# World checklist of hornworts and liverworts

**DOI:** 10.3897/phytokeys.59.6261

**Published:** 2016-01-27

**Authors:** Lars Söderström, Anders Hagborg, Matt von Konrat, Sharon Bartholomew-Began, David Bell, Laura Briscoe, Elizabeth Brown, D. Christine Cargill, Denise P. Costa, Barbara J. Crandall-Stotler, Endymion D. Cooper, Gregorio Dauphin, John J. Engel, Kathrin Feldberg, David Glenny, S. Robbert Gradstein, Xiaolan He, Jochen Heinrichs, Jörn Hentschel, Anna Luiza Ilkiu-Borges, Tomoyuki Katagiri, Nadezhda A. Konstantinova, Juan Larraín, David G. Long, Martin Nebel, Tamás Pócs, Felisa Puche, Elena Reiner-Drehwald, Matt A.M. Renner, Andrea Sass-Gyarmati, Alfons Schäfer-Verwimp, José Gabriel Segarra Moragues, Raymond E. Stotler, Phiangphak Sukkharak, Barbara M. Thiers, Jaime Uribe, Jiří Váňa, Juan Carlos Villarreal, Martin Wigginton, Li Zhang, Rui-Liang Zhu

**Affiliations:** 1 Department of Biology, Norwegian University of Science and Technology, N-7491 Trondheim, Norway; 2 Department of Science and Education, Field Museum, 1400 South Lake Shore Drive, Chicago, IL 60605–2496, United States of America; 3 Department of Biology, West Chester University, West Chester, PA 19383, United States of America; 4 Department of Botany, University of British Columbia, 6270 University Boulevard, Vancouver, BC, V6T 1Z4, Canada; 5 Royal Botanic Gardens and Domain Trust, Mrs Macquaries Road, Sydney NSW2000, Australia; 6 Centre for Australian National Biodiversity Research, Australian National Herbarium, GPO Box 1600, Canberra, ACT 2601, Australia; 7 Instituto de Pesquisas Jardim Botânico do Rio de Janeiro, Rua Pacheco Leão 915, 22460-030, Rio de Janeiro, RJ, Brazil; 8 Department of Plant Biology, Southern Illinois University, Carbondale, Illinois 62901-6509, United States of America; 9 CMNS-Cell Biology and Molecular Genetics, 2107 Bioscience Research Building, University of Maryland, College Park, MD 20742-4451, United States of America; 10 Apartado 5-1500, Acosta, Costa Rica; 11 Systematic Botany and Mycology, Ludwig Maximilian University of Munich, Menzinger Str. 67, 80638 Munich, Germany; 12 Allan Herbarium, Landcare Research, P O Box 69-040, Lincoln 7608, New Zealand; 13 Muséum National d’Histoire Naturelle, Department Systématique et Evolution, C.P. 39, 57 Rue Cuvier, 75231 Paris 05, France; 14 Botany Unit, Finnish Museum of Natural History, University of Helsinki, P.O. Box 7, Helsinki FI-00014, Finland; 15 Department of Systematic Botany with Herbarium Haussknecht and Botanical Garden, Friedrich Schiller University, Fürstengraben 1, 07737 Jena, Germany; 16 Museu Paraense Emilio Goeldi, Coordenaçao de Botanica, Av. Magalhaes Barata 376, 66040-1 70 Belem, Para, Brazil; 17 Department of Biological Science, Graduate School of Science, Hiroshima University, Kagamiyama 1–3–1, Higashihiroshima-shi, Hiroshima 739–8526, Japan; 18 N.A. Avrorin Polar-Alpine Botanical Garden–Institute of Kola SC RAS, 184236 Kirovsk-6, Russia; 19 Royal Botanic Garden, Edinburgh EH3 5LR, United Kingdom; 20 Staatliches Museum für Naturkunde Stuttgart, Rosenstein 1, 70191 Stuttgart, Germany; 21 Botany Department, Institute of Biology, Eszterházy Károly College, Eger, Pf. 43, H-3301, Hungary; 22 Departamento de Botánica, Facultad de Ciencias Biológicas, Universitat de València. C/ Dr. Moliner 50, E-46100, Burjassot (Valencia), Spain; 23 Albrecht-von-Haller-Institut für Pflanzenwissenschaften, Department of Systematics, Biodiversity and Evolution of Plants, Untere Karspüle 2, 37073 Göttingen, Germany; 24 Mittlere Letten 11, 88634 Herdwangen-Schönach, Germany; 25 Centro de Investigaciones sobre Desertificación (CIDE-CSIC-UV-GV), C/ Carretera de Moncada-Náquera Km. 4.5, E-46113, Moncada (Valencia), Spain; 26 Department of Biology, Faculty of Science, Burapha University, Mueang, 20131 Chonburi, Thailand; 27 William and Lynda Steere Herbarium, The New York, Botanical Garden, Bronx, New York 10458-5126, United States of America; 28 Instituto de Ciencias Naturales. Universidad Nacional de Colombia. Apartado 7495, Bogotá D.C., Colombia; 29 Department of Botany, Charles University, Benátská 2, CZ-128 01 Praha 2, Czech Republic; 30 Department of Biology, Ludwig-Maximilians-Universität, Menzinger Str. 67, D-80638, München, Germany; 31 36, Big Green, Warmington, Peterborough PE8 6TU, United Kingdom; 32 Shenzhen Key Laboratory of Southern Subtropical Plant Diversity, Fairylake Botanical Garden, 160 Xianhu Rd., Liantang, Shenzhen 518004, Guangdong, China; 33 Department of Biology, School of Life Sciences, East China Normal University, 3663 Zhong Shan North Road, Shanghai 200062, China*; 34

**Keywords:** Marchantiophyta, Anthocerophyta, nomenclature, taxonomy

## Abstract

A working checklist of accepted taxa worldwide is vital in achieving the goal of developing an online flora of all known plants by 2020 as part of the Global Strategy for Plant Conservation. We here present the first-ever worldwide checklist for liverworts (Marchantiophyta) and hornworts (Anthocerotophyta) that includes 7486 species in 398 genera representing 92 families from the two phyla. The checklist has far reaching implications and applications, including providing a valuable tool for taxonomists and systematists, analyzing phytogeographic and diversity patterns, aiding in the assessment of floristic and taxonomic knowledge, and identifying geographical gaps in our understanding of the global liverwort and hornwort flora. The checklist is derived from a working data set centralizing nomenclature, taxonomy and geography on a global scale. Prior to this effort a lack of centralization has been a major impediment for the study and analysis of species richness, conservation and systematic research at both regional and global scales. The success of this checklist, initiated in 2008, has been underpinned by its community approach involving taxonomic specialists working towards a consensus on taxonomy, nomenclature and distribution.

This checklist is dedicated to Elizabeth Brown and Ray Stotler, who sadly left us during our work with it.

“……Charles Darwin wrote of his desire to provide financial support ‘for the formation of a perfect M.S. catalogue of all known plants’ (Darwin 1881, letter 13570). It is a personal embarrassment to me, and should be chastening to us all, that more than 120 years later we still have not delivered on that commitment.” (Crane 2004).

## Introduction

The natural world is changing fast (Balmford et al. 2005). The Global Strategy for Plant Conservation (GSPC) was adopted by the Conference of Parties (COP) of the Convention on Biological Diversity (CBD) in April 2002 (http://www.cbd.int/decision/cop/?id=7183; accessed 2014.06.02). The Strategy set out 16 outcome-oriented targets that were to be achieved by 2010. The GSPC was designed as a framework for action to halt the loss of plant diversity. Target 1 of the Strategy was to complete “a widely accessible working list of all known plant species, as a step towards a complete world Flora” (Lughadha 2004). As early as 1881 Charles Darwin expressed a wish to have a catalogue of all known plants (Crane 2004). However, over 125 years later, this wish is not yet fulfilled. Without a working checklist, many of the other objectives in the GSPC cannot be met and botanists around the world cannot communicate about plants on a global basis (Crane 2004). A working list of known plant species is critical for (I) its underpinning role in the effective implementation of the other targets through provision of baseline information; (II) increasing the accessibility and use of accurate botanical name information for research, conservation and sustainable use; and (III) real-world politics and how taxonomists respond to the decisions of policy makers (Paton et al. 2008).

Version 1.0 of The Plant List (http://www.theplantlist.org/) was released in December 2010 aimed to be comprehensive for species of vascular plants (flowering plants, conifers, ferns and their allies) and of bryophytes (mosses, liverworts and hornworts), as a response to the 2010 Target 1 of the GSPC and to a clear global need for such data. The Plant List is a broad collaboration, coordinated by the Royal Botanic Gardens, Kew, and the Missouri Botanical Garden (MO), involving diverse partnerships. Paton (2013) noted The Plant List represents a work in progress with future versions planned to improve the quality of names and decrease the number of unresolved names. Target 1 has since been revised and agreed at the 10th COP of the CBD in Nagoya, Japan, with the goal of developing ‘‘an online flora of all known plants’’ by 2020 (http://www.cbd.int/decision/cop/default.shtml?id=12283; accessed 2014.06.02). Paton (2013) identified several factors that might assist in the implementation of the revised target, stressing that achieving a working list for plant taxa worldwide was a vital step to an online flora.

Earlier, Paton et al. (2008) assessed the progress made at that time and discussed prospects for the completion of Target 1. Paton noted that good progress had been made in bryophytes (mosses, liverworts and hornworts), ferns and gymnosperms with widely accessible working lists either complete or almost so for those groups. For bryophytes alone, he tabulated 13,370 accepted species noting that the data was largely derived from Tropicos. However, although Tropicos is an indispensable reference for anyone dealing with bryophyte names and the database is very strong for mosses, the nomenclatural and auxiliary data for liverworts is less complete, especially for larger genera (von Konrat et al. 2008a). The lack of a central source providing a synthesis of nomenclatural data and global distributional data was the impetus toward developing the current checklist of liverworts and hornworts (von Konrat et al. 2008a, 2010d, Söderström et al. 2012e). Checklists are powerful and important tools that can integrate the almost overwhelmingly scattered information concerning taxonomy, systematics, nomenclature, distribution, and even frequency (Söderström et al. 2008).

### Ecological and biological significance of liverworts and hornworts

**Figure 1.**
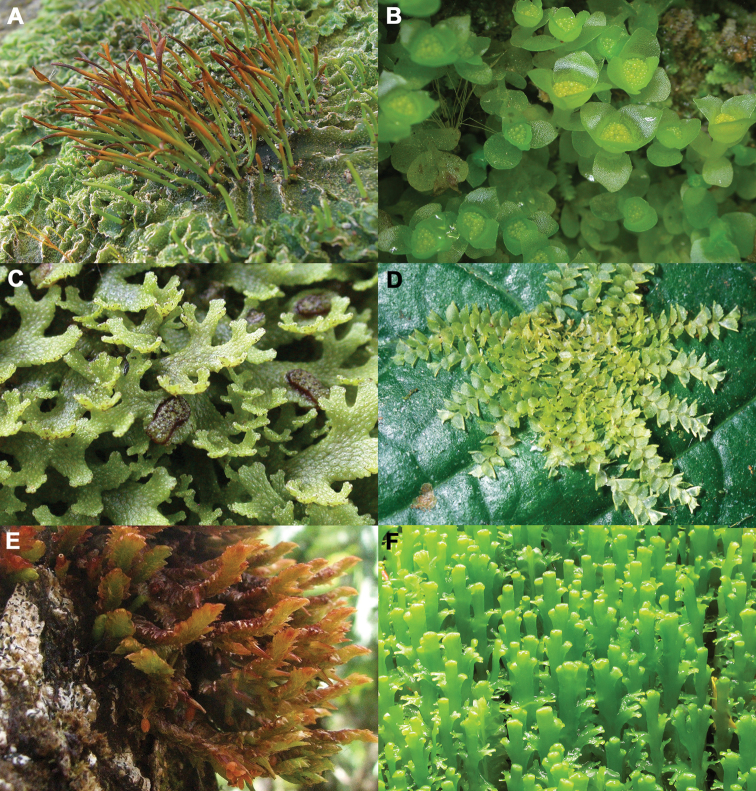
Habit images of selected lineages of Anthocerotophyta (hornworts) and Marchantiophyta (liverworts). **A** Phaeomegaceros squamuliger, photo by J. Hollinger **B** Haplomitrium mnioides, photo by L. Zhang **C** Conocephalum japonicum, photo by D. G. Long **D** Colura vitiensis, photo by T. Pócs **E** Pleurozia gigantea, photo by L. Söderström **F** Riccardia spegazziniana, photo by Juan Larraín.

Liverworts and hornworts (Figure 1) are of critical biological, ecological, and phylogenetic significance (e.g. Asakawa 1998, Longton 1992, Hallingbäck and Hodgetts 2000, Gradstein et al. 2001b, Wellman et al. 2003, Qiu et al. 2007). Liverworts are found on soil, rocks, and trees throughout the world, from coastal Antarctica to the tundra of the Northern Hemisphere, and from semi-arid areas of Australia to the Amazon rainforests (Hallingbäck and Hodgetts 2000). Although there are xero-tolerant taxa, the majority of liverworts are found in relatively humid and shaded terrestrial ecosystems (Gradstein and Costa 2003). Liverworts and hornworts are an important component of the vegetation in many regions of the world, constituting a major part of the biodiversity in moist forest, wetlands, mountain, and tundra ecosystems (Hallingbäck and Hodgetts 2000).

Liverworts, hornworts and mosses offer microhabitats that are critical to the survival of a tremendous diversity of organisms such as single-celled eukaryotes, protozoa, and numerous groups of invertebrates (Gerson 1982). Their structural contribution to levels of diversity might be as significant as that of vascular plants, albeit at a smaller scale (von Konrat et al. 2008a). These plants are also important environmental and ecological indicators (Rao 1982, Pitcairn et al. 1995, Gradstein et al. 2001b, Giordano et al. 2004). Liverworts, in concert with mosses and hornworts, play a significant role in the global carbon budget (O’Neill 2000) and CO_2_ exchange (De Lucia et al. 2003), plant succession (Cremer and Mount 1965), net production and phytomass (Frahm 1990), nutrient cycling (Coxson 1991), and water retention (Pócs 1980a, Gradstein et al. 2001b). These groups of land plants also have been used as indicators of past climate change, to validate climate models, and as potential indicators of global warming (Gignac 2001, Tuba et al. 2011).

### Species numbers

There are estimated to exist only 215 hornwort species. By comparison estimates of liverwort species richness have varied considerably, by as much as 50%, with estimates ranging from 4,500 to 9,000 (e.g. Pearson 1995, Forrest et al. 2006). In the last decade or so estimates in the range of 5,000 to 6,000 liverwort species have been widely accepted (e.g. Gradstein and Costa 2003, Gradstein and Ilkiu-Borges 2009, Heinrichs et al. 2007). von Konrat et al. (2010a) provided a mean estimate of 7,500 for the number of liverwort species based on estimating rates of synonymy in a sample of recently monographed and revised taxa. A standardized global worldwide liverwort checklist with strong community participation, as presented here, coupled with the critical need for ongoing monographs and revisions, will aid in arriving at clearer estimates of liverwort and hornwort diversity. Significantly, a list of standardized names reviewed by taxonomic experts will enable more meaningful geographical and biogeographical species comparative studies. The global checklist of accepted liverwort and hornwort taxa has vast potential, not only in aiding our understanding of liverwort diversity, patterns, and processes, but also to the broader biological community (von Konrat et al. 2008a).

Below we provide context to the worldwide checklist, including a brief historical account and current informational resources, structure and layout, systematic and classification concepts, and methodology, including a brief overview of the underlying data set and nomenclatural elements.

### Major historical works

Schuster (1966d) has provided a detailed account on the history of hepaticology, up to the first half of the 20th century. Earlier, Verdoorn (1934c) also outlined a valuable historical review of important figures in hepaticology. In the early 19th century, possibly the two most influential persons who provided an early framework for taxonomic concepts were W.J. Hooker and G. Raddi. During the middle of the 19th century C.G. Nees von Esenbeck, C.M. Gottsche and J.B.W. Lindenberg provided vastly significant contributions towards developing a classification framework. Frahm and Eggers (2001) provided a brief biography of these workers. This was later followed by R. Spruce and the Austrian-born H. Leitgeb in the second half 19th century. Leitgeb in particular was instrumental in liverwort plant morphology and anatomy including a six part treatise, “Untersuchungen ueber die Lebermoose”, from 1874 to 1881. Towards the end of 19th century and early 20th century V. Schiffner, F. Stephani, and A. Evans were prominent hepaticologists. In the first half of the 20th century A. Evans, H. Buch, F. Verdoorn, and K. Müller (Frib.) were leading influential hepaticologists. In the second half of the 20th century, R. Grolle and R.M. Schuster were of extraordinary influence in working towards contemporary classification schemes. Many of these groundbreaking concepts still serve the foundation from which our current classification scheme is derived. R.M. Schuster’s major contribution to botany and hepaticology lies in the astounding new diversity of liverworts he added to our knowledge. He described a staggering 463 species, 83 genera and 15 families new to science. It is difficult to name another contemporary botanist who discovered this much new diversity of a major clade of land plants (Qiu et al. 2013). R.M. Schuster’s contribution to botany went beyond hepatics. He was one of the first botanists who recognized the importance of Wallace’s Line in plant biogeography, separating Australia of Gondwanaland from Southeast Asia of Laurasia (Cronquist 1988).

**Figure 2.**
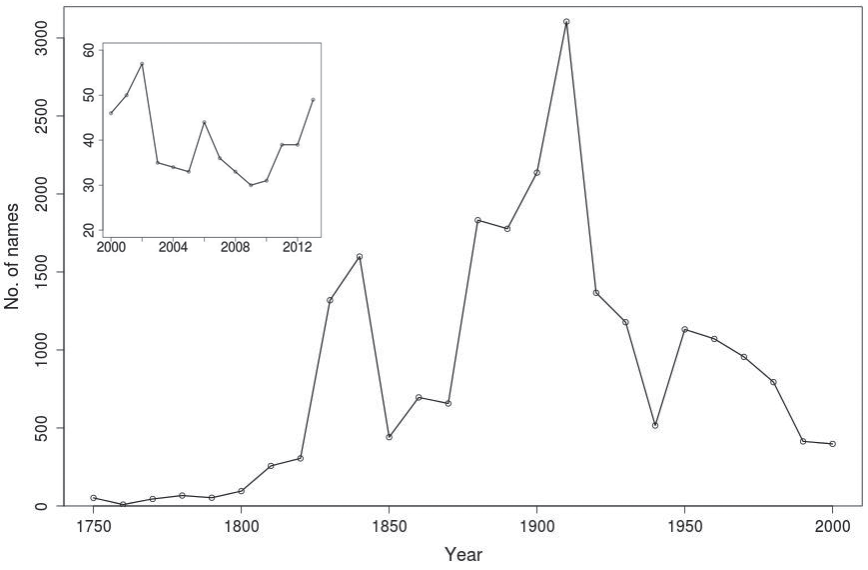
Number of novel liverwort species, excluding new combinations, which have been described over the last 250 years, with an inset of the number described from 2001–2012.

Figure 2 depicts the number of novel liverwort species, excluding new combinations that have been described over the last 250 years. The first major peak corresponds to the works of several early 19th century botanists, including Synopsis Hepaticarum by Gottsche, Lindenberg, and Nees (1844–1847). The three decades leading into, but prior to the highest peak, between 1860 and 1890, correspond to publications by a number of prominent bryologists including W. Mitten, J.D. Hooker, T. Taylor, and V. Schiffner. The second and highest peak of almost 1,200 names, in the early 1900s corresponds largely to the plethora of taxa described by F. Stephani (1898–1924) in his monumental work Species Hepaticarum. The periods of highest rates of new species described in the 1830s and around 1900 are the same for seed plants (Mutke and Barthlott 2005). The third peak over the four decades between 1950 and 1980 can be attributed mainly to the works of R.M. Schuster, H. Inoue, and S. Hattori. H. Inoue and S. Hattori were influential on large species-rich genera such as Plagiochila, Frullania, and Porella. The decline in newly described species since 1970 does not necessarily translate to the conclusion that taxonomists are closer to discovering all known species. The almost 200 novel liverwort species that have been described in the past six years alone still represent a significant number, considering the relatively few liverwort taxonomists and monographers. Moreover, bryological exploration has been very uneven in many parts of the world. For example, many areas of the Neotropics still remain without a single bryophyte record (Gradstein et al. 2001a). Paradoxically, scores of new species are still being discovered and described in relatively well-studied areas such as New Zealand, e.g. over 50 new taxa since 2000 alone (e.g. Engel and Schuster 2001, Engel and Glenny 2008a, Renner and de Lange 2011, von Konrat et al. 2012b). Recent attention to cryptic speciation in bryophytes is also revealing novel liverwort species (e.g. Szweykowski et al. 2005). The combination of collecting in yet-to-be explored areas, the continued discovery of species in well-studied regions, and an increased understanding of the biology of liverworts (including cryptic speciation), will lead to a significant number of newly discovered species in the foreseeable future. The corollary of this, coupled with increased monographic and revisional work, will be the increased discovery and the unravelling of synonymy.

### Contemporary resources

Data availability and information needs associated with liverworts and hornworts have been reviewed extensively by von Konrat et al. (2008a, 2010a). A fundamental problem common to all nomenclatural indexing projects is the dispersed nature of the biological literature, some of which may date back over 250 years (Lughadha 2004). Our own data records, from 1990 to the present alone, indicate there are over 190 periodical titles and non-serials in which liverwort nomenclatural novelties were published. The most useful and successful web-accessible bryophyte nomenclatural database is Tropicos (http://www.tropicos.org), which offers name data with references and type information as well as links to specimen data of its holdings. Tropicos has therefore become an indispensable reference for anyone dealing with bryophyte names. However, as stated above, the database is particularly strong for mosses and far less comprehensive for liverworts. Moreover, Tropicos does not prescribe taxonomic disposition of names, i.e., there is no attempt to adopt a single consistent view on the status of any particular name (Lughadha 2004, von Konrat et al. 2008a).

A major nomenclatural work is the *Index Hepaticarum* (http://www.ville-ge.ch/musinfo/bd/cjb/hepatic/index.php), which includes all effectively published liverwort epithets spanning 12 volumes with the closing date of 1973. The indices were prepared as a purely nomenclatural resource and did not claim to express any particular taxonomic concept. Recently, Crosby and Engel (2006) provided an equally valuable nomenclatural resource and catalog of names at all ranks for liverworts and hornworts published during 1974 to 2000. Subsequently, indices of the citations for names published for bryophytes have been compiled for the years 2001-2004 (Crosby and Magill 2005) and 2005 (Crosby and Magill 2006). The Early Land Plants Today initiative has continued this series (von Konrat et al. 2010c, Söderström et al. 2012b, 2014).

The application of molecular phylogenetics continues to generate new insights into the evolutionary history of liverworts and hornworts. In recent years, inferences made from these phylogenies have especially revolutionized liverwort classification (Crandall-Stotler et al. 2009). Yet, the reconstruction of the phylogenetic history of the Marchantiophyta and Anthocerotophyta remains an ongoing effort and the classification is fluid (von Konrat et al. 2010a). The current checklist largely follows the comprehensive phylogenetic liverwort classification scheme provided by Crandall- Stotler et al. (2009) and the hornwort classification scheme of Stotler and Crandall-Stotler (2005). Thus the checklist largely adheres to their higher classification namely from class to suborder, and the majority of family and genera arrangements provided therein. However, there are some notable exceptions, especially in the systematic treatment of liverworts, which largely either reflect publications subsequent to 2009, or are a slight departure in taxonomic opinion based on earlier works, e.g. the concepts of Saccogynaceae and Stephaniellaceae depart from Crandall-Stotler et al. (2009). Since 2010 there have been a number of realignments and novel hypotheses proposed that were generated from conclusions based on molecular evidence which have influenced the systematic treatment presented here. These include, for example, Adelanthaceae (e.g. Konstantinova and Vilnet 2009, Feldberg et al. 2010b, 2010a, Söderström et al. 2010b, Vilnet et al. 2010), Anastrophyllaceae (e.g. Söderström et. al 2010b), Gymnomitriaceae (e.g. Vilnet et al 2007a, 2010, Váňa et al. 2010b, Shaw et al. 2015), Lepidoziaceae (Cooper 2013), Lophocoleaceae (Söderström et al. 2013b), and Scapaniaceae (e.g. Vilnet et al. 2010, Heinrichs et al. 2012a). In all cases, any such departures, new families, or realignments are noted and very briefly discussed throughout the checklist. As noted by Crandall-Stotler et al. (2009) many small families still remain to be investigated using molecular data, and many of the large families and genera that have been sampled appear to be either polyphyletic or paraphyletic.

The systematic treatment as outlined here also includes the addition of subfamily and infrageneric classification; ranks not presented by Crandall-Stotler et al. (2008b, 2009). Although there are arguably a number of ambiguous placements, especially at the infrageneric rank, and there remain many areas of contention, particularly at the familial and generic level, the systematic treatment itself serves as an excellent synopsis of contemporary taxonomic opinion, including infrageneric ranks. The infrageneric treatments will also aid future efforts in providing a taxonomic framework that will be particularly useful for extremely large genera. In large part, the infrageneric treatments only deal with those taxa that represent phylogenetic clades with strong support. Care has been taken to reduce ambiguity and those taxa where infrageneric placement is weak or without evidence are simply listed under the genus and not placed systematically.

## Structure and format of the checklist

The checklist is presented in a taxonomic framework as outlined above with four main sections. The first is arranged systematically with taxa ordered alphabetically within the nearest higher rank. A brief discussion for selected families is provided in the main body of the text under the respective families. This does not appear uniformly for all families, but typically for larger more complicated families. Rather, a brief description is provided mainly where concepts depart from Crandall-Stotler et al. (2009) or if new information has come to light since that publication. Subfamilies and infrageneric ranks have been incorporated where appropriate. However, if the subgeneric placement is unclear the species are listed under “*Incertae sedis*”. From the rank of genus and below, all entries include a three star confidence ranking (described below), accepted taxon name, authority, abbreviated publication title, page number and date, which is cross- referenced to the full citation in the reference list, as well as the basionym if appropriate. A typical entry appears as follows:

*** Oleolophozia L.Söderstr., De Roo et Hedd., Phytotaxa 3: 50, 2010 (Söderström et al. 2010b).

*** Oleolophozia perssonii (H.Buch et S.W.Arnell) L.Söderstr., De Roo et Hedd., Phytotaxa 3: 51, 2010 (Söderström et al. 2010b). Bas.: Lophozia perssonii H.Buch et S.W.Arnell, Bot. Not. 97: 382, 1944 (Buch 1944).

There are comprehensive footnotes throughout the systematic section of the checklist. These are applied to selected names representing the rank of genus and below only. Footnotes without any literature citation represent observations from the authors of the respective family. Footnotes are wide ranging in their content. Generally, footnotes can be categorized into the following: i) general statements relating to taxonomic opinion, ii) comments on the type specimen, iii) comments about possible conspecificity, including citation of conflicting opinions, and iv) comment about species complexes.

This section is followed by a list of taxa in genera that we do not recognize. Those names represent taxa that have not been studied recently and have not been recognized in any recent treatment. However, many of those names are old and may gain priority over some recognized taxa once their identity is determined.

The next section is an alphabetical list of taxa of the ranks of species and below. The alphabetical list includes the confidence level, the taxon name and authority as well as a reference to the page of the corresponding entry in the systematic section. Thus, the alphabetical list provides a rapid gateway requiring no prior knowledge of higher taxonomy, but also serves as an index to the corresponding name in the systematic section that includes more detailed information.

The last section is a reference list where full bibliographic citations are given to all references for taxa included in the checklist. Titles and references have all been checked and verified, except in a few cases where we could not get hold of the publications.

### Taxon confidence levels

Significantly, each accepted taxon is qualified using a three level ranking system that summarizes our knowledge about a taxon. The coding convention we are adopting largely follows that described by von Konrat et al. (2010d) using one to three stars, which has been applied to recent regional checklists produced by the Early Land Plants Today initiative, e.g. Söderström et al. (2010a, 2011a, 2011b, 2012g, 2013e). The conventions are briefly outlined below coupled with samples illustrating how these conventions are applied in practice. The application of a confidence level to a taxon’s status and whether it represents a genuine “species” that is reached through community consensus may go towards refining species estimates using an evidence-based approach (von Konrat et al. 2008a, 2008b). The confidence levels coupled with the detailed annotations in the form of footnotes may also provide a rapid assessment of taxa and help aid and drive future research into specific taxonomic or nomenclatural problems and issues.

The coding convention:

* Serious doubts. There are doubts about the value of the taxon. It can be that there are conflicting views without any substantial evidence in any direction, conflicting views with substantiating evidence supporting one or both positions, or evidence pointing towards synonymization but it is premature to do it. We have adhered to the principle that it is better to keep a taxon with one star (and preferably a note) than to synonymize it too quickly. Example: Bazzania asymmetrica is conspecific with Bazzania macgregorii in Grolle (1968a), but Kitagawa (1979a) kept them separate. Example: Nardia kamtschatica may be conspecific with Nardia assamica (Váňa 1976c), but the type specimen could not be studied.

** Knowledge problem. The taxon is not well known by the person evaluating it. It may be a newly described species or a species originally not well described and not restudied recently. Example: Jungermannia erectii Ajit P.Singh et V.Nath was recently described and has not been independently studied by someone with a global overview. It is therefore difficult to evaluate.

*** Accepted. A good taxon as currently understood based on personal experience or on taxonomic revisions that have been convincingly performed. Nomenclature and/or taxonomic position may, however, be questioned. Elements may be excluded from the taxon, but the taxon with the current type will still be accepted. Example: H. Bischler-Causse revised the genus Marchantia worldwide in a series of publications. Although she had a broad species concept, she also recognized infraspecific taxa. The taxa that she recognized without doubt should be accepted unless new evidence against it exists. Adopting a narrower species concept so that many of her subspecies are elevated to species does not change her view of what a good taxon is.

### Methodology

The foundation of the checklist is in the underlying data set from which it is derived. It was briefly described by von Konrat et al. (2008a, 2010a). The working data sets now includes a bibliography of 25,000 publications; approximately 39,000 published liverwort names (including “accepted” taxa, synonyms, invalid and illegitimate names). The data quality and standards were outlined by von Konrat et al. (2010d). In summary, for authorities and for the citation abbreviations, we follow the standards set by the on-line version of *Authors of Plant Names* at the Royal Botanical Gardens, Kew Website (http://www.ipni.org), with whom we collaborate closely and provide with updated data records. Publications and journal abbreviations follow *Taxonomic Literature: A selective guide to botanical publications and collections with dates, commentaries and types* (Stafleu and Cowan 1976–2009) (http://www.sil.si.edu/digitalcollections/tl-2/search.cfm) and *Botanico Periodicum Huntianum* Lawrence et al. (1968) (http://huntbot.andrew.cmu.edu/HIBD/Departments/Databases.shtml).

There has been an intense systematic effort focusing on data quality. In all but a few cases nomenclatural data has been verified against original publications. Söderström and Hagborg have checked and confirmed almost every original publication (three publications have not been available) for correct author, title and journal/book citation, date of publication as far as possible, page number for the protologue, and if the name is validly and legitimately published according to the International Code of Nomenclature for algae, fungi and plants (Melbourne Code; McNeill et al. 2012). Most significantly, the checklist has been community driven and collaborative. Broader participation by taxonomic specialists and regional experts has lead to the checklist containing high quality data (cf. Söderström et al. 2008). Development of the checklist has included three international meetings (the first one in 2009) and generated 74 published notes on taxonomy and nomenclature under the auspices of the Early Land Plants Today initiative from 2012 to 2015. The series of meetings were instrumental in providing a framework for direct interaction with taxonomic experts, workshops reviewing names, and helping identify potential participants who were taxonomic experts in specific taxonomic groups, whether these were individual genera or entire families.

The notes, published in the journal Phytotaxa, have included 38 authors from 13 countries. These are the results of several years of revisions of taxonomic groups by taxonomic experts. Following these revisions and working with participants identifying nomenclatural and taxonomic problems that would impact the worldwide checklist, Söderström, Hagborg and von Konrat led editorial efforts in compiling manuscripts with baseline information and nomenclatural or taxonomic issues that required resolving. The work was then coordinated with the taxonomic experts who drove and led the process and often identified further issues. The series of notes on taxonomy and nomenclature of liverworts and hornworts provided updated nomenclature, corrected invalid and illegitimate names and described new taxa based on studies (mainly molecular) that did not draw nomenclatural conclusions. Those notes are all open access and any changes were effective immediately as the Melbourne Code of Botanical Nomenclature allows publishing on internet from 1 Jan. 2012 (Knapp et al. 2011).

This checklist builds on all published taxonomic and nomenclatural papers, that have come to our attention until June 30, 2015.

### Summary statistics

We here present the first-ever worldwide checklist for liverworts (Marchantiophyta) and hornworts (Anthocerotophyta) that includes 8,078 taxa (species and below) in 7,486 species representing 398 genera, 92 families, 20 orders and 7 classes from the two phyla. The list includes 3,533 species with three stars, 2,988 species with two stars and 915 species with one star. The checklist also has extra utility in that it contains 3,106 references in the bibliography that serve as a powerful bibliographic resource for liverwort and hornwort systematic and taxonomic research.

## Concluding remarks

The marked-up publication form of the current checklist by PhytoKeys provides a virtual instrument with a linked environment both internally (e.g. within an article) and externally (GBIF, IPNI, Tropicos, Wikispecies, etc.) that will undoubtedly help accelerate taxonomic research. The published checklist was the first phase in providing a worldwide list of accepted names. The next phase is to establish a generally recognized online repository to augment the huge underlying informational auxiliary data of over 25,000 publications, almost 39,000 published names, and the over 700,000 geographical observations. Several features of Web-based technology make it an essential tool, including 1) a vehicle to facilitate data access, 2) to unify the vastly scattered data on distributional information and nomenclature, 3) offering dynamic rather than static data in a searchable forum. The broader accessibility to the wealth of auxiliary data will help augment monographic and revisional work for many taxonomic groups, aid in identifying the need for increased floristic and survey work in many regions throughout the world, and have broad implications and applications beyond taxonomic research such as conservation science. The current Early Land Plants Today model has strong participation from taxonomic experts and an online resource will provide an opportunity to expand stakeholders to include ecologists, conservationists, scientists from other disciplines and general interest groups. However, such an effort can only be successful if it comes with sustained funding and infrastructure rather than depending on an ad hoc commitment by a few individuals, however dedicated.

The project will help augment monographic and revisional work for many taxonomic groups and aid in identifying the need for increased floristic and survey work in many regions throughout the world. Although there are many challenges ahead to obtain high quality data, quantifying global liverwort diversity is a tractable, multi-faceted and scientifically important goal, and everyone stands to gain by fostering this endeavour. The success of the project will lie on strong collaboration between institutions and the bryological community in general.

## Taxonomic list

### Anthocerotophyta

#### Anthocerotopsida de Bary ex Jancz.

##### Anthocerotidae Rosenv.

###### Anthocerotales Limpr.

####### *** Anthocerotaceae Dumort.

by J.C. Villarreal and D.C. Cargill

Notes on nomenclature and taxonomy can also be found in Cargill et al. (2013b) and Villarreal et al. (2015)

*** **Anthoceros L.**, Sp. Pl. 1: 1139, 1753 (Linnaeus 1753). ^1^

*** Anthoceros adscendens Lehm. et Lindenb., Nov. Stirp. Pug. 4: 24, 1832 (Lehmann 1832).

*** Anthoceros agrestis Paton, J. Bryol. 10 (3): 257, 1979 (Paton 1979a), *nom. conserv*. ^2^

*** Anthoceros alpinus Steph., Sp. Hepat. (Stephani) 6: 425, 1923 (Stephani 1923).

* Anthoceros angustifolius Gottsche, Lindenb. et Nees, Syn. Hepat. 4: 582, 1846 (Gottsche et al. 1846). ^3^

*** Anthoceros angustus Steph., Sp. Hepat. (Stephani) 5: 1001, 1916 (Stephani 1916b).

*** Anthoceros bharadwajii Udar et A.K.Asthana, Proc. Indian Natl. Sci. Acad., B 51 (4): 484, 1985 (Udar and Asthana 1985b).

** Anthoceros buettneri Steph., Sp. Hepat. (Stephani) 5: 997, 1916 (Stephani 1916b).

*** Anthoceros capricornii Cargill et G.A.M.Scott, J. Hattori Bot. Lab. 82: 55, 1997 (Cargill and Scott 1997).

*** Anthoceros caucasicus Steph., Izv. Kavkazsk. Muz. 8: 87, 1914 (Voronov’ 1914).

** Anthoceros cavernosus Steph., Sp. Hepat. (Stephani) 5: 998, 1916 (Stephani 1916b).

* Anthoceros chambensis Kashyap, J. Bombay Nat. Hist. Soc. 25 (2): 281, 1917 (Kashyap 1917).

** Anthoceros chungii Khanna, J. Indian Bot. Soc. 17 (5/6): 316, 1938 (Khanna 1938).

** Anthoceros crispatus Griff., Not. pl. asiat. 2: 349, 1849 (Griffith 1849).

** Anthoceros dimorphus Sim, Trans. Roy. Soc. South Africa 15 (1): 114, 1926 (Sim 1926).

*** Anthoceros erectus Kashyap, New Phytol. 14 (1): 9, 1915 (Kashyap 1915).

** Anthoceros expansus (Steph.) J.C.Villarreal et Cargill, Phytotaxa 208 (1): 92, 2015 (Villarreal et al. 2015). Bas.: Aspiromitus expansus Steph., Sp. Hepat. (Stephani) 5: 961, 1916 (Stephani 1916b).

* Anthoceros ferdinandi-muelleri Steph., Sp. Hepat. (Stephani) 5: 1007, 1916 (Stephani 1916b). ^4^

*** Anthoceros fragilis Steph., Sp. Hepat. (Stephani) 5: 1006, 1916 (Stephani 1916b).

*** Anthoceros fusiformis Austin, Bull. Torrey Bot. Club 6 (4): 28, 1875 [1876] (Austin 1875a).

*** Anthoceros fusiformis var. taiwanensis J.Haseg., Acta Phytotax. Geobot. 44 (2): 100, 1993 (Hasegawa 1993b).

** Anthoceros gasongorii Gola, Mem. Reale Accad. Sci. Torino (ser. 2) 65 (1): 10, 1916 (Gola 1916).

** Anthoceros granulatus Gottsche, Mexik. Leverm.: 275, 1863 (Gottsche 1863).

** Anthoceros harrisanus (Steph.) Parihar, Univ. Allahabad Stud., Bot. 1961-2: 31, 1962 (Parihar 1962). Bas.: Aspiromitus harrisanus Steph., Sp. Hepat. (Stephani) 5: 965, 1916 (Stephani 1916b).

* Anthoceros helmsii Steph., Hedwigia 32 (3): 142, 1893 (Stephani 1893b). ^5^

** Anthoceros jamesonii Taylor ex Mitt., Bot. antarct. voy. II (Fl. Nov.-Zel. 2): 171, 1855 (Mitten 1855).

* Anthoceros javanicoides H.A.Mill., Phytologia 47 (4): 319, 1981 (Miller 1981). *Nom. nov. pro Anthoceros javanicus* Steph., Sp. Hepat. (Stephani) 5: 988, 1916 (Stephani 1916b), *nom. illeg*. ^6^

** Anthoceros jungermannioides Schwein., Spec. Fl. Amer. Crypt.: 25, 1821 (Schweinitz 1821).

* Anthoceros kajumas (K.I.Goebel) Prosk., Bull. Torrey Bot. Club 78 (4): 347, 1951 (Proskauer 1951a). Bas.: Aspiromitus kajumas K.I.Goebel, Ann. Jard. Bot. Buitenzorg 39: 69, 1928 (Goebel 1928).

* Anthoceros koshii Khanna, J. Indian Bot. Soc. 15 (4): 237, 1936 (Khanna 1936).

*** Anthoceros lamellatus Steph., Sp. Hepat. (Stephani) 5: 1000, 1916 (Stephani 1916b).

*** Anthoceros laminifer Steph., J. Linn. Soc., Bot. 29 (201): 266, 1892 (Stephani 1892b).

*** Anthoceros macounii M.Howe, Bull. Torrey Bot. Club 25 (1): 19, 1898 (Howe 1898a).

*** Anthoceros macrosporus Steph., Sp. Hepat. (Stephani) 5: 1005, 1916 (Stephani 1916b).

** Anthoceros maritimus Steph., Sp. Hepat. (Stephani) 5: 984, 1916 (Stephani 1916b).

* Anthoceros megasporus Meijer, J. Hattori Bot. Lab. 18: 6, 1957 (Meijer 1957).

** Anthoceros muscoides Colenso, Trans. & Proc. New Zealand Inst. 16: 361, 1884 (Colenso 1884).

*** Anthoceros myriandroecius Steph., Wiss. Ergebn. Deut. Zentr.-Afr. Exped. (1907-08), Bot. 2: 134, 1911 (Stephani 1911a).

** Anthoceros natalensis Steph., Akad. Wiss. Wien, Math.-Naturwiss. Kl., Denkschr. 88: 732, 1913 (Stephani 1913b).

*** Anthoceros neesii Prosk., Leberm. Eur. 2 (9): 1312, 1958 (Proskauer 1958). *Nom. nov. pro Anthoceros punctatus* α* monocarpus Nees, Naturgesch. Eur. Leberm. 4: 339, 1838 (Nees 1838a).

** Anthoceros niger Steph., Sp. Hepat. (Stephani) 5: 1005, 1916 (Stephani 1916b).

** Anthoceros orizabensis (Steph.) Hässel, Candollea 45 (1): 211, 1990 (Hässel 1990b). Bas.: Aspiromitus orizabensis Steph., Sp. Hepat. (Stephani) 5: 965, 1916 (Stephani 1916b).

*** Anthoceros pandei Udar et A.K.Asthana, J. Indian Bot. Soc. 64: 305, 1985 (Udar and Asthana 1985a).

*** Anthoceros patagonicus Hässel, Candollea 45 (1): 207, 1990 (Hässel 1990b).

*** Anthoceros patagonicus subsp. gremmenii J.C.Villarreal, J.J.Engel et Váňa, Mem. New York Bot. Gard. 105: 32, 2013 (Váňa and Engel 2013).

** Anthoceros peruvianus Steph., Sp. Hepat. (Stephani) 5: 999, 1916 (Stephani 1916b).

** Anthoceros pinnatus Steph., Bol. Soc. Brot. 4: 154 [182], 1885 [1886] (Stephani 1885c).

*** Anthoceros punctatus L., Sp. Pl. 1: 1139, 1753 (Linnaeus 1753). ^7^

* Anthoceros pusillus Colenso, Trans. & Proc. New Zealand Inst. 18: 255, 1886 (Colenso 1886b).

*** Anthoceros rosulans J.Haseg., J. Hattori Bot. Lab. 60: 379, 1986 (Hasegawa 1986b).

*** Anthoceros sambesianus Steph., Sp. Hepat. (Stephani) 5: 996, 1916 (Stephani 1916b).

*** Anthoceros scariosus Austin, Proc. Acad. Nat. Sci. Philadelphia 21: 230, 1869 (Austin 1869).

** Anthoceros schroederi Steph., 52 (5): 306, 1912 (Stephani 1912a).

** Anthoceros serratus Steph., Kungl. Svenska Vetensk.-Akad. Handl. (n.ser.) 46 (9): 90, 1911 (Stephani 1911b).

** Anthoceros simulans M.Howe, Proc. Calif. Acad. Sci. (ser. 4) 21 (17): 204, 1934 (Howe 1934).

** Anthoceros spongiosus Steph., Sp. Hepat. (Stephani) 5: 1003, 1916 (Stephani 1916b).

*** Anthoceros subtilis Steph., Sp. Hepat. (Stephani) 5: 1003, 1916 (Stephani 1916b).

*** Anthoceros telaganus Steph., Sp. Hepat. (Stephani) 5: 1005, 1916 (Stephani 1916b).

*** Anthoceros tristanianus J.C.Villarreal, J.J.Engel et Váňa, Mem. New York Bot. Gard. 105: 33, 2013 (Váňa and Engel 2013).

*** Anthoceros tuberculatus Lehm. et Lindenb., Nov. Stirp. Pug. 4: 25, 1832 (Lehmann 1832).

** Anthoceros venosus Lindenb. et Gottsche, Syn. Hepat. 4: 584, 1846 (Gottsche et al. 1846).

**Excluded from the genus**

* Anthoceros aethyopicus Gola, Ann. Bot. (Rome) 13 (1): 73, 1914 (Gola 1914a). ^8^

* Anthoceros brunnthaleri Steph., Akad. Wiss. Wien, Math.-Naturwiss. Kl., Denkschr. 88: 732, 1913 (Stephani 1913b). ^9^

* Anthoceros floribundus Steph., Sp. Hepat. (Stephani) 5: 977, 1916 (Stephani 1916b). ^10^

* Anthoceros mildbraedii Steph., Sp. Hepat. (Stephani) 6: 428, 1923 (Stephani 1923). ^11^

* Anthoceros parvifrons Steph., 52 (5): 307, 1912 (Stephani 1912a). ^12^

* Anthoceros pseudocostus Steph., Sp. Hepat. (Stephani) 5: 977, 1916 (Stephani 1916b). ^13^

* Anthoceros rossoi Gola, Mem. Reale Accad. Sci. Torino (ser. 2) 65 (1): 10, 1916 (Gola 1916). ^14^

*** **Folioceros D.C.Bharadwaj**, Geophytology 1 (1): 9, 1971 (Bharadwaj 1971).

*** Folioceros amboinensis (Schiffn.) Piippo, Acta Bot. Fenn. 148: 36, 1993 (Piippo 1993b). Bas.: Anthoceros amboinensis Schiffn., Leberm., Forschungsr. Gazelle 4 (4): 45, 1890 (Schiffner 1890).

** Folioceros apiahynus (Steph.) Hässel, Candollea 45 (1): 215, 1990 (Hässel 1990b). Bas.: Anthoceros apiahynus Steph., Sp. Hepat. (Stephani) 5: 999, 1916 (Stephani 1916b).

** Folioceros argillaceus (Steph.) J.C.Villarreal et Cargill, Phytotaxa 208 (1): 93, 2015 (Villarreal et al. 2015). Bas.: Aspiromitus argillaceus Steph., Sp. Hepat. (Stephani) 5: 970, 1916 (Stephani 1916b).

*** Folioceros assamicus D.C.Bharadwaj, Geophytology 1 (1): 10, 1971 (Bharadwaj 1971).

** Folioceros dilatatus (Steph.) J.C.Villarreal et Cargill, Phytotaxa 208 (1): 93, 2015 (Villarreal et al. 2015). Bas.: Anthoceros dilatatus Steph., Bot. Jahrb. Syst. 8 (2): 95, 1886 (Stephani 1886d).

*** Folioceros dixitianus (Mahab.) D.C.Bharadwaj, Geophytology 5 (2): 227, 1975 (Bharadwaj 1975). Bas.: Aspiromitus dixitianus Mahab., Curr. Sci. 10 (12): 532, 1941 (Mahabale 1941).

*** Folioceros fuciformis (Mont.) D.C.Bharadwaj, Geophytology 5 (2): 227, 1975 (Bharadwaj 1975). Bas.: Anthoceros fuciformis Mont., Ann. Sci. Nat. Bot. (sér. 2) 20: 296, 1844 (Montagne 1844b).

*** Folioceros glandulosus (Lehm. et Lindenb.) D.C.Bharadwaj, Geophytology 5 (2): 227, 1975 (Bharadwaj 1975). Bas.: Anthoceros glandulosus Lehm. et Lindenb., Nov. Stirp. Pug. 4: 26, 1832 (Lehmann 1832).

*** Folioceros incurvus (Steph.) D.C.Bharadwaj, Geophytology 5 (2): 227, 1975 (Bharadwaj 1975). Bas.: Anthoceros incurvus Steph., Hedwigia 32 (3): 143, 1893 (Stephani 1893b).

*** Folioceros indicus D.C.Bharadwaj, Geophytology 8 (1): 114, 1978 (Bharadwaj 1978).

*** Folioceros kashyapii S.C.Srivast. et A.K.Asthana, Bryologist 92 (2): 219, 1989 (Srivastava and Asthana 1989).

*** Folioceros mangaloreus (Steph.) D.C.Bharadwaj, Geophytology 5 (2): 227, 1975 (Bharadwaj 1975). Bas.: Aspiromitus mangaloreus Steph., Sp. Hepat. (Stephani) 5: 967, 1916 (Stephani 1916b).

*** Folioceros paliformis D.K.Singh, Bull. Bot. Surv. India 29: 176, 1987 [1989] (Singh 1987b).

*** Folioceros physocladus D.C.Bharadwaj, Geophytology 8 (1): 115, 1978 (Bharadwaj 1978). Based on: Anthoceros physocladus Schiffn. ex Pandé, Proc. Indian Sci. Congr. Assoc. 47 (2): 98, 1960 (Pandé 1960), *nom. inval*.

*** Folioceros pinnilobus (Steph.) D.C.Bharadwaj, Geophytology 5 (2): 227, 1975 (Bharadwaj 1975). Bas.: Anthoceros pinnilobus Steph., Akad. Wiss. Wien, Math.-Naturwiss. Kl., Denkschr. 81: 299, 1907 (Stephani 1907a).

*** Folioceros satpurensis D.C.Bharadwaj et K.P.Srivast., Geophytology 8 (1): 112, 1978 (Bharadwaj 1978). Based on: Anthoceros satpurensis K.P.Srivast., Proc. Indian Sci. Congr. Assoc. 47 (3): 337, 1960 (Srivastava 1960), *nom. inval*.

*** Folioceros udarii A.K.Asthana et S.C.Srivast., Cryptog. Bryol. Lichénol. 7 (2): 151, 1986 (Asthana and Srivastava 1986).

*** Folioceros verruculosus (J.Haseg.) R.L.Zhu et M.J.Lai, Ann. Bot. Fenn. 48 (5): 383, 2011 (Wang et al. 2011). Bas.: Anthoceros verruculosus J.Haseg., Acta Phytotax. Geobot. 44 (2): 103, 1993 (Hasegawa 1993b).

##### Dendrocerotidae R.J.Duff, J.C.Villarreal, Cargill et Renzaglia

###### Dendrocerotales Hässel

####### *** Dendrocerotaceae J.Haseg.

by J.C. Villarreal and D.C. Cargill

*** **Dendroceros Nees**, Syn. Hepat. 4: 579, 1846 (Gottsche et al. 1846).

** **subg. Apoceros R.M.Schust.**, Phytologia 63 (3): 200, 1987 (Schuster 1987b).

*** Dendroceros cavernosus J.Haseg., J. Hattori Bot. Lab. 47: 306, 1980 (Hasegawa 1980).

*** Dendroceros difficilis Steph., Sp. Hepat. (Stephani) 5: 1009, 1917 (Stephani 1917b).

*** Dendroceros muelleri Steph., Hedwigia 28 (2): 133, 1889 (Stephani 1889a).

*** Dendroceros ogeramnangus Piippo, Acta Bot. Fenn. 148: 40, 1993 (Piippo 1993b).

*** Dendroceros pedunculatus Steph., Sitzungsber. Naturf. Ges. Leipzig 36: 14, 1909 (Stephani 1909c).

** Dendroceros subdifficilis S.Hatt., Bot. Mag. (Tokyo) 64 (755/756): 119, 1951 (Hattori 1951c).

** **subg. Dendroceros**

*** Dendroceros acutilobus Steph., Sitzungsber. Naturf. Ges. Leipzig 36: 18, 1909 (Stephani 1909c).

*** Dendroceros borbonicus Steph., Bull. Soc. Roy. Bot. Belgique, Compt. Rend. 32 (2): 31, 1893 [1894] (Stephani 1893e).

*** Dendroceros crispus (Sw.) Nees, Syn. Hepat. 4: 581, 1846 (Gottsche et al. 1846). Bas.: Anthoceros crispus Sw., Prodr. (Swartz): 146, 1788 (Swartz 1788).

*** Dendroceros foliicola J.Haseg., J. Hattori Bot. Lab. 47: 296, 1980 (Hasegawa 1980).

*** Dendroceros japonicus Steph., Sitzungsber. Naturf. Ges. Leipzig 36: 15, 1909 (Stephani 1909c).

*** Dendroceros javanicus (Nees) Nees, Syn. Hepat. 4: 582, 1846 (Gottsche et al. 1846). Bas.: Anthoceros javanicus Nees, Enum. Pl. Crypt. Javae: 1, 1830 (Nees 1830).

*** Dendroceros subplanus Steph., Sitzungsber. Naturf. Ges. Leipzig 36: 20, 1909 (Stephani 1909c).

*** Dendroceros tubercularis S.Hatt., Bot. Mag. (Tokyo) 58 (685): 6, 1944 (Hattori 1944b).

*** Dendroceros validus Steph., Sp. Hepat. (Stephani) 5: 1016, 1917 (Stephani 1917b).

** Dendroceros vesconianus Gottsche, J. Bot. (Morot) 12: 150, 1898 (Bescherelle 1898).

** Dendroceros wattsianus Steph., Sitzungsber. Naturf. Ges. Leipzig 36: 17, 1909 (Stephani 1909c).

***Incertae sedis***

* Dendroceros adglutinatus (Hook.f. et Taylor) Gottsche, Lindenb. et Nees, Syn. Hepat. 4: 580, 1846 (Gottsche et al. 1846). Bas.: Monoclea adglutinata Hook.f. et Taylor, London J. Bot. 4: 96, 1845 (Hooker and Taylor 1845). ^15^

*** Dendroceros africanus Steph., Sitzungsber. Naturf. Ges. Leipzig 36: 18, 1909 (Stephani 1909c).

** Dendroceros allionii Steph., Sp. Hepat. (Stephani) 5: 1014, 1917 (Stephani 1917b).

** Dendroceros australis Steph., Sitzungsber. Naturf. Ges. Leipzig 36: 17, 1909 (Stephani 1909c).

** Dendroceros breutelii Nees, Syn. Hepat. 4: 581, 1846 (Gottsche et al. 1846).

** Dendroceros breutelii var. surinamensis Lindenb. et Gottsche, Linnaea 24 (6): 639, 1851 [1852] (Lindenberg and Gottsche 1851a).

*** Dendroceros cichoraceus (Mont.) Gottsche, Bot. Zeitung (Berlin) Beil. 16: 16, 1858 (Gottsche 1858). Bas.: Anthoceros cichoraceus Mont., Ann. Sci. Nat. Bot. (sér. 3) 4: 355, 1845 (Montagne 1845b).

* Dendroceros crassicostatus Steph., Sp. Hepat. (Stephani) 5: 1015, 1917 (Stephani 1917b).

** Dendroceros crassinervis (Nees) Gottsche, Bot. Zeitung (Berlin) Beil. 16: 18, 1858 (Gottsche 1858). Bas.: Anthoceros crassinervis Nees, Syn. Hepat. 4: 589, 1846 (Gottsche et al. 1846).

*** Dendroceros crispatus (Hook.) Nees, Syn. Hepat. 4: 579, 1846 (Gottsche et al. 1846). Bas.: Monoclea crispata Hook., Bot. Misc. 1: 117, 1830 (Hooker 1830).

** Dendroceros crispatus var. simplicior Spruce, Trans. & Proc. Bot. Soc. Edinburgh 15: 574, 1885 (Spruce 1885).

** Dendroceros cucullatus Steph., Sp. Hepat. (Stephani) 6: 429, 1923 (Stephani 1923). ^16^

* Dendroceros exalatus Steph., Sitzungsber. Naturf. Ges. Leipzig 36: 14, 1909 (Stephani 1909c). ^17^

* Dendroceros gracilis Steph., Sp. Hepat. (Stephani) 5: 1015, 1917 (Stephani 1917b).

*** Dendroceros granulatus Mitt., Fl. vit.: 419, 1871 [1873] (Mitten 1871).

** Dendroceros herasii M.Infante, J. Bryol. 32 (4): 285, 2010 (Infante 2010).

** Dendroceros humboldtensis Hürl., Bauhinia 1 (3): 254, 1960 (Hürlimann 1960).

** Dendroceros paivae C.A.Garcia, Sérgio et J.C.Villarreal, Cryptog. Bryol. 33 (1): 5, 2012 (Garcia et al. 2012).

* Dendroceros rarus Steph., Sp. Hepat. (Stephani) 5: 1014, 1917 (Stephani 1917b).

* Dendroceros reticulus Herzog, Memoranda Soc. Fauna Fl. Fennica 26: 37, 1950 [1951] (Herzog 1950b). ^18^

** Dendroceros rigidus Steph., Sp. Hepat. (Stephani) 5: 1017, 1917 (Stephani 1917b).

*** Dendroceros seramensis J.Haseg., Acta Phytotax. Geobot. 37 (1/3): 10, 1986 (Hasegawa 1986a).

** Dendroceros subtropicus C.J.Wild, Trans. Nat. Hist. Soc. Queensland 1: 49, 1893 (Wild 1893).

** Dendroceros tahitensis Ångstr., Öfvers. Kongl. Vetensk.-Akad. Förh. 30 (5): 138, 1873 (Ångström 1873).

*** **Megaceros Campb.**, Ann. Bot. (Oxford) 21 (4): 484, 1907 (Campbell 1907).

** Megaceros aneuriformis Steph., Sp. Hepat. (Stephani) 5: 949, 1916 (Stephani 1916b).

*** Megaceros austronesophilus Cargill et Seppelt, Austral. Syst. Bot. 26 (5): 372, 2013 (Cargill et al. 2013a).

** Megaceros ciliatus K.I.Goebel, Ann. Jard. Bot. Buitenzorg 39: 74, 1928 (Goebel 1928).

*** Megaceros denticulatus (Lehm.) Steph., Sp. Hepat. (Stephani) 5: 956, 1916 (Stephani 1916b). Bas.: Anthoceros denticulatus Lehm., Nov. Stirp. Pug. 10: 25, 1857 (Lehmann 1857).

*** Megaceros flagellaris (Mitt.) Steph., Sp. Hepat. (Stephani) 5: 951, 1916 (Stephani 1916b). Bas.: Anthoceros flagellaris Mitt., Fl. vit.: 419, 1871 [1873] (Mitten 1871).

*** Megaceros gracilis (Reichardt) Steph., Sp. Hepat. (Stephani) 5: 955, 1916 (Stephani 1916b). Bas.: Anthoceros gracilis Reichardt, Verh. K.K. Zool.-Bot. Ges. Wien 16: 957, 1866 (Reichardt 1866).

** Megaceros leptohymenius (Hook.f. et Taylor) Steph., Sp. Hepat. (Stephani) 5: 955, 1916 (Stephani 1916b). Bas.: Monoclea leptohymenia Hook.f. et Taylor, London J. Bot. 3: 575, 1844 (Hooker and Taylor 1844d).

*** Megaceros pellucidus (Colenso) E.A.Hodgs., J. Roy. Soc. New Zealand 2 (1): 115, 1972 (Hodgson 1972). Bas.: Anthoceros pellucidus Colenso, Trans. & Proc. New Zealand Inst. 17: 263, 1885 (Colenso 1885).

** Megaceros tjibodensis Campb., Ann. Bot. (Oxford) 21 (4): 484, 1907 (Campbell 1907).

**Excluded from the genus** ^19^

* Megaceros flavens (Spruce) Campb., Ann. Bot. (Oxford) 21 (4): 483, 1907 (Campbell 1907). Bas.: Anthoceros flavens Spruce, Trans. & Proc. Bot. Soc. Edinburgh 15: 575, 1885 (Spruce 1885).

* Megaceros jamesonii (Taylor) Steph., Biblioth. Bot. 87 (2): 268, 1916 (Stephani 1916a). Bas.: Dendroceros jamesonii Taylor, London J. Bot. 7: 285, 1848 (Taylor 1848b).

*** **Nothoceros (R.M.Schust.) J.Haseg.**, J. Hattori Bot. Lab. 76: 32, 1994 (Hasegawa 1994b). Bas.: Megaceros subg. Nothoceros R.M.Schust., Phytologia 63 (3): 200, 1987 (Schuster 1987b).

*** Nothoceros aenigmaticus J.C.Villarreal et K.D.McFarland, Bryologist 113 (1): 109, 2010 (Villarreal et al. 2010b). Based on: Megaceros aenigmaticus R.M.Schust., Hepat. Anthocerotae N. Amer. 6: 830, 1992 (Schuster 1992d), *nom. inval*.

*** Nothoceros canaliculatus (Pagán) J.C.Villarreal, Hässel et N.Salazar, Bryologist 110 (2): 283, 2007 (Villarreal et al. 2007). Bas.: Dendroceros canaliculatus Pagán, Bryologist 45 (4): 111, 1942 (Pagán 1942a).

*** Nothoceros endiviifolius (Mont.) J.Haseg. ex J.C.Villarreal, Hässel et N.Salazar, Bryologist 110 (2): 283, 2007 (Villarreal et al. 2007). Bas.: Anthoceros endiviifolius Mont., Voy. Pole Sud, Bot. 1: 211, 1845 (Montagne 1845c).

** Nothoceros fuegiensis (Steph.) J.C.Villarreal, Bryologist 113 (1): 109, 2010 (Villarreal et al. 2010b). Bas.: Megaceros fuegiensis Steph., Kungl. Svenska Vetensk.-Akad. Handl. (n.ser.) 46 (9): 91, 1911 (Stephani 1911b).

*** Nothoceros giganteus (Lehm. et Lindenb.) J.Haseg. ex J.C.Villarreal, Hässel et N.Salazar, Bryologist 110 (2): 283, 2007 (Villarreal et al. 2007). Bas.: Anthoceros giganteus Lehm. et Lindenb., Nov. Stirp. Pug. 4: 25, 1832 (Lehmann 1832).

*** Nothoceros minarum (Nees) J.C.Villarreal, Molec. Phylogen. Evol. 78: 34, 2014 (Villarreal and Renner 2014). Bas.: Anthoceros minarum Nees, Naturgesch. Eur. Leberm. 4: 340, 1838 (Nees 1838a).

** Nothoceros renzagliensis J.C.Villarreal, L.V.Campos et Uribe, Syst. Bot. 37 (1): 32, 2012 (Villarreal et al. 2012).

*** Nothoceros schizophyllus (Steph.) J.C.Villarreal, Molec. Phylogen. Evol. 78: 34, 2014 (Villarreal and Renner 2014). Bas.: Megaceros schizophyllus Steph., Sp. Hepat. (Stephani) 5: 949, 1916 (Stephani 1916b).

*** Nothoceros superbus J.C.Villarreal, Hässel et N.Salazar, Bryologist 110 (2): 280, 2007 (Villarreal et al. 2007).

*** Nothoceros vincentianus (Lehm. et Lindenb.) J.C.Villarreal, Bryologist 113 (1): 111, 2010 (Villarreal et al. 2010b). Bas.: Anthoceros vincentianus Lehm. et Lindenb., Nov. Stirp. Pug. 6: 16, 1834 (Lehmann 1834).

** **Phaeomegaceros R.J.Duff, J.C.Villarreal, Cargill et Renzaglia**, Bryologist 110 (2): 241, 2007 (Duff et al. 2007).

*** Phaeomegaceros coriaceus (Steph.) R.J.Duff, J.C.Villarreal, Cargill et Renzaglia, Bryologist 110 (2): 241, 2007 (Duff et al. 2007). Bas.: Anthoceros coriaceus Steph., Sp. Hepat. (Stephani) 5: 991, 1916 (Stephani 1916b).

*** Phaeomegaceros fimbriatus (Gottsche) R.J.Duff, J.C.Villarreal, Cargill et Renzaglia, Bryologist 110 (2): 241, 2007 (Duff et al. 2007). Bas.: Anthoceros fimbriatus Gottsche, Ann. Sci. Nat. Bot. (sér. 5) 1: 187, 1864 (Gottsche 1864).

*** Phaeomegaceros foveatus (J.Haseg.) J.C.Villarreal, Nova Hedwigia 91 (3/4): 352, 2010 (Villarreal et al. 2010a). Bas.: Phaeoceros foveatus J.Haseg., Bryol. Res. 7 (12): 374, 2001 (Hasegawa 2001).

*** Phaeomegaceros hirticalyx (Steph.) R.J.Duff, J.C.Villarreal, Cargill et Renzaglia, Bryologist 110 (2): 241, 2007 (Duff et al. 2007). Bas.: Aspiromitus hirticalyx Steph., Sp. Hepat. (Stephani) 5: 966, 1916 (Stephani 1916b).

*** Phaeomegaceros plicatus (Mitt.) J.C.Villarreal, J.J.Engel et Váňa, Mem. New York Bot. Gard. 105: 85, 2013 (Váňa and Engel 2013). Bas.: Anthoceros plicatus Mitt., Rep. Challenger, Bot. 1 (2): 178, 1884 (Mitten 1884a).

*** Phaeomegaceros skottsbergii (Steph.) R.J.Duff, J.C.Villarreal, Cargill et Renzaglia, Bryologist 110 (2): 241, 2007 (Duff et al. 2007). Bas.: Anthoceros skottsbergii Steph., Kungl. Svenska Vetensk.-Akad. Handl. (n.ser.) 46 (9): 90, 1911 (Stephani 1911b).

*** Phaeomegaceros squamuliger (Spruce) J.C.Villarreal, Nova Hedwigia 91 (3/4): 351, 2010 (Villarreal et al. 2010a). Bas.: Anthoceros squamuliger Spruce, Trans. & Proc. Bot. Soc. Edinburgh 15: 576, 1885 (Spruce 1885).

** Phaeomegaceros squamuliger subsp. hasselii J.C.Villarreal, Cargill et Goffinet, Nova Hedwigia 91 (3/4): 352, 2010 (Villarreal et al. 2010a).

###### Phymatocerotales R.J.Duff, J.C.Villarreal, Cargill et Renzaglia

####### *** Phymatocerotaceae R.J.Duff, J.C.Villarreal, Cargill et Renzaglia

by J.C. Villarreal and D.C. Cargill

*** **Phymatoceros Stotler, W.T.Doyle et Crand.-Stotl.**, Phytologia 87 (2): 113, 2005 (Stotler et al. 2005).

*** Phymatoceros bulbiculosus (Brot.) Stotler, W.T.Doyle et Crand.-Stotl., Phytologia 87 (2): 114, 2005 (Stotler et al. 2005). Bas.: Anthoceros bulbiculosus Brot., Fl. lusit. 2: 430, 1804 [1805] (Brotero 1804).

*** Phymatoceros phymatodes (M.Howe) R.J.Duff, J.C.Villarreal, Cargill et Renzaglia, Bryologist 110 (2): 240, 2007 (Duff et al. 2007). Bas.: Anthoceros phymatodes M.Howe, Bull. Torrey Bot. Club 25 (1): 12, 1898 (Howe 1898a).

##### Notothylatidae R.J.Duff, J.C.Villarreal, Cargill et Renzaglia

###### Notothyladales Hyvönen et Piippo

####### *** Notothyladaceae Müll.Frib. ex Prosk.

by J.C. Villarreal and D.C. Cargill

######## ** Notothyladoideae Grolle

*** **Notothylas Sull. ex A.Gray**, Amer. J. Sci. Arts (ser. 2) 1 (1): 74, 1846 (Gray 1846).

** **subg. Notothylas**

*** Notothylas anaporata Udar et D.K.Singh, Rev. Bryol. Lichénol. 45 (2): 202, 1979 (Udar and Singh 1979b).

*** Notothylas breutelii (Gottsche) Gottsche, Bot. Zeitung (Berlin) Beil. 16: 21, 1858 (Gottsche 1858). Bas.: Anthoceros breutelii Gottsche, Syn. Hepat. 4: 583, 1846 (Gottsche et al. 1846).

*** Notothylas depressispora J.Haseg., Acta Phytotax. Geobot. 30 (1/3): 26, 1979 (Hasegawa 1979).

*** Notothylas dissecta Steph., Sp. Hepat. (Stephani) 5: 1020, 1917 (Stephani 1917b).

*** Notothylas himalayensis Udar et D.K.Singh, J. Bryol. 11 (3): 451, 1981 (Udar and Singh 1981a).

*** Notothylas indica Kashyap, Proc. Lahore Philos. Soc. 4: 54, 1925 (Kashyap and Dutt 1925).

*** Notothylas javanica (Sande Lac.) Gottsche, Bot. Zeitung (Berlin) Beil. 16: 20, 1858 (Gottsche 1858). Bas.: Blasia javanica Sande Lac., Syn. hepat. jav.: 94, 1856 [1857] (Sande Lacoste 1856b).

*** Notothylas orbicularis (Schwein.) Sull., Amer. J. Sci. Arts (ser. 2) 1 (1): 75, 1846 (Gray 1846). Bas.: Targionia orbicularis Schwein., Spec. Fl. Amer. Crypt.: 23, 1821 (Schweinitz 1821).

*** Notothylas pandei Udar et V.Chandra, Geophytology 7 (2): 142, 1977 (Udar and Chandra 1977).

** **subg. Notothyloides A.K.Asthana et S.C.Srivast.**, Bryophyt. Biblioth. 42: 106, 1991 (Asthana and Srivastava 1991).

*** Notothylas khasiana Udar et D.K.Singh, J. Indian Bot. Soc. 60: 112, 1981 (Udar and Singh 1981b).

*** Notothylas pfleidereri Udar et D.K.Singh, Lindbergia 5 (1): 28, 1979 (Udar and Singh 1979a).

***Incertae sedis***

*** Notothylas decurva (Mitt.) Steph., Cat. Afr. Pl. (Hiern) 2 (2): 320, 1901 (Stephani 1901d). Bas.: Anthoceros decurvus Mitt., Trans. Linn. Soc. London 23 (1): 58, 1860 (Mitten 1860a).

*** Notothylas flabellata Steph., Cat. Afr. Pl. (Hiern) 2 (2): 320, 1901 (Stephani 1901d).

*** Notothylas galapagensis M.Howe, Proc. Calif. Acad. Sci. (ser. 4) 21 (17): 203, 1934 (Howe 1934).

** Notothylas irregularis Chantanaorr., Acta Bot. Hung. 56 (3/4): 270, 2014 (Chantanaorrapint 2014).

*** Notothylas kashyapii D.K.Singh, Indian J. Forest. 23 (4): 386, 2000 (Singh and Semwal 2000).

*** Notothylas nepalensis D.K.Singh, J. Bombay Nat. Hist. Soc. 84 (3): 650, 1987 [1988] (Singh 1987a).

*** Notothylas temperata J.Haseg., Acta Phytotax. Geobot. 30 (1/3): 20, 1979 (Hasegawa 1979).

*** Notothylas udarii D.K.Singh et Semwal, Phytotaxonomy 1: 35, 2001 (Singh and Semwal 2001).

* Notothylas verdoornii Khanna, Rev. Bryol. Lichénol. 6: 118, 1933 (Khanna 1933).

*** Notothylas vitalii Udar et D.K.Singh, Misc. Bryol. Lichenol. 8 (9): 173, 1980 (Udar and Singh 1980).

** Notothylas yunnanensis T.Peng et R.L.Zhu, Phytotaxa 156 (3): 157, 2014 (Peng and Zhu 2014).

######## ** Phaeocerotoideae Hässel

* **Mesoceros Piippo**, Acta Bot. Fenn. 148: 30, 1993 (Piippo 1993b). ^20^

* Mesoceros mesophoros Piippo, Acta Bot. Fenn. 148: 30, 1993 (Piippo 1993b).

* Mesoceros porcatus Piippo, Haussknechtia, Beih. 9: 279, 1999 (Piippo 1999).

** **Paraphymatoceros Hässel**, Phytologia 88 (2): 208, 2006 (Hässel 2006a).

*** Paraphymatoceros diadematus Hässel, Phytologia 88 (2): 209, 2006 (Hässel 2006a).

*** Paraphymatoceros hallii (Austin) Hässel, Phytologia 88 (2): 209, 2006 (Hässel 2006a). Bas.: Anthoceros hallii Austin, Bull. Torrey Bot. Club 6 (4): 26, 1875 [1876] (Austin 1875a).

*** Paraphymatoceros pearsonii (M.Howe) J.C.Villarreal et Cargill, Phytotaxa 208 (1): 94, 2015 (Villarreal et al. 2015). Bas.: Anthoceros pearsonii M.Howe, Bull. Torrey Bot. Club 25 (1): 8, 1898 (Howe 1898a).

*** Paraphymatoceros proskaueri (Stotler, Crand.-Stotl. et W.T.Doyle) J.C.Villarreal et Cargill, Phytotaxa 208 (1): 94, 2015 (Villarreal et al. 2015). Bas.: Phaeoceros proskaueri Stotler, Crand.-Stotl. et W.T.Doyle, Fieldiana, Bot. (n.ser.) 47: 232, 2008 (Crandall-Stotler et al. 2008a).

*** **Phaeoceros Prosk.**, Bull. Torrey Bot. Club 78 (4): 346, 1951 (Proskauer 1951a).

** Phaeoceros austroandinus Hässel, Candollea 44 (2): 721, 1989 (Hässel 1989a).

** Phaeoceros bolusii (Sim) S.W.Arnell, Hepat. South Africa: 401, 1963 (Arnell 1963b). Bas.: Anthoceros bolusii Sim, Trans. Roy. Soc. South Africa 15 (1): 114, 1926 (Sim 1926).

** Phaeoceros brevicapsulus (Steph.) Hässel, Candollea 44 (2): 725, 1989 (Hässel 1989a). Bas.: Anthoceros brevicapsulus Steph., Sp. Hepat. (Stephani) 5: 981, 1916 (Stephani 1916b).

*** Phaeoceros carolinianus (Michx.) Prosk., Bull. Torrey Bot. Club 78 (4): 347, 1951 (Proskauer 1951a). Bas.: Anthoceros carolinianus Michx., Fl. bor.-amer. (Michaux) 2: 280, 1803 (Michaux 1803).

*** Phaeoceros delicatus E.O.Campb. et Outred, New Zealand J. Bot. 33 (3): 285, 1995 (Campbell and Outred 1995).

*** Phaeoceros dendroceroides (Steph.) Hässel, Beih. Nova Hedwigia 134: 487, 2009 (Hässel and Rubies 2009). Bas.: Anthoceros dendroceroides Steph., Sp. Hepat. (Stephani) 5: 984, 1916 (Stephani 1916b). ^21^

*** Phaeoceros engelii Cargill et Fuhrer, Fieldiana, Bot. (n.ser.) 47: 248, 2008 (Cargill and Fuhrer 2008).

** Phaeoceros erectus Udar et D.K.Singh, Geophytology 11 (2): 257, 1981 (Udar and Singh 1981c). *Nom. nov. pro Anthoceros erectus* Steph., Sp. Hepat. (Stephani) 5: 984, 1916 (Stephani 1916b), *nom. illeg*.

*** Phaeoceros evanidus (Steph.) Cargill et Fuhrer, Fieldiana, Bot. (n.ser.) 47: 245, 2008 (Cargill and Fuhrer 2008). Bas.: Anthoceros evanidus Steph., Sp. Hepat. (Stephani) 5: 990, 1916 (Stephani 1916b).

*** Phaeoceros exiguus (Steph.) J.Haseg., J. Hattori Bot. Lab. 60: 387, 1986 (Hasegawa 1986b). Bas.: Anthoceros exiguus Steph., Sp. Hepat. (Stephani) 5: 988, 1916 (Stephani 1916b).

*** Phaeoceros flexivalvis (Gottsche et Nees) Hässel, Candollea 44 (2): 728, 1989 (Hässel 1989a). Bas.: Anthoceros flexivalvis Gottsche et Nees, Syn. Hepat. 4: 586, 1846 (Gottsche et al. 1846).

*** Phaeoceros fulvisporus (Steph.) J.Haseg., Trop. Bryol. 8: 52, 1993 (Hasegawa 1993a). Bas.: Anthoceros fulvisporus Steph., 52 (5): 306, 1912 (Stephani 1912a).

*** Phaeoceros gemmifer (Horik.) J.Haseg., J. Hattori Bot. Lab. 57: 252, 1984 (Hasegawa 1984). Bas.: Anthoceros gemmifer Horik., Sci. Rep. Tôhoku Imp. Univ., Ser. 4, Biol. 4 (2): 426, 1929 (Horikawa 1929a).

** Phaeoceros gualaquizanus (Steph.) Gradst., J. Hattori Bot. Lab. 45: 123, 1979 (Gradstein and Hekking 1979). Bas.: Anthoceros gualaquizanus Steph., Sp. Hepat. (Stephani) 5: 979, 1916 (Stephani 1916b).

*** Phaeoceros himalayensis (Kashyap) Prosk. ex Bapna et G.G.Vyas, J. Hattori Bot. Lab. 25: 88, 1962 (Bapna and Vyas 1962). Bas.: Anthoceros himalayensis Kashyap, New Phytol. 14 (1): 8, 1915 (Kashyap 1915).

*** Phaeoceros huebschmannii Hässel, Veröff. Geobot. Inst. ETH Stiftung Rübel Zürich 91: 301, 1986 (Hässel 1986b).

*** Phaeoceros inflatus (Steph.) Cargill et Fuhrer, Fieldiana, Bot. (n.ser.) 47: 246, 2008 (Cargill and Fuhrer 2008). Bas.: Anthoceros inflatus Steph., Sp. Hepat. (Stephani) 5: 990, 1916 (Stephani 1916b).

*** Phaeoceros kashyapii A.K.Asthana et S.C.Srivast., Bryophyt. Biblioth. 42: 129, 1991 (Asthana and Srivastava 1991).

*** Phaeoceros laevis (L.) Prosk., Bull. Torrey Bot. Club 78 (4): 347, 1951 (Proskauer 1951a). Bas.: Anthoceros laevis L., Sp. Pl. 1: 1139, 1753 (Linnaeus 1753).

** Phaeoceros maranguensis (Steph.) Bapna, Univ. Udaipur Res. Stud. 3: 138, 1966 (Bapna 1966). Bas.: Aspiromitus maranguensis Steph., Sp. Hepat. (Stephani) 5: 960, 1916 (Stephani 1916b).

* Phaeoceros microsporus (Steph.) Hässel, Candollea 44 (2): 721, 1989 (Hässel 1989a). Bas.: Aspiromitus microsporus Steph., Sp. Hepat. (Stephani) 5: 963, 1916 (Stephani 1916b). ^22^

*** Phaeoceros minutus (Mitt.) S.W.Arnell, Hepat. South Africa: 403, 1963 (Arnell 1963b). Bas.: Anthoceros minutus Mitt., J. Linn. Soc., Bot. 16 (91): 195, 1877 (Mitten 1877).

*** Phaeoceros mohrii (Austin) Hässel, Candollea 44 (2): 721, 1989 (Hässel 1989a). Bas.: Anthoceros mohrii Austin, Bull. Torrey Bot. Club 6 (52): 304, 1879 (Austin 1879).

*** Phaeoceros oreganus (Austin) Hässel, Candollea 44 (2): 718, 1989 (Hässel 1989a). Bas.: Anthoceros oreganus Austin, Bull. Torrey Bot. Club 6 (4): 26, 1875 [1876] (Austin 1875a).

*** Phaeoceros parvulus (Schiffn.) J.Haseg., J. Hattori Bot. Lab. 57: 248, 1984 (Hasegawa 1984). Bas.: Anthoceros parvulus Schiffn., Österr. Bot. Z. 49 (11): 391, 1899 (Schiffner 1899c).

*** Phaeoceros perpusillus Chantanaorr., Acta Bot. Hung. 51 (1/2): 30, 2009 (Chantanaorrapint 2009).

** Phaeoceros pichinchensis (Spruce) Hässel, Candollea 44 (2): 725, 1989 (Hässel 1989a). Bas.: Anthoceros pichinchensis Spruce, Trans. & Proc. Bot. Soc. Edinburgh 15: 577, 1885 (Spruce 1885).

** Phaeoceros propagulifer (Steph.) Prosk., Bull. Torrey Bot. Club 78 (4): 347, 1951 (Proskauer 1951a). Bas.: Anthoceros propagulifer Steph., Sp. Hepat. (Stephani) 5: 1001, 1916 (Stephani 1916b).

* Phaeoceros striatisporus J.Haseg., J. Hattori Bot. Lab. 75, 268 (Hasegawa 1994a).^23^

*** Phaeoceros tenuis (Spruce) Hässel, Veröff. Geobot. Inst. ETH Stiftung Rübel Zürich 91: 303, 1986 (Hässel 1986b). Bas.: Anthoceros tenuis Spruce, Bull. Soc. Bot. France (Congr. Bot.) 36: cxcvi, 1889 [1890] (Spruce 1889).

** Phaeoceros tigrinus (Gola) J.C.Villarreal, Phytotaxa 208 (1): 95, 2015 (Villarreal et al. 2015). Bas.: Anthoceros tigrinus Gola, Ann. Bot. (Rome) 13 (1): 72, 1914 (Gola 1914a).

*** Phaeoceros tuberosus (Taylor) Prosk., J. Indian Bot. Soc. 42A: 185, 1964 (Proskauer 1964). Bas.: Anthoceros tuberosus Taylor, London J. Bot. 5: 412, 1846 (Taylor 1846b).

*** Phaeoceros udarii A.K.Asthana et V.Nath, Proc. Natl. Acad. Sci. India, B 63 (4): 461, 1993 (Asthana and Nath 1993).

** Phaeoceros wrightii (Steph.) Hässel, Candollea 44 (2): 722, 1989 (Hässel 1989a). Bas.: Anthoceros wrightii Steph., Sp. Hepat. (Stephani) 5: 999, 1916 (Stephani 1916b).

#### Leiosporocerotopsida Stotler et Crand.-Stotl.

##### Leiosporocerotales Hässel

###### *** Leiosporocerotaceae Hässel ex Ochyra

by J.C. Villarreal and D.C. Cargill

*** **Leiosporoceros Hässel**, J. Bryol. 14 (2): 255, 1986 (Hässel 1986a).

*** Leiosporoceros dussii (Steph.) Hässel, J. Bryol. 14 (2): 255, 1986 (Hässel 1986a). Bas.: Anthoceros dussii Steph., Hedwigia 32 (3): 142, 1893 (Stephani 1893b).

### Marchantiophyta

#### Haplomitriopsida Stotler et Crand.-Stotl.

##### Haplomitriidae Stotler et Crand.-Stotl.

###### Calobryales Hamlin

####### *** Haplomitriaceae Dědeček

by S. Bartholomew-Began

The treatment of Haplomitriaceae follows Bartholomew-Began (1991). Morphogenetic and molecular studies support the infrageneric ranks of Haplomitrium (Crandall-Stotler et al. 2009, Forrest et al. 2006).

*** **Haplomitrium Nees**, Naturgesch. Eur. Leberm. 1: 109, 1833 (Nees 1833c) nom. conserv.

*** **subg. Calobryum (Nees) R.M.Schust.**, Nova Hedwigia 13 (1/2): 40, 1967 (Schuster 1967c). Bas.: Calobryum Nees, Syn. Hepat. 4: 507, 1846 (Gottsche et al. 1846).

*** Haplomitrium blumei (Nees) R.M.Schust., J. Hattori Bot. Lab. 26: 225, 1963 (Schuster 1963b). Bas.: Monoclea blumei Nees, Enum. Pl. Crypt. Javae: 2, 1830 (Nees 1830).

*** Haplomitrium mnioides (Lindb.) R.M.Schust., J. Hattori Bot. Lab. 26: 225, 1963 (Schuster 1963b). Bas.: Rhopalanthus mnioides Lindb., Not. Sällsk. Fauna Fl. Fenn. Förh. 13: 391, 1874 (Lindberg 1874a).

*** **subg. Haplomitrium**

** **sect. Archibryum (R.M.Schust.) J.J.Engel**, Ann. Missouri Bot. Gard. 68 (4): 675, 1981 (Engel 1981). Bas.: Haplomitrium subg. Archibryum R.M.Schust., Nova Hedwigia 13 (1/2): 28, 1967 (Schuster 1967c).

*** Haplomitrium gibbsiae (Steph.) R.M.Schust., J. Hattori Bot. Lab. 26: 225, 1963 (Schuster 1963b). Bas.: Calobryum gibbsiae Steph., Sp. Hepat. (Stephani) 6: 76, 1917 (Stephani 1917a).

*** Haplomitrium intermedium Berrie, Proc. Linn. Soc. New South Wales (ser. 2) 87 (399): 191, 1963 (Berrie 1963).

** **sect. Haplomitrium**

*** Haplomitrium hookeri (Lyell ex Sm.) Nees, Naturgesch. Eur. Leberm. 1: 111, 1833 (Nees 1833c). Bas.: Jungermannia hookeri Lyell ex Sm., Engl. Bot. 35: tab. 2555, 1814 (Smith and Sowerby 1814).

*** Haplomitrium hookeri var. minutum (E.O.Campb.) Barthol.-Began, Bryophyt. Biblioth. 41: 230, 1991 (Bartholomew-Began 1991). Bas.: Steereomitrium minutum E.O.Campb., Mem. New York Bot. Gard. 45: 569, 1987 (Campbell 1987).

*** Haplomitrium monoicum J.J.Engel, Ann. Missouri Bot. Gard. 68 (4): 668, 1981 [1982] (Engel 1981).

*** Haplomitrium ovalifolium R.M.Schust., Bryologist 74 (2): 136, 1971 (Schuster 1971d).

##### Treubiidae Stotler et Crand.-Stotl.

###### Treubiales Schljakov

####### *** Treubiaceae Verd.

by R. Stotler and B.J. Crandall-Stotler

Stech et al. (2002) showed a clear molecular distinction between Treubia and Apotreubia, which is in accordance with their morphological differences and supports their recognition as separate genera.

*** **Apotreubia S.Hatt. et Mizut.**, Bryologist 69 (4): 491, 1966 [1967] (Hattori et al. 1966).

*** Apotreubia hortoniae Konstant., Phytotaxa 76 (3): 33, 2013 (Konstantinova et al. 2013b). Based on: Apotreubia hortoniae R.M.Schust. et Konstant., J. Hattori Bot. Lab. 78: 55, 1995 (Schuster and Konstantinova 1995), *nom. inval*.

*** Apotreubia nana (S.Hatt. et Inoue) S.Hatt. et Mizut., Bryologist 69 (4): 492, 1966 [1967] (Hattori et al. 1966). Bas.: Treubia nana S.Hatt. et Inoue, J. Hattori Bot. Lab. 11: 99, 1954 (Hattori and Inoue 1954).

** Apotreubia pusilla (R.M.Schust.) Grolle, Acta Bot. Fenn. 125: 63, 1984 (Grolle and Piippo 1984). Bas.: Treubia pusilla R.M.Schust., Nova Hedwigia 15: 515, 1968 (Schuster 1968b).

*** Apotreubia yunnanensis Higuchi, Cryptog. Bryol. Lichénol. 19 (4): 321, 1998 (Higuchi 1998).

*** **Treubia K.I.Goebel**, Ann. Jard. Bot. Buitenzorg 9 (1): 1, 1890 [1891] (Goebel 1890) nom. conserv.

*** Treubia insignis K.I.Goebel, Ann. Jard. Bot. Buitenzorg 9 (1): 1, 1890 [1891] (Goebel 1890).

** Treubia insignis subsp. bracteata (Steph.) R.M.Schust. et G.A.M.Scott, J. Hattori Bot. Lab. 32: 241, 1969 (Schuster and Scott 1969). Bas.: Treubia bracteata Steph., Bot. Jahrb. Syst. 23 (1/2, 3): 302, 1896 (Stephani 1896a).

** Treubia insignis subsp. caledonica R.M.Schust. et G.A.M.Scott, J. Hattori Bot. Lab. 32: 243, 1969 (Schuster and Scott 1969).

** Treubia insignis subsp. vitiensis R.M.Schust. et G.A.M.Scott, J. Hattori Bot. Lab. 32: 242, 1969 (Schuster and Scott 1969).

*** Treubia lacunosa (Colenso) Prosk., Bryologist 58 (3): 199, 1955 (Proskauer 1955). Bas.: Noteroclada lacunosa Colenso, Trans. & Proc. New Zealand Inst. 18: 248, 1886 (Colenso 1886b).

** Treubia lacunosoides T.Pfeiff., W.Frey et M.Stech, Nova Hedwigia 75 (1/2): 249, 2002 (Pfeiffer et al. 2002).

*** Treubia pygmaea R.M.Schust., Phytologia 56 (7): 460, 1985 (Schuster 1985c).

*** Treubia scapanioides R.M.Schust., J. Hattori Bot. Lab. 32: 246, 1969 (Schuster and Scott 1969).

*** Treubia tahitensis (Nadeaud) Besch., J. Bot. (Morot) 12: 147, 1898 (Bescherelle 1898). Bas.: Gottschea tahitensis Nadeaud, Énum. Pl. Tahiti: 7, 1873 (Nadeaud 1873).

*** Treubia tasmanica R.M.Schust. et G.A.M.Scott, J. Hattori Bot. Lab. 32: 248, 1969 (Schuster and Scott 1969).

#### Jungermanniopsida Stotler et Crand.-Stotl.

##### Jungermanniidae Engl.

###### Jungermanniales H.Klinggr.

####### Cephaloziineae Schljakov

######## *** Adelanthaceae Grolle

by K. Feldberg, J. Váňa and J. Heinrichs

The subfamily Jamesonielloideae was excluded from Lophoziaceae/Scapaniaceae by De Roo et al. (2007) and this status was confirmed by e.g. Vilnet et al. (2010). The family was described and defined by Feldberg et al. (2010a). Some taxonomic and nomenclatural notes can also be found in Feldberg et al. (2010b, 2011), Váňa et al. (2014d).

*** Adelanthoideae K.Feldberg, Heinrichs et Váňa

*** **Adelanthus Mitt.**, J. Proc. Linn. Soc., Bot. 7 (28): 243, 1864 (Mitten 1864b) nom. conserv.

** **sect. Adelanthus**

*** Adelanthus falcatus (Hook.) Mitt., J. Proc. Linn. Soc., Bot. 7 (28): 243, 1864 (Mitten 1864b). Bas.: Jungermannia falcata Hook., Musci Exot. 1: tab. 89, 1818 (Hooker 1818).

*** Adelanthus occlusus (Hook.f. et Taylor) Carrington, Trans. Bot. Soc. Edinburgh 10: 381, 1870 (Carrington 1870). Bas.: Jungermannia occlusa Hook.f. et Taylor, London J. Bot. 3: 369, 1844 (Hooker and Taylor 1844a).

** **sect. Calyptrocolea (R.M.Schust.) Grolle**, J. Hattori Bot. Lab. 35: 331, 1972 (Grolle 1972c). Bas.: Calyptrocolea R.M.Schust., Rev. Bryol. Lichénol. 34 (3/4): 685, 1966 (Schuster 1966a).

*** Adelanthus aureomarginatus R.M.Schust., Phytologia 39 (4): 250, 1978 (Schuster 1978a).

*** Adelanthus gemmiparus (R.M.Schust.) E.A.Hodgs., Trans. Roy. Soc. New Zealand, Biol. Sci. 11 (18): 241, 1970 (Hodgson 1970). Bas.: Calyptrocolea gemmipara R.M.Schust., Rev. Bryol. Lichénol. 34 (3/4): 695, 1966 [1967] (Schuster 1966a).

** Adelanthus lingulatus J.J.Engel et Váňa, Mem. New York Bot. Gard. 105: 26, 2013 (Váňa and Engel 2013).

*** Adelanthus tenuis J.J.Engel et Grolle, J. Hattori Bot. Lab. 35: 333, 1972 (Grolle 1972c).

** **sect. Lindenbergiani Grolle**, J. Hattori Bot. Lab. 35: 331, 1972 (Grolle 1972c).

*** Adelanthus carabayensis (Mont.) Grolle, J. Hattori Bot. Lab. 35: 348, 1972 (Grolle 1972c). Bas.: Plagiochila carabayensis Mont., Ann. Sci. Nat. Bot. (sér. 4) 5: 348, 1856 (Montagne 1856c).

*** Adelanthus integerrimus Grolle, J. Hattori Bot. Lab. 35: 340, 1972 (Grolle 1972c).

*** Adelanthus lindenbergianus (Lehm.) Mitt., J. Proc. Linn. Soc., Bot. 7 (28): 244, 1864 (Mitten 1864b). Bas.: Jungermannia lindenbergiana Lehm., Linnaea 4: 367, 1829 (Lehmann 1829).

** **sect. Pittieri Grolle**, J. Hattori Bot. Lab. 35: 331, 1972 (Grolle 1972c).

*** Adelanthus pittieri (Steph.) Grolle, J. Hattori Bot. Lab. 35: 337, 1972 (Grolle 1972c). Bas.: Tylimanthus pittieri Steph., Sp. Hepat. (Stephani) 6: 250, 1922 (Stephani 1922).

*** Adelanthus squarrosus Grolle, J. Hattori Bot. Lab. 67: 243, 1989 (Grolle 1989d).

*** **Pseudomarsupidium Herzog**, Svensk Bot. Tidskr. 47 (1): 42, 1953 (Herzog 1953b).

*** Pseudomarsupidium aureocinctum (R.M.Schust.) J.J.Engel, Novon 17 (3): 312, 2007 (Engel 2007). Bas.: Adelanthus decipiens subsp. aureocinctus R.M.Schust., Phytologia 39 (4): 250, 1978 (Schuster 1978a).

*** Pseudomarsupidium borneensis (Grolle) Váňa, L.Söderstr., A.Hagborg et von Konrat, Phytotaxa 65: 60, 2012 (Váňa et al. 2012f). Bas.: Adelanthus borneensis Grolle, J. Hattori Bot. Lab. 35: 362, 1972 (Grolle 1972c).

*** Pseudomarsupidium decipiens (Hook.) Grolle, Trans. Brit. Bryol. Soc. 4 (3): 443, 1963 (Grolle 1963a). Bas.: Jungermannia decipiens Hook., Brit. Jungermann.: tab. 50, 1813 (Hooker 1813).

*** Pseudomarsupidium piliferum (Steph.) Herzog ex Grolle, Trans. Brit. Bryol. Soc. 4 (3): 443, 1963 (Grolle 1963a). Bas.: Marsupidium piliferum Steph., Bull. Herb. Boissier (sér. 2) 8 (8): 602 (386), 1908 (Stephani 1908e).

*** **Wettsteinia Schiffn.**, Ann. Jard. Bot. Buitenzorg, suppl. 2: 44, 1898 (Schiffner 1898c).

*** Wettsteinia densiretis (Herzog) Grolle, J. Hattori Bot. Lab. 28: 99, 1965 (Grolle 1965f). Bas.: Tylimanthus densiretis Herzog, Nat. Hist. Juan Fernandez (Botany) 2 (5): 712, 1942 (Herzog 1942a).

*** Wettsteinia inversa (Sande Lac.) Schiffn., Ann. Jard. Bot. Buitenzorg, suppl. 2: 45, 1898 (Schiffner 1898c). Bas.: Plagiochila inversa Sande Lac., Ann. Mus. Bot. Lugduno-Batavi 1: 289, 1864 (Sande Lacoste 1864).

*** Wettsteinia rotundifolia (Horik.) Grolle, J. Hattori Bot. Lab. 28: 100, 1965 (Grolle 1965f). Bas.: Adelanthus rotundifolius Horik., J. Sci. Hiroshima Univ., Ser. B, Div. 2, Bot. 2: 181, 1934 (Horikawa 1934).

*** Wettsteinia schusteriana Grolle, J. Hattori Bot. Lab. 28: 99, 1965 (Grolle 1965f).

** Jamesonielloideae Inoue

*** **Cuspidatula Steph.**, Bull. Herb. Boissier (sér. 2) 1 (10): 1141 (124), 1901 (Stephani 1901c).

*** Cuspidatula contracta (Reinw., Blume et Nees) Steph., Bull. Herb. Boissier (sér. 2) 1 (11): 1141 (124), 1901 (Stephani 1901c). Bas.: Jungermannia contracta Reinw., Blume et Nees, Nova Acta Phys.-Med. Acad. Caes. Leop.-Carol. Nat. Cur. 12 (1): 233, 1824 [1825] (Reinwardt et al. 1824a).

*** Cuspidatula flaccida (Steph.) K.Feldberg, Váňa, Hentschel et Heinrichs, Cryptog. Bryol. 31 (2): 142, 2010 (Feldberg et al. 2010b). Bas.: Anastrophyllum flaccidum Steph., Sp. Hepat. (Stephani) 6: 105, 1917 (Stephani 1917a).

*** Cuspidatula flexicaulis (Nees) Váňa et L.Söderstr., Phytotaxa 76 (3): 35, 2013 (Váňa et al. 2013h). Bas.: Jungermannia flexicaulis Nees, Linnaea 6 (4): 604, 1831 (Nees 1831).

*** Cuspidatula kirkii (Steph.) K.Feldberg, Váňa, Hentschel et Heinrichs, Cryptog. Bryol. 31 (2): 142, 2010 (Feldberg et al. 2010b). Bas.: Jamesoniella kirkii Steph., Hedwigia 34 (2): 47, 1895 (Stephani 1895c).

*** Cuspidatula monodon (Taylor) Steph., Bull. Herb. Boissier (sér. 2) 1 (11): 1143 (126), 1901 (Stephani 1901c). Bas.: Jungermannia monodon Taylor, Nov. Stirp. Pug. 8: 7, 1844 (Lehmann 1844).

*** Cuspidatula orbicularis (Grolle) Váňa et L.Söderstr., Phytotaxa 76 (3): 36, 2013 (Váňa et al. 2013h). Bas.: Jamesoniella orbicularis Grolle, Feddes Repert. 82 (1): 42, 1971 (Grolle 1971b).

*** Cuspidatula robusta (Austin) Váňa et L.Söderstr., Phytotaxa 76 (3): 36, 2013 (Váňa et al. 2013h). Bas.: Jungermannia robusta Austin, Proc. Acad. Nat. Sci. Philadelphia 21: 219, 1869 (Austin 1869).

** **Denotarisia Grolle**, Feddes Repert. 82 (1): 6, 1971 (Grolle 1971b).

*** Denotarisia linguifolia (De Not.) Grolle, Feddes Repert. 82 (1): 6, 1971 (Grolle 1971b). Bas.: Plagiochila linguifolia De Not., Epat. Borneo: 13, 1874 (De Notaris 1874).

** **Nothostrepta R.M.Schust.**, Phytologia 45 (5): 420, 1980 (Schuster 1980b).

*** Nothostrepta bifida (Steph.) R.M.Schust., Phytologia 45 (5): 420, 1980 (Schuster 1980b). Bas.: Plagiochila bifida Steph., Annuario Reale Ist. Bot. Roma 2: 86, 1885 [1886] (Stephani 1885d).

*** Nothostrepta longissima (Steph.) R.M.Schust., Phytologia 45 (5): 420, 1980 (Schuster 1980b). Bas.: Anastrophyllum longissimum Steph., Bih. Kongl. Svenska Vetensk.-Akad. Handl. 26 (III, 17): 13, 1901 (Stephani 1901b).

** **Pisanoa Hässel**, Lindbergia 14 (3): 179, 1988 [1989] (Hässel 1988).

*** Pisanoa chilensis Hässel, Lindbergia 14 (3): 179, 1988 [1989] (Hässel 1988).

** **Protosyzygiella (Inoue) R.M.Schust.**, Hepat. Anthocerotae N. Amer. 4: 334, 1980 (Schuster 1980c). Bas.: Syzygiella subg. Protosyzygiella Inoue, J. Hattori Bot. Lab. 29: 180, 1966 (Inoue 1966c).

*** Protosyzygiella pseudoconnexa (Herzog) R.M.Schust., Hepat. Anthocerotae N. Amer. 4: 334, 1980 (Schuster 1980c). Bas.: Plagiochila pseudoconnexa Herzog, Rev. Bryol. Lichénol. 21 (3/4): 259, 1952 [1953] (Herzog 1952d).

*** **Syzygiella Spruce**, J. Bot. 14: 234, 1876 (Spruce 1876a). ^24^

*** **subg. Anomalae (Inoue) K.Feldberg, Váňa, Hentschel et Heinrichs**, Cryptog. Bryol. 31 (2): 143, 2010 (Feldberg et al. 2010b). Bas.: Syzygiella sect. Anomalae Inoue, J. Hattori Bot. Lab. 29: 183, 1966 (Inoue 1966c).

*** Syzygiella anomala (Lindenb. et Gottsche) Steph., Bull. Herb. Boissier (sér. 2) 2 (5): 471 (190), 1902 (Stephani 1902f). Bas.: Plagiochila anomala Lindenb. et Gottsche, Syn. Hepat. 5: 646, 1847 (Gottsche et al. 1847).

*** Syzygiella bilobata Inoue, J. Hattori Bot. Lab. 29: 186, 1966 (Inoue 1966c).

** Syzygiella ciliata Gradst. et A.R.Benitez, Nova Hedwigia 99 (1/2): 115, 2014 (Gradstein and Benitez 2014).

*** Syzygiella concreta (Gottsche) Spruce, J. Bot. 14: 234, 1876 (Spruce 1876a). Bas.: Jungermannia concreta Gottsche, Mexik. Leverm.: 82, 1863 (Gottsche 1863).

*** Syzygiella manca (Mont.) Steph., Hedwigia 31 (1): 14, 1892 (Jack and Stephani 1892). Bas.: Chiloscyphus mancus Mont., Syll. Gen. Sp. Crypt.: 63, 1856 (Montagne 1856b).

*** Syzygiella pectiniformis Spruce, Trans. & Proc. Bot. Soc. Edinburgh 15: 501, 1885 (Spruce 1885).

*** Syzygiella tonduzana Steph., Sp. Hepat. (Stephani) 6: 118, 1917 (Stephani 1917a).

*** Syzygiella trigonifolia (Steph.) Herzog, Hedwigia 74 (2): 87, 1934 (Herzog 1934a). Bas.: Jamesoniella trigonifolia Steph., Biblioth. Bot. 87 (2): 185, 1916 (Stephani 1916a).

*** **subg. Cryptochila (R.M.Schust.) K.Feldberg, Váňa, Hentschel et Heinrichs**, Cryptog. Bryol. 31 (2): 143, 2010 (Feldberg et al. 2010b). Bas.: Cryptochila R.M.Schust., J. Hattori Bot. Lab. 26: 284, 1963 (Schuster 1963b).

*** Syzygiella acinacifolia (Hook.f. et Taylor) K.Feldberg, Váňa, Hentschel et Heinrichs, Cryptog. Bryol. 31 (2): 143, 2010 (Feldberg et al. 2010b). Bas.: Jungermannia acinacifolia Hook.f. et Taylor, London J. Bot. 3: 367, 1844 (Hooker and Taylor 1844a).

*** Syzygiella nigrescens (Steph.) K.Feldberg, Váňa, Hentschel et Heinrichs, Cryptog. Bryol. 31 (2): 143, 2010 (Feldberg et al. 2010b). Bas.: Jamesoniella nigrescens Steph., Hedwigia 34 (2): 48, 1895 (Stephani 1895c).

*** Syzygiella paludosa (Steph.) K.Feldberg, Váňa, Hentschel et Heinrichs, Cryptog. Bryol. 31 (2): 143, 2010 (Feldberg et al. 2010b). Bas.: Jamesoniella paludosa Steph., Bih. Kongl. Svenska Vetensk.-Akad. Handl. 26 (III, 17): 11, 1901 (Stephani 1901b).

*** Syzygiella pseudocclusa (E.A.Hodgs.) K.Feldberg, Váňa, Hentschel et Heinrichs, Cryptog. Bryol. 31 (2): 143, 2010 (Feldberg et al. 2010b). Bas.: Jamesoniella pseudocclusa E.A.Hodgs., Trans. Roy. Soc. New Zealand 85 (4): 583, 1958 (Hodgson 1958).

*** Syzygiella sonderi (Gottsche) K.Feldberg, Váňa, Hentschel et Heinrichs, Cryptog. Bryol. 31 (2): 143, 2010 (Feldberg et al. 2010b). Bas.: Jungermannia sonderi Gottsche, Linnaea 28 (5): 550, 1856 [1857] (Gottsche 1856).

*** Syzygiella spegazziniana (Spruce ex C.Massal.) K.Feldberg, Váňa, Hentschel et Heinrichs, Cryptog. Bryol. 31 (2): 144, 2010 (Feldberg et al. 2010b). Bas.: Jungermannia spegazziniana Spruce ex C.Massal., Nuovo Giorn. Bot. Ital. 17 (3): 216, 1885 (Massalongo 1885).

*** **subg. Pseudoplagiochila Inoue**, J. Hattori Bot. Lab. 29: 182, 1966 (Inoue 1966c).

*** Syzygiella ovalifolia Inoue, J. Hattori Bot. Lab. 29: 191, 1966 (Inoue 1966c).

* Syzygiella securifolia (Nees) Inoue, J. Hattori Bot. Lab. 46: 232, 1979 (Inoue 1979a). Bas.: Plagiochila securifolia Nees, Sp. Hepat. (Lindenberg) 2-4: 58, 1840 (Lindenberg 1840). ^25^

*** Syzygiella subintegerrima (Reinw., Blume et Nees) Spruce, J. Linn. Soc., Bot. 30 (210): 362, 1895 (Gepp 1895b). Bas.: Jungermannia subintegerrima Reinw., Blume et Nees, Nova Acta Phys.-Med. Acad. Caes. Leop.-Carol. Nat. Cur. 12 (1): 238, 1824 [1825] (Reinwardt et al. 1824a).

*** Syzygiella tasmanica (Hook.f. et Taylor) K.Feldberg, Váňa, Hentschel et Heinrichs, Cryptog. Bryol. 31 (2): 144, 2010 (Feldberg et al. 2010b). Bas.: Jungermannia tasmanica Hook.f. et Taylor, London J. Bot. 5: 274, 1846 (Taylor 1846a).

*** **subg. Roivainenia (Perss.) K.Feldberg, Váňa, Hentschel et Heinrichs**, Cryptog. Bryol. 31 (2): 144, 2010 (Feldberg et al. 2010b). Bas.: Roivainenia Perss., Nova Hedwigia 3 (1): 43, 1961 (Persson and Grolle 1961).

*** Syzygiella jacquinotii (Mont.) Hentschel, K.Feldberg, Váňa et Heinrichs, Cryptog. Bryol. 31 (2): 144, 2010 (Feldberg et al. 2010b). Bas.: Jungermannia jacquinotii Mont., Ann. Sci. Nat. Bot. (sér. 2) 19: 250, 1843 (Montagne 1843).

*** **subg. Syzygiella**

*** Syzygiella autumnalis (DC.) K.Feldberg, Váňa, Hentschel et Heinrichs, Cryptog. Bryol. 31 (2): 144, 2010 (Feldberg et al. 2010b). Bas.: Jungermannia autumnalis DC., Fl. Franç. (DC. & Lamarck), 5 (6): 202, 1815 (De Candolle and Lamarck 1815).

*** Syzygiella campanulata Herzog, Rev. Bryol. Lichénol. 11 (1): 9, 1938 [1939] (Herzog 1938a).

*** Syzygiella colorata (Lehm.) K.Feldberg, Váňa, Hentschel et Heinrichs, Cryptog. Bryol. 31 (2): 144, 2010 (Feldberg et al. 2010b). Bas.: Jungermannia colorata Lehm., Linnaea 4: 366, 1829 (Lehmann 1829).

** Syzygiella colorata var. collenchymata J.J.Engel et Váňa, Mem. New York Bot. Gard. 105: 102, 2013 (Váňa and Engel 2013).

*** Syzygiella contigua Steph., Bull. Herb. Boissier (sér. 2) 2 (5): 470 (189), 1902 (Stephani 1902f). *Nom. nov. pro Jungermannia contigua* Gottsche, Ann. Sci. Nat. Bot. (sér. 5) 1: 118, 1864 (Gottsche 1864), *nom. illeg*.

*** Syzygiella elongella (Taylor) K.Feldberg, Váňa, Hentschel et Heinrichs, Cryptog. Bryol. 31 (2): 144, 2010 (Feldberg et al. 2010b). Bas.: Jungermannia elongella Taylor, London J. Bot. 5: 274, 1846 (Taylor 1846a).

*** Syzygiella macrocalyx (Mont.) Spruce, J. Bot. 14: 234, 1876 (Spruce 1876a). Bas.: Jungermannia macrocalyx Mont., Ann. Sci. Nat. Bot. (sér. 2) 19: 248, 1843 (Montagne 1843).

*** Syzygiella nipponica (S.Hatt.) K.Feldberg, Váňa, Hentschel et Heinrichs, Cryptog. Bryol. 31 (2): 145, 2010 (Feldberg et al. 2010b). Bas.: Jamesoniella nipponica S.Hatt., J. Jap. Bot. 19 (11): 350, 1943 (Hattori 1943b).

** Syzygiella oenops (Lindenb. et Gottsche) K.Feldberg, Váňa, Hentschel et Heinrichs, Cryptog. Bryol. 31 (2): 145, 2010 (Feldberg et al. 2010b). Bas.: Jungermannia oenops Lindenb. et Gottsche, Syn. Hepat. 5: 673, 1847 (Gottsche et al. 1847).

*** Syzygiella perfoliata (Sw.) Spruce, J. Bot. 14: 234, 1876 (Spruce 1876a). Bas.: Jungermannia perfoliata Sw., Prodr. (Swartz): 143, 1788 (Swartz 1788).

*** Syzygiella purpurascens (Steph.) K.Feldberg, Váňa, Hentschel et Heinrichs, Cryptog. Bryol. 31 (2): 145, 2010 (Feldberg et al. 2010b). Bas.: Jamesoniella purpurascens Steph., Bull. Soc. Roy. Bot. Belgique, Compt. Rend. 30 (2): 200, 1891 [1892] (Stephani 1891b).

*** Syzygiella rubricaulis (Nees) Steph., Bull. Herb. Boissier (sér. 2) 2 (5): 468 (187), 1902 (Stephani 1902f). Bas.: Jungermannia rubricaulis Nees, Fl. Bras. (Martius) 1 (1): 344, 1833 (Nees 1833a).

*** Syzygiella setulosa Steph., Bull. Herb. Boissier (sér. 2) 2 (5): 469 (188), 1902 (Stephani 1902f).

*** Syzygiella teres (Carrington et Pearson) Váňa, Phytotaxa 76 (3): 35, 2013 (Váňa et al. 2013h). Bas.: Jungermannia teres Carrington et Pearson, Pap. & Proc. Roy. Soc. Tasmania 1887: 9, 1888 (Carrington and Pearson 1888b).

*** Syzygiella undata (Mont.) K.Feldberg, Váňa, Hentschel et Heinrichs, Cryptog. Bryol. 31 (2): 145, 2010 (Feldberg et al. 2010b). Bas.: Jungermannia undata Mont., Ann. Sci. Nat. Bot. (sér. 4) 14: 183, 1860 (Montagne 1860).

***Incertae sedis***

*** Syzygiella eatonii (Austin) Inoue, J. Jap. Bot. 37 (12): 359, 1962 (Inoue 1962a). Bas.: Plagiochila eatonii Austin, Trans. Connecticut Acad. Arts 8 (15): 257, 1891 (Evans 1891).

*** Syzygiella uleana Steph., Hedwigia 44 (4): 224, 1905 (Stephani 1905a).

** **Vanaea (Inoue et Gradst.) Inoue et Gradst.**, Trop. Bryol. 1: 33, 1989 (Gradstein and Florschütz-de Waard 1989). Bas.: Anastrophyllum subg. Vanaea Inoue et Gradst., Bull. Natl. Sci. Mus. Tokyo, B 14 (3): 88, 1988 (Inoue and Gradstein 1988).

*** Vanaea plagiochiloides (Inoue et Gradst.) Inoue et Gradst., Trop. Bryol. 1: 33, 1989 (Gradstein and Florschütz-de Waard 1989). Bas.: Anastrophyllum plagiochiloides Inoue et Gradst., Bull. Natl. Sci. Mus. Tokyo, B 14 (3): 88, 1988 (Inoue and Gradstein 1988).

######## *** Anastrophyllaceae L.Söderstr., De Roo et Hedd.

by J. Váňa and L. Söderström

Anastrophyllaceae was described by Söderström et. al (2010b) from elements usually included in Lophoziaceae. Further taxonomic and nomenclatural notes can be found in Váňa et al. (2013k, 2013a). The complex of Chandonanthus/Plicanthus/Tetralophozia should be checked using molecular methods before generic and specific status of most of the species can be confirmed. The placement of Hattoria and Zantenia in the family is still provisional. The placement of Isopaches is also unclear, de Roo et al. (2007) placed it in the family, but the study of Vilnet et al. (2010) showed that it can not be placed there.

*** **Anastrepta (Lindb.) Schiffn.**, Hepat. (Engl.-Prantl): 85, 1893 (Schiffner 1893b). Bas.: Jungermannia sect. Anastrepta Lindb., Kongl. Svenska Vetensk.-Akad. Handl. (n.ser.) 23 (5): 40, 1889 (Lindberg and Arnell 1889).

*** Anastrepta orcadensis (Hook.) Schiffn., Hepat. (Engl.-Prantl): 85, 1893 (Schiffner 1893b). Bas.: Jungermannia orcadensis Hook., Brit. Jungermann.: tab. 71, 1815 (Hooker 1815).

*** **Anastrophyllum (Spruce) Steph.**, Hedwigia 32 (3): 139, 1893 (Stephani 1893b). Bas.: Jungermannia subg. Anastrophyllum Spruce, J. Bot. 14: 235, 1876 (Spruce 1876a).

*** Anastrophyllum alpinum Steph., Sp. Hepat. (Stephani) 6: 103, 1917 (Stephani 1917a).

*** Anastrophyllum assimile (Mitt.) Steph., Hedwigia 32 (3): 140, 1893 (Stephani 1893b). Bas.: Jungermannia assimilis Mitt., J. Proc. Linn. Soc., Bot. 5 (18): 93, 1860 [1861] (Mitten 1860c).

*** Anastrophyllum auritum (Lehm.) Steph., Bull. Herb. Boissier (sér. 2) 1 (11): 1137 (120), 1901 (Stephani 1901c). Bas.: Jungermannia aurita Lehm., Linnaea 4: 368, 1829 (Lehmann 1829).

*** Anastrophyllum ciliatum Steph., Hedwigia 32 (3): 139, 1893 (Stephani 1893b).

*** Anastrophyllum donnianum (Hook.) Steph., Hedwigia 32 (3): 140, 1893 (Stephani 1893b). Bas.: Jungermannia donniana Hook., Brit. Jungermann.: tab. 39, 1813 (Hooker 1813).

*** Anastrophyllum ellipticum Inoue, Bull. Natl. Sci. Mus. Tokyo, B 4 (1): 13, 1978 (Inoue 1978b). ^26^

** Anastrophyllum esenbeckii (Mont.) Steph., Hedwigia 32 (3): 140, 1893 (Stephani 1893b). Bas.: Jungermannia esenbeckii Mont., Ann. Sci. Nat. Bot. (sér. 2) 19: 247, 1843 (Montagne 1843).

*** Anastrophyllum fissum Steph., Bull. Herb. Boissier 5 (10): 845, 1897 (Stephani 1897c).

*** Anastrophyllum joergensenii Schiffn., Hedwigia 49 (4): 396, 1910 (Schiffner 1910b).

* Anastrophyllum lignicola D.B.Schill et D.G.Long, Ann. Bot. Fenn. 39 (2): 130, 2002 (Schill and Long 2002). ^27^

*** Anastrophyllum michauxii (F.Weber) H.Buch, Memoranda Soc. Fauna Fl. Fennica 8: 289, 1932 [1933] (Buch 1932). Bas.: Jungermannia michauxii F.Weber, Hist. Musc. Hepat. Prodr.: 76, 1815 (Weber 1815).

*** Anastrophyllum nigrescens (Mitt.) Steph., Hedwigia 32 (3): 140, 1893 (Stephani 1893b). Bas.: Jungermannia nigrescens Mitt., Hooker’s J. Bot. Kew Gard. Misc. 3: 358, 1851 (Mitten 1851).

*** Anastrophyllum obtusum Herzog, Trans. Brit. Bryol. Soc. 1 (4): 285, 1950 (Herzog 1950a).

*** Anastrophyllum piligerum (Nees) Steph., Hedwigia 32 (3): 140, 1893 (Stephani 1893b). Bas.: Jungermannia piligera Nees, Nova Acta Phys.-Med. Acad. Caes. Leop.-Carol. Nat. Cur. 12 (1): 414, 1824 [1825] (Reinwardt et al. 1824b).

*** Anastrophyllum squarrosum Herzog, Ann. Bryol. 5: 72, 1932 (Herzog 1932b).

*** Anastrophyllum stellatum R.M.Schust., Phytologia 39 (4): 243, 1978 (Schuster 1978a).

*** Anastrophyllum tubulosum (Nees) Grolle, J. Hattori Bot. Lab. 28: 101, 1965 (Grolle 1965e). Bas.: Jungermannia tubulosa Nees, Enum. Pl. Crypt. Javae: 32, 1830 (Nees 1830).

*** **Barbilophozia Loeske**, Verh. Bot. Vereins Prov. Brandenburg 49 (1): 37, 1908 (Loeske 1908).

** **subg. Barbilophozia**

*** Barbilophozia barbata (Schmidel ex Schreb.) Loeske, Verh. Bot. Vereins Prov. Brandenburg 49 (1): 37, 1908 (Loeske 1908). Bas.: Jungermannia barbata Schmidel ex Schreb., Spic. Fl. Lips.: 107, 1771 (Schreber 1771).

*** Barbilophozia hatcheri (A.Evans) Loeske, Verh. Bot. Vereins Prov. Brandenburg 49 (1): 37, 1908 (Loeske 1908). Bas.: Jungermannia hatcheri A.Evans, Bull. Torrey Bot. Club 25 (8): 417, 1898 (Evans 1898).

*** Barbilophozia lycopodioides (Wallr.) Loeske, Verh. Bot. Vereins Prov. Brandenburg 49 (1): 37, 1908 (Loeske 1908). Bas.: Jungermannia lycopodioides Wallr., Comp. fl. Germ. 2 (III): 76, 1831 (Bluff and Fingerhuth 1831).

*** Barbilophozia rubescens (R.M.Schust. et Damsh.) Kartt. et L.Söderstr., Ann. Bot. Fenn. 29 (2): 120, 1992 (Söderström et al. 1992). Bas.: Lophozia rubescens R.M.Schust. et Damsh., Phytologia 63 (5): 325, 1987 (Schuster and Damsholt 1987).

** **subg. Sudeticae (Schljakov) L.Söderstr., De Roo et Hedd.**, Phytotaxa 3: 50, 2010 (Söderström et al. 2010b). Bas.: Lophozia sect. Sudeticae Schljakov, Pečen. Mchi Sev. SSSR 3: 113, 1980 (Shliakov 1980a).

*** Barbilophozia sudetica (Nees ex Huebener) L.Söderstr., De Roo et Hedd., Phytotaxa 3: 50, 2010 (Söderström et al. 2010b). Bas.: Jungermannia sudetica Nees ex Huebener, Hepaticol. germ.: 142, 1834 (Hübener 1834). ^28^

*** **Biantheridion (Grolle) Konstant. et Vilnet**, Arctoa 18: 67, 2009 [2010] (Konstantinova and Vilnet 2009). Bas.: Jamesoniella sect. Biantheridion Grolle, Trans. Brit. Bryol. Soc. 4 (4): 662, 1964 (Grolle 1964i).

*** Biantheridion undulifolium (Nees) Konstant. et Vilnet, Arctoa 18: 67, 2009 [2010] (Konstantinova and Vilnet 2009). Bas.: Jungermannia schraderi β undulifolia Nees, Naturgesch. Eur. Leberm. 1: 306, 1833 (Nees 1833c).

*** **Chandonanthus Mitt.**, Handb. N. Zeal. fl. 2: 753, 1867 (Hooker 1867).

*** Chandonanthus squarrosus (Menzies) Mitt., Handb. N. Zeal. fl. 2: 753, 1867 (Hooker 1867). Bas.: Jungermannia squarrosa Menzies, Musci Exot. 1: tab. 78, 1818 (Hooker 1818).

*** **Crossocalyx Meyl.**, Bull. Soc. Vaud. Sci. Nat. 60 (249): 266, 1939 (Meylan 1939).

*** Crossocalyx hellerianus (Nees ex Lindenb.) Meyl., Bull. Soc. Vaud. Sci. Nat. 60 (249): 266, 1939 (Meylan 1939). Bas.: Jungermannia helleriana Nees ex Lindenb., Syn. hepat. eur: 64, 1829 (Lindenberg 1829).

*** Crossocalyx tenuis (Harry Williams) Schljakov, Novosti Sist. Nizš. Rast. 15: 246, 1978 (Shliakov 1978). Bas.: Anastrophyllum tenue Harry Williams, Bryologist 71 (1): 34, 1968 (Williams 1968).

*** **Gymnocolea (Dumort.) Dumort.**, Recueil Observ. Jungerm.: 17, 1835 (Dumortier 1835). Bas.: Jungermannia sect. Gymnocolea Dumort., Syll. Jungerm. Europ.: 52, 1831 (Dumortier 1831).

*** Gymnocolea borealis (Frisvoll et Moen) R.M.Schust., Lindbergia 12 (1): 7, 1986 (Schuster 1986). Bas.: Lophozia borealis Frisvoll et Moen, Lindbergia 6 (2): 138, 1980 [1981] (Frisvoll and Moen 1980).

** Gymnocolea fascinifera Potemkin, Arctoa 2: 76, 1993 (Potemkin 1993).

*** Gymnocolea inflata (Huds.) Dumort., Recueil Observ. Jungerm.: 17, 1835 (Dumortier 1835). Bas.: Jungermannia inflata Huds., Fl. Angl. (Hudson), ed. 2: 511, 1778 (Hudson 1778).

* Gymnocolea inflata subsp. acutiloba (Schiffn.) R.M.Schust. et Damsh. ex L.Söderstr. et Váňa, Lindbergia 27 (1): 43, 2002 (Söderström et al. 2002). Bas.: Lophozia acutiloba Schiffn., Hedwigia 48 (3): 187, 1909 (Schiffner 1909a). ^29^

*** **Hamatostrepta Váňa et D.G.Long**, Fieldiana, Bot. (n.ser.) 47: 134, 2008 (Váňa and Long 2008).

*** Hamatostrepta concinna Váňa et D.G.Long, Fieldiana, Bot. (n.ser.) 47: 134, 2008 (Váňa and Long 2008).

** **Hattoria R.M.Schust.**, Rev. Bryol. Lichénol. 30 (1/2): 69, 1961 (Schuster 1961a).

*** Hattoria yakushimensis (Horik.) R.M.Schust., Rev. Bryol. Lichénol. 30 (1/2): 70, 1961 (Schuster 1961a). Bas.: Anastrophyllum yakushimense Horik., J. Sci. Hiroshima Univ., Ser. B, Div. 2, Bot. 2: 149, 1934 (Horikawa 1934).

*** **Isopaches H.Buch**, Memoranda Soc. Fauna Fl. Fennica 8: 287, 1932 [1933] (Buch 1932).

*** Isopaches alboviridis (R.M.Schust.) Schljakov, Novosti Sist. Nizš. Rast. 16: 205, 1979 (Shliakov 1979). Bas.: Lophozia alboviridis R.M.Schust., Hepat. Anthocerotae N. Amer. 2: 487, 1969 (Schuster 1969b).

*** Isopaches bicrenatus (Schmidel ex Hoffm.) H.Buch, Memoranda Soc. Fauna Fl. Fennica 8: 288, 1932 [1933] (Buch 1932). Bas.: Jungermannia bicrenata Schmidel ex Hoffm., Deutschl. Fl., Theil 2 (Hoffm.): 11 (addenda), 1795 [1796] (Hoffmann 1795).

*** Isopaches decolorans (Limpr.) H.Buch, Memoranda Soc. Fauna Fl. Fennica 8: 288, 1932 [1933] (Buch 1932). Bas.: Jungermannia decolorans Limpr., Jahresber. Schles. Ges. Vaterl. Cult. 57: 316, 1879 [1880] (Limpricht 1879).

*** Isopaches pumicicola (Berggr.) Bakalin, Arctoa 17: 162, 2008 [2009] (Bakalin 2008b). Bas.: Lophozia pumicicola Berggr., New Zealand Hepat.: 21, 1898 (Berggren 1898).

*** **Neoorthocaulis L.Söderstr., De Roo et Hedd.**, Phytotaxa 3: 49, 2010 (Söderström et al. 2010b).

*** Neoorthocaulis attenuatus (Mart.) L.Söderstr., De Roo et Hedd., Phytotaxa 3: 49, 2010 (Söderström et al. 2010b). Bas.: Jungermannia quinquedentata δ attenuata Mart., Fl. crypt. erlang.: 177, 1817 (Martius 1817).

*** Neoorthocaulis binsteadii (Kaal.) L.Söderstr., De Roo et Hedd., Phytotaxa 3: 49, 2010 (Söderström et al. 2010b). Bas.: Jungermannia binsteadii Kaal., Skr. Vidensk.-Selsk. Christiana, Math.-Naturvidensk. Kl. 1898 (9): 9, 1898 (Kaalaas 1898).

*** Neoorthocaulis floerkei (F.Weber et D.Mohr) L.Söderstr., De Roo et Hedd., Phytotaxa 3: 50, 2010 (Söderström et al. 2010b). Bas.: Jungermannia floerkei F.Weber et D.Mohr, Bot. Taschenb. (Weber): 410, 1807 (Weber and Mohr 1807).

** Neoorthocaulis hyperboreus (R.M.Schust.) L.Söderstr., De Roo et Hedd., Phytotaxa 3: 50, 2010 (Söderström et al. 2010b). Bas.: Lophozia floerkei var. hyperborea R.M.Schust., Bull. Natl. Mus. Canada 164: 21, 1959 (Schuster et al. 1959).

*** **Orthocaulis H.Buch**, Memoranda Soc. Fauna Fl. Fennica 8: 293, 1932 [1933] (Buch 1932).

*** Orthocaulis atlanticus (Kaal.) H.Buch, Memoranda Soc. Fauna Fl. Fennica 8: 294, 1932 [1933] (Buch 1932). Bas.: Jungermannia atlantica Kaal., Skr. Vidensk.-Selsk. Christiana, Math.-Naturvidensk. Kl. 1898 (9): 11, 1898 (Kaalaas 1898).

* Orthocaulis cavifolius H.Buch et S.W.Arnell, Memoranda Soc. Fauna Fl. Fennica 26: 71, 1951 (Buch 1951).

** **Plicanthus R.M.Schust.**, Nova Hedwigia 74 (3/4): 484, 2002 (Schuster 2002a).

*** Plicanthus birmensis (Steph.) R.M.Schust., Beih. Nova Hedwigia 119: 223, 2002 (Schuster 2002b). Bas.: Chandonanthus birmensis Steph., Bull. Soc. Roy. Bot. Belgique 38 (1): 43, 1899 (Stephani 1899h). ^30^

** Plicanthus difficilis (Steph.) L.Söderstr. et Váňa, Phytotaxa 81 (1): 30, 2013 (Váňa et al. 2013a). Bas.: Chandonanthus difficilis Steph., J. & Proc. Roy. Soc. New South Wales 48 (1/2): 101, 1914 (Stephani and Watts 1914).

** Plicanthus giganteus (Steph.) R.M.Schust., Nova Hedwigia 74 (3/4): 485, 2002 (Schuster 2002a). Bas.: Chandonanthus giganteus Steph., Wiss. Ergebn. Deut. Zentr.-Afr. Exped. (1907-08), Bot. 2: 124, 1911 (Stephani 1911a). ^31^

*** Plicanthus hirtellus (F.Weber) R.M.Schust., Nova Hedwigia 74 (3/4): 492, 2002 (Schuster 2002a). Bas.: Jungermannia hirtella F.Weber, Hist. Musc. Hepat. Prodr.: 50, 1815 (Weber 1815).

*** **Schizophyllopsis Váňa et L.Söderstr.**, Phytotaxa 152 (1): 48, 2013 (Váňa et al. 2013f). *Nom. nov. pro Anastrophyllum* subg. Schizophyllum R.M.Schust., Hepat. Anthocerotae N. Amer. 2: 739, 1969 (Schuster 1969b). ^32^

** Schizophyllopsis aristata (Herzog ex N.Kitag.) Váňa et L.Söderstr., Phytotaxa 152 (1): 48, 2013 (Váňa et al. 2013f). Bas.: Anastrophyllum bidens var. aristatum Herzog ex N.Kitag., J. Hattori Bot. Lab. 33: 216, 1970 (Kitagawa 1970).

*** Schizophyllopsis bidens (Reinw., Blume et Nees) Váňa et L.Söderstr., Phytotaxa 152 (1): 48, 2013 (Váňa et al. 2013f). Bas.: Jungermannia bidens Reinw., Blume et Nees, Nova Acta Phys.-Med. Acad. Caes. Leop.-Carol. Nat. Cur. 12 (1): 208, 1824 [1825] (Reinwardt et al. 1824a).

** Schizophyllopsis lanciloba (Steph.) Váňa et L.Söderstr., Phytotaxa 152 (1): 48, 2013 (Váňa et al. 2013f). Bas.: Anastrophyllum lancilobum Steph., Sp. Hepat. (Stephani) 6: 107, 1917 (Stephani 1917a).

*** Schizophyllopsis papillosa (J.J.Engel et Braggins) Váňa et L.Söderstr., Phytotaxa 152 (1): 48, 2013 (Váňa et al. 2013f). Bas.: Anastrophyllum papillosum J.J.Engel et Braggins, J. Bryol. 20 (2): 381, 1998 (Engel and Braggins 1998).

*** Schizophyllopsis sphenoloboides (R.M.Schust.) Váňa et L.Söderstr., Phytotaxa 152 (1): 49, 2013 (Váňa et al. 2013f). Bas.: Anastrophyllum sphenoloboides R.M.Schust., Hepat. Anthocerotae N. Amer. 2: 741, 1969 (Schuster 1969b).

*** **Schljakovia Konstant. et Vilnet**, Arctoa 18: 66, 2009 [2010] (Konstantinova and Vilnet 2009).

*** Schljakovia kunzeana (Huebener) Konstant. et Vilnet, Arctoa 18: 66, 2009 [2010] (Konstantinova and Vilnet 2009). Bas.: Jungermannia kunzeana Huebener, Hepaticol. germ.: 115, 1834 (Hübener 1834).

*** **Schljakovianthus Konstant. et Vilnet**, Arctoa 18: 66, 2009 [2010] (Konstantinova and Vilnet 2009).

*** Schljakovianthus quadrilobus (Lindb.) Konstant. et Vilnet, Arctoa 18: 66, 2009 [2010] (Konstantinova and Vilnet 2009). Bas.: Jungermannia quadriloba Lindb., Kongl. Svenska Vetensk.-Akad. Handl. (n.ser.) 23 (5): 55, 1889 (Lindberg and Arnell 1889).

*** **Sphenolobopsis R.M.Schust. et N.Kitag.**, Nova Hedwigia 22: 152, 1971 [1972] (Schuster 1971b).

*** Sphenolobopsis pearsonii (Spruce) R.M.Schust., Nova Hedwigia 22: 153, 1971 [1972] (Schuster 1971b). Bas.: Jungermannia pearsonii Spruce, J. Bot. 19: 33, 1881 (Spruce 1881b).

*** **Sphenolobus (Lindb.) Berggr.**, New Zealand Hepat.: 22, 1898 (Berggren 1898). Bas.: Jungermannia sect. Sphenolobus Lindb., Not. Sällsk. Fauna Fl. Fenn. Förh. 13: 369, 1874 (Lindberg 1874a).

*** Sphenolobus austroamericanus (Váňa) Váňa, Phytotaxa 81 (1): 30, 2013 (Váňa et al. 2013a). Bas.: Anastrophyllum austroamericanum Váňa, J. Hattori Bot. Lab. 48: 225, 1980 (Váňa 1980).

*** Sphenolobus minutus (Schreb. ex D.Crantz) Berggr., New Zealand Hepat.: 22, 1898 (Berggren 1898). Bas.: Jungermannia minuta Schreb. ex D.Crantz, Forts. Hist. Grönland: 285, 1770 (Crantz 1770; non vidi).

*** Sphenolobus saxicola (Schrad.) Steph., Bull. Herb. Boissier (sér. 2) 2 (2): 168 (160), 1902 (Stephani 1902d). Bas.: Jungermannia saxicola Schrad., Syst. Samml. Crypt. Gew. 2: 4, 1797 (Schrader 1797).

** **Tetralophozia (R.M.Schust.) Schljakov**, Novosti Sist. Nizš. Rast. 13: 227, 1976 (Shliakov 1976). Bas.: Chandonanthus subg. Tetralophozia R.M.Schust., J. Hattori Bot. Lab. 23: 206, 1960 [1961] (Schuster 1960a).

*** Tetralophozia cavallii (Gola) Váňa, Trop. Bryol. 8: 102, 1993 (Váňa 1993). Bas.: Blepharostomum cavallii Gola, Ann. Bot. (Rome) 6 (2): 274, 1907 (Gola 1907).

*** Tetralophozia filiformis (Steph.) Urmi, J. Bryol. 12 (3): 394, 1983 (Urmi 1983). Bas.: Chandonanthus filiformis Steph., Sp. Hepat. (Stephani) 3: 644, 1909 (Stephani 1909a).

** Tetralophozia pilifera (Steph.) R.M.Schust., Nova Hedwigia 74 (3/4): 482, 2002 (Schuster 2002a). Bas.: Chandonanthus pilifer Steph., Sp. Hepat. (Stephani) 3: 644, 1909 (Stephani 1909a).

*** Tetralophozia setiformis (Ehrh.) Schljakov, Novosti Sist. Nizš. Rast. 13: 228, 1976 (Shliakov 1976). Bas.: Jungermannia setiformis Ehrh., Hannover. Mag. 22 (8): 142, 1784 (Ehrhart 1784).

*** **Zantenia (S.Hatt.) Váňa et J.J.Engel**, Mem. New York Bot. Gard. 105: 29, 2013 (Váňa and Engel 2013). Bas.: Anastrophyllum subg. Zantenia S.Hatt., Bot. Mag. (Tokyo) 79 (937): 342, 1966 (Hattori 1966a).

*** Zantenia borneensis (Herzog) Váňa et J.J.Engel, Mem. New York Bot. Gard. 105: 29, 2013 (Váňa and Engel 2013). Bas.: Anastrophyllum borneense Herzog, Trans. Brit. Bryol. Soc. 1 (4): 282, 1950 (Herzog 1950a).

*** Zantenia denticulata (Grolle) Váňa et J.J.Engel, Mem. New York Bot. Gard. 105: 30, 2013 (Váňa and Engel 2013). Bas.: Anastrophyllum denticulatum Grolle, Nova Hedwigia 16: 148, 1968 (Grolle 1968d).

*** Zantenia karstenii (Schiffn.) Váňa et J.J.Engel, Mem. New York Bot. Gard. 105: 30, 2013 (Váňa and Engel 2013). Bas.: Anastrophyllum karstenii Schiffn., Nova Acta Acad. Caes. Leop.-Carol. German. Nat. Cur. 60 (2): 268, 1893 (Schiffner 1893a).

*** Zantenia prionophylla (S.Hatt.) Váňa et J.J.Engel, Mem. New York Bot. Gard. 105: 30, 2013 (Váňa and Engel 2013). Bas.: Anastrophyllum prionophyllum S.Hatt., Bot. Mag. (Tokyo) 79 (937): 342, 1966 (Hattori 1966a).

######## *** Cephaloziaceae Mig.

by J. Váňa with contributions by S.R. Gradstein (Odontoschisma)

Cephaloziaceae was recently studied by Vilnet et al. (2012) and Feldberg et al. (2013). Some taxonomic and nomenclatural notes were published by Váňa et al. (2013g, 2013i) and Gradstein et al. (2014b).

######### ** Alobielloideae R.M.Schust.

** **Alobiella (Spruce) Schiffn.**, Hepat. (Engl.-Prantl): 98, 1893 (Schiffner 1893b). Bas.: Cephalozia subg. Alobiella Spruce, Cephalozia: 28, 1882 (Spruce 1882).

*** Alobiella husnotii (Spruce) Schiffn., Hepat. (Engl.-Prantl): 98, 1893 (Schiffner 1893b). Bas.: Cephalozia husnotii Spruce, Cephalozia: 30, 1882 (Spruce 1882).

** **Alobiellopsis R.M.Schust.**, Nova Hedwigia 10 (1/2): 25, 1965 (Schuster 1965b).

*** Alobiellopsis acroscypha (Spruce) R.M.Schust., Nova Hedwigia 10 (1/2): 25, 1965 (Schuster 1965b). Bas.: Cephalozia acroscypha Spruce, Cephalozia: 30, 1882 (Spruce 1882).

*** Alobiellopsis dominicensis (Spruce) Fulford, Mem. New York Bot. Gard. 11 (3): 350, 1968 (Fulford 1968). Bas.: Alobiella dominicensis Spruce, J. Linn. Soc., Bot. 30 (210): 355, 1895 (Gepp 1895b).

*** Alobiellopsis heteromorpha (Lehm.) R.M.Schust., Bull. Natl. Sci. Mus. Tokyo (n.ser.) 12 (3): 682, 1969 (Schuster 1969a). Bas.: Jungermannia heteromorpha Lehm., Linnaea 4: 362, 1829 (Lehmann 1829).

*** Alobiellopsis parvifolia (Steph.) R.M.Schust., Bull. Natl. Sci. Mus. Tokyo (n.ser.) 12 (3): 679, 1969 (Schuster 1969a). Bas.: Alobiella parvifolia Steph., Bull. Herb. Boissier (sér. 2) 8 (8): 568 (352), 1908 (Stephani 1908e).

*** Alobiellopsis pillansii (Sim) R.M.Schust., Bull. Natl. Sci. Mus. Tokyo (n.ser.) 12 (3): 683, 1969 (Schuster 1969a). Bas.: Cephalozia pillansii Sim, Trans. Roy. Soc. South Africa 15 (1): 87, 1926 (Sim 1926).

######### ** Cephalozioideae Müll.Frib.

*** **Cephalozia (Dumort.) Dumort.**, Recueil Observ. Jungerm.: 18, 1835 (Dumortier 1835). Bas.: Jungermannia sect. Cephalozia Dumort., Syll. Jungerm. Europ.: 60, 1831 (Dumortier 1831).

*** Cephalozia acuminata (Herzog) Váňa, Phytotaxa 112 (1): 11, 2013 (Váňa et al. 2013g). Bas.: Hygrobiella acuminata Herzog, Trans. Brit. Bryol. Soc. 1 (4): 292, 1950 (Herzog 1950a).

** Cephalozia acutiloba (Inoue) Váňa, Phytotaxa 112 (1): 11, 2013 (Váňa et al. 2013g). Bas.: Metahygrobiella acutiloba Inoue, Bull. Natl. Sci. Mus. Tokyo (n.ser.) 10 (2): 155, 1967 (Inoue 1967c).

*** Cephalozia albula Steph., Hedwigia 32 (5): 318, 1893 (Stephani 1893d). *Nom. nov. pro Jungermannia albula* Mitt., J. Proc. Linn. Soc., Bot. 5 (18): 93, 1860 [1861] (Mitten 1860c), *nom. illeg*.

*** Cephalozia ambigua C.Massal., Malpighia 21 (7/8): 310, 1907 (Massalongo 1907).

*** Cephalozia austrigena R.M.Schust. ex J.J.Engel, Novon 17 (3): 312, 2007 (Engel 2007). Based on: Cephalozia bicuspidata subsp. austrigena R.M.Schust., Hepat. Anthocerotae N. Amer. 3: 712, 1974 (Schuster 1974), *nom. inval*.

*** Cephalozia badia (Gottsche) Steph., Bull. Herb. Boissier (sér. 2) 8 (7): 483 (313), 1908 (Stephani 1908f). Bas.: Jungermannia badia Gottsche, Int. Polarforsch., Deutsch. Exped. 2: 452, 1890 (Gottsche 1890).

*** Cephalozia bicuspidata (L.) Dumort., Recueil Observ. Jungerm.: 18, 1835 (Dumortier 1835). Bas.: Jungermannia bicuspidata L., Sp. Pl. 1: 1132, 1753 (Linnaeus 1753).

* Cephalozia bicuspidata subsp. lammersiana (Huebener) R.M.Schust., Hepat. Anthocerotae N. Amer. 3: 730, 1974 (Schuster 1974). Bas.: Jungermannia lammersiana Huebener, Flora 15 (20): 306, 1832 (Hübener 1832).

*** Cephalozia chilensis (J.J.Engel et R.M.Schust.) R.M.Schust., Beih. Nova Hedwigia 119: 29, 2002 (Schuster 2002b). Bas.: Metahygrobiella chilensis J.J.Engel et R.M.Schust., Brittonia 40 (2): 203, 1988 (Engel and Schuster 1988).

*** Cephalozia conchata (Grolle et Váňa) Váňa, Syst. Bot. 40 (1): 38, 2015 (Shaw et al. 2015). Bas.: Jungermannia conchata Grolle et Váňa, Fragm. Florist. Geobot. 37 (1): 3, 1992 (Grolle and Váňa 1992).

*** Cephalozia crossii Spruce, Cephalozia: 46, 1882 (Spruce 1882).

*** Cephalozia darjeelingensis Udar et D.Kumar, Geophytology 6 (1): 36, 1976 (Udar and Kumar 1976).

*** Cephalozia drucei (R.M.Schust.) Váňa, Phytotaxa 112 (1): 11, 2013 (Váňa et al. 2013g). Bas.: Metahygrobiella drucei R.M.Schust., J. Hattori Bot. Lab. 26: 273, 1963 (Schuster 1963b).

*** Cephalozia fuegiensis Váňa, Phytotaxa 112 (1): 12, 2013 (Váňa et al. 2013g). *Nom. nov. pro Hygrobiella dusenii* Steph., Sp. Hepat. (Stephani) 6: 444, 1924 (Stephani 1924).

*** Cephalozia hamatiloba Steph., Bull. Herb. Boissier (sér. 2) 8 (6): 427 (303), 1908 (Stephani 1908g).

*** Cephalozia hamatiloba subsp. siamensis (N.Kitag.) Váňa, Acta Bot. Fenn. 177: 16, 2004 (Koponen et al. 2004). Bas.: Cephalozia siamensis N.Kitag., J. Hattori Bot. Lab. 32: 293, 1969 (Kitagawa 1969c).

*** Cephalozia lacinulata (J.B.Jack ex Gottsche et Rabenh.) Spruce, Cephalozia: 45, 1882 (Spruce 1882). Bas.: Jungermannia lacinulata J.B.Jack ex Gottsche et Rabenh., Hepat. Eur., Leberm. 62-64: no. 624, 1877 (Gottsche and Rabenhorst 1877).

*** Cephalozia lucens (A.Evans) Steph., Bull. Herb. Boissier (sér. 2) 8 (7): 496 (326), 1908 (Stephani 1908f). Bas.: Jungermannia lucens A.Evans, Trans. Connecticut Acad. Arts 8 (15): 258, 1891 (Evans 1891).

*** Cephalozia macgregorii (Steph.) Váňa, Phytotaxa 112 (1): 12, 2013 (Váňa et al. 2013g). Bas.: Hygrobiella macgregorii Steph., Hedwigia 34 (2): 45, 1895 (Stephani 1895c).

*** Cephalozia macounii (Austin) Austin, Hepat. bor.-amer.: 14, 1873 (Austin 1873). Bas.: Jungermannia macounii Austin, Proc. Acad. Nat. Sci. Philadelphia 21: 222, 1869 (Austin 1869).

*** Cephalozia maxima Steph., Sp. Hepat. (Stephani) 6: 441, 1924 (Stephani 1924).

*** Cephalozia mollusca (De Not.) Váňa, Phytotaxa 112 (1): 12, 2013 (Váňa et al. 2013g). Bas.: Jungermannia mollusca De Not., Epat. Borneo: 16, 1874 (De Notaris 1874).

* Cephalozia neesiana Steph., Bull. Herb. Boissier (sér. 2) 8 (6): 429 (305), 1908 (Stephani 1908g). ^33^

*** Cephalozia nishimurae (N.Kitag.) Váňa, Phytotaxa 112 (1): 12, 2013 (Váňa et al. 2013g). Bas.: Hygrobiella nishimurae N.Kitag., Misc. Bryol. Lichenol. 9 (4): 69, 1982 (Kitagawa 1982).

** Cephalozia pachygyna R.M.Schust. ex J.J.Engel, Novon 17 (3): 313, 2007 (Engel 2007).

** Cephalozia physocaula (Hook.f. et Taylor) Steph., Bull. Herb. Boissier (sér. 2) 8 (7): 485 (315), 1908 (Stephani 1908f). Bas.: Jungermannia physocaula Hook.f. et Taylor, London J. Bot. 3: 455, 1844 (Hooker and Taylor 1844b).

** Cephalozia schusteriana J.J.Engel, Novon 17 (3): 313, 2007 (Engel 2007).

*** Cephalozia stolonacea (Herzog) Váňa, Phytotaxa 112 (1): 12, 2013 (Váňa et al. 2013g). Bas.: Hygrobiella stolonacea Herzog, Trans. Brit. Bryol. Soc. 1 (4): 293, 1950 (Herzog 1950a).

*** Cephalozia tubulata (Hook.f. et Taylor) Trevis., Mem. Reale Ist. Lombardo Sci. (Ser. 3), C. Sci. Mat. 4 (13): 417, 1877 (Trevisan 1877). Bas.: Jungermannia tubulata Hook.f. et Taylor, London J. Bot. 3: 463, 1844 (Hooker and Taylor 1844b).

**Excluded from the genus**

* Cephalozia hians Steph., Sp. Hepat. (Stephani) 6: 441, 1924 (Stephani 1924). ^34^

* Cephalozia indica Udar et D.Kumar, Geophytology 6 (1): 42, 1976 (Udar and Kumar 1976). ^35^

* Cephalozia kodaikanalensis G.Asthana et Saumya Srivast., Geophytology 43 (1): 63, 2013 (Asthana and Srivastava 2013). ^36^

* Cephalozia parvifolia Arnell, Rev. Bryol. 25 (1): 1, 1898 (Arnell 1898). ^37^

* Cephalozia tricuspidata (Nees) Trevis., Mem. Reale Ist. Lombardo Sci. (Ser. 3), C. Sci. Mat. 4 (13): 417, 1877 (Trevisan 1877). Bas.: Jungermannia tricuspidata Nees, Enum. Pl. Crypt. Javae: 31, 1830 (Nees 1830). ^38^

*** **Fuscocephaloziopsis Fulford**, Mem. New York Bot. Gard. 11 (3): 353, 1968 (Fulford 1968).

** Fuscocephaloziopsis affinis (Lindb. ex Steph.) Váňa et L.Söderstr., Phytotaxa 112 (1): 9, 2013 (Váňa et al. 2013g). Bas.: Cephalozia affinis Lindb. ex Steph., Bull. Herb. Boissier (sér. 2) 8 (4): 277 (291), 1908 (Stephani 1908j).

*** Fuscocephaloziopsis africana (Váňa) Váňa et L.Söderstr., Phytotaxa 112 (1): 9, 2013 (Váňa et al. 2013g). Bas.: Cephalozia africana Váňa, Beih. Nova Hedwigia 90: 187, 1988 (Váňa 1988).

*** Fuscocephaloziopsis albescens (Hook.) Váňa et L.Söderstr., Phytotaxa 112 (1): 9, 2013 (Váňa et al. 2013g). Bas.: Jungermannia albescens Hook., Brit. Jungermann.: tab. 72, 1815 (Hooker 1815).

* Fuscocephaloziopsis albescens var. islandica (Nees) Váňa et L.Söderstr., Phytotaxa 112 (1): 9, 2013 (Váňa et al. 2013g). Bas.: Jungermannia islandica Nees, Naturgesch. Eur. Leberm. 2: 29, 1836 (Nees 1836).

*** Fuscocephaloziopsis baldwinii (C.M.Cooke) Váňa et L.Söderstr., Phytotaxa 112 (1): 9, 2013 (Váňa et al. 2013g). Bas.: Cephalozia baldwinii C.M.Cooke, Trans. Connecticut Acad. Arts 12 (1): 35, 1904 (Cooke 1904).

** Fuscocephaloziopsis biloba (Herzog) Fulford, Mem. New York Bot. Gard. 11 (3): 355, 1968 (Fulford 1968). Bas.: Alobiella biloba Herzog, Feddes Repert. Spec. Nov. Regni Veg. 57 (1/2): 166, 1955 (Herzog 1955).

*** Fuscocephaloziopsis catenulata (Huebener) Váňa et L.Söderstr., Phytotaxa 112 (1): 9, 2013 (Váňa et al. 2013g). Bas.: Jungermannia catenulata Huebener, Hepaticol. germ.: 169, 1834 (Hübener 1834).

*** Fuscocephaloziopsis catenulata subsp. nipponica (S.Hatt.) Váňa et L.Söderstr., Phytotaxa 112 (1): 9, 2013 (Váňa et al. 2013g). Bas.: Cephalozia nipponica S.Hatt., Bull. Tokyo Sci. Mus. 11: 74, 1944 (Hattori 1944d).

*** Fuscocephaloziopsis connivens (Dicks.) Váňa et L.Söderstr., Phytotaxa 112 (1): 9, 2013 (Váňa et al. 2013g). Bas.: Jungermannia connivens Dicks., Fasc. Pl. Crypt. Brit. 4: 19, 1801 (Dickson 1801).

*** Fuscocephaloziopsis connivens subsp. fissa (Steph.) Váňa et L.Söderstr., Phytotaxa 112 (1): 9, 2013 (Váňa et al. 2013g). Bas.: Cephalozia fissa Steph., Hedwigia 30 (5): 204, 1891 (Stephani 1891a).

*** Fuscocephaloziopsis connivens subsp. sandwicensis (Mont.) Váňa et L.Söderstr., Phytotaxa 112 (1): 9, 2013 (Váňa et al. 2013g). Bas.: Jungermannia sandwicensis Mont., Ann. Sci. Nat. Bot. (sér. 2) 19: 249, 1843 (Montagne 1843).

*** Fuscocephaloziopsis crassifolia (Lindenb. et Gottsche) Váňa et L.Söderstr., Phytotaxa 112 (1): 10, 2013 (Váňa et al. 2013g). Bas.: Jungermannia crassifolia Lindenb. et Gottsche, Syn. Hepat. 5: 685, 1847 (Gottsche et al. 1847).

*** Fuscocephaloziopsis gollanii (Steph.) Váňa et L.Söderstr., Phytotaxa 112 (1): 10, 2013 (Váňa et al. 2013g). Bas.: Cephalozia gollanii Steph., Bull. Herb. Boissier (sér. 2) 8 (6): 428 (304), 1908 (Stephani 1908g).

*** Fuscocephaloziopsis leucantha (Spruce) Váňa et L.Söderstr., Phytotaxa 112 (1): 10, 2013 (Váňa et al. 2013g). Bas.: Cephalozia leucantha Spruce, Cephalozia: 68, 1882 (Spruce 1882).

*** Fuscocephaloziopsis loitlesbergeri (Schiffn.) Váňa et L.Söderstr., Phytotaxa 112 (1): 10, 2013 (Váňa et al. 2013g). Bas.: Cephalozia loitlesbergeri Schiffn., Österr. Bot. Z. 62 (1): 10, 1912 (Schiffner 1912b).

*** Fuscocephaloziopsis lunulifolia (Dumort.) Váňa et L.Söderstr., Phytotaxa 112 (1): 10, 2013 (Váňa et al. 2013g). Bas.: Jungermannia lunulifolia Dumort., Syll. Jungerm. Europ.: 61, 1831 (Dumortier 1831). ^39^

*** Fuscocephaloziopsis macrostachya (Kaal.) Váňa et L.Söderstr., Phytotaxa 112 (1): 10, 2013 (Váňa et al. 2013g). Bas.: Cephalozia macrostachya Kaal., Rev. Bryol. 29 (1): 8, 1902 (Kaalaas 1902).

*** Fuscocephaloziopsis macrostachya subsp. australis (R.M.Schust.) Váňa et L.Söderstr., Phytotaxa 112 (1): 10, 2013 (Váňa et al. 2013g). Bas.: Cephalozia macrostachya subsp. australis R.M.Schust., Hepat. Anthocerotae N. Amer. 3: 754, 1974 (Schuster 1974).

*** Fuscocephaloziopsis macrostachya subsp. macrostachya var. spiniflora (Schiffn.) Váňa et L.Söderstr., Phytotaxa 112 (1): 10, 2013 (Váňa et al. 2013g). Bas.: Cephalozia spiniflora Schiffn., Hedwigia 54 (6): 323, 1914 (Schiffner 1914a).

*** Fuscocephaloziopsis monticola (J.D.Godfrey) Váňa et L.Söderstr., Phytotaxa 112 (1): 11, 2013 (Váňa et al. 2013g). Bas.: Schofieldia monticola J.D.Godfrey, Bryologist 79 (3): 315, 1976 (Godfrey 1976).

*** Fuscocephaloziopsis pachycaulis (R.M.Schust.) Váňa et L.Söderstr., Phytotaxa 112 (1): 11, 2013 (Váňa et al. 2013g). Bas.: Cephalozia pachycaulis R.M.Schust., Bryologist 96 (4): 623, 1993 (Schuster 1993b).

*** Fuscocephaloziopsis pleniceps (Austin) Váňa et L.Söderstr., Phytotaxa 112 (1): 11, 2013 (Váňa et al. 2013g). Bas.: Jungermannia pleniceps Austin, Proc. Acad. Nat. Sci. Philadelphia 21: 222, 1869 (Austin 1869).

** Fuscocephaloziopsis pleniceps var. caroliniana (R.M.Schust.) Váňa et L.Söderstr., Phytotaxa 112 (1): 11, 2013 (Váňa et al. 2013g). Bas.: Cephalozia pleniceps var. caroliniana R.M.Schust., Hepat. Anthocerotae N. Amer. 3: 780, 1974 (Schuster 1974).

*** Fuscocephaloziopsis pulvinata (Steph.) Fulford, Mem. New York Bot. Gard. 11 (3): 355, 1968 (Fulford 1968). Bas.: Alobiella pulvinata Steph., Bull. Herb. Boissier (sér. 2) 8 (8): 572 (356), 1908 (Stephani 1908e).

* Fuscocephaloziopsis schusteri (Sushil K.Singh et D.K.Singh) Váňa, Phytotaxa 183 (4): 291, 2014 (Váňa et al. 2014a). Bas.: Cephalozia schusteri Sushil K.Singh et D.K.Singh, Lindbergia 32 (1): 1, 2007 (Singh and Singh 2007a).

*** Fuscocephaloziopsis subintegra Gradst. et Váňa, Cryptog. Bryol. 25 (3): 274, 2004 (Parolly et al. 2004).

*** Fuscocephaloziopsis zoopsioides (Horik.) Váňa et L.Söderstr., Phytotaxa 112 (1): 11, 2013 (Váňa et al. 2013g). Bas.: Cephalozia zoopsioides Horik., J. Sci. Hiroshima Univ., Ser. B, Div. 2, Bot. 2: 178, 1934 (Horikawa 1934).

*** **Nowellia Mitt.**, Nat. hist. Azores: 321, 1870 (Mitten 1870).

** **sect. Acronowellia R.M.Schust.**, Hepat. Anthocerotae N. Amer. 3: 816, 1974 (Schuster 1974).

*** Nowellia reedii H.Rob., Bryologist 73 (1): 150, 1970 (Robinson 1970).

*** Nowellia yunckeri Fulford, Mem. New York Bot. Gard. 11 (3): 329, 1968 (Fulford 1968).

** **sect. Metanowellia (Grolle) R.M.Schust.**, Hepat. Anthocerotae N. Amer. 3: 816, 1974 (Schuster 1974). Bas.: Nowellia subg. Metanowellia Grolle, J. Hattori Bot. Lab. 31: 33, 1968 (Grolle 1968b).

*** Nowellia borneensis (De Not.) Schiffn., Hepat. (Engl.-Prantl): 98, 1893 (Schiffner 1893b). Bas.: Jungermannia curvifolia var. borneensis De Not., Epat. Borneo: 19, 1874 (De Notaris 1874).

*** Nowellia dominicensis Steph., Sp. Hepat. (Stephani) 6: 443, 1924 (Stephani 1924).

*** Nowellia evansii Grolle, J. Hattori Bot. Lab. 31: 33, 1968 (Grolle 1968b).

*** Nowellia langii Pearson, J. Linn. Soc., Bot. 46 (305): 25, 1922 (Pearson 1922b).

*** Nowellia pusilla Grolle, J. Hattori Bot. Lab. 31: 37, 1968 (Grolle 1968b).

*** Nowellia wrightii (Gottsche ex Spruce) Steph. ex Duss, Enum. musc. Antilles franç., Hép.: 21, 1903 (Duss 1903). Bas.: Cephalozia wrightii Gottsche ex Spruce, J. Linn. Soc., Bot. 30 (210): 354, 1895 (Gepp 1895b).

** **sect. Nowellia**

*** Nowellia aciliata (P.C.Chen et P.C.Wu) Mizut., Hikobia 11: 469, 1994 (Mizutani 1994). Bas.: Nowellia curvifolia var. aciliata P.C.Chen et P.C.Wu, Obs. fl. Hwangs.: 6, 1965 (Chen and Wu 1965).

*** Nowellia curvifolia (Dicks.) Mitt., Nat. hist. Azores: 321, 1870 (Mitten 1870). Bas.: Jungermannia curvifolia Dicks., Fasc. Pl. Crypt. Brit. 2: 15, 1790 (Dickson 1790).

######### ** Odontoschismatoideae H.Buch ex Grolle

*** **Odontoschisma (Dumort.) Dumort.**, Recueil Observ. Jungerm.: 19, 1835 (Dumortier 1835). Bas.: Pleuroschisma sect. Odontoschisma Dumort., Syll. Jungerm. Europ.: 68, 1831 (Dumortier 1831).

** **sect. Cladopodiella (H.Buch) Gradst., S.C.Aranda et Vanderp.**, Taxon 63 (5): 1017, 2014 (Aranda et al. 2014). Bas.: Cladopodiella H.Buch, Memoranda Soc. Fauna Fl. Fennica 1: 89, 1927 (Buch 1927).

*** Odontoschisma francisci (Hook.) L.Söderstr. et Váňa, Phytotaxa 112 (1): 12, 2013 (Váňa et al. 2013g). Bas.: Jungermannia francisci Hook., Brit. Jungermann.: tab. 49, 1813 (Hooker 1813).

** **sect. Denudata R.M.Schust.**, Hepat. Anthocerotae N. Amer. 3: 833, 1974 (Schuster 1974).

*** Odontoschisma brasiliense Steph., Bull. Herb. Boissier (sér. 2) 8 (8): 585 (369), 1908 (Stephani 1908e).

** Odontoschisma cleefii Gradst., S.C.Aranda et Vanderp., Taxon 63 (5): 1017, 2014 (Aranda et al. 2014).

*** Odontoschisma denudatum (Mart.) Dumort., Recueil Observ. Jungerm.: 19, 1835 (Dumortier 1835). Bas.: Jungermannia scalaris var. denudata Mart., Fl. crypt. erlang.: 183, 1817 (Martius 1817).

** Odontoschisma denudatum subsp. naviculare (Steph.) Gradst., S.C.Aranda et Vanderp., Taxon 63 (5): 1019, 2014 (Aranda et al. 2014). Bas.: Jamesoniella navicularis Steph., Sp. Hepat. (Stephani) 6: 101, 1917 (Stephani 1917a).

** Odontoschisma denudatum subsp. sandvicense (Ångstr.) Gradst., S.C.Aranda et Vanderp., Taxon 63 (5): 1019, 2014 (Aranda et al. 2014). Bas.: Sphagnoecetis sandvicensis Ångstr., Öfvers. Kongl. Vetensk.-Akad. Förh. 29 (4): 22, 1872 (Ångström 1872).

*** Odontoschisma elongatum (Lindb.) A.Evans, Rhodora 14 (157): 13, 1912 (Evans 1912b). Bas.: Odontoschisma denudatum f. elongatum Lindb., Helsingf. Dagbl. 1874 (45, 16 Feb): 2, 1874 (Lindberg 1874b).

*** Odontoschisma engelii Gradst. et Burghardt, Fieldiana, Bot. (n.ser.) 47: 194, 2008 (Gradstein and Burghardt 2008).

*** Odontoschisma longiflorum (Taylor) Trevis., Mem. Reale Ist. Lombardo Sci. (Ser. 3), C. Sci. Mat. 4 (13): 419, 1877 (Trevisan 1877). Bas.: Sphagnoecetis longiflora Taylor, London J. Bot. 5: 281, 1846 (Taylor 1846a).

*** Odontoschisma macounii (Austin) Underw., Bull. Illinois State Lab. Nat. Hist. 2 (1): 92, 1884 (Underwood 1884). Bas.: Sphagnoecetis macounii Austin, Bull. Torrey Bot. Club 3 (3): 13, 1872 (Austin 1872).

*** Odontoschisma portoricense (Hampe et Gottsche) Steph., Hedwigia 27 (11/12): 296, 1888 (Stephani 1888c). Bas.: Sphagnoecetis portoricensis Hampe et Gottsche, Linnaea 25 (3): 343, 1852 [1853] (Hampe and Gottsche 1852).

** Odontoschisma pseudogrosseverrucosum Gradst., S.C.Aranda et Vanderp., Taxon 63 (5): 1019, 2014 (Aranda et al. 2014).

*** Odontoschisma purpuratum Herzog, Trans. Brit. Bryol. Soc. 1 (4): 297, 1950 (Herzog 1950a).

*** Odontoschisma soratamum Fulford, Mem. New York Bot. Gard. 11 (3): 338, 1968 (Fulford 1968).

*** Odontoschisma variabile (Lindenb. et Gottsche) Trevis., Mem. Reale Ist. Lombardo Sci. (Ser. 3), C. Sci. Mat. 4 (13): 419, 1877 (Trevisan 1877). Bas.: Sphagnoecetis variabilis Lindenb. et Gottsche, Syn. Hepat. 5: 688, 1847 (Gottsche et al. 1847).

** Odontoschisma zhui Gradst., S.C.Aranda et Vanderp., Taxon 63 (5): 1020, 2014 (Aranda et al. 2014).

** **sect. Iwatsukia (N.Kitag.) Gradst., S.C.Aranda et Vanderp.**, Phytotaxa 162 (4): 232, 2014 (Gradstein et al. 2014b). Bas.: Iwatsukia N.Kitag., J. Hattori Bot. Lab. 27: 178, 1964 (Kitagawa 1964).

*** Odontoschisma bifidum (Fulford) Gradst., S.C.Aranda et Vanderp., Phytotaxa 162 (4): 232, 2014 (Gradstein et al. 2014b). Bas.: Cladomastigum bifidum Fulford, Acta Bot. Venez. 2 (5/8): 80, 1967 (Fulford 1967).

*** Odontoschisma jishibae (Steph.) L.Söderstr. et Váňa, Phytotaxa 112 (1): 13, 2013 (Váňa et al. 2013g). Bas.: Cephalozia jishibae Steph., Sp. Hepat. (Stephani) 6: 437, 1924 (Stephani 1924).

*** Odontoschisma spinosum (Fulford) Gradst., S.C.Aranda et Vanderp., Phytotaxa 162 (4): 232, 2014 (Gradstein et al. 2014b). Bas.: Cladomastigum spinosum Fulford, Mem. New York Bot. Gard. 23: 840, 1972 (Fulford 1972).

** **sect. Neesia Gradst., S.C.Aranda et Vanderp.**, Taxon 63 (5): 1017, 2014 (Aranda et al. 2014).

*** Odontoschisma fluitans (Nees) L.Söderstr. et Váňa, Phytotaxa 112 (1): 12, 2013 (Váňa et al. 2013g). Bas.: Jungermannia fluitans Nees, Flora 6 (2): 30, 1823 (Link 1823).

** **sect. Odontoschisma**

*** Odontoschisma grosseverrucosum Steph., Bull. Herb. Boissier (sér. 2) 8 (8): 593 (377), 1908 (Stephani 1908e).

*** Odontoschisma sphagni (Dicks.) Dumort., Recueil Observ. Jungerm.: 19, 1835 (Dumortier 1835). Bas.: Jungermannia sphagni Dicks., Fasc. Pl. Crypt. Brit. 1: 6, 1785 (Dickson 1785).

** Odontoschisma steyermarkii Gradst. et Ilk.-Borg., Nova Hedwigia 100 (1/2): 38, 2015 [2014 online] (Gradstein and Ilkiu-Borges 2015).

***Incertae sedis***

* Odontoschisma obcordatum (Spruce) Steph., Bull. Herb. Boissier (sér. 2) 8 (8): 586 (370), 1908 (Stephani 1908e). Bas.: Cephalozia obcordata Spruce, Cephalozia: 61, 1882 (Spruce 1882). ^40^

######### ** Schiffnerioideae R.M.Schust.

** **Schiffneria Steph.**, Österr. Bot. Z. 44 (1): 1, 1894 (Stephani 1894c).

*** Schiffneria hyalina Steph., Österr. Bot. Z. 44 (1): 1, 1894 (Stephani 1894c).

######### ** Trabacelluloideae R.M.Schust.

** **Haesselia Grolle et Gradst.**, J. Hattori Bot. Lab. 64: 327, 1988 (Grolle and Gradstein 1988).

*** Haesselia acuminata Gradst., Trop. Bryol. 1: 30, 1989 (Gradstein and Florschütz-de Waard 1989).

*** Haesselia roraimensis Grolle et Gradst., J. Hattori Bot. Lab. 64: 327, 1988 (Grolle and Gradstein 1988).

** **Trabacellula Fulford**, Acta Bot. Venez. 2 (5/8): 86, 1967 (Fulford 1967).

*** Trabacellula tumidula Fulford, Acta Bot. Venez. 2 (5/8): 86, 1967 (Fulford 1967).

######## *** Cephaloziellaceae Douin

by J. Váňa

The treatment of Cephaloziellaceae follows what was outlined in Váňa et al. (2013e). Some nomenclatural and taxonomic notes can also be found in Söderström et al. (2013a) and Váňa et al. (2013b, 2013l). The placement of Phycolepidozia follows Gradstein et al. (2014a).

*** **Allisoniella E.A.Hodgs.**, Trans. Roy. Soc. New Zealand, Bot. 3 (4): 80, 1965 (Hodgson 1965).

** **sect. Allisoniella**

*** Allisoniella nigra (Rodway) R.M.Schust., Nova Hedwigia 22: 137, 1971 [1972] (Schuster 1971b). Bas.: Sphenolobus niger Rodway, Tasm. Bryoph.: 33, 1917 (Rodway 1917b).

** Allisoniella nigra var. acutiloba J.J.Engel, Novon 17 (3): 313, 2007 (Engel 2007).

** Allisoniella nigra subsp. novaezelandiae R.M.Schust., Nova Hedwigia 22: 143, 1971 [1972] (Schuster 1971b).

*** Allisoniella recurva R.M.Schust., Nova Hedwigia 22: 146, 1971 [1972] (Schuster 1971b).

*** Allisoniella subbipartita (C.Massal.) R.M.Schust. et J.J.Engel, Nova Hedwigia 22: 147, 1971 [1972] (Schuster 1971b). Bas.: Cephalozia subbipartita C.Massal., Nuovo Giorn. Bot. Ital. 17 (3): 235, 1885 (Massalongo 1885).

*** Allisoniella tasmanica R.M.Schust., Nova Hedwigia 22: 145, 1971 [1972] (Schuster 1971b).

** **sect. Protomarsupella (R.M.Schust.) R.M.Schust.**, Nova Hedwigia 22: 150, 1971 (Schuster 1971b). Bas.: Protomarsupella R.M.Schust., Rev. Bryol. Lichénol. 34 (1/2): 264, 1966 (Schuster 1966b).

*** Allisoniella scottii (R.M.Schust.) R.M.Schust., Nova Hedwigia 22: 151, 1971 [1972] (Schuster 1971b). Bas.: Protomarsupella scottii R.M.Schust., Rev. Bryol. Lichénol. 34 (1/2): 267, 1966 (Schuster 1966b).

*** **Amphicephalozia R.M.Schust.**, Nova Hedwigia 22: 131, 1971 [1972] (Schuster 1971b).

*** Amphicephalozia africana Váňa et M.Wigginton, J. Bryol. 30 (1): 55, 2008 (Váňa and Wigginton 2008).

*** Amphicephalozia amplexicaulis R.M.Schust., Nova Hedwigia 22: 133, 1971 [1972] (Schuster 1971b).

*** Amphicephalozia geisslerae Pócs et Váňa, Polish Bot. J. 46 (2): 145, 2001 (Pócs and Váňa 2001).

*** **Anastrophyllopsis (R.M.Schust.) Váňa et L.Söderstr.**, Phytotaxa 81 (1): 15, 2013 (Váňa et al. 2013k). Bas.: Anastrophyllum sect. Anastrophyllopsis R.M.Schust., Beih. Nova Hedwigia 119: 310, 2002 (Schuster 2002b).

*** Anastrophyllopsis involutifolia (Mont. ex Gottsche, Lindenb. et Nees) Váňa et L.Söderstr., Phytotaxa 81 (1): 15, 2013 (Váňa et al. 2013k). Bas.: Jungermannia involutifolia Mont. ex Gottsche, Lindenb. et Nees, Syn. Hepat. 1: 81, 1844 (Gottsche et al. 1844).

*** Anastrophyllopsis revoluta (Steph.) Váňa et L.Söderstr., Phytotaxa 81 (1): 16, 2013 (Váňa et al. 2013k). Bas.: Anastrophyllum revolutum Steph., Hedwigia 32 (3): 139, 1893 (Stephani 1893b).

*** Anastrophyllopsis subcomplicata (Lehm. et Lindenb.) Váňa et L.Söderstr., Phytotaxa 81 (1): 16, 2013 (Váňa et al. 2013k). Bas.: Jungermannia subcomplicata Lehm. et Lindenb., Nov. Stirp. Pug. 7: 4, 1838 (Lehmann 1838).

** **Cephalojonesia Grolle**, Rev. Bryol. Lichénol. 37 (4): 763, 1970 [1971] (Grolle and Vanden Berghen 1970).

*** Cephalojonesia incuba Grolle et Vanden Berghen, Rev. Bryol. Lichénol. 37 (4): 764, 1970 [1971] (Grolle and Vanden Berghen 1970).

*** Cephalojonesia incuba subsp. mexicana Burghardt, Gradst. et Váňa, J. Hattori Bot. Lab. 100: 35, 2006 (Burghardt et al. 2006).

** **Cephalomitrion R.M.Schust.**, Nova Hedwigia 61 (3/4): 550, 1995 (Schuster 1995b).

*** Cephalomitrion aterrimum (Steph.) R.M.Schust., Nova Hedwigia 61 (3/4): 554, 1995 (Schuster 1995b). Bas.: Cephalozia aterrima Steph., Bull. Herb. Boissier (sér. 2) 8 (7): 501 (331), 1908 (Stephani 1908f).

*** **Cephaloziella (Spruce) Schiffn.**, Hepat. (Engl.-Prantl): 98, 1893 (Schiffner 1893b) nom. conserv. Bas.: Cephalozia subg. Cephaloziella Spruce, Cephalozia: 62, 1882 (Spruce 1882). ^41^

** **subg. Cephaloziella**

*** Cephaloziella aenigmatica R.M.Schust., Nova Hedwigia 63 (1/2): 20, 1996 (Schuster 1996d).

*** Cephaloziella anthelioides S.W.Arnell, Bot. Not. 105: 322, 1952 (Arnell 1952a).

*** Cephaloziella arctogena (R.M.Schust.) Konstant., Arctoa 3: 126, 1994 (Konstantinova and Vasil’ev 1994). Bas.: Cephaloziella rubella var. arctogena R.M.Schust., Hepat. Anthocerotae N. Amer. 4: 125, 1980 (Schuster 1980c).

** Cephaloziella arenaria (Steph.) R.M.Schust., J. Hattori Bot. Lab. 26: 280, 1963 (Schuster 1963b). Bas.: Cephalozia arenaria Steph., Bull. Herb. Boissier (sér. 2) 8 (7): 504 (334), 1908 (Stephani 1908f).

*** Cephaloziella aspericaulis Jørg., Bergens Mus. Skr. (n.ser.) 16: 197, 1934 (Jørgensen 1934).

*** Cephaloziella baumgartneri Schiffn., Verh. K.K. Zool.-Bot. Ges. Wien 56 (3): 273, 1906 (Schiffner 1906c).

** Cephaloziella breviperianthia C.Gao, Fl. Hepat. Chin. Boreali-Orient.: 208, 1981 (Gao and Chang 1981).

** Cephaloziella brinkmannii Douin, Mém. Soc. Bot. France 29: 75, 1920 (Douin 1920).

*** Cephaloziella capensis (Sim) S.W.Arnell, Bot. Not. 105: 326, 1952 (Arnell 1952a). Bas.: Cephalozia capensis Sim, Trans. Roy. Soc. South Africa 15 (1): 87, 1926 (Sim 1926).

*** Cephaloziella capillaris (Steph.) Douin, Mém. Soc. Bot. France 29: 59, 1920 (Douin 1920). Bas.: Cephalozia capillaris Steph., Bull. Herb. Boissier (sér. 2) 8 (7): 496 (326), 1908 (Stephani 1908f).

*** Cephaloziella crassigyna (R.M.Schust.) R.M.Schust., Nova Hedwigia 61 (3/4): 556, 1995 (Schuster 1995b). Bas.: Cephaloziella aterrima var. crassigyna R.M.Schust., Nova Hedwigia 22: 211, 1971 [1972] (Schuster 1971b).

*** Cephaloziella crispata N.Kitag., J. Hattori Bot. Lab. 32: 301, 1969 (Kitagawa 1969c).

*** Cephaloziella densifolia R.M.Schust., Nova Hedwigia 22: 199, 1971 [1972] (Schuster 1971b).

** Cephaloziella densifolia var. dubia R.M.Schust., Nova Hedwigia 22: 202, 1971 [1972] (Schuster 1971b).

*** Cephaloziella divaricata (Sm.) Schiffn., Hepat. (Engl.-Prantl): 99, 1893 (Schiffner 1893b). Bas.: Jungermannia divaricata Sm., Engl. Bot. 10: tab. 719, 1800 (Smith and Sowerby 1800).

** Cephaloziella divaricata var. scabra (M.Howe) Haynes, Bryologist 12 (4): 68, 1909 (Haynes 1909). Bas.: Cephalozia divaricata var. scabra M.Howe, Mem. Torrey Bot. Club 7: 129, 1899 (Howe 1899). ^42^

** Cephaloziella dusenii Steph., Bih. Kongl. Svenska Vetensk.-Akad. Handl. 26 (III, 6): 49, 1900 (Stephani 1900b).

*** Cephaloziella elachista (J.B.Jack ex Gottsche et Rabenh.) Schiffn., Sitzungsber. deutsch. naturwiss.-med. Vereins Böhmen “Lotos” Prag 48: 336, 1900 (Schiffner 1900d). Bas.: Jungermannia elachista J.B.Jack ex Gottsche et Rabenh., Hepat. Eur., Leberm. 58-59: no. 574, 1873 (Gottsche and Rabenhorst 1873a).

** Cephaloziella elachista var. spinophylla (C.Gao) C.Gao, Fl. Bryoph. Sin. 9: 181, 2003 (Gao 2003). Bas.: Cephaloziella spinophylla C.Gao, Fl. Hepat. Chin. Boreali-Orient.: 208, 1981 (Gao and Chang 1981).

** Cephaloziella elegans (Heeg) Schiffn., Sitzungsber. deutsch. naturwiss.-med. Vereins Böhmen “Lotos” Prag 48: 336, 1900 (Schiffner 1900d). Bas.: Cephalozia elegans Heeg, Rev. Bryol. 20 (5): 82, 1893 (Heeg 1893).

*** Cephaloziella exigua R.M.Schust., Nova Hedwigia 63 (1/2): 49, 1996 (Schuster 1996d).

*** Cephaloziella exiliflora (Taylor) Douin, Mém. Soc. Bot. France 29: 72, 1920 (Douin 1920). Bas.: Jungermannia exiliflora Taylor, London J. Bot. 5: 279, 1846 (Taylor 1846a).

** Cephaloziella fragillima (Spruce) Fulford, Mem. New York Bot. Gard. 11 (4): 409, 1976 (Fulford 1976). Bas.: Cephalozia fragillima Spruce, Mem. Torrey Bot. Club 1 (3): 131, 1890 (Spruce 1890).

*** Cephaloziella garsidei S.W.Arnell, Rev. Bryol. Lichénol. 23 (1/2): 173, 1954 (Arnell 1954a).

*** Cephaloziella grandiretis (R.M.Schust.) R.M.Schust., Nova Hedwigia 63 (1/2): 35, 1996 (Schuster 1996d). Bas.: Cephaloziella byssacea subsp. grandiretis R.M.Schust., Nova Hedwigia 22: 195, 1971 [1972] (Schuster 1971b).

*** Cephaloziella grimsulana (J.B.Jack ex Gottsche et Rabenh.) Lacout., Hépat. France: 52, 1905 (Lacouture 1905). Bas.: Jungermannia grimsulana J.B.Jack ex Gottsche et Rabenh., Hepat. Eur., Leberm. 53–55: no. 526, 1872 (Gottsche and Rabenhorst 1872).

*** Cephaloziella hampeana (Nees) Schiffn. ex Loeske, Moosfl. Harz.: 92, 1903 (Loeske 1903). Bas.: Jungermannia hampeana Nees, Naturgesch. Eur. Leberm. 3: 560, 1838 (Nees 1838b).

** Cephaloziella hebridensis Steph., Hedwigia 32 (5): 316, 1893 (Stephani 1893d).

*** Cephaloziella herzogiana (Pandé et K.P.Srivast.) Udar et D.Kumar, Geophytology 6 (1): 45, 1976 (Udar and Kumar 1976). Bas.: Cephalozia herzogiana Pandé et K.P.Srivast., Feddes Repert. Spec. Nov. Regni Veg. 58: 75, 1955 (Pandé and Srivastava 1955).

** Cephaloziella heteroica (C.M.Cooke) Douin, Mém. Soc. Bot. France 29: 76, 1920 (Douin 1920). Bas.: Cephalozia heteroica C.M.Cooke, Trans. Connecticut Acad. Arts 12 (1): 38, 1904 (Cooke 1904).

** Cephaloziella hyalina Douin, Mém. Soc. Bot. France 29: 77, 1920 (Douin 1920).

* Cephaloziella hyalina var. rappii (Douin) R.M.Schust., Hepat. Anthocerotae N. Amer. 4: 56, 1980 (Schuster 1980c). Bas.: Cephaloziella rappii Douin, Mém. Soc. Bot. France 29: 77, 1920 (Douin 1920).

*** Cephaloziella inaequalis R.M.Schust., Nova Hedwigia 22: 186, 1971 [1972] (Schuster 1971b).

*** Cephaloziella invisa R.M.Schust., Nova Hedwigia 63 (1/2): 30, 1996 (Schuster 1996d).

** Cephaloziella kilohanensis (C.M.Cooke) Douin, Mém. Soc. Bot. France 29: 85, 1920 (Douin 1920). Bas.: Cephalozia kilohanensis C.M.Cooke, Trans. Connecticut Acad. Arts 12 (1): 37, 1904 (Cooke 1904).

*** Cephaloziella longii Váňa, Folia Geobot. Phytotax. 27 (2): 193, 1992 (Váňa 1992).

*** Cephaloziella lycopodioides (Sim) S.W.Arnell, Bot. Not. 105: 321, 1952 (Arnell 1952a). Bas.: Cephalozia lycopodioides Sim, Trans. Roy. Soc. South Africa 15 (1): 85, 1926 (Sim 1926).

** Cephaloziella mammillifera R.M.Schust. et Damsh., Phytologia 63 (5): 327, 1987 (Schuster and Damsholt 1987).

*** Cephaloziella massalongi (Spruce) Müll.Frib., Lebermoose 2 (17): 191, 1913 (Müller 1913a). Bas.: Cephalozia massalongi Spruce, Cephalozia: 71, 1882 (Spruce 1882).

*** Cephaloziella muelleriana R.M.Schust., Nova Hedwigia 63 (1/2): 24, 1996 (Schuster 1996d).

*** Cephaloziella natalensis (Sim) S.W.Arnell, Hepat. South Africa: 338, 1963 (Arnell 1963b). Bas.: Cephalozia natalensis Sim, Trans. Roy. Soc. South Africa 15 (1): 86, 1926 (Sim 1926).

** Cephaloziella nicholsonii Douin, Rev. Bryol. 40 (6): 81, 1913 (Douin 1913b).

*** Cephaloziella nothogena R.M.Schust., Nova Hedwigia 63 (1/2): 37, 1996 (Schuster 1996d).

** Cephaloziella obtusilobula R.M.Schust., Hepat. Anthocerotae N. Amer. 4: 108, 1980 (Schuster 1980c).

** Cephaloziella patulifolia (Steph.) Douin, Mém. Soc. Bot. France 29: 70, 1920 (Douin 1920). Bas.: Cephalozia patulifolia Steph., Bull. Herb. Boissier (sér. 2) 8 (7): 509 (339), 1908 (Stephani 1908f).

*** Cephaloziella phyllacantha (C.Massal. et Carestia) Müll.Frib., Lebermoose 2 (17): 194, 1913 (Müller 1913a). Bas.: Anthelia phyllacantha C.Massal. et Carestia, Nuovo Giorn. Bot. Ital. 12 (4): 340, 1880 (Massalongo and Carestia 1880).

*** Cephaloziella polystratosa (R.M.Schust. et Damsh.) Konstant., Bot. Zhurn. (Moscow & Leningrad) 85 (10): 127, 2000 (Konstantinova 2000). Bas.: Cephaloziella byssacea var. polystratosa R.M.Schust. et Damsh., Phytologia 63 (5): 327, 1987 (Schuster and Damsholt 1987).

*** Cephaloziella pseudocrassigyna R.M.Schust. ex J.J.Engel, Novon 17 (3): 313, 2007 (Engel 2007).

*** Cephaloziella pulcherrima R.M.Schust., Nova Hedwigia 22: 203, 1971 [1972] (Schuster 1971b).

** Cephaloziella pulcherrima subsp. sphagnicola R.M.Schust., Nova Hedwigia 22: 205, 1971 [1972] (Schuster 1971b).

** Cephaloziella pungens Steph. ex Fulford, Mem. New York Bot. Gard. 11 (4): 410, 1976 (Fulford 1976).

*** Cephaloziella rubella (Nees) Warnst., Krypt.-Fl. Brandenburg, Leber- & Torfmoose: 231, 1902 (Warnstorf 1902). Bas.: Jungermannia rubella Nees, Naturgesch. Eur. Leberm. 2: 236, 1836 (Nees 1836).

*** Cephaloziella schelpei S.W.Arnell, Bot. Not. 110 (1): 18, 1957 (Arnell 1957a).

*** Cephaloziella spegazziniana (C.Massal.) Douin, Mém. Soc. Bot. France 29: 69, 1920 (Douin 1920). Bas.: Cephalozia spegazziniana C.Massal., Nuovo Giorn. Bot. Ital. 17 (3): 234, 1885 (Massalongo 1885).

*** Cephaloziella spinicaulis Douin, Mém. Soc. Bot. France 29: 62, 1920 (Douin 1920).

*** Cephaloziella spinigera (Lindb.) Jørg., Bergens Mus. Skr. (n.ser.) 16: 189, 1934 (Jørgensen 1934). Bas.: Cephalozia spinigera Lindb., Musci Scand.: 4, 1879 (Lindberg 1879).

*** Cephaloziella stellulifera (Taylor ex Carrington et Pearson) Croz., Rev. Bryol. 30 (2): 31, 1903 (Crozals 1903a). Bas.: Jungermannia stellulifera Taylor ex Carrington et Pearson, Hepat. Brit. Exsicc. Fasc. I: no. 32, 1878 (Carrington and Pearson 1878).

*** Cephaloziella stephanii Schiffn. ex Douin, Mém. Soc. Bot. France 29: 85, 1920 (Douin 1920).

** Cephaloziella stolonifera R.M.Schust., Phytologia 39 (6): 426, 1978 (Schuster 1978b).

** Cephaloziella subtilis (Lindenb. et Gottsche) Steph., Hedwigia 32 (5): 318, 1893 (Stephani 1893d). Bas.: Jungermannia subtilis Lindenb. et Gottsche, Syn. Hepat. 5: 685, 1847 (Gottsche et al. 1847).

*** Cephaloziella sumatrana Schiffn. ex Douin, Mém. Soc. Bot. France 29: 59, 1920 (Douin 1920).

*** Cephaloziella tabularis S.W.Arnell, Bot. Not. 105: 318, 1952 (Arnell 1952a).

*** Cephaloziella transvaalensis S.W.Arnell, Bot. Not. 110 (1): 19, 1957 (Arnell 1957a).

*** Cephaloziella triplicata S.W.Arnell, Bot. Not. 115: 203, 1962 (Arnell 1962a).

*** Cephaloziella umtaliensis S.W.Arnell, Bot. Not. 110 (1): 20, 1957 (Arnell 1957a).

*** Cephaloziella uncinata R.M.Schust., Meddel. Grønland 199 (1): 316, 1974 (Schuster and Damsholt 1974).

** Cephaloziella uncinata var. brevigyna R.M.Schust. et Damsh., Phytologia 63 (5): 327, 1987 (Schuster and Damsholt 1987).

** Cephaloziella uncinata var. sphagnicola R.M.Schust., Meddel. Grønland 199 (1): 323, 1974 (Schuster and Damsholt 1974).

*** Cephaloziella vaginans Steph., Wiss. Ergebn. Deut. Zentr.-Afr. Exped. (1907-08), Bot. 2: 119, 1911 (Stephani 1911a).

*** Cephaloziella varians (Gottsche) Steph., Wiss. Ergebn. Schwed. Südpolar-Exped. [1901–1903] 4 (1): 4, 1905 (Stephani 1905e). Bas.: Jungermannia varians Gottsche, Int. Polarforsch., Deutsch. Exped. 2: 452, 1890 (Gottsche 1890).

*** Cephaloziella verrucosa Steph., Hedwigia 32 (5): 318, 1893 (Stephani 1893d).

* Cephaloziella villaumei (Steph.) Váňa, Phytotaxa 112 (1): 3, 2013 (Váňa et al. 2013e). Bas.: Cephalozia villaumei Steph., Sp. Hepat. (Stephani) 6: 437, 1924 (Stephani 1924). ^43^

** Cephaloziella violacea Schljakov, Novosti Sist. Nizš. Rast. 15: 242, 1978 (Shliakov 1978).

** Cephaloziella welwitschii (Steph.) Douin, Mém. Soc. Bot. France 29: 58, 1920 (Douin 1920). Bas.: Cephalozia welwitschii Steph., Bull. Herb. Boissier (sér. 2) 8 (6): 432 (308), 1908 (Stephani 1908g).

** **subg. Dichiton (Mont.) Müll.Frib.**, Lebermoose 2 (27): 787, 1916 (Müller 1916). Bas.: Dichiton Mont., Syll. Gen. Sp. Crypt.: 52, 1856 (Montagne 1856b).

*** Cephaloziella calyculata (Durieu et Mont.) Müll.Frib., Lebermoose 2 (27): 787, 1916 (Müller 1916). Bas.: Jungermannia calyculata Durieu et Mont., Ann. Sci. Nat. Bot. (sér. 3) 11: 34, 1849 (Montagne 1849).

*** Cephaloziella integerrima (Lindb.) Warnst., Krypt.-Fl. Brandenburg, Leber- & Torfmoose: 232, 1902 (Warnstorf 1902). Bas.: Cephalozia integerrima Lindb., Acta Soc. Sci. Fenn. 10: 502, 1875 (Lindberg 1875).

** **subg. Evansia (Douin et Schiffn.) Müll.Frib.**, Lebermoose 2 (27): 787, 1916 (Müller 1916). Bas.: Evansia Douin et Schiffn., Rev. Bryol. 40 (5): 66, 1913 (Douin 1913a).

*** Cephaloziella antillana (Besch. et Spruce) Fulford, Mem. New York Bot. Gard. 11 (4): 405, 1976 (Fulford 1976). Bas.: Cephalozia antillana Besch. et Spruce, Bull. Soc. Bot. France (Congr. Bot.) 36: clxxxiii, 1889 [1890] (Bescherelle and Spruce 1889).

*** Cephaloziella dentata (Raddi) Steph., Bull. Herb. Boissier 5 (2): 78, 1897 (Stephani 1897b). Bas.: Jungermannia dentata Raddi, Jungermanniogr. Etrusca: 21, 1818 (Raddi 1818a).

*** Cephaloziella hirta (Steph.) R.M.Schust., J. Hattori Bot. Lab. 26: 280, 1963 (Schuster 1963b). Bas.: Cephalozia hirta Steph., Bull. Herb. Boissier (sér. 2) 8 (8): 561 (345), 1908 (Stephani 1908e).

** Cephaloziella squarrosula (Trevis.) Steph., Hedwigia 32 (5): 318, 1893 (Stephani 1893d). Bas.: Cephalozia squarrosula Trevis., Mem. Reale Ist. Lombardo Sci. (Ser. 3), C. Sci. Mat. 4 (13): 417, 1877 (Trevisan 1877).

*** Cephaloziella subspinosa R.M.Schust., Nova Hedwigia 22: 210, 1971 [1972] (Schuster 1971b).

** **subg. Prionolobus (Spruce) Müll.Frib.**, Lebermoose 2 (16): 110, 1912 (Müller 1912). Bas.: Cephalozia subg. Prionolobus Spruce, Trans. & Proc. Bot. Soc. Edinburgh 15: 508, 1885 (Spruce 1885).

*** Cephaloziella acanthophora (S.Hatt.) Horik., Hikobia 1 (2): 79, 1951 (Horikawa 1951c). Bas.: Prionolobus acanthophorus S.Hatt., Bull. Tokyo Sci. Mus. 11: 29, 1944 (Hattori 1944d).

*** Cephaloziella biokoensis Váňa et Frank Müll., Trop. Bryol. 24: 1, 2003 (Váňa and Müller 2003).

*** Cephaloziella granatensis (J.B.Jack ex Steph.) Fulford, Mem. New York Bot. Gard. 11 (4): 411, 1976 (Fulford 1976). Bas.: Cephalozia granatensis J.B.Jack ex Steph., Bull. Herb. Boissier (sér. 2) 8 (7): 500 (330), 1908 (Stephani 1908f).

*** Cephaloziella grisea R.M.Schust., Phytologia 39 (6): 425, 1978 (Schuster 1978b).

* Cephaloziella meghalayensis Udar et Ad.Kumar, Lindbergia 8 (1): 34, 1982 (Udar and Kumar 1982d). ^44^

*** Cephaloziella microphylla (Steph.) Douin, Mém. Soc. Bot. France 29: 59, 1920 (Douin 1920). Bas.: Cephalozia microphylla Steph., Bull. Herb. Boissier (sér. 2) 8 (7): 513 (343), 1908 (Stephani 1908f).

*** Cephaloziella tenuissima (Lehm.) Steph., Hedwigia 32 (5): 318, 1893 (Stephani 1893d). Bas.: Jungermannia tenuissima Lehm., Linnaea 4: 367, 1829 (Lehmann 1829).

*** Cephaloziella turneri (Hook.) Müll.Frib., Lebermoose 2 (17): 202, 1913 (Müller 1913a). Bas.: Jungermannia turneri Hook., Brit. Jungermann.: tab. 29, 1812 (Hooker 1812).

***Incertae sedis***

** Cephaloziella dentifolia Udar et Ad.Kumar, Lindbergia 8 (1): 30, 1982 (Udar and Kumar 1982d).

** Cephaloziella filum (Trevis.) Steph., Hedwigia 32 (5): 318, 1893 (Stephani 1893d). Bas.: Cephalozia filum Trevis., Mem. Reale Ist. Lombardo Sci. (Ser. 3), C. Sci. Mat. 4 (13): 417, 1877 (Trevisan 1877).

* Cephaloziella flexuosa C.Gao et K.C.Chang, Bull. Bot. Res., Harbin 4 (3): 88, 1984 (Chang and Gao 1984). ^45^

** Cephaloziella intricata Schiffn. ex Douin, Mém. Soc. Bot. France 29: 59, 1920 (Douin 1920).

** Cephaloziella levieri Schiffn. ex Douin, Mém. Soc. Bot. France 29: 80, 1920 (Douin 1920).

*** Cephaloziella pellucida R.M.Schust., Nova Hedwigia 63 (1/2): 57, 1996 (Schuster 1996d). ^46^

* Cephaloziella pygmaea (Spruce) Váňa, Phytotaxa 183 (4): 290, 2014 (Váňa et al. 2014a). Bas.: Cephalozia pygmaea Spruce, Cephalozia: 69, 1882 (Spruce 1882). ^47^

* Cephaloziella secundifolia Pearson, Ann. Bryol. 4: 106, 1931 (Pearson 1931b). ^48^

** Cephaloziella sinensis Douin, Rev. Bryol. 41 (1): 8, 1914 (Douin 1914).

** **Cephaloziopsis (Spruce) Schiffn.**, Hepat. (Engl.-Prantl): 85, 1893 (Schiffner 1893b). Bas.: Jungermannia sect. Cephaloziopsis Spruce, Trans. & Proc. Bot. Soc. Edinburgh 15: 511, 1885 (Spruce 1885).

*** Cephaloziopsis intertexta (Gottsche) R.M.Schust., Nova Hedwigia 22: 183, 1971 [1972] (Schuster 1971b). Bas.: Jungermannia intertexta Gottsche, Syn. Hepat. 1: 107, 1844 (Gottsche et al. 1844).

** **Chaetophyllopsis R.M.Schust.**, J. Hattori Bot. Lab. 23: 69, 1960 [1961] (Schuster 1960b).

*** Chaetophyllopsis whiteleggei (Carrington et Pearson) R.M.Schust. ex Hamlin, Rec. Domin. Mus. 7: 255, 1972 (Hamlin 1972). Bas.: Jungermannia whiteleggei Carrington et Pearson, Proc. Linn. Soc. New South Wales (ser. 2) 2 (4): 1051, 1888 (Carrington and Pearson 1888a).

** **Cylindrocolea R.M.Schust.**, Bull. Natl. Sci. Mus. Tokyo (n.ser.) 12 (3): 666, 1969 (Schuster 1969a).

** **subg. Cylindrocolea**

*** Cylindrocolea kiaeri (Austin) Váňa, Phytotaxa 112 (1): 2, 2013 (Váňa et al. 2013e). Bas.: Jungermannia kiaeri Austin, Bull. Torrey Bot. Club 6 (3): 18, 1875 (Austin 1875b).

*** Cylindrocolea sanctae-helenae M.Wigginton, Polish Bot. J. 58 (1): 107, 2013 (Wigginton 2013).

** **sect. Cylindrocolea**

*** Cylindrocolea andersonii R.M.Schust., Hepat. Anthocerotae N. Amer. 4: 33, 1980 (Schuster 1980c).

*** Cylindrocolea brasiliensis D.P.Costa, N.D.Santos et Váňa, Bryologist 111 (4): 667, 2008 (Costa et al. 2008).

*** Cylindrocolea chevalieri (Steph.) R.M.Schust., Bull. Natl. Sci. Mus. Tokyo (n.ser.) 12 (3): 666, 1969 (Schuster 1969a). Bas.: Alobiella chevalieri Steph., Bull. Herb. Boissier (sér. 2) 8 (8): 567 (351), 1908 (Stephani 1908e).

*** Cylindrocolea gittinsii (E.W.Jones) R.M.Schust., Nova Hedwigia 22: 172, 1971 [1972] (Schuster 1971b). Bas.: Cephaloziella gittinsii E.W.Jones, Trans. Brit. Bryol. Soc. 3 (3): 437, 1958 (Jones 1958).

*** Cylindrocolea madagascariensis (Steph.) R.M.Schust., Bull. Natl. Sci. Mus. Tokyo (n.ser.) 12 (3): 666, 1969 (Schuster 1969a). Bas.: Lophozia madagascariensis Steph., Sp. Hepat. (Stephani) 6: 112, 1917 (Stephani 1917a).

*** Cylindrocolea nigerica (E.W.Jones) R.M.Schust., Nova Hedwigia 22: 172, 1971 [1972] (Schuster 1971b). Bas.: Cephaloziella nigerica E.W.Jones, Trans. Brit. Bryol. Soc. 3 (3): 435, 1958 (Jones 1958).

*** Cylindrocolea novae-caledoniae (Grolle) R.M.Schust., Nova Hedwigia 22: 161, 1971 [1972] (Schuster 1971b). Bas.: Cephaloziella novae-caledoniae Grolle, Rev. Bryol. Lichénol. 29 (3/4): 208, 1960 [1961] (Grolle 1960a).

*** Cylindrocolea planifolia (Steph.) R.M.Schust., Nova Hedwigia 22: 164, 1971 [1972] (Schuster 1971b). Bas.: Cephaloziella planifolia Steph., Hedwigia 32 (5): 317, 1893 (Stephani 1893d).

*** Cylindrocolea sprucei R.M.Schust., Nova Hedwigia 22: 163, 1971 [1972] (Schuster 1971b).

*** Cylindrocolea ugandica (E.W.Jones) R.M.Schust., Nova Hedwigia 22: 171, 1971 [1972] (Schuster 1971b). Bas.: Cephaloziella ugandica E.W.Jones, Trans. Brit. Bryol. Soc. 3 (3): 433, 1958 (Jones 1958).

** **sect. Platycaulis R.M.Schust.**, Nova Hedwigia 22: 173, 1971 (Schuster 1971b).

*** Cylindrocolea recurvifolia (Steph.) Inoue, J. Jap. Bot. 47 (11): 348, 1972 (Inoue 1972a). Bas.: Cephalozia recurvifolia Steph., Bull. Herb. Boissier (sér. 2) 8 (7): 497 (327), 1908 (Stephani 1908f).

*** Cylindrocolea tagawae (N.Kitag.) R.M.Schust., Nova Hedwigia 22: 174, 1971 [1972] (Schuster 1971b). Bas.: Cephaloziella tagawae N.Kitag., J. Hattori Bot. Lab. 32: 303, 1969 (Kitagawa 1969c).

** **subg. Cylindroscyphus (Douin) R.M.Schust.**, Hepat. Anthocerotae N. Amer. 4: 20, 1980 (Schuster 1980c). Bas.: Cephaloziella subg. Cylindroscyphus Douin, Mém. Soc. Bot. France 29: 56, 1920 (Douin 1920).

*** Cylindrocolea abyssinica (Gola) Váňa, Phytotaxa 112 (1): 2, 2013 (Váňa et al. 2013e). Bas.: Cephaloziella abyssinica Gola, Ann. Bot. (Rome) 13 (1): 68, 1914 (Gola 1914a).

** Cylindrocolea obtusifolia Fulford, Mem. New York Bot. Gard. 11 (4): 401, 1976 (Fulford 1976).

** Cylindrocolea reticulata Udar et Ad.Kumar, Lindbergia 8 (3): 181, 1982 [1983] (Udar and Kumar 1982c).

*** Cylindrocolea rhizantha (Mont.) R.M.Schust., Nova Hedwigia 22: 175, 1971 [1972] (Schuster 1971b). Bas.: Jungermannia rhizantha Mont., Hist. Phys. Cuba, Bot., Pl. Cell.: 454, 1842 (Montagne 1842a).

*** **Gottschelia Grolle**, J. Hattori Bot. Lab. 31: 13, 1968 (Grolle 1968c).

*** Gottschelia maxima (Steph.) Grolle, J. Bryol. 25 (1): 6, 2003 (Grolle et al. 2003). Bas.: Tylimanthus maximus Steph., Sp. Hepat. (Stephani) 6: 249, 1922 (Stephani 1922).

*** Gottschelia schizopleura (Spruce) Grolle, J. Hattori Bot. Lab. 31: 16, 1968 (Grolle 1968c). Bas.: Jungermannia schizopleura Spruce, Trans. & Proc. Bot. Soc. Edinburgh 15: 517, 1885 (Spruce 1885).

** **Gymnocoleopsis (R.M.Schust.) R.M.Schust.**, Phytologia 39 (4): 243, 1978 (Schuster 1978a). Bas.: Gymnocolea subg. Gymnocoleopsis R.M.Schust., Bryologist 70 (1): 111, 1967 (Schuster 1967b).

*** Gymnocoleopsis capensis (S.W.Arnell) R.M.Schust., J. Hattori Bot. Lab. 78: 123, 1995 (Schuster 1995c). Bas.: Lophozia capensis S.W.Arnell, Svensk Bot. Tidskr. 47 (1): 112, 1953 (Arnell 1953a).

*** Gymnocoleopsis cylindriformis (Mitt.) R.M.Schust., J. Hattori Bot. Lab. 78: 126, 1995 (Schuster 1995c). Bas.: Jungermannia cylindriformis Mitt., J. Linn. Soc., Bot. 15 (84): 196, 1876 (Mitten 1876b).

*** **Herzogobryum Grolle**, Rev. Bryol. Lichénol. 32 (3/4): 160, 1963 [1964] (Grolle 1963d). *Nom. nov. pro Chondrophyllum* Herzog, Rev. Bryol. Lichénol. 21 (1/2): 46, 1952 (Herzog 1952f).

*** Herzogobryum atrocapillum (Hook.f. et Taylor) Grolle, Österr. Bot. Z. 113 (2): 228, 1966 (Grolle 1966j). Bas.: Gymnomitrion atrocapillum Hook.f. et Taylor, London J. Bot. 5: 258, 1846 (Taylor 1846a).

*** Herzogobryum filiforme R.M.Schust., Phytologia 45 (5): 422, 1980 (Schuster 1980b).

*** Herzogobryum molle Grolle, Österr. Bot. Z. 113 (2): 226, 1966 (Grolle 1966j).

*** Herzogobryum vermiculare (Schiffn.) Grolle, J. Hattori Bot. Lab. 28: 103, 1965 (Grolle 1965e). Bas.: Gymnomitrion vermiculare Schiffn., Leberm., Forschungsr. Gazelle 4 (4): 2, 1890 (Schiffner 1890).

** **Kymatocalyx Herzog**, Memoranda Soc. Fauna Fl. Fennica 25: 56, 1950 (Herzog 1950c).

*** Kymatocalyx africanus Váňa et M.Wigginton, Haussknechtia, Beih. 9: 158, 1999 (Gradstein and Váňa 1999).

*** Kymatocalyx dominicensis (Spruce) Váňa, Österr. Bot. Z. 118 (5): 575, 1970 (Váňa 1970b). Bas.: Jungermannia dominicensis Spruce, J. Linn. Soc., Bot. 30 (210): 363, 1895 (Gepp 1895b).

*** Kymatocalyx madagascariensis (Steph.) Gradst. et Váňa, Haussknechtia, Beih. 9: 164, 1999 (Gradstein and Váňa 1999). Bas.: Acrobolbus madagascariensis Steph., Bull. Herb. Boissier (sér. 2) 2 (5): 460 (179), 1902 (Stephani 1902f).

*** Kymatocalyx rhizomaticus (Herzog) Gradst. et Váňa, Haussknechtia, Beih. 9: 166, 1999 (Gradstein and Váňa 1999). Bas.: Stenorrhipis rhizomatica Herzog, Trans. Brit. Bryol. Soc. 1 (4): 290, 1950 (Herzog 1950a).

*** **Lophonardia R.M.Schust.**, Phytologia 39 (4): 244, 1978 (Schuster 1978a).

*** Lophonardia jamesonii (Mont.) L.Söderstr. et Váňa, Phytotaxa 81 (1): 19, 2013 (Söderström et al. 2013a). Bas.: Jungermannia jamesonii Mont., Syll. Gen. Sp. Crypt.: 60, 1856 (Montagne 1856b).

*** Lophonardia laxifolia (Mont.) L.Söderstr. et Váňa, Phytotaxa 81 (1): 20, 2013 (Söderström et al. 2013a). Bas.: Sarcocyphos laxifolius Mont., Ann. Sci. Nat. Bot. (sér. 3) 4: 346, 1845 (Montagne 1845b).

*** Lophonardia tristaniana (S.W.Arnell) L.Söderstr. et Váňa, Phytotaxa 81 (1): 20, 2013 (Söderström et al. 2013a). Bas.: Jungermannia tristaniana S.W.Arnell, Results Norweg. Sci. Exped. Tristan da Cunha 42: 12, 1958 (Arnell 1958b).

*** **Nothogymnomitrion R.M.Schust.**, J. Hattori Bot. Lab. 80: 43, 1996 (Schuster 1996a).

*** Nothogymnomitrion erosum (Carrington et Pearson) R.M.Schust., J. Hattori Bot. Lab. 80: 44, 1996 (Schuster 1996a). Bas.: Cesius erosus Carrington et Pearson, Pap. & Proc. Roy. Soc. Tasmania 1887: 8, 1888 (Carrington and Pearson 1888b).

*** **Obtusifolium S.W.Arnell**, Ill. Moss Fl. Fennosc. Hep.: 309, 1956 (Arnell 1956b).

*** Obtusifolium obtusum (Lindb.) S.W.Arnell, Ill. Moss Fl. Fennosc. Hep.: 133, 1956 (Arnell 1956b). Bas.: Jungermannia obtusa Lindb., Musci Scand.: 7, 1879 (Lindberg 1879).

*** **Oleolophozia L.Söderstr., De Roo et Hedd.**, Phytotaxa 3: 50, 2010 (Söderström et al. 2010b).

*** Oleolophozia perssonii (H.Buch et S.W.Arnell) L.Söderstr., De Roo et Hedd., Phytotaxa 3: 51, 2010 (Söderström et al. 2010b). Bas.: Lophozia perssonii H.Buch et S.W.Arnell, Bot. Not. 97: 382, 1944 (Buch 1944).

** **Phycolepidozia R.M.Schust.**, Bull. Torrey Bot. Club 93 (6): 438, 1966 [1967] (Schuster 1966f).

** **subg. Metaphycolepidozia Gradst., J.-P.Frahm et U.Schwarz**, Taxon 63 (3): 506, 2014 (Gradstein et al. 2014a).

** Phycolepidozia indica Gradst., J.-P.Frahm et U.Schwarz, Taxon 63 (3): 499, 2014 (Gradstein et al. 2014a).

** **subg. Phycolepidozia**

*** Phycolepidozia exigua R.M.Schust., Bull. Torrey Bot. Club 93 (6): 440, 1966 [1967] (Schuster 1966f).

** **Protolophozia (R.M.Schust.) Schljakov**, Novosti Sist. Nizš. Rast. 16: 204, 1979 (Shliakov 1979). Bas.: Lophozia subg. Protolophozia R.M.Schust., Nova Hedwigia 15: 472, 1968 (Schuster 1968b).

** Protolophozia androgyna R.M.Schust. ex Váňa et L.Söderstr., Phytotaxa 76 (3): 51, 2013 (Váňa et al. 2013l). Based on: Lophozia androgyna R.M.Schust., Beih. Nova Hedwigia 119: 266, 2002 (Schuster 2002b), *nom. inval*.

** Protolophozia autoica (R.M.Schust.) Váňa et L.Söderstr., Phytotaxa 76 (3): 51, 2013 (Váňa et al. 2013l). Bas.: Lophozia autoica R.M.Schust., Nova Hedwigia 15: 479, 1968 (Schuster 1968b).

** Protolophozia crispata (R.M.Schust.) Váňa et L.Söderstr., Phytotaxa 76 (3): 51, 2013 (Váňa et al. 2013l). Bas.: Lophozia crispata R.M.Schust., Nova Hedwigia 15: 474, 1968 (Schuster 1968b).

*** Protolophozia druceae (Grolle et E.A.Hodgs.) Váňa et L.Söderstr., Phytotaxa 76 (3): 51, 2013 (Váňa et al. 2013l). Bas.: Lophozia druceae Grolle et E.A.Hodgs., J. Roy. Soc. New Zealand 2 (1): 112, 1972 (Hodgson 1972).

*** Protolophozia elongata (Steph.) Schljakov, Novosti Sist. Nizš. Rast. 16: 204, 1979 (Shliakov 1979). Bas.: Lophozia elongata Steph., Bull. Herb. Boissier (sér. 2) 2 (1): 41 (141), 1902 (Stephani 1902c).

*** Protolophozia herzogiana (E.A.Hodgs. et Grolle) Váňa et L.Söderstr., Phytotaxa 76 (3): 51, 2013 (Váňa et al. 2013l). Bas.: Lophozia herzogiana E.A.Hodgs. et Grolle, Rev. Bryol. Lichénol. 31 (3/4): 152, 1962 [1963] (Grolle 1962c).

*** Protolophozia lancistipa (Grolle) Váňa et L.Söderstr., Phytotaxa 76 (3): 51, 2013 (Váňa et al. 2013l). Bas.: Andrewsianthus lancistipus Grolle, Marion Prince Edw. Is: 230, 1971 (Grolle 1971d).

*** Protolophozia leucorhiza (Mitt.) Váňa et L.Söderstr., Phytotaxa 76 (3): 51, 2013 (Váňa et al. 2013l). Bas.: Jungermannia leucorhiza Mitt., J. Linn. Soc., Bot. 15 (82): 68, 1876 (Mitten 1876a).

*** Protolophozia longiflora (Herzog) L.Söderstr. et Váňa, Phytotaxa 76 (3): 51, 2013 (Váňa et al. 2013l). Bas.: Orthocaulis longiflorus Herzog, Rev. Bryol. Lichénol. 23 (1/2): 32, 1954 (Herzog 1954).

** Protolophozia monoica (E.A.Hodgs.) Váňa et L.Söderstr., Phytotaxa 76 (3): 52, 2013 (Váňa et al. 2013l). Bas.: Metahygrobiella monoica E.A.Hodgs., Trans. Roy. Soc. New Zealand, Bot. 3 (4): 76, 1965 (Hodgson 1965).

*** Protolophozia multicuspidata (Hook.f. et Taylor) Váňa et L.Söderstr., Phytotaxa 76 (3): 52, 2013 (Váňa et al. 2013l). Bas.: Jungermannia multicuspidata Hook.f. et Taylor, London J. Bot. 3: 375, 1844 (Hooker and Taylor 1844a).

** Protolophozia nivicola (R.M.Schust.) Váňa et L.Söderstr., Phytotaxa 76 (3): 52, 2013 (Váňa et al. 2013l). Bas.: Lophozia nivicola R.M.Schust., Nova Hedwigia 15: 477, 1968 (Schuster 1968b).

*** Protolophozia perssoniana (H.A.Mill.) Váňa et L.Söderstr., Phytotaxa 76 (3): 52, 2013 (Váňa et al. 2013l). Bas.: Lophozia perssoniana H.A.Mill., Ark. Bot. (n.ser.) 5 (2): 508, 1963 (Miller 1963).

** Protolophozia subalpina (R.M.Schust.) Váňa et L.Söderstr., Phytotaxa 76 (3): 52, 2013 (Váňa et al. 2013l). Bas.: Lophozia autoica var. subalpina R.M.Schust., Nova Hedwigia 15: 482, 1968 (Schuster 1968b).

*** Protolophozia tasmanica (R.M.Schust.) Váňa et L.Söderstr., Phytotaxa 76 (3): 52, 2013 (Váňa et al. 2013l). Bas.: Lophozia tasmanica R.M.Schust., Nova Hedwigia 15: 484, 1968 (Schuster 1968b).

*** Protolophozia verruculosa (R.M.Schust.) Váňa et L.Söderstr., Phytotaxa 76 (3): 52, 2013 (Váňa et al. 2013l). Bas.: Lophozia verruculosa R.M.Schust., Phytologia 39 (4): 242, 1978 (Schuster 1978a).

######## *** Lophoziaceae Cavers

by L. Söderström and J. Váňa

Lophoziaceae was broadly defined by de Roo et al. (2007) excluding Anastrophyllaceae and Jamesonielloideae and also moving several traditionally included genera to other families. Vilnet et al. (2010) further refined the family. Nomenclatural and taxonomic notes can also be found in Vilnet et al. (2007b, 2008), Söderström et al. (2013c) and Váňa et al. (2013m). The placement of Gerhildiella and Pseudocephaloziella in the family is provisional.

*** **Andrewsianthus R.M.Schust.**, Rev. Bryol. Lichénol. 30 (1/2): 66, 1961 (Schuster 1961a).

** **subg. Andrewsianthus**

*** Andrewsianthus aberrans (Nees et Mont.) Grolle, Trans. Brit. Bryol. Soc. 4 (3): 440, 1963 (Grolle 1963a). Bas.: Jungermannia aberrans Nees et Mont., Ann. Sci. Nat. Bot. (sér. 2) 19: 250, 1843 (Montagne 1843).

*** Andrewsianthus bidens (Mitt. ex Steph.) R.M.Schust., Nova Hedwigia 15: 492, 1968 (Schuster 1968b). Bas.: Lophozia bidens Mitt. ex Steph., Bull. Herb. Boissier (sér. 2) 2 (1): 41 (141), 1902 (Stephani 1902c).

*** Andrewsianthus bilobus (Mitt.) Grolle, Trans. Brit. Bryol. Soc. 4 (3): 437, 1963 (Grolle 1963a). Bas.: Gymnanthe biloba Mitt., J. Proc. Linn. Soc., Bot. 7 (27): 166, 1863 (Mitten 1863).

*** Andrewsianthus carinatus Grolle, Marion Prince Edw. Is: 229, 1971 (Grolle 1971d).

*** Andrewsianthus cavifolius Grolle et Váňa, J. Hattori Bot. Lab. 38: 640, 1974 (Váňa 1974b).

*** Andrewsianthus chimbuensis R.M.Schust., Nova Hedwigia 15: 491, 1968 (Schuster 1968b).

*** Andrewsianthus kinabaluensis N.Kitag., J. Hattori Bot. Lab. 33: 205, 1970 (Kitagawa 1970).

*** Andrewsianthus koponenii Váňa et Piippo, Ann. Bot. Fenn. 26 (3): 284, 1989 (Váňa and Piippo 1989).

*** Andrewsianthus mizutanii N.Kitag., J. Hattori Bot. Lab. 32: 307, 1969 (Kitagawa 1969a).

*** Andrewsianthus papillosus N.Kitag., J. Hattori Bot. Lab. 33: 207, 1970 (Kitagawa 1970).

*** Andrewsianthus perigonialis (Hook.f. et Taylor) R.M.Schust., Beih. Nova Hedwigia 119: 336, 2002 (Schuster 2002b). Bas.: Jungermannia perigonialis Hook.f. et Taylor, London J. Bot. 3: 368, 1844 (Hooker and Taylor 1844a).

*** Andrewsianthus puniceus (Nees) R.M.Schust. ex Grolle, Trans. Brit. Bryol. Soc. 4 (3): 439, 1963 (Grolle 1963a). Bas.: Jungermannia punicea Nees, Enum. Pl. Crypt. Javae: 32, 1830 (Nees 1830).

*** Andrewsianthus recurvifolius (Nees) R.M.Schust., Hepat. Anthocerotae N. Amer. 2: 710, 1969 (Schuster 1969b). Bas.: Jungermannia recurvifolia Nees, Enum. Pl. Crypt. Javae: 32, 1830 (Nees 1830).

*** Andrewsianthus sundaicus (Schiffn.) R.M.Schust., Hepat. Anthocerotae N. Amer. 2: 710, 1969 (Schuster 1969b). Bas.: Anastrophyllum sundaicum Schiffn., Denkschr. Kaiserl. Akad. Wiss., Math.-Naturwiss. Kl. 67: 202, 1898 (Schiffner 1898a).

*** Andrewsianthus zantenii Váňa, J. Hattori Bot. Lab. 38: 645, 1974 (Váňa 1974b).

** **subg. Cephalolobus (R.M.Schust.) R.M.Schust.**, Beih. Nova Hedwigia 119: 326, 2002 (Schuster 2002b). Bas.: Cephalolobus R.M.Schust., Rev. Bryol. Lichénol. 34 (1/2): 244, 1966 (Schuster 1966b).

*** Andrewsianthus hodgsoniae (R.M.Schust.) R.M.Schust. ex J.J.Engel, Novon 17 (3): 311, 2007 (Engel 2007). Bas.: Cephalolobus hodgsoniae R.M.Schust., Rev. Bryol. Lichénol. 34 (1/2): 254, 1966 (Schuster 1966b).

*** Andrewsianthus marionensis (S.W.Arnell) Grolle, Marion Prince Edw. Is: 232, 1971 (Grolle 1971d). Bas.: Lophozia marionensis S.W.Arnell, Svensk Bot. Tidskr. 47 (3): 421, 1953 (Arnell 1953c).

*** Andrewsianthus scabrellus (C.Massal.) R.M.Schust. ex J.J.Engel, Novon 17 (3): 311, 2007 (Engel 2007). Bas.: Cephalozia scabrella C.Massal., Nuovo Giorn. Bot. Ital. 17 (3): 233, 1885 (Massalongo 1885).

** Andrewsianthus sphenoloboides (R.M.Schust.) R.M.Schust. ex J.J.Engel, Novon 17 (3): 311, 2007 (Engel 2007). Bas.: Cephalolobus sphenoloboides R.M.Schust., Rev. Bryol. Lichénol. 34 (1/2): 251, 1966 (Schuster 1966b).

** **Gerhildiella Grolle**, Rev. Bryol. Lichénol. 34 (1/2): 187, 1966 (Grolle 1966a).

*** Gerhildiella rossneriana Grolle, Rev. Bryol. Lichénol. 34 (1/2): 187, 1966 (Grolle 1966a).

*** **Heterogemma (Jørg.) Konstant. et Vilnet**, Arctoa 18: 67, 2009 [2010] (Konstantinova and Vilnet 2009). Bas.: Lophozia sect. Heterogemma Jørg., Bergens Mus. Skr. (n.ser.) 16: 146, 1934 (Jørgensen 1934).

*** Heterogemma capitata (Hook.) Konstant. et Vilnet, Arctoa 18: 67, 2009 [2010] (Konstantinova and Vilnet 2009). Bas.: Jungermannia capitata Hook., Brit. Jungermann.: tab. 80, 1816 (Hooker 1816a).

*** Heterogemma laxa (Lindb.) Konstant. et Vilnet, Arctoa 18: 67, 2009 [2010] (Konstantinova and Vilnet 2009). Bas.: Jungermannia laxa Lindb., Acta Soc. Sci. Fenn. 10: 529, 1875 (Lindberg 1875).

*** Heterogemma patagonica (Herzog et Grolle) L.Söderstr. et Váňa, Phytotaxa 97 (2): 29, 2013 (Söderström et al. 2013c). Bas.: Lophozia patagonica Herzog et Grolle, Rev. Bryol. Lichénol. 28 (3/4): 343, 1959 [1960] (Grolle 1959a).

*** **Lophozia (Dumort.) Dumort.**, Recueil Observ. Jungerm.: 17, 1835 (Dumortier 1835). Bas.: Jungermannia sect. Lophozia Dumort., Syll. Jungerm. Europ.: 53, 1831 (Dumortier 1831). ^49^

*** Lophozia ascendens (Warnst.) R.M.Schust., Bryologist 55 (3): 180, 1952 (Schuster 1952). Bas.: Sphenolobus ascendens Warnst., Hedwigia 57 (1/2): 63, 1916 (Warnstorf 1916).

* Lophozia austrosibirica Bakalin, Ann. Bot. Fenn. 40 (1): 49, 2003 (Bakalin 2003).

** Lophozia ciliata Damsh., L.Söderstr. et H.Weibull, Lindbergia 25 (1): 3, 2000 (Söderström et al. 2000).

*** Lophozia guttulata (Lindb. et Arnell) A.Evans, Proc. Wash. Acad. Sci. 2: 302, 1900 (Evans 1900c). Bas.: Jungermannia guttulata Lindb. et Arnell, Kongl. Svenska Vetensk.-Akad. Handl. (n.ser.) 23 (5): 51, 1889 (Lindberg and Arnell 1889).

* Lophozia jamaicensis (Nees) Steph., Bull. Herb. Boissier (sér. 2) 1 (2): 128 (155), 1901 (Stephani 1901h). Bas.: Jungermannia jamaicensis Nees, Syn. Hepat. 1: 105, 1844 (Gottsche et al. 1844). ^50^

** Lophozia lacerata N.Kitag., Hikobia 3 (3): 172, 1963 (Kitagawa 1963a).

** Lophozia lantratoviae Bakalin, Ann. Bot. Fenn. 40 (1): 47, 2003 (Bakalin 2003).

** Lophozia murmanica Kaal., Rep. Second Norweg. Arctic Exped. 11: 34, 1906 (Bryhn 1906).

** Lophozia pacifica Bakalin, Bryologist 114 (2): 302, 2011 (Bakalin 2011).

* Lophozia pallida (Steph.) Grolle, J. Jap. Bot. 39 (6): 174, 1964 (Grolle 1964a). Bas.: Anastrophyllum pallidum Steph., Bull. Herb. Boissier (sér. 2) 1 (10): 1131 (114), 1901 (Stephani 1901c). ^51^

** Lophozia savicziae Schljakov, Novosti Sist. Nizš. Rast. 10: 299, 1973 (Shliakov 1973).

* Lophozia schusterana Schljakov, Novosti Sist. Nizš. Rast. 12: 320, 1975 (Shliakov 1975).

*** Lophozia silvicola H.Buch, Beret. 18 Skand. Naturforskarmøde: 228, 1929 (Buch 1929).

*** Lophozia silvicoloides N.Kitag., J. Hattori Bot. Lab. 28: 276, 1965 (Kitagawa 1965).

* Lophozia subapiculata R.M.Schust. et Damsh., Meddel. Grønland 199 (1): 104, 1974 (Schuster and Damsholt 1974). ^52^

** Lophozia udarii S.Srivast., S.C.Srivast. et K.K.Rawat, Nelumbo 55: 130, 2013 (Srivastava et al. 2013). ^53^

*** Lophozia ventricosa (Dicks.) Dumort., Recueil Observ. Jungerm.: 17, 1835 (Dumortier 1835). Bas.: Jungermannia ventricosa Dicks., Fasc. Pl. Crypt. Brit. 2: 14, 1790 (Dickson 1790). ^54^

* Lophozia wenzelii (Nees) Steph., Bull. Herb. Boissier (sér. 2) 2 (1): 35 (135), 1902 (Stephani 1902c). Bas.: Jungermannia wenzelii Nees, Naturgesch. Eur. Leberm. 2: 58, 1836 (Nees 1836). ^55^

**Excluded from the genus**

* Lophozia serpens (Dumort.) Dumort., Recueil Observ. Jungerm.: 17, 1835 (Dumortier 1835). Bas.: Jungermannia serpens Dumort., Syll. Jungerm. Europ.: 56, 1831 (Dumortier 1831). ^56^

** **Lophoziopsis Konstant. et Vilnet**, Arctoa 18: 66, 2009 [2010] (Konstantinova and Vilnet 2009).

*** Lophoziopsis excisa (Dicks.) Konstant. et Vilnet, Arctoa 18: 66, 2009 [2010] (Konstantinova and Vilnet 2009). Bas.: Jungermannia excisa Dicks., Fasc. Pl. Crypt. Brit. 3: 11, 1793 (Dickson 1793).

** Lophoziopsis excisa var. elegans (R.M.Schust.) Konstant. et Vilnet, Arctoa 18: 66, 2009 [2010] (Konstantinova and Vilnet 2009). Bas.: Lophozia excisa var. elegans R.M.Schust., Hepat. Anthocerotae N. Amer. 2: 522, 1969 (Schuster 1969b).

** Lophoziopsis excisa var. infuscata (R.M.Schust. et Damsh.) Konstant. et Vilnet, Arctoa 18: 66, 2009 [2010] (Konstantinova and Vilnet 2009). Bas.: Lophozia excisa var. infuscata R.M.Schust. et Damsh., Meddel. Grønland 199 (1): 94, 1974 (Schuster and Damsholt 1974).

** Lophoziopsis excisa var. succulenta (R.M.Schust. et Damsh.) Konstant. et Vilnet, Arctoa 18: 66, 2009 [2010] (Konstantinova and Vilnet 2009). Bas.: Lophozia excisa var. succulenta R.M.Schust. et Damsh., Meddel. Grønland 199 (1): 96, 1974 (Schuster and Damsholt 1974).

*** Lophoziopsis longidens (Lindb.) Konstant. et Vilnet, Arctoa 18: 66, 2009 [2010] (Konstantinova and Vilnet 2009). Bas.: Jungermannia longidens Lindb., Helsingf. Dagbl. 1876 (323, 26 Nov.): 2, 1876 (Lindberg 1876a).

** Lophoziopsis longidens subsp. arctica (R.M.Schust.) Váňa et L.Söderstr., Phytotaxa 97 (2): 28, 2013 (Söderström et al. 2013c). Bas.: Lophozia longidens subsp. arctica R.M.Schust., Hepat. Anthocerotae N. Amer. 2: 539, 1969 (Schuster 1969b). ^57^

*** Lophoziopsis pellucida (R.M.Schust.) Konstant. et Vilnet, Arctoa 18: 67, 2009 [2010] (Konstantinova and Vilnet 2009). Bas.: Lophozia pellucida R.M.Schust., Canad. J. Bot. 39 (4): 978, 1961 (Schuster 1961b).

** Lophoziopsis pellucida var. minor (R.M.Schust.) L.Söderstr. et Váňa, Phytotaxa 97 (2): 28, 2013 (Söderström et al. 2013c). Bas.: Lophozia pellucida var. minor R.M.Schust., Canad. J. Bot. 39 (4): 984, 1961 (Schuster 1961b).

*** Lophoziopsis polaris (R.M.Schust.) Konstant. et Vilnet, Arctoa 18: 67, 2009 [2010] (Konstantinova and Vilnet 2009). Bas.: Lophozia alpestris subsp. polaris R.M.Schust., Hepat. Anthocerotae N. Amer. 2: 614, 1969 (Schuster 1969b).

** Lophoziopsis polaris var. sphagnorum (R.M.Schust.) Konstant. et Vilnet, Arctoa 18: 67, 2009 [2010] (Konstantinova and Vilnet 2009). Bas.: Lophozia alpestris f. sphagnorum R.M.Schust., Hepat. Anthocerotae N. Amer. 2: 619, 1969 (Schuster 1969b).

* Lophoziopsis propagulifera (Gottsche) Konstant. et Vilnet, Arctoa 18: 67, 2009 [2010] (Konstantinova and Vilnet 2009). Bas.: Jungermannia propagulifera Gottsche, Int. Polarforsch., Deutsch. Exped. 2: 451, 1890 (Gottsche 1890). ^58^

** Lophoziopsis rubrigemma (R.M.Schust.) Konstant. et Vilnet, Arctoa 18: 67, 2009 [2010] (Konstantinova and Vilnet 2009). Bas.: Lophozia rubrigemma R.M.Schust., Hepat. Anthocerotae N. Amer. 2: 621, 1969 (Schuster 1969b).

** **Pseudocephaloziella R.M.Schust.**, Phytologia 39 (4): 243, 1978 (Schuster 1978a).

*** Pseudocephaloziella epiphytica R.M.Schust., Phytologia 39 (4): 243, 1978 (Schuster 1978a).

*** **Trilophozia (R.M.Schust.) Bakalin**, Monogr. Lophozia: 34, 2005 (Bakalin 2005). Bas.: Tritomaria subg. Trilophozia R.M.Schust., Amer. Midl. Naturalist 49 (2): 382, 1953 (Schuster 1953).

*** Trilophozia quinquedentata (Huds.) Bakalin, Monogr. Lophozia: 34, 2005 (Bakalin 2005). Bas.: Jungermannia quinquedentata Huds., Fl. Angl. (Hudson): 433, 1762 (Hudson 1762).

** Trilophozia quinquedentata var. asymmetrica (Horik.) L.Söderstr. et Váňa, Phytotaxa 97 (2): 32, 2013 (Söderström et al. 2013c). Bas.: Lophozia asymmetrica Horik., J. Sci. Hiroshima Univ., Ser. B, Div. 2, Bot. 2: 153, 1934 (Horikawa 1934).

*** **Tritomaria Schiffn. ex Loeske**, Hedwigia 49 (1/2): 13, 1909 (Loeske 1909). Based on: Tritomaria Schiffn., Ber. Naturwiss.-Med. Vereins Innsbruck 31 [Beilage]: 12, 1908 (Schiffner 1908a).

*** Tritomaria exsecta (Schmidel) Schiffn. ex Loeske, Hedwigia 49 (1/2): 13, 1909 (Loeske 1909). Bas.: Jungermannia exsecta Schmidel, Syst. Samml. Crypt. Gew. 2: 5, 1797 (Schrader 1797), *nom. conserv*.

** Tritomaria exsecta subsp. novaezelandiae J.J.Engel, Bryologist 109 (1): 61, 2006 (Engel 2006b).

*** Tritomaria exsectiformis (Breidl.) Schiffn. ex Loeske, Hedwigia 49 (1/2): 13, 1909 (Loeske 1909). Bas.: Jungermannia exsectiformis Breidl., Mitt. Naturwiss. Vereins Steiermark 30: 321, 1894 (Breidler 1894).

** Tritomaria exsectiformis subsp. arctica R.M.Schust., Hepat. Anthocerotae N. Amer. 2: 661, 1969 (Schuster 1969b).

** Tritomaria exsectiformis subsp. camerunensis S.W.Arnell ex Váňa, Phytotaxa 167 (2): 216, 2014 (Váňa et al. 2014b). Based on: Tritomaria camerunensis S.W.Arnell, Svensk Bot. Tidskr. 52 (1): 64, 1958 (Arnell 1958a), *nom. inval*.

*** Tritomaria ferruginea (Grolle) Váňa, Phytotaxa 81 (1): 24, 2013 (Váňa et al. 2013m). Bas.: Andrewsianthus ferrugineus Grolle, Khumbu Himal 1 (4): 275, 1966 (Grolle 1966k).

* Tritomaria koreana Bakalin, S.S.Choi et B.Y.Sun, Arctoa 18: 163, 2009 [2010] (Bakalin et al. 2009a).

* Tritomaria mexicana Bakalin, Arctoa 17: 162, 2008 [2009] (Bakalin 2008b).

*** Tritomaria scitula (Taylor) Jørg., Bergens Mus. Aarbok 1919/20 (7): 3, 1922 (Jørgensen 1922). Bas.: Jungermannia scitula Taylor, London J. Bot. 5: 274, 1846 (Taylor 1846a).

######## *** Scapaniaceae Mig.

by J. Váňa

Scapaniaceae , in its classical concept, was recognized as monophyletic and nested within Lophoziaceae s. lat. (cf. Davis 2004, Schill et al. 2004, Yatsentyuk et al. 2004, He-Nygrén et al. 2006). More recent molecular studies classified Scapaniaceae as a sister clade to Lophoziaceae, and the genus Schistochilopsis, a segregate of the genus Lophozia s. lat., more closely related to Scapaniaceae than to Lophoziaceae (cf. de Roo et al. 2007, Vilnet et al. 2007). The genus Scapania was recently studied by morphological methods by Potemkin (2002), by molecular methods by Vilnet et al. (2007) and especially Heinrichs et al. (2012a). Some taxonomic and nomenclatural notes can be found in Váňa et al. (2012a, 2013j, 2015) and Konstantinova et al. (2013a). The placement of Pseudotritomaria, Saccobasis and Schistochilopsis in the family follows Vilnet et al. (2010).

*** **Diplophyllum (Dumort.) Dumort.**, Recueil Observ. Jungerm.: 15, 1835 (Dumortier 1835) nom. conserv. Bas.: Jungermannia sect. Diplophyllum Dumort., Syll. Jungerm. Europ.: 44, 1831 (Dumortier 1831).

*** **subg. Austrodiplophyllum R.M.Schust.**, Bull. Natl. Sci. Mus. Tokyo (n.ser.) 11 (1): 18, 1968 (Schuster 1968a).

** Diplophyllum recurvifolium C.Massal., Atti Reale Ist. Veneto Sci. Lett. Arti 87 (2): 221, 1928 (Massalongo 1928).

*** Diplophyllum squarrosum Steph., Sp. Hepat. (Stephani) 4: 116, 1910 (Stephani 1910b).

*** Diplophyllum verrucosum R.M.Schust., Bull. Natl. Sci. Mus. Tokyo (n.ser.) 11 (1): 19, 1968 (Schuster 1968a).

*** **subg. Diplophyllum**

*** **sect. Diplophyllum**

*** Diplophyllum albicans (L.) Dumort., Recueil Observ. Jungerm.: 16, 1835 (Dumortier 1835). Bas.: Jungermannia albicans L., Sp. Pl. 1: 1133, 1753 (Linnaeus 1753).

*** **sect. Protodiplophyllum (R.M.Schust.) Váňa et L.Söderstr.**, Phytotaxa 76 (3): 29, 2013 (Váňa et al. 2013j). Bas.: Diplophyllum subg. Protodiplophyllum R.M.Schust., Hepat. Anthocerotae N. Amer. 3: 192, 1974 (Schuster 1974).

*** Diplophyllum africanum S.W.Arnell, Ark. Bot. (n.ser.) 3 (16): 527, 1956 (Arnell 1956e).

*** Diplophyllum andicola R.M.Schust., Phytologia 39 (4): 248, 1978 (Schuster 1978a).

*** Diplophyllum andrewsii A.Evans, Bryologist 25 (2): 28, 1922 (Evans 1922).

* Diplophyllum androgynum J.J.Engel et G.L.Merr., J. Hattori Bot. Lab. 84: 277, 1998 (Engel and Smith Merrill 1998).

* Diplophyllum angustifolium J.J.Engel et G.L.Merr., J. Hattori Bot. Lab. 84: 262, 1998 (Engel and Smith Merrill 1998).

*** Diplophyllum apiculatum (A.Evans) Steph., Sp. Hepat. (Stephani) 4: 110, 1910 (Stephani 1910b). Bas.: Diplophylleia apiculata A.Evans, Bot. Gaz. 34 (5): 372, 1902 (Evans 1902b).

** Diplophyllum dioicum R.M.Schust., Bull. Natl. Sci. Mus. Tokyo (n.ser.) 11 (1): 20, 1968 (Schuster 1968a).

** Diplophyllum exiguum Steph., Sp. Hepat. (Stephani) 6: 500, 1924 (Stephani 1924).

* Diplophyllum gemmiparum J.J.Engel et G.L.Merr., J. Hattori Bot. Lab. 84: 255, 1998 (Engel and Smith Merrill 1998).

* Diplophyllum incrassatum J.J.Engel et G.L.Merr., J. Hattori Bot. Lab. 84: 265, 1998 (Engel and Smith Merrill 1998).

*** Diplophyllum nanum Herzog, Memoranda Soc. Fauna Fl. Fennica 26: 48, 1950 [1951] (Herzog 1950b).

* Diplophyllum novum J.J.Engel et G.L.Merr., J. Hattori Bot. Lab. 84: 274, 1998 (Engel and Smith Merrill 1998).

*** Diplophyllum obtusatum (R.M.Schust.) R.M.Schust., Hepat. Anthocerotae N. Amer. 3: 215, 1974 (Schuster 1974). Bas.: Diplophyllum apiculatum var. obtusatum R.M.Schust., Amer. Midl. Naturalist 49 (2): 432, 1953 (Schuster 1953).

*** Diplophyllum obtusifolium (Hook.) Dumort., Recueil Observ. Jungerm.: 16, 1835 (Dumortier 1835). Bas.: Jungermannia obtusifolia Hook., Brit. Jungermann.: tab. 26, 1812 (Hooker 1812). ^59^

** Diplophyllum obtusifolium subsp. domesticum (Gottsche) Váňa, Phytotaxa 76 (3): 29, 2013 (Váňa et al. 2013j). Bas.: Jungermannia domestica Gottsche, Linnaea 28 (5): 548, 1856 [1857] (Gottsche 1856).

*** Diplophyllum serrulatum (Müll.Frib.) Steph., Sp. Hepat. (Stephani) 4: 112, 1910 (Stephani 1910b). Bas.: Diplophylleia serrulata Müll.Frib., Bull. Herb. Boissier (sér. 2) 3 (1): 34, 1903 (Müller 1903).

*** Diplophyllum taxifolium (Wahlenb.) Dumort., Recueil Observ. Jungerm.: 16, 1835 (Dumortier 1835). Bas.: Jungermannia taxifolia Wahlenb., Fl. Lapp. (Wahlenberg): 389, 1812 (Wahlenberg 1812).

** Diplophyllum taxifolium var. mucronatum R.M.Schust., Hepat. Anthocerotae N. Amer. 3: 203, 1974 (Schuster 1974).

*** Diplophyllum trollii Grolle, Khumbu Himal 1 (4): 273, 1966 (Grolle 1966k).

*** **Douinia (C.E.O.Jensen) H.Buch**, Scapan. N.-Eur. Sib.: 13, 1928 (Buch 1928). Bas.: Diplophylleia subg. Douinia C.E.O.Jensen, Danmarks mosser: 145, 1915 (Jensen 1915).

*** Douinia imbricata (M.Howe) Konstant. et Vilnet, Phytotaxa 76 (3): 31, 2013 (Konstantinova et al. 2013a). Bas.: Scapania imbricata M.Howe, Bull. New York Bot. Gard. 2 (6): 104, 1901 (Howe 1901b).

*** Douinia ovata (Dicks.) H.Buch, Scapan. N.-Eur. Sib.: 14, 1928 (Buch 1928). Bas.: Jungermannia ovata Dicks., Fasc. Pl. Crypt. Brit. 3: 11, 1793 (Dickson 1793).

*** Douinia plicata (Lindb.) Konstant. et Vilnet, Phytotaxa 76 (3): 31, 2013 (Konstantinova et al. 2013a). Bas.: Diplophyllum plicatum Lindb., Acta Soc. Sci. Fenn. 10: 235, 1872 [1873] (Lindberg 1872b).

** **Pseudotritomaria Konstant. et Vilnet**, Arctoa 18: 66, 2009 [2010] (Konstantinova and Vilnet 2009).

*** Pseudotritomaria heterophylla (R.M.Schust.) Konstant. et Vilnet, Arctoa 18: 66, 2009 [2010] (Konstantinova and Vilnet 2009). Bas.: Tritomaria heterophylla R.M.Schust., Canad. J. Bot. 36 (2): 272, 1958 (Schuster 1958b).

** **Saccobasis H.Buch**, Memoranda Soc. Fauna Fl. Fennica 8: 291, 1932 [1933] (Buch 1932).

*** Saccobasis polita (Nees) H.Buch, Memoranda Soc. Fauna Fl. Fennica 8: 292, 1932 [1933] (Buch 1932). Bas.: Jungermannia polita Nees, Naturgesch. Eur. Leberm. 2: 145, 1836 (Nees 1836).

** Saccobasis polymorpha (R.M.Schust.) Schljakov, Novosti Sist. Nizš. Rast. 16: 205, 1979 (Shliakov 1979). Bas.: Tritomaria polita subsp. polymorpha R.M.Schust., Hepat. Anthocerotae N. Amer. 2: 700, 1969 (Schuster 1969b).

*** **Scapania (Dumort.) Dumort.**, Recueil Observ. Jungerm.: 14, 1835 (Dumortier 1835) nom. conserv. Bas.: Radula sect. Scapania Dumort., Syll. Jungerm. Europ.: 38, 1831 (Dumortier 1831).

** **subg. Ascapania Grolle**, Khumbu Himal 1 (4): 268, 1966 (Grolle 1966k).

*** Scapania contorta Mitt., J. Proc. Linn. Soc., Bot. 5 (18): 101, 1860 [1861] (Mitten 1860c).

** **subg. Gracilidae (H.Buch) Váňa, Hentschel, Joch.Müll. et Heinrichs**, PhytoKeys 10: 15, 2012 (Váňa et al. 2012a). Bas.: Scapania sect. Gracilidae H.Buch, Scapan. N.-Eur. Sib.: 106, 1928 (Buch 1928).

*** Scapania ampliata Steph., Bull. Herb. Boissier 5 (2): 106, 1897 (Stephani 1897b).

** Scapania ampliata subsp. queenslandica M.L.Hicks, J. Hattori Bot. Lab. 69: 130, 1991 (Hicks 1991).

*** Scapania bolanderi Austin, Proc. Acad. Nat. Sci. Philadelphia 21: 218, 1869 (Austin 1869).

*** Scapania gracilis Lindb., Morgonbladet (Helsinki) 1873 (286, 9 Dec): 2, 1873 (Lindberg 1873a).

** Scapania macroparaphyllia T.Cao, C.Gao et J.Sun, Acta Phytotax. Sin. 42 (2): 180, 2004 (Cao et al. 2004).

*** Scapania maxima Horik., J. Sci. Hiroshima Univ., Ser. B, Div. 2, Bot. 2: 223, 1934 (Horikawa 1934).

*** Scapania nipponica (Amakawa et S.Hatt.) Amakawa, J. Hattori Bot. Lab. 30: 319, 1967 (Amakawa 1967a). Bas.: Scapania bolanderi var. nipponica Amakawa et S.Hatt., J. Hattori Bot. Lab. 14: 83, 1955 (Amakawa and Hattori 1955).

** Scapania paraphyllia T.Cao, C.Gao, J.Sun et B.R.Zuo, Acta Phytotax. Sin. 45 (3): 311, 2007 (Zuo et al. 2007b).

*** Scapania subnimbosa Steph., Sp. Hepat. (Stephani) 4: 150, 1910 (Stephani 1910b).

** **subg. Macroscapania R.M.Schust.**, Hepat. Anthocerotae N. Amer. 3: 248, 1974 (Schuster 1974).

* Scapania geppii Steph., Hedwigia 44 (1): 14, 1904 (Stephani 1904i).

*** Scapania portoricensis Hampe et Gottsche, Linnaea 25 (3): 342, 1852 [1853] (Hampe and Gottsche 1852).

** Scapania portoricensis var. boliviensis (Steph.) Herzog, Ann. Bryol. 1: 110, 1928 (Herzog 1928). Bas.: Scapania boliviensis Steph., Biblioth. Bot. 87 (2): 231, 1916 (Stephani 1916a).

** Scapania portoricensis var. organensis (Herzog) Herzog, Ann. Bryol. 1: 110, 1928 (Herzog 1928). Bas.: Scapania organensis Herzog, Repert. Spec. Nov. Regni Veg. 21 (1/7): 27, 1925 (Herzog 1925a).

** Scapania portoricensis var. roraimensis Warnst., Hedwigia 63 (2): 109, 1921 (Warnstorf 1921).

** **subg. Plicaticalyx Müll.Frib.**, Bull. Herb. Boissier (sér. 2) 3 (1): 36, 1903 (Müller 1903).

** **sect. Planifoliae (Müll.Frib.) Potemkin**, J. Hattori Bot. Lab. 85: 56, 1998 (Potemkin 1998). Bas.: Scapania Gruppe Planifoliae Müll.Frib., Nova Acta Acad. Caes. Leop.-Carol. German. Nat. Cur. 83: 286, 1905 (Müller 1905).

*** Scapania davidii Potemkin, Ann. Bot. Fenn. 38 (2): 83, 2001 (Potemkin 2001).

** Scapania ferruginaeoides T.Cao, C.Gao et J.Sun, Guihaia 24 (1): 23, 2004 (Sun et al. 2004).

** Scapania gaochii X.Fu ex T.Cao, Phytotaxa 97 (2): 26, 2013 (Cao et al. 2013). Based on: Scapania gaochii X.Fu ex T.Cao, Acta Bot. Yunnan. 25 (5): 541, 2003 (Cao et al. 2003), *nom. inval*.

*** Scapania harae Amakawa, J. Hattori Bot. Lab. 27: 5, 1964 (Amakawa 1964a).

*** Scapania nimbosa Taylor, Nov. Stirp. Pug. 8: 6, 1844 (Lehmann 1844).

*** Scapania ornithopodioides (With.) Waddell, Moss Exch. Club Cat. Hepat.: 4, 1897 (Waddell 1897). Bas.: Jungermannia ornithopodioides With., Bot. arr. veg. Gr. Brit. 2: 695, 1776 (Withering 1776).

*** Scapania rotundifolia W.E.Nicholson, Symb. Sin. 5: 31, 1930 (Nicholson et al. 1930).

*** Scapania secunda Steph., Mém. Soc. Nat. Sci. Nat. Math. Cherbourg 29: 226, 1894 (Stephani 1894b).

*** Scapania zhukovae Potemkin, Arctoa 9: 121, 2000 (Potemkin 2000b).

** **sect. Plicaticalyx (Müll.Frib.) Potemkin**, Ann. Bot. Fenn. 39 (4): 326, 2002 (Potemkin 2002). Bas.: Scapania subg. Plicaticalyx Müll.Frib., Bull. Herb. Boissier (sér. 2) 3 (1): 36, 1903 (Müller 1903).

*** Scapania ciliatospinosa Horik., J. Sci. Hiroshima Univ., Ser. B, Div. 2, Bot. 2: 222, 1934 (Horikawa 1934).

*** Scapania ferruginea (Lehm. et Lindenb.) Lehm. et Lindenb., Syn. Hepat. 1: 72, 1844 (Gottsche et al. 1844). Bas.: Jungermannia ferruginea Lehm. et Lindenb., Nov. Stirp. Pug. 4: 20, 1832 (Lehmann 1832).

*** Scapania hians Steph. ex Müll.Frib., Nova Acta Acad. Caes. Leop.-Carol. German. Nat. Cur. 83: 223, 1905 (Müller 1905).

** Scapania hians subsp. salishensis J.D.Godfrey et G.Godfrey, Bryologist 81 (3): 362, 1978 (Godfrey and Godfrey 1978).

*** Scapania orientalis Steph. ex Müll.Frib., Bull. Herb. Boissier (sér. 2) 1 (6): 606, 1901 (Müller 1901a).

*** Scapania pseudocontorta Potemkin, Arctoa 9: 115, 2000 (Potemkin 2000b).

*** Scapania sinikkae Potemkin, Ann. Bot. Fenn. 38 (2): 85, 2001 (Potemkin 2001).

*** Scapania spiniloba Potemkin, Arctoa 9: 117, 2000 (Potemkin 2000b).

** **subg. Pseudomacrodiplophyllum Váňa, Hentschel, Joch.Müll. et Heinrichs**, PhytoKeys 10: 15, 2012 (Váňa et al. 2012a).

*** Scapania microdonta (Mitt.) Müll.Frib., Nova Acta Acad. Caes. Leop.-Carol. German. Nat. Cur. 83: 262, 1905 (Müller 1905). Bas.: Martinellius microdontus Mitt., Trans. Linn. Soc. London, Bot. 3 (3): 196, 1891 (Mitten 1891).

*** **subg. Scapania**

** **sect. Aequilobae (Müll.Frib.) H.Buch**, Scapan. N.-Eur. Sib.: 110, 1928 (Buch 1928). Bas.: Scapania Gruppe Aequilobae Müll.Frib., Nova Acta Acad. Caes. Leop.-Carol. German. Nat. Cur. 83: 219, 1905 (Müller 1905).

*** Scapania aequiloba (Schwägr.) Dumort., Recueil Observ. Jungerm.: 14, 1835 (Dumortier 1835). Bas.: Jungermannia aequiloba Schwägr., Hist. Musc. Hepat. Prodr.: 24, 1814 (Schwägrichen 1814).

*** Scapania aspera M.Bernet et Bernet, Cat. hép. Suisse: 42, 1888 (Bernet 1888).

** **sect. Americanae Váňa, Hentschel, Joch.Müll. et Heinrichs**, PhytoKeys 10: 15, 2012 (Váňa et al. 2012a).

*** Scapania americana Müll.Frib., Bull. Herb. Boissier (sér. 2) 3 (1): 44, 1903 (Müller 1903).

** **sect. Apiculatae H.Buch**, Scapan. N.-Eur. Sib.: 53, 1928 (Buch 1928).

*** Scapania apiculata Spruce, Hep. Pyr. Exsic.: no. 15, 1847 (Spruce 1847).

*** Scapania carinthiaca J.B.Jack ex Lindb., Rev. Bryol. 7 (4): 77, 1880 (Lindberg 1880a).

*** Scapania carinthiaca var. massalongi Müll.Frib., Bull. Herb. Boissier (sér. 2) 1 (6): 598, 1901 (Müller 1901a).

*** Scapania umbrosa (Schrad.) Dumort., Recueil Observ. Jungerm.: 14, 1835 (Dumortier 1835). Bas.: Jungermannia umbrosa Schrad., Syst. Samml. Crypt. Gew. 2: 5, 1797 (Schrader 1797).

** **sect. Ciliatae Grolle**, Khumbu Himal 1 (4): 272, 1966 (Grolle 1966k).

*** Scapania bhutanensis Amakawa, Fl. E. Himalaya 2: 230, 1971 (Hattori 1971a).

*** Scapania ciliata Sande Lac., Prolus. fl. jap.: 209, 1867 (Sande Lacoste 1867).

*** Scapania ciliata subsp. hawaiica (Müll.Frib.) Potemkin, Ann. Bot. Fenn. 39 (4): 321, 2002 (Potemkin 2002). Bas.: Scapania hawaiica Müll.Frib., Nova Acta Acad. Caes. Leop.-Carol. German. Nat. Cur. 83: 160, 1905 (Müller 1905).

*** Scapania hirosakiensis Steph. ex Müll.Frib., Nova Acta Acad. Caes. Leop.-Carol. German. Nat. Cur. 83: 120, 1905 (Müller 1905).

*** Scapania hollandiae W.S.Hong, Bryologist 83 (1): 56, 1980 (Hong 1980).

*** Scapania koponenii Potemkin, Ann. Bot. Fenn. 37 (1): 41, 2000 (Potemkin 2000c).

*** Scapania lepida Mitt., J. Proc. Linn. Soc., Bot. 5 (18): 101, 1860 [1861] (Mitten 1860c).

*** Scapania sandei Schiffn. ex Müll.Frib., Bull. Herb. Boissier (sér. 2) 1 (6): 612, 1901 (Müller 1901a).

** **sect. Compactae (Müll.Frib.) H.Buch**, Scapan. N.-Eur. Sib.: 101, 1928 (Buch 1928). Bas.: Scapania Gruppe Compactae Müll.Frib., Nova Acta Acad. Caes. Leop.-Carol. German. Nat. Cur. 83: 53, 1905 (Müller 1905).

*** Scapania compacta (Roth) Dumort., Recueil Observ. Jungerm.: 14, 1835 (Dumortier 1835). Bas.: Jungermannia compacta Roth, Tent. Fl. Germ. 3: 375, 1800 (Roth 1800).

*** Scapania kaurinii Ryan, Bot. Not. 42: 210, 1889 (Ryan 1889).

*** Scapania spitsbergensis (Lindb.) Müll.Frib., Bull. Herb. Boissier (sér. 2) 1 (6): 607, 1901 (Müller 1901a). Bas.: Martinellius spitsbergensis Lindb., Kongl. Svenska Vetensk.-Akad. Handl. (n.ser.) 23 (5): 31, 1889 (Lindberg and Arnell 1889).

** **sect. Curtae (Müll.Frib.) H.Buch**, Scapan. N.-Eur. Sib.: 55, 1928 (Buch 1928). Bas.: Scapania Gruppe Curtae Müll.Frib., Nova Acta Acad. Caes. Leop.-Carol. German. Nat. Cur. 83: 245, 1905 (Müller 1905).

*** Scapania curta (Mart.) Dumort., Recueil Observ. Jungerm.: 14, 1835 (Dumortier 1835). Bas.: Jungermannia curta Mart., Fl. crypt. erlang.: 148, 1817 (Martius 1817).

** Scapania curta var. grandiretis R.M.Schust., Hepat. Anthocerotae N. Amer. 3: 393, 1974 (Schuster 1974).

** Scapania curta var. isoloba R.M.Schust., Hepat. Anthocerotae N. Amer. 3: 396, 1974 (Schuster 1974).

*** Scapania diplophylloides Amakawa et S.Hatt., J. Hattori Bot. Lab. 9: 59, 1953 (Amakawa and Hattori 1953).

*** Scapania esterhuyseniae S.W.Arnell, Bot. Not. 110 (1): 26, 1957 (Arnell 1957a).

*** Scapania fulfordiae W.S.Hong, Bryologist 83 (1): 46, 1980 (Hong 1980).

*** Scapania gamundiae R.M.Schust., Bull. Natl. Sci. Mus. Tokyo (n.ser.) 11 (1): 14, 1968 (Schuster 1968a).

*** Scapania helvetica Gottsche, Hepat. Eur., Leberm. 42-44: no. 426, 1868 (Gottsche and Rabenhorst 1868).

*** Scapania irrigua (Nees) Nees, Syn. Hepat. 1: 67, 1844 (Gottsche et al. 1844). Bas.: Jungermannia irrigua Nees, Naturgesch. Eur. Leberm. 1: 193, 1833 (Nees 1833c).

** Scapania irrigua subsp. rufescens (Loeske) R.M.Schust., Hepat. Anthocerotae N. Amer. 3: 471, 1974 (Schuster 1974). Bas.: Scapania irrigua f. rufescens Loeske, Moosfl. Harz.: 71, 1903 (Loeske 1903).

*** Scapania lingulata H.Buch, Meddel. Soc. Fauna Fl. Fenn. 42: 92, 1916 (Buch 1916).

** Scapania lingulata var. microphylla (Warnst.) R.M.Schust., Hepat. Anthocerotae N. Amer. 3: 415, 1974 (Schuster 1974). Bas.: Scapania microphylla Warnst., Hedwigia 63 (2): 75, 1921 (Warnstorf 1921).

** Scapania magadanica S.S.Choi, Bakalin et B.Y.Sun, Bot. Pacifica 1: 46, 2012 (Choi et al. 2012).

*** Scapania mucronata H.Buch, Meddel. Soc. Fauna Fl. Fenn. 42: 91, 1916 (Buch 1916).

*** Scapania obcordata (Berggr.) S.W.Arnell, Ark. Bot. (n.ser.) 4 (6): 117, 1959 (Arnell and Mårtensson 1959). Bas.: Sarcocyphos obcordatus Berggr., Kongl. Svenska Vetensk.-Akad. Handl. (n.ser.) 13 (7): 96, 1875 (Berggren 1875).

** Scapania parvifolia Warnst., Hedwigia 63 (2): 78, 1921 (Warnstorf 1921).

*** Scapania praetervisa Meyl., Jahresber. Naturf. Ges. Graubündens (n.f.) 64: 364, 1926 (Meylan 1926).

*** Scapania scandica (Arnell et H.Buch) Macvicar, Stud. handb. Brit. hepat. (ed. 2): 394, 1926 (Macvicar 1926). Bas.: Martinellius scandicus Arnell et H.Buch, Bot. Not. 74: 1, 1921 (Arnell and Buch 1921).

** Scapania scandica var. argutedentata H.Buch, Scapan. N.-Eur. Sib.: 75, 1928 (Buch 1928).

** Scapania scandica var. dimorpha R.M.Schust., Hepat. Anthocerotae N. Amer. 3: 453, 1974 (Schuster 1974).

** Scapania scandica var. grandiretis (Schljakov) Schljakov, Pečen. Mchi Sev. SSSR 4: 152, 1981 (Shliakov 1981). Bas.: Scapania parvifolia var. grandiretis Schljakov, Novosti Sist. Nizš. Rast. 8: 332, 1971 (Shliakov 1971).

*** Scapania uliginosa (Lindenb.) Dumort., Recueil Observ. Jungerm.: 14, 1835 (Dumortier 1835). Bas.: Jungermannia undulata var. uliginosa Lindenb., Syn. hepat. eur: 58, 1829 (Lindenberg 1829).

*** Scapania valdonii Váňa, Bedn.-Ochyra et Cykowska, Nova Hedwigia 89 (1/2): 126, 2009 (Váňa et al. 2009).

*** Scapania zemliae S.W.Arnell, Svensk Bot. Tidskr. 41: 215, 1947 (Arnell 1947).

** **sect. Cuspiduligerae H.Buch**, Scapan. N.-Eur. Sib.: 125, 1928 (Buch 1928).

*** Scapania cuspiduligera (Nees) Müll.Frib., Lebermoose 2 (22): 472, 1915 (Müller 1915a). Bas.: Jungermannia cuspiduligera Nees, Naturgesch. Eur. Leberm. 1: 180, 1833 (Nees 1833c).

** Scapania cuspiduligera var. diplophyllopsis R.M.Schust., Hepat. Anthocerotae N. Amer. 3: 361, 1974 (Schuster 1974).

** **sect. Grolleoscapania Potemkin**, Ann. Bot. Fenn. 39 (4): 329, 2002 (Potemkin 2002).

*** Scapania karl-muelleri Grolle, Khumbu Himal 1 (4): 270, 1966 (Grolle 1966k).

** **sect. Hyperboreae Váňa, Hentschel, Joch.Müll. et Heinrichs**, PhytoKeys 10: 16, 2012 (Váňa et al. 2012a).

*** Scapania hyperborea Jørg., Forh. Vidensk.-Selsk. Kristiania 1894 (8): 56, 1894 (Jørgensen 1894).

*** Scapania paludicola Loeske et Müll.Frib., Lebermoose 2 (21): 425, 1915 (Müller 1915b).

** Scapania paludicola var. viridigemma R.M.Schust., Bull. Natl. Mus. Canada 122: 20, 1950 [1951] (Schuster 1950).

*** Scapania tundrae (Arnell) H.Buch, Scapan. N.-Eur. Sib.: 99, 1928 (Buch 1928). Bas.: Martinellius tundrae Arnell, Bot. Not. 74: 289, 1921 (Arnell 1921).

** **sect. Kaalaasia (H.Buch) Jørg.**, Bergens Mus. Skr. (n.ser.) 16: 210, 1934 (Jørgensen 1934). Bas.: Scapania subg. Kaalaasia H.Buch, Scapan. N.-Eur. Sib.: 47, 1928 (Buch 1928).

*** Scapania calcicola (Arnell et J.Perss.) Ingham, Naturalist (Hull) 564: 11, 1904 (Ingham 1904). Bas.: Martinellius calcicola Arnell et J.Perss., Rev. Bryol. 30 (6): 97, 1903 (Arnell 1903).

*** Scapania gymnostomophila Kaal., Bot. Not. 49: 21, 1896 (Kaalaas 1896).

*** Scapania ligulifolia R.M.Schust., Hepat. Anthocerotae N. Amer. 3: 306, 1974 (Schuster 1974).

*** Scapania pseudocalcicola R.M.Schust., Phytologia 63 (5): 327, 1987 (Schuster and Damsholt 1987).

* **Scapania sect. Muelleria Potemkin**, Ann. Bot. Fenn. 39 (4): 320, 2002 (Potemkin 2002).

*** Scapania himalayica Müll.Frib. ex Herzog, Ann. Bryol. 12: 81, 1939 (Herzog 1939b).

*** Scapania schljakovii Potemkin, Ann. Bot. Fenn. 38 (2): 87, 2001 (Potemkin 2001).

** **sect. Nemorosae (Müll.Frib.) H.Buch**, Scapan. N.-Eur. Sib.: 152, 1928 (Buch 1928). Bas.: Scapania Gruppe Nemorosae Müll.Frib., Nova Acta Acad. Caes. Leop.-Carol. German. Nat. Cur. 83: 155, 1905 (Müller 1905).

*** Scapania brevicaulis Taylor, London J. Bot. 5: 272, 1846 (Taylor 1846a).

*** Scapania crassiretis Bryhn, Rev. Bryol. 19 (1): 7, 1892 (Bryhn 1892).

* Scapania degenii Schiffn. ex Müll.Frib., Lebermoose 2 (22): 497, 1915 (Müller 1915a). ^60^

* Scapania glaucoviridis Horik., J. Sci. Hiroshima Univ., Ser. B, Div. 2, Bot. 2: 221, 1934 (Horikawa 1934). ^61^

** Scapania grossidens Steph. ex Müll.Frib., Nova Acta Acad. Caes. Leop.-Carol. German. Nat. Cur. 83: 146, 1905 (Müller 1905).

*** Scapania hedbergii S.W.Arnell, Ark. Bot. (n.ser.) 3 (16): 556, 1956 (Arnell 1956e).

*** Scapania integerrima Steph., Sp. Hepat. (Stephani) 4: 148, 1910 (Stephani 1910b).

*** Scapania matveyevae Potemkin, Arctoa 9: 97, 2000 (Potemkin 2000a).

*** Scapania nemorea (L.) Grolle, Rev. Bryol. Lichénol. 32 (3/4): 160, 1963 [1964] (Grolle 1963d). Bas.: Jungermannia nemorea L., Syst. Nat., ed. 10., 2: 1337, 1759 (Linnaeus 1759).

** Scapania parvidens Steph., Hedwigia 44 (1): 15, 1904 (Stephani 1904i). ^62^

*** Scapania parvitexta Steph., Bull. Herb. Boissier 5 (2): 107, 1897 (Stephani 1897b).

*** Scapania rigida Nees, Syn. Hepat. 1: 69, 1844 (Gottsche et al. 1844).

** **sect. Scapania**

*** Scapania gigantea Horik., J. Sci. Hiroshima Univ., Ser. B, Div. 2, Bot. 1: 15, 1931 (Horikawa 1931b).

** Scapania grandiloba Steph., Sp. Hepat. (Stephani) 6: 502, 1924 (Stephani 1924).

*** Scapania komagadakensis Amakawa, J. Hattori Bot. Lab. 31: 96, 1968 (Amakawa 1968a).

*** Scapania obscura (Arnell et C.E.O.Jensen) Schiffn., Österr. Bot. Z. 58 (10): 377, 1908 (Schiffner 1908b). Bas.: Martinellius obscurus Arnell et C.E.O.Jensen, Moose Sarekgeb.: 91, 1907 (Arnell and Jensen 1907).

** Scapania paludosa (Müll.Frib.) Müll.Frib., Mitt. Bad. Bot. Vereins 4 (182/183): 287, 1902 (Müller 1902). Bas.: Scapania undulata var. paludosa Müll.Frib., Beih. Bot. Centralbl. 10 (4/5): 220, 1901 (Müller 1901b).

*** Scapania rufidula Warnst., Hedwigia 63 (2): 94, 1921 (Warnstorf 1921).

*** Scapania serrulata R.M.Schust., Hepat. Anthocerotae N. Amer. 3: 539, 1974 (Schuster 1974).

*** Scapania subalpina (Nees ex Lindenb.) Dumort., Recueil Observ. Jungerm.: 14, 1835 (Dumortier 1835). Bas.: Jungermannia subalpina Nees ex Lindenb., Syn. hepat. eur: 55, 1829 (Lindenberg 1829).

** Scapania subalpina var. haynesiae Frye et L.Clark, Univ. Wash. Publ. Biol. 6 (4): 638, 1946 (Frye and Clark 1946).

** Scapania subalpina var. muddiae C.D.Bird et W.S.Hong, Bryologist 83 (1): 51, 1980 (Hong 1980).

*** Scapania undulata (L.) Dumort., Recueil Observ. Jungerm.: 14, 1835 (Dumortier 1835). Bas.: Jungermannia undulata L., Sp. Pl. 1: 1132, 1753 (Linnaeus 1753).

** **sect. Scapaniella (H.Buch) Potemkin**, J. Hattori Bot. Lab. 85: 43, 1998 (Potemkin 1998). Bas.: Scapaniella H.Buch, Scapan. N.-Eur. Sib.: 33, 1928 (Buch 1928).

*** Scapania glaucocephala (Taylor) Austin, Bull. Torrey Bot. Club 6 (16): 85, 1876 (Austin 1876c). Bas.: Jungermannia glaucocephala Taylor, London J. Bot. 5: 277, 1846 (Taylor 1846a).

*** Scapania glaucocephala var. saxicola (R.M.Schust.) Potemkin, Bryologist 102 (1): 36, 1999 (Potemkin 1999). Bas.: Scapania saxicola R.M.Schust., Amer. Midl. Naturalist 49 (2): 448, 1953 (Schuster 1953).

* Scapania scapanioides (C.Massal.) Grolle, Feddes Repert. 87 (3/4): 235, 1976 (Grolle 1976a). Bas.: Jungermannia scapanioides C.Massal., Hepaticol. ven.: 64, 1879 (Massalongo 1879). ^63^

** **sect. Simmonsiae (R.M.Schust.) Váňa, Hentschel, Joch.Müll. et Heinrichs**, PhytoKeys 10: 16, 2012 (Váňa et al. 2012a). Bas.: Scapania subsect. Simmonsiae R.M.Schust., Hepat. Anthocerotae N. Amer. 3: 612, 1974 (Schuster 1974).

*** Scapania simmonsii Bryhn et Kaal., Rep. Second Norweg. Arctic Exped. 11: 51, 1906 (Bryhn 1906).

** **sect. Sphaeriferae Konstant. et Potemkin**, Ann. Bot. Fenn. 31 (2): 125, 1994 (Konstantinova and Potemkin 1994).

*** Scapania sphaerifera H.Buch et Tuom., Memoranda Soc. Fauna Fl. Fennica 11: 227, 1936 (Buch and Tuomikoski 1936).

** **sect. Stephaniae Potemkin**, J. Hattori Bot. Lab. 85: 57, 1998 (Potemkin 1998). Based on: Scapania sect. Stephaniae Amakawa et S.Hatt., J. Hattori Bot. Lab. 12: 94, 1954 (Amakawa and Hattori 1954). *nom. inval*.

*** Scapania griffithii Schiffn., Österr. Bot. Z. 49 (6): 204, 1899 (Schiffner 1899a).

*** Scapania javanica Gottsche, Natuurk. Tijdschr. Ned.-Indië 4: 575, 1853 (Gottsche 1853).

* Scapania javanica var. scabra Schiffn., Arch. Hydrobiol., suppl. 21 (3/4): 397, 1955 (Schiffner 1955).

*** Scapania ligulata Steph., Hedwigia 44 (1): 14, 1904 (Stephani 1904i).

** Scapania ligulata subsp. stephanii (Müll.Frib.) Potemkin, Piippo et T.J.Kop., Ann. Bot. Fenn. 41 (6): 423, 2004 (Potemkin et al. 2004). Bas.: Scapania stephanii Müll.Frib., Nova Acta Acad. Caes. Leop.-Carol. German. Nat. Cur. 83: 273, 1905 (Müller 1905).

** **sect. Verrucosae Potemkin**, J. Hattori Bot. Lab. 85: 54, 1998 (Potemkin 1998).

*** Scapania udarii S.C.Srivast. et A.Srivast., J. Indian Bot. Soc. 72: 237, 1993 (Srivastava and Srivastava 1993).

*** Scapania verrucosa Heeg, Rev. Bryol. 20 (5): 81, 1893 (Heeg 1893).

*** **Schistochilopsis (N.Kitag.) Konstant.**, Arctoa 3: 125, 1994 (Konstantinova and Vasil’ev 1994). Bas.: Lophozia subg. Schistochilopsis N.Kitag., J. Hattori Bot. Lab. 28: 289, 1965 (Kitagawa 1965).

*** Schistochilopsis cornuta (Steph.) Konstant., Arctoa 3: 125, 1994 (Konstantinova and Vasil’ev 1994). Bas.: Schistochila cornuta Steph., Sp. Hepat. (Stephani) 4: 84, 1909 (Stephani 1909d).

*** Schistochilopsis grandiretis (Lindb. ex Kaal.) Konstant., Arctoa 3: 125, 1994 (Konstantinova and Vasil’ev 1994). Bas.: Jungermannia grandiretis Lindb. ex Kaal., Nyt Mag. Naturvidensk. 33 (4/5): 322, 1893 (Kaalaas 1893b).

** Schistochilopsis hyperarctica Konstant. et L.Söderstr., Phytotaxa 162 (4): 240, 2014 (Konstantinova et al. 2014b). Based on: Lophozia hyperarctica R.M.Schust., Canad. J. Bot. 39 (4): 967, 1961 (Schuster 1961b), *nom. inval*.

*** Schistochilopsis incisa (Schrad.) Konstant., Arctoa 3: 125, 1994 (Konstantinova and Vasil’ev 1994). Bas.: Jungermannia incisa Schrad., Syst. Samml. Crypt. Gew. 2: 5, 1797 (Schrader 1797).

* Schistochilopsis nakanishii (Inoue) Konstant., Arctoa 3: 125, 1994 (Konstantinova and Vasil’ev 1994). Bas.: Lophozia nakanishii Inoue, Bull. Natl. Sci. Mus. Tokyo (n.ser.) 9 (1): 37, 1966 (Inoue 1966a). ^64^

** Schistochilopsis opacifolia (Culm. ex Meyl.) Konstant., Arctoa 3: 125, 1994 (Konstantinova and Vasil’ev 1994). Bas.: Lophozia opacifolia Culm. ex Meyl., Beitr. Kryptogamenfl. Schweiz 6 (4): 174, 1924 (Meylan 1924). ^65^

*** Schistochilopsis setosa (Mitt.) Konstant., Arctoa 3: 125, 1994 (Konstantinova and Vasil’ev 1994). Bas.: Jungermannia setosa Mitt., J. Proc. Linn. Soc., Bot. 5 (18): 92, 1860 [1861] (Mitten 1860c).

####### Jungermanniineae R.M.Schust. ex Stotler et Crand.-Stotl.

######## *** Acrobolbaceae E.A.Hodgs.

by L. Briscoe and J.J. Engel

** **Enigmella G.A.M.Scott et K.G.Beckm.**, J. Bryol. 17 (2): 297, 1992 (Beckmann and Scott 1992).

** Enigmella thallina G.A.M.Scott et K.G.Beckm., J. Bryol. 17 (2): 297, 1992 (Beckmann and Scott 1992).

######### ** Acrobolboideae R.M.Schust. ex Briscoe

*** **Acrobolbus Nees**, Syn. Hepat. 1: 5, 1844 (Gottsche et al. 1844).

** Acrobolbus africanus (Pearson) Briscoe, Phytotaxa 202 (1): 59, 2015 (Briscoe et al. 2015). Bas.: Tylimanthus africanus Pearson, Forh. Vidensk.-Selsk. Kristiania 1887 (9): 14, 1887 (Pearson 1887b).

*** Acrobolbus anisodontus (Hook.f. et Taylor) Briscoe, Phytotaxa 202 (1): 59, 2015 (Briscoe et al. 2015). Bas.: Jungermannia anisodonta Hook.f. et Taylor, London J. Bot. 4: 79, 1845 (Hooker and Taylor 1845).

** Acrobolbus antillanus R.M.Schust., J. Hattori Bot. Lab. 90: 143, 2001 (Schuster 2001a).

** Acrobolbus azoricus (Grolle et Perss.) Briscoe, Phytotaxa 202 (1): 59, 2015 (Briscoe et al. 2015). Bas.: Tylimanthus azoricus Grolle et Perss., Svensk Bot. Tidskr. 60 (1): 169, 1966 (Grolle and Persson 1966).

*** Acrobolbus caducifolius R.M.Schust., J. Hattori Bot. Lab. 90: 154, 2001 (Schuster 2001a).

*** Acrobolbus ciliatus (Mitt.) Schiffn., Hepat. (Engl.-Prantl): 86, 1893 (Schiffner 1893b). Bas.: Gymnanthe ciliata Mitt., J. Proc. Linn. Soc., Bot. 5 (18): 100, 1860 [1861] (Mitten 1860c).

*** Acrobolbus cinerascens (Lehm. et Lindenb.) Bastow, Pap. & Proc. Roy. Soc. Tasmania 1887: 242, 1888 (Bastow 1888). Bas.: Jungermannia cinerascens Lehm. et Lindenb., Nov. Stirp. Pug. 4: 46, 1832 (Lehmann 1832).

*** Acrobolbus concinnus (Mitt.) Steph., Trans. & Proc. New Zealand Inst. 24: 399, 1892 (Colenso 1892). Bas.: Gymnanthe concinna Mitt., Bot. antarct. voy. III (Fl. Tasman. 2): 230, 1860 (Mitten 1860b).

** Acrobolbus cuneifolius (Steph.) Briscoe, Phytotaxa 202 (1): 59, 2015 (Briscoe et al. 2015). Bas.: Tylimanthus cuneifolius Steph., Bull. Herb. Boissier (sér. 2) 5 (12): 1138 (10), 1905 (Stephani 1905b).

*** Acrobolbus diversifolius R.M.Schust., J. Hattori Bot. Lab. 90: 150, 2001 (Schuster 2001a).

*** Acrobolbus epiphytus (Colenso) Briscoe, Phytotaxa 202 (1): 59, 2015 (Briscoe et al. 2015). Bas.: Marsupidium epiphytum Colenso, Trans. & Proc. New Zealand Inst. 21: 64, 1889 (Colenso 1889).

*** Acrobolbus flavicans (J.J.Engel et Grolle) Briscoe et J.J.Engel, Phytotaxa 202 (1): 59, 2015 (Briscoe et al. 2015). Bas.: Marsupidium flavicans J.J.Engel et Grolle, J. Hattori Bot. Lab. 34: 438, 1971 (Engel and Grolle 1971).

*** Acrobolbus gradsteinii (Grolle) Briscoe, Phytotaxa 202 (1): 59, 2015 (Briscoe et al. 2015). Bas.: Marsupidium gradsteinii Grolle, J. Hattori Bot. Lab. 66: 337, 1989 (Grolle 1989a).

** Acrobolbus integrifolius (A.Evans) Briscoe, Phytotaxa 202 (1): 59, 2015 (Briscoe et al. 2015). Bas.: Tylimanthus integrifolius A.Evans, Trans. Connecticut Acad. Arts 8 (15): 259, 1891 (Evans 1891).

*** Acrobolbus knightii (Mitt.) Briscoe, Phytotaxa 202 (1): 59, 2015 (Briscoe et al. 2015). Bas.: Marsupidium knightii Mitt., Handb. N. Zeal. fl. 2: 753, 1867 (Hooker 1867).

** Acrobolbus kunkelii (Hässel et Solari) Briscoe et J.J.Engel, Phytotaxa 202 (1): 60, 2015 (Briscoe et al. 2015). Bas.: Tylimanthus kunkelii Hässel et Solari, Darwiniana 17: 574, 1972 (Hässel and Solari 1972).

*** Acrobolbus laxus (Lehm. et Lindenb.) Briscoe, Phytotaxa 202 (1): 60, 2015 (Briscoe et al. 2015). Bas.: Plagiochila laxa Lehm. et Lindenb., Sp. Hepat. (Lindenberg) 2-4: 68, 1840 (Lindenberg 1840).

*** Acrobolbus limbatus (Steph.) Briscoe et J.J.Engel, Phytotaxa 202 (1): 60, 2015 (Briscoe et al. 2015). Bas.: Tylimanthus limbatus Steph., Kungl. Svenska Vetensk.-Akad. Handl. (n.ser.) 46 (9): 25, 1911 (Stephani 1911b).

*** Acrobolbus lophocoleoides (Mitt.) Mitt., Handb. N. Zeal. fl. 2: 753, 1867 (Hooker 1867). Bas.: Gymnanthe lophocoleoides Mitt., Bot. antarct. voy. II (Fl. Nov.-Zel. 2): 144, 1854 (Mitten 1854).

** Acrobolbus madeirensis (Grolle et Perss.) Briscoe, Phytotaxa 202 (1): 60, 2015 (Briscoe et al. 2015). Bas.: Tylimanthus madeirensis Grolle et Perss., Svensk Bot. Tidskr. 60 (1): 166, 1966 (Grolle and Persson 1966).

** Acrobolbus mittenii Steph., Bull. Herb. Boissier (sér. 2) 2 (5): 460 (179), 1902 (Stephani 1902f).

*** Acrobolbus ochrophyllus (Hook.f. et Taylor) R.M.Schust., Rev. Bryol. Lichénol. 30 (1/2): 64, 1961 (Schuster 1961a). Bas.: Jungermannia ochrophylla Hook.f. et Taylor, London J. Bot. 3: 368, 1844 (Hooker and Taylor 1844a).

*** Acrobolbus papillosus (J.J.Engel et Glenny) Briscoe, Phytotaxa 202 (1): 60, 2015 (Briscoe et al. 2015). Bas.: Marsupidium papillosum J.J.Engel et Glenny, Nova Hedwigia 87 (3/4): 289, 2008 (Engel and Glenny 2008c).

*** Acrobolbus perpusillus (Colenso) Briscoe, Phytotaxa 202 (1): 60, 2015 (Briscoe et al. 2015). Bas.: Tylimanthus perpusillus Colenso, Trans. & Proc. New Zealand Inst. 19: 286, 1887 (Colenso 1887).

*** Acrobolbus perpusillus var. denticulatus (J.J.Engel et Glenny) Briscoe, Phytotaxa 202 (1): 60, 2015 (Briscoe et al. 2015). Bas.: Marsupidium perpusillum var. denticulatum J.J.Engel et Glenny, Nova Hedwigia 87 (3/4): 284, 2008 (Engel and Glenny 2008c).

*** Acrobolbus plagiochiloides (J.J.Engel et Glenny) Briscoe, Phytotaxa 202 (1): 60, 2015 (Briscoe et al. 2015). Bas.: Marsupidium plagiochiloides J.J.Engel et Glenny, Nova Hedwigia 87 (3/4): 284, 2008 (Engel and Glenny 2008c).

*** Acrobolbus pseudosaccatus (Grolle) Briscoe, Phytotaxa 202 (1): 60, 2015 (Briscoe et al. 2015). Bas.: Tylimanthus pseudosaccatus Grolle, Nova Hedwigia 6 (3/4): 391, 1963 (Grolle 1963c).

*** Acrobolbus renifolius (Hässel et Solari) Briscoe et J.J.Engel, Phytotaxa 202 (1): 60, 2015 (Briscoe et al. 2015). Bas.: Tylimanthus renifolius Hässel et Solari, Darwiniana 17: 583, 1972 (Hässel and Solari 1972).

** Acrobolbus ruwenzorensis (S.W.Arnell) Briscoe, Phytotaxa 202 (1): 60, 2015 (Briscoe et al. 2015). Bas.: Tylimanthus ruwenzorensis S.W.Arnell, Ark. Bot. (n.ser.) 3 (16): 560, 1956 (Arnell 1956e).

*** Acrobolbus saccatus (Hook.) Trevis., Mem. Reale Ist. Lombardo Sci. (Ser. 3), C. Sci. Mat. 4 (13): 423, 1877 (Trevisan 1877). Bas.: Jungermannia saccata Hook., Musci Exot. 1: tab. 16, 1818 (Hooker 1818).

*** Acrobolbus setulosus (Mitt.) Briscoe, Phytotaxa 202 (1): 60, 2015 (Briscoe et al. 2015). Bas.: Gymnanthe setulosa Mitt., Bot. antarct. voy. II (Fl. Nov.-Zel. 2): 144, 1854 (Mitten 1854).

*** Acrobolbus spinifolius R.M.Schust., J. Hattori Bot. Lab. 90: 137, 2001 (Schuster 2001a).

*** Acrobolbus sumatranus (Schiffn.) Briscoe, Phytotaxa 202 (1): 61, 2015 (Briscoe et al. 2015). Bas.: Lophozia sumatrana Schiffn., Denkschr. Kaiserl. Akad. Wiss., Math.-Naturwiss. Kl. 67: 203, 1898 (Schiffner 1898a).

*** Acrobolbus surculosus (Nees) Trevis., Mem. Reale Ist. Lombardo Sci. (Ser. 3), C. Sci. Mat. 4 (13): 423, 1877 (Trevisan 1877). Bas.: Scapania surculosa Nees, Syn. Hepat. 1: 62, 1844 (Gottsche et al. 1844).

*** Acrobolbus tenellus (Taylor) Trevis., Mem. Reale Ist. Lombardo Sci. (Ser. 3), C. Sci. Mat. 4 (13): 423, 1877 (Trevisan 1877). Bas.: Gymnanthe tenella Taylor, Nov. Stirp. Pug. 8: 1, 1844 (Lehmann 1844).

*** Acrobolbus tenellus var. diversifolius (E.A.Hodgs.) Briscoe, Phytotaxa 202 (1): 61, 2015 (Briscoe et al. 2015). Bas.: Tylimanthus diversifolius E.A.Hodgs., Trans. Roy. Soc. New Zealand 85 (4): 575, 1958 (Hodgson 1958).

*** Acrobolbus urvilleanus (Mont.) Trevis., Mem. Reale Ist. Lombardo Sci. (Ser. 3), C. Sci. Mat. 4 (13): 423, 1877 (Trevisan 1877). Bas.: Plagiochila urvilleana Mont., Ann. Sci. Nat. Bot. (sér. 2) 19: 247, 1843 (Montagne 1843).

*** Acrobolbus viridis (Mitt.) Briscoe et J.J.Engel, Phytotaxa 202 (1): 61, 2015 (Briscoe et al. 2015). Bas.: Tylimanthus viridis Mitt., J. Linn. Soc., Bot. 15 (84): 197, 1876 (Mitten 1876b).

*** Acrobolbus wilsonii Nees, Syn. Hepat. 1: 5, 1844 (Gottsche et al. 1844).

** Acrobolbus wilsonii var. andinus Spruce, Trans. & Proc. Bot. Soc. Edinburgh 15: 522, 1885 (Spruce 1885).

**Excluded from the genus**

* Acrobolbus bispinosus (J.B.Jack et Steph.) Steph., Bull. Herb. Boissier (sér. 2) 2 (5): 459 (178), 1902 (Stephani 1902f). Bas.: Tylimanthus bispinosus J.B.Jack et Steph., Hedwigia 31 (1): 26, 1892 (Jack and Stephani 1892). ^66^

######### ** Austrolophozioideae R.M.Schust. ex Crand.-Stotl., Váňa et Stotler

** **Austrolophozia R.M.Schust.**, J. Hattori Bot. Lab. 26: 282, 1963 (Schuster 1963b).

** Austrolophozia andina R.M.Schust., Nova Hedwigia 15: 495, 1968 (Schuster 1968b).

*** Austrolophozia camensis (Steph.) Grolle ex Hässel et Solari, Revista Mus. Argent. Ci. Nat., Bernardino Rivadavia Inst. Nac. Invest. Ci. Nat. Bot. 3 (6): 240, 1970 (Hässel and Solari 1970). Bas.: Tylimanthus camensis Steph., Kungl. Svenska Vetensk.-Akad. Handl. (n.ser.) 46 (9): 24, 1911 (Stephani 1911b).

*** Austrolophozia paradoxa R.M.Schust., J. Hattori Bot. Lab. 26: 282, 1963 (Schuster 1963b).

*** **Goebelobryum Grolle**, J. Hattori Bot. Lab. 25: 135, 1962 (Grolle 1962b).

*** Goebelobryum grossitextum (Steph.) Grolle, J. Hattori Bot. Lab. 25: 137, 1962 (Grolle 1962b). Bas.: Marsupidium grossitextum Steph., Sp. Hepat. (Stephani) 6: 446, 1924 (Stephani 1924).

*** Goebelobryum unguiculatum (Hook.f. et Taylor) Grolle, J. Hattori Bot. Lab. 25: 137, 1962 (Grolle 1962b). Bas.: Jungermannia unguiculata Hook.f. et Taylor, London J. Bot. 5: 279, 1846 (Taylor 1846a).

*** Goebelobryum vermiculare J.J.Engel et Glenny, Nova Hedwigia 95 (3/4): 320, 2012 (Engel and Glenny 2012).

######### ** Lethocoleoideae Grolle

*** **Lethocolea Mitt.**, Handb. N. Zeal. fl. 2: 751, 1867 (Hooker 1867) nom. conserv.

** Lethocolea congesta (Lehm.) S.W.Arnell, Bot. Not. 108: 311, 1955 (Arnell 1955b). Bas.: Jungermannia congesta Lehm., Linnaea 4: 365, 1829 (Lehmann 1829).

*** Lethocolea glossophylla (Spruce) Grolle, Bot. Mag. (Tokyo) 78 (921): 83, 1965 (Grolle 1965c). Bas.: Symphyomitra glossophylla Spruce, Trans. & Proc. Bot. Soc. Edinburgh 15: 503, 1885 (Spruce 1885).

** Lethocolea indica G.Asthana et Maurya, Natl. Acad. Sci. Lett. 37 (6): 535, 2014 (Asthana and Maurya 2014).

*** Lethocolea javanica (Schiffn.) Grolle, Bot. Mag. (Tokyo) 78 (921): 83, 1965 (Grolle 1965c). Bas.: Symphyomitra javanica Schiffn., Denkschr. Kaiserl. Akad. Wiss., Math.-Naturwiss. Kl. 67: 193, 1898 (Schiffner 1898a).

** Lethocolea naruto-toganensis Furuki, Bryologist 104 (2): 306, 2001 (Furuki 2001).

*** Lethocolea pansa (Taylor) G.A.M.Scott et K.G.Beckm., Symp. Biol. Hung. 35: 212, 1987 (Scott and Beckmann 1987). Bas.: Jungermannia pansa Taylor, London J. Bot. 5: 275, 1846 (Taylor 1846a).

*** Lethocolea radicosa (Lehm. et Lindenb.) Grolle, Bot. Mag. (Tokyo) 78 (921): 83, 1965 (Grolle 1965c). Bas.: Jungermannia radicosa Lehm. et Lindenb., Nov. Stirp. Pug. 6: 35, 1834 (Lehmann 1834).

* Lethocolea repens S.Winkl., Mitt. Inst. Colombo-Alemán Invest. Ci. 3: 67, 1969 (Winkler 1969).

######### ** Saccogynidioideae Crand.-Stotl., Váňa et Stotler

*** **Saccogynidium Grolle**, J. Hattori Bot. Lab. 23: 43, 1960 [1961] (Grolle 1960d).

** **sect. Decurvum Grolle**, J. Hattori Bot. Lab. 23: 59, 1960 (Grolle 1960d).

*** Saccogynidium decurvum (Mitt.) Grolle, J. Hattori Bot. Lab. 23: 59, 1960 [1961] (Grolle 1960d). Bas.: Lophocolea decurva Mitt., Bot. antarct. voy. III (Fl. Tasman. 2): 227, 1860 (Mitten 1860b).

** **sect. Jugata Grolle**, J. Hattori Bot. Lab. 23: 55, 1960 (Grolle 1960d).

** Saccogynidium rigidulum (Nees) Grolle, J. Hattori Bot. Lab. 23: 52, 1960 [1961] (Grolle 1960d). Bas.: Jungermannia rigidula Nees, Enum. Pl. Crypt. Javae: 25, 1830 (Nees 1830).

** **sect. Saccogynidium**

*** Saccogynidium australe (Mitt.) Grolle, J. Hattori Bot. Lab. 23: 49, 1960 [1961] (Grolle 1960d). Bas.: Saccogyna australis Mitt., Bot. antarct. voy. II (Fl. Nov.-Zel. 2): 145, 1854 (Mitten 1854).

** Saccogynidium caldense (Ångstr.) Grolle, J. Hattori Bot. Lab. 23: 44, 1960 [1961] (Grolle 1960d). Bas.: Chiloscyphus caldensis Ångstr., Öfvers. Kongl. Vetensk.-Akad. Förh. 33 (7): 80, 1876 [1877] (Ångström 1876).

** Saccogynidium goebelii (Herzog) Grolle, J. Hattori Bot. Lab. 23: 52, 1960 [1961] (Grolle 1960d). Bas.: Leioscyphus goebelii Herzog, Ann. Bryol. 5: 89, 1932 (Herzog 1932a).

** Saccogynidium muricellum (De Not.) Grolle, J. Hattori Bot. Lab. 36: 80, 1972 [1973] (Grolle and Schultze-Motel 1972). Bas.: Chiloscyphus muricellus De Not., Epat. Borneo: 24, 1874 (De Notaris 1874).

*** Saccogynidium vasculosum (Hook.f. et Taylor) Grolle, J. Hattori Bot. Lab. 23: 46, 1960 [1961] (Grolle 1960d). Bas.: Jungermannia vasculosa Hook.f. et Taylor, London J. Bot. 3: 461, 1844 (Hooker and Taylor 1844b).

***Incertae sedis***

** Saccogynidium chiloscyphoides R.M.Schust., J. Hattori Bot. Lab. 26: 272, 1963 (Schuster 1963b).

** Saccogynidium irregularospinum C.Gao, T.Cao et M.J.Lai, Bryologist 104 (1): 129, 2001 (Gao et al. 2001).

######## *** Antheliaceae R.M.Schust.

*** **Anthelia (Dumort.) Dumort.**, Recueil Observ. Jungerm.: 18, 1835 (Dumortier 1835). Bas.: Jungermannia sect. Anthelia Dumort., Syll. Jungerm. Europ.: 63, 1831 (Dumortier 1831).

*** Anthelia julacea (L.) Dumort., Recueil Observ. Jungerm.: 18, 1835 (Dumortier 1835). Bas.: Jungermannia julacea L., Sp. Pl., ed. 2: 1601, 1763 (Linnaeus 1763).

** Anthelia juratzkana (Limpr.) Trevis., Mem. Reale Ist. Lombardo Sci. (Ser. 3), C. Sci. Mat. 4 (13): 416, 1877 (Trevisan 1877). Bas.: Jungermannia juratzkana Limpr., Hedwigia 15 (2): 18, 1876 (Limpricht 1876).

######## *** Arnelliaceae Nakai

Crandall-Stotler et al. (2009) placed Stephaniella and Stephaniellidium in Arnelliaceae following de Roo et al. (2007). However, Váňa et al. (2012e) argued based on further molecular evidence that Arnelliaceae should be retained as a monotypic family with a single species.

*** **Arnellia Lindb.**, Kongl. Svenska Vetensk.-Akad. Handl. (n.ser.) 23 (5): 35, 1889 (Lindberg and Arnell 1889).

*** Arnellia fennica (Gottsche) Lindb., Kongl. Svenska Vetensk.-Akad. Handl. (n.ser.) 23 (5): 35, 1889 (Lindberg and Arnell 1889). Bas.: Jungermannia fennica Gottsche, Hepat. Eur., Leberm. 42-44: no. 418, 1868 (Gottsche and Rabenhorst 1868).

######## *** Balantiopsidaceae H.Buch

by J.J. Engel with contribution by J. Váňa (Neesioscyphus)

The placement of Pseudoisotachis in Balantiopsidaceae is only preliminary (cf. Váňa 2013).

*** **Acroscyphella N.Kitag. et Grolle**, Acta Phytotax. Geobot. 36 (1/3): 58, 1985 (Kitagawa and Grolle 1985). *Nom. nov. pro Acroscyphus* N.Kitag., Acta Phytotax. Geobot. 35 (1/3): 1, 1984 (Kitagawa 1984).

*** Acroscyphella iwatsukii (N.Kitag.) N.Kitag. et Grolle, Acta Phytotax. Geobot. 36 (1/3): 58, 1985 (Kitagawa and Grolle 1985). Bas.: Acroscyphus iwatsukii N.Kitag., Acta Phytotax. Geobot. 35 (1/3): 3, 1984 (Kitagawa 1984).

*** Acroscyphella phoenicorhiza (Grolle) N.Kitag. et Grolle, Acta Phytotax. Geobot. 36 (1/3): 58, 1985 (Kitagawa and Grolle 1985). Bas.: Neesioscyphus phoenicorhizus Grolle, Österr. Bot. Z. 111 (1): 27, 1964 (Grolle 1964e).

*** Acroscyphella tjiwideiensis (Sande Lac.) N.Kitag. et Grolle, Acta Phytotax. Geobot. 36 (1/3): 58, 1985 (Kitagawa and Grolle 1985). Bas.: Chiloscyphus tjiwideiensis Sande Lac., Ned. Kruidk. Arch. 3: 418, 1854 [1855] (Sande Lacoste 1854).

** **Pseudoisotachis Váňa**, Polish Bot. J. 58 (1): 55, 2013 (Váňa 2013).

** Pseudoisotachis pocsii Váňa, Polish Bot. J. 58 (1): 55, 2013 (Váňa 2013).

######### *** Balantiopsidoideae J.J.Engel et Váňa

*** **Balantiopsis Mitt.**, Handb. N. Zeal. fl. 2: 751, 1867 (Hooker 1867).

*** Balantiopsis asymmetrica (Herzog) J.J.Engel, Nova Hedwigia 16: 93, 1968 (Engel 1968). Bas.: Balantiopsis latifolia var. asymmetrica Herzog, Rev. Bryol. Lichénol. 23 (1/2): 53, 1954 (Herzog 1954).

*** Balantiopsis bisbifida (Steph.) Steph., Sp. Hepat. (Stephani) 4: 101, 1910 (Stephani 1910b). Bas.: Isotachis bisbifida Steph., Bih. Kongl. Svenska Vetensk.-Akad. Handl. 26 (III, 17): 24, 1901 (Stephani 1901b).

*** Balantiopsis brasiliensis Steph., Sp. Hepat. (Stephani) 4: 104, 1910 (Stephani 1910b).

*** Balantiopsis cancellata (Nees) Steph., Sp. Hepat. (Stephani) 4: 103, 1910 (Stephani 1910b). Bas.: Ptilidium cancellatum Nees, Syn. Hepat. 2: 251, 1845 (Gottsche et al. 1845a).

*** Balantiopsis ciliaris S.Hatt., J. Jap. Bot. 41 (5): 129, 1966 (Hattori 1966b).

** Balantiopsis ciliaris subsp. novoguineensis S.Hatt., J. Jap. Bot. 41 (5): 131, 1966 (Hattori 1966b).

*** Balantiopsis convexiuscula Berggr., New Zealand Hepat.: 44, 1898 (Berggren 1898).

*** Balantiopsis crocea Herzog, Beih. Bot. Centralbl. 60B (1/2): 12, 1939 (Herzog 1939c).

*** Balantiopsis diplophylla (Hook.f. et Taylor) Mitt., Handb. N. Zeal. fl. 2: 753, 1867 (Hooker 1867). Bas.: Jungermannia diplophylla Hook.f. et Taylor, London J. Bot. 3: 377, 1844 (Hooker and Taylor 1844a).

** Balantiopsis diplophylla var. hockenii (Berggr.) J.J.Engel et G.L.Merr., Fieldiana, Bot. (n.ser.) 37: 11, 1997 (Engel and Smith Merrill 1997). Bas.: Balantiopsis hockenii Berggr., New Zealand Hepat.: 46, 1898 (Berggren 1898).

*** Balantiopsis erinacea (Hook.f. et Taylor) Mitt., Handb. N. Zeal. fl. 2: 753, 1867 (Hooker 1867). Bas.: Jungermannia erinacea Hook.f. et Taylor, London J. Bot. 3: 462, 1844 (Hooker and Taylor 1844b).

*** Balantiopsis lingulata R.M.Schust., Bull. Natl. Sci. Mus. Tokyo (n.ser.) 11 (1): 26, 1968 (Schuster 1968a).

*** Balantiopsis montana (Colenso) J.J.Engel et G.L.Merr., Fieldiana, Bot. (n.ser.) 37: 12, 1997 (Engel and Smith Merrill 1997). Bas.: Chiloscyphus montanus Colenso, Trans. & Proc. New Zealand Inst. 21: 62, 1889 (Colenso 1889).

** Balantiopsis neocaledonica Pearson, J. Linn. Soc., Bot. 46 (305): 28, 1922 (Pearson 1922b).

** Balantiopsis paucidens Steph., Bih. Kongl. Svenska Vetensk.-Akad. Handl. 26 (III, 17): 29, 1901 (Stephani 1901b).

*** Balantiopsis purpurata Mitt., Rep. Challenger, Bot. 1 (3, 1): 86, 1884 (Mitten 1884b).

*** Balantiopsis rosea Berggr., New Zealand Hepat.: 43, 1898 (Berggren 1898).

*** Balantiopsis splendens (Steph.) J.J.Engel et G.L.Merr., Fieldiana, Bot. (n.ser.) 37: 55, 1997 (Engel and Smith Merrill 1997). Bas.: Isotachis splendens Steph., Hedwigia 34 (2): 49, 1895 (Stephani 1895c).

*** Balantiopsis tumida Berggr., New Zealand Hepat.: 45, 1898 (Berggren 1898).

*** Balantiopsis verrucosa J.J.Engel et G.L.Merr., Fieldiana, Bot. (n.ser.) 37: 16, 1997 (Engel and Smith Merrill 1997).

######### *** Isotachidoideae Grolle

*** **Isotachis Mitt.**, Bot. antarct. voy. II (Fl. Nov.-Zel. 2): 148, 1854 (Mitten 1854).

*** Isotachis armata (Nees) Gottsche, Ann. Sci. Nat. Bot. (sér. 5) 1: 121, 1864 (Gottsche 1864). Bas.: Jungermannia armata Nees, Syn. Hepat. 1: 129, 1844 (Gottsche et al. 1844).

*** Isotachis aubertii (Schwägr.) Mitt., J. Linn. Soc., Bot. 22 (146): 322, 1886 (Mitten 1886b). Bas.: Jungermannia aubertii Schwägr., Hist. Musc. Hepat. Prodr.: 19, 1814 (Schwägrichen 1814).

* Isotachis boliviensis Gottsche, Sp. Hepat. (Stephani) 3: 670, 1909 (Stephani 1909a).

** Isotachis chinensis C.Gao, T.Cao et J.Sun, Bryologist 105 (4): 694, 2002 [2003] (Gao et al. 2002).

** Isotachis erythrorhiza (Lehm. et Lindenb.) Spruce, Trans. & Proc. Bot. Soc. Edinburgh 15: 338, 1885 (Spruce 1885). Bas.: Jungermannia erythrorhiza Lehm. et Lindenb., Nov. Stirp. Pug. 4: 44, 1832 (Lehmann 1832).

*** Isotachis fragilis Steph., Kungl. Svenska Vetensk.-Akad. Handl. (n.ser.) 46 (9): 67, 1911 (Stephani 1911b).

** Isotachis grandis Carrington et Pearson, Proc. Linn. Soc. New South Wales (ser. 2) 2 (4): 1041, 1888 (Carrington and Pearson 1888a).

*** Isotachis grossidens Steph., Kungl. Svenska Vetensk.-Akad. Handl. (n.ser.) 46 (9): 69, 1911 (Stephani 1911b).

** Isotachis hastatistipula (Steph.) J.J.Engel, Phytotaxa 183 (4): 299, 2014 (Engel et al. 2014). Bas.: Balantiopsis hastatistipula Steph., J. & Proc. Roy. Soc. New South Wales 48 (1/2): 98, 1914 (Stephani and Watts 1914).

* Isotachis hians Steph., Sp. Hepat. (Stephani) 3: 665, 1909 (Stephani 1909a).

*** Isotachis humectata (Hook.f. et Taylor) Steph., Sp. Hepat. (Stephani) 3: 654, 1909 (Stephani 1909a). Bas.: Jungermannia humectata Hook.f. et Taylor, London J. Bot. 3: 462, 1844 (Hooker and Taylor 1844b).

** Isotachis inflata Steph., Arch. Mus. Nac. Rio de Janeiro 13: 113, 1905 (Stephani 1905c).

*** Isotachis intortifolia (Hook.f. et Taylor) Gottsche, Ann. Sci. Nat. Bot. (sér. 5) 1: 121, 1864 (Gottsche 1864). Bas.: Jungermannia intortifolia Hook.f. et Taylor, London J. Bot. 3: 374, 1844 (Hooker and Taylor 1844a).

** Isotachis japonica Steph., Sp. Hepat. (Stephani) 3: 652, 1909 (Stephani 1909a).

* Isotachis lacustris Herzog, Hedwigia 74 (2): 94, 1934 (Herzog 1934a).

*** Isotachis lopezii (R.M.Schust.) Gradst., Mem. New York Bot. Gard. 84: 66, 1999 (Gradstein 1999). Bas.: Ruizanthus lopezii R.M.Schust., Phytologia 39 (4): 241, 1978 (Schuster 1978a).

*** Isotachis lyallii Mitt., Bot. antarct. voy. II (Fl. Nov.-Zel. 2): 149, 1854 (Mitten 1854).

*** Isotachis minima Pearson, Univ. Calif. Publ. Bot. 10 (4): 322, 1923 (Pearson 1923).

*** Isotachis montana Colenso, Trans. & Proc. New Zealand Inst. 21: 68, 1889 (Colenso 1889).

*** Isotachis multiceps (Lindenb. et Gottsche) Gottsche, Mexik. Leverm.: 105, 1863 (Gottsche 1863). Bas.: Jungermannia multiceps Lindenb. et Gottsche, Syn. Hepat. 5: 687, 1847 (Gottsche et al. 1847). ^67^

** Isotachis multiceps var. fendleri Gottsche, Ann. Sci. Nat. Bot. (sér. 5) 1: 125, 1864 (Gottsche 1864).

** Isotachis nigella Herzog, Memoranda Soc. Fauna Fl. Fennica 27: 107, 1952 (Herzog 1952c).

*** Isotachis obtusa Steph., Sp. Hepat. (Stephani) 6: 354, 1922 (Stephani 1922).

** Isotachis olivacea R.M.Schust., J. Hattori Bot. Lab. 83: 207, 1997 (Schuster and Engel 1997).

*** Isotachis plicata J.J.Engel, J. Hattori Bot. Lab. 83: 210, 1997 (Schuster and Engel 1997).

** Isotachis pusilla Steph., Sp. Hepat. (Stephani) 3: 655, 1909 (Stephani 1909a).

** Isotachis riparia Rodway, Tasm. Bryoph.: 63, 1917 (Rodway 1917b).

*** Isotachis serrulata (Sw.) Gottsche, Ann. Sci. Nat. Bot. (sér. 5) 1: 121, 1864 (Gottsche 1864). Bas.: Jungermannia serrulata Sw., Prodr. (Swartz): 143, 1788 (Swartz 1788).

*** Isotachis spegazziniana C.Massal., Nuovo Giorn. Bot. Ital. 17 (3): 220, 1885 (Massalongo 1885).

* Isotachis sprucei Beauverd, Sp. Hepat. (Stephani) 6: 572, 1924 (Stephani 1924). *Nom. nov. pro Isotachis trifida* Steph., Sp. Hepat. (Stephani) 6: 356, 1922 (Stephani 1922), *nom. illeg*.

*** Isotachis stephanii E.S.Salmon, Rev. Bryol. 28 (4): 75, 1901 (Salmon 1901).

* Isotachis vexans Steph., Sp. Hepat. (Stephani) 3: 662, 1909 (Stephani 1909a).

*** Isotachis westlandica (E.A.Hodgs.) R.M.Schust., Nova Hedwigia 15: 455, 1968 (Schuster 1968b). Bas.: Rhizocaulia westlandica E.A.Hodgs., Trans. Roy. Soc. New Zealand, Bot. 3 (4): 78, 1965 (Hodgson 1965).

*** **Neesioscyphus Grolle**, Österr. Bot. Z. 111 (1): 19, 1964 (Grolle 1964e).

*** Neesioscyphus allionii (Steph.) Grolle, Rev. Bryol. Lichénol. 34 (1/2): 185, 1966 (Grolle 1966c). Bas.: Isotachis allionii Steph., Sp. Hepat. (Stephani) 6: 350, 1922 (Stephani 1922).

*** Neesioscyphus argillaceus (Nees) Grolle, Österr. Bot. Z. 111 (1): 24, 1964 (Grolle 1964e). Bas.: Jungermannia argillacea Nees, Fl. Bras. (Martius) 1 (1): 338, 1833 (Nees 1833a).

*** Neesioscyphus bicuspidatus (Steph.) Grolle, Rev. Bryol. Lichénol. 34 (1/2): 182, 1966 (Grolle 1966c). Bas.: Isotachis bicuspidata Steph., Symb. Antill. 2: 471, 1901 (Stephani 1901f).

*** Neesioscyphus carneus (Nees) Grolle, Österr. Bot. Z. 111 (1): 20, 1964 (Grolle 1964e). Bas.: Jungermannia carnea Nees, Fl. Bras. (Martius) 1 (1): 337, 1833 (Nees 1833a).

*** Neesioscyphus homophyllus (Nees) Grolle, Österr. Bot. Z. 111 (2/3): 188, 1964 (Grolle 1964f). Bas.: Jungermannia homophylla Nees, Fl. Bras. (Martius) 1 (1): 336, 1833 (Nees 1833a).

######### ** Ruizanthoideae R.M.Schust. ex J.J.Engel et G.L.Merr.

*** **Ruizanthus R.M.Schust.**, Phytologia 39 (4): 240, 1978 (Schuster 1978a).

*** Ruizanthus venezuelanus R.M.Schust., Phytologia 39 (4): 240, 1978 (Schuster 1978a).

######## *** Blepharidophyllaceae R.M.Schust. ex J.J.Engel

by J.J. Engel

*** **Blepharidophyllum Ångstr.**, Öfvers. Kongl. Vetensk.-Akad. Förh. 30 (5): 151, 1873 (Ångström 1873).

*** Blepharidophyllum densifolium (Hook.) Ångstr. ex C.Massal., Nuovo Giorn. Bot. Ital. 17 (3): 208, 1885 (Massalongo 1885). Bas.: Jungermannia densifolia Hook., Musci Exot. 1: tab. 36, 1818 (Hooker 1818).

*** Blepharidophyllum vertebrale (Gottsche) Ångstr. ex C.Massal., Nuovo Giorn. Bot. Ital. 17 (3): 208, 1885 (Massalongo 1885). Bas.: Scapania vertebralis Gottsche, Syn. Hepat. 1: 72, 1844 (Gottsche et al. 1844).

*** **Clandarium (Grolle) R.M.Schust.**, New Manual Bryol. 1: 541, 1983 [1984] (Schuster 1983a). Bas.: Blepharidophyllum subg. Clandarium Grolle, J. Hattori Bot. Lab. 28: 65, 1965 (Grolle 1965a).

*** Clandarium clandestinum (Mont.) R.M.Schust., New Manual Bryol. 1: 541, 1983 [1984] (Schuster 1983a). Bas.: Plagiochila clandestina Mont., Ann. Sci. Nat. Bot. (sér. 2) 19: 247, 1843 (Montagne 1843).

*** Clandarium gottscheanum (Grolle) R.M.Schust., New Manual Bryol. 1: 541, 1983 [1984] (Schuster 1983a). Bas.: Blepharidophyllum gottscheanum Grolle, J. Hattori Bot. Lab. 28: 69, 1965 (Grolle 1965a).

*** Clandarium xiphophyllum (Grolle) R.M.Schust., Phytologia 56 (2): 68, 1984 (Schuster 1984). Bas.: Blepharidophyllum xiphophyllum Grolle, J. Hattori Bot. Lab. 28: 65, 1965 (Grolle 1965a).

######## *** Calypogeiaceae Arnell

by M.A.M. Renner

Calypogeiaceae is shown to be monophyletic (Masuzaki et al. 2010b) and the classification follows Schuster (2000a) and Masuzaki et al. (2010b).

*** **Calypogeia Raddi**, Jungermanniogr. Etrusca: 31, 1818 (Raddi 1818a) nom. conserv.

** **subg. Asperifoliae (Warnst.) R.M.Schust.**, Hepat. Anthocerotae N. Amer. 2: 115, 1969 (Schuster 1969b). Bas.: Calypogeia [unranked] Asperifoliae Warnst., Bryol. Z. 1 (7): 111, 1917 (Warnstorf 1917).

*** Calypogeia arguta Nees et Mont., Naturgesch. Eur. Leberm. 3: 24, 1838 (Nees 1838b).

** Calypogeia sullivantii Austin, Hepat. bor.-amer.: 19, 1873 (Austin 1873).

** **subg. Calypogeia**

*** Calypogeia andicola Bischl., Candollea 18: 79, 1962 (Bischler 1962a).

** Calypogeia annabonensis Steph., Sp. Hepat. (Stephani) 6: 447, 1924 (Stephani 1924).

** Calypogeia azorica Bischl., Rev. Bryol. Lichénol. 37 (1): 116, 1970 (Bischler 1970).

*** Calypogeia azurea Stotler et Crotz, Taxon 32 (1): 74, 1983 (Stotler and Crotz 1983).

*** Calypogeia bidentula (F.Weber) Nees, Syn. Hepat. 2: 199, 1845 (Gottsche et al. 1845a). Bas.: Jungermannia bidentula F.Weber, Hist. Musc. Hepat. Prodr.: 38, 1815 (Weber 1815).

** Calypogeia falcata Bischl., Candollea 18: 112, 1962 (Bischler 1962c).

*** Calypogeia fissa (L.) Raddi, Jungermanniogr. Etrusca: 33, 1818 (Raddi 1818a). Bas.: Mnium fissum L., Sp. Pl. 1: 1114, 1753 (Linnaeus 1753), *nom. conserv*. ^68^

** Calypogeia fissa subsp. neogaea R.M.Schust., Hepat. Anthocerotae N. Amer. 2: 169, 1969 (Schuster 1969b).

** Calypogeia goebelii (Schiffn.) Steph., Bull. Herb. Boissier (sér. 2) 8 (9): 677 (409), 1908 (Stephani 1908d). Bas.: Kantius goebelii Schiffn., Nova Acta Acad. Caes. Leop.-Carol. German. Nat. Cur. 60 (2): 260, 1893 (Schiffner 1893a).

** Calypogeia goebelii var. siamensis N.Kitag., Beih. Nova Hedwigia 90: 165, 1988 (Kitagawa 1988).

*** Calypogeia grandistipula (Steph.) Steph., Bull. Herb. Boissier (sér. 2) 8 (9): 669 (401), 1908 (Stephani 1908d). Bas.: Kantius grandistipulus Steph., Hedwigia 34 (2): 52, 1895 (Stephani 1895c).

*** Calypogeia integristipula Steph., Bull. Herb. Boissier (sér. 2) 8 (9): 662 (394), 1908 (Stephani 1908d).

*** Calypogeia laxa Gottsche et Lindenb., Syn. Hepat. 5: 713, 1847 (Gottsche et al. 1847).

*** Calypogeia lechleri (Steph.) Steph., Bull. Herb. Boissier (sér. 2) 8 (9): 680 (412), 1908 (Stephani 1908d). Bas.: Kantius lechleri Steph., Hedwigia 34 (2): 53, 1895 (Stephani 1895c). ^69^

** Calypogeia lechleri var. densifolia (Steph.) Bischl., Candollea 18: 101, 1962 (Bischler 1962c). Bas.: Kantius densifolius Steph., Hedwigia 34 (2): 52, 1895 (Stephani 1895c).

** Calypogeia longifolia Steph., Sp. Hepat. (Stephani) 6: 449, 1924 (Stephani 1924).

** Calypogeia lophocoleoides Steph., Bull. Herb. Boissier (sér. 2) 8 (9): 677 (409), 1908 (Stephani 1908d).

** Calypogeia mascarenensis Bischl., Rev. Bryol. Lichénol. 37 (1): 89, 1970 (Bischler 1970).

** Calypogeia microstipula (Steph.) Steph., Bull. Herb. Boissier (sér. 2) 8 (9): 670 (402), 1908 (Stephani 1908d). Bas.: Kantius microstipulus Steph., Hedwigia 34 (2): 53, 1895 (Stephani 1895c).

*** Calypogeia miquelii Mont. ex Gottsche, Lindenb. et Nees, Syn. Hepat. 2: 200, 1845 (Gottsche et al. 1845a).

*** Calypogeia muelleriana (Schiffn.) Müll.Frib., Beih. Bot. Centralbl. 10 (4/5): 217, 1901 (Müller 1901b). Bas.: Kantius muellerianus Schiffn., Sitzungsber. deutsch. naturwiss.-med. Vereins Böhmen “Lotos” Prag 48: 342, 1900 (Schiffner 1900d). ^70^

** Calypogeia muelleriana subsp. blomquistii R.M.Schust., Hepat. Anthocerotae N. Amer. 2: 187, 1969 (Schuster 1969b).

*** Calypogeia neesiana (C.Massal. et Carestia) Müll.Frib., Verh. Bot. Vereins Prov. Brandenburg 47: 320, 1905 (Loeske 1905). Bas.: Kantius trichomanis var. neesianus C.Massal. et Carestia, Nuovo Giorn. Bot. Ital. 12 (4): 351, 1880 (Massalongo and Carestia 1880).

** Calypogeia neesiana subsp. subalpina (Inoue) Inoue, Mem. Natl. Sci. Mus. (Tokyo) 4: 58, 1971 (Inoue 1971a). Bas.: Calypogeia subalpina Inoue, J. Jap. Bot. 37 (4): 103, 1962 (Inoue 1962b).

*** Calypogeia oblata Herzog, Svensk Bot. Tidskr. 51 (1): 189, 1957 (Herzog 1957a).

*** Calypogeia peruviana Nees et Mont., Ann. Sci. Nat. Bot. (sér. 2) 9: 47, 1838 (Montagne 1838).

*** Calypogeia rhombifolia (Spruce) Steph., Bull. Herb. Boissier (sér. 2) 8 (9): 667 (399), 1908 (Stephani 1908d). Bas.: Kantius rhombifolius Spruce, Trans. & Proc. Bot. Soc. Edinburgh 15: 413, 1885 (Spruce 1885).

** Calypogeia rhombifolia var. colombiana Bischl., Candollea 18: 104, 1962 (Bischler 1962c).

*** Calypogeia sphagnicola (Arnell et J.Perss.) Warnst. et Loeske, Verh. Bot. Vereins Prov. Brandenburg 47: 320, 1905 (Loeske 1905). Bas.: Kantius sphagnicola Arnell et J.Perss., Rev. Bryol. 29 (2): 26, 1902 (Arnell 1902). ^71^

*** Calypogeia subintegra (Gottsche, Lindenb. et Nees) Bischl., Candollea 18: 75, 1962 (Bischler 1962a). Bas.: Calypogeia peruviana β subintegra Gottsche, Lindenb. et Nees, Syn. Hepat. 5: 712, 1847 (Gottsche et al. 1847).

** Calypogeia subintegra var. dussiana (Steph.) Bischl., Candollea 18: 77, 1962 (Bischler 1962a). Bas.: Calypogeia dussiana Steph., Bull. Herb. Boissier (sér. 2) 8 (9): 672 (404), 1908 (Stephani 1908d).

*** Calypogeia suecica (Arnell et J.Perss.) Müll.Frib., Beih. Bot. Centralbl. 17 (2): 224, 1904 (Müller 1904). Bas.: Kantius suecicus Arnell et J.Perss., Rev. Bryol. 29 (2): 29, 1902 (Arnell 1902).

*** Calypogeia tenax (Spruce) Steph., Bull. Herb. Boissier (sér. 2) 8 (9): 664 (396), 1908 (Stephani 1908d). Bas.: Kantius tenax Spruce, Trans. & Proc. Bot. Soc. Edinburgh 15: 416, 1885 (Spruce 1885).

** Calypogeia uncinulatula Herzog, Hedwigia 67 (6): 250, 1927 (Herzog 1927).

***Incertae sedis***

*** Calypogeia aeruginosa Mitt., J. Proc. Linn. Soc., Bot. 5 (18): 107, 1860 [1861] (Mitten 1860c).

* Calypogeia amazonica (Spruce) Steph., Bull. Herb. Boissier (sér. 2) 8 (9): 680 (412), 1908 (Stephani 1908d). Bas.: Kantius amazonicus Spruce, Trans. & Proc. Bot. Soc. Edinburgh 15: 415, 1885 (Spruce 1885). ^72^

** Calypogeia angusta Steph., Bull. Herb. Boissier (sér. 2) 8 (9): 663 (395), 1908 (Stephani 1908d).

** Calypogeia apiculata (Steph.) Steph., Bull. Herb. Boissier (sér. 2) 8 (9): 668 (400), 1908 (Stephani 1908d). Bas.: Kantius apiculatus Steph., Hedwigia 34 (2): 51, 1895 (Stephani 1895c).

** Calypogeia asakawana S.Hatt. ex Inoue, J. Jap. Bot. 39 (4): 107, 1964 (Inoue 1964a).

** Calypogeia ceylanica S.Hatt. et Mizut., Candollea 23: 288, 1968 (Hattori 1968).

** Calypogeia contracta Inoue, Bull. Natl. Sci. Mus. Tokyo, B 1 (4): 139, 1975 (Inoue 1975b).

** Calypogeia cuspidata (Steph.) Steph., Bull. Herb. Boissier (sér. 2) 8 (9): 669 (401), 1908 (Stephani 1908d). Bas.: Kantius cuspidatus Steph., Bull. Herb. Boissier 5 (10): 846, 1897 (Stephani 1897c).

* Calypogeia decurrens (Steph.) Steph., Bull. Herb. Boissier (sér. 2) 8 (9): 675 (407), 1908 (Stephani 1908d). Bas.: Kantius decurrens Steph., Hedwigia 34 (2): 52, 1895 (Stephani 1895c).

** Calypogeia formosana Horik., J. Sci. Hiroshima Univ., Ser. B, Div. 2, Bot. 2: 186, 1934 (Horikawa 1934).

** Calypogeia fujisana Inoue, Bull. Natl. Sci. Mus. Tokyo, B 1 (4): 135, 1975 (Inoue 1975b).

** Calypogeia granulata Inoue, J. Jap. Bot. 43 (10/11): 468, 1968 (Inoue 1968b).

** Calypogeia japonica Steph., Sp. Hepat. (Stephani) 6: 448, 1924 (Stephani 1924).

** Calypogeia khasiana Ajit P.Singh et V.Nath, Taiwania 52 (4): 320, 2007 (Singh and Nath 2007a).

** Calypogeia latissima Steph., Sp. Hepat. (Stephani) 6: 449, 1924 (Stephani 1924).

*** Calypogeia lunata Mitt., J. Proc. Linn. Soc., Bot. 5 (18): 107, 1860 [1861] (Mitten 1860c).

** Calypogeia marginella Mitt., J. Proc. Linn. Soc., Bot. 5 (18): 106, 1860 [1861] (Mitten 1860c).

** Calypogeia obovata R.M.Schust., Phytologia 39 (4): 242, 1978 (Schuster 1978a).

* Calypogeia steyermarkii Fulford, Mem. New York Bot. Gard. 11 (3): 305, 1968 (Fulford 1968).

** Calypogeia tosana (Steph.) Steph., Bull. Herb. Boissier (sér. 2) 8 (9): 678 (410), 1908 (Stephani 1908d). Bas.: Kantius tosanus Steph., Hedwigia 34 (2): 54, 1895 (Stephani 1895c).

** Calypogeia udarii Sudipa Das et D.K.Singh, Nelumbo 53: 194, 2011 (Das and Singh 2011).

** **Eocalypogeia (R.M.Schust.) R.M.Schust.**, Fragm. Florist. Geobot. 40 (2): 861, 1995 (Schuster 1995a). Bas.: Metacalypogeia subg. Eocalypogeia R.M.Schust., Hepat. Anthocerotae N. Amer. 2: 107, 1969 (Schuster 1969b).

** Eocalypogeia quelpaertensis (S.Hatt. et Inoue) R.M.Schust., Fragm. Florist. Geobot. 40 (2): 861, 1995 (Schuster 1995a). Bas.: Metacalypogeia quelpaertensis S.Hatt. et Inoue, J. Hattori Bot. Lab. 25: 129, 1962 (Hattori et al. 1962).

** Eocalypogeia schusterana (S.Hatt. et Mizut.) R.M.Schust., Fragm. Florist. Geobot. 40 (2): 861, 1995 (Schuster 1995a). Bas.: Metacalypogeia schusterana S.Hatt. et Mizut., Misc. Bryol. Lichenol. 4 (8): 121, 1967 (Hattori and Mizutani 1967).

** **Metacalypogeia (S.Hatt.) Inoue**, J. Hattori Bot. Lab. 21: 231, 1959 (Inoue 1959b). Bas.: Calypogeia subg. Metacalypogeia S.Hatt., J. Hattori Bot. Lab. 18: 83, 1957 (Hattori 1957b).

*** Metacalypogeia alternifolia (Nees) Grolle, Österr. Bot. Z. 111 (2/3): 185, 1964 (Grolle 1964f). Bas.: Mastigobryum alternifolium Nees, Syn. Hepat. 2: 216, 1845 (Gottsche et al. 1845a).

** Metacalypogeia cordifolia (Steph.) Inoue, J. Hattori Bot. Lab. 21: 233, 1959 (Inoue 1959b). Bas.: Calypogeia cordifolia Steph., Bull. Herb. Boissier (sér. 2) 8 (9): 661 (393), 1908 (Stephani 1908d).

** **Mizutania Furuki et Z.Iwats.**, J. Hattori Bot. Lab. 67: 291, 1989 (Furuki and Iwatsuki 1989).

*** Mizutania riccardioides Furuki et Z.Iwats., J. Hattori Bot. Lab. 67: 291, 1989 (Furuki and Iwatsuki 1989).

*** **Mnioloma Herzog**, Ann. Bryol. 3: 115, 1930 (Herzog 1930a).

** **subg. Caracoma (Bischl.) R.M.Schust.**, Fragm. Florist. Geobot. 40 (2): 833, 1995 (Schuster 1995a). Bas.: Calypogeia subg. Caracoma Bischl., Candollea 18: 26, 1962 (Bischler 1962b).

** Mnioloma bolivianum (Fulford) R.M.Schust., Beih. Nova Hedwigia 118: 509, 2000 (Schuster 2000a). Bas.: Calypogeia boliviana Fulford, Mem. New York Bot. Gard. 11 (3): 291, 1968 (Fulford 1968).

*** Mnioloma caespitosum (Spruce) R.M.Schust., Fragm. Florist. Geobot. 40 (2): 839, 1995 (Schuster 1995a). Bas.: Kantius caespitosus Spruce, Trans. & Proc. Bot. Soc. Edinburgh 15: 412, 1885 (Spruce 1885).

*** Mnioloma cellulosum (Spreng.) R.M.Schust., Fragm. Florist. Geobot. 40 (2): 836, 1995 (Schuster 1995a). Bas.: Jungermannia cellulosa Spreng. Syst. Veg. (ed. 16) [Sprengel] 4 (1): 232, 1827 (Sprengel 1827a).

*** Mnioloma crenulatum (Bischl.) R.M.Schust., Fragm. Florist. Geobot. 40 (2): 839, 1995 (Schuster 1995a). Bas.: Calypogeia crenulata Bischl., Candollea 18: 35, 1962 (Bischler 1962b).

*** Mnioloma cyclostipum (Spruce) R.M.Schust., Fragm. Florist. Geobot. 40 (2): 843, 1995 (Schuster 1995a). Bas.: Kantius cyclostipus Spruce, Trans. & Proc. Bot. Soc. Edinburgh 15: 411, 1885 (Spruce 1885).

** Mnioloma elliottii (Steph.) R.M.Schust., Fragm. Florist. Geobot. 40 (2): 841, 1995 (Schuster 1995a). Bas.: Calypogeia elliottii Steph., Bull. Herb. Boissier (sér. 2) 8 (9): 663 (395), 1908 (Stephani 1908d).

** Mnioloma fissistipulum (Bischl.) R.M.Schust., Fragm. Florist. Geobot. 40 (2): 847, 1995 (Schuster 1995a). Bas.: Calypogeia fissistipula Bischl., Candollea 18: 47, 1962 (Bischler 1962b).

*** Mnioloma fuscum (Lehm.) R.M.Schust., Fragm. Florist. Geobot. 40 (2): 848, 1995 (Schuster 1995a). Bas.: Jungermannia fusca Lehm., Linnaea 4: 360, 1829 (Lehmann 1829).

*** Mnioloma nephrostipum (Spruce) R.M.Schust., Fragm. Florist. Geobot. 40 (2): 847, 1995 (Schuster 1995a). Bas.: Kantius nephrostipus Spruce, Trans. & Proc. Bot. Soc. Edinburgh 15: 412, 1885 (Spruce 1885).

** Mnioloma novaezelandiae J.J.Engel, Cryptog. Bryol. 27 (1): 111, 2006 (Engel 2006a).

*** Mnioloma parallelogramum (Spruce) R.M.Schust., Fragm. Florist. Geobot. 40 (2): 847, 1995 (Schuster 1995a). Bas.: Kantius parallelogramus Spruce, Trans. & Proc. Bot. Soc. Edinburgh 15: 413, 1885 (Spruce 1885).

** Mnioloma retusum (Bischl.) R.M.Schust., Fragm. Florist. Geobot. 40 (2): 839, 1995 (Schuster 1995a). Bas.: Calypogeia retusa Bischl., Candollea 18: 33, 1962 (Bischler 1962b).

** Mnioloma stamatotonum M.A.M.Renner et E.A.Br., Fieldiana, Bot. (n.ser.) 47: 173, 2008 (Renner and Brown 2008).

*** Mnioloma venezuelanum (Fulford) R.M.Schust., Beih. Nova Hedwigia 118: 509, 2000 (Schuster 2000a). Bas.: Calypogeia venezuelana Fulford, Mem. New York Bot. Gard. 11 (3): 287, 1968 (Fulford 1968).

** **subg. Mnioloma**, Fragm. Florist. Geobot. 40 (2): 833, 1995 (Schuster 1995a).

*** Mnioloma rhynchophyllum Herzog, Ann. Bryol. 3: 120, 1930 (Herzog 1930a).

######## *** Endogemmataceae Konstant., Vilnet et A.V.Troitsky

by N.A. Konstantinova

Vilnet et al. (2011) described the monotypic family Endogemmataceae based on molecular evidence after a re-evaluation of Solenostomataceae.

*** **Endogemma Konstant., Vilnet et A.V.Troitsky**, Folia Cryptog. Estonica 48: 132, 2011 (Vilnet et al. 2011).

*** Endogemma caespiticia (Lindenb.) Konstant., Vilnet et A.V.Troitsky, Folia Cryptog. Estonica 48: 132, 2011 (Vilnet et al. 2011). Bas.: Jungermannia caespiticia Lindenb., Syn. hepat. eur: 67, 1829 (Lindenberg 1829).

######## *** Geocalycaceae H.Klinggr.

Placement of Geocalycaceae in Jungermanniinae follows Shaw et al. (2015).

*** **Geocalyx Nees**, Naturgesch. Eur. Leberm. 1: 97, 1833 (Nees 1833c).

*** Geocalyx caledonicus Steph., Bull. Herb. Boissier (sér. 2) 8 (3): 205 (265), 1908 (Stephani 1908h).

*** Geocalyx graveolens (Schrad.) Nees, Naturgesch. Eur. Leberm. 2: 397, 1836 (Nees 1836). Bas.: Jungermannia graveolens Schrad., Syst. Samml. Crypt. Gew. 2: 6, 1797 (Schrader 1797).

** Geocalyx lancistipulus (Steph.) S.Hatt., J. Jap. Bot. 28 (8): 234, 1953 (Hattori 1953a). Bas.: Lophocolea lancistipula Steph., Sp. Hepat. (Stephani) 6: 281, 1922 (Stephani 1922).

** Geocalyx orientalis Besch. et Spruce, Bull. Soc. Bot. France (Congr. Bot.) 36: clxxxix, 1889 [1890] (Bescherelle and Spruce 1889).

######## *** Gymnomitriaceae H.Klinggr.

by J. Váňa

The treatment of the family follows Váňa et al. (2010b) with some modifications. Nardioideae is included following Vilnet et al. (2010) and Váňa et al. (2014c). Some re-arrangements in Apomarsupella, Gymnomitrion and Marsupella were done by Vilnet et al (2010) and Shaw et al. (2015). Further notes on nomenclature and taxonomy can be found in Váňa et al. (2013d). Herzogobryum and Nothogymnomitrion were removed from the family by Váňa et al. (2013e). The placement of Acrolophozia, Nanomarsupella and Paramomitrion is provisional. Inclusion of Cryptocoleopsis follows Shaw et al (2015).

*** **Acrolophozia R.M.Schust.**, Rev. Bryol. Lichénol. 34 (1/2): 259, 1966 (Schuster 1966b).

*** Acrolophozia fuegiana R.M.Schust., Nova Hedwigia 15: 499, 1968 (Schuster 1968b).

*** Acrolophozia pectinata R.M.Schust., Rev. Bryol. Lichénol. 34 (1/2): 261, 1966 (Schuster 1966b).

*** Acrolophozia sulcata Hässel, J. Bryol. 11 (1): 108, 1980 (Hässel 1980).

** **Nanomarsupella R.M.Schust. ex A.Hagborg, L.Söderstr. et von Konrat**, Phytotaxa 112 (1): 16, 2013 (Hagborg et al. 2013). Based on: Marsupella subg. Nanomarsupella R.M.Schust., Phytologia 39 (4): 248, 1978 (Schuster 1978a).

*** Nanomarsupella xenophylla (R.M.Schust.) R.M.Schust. ex A.Hagborg, L.Söderstr. et von Konrat, Phytotaxa 112 (1): 16, 2013 (Hagborg et al. 2013). Bas.: Marsupella xenophylla R.M.Schust., Phytologia 39 (4): 248, 1978 (Schuster 1978a).

** **Paramomitrion R.M.Schust.**, J. Hattori Bot. Lab. 80: 134, 1996 (Schuster 1996a).

*** Paramomitrion paradoxum R.M.Schust., J. Hattori Bot. Lab. 80: 135, 1996 (Schuster 1996a).

######### *** Gymnomitrioideae T.Jensen

** **Cryptocoleopsis Amakawa**, J. Hattori Bot. Lab. 21: 274, 1959 (Amakawa 1959a).

*** Cryptocoleopsis imbricata Amakawa, J. Hattori Bot. Lab. 21: 274, 1959 (Amakawa 1959a).

*** **Gymnomitrion Corda**, Gen. hepat.: 651, 1829 (Corda 1829) nom. conserv.

*** Gymnomitrion adustum Nees, Naturgesch. Eur. Leberm. 1: 120, 1833 (Nees 1833c).

*** Gymnomitrion africanum (Steph.) Horik., Acta Phytotax. Geobot. 13: 212, 1943 (Horikawa 1943). Bas.: Acolea africana Steph., Sp. Hepat. (Stephani) 6: 77, 1917 (Stephani 1917a).

*** Gymnomitrion alpinum (Gottsche ex Husn.) Schiffn., Österr. Bot. Z. 53 (7): 280, 1903 (Schiffner 1903a). Bas.: Sarcocyphos alpinus Gottsche ex Husn., Hepaticol. gall. 1: 13, 1875 (Husnot 1875).

*** Gymnomitrion asperulatum R.M.Schust., Acta Acad. Ped. Agr., Sect. Biol. 24: 114, 2003 (Váňa 2003).

*** Gymnomitrion atrofilum Váňa, J. Hattori Bot. Lab. 41: 411, 1976 (Váňa 1976b).

*** Gymnomitrion bolivianum (Steph.) Váňa, Novon 20 (2): 225, 2010 (Váňa et al. 2010c). Bas.: Anastrophyllum bolivianum Steph., Biblioth. Bot. 87 (2): 186, 1916 (Stephani 1916a).

*** Gymnomitrion brevissimum (Dumort.) Warnst., Hedwigia 53 (3): 196, 1913 (Warnstorf 1913). Bas.: Acolea brevissima Dumort., Syll. Jungerm. Europ.: 76, 1831 (Dumortier 1831).

*** Gymnomitrion commutatum (Limpr.) Schiffn., Magyar Bot. Lapok 13: 304, 1914 [1915] (Schiffner 1914b). Bas.: Sarcocyphos commutatus Limpr., Jahresber. Schles. Ges. Vaterl. Cult. 57: 314, 1879 [1880] (Limpricht 1879).

*** Gymnomitrion concinnatum (Lightf.) Corda, Gen. hepat.: 651, 1829 (Corda 1829). Bas.: Jungermannia concinnata Lightf., Fl. Scot. 2: 786, 1777 (Lightfoot 1777), *nom. conserv*.

*** Gymnomitrion corallioides Nees, Naturgesch. Eur. Leberm. 1: 118, 1833 (Nees 1833c).

*** Gymnomitrion crenatilobum Grolle, Khumbu Himal 1 (4): 278, 1966 (Grolle 1966k).

*** Gymnomitrion crenulatum Gottsche ex Carrington, Trans. Bot. Soc. Edinburgh 7 (3): 444, 1863 (Carrington 1863).

*** Gymnomitrion crystallocaulon (Grolle) Váňa, Crand.-Stotl. et Stotler, Syst. Bot. 40 (1): 39, 2015 (Shaw et al. 2015). Bas.: Marsupella crystallocaulon Grolle, Khumbu Himal 1 (4): 281, 1966 (Grolle 1966k).

** Gymnomitrion incompletum (Gottsche) R.M.Schust. ex Váňa, J. Hattori Bot. Lab. 40: 186, 1976 (Váňa 1976a). Bas.: Jungermannia incompleta Gottsche, Linnaea 28 (5): 551, 1856 [1857] (Gottsche 1856).

*** Gymnomitrion laceratum (Steph.) Horik., Acta Phytotax. Geobot. 13: 212, 1943 (Horikawa 1943). Bas.: Acolea lacerata Steph., Sp. Hepat. (Stephani) 6: 78, 1917 (Stephani 1917a).

*** Gymnomitrion miniatum Lindenb. et Gottsche, Syn. Hepat. 4: 617, 1846 (Gottsche et al. 1846).

*** Gymnomitrion minutulum (Hässel) Váňa, Novon 20 (2): 225, 2010 (Váňa et al. 2010c). Bas.: Marsupella minutula Hässel, J. Bryol. 11 (1): 123, 1980 (Hässel 1980).

** Gymnomitrion moralesae Váňa, J. Hattori Bot. Lab. 48: 230, 1980 (Váňa 1980).

*** Gymnomitrion mucronulatum (N.Kitag.) N.Kitag., Acta Phytotax. Geobot. 19 (2/3): 53, 1962 (Kitagawa 1962a). Bas.: Gymnomitrion concinnatum var. mucronulatum N.Kitag., Acta Phytotax. Geobot. 18 (2/3): 38, 1959 (Kitagawa 1959).

** Gymnomitrion mucrophorum R.M.Schust., Bryologist 98 (2): 243, 1995 (Schuster 1995d).

*** Gymnomitrion nigrum (Grolle et Váňa) Váňa, Novon 20 (2): 225, 2010 (Váňa et al. 2010c). Bas.: Marsupella nigra Grolle et Váňa, J. Hattori Bot. Lab. 40: 186, 1976 (Váňa 1976a).

*** Gymnomitrion noguchianum S.Hatt., J. Jap. Bot. 27 (2): 55, 1952 (Hattori 1952b).

*** Gymnomitrion obtusilobum N.Kitag., Bull. Univ. Mus. Univ. Tokyo 8: 229, 1975 (Hattori 1975e).

*** Gymnomitrion obtusum Lindb., Morgonbladet (Helsinki) 1877 (30, 6 Feb): 2, 1877 (Lindberg 1877a).

*** Gymnomitrion pacificum Grolle, Trans. Brit. Bryol. Soc. 5 (1): 92, 1966 (Grolle 1966f).

*** Gymnomitrion revolutum (Nees) H.Philib., Rev. Bryol. 17 (3): 34, 1890 (Philibert 1890). Bas.: Sarcocyphos revolutus Nees, Naturgesch. Eur. Leberm. 2: 419, 1836 (Nees 1836).

** Gymnomitrion revolutum subsp. novoguineanensis (R.M.Schust.) Váňa, Crand.-Stotl. et Stotler, Syst. Bot. 40 (1): 39, 2015 (Shaw et al. 2015). Bas.: Apomarsupella revoluta subsp. novoguineanensis R.M.Schust., J. Hattori Bot. Lab. 80: 90, 1996 (Schuster 1996a).

*** Gymnomitrion rubidum (Mitt.) Váňa, Crand.-Stotl. et Stotler, Syst. Bot. 40 (1): 39, 2015 (Shaw et al. 2015). Bas.: Jungermannia rubida Mitt., J. Proc. Linn. Soc., Bot. 5 (18): 90, 1860 [1861] (Mitten 1860c).

*** Gymnomitrion setaceum Grolle et Váňa, J. Hattori Bot. Lab. 41: 411, 1976 (Váňa 1976b).

*** Gymnomitrion sinense Müll.Frib., Rev. Bryol. Lichénol. 20 (1/2): 176, 1951 (Müller 1951b).

** Gymnomitrion strictum (Berggr.) R.M.Schust., J. Hattori Bot. Lab. 26: 280, 1963 (Schuster 1963b). Bas.: Cesius strictus Berggr., New Zealand Hepat.: 2, 1898 (Berggren 1898).

** Gymnomitrion strictum var. inaequale R.M.Schust., J. Hattori Bot. Lab. 80: 118, 1996 (Schuster 1996a).

*** Gymnomitrion subintegrum (S.W.Arnell) Váňa, Novon 20 (2): 225, 2010 (Váňa et al. 2010c). Bas.: Marsupella subintegra S.W.Arnell, Ark. Bot. (n.ser.) 3 (16): 545, 1956 (Arnell 1956e).

*** Gymnomitrion truncatoapiculatum Herzog, Hedwigia 74 (2): 81, 1934 (Herzog 1934a).

*** Gymnomitrion verrucosum W.E.Nicholson, Symb. Sin. 5: 10, 1930 (Nicholson et al. 1930).

*** **Marsupella Dumort.**, Commentat. Bot. (Dumortier): 114, 1822 (Dumortier 1822).

*** Marsupella alata S.Hatt. et N.Kitag., Mem. Coll. Sci. Kyoto Imp. Univ., Ser. B, Biol. 27: 79, 1960 (Kitagawa 1960b).

*** Marsupella andreaeoides (Lindb.) Müll.Frib., Feddes Repert. Spec. Nov. Regni Veg. 54 (2/3): 214, 1951 (Müller 1951a). Bas.: Cesius andreaeoides Lindb., Meddel. Soc. Fauna Fl. Fenn. 14: 68, 1887 (Lindberg 1887a).

*** Marsupella apiculata Schiffn., Österr. Bot. Z. 53 (6): 249, 1903 (Schiffner 1903b).

*** Marsupella aquatica (Lindenb.) Schiffn., Sitzungsber. deutsch. naturwiss.-med. Vereins Böhmen “Lotos” Prag 44 (8): 267, 1896 [1897] (Schiffner 1896a). Bas.: Jungermannia emarginata var. aquatica Lindenb., Syn. hepat. eur: 75, 1829 (Lindenberg 1829).

*** Marsupella arctica (Berggr.) Bryhn et Kaal., Rep. Second Norweg. Arctic Exped. 11: 26, 1906 (Bryhn 1906). Bas.: Sarcocyphos emarginatus var. arcticus Berggr., Kongl. Svenska Vetensk.-Akad. Handl. (n.ser.) 13 (7): 96, 1875 (Berggren 1875).

*** Marsupella boeckii (Austin) Lindb. ex Kaal., Nyt Mag. Naturvidensk. 33 (4/5): 409, 1893 (Kaalaas 1893b). Bas.: Sarcocyphos boeckii Austin, Bull. Torrey Bot. Club 3 (3): 9, 1872 (Austin 1872).

*** Marsupella bolanderi (Austin) Underw., Zoe 1 (12): 365, 1891 (Underwood 1891). Bas.: Sarcocyphos bolanderi Austin, Bull. Torrey Bot. Club 3 (3): 9, 1872 (Austin 1872).

*** Marsupella condensata (Ångstr. ex C.Hartm.) Lindb. ex Kaal., Nyt Mag. Naturvidensk. 33 (4/5): 420, 1893 (Kaalaas 1893b). Bas.: Gymnomitrion condensatum Ångstr. ex C.Hartm., Handb. Skand. fl. (ed. 10): 128, 1871 (Hartman 1871).

*** Marsupella disticha Steph., Bull. Herb. Boissier (sér. 2) 1 (2): 164 (25), 1901 (Stephani 1901h).

*** Marsupella emarginata (Ehrh.) Dumort., Recueil Observ. Jungerm.: 24, 1835 (Dumortier 1835). Bas.: Jungermannia emarginata Ehrh., Hannover. Mag. 22 (8): 141, 1784 (Ehrhart 1784).

** Marsupella emarginata subsp. tubulosa (Steph.) N.Kitag., Mem. Coll. Sci. Kyoto Imp. Univ., Ser. B, Biol. 27: 76, 1960 (Kitagawa 1960b). Bas.: Marsupella tubulosa Steph., Bull. Herb. Boissier 5 (2): 99, 1897 (Stephani 1897b).

** Marsupella emarginata subsp. tubulosa var. apertifolia (Steph.) N.Kitag., J. Hattori Bot. Lab. 26: 89, 1963 (Kitagawa 1963b). Bas.: Marsupella apertifolia Steph., Bull. Herb. Boissier (sér. 2) 1 (2): 162 (23), 1901 (Stephani 1901h).

** Marsupella emarginata subsp. tubulosa var. patens N.Kitag., Mem. Coll. Sci. Kyoto Imp. Univ., Ser. B, Biol. 27: 77, 1960 (Kitagawa 1960b).

** Marsupella emarginata subsp. tubulosa var. tubulosa (Steph.) N.Kitag. ex Váňa et L.Söderstr., Phytotaxa 183 (4): 288, 2014 (Váňa et al. 2014c). Bas.: Marsupella tubulosa Steph., Bull. Herb. Boissier 5 (2): 99, 1897 (Stephani 1897b).

*** Marsupella funckii (F.Weber et D.Mohr) Dumort., Recueil Observ. Jungerm.: 24, 1835 (Dumortier 1835). Bas.: Jungermannia funckii F.Weber et D.Mohr, Bot. Taschenb. (Weber): 422, 1807 (Weber and Mohr 1807).

*** Marsupella microphylla R.M.Schust., Phytologia 39 (4): 249, 1978 (Schuster 1978a).

** Marsupella minutissima N.Kitag., Mem. Coll. Sci. Kyoto Imp. Univ., Ser. B, Biol. 27: 81, 1960 (Kitagawa 1960b).

*** Marsupella neesii Sande Lac. ex Schiffn., Consp. Hepat. Arch. Ind.: 70, 1898 (Schiffner 1898b). Based on: Sarcocyphos neesii Nees ex Sande Lac., Ann. Mus. Bot. Lugduno-Batavi 1: 288, 1864 (Sande Lacoste 1864), *nom. inval*.

*** Marsupella paroica R.M.Schust., Bryologist 60 (2): 145, 1957 (Schuster 1957b).

*** Marsupella profunda Lindb., Rev. Bryol. 14 (2): 19, 1887 (Lindberg 1887b).

*** Marsupella pseudofunckii S.Hatt., J. Hattori Bot. Lab. 4: 63, 1950 (Hattori 1950).

*** Marsupella sparsifolia (Lindb.) Dumort., Bull. Soc. Roy. Bot. Belgique 13: 128, 1874 (Dumortier 1874). Bas.: Sarcocyphos sparsifolius Lindb., Not. Sällsk. Fauna Fl. Fenn. Förh. 9: 280, 1868 (Lindberg 1868b).

** Marsupella sparsifolia subsp. childii R.M.Schust., Phytotaxa 183 (4): 288, 2014 (Váňa et al. 2014c). Based on: Marsupella sparsifolia subsp. childii R.M.Schust., J. Hattori Bot. Lab. 80: 61, 1996 (Schuster 1996a), *nom. inval*.

*** Marsupella sphacelata (Giesecke ex Lindenb.) Dumort., Recueil Observ. Jungerm.: 24, 1835 (Dumortier 1835). Bas.: Jungermannia sphacelata Giesecke ex Lindenb., Syn. hepat. eur: 76, 1829 (Lindenberg 1829).

*** Marsupella spiniloba R.M.Schust. et Damsh., Phytologia 63 (5): 326, 1987 (Schuster and Damsholt 1987).

*** Marsupella sprucei (Limpr.) Bernet, Cat. hép. Suisse: 33, 1888 (Bernet 1888). Bas.: Sarcocyphos sprucei Limpr., Flora 64 (5): 72, 1881 (Limpricht 1881).

** Marsupella stableri Spruce, Rev. Bryol. 8 (6): 96, 1881 (Spruce 1881a).

*** Marsupella stoloniformis N.Kitag., J. Hattori Bot. Lab. 30: 201, 1967 (Kitagawa 1967b).

** Marsupella stoloniformis subsp. vermiformis R.M.Schust., J. Hattori Bot. Lab. 80: 72, 1996 (Schuster 1996a).

*** Marsupella yakushimensis (Horik.) S.Hatt., Bull. Tokyo Sci. Mus. 11: 80, 1944 (Hattori 1944d). Bas.: Sphenolobus yakushimensis Horik., J. Sci. Hiroshima Univ., Ser. B, Div. 2, Bot. 2: 156, 1934 (Horikawa 1934).

*** **Poeltia Grolle**, Khumbu Himal 1 (4): 280, 1966 (Grolle 1966k).

*** Poeltia campylata Grolle, Khumbu Himal 1 (4): 280, 1966 (Grolle 1966k).

*** **Prasanthus Lindb.**, Kongl. Svenska Vetensk.-Akad. Handl. (n.ser.) 23 (5): 62, 1889 (Lindberg and Arnell 1889).

** Prasanthus jamalicus Potemkin, Ann. Bot. Fenn. 29 (4): 319, 1992 (Potemkin 1992).

*** Prasanthus suecicus (Gottsche) Lindb., Kongl. Svenska Vetensk.-Akad. Handl. (n.ser.) 23 (5): 62, 1889 (Lindberg and Arnell 1889). Bas.: Gymnomitrion suecicum Gottsche, Fl. Danica 16 (48): 20, 1871 (Lange 1871).

######### *** Nardioideae Váňa

*** **Nardia Gray**, Nat. Arr. Brit. Pl. 1: 694, 1821 (Gray 1821) nom. conserv.

*** Nardia arnelliana Grolle, Bot. Mag. (Tokyo) 77 (914): 297, 1964 (Grolle 1964b).

*** Nardia assamica (Mitt.) Amakawa, J. Hattori Bot. Lab. 26: 23, 1963 (Amakawa 1963). Bas.: Jungermannia assamica Mitt., J. Proc. Linn. Soc., Bot. 5 (18): 91, 1860 [1861] (Mitten 1860c).

*** Nardia breidleri (Limpr.) Lindb., Helsingf. Dagbl. 1880 (311, 15 Nov.): 2, 1880 (Lindberg 1880b). Bas.: Alicularia breidleri Limpr., Jahresber. Schles. Ges. Vaterl. Cult. 57: 311, 1879 [1880] (Limpricht 1879).

*** Nardia compressa (Hook.) Gray, Nat. Arr. Brit. Pl. 1: 694, 1821 (Gray 1821). Bas.: Jungermannia compressa Hook., Brit. Jungermann.: tab. 58, 1813 (Hooker 1813).

*** Nardia flagelliformis Inoue, J. Jap. Bot. 46 (1): 1, 1971 (Inoue 1971b).

*** Nardia geoscyphus (De Not.) Lindb., Not. Sällsk. Fauna Fl. Fenn. Förh. 13: 371, 1874 (Lindberg 1874a). Bas.: Alicularia geoscyphus De Not., Mem. Reale Accad. Sci. Torino (ser. 2) 18: 486, 1859 (De Notaris 1859).

* Nardia geoscyphus var. dioica Bakalin, Arctoa 18: 87, 2009 [2010] (Bakalin et al. 2009b).

* Nardia geoscyphus var. suberecta (Lindb. ex Kaal.) Váňa, Phytotaxa 76 (3): 37, 2013 (Váňa et al. 2013d). Bas.: Nardia haematosticta var. suberecta Kaal., Nyt Mag. Naturvidensk. 33 (4/5): 395, 1893 (Kaalaas 1893b).

*** Nardia grollei Váňa et D.G.Long, Nova Hedwigia 89 (3/4): 491, 2009 (Váňa and Long 2009).

*** Nardia insecta Lindb., Helsingf. Dagbl. 1878 (315, 18 Nov.): 2, 1878 (Lindberg 1878).

*** Nardia japonica Steph., Bull. Herb. Boissier 5 (2): 101, 1897 (Stephani 1897b).

* Nardia kamtschatica Arnell et C.E.O.Jensen, Hedwigia 67 (1/2): 111, 1927 (Arnell 1927). ^73^

* Nardia leptocaulis C.Gao, Fl. Hepat. Chin. Boreali-Orient.: 205, 1981 (Gao and Chang 1981).

*** Nardia lescurii (Austin) Underw., Bull. Illinois State Lab. Nat. Hist. 2 (1): 115, 1884 (Underwood 1884). Bas.: Alicularia lescurii Austin, Hepat. bor.-amer.: 4, 1873 (Austin 1873).

** Nardia minutifolia Furuki, Bryol. Res. 9 (3): 73, 2006 (Furuki 2006b).

** Nardia nuda (Lindenb. et Gottsche) Váňa, Folia Geobot. Phytotax. 8 (2): 193, 1973 (Váňa 1973b). Bas.: Jungermannia nuda Lindenb. et Gottsche, Syn. Hepat. 5: 668, 1847 (Gottsche et al. 1847).

*** Nardia poeltii Váňa, J. Hattori Bot. Lab. 36: 73, 1972 [1973] (Váňa 1972b).

*** Nardia scalaris Gray, Nat. Arr. Brit. Pl. 1: 694, 1821 (Gray 1821).

* Nardia scalaris var. botryoidea (R.M.Schust.) Váňa, Phytotaxa 76 (3): 38, 2013 (Váňa et al. 2013d). Bas.: Nardia scalaris subsp. botryoidea R.M.Schust., Hepat. Anthocerotae N. Amer. 2: 862, 1969 (Schuster 1969b).

* Nardia scalaris var. harae (Amakawa) Váňa, Phytotaxa 76 (3): 38, 2013 (Váňa et al. 2013d). Bas.: Nardia harae Amakawa, J. Jap. Bot. 32 (2): 38, 1957 (Amakawa 1957c).

*** Nardia subclavata (Steph.) Amakawa, J. Jap. Bot. 32 (2): 40, 1957 (Amakawa 1957c). Bas.: Jungermannia subclavata Steph., Sp. Hepat. (Stephani) 6: 93, 1917 (Stephani 1917a).

*** Nardia succulenta (A.Rich.) Spruce, Trans. & Proc. Bot. Soc. Edinburgh 15: 519, 1885 (Spruce 1885). Bas.: Jungermannia succulenta A.Rich., Nov. Stirp. Pug. 4: 43, 1832 (Lehmann 1832).

*** Nardia unispiralis Amakawa, J. Jap. Bot. 32 (6): 167, 1957 (Amakawa 1957a).

######## ** Gyrothyraceae R.M.Schust.

Placement of Gyrothyraceae in Jungermanniinae follows Shaw et al. (2015).

** **Gyrothyra M.Howe**, Bull. Torrey Bot. Club 24 (4): 201, 1897 (Howe 1897a).

*** Gyrothyra underwoodiana M.Howe, Bull. Torrey Bot. Club 24 (4): 202, 1897 (Howe 1897a).

######## *** Harpanthaceae Arnell

Davis (2004) and Hentschel et al. (2006) resolved Harpanthus as an independent lineage which is recognized here as a monogeneric family as originally construed by Arnell (1928).

*** **Harpanthus Nees**, Naturgesch. Eur. Leberm. 2: 351, 1836 (Nees 1836).

*** Harpanthus drummondii (Taylor) Grolle, Österr. Bot. Z. 112 (3): 274, 1965 (Grolle 1965g). Bas.: Chiloscyphus drummondii Taylor, London J. Bot. 5: 283, 1846 (Taylor 1846a).

*** Harpanthus flotovianus (Nees) Nees, Naturgesch. Eur. Leberm. 2: 353, 1836 (Nees 1836). Bas.: Jungermannia flotoviana Nees, Flora 16 (26): 408, 1833 (Nees 1833b).

*** Harpanthus scutatus (F.Weber et D.Mohr) Spruce, Trans. Bot. Soc. Edinburgh 3 (1/4): 209, 1850 (Spruce 1850). Bas.: Jungermannia scutata F.Weber et D.Mohr, Bot. Taschenb. (Weber): 408, 1807 (Weber and Mohr 1807).

######## ** Hygrobiellaceae Konstant. et Vilnet

by N. Konstantinova

The family Hygrobiellaceae was validated by Konstantinova et al. (2014a).

*** **Hygrobiella Spruce**, Cephalozia: 73, 1882 (Spruce 1882).

** Hygrobiella intermedia Bakalin et Vilnet, Pl. Syst. Evol. 300 (10): 2286, 2014 (Bakalin and Vilnet 2014).

*** Hygrobiella laxifolia (Hook.) Spruce, Cephalozia: 74, 1882 (Spruce 1882). Bas.: Jungermannia laxifolia Hook., Brit. Jungermann.: tab. 59, 1813 (Hooker 1813).

** Hygrobiella squamosa Bakalin et Vilnet, Pl. Syst. Evol. 300 (10): 2286, 2014 (Bakalin and Vilnet 2014).

######## *** Jackiellaceae R.M.Schust.

by J. Váňa

The monogeneric status of Jackiella is supported by a molecular study based on three loci by Hendry et al. (2007).

*** **Jackiella Schiffn.**, Hep. Fl. Buitenzorg: 211, 1900 (Schiffner 1900a).

** Jackiella angustifolia Herzog, Trans. Brit. Bryol. Soc. 1 (4): 296, 1950 (Herzog 1950a).

** Jackiella ceylanica Schiffn. ex Steph., Bull. Herb. Boissier (sér. 2) 8 (3): 212 (272), 1908 (Stephani 1908h).

*** Jackiella curvata E.A.Hodgs. et Allison, Trans. Roy. Soc. New Zealand 85 (4): 571, 1958 (Hodgson 1958).

*** Jackiella javanica Schiffn., Hep. Fl. Buitenzorg: 212, 1900 (Schiffner 1900a).

** Jackiella javanica var. cavifolia Schiffn., Hep. Fl. Buitenzorg: 213, 1900 (Schiffner 1900a).

** Jackiella javanica var. cordifolia Schiffn., Hep. Fl. Buitenzorg: 213, 1900 (Schiffner 1900a).

** Jackiella renifolia Schiffn., Denkschr. Kaiserl. Akad. Wiss., Math.-Naturwiss. Kl. 70: 218, 1900 [1901] (Schiffner 1900c).

** Jackiella sinensis (W.E.Nicholson) Grolle, Österr. Bot. Z. 111 (2/3): 186, 1964 (Grolle 1964f). Bas.: Aplozia sinensis W.E.Nicholson, Symb. Sin. 5: 12, 1930 (Nicholson et al. 1930).

** Jackiella singapurensis Schiffn., Denkschr. Kaiserl. Akad. Wiss., Math.-Naturwiss. Kl. 70: 218, 1900 [1901] (Schiffner 1900c).

** Jackiella singapurensis var. philippinensis N.Kitag., Misc. Bryol. Lichenol. 9 (1): 9, 1981 (Kitagawa 1981b).

**Excluded from the genus**

* Jackiella unica Steph., Sp. Hepat. (Stephani) 6: 318, 1922 (Stephani 1922). ^74^

######## *** Jungermanniaceae Rchb.

by J. Váňa

The treatment of Jungermanniaceae follows Shaw et al. (2015). Many old names in Jungermannia are still neither synonymized nor transferred and we do not know their value. Some of them may prove to be older names of currently accepted taxa. We list those doubtful names in a separate section below.

######### ** Delavayelloideae Grolle

** **Delavayella Steph.**, Hedwigia 33 (1): 4, 1894 (Stephani 1894a).

** Delavayella serrata Steph., Mém. Soc. Nat. Sci. Nat. Math. Cherbourg 29: 211, 1894 (Stephani 1894b).

** Delavayella serrata var. purpurea P.C.Chen, Feddes Repert. Spec. Nov. Regni Veg. 58: 38, 1955 (Chen 1955).

** **Liochlaena Nees**, Syn. Hepat. 2: 150, 1845 (Gottsche et al. 1845a).

*** Liochlaena lanceolata Nees, Syn. Hepat. 2: 150, 1845 (Gottsche et al. 1845a).

*** Liochlaena subulata (A.Evans) Schljakov, Pečen. Mchi Sev. SSSR 4: 71, 1981 (Shliakov 1981). Bas.: Jungermannia subulata A.Evans, Trans. Connecticut Acad. Arts 8 (15): 258, 1891 (Evans 1891).

######### ** Jungermannioideae Dumort.

** **Eremonotus Lindb. et Kaal. ex Pearson**, Hepat. Br. Isl. 1 (6-15): 200, 1900 (Pearson 1900).

*** Eremonotus myriocarpus (Carrington) Lindb. et Kaal. ex Pearson, Hepat. Br. Isl. 1 (6-15): 201, 1900 (Pearson 1900). Bas.: Jungermannia myriocarpa Carrington, Hepat. Brit. Exsicc. Fasc. II: no. 96, 1879 (Carrington and Pearson 1879).

*** **Jungermannia L.**, Sp. Pl. 1: 1131, 1753 (Linnaeus 1753). ^75^

*** Jungermannia atrovirens Dumort., Syll. Jungerm. Europ.: 51, 1831 (Dumortier 1831).

*** Jungermannia borealis Damsh. et Váňa, Lindbergia 4 (1/2): 5, 1977 (Damsholt and Váňa 1977).

* Jungermannia erectii Ajit P.Singh et V.Nath, Hepat. Khasi Jaintia Hills: E. Himal.: 117, 2007 (Singh and Nath 2007b). ^76^

*** Jungermannia exsertifolia Steph., Sp. Hepat. (Stephani) 6: 86, 1917 (Stephani 1917a).

** Jungermannia exsertifolia subsp. cordifolia (Dumort.) Váňa, Folia Geobot. Phytotax. 8 (3): 268, 1973 (Váňa 1973c). Bas.: Aplozia cordifolia Dumort., Bull. Soc. Roy. Bot. Belgique 13: 59, 1874 (Dumortier 1874).

*** Jungermannia gollanii Steph., Sp. Hepat. (Stephani) 6: 86, 1917 (Stephani 1917a).

** Jungermannia konstantinovae Bakalin et Vilnet, Arctoa 18: 161, 2009 [2010] (Bakalin and Vilnet 2009).

*** Jungermannia ovatotrigona (Steph.) Grolle, Feddes Repert. 82 (1): 90, 1971 (Grolle 1971b). Bas.: Jamesoniella ovatotrigona Steph., Biblioth. Bot. 87 (2): 184, 1916 (Stephani 1916a).

*** Jungermannia polaris Lindb., Öfvers. Kongl. Vetensk.-Akad. Förh. 23 (10): 560, 1866 [1867] (Lindberg 1866).

*** Jungermannia pumila With., Arr. Brit. Pl., ed. 3, 3: 883, 1796 (Withering 1796).

**Taxa doubtfully belonging to the genus ^77^**

* Jungermannia amentacea Bertol., Mem. Reale Accad. Sci. Ist. Bologna (ser. 2) 1: 19, 1862 (Bertoloni 1862).

* Jungermannia brasiliensis Raddi, Critt. Brasil.: 15, 1822 (Raddi 1822). ^78^

* Jungermannia chinensis Osbeck, Dagb. Ostind. Resa: 221, 1757 (Osbeck 1757). ^79^

* Jungermannia cordata Vill., Hist. Pl. Dauphiné (Villars) 3: 923, 1789 (Villars 1789).

* Jungermannia crenulata Schmidel, Jungerm. Char.: 20, 1760 (Schmidel 1760).

* Jungermannia creutzeri Kremer, Monogr. hépat. Moselle: 26, 1837 (Krémer 1837).

* Jungermannia digitata C.F.W.Meissn. ex Spreng. Syst. Veg. (ed. 16) [Sprengel] 4 (2): 326, 1827 (Sprengel 1827b).

* Jungermannia dubioides H.A.Mill., Phytologia 47 (4): 322, 1981 (Miller 1981). *Nom. nov. pro Jungermannia dubia* Nees, Prodr. Fl. Norfolk.: 5, 1833 (Endlicher 1833), *nom. illeg*.

* Jungermannia fernandeziana Mitt., Rep. Challenger, Bot. 1 (3, 1): 85, 1884 (Mitten 1884b).

* Jungermannia hexagona Schwägr., Hist. Musc. Hepat. Prodr.: 18, 1814 (Schwägrichen 1814).

* Jungermannia holandriana Kremer, Monogr. hépat. Moselle: 25, 1837 (Krémer 1837).

* Jungermannia incerta Gottsche, Abh. Naturwiss. Vereins Bremen 7: 344, 1882 (Gottsche 1882). ^80^

* Jungermannia lateriflora Hampe ex Gottsche, Mexik. Leverm.: 82, 1863 (Gottsche 1863).

* Jungermannia lescuriana Austin, Rep. (Annual) Regents Univ. State New York State Cab. Nat. Hist. 19: 67, 1866 (Peck 1866). ^81^

* Jungermannia longiretis Besch. et Spruce, Bull. Soc. Bot. France (Congr. Bot.) 36: clxxxv, 1889 [1890] (Bescherelle and Spruce 1889). ^82^

* Jungermannia mastigophora Spreng. Neue Entdeck. Pflanzenk. 2: 99, 1821 (Sprengel 1821). ^83^

* Jungermannia michelii Mérat, Nouv. fl. env. Paris (ed. 2) 1: 219, 1821 (Mérat 1821). ^84^

* Jungermannia minima Scop., Fl. Carniol. (ed. 2) 2: 350, 1772 (Scopoli 1772).

* Jungermannia odorata With., Bot. arr. veg. Gr. Brit. 2: 693, 1776 (Withering 1776).

* Jungermannia peltata Schmidel, Jungerm. Char.: 14, 1760 (Schmidel 1760).

* Jungermannia quadridigitata Griff., Not. pl. asiat. 2: 314, 1849 (Griffith 1849). ^85^

* Jungermannia sauteri De Not. ex Rabenh., Hedwigia 1 (20): 121, 1857 (Rabenhorst 1857).

* Jungermannia secunda Hampe ex Gottsche, Mexik. Leverm.: 82, 1863 (Gottsche 1863).

* Jungermannia stereocaulis Bory, Voy. Uranie, Bot. 4: 130, 1827 (Gaudichaud 1827). ^86^

* Jungermannia submersa Kremer, Monogr. hépat. Moselle: 36, 1837 (Krémer 1837).

* Jungermannia sullivantiana Austin, Rep. (Annual) Regents Univ. State New York State Cab. Nat. Hist. 19: 66, 1866 (Peck 1866). ^87^

* Jungermannia supina Hoffm., Deutschl. Fl., Theil 2 (Hoffm.): 86, 1795 [1796] (Hoffmann 1795).

* Jungermannia tenuis Ehrh., Beitr. Naturk. (Ehrhart) 4: 45, 1789 (Ehrhart 1789). ^88^

* Jungermannia uncifolia Steph., Hedwigia 34 (2): 51, 1895 (Stephani 1895c).

* Jungermannia vernicosa Cass. ex Mérat, Nouv. fl. env. Paris (ed. 2) 1: 221, 1821 (Mérat 1821).

######### ** Mesoptychioideae R.M.Schust.

*** **Mesoptychia (Lindb.) A.Evans**, Ottawa Naturalist 17: 15, 1903 (Evans 1903a). Bas.: Jungermannia sect. Mesoptychia Lindb., Kongl. Svenska Vetensk.-Akad. Handl. (n.ser.) 23 (5): 39, 1889 (Lindberg and Arnell 1889).

*** Mesoptychia badensis (Gottsche ex Rabenh.) L.Söderstr. et Váňa, Phytotaxa 65: 52, 2012 (Váňa et al. 2012b). Bas.: Jungermannia badensis Gottsche ex Rabenh., Hepat. Eur., Leberm. 9-10: no. 95, 1859 (Rabenhorst 1859).

*** Mesoptychia bantriensis (Hook.) L.Söderstr. et Váňa, Phytotaxa 65: 52, 2012 (Váňa et al. 2012b). Bas.: Jungermannia bantriensis Hook., Brit. Jungermann.: tab. 41, 1813 (Hooker 1813).

** Mesoptychia bantriensis subsp. wallfischii (Ştefănuţ) L.Söderstr. et Váňa, Phytotaxa 65: 52, 2012 (Váňa et al. 2012b). Bas.: Leiocolea bantriensis subsp. wallfischii Ştefănuţ, Hornwort Liverwort Romania: 21, 2008 (Ştefănuţ 2008).

* Mesoptychia chichibuensis (Inoue) L.Söderstr. et Váňa, Phytotaxa 65: 53, 2012 (Váňa et al. 2012b). Bas.: Lophozia chichibuensis Inoue, J. Jap. Bot. 36 (2): 41, 1961 (Inoue 1961b).

* Mesoptychia collaris (Nees) L.Söderstr. et Váňa, Phytotaxa 65: 53, 2012 (Váňa et al. 2012b). Bas.: Jungermannia collaris Nees, Fl. crypt. erlang.: xv, 1817 (Martius 1817).

* Mesoptychia fitzgeraldiae (Paton et A.R.Perry) L.Söderstr. et Váňa, Phytotaxa 65: 53, 2012 (Váňa et al. 2012b). Bas.: Leiocolea fitzgeraldiae Paton et A.R.Perry, J. Bryol. 18 (3): 470, 1995 (Paton and Perry 1995).

*** Mesoptychia gillmanii (Austin) L.Söderstr. et Váňa, Phytotaxa 65: 53, 2012 (Váňa et al. 2012b). Bas.: Jungermannia gillmanii Austin, Bull. Torrey Bot. Club 3 (3): 12, 1872 (Austin 1872).

*** Mesoptychia heterocolpos (Thed. ex Hartm.) L.Söderstr. et Váňa, Phytotaxa 65: 53, 2012 (Váňa et al. 2012b). Bas.: Jungermannia heterocolpos Thed. ex Hartm., Handb. Skand. fl. (ed. 3): 328, 1838 (Hartman 1838).

** Mesoptychia heterocolpos var. arctica (S.W.Arnell) L.Söderstr. et Váňa, Phytotaxa 65: 53, 2012 (Váňa et al. 2012b). Bas.: Leiocolea arctica S.W.Arnell, Svensk Bot. Tidskr. 44 (2): 374, 1950 (Arnell 1950).

** Mesoptychia heterocolpos var. harpanthoides (Bryhn et Kaal.) L.Söderstr. et Váňa, Phytotaxa 65: 53, 2012 (Váňa et al. 2012b). Bas.: Lophozia harpanthoides Bryhn et Kaal., Rep. Second Norweg. Arctic Exped. 11: 31, 1906 (Bryhn 1906).

*** Mesoptychia igiana (S.Hatt.) L.Söderstr. et Váňa, Phytotaxa 65: 54, 2012 (Váňa et al. 2012b). Bas.: Lophozia igiana S.Hatt., J. Jap. Bot. 31 (7): 201, 1956 (Hattori 1956a).

* Mesoptychia mamatkulovii (Duda) L.Söderstr. et Váňa, Phytotaxa 65: 54, 2012 (Váňa et al. 2012b). Bas.: Lophozia mamatkulovii Duda, Trans. Brit. Bryol. Soc. 6 (1): 82, 1970 (Duda 1970).

*** Mesoptychia mayebarae (S.Hatt.) L.Söderstr. et Váňa, Phytotaxa 65: 54, 2012 (Váňa et al. 2012b). Bas.: Cephalozia mayebarae S.Hatt., J. Hattori Bot. Lab. 3: 37, 1948 [1950] (Hattori 1948a).

*** Mesoptychia morrisoncola (Horik.) L.Söderstr. et Váňa, Phytotaxa 65: 54, 2012 (Váňa et al. 2012b). Bas.: Lophozia morrisoncola Horik., J. Sci. Hiroshima Univ., Ser. B, Div. 2, Bot. 2: 150, 1934 (Horikawa 1934).

* Mesoptychia polymorpha Stotler, Crand.-Stotl. et Bakalin, Polish Bot. J. 58 (1): 82, 2013 (Crandall-Stotler et al. 2013).

*** Mesoptychia rutheana (Limpr.) L.Söderstr. et Váňa, Phytotaxa 65: 54, 2012 (Váňa et al. 2012b). Bas.: Jungermannia rutheana Limpr., Jahresber. Schles. Ges. Vaterl. Cult. 61: 207, 1884 (Limpricht 1884).

** Mesoptychia rutheana var. laxa (Schiffn. ex Burrell) L.Söderstr. et Váňa, Phytotaxa 65: 54, 2012 (Váňa et al. 2012b). Bas.: Lophozia schultzii var. laxa Schiffn. ex Burrell, J. Bot. 49: 217, 1911 (Burrell 1911).

*** Mesoptychia sahlbergii (Lindb. et Arnell) A.Evans, Ottawa Naturalist 17: 15, 1903 (Evans 1903a). Bas.: Jungermannia sahlbergii Lindb. et Arnell, Kongl. Svenska Vetensk.-Akad. Handl. (n.ser.) 23 (5): 40, 1889 (Lindberg and Arnell 1889).

*** Mesoptychia subcrispa (Herzog) L.Söderstr. et Váňa, Phytotaxa 65: 54, 2012 (Váňa et al. 2012b). Bas.: Lophozia subcrispa Herzog, Ann. Naturhist. Mus. Wien 53 (1): 362, 1942 [1943] (Herzog 1942b).

*** Mesoptychia turbinata (Raddi) L.Söderstr. et Váňa, Phytotaxa 65: 55, 2012 (Váňa et al. 2012b). Bas.: Jungermannia turbinata Raddi, Jungermanniogr. Etrusca: 18, 1818 (Raddi 1818a).

* Mesoptychia ussuriensis (Bakalin) L.Söderstr. et Váňa, Phytotaxa 65: 55, 2012 (Váňa et al. 2012b). Bas.: Leiocolea ussuriensis Bakalin, Arctoa 17: 103, 2008 [2009] (Bakalin 2008c).

** **Rivulariella D.H.Wagner**, Phytoneuron 2013 (10): 2, 2013 (Wagner 2013).

** Rivulariella gemmipara (A.Evans) D.H.Wagner, Phytoneuron 2013 (10): 2, 2013 (Wagner 2013). Bas.: Chiloscyphus gemmiparus A.Evans, Bryologist 41 (3): 50, 1938 (Evans 1938b).

######## ** Notoscyphaceae Crand.-Stotl., Váňa et Stotler

Shaw et al. (2015) circumscribed Notoscyphaceae as monogeneric based on molecular and morphological evidence.

*** **Notoscyphus Mitt.**, Fl. vit.: 407, 1871 [1873] (Mitten 1871).

*** Notoscyphus lutescens (Lehm. et Lindenb.) Mitt., Fl. vit.: 407, 1871 [1873] (Mitten 1871). Bas.: Jungermannia lutescens Lehm. et Lindenb., Nov. Stirp. Pug. 4: 16, 1832 (Lehmann 1832).

######## ** Saccogynaceae Heeg

Shaw et al. (2015) recognized Saccogynaceae as being monogeneric.

*** **Saccogyna Dumort.**, Commentat. Bot. (Dumortier): 113, 1822 (Dumortier 1822) nom. conserv.

* Saccogyna ligulata Steph., Bull. Herb. Boissier (sér. 2) 8 (3): 207 (267), 1908 (Stephani 1908h).

** Saccogyna subacuta Steph., Sp. Hepat. (Stephani) 6: 316, 1922 (Stephani 1922).

*** Saccogyna viticulosa (L.) Dumort., Syll. Jungerm. Europ.: 74, 1831 (Dumortier 1831). Bas.: Jungermannia viticulosa L., Sp. Pl. 1: 1131, 1753 (Linnaeus 1753).

**Excluded from the genus**

* Saccogyna tridens Steph., Sp. Hepat. (Stephani) 6: 317, 1922 (Stephani 1922). ^89^

######## *** Solenostomataceae Stotler et Crand.-Stotl.

by J. Váňa

Solenostomataceae was first defined by Crandall-Stotler et al. (2009) and refined to the current circumscription in Shaw et al (2015). The placement of Aponardia is provisional following Váňa et al. (2012c) and the placement of Arctoscyphus, Cryptocolea and Diplocolea in the family is also provisional. Taxonomic and nomenclatural notes can also be found in Váňa and Long (2009) and Váňa et al. (2012d, 2013c).

** **Aponardia (R.M.Schust.) Váňa**, Phytotaxa 65: 46, 2012 (Váňa et al. 2012c). Bas.: Nardia subg. Aponardia R.M.Schust., Beih. Nova Hedwigia 119: 360, 2002 (Schuster 2002b).

*** Aponardia huerlimannii (Váňa et Grolle) Váňa, Phytotaxa 65: 46, 2012 (Váňa et al. 2012c). Bas.: Nardia huerlimannii Váňa et Grolle, Österr. Bot. Z. 118 (3): 233, 1970 (Váňa 1970c).

** **Arctoscyphus Hässel**, Lindbergia 16 (4): 133, 1990 [1992] (Hässel 1990a).

** Arctoscyphus fuegiensis (C.Massal.) Hässel, Cryptog. Bryol. Lichénol. 17 (3): 164, 1996 (Hässel 1996). Bas.: Leioscyphus repens var. fuegiensis C.Massal., Nuovo Giorn. Bot. Ital. 17 (3): 212, 1885 (Massalongo 1885).

** Arctoscyphus ronsmithii Hässel, Lindbergia 16 (4): 133, 1990 [1992] (Hässel 1990a).

** **Cryptocolea R.M.Schust.**, Amer. Midl. Naturalist 49 (2): 414, 1953 (Schuster 1953).

*** Cryptocolea imbricata R.M.Schust., Amer. Midl. Naturalist 49 (2): 417, 1953 (Schuster 1953).

** **Diplocolea Amakawa**, J. Jap. Bot. 37 (9): 274, 1962 (Amakawa 1962).

*** Diplocolea sikkimensis Amakawa, J. Jap. Bot. 37 (9): 274, 1962 (Amakawa 1962).

*** **Solenostoma Mitt.**, J. Proc. Linn. Soc., Bot. 8 (29): 51, 1864 [1865] (Mitten 1864c) nom. conserv. ^90^

** **subg. Eucalyx (Lindb.) Váňa, Crand.-Stotl. et Stotler**, Syst. Bot. 40 (1): 38, 2015 (Shaw et al. 2015). Bas.: Nardia sect. Eucalyx Lindb., Acta Soc. Sci. Fenn. 10: 525, 1875 (Lindberg 1875).

*** Solenostoma bilobum (S.Hatt. ex Amakawa) Potemkin et Nyushko, Liverworts and Hornworts of Russia 1: 286, 2009 (Potemkin and Sofronova 2009). Bas.: Plectocolea biloba S.Hatt. ex Amakawa, J. Jap. Bot. 32 (7): 216, 1957 (Amakawa 1957d).

*** Solenostoma emarginatum (Amakawa) Váňa, Hentschel et Heinrichs, Cryptog. Bryol. 31 (2): 136, 2010 (Váňa et al. 2010a). Bas.: Plectocolea emarginata Amakawa, J. Jap. Bot. 33 (11): 340, 1958 (Amakawa 1958b).

*** Solenostoma flagellatum (S.Hatt.) Váňa et D.G.Long, Nova Hedwigia 89 (3/4): 501, 2009 (Váňa and Long 2009). Bas.: Plectocolea flagellata S.Hatt., J. Hattori Bot. Lab. 3: 13, 1948 [1950] (Hattori 1948b).

*** Solenostoma hokkaidense (Váňa) Váňa, Hentschel et Heinrichs, Cryptog. Bryol. 31 (2): 137, 2010 (Váňa et al. 2010a). Bas.: Jungermannia hokkaidensis Váňa, J. Hattori Bot. Lab. 35: 314, 1972 (Váňa 1972a).

*** Solenostoma obovatum (Nees) C.Massal., Epat. erb. critt. ital.: 17, 1903 (Massalongo 1903). Bas.: Jungermannia obovata Nees, Naturgesch. Eur. Leberm. 1: 332, 1833 (Nees 1833c).

*** Solenostoma obscurum (A.Evans) R.M.Schust., Hepat. Anthocerotae N. Amer. 2: 1013, 1969 (Schuster 1969b). Bas.: Nardia obscura A.Evans, Rhodora 21 (249): 159, 1919 (Evans 1919a).

*** Solenostoma schusteranum (J.D.Godfrey et G.Godfrey) Váňa, Hentschel et Heinrichs, Cryptog. Bryol. 31 (2): 137, 2010 (Váňa et al. 2010a). Bas.: Jungermannia schusterana J.D.Godfrey et G.Godfrey, J. Hattori Bot. Lab. 46: 109, 1979 (Godfrey and Godfrey 1979).

*** Solenostoma subtilissimum (Schiffn.) R.M.Schust., Hepat. Anthocerotae N. Amer. 2: 1027, 1969 (Schuster 1969b). Bas.: Nardia subtilissima Schiffn., Ann. K. K. Naturhist. Hofmus. 23: 136, 1909 (Schiffner 1909b).

** **subg. Metasolenostoma Váňa, Crand.-Stotl. et Stotler**, Syst. Bot. 40 (1): 38, 2015 (Shaw et al. 2015).

*** Solenostoma fusiforme (Steph.) R.M.Schust., Hepat. Anthocerotae N. Amer. 2: 944, 1969 (Schuster 1969b). Bas.: Nardia fusiformis Steph., Bull. Herb. Boissier 5 (2): 99, 1897 (Stephani 1897b).

*** Solenostoma gracillimum (Sm.) R.M.Schust., Hepat. Anthocerotae N. Amer. 2: 972, 1969 (Schuster 1969b). Bas.: Jungermannia gracillima Sm., Engl. Bot. 32: tab. 2238, 1811 (Smith and Sowerby 1811).

*** Solenostoma handelii (Schiffn.) Müll.Frib., Beitr. Kryptogamenfl. Schweiz 10 (2): 38, 1947 (Müller 1947). Bas.: Nardia handelii Schiffn., Ann. K. K. Naturhist. Hofmus. 23: 135, 1909 (Schiffner 1909b).

** Solenostoma lignicola (Schiffn.) Váňa, Hentschel et Heinrichs, Cryptog. Bryol. 31 (2): 137, 2010 (Váňa et al. 2010a). Bas.: Nardia lignicola Schiffn., Ann. K. K. Naturhist. Hofmus. 23: 137, 1909 (Schiffner 1909b).

*** Solenostoma limbatifolium (Amakawa) Váňa et D.G.Long, Nova Hedwigia 89 (3/4): 504, 2009 (Váňa and Long 2009). Bas.: Jungermannia limbatifolia Amakawa, J. Hattori Bot. Lab. 31: 112, 1968 (Amakawa 1968b).

** Solenostoma philippinense Váňa, Phytotaxa 152 (1): 43, 2013 (Váňa et al. 2013c). Based on: Solenostoma gracillimum subsp. camiguinense Bakalin, Polish Bot. J. 58 (1): 134, 2013 (Bakalin 2013).

*** Solenostoma rubrum (Gottsche) R.M.Schust., Hepat. Anthocerotae N. Amer. 2: 975, 1969 (Schuster 1969b). Bas.: Jungermannia rubra Gottsche, Bot. Gaz. 13 (5): 113, 1888 (Underwood 1888).

*** Solenostoma suborbiculatum (Amakawa) Váňa et D.G.Long, Nova Hedwigia 89 (3/4): 508, 2009 (Váňa and Long 2009). Bas.: Jungermannia suborbiculata Amakawa, J. Hattori Bot. Lab. 31: 112, 1968 (Amakawa 1968b).

** **subg. Plectocolea Mitt.**, J. Linn. Soc., Bot. 8 (31): 156, 1864 (Mitten 1864a).

*** Solenostoma balfourii (Váňa) Váňa, Hentschel et Heinrichs, Cryptog. Bryol. 31 (2): 136, 2010 (Váňa et al. 2010a). Bas.: Jungermannia balfourii Váňa, Folia Geobot. Phytotax. 9 (3): 279, 1974 (Váňa 1974a).

*** Solenostoma borneense (Amakawa) Váňa, Hentschel et Heinrichs, Cryptog. Bryol. 31 (2): 136, 2010 (Váňa et al. 2010a). Bas.: Jungermannia borneensis Amakawa, J. Hattori Bot. Lab. 33: 160, 1970 (Amakawa 1970).

*** Solenostoma callithrix (Lindenb. et Gottsche) Steph., Bull. Herb. Boissier (sér. 2) 1 (5): 486 (48), 1901 (Stephani 1901a). Bas.: Jungermannia callithrix Lindenb. et Gottsche, Syn. Hepat. 5: 673, 1847 (Gottsche et al. 1847).

*** Solenostoma champawatense (S.N.Srivast. et Amakawa) Váňa et D.G.Long, Nova Hedwigia 89 (3/4): 496, 2009 (Váňa and Long 2009). Bas.: Jungermannia champawatensis S.N.Srivast. et Amakawa, Proc. Natl. Acad. Sci. India, B 61 (2): 205, 1991 (Srivastava and Amakawa 1991).

*** Solenostoma comatum (Nees) C.Gao, Fl. Hepat. Chin. Boreali-Orient.: 73, 1981 (Gao and Chang 1981). Bas.: Jungermannia comata Nees, Enum. Pl. Crypt. Javae: 78, 1830 (Nees 1830).

*** Solenostoma comatum var. novae-guineae (Váňa) Váňa, Hentschel et Heinrichs, Cryptog. Bryol. 31 (2): 136, 2010 (Váňa et al. 2010a). Bas.: Jungermannia comata var. novae-guineae Váňa, J. Hattori Bot. Lab. 37: 187, 1973 (Váňa 1973a).

*** Solenostoma crenuliforme (Austin) Steph., Bull. Herb. Boissier (sér. 2) 1 (5): 494 (56), 1901 (Stephani 1901a). Bas.: Jungermannia crenuliformis Austin, Bull. Torrey Bot. Club 3 (3): 10, 1872 (Austin 1872).

*** Solenostoma decolor (Schiffn.) R.M.Schust. ex Váňa, Hentschel et Heinrichs, Cryptog. Bryol. 31 (2): 136, 2010 (Váňa et al. 2010a). Bas.: Jungermannia decolor Schiffn., Leberm., Forschungsr. Gazelle 4 (4): 10, 1890 (Schiffner 1890).

*** Solenostoma dusenii (Steph.) Váňa, Hentschel et Heinrichs, Cryptog. Bryol. 31 (2): 136, 2010 (Váňa et al. 2010a). Bas.: Nardia dusenii Steph., Hedwigia 30 (5): 209, 1891 (Stephani 1891a).

*** Solenostoma erectum (Amakawa) C.Gao, Fl. Hepat. Chin. Boreali-Orient.: 66, 1981 (Gao and Chang 1981). Bas.: Plectocolea erecta Amakawa, J. Jap. Bot. 32 (10): 307, 1957 (Amakawa 1957b).

* Solenostoma flagellalioides C.Gao, Fl. Hepat. Chin. Boreali-Orient.: 205, 1981 (Gao and Chang 1981).

*** Solenostoma flavialbicans (Amakawa et Grolle) Váňa et D.G.Long, Nova Hedwigia 89 (3/4): 501, 2009 (Váňa and Long 2009). Bas.: Jungermannia flavialbicans Amakawa et Grolle, J. Hattori Bot. Lab. 31: 108, 1968 (Amakawa 1968b).

*** Solenostoma fossombronioides (Austin) R.M.Schust., Hepat. Anthocerotae N. Amer. 2: 1027, 1969 (Schuster 1969b). Bas.: Jungermannia fossombronioides Austin, Proc. Acad. Nat. Sci. Philadelphia 21: 220, 1869 (Austin 1869).

*** Solenostoma glaucum (Amakawa) Váňa et D.G.Long, Nova Hedwigia 89 (3/4): 502, 2009 (Váňa and Long 2009). Bas.: Jungermannia glauca Amakawa, Fl. E. Himalaya: 511, 1966 (Hattori 1966c).

* Solenostoma gongshanense (C.Gao et J.Sun) Váňa et D.G.Long, Nova Hedwigia 89 (3/4): 502, 2009 (Váňa and Long 2009). Bas.: Jungermannia gongshanensis C.Gao et J.Sun, Bull. Bot. Res., Harbin 27 (2): 140, 2007 (Sun and Duan 2007).

*** Solenostoma haskarlianum (Nees) R.M.Schust. ex Váňa et D.G.Long, Nova Hedwigia 89 (3/4): 502, 2009 (Váňa and Long 2009). Bas.: Alicularia haskarliana Nees, Syn. Hepat. 1: 12, 1844 (Gottsche et al. 1844).

*** Solenostoma hattorianum (Amakawa) Potemkin et Nyushko, Liverworts and Hornworts of Russia 1: 287, 2009 (Potemkin and Sofronova 2009). Bas.: Plectocolea hattoriana Amakawa, J. Jap. Bot. 33 (11): 341, 1958 (Amakawa 1958b).

*** Solenostoma hirticalyx (Steph.) R.M.Schust. ex Váňa, Hentschel et Heinrichs, Cryptog. Bryol. 31 (2): 137, 2010 (Váňa et al. 2010a). Bas.: Jungermannia hirticalyx Steph., Sp. Hepat. (Stephani) 6: 87, 1917 (Stephani 1917a).

*** Solenostoma horikawanum (Amakawa) Váňa, Hentschel et Heinrichs, Cryptog. Bryol. 31 (2): 137, 2010 (Váňa et al. 2010a). Bas.: Plectocolea horikawana Amakawa, J. Jap. Bot. 32 (7): 219, 1957 (Amakawa 1957d).

*** Solenostoma hyalinum (Lyell) Mitt., Nat. hist. Azores: 319, 1870 (Mitten 1870). Bas.: Jungermannia hyalina Lyell, Brit. Jungermann.: tab. 63, 1814 (Hooker 1814).

*** Solenostoma infuscum (Mitt.) Hentschel, Pl. Syst. Evol. 268 (1/4): 152, 2007 (Hentschel et al. 2007a). Bas.: Plectocolea infusca Mitt., Trans. Linn. Soc. London, Bot. 3 (3): 196, 1891 (Mitten 1891).

*** Solenostoma infuscum var. ovicalyx (Steph.) Potemkin et Sofronova, Liverworts and Hornworts of Russia 1: 288, 2009 (Potemkin and Sofronova 2009). Bas.: Solenostoma ovicalyx Steph., Sp. Hepat. (Stephani) 6: 82, 1917 (Stephani 1917a).

** Solenostoma kurilense (Bakalin) Váňa, Phytotaxa 152 (1): 40, 2013 (Váňa et al. 2013c). Bas.: Plectocolea flagellata var. kurilensis Bakalin, Arctoa 18: 90, 2009 [2010] (Bakalin et al. 2009b).

* Solenostoma lixingjiangii (C.Gao et X.L.Bai) Váňa et D.G.Long, Nova Hedwigia 89 (3/4): 504, 2009 (Váňa and Long 2009). Bas.: Jungermannia lixingjiangii C.Gao et X.L.Bai, Philipp. Scientist 38: 128, 2001 (Gao and Bai 2001).

*** Solenostoma marginatum (S.Hatt.) R.M.Schust., Hepat. Anthocerotae N. Amer. 2: 983, 1969 (Schuster 1969b). Bas.: Plectocolea marginata S.Hatt., J. Hattori Bot. Lab. 3: 40, 1948 [1950] (Hattori 1948a).

*** Solenostoma micranthum (Mitt.) Váňa, Hentschel et Heinrichs, Cryptog. Bryol. 31 (2): 137, 2010 (Váňa et al. 2010a). Bas.: Plectocolea micrantha Mitt., Fl. vit.: 405, 1871 [1873] (Mitten 1871).

** Solenostoma montanum (Steph.) Váňa, Phytotaxa 65: 44, 2012 (Váňa et al. 2012d). Bas.: Nardia montana Steph., Hedwigia 28 (3): 164, 1889 (Stephani 1889d).

* Solenostoma multicarpum (C.Gao et J.Sun) Váňa et D.G.Long, Nova Hedwigia 89 (3/4): 505, 2009 (Váňa and Long 2009). Bas.: Jungermannia multicarpa C.Gao et J.Sun, Bull. Bot. Res., Harbin 27 (2): 139, 2007 (Sun and Duan 2007).

** Solenostoma nilgiriense (A.Alam, Ad.Kumar et S.C.Srivast.) Váňa et D.G.Long, Nova Hedwigia 89 (3/4): 505, 2009 (Váňa and Long 2009). Bas.: Jungermannia nilgiriensis A.Alam, Ad.Kumar et S.C.Srivast., Bull. Bot. Surv. India 49 (1/4): 220, 2007 (Alam et al. 2007).

*** Solenostoma obliquifolium (Schiffn.) R.M.Schust. ex Váňa, Hentschel et Heinrichs, Cryptog. Bryol. 31 (2): 137, 2010 (Váňa et al. 2010a). Bas.: Nardia obliquifolia Schiffn., Denkschr. Kaiserl. Akad. Wiss., Math.-Naturwiss. Kl. 67: 191, 1898 (Schiffner 1898a).

*** Solenostoma onraedtii (Váňa) Váňa, Hentschel et Heinrichs, Cryptog. Bryol. 31 (2): 137, 2010 (Váňa et al. 2010a). Bas.: Jungermannia onraedtii Váňa, Folia Geobot. Phytotax. 9 (3): 282, 1974 (Váňa 1974a).

* Solenostoma orbicularifolium (Piippo ex C.Gao et Bai) Váňa, Phytotaxa 222 (2): 199, 2015 (Söderström et al. 2015d). Bas.: Jungermannia orbicularifolia Piippo ex C.Gao et Bai, Philipp. Scientist 38: 129, 2001 (Gao and Bai 2001).

*** Solenostoma otianum (S.Hatt.) R.M.Schust., Hepat. Anthocerotae N. Amer. 2: 984, 1969 (Schuster 1969b). Bas.: Plectocolea otiana S.Hatt., J. Jap. Bot. 28 (6): 183, 1953 (Hattori 1953b).

*** Solenostoma ovalifolium (Amakawa) Váňa, Phytotaxa 152 (1): 40, 2013 (Váňa et al. 2013c). Bas.: Plectocolea infusca var. ovalifolia Amakawa, J. Jap. Bot. 34 (4): 115, 1959 (Amakawa 1959b).

*** Solenostoma paroicum (Schiffn.) R.M.Schust., Amer. Midl. Naturalist 49 (2): 402, 1953 (Schuster 1953). Bas.: Nardia paroica Schiffn., Lotos 58: 320, 1910 (Schiffner 1910d).

*** Solenostoma plagiochilaceum (Grolle) Váňa et D.G.Long, Nova Hedwigia 89 (3/4): 505, 2009 (Váňa and Long 2009). Bas.: Jungermannia plagiochilacea Grolle, J. Hattori Bot. Lab. 58: 197, 1985 (Grolle 1985b).

*** Solenostoma polyrhizoides (Grolle ex Amakawa) Váňa et D.G.Long, Nova Hedwigia 89 (3/4): 505, 2009 (Váňa and Long 2009). Bas.: Jungermannia polyrhizoides Grolle ex Amakawa, J. Hattori Bot. Lab. 29: 262, 1966 (Amakawa 1966).

*** Solenostoma radicellosum Mitt., J. Linn. Soc., Bot. 8 (31): 156, 1864 [1865] (Mitten 1864a).

*** Solenostoma renauldii (Steph.) Váňa, Hentschel et Heinrichs, Cryptog. Bryol. 31 (2): 137, 2010 (Váňa et al. 2010a). Bas.: Jungermannia renauldii Steph., Bull. Soc. Roy. Bot. Belgique, Compt. Rend. 30 (2): 201, 1891 [1892] (Stephani 1891b).

*** Solenostoma rigidulum (S.Hatt.) R.M.Schust., Hepat. Anthocerotae N. Amer. 2: 983, 1969 (Schuster 1969b). Bas.: Plectocolea rigidula S.Hatt., J. Jap. Bot. 27 (2): 53, 1952 (Hattori 1952b).

*** Solenostoma rosulans (Steph.) Váňa et D.G.Long, Nova Hedwigia 89 (3/4): 507, 2009 (Váňa and Long 2009). Bas.: Nardia rosulans Steph., Bull. Herb. Boissier 5 (2): 101, 1897 (Stephani 1897b).

*** Solenostoma rotundatum Amakawa, J. Jap. Bot. 31 (2): 50, 1956 (Amakawa 1956).

*** Solenostoma rubripunctatum (S.Hatt.) R.M.Schust., Hepat. Anthocerotae N. Amer. 2: 898, 1969 (Schuster 1969b). Bas.: Plectocolea rubripunctata S.Hatt., J. Hattori Bot. Lab. 3: 41, 1948 [1950] (Hattori 1948a).

* Solenostoma rupicola (Amakawa) Váňa et D.G.Long, Nova Hedwigia 89 (3/4): 507, 2009 (Váňa and Long 2009). Bas.: Jungermannia rupicola Amakawa, J. Hattori Bot. Lab. 22: 23, 1960 (Amakawa 1960b). ^91^

*** Solenostoma sikkimense (Schiffn. ex Steph.) Váňa et D.G.Long, Nova Hedwigia 89 (3/4): 508, 2009 (Váňa and Long 2009). Bas.: Jungermannia sikkimensis Schiffn. ex Steph., Sp. Hepat. (Stephani) 6: 92, 1917 (Stephani 1917a).

*** Solenostoma tetragonum (Lindenb.) R.M.Schust. ex Váňa et D.G.Long, Nova Hedwigia 89 (3/4): 509, 2009 (Váňa and Long 2009). Bas.: Jungermannia tetragona Lindenb., Bot. Zeitung (Berlin) 6 (25): 462, 1848 (Meissner 1848).

* Solenostoma tetragonum var. kodaikanalense A.Alam, D.Sharma et So.Yadav, Phytotaxonomy 12: 70, 2012 (Alam et al. 2012).

*** Solenostoma torticalyx (Steph.) C.Gao, Fl. Hepat. Chin. Boreali-Orient.: 69, 1981 (Gao and Chang 1981). Bas.: Jungermannia torticalyx Steph., Sp. Hepat. (Stephani) 6: 94, 1917 (Stephani 1917a).

*** Solenostoma truncatum (Nees) R.M.Schust. ex Váňa et D.G.Long, Nova Hedwigia 89 (3/4): 509, 2009 (Váňa and Long 2009). Bas.: Jungermannia truncata Nees, Enum. Pl. Crypt. Javae: 29, 1830 (Nees 1830).

** Solenostoma truncatum var. setulosum (Herzog) Váňa et D.G.Long, Nova Hedwigia 89 (3/4): 510, 2009 (Váňa and Long 2009). Bas.: Plectocolea setulosa Herzog, J. Hattori Bot. Lab. 14: 33, 1955 (Herzog and Noguchi 1955).

*** Solenostoma tuberculiferum (Herzog) Váňa, Hentschel et Heinrichs, Cryptog. Bryol. 31 (2): 138, 2010 (Váňa et al. 2010a). Bas.: Aplozia tuberculifera Herzog, Ann. Bryol. 5: 84, 1932 (Herzog 1932a).

*** Solenostoma unispire (Amakawa) Váňa, Hentschel et Heinrichs, Cryptog. Bryol. 31 (2): 138, 2010 (Váňa et al. 2010a). Bas.: Plectocolea unispiris Amakawa, J. Jap. Bot. 29 (6): 178, 1954 (Amakawa 1954).

*** Solenostoma virgatum (Mitt.) Váňa et D.G.Long, Nova Hedwigia 89 (3/4): 510, 2009 (Váňa and Long 2009). Bas.: Plectocolea virgata Mitt., Trans. Linn. Soc. London, Bot. 3 (3): 197, 1891 (Mitten 1891).

*** Solenostoma vulcanicola (Schiffn.) Nyushko ex Potemkin et Sofronova, Liverworts and Hornworts of Russia 1: 289, 2009 (Potemkin and Sofronova 2009). Bas.: Nardia vulcanicola Schiffn., Denkschr. Kaiserl. Akad. Wiss., Math.-Naturwiss. Kl. 67: 191, 1898 (Schiffner 1898a).

* Solenostoma zangmuii (C.Gao et X.L.Bai) Váňa et D.G.Long, Nova Hedwigia 89 (3/4): 510, 2009 (Váňa and Long 2009). Bas.: Jungermannia zangmuii C.Gao et X.L.Bai, Philipp. Scientist 38: 135, 2001 (Gao and Bai 2001).

** **subg. Solenostoma**

*** Solenostoma amoenum (Lindenb. et Gottsche) R.M.Schust. ex Váňa, Hentschel et Heinrichs, Cryptog. Bryol. 31 (2): 136, 2010 (Váňa et al. 2010a). Bas.: Jungermannia amoena Lindenb. et Gottsche, Syn. Hepat. 5: 674, 1847 (Gottsche et al. 1847).

*** Solenostoma amplexifolium (Hampe) Váňa et Schäf.-Verw., Acta Bot. Hung. 51 (3/4): 408, 2009 (Schäfer-Verwimp and Pócs 2009). Bas.: Plagiochila amplexifolia Hampe, Nov. Stirp. Pug. 7: 6, 1838 (Lehmann 1838).

** Solenostoma appalachianum R.M.Schust. ex Bakalin, Arctoa 23: 127, 2014 (Bakalin 2014). Based on: Solenostoma appalachianum R.M.Schust., Rhodora 60 (717): 246, 1958 (Schuster 1958a), *nom. inval*. ^92^

*** Solenostoma appressifolium (Mitt.) Váňa et D.G.Long, Nova Hedwigia 89 (3/4): 494, 2009 (Váňa and Long 2009). Bas.: Jungermannia appressifolia Mitt., J. Proc. Linn. Soc., Bot. 5 (18): 91, 1860 [1861] (Mitten 1860c). ^93^

*** Solenostoma appressifolium var. minor (Amakawa) Váňa et D.G.Long, Nova Hedwigia 89 (3/4): 495, 2009 (Váňa and Long 2009). Bas.: Jungermannia clavellata var. minor Amakawa, J. Hattori Bot. Lab. 26: 25, 1963 (Amakawa 1963).

*** Solenostoma appressifolium var. nigricans (Amakawa) Váňa et D.G.Long, Nova Hedwigia 89 (3/4): 495, 2009 (Váňa and Long 2009). Bas.: Jungermannia decolyana var. nigricans Amakawa, J. Hattori Bot. Lab. 30: 185, 1967 (Amakawa 1967b).

*** Solenostoma ariadne (Taylor) R.M.Schust. ex Váňa et D.G.Long, Nova Hedwigia 89 (3/4): 495, 2009 (Váňa and Long 2009). Bas.: Jungermannia ariadne Taylor, Nov. Stirp. Pug. 8: 9, 1844 (Lehmann 1844).

*** Solenostoma atrobrunneum (Amakawa) Váňa et D.G.Long, Nova Hedwigia 89 (3/4): 495, 2009 (Váňa and Long 2009). Bas.: Jungermannia atrobrunnea Amakawa, J. Hattori Bot. Lab. 30: 192, 1967 (Amakawa 1967b).

*** Solenostoma atrorevolutum (Grolle ex Amakawa) Váňa et D.G.Long, Nova Hedwigia 89 (3/4): 496, 2009 (Váňa and Long 2009). Bas.: Jungermannia atrorevoluta Grolle ex Amakawa, J. Hattori Bot. Lab. 29: 255, 1966 (Amakawa 1966).

*** Solenostoma atrovirens Steph., Bull. Herb. Boissier (sér. 2) 1 (5): 493 (55), 1901 (Stephani 1901a).

*** Solenostoma baueri (Schiffn.) Steph., Bull. Herb. Boissier (sér. 2) 1 (5): 495 (57), 1901 (Stephani 1901a). Bas.: Aplozia baueri Schiffn., Denkschr. Kaiserl. Akad. Wiss., Math.-Naturwiss. Kl. 67: 195, 1898 (Schiffner 1898a).

*** Solenostoma bengalense (Amakawa) Váňa et D.G.Long, Nova Hedwigia 89 (3/4): 496, 2009 (Váňa and Long 2009). Bas.: Jungermannia bengalensis Amakawa, J. Hattori Bot. Lab. 31: 112, 1968 (Amakawa 1968b).

** Solenostoma breviflorum Kashyap et R.S.Chopra, Liverworts W. Himal. 2: 85, 1932 (Kashyap and Chopra 1932).

*** Solenostoma caeleste (Inoue et Váňa) Váňa, Hentschel et Heinrichs, Cryptog. Bryol. 31 (2): 136, 2010 (Váňa et al. 2010a). Bas.: Jungermannia caelestis Inoue et Váňa, Stud. Cryptog. Papua N. Guinea: 16, 1979 (Inoue 1979b).

* Solenostoma caoi (C.Gao et X.L.Bai) Váňa et D.G.Long, Nova Hedwigia 89 (3/4): 496, 2009 (Váňa and Long 2009). Bas.: Jungermannia caoi C.Gao et X.L.Bai, Philipp. Scientist 38: 152, 2001 (Gao and Bai 2001).

*** Solenostoma caucasicum (Váňa) Konstant., Arctoa 1: 123, 1992 (Konstantinova et al. 1992). Bas.: Jungermannia caucasica Váňa, Preslia 42: 96, 1970 (Váňa 1970a).

*** Solenostoma chenianum (C.Gao, Y.H.Wu et Grolle) Váňa et D.G.Long, Nova Hedwigia 89 (3/4): 496, 2009 (Váňa and Long 2009). Bas.: Jungermannia cheniana C.Gao, Y.H.Wu et Grolle, Nova Hedwigia 77 (1/2): 190, 2003 (Gao et al. 2003).

*** Solenostoma clavellatum Mitt. ex Steph., Bull. Herb. Boissier (sér. 2) 1 (5): 491 (53), 1901 (Stephani 1901a).

*** Solenostoma confertissimum (Nees) Schljakov, Novosti Sist. Nizš. Rast. 17: 239, 1980 (Shliakov 1980b). Bas.: Jungermannia confertissima Nees, Naturgesch. Eur. Leberm. 1: 291, 1833 (Nees 1833c).

*** Solenostoma coniflorum (Schiffn.) Steph., Bull. Herb. Boissier (sér. 2) 1 (5): 497 (59), 1901 (Stephani 1901a). Bas.: Jungermannia coniflora Schiffn., Leberm., Forschungsr. Gazelle 4 (4): 10, 1890 (Schiffner 1890).

*** Solenostoma crassulum (Nees et Mont.) Steph., Bull. Herb. Boissier (sér. 2) 1 (5): 497 (59), 1901 (Stephani 1901a). Bas.: Jungermannia crassula Nees et Mont., Ann. Sci. Nat. Bot. (sér. 2) 5: 54, 1836 (Nees and Montagne 1836).

** Solenostoma cryptogynum R.M.Schust. ex J.J.Engel, Novon 17 (3): 311, 2007 (Engel 2007). Based on: Solenostoma cryptogynum R.M.Schust., Beih. Nova Hedwigia 119: 380, 2002 (Schuster 2002b), *nom. inval*.

*** Solenostoma cyclops (S.Hatt.) R.M.Schust., Hepat. Anthocerotae N. Amer. 2: 945, 1969 (Schuster 1969b). Bas.: Jungermannia cyclops S.Hatt., J. Hattori Bot. Lab. 3: 5, 1948 [1950] (Hattori 1948b).

*** Solenostoma diversiclavellatum (Amakawa et Grolle) R.M.Schust. ex Váňa et D.G.Long, Nova Hedwigia 89 (3/4): 501, 2009 (Váňa and Long 2009). Bas.: Jungermannia diversiclavellata Amakawa et Grolle, J. Hattori Bot. Lab. 31: 107, 1968 (Amakawa 1968b).

*** Solenostoma dulongense Váňa et D.G.Long, Nova Hedwigia 89 (3/4): 497, 2009 (Váňa and Long 2009).

*** Solenostoma exsertum (A.Evans) Steph., Bull. Herb. Boissier (sér. 2) 1 (5): 490 (52), 1901 (Stephani 1901a). Bas.: Nardia exserta A.Evans, Trans. Connecticut Acad. Arts 8 (15): 259, 1891 (Evans 1891).

*** Solenostoma faurieanum (Beauverd) R.M.Schust., Hepat. Anthocerotae N. Amer. 2: 945, 1969 (Schuster 1969b). Bas.: Jungermannia faurieana Beauverd, Sp. Hepat. (Stephani) 6: 571, 1924 (Stephani 1924).

*** Solenostoma flagellare (Amakawa) Váňa et D.G.Long, Nova Hedwigia 89 (3/4): 501, 2009 (Váňa and Long 2009). Bas.: Jungermannia flagellaris Amakawa, J. Hattori Bot. Lab. 29: 258, 1966 (Amakawa 1966).

*** Solenostoma flavorevolutum (Váňa) Váňa et D.G.Long, Nova Hedwigia 89 (3/4): 501, 2009 (Váňa and Long 2009). Bas.: Jungermannia flavorevoluta Váňa, J. Hattori Bot. Lab. 36: 63, 1972 [1973] (Váňa 1972b).

*** Solenostoma grollei (D.G.Long et Váňa) K.Feldberg, Hentschel, Bombosch, D.G.Long, Váňa et Heinrichs, Pl. Syst. Evol. 280 (3/4): 244, 2009 (Feldberg et al. 2009). Bas.: Gottschelia grollei D.G.Long et Váňa, J. Bryol. 29 (3): 167, 2007 (Long and Váňa 2007).

*** Solenostoma grosseverrucosum (Amakawa et S.Hatt.) Váňa, Crand.-Stotl. et Stotler, Syst. Bot. 40 (1): 39, 2015 (Shaw et al. 2015). Bas.: Horikawaella grosseverrucosa Amakawa et S.Hatt., Bull. Univ. Mus. Univ. Tokyo 8: 216, 1975 (Hattori 1975e).

*** Solenostoma heterolimbatum (Amakawa) Váňa et D.G.Long, Nova Hedwigia 89 (3/4): 503, 2009 (Váňa and Long 2009). Bas.: Jungermannia heterolimbata Amakawa, J. Hattori Bot. Lab. 30: 183, 1967 (Amakawa 1967b).

*** Solenostoma hewsoniae (Amakawa et Grolle) R.M.Schust. ex Váňa, Hentschel et Heinrichs, Cryptog. Bryol. 31 (2): 136, 2010 (Váňa et al. 2010a). Bas.: Jungermannia hewsoniae Amakawa et Grolle, J. Hattori Bot. Lab. 31: 108, 1968 (Amakawa 1968b).

*** Solenostoma hiugaense Amakawa, J. Jap. Bot. 31 (2): 47, 1956 (Amakawa 1956).

*** Solenostoma indrodayanum (Sushil K.Singh et D.K.Singh) Váňa et D.G.Long, Nova Hedwigia 89 (3/4): 503, 2009 (Váňa and Long 2009). Bas.: Jungermannia indrodayana Sushil K.Singh et D.K.Singh, Cryptog. Bryol. 28 (2): 103, 2007 (Singh and Singh 2007b).

*** Solenostoma inundatum (Hook.f. et Taylor) Mitt. ex Steph., Bull. Herb. Boissier (sér. 2) 1 (5): 490 (52), 1901 (Stephani 1901a). Bas.: Jungermannia inundata Hook.f. et Taylor, London J. Bot. 3: 559, 1844 (Hooker and Taylor 1844d).

*** Solenostoma javanicum (Schiffn.) Steph., Bull. Herb. Boissier (sér. 2) 1 (5): 494 (56), 1901 (Stephani 1901a). Bas.: Aplozia javanica Schiffn., Denkschr. Kaiserl. Akad. Wiss., Math.-Naturwiss. Kl. 67: 193, 1898 (Schiffner 1898a).

** Solenostoma kanaii (Amakawa) Váňa et D.G.Long, Nova Hedwigia 89 (3/4): 503, 2009 (Váňa and Long 2009). Bas.: Jungermannia kanaii Amakawa, J. Hattori Bot. Lab. 30: 194, 1967 (Amakawa 1967b).

*** Solenostoma kashyapii (S.C.Srivast., S.Srivast. et D.Sharma) Váňa et D.G.Long, Nova Hedwigia 89 (3/4): 503, 2009 (Váňa and Long 2009). Bas.: Jungermannia kashyapii S.C.Srivast., S.Srivast. et D.Sharma, Lindbergia 28 (3): 131, 2003 (Srivastava et al. 2003).

*** Solenostoma lanigerum (Mitt.) Váňa et D.G.Long, Nova Hedwigia 89 (3/4): 503, 2009 (Váňa and Long 2009). Bas.: Jungermannia lanigera Mitt., J. Proc. Linn. Soc., Bot. 5 (18): 91, 1860 [1861] (Mitten 1860c).

*** Solenostoma macrocarpum (Schiffn. ex Steph.) Váňa et D.G.Long, Nova Hedwigia 89 (3/4): 504, 2009 (Váňa and Long 2009). Bas.: Jungermannia macrocarpa Schiffn. ex Steph., Sp. Hepat. (Stephani) 6: 87, 1917 (Stephani 1917a).

*** Solenostoma mamatkulovii (Váňa et Zerov) Váňa, Hentschel et Heinrichs, Cryptog. Bryol. 31 (2): 137, 2010 (Váňa et al. 2010a). Bas.: Jungermannia mamatkulovii Váňa et Zerov, Preslia 49: 181, 1977 (Váňa 1977).

* Solenostoma microphyllum C.Gao, Fl. Hepat. Chin. Boreali-Orient.: 206, 1981 (Gao and Chang 1981).

* Solenostoma microrevolutum (C.Gao et X.L.Bai) Váňa et D.G.Long, Nova Hedwigia 89 (3/4): 505, 2009 (Váňa and Long 2009). Bas.: Jungermannia microrevoluta C.Gao et X.L.Bai, Philipp. Scientist 38: 146, 2001 (Gao and Bai 2001).

*** Solenostoma mildbraedii (Steph.) R.M.Schust., Beih. Nova Hedwigia 119: 387, 2002 (Schuster 2002b). Bas.: Jungermannia mildbraedii Steph., Wiss. Ergebn. Deut. Zentr.-Afr. Exped. (1907-08), Bot. 2: 113, 1911 (Stephani 1911a).

*** Solenostoma niveum (Grolle) R.M.Schust. ex Váňa, Hentschel et Heinrichs, Cryptog. Bryol. 31 (2): 137, 2010 (Váňa et al. 2010a). Bas.: Jungermannia nivea Grolle, Misc. Bryol. Lichenol. 6 (1): 1, 1971 (Grolle 1971c).

* Solenostoma novazelandiae R.M.Schust., Beih. Nova Hedwigia 119: 380, 2002 (Schuster 2002b). ^94^

*** Solenostoma ohbae (Amakawa) C.Gao, Bryofl. Xizang: 495, 1985 (Li 1985). Bas.: Jungermannia ohbae Amakawa, Bull. Univ. Mus. Univ. Tokyo 8: 218, 1975 (Hattori 1975e).

*** Solenostoma orbiculatum (Colenso) R.M.Schust., Beih. Nova Hedwigia 119: 380, 2002 (Schuster 2002b). Bas.: Gymnomitrion orbiculatum Colenso, Trans. & Proc. New Zealand Inst. 18: 236, 1886 (Colenso 1886b).

*** Solenostoma parvitextum (Amakawa) Váňa et D.G.Long, Nova Hedwigia 89 (3/4): 505, 2009 (Váňa and Long 2009). Bas.: Jungermannia parvitexta Amakawa, J. Hattori Bot. Lab. 30: 187, 1967 (Amakawa 1967b).

*** Solenostoma patoniae (Grolle, D.B.Schill et D.G.Long) K.Feldberg, Hentschel, Bombosch, D.G.Long, Váňa et Heinrichs, Pl. Syst. Evol. 280 (3/4): 244, 2009 (Feldberg et al. 2009). Bas.: Gottschelia patoniae Grolle, D.B.Schill et D.G.Long, J. Bryol. 25 (1): 3, 2003 (Grolle et al. 2003).

*** Solenostoma pocsii (Váňa) Bakalin, Arctoa 18: 160, 2009 [2010] (Bakalin and Vilnet 2009). Bas.: Jungermannia pocsii Váňa, Folia Geobot. Phytotax. 10 (4): 365, 1975 (Váňa 1975b).

*** Solenostoma poeltii (Amakawa) Váňa et D.G.Long, Nova Hedwigia 89 (3/4): 505, 2009 (Váňa and Long 2009). Bas.: Jungermannia poeltii Amakawa, J. Hattori Bot. Lab. 29: 258, 1966 (Amakawa 1966).

*** Solenostoma pseudocyclops (Inoue) Váňa et D.G.Long, Nova Hedwigia 89 (3/4): 506, 2009 (Váňa and Long 2009). Bas.: Jungermannia pseudocyclops Inoue, Bull. Natl. Sci. Mus. Tokyo (n.ser.) 9 (1): 37, 1966 (Inoue 1966a).

** Solenostoma pseudopyriflorum Bakalin et Vilnet, Arctoa 18: 159, 2009 [2010] (Bakalin and Vilnet 2009).

*** Solenostoma purpuratum (Mitt.) Steph., Bull. Herb. Boissier (sér. 2) 1 (5): 489 (51), 1901 (Stephani 1901a). Bas.: Jungermannia purpurata Mitt., J. Proc. Linn. Soc., Bot. 5 (18): 91, 1860 [1861] (Mitten 1860c).

*** Solenostoma pyriflorum Steph., Sp. Hepat. (Stephani) 6: 83, 1917 (Stephani 1917a). ^95^

*** Solenostoma pyriflorum var. gracillimum (Amakawa) Váňa et D.G.Long, Nova Hedwigia 89 (3/4): 506, 2009 (Váňa and Long 2009). Bas.: Jungermannia pyriflora var. gracillima Amakawa, Fl. E. Himalaya 2: 228, 1971 (Hattori 1971a).

** Solenostoma pyriflorum var. major (S.Hatt.) Bakalin, Arctoa 17: 230, 2008 [2009] (Bakalin 2008a). Bas.: Jungermannia monticola f. major S.Hatt., J. Hattori Bot. Lab. 3: 8, 1948 [1950] (Hattori 1948b).

*** Solenostoma pyriflorum var. minutissimum (Amakawa) Bakalin, Arctoa 16: 208, 2007 [2008] (Bakalin 2007). Bas.: Jungermannia pyriflora var. minutissima Amakawa, J. Hattori Bot. Lab. 22: 61, 1960 (Amakawa 1960b). ^96^

*** Solenostoma raujeanum (Grolle ex Amakawa) Váňa et D.G.Long, Nova Hedwigia 89 (3/4): 507, 2009 (Váňa and Long 2009). Bas.: Jungermannia raujeana Grolle ex Amakawa, J. Hattori Bot. Lab. 29: 262, 1966 (Amakawa 1966).

*** Solenostoma riclefii Váňa et D.G.Long, Nova Hedwigia 89 (3/4): 507, 2009 (Váňa and Long 2009). *Nom. nov. pro Jungermannia grollei* Amakawa, J. Hattori Bot. Lab. 29: 260, 1966 (Amakawa 1966).

*** Solenostoma sanguinolentum (Griff.) Steph., Bull. Herb. Boissier (sér. 2) 1 (5): 489 (51), 1901 (Stephani 1901a). Bas.: Jungermannia sanguinolenta Griff., Not. pl. asiat. 2: 302, 1849 (Griffith 1849).

*** Solenostoma schaulianum (Steph.) Váňa et D.G.Long, Nova Hedwigia 89 (3/4): 508, 2009 (Váňa and Long 2009). Bas.: Jungermannia schauliana Steph., Sp. Hepat. (Stephani) 6: 90, 1917 (Stephani 1917a).

*** Solenostoma shimizuanum (S.Hatt. ex Váňa) Váňa, Hentschel et Heinrichs, Cryptog. Bryol. 31 (2): 138, 2010 (Váňa et al. 2010a). Bas.: Jungermannia shimizuana S.Hatt. ex Váňa, J. Hattori Bot. Lab. 35: 315, 1972 (Váňa 1972a).

*** Solenostoma speciosum (Horik.) Hentschel, K.Feldberg, Bombosch, D.G.Long, Váňa et Heinrichs, Pl. Syst. Evol. 280 (3/4): 244, 2009 (Feldberg et al. 2009). Bas.: Anastrophyllum speciosum Horik., J. Sci. Hiroshima Univ., Ser. B, Div. 2, Bot. 2: 147, 1934 (Horikawa 1934).

** Solenostoma speciosum subsp. villosum (R.M.Schust.) Hentschel, K.Feldberg, Bombosch, D.G.Long, Váňa et Heinrichs, Pl. Syst. Evol. 280 (3/4): 246, 2009 (Feldberg et al. 2009). Bas.: Scaphophyllum speciosum subsp. villosum R.M.Schust., Bryologist 101 (3): 434, 1998 (Schuster 1998b).

*** Solenostoma sphaerocarpum (Hook.) Steph., Bull. Herb. Boissier (sér. 2) 1 (5): 499 (61), 1901 (Stephani 1901a). Bas.: Jungermannia sphaerocarpa Hook., Brit. Jungermann.: tab. 74, 1815 (Hooker 1815).

*** Solenostoma stephanii (Schiffn.) Steph., Bull. Herb. Boissier (sér. 2) 1 (5): 496 (58), 1901 (Stephani 1901a). Bas.: Aplozia stephanii Schiffn., Denkschr. Kaiserl. Akad. Wiss., Math.-Naturwiss. Kl. 67: 195, 1898 (Schiffner 1898a).

*** Solenostoma stoloniferum (Steph.) S.W.Arnell, Hepat. South Africa: 316, 1963 (Arnell 1963b). Bas.: Nardia stolonifera Steph., Hedwigia 31 (3): 128, 1892 (Stephani 1892d).

*** Solenostoma strictum (Schiffn.) Váňa, Hentschel et Heinrichs, Cryptog. Bryol. 31 (2): 138, 2010 (Váňa et al. 2010a). Bas.: Aplozia stricta Schiffn., Denkschr. Kaiserl. Akad. Wiss., Math.-Naturwiss. Kl. 67: 194, 1898 (Schiffner 1898a).

*** Solenostoma subacutum (Herzog) Váňa, Crand.-Stotl. et Stotler, Syst. Bot. 40 (1): 39, 2015 (Shaw et al. 2015). Bas.: Anastrophyllum subacutum Herzog, Ann. Bryol. 12: 75, 1939 (Herzog 1939b).

*** Solenostoma subrubrum (Schiffn. ex Steph.) Váňa et D.G.Long, Nova Hedwigia 89 (3/4): 508, 2009 (Váňa and Long 2009). Bas.: Jungermannia subrubra Schiffn. ex Steph., Sp. Hepat. (Stephani) 6: 93, 1917 (Stephani 1917a).

* Solenostoma sunii Bakalin et Vilnet, Bot. Pacifica 3 (2): 15, 2014 (Bakalin et al. 2014).

* Solenostoma totopapillosum (E.A.Hodgs.) R.M.Schust., Bryologist 100 (3): 366, 1997 (Schuster 1997a). Bas.: Jungermannia totopapillosa E.A.Hodgs., J. Roy. Soc. New Zealand 2 (1): 111, 1972 (Hodgson 1972). ^97^

*** Solenostoma udarii (S.C.Srivast. et P.Singh) Váňa et D.G.Long, Nova Hedwigia 89 (3/4): 510, 2009 (Váňa and Long 2009). Bas.: Jungermannia udarii S.C.Srivast. et P.Singh, Recent Stud. Indian Bryoph.: 152, 1995 (Srivastava and Singh 1995).

* Solenostoma ventroversum (Grolle) Váňa et D.G.Long, Nova Hedwigia 89 (3/4): 510, 2009 (Váňa and Long 2009). Bas.: Jungermannia ventroversa Grolle, Khumbu Himal 1 (4): 284, 1966 (Grolle 1966k). ^98^

*** Solenostoma zantenii (Amakawa) R.M.Schust. ex Váňa et D.G.Long, Nova Hedwigia 89 (3/4): 510, 2009 (Váňa and Long 2009). Bas.: Jungermannia zantenii Amakawa, J. Hattori Bot. Lab. 31: 110, 1968 (Amakawa 1968b).

* Solenostoma zengii (C.Gao et X.L.Bai) Váňa et D.G.Long, Nova Hedwigia 89 (3/4): 510, 2009 (Váňa and Long 2009). Bas.: Jungermannia zengii C.Gao et X.L.Bai, Philipp. Scientist 38: 151, 2001 (Gao and Bai 2001).

######## *** Southbyaceae Váňa, Crand.-Stotl., Stotler et D.G.Long

by J. Váňa

Southbyaceae has traditionally been placed in Arnelliaceae (e.g. Crandall-Stotler et al. 2009) or kept as a separate family (e.g. Vanden Berghen 1957) that has never been validated. However, it was re-established, rearranged and validated in the study by Váňa et al. (2012e).

*** **Gongylanthus Nees**, Naturgesch. Eur. Leberm. 2: 405, 1836 (Nees 1836).

*** Gongylanthus dusenii Steph., Bull. Herb. Boissier (sér. 2) 6 (5): 385 (41), 1906 (Stephani 1906a).

*** Gongylanthus ericetorum (Raddi) Nees, Naturgesch. Eur. Leberm. 2: 407, 1836 (Nees 1836). Bas.: Calypogeia ericetorum Raddi, Jungermanniogr. Etrusca: 31, 1818 (Raddi 1818a).

*** Gongylanthus granatensis (Gottsche) Steph., Bull. Herb. Boissier (sér. 2) 6 (5): 385 (41), 1906 (Stephani 1906a). Bas.: Lindigina granatensis Gottsche, Ann. Sci. Nat. Bot. (sér. 5) 1: 138, 1864 (Gottsche 1864).

*** Gongylanthus himalayensis Grolle, Khumbu Himal 1 (4): 285, 1966 (Grolle 1966k).

*** Gongylanthus javanicus Grolle, J. Jap. Bot. 40 (7): 206, 1965 (Grolle 1965d).

*** Gongylanthus liebmanianus (Lindenb. et Gottsche) Steph., Bull. Herb. Boissier (sér. 2) 6 (5): 388 (44), 1906 (Stephani 1906a). Bas.: Gymnanthe liebmaniana Lindenb. et Gottsche, Syn. Hepat. 5: 712, 1847 (Gottsche et al. 1847).

*** Gongylanthus limbatus (Herzog) Grolle et Váňa, Folia Geobot. Phytotax. 9 (2): 198, 1974 (Váňa 1974c). Bas.: Aplozia limbata Herzog, Beih. Bot. Centralbl. 61B (3): 561, 1942 (Herzog 1942d).

*** Gongylanthus muelleri (Gottsche) Steph., Bull. Herb. Boissier (sér. 2) 6 (5): 388 (44), 1906 (Stephani 1906a). Bas.: Lindigia muelleri Gottsche, Mexik. Leverm.: 121, 1863 (Gottsche 1863).

** Gongylanthus oniscoides (Spruce) Steph., Bull. Herb. Boissier (sér. 2) 6 (5): 386 (42), 1906 (Stephani 1906a). Bas.: Calypogeia oniscoides Spruce, Trans. & Proc. Bot. Soc. Edinburgh 15: 448, 1885 (Spruce 1885).

*** Gongylanthus richardsii E.W.Jones, Trans. Brit. Bryol. Soc. 4 (4): 650, 1964 (Jones 1964).

*** **Southbya Spruce**, Ann. Mag. Nat. Hist. (ser. 2) 3 (18): 501, 1849 (Spruce 1849).

*** Southbya gollanii Steph., Bull. Herb. Boissier (sér. 2) 6 (5): 381 (37), 1906 (Stephani 1906a).

*** Southbya nigrella (De Not.) Henriq., Bol. Soc. Brot. 4: 244, 1886 [1887] (Henriques 1886). Bas.: Jungermannia nigrella De Not., Mem. Reale Accad. Sci. Torino (ser. 2) 1: 315, 1838 (De Notaris 1838).

*** Southbya organensis Herzog, Memoranda Soc. Fauna Fl. Fennica 25: 53, 1950 (Herzog 1950c).

*** Southbya tophacea (Spruce) Spruce, Ann. Mag. Nat. Hist. (ser. 2) 3 (18): 501, 1849 (Spruce 1849). Bas.: Jungermannia tophacea Spruce, Hep. Pyr. Exsic.: no. 23, 1847 (Spruce 1847).

######## ** Stephaniellaceae R.M.Schust.

by J. Váňa

In the absence of molecular data, the phylogenetic affinities of Stephaniella and Stephaniellidium remain equivocal, but there is no evidence to support their placement in either the Arnelliaceae or Southbyaceae where they have been placed recently. Thus the family as construed by Schuster (2002b) is retained.

*** **Stephaniella J.B.Jack**, Hedwigia 33 (1): 11, 1894 (Jack 1894).

* Stephaniella boliviensis Steph., Biblioth. Bot. 87 (2): 182, 1916 (Stephani 1916a).

*** Stephaniella hamata Steph., Bull. Herb. Boissier (sér. 2) 1 (10): 1024 (87), 1901 (Stephani 1901c).

*** Stephaniella paraphyllina J.B.Jack, Hedwigia 33 (1): 11, 1894 (Jack 1894).

*** Stephaniella rostrata U.Schmitt, Österr. Bot. Z. 115 (2): 124, 1968 (Schmitt and Winkler 1968).

*** Stephaniella uncifolia S.Winkl., Österr. Bot. Z. 115 (2): 124, 1968 (Schmitt and Winkler 1968).

** **Stephaniellidium S.Winkl. ex Grolle**, Acta Bot. Fenn. 121: 38, 1983 (Grolle 1983b). Based on: Stephaniellidium S.Winkl., Mitt. Inst. Colombo-Alemán Invest. Ci. 3: 60, 1969 (Winkler 1969).

*** Stephaniellidium sleumeri (Müll.Frib.) S.Winkl. ex Grolle, Acta Bot. Fenn. 121: 38, 1983 (Grolle 1983b). Bas.: Stephaniella sleumeri Müll.Frib., Rev. Bryol. Lichénol. 20 (1/2): 177, 1951 (Müller 1951b).

######## *** Trichotemnomataceae R.M.Schust.

*** **Trichotemnoma R.M.Schust.**, Nova Hedwigia 15: 440, 1968 (Schuster 1968b).

*** Trichotemnoma corrugatum (Steph.) R.M.Schust., Nova Hedwigia 15: 440, 1968 (Schuster 1968b). Bas.: Blepharostoma corrugatum Steph., Hedwigia 32 (5): 315, 1893 (Stephani 1893d).

####### Lophocoleineae Schljakov

######## *** Blepharostomataceae W.Frey et M.Stech

The recognition of Blepharostomataceae follows Frey and Stech (2008) following reconsideration of molecular and morphological data available at that time.

*** **Blepharostoma (Dumort.) Dumort.**, Recueil Observ. Jungerm.: 18, 1835 (Dumortier 1835). Bas.: Jungermannia sect. Blepharostoma Dumort., Syll. Jungerm. Europ.: 65, 1831 (Dumortier 1831).

** Blepharostoma arachnoideum M.Howe, Mem. Torrey Bot. Club 7: 140, 1899 (Howe 1899).

** Blepharostoma indicum G.Asthana, M.Saxena et Maurya, J. Bryol. 35 (4): 267, 2013 (Asthana et al. 2013).

** Blepharostoma minor Horik., Hikobia 1 (2): 104, 1951 [1952] (Horikawa 1951b).

*** Blepharostoma trichophyllum (L.) Dumort., Recueil Observ. Jungerm.: 18, 1835 (Dumortier 1835). Bas.: Jungermannia trichophylla L., Sp. Pl. 1: 1135, 1753 (Linnaeus 1753).

** Blepharostoma trichophyllum subsp. brevirete (Bryhn et Kaal.) R.M.Schust., Bull. Natl. Mus. Canada 164: 16, 1959 (Schuster et al. 1959). Bas.: Blepharostoma trichophyllum var. brevirete Bryhn et Kaal., Rep. Second Norweg. Arctic Exped. 11: 46, 1906 (Bryhn 1906).

######## *** Brevianthaceae J.J.Engel et R.M.Schust.

by L. Söderström, J. Váňa, R. Stotler, B.J. Crandall-Stotler and J.J. Engel

The treatment of Brevianthaceae follows Söderström et al. (2013b).

*** **Brevianthus J.J.Engel et R.M.Schust.**, Phytologia 47 (4): 317, 1981 (Engel and Schuster 1981).

*** Brevianthus flavus (Grolle) J.J.Engel et R.M.Schust., Phytologia 47 (4): 318, 1981 (Engel and Schuster 1981). Bas.: Jackiella flava Grolle, J. Hattori Bot. Lab. 33: 222, 1970 (Grolle 1970b).

** Brevianthus flavus subsp. crenulatus J.J.Engel, Nova Hedwigia 93 (3/4): 406, 2011 (Engel 2011).

* Brevianthus hypocanthidium M.A.M.Renner et J.J.Engel, PhytoKeys 50: 46, 2015 (Renner et al. 2015).

*** **Tetracymbaliella Grolle**, Nova Hedwigia 3 (1): 48, 1961 (Grolle 1961b).

** Tetracymbaliella comptonii (Pearson) Grolle, Rev. Bryol. Lichénol. 32 (3/4): 164, 1963 [1964] (Grolle 1963d). Bas.: Chiloscyphus comptonii Pearson, J. Linn. Soc., Bot. 46 (305): 23, 1922 (Pearson 1922b).

*** Tetracymbaliella cymbalifera (Hook.f. et Taylor) Grolle, Nova Hedwigia 3 (1): 50, 1961 (Grolle 1961b). Bas.: Jungermannia cymbalifera Hook.f. et Taylor, Bot. Antarct. Voy. I (Fl. Antarct. 1): 151, 1845 (Taylor and Hooker 1845).

*** Tetracymbaliella decipiens (Gottsche) Grolle, Nova Hedwigia 3 (1): 49, 1961 (Grolle 1961b). Bas.: Chiloscyphus decipiens Gottsche, Syn. Hepat. 2: 176, 1845 (Gottsche et al. 1845a).

*** Tetracymbaliella subsimplex (Austin) J.J.Engel, Phytotaxa 207 (2): 185, 2015 (Engel 2015b). Bas.: Polyotus subsimplex Austin, Bull. Torrey Bot. Club 6 (7): 46, 1875 (Austin 1875c).

######## ** Chonecoleaceae R.M.Schust. ex Grolle

** **Chonecolea Grolle**, Rev. Bryol. Lichénol. 25 (3/4): 294, 1956 [1957] (Grolle 1956).

** Chonecolea acutiloba (Schiffn.) R.M.Schust., Hepat. Anthocerotae N. Amer. 4: 321, 1980 (Schuster 1980c). Bas.: Clasmatocolea acutiloba Schiffn., Österr. Akad. Wiss., Math.-Naturwiss. Kl., Denkschr. 111: 63, 1964 (Schiffner and Arnell 1964).

** Chonecolea andina Grolle et Váňa, J. Hattori Bot. Lab. 48: 229, 1980 (Váňa 1980).

** Chonecolea doellingeri (Nees) Grolle, Rev. Bryol. Lichénol. 25 (3/4): 295, 1956 [1957] (Grolle 1956). Bas.: Jungermannia doellingeri Nees, Syn. Hepat. 1: 104, 1844 (Gottsche et al. 1844).

** Chonecolea ruwenzorensis E.W.Jones, J. Bryol. 13 (4): 498, 1986 (Jones 1986).

** Chonecolea schusteri Udar et Ad.Kumar, Bryologist 85 (3): 315, 1982 (Udar and Kumar 1982a).

** Chonecolea verae Potemkin, Proc. int. meeting 90th anniv. Abramova: 165, 2005 (Potemkin 2005).

######## ** Grolleaceae Solari ex R.M.Schust.

** **Grollea R.M.Schust.**, Nova Hedwigia 8 (3/4): 288, 1964 (Schuster 1964b).

** Grollea antheliopsis R.M.Schust., Nova Hedwigia 8 (3/4): 288, 1964 (Schuster 1964b).

######## *** Herbertaceae Müll.Frib. ex Fulford et Hatcher

by D. Bell

*** **Herbertus Gray**, Nat. Arr. Brit. Pl. 1: 705, 1821 (Gray 1821). ^99^

*** Herbertus aduncus (Dicks.) Gray, Nat. Arr. Brit. Pl. 1: 705, 1821 (Gray 1821). Bas.: Jungermannia adunca Dicks., Fasc. Pl. Crypt. Brit. 3: 12, 1793 (Dickson 1793).

** Herbertus arcticus (Inoue et Steere) Schljakov, Novosti Sist. Nizš. Rast. 19: 209, 1982 (Shliakov 1982). Bas.: Herbertus sakuraii subsp. arcticus Inoue et Steere, J. Hattori Bot. Lab. 44: 266, 1978 (Steere and Inoue 1978).

** Herbertus armitanus (Steph.) H.A.Mill., J. Hattori Bot. Lab. 28: 324, 1965 (Miller 1965). Bas.: Schisma armitanum Steph., Sp. Hepat. (Stephani) 4: 28, 1909 (Stephani 1909d).

** Herbertus asparus Tixier, Bryophyt. Biblioth. 18: 64, 1979 (Tixier 1979a).

*** Herbertus bivittatus Spruce, Trans. & Proc. Bot. Soc. Edinburgh 15: 343, 1885 (Spruce 1885).

** Herbertus borealis Crundw., Trans. Brit. Bryol. Soc. 6 (1): 41, 1970 (Crundwell 1970).

** Herbertus buchii Juslén, Ann. Bot. Fenn. 43 (6): 416, 2006 (Juslén 2006a).

** Herbertus ceylanicus (Steph.) Abeyw., Ceylon J. Sci., Biol. Sci. 2 (1): 43, 1959 (Abeywickrama 1959). Bas.: Schisma ceylanicum Steph., Sp. Hepat. (Stephani) 4: 22, 1909 (Stephani 1909d).

** Herbertus circinatus (Steph.) H.A.Mill., J. Hattori Bot. Lab. 28: 318, 1965 (Miller 1965). Bas.: Schisma circinatum Steph., Sp. Hepat. (Stephani) 4: 25, 1909 (Stephani 1909d).

*** Herbertus delavayi (Steph.) Steph., Hedwigia 34 (2): 43, 1895 (Stephani 1895c). Bas.: Schisma delavayi Steph., Mém. Soc. Nat. Sci. Nat. Math. Cherbourg 29: 228, 1894 (Stephani 1894b). ^100^

*** Herbertus dicranus (Gottsche, Lindenb. et Nees) Trevis., Mem. Reale Ist. Lombardo Sci. (Ser. 3), C. Sci. Mat. 4 (13): 397, 1877 (Trevisan 1877). Bas.: Sendtnera dicrana Gottsche, Lindenb. et Nees, Syn. Hepat. 2: 239, 1845 (Gottsche et al. 1845a).

** Herbertus durandii (Steph.) Herzog, Rev. Bryol. Lichénol. 11 (1): 25, 1938 [1939] (Herzog 1938a). Bas.: Schisma durandii Steph., Trans. Linn. Soc. London, Bot. 6 (1): 99, 1901 (Stephani 1901e).

* Herbertus evittatus (Steph.) H.A.Mill., J. Hattori Bot. Lab. 28: 327, 1965 (Miller 1965). Bas.: Schisma evittatum Steph., Sp. Hepat. (Stephani) 6: 357, 1922 (Stephani 1922).

** Herbertus gaochienii X.Fu, Fl. Bryoph. Sin. 9: 38, 2003 (Gao 2003).

** Herbertus gracilis (Mont.) Steph., Bull. Herb. Boissier 5 (10): 842, 1897 (Stephani 1897c). Bas.: Mastigophora gracilis Mont., Ann. Sci. Nat. Bot. (sér. 2) 19: 254, 1843 (Montagne 1843).

** Herbertus guangdongii P.J.Lin ex Piippo, Bryobrothera 1: 206, 1992 (Lin et al. 1992).

** Herbertus hawaiiensis H.A.Mill., J. Hattori Bot. Lab. 28: 317, 1965 (Miller 1965).

** Herbertus helleri (Steph.) W.E.Nicholson, Rev. Bryol. Lichénol. 13: 143, 1942 (Nicholson 1942). Bas.: Schisma helleri Steph., Sp. Hepat. (Stephani) 4: 29, 1909 (Stephani 1909d).

** Herbertus herpocladioides E.B.Scott et H.A.Mill., Bryologist 62 (2): 116, 1959 (Scott and Miller 1959).

*** Herbertus hutchinsiae (Gottsche et Rabenh.) A.Evans, Bull. Torrey Bot. Club 44 (4): 214, 1917 (Evans 1917c). Bas.: Sendtnera adunca var. hutchinsiae Gottsche et Rabenh., Hepat. Eur., Leberm. 21-22: no. 210, 1862 (Rabenhorst 1862).

*** Herbertus juniperoideus (Sw.) Grolle, Rev. Bryol. Lichénol. 30 (1/2): 80, 1961 (Grolle 1961a). Bas.: Jungermannia juniperoidea Sw., Prodr. (Swartz): 144, 1788 (Swartz 1788). ^101^

*** Herbertus juniperoideus subsp. acanthelius (Spruce) K.Feldberg et Heinrichs, Bot. J. Linn. Soc. 151: 326, 2006 (Feldberg and Heinrichs 2006). Bas.: Herbertus acanthelius Spruce, Trans. & Proc. Bot. Soc. Edinburgh 15: 341, 1885 (Spruce 1885).

*** Herbertus juniperoideus subsp. pensilis (Taylor) K.Feldberg et Heinrichs, Bot. J. Linn. Soc. 151: 329, 2006 (Feldberg and Heinrichs 2006). Bas.: Sendtnera pensilis Taylor, London J. Bot. 5: 372, 1846 (Taylor 1846b).

** Herbertus kurzii (Steph.) R.S.Chopra, J. Indian Bot. Soc. 22: 247, 1943 (Chopra 1943). Bas.: Schisma kurzii Steph., Sp. Hepat. (Stephani) 4: 24, 1909 (Stephani 1909d).

** Herbertus leratii (Steph.) H.A.Mill., J. Hattori Bot. Lab. 28: 327, 1965 (Miller 1965). Bas.: Schisma leratii Steph., Sp. Hepat. (Stephani) 6: 360, 1922 (Stephani 1922).

** Herbertus lonchobasis H.A.Mill., J. Hattori Bot. Lab. 28: 306, 1965 (Miller 1965).

** Herbertus longifissus Steph., Hedwigia 34 (2): 44, 1895 (Stephani 1895c).

** Herbertus longispinus J.B.Jack et Steph., Hedwigia 31 (1): 15, 1892 (Jack and Stephani 1892).

** Herbertus mauritianus N.G.Hodgetts, J. Bryol. 30 (4): 247, 2008 (Hodgetts 2008).

*** Herbertus norenus D.G.Long, D.Bell et H.H.Blom, Molec. Ecol. Res. 12: 44, 2012 (Bell et al. 2012).

** Herbertus oldfieldianus (Steph.) Rodway, Tasm. Bryoph.: 72, 1917 (Rodway 1917b). Bas.: Schisma oldfieldianum Steph., Sp. Hepat. (Stephani) 4: 20, 1909 (Stephani 1909d).

** Herbertus pilifer Schiffn., Nova Acta Acad. Caes. Leop.-Carol. German. Nat. Cur. 60 (2): 252, 1893 (Schiffner 1893a).

** Herbertus pocsii N.G.Hodgetts, J. Bryol. 30 (4): 249, 2008 (Hodgetts 2008).

** Herbertus pumilus Steph., Hedwigia 34 (2): 44, 1895 (Stephani 1895c).

** Herbertus ramosus (Steph.) H.A.Mill., J. Hattori Bot. Lab. 28: 314, 1965 (Miller 1965). Bas.: Schisma ramosum Steph., Sp. Hepat. (Stephani) 4: 23, 1909 (Stephani 1909d).

** Herbertus runcinatus (Taylor) Trevis., Mem. Reale Ist. Lombardo Sci. (Ser. 3), C. Sci. Mat. 4 (13): 397, 1877 (Trevisan 1877). Bas.: Sendtnera runcinata Taylor, London J. Bot. 5: 372, 1846 (Taylor 1846b).

*** Herbertus sendtneri (Nees) Lindb., Hepat. Scand. Exsicc.: no. 4, 1874 (Lindberg and Lackström 1874). Bas.: Schisma sendtneri Nees, Naturgesch. Eur. Leberm. 3: 575, 1838 (Nees 1838b).

** Herbertus spicatus N.G.Hodgetts, J. Bryol. 30 (4): 244, 2008 (Hodgetts 2008).

*** Herbertus stramineus (Dumort.) Trevis., Mem. Reale Ist. Lombardo Sci. (Ser. 3), C. Sci. Mat. 4 (13): 397, 1877 (Trevisan 1877). Bas.: Schisma stramineum Dumort., Syll. Jungerm. Europ.: 77, 1831 (Dumortier 1831).

** Herbertus streimannii M.L.So, Syst. Bot. 28 (1): 13, 2003 (So 2003).

*** Herbertus tenuis A.Evans, Bull. Torrey Bot. Club 44 (4): 219, 1917 (Evans 1917c).

** Herbertus udarii D.Kumar et N.Manocha, Geophytology 29 (1/2): 105, 1999 [2000] (Kumar and Manocha 1999).

**Excluded from the genus**

* Herbertus subrotundatus X.Fu et Y.J.Yi, Acta Phytotax. Sin. 39 (1): 89, 2001 (Yi et al. 2001). ^102^

*** **Triandrophyllum Fulford et Hatcher**, Bryologist 64 (4): 349, 1961 [1962] (Fulford and Hatcher 1961). Based on: Triandrophyllum Fulford et Hatcher, Bryologist 61 (4): 277, 1958 [1959] (Fulford and Hatcher 1958).

*** Triandrophyllum eophyllum (R.M.Schust.) Gradst., Mem. New York Bot. Gard. 86: 104, 2001 (Gradstein et al. 2001a). Bas.: Olgantha eophylla R.M.Schust., Nova Hedwigia 63 (3/4): 535, 1996 (Schuster 1996b).

** Triandrophyllum fernandeziense (S.W.Arnell) Grolle ex Fulford et Hatcher, Bryologist 64 (4): 351, 1961 [1962] (Fulford and Hatcher 1961). Bas.: Acromastigum fernandeziense S.W.Arnell, Ark. Bot. (n.ser.) 4 (1): 10, 1957 (Arnell 1957b).

** Triandrophyllum heterophyllum (Steph.) Grolle, J. Jap. Bot. 39 (8): 238, 1964 (Grolle 1964g). Bas.: Mastigophora heterophylla Steph., Sp. Hepat. (Stephani) 6: 367, 1922 (Stephani 1922).

*** Triandrophyllum subtrifidum (Hook.f. et Taylor) Fulford et Hatcher, Bryologist 64 (4): 350, 1961 [1962] (Fulford and Hatcher 1961). Bas.: Jungermannia subtrifida Hook.f. et Taylor, London J. Bot. 3: 579, 1844 (Hooker and Taylor 1844c).

** Triandrophyllum subtrifidum var. trifidum (Gottsche) Solari, Bol. Soc. Argent. Bot. 15 (2/3): 201, 1973 (Solari 1973). Bas.: Sendtnera trifida Gottsche, Ann. Sci. Nat. Bot. (sér. 5) 1: 142, 1864 (Gottsche 1864).

** Triandrophyllum symmetricum J.J.Engel, Haussknechtia, Beih. 9: 115, 1999 (Engel 1999b).

######## *** Lepicoleaceae R.M.Schust.

by M. von Konrat

Lepicoleaceae has conventionally been considered a monogeneric family only containing Lepicolea (e.g. Crandall-Stotler et al. 2009). However, we expand Lepicoleaceae here to include the monogeneric family Vetaformaceae. A number of molecular analyses have shown that Vetaforma dusenii is sister to Lepicolea (e.g. He-Nygrén et al. 2006, Juslén 2006b) together forming a robust clade.

*** **Lepicolea Dumort.**, Recueil Observ. Jungerm.: 20, 1835 (Dumortier 1835).

*** Lepicolea attenuata (Mitt.) Steph., J. Linn. Soc., Bot. 29 (201): 276, 1892 (Stephani 1892b). Bas.: Sendtnera attenuata Mitt., Bot. antarct. voy. II (Fl. Nov.-Zel. 2): 153, 1854 (Mitten 1854).

*** Lepicolea magellanica (Gola) Solari, Lindbergia 9 (2): 86, 1983 (Solari 1983b). Bas.: Lepicolea scolopendra var. magellanica Gola, Nuovo Giorn. Bot. Ital. (n.ser.) 29 (1/4): 170, 1922 [1923] (Gola 1922).

*** Lepicolea norrisii Piippo, Ann. Bot. Fenn. 25 (1): 55, 1988 (Piippo 1988).

*** Lepicolea ochroleuca (Spreng.) Spruce, Trans. & Proc. Bot. Soc. Edinburgh 15: 345, 1885 (Spruce 1885). Bas.: Jungermannia ochroleuca Spreng. Syst. Veg. (ed. 16) [Sprengel] 4 (2): 325, 1827 (Sprengel 1827b).

*** Lepicolea pruinosa (Taylor) Spruce, Trans. & Proc. Bot. Soc. Edinburgh 15: 345, 1885 (Spruce 1885). Bas.: Sendtnera pruinosa Taylor, London J. Bot. 5: 373, 1846 (Taylor 1846b).

*** Lepicolea ramentifissa Herzog, Biblioth. Bot. 88: 30, 1920 [1921] (Herzog 1920).

*** Lepicolea rara (Steph.) Grolle, Nova Hedwigia 16: 152, 1968 (Grolle 1968d). Bas.: Lepidozia rara Steph., Sp. Hepat. (Stephani) 3: 618, 1909 (Stephani 1909a). ^103^

*** Lepicolea rigida (De Not.) E.B.Scott, Nova Hedwigia 2: 148, 1960 (Scott 1960). Bas.: Sendtnera rigida De Not., Mem. Reale Accad. Sci. Torino (ser. 2) 16: 229, 1857 (De Notaris 1857).

*** Lepicolea scolopendra (Hook.) Dumort. ex Trevis., Mem. Reale Ist. Lombardo Sci. (Ser. 3), C. Sci. Mat. 4 (13): 398, 1877 (Trevisan 1877). Bas.: Jungermannia scolopendra Hook., Musci Exot. 1: tab. 40, 1818 (Hooker 1818).

*** Lepicolea yakusimensis (S.Hatt.) S.Hatt., J. Hattori Bot. Lab. 10: 42, 1953 (Hattori 1953c). Bas.: Lepicolea scolopendra var. yakusimensis S.Hatt., J. Hattori Bot. Lab. 2: 9, 1947 [1948] (Hattori 1947b).

*** **Vetaforma Fulford et J.Taylor**, Nova Hedwigia 4 (1/2): 81, 1962 (Fulford 1962b).

*** Vetaforma dusenii (Steph.) Fulford et J.Taylor, Nova Hedwigia 4 (1/2): 82, 1962 (Fulford 1962b). Bas.: Lepidozia dusenii Steph., Bih. Kongl. Svenska Vetensk.-Akad. Handl. 26 (III, 6): 52, 1900 (Stephani 1900b).

######## *** Lepidoziaceae Limpr.

by E.D. Cooper

The classification within Lepidoziaceae is in a state of flux. The treatment provided here follows the interim classification proposed by Cooper (2013) based on recent molecular phylogenetic studies (Heslewood and Brown 2007, Cooper et al. 2011, 2012a, 2012b) incorporating nomenclatural changes from Cooper et al. (2013). Several higher taxa are unlikely to represent monophyletic units, but the phylogenetic data currently available are insufficient to re-circumscribe the doubtful subfamilies and genera (Cooper 2013). Infrageneric taxa have been retained where phylogenetic data are absent or inconclusive.

** **Meinungeria Frank Müll.**, Bryologist 110 (3): 494, 2007 (Müller 2007).

** Meinungeria mouensis Frank Müll., Bryologist 110 (3): 494, 2007 (Müller 2007).

######### *** Bazzanioideae Rodway

*** **Acromastigum A.Evans**, Bull. Torrey Bot. Club 27 (3): 103, 1900 (Evans 1900b). ^104^

** **subg. Acromastigum**

*** Acromastigum caledonicum (Steph.) Grolle, Österr. Bot. Z. 111 (2/3): 243, 1964 (Grolle 1964h). Bas.: Acolea caledonica Steph., Nova Caledonia, Bot. 1: 19, 1914 (Stephani 1914c).

*** Acromastigum cavifolium R.M.Schust., J. Hattori Bot. Lab. 26: 257, 1963 (Schuster 1963b).

** Acromastigum herzogii Grolle, Österr. Bot. Z. 111 (2/3): 250, 1964 (Grolle 1964h).

*** Acromastigum homodictyon (Herzog) Grolle, Österr. Bot. Z. 111 (2/3): 245, 1964 (Grolle 1964h). Bas.: Acromastigum integrifolium var. homodictyon Herzog, Ark. Bot. (n.ser.) 3 (3): 44, 1953 (Herzog 1953a).

** Acromastigum integrifolium (Austin) A.Evans, Bull. Torrey Bot. Club 27 (3): 103, 1900 (Evans 1900b). Bas.: Mastigobryum integrifolium Austin, Bot. Bull. (Hanover) 1 (7): 32, 1876 (Austin 1876b).

*** Acromastigum stellare N.Kitag., Acta Phytotax. Geobot. 36 (4/6): 109, 1985 (Kitagawa 1985).

** Acromastigum stenophyllum R.M.Schust., Nova Hedwigia 15: 465, 1968 (Schuster 1968b).

*** Acromastigum verticale (Steph.) E.A.Hodgs., Trans. Roy. Soc. New Zealand 82 (1): 18, 1954 (Hodgson 1954). Bas.: Bazzania verticalis Steph., Hedwigia 32 (4): 214, 1893 (Stephani 1893c).

** **subg. Inaequilatera (Schiffn.) Grolle**, J. Hattori Bot. Lab. 44: 2, 1978 (Grolle 1978b). Bas.: Bazzania sect. Inaequilaterae Schiffn., Hepat. (Engl.-Prantl): 101, 1893 (Schiffner 1893b).

*** Acromastigum adaptatum Hürl., Bauhinia 7 (4): 263, 1983 (Hürlimann 1983).

*** Acromastigum anisostomum (Lehm. et Lindenb.) A.Evans, Ann. Bryol., Suppl. 3: 48, 1934 (Evans 1934). Bas.: Jungermannia anisostoma Lehm. et Lindenb., Nov. Stirp. Pug. 6: 57, 1834 (Lehmann 1834).

** Acromastigum anisostomum var. minutum E.A.Hodgs., Trans. Roy. Soc. New Zealand, Bot. 3 (4): 71, 1965 (Hodgson 1965).

** Acromastigum aurescens A.Evans, Ann. Bryol., Suppl. 3: 45, 1934 (Evans 1934).

** Acromastigum bancanum (Sande Lac.) A.Evans, Ann. Bryol., Suppl. 3: 20, 1934 (Evans 1934). Bas.: Mastigobryum bancanum Sande Lac., Ann. Mus. Bot. Lugduno-Batavi 1: 301, 1864 (Sande Lacoste 1864).

** Acromastigum brotheri (Steph.) A.Evans, Ann. Bryol., Suppl. 3: 70, 1934 (Evans 1934). Bas.: Mastigobryum brotheri Steph., Sp. Hepat. (Stephani) 3: 536, 1909 (Stephani 1909a).

** Acromastigum capillare (Steph.) A.Evans, Ann. Bryol., Suppl. 3: 37, 1934 (Evans 1934). Bas.: Mastigobryum capillare Steph., Sp. Hepat. (Stephani) 6: 457, 1924 (Stephani 1924).

*** Acromastigum colensoanum (Mitt.) A.Evans ex Reimers, Hedwigia 73 (3/4): 142, 1933 (Reimers 1933). Bas.: Mastigobryum colensoanum Mitt., Bot. antarct. voy. II (Fl. Nov.-Zel. 2): 147, 1854 (Mitten 1854).

*** Acromastigum cunninghamii (Steph.) A.Evans, Ann. Bryol., Suppl. 3: 106, 1934 (Evans 1934). Bas.: Bazzania cunninghamii Steph., Hedwigia 32 (4): 205, 1893 (Stephani 1893c).

** Acromastigum curtilobum A.Evans, Ann. Bryol., Suppl. 3: 97, 1934 (Evans 1934).

*** Acromastigum divaricatum (Nees) A.Evans ex Reimers, Hedwigia 73 (3/4): 142, 1933 (Reimers 1933). Bas.: Mastigobryum divaricatum Nees, Syn. Hepat. 2: 219, 1845 (Gottsche et al. 1845a).

** Acromastigum echinatiforme (De Not.) A.Evans, Ann. Bryol., Suppl. 3: 64, 1934 (Evans 1934). Bas.: Mastigobryum echinatiforme De Not., Epat. Borneo: 38, 1874 (De Notaris 1874).

* Acromastigum echinatum (Gottsche) A.Evans, Ann. Bryol., Suppl. 3: 147, 1934 (Evans 1934). Bas.: Mastigobryum echinatum Gottsche, Syn. Hepat. 2: 218, 1845 (Gottsche et al. 1845a). ^105^

*** Acromastigum exiguum (Steph.) A.Evans, Ann. Bryol., Suppl. 3: 75, 1934 (Evans 1934). Bas.: Mastigobryum exiguum Steph., Hedwigia 25 (1): 6, 1886 (Stephani 1886e).

** Acromastigum exile (Lindenb.) A.Evans, Ann. Bryol., Suppl. 3: 24, 1934 (Evans 1934). Bas.: Mastigobryum exile Lindenb., Syn. Hepat. 2: 217, 1845 (Gottsche et al. 1845a).

*** Acromastigum filum (Steph.) A.Evans, Ann. Bryol., Suppl. 3: 31, 1934 (Evans 1934). Bas.: Bazzania filum Steph., Hedwigia 32 (4): 206, 1893 (Stephani 1893c).

*** Acromastigum filum var. papillosum N.Kitag., Acta Phytotax. Geobot. 36 (4/6): 112, 1985 (Kitagawa 1985).

** Acromastigum fimbriatum (Steph.) A.Evans, Ann. Bryol., Suppl. 3: 153, 1934 (Evans 1934). Bas.: Mastigobryum fimbriatum Steph., Sp. Hepat. (Stephani) 3: 538, 1909 (Stephani 1909a).

** Acromastigum furcatifolium (Steph.) E.A.Br., Phytotaxa 65: 58, 2012 (Brown et al. 2012). Bas.: Lepidozia furcatifolia Steph., J. & Proc. Roy. Soc. New South Wales 48 (1/2): 112, 1914 (Stephani and Watts 1914).

*** Acromastigum inaequilaterum (Lehm. et Lindenb.) A.Evans, Ann. Bryol., Suppl. 3: 129, 1934 (Evans 1934). Bas.: Jungermannia inaequilatera Lehm. et Lindenb., Nov. Stirp. Pug. 6: 56, 1834 (Lehmann 1834).

** Acromastigum interstisiale E.A.Br. et M.A.M.Renner, Telopea 17: 268, 2014 (Brown and Renner 2014).

** Acromastigum laetevirens (Sande Lac. ex Steph.) A.Evans, Ann. Bryol., Suppl. 3: 94, 1934 (Evans 1934). Bas.: Mastigobryum laetevirens Sande Lac. ex Steph., Hedwigia 25 (4): 133, 1886 (Stephani 1886b).

** Acromastigum laevigatum A.Evans, Ann. Bryol., Suppl. 3: 101, 1934 (Evans 1934).

** Acromastigum linganum (De Not.) A.Evans, Ann. Bryol., Suppl. 3: 118, 1934 (Evans 1934). Bas.: Mastigobryum linganum De Not., Epat. Borneo: 37, 1874 (De Notaris 1874).

** Acromastigum lobuliferum A.Evans, Ann. Bryol., Suppl. 3: 157, 1934 (Evans 1934).

** Acromastigum longirete Grolle, J. Hattori Bot. Lab. 44: 11, 1978 (Grolle 1978b).

*** Acromastigum mooreanum (Steph.) E.A.Hodgs., Trans. Roy. Soc. New Zealand 82 (1): 19, 1954 (Hodgson 1954). Bas.: Bazzania mooreana Steph., Hedwigia 33 (1): 1, 1894 (Stephani 1894a).

*** Acromastigum moratii N.Kitag., Acta Phytotax. Geobot. 36 (4/6): 119, 1985 (Kitagawa 1985).

** Acromastigum obliquatum (Mitt.) A.Evans, Ann. Bryol., Suppl. 3: 110, 1934 (Evans 1934). Bas.: Bazzania obliquata Mitt., Hedwigia 32 (4): 211, 1893 (Stephani 1893c).

** Acromastigum tenax (Steph.) A.Evans, Ann. Bryol., Suppl. 3: 41, 1934 (Evans 1934). Bas.: Mastigobryum tenax Steph., Sp. Hepat. (Stephani) 6: 483, 1924 (Stephani 1924).

***Incertae sedis***

** Acromastigum fumosum E.A.Br. et M.A.M.Renner, Telopea 17: 274, 2014 (Brown and Renner 2014).

** Acromastigum leptophyllum Herzog, Trans. Brit. Bryol. Soc. 1 (4): 309, 1950 (Herzog 1950a).

*** Acromastigum marginatum E.A.Hodgs., Trans. Roy. Soc. New Zealand 82 (1): 22, 1954 (Hodgson 1954).

** Acromastigum microstictum A.Evans, Ann. Bryol., Suppl. 3: 115, 1934 (Evans 1934).

** Acromastigum prismaticale E.A.Br. et M.A.M.Renner, Telopea 17: 281, 2014 (Brown and Renner 2014).

** Acromastigum pusillum N.Kitag., Acta Phytotax. Geobot. 36 (4/6): 116, 1985 (Kitagawa 1985).

** Acromastigum rigidum R.M.Schust., Nova Hedwigia 64 (3/4): 617, 1997 (Schuster 1997b).

** Acromastigum subechinatiforme Hürl., Bauhinia 7 (4): 266, 1983 (Hürlimann 1983).

*** **Bazzania Gray**, Nat. Arr. Brit. Pl. 1: 704, 1821 (Gray 1821) nom. conserv. ^106^

*** Bazzania acanthostipa Spruce, Trans. & Proc. Bot. Soc. Edinburgh 15: 381, 1885 (Spruce 1885).

*** Bazzania accreta (Lehm. et Lindenb.) Trevis., Mem. Reale Ist. Lombardo Sci. (Ser. 3), C. Sci. Mat. 4 (13): 414, 1877 (Trevisan 1877). Bas.: Mastigobryum accretum Lehm. et Lindenb., Syn. Hepat. 2: 222, 1845 (Gottsche et al. 1845a).

** Bazzania acinaciformis Steph., Akad. Wiss. Wien, Math.-Naturwiss. Kl., Denkschr. 81: 290, 1907 (Stephani 1907a).

** Bazzania acuminata (Lindenb. et Gottsche) Trevis., Mem. Reale Ist. Lombardo Sci. (Ser. 3), C. Sci. Mat. 4 (13): 414, 1877 (Trevisan 1877). Bas.: Mastigobryum acuminatum Lindenb. et Gottsche, Syn. Hepat. 5: 719, 1847 (Gottsche et al. 1847).

** Bazzania acutifolia (Steph.) Schiffn., Consp. Hepat. Arch. Ind.: 147, 1898 (Schiffner 1898b). Bas.: Mastigobryum acutifolium Steph., Hedwigia 24 (5): 214, 1885 (Stephani 1885a).

*** Bazzania adnexa (Lehm. et Lindenb.) Trevis., Mem. Reale Ist. Lombardo Sci. (Ser. 3), C. Sci. Mat. 4 (13): 414, 1877 (Trevisan 1877). Bas.: Jungermannia adnexa Lehm. et Lindenb., Nov. Stirp. Pug. 4: 58, 1832 (Lehmann 1832).

** Bazzania adnexa var. aucklandica (Lindenb. et Gottsche) J.J.Engel et G.L.Merr., Bryologist 97 (3): 319, 1994 (Engel and Smith Merrill 1994). Bas.: Mastigobryum novae-hollandiae f. aucklandicum Lindenb. et Gottsche, Sp. Hepat. (Lindenberg) 8-11: 33, 1851 (Lindenberg and Gottsche 1851b).

*** Bazzania affinis (Lindenb. et Gottsche) Trevis., Mem. Reale Ist. Lombardo Sci. (Ser. 3), C. Sci. Mat. 4 (13): 415, 1877 (Trevisan 1877). Bas.: Mastigobryum affine Lindenb. et Gottsche, Syn. Hepat. 5: 720, 1847 (Gottsche et al. 1847).

** Bazzania albifolia Horik., J. Sci. Hiroshima Univ., Ser. B, Div. 2, Bot. 2: 195, 1934 (Horikawa 1934).

** Bazzania ambigua (Lindenb.) Trevis., Mem. Reale Ist. Lombardo Sci. (Ser. 3), C. Sci. Mat. 4 (13): 414, 1877 (Trevisan 1877). Bas.: Mastigobryum ambiguum Lindenb., Syn. Hepat. 2: 217, 1845 (Gottsche et al. 1845a).

*** Bazzania amblyphylla Meagher, Nova Hedwigia 92 (3/4): 487, 2011 (Meagher 2011).

** Bazzania aneityensis (Steph.) Tixier, Bull. Mus. Natl. Hist. Nat. (Sér. 3), Bot. 10 (190): 80, 1973 (Tixier 1973d). Bas.: Mastigobryum aneityense Steph., J. & Proc. Roy. Soc. New South Wales 48 (1/2): 121, 1914 (Stephani and Watts 1914).

** Bazzania angusta (Steph.) Herzog, Trans. Brit. Bryol. Soc. 1 (4): 307, 1950 (Herzog 1950a). Bas.: Mastigobryum angustum Steph., Sp. Hepat. (Stephani) 6: 453, 1924 (Stephani 1924).

** Bazzania angustifalcata Herzog, Feddes Repert. Spec. Nov. Regni Veg. 57 (1/2): 166, 1955 (Herzog 1955).

** Bazzania angustifolia Horik., J. Sci. Hiroshima Univ., Ser. B, Div. 2, Bot. 2: 198, 1934 (Horikawa 1934).

** Bazzania angustisedens (Steph.) N.Kitag., J. Hattori Bot. Lab. 36: 445, 1972 [1973] (Kitagawa 1972). Bas.: Mastigobryum angustisedens Steph., Bull. Herb. Boissier (sér. 2) 8 (10): 745 (429), 1908 (Stephani 1908c).

** Bazzania angustistipula N.Kitag., J. Hattori Bot. Lab. 30: 268, 1967 (Kitagawa 1967a).

*** Bazzania appendiculata (Mitt.) S.Hatt., Fl. E. Himalaya: 505, 1966 (Hattori 1966c). Bas.: Mastigobryum appendiculatum Mitt., J. Proc. Linn. Soc., Bot. 5 (18): 105, 1860 [1861] (Mitten 1860c).

** Bazzania approximata Onr., Bull. Jard. Bot. Natl. Belg. 47 (1/2): 139, 1977 (Onraedt 1977).

** Bazzania arcuata (Lindenb. et Gottsche) Trevis., Mem. Reale Ist. Lombardo Sci. (Ser. 3), C. Sci. Mat. 4 (13): 414, 1877 (Trevisan 1877). Bas.: Mastigobryum arcuatum Lindenb. et Gottsche, Syn. Hepat. 5: 718, 1847 (Gottsche et al. 1847).

** Bazzania arcuata var. mamillosa Gradst. et A.R.Benitez, Nova Hedwigia 99 (1/2): 113, 2014 (Gradstein and Benitez 2014).

** Bazzania armatistipula (Steph.) Fulford, Ann. Cryptog. Phytopathol. 3: 114, 1946 (Fulford 1946). Bas.: Mastigobryum armatistipulum Steph., Bull. Herb. Boissier (sér. 2) 8 (11): 866 (490), 1908 (Stephani 1908a).

** Bazzania asperrima Steph., Rev. Bryol. 34 (3): 48, 1907 (Paris 1907).

* Bazzania asymmetrica (Steph.) N.Kitag., Bull. Nara Univ. Educ., B 28 (2): 77, 1979 (Kitagawa 1979a). Bas.: Mastigobryum asymmetricum Steph., Sp. Hepat. (Stephani) 6: 454, 1924 (Stephani 1924). ^107^

** Bazzania aterrima (Steph.) N.Kitag., Bull. Nara Univ. Educ., B 26 (2): 77, 1977 (Kitagawa 1977). Bas.: Mastigobryum aterrimum Steph., Sp. Hepat. (Stephani) 6: 454, 1924 (Stephani 1924).

*** Bazzania aurescens Spruce, Trans. & Proc. Bot. Soc. Edinburgh 15: 374, 1885 (Spruce 1885).

** Bazzania avia Meagher, Nova Hedwigia 97 (3/4): 529, 2013 (Meagher 2013).

** Bazzania azorica H.Buch et Perss., Bryophyt. Azoren Madeira: 3, 1941 (Buch and Persson 1941).

** Bazzania baldwinii Austin, Trans. Connecticut Acad. Arts 8 (15): 255, 1891 (Evans 1891).

** Bazzania bernieri (Steph.) Inoue et H.A.Mill., Bull. Natl. Sci. Mus. Tokyo (n.ser.) 8 (2): 142, 1965 (Inoue and Miller 1965). Bas.: Mastigobryum bernieri Steph., Bull. Herb. Boissier (sér. 2) 8 (11): 852 (476), 1908 (Stephani 1908a).

*** Bazzania bescherellei Steph., Hedwigia 32 (4): 204, 1893 (Stephani 1893c).

** Bazzania bhutanica N.Kitag. et Grolle, J. Hattori Bot. Lab. 61: 269, 1986 [1987] (Kitagawa and Grolle 1986).

** Bazzania bicrenata N.Kitag., J. Hattori Bot. Lab. 47: 127, 1980 (Kitagawa 1980).

*** Bazzania bidens (Gottsche et Lindenb.) Trevis., Mem. Reale Ist. Lombardo Sci. (Ser. 3), C. Sci. Mat. 4 (13): 415, 1877 (Trevisan 1877). Bas.: Mastigobryum bidens Gottsche et Lindenb., Syn. Hepat. 2: 228, 1845 (Gottsche et al. 1845a).

** Bazzania bidens var. heterodonta Spruce, Trans. & Proc. Bot. Soc. Edinburgh 15: 372, 1885 (Spruce 1885).

** Bazzania bidentula (Steph.) Yasuda, Shokubutsugaku Kakuron: 711, 1911 (Yasuda 1911). Bas.: Pleuroschisma bidentulum Steph., Mém. Soc. Nat. Sci. Nat. Math. Cherbourg 29: 222, 1894 (Stephani 1894b).

*** Bazzania bilobata N.Kitag., J. Hattori Bot. Lab. 30: 257, 1967 (Kitagawa 1967a).

** Bazzania borneensis N.Kitag., J. Hattori Bot. Lab. 37: 263, 1973 (Kitagawa 1973). *Nom. nov. pro Mastigobryum borneense* Steph., Bull. Herb. Boissier (sér. 2) 8 (11): 839 (463), 1908 (Stephani 1908a), *nom. illeg*.

** Bazzania brasiliensis (Gottsche et Lindenb.) Trevis., Mem. Reale Ist. Lombardo Sci. (Ser. 3), C. Sci. Mat. 4 (13): 415, 1877 (Trevisan 1877). Bas.: Mastigobryum brasiliense Gottsche et Lindenb., Syn. Hepat. 2: 227, 1845 (Gottsche et al. 1845a).

** Bazzania brighamii (Austin) A.Evans, Trans. Connecticut Acad. Arts 8 (15): 255, 1891 (Evans 1891). Bas.: Mastigobryum brighamii Austin, Bull. Torrey Bot. Club 5 (3): 16, 1874 (Austin 1874).

** Bazzania cadens N.Kitag., J. Hattori Bot. Lab. 47: 129, 1980 (Kitagawa 1980).

*** Bazzania calcarata (Sande Lac.) Schiffn., Consp. Hepat. Arch. Ind.: 149, 1898 (Schiffner 1898b). Bas.: Mastigobryum calcaratum Sande Lac., Ann. Mus. Bot. Lugduno-Batavi 1: 304, 1864 (Sande Lacoste 1864).

** Bazzania callida (Sande Lac. ex Steph.) Abeyw., Ceylon J. Sci., Biol. Sci. 2 (1): 45, 1959 (Abeywickrama 1959). Bas.: Mastigobryum callidum Sande Lac. ex Steph., Hedwigia 24 (6): 246, 1885 (Stephani 1885b).

*** Bazzania canelensis (Steph.) Fulford, Ann. Cryptog. Phytopathol. 3: 152, 1946 (Fulford 1946). Bas.: Mastigobryum canelense Steph., Sp. Hepat. (Stephani) 3: 518, 1909 (Stephani 1909a).

** Bazzania caudata (Steph.) Herzog, Trans. Brit. Bryol. Soc. 1 (4): 301, 1950 (Herzog 1950a). Bas.: Mastigobryum caudatum Steph., Bull. Herb. Boissier (sér. 2) 8 (12): 947 (497), 1908 (Stephani 1908b).

*** Bazzania caudistipula (Steph.) Inoue et H.A.Mill., Bull. Natl. Sci. Mus. Tokyo (n.ser.) 8 (2): 141, 1965 (Inoue and Miller 1965). Bas.: Mastigobryum caudistipulum Steph., Bull. Herb. Boissier (sér. 2) 8 (12): 945 (495), 1908 (Stephani 1908b).

** Bazzania ceylanica (Mitt.) Steph., Bot. Jahrb. Syst. 23 (1/2, 3): 306, 1896 (Stephani 1896a). Bas.: Mastigobryum ceylanicum Mitt., J. Proc. Linn. Soc., Bot. 5 (18): 105, 1860 [1861] (Mitten 1860c).

** Bazzania chilensis (Steph.) Fulford, Ann. Cryptog. Phytopathol. 3: 51, 1946 (Fulford 1946). Bas.: Mastigobryum chilense Steph., Hedwigia 24 (6): 247, 1885 (Stephani 1885b).

*** Bazzania chimantensis Fulford, Bryologist 63 (2): 89, 1960 (Fulford 1960).

** Bazzania cincinnata (De Not.) Trevis., Mem. Reale Ist. Lombardo Sci. (Ser. 3), C. Sci. Mat. 4 (13): 414, 1877 (Trevisan 1877). Bas.: Mastigobryum cincinnatum De Not., Epat. Borneo: 34, 1874 (De Notaris 1874).

*** Bazzania citharodes Meagher, Nova Hedwigia 86 (3/4): 481, 2008 (Meagher 2008).

** Bazzania combinata (J.B.Jack et Steph.) Steph., Bot. Jahrb. Syst. 23 (1/2, 3): 306, 1896 (Stephani 1896a). Bas.: Mastigobryum combinatum J.B.Jack et Steph., Bot. Centralbl. 60 (4): 102, 1894 (Jack and Stephani 1894).

** Bazzania commutata (Lindenb. et Gottsche) Schiffn., Consp. Hepat. Arch. Ind.: 149, 1898 (Schiffner 1898b). Bas.: Mastigobryum commutatum Lindenb. et Gottsche, Sp. Hepat. (Lindenberg) 8-11: 97, 1851 (Lindenberg and Gottsche 1851b).

* Bazzania comorensis Steph., Bull. Soc. Roy. Bot. Belgique, Compt. Rend. 30 (2): 197, 1891 [1892] (Stephani 1891b).

** Bazzania confertifolia (Steph.) Herzog, Trans. Brit. Bryol. Soc. 1 (4): 298, 1950 (Herzog 1950a). Bas.: Mastigobryum confertifolium Steph., Bull. Herb. Boissier (sér. 2) 8 (11): 858 (482), 1908 (Stephani 1908a).

** Bazzania conistipula (Steph.) H.A.Mill., Phytologia 47 (4): 320, 1981 (Miller 1981). Bas.: Mastigobryum conistipulum Steph., Sp. Hepat. (Stephani) 6: 458, 1924 (Stephani 1924).

** Bazzania conophylla (Sande Lac.) Schiffn., Consp. Hepat. Arch. Ind.: 150, 1898 (Schiffner 1898b). Bas.: Mastigobryum conophyllum Sande Lac., Ann. Mus. Bot. Lugduno-Batavi 1: 304, 1864 (Sande Lacoste 1864).

** Bazzania consanguinea (Hampe et Lindenb.) Trevis., Mem. Reale Ist. Lombardo Sci. (Ser. 3), C. Sci. Mat. 4 (13): 414, 1877 (Trevisan 1877). Bas.: Mastigobryum consanguineum Hampe et Lindenb., Linnaea 20 (3): 327, 1847 (Hampe 1847).

** Bazzania consociata (Steph.) H.A.Mill., Phytologia 47 (4): 320, 1981 (Miller 1981). Bas.: Mastigobryum consociatum Steph., Sp. Hepat. (Stephani) 6: 458, 1924 (Stephani 1924).

* Bazzania corbieri (Steph.) Meagher, Nova Hedwigia 86 (3/4): 483, 2008 (Meagher 2008). Bas.: Mastigobryum corbieri Steph., J. & Proc. Roy. Soc. New South Wales 48 (1/2): 122, 1914 (Stephani and Watts 1914). ^108^

** Bazzania crassidentata Fulford, Bull. Torrey Bot. Club 86 (5): 338, 1959 (Fulford 1959b).

** Bazzania crassitexta Steph., Hedwigia 32 (4): 205, 1893 (Stephani 1893c).

* Bazzania crenata (Trevis.) Trevis., Mem. Reale Ist. Lombardo Sci. (Ser. 3), C. Sci. Mat. 4 (13): 415, 1877 (Trevisan 1877). Bas.: Pleuroschisma crenatum Trevis., Herb. Crypt. Trev. 2: 30, 1853 (Trevisan 1853; non vidi). ^109^

** Bazzania cubensis (Gottsche ex Steph.) Pagán, Bryologist 42 (2): 38, 1939 (Pagán 1939b). Bas.: Mastigobryum cubense Gottsche ex Steph., Hedwigia 24 (6): 248, 1885 (Stephani 1885b).

** Bazzania cucullata Onr., Bull. Jard. Bot. Natl. Belg. 47 (1/2): 142, 1977 (Onraedt 1977).

*** Bazzania cuneistipula (Gottsche et Lindenb.) Trevis., Mem. Reale Ist. Lombardo Sci. (Ser. 3), C. Sci. Mat. 4 (13): 414, 1877 (Trevisan 1877). Bas.: Mastigobryum cuneistipulum Gottsche et Lindenb., Syn. Hepat. 2: 225, 1845 (Gottsche et al. 1845a).

* Bazzania curvidens Steph., Bull. Soc. Roy. Bot. Belgique, Compt. Rend. 30 (2): 197, 1891 [1892] (Stephani 1891b).

** Bazzania debilis N.Kitag., J. Hattori Bot. Lab. 30: 256, 1967 (Kitagawa 1967a).

** Bazzania deciduifolia Onr., Bull. Jard. Bot. Natl. Belg. 47 (1/2): 144, 1977 (Onraedt 1977).

*** Bazzania decrescens (Lehm. et Lindenb.) Trevis., Mem. Reale Ist. Lombardo Sci. (Ser. 3), C. Sci. Mat. 4 (13): 414, 1877 (Trevisan 1877). Bas.: Jungermannia decrescens Lehm. et Lindenb., Nov. Stirp. Pug. 4: 57, 1832 (Lehmann 1832). ^110^

** Bazzania decrescens var. dentistipula Kiaer et Pearson, Forh. Vidensk.-Selsk. Kristiania 1892 (14): 6, 1893 (Pearson 1893).

** Bazzania decrescens subsp. molleri (Steph.) E.W.Jones, J. Bryol. 8 (3): 303, 1975 (Jones 1975). Bas.: Mastigobryum molleri Steph., Bot. Jahrb. Syst. 8 (2): 84, 1886 (Stephani 1886d).

** Bazzania decrescens subsp. pumila (Mitt.) Pócs, Trop. Bryol. 9: 129, 1994 (Pócs 1994c). Bas.: Bazzania pumila Mitt., J. Linn. Soc., Bot. 22 (146): 322, 1886 (Mitten 1886b).

** Bazzania densa (Sande Lac.) Schiffn., Consp. Hepat. Arch. Ind.: 151, 1898 (Schiffner 1898b). Bas.: Mastigobryum densum Sande Lac., Ned. Kruidk. Arch. 3: 418, 1854 [1855] (Sande Lacoste 1854).

** Bazzania densa var. connata (Sande Lac.) Schiffn., Consp. Hepat. Arch. Ind.: 151, 1898 (Schiffner 1898b). Bas.: Mastigobryum densum β connatum Sande Lac., Ann. Mus. Bot. Lugduno-Batavi 1: 302, 1864 (Sande Lacoste 1864).

*** Bazzania denticulata (Lindenb. et Gottsche) Trevis., Mem. Reale Ist. Lombardo Sci. (Ser. 3), C. Sci. Mat. 4 (13): 414, 1877 (Trevisan 1877). Bas.: Mastigobryum denticulatum Lindenb. et Gottsche, Syn. Hepat. 5: 718, 1847 (Gottsche et al. 1847).

** Bazzania denticulifera Mägd., Nova Hedwigia 38: 53, 1983 (Mägdefrau 1983).

** Bazzania denudata (Lindenb. et Gottsche) Trevis., Mem. Reale Ist. Lombardo Sci. (Ser. 3), C. Sci. Mat. 4 (13): 414, 1877 (Trevisan 1877). Bas.: Mastigobryum denudatum Lindenb. et Gottsche, Syn. Hepat. 2: 216, 1845 (Gottsche et al. 1845a).

*** Bazzania deplanchei (Gottsche) Jovet-Ast, Rev. Bryol. Lichénol. 18 (1/2): 83, 1949 (Jovet-Ast 1949b). Bas.: Mastigobryum deplanchei Gottsche, Bull. Herb. Boissier (sér. 2) 8 (12): 955 (505), 1908 (Stephani 1908b).

** Bazzania deplanchei var. filamentosa Tixier, Cryptog. Bryol. Lichénol. 6 (2): 179, 1985 (Tixier 1985b).

** Bazzania desciscens (Steph.) Meijer, Blumea 10 (2): 382, 1960 (Meijer 1960). Bas.: Mastigobryum desciscens Steph., Bull. Herb. Boissier (sér. 2) 8 (11): 862 (486), 1908 (Stephani 1908a).

** Bazzania didericiana (Gottsche ex Steph.) Steph., Bull. Herb. Boissier 5 (10): 841, 1897 (Stephani 1897c). Bas.: Mastigobryum didericianum Gottsche ex Steph., Hedwigia 24 (6): 249, 1885 (Stephani 1885b).

** Bazzania diminuta Herzog, Trans. Brit. Bryol. Soc. 1 (4): 305, 1950 (Herzog 1950a).

* Bazzania distans (Nees) Trevis., Mem. Reale Ist. Lombardo Sci. (Ser. 3), C. Sci. Mat. 4 (13): 414, 1877 (Trevisan 1877). Bas.: Mastigobryum distans Nees, Syn. Hepat. 2: 216, 1845 (Gottsche et al. 1845a). ^111^

*** Bazzania diversicuspis Spruce, Trans. & Proc. Bot. Soc. Edinburgh 15: 373, 1885 (Spruce 1885).

** Bazzania drepanophylla Herzog, Trans. Brit. Bryol. Soc. 1 (4): 301, 1950 (Herzog 1950a).

** Bazzania dulitensis Herzog, Trans. Brit. Bryol. Soc. 1 (4): 299, 1950 (Herzog 1950a).

** Bazzania dulongensis L.P.Zhou et Li Zhang, J. Bryol. 34 (1): 22, 2012 (Zhou et al. 2012).

** Bazzania eggersiana (Steph.) Pagán, Bryologist 42 (2): 39, 1939 (Pagán 1939b). Bas.: Mastigobryum eggersianum Steph., Bull. Herb. Boissier (sér. 2) 8 (11): 844 (468), 1908 (Stephani 1908a).

** Bazzania elmeri (Steph.) N.Kitag., J. Hattori Bot. Lab. 36: 446, 1972 [1973] (Kitagawa 1972). Bas.: Mastigobryum elmeri Steph., Leafl. Philipp. Bot. 6: 2289, 1914 (Stephani 1914a).

** Bazzania elongata Fulford, Bull. Torrey Bot. Club 86 (5): 337, 1959 (Fulford 1959b).

** Bazzania emarginata (Steph.) C.M.Cooke, Trans. Connecticut Acad. Arts 12 (1): 17, 1904 (Cooke 1904). Bas.: Mastigobryum didericianum var. emarginatum Steph., Hedwigia 24 (6): 249, 1885 (Stephani 1885b).

** Bazzania engelii Glenny, Fieldiana, Bot. (n.ser.) 47: 176, 2007 (Glenny and Bartlett 2007).

*** Bazzania erosa (Reinw., Blume et Nees) Trevis., Mem. Reale Ist. Lombardo Sci. (Ser. 3), C. Sci. Mat. 4 (13): 415, 1877 (Trevisan 1877). Bas.: Jungermannia erosa Reinw., Blume et Nees, Nova Acta Phys.-Med. Acad. Caes. Leop.-Carol. Nat. Cur. 12 (1): 230, 1824 [1825] (Reinwardt et al. 1824a).

** Bazzania erosa var. pulopenangensis (Lindenb. et Gottsche) Schiffn., Consp. Hepat. Arch. Ind.: 157, 1898 (Schiffner 1898b). Bas.: Mastigobryum erosum δ pulopenangense Lindenb. et Gottsche, Sp. Hepat. (Lindenberg) 8-11: 99, 1851 (Lindenberg and Gottsche 1851b).

*** Bazzania exempta J.J.Engel, J. Hattori Bot. Lab. 99: 197, 2006 (Engel 2006c).

*** Bazzania falcata (Lindenb.) Trevis., Mem. Reale Ist. Lombardo Sci. (Ser. 3), C. Sci. Mat. 4 (13): 415, 1877 (Trevisan 1877). Bas.: Mastigobryum falcatum Lindenb., Syn. Hepat. 2: 231, 1845 (Gottsche et al. 1845a).

** Bazzania falcifolia (Steph.) H.A.Mill., Phytologia 47 (4): 320, 1981 (Miller 1981). Bas.: Mastigobryum falcifolium Steph., Akad. Wiss. Wien, Math.-Naturwiss. Kl., Denkschr. 88: 33, 1911 (Stephani 1911d).

* Bazzania fallax (Sande Lac.) Schiffn., Consp. Hepat. Arch. Ind.: 158, 1898 (Schiffner 1898b). Bas.: Mastigobryum fallax Sande Lac., Ann. Mus. Bot. Lugduno-Batavi 1: 304, 1864 (Sande Lacoste 1864).

*** Bazzania fasciculata (Steph.) Meagher, Australas. Bryol. Newslett. 46: 6, 2002 (Meagher 2002). Bas.: Mastigobryum fasciculatum Steph., Bull. Herb. Boissier (sér. 2) 8 (10): 748 (432), 1908 (Stephani 1908c).

** Bazzania fauriana (Steph.) S.Hatt., Bot. Mag. (Tokyo) 59 (693/694): 27, 1946 (Hattori 1946). Bas.: Mastigobryum faurianum Steph., Bull. Herb. Boissier (sér. 2) 8 (11): 843 (467), 1908 (Stephani 1908a).

** Bazzania filiformis Steph., Hedwigia 28 (2): 131, 1889 (Stephani 1889a).

*** Bazzania flaccida (Dumort.) Grolle, Lindbergia 1 (3/4): 200, 1972 [1973] (Grolle 1972b). Bas.: Pleuroschisma flaccidum Dumort., Syll. Jungerm. Europ.: 71, 1831 (Dumortier 1831).

** Bazzania flavescens (Sande Lac. ex Steph.) Schiffn., Consp. Hepat. Arch. Ind.: 158, 1898 (Schiffner 1898b). Bas.: Mastigobryum flavescens Sande Lac. ex Steph., Hedwigia 25 (1): 6, 1886 (Stephani 1886e).

** Bazzania fleischeri (Steph.) Abeyw., Ceylon J. Sci., Biol. Sci. 2 (1): 45, 1959 (Abeywickrama 1959). Bas.: Mastigobryum fleischeri Steph., Bull. Herb. Boissier (sér. 2) 8 (10): 773 (457), 1908 (Stephani 1908c).

* Bazzania francana (Steph.) N.Kitag., J. Hattori Bot. Lab. 36: 446, 1972 [1973] (Kitagawa 1972). Bas.: Mastigobryum francanum Steph., Sp. Hepat. (Stephani) 6: 463, 1924 (Stephani 1924).

** Bazzania friabilis N.Kitag. et T.Kodama, J. Hattori Bot. Lab. 39: 67, 1975 (Kitagawa and Kodama 1975b).

*** Bazzania fuhreri Meagher, Nova Hedwigia 92 (3/4): 488, 2011 (Meagher 2011).

** Bazzania fuscescens A.Evans, Pap. Michigan Acad. Sci. 17: 85, 1932 [1933] (Evans 1932b).

*** Bazzania gamscottii Meagher, Nova Hedwigia 92 (3/4): 492, 2011 (Meagher 2011).

*** Bazzania gedeana (Steph.) Meijer, Blumea 10 (2): 378, 1960 (Meijer 1960). Bas.: Mastigobryum gedeanum Steph., Sp. Hepat. (Stephani) 3: 540, 1909 (Stephani 1909a).

*** Bazzania gracilis (Hampe et Gottsche) Steph., Hedwigia 27 (11/12): 279, 1888 (Stephani 1888c). Bas.: Mastigobryum gracile Hampe et Gottsche, Linnaea 25 (3): 346, 1852 [1853] (Hampe and Gottsche 1852).

** Bazzania grandiretis (Steph.) Herzog, Trans. Brit. Bryol. Soc. 1 (4): 298, 1950 (Herzog 1950a). Bas.: Mastigobryum grandirete Steph., Bull. Herb. Boissier (sér. 2) 8 (10): 747 (431), 1908 (Stephani 1908c).

** Bazzania griffithiana (Steph.) Mizut., J. Hattori Bot. Lab. 30: 82, 1967 (Mizutani 1967). Bas.: Mastigobryum griffithianum Steph., Bull. Herb. Boissier (sér. 2) 8 (12): 959 (509), 1908 (Stephani 1908b).

** Bazzania gunniana (Steph.) H.A.Mill., Phytologia 47 (4): 320, 1981 (Miller 1981). Bas.: Mastigobryum gunnianum Steph., J. & Proc. Roy. Soc. New South Wales 48 (1/2): 123, 1914 (Stephani and Watts 1914).

** Bazzania hainanensis L.P.Zhou et Li Zhang, J. Bryol. 34 (1): 25, 2012 (Zhou et al. 2012).

** Bazzania halconiensis (Steph.) N.Kitag., J. Hattori Bot. Lab. 36: 447, 1972 [1973] (Kitagawa 1972). Bas.: Mastigobryum halconiense Steph., Bull. Herb. Boissier (sér. 2) 8 (10): 759 (443), 1908 (Stephani 1908c).

** Bazzania hamatifolia (Steph.) H.A.Mill., Phytologia 47 (4): 320, 1981 (Miller 1981). Bas.: Mastigobryum hamatifolium Steph., Bull. Herb. Boissier (sér. 2) 8 (11): 862 (486), 1908 (Stephani 1908a).

** Bazzania harpago (De Not.) Schiffn., Consp. Hepat. Arch. Ind.: 159, 1898 (Schiffner 1898b). Bas.: Mastigobryum harpago De Not., Epat. Borneo: 29, 1874 (De Notaris 1874).

** Bazzania hebridensis (Steph.) H.A.Mill., Phytologia 47 (4): 320, 1981 (Miller 1981). Bas.: Mastigobryum hebridense Steph., J. & Proc. Roy. Soc. New South Wales 48 (1/2): 124, 1914 (Stephani and Watts 1914).

** Bazzania herminieri (Gottsche ex Steph.) Pagán, Bryologist 45 (4): 90, 1942 (Pagán 1942b). Bas.: Mastigobryum herminieri Gottsche ex Steph., Hedwigia 25 (1): 8, 1886 (Stephani 1886e).

** Bazzania herzogiana Meijer, Blumea 10 (2): 371, 1960 (Meijer 1960). *Nom. nov. pro Bazzania remotifolia* Herzog, Trans. Brit. Bryol. Soc. 1 (4): 304, 1950 (Herzog 1950a), *nom. illeg*.

** Bazzania heterostipa (Steph.) Fulford, Bull. Torrey Bot. Club 86 (6): 410, 1959 (Fulford 1959a). Bas.: Mastigobryum heterostipum Steph., Sp. Hepat. (Stephani) 3: 532, 1909 (Stephani 1909a).

*** Bazzania himalayana (Mitt.) Schiffn., Österr. Bot. Z. 49 (4): 132, 1899 (Schiffner 1899b). Bas.: Mastigobryum himalayanum Mitt., J. Proc. Linn. Soc., Bot. 5 (18): 105, 1860 [1861] (Mitten 1860c).

*** Bazzania hochstetteri (Reichardt) E.A.Hodgs., Trans. Roy. Soc. New Zealand 82 (1): 11, 1954 (Hodgson 1954). Bas.: Mastigobryum hochstetteri Reichardt, Verh. K.K. Zool.-Bot. Ges. Wien 16: 959, 1866 (Reichardt 1866).

*** Bazzania hookeri (Lindenb.) Trevis., Mem. Reale Ist. Lombardo Sci. (Ser. 3), C. Sci. Mat. 4 (13): 414, 1877 (Trevisan 1877). Bas.: Mastigobryum hookeri Lindenb., Syn. Hepat. 2: 226, 1845 (Gottsche et al. 1845a).

** Bazzania horridula Schiffn., Nova Acta Acad. Caes. Leop.-Carol. German. Nat. Cur. 60 (2): 258, 1893 (Schiffner 1893a).

** Bazzania inaequabilis Steph., Trans. Connecticut Acad. Arts 12 (1): 21, 1904 (Cooke 1904).

** Bazzania inaequitexta Steph., Hedwigia 32 (4): 208, 1893 (Stephani 1893c).

** Bazzania incrassata (Steph.) N.Kitag., J. Hattori Bot. Lab. 36: 448, 1972 [1973] (Kitagawa 1972). Bas.: Mastigobryum incrassatum Steph., Sp. Hepat. (Stephani) 6: 469, 1924 (Stephani 1924).

** Bazzania indica (Gottsche et Lindenb.) Trevis., Mem. Reale Ist. Lombardo Sci. (Ser. 3), C. Sci. Mat. 4 (13): 415, 1877 (Trevisan 1877). Bas.: Mastigobryum indicum Gottsche et Lindenb., Syn. Hepat. 2: 230, 1845 (Gottsche et al. 1845a).

** Bazzania indigenarum (Steph.) N.Kitag., Bull. Nara Univ. Educ., B 26 (2): 82, 1977 (Kitagawa 1977). Bas.: Mastigobryum indigenarum Steph., J. & Proc. Roy. Soc. New South Wales 48 (1/2): 124, 1914 (Stephani and Watts 1914).

** Bazzania insignis (De Not.) Trevis., Mem. Reale Ist. Lombardo Sci. (Ser. 3), C. Sci. Mat. 4 (13): 414, 1877 (Trevisan 1877). Bas.: Mastigobryum insigne De Not., Epat. Borneo: 26, 1874 (De Notaris 1874).

* Bazzania intermedia (Gottsche et Lindenb.) Trevis., Mem. Reale Ist. Lombardo Sci. (Ser. 3), C. Sci. Mat. 4 (13): 415, 1877 (Trevisan 1877). Bas.: Mastigobryum intermedium Gottsche et Lindenb., Sp. Hepat. (Lindenberg) 8-11: 82, 1851 (Lindenberg and Gottsche 1851b).

** Bazzania intermedia var. sarawakiana (De Not.) Schiffn., Consp. Hepat. Arch. Ind.: 162, 1898 (Schiffner 1898b). Bas.: Mastigobryum intermedium var. sarawakianum De Not., Epat. Borneo: 32, 1874 (De Notaris 1874).

** Bazzania involuta (Mont.) Trevis., Mem. Reale Ist. Lombardo Sci. (Ser. 3), C. Sci. Mat. 4 (13): 414, 1877 (Trevisan 1877). Bas.: Herpetium involutum Mont., Ann. Sci. Nat. Bot. (sér. 2) 19: 253, 1843 (Montagne 1843).

** Bazzania involuta var. submutica (Lindenb. et Gottsche) J.J.Engel et G.L.Merr., Bryologist 97 (3): 314, 1994 (Engel and Smith Merrill 1994). Bas.: Mastigobryum novae-hollandiae γ3 submuticum Lindenb. et Gottsche, Sp. Hepat. (Lindenberg) 8-11: 33, 1851 (Lindenberg and Gottsche 1851b).

** Bazzania involutiformis (De Not.) Trevis., Mem. Reale Ist. Lombardo Sci. (Ser. 3), C. Sci. Mat. 4 (13): 414, 1877 (Trevisan 1877). Bas.: Mastigobryum involutiforme De Not., Epat. Borneo: 28, 1874 (De Notaris 1874).

** Bazzania irregularis (Steph.) Schiffn., Consp. Hepat. Arch. Ind.: 163, 1898 (Schiffner 1898b). Bas.: Mastigobryum irregulare Steph., Hedwigia 25 (4): 133, 1886 (Stephani 1886b).

*** Bazzania jamaicensis (Lehm. et Lindenb.) Trevis., Mem. Reale Ist. Lombardo Sci. (Ser. 3), C. Sci. Mat. 4 (13): 414, 1877 (Trevisan 1877). Bas.: Herpetium jamaicense Lehm. et Lindenb., Nov. Stirp. Pug. 7: 7, 1838 (Lehmann 1838).

** Bazzania japonica (Sande Lac.) Lindb., Acta Soc. Sci. Fenn. 10: 224, 1872 [1873] (Lindberg 1872b). Bas.: Mastigobryum japonicum Sande Lac., Ann. Mus. Bot. Lugduno-Batavi 1: 303, 1864 (Sande Lacoste 1864).

** Bazzania japonica var. sumatrana Herzog, Ann. Naturhist. Mus. Wien 53 (1): 366, 1942 [1943] (Herzog 1942b).

** Bazzania javanica (Sande Lac.) Schiffn., Consp. Hepat. Arch. Ind.: 163, 1898 (Schiffner 1898b). Bas.: Mastigobryum javanicum Sande Lac., Ned. Kruidk. Arch. 3: 418, 1854 [1855] (Sande Lacoste 1854).

** Bazzania kernii Steph., Hedwigia 32 (4): 208, 1893 (Stephani 1893c).

** Bazzania kokawana N.Kitag. et T.Kodama, J. Jap. Bot. 50 (1): 11, 1975 (Kitagawa and Kodama 1975a).

** Bazzania latifolia Steph., Hedwigia 32 (4): 209, 1893 (Stephani 1893c).

* Bazzania lehmanniana (Lindenb.) Trevis., Mem. Reale Ist. Lombardo Sci. (Ser. 3), C. Sci. Mat. 4 (13): 414, 1877 (Trevisan 1877). Bas.: Mastigobryum lehmannianum Lindenb., Syn. Hepat. 2: 223, 1845 (Gottsche et al. 1845a). ^112^

** Bazzania leratii (Beauverd) H.A.Mill., Phytologia 47 (4): 320, 1981 (Miller 1981). Bas.: Mastigobryum leratii Beauverd, Sp. Hepat. (Stephani) 6: 477, 1924 (Stephani 1924).

** Bazzania lessonii (Steph.) H.A.Mill., Phytologia 47 (4): 321, 1981 (Miller 1981). Bas.: Mastigobryum lessonii Steph., Sp. Hepat. (Stephani) 3: 531, 1909 (Stephani 1909a).

** Bazzania levieri (Steph.) N.Kitag., J. Hattori Bot. Lab. 36: 448, 1972 [1973] (Kitagawa 1972). Bas.: Mastigobryum levieri Steph., Bull. Herb. Boissier (sér. 2) 8 (12): 944 (494), 1908 (Stephani 1908b).

** Bazzania liebmaniana (Lindenb. et Gottsche) Trevis., Mem. Reale Ist. Lombardo Sci. (Ser. 3), C. Sci. Mat. 4 (13): 414, 1877 (Trevisan 1877). Bas.: Mastigobryum liebmanianum Lindenb. et Gottsche, Syn. Hepat. 5: 719, 1847 (Gottsche et al. 1847).

** Bazzania linearis Herzog, Trans. Brit. Bryol. Soc. 1 (4): 303, 1950 (Herzog 1950a).

** Bazzania linguiformis (Sande Lac.) Trevis., Mem. Reale Ist. Lombardo Sci. (Ser. 3), C. Sci. Mat. 4 (13): 414, 1877 (Trevisan 1877). Bas.: Mastigobryum linguiforme Sande Lac., Plagiochila Sandei: 8, 1856 (Sande Lacoste 1856c).

*** Bazzania longa (Nees) Trevis., Mem. Reale Ist. Lombardo Sci. (Ser. 3), C. Sci. Mat. 4 (13): 415, 1877 (Trevisan 1877). Bas.: Jungermannia longa Nees, Linnaea 6 (4): 623, 1831 (Nees 1831).

** Bazzania longa var. papillata (Steph.) Fulford, Ann. Cryptog. Phytopathol. 3: 94, 1946 (Fulford 1946). Bas.: Mastigobryum papillatum Steph., Sp. Hepat. (Stephani) 3: 526, 1909 (Stephani 1909a).

*** Bazzania longicaulis (Sande Lac.) Schiffn., Consp. Hepat. Arch. Ind.: 165, 1898 (Schiffner 1898b). Bas.: Mastigobryum longicaule Sande Lac., Ann. Mus. Bot. Lugduno-Batavi 1: 303, 1864 (Sande Lacoste 1864).

** Bazzania longicaulis var. latiareata Herzog, Memoranda Soc. Fauna Fl. Fennica 26: 46, 1950 [1951] (Herzog 1950b).

*** Bazzania longistipula (Lindenb.) Trevis., Mem. Reale Ist. Lombardo Sci. (Ser. 3), C. Sci. Mat. 4 (13): 415, 1877 (Trevisan 1877). Bas.: Mastigobryum longistipulum Lindenb., Syn. Hepat. 2: 228, 1845 (Gottsche et al. 1845a).

*** Bazzania loricata (Reinw., Blume et Nees) Trevis., Mem. Reale Ist. Lombardo Sci. (Ser. 3), C. Sci. Mat. 4 (13): 414, 1877 (Trevisan 1877). Bas.: Jungermannia loricata Reinw., Blume et Nees, Nova Acta Phys.-Med. Acad. Caes. Leop.-Carol. Nat. Cur. 12 (1): 233, 1824 [1825] (Reinwardt et al. 1824a).

** Bazzania lowii (Sande Lac. ex Steph.) Schiffn., Consp. Hepat. Arch. Ind.: 166, 1898 (Schiffner 1898b). Bas.: Mastigobryum lowii Sande Lac. ex Steph., Hedwigia 25 (5): 204, 1886 (Stephani 1886f).

** Bazzania luzonensis (Steph.) Del Ros., Philipp. J. Sci. 100 (3/4): 231, 1971 (Del Rosario 1971). Bas.: Mastigobryum luzonense Steph., Sp. Hepat. (Stephani) 6: 472, 1924 (Stephani 1924).

** Bazzania macgregorii Steph., Hedwigia 32 (4): 210, 1893 (Stephani 1893c).

** Bazzania macrostipula Fulford, Bull. Torrey Bot. Club 86 (6): 407, 1959 (Fulford 1959a).

** Bazzania magna Horik., J. Sci. Hiroshima Univ., Ser. B, Div. 2, Bot. 2: 197, 1934 (Horikawa 1934).

** Bazzania magnistipula N.Kitag., J. Hattori Bot. Lab. 47: 132, 1980 (Kitagawa 1980).

** Bazzania malaccensis (Steph.) Tixier, Gard. Bull. Singapore 25 (3): 342, 1971 (Tixier 1971). Bas.: Mastigobryum malaccense Steph., Bull. Herb. Boissier (sér. 2) 8 (12): 944 (494), 1908 (Stephani 1908b).

** Bazzania manillana (Gottsche ex Steph.) S.Hatt., Bot. Mag. (Tokyo) 64 (755/756): 113, 1951 (Hattori 1951c). Bas.: Mastigobryum manillanum Gottsche ex Steph., Hedwigia 25 (5): 204, 1886 (Stephani 1886f).

* Bazzania marginata (Steph.) N.Kitag., J. Hattori Bot. Lab. 36: 449, 1972 [1973] (Kitagawa 1972). Bas.: Mastigobryum marginatum Steph., Rev. Bryol. 35 (2): 31, 1908 (Stephani 1908l).

** Bazzania marginella (Herzog) N.Kitag. et T.Kodama, Bull. Osaka Mus. Nat. Hist. 27: 17, 1973 (Kitagawa and Kodama 1973). Bas.: Mastigobryum marginellum Herzog, Ann. Bryol. 5: 91, 1932 (Herzog 1932a).

* Bazzania mascarena (Steph.) Herzog, Bot. Not. 100 (4): 334, 1947 (Herzog 1947). Bas.: Mastigobryum mascarenum Steph., Hedwigia 25 (5): 205, 1886 (Stephani 1886f).

** Bazzania mayebarae S.Hatt., J. Hattori Bot. Lab. 19: 91, 1958 (Hattori and Mizutani 1958).

* Bazzania menzelii E.D.Cooper, Phytotaxa 97 (2): 52, 2013 (Cooper et al. 2013). *Nom. nov. pro Acromastigum emarginatum* Herzog, Trans. Brit. Bryol. Soc. 1 (4): 309, 1950 (Herzog 1950a).

** Bazzania merrillana (Steph.) Inoue ex Bonner, Index Hepat. 3: 359, 1963 (Bonner 1963). Bas.: Mastigobryum merrillanum Steph., Bull. Herb. Boissier (sér. 2) 8 (12): 944 (494), 1908 (Stephani 1908b).

** Bazzania minuta (Austin) A.Evans, Trans. Connecticut Acad. Arts 8 (15): 255, 1891 (Evans 1891). Bas.: Mastigobryum minutum Austin, Bull. Torrey Bot. Club 5 (3): 17, 1874 (Austin 1874).

** Bazzania minutidens (Steph.) Inoue et H.A.Mill., Bull. Natl. Sci. Mus. Tokyo (n.ser.) 8 (2): 142, 1965 (Inoue and Miller 1965). Bas.: Mastigobryum minutidens Steph., Sp. Hepat. (Stephani) 6: 474, 1924 (Stephani 1924).

** Bazzania minutiserra (Steph.) N.Kitag., Bull. Nara Univ. Educ., B 26 (2): 77, 1977 (Kitagawa 1977). Bas.: Mastigobryum minutiserrum Steph., Sp. Hepat. (Stephani) 6: 474, 1924 (Stephani 1924).

** Bazzania missionum (Herzog) Jovet-Ast, Rev. Bryol. Lichénol. 20 (1/2): 96, 1951 (Jovet-Ast 1951). Bas.: Mastigobryum missionum Herzog, Ann. Bryol. 5: 93, 1932 (Herzog 1932a).

** Bazzania mittenii (Steph.) Steph., Hedwigia 28 (2): 132, 1889 (Stephani 1889a). Bas.: Mastigobryum mittenii Steph., Hedwigia 25 (6): 245, 1886 (Stephani 1886c).

*** Bazzania monilinervis (Lehm. et Lindenb.) Trevis., Mem. Reale Ist. Lombardo Sci. (Ser. 3), C. Sci. Mat. 4 (13): 414, 1877 (Trevisan 1877). Bas.: Jungermannia monilinervis Lehm. et Lindenb., Nov. Stirp. Pug. 4: 56, 1832 (Lehmann 1832).

** Bazzania morokensis (Steph.) Grolle, J. Hattori Bot. Lab. 31: 1, 1968 (Grolle 1968a). Bas.: Mastigobryum morokense Steph., Bull. Herb. Boissier (sér. 2) 8 (10): 765 (449), 1908 (Stephani 1908c).

*** Bazzania nitida (F.Weber) Grolle, Rev. Bryol. Lichénol. 29 (3/4): 210, 1960 [1961] (Grolle 1960a). Bas.: Jungermannia nitida F.Weber, Hist. Musc. Hepat. Prodr.: 43, 1815 (Weber 1815).

*** Bazzania novae-zelandiae (Mitt.) Besch. et C.Massal., Miss. sci. Cape Horn, Bot. 5: 233, 1889 (Bescherelle and Massalongo 1889). Bas.: Mastigobryum novae-zelandiae Mitt., Bot. antarct. voy. II (Fl. Nov.-Zel. 2): 148, 1854 (Mitten 1854).

** Bazzania nudicaulis A.Evans, Bryologist 26 (6): 62, 1923 [1924] (Evans 1923b).

** Bazzania nuuanuensis C.M.Cooke, Trans. Connecticut Acad. Arts 12 (1): 15, 1904 (Cooke 1904).

** Bazzania obcuneata (Steph.) H.A.Mill., Phytologia 47 (4): 321, 1981 (Miller 1981). Bas.: Mastigobryum obcuneatum Steph., Bull. Herb. Boissier (sér. 2) 8 (11): 863 (487), 1908 (Stephani 1908a).

** Bazzania obtusata (Mitt.) Abeyw., Ceylon J. Sci., Biol. Sci. 2 (1): 45, 1959 (Abeywickrama 1959). Bas.: Mastigobryum obtusatum Mitt., J. Proc. Linn. Soc., Bot. 5 (18): 106, 1860 [1861] (Mitten 1860c).

** Bazzania okaritana Meagher et Glenny, J. Bryol. 29 (1): 60, 2007 (Meagher and Glenny 2007).

** Bazzania orbanii Pócs, Acta Biol. Pl. Agr. 1: 16, 2010 [2011] (Pócs 2010c).

** Bazzania ovistipula (Steph.) Abeyw., Ceylon J. Sci., Biol. Sci. 2 (1): 45, 1959 (Abeywickrama 1959). Bas.: Mastigobryum ovistipulum Steph., Bull. Herb. Boissier (sér. 2) 8 (10): 760 (444), 1908 (Stephani 1908c).

** Bazzania pallidevirens (Steph.) Fulford, Ann. Cryptog. Phytopathol. 3: 42, 1946 (Fulford 1946). Bas.: Mastigobryum pallidevirens Steph., Bull. Herb. Boissier (sér. 2) 8 (11): 849 (473), 1908 (Stephani 1908a).

** Bazzania papillosa S.W.Arnell, Svensk Bot. Tidskr. 59 (1): 67, 1965 (Arnell 1965).

** Bazzania paradoxa (Sande Lac.) Steph., Bot. Jahrb. Syst. 23 (1/2, 3): 307, 1896 (Stephani 1896a). Bas.: Mastigobryum paradoxum Sande Lac., Ned. Kruidk. Arch. 3: 419, 1854 [1855] (Sande Lacoste 1854).

*** Bazzania parisii (Steph.) N.Kitag., J. Hattori Bot. Lab. 47: 135, 1980 (Kitagawa 1980). Bas.: Mastigobryum parisii Steph., Bull. Herb. Boissier (sér. 2) 8 (10): 769 (453), 1908 (Stephani 1908c).

** Bazzania parvitexta Steph., Hedwigia 32 (4): 211, 1893 (Stephani 1893c).

** Bazzania patens (Mont.) Trevis., Mem. Reale Ist. Lombardo Sci. (Ser. 3), C. Sci. Mat. 4 (13): 414, 1877 (Trevisan 1877). Bas.: Herpetium patens Mont., Ann. Sci. Nat. Bot. (sér. 2) 20: 295, 1844 (Montagne 1844b).

** Bazzania patentistipa (Sande Lac.) Schiffn., Consp. Hepat. Arch. Ind.: 168, 1898 (Schiffner 1898b). Bas.: Mastigobryum patentistipum Sande Lac., Ann. Mus. Bot. Lugduno-Batavi 1: 302, 1864 (Sande Lacoste 1864).

** Bazzania paucidens (Steph.) H.A.Mill., Phytologia 47 (4): 321, 1981 (Miller 1981). Bas.: Mastigobryum paucidens Steph., Bull. Herb. Boissier (sér. 2) 8 (11): 860 (484), 1908 (Stephani 1908a).

** Bazzania pearsonii Steph., Hedwigia 32 (4): 212, 1893 (Stephani 1893c).

*** Bazzania pectinata (Lindenb. et Gottsche) Schiffn., Nova Acta Acad. Caes. Leop.-Carol. German. Nat. Cur. 60 (2): 259, 1893 (Schiffner 1893a). Bas.: Mastigobryum pectinatum Lindenb. et Gottsche, Sp. Hepat. (Lindenberg) 8-11: 84, 1851 (Lindenberg and Gottsche 1851b).

** Bazzania perfalcata N.Kitag., J. Hattori Bot. Lab. 47: 135, 1980 (Kitagawa 1980).

** Bazzania perrotana E.W.Jones, J. Bryol. 8 (3): 310, 1975 (Jones 1975). *Nom. nov. pro Mastigobryum perrotanum* Steph., Sp. Hepat. (Stephani) 6: 476, 1924 (Stephani 1924), *nom. illeg*.

** Bazzania peruviana (Nees) Trevis., Mem. Reale Ist. Lombardo Sci. (Ser. 3), C. Sci. Mat. 4 (13): 414, 1877 (Trevisan 1877). Bas.: Mastigobryum peruvianum Nees, Syn. Hepat. 2: 220, 1845 (Gottsche et al. 1845a).

*** Bazzania phyllobola Spruce, Trans. & Proc. Bot. Soc. Edinburgh 15: 372, 1885 (Spruce 1885).

*** Bazzania placophylla (Taylor) Grolle, Rev. Bryol. Lichénol. 27 (1/2): 54, 1958 (Grolle 1958). Bas.: Jungermannia placophylla Taylor, London J. Bot. 5: 276, 1846 (Taylor 1846a).

** Bazzania platycnema (Schwägr. ex Steph.) H.A.Mill., Bryologist 63 (2): 121, 1960 (Miller 1960). Bas.: Mastigobryum platycnemum Schwägr. ex Steph., Bull. Herb. Boissier (sér. 2) 8 (10): 776 (460), 1908 (Stephani 1908c).

** Bazzania pompeana (Sande Lac.) Mitt., Trans. Linn. Soc. London, Bot. 3 (3): 200, 1891 (Mitten 1891). Bas.: Mastigobryum pompeanum Sande Lac., Ann. Mus. Bot. Lugduno-Batavi 1: 304, 1864 (Sande Lacoste 1864).

*** Bazzania praerupta (Reinw., Blume et Nees) Trevis., Mem. Reale Ist. Lombardo Sci. (Ser. 3), C. Sci. Mat. 4 (13): 414, 1877 (Trevisan 1877). Bas.: Jungermannia praerupta Reinw., Blume et Nees, Nova Acta Phys.-Med. Acad. Caes. Leop.-Carol. Nat. Cur. 12 (1): 229, 1824 [1825] (Reinwardt et al. 1824a).

* Bazzania praerupta var. obliquata (Nees) Schiffn., Consp. Hepat. Arch. Ind.: 170, 1898 (Schiffner 1898b). Bas.: Jungermannia obliquata Nees, Enum. Pl. Crypt. Javae: 62, 1830 (Nees 1830).

** Bazzania praerupta var. thermalis Schiffn., Arch. Hydrobiol., suppl. 21 (3/4): 396, 1955 (Schiffner 1955).

** Bazzania pseudovittata N.Kitag. et T.Kodama, J. Hattori Bot. Lab. 39: 69, 1975 (Kitagawa and Kodama 1975b).

** Bazzania pusilla (Mitt.) Steph., Bot. Jahrb. Syst. 23 (1/2, 3): 307, 1896 (Stephani 1896a). Bas.: Mastigobryum pusillum Mitt., Fl. vit.: 406, 1871 [1873] (Mitten 1871).

** Bazzania pycnophylla (Taylor) Trevis., Mem. Reale Ist. Lombardo Sci. (Ser. 3), C. Sci. Mat. 4 (13): 414, 1877 (Trevisan 1877). Bas.: Mastigobryum pycnophyllum Taylor, London J. Bot. 5: 371, 1846 (Taylor 1846b).

** Bazzania quadratistipula H.A.Mill., Phytologia 47 (4): 321, 1981 (Miller 1981). *Nom. nov. pro Mastigobryum quadratum* Steph., Sp. Hepat. (Stephani) 6: 477, 1924 (Stephani 1924), *nom. illeg*.

** Bazzania rabenhorstii (Steph.) Abeyw., Ceylon J. Sci., Biol. Sci. 2 (1): 45, 1959 (Abeywickrama 1959). Bas.: Mastigobryum rabenhorstii Steph., Bull. Herb. Boissier (sér. 2) 8 (10): 774 (458), 1908 (Stephani 1908c).

** Bazzania recurva (Mont.) Trevis., Mem. Reale Ist. Lombardo Sci. (Ser. 3), C. Sci. Mat. 4 (13): 414, 1877 (Trevisan 1877). Bas.: Herpetium recurvum Mont., Ann. Sci. Nat. Bot. (sér. 2) 19: 253, 1843 (Montagne 1843).

* Bazzania recurva var. major (Sande Lac.) Schiffn., Consp. Hepat. Arch. Ind.: 172, 1898 (Schiffner 1898b). Bas.: Mastigobryum recurvum var. majus Sande Lac., Ann. Mus. Bot. Lugduno-Batavi 1: 304, 1864 (Sande Lacoste 1864).

** Bazzania reflexa (Gottsche) Steph., Rev. Bryol. 18 (4): 56, 1891 (Renauld and Cardot 1891). Bas.: Mastigobryum reflexum Gottsche, Abh. Naturwiss. Vereins Bremen 7: 347, 1882 (Gottsche 1882).

** Bazzania reinwardtii (Sande Lac.) Schiffn., Consp. Hepat. Arch. Ind.: 172, 1898 (Schiffner 1898b). Bas.: Mastigobryum reinwardtii Sande Lac., Pl. Ind. Batav. Orient.: 22, 1856 (Sande Lacoste 1856a).

** Bazzania renistipula Steph., Hedwigia 32 (4): 212, 1893 (Stephani 1893c).

** Bazzania revoluta (Steph.) N.Kitag., J. Hattori Bot. Lab. 36: 450, 1972 [1973] (Kitagawa 1972). Bas.: Mastigobryum revolutum Steph., Bull. Herb. Boissier (sér. 2) 8 (12): 961 (511), 1908 (Stephani 1908b).

*** Bazzania rimosa Meagher, Nova Hedwigia 86 (3/4): 489, 2008 (Meagher 2008).

** Bazzania roccatii Gola, Ann. Bot. (Rome) 6 (2): 273, 1907 (Gola 1907).

*** Bazzania roraimensis (Steph.) Fulford, Ann. Cryptog. Phytopathol. 3: 27, 1946 (Fulford 1946). Bas.: Mastigobryum roraimense Steph., Trans. Linn. Soc. London, Bot. 6 (1): 97, 1901 (Stephani 1901e).

** Bazzania sandvicensis (Gottsche ex Steph.) Steph., Bull. Herb. Boissier 5 (10): 841, 1897 (Stephani 1897c). Bas.: Mastigobryum sandvicense Gottsche ex Steph., Hedwigia 25 (5): 207, 1886 (Stephani 1886f).

** Bazzania sauropoda Meagher, Austrobaileya 7 (1): 129, 2005 (Meagher 2005a).

*** Bazzania scalaris Meagher, Telopea 11 (3): 247, 2006 (Meagher 2006).

*** Bazzania schlimiana (Gottsche) Fulford, Bull. Torrey Bot. Club 86 (6): 401, 1959 (Fulford 1959a). Bas.: Mastigobryum schlimianum Gottsche, Ann. Sci. Nat. Bot. (sér. 5) 1: 140, 1864 (Gottsche 1864).

** Bazzania schultze-motelii N.Kitag., J. Hattori Bot. Lab. 47: 138, 1980 (Kitagawa 1980).

** Bazzania schusterana N.Kitag., J. Hattori Bot. Lab. 47: 139, 1980 (Kitagawa 1980).

*** Bazzania schwaneckiana (Hampe et Gottsche) Trevis., Mem. Reale Ist. Lombardo Sci. (Ser. 3), C. Sci. Mat. 4 (13): 414, 1877 (Trevisan 1877). Bas.: Mastigobryum schwaneckianum Hampe et Gottsche, Linnaea 25 (3): 345, 1852 [1853] (Hampe and Gottsche 1852).

* Bazzania scutigera (Nees et Mont.) Trevis., Mem. Reale Ist. Lombardo Sci. (Ser. 3), C. Sci. Mat. 4 (13): 414, 1877 (Trevisan 1877). Bas.: Herpetium scutigerum Nees et Mont., Ann. Sci. Nat. Bot. (sér. 2) 9: 44, 1838 (Montagne 1838).

** Bazzania semicordata (Lindenb. et Gottsche) Kuntze, Revis. Gen. Pl. 2: 832, 1891 (Kuntze 1891). Bas.: Mastigobryum semicordatum Lindenb. et Gottsche, Syn. Hepat. 5: 720, 1847 (Gottsche et al. 1847).

* Bazzania serpentina (Nees) Trevis., Mem. Reale Ist. Lombardo Sci. (Ser. 3), C. Sci. Mat. 4 (13): 415, 1877 (Trevisan 1877). Bas.: Jungermannia serpentina Nees, Enum. Pl. Crypt. Javae: 62, 1830 (Nees 1830). ^113^

** Bazzania serrapiculata Inoue et H.A.Mill., Bull. Natl. Sci. Mus. Tokyo (n.ser.) 8 (2): 141, 1965 (Inoue and Miller 1965).

** Bazzania serrata Fulford, Bull. Torrey Bot. Club 86 (5): 321, 1959 (Fulford 1959b).

** Bazzania serrulatoides Horik., J. Sci. Hiroshima Univ., Ser. B, Div. 2, Bot. 2: 200, 1934 (Horikawa 1934).

* Bazzania sikkimensis (Steph.) Herzog, Ann. Bryol. 12: 78, 1939 (Herzog 1939b). Bas.: Mastigobryum sikkimense Steph., Hedwigia 44 (2): 73, 1905 (Stephani 1905h). ^114^

** Bazzania spinosa S.Okamura, J. Coll. Sci. Imp. Univ. Tokyo 38 (4): 2, 1916 (Okamura 1916).

** Bazzania spiralis (Reinw., Blume et Nees) Meijer, Blumea 10 (2): 381, 1960 (Meijer 1960). Bas.: Jungermannia spiralis Reinw., Blume et Nees, Nova Acta Phys.-Med. Acad. Caes. Leop.-Carol. Nat. Cur. 12 (1): 231, 1824 [1825] (Reinwardt et al. 1824a).

** Bazzania spruceana Steph., Hedwigia 32 (4): 213, 1893 (Stephani 1893c).

** Bazzania squarrosa (Steph.) H.A.Mill., Phytologia 47 (4): 321, 1981 (Miller 1981). Bas.: Mastigobryum squarrosum Steph., Bull. Herb. Boissier (sér. 2) 8 (10): 776 (460), 1908 (Stephani 1908c).

*** Bazzania stolonifera (Sw.) Trevis., Mem. Reale Ist. Lombardo Sci. (Ser. 3), C. Sci. Mat. 4 (13): 415, 1877 (Trevisan 1877). Bas.: Jungermannia stolonifera Sw., Prodr. (Swartz): 144, 1788 (Swartz 1788).

* Bazzania stolonifera var. granatensis (Gottsche) Fulford, Ann. Cryptog. Phytopathol. 3: 51, 1946 (Fulford 1946). Bas.: Mastigobryum stoloniferum var. granatense Gottsche, Ann. Sci. Nat. Bot. (sér. 5) 1: 141, 1864 (Gottsche 1864).

** Bazzania stresemannii (Herzog) N.Kitag., Bull. Nara Univ. Educ., B 28 (2): 77, 1979 (Kitagawa 1979a). Bas.: Mastigobryum stresemannii Herzog, Beih. Bot. Centralbl. 38 (2): 324, 1921 (Herzog 1921).

** Bazzania subacuta (Mitt.) Steph., Bot. Jahrb. Syst. 23 (1/2, 3): 307, 1896 (Stephani 1896a). Bas.: Mastigobryum subacutum Mitt., Fl. vit.: 406, 1871 [1873] (Mitten 1871).

** Bazzania subaequitexta (Steph.) N.Kitag., Bull. Nara Univ. Educ., B 28 (2): 81, 1979 (Kitagawa 1979a). Bas.: Mastigobryum subaequitextum Steph., Bull. Herb. Boissier (sér. 2) 8 (11): 859 (483), 1908 (Stephani 1908a).

** Bazzania subintegra (Steph.) L.Söderstr. et A.Hagborg, Phytotaxa 202 (1): 69, 2015 (Söderström et al. 2015c). Bas.: Mastigobryum subintegrum Steph., Bull. Herb. Boissier (sér. 2) 8 (10): 775 (459), 1908 (Stephani 1908c).

** Bazzania sublonga Fulford, Bull. Torrey Bot. Club 86 (5): 334, 1959 (Fulford 1959b).

** Bazzania subserrifolia (Beauverd) H.A.Mill., Phytologia 47 (4): 321, 1981 (Miller 1981). Bas.: Mastigobryum subserrifolium Beauverd, Sp. Hepat. (Stephani) 6: 480, 1924 (Stephani 1924).

** Bazzania subserrulata A.Evans, Pap. Michigan Acad. Sci. 17: 97, 1932 [1933] (Evans 1932b).

*** Bazzania subtilis (Sande Lac.) Trevis., Mem. Reale Ist. Lombardo Sci. (Ser. 3), C. Sci. Mat. 4 (13): 414, 1877 (Trevisan 1877). Bas.: Mastigobryum subtile Sande Lac., Ann. Mus. Bot. Lugduno-Batavi 1: 302, 1864 (Sande Lacoste 1864).

** Bazzania succulenta N.Kitag., J. Hattori Bot. Lab. 47: 141, 1980 (Kitagawa 1980).

** Bazzania sumatrana (Sande Lac. ex Steph.) Steph., Hedwigia 32 (4): 209, 1893 (Stephani 1893c). Bas.: Mastigobryum sumatranum Sande Lac. ex Steph., Hedwigia 25 (6): 234, 1886 (Stephani 1886c).

* Bazzania sumbavensis (Gottsche ex Steph.) Steph., Hedwigia 32 (4): 204, 1893 (Stephani 1893c). Bas.: Mastigobryum sumbavense Gottsche ex Steph., Hedwigia 25 (6): 236, 1886 (Stephani 1886c). ^115^

*** Bazzania taleana (Gottsche) Fulford, Ann. Cryptog. Phytopathol. 3: 54, 1946 (Fulford 1946). Bas.: Mastigobryum taleanum Gottsche, Mexik. Leverm.: 131, 1863 (Gottsche 1863).

*** Bazzania tayloriana (Mitt.) Kuntze, Revis. Gen. Pl. 2: 832, 1891 (Kuntze 1891). Bas.: Mastigobryum taylorianum Mitt., Bot. antarct. voy. II (Fl. Nov.-Zel. 2): 147, 1854 (Mitten 1854).

** Bazzania temariana (Steph.) H.A.Mill., Phytologia 47 (4): 321, 1981 (Miller 1981). Bas.: Mastigobryum temarianum Steph., Sp. Hepat. (Stephani) 3: 532, 1909 (Stephani 1909a).

*** Bazzania tessellata Meagher, Nova Hedwigia 92 (3/4): 492, 2011 (Meagher 2011).

** Bazzania tiaoloensis Mizut. et K.C.Chang, J. Hattori Bot. Lab. 60: 432, 1986 (Mizutani and Chang 1986).

*** Bazzania tricrenata (Wahlenb.) Lindb., Musci Fenn. Exsic., fasc. 2: [2 (adnot.)], 1872 (Brotherus 1872). Bas.: Jungermannia tricrenata Wahlenb., Fl. Carpat. Princ.: 364, 1814 (Wahlenberg 1814).

** Bazzania tricrenata var. fulfordiae W.S.Hong, Bryologist 91 (4): 331, 1988 (Hong 1988).

*** Bazzania tridens (Reinw., Blume et Nees) Trevis., Mem. Reale Ist. Lombardo Sci. (Ser. 3), C. Sci. Mat. 4 (13): 415, 1877 (Trevisan 1877). Bas.: Jungermannia tridens Reinw., Blume et Nees, Nova Acta Phys.-Med. Acad. Caes. Leop.-Carol. Nat. Cur. 12 (1): 228, 1824 [1825] (Reinwardt et al. 1824a).

*** Bazzania tridens var. assamica (Steph.) Pócs, J. Hattori Bot. Lab. 32: 86, 1969 (Pócs 1969). Bas.: Mastigobryum assamicum Steph., Hedwigia 24 (5): 216, 1885 (Stephani 1885a).

** Bazzania tridens var. cornutistipula (Steph.) Pócs, J. Hattori Bot. Lab. 32: 83, 1969 (Pócs 1969). Bas.: Mastigobryum cornutistipulum Steph., Rev. Bryol. 35 (2): 35, 1908 (Stephani 1908l).

*** Bazzania trilobata (L.) Gray, Nat. Arr. Brit. Pl. 1: 704, 1821 (Gray 1821). Bas.: Jungermannia trilobata L., Sp. Pl. 1: 1133, 1753 (Linnaeus 1753).

** Bazzania trilobata var. depauperata (Müll.Frib.) Grolle, Lindbergia 1 (3/4): 197, 1972 [1973] (Grolle 1972b). Bas.: Pleuroschisma trilobatum var. depauperatum Müll.Frib., Lebermoose 2 (18): 266, 1913 (Müller 1913b).

*** Bazzania uncigera (Reinw., Blume et Nees) Trevis., Mem. Reale Ist. Lombardo Sci. (Ser. 3), C. Sci. Mat. 4 (13): 415, 1877 (Trevisan 1877). Bas.: Jungermannia uncigera Reinw., Blume et Nees, Nova Acta Phys.-Med. Acad. Caes. Leop.-Carol. Nat. Cur. 12 (1): 230, 1824 [1825] (Reinwardt et al. 1824a).

** Bazzania uncigera var. brevifolia Herzog, Trans. Brit. Bryol. Soc. 1 (4): 301, 1950 (Herzog 1950a).

** Bazzania uncigera var. gibba (Sande Lac.) Meijer, Blumea 10 (2): 378, 1960 (Meijer 1960). Bas.: Mastigobryum gibbum Sande Lac., Plagiochila Sandei: 8, 1856 (Sande Lacoste 1856c).

** Bazzania undulata Herzog, Trans. Brit. Bryol. Soc. 1 (4): 306, 1950 (Herzog 1950a).

** Bazzania vietnamica Pócs, J. Hattori Bot. Lab. 32: 90, 1969 (Pócs 1969).

** Bazzania vitiana Mitt., Hedwigia 32 (4): 214, 1893 (Stephani 1893c).

*** Bazzania vittata (Gottsche) Trevis., Mem. Reale Ist. Lombardo Sci. (Ser. 3), C. Sci. Mat. 4 (13): 414, 1877 (Trevisan 1877). Bas.: Mastigobryum vittatum Gottsche, Syn. Hepat. 2: 216, 1845 (Gottsche et al. 1845a).

** Bazzania vittata var. luxurians (De Not.) Schiffn., Consp. Hepat. Arch. Ind.: 178, 1898 (Schiffner 1898b). Bas.: Mastigobryum vittatum var. luxurians De Not., Epat. Borneo: 29, 1874 (De Notaris 1874).

** Bazzania wallichiana (Lindenb.) Trevis., Mem. Reale Ist. Lombardo Sci. (Ser. 3), C. Sci. Mat. 4 (13): 415, 1877 (Trevisan 1877). Bas.: Mastigobryum wallichianum Lindenb., Syn. Hepat. 2: 229, 1845 (Gottsche et al. 1845a). ^116^

** Bazzania watanabei Inoue, J. Jap. Bot. 34 (9): 269, 1959 (Inoue 1959a).

* Bazzania wattsiana (Steph.) Meagher, Australas. Bryol. Newslett. 50: 8, 2005 (Meagher 2005b). Bas.: Mastigobryum wattsianum Steph., Bull. Herb. Boissier (sér. 2) 8 (11): 850 (474), 1908 (Stephani 1908a). ^117^

** Bazzania wiltensii (Sande Lac. ex Steph.) Schiffn., Consp. Hepat. Arch. Ind.: 180, 1898 (Schiffner 1898b). Bas.: Mastigobryum wiltensii Sande Lac. ex Steph., Hedwigia 25 (6): 237, 1886 (Stephani 1886c).

** Bazzania wooroonooran Meagher, Nova Hedwigia 100 (3/4): 549, 2015 (Meagher 2015).

** Bazzania wrightii (Gottsche ex Steph.) Steph., Hedwigia 27 (11/12): 279, 1888 (Stephani 1888c). Bas.: Mastigobryum wrightii Gottsche ex Steph., Hedwigia 25 (6): 237, 1886 (Stephani 1886c).

** Bazzania yoshinagana (Steph.) Yasuda, Shokubutsugaku Kakuron: 711, 1911 (Yasuda 1911). Bas.: Mastigobryum yoshinaganum Steph., Bull. Herb. Boissier (sér. 2) 8 (11): 866 (490), 1908 (Stephani 1908a).

** Bazzania zollingeri (Lindenb.) Trevis., Mem. Reale Ist. Lombardo Sci. (Ser. 3), C. Sci. Mat. 4 (13): 414, 1877 (Trevisan 1877). Bas.: Mastigobryum zollingeri Lindenb., Bot. Zeitung (Berlin) 6 (25): 462, 1848 (Meissner 1848).

*** Bazzania zonulata Meagher, Nova Hedwigia 86 (3/4): 491, 2008 (Meagher 2008).

* **Mastigopelma Mitt.**, Fl. vit.: 406, 1871 [1873] (Mitten 1871). ^118^

** Mastigopelma fragile (Steph.) N.Kitag., J. Hattori Bot. Lab. 36: 454, 1972 [1973] (Kitagawa 1972). Bas.: Mastigobryum fragile Steph., Sp. Hepat. (Stephani) 6: 463, 1924 (Stephani 1924).

** Mastigopelma pulvinulatum (De Not.) Grolle, J. Hattori Bot. Lab. 33: 39, 1970 (Grolle 1970c). Bas.: Mastigobryum pulvinulatum De Not., Epat. Borneo: 40, 1874 (De Notaris 1874).

** Mastigopelma simplex Mitt., Fl. vit.: 406, 1871 [1873] (Mitten 1871).

** Mastigopelma subfissum Grolle, J. Hattori Bot. Lab. 33: 36, 1970 (Grolle 1970c).

######### *** Drucelloideae R.M.Schust.

*** **Drucella E.A.Hodgs.**, Trans. Roy. Soc. New Zealand, Bot. 2 (3): 45, 1962 (Hodgson 1962b).

*** Drucella integristipula (Steph.) E.A.Hodgs., Trans. Roy. Soc. New Zealand, Bot. 2 (3): 45, 1962 (Hodgson 1962b). Bas.: Lepidozia integristipula Steph., Sp. Hepat. (Stephani) 6: 331, 1922 (Stephani 1922).

######### *** Lembidioideae R.M.Schust.

** **Dendrolembidium Herzog**, Ark. Bot. (n.ser.) 1 (13): 497, 1951 (Herzog 1951b).

*** Dendrolembidium dendroides (Carrington et Pearson) Herzog, Ark. Bot. (n.ser.) 1 (13): 500, 1951 (Herzog 1951b). Bas.: Lembidium dendroides Carrington et Pearson, Proc. Linn. Soc. New South Wales (ser. 2) 2 (4): 1047, 1888 (Carrington and Pearson 1888a).

*** Dendrolembidium tenax (Grev.) Herzog, Ark. Bot. (n.ser.) 1 (13): 499, 1951 (Herzog 1951b). Bas.: Jungermannia tenax Grev., Ann. Lyceum Nat. Hist. New York 1 (2): 277, 1825 (Greville 1825).

** **Hygrolembidium R.M.Schust.**, J. Hattori Bot. Lab. 26: 277, 1963 (Schuster 1963b).

*** Hygrolembidium acrocladum (Berggr.) R.M.Schust., J. Hattori Bot. Lab. 26: 277, 1963 (Schuster 1963b). Bas.: Aplozia acroclada Berggr., New Zealand Hepat.: 9, 1898 (Berggren 1898).

** Hygrolembidium andinum (Herzog) R.M.Schust., Nova Hedwigia 10 (1/2): 23, 1965 (Schuster 1965b). Bas.: Lembidium andinum Herzog, Rev. Bryol. Lichénol. 23 (1/2): 48, 1954 (Herzog 1954).

*** Hygrolembidium australe (Steph.) Grolle, J. Jap. Bot. 41 (8): 229, 1966 (Grolle 1966d). Bas.: Hygrobiella australis Steph., Bull. Herb. Boissier (sér. 2) 8 (8): 574 (358), 1908 (Stephani 1908e).

*** Hygrolembidium boschianum (Sande Lac.) R.M.Schust., J. Hattori Bot. Lab. 26: 277, 1963 (Schuster 1963b). Bas.: Jungermannia boschiana Sande Lac., Ned. Kruidk. Arch. 3: 521, 1855 (Sande Lacoste 1855).

** Hygrolembidium isophyllum R.M.Schust., Nova Hedwigia 15: 467, 1968 (Schuster 1968b).

*** Hygrolembidium rigidum R.M.Schust. et J.J.Engel, Phytologia 62 (1): 9, 1987 (Schuster and Engel 1987a).

*** Hygrolembidium triquetrum J.J.Engel et R.M.Schust., Phytologia 62 (1): 11, 1987 (Schuster and Engel 1987a).

** Hygrolembidium ventrosum (Mitt.) Grolle, Marion Prince Edw. Is: 233, 1971 (Grolle 1971d). Bas.: Lembidium ventrosum Mitt., J. Linn. Soc., Bot. 15 (82): 69, 1876 (Mitten 1876a).

** **Isolembidium R.M.Schust.**, Nova Hedwigia 15: 466, 1968 (Schuster 1968b).

*** Isolembidium anomalum (Rodway) Grolle, J. Bryol. 10 (3): 264, 1979 (Grolle 1979b). Bas.: Lembidium anomalum Rodway, Tasm. Bryoph.: 70, 1917 (Rodway 1917b).

** Isolembidium anomalum var. cucullatum (E.A.Hodgs.) J.J.Engel et R.M.Schust., J. Hattori Bot. Lab. 63: 268, 1987 (Schuster and Engel 1987b). Bas.: Lembidium cucullatum E.A.Hodgs., Rec. Domin. Mus. 4 (11): 110, 1962 (Hodgson 1962a).

*** **Kurzia G.Martens**, Flora 53 (27): 417, 1870 (0).

** **subg. Kurzia**

*** Kurzia capillaris (Sw.) Grolle, Rev. Bryol. Lichénol. 32 (3/4): 173, 1963 [1964] (Grolle 1963b). Bas.: Jungermannia capillaris Sw., Prodr. (Swartz): 144, 1788 (Swartz 1788).

*** Kurzia capillaris subsp. capillaris var. verrucosa (Steph.) Pócs, Proc. Third Meeting Bryol. C. & E. Europe: 111, 1984 (Pócs 1984b). Bas.: Lepidozia verrucosa Steph., Hedwigia 24 (4): 167, 1885 (Stephani 1885f).

*** Kurzia capillaris subsp. paramicola Pócs, Acta Biol. Pl. Agr. 2: 102, 2012 (Pócs 2012a).

*** Kurzia capillaris subsp. stephanii (Renauld ex Steph.) Pócs, Proc. Third Meeting Bryol. C. & E. Europe: 111, 1984 (Pócs 1984b). Bas.: Lepidozia stephanii Renauld ex Steph., Bot. Gaz. 15 (11): 287, 1890 (Stephani 1890c).

*** Kurzia gonyotricha (Sande Lac.) Grolle, Rev. Bryol. Lichénol. 32 (3/4): 167, 1963 [1964] (Grolle 1963b). Bas.: Lepidozia gonyotricha Sande Lac., Ned. Kruidk. Arch. 3: 521, 1855 (Sande Lacoste 1855).

** Kurzia nemoides (Hook.f. et Taylor) Grolle, Rev. Bryol. Lichénol. 32 (3/4): 173, 1963 [1964] (Grolle 1963b). Bas.: Jungermannia nemoides Hook.f. et Taylor, London J. Bot. 4: 84, 1845 (Hooker and Taylor 1845).

** **subg. Micrisophylla (Fulford) J.J.Engel ex R.M.Schust.**, J. Hattori Bot. Lab. 48: 347, 1980 (Schuster 1980a). Bas.: Micrisophylla Fulford, Brittonia 14 (1): 124, 1962 (Fulford 1962a).

** Kurzia saddlensis (Besch. et C.Massal.) Grolle, Rev. Bryol. Lichénol. 32 (3/4): 174, 1963 [1964] (Grolle 1963b). Bas.: Lepidozia saddlensis Besch. et C.Massal., Bull. Mens. Soc. Linn. Paris 1 (79): 637, 1886 (Bescherelle and Massalongo 1886).

** **subg. Microlepidozia (Spruce) R.M.Schust.**, J. Hattori Bot. Lab. 48: 355, 1980 (Schuster 1980a). Bas.: Lepidozia subg. Microlepidozia Spruce, J. Bot. 14: 165, 1876 (Spruce 1876b).

** Kurzia calcarata (Steph.) Grolle, Rev. Bryol. Lichénol. 32 (3/4): 178, 1963 [1964] (Grolle 1963b). Bas.: Lepidozia calcarata Steph., Sp. Hepat. (Stephani) 3: 592, 1909 (Stephani 1909a).

** Kurzia compacta (Steph.) Grolle, Rev. Bryol. Lichénol. 32 (3/4): 178, 1963 [1964] (Grolle 1963b). Bas.: Lepidozia compacta Steph., Sp. Hepat. (Stephani) 3: 592, 1909 (Stephani 1909a).

** Kurzia helophila R.M.Schust., J. Hattori Bot. Lab. 48: 368, 1980 (Schuster 1980a).

** Kurzia helophila var. flaccida R.M.Schust. ex J.J.Engel, Novon 17 (3): 310, 2007 (Engel 2007).

*** Kurzia hippurioides (Hook.f. et Taylor) Grolle, Rev. Bryol. Lichénol. 32 (3/4): 178, 1963 [1964] (Grolle 1963b). Bas.: Jungermannia hippurioides Hook.f. et Taylor, London J. Bot. 3: 287 [387], 1844 (Hooker and Taylor 1844a).

** Kurzia hippurioides var. ornata J.J.Engel et G.L.Merr., J. Hattori Bot. Lab. 80: 228, 1996 (Engel and Smith Merrill 1996).

** Kurzia irregularis (Steph.) Grolle, J. Hattori Bot. Lab. 36: 548, 1972 [1973] (Grolle 1972a). Bas.: Lepidozia irregularis Steph., Wiss. Ergebn. Deut. Zentr.-Afr. Exped. (1907-08), Bot. 2: 120, 1911 (Stephani 1911a).

** Kurzia moniliformis J.J.Engel, Cryptog. Bryol. 26 (1): 73, 2005 (Engel 2005).

*** Kurzia pauciflora (Dicks.) Grolle, Rev. Bryol. Lichénol. 32 (3/4): 171, 1963 [1964] (Grolle 1963b). Bas.: Jungermannia pauciflora Dicks., Fasc. Pl. Crypt. Brit. 2: 15, 1790 (Dickson 1790).

***Incertae sedis***

** Kurzia abbreviata Mizut., J. Hattori Bot. Lab. 38: 379, 1974 (Mizutani 1974).

** Kurzia abietinella (Herzog) Grolle, Rev. Bryol. Lichénol. 32 (3/4): 170, 1963 [1964] (Grolle 1963b). Bas.: Lepidozia abietinella Herzog, Trans. Brit. Bryol. Soc. 1 (4): 311, 1950 (Herzog 1950a).

*** Kurzia bisetula (Steph.) Grolle, Rev. Bryol. Lichénol. 32 (3/4): 170, 1963 [1964] (Grolle 1963b). Bas.: Lepidozia bisetula Steph., Sp. Hepat. (Stephani) 6: 323, 1922 (Stephani 1922).

** Kurzia borneensis Mizut., J. Hattori Bot. Lab. 38: 377, 1974 (Mizutani 1974).

** Kurzia brasiliensis (Steph.) Grolle, Rev. Bryol. Lichénol. 32 (3/4): 174, 1963 [1964] (Grolle 1963b). Bas.: Psiloclada brasiliensis Steph., Sp. Hepat. (Stephani) 3: 550, 1909 (Stephani 1909a).

** Kurzia brevicalycina (Steph.) Grolle, Rev. Bryol. Lichénol. 32 (3/4): 175, 1963 [1964] (Grolle 1963b). Bas.: Lepidozia brevicalycina Steph., Sp. Hepat. (Stephani) 3: 580, 1909 (Stephani 1909a).

* Kurzia caduciloba R.M.Schust., Beih. Nova Hedwigia 118: 273, 2000 (Schuster 2000a).

* Kurzia cucullifolia (Steph.) R.M.Schust., J. Hattori Bot. Lab. 48: 350, 1980 (Schuster 1980a). Bas.: Lepidozia cucullifolia Steph., Bih. Kongl. Svenska Vetensk.-Akad. Handl. 26 (III, 6): 51, 1900 (Stephani 1900b). ^119^

*** Kurzia flagellifera (Steph.) Grolle, J. Jap. Bot. 39 (3): 80, 1964 (Grolle 1964c). Bas.: Lepidozia flagellifera Steph., Sp. Hepat. (Stephani) 3: 571, 1909 (Stephani 1909a).

*** Kurzia fragilifolia R.M.Schust., J. Hattori Bot. Lab. 48: 364, 1980 (Schuster 1980a).

** Kurzia fragillima (Herzog) Grolle, Rev. Bryol. Lichénol. 32 (3/4): 174, 1963 [1964] (Grolle 1963b). Bas.: Lepidozia fragillima Herzog, Nat. Hist. Juan Fernandez (Botany) 2 (5): 726, 1942 (Herzog 1942a), *nom. illeg*.

** Kurzia geniculata Mizut., J. Hattori Bot. Lab. 38: 383, 1974 (Mizutani 1974).

** Kurzia hawaica (C.M.Cooke) Grolle, Rev. Bryol. Lichénol. 32 (3/4): 170, 1963 [1964] (Grolle 1963b). Bas.: Lepidozia hawaica C.M.Cooke, Trans. Connecticut Acad. Arts 12 (1): 8, 1904 (Cooke 1904).

** Kurzia hispida (Steph.) Grolle, Rev. Bryol. Lichénol. 32 (3/4): 170, 1963 [1964] (Grolle 1963b). Bas.: Lepidozia hispida Steph., Sp. Hepat. (Stephani) 3: 607, 1909 (Stephani 1909a).

** Kurzia lateconica (Steph.) Grolle, Rev. Bryol. Lichénol. 32 (3/4): 175, 1963 [1964] (Grolle 1963b). Bas.: Lepidozia lateconica Steph., J. & Proc. Roy. Soc. New South Wales 48 (1/2): 113, 1914 (Stephani and Watts 1914).

** Kurzia lineariloba Mizut., J. Hattori Bot. Lab. 38: 382, 1974 (Mizutani 1974).

*** Kurzia longicaulis Piippo, Acta Bot. Fenn. 131: 174, 1985 (Piippo 1985b).

** Kurzia makinoana (Steph.) Grolle, Rev. Bryol. Lichénol. 32 (3/4): 171, 1963 [1964] (Grolle 1963b). Bas.: Lepidozia makinoana Steph., Bull. Herb. Boissier 5 (2): 94, 1897 (Stephani 1897b).

** Kurzia mauiensis (H.A.Mill.) H.A.Mill., J. Hattori Bot. Lab. 30: 274, 1967 (Miller 1967). Bas.: Microlepidozia mauiensis H.A.Mill., Ark. Bot. (n.ser.) 5 (2): 496, 1963 (Miller 1963).

** Kurzia mollis (Steph.) J.J.Engel et R.M.Schust., Bryologist 79 (4): 514, 1976 [1977] (Engel 1976b). Bas.: Lepidozia mollis Steph., Sp. Hepat. (Stephani) 3: 601, 1909 (Stephani 1909a).

*** Kurzia nivicola (R.M.Schust.) E.D.Cooper, Phytotaxa 97 (2): 52, 2013 (Cooper et al. 2013). Bas.: Telaranea nivicola R.M.Schust., Nova Hedwigia 15: 460, 1968 (Schuster 1968b).

*** Kurzia pallescens Grolle, Rev. Bryol. Lichénol. 32 (3/4): 177, 1963 [1964] (Grolle 1963b).

** Kurzia pallida Piippo, Acta Bot. Fenn. 131: 178, 1985 (Piippo 1985b).

*** Kurzia quinquespina J.J.Engel et G.L.Merr., J. Hattori Bot. Lab. 80: 217, 1996 (Engel and Smith Merrill 1996).

** Kurzia reversa (Carrington et Pearson) Grolle, Rev. Bryol. Lichénol. 32 (3/4): 175, 1963 [1964] (Grolle 1963b). Bas.: Lepidozia reversa Carrington et Pearson, J. Bot. 27: 225, 1889 (Carrington and Pearson 1889).

** Kurzia setiformis (De Not.) J.J.Engel et R.M.Schust., Bryologist 79 (4): 514, 1976 [1977] (Engel 1976b). Bas.: Lepidozia setiformis De Not., Mem. Reale Accad. Sci. Torino (ser. 2) 16: 225, 1857 (De Notaris 1857).

** Kurzia sexfida (Steph.) Grolle, Rev. Bryol. Lichénol. 32 (3/4): 178, 1963 [1964] (Grolle 1963b). Bas.: Lepidozia sexfida Steph., Sp. Hepat. (Stephani) 3: 582, 1909 (Stephani 1909a).

** Kurzia sinensis K.C.Chang, Bull. Bot. Res., Harbin 4 (3): 83, 1984 (Chang and Gao 1984).

** Kurzia sylvatica (A.Evans) Grolle, Herzogia 3: 77, 1973 (Grolle 1973a). Bas.: Lepidozia sylvatica A.Evans, Rhodora 6 (69): 186, 1904 (Evans 1904b).

*** Kurzia tasmanica (Steph.) E.D.Cooper, Phytotaxa 97 (2): 52, 2013 (Cooper et al. 2013). Bas.: Lepidozia tasmanica Steph., Sp. Hepat. (Stephani) 3: 580, 1909 (Stephani 1909a).

** Kurzia tayloriana (H.A.Mill.) H.A.Mill., J. Hattori Bot. Lab. 30: 274, 1967 (Miller 1967). Bas.: Microlepidozia tayloriana H.A.Mill., Ark. Bot. (n.ser.) 5 (2): 497, 1963 (Miller 1963).

** Kurzia tenerrima (Mitt.) Grolle, Rev. Bryol. Lichénol. 32 (3/4): 171, 1963 [1964] (Grolle 1963b). Bas.: Lepidozia tenerrima Mitt., Sp. Hepat. (Stephani) 3: 607, 1909 (Stephani 1909a).

** Kurzia touwii N.Kitag., Acta Phytotax. Geobot. 29 (1/5): 56, 1978 (Kitagawa 1978).

** Kurzia trichoclados (Müll.Frib.) Grolle, Rev. Bryol. Lichénol. 32 (3/4): 171, 1963 [1964] (Grolle 1963b). Bas.: Lepidozia trichoclados Müll.Frib., Hedwigia 38 (4): 197, 1899 (Müller 1899).

*** Kurzia trilobata (R.M.Schust.) R.M.Schust., Beih. Nova Hedwigia 118: 270, 2000 (Schuster 2000a). Bas.: Kurzia quadriseta var. trilobata R.M.Schust., J. Hattori Bot. Lab. 48: 363, 1980 (Schuster 1980a).

** Kurzia verticellata (Carrington) Grolle, Rev. Bryol. Lichénol. 32 (3/4): 178, 1963 [1964] (Grolle 1963b). Bas.: Lepidozia verticellata Carrington, Pap. & Proc. Roy. Soc. Tasmania 1887: 3, 1888 (Carrington and Pearson 1888b).

** **Lembidium Mitt.**, Handb. N. Zeal. fl. 2: 754, 1867 (Hooker 1867) nom. conserv.

*** Lembidium berggrenii Herzog, Ark. Bot. (n.ser.) 1 (13): 485, 1951 (Herzog 1951b).

*** Lembidium longifolium R.M.Schust., Phytologia 45 (5): 420, 1980 (Schuster 1980b).

*** Lembidium nutans (Hook.f. et Taylor) Mitt., Handb. N. Zeal. Fl. 2: 754, 1867 (Mitten 1867). Bas.: Jungermannia nutans Hook.f. et Taylor, London J. Bot. 3: 289 [389], 1844 (Hooker and Taylor 1844a).

** Lembidium nutans var. flagelliferum E.A.Hodgs., Rec. Domin. Mus. 4 (11): 110, 1962 (Hodgson 1962a).

** **Megalembidium R.M.Schust.**, J. Hattori Bot. Lab. 26: 258, 1963 (Schuster 1963b).

*** Megalembidium insulanum (W.Martin et E.A.Hodgs.) R.M.Schust., J. Hattori Bot. Lab. 26: 258, 1963 (Schuster 1963b). Bas.: Lembidium insulanum W.Martin et E.A.Hodgs., Trans. & Proc. Roy. Soc. New Zealand 78 (4): 497, 1950 (Martin 1950).

** **Pseudocephalozia R.M.Schust.**, Nova Hedwigia 10 (1/2): 21, 1965 (Schuster 1965b).

** **sect. Lobulatae R.M.Schust.**, J. Hattori Bot. Lab. 36: 371, 1972 (Schuster 1972).

** Pseudocephalozia cucullata J.J.Engel et R.M.Schust., J. Hattori Bot. Lab. 38: 694, 1974 (Schuster and Engel 1974).

*** Pseudocephalozia lobulata (Herzog) R.M.Schust., J. Hattori Bot. Lab. 36: 371, 1972 [1973] (Schuster 1972). Bas.: Lembidium lobulatum Herzog, Arch. Esc. Fárm. Fac. Ci. Méd. Córdoba 7: 24, 1938 (Herzog and Hosseus 1938).

*** Pseudocephalozia quadriloba (Steph.) R.M.Schust., J. Hattori Bot. Lab. 36: 371, 1972 [1973] (Schuster 1972). Bas.: Isotachis quadriloba Steph., Bih. Kongl. Svenska Vetensk.-Akad. Handl. 26 (III, 6): 54, 1900 (Stephani 1900b).

** **sect. Pseudocephalozia**

** Pseudocephalozia lepidozioides R.M.Schust., Nova Hedwigia 10 (1/2): 22, 1965 (Schuster 1965b).

** Pseudocephalozia leptodictyon R.M.Schust., J. Hattori Bot. Lab. 36: 369, 1972 [1973] (Schuster 1972).

*** Pseudocephalozia paludicola R.M.Schust., Nova Hedwigia 10 (1/2): 21, 1965 (Schuster 1965b).

######### *** Lepidozioideae Müll.Frib.

*** **Ceramanus E.D.Cooper**, Phytotaxa 97 (2): 53, 2013 (Cooper et al. 2013).

*** Ceramanus centipes (Lindenb. et Gottsche) E.D.Cooper, Phytotaxa 97 (2): 54, 2013 (Cooper et al. 2013). Bas.: Lepidozia centipes Lindenb. et Gottsche, Syn. Hepat. 2: 204, 1845 (Gottsche et al. 1845a).

*** Ceramanus clatritexta (Steph.) E.D.Cooper, Phytotaxa 97 (2): 54, 2013 (Cooper et al. 2013). Bas.: Lepidozia clatritexta Steph., Sp. Hepat. (Stephani) 3: 583, 1909 (Stephani 1909a).

*** Ceramanus elegans (Colenso) E.D.Cooper, Phytotaxa 97 (2): 54, 2013 (Cooper et al. 2013). Bas.: Lepidozia elegans Colenso, Trans. & Proc. New Zealand Inst. 21: 65, 1889 (Colenso 1889).

*** Ceramanus grossiseta (Steph.) E.D.Cooper, Phytotaxa 97 (2): 54, 2013 (Cooper et al. 2013). Bas.: Lepidozia grossiseta Steph., Sp. Hepat. (Stephani) 3: 584, 1909 (Stephani 1909a).

*** Ceramanus perfragilis (J.J.Engel et G.L.Merr.) E.D.Cooper, Phytotaxa 97 (2): 54, 2013 (Cooper et al. 2013). Bas.: Telaranea perfragilis J.J.Engel et G.L.Merr., Fieldiana, Bot. (n.ser.) 44: 72, 2004 (Engel and Smith Merrill 2004).

*** Ceramanus pruinosa (Herzog) E.D.Cooper, Phytotaxa 97 (2): 54, 2013 (Cooper et al. 2013). Bas.: Lepidozia pruinosa Herzog, Memoranda Soc. Fauna Fl. Fennica 27: 93, 1952 (Herzog 1952c).

*** Ceramanus tuberifera (J.J.Engel et R.M.Schust.) E.D.Cooper, Phytotaxa 97 (2): 54, 2013 (Cooper et al. 2013). Bas.: Telaranea tuberifera J.J.Engel et R.M.Schust., Fieldiana, Bot. (n.ser.) 14: 2, 1983 (Engel and Schuster 1983).

*** **Lepidozia (Dumort.) Dumort.**, Recueil Observ. Jungerm.: 19, 1835 (Dumortier 1835) nom. conserv. Bas.: Pleuroschisma sect. Lepidozia Dumort., Syll. Jungerm. Europ.: 69, 1831 (Dumortier 1831). ^120^

** Lepidozia acantha J.J.Engel, Fieldiana, Bot. (n.ser.) 42: 71, 2001 (Engel and Schuster 2001).

** Lepidozia aequiloba Steph., Sp. Hepat. (Stephani) 6: 319, 1922 (Stephani 1922).

** Lepidozia africana Steph., Sp. Hepat. (Stephani) 6: 320, 1922 (Stephani 1922).

*** Lepidozia alstonii Fulford, Mem. New York Bot. Gard. 11 (2): 211, 1966 (Fulford 1966).

** Lepidozia ambigua De Not., Epat. Borneo: 25, 1874 (De Notaris 1874).

** Lepidozia andicola Beauverd, Sp. Hepat. (Stephani) 6: 572, 1924 (Stephani 1924). *Nom. nov. pro Lepidozia appendiculata* Steph., Biblioth. Bot. 87 (2): 225, 1916 (Stephani 1916a), *nom. illeg*.

** Lepidozia appressifolia Steph., Sp. Hepat. (Stephani) 3: 583, 1909 (Stephani 1909a).

*** Lepidozia armata Steph., Sp. Hepat. (Stephani) 3: 567, 1909 (Stephani 1909a).

** Lepidozia asymmetrica Steph., Sp. Hepat. (Stephani) 3: 586, 1909 (Stephani 1909a).

** Lepidozia auriculata Mitt., Sp. Hepat. (Stephani) 3: 579, 1909 (Stephani 1909a).

** Lepidozia australis (Lehm. et Lindenb.) Mitt., Fl. vit.: 406, 1871 [1873] (Mitten 1871). Bas.: Jungermannia australis Lehm. et Lindenb., Nov. Stirp. Pug. 6: 28, 1834 (Lehmann 1834).

** Lepidozia bidens J.J.Engel, Fieldiana, Bot. (n.ser.) 42: 79, 2001 (Engel and Schuster 2001).

** Lepidozia biloba Herzog, Ann. Bryol. 4: 83, 1931 (Herzog 1931b).

*** Lepidozia bisbifida Steph., Sp. Hepat. (Stephani) 3: 593, 1909 (Stephani 1909a).

** Lepidozia borneensis Steph., Sp. Hepat. (Stephani) 3: 625, 1909 (Stephani 1909a).

** Lepidozia bragginsiana E.D.Cooper et M.A.M.Renner, Phytotaxa 173 (2): 118, 2014 (Cooper and Renner 2014).

* Lepidozia brasiliensis Steph., Sp. Hepat. (Stephani) 3: 571, 1909 (Stephani 1909a). ^121^

** Lepidozia brevidentata Mitt., Fl. vit.: 406, 1871 [1873] (Mitten 1871).

** Lepidozia brevifolia Mitt., J. Proc. Linn. Soc., Bot. 5 (18): 104, 1860 [1861] (Mitten 1860c).

** Lepidozia brevifolia var. planifolia Schiffn., Ann. Bryol. 8: 155, 1935 (Verdoorn 1935).

** Lepidozia brotheri Steph., Sp. Hepat. (Stephani) 3: 623, 1909 (Stephani 1909a).

** Lepidozia buffalona Steph., J. & Proc. Roy. Soc. New South Wales 48 (1/2): 111, 1914 (Stephani and Watts 1914).

** Lepidozia bursifera S.Hatt. et Grolle, J. Hattori Bot. Lab. 30: 115, 1967 (Grolle 1967b).

** Lepidozia caespitosa Spruce, Trans. & Proc. Bot. Soc. Edinburgh 15: 362, 1885 (Spruce 1885).

** Lepidozia caledonica Steph., Rev. Bryol. 35 (2): 31, 1908 (Stephani 1908l).

** Lepidozia caledonica var. tenuisecta Hürl., Bauhinia 8 (2): 109, 1985 (Hürlimann 1985).

* Lepidozia ceramensis Herzog, Hedwigia 66 (6): 340, 1926 (Herzog 1926).

** Lepidozia cherydrion Hürl., Bauhinia 8 (2): 109, 1985 (Hürlimann 1985).

** Lepidozia chiloensis Steph., Sp. Hepat. (Stephani) 6: 322, 1922 (Stephani 1922).

** Lepidozia chordulifera Taylor, London J. Bot. 5: 371, 1846 (Taylor 1846b).

*** Lepidozia cladorhiza (Reinw., Blume et Nees) Nees, Syn. Hepat. 2: 210, 1845 (Gottsche et al. 1845a). Bas.: Jungermannia cladorhiza Reinw., Blume et Nees, Nova Acta Phys.-Med. Acad. Caes. Leop.-Carol. Nat. Cur. 12 (1): 203, 1824 [1825] (Reinwardt et al. 1824a).

*** Lepidozia coilophylla Taylor, London J. Bot. 5: 370, 1846 (Taylor 1846b).

** Lepidozia coilophylla var. apiculiloba (Steph.) Fulford, Mem. New York Bot. Gard. 11 (2): 194, 1966 (Fulford 1966). Bas.: Lepidozia apiculiloba Steph., Sp. Hepat. (Stephani) 6: 321, 1922 (Stephani 1922).

** Lepidozia communis Steph., J. & Proc. Roy. Soc. New South Wales 48 (1/2): 111, 1914 (Stephani and Watts 1914).

*** Lepidozia concinna Colenso, Trans. & Proc. New Zealand Inst. 18: 244, 1886 (Colenso 1886b).

** Lepidozia cordata Lindenb., Syn. Hepat. 2: 207, 1845 (Gottsche et al. 1845a).

* Lepidozia cordistipula Steph., Sp. Hepat. (Stephani) 6: 345, 1922 (Stephani 1922).

** Lepidozia crassitexta Steph., J. & Proc. Roy. Soc. New South Wales 48 (1/2): 111, 1914 (Stephani and Watts 1914).

*** Lepidozia cupressina (Sw.) Lindenb., Syn. Hepat. 2: 207, 1845 (Gottsche et al. 1845a). Bas.: Jungermannia cupressina Sw., Prodr. (Swartz): 144, 1788 (Swartz 1788).

** Lepidozia cupressina subsp. natalensis (Steph.) Pócs, Proc. Third Meeting Bryol. C. & E. Europe: 109, 1984 (Pócs 1984b). Bas.: Lepidozia natalensis Steph., Sp. Hepat. (Stephani) 3: 562, 1909 (Stephani 1909a).

* Lepidozia cupressina subsp. pinnata (Hook.) Pócs, Proc. Third Meeting Bryol. C. & E. Europe: 109, 1984 (Pócs 1984b). Bas.: Jungermannia reptans var. pinnata Hook., Brit. Jungermann.: tab. 75, 1815 (Hooker 1815).

* Lepidozia cupressina subsp. quinquefida (Steph.) Pócs, Proc. Third Meeting Bryol. C. & E. Europe: 109, 1984 (Pócs 1984b). Bas.: Lepidozia quinquefida Steph., Wiss. Ergebn. Deut. Zentr.-Afr. Exped. (1907-08), Bot. 2: 123, 1911 (Stephani 1911a).

** Lepidozia decaisnei Steph., Sp. Hepat. (Stephani) 3: 588, 1909 (Stephani 1909a).

** Lepidozia dendritica Spruce, Trans. & Proc. Bot. Soc. Edinburgh 15: 362, 1885 (Spruce 1885).

** Lepidozia densa Herzog, Repert. Spec. Nov. Regni Veg. 21 (1/7): 26, 1925 (Herzog 1925a).

*** Lepidozia digitata Herzog, Trans. & Proc. Roy. Soc. New Zealand 68 (1): 45, 1938 (Herzog 1938c).

*** Lepidozia eenii S.W.Arnell, Svensk Bot. Tidskr. 57 (2): 190, 1963 (Arnell 1963a).

** Lepidozia elobata R.M.Schust., Fieldiana, Bot. (n.ser.) 42: 74, 2001 (Engel and Schuster 2001).

** Lepidozia erosa Steph., Sp. Hepat. (Stephani) 3: 621, 1909 (Stephani 1909a).

** Lepidozia erronea Herzog, Nat. Hist. Juan Fernandez (Botany) 2 (5): 725, 1942 (Herzog 1942a). *Nom. nov. pro Lepidozia fernandeziensis* Steph., Sp. Hepat. (Stephani) 6: 326, 1922 (Stephani 1922), *nom. illeg*.

** Lepidozia everettii Steph., Sp. Hepat. (Stephani) 3: 622, 1909 (Stephani 1909a).

* Lepidozia everettii var. javensis Herzog, Ann. Bryol. 5: 78, 1932 (Herzog 1932b).

** Lepidozia fauriana Steph., Sp. Hepat. (Stephani) 3: 631, 1909 (Stephani 1909a).

*** Lepidozia ferdinandi-muelleri Steph., Sp. Hepat. (Stephani) 3: 614, 1909 (Stephani 1909a).

** Lepidozia filamentosa (Lehm. et Lindenb.) Lehm. et Lindenb., Syn. Hepat. 2: 206, 1845 (Gottsche et al. 1845a). Bas.: Jungermannia filamentosa Lehm. et Lindenb., Nov. Stirp. Pug. 6: 29, 1834 (Lehmann 1834).

** Lepidozia fistulosa Mitt., Fl. vit.: 406, 1871 [1873] (Mitten 1871).

** Lepidozia flexuosa Mitt., J. Proc. Linn. Soc., Bot. 5 (18): 103, 1860 [1861] (Mitten 1860c).

** Lepidozia fuegiensis Steph., Kungl. Svenska Vetensk.-Akad. Handl. (n.ser.) 46 (9): 63, 1911 (Stephani 1911b).

** Lepidozia fugax J.J.Engel, Fieldiana, Bot. (n.ser.) 42: 63, 2001 (Engel and Schuster 2001).

* Lepidozia gedena Steph., Sp. Hepat. (Stephani) 6: 327, 1922 (Stephani 1922).

*** Lepidozia glaucescens J.J.Engel, Fieldiana, Bot. (n.ser.) 42: 101, 2001 (Engel and Schuster 2001).

*** Lepidozia glaucophylla (Hook.f. et Taylor) Gottsche, Lindenb. et Nees, Syn. Hepat. 2: 207, 1845 (Gottsche et al. 1845a). Bas.: Jungermannia glaucophylla Hook.f. et Taylor, London J. Bot. 3: 580, 1844 (Hooker and Taylor 1844c).

** Lepidozia grandifolia Steph., Sp. Hepat. (Stephani) 3: 625, 1909 (Stephani 1909a).

* Lepidozia griseola Herzog, Hedwigia 66 (6): 340, 1926 (Herzog 1926).

** Lepidozia groenlandica Lehm., Nov. Stirp. Pug. 10: 7, 1857 (Lehmann 1857).

** Lepidozia gwamii Piippo, Ann. Bot. Fenn. 21 (4): 311, 1984 (Piippo 1984b).

** Lepidozia hampeana Lindenb., Syn. Hepat. 2: 208, 1845 (Gottsche et al. 1845a).

*** Lepidozia haskarliana (Gottsche, Lindenb. et Nees) Steph., Sp. Hepat. (Stephani) 3: 614, 1909 (Stephani 1909a). Bas.: Lepidozia supradecomposita β haskarliana Gottsche, Lindenb. et Nees, Syn. Hepat. 2: 202, 1845 (Gottsche et al. 1845a).

** Lepidozia hastatistipula Steph., J. & Proc. Roy. Soc. New South Wales 48 (1/2): 113, 1914 (Stephani and Watts 1914).

*** Lepidozia hirta Steph., Sp. Hepat. (Stephani) 3: 599, 1909 (Stephani 1909a).

*** Lepidozia holorhiza (Reinw., Blume et Nees) Nees, Syn. Hepat. 2: 210, 1845 (Gottsche et al. 1845a). Bas.: Jungermannia holorhiza Reinw., Blume et Nees, Nova Acta Phys.-Med. Acad. Caes. Leop.-Carol. Nat. Cur. 12 (1): 204, 1824 [1825] (Reinwardt et al. 1824a).

** Lepidozia holorhiza var. laxa (Nees) Schiffn., Consp. Hepat. Arch. Ind.: 186, 1898 (Schiffner 1898b). Bas.: Jungermannia holorhiza β laxa Nees, Enum. Pl. Crypt. Javae: 14, 1830 (Nees 1830).

** Lepidozia inaequalis (Lehm. et Lindenb.) Lehm. et Lindenb., Syn. Hepat. 2: 209, 1845 (Gottsche et al. 1845a). Bas.: Jungermannia inaequalis Lehm. et Lindenb., Nov. Stirp. Pug. 5: 1, 1833 (Lehmann 1833).

*** Lepidozia incurvata Lindenb., Syn. Hepat. 2: 203, 1845 (Gottsche et al. 1845a).

** Lepidozia infuscata Mitt., Fl. vit.: 406, 1871 [1873] (Mitten 1871).

** Lepidozia integrifolia Doei, J. Hattori Bot. Lab. 63: 421, 1987 (Doei 1987).

*** Lepidozia jamaicensis Steph., Sp. Hepat. (Stephani) 3: 568, 1909 (Stephani 1909a).

** Lepidozia kashyapii D.Singh et D.K.Singh, Nova Hedwigia 94 (1/2): 222, 2012 (Singh and Singh 2012).

** Lepidozia kinabaluensis Mizut., J. Hattori Bot. Lab. 38: 372, 1974 (Mizutani 1974).

*** Lepidozia kirkii Steph., Sp. Hepat. (Stephani) 3: 598, 1909 (Stephani 1909a).

** Lepidozia lacerifolia Steph., Sp. Hepat. (Stephani) 6: 332, 1922 (Stephani 1922).

*** Lepidozia laevifolia (Hook.f. et Taylor) Gottsche, Lindenb. et Nees, Syn. Hepat. 2: 208, 1845 (Gottsche et al. 1845a). Bas.: Jungermannia laevifolia Hook.f. et Taylor, London J. Bot. 3: 285 [385], 1844 (Hooker and Taylor 1844a).

** Lepidozia laevifolia var. acutiloba J.J.Engel, Fieldiana, Bot. (n.ser.) 42: 61, 2001 (Engel and Schuster 2001).

** Lepidozia laevifolia var. alpina R.M.Schust. et J.J.Engel, Fieldiana, Bot. (n.ser.) 42: 62, 2001 (Engel and Schuster 2001).

** Lepidozia lindigiana Steph., Sp. Hepat. (Stephani) 3: 573, 1909 (Stephani 1909a).

** Lepidozia loheri Steph., Sp. Hepat. (Stephani) 3: 621, 1909 (Stephani 1909a).

** Lepidozia longifolia Steph., Sp. Hepat. (Stephani) 3: 606, 1909 (Stephani 1909a).

** Lepidozia loriana Steph., Sp. Hepat. (Stephani) 6: 333, 1922 (Stephani 1922).

*** Lepidozia macrocolea Spruce, Trans. & Proc. Bot. Soc. Edinburgh 15: 363, 1885 (Spruce 1885).

* Lepidozia massartiana Schiffn. ex Steph., Sp. Hepat. (Stephani) 3: 611, 1909 (Stephani 1909a). Based on: Lepidozia massartiana Schiffn., Hedwigia 39 (4): 196, 1900 (Schiffner 1900b), *nom. inval*. ^122^

*** Lepidozia microphylla (Hook.) Lindenb., Syn. Hepat. 2: 202, 1845 (Gottsche et al. 1845a). Bas.: Jungermannia microphylla Hook., Musci Exot. 1: tab. 80, 1818 (Hooker 1818).

** Lepidozia microstipula Steph., J. & Proc. Roy. Soc. New South Wales 48 (1/2): 114, 1914 (Stephani and Watts 1914).

** Lepidozia minima Steph., Sp. Hepat. (Stephani) 6: 335, 1922 (Stephani 1922).

* Lepidozia minor (Gottsche, Lindenb. et Nees) Solari, Boll. Mus. Civico Storia Nat. Verona 10: 203, 1983 [1985] (Solari and Hässel 1983). Bas.: Lepidozia truncatella β minor Gottsche, Lindenb. et Nees, Syn. Hepat. 2: 209, 1845 (Gottsche et al. 1845a). ^123^

** Lepidozia miqueliana Sande Lac., Ann. Mus. Bot. Lugduno-Batavi 1: 301, 1864 (Sande Lacoste 1864).

** Lepidozia montana Steph., Sp. Hepat. (Stephani) 3: 587, 1909 (Stephani 1909a).

* Lepidozia newtonii Steph., Sp. Hepat. (Stephani) 3: 623, 1909 (Stephani 1909a).

* Lepidozia nova Steph., J. & Proc. Roy. Soc. New South Wales 48 (1/2): 115, 1914 (Stephani and Watts 1914).

*** Lepidozia novae-zelandiae Steph., Sp. Hepat. (Stephani) 3: 595, 1909 (Stephani 1909a).

** Lepidozia novae-zelandiae var. heterostipa R.M.Schust., Fieldiana, Bot. (n.ser.) 42: 70, 2001 (Engel and Schuster 2001).

** Lepidozia novae-zelandiae var. minima R.M.Schust., Fieldiana, Bot. (n.ser.) 42: 71, 2001 (Engel and Schuster 2001).

*** Lepidozia obtusiloba Steph., Sp. Hepat. (Stephani) 3: 598, 1909 (Stephani 1909a).

*** Lepidozia obtusiloba var. parvula J.J.Engel, Fieldiana, Bot. (n.ser.) 42: 48, 2001 (Engel and Schuster 2001).

** Lepidozia omeiensis P.C.Chen ex Mizut. et K.C.Chang, J. Hattori Bot. Lab. 60: 421, 1986 (Mizutani and Chang 1986).

*** Lepidozia ornata J.J.Engel, Fieldiana, Bot. (n.ser.) 42: 49, 2001 (Engel and Schuster 2001).

* Lepidozia pallida Steph., Sp. Hepat. (Stephani) 3: 604, 1909 (Stephani 1909a). ^124^

* Lepidozia palmicola Steph., Sp. Hepat. (Stephani) 6: 346, 1922 (Stephani 1922).

** Lepidozia paschalis Steph., Sp. Hepat. (Stephani) 6: 336, 1922 (Stephani 1922).

*** Lepidozia patens Lindenb., Syn. Hepat. 2: 202, 1845 (Gottsche et al. 1845a).

* Lepidozia paucifolia Steph., Sp. Hepat. (Stephani) 3: 610, 1909 (Stephani 1909a). ^125^

** Lepidozia paupercula Steph., Sp. Hepat. (Stephani) 6: 337, 1922 (Stephani 1922).

** Lepidozia pearsonii Spruce, J. Bot. 19: 34, 1881 (Spruce 1881b).

*** Lepidozia pendulina (Hook.) Lindenb., Syn. Hepat. 2: 208, 1845 (Gottsche et al. 1845a). Bas.: Jungermannia pendulina Hook., Musci Exot. 1: tab. 60, 1818 (Hooker 1818).

** Lepidozia peruviensis Steph., Sp. Hepat. (Stephani) 3: 575, 1909 (Stephani 1909a).

*** Lepidozia pinnaticruris Spruce ex Steph., Sp. Hepat. (Stephani) 3: 579, 1909 (Stephani 1909a).

* Lepidozia plumula Herzog, Beih. Bot. Centralbl. 38 (2): 331, 1921 (Herzog 1921).

** Lepidozia portoricensis Fulford, Mem. New York Bot. Gard. 11 (2): 187, 1966 (Fulford 1966).

*** Lepidozia procera Mitt., Bot. antarct. voy. III (Fl. Tasman. 2): 231, 1860 (Mitten 1860b).

* Lepidozia pseudocupressina Schiffn., Krit. Bemerk. Eur. Lebermoose 14: 9, 1919 (Schiffner 1919).

*** Lepidozia pumila J.J.Engel, Fieldiana, Bot. (n.ser.) 42: 76, 2001 (Engel and Schuster 2001).

*** Lepidozia quadridens (Nees) Nees, Syn. Hepat. 2: 209, 1845 (Gottsche et al. 1845a). Bas.: Jungermannia quadridens Nees, Enum. Pl. Crypt. Javae: 18, 1830 (Nees 1830).

** Lepidozia quadrifida Lindenb., Syn. Hepat. 2: 203, 1845 (Gottsche et al. 1845a).

*** Lepidozia reptans (L.) Dumort., Recueil Observ. Jungerm.: 19, 1835 (Dumortier 1835). Bas.: Jungermannia reptans L., Sp. Pl. 1: 1133, 1753 (Linnaeus 1753).

** Lepidozia richardsii Herzog, Trans. Brit. Bryol. Soc. 1 (4): 313, 1950 (Herzog 1950a).

** Lepidozia rigida Steph., Sp. Hepat. (Stephani) 6: 340, 1922 (Stephani 1922).

** Lepidozia robusta Steph., Mém. Soc. Nat. Sci. Nat. Math. Cherbourg 29: 217, 1894 (Stephani 1894b).

* Lepidozia rufescens Steph., Biblioth. Bot. 87 (2): 226, 1916 (Stephani 1916a).

** Lepidozia sandvicensis Lindenb., Syn. Hepat. 2: 201, 1845 (Gottsche et al. 1845a).

** Lepidozia schwabei Herzog, Rev. Bryol. Lichénol. 23 (1/2): 49, 1954 (Herzog 1954).

** Lepidozia sellingiana H.A.Mill., Ark. Bot. (n.ser.) 5 (2): 494, 1963 (Miller 1963).

*** Lepidozia septemfida Steph., Sp. Hepat. (Stephani) 3: 588, 1909 (Stephani 1909a).

** Lepidozia serpens Spruce, Trans. & Proc. Bot. Soc. Edinburgh 15: 364, 1885 (Spruce 1885).

*** Lepidozia serrulata J.J.Engel, J. Hattori Bot. Lab. 96: 273, 2004 (Engel 2004a).

*** Lepidozia setigera Steph., Sp. Hepat. (Stephani) 3: 599, 1909 (Stephani 1909a).

** Lepidozia sikkimensis Steph., Sp. Hepat. (Stephani) 6: 341, 1922 (Stephani 1922).

*** Lepidozia spinosissima (Hook.f. et Taylor) Mitt., Bot. antarct. voy. II (Fl. Nov.-Zel. 2): 146, 1854 (Mitten 1854). Bas.: Sendtnera spinosissima Hook.f. et Taylor, London J. Bot. 5: 373, 1846 (Taylor 1846b).

* Lepidozia squamifolia Steph., Sp. Hepat. (Stephani) 6: 341, 1922 (Stephani 1922).

*** Lepidozia squarrosa Steph., Sp. Hepat. (Stephani) 3: 573, 1909 (Stephani 1909a).

** Lepidozia stahlii Steph., Sp. Hepat. (Stephani) 3: 616, 1909 (Stephani 1909a).

** Lepidozia stuhlmannii Steph., Bot. Jahrb. Syst. 20 (3): 308, 1895 (Stephani 1895a).

** Lepidozia stuhlmannii var. carnosa (Steph.) Pócs, Lidia 4 (1): 22, 1997 (Lye and Pócs 1997). Bas.: Lepidozia carnosa Steph., Wiss. Ergebn. Deut. Zentr.-Afr. Exped. (1907-08), Bot. 2: 122, 1911 (Stephani 1911a).

** Lepidozia stuhlmannii subsp. pulvinata (Steph.) Pócs, Trop. Bryol. 9: 127, 1994 (Pócs 1994c). Bas.: Lepidozia pulvinata Steph., Wiss. Ergebn. Deut. Zentr.-Afr. Exped. (1907-08), Bot. 2: 121, 1911 (Stephani 1911a).

** Lepidozia subdichotoma Spruce, Trans. & Proc. Bot. Soc. Edinburgh 15: 361, 1885 (Spruce 1885).

** Lepidozia subintegra Lindenb., Syn. Hepat. 2: 201, 1845 (Gottsche et al. 1845a).

** Lepidozia subtransversa Steph., Bull. Herb. Boissier 5 (2): 95, 1897 (Stephani 1897b).

** Lepidozia subtrichodes Steph., Sp. Hepat. (Stephani) 3: 615, 1909 (Stephani 1909a).

** Lepidozia succida Mitt., Trans. Linn. Soc. London 23 (1): 57, 1860 (Mitten 1860a).

** Lepidozia supradecomposita Lindenb., Syn. Hepat. 2: 202, 1845 (Gottsche et al. 1845a).

* Lepidozia supradecomposita var. falcifolia Herzog, Mitt. Inst. Allg. Bot. Hamburg 7 (3): 191, 1931 (Herzog 1931a).

** Lepidozia suyungii C.Gao et X.L.Bai, J. Hattori Bot. Lab. 92: 192, 2002 (Gao and Bai 2002).

** Lepidozia terricola Steph., Sp. Hepat. (Stephani) 3: 585, 1909 (Stephani 1909a).

** Lepidozia triangulifolia Steph., Sp. Hepat. (Stephani) 6: 344, 1922 (Stephani 1922).

*** Lepidozia trichodes (Reinw., Blume et Nees) Nees, Syn. Hepat. 2: 203, 1845 (Gottsche et al. 1845a). Bas.: Jungermannia trichodes Reinw., Blume et Nees, Nova Acta Phys.-Med. Acad. Caes. Leop.-Carol. Nat. Cur. 12 (1): 199, 1824 [1825] (Reinwardt et al. 1824a).

* Lepidozia tricuspidata Steph., Sp. Hepat. (Stephani) 6: 344, 1922 (Stephani 1922).

** Lepidozia tunguraguae Steph., Sp. Hepat. (Stephani) 6: 345, 1922 (Stephani 1922).

** Lepidozia ubangiensis Steph., Sp. Hepat. (Stephani) 3: 561, 1909 (Stephani 1909a).

*** Lepidozia udarii S.C.Srivast., D.Kumar et D.Sharma, Cryptog. Bryol. Lichénol. 9 (3): 237, 1988 (Srivastava et al. 1988).

*** Lepidozia ulothrix (Schwägr.) Lindenb., Syn. Hepat. 2: 210, 1845 (Gottsche et al. 1845a). Bas.: Jungermannia ulothrix Schwägr., Hist. Musc. Hepat. Prodr.: 21, 1814 (Schwägrichen 1814).

** Lepidozia vitrea Steph., Bull. Herb. Boissier 5 (2): 96, 1897 (Stephani 1897b).

** Lepidozia wattsiana Steph., Sp. Hepat. (Stephani) 3: 586, 1909 (Stephani 1909a).

** Lepidozia weymouthiana Steph., J. & Proc. Roy. Soc. New South Wales 48 (1/2): 117, 1914 (Stephani and Watts 1914).

**Excluded from the genus**

* Lepidozia hexiloba Pearson, Ann. Cryptog. Exot. 4 (2): 67, 1931 (Pearson 1931a). ^126^

* Lepidozia parvistipa Taylor, London J. Bot. 5: 370, 1846 (Taylor 1846b). ^127^

*** **Neolepidozia Fulford et J.Taylor**, Brittonia 11 (2): 81, 1959 (Fulford and Taylor 1959a).

*** Neolepidozia aubertii (Jovet-Ast) E.D.Cooper, Phytotaxa 97 (2): 55, 2013 (Cooper et al. 2013). Bas.: Lepidozia aubertii Jovet-Ast, Candollea 11: 35, 1947 (Jovet-Ast 1947a).

*** Neolepidozia autoica (J.J.Engel et G.L.Merr.) E.D.Cooper, Phytotaxa 97 (2): 55, 2013 (Cooper et al. 2013). Bas.: Telaranea autoica J.J.Engel et G.L.Merr., Fieldiana, Bot. (n.ser.) 44: 124, 2004 (Engel and Smith Merrill 2004).

*** Neolepidozia capilligera (Schwägr.) Fulford et J.Taylor, Brittonia 11 (2): 84, 1959 (Fulford and Taylor 1959a). Bas.: Jungermannia capilligera Schwägr., Hist. Musc. Hepat. Prodr.: 21, 1814 (Schwägrichen 1814).

*** Neolepidozia consobrina (J.J.Engel et G.L.Merr.) E.D.Cooper, Phytotaxa 97 (2): 55, 2013 (Cooper et al. 2013). Bas.: Telaranea consobrina J.J.Engel et G.L.Merr., Novon 9 (3): 339, 1999 (Engel and Smith Merrill 1999a).

*** Neolepidozia cuneifolia (Steph.) Fulford et J.Taylor, Brittonia 11 (2): 85, 1959 (Fulford and Taylor 1959a). Bas.: Lepidozia cuneifolia Steph., Sp. Hepat. (Stephani) 3: 618, 1909 (Stephani 1909a).

*** Neolepidozia disparata (J.J.Engel et G.L.Merr.) E.D.Cooper, Phytotaxa 97 (2): 55, 2013 (Cooper et al. 2013). Bas.: Telaranea disparata J.J.Engel et G.L.Merr., Fieldiana, Bot. (n.ser.) 44: 147, 2004 (Engel and Smith Merrill 2004).

*** Neolepidozia disticha (Steph.) Fulford et J.Taylor, Brittonia 11 (2): 85, 1959 (Fulford and Taylor 1959a). Bas.: Lepidozia disticha Steph., Kungl. Svenska Vetensk.-Akad. Handl. (n.ser.) 46 (9): 62, 1911 (Stephani 1911b).

*** Neolepidozia gibbsiana (Steph.) E.D.Cooper, Phytotaxa 97 (2): 55, 2013 (Cooper et al. 2013). Bas.: Lepidozia gibbsiana Steph., Sp. Hepat. (Stephani) 6: 328, 1922 (Stephani 1922).

*** Neolepidozia heterotexta (Steph.) E.D.Cooper, Phytotaxa 97 (2): 55, 2013 (Cooper et al. 2013). Bas.: Lepidozia heterotexta Steph., Sp. Hepat. (Stephani) 6: 329, 1922 (Stephani 1922).

*** Neolepidozia hodgsoniae (J.J.Engel et G.L.Merr.) E.D.Cooper, Phytotaxa 97 (2): 55, 2013 (Cooper et al. 2013). Bas.: Telaranea hodgsoniae J.J.Engel et G.L.Merr., Phytologia 79 (3): 251, 1995 [1996] (Engel and Smith Merrill 1995).

*** Neolepidozia longitudinalis (Herzog) E.D.Cooper, Phytotaxa 97 (2): 56, 2013 (Cooper et al. 2013). Bas.: Lepidozia longitudinalis Herzog, Trans. Brit. Bryol. Soc. 1 (4): 312, 1950 (Herzog 1950a).

*** Neolepidozia mamillosa (Schiffn.) E.D.Cooper, Phytotaxa 97 (2): 56, 2013 (Cooper et al. 2013). Bas.: Lepidozia mamillosa Schiffn., Nova Acta Acad. Caes. Leop.-Carol. German. Nat. Cur. 60 (2): 254, 1893 (Schiffner 1893a).

*** Neolepidozia meridiana (E.A.Hodgs.) E.D.Cooper, Phytotaxa 97 (2): 56, 2013 (Cooper et al. 2013). Bas.: Lepidozia meridiana E.A.Hodgs., Trans. Roy. Soc. New Zealand 83 (4): 611, 1956 (Hodgson 1956).

*** Neolepidozia oligophylla (Lehm. et Lindenb.) Fulford et J.Taylor, Brittonia 11 (2): 84, 1959 (Fulford and Taylor 1959a). Bas.: Jungermannia oligophylla Lehm. et Lindenb., Nov. Stirp. Pug. 6: 26, 1834 (Lehmann 1834).

*** Neolepidozia ophiria (Gottsche) E.D.Cooper, Phytotaxa 97 (2): 56, 2013 (Cooper et al. 2013). Bas.: Lepidozia ophiria Gottsche, Sp. Hepat. (Stephani) 3: 611, 1909 (Stephani 1909a).

*** Neolepidozia palmata (J.J.Engel et G.L.Merr.) E.D.Cooper, Phytotaxa 97 (2): 56, 2013 (Cooper et al. 2013). Bas.: Telaranea palmata J.J.Engel et G.L.Merr., Novon 9 (3): 344, 1999 (Engel and Smith Merrill 1999a).

*** Neolepidozia paludicola (E.A.Hodgs.) E.D.Cooper, Phytotaxa 97 (2): 56, 2013 (Cooper et al. 2013). Bas.: Lepidozia meridiana var. paludicola E.A.Hodgs., Trans. Roy. Soc. New Zealand 83 (4): 611, 1956 (Hodgson 1956).

*** Neolepidozia papulosa (Steph.) Fulford et J.Taylor, Brittonia 11 (2): 85, 1959 (Fulford and Taylor 1959a). Bas.: Lepidozia papulosa Steph., Sp. Hepat. (Stephani) 3: 609, 1909 (Stephani 1909a).

*** Neolepidozia parvifolia (Steph.) Fulford et J.Taylor, Brittonia 11 (2): 85, 1959 (Fulford and Taylor 1959a). Bas.: Lepidozia parvifolia Steph., Sp. Hepat. (Stephani) 6: 337, 1922 (Stephani 1922).

*** Neolepidozia patentissima (Hook.f. et Taylor) E.D.Cooper, Phytotaxa 97 (2): 56, 2013 (Cooper et al. 2013). Bas.: Jungermannia patentissima Hook.f. et Taylor, London J. Bot. 3: 286 [386], 1844 (Hooker and Taylor 1844a).

*** Neolepidozia patentissima var. ampliata (J.J.Engel et G.L.Merr.) E.D.Cooper, Phytotaxa 97 (2): 56, 2013 (Cooper et al. 2013). Bas.: Telaranea patentissima var. ampliata J.J.Engel et G.L.Merr., Fieldiana, Bot. (n.ser.) 44: 48, 2004 (Engel and Smith Merrill 2004).

*** Neolepidozia patentissima var. zebrina (J.J.Engel et G.L.Merr.) E.D.Cooper, Phytotaxa 97 (2): 57, 2013 (Cooper et al. 2013). Bas.: Telaranea patentissima var. zebrina J.J.Engel et G.L.Merr., Fieldiana, Bot. (n.ser.) 44: 50, 2004 (Engel and Smith Merrill 2004).

*** Neolepidozia pennata (J.J.Engel et G.L.Merr.) E.D.Cooper, Phytotaxa 97 (2): 57, 2013 (Cooper et al. 2013). Bas.: Telaranea pennata J.J.Engel et G.L.Merr., Phytologia 79 (3): 252, 1995 [1996] (Engel and Smith Merrill 1995).

*** Neolepidozia planifolia (Steph.) E.D.Cooper, Phytotaxa 97 (2): 57, 2013 (Cooper et al. 2013). Bas.: Lepidozia planifolia Steph., Sp. Hepat. (Stephani) 3: 629, 1909 (Stephani 1909a).

*** Neolepidozia praenitens (Lehm. et Lindenb.) E.D.Cooper, Phytotaxa 97 (2): 57, 2013 (Cooper et al. 2013). Bas.: Jungermannia praenitens Lehm. et Lindenb., Nov. Stirp. Pug. 6: 27, 1834 (Lehmann 1834).

*** Neolepidozia praenitens var. dentifolia (J.J.Engel et G.L.Merr.) E.D.Cooper, Phytotaxa 97 (2): 57, 2013 (Cooper et al. 2013). Bas.: Telaranea praenitens var. dentifolia J.J.Engel et G.L.Merr., Phytologia 79 (3): 253, 1995 [1996] (Engel and Smith Merrill 1995).

*** Neolepidozia quadristipula (Steph.) E.D.Cooper, Phytotaxa 97 (2): 57, 2013 (Cooper et al. 2013). Bas.: Lepidozia quadristipula Steph., J. & Proc. Roy. Soc. New South Wales 48 (1/2): 115, 1914 (Stephani and Watts 1914).

*** Neolepidozia rectangularis (R.M.Schust.) E.D.Cooper, Phytotaxa 97 (2): 57, 2013 (Cooper et al. 2013). Bas.: Telaranea rectangularis R.M.Schust., Phytologia 39 (4): 241, 1978 (Schuster 1978a).

*** Neolepidozia seriatitexta (Steph.) Fulford, Mem. New York Bot. Gard. 11 (2): 215, 1966 (Fulford 1966). Bas.: Lepidozia seriatitexta Steph., Bih. Kongl. Svenska Vetensk.-Akad. Handl. 26 (III, 6): 53, 1900 (Stephani 1900b).

*** Neolepidozia tetrapila (Hook.f. et Taylor) E.D.Cooper, Phytotaxa 97 (2): 57, 2013 (Cooper et al. 2013). Bas.: Lepidozia tetrapila Hook.f. et Taylor, London J. Bot. 5: 370, 1846 (Taylor 1846b).

*** Neolepidozia tetrapila var. cancellata (Colenso) E.D.Cooper, Phytotaxa 97 (2): 57, 2013 (Cooper et al. 2013). Bas.: Lepidozia cancellata Colenso, Trans. & Proc. New Zealand Inst. 18: 244, 1886 (Colenso 1886b).

*** Neolepidozia tetrapila var. roseana (Steph.) E.D.Cooper, Phytotaxa 97 (2): 58, 2013 (Cooper et al. 2013). Bas.: Lepidozia roseana Steph., Sp. Hepat. (Stephani) 3: 590, 1909 (Stephani 1909a).

*** Neolepidozia tridactylis (Lehm. et Lindenb.) E.D.Cooper, Phytotaxa 97 (2): 58, 2013 (Cooper et al. 2013). Bas.: Jungermannia tridactylis Lehm. et Lindenb., Nov. Stirp. Pug. 4: 41, 1832 (Lehmann 1832).

*** Neolepidozia trifida (Steph.) E.D.Cooper, Phytotaxa 97 (2): 58, 2013 (Cooper et al. 2013). Bas.: Lepidozia trifida Steph., Wiss. Ergebn. Deut. Zentr.-Afr. Exped. (1907-08), Bot. 2: 120, 1911 (Stephani 1911a).

*** Neolepidozia verruculosa (J.J.Engel et G.L.Merr.) E.D.Cooper, Phytotaxa 97 (2): 58, 2013 (Cooper et al. 2013). Bas.: Telaranea verruculosa J.J.Engel et G.L.Merr., Fieldiana, Bot. (n.ser.) 44: 197, 2004 (Engel and Smith Merrill 2004).

*** Neolepidozia wallichiana (Gottsche) Fulford et J.Taylor, Brittonia 11 (2): 84, 1959 (Fulford and Taylor 1959a). Bas.: Lepidozia wallichiana Gottsche, Syn. Hepat. 2: 204, 1845 (Gottsche et al. 1845a).

*** **Tricholepidozia (R.M.Schust.) E.D.Cooper**, Phytotaxa 97 (2): 58, 2013 (Cooper et al. 2013). Bas.: Telaranea subg. Tricholepidozia R.M.Schust., J. Hattori Bot. Lab. 26: 256, 1963 (Schuster 1963b).

*** Tricholepidozia chaetocarpa (Pearson) E.D.Cooper, Phytotaxa 97 (2): 58, 2013 (Cooper et al. 2013). Bas.: Lepidozia chaetocarpa Pearson, J. Linn. Soc., Bot. 46 (305): 27, 1922 (Pearson 1922b).

*** Tricholepidozia fernandeziensis (Steph.) E.D.Cooper, Phytotaxa 97 (2): 58, 2013 (Cooper et al. 2013). Bas.: Lepidozia fernandeziensis Steph., Kungl. Svenska Vetensk.-Akad. Handl. (n.ser.) 46 (9): 63, 1911 (Stephani 1911b).

*** Tricholepidozia ferruginea (J.J.Engel et G.L.Merr.) E.D.Cooper, Phytotaxa 97 (2): 58, 2013 (Cooper et al. 2013). Bas.: Telaranea ferruginea J.J.Engel et G.L.Merr., Fieldiana, Bot. (n.ser.) 44: 159, 2004 (Engel and Smith Merrill 2004).

*** Tricholepidozia fissifolia (Steph.) E.D.Cooper, Phytotaxa 97 (2): 58, 2013 (Cooper et al. 2013). Bas.: Lepidozia fissifolia Steph., Sp. Hepat. (Stephani) 3: 610, 1909 (Stephani 1909a).

*** Tricholepidozia jowettiana (H.A.Mill.) E.D.Cooper, Phytotaxa 97 (2): 59, 2013 (Cooper et al. 2013). Bas.: Telaranea jowettiana H.A.Mill., J. Bryol. 14 (2): 235, 1986 (Miller 1986).

*** Tricholepidozia kaindina (Grolle) E.D.Cooper, Phytotaxa 97 (2): 59, 2013 (Cooper et al. 2013). Bas.: Telaranea kaindina Grolle, J. Hattori Bot. Lab. 31: 9, 1968 (Grolle 1968a).

*** Tricholepidozia kogiana (Steph.) E.D.Cooper, Phytotaxa 97 (2): 59, 2013 (Cooper et al. 2013). Bas.: Lepidozia kogiana Steph., Sp. Hepat. (Stephani) 6: 332, 1922 (Stephani 1922).

*** Tricholepidozia lawesii (Steph.) E.D.Cooper, Phytotaxa 97 (2): 59, 2013 (Cooper et al. 2013). Bas.: Lepidozia lawesii Steph., Hedwigia 28 (4): 264, 1889 (Stephani 1889c).

*** Tricholepidozia leratii (Steph.) E.D.Cooper, Phytotaxa 167 (2): 218, 2014 (Cooper et al. 2014). Bas.: Lepidozia leratii Steph., Sp. Hepat. (Stephani) 6: 333, 1922 (Stephani 1922).

*** Tricholepidozia lindenbergii (Gottsche) E.D.Cooper, Phytotaxa 97 (2): 59, 2013 (Cooper et al. 2013). Bas.: Lepidozia lindenbergii Gottsche, Syn. Hepat. 2: 213, 1845 (Gottsche et al. 1845a).

*** Tricholepidozia lindenbergii var. complanata (J.J.Engel et G.L.Merr.) E.D.Cooper, Phytotaxa 97 (2): 59, 2013 (Cooper et al. 2013). Bas.: Telaranea lindenbergii var. complanata J.J.Engel et G.L.Merr., Phytologia 79 (3): 252, 1995 [1996] (Engel and Smith Merrill 1995).

*** Tricholepidozia lindenbergii var. mellea (J.J.Engel et G.L.Merr.) E.D.Cooper, Phytotaxa 97 (2): 59, 2013 (Cooper et al. 2013). Bas.: Telaranea lindenbergii var. mellea J.J.Engel et G.L.Merr., Phytologia 79 (3): 252, 1995 [1996] (Engel and Smith Merrill 1995).

*** Tricholepidozia lindenbergii var. papillata (J.J.Engel et G.L.Merr.) E.D.Cooper, Phytotaxa 97 (2): 59, 2013 (Cooper et al. 2013). Bas.: Telaranea lindenbergii var. papillata J.J.Engel et G.L.Merr., Fieldiana, Bot. (n.ser.) 44: 83, 2004 (Engel and Smith Merrill 2004).

*** Tricholepidozia marginata (J.J.Engel et G.L.Merr.) E.D.Cooper, Phytotaxa 97 (2): 59, 2013 (Cooper et al. 2013). Bas.: Telaranea marginata J.J.Engel et G.L.Merr., Fieldiana, Bot. (n.ser.) 44: 166, 2004 (Engel and Smith Merrill 2004).

*** Tricholepidozia martinii (E.A.Hodgs.) E.D.Cooper, Phytotaxa 97 (2): 59, 2013 (Cooper et al. 2013). Bas.: Lepidozia martinii E.A.Hodgs., Trans. Roy. Soc. New Zealand 83 (4): 602, 1956 (Hodgson 1956).

*** Tricholepidozia melanesica (H.A.Mill.) E.D.Cooper, Phytotaxa 97 (2): 60, 2013 (Cooper et al. 2013). Bas.: Telaranea melanesica H.A.Mill., J. Bryol. 14 (2): 237, 1986 (Miller 1986).

*** Tricholepidozia neesii (Lindenb.) E.D.Cooper, Phytotaxa 97 (2): 60, 2013 (Cooper et al. 2013). Bas.: Lepidozia neesii Lindenb., Syn. Hepat. 2: 212, 1845 (Gottsche et al. 1845a).

*** Tricholepidozia octoloba (Del Ros.) E.D.Cooper, Phytotaxa 97 (2): 60, 2013 (Cooper et al. 2013). Bas.: Telaranea octoloba Del Ros., Philipp. J. Sci. 100 (3/4): 239, 1971 (Del Rosario 1971).

*** Tricholepidozia plumulosa (Lehm. et Lindenb.) E.D.Cooper, Phytotaxa 97 (2): 60, 2013 (Cooper et al. 2013). Bas.: Jungermannia plumulosa Lehm. et Lindenb., Nov. Stirp. Pug. 6: 30, 1834 (Lehmann 1834).

*** Tricholepidozia pulcherrima (Steph.) E.D.Cooper, Phytotaxa 97 (2): 60, 2013 (Cooper et al. 2013). Bas.: Lepidozia pulcherrima Steph., Sp. Hepat. (Stephani) 3: 600, 1909 (Stephani 1909a).

*** Tricholepidozia pulcherrima var. mooreana (Steph.) E.D.Cooper, Phytotaxa 97 (2): 60, 2013 (Cooper et al. 2013). Bas.: Lepidozia mooreana Steph., Sp. Hepat. (Stephani) 3: 585, 1909 (Stephani 1909a).

*** Tricholepidozia quadriseta (Steph.) E.D.Cooper, Phytotaxa 97 (2): 60, 2013 (Cooper et al. 2013). Bas.: Lepidozia quadriseta Steph., Sp. Hepat. (Stephani) 3: 582, 1909 (Stephani 1909a).

*** Tricholepidozia remotifolia (E.A.Hodgs.) E.D.Cooper, Phytotaxa 97 (2): 60, 2013 (Cooper et al. 2013). Bas.: Telaranea remotifolia E.A.Hodgs., Rec. Domin. Mus. 4 (11): 107, 1962 (Hodgson 1962a).

*** Tricholepidozia semperiana (Steph.) E.D.Cooper, Phytotaxa 97 (2): 60, 2013 (Cooper et al. 2013). Bas.: Lepidozia semperiana Steph., Sp. Hepat. (Stephani) 3: 612, 1909 (Stephani 1909a).

*** Tricholepidozia tetradactyla (Hook.f. et Taylor) E.D.Cooper, Phytotaxa 97 (2): 61, 2013 (Cooper et al. 2013). Bas.: Jungermannia tetradactyla Hook.f. et Taylor, London J. Bot. 3: 286 [386], 1844 (Hooker and Taylor 1844a).

*** Tricholepidozia trichocoleoides (Herzog) E.D.Cooper, Phytotaxa 97 (2): 61, 2013 (Cooper et al. 2013). Bas.: Lepidozia trichocoleoides Herzog, Trans. Brit. Bryol. Soc. 1 (4): 314, 1950 (Herzog 1950a).

######### ** Micropterygioideae Grolle

** **Micropterygium Gottsche, Lindenb. et Nees**, Syn. Hepat. 2: 233, 1845 (Gottsche et al. 1845a).

** Micropterygium angustistipulum Spruce, Trans. & Proc. Bot. Soc. Edinburgh 15: 385, 1885 (Spruce 1885).

*** Micropterygium bialatum Fulford, Mem. New York Bot. Gard. 11 (2): 268, 1966 (Fulford 1966).

*** Micropterygium bolivarense Fulford, Mem. New York Bot. Gard. 11 (2): 263, 1966 (Fulford 1966).

*** Micropterygium campanense Spruce, Hedwigia 73 (3/4): 157, 1933 (Reimers 1933).

*** Micropterygium carinatum (Grev.) Reimers, Hedwigia 76 (3): 166, 1936 (Reimers 1936). Bas.: Jungermannia carinata Grev., Ann. Lyceum Nat. Hist. New York 1 (2): 276, 1825 (Greville 1825).

*** Micropterygium conchifolium Reimers, Hedwigia 73 (3/4): 155, 1933 (Reimers 1933).

*** Micropterygium duidae Reimers, Hedwigia 73 (3/4): 147, 1933 (Reimers 1933).

** Micropterygium exalatum Steph., Sp. Hepat. (Stephani) 3: 547, 1909 (Stephani 1909a).

** Micropterygium grandistipulum Steph., Trans. Linn. Soc. London, Bot. 6 (1): 98, 1901 (Stephani 1901e).

** Micropterygium laeve H.Rob., Bol. Soc. Venez. Ci. Nat. 32 (132/133): 252, 1976 (Robinson 1976b).

** Micropterygium lechleri Reimers, Hedwigia 73 (3/4): 184, 1933 (Reimers 1933).

*** Micropterygium leiophyllum Spruce, Trans. & Proc. Bot. Soc. Edinburgh 15: 386, 1885 (Spruce 1885).

** Micropterygium longicellulatum Uribe et E.L.Linares, Phytotaxa 213 (3): 297, 2015 (Uribe and Linares 2015).

** Micropterygium parvistipulum Spruce, Trans. & Proc. Bot. Soc. Edinburgh 15: 383, 1885 (Spruce 1885).

*** Micropterygium pterygophyllum (Nees) Trevis., Mem. Reale Ist. Lombardo Sci. (Ser. 3), C. Sci. Mat. 4 (13): 413, 1877 (Trevisan 1877). Bas.: Jungermannia pterygophylla Nees, Fl. Bras. (Martius) 1 (1): 377, 1833 (Nees 1833a).

* Micropterygium pterygophyllum var. lancifolium Reimers, Hedwigia 73 (3/4): 176, 1933 (Reimers 1933).

** Micropterygium reimersianum Herzog, Hedwigia 81 (5/6): 226, 1943 (Herzog 1943b).

*** Micropterygium steyermarkii Fulford, Mem. New York Bot. Gard. 11 (2): 270, 1966 (Fulford 1966).

*** Micropterygium tatei Reimers, Hedwigia 73 (3/4): 150, 1933 (Reimers 1933).

*** Micropterygium tenax (Steph.) Grolle, J. Bryol. 10 (3): 265, 1979 (Grolle 1979b). Bas.: Harpalejeunea tenax Steph., Trans. Linn. Soc. London, Bot. 6 (1): 100, 1901 (Stephani 1901e).

*** Micropterygium trachyphyllum Reimers, Hedwigia 73 (3/4): 186, 1933 (Reimers 1933). ^128^

* Micropterygium trachyphyllum var. brasiliense Reimers, Hedwigia 73 (3/4): 195, 1933 (Reimers 1933).

* Micropterygium trachyphyllum var. cubense Reimers, Hedwigia 73 (3/4): 188, 1933 (Reimers 1933).

* Micropterygium trachyphyllum var. guadeloupense Reimers, Hedwigia 73 (3/4): 190, 1933 (Reimers 1933).

* Micropterygium trachyphyllum var. jamaicense Reimers, Hedwigia 73 (3/4): 190, 1933 (Reimers 1933).

*** Micropterygium tumidulum Fulford, Mem. New York Bot. Gard. 11 (2): 272, 1966 (Fulford 1966).

** **Mytilopsis Spruce**, Cephalozia: 90, 1882 (Spruce 1882).

*** Mytilopsis albifrons Spruce, Cephalozia: 91, 1882 (Spruce 1882).

######### ** Protocephalozioideae R.M.Schust.

** **Protocephalozia (Spruce) K.I.Goebel**, Flora 77 (2): 83, 1893 (Goebel 1893b). Bas.: Cephalozia subg. Protocephalozia Spruce, Cephalozia: 24, 1882 (Spruce 1882).

*** Protocephalozia ephemeroides (Spruce) K.I.Goebel, Flora 77 (2): 83, 1893 (Goebel 1893b). Bas.: Cephalozia ephemeroides Spruce, Cephalozia: 24, 1882 (Spruce 1882).

######### *** Zoopsidoideae R.M.Schust.

** **Amazoopsis J.J.Engel et G.L.Merr.**, Fieldiana, Bot. (n.ser.) 44: 242, 2004 (Engel and Smith Merrill 2004).

*** Amazoopsis diplopoda (Pócs) J.J.Engel et G.L.Merr., Fieldiana, Bot. (n.ser.) 44: 245, 2004 (Engel and Smith Merrill 2004). Bas.: Arachniopsis diplopoda Pócs, Proc. Third Meeting Bryol. C. & E. Europe: 114, 1984 (Pócs 1984b).

*** Amazoopsis dissotricha (Spruce) J.J.Engel et G.L.Merr., Fieldiana, Bot. (n.ser.) 44: 247, 2004 (Engel and Smith Merrill 2004). Bas.: Arachniopsis dissotricha Spruce, Cephalozia: 86, 1882 (Spruce 1882).

*** Amazoopsis gracilis J.J.Engel et G.L.Merr., Fieldiana, Bot. (n.ser.) 44: 246, 2004 (Engel and Smith Merrill 2004).

* **Hyalolepidozia S.W.Arnell ex Grolle**, Rev. Bryol. Lichénol. 32 (3/4): 179, 1963 [1964] (Grolle 1963b). Based on: Hyalolepidozia S.W.Arnell, Bot. Not. 115: 203, 1962 (Arnell 1962a).

** Hyalolepidozia bicuspidata (C.Massal.) S.W.Arnell ex Grolle, Rev. Bryol. Lichénol. 32 (3/4): 179, 1963 [1964] (Grolle 1963b). Bas.: Lepidozia bicuspidata C.Massal., Nuovo Giorn. Bot. Ital. 17 (3): 239, 1885 (Massalongo 1885).

* **Monodactylopsis (R.M.Schust.) R.M.Schust.**, Nova Hedwigia 56 (1/2): 45, 1993 (Schuster 1993a). Bas.: Arachniopsis subg. Monodactylopsis R.M.Schust., Nova Hedwigia 10 (1/2): 24, 1965 (Schuster 1965b).

** Monodactylopsis monodactyla (Spruce) R.M.Schust., Nova Hedwigia 69 (3/4): 523, 1999 (Schuster 1999a). Bas.: Cephalozia monodactyla Spruce, Cephalozia: 28, 1882 (Spruce 1882).

** **Neogrollea E.A.Hodgs.**, Trans. Roy. Soc. New Zealand, Bot. 3 (4): 70, 1965 (Hodgson 1965).

*** Neogrollea notabilis E.A.Hodgs., Trans. Roy. Soc. New Zealand, Bot. 3 (4): 70, 1965 (Hodgson 1965).

* **Odontoseries Fulford**, Mem. New York Bot. Gard. 11 (3): 364, 1968 (Fulford 1968).

* Odontoseries chimantana Fulford, Mem. New York Bot. Gard. 11 (3): 366, 1968 (Fulford 1968).

*** **Paracromastigum Fulford et J.Taylor**, Brittonia 13 (4): 336, 1961 (Fulford and Taylor 1961).

*** Paracromastigum denticulatum (Steph.) E.D.Cooper, Phytotaxa 97 (2): 61, 2013 (Cooper et al. 2013). Bas.: Lembidium denticulatum Steph., Sp. Hepat. (Stephani) 6: 444, 1924 (Stephani 1924).

*** Paracromastigum drucei (R.M.Schust.) R.M.Schust., J. Hattori Bot. Lab. 38: 700, 1974 (Schuster and Engel 1974). Bas.: Pseudocephalozia drucei R.M.Schust., Nova Hedwigia 10 (1/2): 22, 1965 (Schuster 1965b).

*** Paracromastigum dusenii (Steph.) R.M.Schust., J. Hattori Bot. Lab. 38: 700, 1974 (Schuster and Engel 1974). Bas.: Alobiella dusenii Steph., Bih. Kongl. Svenska Vetensk.-Akad. Handl. 26 (III, 6): 48, 1900 (Stephani 1900b).

** Paracromastigum fiordlandiae R.M.Schust. et J.J.Engel, Brittonia 48 (2): 167, 1996 (Schuster and Engel 1996).

*** Paracromastigum furcifolium (Steph.) R.M.Schust., J. Hattori Bot. Lab. 26: 276, 1963 (Schuster 1963b). Bas.: Cephalozia furcifolia Steph., Bull. Herb. Boissier (sér. 2) 8 (7): 485 (315), 1908 (Stephani 1908f).

** Paracromastigum granatense (Gottsche) R.M.Schust., J. Hattori Bot. Lab. 48: 341, 1980 (Schuster 1980a). Bas.: Lepidozia granatensis Gottsche, Ann. Sci. Nat. Bot. (sér. 5) 1: 139, 1864 (Gottsche 1864).

** Paracromastigum longiscyphum (Taylor) R.M.Schust. et J.J.Engel, Brittonia 48 (2): 167, 1996 (Schuster and Engel 1996). Bas.: Jungermannia longiscypha Taylor, London J. Bot. 5: 280, 1846 (Taylor 1846a).

** Paracromastigum macrostipum (Steph.) R.M.Schust., J. Hattori Bot. Lab. 26: 276, 1963 (Schuster 1963b). Bas.: Cephalozia macrostipa Steph., Hedwigia 32 (5): 315, 1893 (Stephani 1893d).

** Paracromastigum micromera (Spruce) R.M.Schust., J. Hattori Bot. Lab. 26: 276, 1963 (Schuster 1963b). Bas.: Cephalozia micromera Spruce, Cephalozia: 32, 1882 (Spruce 1882).

** Paracromastigum microphyllum (R.M.Schust. ex J.J.Engel) E.D.Cooper, Phytotaxa 97 (2): 61, 2013 (Cooper et al. 2013). Bas.: Hyalolepidozia microphylla R.M.Schust. ex J.J.Engel, Novon 17 (3): 310, 2007 (Engel 2007).

*** Paracromastigum pachyrhizum (Nees) Fulford, Mem. New York Bot. Gard. 11 (3): 390, 1968 (Fulford 1968). Bas.: Jungermannia pachyrhiza Nees, Fl. Bras. (Martius) 1 (1): 339, 1833 (Nees 1833a).

** Paracromastigum ryszardii Váňa, Bedn.-Ochyra et Cykowska, Nova Hedwigia 89 (1/2): 122, 2009 (Váňa et al. 2009).

** Paracromastigum stipulatum (Herzog) Fulford, Mem. New York Bot. Gard. 11 (3): 390, 1968 (Fulford 1968). Bas.: Cephalozia stipulata Herzog, Memoranda Soc. Fauna Fl. Fennica 27: 105, 1952 (Herzog 1952c).

** Paracromastigum subsimplex (Steph.) Fulford et J.Taylor, Brittonia 13 (4): 336, 1961 (Fulford and Taylor 1961). Bas.: Lepidozia subsimplex Steph., Kungl. Svenska Vetensk.-Akad. Handl. (n.ser.) 46 (9): 66, 1911 (Stephani 1911b).

** Paracromastigum succulentum (Sim) J.J.Engel et G.L.Merr., Bryologist 104 (1): 151, 2001 (Engel and Smith Merrill 2001). Bas.: Lepidozia succulenta Sim, Trans. Roy. Soc. South Africa 15 (1): 90, 1926 (Sim 1926).

** Paracromastigum tristanianum (R.M.Schust.) J.J.Engel et R.M.Schust., J. Hattori Bot. Lab. 38: 700, 1974 (Schuster and Engel 1974). Bas.: Pseudocephalozia tristaniana R.M.Schust., Nova Hedwigia 10 (1/2): 23, 1965 (Schuster 1965b).

** Paracromastigum vastilobum (Steph.) J.J.Engel et G.L.Merr., Fieldiana, Bot. (n.ser.) 44: 255, 2004 (Engel and Smith Merrill 2004). Bas.: Lepidozia vastiloba Steph., Sp. Hepat. (Stephani) 3: 581, 1909 (Stephani 1909a).

** **Psiloclada Mitt.**, Bot. antarct. voy. II (Fl. Nov.-Zel. 2): 143, 1854 (Mitten 1854).

*** Psiloclada clandestina Mitt., Bot. antarct. voy. II (Fl. Nov.-Zel. 2): 143, 1854 (Mitten 1854).

** Psiloclada clandestina subsp. melanesica R.M.Schust., J. Hattori Bot. Lab. 48: 411, 1980 (Schuster 1980a).

** Psiloclada clandestina subsp. spinosa (S.W.Arnell) R.M.Schust., J. Hattori Bot. Lab. 48: 410, 1980 (Schuster 1980a). Bas.: Lepidozia spinosa S.W.Arnell, Bot. Not. 107: 427, 1954 (Arnell 1954c).

** **Pteropsiella Spruce**, J. Bot. 14: 161, 1876 (Spruce 1876b).

*** Pteropsiella frondiformis Spruce, J. Bot. 14: 161, 1876 (Spruce 1876b).

** Pteropsiella metzgeriiformis R.M.Schust., Nova Hedwigia 10 (1/2): 25, 1965 (Schuster 1965b).

** **Telaranea Spruce ex Schiffn.**, Hepat. (Engl.-Prantl): 103, 1893 (Schiffner 1893b) nom. conserv. Based on: Telaranea Spruce, Trans. & Proc. Bot. Soc. Edinburgh 15: 365, 1885 (Spruce 1885). ^129^

* **sect. Telaranea**

*** Telaranea apiahyna (Steph.) Fulford, Brittonia 15 (1): 71, 1963 (Fulford 1963). Bas.: Lepidozia apiahyna Steph., Sp. Hepat. (Stephani) 3: 572, 1909 (Stephani 1909a).

*** Telaranea bicruris (Steph.) M.Howe, Bull. Torrey Bot. Club 29 (5): 287, 1902 (Howe 1902). Bas.: Lepidozia bicruris Steph., Hedwigia 24 (4): 166, 1885 (Stephani 1885f).

*** Telaranea blepharostoma (Steph.) Fulford, Brittonia 15 (1): 73, 1963 (Fulford 1963). Bas.: Lepidozia blepharostoma Steph., Bih. Kongl. Svenska Vetensk.-Akad. Handl. 26 (III, 17): 22, 1901 (Stephani 1901b).

*** Telaranea breviseta (Herzog) J.J.Engel et G.L.Merr., Fieldiana, Bot. (n.ser.) 44: 131, 2004 (Engel and Smith Merrill 2004). Bas.: Lepidozia sejuncta var. breviseta Herzog, Nat. Hist. Juan Fernandez (Botany) 2 (5): 723, 1942 (Herzog 1942a).

*** Telaranea chaetophylla (Spruce) Schiffn., Hepat. (Engl.-Prantl): 103, 1893 (Schiffner 1893b). Bas.: Lepidozia chaetophylla Spruce, Trans. & Proc. Bot. Soc. Edinburgh 15: 365, 1885 (Spruce 1885).

*** Telaranea europaea J.J.Engel et G.L.Merr., Fieldiana, Bot. (n.ser.) 44: 150, 2004 (Engel and Smith Merrill 2004).

*** Telaranea fragilis Mizut., J. Hattori Bot. Lab. 40: 449, 1976 (Mizutani 1976a).

*** Telaranea granulata J.J.Engel et G.L.Merr., Fieldiana, Bot. (n.ser.) 44: 103, 2004 (Engel and Smith Merrill 2004).

*** Telaranea longifolia (M.Howe) J.J.Engel et G.L.Merr., Fieldiana, Bot. (n.ser.) 44: 163, 2004 (Engel and Smith Merrill 2004). Bas.: Telaranea nematodes var. longifolia M.Howe, Bull. Torrey Bot. Club 29 (5): 286, 1902 (Howe 1902).

*** Telaranea nematodes (Gottsche ex Austin) M.Howe, Bull. Torrey Bot. Club 29 (5): 284, 1902 (Howe 1902). Bas.: Cephalozia nematodes Gottsche ex Austin, Bull. Torrey Bot. Club 6 (52): 302, 1879 (Austin 1879).

*** Telaranea panchoi Del Ros., Philipp. J. Sci. 100 (3/4): 238, 1971 (Del Rosario 1971).

*** Telaranea pellucida J.J.Engel et G.L.Merr., Fieldiana, Bot. (n.ser.) 44: 179, 2004 (Engel and Smith Merrill 2004).

*** Telaranea pseudozoopsis (Herzog) Fulford, Brittonia 15 (1): 71, 1963 (Fulford 1963). Bas.: Lepidozia pseudozoopsis Herzog, Nat. Hist. Juan Fernandez (Botany) 2 (5): 723, 1942 (Herzog 1942a).

*** Telaranea redacta (Steph.) J.J.Engel et G.L.Merr., Fieldiana, Bot. (n.ser.) 44: 186, 2004 (Engel and Smith Merrill 2004). Bas.: Lepidozia redacta Steph., Wiss. Ergebn. Deut. Zentr.-Afr. Exped. (1907-08), Bot. 2: 119, 1911 (Stephani 1911a).

*** Telaranea rosarioana H.A.Mill., J. Bryol. 14 (2): 240, 1986 (Miller 1986).

*** Telaranea setosa J.J.Engel et G.L.Merr., Fieldiana, Bot. (n.ser.) 44: 192, 2004 (Engel and Smith Merrill 2004).

*** Telaranea trisetosa (Steph.) Grolle, J. Hattori Bot. Lab. 29: 280, 1966 (Grolle 1966g). Bas.: Lepidozia trisetosa Steph., Sp. Hepat. (Stephani) 3: 607, 1909 (Stephani 1909a).

* **sect. Tenuifoliae (R.M.Schust.) J.J.Engel et G.L.Merr.**, Fieldiana, Bot. (n.ser.) 44: 112, 2004 (Engel and Smith Merrill 2004). Bas.: Arachniopsis sect. Tenuifoliae R.M.Schust., Beih. Nova Hedwigia 118: 461, 2000 (Schuster 2000a).

*** Telaranea anomala R.M.Schust. ex J.J.Engel et G.L.Merr., Fieldiana, Bot. (n.ser.) 44: 121, 2004 (Engel and Smith Merrill 2004).

** Telaranea bischleriana Pócs, Acta Bot. Hung. 48 (1/2): 120, 2006 (Pócs 2006c).

*** Telaranea coactilis (Spruce) J.J.Engel et G.L.Merr., Fieldiana, Bot. (n.ser.) 44: 140, 2004 (Engel and Smith Merrill 2004). Bas.: Arachniopsis coactilis Spruce, Cephalozia: 85, 1882 (Spruce 1882).

*** Telaranea confervoides J.J.Engel et G.L.Merr., Fieldiana, Bot. (n.ser.) 44: 143, 2004 (Engel and Smith Merrill 2004). Based on: Arachniopsis pecten var. confervoides R.M.Schust., Beih. Nova Hedwigia 118: 455, 2000 (Schuster 2000a), *nom. inval*.

*** Telaranea diacantha (Mont.) J.J.Engel et G.L.Merr., Fieldiana, Bot. (n.ser.) 44: 145, 2004 (Engel and Smith Merrill 2004). Bas.: Jungermannia diacantha Mont., Ann. Sci. Nat. Bot. (sér. 4) 5: 349, 1856 (Montagne 1856c).

*** Telaranea herzogii (E.A.Hodgs.) E.A.Hodgs., Rec. Domin. Mus. 4 (11): 106, 1962 (Hodgson 1962a). Bas.: Lepidozia herzogii E.A.Hodgs., Trans. & Proc. Roy. Soc. New Zealand 78 (4): 500, 1950 (Martin 1950).

*** Telaranea inaequalis R.M.Schust. ex J.J.Engel et G.L.Merr., Fieldiana, Bot. (n.ser.) 44: 117, 2004 (Engel and Smith Merrill 2004).

** Telaranea maorensis Pócs, Acta Bot. Hung. 48 (1/2): 124, 2006 (Pócs 2006c).

*** Telaranea microstipulata R.M.Schust., Phytologia 39 (4): 241, 1978 (Schuster 1978a).

*** Telaranea monocera (Mitt. ex R.M.Schust. et Grolle) J.J.Engel et G.L.Merr., Fieldiana, Bot. (n.ser.) 44: 168, 2004 (Engel and Smith Merrill 2004). Bas.: Arachniopsis monocera Mitt. ex R.M.Schust. et Grolle, Nova Hedwigia 10 (1/2): 25, 1965 (Schuster 1965b).

*** Telaranea pecten (Spruce) J.J.Engel et G.L.Merr., Fieldiana, Bot. (n.ser.) 44: 178, 2004 (Engel and Smith Merrill 2004). Bas.: Arachniopsis pecten Spruce, Cephalozia: 85, 1882 (Spruce 1882).

*** Telaranea sejuncta (Ångstr.) S.W.Arnell, Bot. Not. 110 (1): 18, 1957 (Arnell 1957a). Bas.: Blepharostoma sejunctum Ångstr., Öfvers. Kongl. Vetensk.-Akad. Förh. 33 (7): 78, 1876 [1877] (Ångström 1876).

*** Telaranea tenuifolia J.J.Engel et G.L.Merr., Fieldiana, Bot. (n.ser.) 44: 193, 2004 (Engel and Smith Merrill 2004). Based on: Arachniopsis tenuifolia R.M.Schust., Beih. Nova Hedwigia 118: 461, 2000 (Schuster 2000a), *nom. inval*.

***Incertae sedis***

** Telaranea azorica (H.Buch et Perss.) Pócs in Schumacker et Váňa, Identif. keys liverw. hornw. Europe: 160, 2005 (Schumacker and Váňa 2005). Bas.: Lepidozia azorica H.Buch et Perss., Bryophyt. Azoren Madeira: 4, 1941 (Buch and Persson 1941).

** Telaranea indica (S.C.Srivast. et P.K.Verma) A.E.D.Daniels et P.Daniel, Bull. Bot. Surv. India 49 (1/4): 231, 2007 (Daniels and Daniel 2007). Bas.: Arachniopsis indica S.C.Srivast. et P.K.Verma, Natl. Acad. Sci. Lett. 27 (7/8): 270, 2004 [2006] (Srivastava and Verma 2004).

** Telaranea major (Herzog) J.J.Engel et G.L.Merr., Fieldiana, Bot. (n.ser.) 44: 165, 2004 (Engel and Smith Merrill 2004). Bas.: Arachniopsis major Herzog, Trans. Brit. Bryol. Soc. 1 (4): 294, 1950 (Herzog 1950a).

** **Zoopsidella R.M.Schust.**, Nova Hedwigia 10 (1/2): 24, 1965 (Schuster 1965b).

** Zoopsidella antillana (Steph.) H.Rob., Bol. Soc. Venez. Ci. Nat. 32 (132/133): 254, 1976 (Robinson 1976b). Bas.: Zoopsis antillana Steph., Bull. Herb. Boissier (sér. 2) 8 (4): 268 (282), 1908 (Stephani 1908j).

** Zoopsidella antillana subsp. jamaicensis R.M.Schust., Nova Hedwigia 69 (1/2): 132, 1999 (Schuster 1999b).

*** Zoopsidella caledonica (Steph.) R.M.Schust., Taxon 18 (1): 57, 1969 (Schuster 1969c). Bas.: Zoopsis caledonica Steph., Sp. Hepat. (Stephani) 6: 318, 1922 (Stephani 1922).

** Zoopsidella cynosurandra (Steph.) R.M.Schust., Nova Hedwigia 10 (1/2): 24, 1965 (Schuster 1965b). Bas.: Zoopsis cynosurandra Steph., Bull. Herb. Boissier (sér. 2) 8 (4): 269 (283), 1908 (Stephani 1908j).

*** Zoopsidella integrifolia (Spruce) R.M.Schust., Bull. Natl. Sci. Mus. Tokyo (n.ser.) 12 (3): 666, 1969 (Schuster 1969a). Bas.: Cephalozia integrifolia Spruce, Cephalozia: 29, 1882 (Spruce 1882).

** Zoopsidella macella (Spruce) R.M.Schust., Bull. Natl. Sci. Mus. Tokyo (n.ser.) 12 (3): 666, 1969 (Schuster 1969a). Bas.: Cephalozia macella Spruce, Cephalozia: 29, 1882 (Spruce 1882).

*** Zoopsidella serra (Spruce) R.M.Schust., Bull. Natl. Sci. Mus. Tokyo (n.ser.) 12 (3): 666, 1969 (Schuster 1969a). Bas.: Cephalozia serra Spruce, Cephalozia: 32, 1882 (Spruce 1882).

** **Zoopsis Hook.f. ex Gottsche, Lindenb. et Nees**, Syn. Hepat. 4: 473, 1846 (Gottsche et al. 1846).

** **subg. Eozoopsis R.M.Schust.**, J. Hattori Bot. Lab. 36: 373, 1972 (Schuster 1972).

*** Zoopsis leitgebiana (Carrington et Pearson) Bastow, Pap. & Proc. Roy. Soc. Tasmania 1887: 269, 1888 (Bastow 1888). Bas.: Cephalozia leitgebiana Carrington et Pearson, Pap. & Proc. Roy. Soc. Tasmania 1887: 3, 1888 (Carrington and Pearson 1888b).

*** Zoopsis macrophylla R.M.Schust., Nova Hedwigia 68 (1/2): 14, 1999 (Schuster 1999d).

** **subg. Zoopsis**

*** Zoopsis argentea (Hook.f. et Taylor) Gottsche, Lindenb. et Nees, Syn. Hepat. 4: 473, 1846 (Gottsche et al. 1846). Bas.: Jungermannia argentea Hook.f. et Taylor, London J. Bot. 3: 400, 1844 (Hooker and Taylor 1844a).

** Zoopsis argentea var. flagelliformis (Colenso) R.M.Schust., Nova Hedwigia 68 (1/2): 38, 1999 (Schuster 1999d). Bas.: Zoopsis flagelliformis Colenso, Trans. & Proc. New Zealand Inst. 18: 250, 1886 (Colenso 1886b).

*** Zoopsis bicruris Glenny et E.A.Br., J. Bryol. 28 (4): 332, 2006 (Renner et al. 2006).

** Zoopsis liukiuensis Horik., J. Sci. Hiroshima Univ., Ser. B, Div. 2, Bot. 1: 65, 1931 (Horikawa 1931a).

*** Zoopsis matawaia M.A.M.Renner, J. Bryol. 28 (4): 334, 2006 (Renner et al. 2006).

*** Zoopsis nitida Glenny, Braggins et R.M.Schust., J. Bryol. 19 (4): 776, 1997 (Glenny et al. 1997).

*** Zoopsis setulosa Leitg., Mitt. Naturwiss. Vereins Steiermark 13: 24, 1876 (Leitgeb 1876).

***Incertae sedis***

*** Zoopsis ceratophylla (Spruce) Hamlin, Rec. Domin. Mus. 7: 311, 1972 (Hamlin 1972). Bas.: Cephalozia ceratophylla Spruce, Cephalozia: 32, 1882 (Spruce 1882).

* Zoopsis ciliata Colenso, Trans. & Proc. New Zealand Inst. 20: 253, 1888 (Colenso 1888). ^130^

* Zoopsis martinicensis Steph., Bull. Herb. Boissier (sér. 2) 8 (4): 268 (282), 1908 (Stephani 1908j).

** Zoopsis setigera K.I.Goebel, Flora 77 (2): 93, 1893 (Goebel 1893b).

** Zoopsis uleana Steph., Hedwigia 44 (4): 225, 1905 (Stephani 1905a).

######## *** Lophocoleaceae Vanden Berghen

by B.J. Crandall-Stotler, R. Stotler, J. Váňa, J.J.Engel and L. Söderström

Söderström et al. (2013b) outlined the current status of Lophocoleaceae noting that several taxonomic problems in delimitating genera remain. The placement of several species is also unclear. Nomenclatural and taxonomic notes can be found in Söderström et al. (2013b, 2013f).

** **Bragginsella R.M.Schust.**, Bryologist 100 (3): 363, 1997 (Schuster 1997a).

*** Bragginsella anomala R.M.Schust., Bryologist 100 (3): 363, 1997 (Schuster 1997a).

*** **Chiloscyphus Corda**, Gen. hepat.: 651, 1829 (Corda 1829) nom. conserv.

** Chiloscyphus kashyapii A.Srivast. et S.C.Srivast., Indian Geocalyc.: 34, 2002 (Srivastava and Srivastava 2002).

*** Chiloscyphus pallescens (Ehrh.) Dumort., Syll. Jungerm. Europ.: 67, 1831 (Dumortier 1831). Bas.: Jungermannia pallescens Ehrh., Deutschl. Fl., Theil 2 (Hoffm.): 87, 1795 [1796] (Hoffmann 1795).

* Chiloscyphus pallescens var. fragilis (Roth) Müll.Frib., Ber. Deutsch. Bot. Ges. 59 (10): 429, 1942 (Müller 1942). Bas.: Jungermannia fragilis Roth, Tent. Fl. Germ. 3: 370, 1800 (Roth 1800). ^131^

*** Chiloscyphus polyanthos (L.) Corda, Gen. hepat.: 651, 1829 (Corda 1829). Bas.: Jungermannia polyanthos L., Sp. Pl. 1: 1131, 1753 (Linnaeus 1753).

* Chiloscyphus polyanthos var. rivularis (Schrad.) Lindb. et Arnell, Kongl. Svenska Vetensk.-Akad. Handl. (n.ser.) 23 (5): 24, 1889 (Lindberg and Arnell 1889). Bas.: Jungermannia pallescens var. rivularis Schrad., Syst. Samml. Crypt. Gew. 2: 7, 1797 (Schrader 1797). ^132^

***Incertae sedis*** ^133^

** Chiloscyphus acutus Steph., Sp. Hepat. (Stephani) 6: 302, 1922 (Stephani 1922).

** Chiloscyphus alpicola J.J.Engel, Phytotaxa 207 (2): 181, 2015 (Engel 2015b).

** Chiloscyphus beesleyanus Pearson, J. Linn. Soc., Bot. 46 (305): 22, 1922 (Pearson 1922b).

* Chiloscyphus bifidus Schiffn., Hep. Fl. Buitenzorg: 200, 1900 (Schiffner 1900a). ^134^

** Chiloscyphus breviculus B.Y.Yang et W.C.Lee, Bot. Bull. Acad. Sin. (n.ser.) 5 (2): 190, 1964 (Yang and Lee 1964).

* Chiloscyphus brevistipulus Steph., Sp. Hepat. (Stephani) 6: 303, 1922 (Stephani 1922).

** Chiloscyphus chinnarensis Manju, K.P.Rajesh et Madhus., Acta Bot. Hung. 53 (1/2): 152, 2011 (Manju et al. 2011).

** Chiloscyphus confertifolius Steph., Sp. Hepat. (Stephani) 6: 304, 1922 (Stephani 1922).

** Chiloscyphus confertus Steph., Sp. Hepat. (Stephani) 6: 305, 1922 (Stephani 1922).

** Chiloscyphus cornutistipulus Steph., Sp. Hepat. (Stephani) 6: 303, 1922 (Stephani 1922).

* Chiloscyphus durus (Steph.) Hässel, Revista Mus. Argent. Ci. Nat. (n.ser.) 1 (2): 122, 1999 (Hässel 1999). Bas.: Lophocolea dura Steph., Kungl. Svenska Vetensk.-Akad. Handl. (n.ser.) 46 (9): 43, 1911 (Stephani 1911b). ^135^

* Chiloscyphus ernstianus Steph., Sp. Hepat. (Stephani) 6: 306, 1922 (Stephani 1922).

** Chiloscyphus etesseanus Steph., Bull. Herb. Boissier (sér. 2) 7 (10): 845 (217), 1907 (Stephani 1907b).

** Chiloscyphus francanus Steph., Sp. Hepat. (Stephani) 6: 306, 1922 (Stephani 1922).

** Chiloscyphus graeffeanus Steph., Sp. Hepat. (Stephani) 6: 307, 1922 (Stephani 1922).

** Chiloscyphus greenwelliae (H.A.Mill.) H.A.Mill., J. Hattori Bot. Lab. 30: 275, 1967 (Miller 1967). Bas.: Lophocolea greenwelliae H.A.Mill., Ark. Bot. (n.ser.) 5 (2): 504, 1963 (Miller 1963).

** Chiloscyphus hookeri J.J.Engel, J. Hattori Bot. Lab. 36: 150, 1972 [1973] (Engel 1972).

* Chiloscyphus hookeri var. constantifolius J.J.Engel, J. Hattori Bot. Lab. 36: 155, 1972 [1973] (Engel 1972).

** Chiloscyphus integerrimus Schiffn., Hep. Fl. Buitenzorg: 197, 1900 (Schiffner 1900a).

** Chiloscyphus kehdingianus (Steph.) N.Kitag., Hikobia, Suppl. 1: 68, 1981 (Kitagawa 1981a). Bas.: Lophocolea kehdingiana Steph., Sp. Hepat. (Stephani) 6: 278, 1922 (Stephani 1922).

** Chiloscyphus kilauensis Steph., Sp. Hepat. (Stephani) 6: 309, 1922 (Stephani 1922).

** Chiloscyphus koeppensis (Gottsche) Steph., Bull. Herb. Boissier (sér. 2) 8 (2): 139 (255), 1908 (Stephani 1908i). Bas.: Jungermannia koeppensis Gottsche, Int. Polarforsch., Deutsch. Exped. 2: 452, 1890 (Gottsche 1890).

** Chiloscyphus laceratus Steph., Sp. Hepat. (Stephani) 6: 310, 1922 (Stephani 1922).

** Chiloscyphus lambertonii H.A.Mill., Ark. Bot. (n.ser.) 5 (2): 506, 1963 (Miller 1963).

** Chiloscyphus latistipus Steph., Sp. Hepat. (Stephani) 6: 309, 1922 (Stephani 1922).

** Chiloscyphus lepervanchei (Steph.) J.J.Engel et R.M.Schust., Nova Hedwigia 39: 418, 1984 [1985] (Engel and Schuster 1984). Bas.: Lophocolea lepervanchei Steph., Bull. Herb. Boissier (sér. 2) 7 (4): 310 (174), 1907 (Stephani 1907d).

** Chiloscyphus longifissus Steph., Sp. Hepat. (Stephani) 6: 310, 1922 (Stephani 1922).

** Chiloscyphus propagulifer Schiffn., Hep. Fl. Buitenzorg: 208, 1900 (Schiffner 1900a).

** Chiloscyphus purpureus Steph., Sp. Hepat. (Stephani) 6: 312, 1922 (Stephani 1922).

** Chiloscyphus quadricilius Steph., Sp. Hepat. (Stephani) 6: 312, 1922 (Stephani 1922).

*** Chiloscyphus quadridentatus (Spruce) J.J.Engel et R.M.Schust., Nova Hedwigia 39: 422, 1984 [1985] (Engel and Schuster 1984). Bas.: Lophocolea quadridentata Spruce, Mem. Torrey Bot. Club 1 (3): 137, 1890 (Spruce 1890).

** Chiloscyphus rotundifolius Mitt., Rep. Challenger, Bot. 1 (3, 1): 85, 1884 (Mitten 1884b).

** Chiloscyphus rotundiphyllus H.A.Mill., Phytologia 47 (4): 321, 1981 (Miller 1981). *Nom. nov. pro Chiloscyphus rotundifolius* Steph., Sp. Hepat. (Stephani) 6: 313, 1922 (Stephani 1922), *nom. illeg*.

** Chiloscyphus scaberulus Spruce, Bull. Soc. Bot. France (Congr. Bot.) 36: cc, 1889 [1890] (Spruce 1889).

** Chiloscyphus septatus J.J.Engel, Fieldiana, Bot. (n.ser.) 48: 125, 2010 (Engel 2010).

** Chiloscyphus similis Steph., Rev. Bryol. 35 (2): 28, 1908 (Stephani 1908l).

** Chiloscyphus skottsbergianus H.A.Mill., Ark. Bot. (n.ser.) 5 (2): 508, 1963 (Miller 1963).

** Chiloscyphus subacuminatus Herzog, Ark. Bot. (n.ser.) 3 (3): 49, 1953 (Herzog 1953a).

** Chiloscyphus subsimilis Steph., Sp. Hepat. (Stephani) 6: 314, 1922 (Stephani 1922).

* Chiloscyphus tridens Steph., Sp. Hepat. (Stephani) 6: 315, 1922 (Stephani 1922).

** Chiloscyphus trigonifolius Steph., Sp. Hepat. (Stephani) 6: 316, 1922 (Stephani 1922).

* Chiloscyphus venustulus Colenso, Trans. & Proc. New Zealand Inst. 21: 60, 1889 (Colenso 1889).

** **Clasmatocolea Spruce**, Trans. & Proc. Bot. Soc. Edinburgh 15: 440, 1885 (Spruce 1885). ^136^

*** Clasmatocolea bisexualis Glenny et J.J.Engel, New Zealand J. Bot. 51 (1): 23, 2013 (Glenny and Engel 2013).

*** Clasmatocolea crassiretis (Herzog) Grolle, Nova Acta Leop. (n.ser.) 25 (161): 69, 1962 [1963] (Grolle 1962a). Bas.: Lophocolea crassiretis Herzog, Trans. & Proc. Roy. Soc. New Zealand 65 (3): 354, 1936 (Herzog 1936b).

*** Clasmatocolea ctenophylla (Schiffn.) Grolle, Rev. Bryol. Lichénol. 29 (1/2): 71, 1960 (Grolle 1960c). Bas.: Lophocolea ctenophylla Schiffn., Leberm., Forschungsr. Gazelle 4 (4): 12, 1890 (Schiffner 1890).

*** Clasmatocolea cucullistipula (Steph.) Grolle, Rev. Bryol. Lichénol. 29 (1/2): 71, 1960 (Grolle 1960c). Bas.: Lophocolea cucullistipula Steph., Bih. Kongl. Svenska Vetensk.-Akad. Handl. 26 (III, 6): 37, 1900 (Stephani 1900b).

*** Clasmatocolea fasciculata (Nees) Grolle, Rev. Bryol. Lichénol. 29 (1/2): 72, 1960 (Grolle 1960c). Bas.: Jungermannia fasciculata Nees, Horae Phys. Berol.: 46, 1820 (Nees 1820).

*** Clasmatocolea fulvella (Hook.f. et Taylor) Grolle, Rev. Bryol. Lichénol. 29 (1/2): 72, 1960 (Grolle 1960c). Bas.: Jungermannia fulvella Hook.f. et Taylor, London J. Bot. 3: 464, 1844 (Hooker and Taylor 1844b).

*** Clasmatocolea gayana (Mont.) Grolle, Rev. Bryol. Lichénol. 29 (1/2): 72, 1960 (Grolle 1960c). Bas.: Jungermannia gayana Mont., Ann. Sci. Nat. Bot. (sér. 3) 4: 349, 1845 (Montagne 1845b).

*** Clasmatocolea humilis (Hook.f. et Taylor) Grolle, Rev. Bryol. Lichénol. 29 (1/2): 72, 1960 (Grolle 1960c). Bas.: Jungermannia humilis Hook.f. et Taylor, London J. Bot. 3: 468, 1844 (Hooker and Taylor 1844b).

*** Clasmatocolea humilis var. polymorpha J.J.Engel, Phytologia 41 (5): 309, 1979 (Engel 1979a).

*** Clasmatocolea humilis var. suspecta (C.Massal.) J.J.Engel, Phytologia 41 (5): 309, 1979 (Engel 1979a). Bas.: Lophocolea puccioana β suspecta C.Massal., Nuovo Giorn. Bot. Ital. 17 (3): 228, 1885 (Massalongo 1885).

*** Clasmatocolea inflexispina (Hook.f. et Taylor) J.J.Engel, Bryologist 94 (4): 436, 1991 (Engel 1991b). Bas.: Jungermannia inflexispina Hook.f. et Taylor, London J. Bot. 4: 82, 1845 (Hooker and Taylor 1845).

*** Clasmatocolea marginata (Steph.) Grolle, Nova Acta Leop. (n.ser.) 25 (161): 73, 1962 [1963] (Grolle 1962a). Bas.: Leioscyphus marginatus Steph., Bull. Herb. Boissier (sér. 2) 6 (3): 223 (23), 1906 (Stephani 1906g).

*** Clasmatocolea minutiretis J.J.Engel et Grolle, Phytologia 41 (5): 309, 1979 (Engel 1979a).

*** Clasmatocolea moniliformis J.J.Engel, Phytologia 41 (5): 310, 1979 (Engel 1979a).

*** Clasmatocolea navistipula (Steph.) Grolle, Feddes Repert. 82 (1): 88, 1971 (Grolle 1971b). Bas.: Lophocolea navistipula Steph., Bull. Herb. Boissier (sér. 2) 6 (7): 543 (57), 1906 (Stephani 1906f).

*** Clasmatocolea navistipula var. parceramosa J.J.Engel, Phytologia 41 (5): 311, 1979 (Engel 1979a).

*** Clasmatocolea notophylla (Hook.f. et Taylor) Grolle, J. Jap. Bot. 41 (8): 228, 1966 (Grolle 1966d). Bas.: Jungermannia notophylla Hook.f. et Taylor, London J. Bot. 3: 376, 1844 (Hooker and Taylor 1844a).

*** Clasmatocolea obvoluta (Hook.f. et Taylor) Grolle, Rev. Bryol. Lichénol. 29 (1/2): 72, 1960 (Grolle 1960c). Bas.: Jungermannia obvoluta Hook.f. et Taylor, London J. Bot. 4: 80, 1845 (Hooker and Taylor 1845).

*** Clasmatocolea obvoluta var. cookiana (C.Massal.) J.J.Engel, Phytologia 41 (5): 311, 1979 (Engel 1979a). Bas.: Lophocolea cookiana C.Massal., Nuovo Giorn. Bot. Ital. 17 (3): 224, 1885 (Massalongo 1885).

*** Clasmatocolea puccioana (De Not.) Grolle, Rev. Bryol. Lichénol. 29 (1/2): 72, 1960 (Grolle 1960c). Bas.: Jungermannia puccioana De Not., Mem. Reale Accad. Sci. Torino (ser. 2) 16: 221, 1857 (De Notaris 1857).

*** Clasmatocolea rigens (Hook.f. et Taylor) J.J.Engel, J. Hattori Bot. Lab. 36: 156, 1972 [1973] (Engel 1972). Bas.: Jungermannia rigens Hook.f. et Taylor, London J. Bot. 3: 461, 1844 (Hooker and Taylor 1844b).

*** Clasmatocolea strongylophylla (Hook.f. et Taylor) Grolle, Rev. Bryol. Lichénol. 29 (1/2): 73, 1960 (Grolle 1960c). Bas.: Jungermannia strongylophylla Hook.f. et Taylor, London J. Bot. 3: 370, 1844 (Hooker and Taylor 1844a).

*** Clasmatocolea trachyopa (Hook.f. et Taylor) Grolle, Rev. Bryol. Lichénol. 29 (1/2): 73, 1960 (Grolle 1960c). Bas.: Jungermannia trachyopa Hook.f. et Taylor, London J. Bot. 3: 471, 1844 (Hooker and Taylor 1844b).

*** Clasmatocolea vermicularis (Lehm.) Grolle, Rev. Bryol. Lichénol. 29 (1/2): 78, 1960 (Grolle 1960c). Bas.: Jungermannia vermicularis Lehm., Linnaea 4: 361, 1829 (Lehmann 1829).

*** Clasmatocolea verrucosa J.J.Engel, Bryologist 83 (2): 220, 1980 (Engel 1980b).

*** **Conoscyphus Mitt.**, Fl. vit.: 404, 1871 [1873] (Mitten 1871).

** Conoscyphus koponenii Piippo, Mamontov et Potemkin, Acta Bryolichenol. Asiat. 5: 20, 2014 (Piippo et al. 2014).

*** Conoscyphus trapezioides (Sande Lac.) Schiffn., Consp. Hepat. Arch. Ind.: 125, 1898 (Schiffner 1898b). Bas.: Chiloscyphus trapezioides Sande Lac., Ned. Kruidk. Arch. 3: 417, 1854 [1855] (Sande Lacoste 1854).

*** **Cryptolophocolea L.Söderstr., Crand.-Stotl., Stotler et Váňa**, Phytotaxa 97 (2): 39, 2013 (Söderström et al. 2013b). Based on: Plagiochila sect. Connatae Lindenb., Monogr. hep. gen. Plagiochilae: xxix, 1844 [1843] (Lindenberg 1844).

*** Cryptolophocolea aculeata (Mitt.) L.Söderstr., Phytotaxa 112 (1): 21, 2013 (Söderström et al. 2013f). Bas.: Chiloscyphus aculeatus Mitt., Bot. antarct. voy. II (Fl. Nov.-Zel. 2): 140, 1854 (Mitten 1854).

** Cryptolophocolea chiloscyphoidea (Lindenb.) L.Söderstr. et Crand.-Stotl., Phytotaxa 112 (1): 18, 2013 (Söderström et al. 2013f). Bas.: Plagiochila chiloscyphoidea Lindenb., Nov. Stirp. Pug. 8: 4, 1844 (Lehmann 1844).

*** Cryptolophocolea ciliolata (Nees) L.Söderstr., Crand.-Stotl., Stotler et Váňa, Phytotaxa 97 (2): 39, 2013 (Söderström et al. 2013b). Bas.: Jungermannia ciliolata Nees, Enum. Pl. Crypt. Javae: 68, 1830 (Nees 1830).

** Cryptolophocolea compacta (Mitt.) L.Söderstr., Phytotaxa 112 (1): 19, 2013 (Söderström et al. 2013f). Bas.: Lophocolea compacta Mitt., Trans. Linn. Soc. London, Bot. 3 (3): 198, 1891 (Mitten 1891).

*** Cryptolophocolea connata (Sw.) L.Söderstr. et Váňa, Phytotaxa 112 (1): 19, 2013 (Söderström et al. 2013f). Bas.: Jungermannia connata Sw., Prodr. (Swartz): 143, 1788 (Swartz 1788).

*** Cryptolophocolea connatifolia (J.J.Engel) L.Söderstr., Phytotaxa 112 (1): 21, 2013 (Söderström et al. 2013f). Bas.: Chiloscyphus connatifolius J.J.Engel, Phytologia 83 (1): 42, 1997 [1998] (Engel 1997).

*** Cryptolophocolea costata (Nees) L.Söderstr., Phytotaxa 112 (1): 19, 2013 (Söderström et al. 2013f). Bas.: Jungermannia costata Nees, Enum. Pl. Crypt. Javae: 69, 1830 (Nees 1830).

*** Cryptolophocolea edentata (J.J.Engel) L.Söderstr., Phytotaxa 112 (1): 21, 2013 (Söderström et al. 2013f). Bas.: Chiloscyphus edentatus J.J.Engel, Phytologia 83 (1): 43, 1997 [1998] (Engel 1997).

** Cryptolophocolea explanata (Mitt.) Váňa et Crand.-Stotl., Phytotaxa 202 (1): 69, 2015 (Söderström et al. 2015c). Bas.: Lophocolea explanata Mitt., Fl. vit.: 404, 1871 [1873] (Mitten 1871).

** Cryptolophocolea fleischeri (Steph.) L.Söderstr., Phytotaxa 112 (1): 21, 2013 (Söderström et al. 2013f). Bas.: Lophocolea fleischeri Steph., Bull. Herb. Boissier (sér. 2) 6 (11): 952 (132), 1906 (Stephani 1906c).

*** Cryptolophocolea guadalupensis (Steph.) L.Söderstr. et Váňa, Phytotaxa 112 (1): 19, 2013 (Söderström et al. 2013f). Bas.: Lophocolea guadalupensis Steph., Bull. Herb. Boissier (sér. 2) 7 (1): 65 (153), 1907 (Stephani 1907c).

*** Cryptolophocolea helmsiana (Steph.) L.Söderstr., Phytotaxa 112 (1): 19, 2013 (Söderström et al. 2013f). Bas.: Lophocolea helmsiana Steph., Bull. Herb. Boissier (sér. 2) 6 (9): 794 (94), 1906 (Stephani 1906e).

*** Cryptolophocolea leucophylla (Hook.f. et Taylor) L.Söderstr., Phytotaxa 112 (1): 19, 2013 (Söderström et al. 2013f). Bas.: Jungermannia leucophylla Hook.f. et Taylor, London J. Bot. 3: 384, 1844 (Hooker and Taylor 1844a).

* Cryptolophocolea levieri (Schiffn.) L.Söderstr., Phytotaxa 112 (1): 21, 2013 (Söderström et al. 2013f). Bas.: Lophocolea levieri Schiffn., Hep. Fl. Buitenzorg: 182, 1900 (Schiffner 1900a). ^137^

* Cryptolophocolea lilliena (Steph.) L.Söderstr., Phytotaxa 112 (1): 21, 2013 (Söderström et al. 2013f). Bas.: Lophocolea lilliena Steph., Sp. Hepat. (Stephani) 6: 282, 1922 (Stephani 1922). ^138^

*** Cryptolophocolea martiana (Nees) L.Söderstr., Crand.-Stotl. et Stotler, Phytotaxa 112 (1): 20, 2013 (Söderström et al. 2013f). Bas.: Lophocolea martiana Nees, Syn. Hepat. 2: 152, 1845 (Gottsche et al. 1845a).

** Cryptolophocolea martiana subsp. bidentula (Nees) L.Söderstr., Crand.-Stotl. et Stotler, Phytotaxa 112 (1): 20, 2013 (Söderström et al. 2013f). Bas.: Chiloscyphus bidentulus Nees, Syn. Hepat. 2: 181, 1845 (Gottsche et al. 1845a).

** Cryptolophocolea martiana var. perissodonta (Spruce) Gradst., Phytoneuron 2015 (22): 1, 2015 (Bernal et al. 2015). Bas.: Lophocolea martiana var. perissodonta Spruce, Trans. & Proc. Bot. Soc. Edinburgh 15: 432, 1885 (Spruce 1885).

* Cryptolophocolea massalongoana (Schiffn.) L.Söderstr., Phytotaxa 112 (1): 20, 2013 (Söderström et al. 2013f). Bas.: Lophocolea massalongoana Schiffn., Hep. Fl. Buitenzorg: 183, 1900 (Schiffner 1900a). ^139^

*** Cryptolophocolea mitteniana (Colenso) L.Söderstr., Phytotaxa 112 (1): 21, 2013 (Söderström et al. 2013f). Bas.: Isotachis mitteniana Colenso, Trans. & Proc. New Zealand Inst. 21: 69, 1889 (Colenso 1889).

*** Cryptolophocolea mitteniana var. obtusa (J.J.Engel) L.Söderstr., Phytotaxa 112 (1): 21, 2013 (Söderström et al. 2013f). Bas.: Chiloscyphus mittenianus var. obtusus J.J.Engel, Phytologia 83 (1): 44, 1997 [1998] (Engel 1997).

*** Cryptolophocolea mitteniana var. symmetrica (J.J.Engel) L.Söderstr., Phytotaxa 112 (1): 22, 2013 (Söderström et al. 2013f). Bas.: Chiloscyphus mittenianus var. symmetricus J.J.Engel, Phytologia 83 (1): 44, 1997 [1998] (Engel 1997).

*** Cryptolophocolea pallida (Mitt.) L.Söderstr., Phytotaxa 112 (1): 22, 2013 (Söderström et al. 2013f). Bas.: Lophocolea pallida Mitt., Bot. antarct. voy. II (Fl. Nov.-Zel. 2): 135, 1854 (Mitten 1854).

** Cryptolophocolea pallidovirens (Hook.f. et Taylor) L.Söderstr., Phytotaxa 112 (1): 20, 2013 (Söderström et al. 2013f). Bas.: Jungermannia pallidovirens Hook.f. et Taylor, London J. Bot. 3: 473, 1844 (Hooker and Taylor 1844b).

* Cryptolophocolea proteus (Herzog) L.Söderstr., Phytotaxa 112 (1): 22, 2013 (Söderström et al. 2013f). Bas.: Lophocolea proteus Herzog, Feddes Repert. Spec. Nov. Regni Veg. 57 (1/2): 164, 1955 (Herzog 1955).

* Cryptolophocolea pycnophylla (Spruce) L.Söderstr., Phytotaxa 112 (1): 22, 2013 (Söderström et al. 2013f). Bas.: Lophocolea pycnophylla Spruce, Trans. & Proc. Bot. Soc. Edinburgh 15: 434, 1885 (Spruce 1885).

*** Cryptolophocolea regularis (Steph.) L.Söderstr., Phytotaxa 112 (1): 23, 2013 (Söderström et al. 2013f). Bas.: Chiloscyphus regularis Steph., Hedwigia 32 (5): 325, 1893 (Stephani 1893d).

*** Cryptolophocolea spinifera (Hook.f. et Taylor) L.Söderstr., Phytotaxa 112 (1): 20, 2013 (Söderström et al. 2013f). Bas.: Jungermannia spinifera Hook.f. et Taylor, London J. Bot. 3: 381, 1844 (Hooker and Taylor 1844a).

* Cryptolophocolea stephanii (Schiffn.) L.Söderstr., Phytotaxa 112 (1): 23, 2013 (Söderström et al. 2013f). Bas.: Lophocolea stephanii Schiffn., Hep. Fl. Buitenzorg: 181, 1900 (Schiffner 1900a). ^140^

*** Cryptolophocolea subopposita (J.J.Engel) L.Söderstr., Phytotaxa 112 (1): 23, 2013 (Söderström et al. 2013f). Bas.: Chiloscyphus suboppositus J.J.Engel, Phytologia 83 (1): 45, 1997 [1998] (Engel 1997).

* Cryptolophocolea thermarum (Schiffn.) L.Söderstr., Phytotaxa 112 (1): 23, 2013 (Söderström et al. 2013f). Bas.: Lophocolea thermarum Schiffn., Hep. Fl. Buitenzorg: 180, 1900 (Schiffner 1900a). ^141^

*** Cryptolophocolea trialata (Gottsche) L.Söderstr., Phytotaxa 112 (1): 23, 2013 (Söderström et al. 2013f). Bas.: Lophocolea trialata Gottsche, Linnaea 28 (5): 552, 1856 [1857] (Gottsche 1856).

** Cryptolophocolea tricorata (Hässel) Crand.-Stotl. et Stotler, Phytotaxa 112 (1): 23, 2013 (Söderström et al. 2013f). Bas.: Chiloscyphus tricoratus Hässel, Nova Hedwigia 70 (3/4): 456, 2000 (Hässel 2000).

*** Cryptolophocolea tuberculata (J.J.Engel) L.Söderstr., Phytotaxa 112 (1): 23, 2013 (Söderström et al. 2013f). Bas.: Chiloscyphus tuberculatus J.J.Engel, Phytologia 83 (1): 45, 1997 [1998] (Engel 1997).

* Cryptolophocolea whittieriana (Inoue et H.A.Mill.) L.Söderstr., Phytotaxa 112 (1): 24, 2013 (Söderström et al. 2013f). Bas.: Lophocolea whittieriana Inoue et H.A.Mill., Bull. Natl. Sci. Mus. Tokyo (n.ser.) 8 (2): 143, 1965 (Inoue and Miller 1965). ^142^

*** **Deceptifrons J.J.Engel et Váňa**, Mem. New York Bot. Gard. 105: 54, 2013 (Váňa and Engel 2013).

*** Deceptifrons plagiochiloides J.J.Engel et Váňa, Mem. New York Bot. Gard. 105: 54, 2013 (Váňa and Engel 2013).

*** **Evansianthus R.M.Schust. et J.J.Engel**, Bryologist 76 (4): 516, 1973 (Schuster and Engel 1973).

*** Evansianthus georgiensis (Gottsche) R.M.Schust. et J.J.Engel, Bryologist 76 (4): 518, 1973 (Schuster and Engel 1973). Bas.: Lophocolea georgiensis Gottsche, Int. Polarforsch., Deutsch. Exped. 2: 453, 1890 (Gottsche 1890).

*** **Hepatostolonophora J.J.Engel et R.M.Schust.**, J. Hattori Bot. Lab. 46: 91, 1979 (Engel 1979b). *Nom. nov. pro Stolonophora* J.J.Engel et R.M.Schust., Fieldiana, Bot. 36 (11): 111, 1975 (Engel and Schuster 1975).

*** Hepatostolonophora abnormis (Besch. et C.Massal.) J.J.Engel et R.M.Schust., J. Hattori Bot. Lab. 46: 94, 1979 (Engel 1979b). Bas.: Leioscyphus abnormis Besch. et C.Massal., Bull. Mens. Soc. Linn. Paris 1 (79): 629, 1886 (Bescherelle and Massalongo 1886).

** Hepatostolonophora conica (Steph.) Hässel, Bol. Soc. Argent. Bot. 22 (1/4): 123, 1983 (Hässel 1983). Bas.: Plagiochila conica Steph., Kungl. Svenska Vetensk.-Akad. Handl. (n.ser.) 46 (9): 28, 1911 (Stephani 1911b).

*** Hepatostolonophora paucistipula (Rodway) J.J.Engel, J. Hattori Bot. Lab. 46: 103, 1979 (Engel 1979b). Bas.: Lophocolea paucistipula Rodway, Pap. & Proc. Roy. Soc. Tasmania 1916: 46, 1917 (Rodway 1917a).

*** Hepatostolonophora rotata (Hook.f. et Taylor) J.J.Engel, J. Hattori Bot. Lab. 46: 98, 1979 (Engel 1979b). Bas.: Jungermannia rotata Hook.f. et Taylor, London J. Bot. 3: 560, 1844 (Hooker and Taylor 1844d).

*** Hepatostolonophora rotata var. perssonii (R.M.Schust.) J.J.Engel, J. Hattori Bot. Lab. 46: 103, 1979 (Engel 1979b). Bas.: Calyptrocolea perssonii R.M.Schust., Rev. Bryol. Lichénol. 34 (3/4): 699, 1966 [1967] (Schuster 1966a).

*** **Heteroscyphus Schiffn.**, Österr. Bot. Z. 60 (5): 171, 1910 (Schiffner 1910a) nom. conserv.

** Heteroscyphus acutangulus (Schiffn.) Schiffn., Österr. Bot. Z. 60 (5): 172, 1910 (Schiffner 1910a). Bas.: Chiloscyphus acutangulus Schiffn., Hep. Fl. Buitenzorg: 200, 1900 (Schiffner 1900a).

** Heteroscyphus allodontus (Hook.f. et Taylor) J.J.Engel et R.M.Schust., Nova Hedwigia 39: 399, 1984 [1985] (Engel and Schuster 1984). Bas.: Jungermannia allodonta Hook.f. et Taylor, London J. Bot. 3: 382, 1844 (Hooker and Taylor 1844a).

** Heteroscyphus amboinensis (Schiffn.) Schiffn., Österr. Bot. Z. 60 (5): 172, 1910 (Schiffner 1910a). Bas.: Chiloscyphus endlicherianus var. amboinensis Schiffn., Leberm., Forschungsr. Gazelle 4 (4): 15, 1890 (Schiffner 1890).

** Heteroscyphus ammophilus (Colenso) R.M.Schust., Hepat. Anthocerotae N. Amer. 4: 248, 1980 (Schuster 1980c). Bas.: Chiloscyphus ammophilus Colenso, Trans. & Proc. New Zealand Inst. 21: 59, 1889 (Colenso 1889).

** Heteroscyphus ammophilus var. obtusifolius J.J.Engel et G.L.Merr., Nova Hedwigia 99 (1/2): 158, 2014 (Engel 2014).

*** Heteroscyphus argutus (Reinw., Blume et Nees) Schiffn., Österr. Bot. Z. 60 (5): 172, 1910 (Schiffner 1910a). Bas.: Jungermannia arguta Reinw., Blume et Nees, Nova Acta Phys.-Med. Acad. Caes. Leop.-Carol. Nat. Cur. 12 (1): 206, 1824 [1825] (Reinwardt et al. 1824a).

** Heteroscyphus argutus var. brevidens (Schiffn.) Herzog et Nog., J. Hattori Bot. Lab. 14: 40, 1955 (Herzog and Noguchi 1955). Bas.: Chiloscyphus argutus var. brevidens Schiffn., Hep. Fl. Buitenzorg: 195, 1900 (Schiffner 1900a).

*** Heteroscyphus aselliformis (Reinw., Blume et Nees) Schiffn., Österr. Bot. Z. 60 (5): 172, 1910 (Schiffner 1910a). Bas.: Jungermannia aselliformis Reinw., Blume et Nees, Nova Acta Phys.-Med. Acad. Caes. Leop.-Carol. Nat. Cur. 12 (1): 412, 1824 [1825] (Reinwardt et al. 1824b).

** Heteroscyphus assurgentifolius J.J.Engel, Nova Hedwigia 99 (1/2): 158, 2014 (Engel 2014).

** Heteroscyphus baduinus (Nees) Schiffn., Österr. Bot. Z. 60 (5): 171, 1910 (Schiffner 1910a). Bas.: Jungermannia baduina Nees, Enum. Pl. Crypt. Javae: 26, 1830 (Nees 1830).

** Heteroscyphus balnetii (Herzog) Grolle, Acta Bot. Fenn. 125: 68, 1984 (Grolle and Piippo 1984). Bas.: Chiloscyphus balnetii Herzog, Ann. Bryol. 5: 89, 1932 (Herzog 1932a).

*** Heteroscyphus billardierei (Schwägr.) Schiffn., Österr. Bot. Z. 60 (5): 172, 1910 (Schiffner 1910a). Bas.: Jungermannia billardierei Schwägr., Hist. Musc. Hepat. Prodr.: 19, 1814 (Schwägrichen 1814).

** Heteroscyphus billardierei var. clasmatocoleoides (J.J.Engel et G.L.Merr.) J.J.Engel, Nova Hedwigia 100 (3/4): 565, 2015 (Engel 2015a). Bas.: Heteroscyphus circumdentatus var. clasmatocoleoides J.J.Engel et G.L.Merr., Polish Bot. J. 58 (1): 95, 2013 (Engel 2013b).

** Heteroscyphus brassii (Grolle) Grolle, Acta Bot. Fenn. 125: 68, 1984 (Grolle and Piippo 1984). Bas.: Chiloscyphus brassii Grolle, J. Hattori Bot. Lab. 31: 2, 1968 (Grolle 1968a).

* Heteroscyphus caesius (Schiffn.) Schiffn., Österr. Bot. Z. 60 (5): 172, 1910 (Schiffner 1910a). Bas.: Chiloscyphus caesius Schiffn., Hep. Fl. Buitenzorg: 207, 1900 (Schiffner 1900a). ^143^

* Heteroscyphus caledonicus (Steph.) Schiffn., Österr. Bot. Z. 60 (5): 172, 1910 (Schiffner 1910a). Bas.: Chiloscyphus caledonicus Steph., Bull. Herb. Boissier (sér. 2) 7 (10): 844 (216), 1907 (Stephani 1907b). ^144^

** Heteroscyphus chlorophyllus (Hook.f. et Taylor) Schiffn., Österr. Bot. Z. 60 (5): 172, 1910 (Schiffner 1910a). Bas.: Jungermannia chlorophylla Hook.f. et Taylor, London J. Bot. 3: 562, 1844 (Hooker and Taylor 1844d).

** Heteroscyphus ciliatus (Steph.) Schiffn., Österr. Bot. Z. 60 (5): 172, 1910 (Schiffner 1910a). Bas.: Chiloscyphus ciliatus Steph., Hedwigia 32 (5): 320, 1893 (Stephani 1893d).

*** Heteroscyphus coalitus (Hook.) Schiffn., Österr. Bot. Z. 60 (5): 172, 1910 (Schiffner 1910a). Bas.: Jungermannia coalita Hook., Musci Exot. 2: tab. 123, 1820 (Hooker 1820).

** Heteroscyphus coalitus var. simplicifolius J.J.Engel, Nova Hedwigia 100 (3/4): 572, 2015 (Engel 2015a).

** Heteroscyphus combinatus (Nees) Schiffn., Österr. Bot. Z. 60 (5): 172, 1910 (Schiffner 1910a). Bas.: Jungermannia combinata Nees, Enum. Pl. Crypt. Javae: 22, 1830 (Nees 1830).

*** Heteroscyphus conjugatus (Mitt.) J.J.Engel et R.M.Schust., Nova Hedwigia 39: 399, 1984 [1985] (Engel and Schuster 1984). Bas.: Chiloscyphus conjugatus Mitt., Bot. antarct. voy. III (Fl. Tasman. 2): 227, 1860 (Mitten 1860b).

*** Heteroscyphus contortuplicatus (Nees et Mont.) Grolle, J. Hattori Bot. Lab. 55: 504, 1984 (Grolle 1984a). Bas.: Jungermannia contortuplicata Nees et Mont., Ann. Sci. Nat. Bot. (sér. 2) 5: 54, 1836 (Nees and Montagne 1836).

*** Heteroscyphus cuneistipulus (Steph.) Schiffn., Österr. Bot. Z. 60 (5): 172, 1910 (Schiffner 1910a). Bas.: Chiloscyphus cuneistipulus Steph., Hedwigia 32 (5): 322, 1893 (Stephani 1893d).

** Heteroscyphus darjeelingensis A.Srivast. et S.C.Srivast., Indian Geocalyc.: 115, 2002 (Srivastava and Srivastava 2002).

** Heteroscyphus deceptifrons J.J.Engel, Nova Hedwigia 99 (1/2): 162, 2014 (Engel 2014).

*** Heteroscyphus dentammophilus J.J.Engel et G.L.Merr., Polish Bot. J. 58 (1): 96, 2013 (Engel 2013b).

** Heteroscyphus denticulatus (Mitt.) Schiffn., Beih. Bot. Centralbl. 29: 106, 1912 (Schiffner 1912c). Bas.: Chiloscyphus denticulatus Mitt., Nat. hist. Azores: 320, 1870 (Mitten 1870).

** Heteroscyphus deplanchei (Steph.) Schiffn., Österr. Bot. Z. 60 (5): 172, 1910 (Schiffner 1910a). Bas.: Chiloscyphus deplanchei Steph., Bull. Herb. Boissier (sér. 2) 7 (8): 693 (203), 1907 (Stephani 1907e).

** Heteroscyphus diestianus (Sande Lac.) Piippo, Acta Bot. Fenn. 131: 165, 1985 (Piippo 1985a). Bas.: Chiloscyphus diestianus Sande Lac., Ann. Mus. Bot. Lugduno-Batavi 1: 296, 1864 (Sande Lacoste 1864).

** Heteroscyphus divergenticiliatus (Steph.) Fulford, Mem. New York Bot. Gard. 11 (4): 501, 1976 (Fulford 1976). Bas.: Lophocolea divergenticiliata Steph., Bih. Kongl. Svenska Vetensk.-Akad. Handl. 26 (III, 6): 38, 1900 (Stephani 1900b).

** Heteroscyphus dubius (Gottsche) Schiffn., Österr. Bot. Z. 60 (5): 172, 1910 (Schiffner 1910a). Bas.: Chiloscyphus dubius Gottsche, Abh. Naturwiss. Vereins Bremen 7: 346, 1882 (Gottsche 1882).

*** Heteroscyphus echinellus (Lindenb. et Gottsche) J.J.Engel et Xiao L.He, Bryologist 113 (1): 155, 2010 (Engel and He 2010). Bas.: Lophocolea echinella Lindenb. et Gottsche, Syn. Hepat. 5: 703, 1847 (Gottsche et al. 1847).

** Heteroscyphus echinellus var. hyalinus J.J.Engel, Bryologist 113 (1): 161, 2010 (Engel and He 2010).

** Heteroscyphus elliottii (Steph.) Pagán, Bryologist 42 (1): 7, 1939 (Pagán 1939a). Bas.: Chiloscyphus elliottii Steph., Bull. Herb. Boissier (sér. 2) 8 (1): 55 (231), 1908 (Stephani 1908k).

* Heteroscyphus elliottii var. portoricensis Fulford, Mem. New York Bot. Gard. 11 (4): 489, 1976 (Fulford 1976).

* Heteroscyphus falcifolius (Steph.) Schiffn., Österr. Bot. Z. 60 (5): 172, 1910 (Schiffner 1910a). Bas.: Chiloscyphus falcifolius Steph., Bull. Herb. Boissier (sér. 2) 7 (8): 698 (208), 1907 (Stephani 1907e).

*** Heteroscyphus fissistipus (Hook.f. et Taylor) Schiffn., Österr. Bot. Z. 60 (5): 172, 1910 (Schiffner 1910a). Bas.: Jungermannia fissistipa Hook.f. et Taylor, London J. Bot. 3: 384, 1844 (Hooker and Taylor 1844a).

** Heteroscyphus fissistipus var. multispinus (E.A.Hodgs. et Allison) J.J.Engel, Nova Hedwigia 99 (1/2): 165, 2014 (Engel 2014). Bas.: Chiloscyphus multispinus E.A.Hodgs. et Allison, Trans. & Proc. Roy. Soc. New Zealand 73 (1): 37, 1943 (Hodgson 1943).

** Heteroscyphus fissistipus var. repandus J.J.Engel, Nova Hedwigia 99 (1/2): 165, 2014 (Engel 2014).

** Heteroscyphus flaccidus (Mitt.) A.Srivast. et S.C.Srivast., Indian Geocalyc.: 85, 2002 (Srivastava and Srivastava 2002). Bas.: Lophocolea flaccida Mitt., J. Proc. Linn. Soc., Bot. 5 (18): 99, 1860 [1861] (Mitten 1860c).

** Heteroscyphus fleischeri (Steph.) D.G.Long et Rubas., Ceylon J. Sci., Biol. Sci. 43 (1): 26, 2014 (Long and Rubasinghe 2014). Bas.: Chiloscyphus fleischeri Steph., Sp. Hepat. (Stephani) 6: 306, 1922 (Stephani 1922).

* Heteroscyphus fragilicilius (Schiffn.) Schiffn., Österr. Bot. Z. 60 (5): 172, 1910 (Schiffner 1910a). Bas.: Chiloscyphus fragilicilius Schiffn., Denkschr. Kaiserl. Akad. Wiss., Math.-Naturwiss. Kl. 70: 210, 1900 [1901] (Schiffner 1900c).

** Heteroscyphus furcistipulus (E.A.Hodgs.) J.J.Engel et R.M.Schust., Nova Hedwigia 39: 400, 1984 [1985] (Engel and Schuster 1984). Bas.: Chiloscyphus furcistipulus E.A.Hodgs., Trans. Roy. Soc. New Zealand, Bot. 3 (4): 79, 1965 (Hodgson 1965).

** Heteroscyphus giganteus (Steph.) Hürl., Bauhinia 12 (1/2): 114, 1998 (Hürlimann 1998). Bas.: Chiloscyphus giganteus Steph., Sp. Hepat. (Stephani) 6: 307, 1922 (Stephani 1922).

** Heteroscyphus graeffei (J.B.Jack et Steph.) Grolle, Wiss. Z. Friedrich-Schiller-Univ. Jena, Math.-Naturwiss. Reihe 29 (4): 645, 1980 (Grolle 1980d). Bas.: Lophocolea graeffei J.B.Jack et Steph., Bot. Centralbl. 60 (4): 101, 1894 (Jack and Stephani 1894).

** Heteroscyphus grandiflorus (Steph.) Hürl., Bauhinia 12 (1/2): 114, 1998 (Hürlimann 1998). Bas.: Chiloscyphus grandiflorus Steph., Sp. Hepat. (Stephani) 6: 307, 1922 (Stephani 1922).

** Heteroscyphus grandistipus (Steph.) Schiffn., Österr. Bot. Z. 60 (5): 172, 1910 (Schiffner 1910a). Bas.: Chiloscyphus grandistipus Steph., Bot. Gaz. 15 (11): 283, 1890 (Stephani 1890c).

** Heteroscyphus gunnianus (Mitt.) J.J.Engel et R.M.Schust., Nova Hedwigia 39: 400, 1984 [1985] (Engel and Schuster 1984). Bas.: Chiloscyphus gunnianus Mitt., Bot. antarct. voy. III (Fl. Tasman. 2): 228, 1860 (Mitten 1860b).

** Heteroscyphus hastatus (E.A.Hodgs.) J.J.Engel et R.M.Schust., Nova Hedwigia 39: 400, 1984 [1985] (Engel and Schuster 1984). Bas.: Chiloscyphus hastatus E.A.Hodgs., Trans. Roy. Soc. New Zealand, Bot. 3 (11): 181, 1967 (Hodgson 1967).

** Heteroscyphus hebridensis (Steph.) Schiffn., Österr. Bot. Z. 60 (5): 172, 1910 (Schiffner 1910a). Bas.: Chiloscyphus hebridensis Steph., Hedwigia 32 (5): 323, 1893 (Stephani 1893d).

** Heteroscyphus heterophyllus (Steph.) J.J.Engel et R.M.Schust., Nova Hedwigia 39: 400, 1984 [1985] (Engel and Schuster 1984). Bas.: Chiloscyphus heterophyllus Steph., Sp. Hepat. (Stephani) 6: 308, 1922 (Stephani 1922).

** Heteroscyphus hyalinus (Steph.) A.Srivast. et S.C.Srivast., Indian Geocalyc.: 118, 2002 (Srivastava and Srivastava 2002). Bas.: Lophocolea hyalina Steph., Bull. Soc. Roy. Bot. Belgique 38 (1): 46, 1899 (Stephani 1899h).

*** Heteroscyphus integrifolius (Lehm. et Lindenb.) Fulford, Mem. New York Bot. Gard. 11 (4): 495, 1976 (Fulford 1976). Bas.: Jungermannia integrifolia Lehm. et Lindenb., Nov. Stirp. Pug. 6: 32, 1834 (Lehmann 1834).

** Heteroscyphus iwatsukii (S.Hatt.) Piippo, Willdenowia 18 (2): 522, 1989 (Piippo 1989a). Bas.: Saccogynidium iwatsukii S.Hatt., J. Jap. Bot. 39 (7): 206, 1964 (Hattori 1964).

** Heteroscyphus jackii (Steph.) Schiffn., Österr. Bot. Z. 60 (5): 172, 1910 (Schiffner 1910a). Bas.: Chiloscyphus jackii Steph., Bot. Centralbl. 60 (4): 102, 1894 (Jack and Stephani 1894).

*** Heteroscyphus knightii (Steph.) Grolle, J. Hattori Bot. Lab. 61: 251, 1986 [1987] (Grolle 1986a). Bas.: Chiloscyphus knightii Steph., Bull. Herb. Boissier (sér. 2) 8 (2): 129 (245), 1908 (Stephani 1908i).

** Heteroscyphus levieri (Steph.) Schiffn., Österr. Bot. Z. 60 (5): 172, 1910 (Schiffner 1910a). Bas.: Chiloscyphus levieri Steph., Bull. Herb. Boissier (sér. 2) 8 (2): 132 (248), 1908 (Stephani 1908i).

** Heteroscyphus limosus (Carrington et Pearson) Schiffn., Österr. Bot. Z. 60 (5): 172, 1910 (Schiffner 1910a). Bas.: Chiloscyphus limosus Carrington et Pearson, Pap. & Proc. Roy. Soc. Tasmania 1887: 6, 1888 (Carrington and Pearson 1888b).

** Heteroscyphus lingulatus (Colenso) J.J.Engel et R.M.Schust., Nova Hedwigia 39: 400, 1984 [1985] (Engel and Schuster 1984). Bas.: Chiloscyphus lingulatus Colenso, Trans. & Proc. New Zealand Inst. 21: 61, 1889 (Colenso 1889).

** Heteroscyphus lophocoleoides S.Hatt., Bull. Tokyo Sci. Mus. 11: 45, 1944 (Hattori 1944d).

*** Heteroscyphus lyallii (Mitt.) R.M.Schust., Hepat. Anthocerotae N. Amer. 4: 248, 1980 (Schuster 1980c). Bas.: Chiloscyphus lyallii Mitt., Bot. antarct. voy. II (Fl. Nov.-Zel. 2): 140, 1854 (Mitten 1854).

*** Heteroscyphus magellanicus (Steph.) J.J.Engel et R.M.Schust., Nova Hedwigia 39: 400, 1984 [1985] (Engel and Schuster 1984). Bas.: Chiloscyphus magellanicus Steph., Bull. Herb. Boissier (sér. 2) 8 (2): 140 (256), 1908 (Stephani 1908i).

** Heteroscyphus mamillatus Piippo, Nova Hedwigia 56 (3/4): 360, 1993 (Piippo 1993a).

** Heteroscyphus marginatus (Steph.) Fulford, Mem. New York Bot. Gard. 11 (4): 491, 1976 (Fulford 1976). Bas.: Lophocolea marginata Steph., Sp. Hepat. (Stephani) 6: 282, 1922 (Stephani 1922).

*** Heteroscyphus menziesii (Mitt.) J.J.Engel, Polish Bot. J. 58 (1): 99, 2013 (Engel 2013b). Bas.: Chiloscyphus menziesii Mitt., Bot. antarct. voy. II (Fl. Nov.-Zel. 2): 139, 1854 (Mitten 1854).

** Heteroscyphus merapiensis (Steph.) Piippo, Ann. Bot. Fenn. 30 (3): 200, 1993 (Piippo 1993c). Bas.: Chiloscyphus merapiensis Steph., Sp. Hepat. (Stephani) 6: 311, 1922 (Stephani 1922).

** Heteroscyphus miradorensis (Steph.) Schiffn., Österr. Bot. Z. 60 (5): 172, 1910 (Schiffner 1910a). Bas.: Chiloscyphus miradorensis Steph., Bull. Herb. Boissier (sér. 2) 8 (1): 56 (232), 1908 (Stephani 1908k).

*** Heteroscyphus mononuculus J.J.Engel, J. Hattori Bot. Lab. 90: 241, 2001 (Engel 2001).

** Heteroscyphus mononuculus var. ammophilopsis J.J.Engel, Nova Hedwigia 99 (1/2): 166, 2014 (Engel 2014).

** Heteroscyphus mononuculus var. bilobus J.J.Engel, Nova Hedwigia 99 (1/2): 167, 2014 (Engel 2014).

** Heteroscyphus montagnei (Steph.) Fulford, Mem. New York Bot. Gard. 11 (4): 497, 1976 (Fulford 1976). Bas.: Chiloscyphus montagnei Steph., Bull. Herb. Boissier (sér. 2) 8 (2): 141 (257), 1908 (Stephani 1908i).

** Heteroscyphus multifidus (Steph.) J.J.Engel et R.M.Schust., Nova Hedwigia 39: 400, 1984 [1985] (Engel and Schuster 1984). Bas.: Chiloscyphus multifidus Steph., Bull. Herb. Boissier (sér. 2) 8 (2): 132 (248), 1908 (Stephani 1908i).

** Heteroscyphus multifidus var. subintegerrimus J.J.Engel, Nova Hedwigia 100 (3/4): 562, 2015 (Engel 2015a).

** Heteroscyphus nadeaudii (Steph.) Schiffn., Österr. Bot. Z. 60 (5): 172, 1910 (Schiffner 1910a). Bas.: Chiloscyphus nadeaudii Steph., Bull. Herb. Boissier (sér. 2) 7 (10): 848 (220), 1907 (Stephani 1907b).

** Heteroscyphus orbiculatus A.Srivast. et S.C.Srivast., Indian Geocalyc.: 140, 2002 (Srivastava and Srivastava 2002).

** Heteroscyphus palniensis A.Srivast. et S.C.Srivast., Indian Geocalyc.: 130, 2002 (Srivastava and Srivastava 2002).

** Heteroscyphus pandei S.C.Srivast. et A.Srivast., Lindbergia 15 (6): 197, 1989 [1991] (Srivastava and Srivastava 1989b).

*** Heteroscyphus parallelifolius J.J.Engel, Polish Bot. J. 58 (1): 99, 2013 (Engel 2013b).

** Heteroscyphus parvulus (Schiffn.) Schiffn., Österr. Bot. Z. 60 (5): 171, 1910 (Schiffner 1910a). Bas.: Chiloscyphus parvulus Schiffn., Hep. Fl. Buitenzorg: 206, 1900 (Schiffner 1900a).

** Heteroscyphus parvus A.Srivast. et S.C.Srivast., Indian Geocalyc.: 112, 2002 (Srivastava and Srivastava 2002).

** Heteroscyphus perfoliatus (Mont.) Schiffn., Österr. Bot. Z. 60 (5): 171, 1910 (Schiffner 1910a). Bas.: Lophocolea perfoliata Mont., Ann. Sci. Nat. Bot. (sér. 2) 18: 12, 1842 (Montagne 1842b).

** Heteroscyphus pertusus (Lehm.) Fulford, Mem. New York Bot. Gard. 11 (4): 505, 1976 (Fulford 1976). Bas.: Chiloscyphus pertusus Lehm., Nov. Stirp. Pug. 10: 7, 1857 (Lehmann 1857).

*** Heteroscyphus planiusculus (Hook.f. et Taylor) J.J.Engel, J. Hattori Bot. Lab. 68: 315, 1990 (Engel 1990a). Bas.: Jungermannia planiuscula Hook.f. et Taylor, London J. Bot. 3: 382, 1844 (Hooker and Taylor 1844a).

** Heteroscyphus planus (Mitt.) Schiffn., Österr. Bot. Z. 60 (5): 171, 1910 (Schiffner 1910a). Bas.: Chiloscyphus planus Mitt., J. Linn. Soc., Bot. 8 (31): 157, 1864 [1865] (Mitten 1864a).

*** Heteroscyphus polyblepharis (Spruce) Schiffn., Österr. Bot. Z. 60 (5): 172, 1910 (Schiffner 1910a). Bas.: Chiloscyphus polyblepharis Spruce, Trans. & Proc. Bot. Soc. Edinburgh 15: 442, 1885 (Spruce 1885).

*** Heteroscyphus polychaetus (Spruce) Hentschel et Heinrichs, Taxon 56 (4): 1139, 2007 (Hentschel et al. 2007c). Bas.: Lophocolea polychaeta Spruce, Trans. & Proc. Bot. Soc. Edinburgh 15: 436, 1885 (Spruce 1885).

** Heteroscyphus polycladus (Hook.f. et Lév.) R.M.Schust., Hepat. Anthocerotae N. Amer. 4: 248, 1980 (Schuster 1980c). Bas.: Jungermannia polyclada Hook.f. et Lév., Choix Pl. Nouv.-Zel.: 8, 1846 (Raoul 1846).

** Heteroscyphus rectangulatus (Herzog) Piippo, Ann. Bot. Fenn. 30 (3): 200, 1993 (Piippo 1993c). Bas.: Chiloscyphus rectangulatus Herzog, Ann. Naturhist. Mus. Wien 53 (1): 364, 1942 [1943] (Herzog 1942b).

** Heteroscyphus saccogynoides Herzog, J. Hattori Bot. Lab. 14: 40, 1955 (Herzog and Noguchi 1955).

** Heteroscyphus sarawaketanus Piippo, Acta Bot. Fenn. 131: 143, 1985 (Piippo 1985a).

*** Heteroscyphus sinuosus (Hook.) Schiffn., Österr. Bot. Z. 60 (5): 172, 1910 (Schiffner 1910a). Bas.: Jungermannia sinuosa Hook., Musci Exot. 2: tab. 113, 1820 (Hooker 1820).

** Heteroscyphus spectabilis (Steph.) Schiffn., Österr. Bot. Z. 60 (5): 172, 1910 (Schiffner 1910a). Bas.: Chiloscyphus spectabilis Steph., Hedwigia 30 (5): 205, 1891 (Stephani 1891a).

** Heteroscyphus spinifer C.Gao, T.Cao et Y.H.Wu, J. Bryol. 26 (2): 97, 2004 (Gao et al. 2004).

*** Heteroscyphus splendens (Lehm. et Lindenb.) Grolle, Acta Bot. Fenn. 125: 68, 1984 (Grolle and Piippo 1984). Bas.: Jungermannia splendens Lehm. et Lindenb., Nov. Stirp. Pug. 4: 22, 1832 (Lehmann 1832).

** Heteroscyphus splendidus (E.A.Hodgs.) J.J.Engel et R.M.Schust., Nova Hedwigia 39: 400, 1984 [1985] (Engel and Schuster 1984). Bas.: Chiloscyphus splendidus E.A.Hodgs., Trans. Roy. Soc. New Zealand, Bot. 3 (11): 180, 1967 (Hodgson 1967).

*** Heteroscyphus stolonifer J.J.Engel, Polish Bot. J. 58 (1): 102, 2013 (Engel 2013b).

*** Heteroscyphus succulentus (Gottsche) Schiffn., Österr. Bot. Z. 60 (5): 171, 1910 (Schiffner 1910a). Bas.: Chiloscyphus succulentus Gottsche, Natuurk. Tijdschr. Ned.-Indië 4: 576, 1853 (Gottsche 1853).

** Heteroscyphus supinus (Hook.f. et Taylor) R.M.Schust., Hepat. Anthocerotae N. Amer. 4: 248, 1980 (Schuster 1980c). Bas.: Chiloscyphus supinus Hook.f. et Taylor, London J. Bot. 5: 284, 1846 (Taylor 1846a).

** Heteroscyphus tener (Steph.) Schiffn., Österr. Bot. Z. 60 (5): 172, 1910 (Schiffner 1910a). Bas.: Chiloscyphus tener Steph., Bull. Herb. Boissier (sér. 2) 7 (8): 695 (205), 1907 (Stephani 1907e).

*** Heteroscyphus thraustus (Spruce) Fulford, Mem. New York Bot. Gard. 11 (4): 495, 1976 (Fulford 1976). Bas.: Lophocolea thrausta Spruce, Trans. & Proc. Bot. Soc. Edinburgh 15: 437, 1885 (Spruce 1885).

** Heteroscyphus timppae Piippo, Ann. Bot. Fenn. 29 (3): 246, 1992 (Piippo 1992).

*** Heteroscyphus triacanthus (Hook.f. et Lév.) Schiffn., Österr. Bot. Z. 60 (5): 172, 1910 (Schiffner 1910a). Bas.: Jungermannia triacantha Hook.f. et Lév., Choix Pl. Nouv.-Zel.: 8, 1846 (Raoul 1846).

** Heteroscyphus triacanthus var. magnistipulatus J.J.Engel, Nova Hedwigia 99 (1/2): 167, 2014 (Engel 2014).

** Heteroscyphus tridentatus (Sande Lac.) Grolle, Acta Bot. Fenn. 125: 68, 1984 (Grolle and Piippo 1984). Bas.: Lophocolea tridentata Sande Lac., Ann. Mus. Bot. Lugduno-Batavi 1: 296, 1864 (Sande Lacoste 1864).

** Heteroscyphus turgidus (Schiffn.) Schiffn., Österr. Bot. Z. 60 (5): 171, 1910 (Schiffner 1910a). Bas.: Chiloscyphus turgidus Schiffn., Denkschr. Kaiserl. Akad. Wiss., Math.-Naturwiss. Kl. 70: 212, 1900 [1901] (Schiffner 1900c).

*** Heteroscyphus valdiviensis (Mont.) Schiffn., Österr. Bot. Z. 60 (5): 172, 1910 (Schiffner 1910a). Bas.: Chiloscyphus valdiviensis Mont., Ann. Sci. Nat. Bot. (sér. 3) 4: 351, 1845 (Montagne 1845b).

** Heteroscyphus varians (Steph.) J.J.Engel, J. Hattori Bot. Lab. 68: 315, 1990 (Engel 1990a). Bas.: Lophocolea varians Steph., J. & Proc. Roy. Soc. New South Wales 48 (1/2): 119, 1914 (Stephani and Watts 1914).

** Heteroscyphus wettsteinii (Schiffn.) Schiffn., Österr. Bot. Z. 60 (5): 172, 1910 (Schiffner 1910a). Bas.: Chiloscyphus wettsteinii Schiffn., Hep. Fl. Buitenzorg: 202, 1900 (Schiffner 1900a).

** Heteroscyphus zollingeri (Gottsche) Schiffn., Österr. Bot. Z. 60 (5): 171, 1910 (Schiffner 1910a). Bas.: Chiloscyphus zollingeri Gottsche, Natuurk. Tijdschr. Ned.-Indië 4: 576, 1853 (Gottsche 1853).

*** **Lamellocolea J.J.Engel**, J. Hattori Bot. Lab. 70: 65, 1991 (Engel 1991c).

*** Lamellocolea granditexta (Steph.) J.J.Engel, J. Hattori Bot. Lab. 70: 66, 1991 (Engel 1991c). Bas.: Lophocolea granditexta Steph., Bull. Herb. Boissier (sér. 2) 6 (10): 881 (106), 1906 (Stephani 1906d).

*** Lamellocolea integrostia J.J.Engel et Glenny, Bryologist 114 (1): 23, 2011 (Engel and Glenny 2011).

*** **Leptophyllopsis R.M.Schust.**, J. Hattori Bot. Lab. 26: 269, 1963 (Schuster 1963b).

*** Leptophyllopsis laxa (Mitt.) R.M.Schust. ex Hamlin, Rec. Domin. Mus. 7: 284, 1972 (Hamlin 1972). Bas.: Chiloscyphus laxus Mitt., Bot. antarct. voy. II (Fl. Nov.-Zel. 2): 142, 1854 (Mitten 1854).

*** **Leptoscyphopsis R.M.Schust.**, Phytologia 39 (4): 246, 1978 (Schuster 1978a).

*** Leptoscyphopsis paradoxa R.M.Schust., Phytologia 39 (4): 246, 1978 (Schuster 1978a).

*** **Leptoscyphus Mitt.**, Hooker’s J. Bot. Kew Gard. Misc. 3: 358, 1851 (Mitten 1851). ^145^

** **subg. Anomylia (R.M.Schust.) R.M.Schust.**, Hepat. Anthocerotae N. Amer. 4: 272, 1980 (Schuster 1980c). Bas.: Anomylia R.M.Schust., Amer. Midl. Naturalist 62 (1): 51, 1959 (Schuster 1959a).

*** Leptoscyphus cuneifolius (Hook.) Mitt., Hooker’s J. Bot. Kew Gard. Misc. 3: 358, 1851 (Mitten 1851). Bas.: Jungermannia cuneifolia Hook., Brit. Jungermann.: tab. 64, 1814 (Hooker 1814).

** Leptoscyphus cuneifolius subsp. fragilis (J.B.Jack et Steph.) Grolle, Nova Acta Leop. (n.ser.) 25 (161): 28, 1962 [1963] (Grolle 1962a). Bas.: Leioscyphus fragilis J.B.Jack et Steph., Hedwigia 31 (1): 20, 1892 (Jack and Stephani 1892).

** **subg. Austroleptoscyphus Vanderp., Schäf.-Verw. et D.G.Long**, Taxon 59 (1): 183, 2010 (Vanderpoorten et al. 2010).

*** Leptoscyphus antarcticus (C.Massal.) Solari, Cryptog. Bryol. Lichénol. 7 (3): 219, 1986 (Solari 1986). Bas.: Leioscyphus antarcticus C.Massal., Atti Reale Ist. Veneto Sci. Lett. Arti 87 (2): 229, 1928 (Massalongo 1928).

*** Leptoscyphus australis (Gottsche, Lindenb. et Nees) R.M.Schust., J. Hattori Bot. Lab. 26: 270, 1963 (Schuster 1963b). Bas.: Chiloscyphus australis Gottsche, Lindenb. et Nees, Syn. Hepat. 2: 189, 1845 (Gottsche et al. 1845a).

*** Leptoscyphus belmoranus (Steph.) J.J.Engel, J. Hattori Bot. Lab. 74: 33, 1993 (Engel 1993). Bas.: Lophocolea belmorana Steph., J. & Proc. Roy. Soc. New South Wales 48 (1/2): 117, 1914 (Stephani and Watts 1914).

*** Leptoscyphus excipulatus (Steph.) J.J.Engel, J. Hattori Bot. Lab. 74: 33, 1993 (Engel 1993). Bas.: Lophocolea excipulata Steph., Bull. Herb. Boissier (sér. 2) 6 (9): 790 (90), 1906 (Stephani 1906e).

*** Leptoscyphus innovatus (E.A.Hodgs.) J.J.Engel, J. Hattori Bot. Lab. 74: 33, 1993 (Engel 1993). Bas.: Lophocolea innovata E.A.Hodgs., Trans. Roy. Soc. New Zealand 80 (3/4): 347, 1952 [1953] (Hodgson 1952).

*** Leptoscyphus longistipulus (Steph.) J.J.Engel, Bryologist 94 (4): 436, 1991 (Engel 1991b). Bas.: Lophocolea longistipula Steph., Bull. Herb. Boissier (sér. 2) 6 (10): 884 (109), 1906 (Stephani 1906d).

** **subg. Leptoscyphus**

** **sect. Hexagonistipa Grolle**, Nova Acta Leop. (n.ser.) 25 (161): 46, 1962 (Grolle 1962a).

*** Leptoscyphus gibbosus (Taylor) Mitt., Hooker’s J. Bot. Kew Gard. Misc. 3: 358, 1851 (Mitten 1851). Bas.: Chiloscyphus gibbosus Taylor, London J. Bot. 5: 283, 1846 (Taylor 1846a).

*** Leptoscyphus gradsteinii Vanderp., Schäf.-Verw. et D.G.Long, Taxon 59 (1): 182, 2010 (Vanderpoorten et al. 2010).

*** Leptoscyphus hexagonus (Nees) Grolle, Nova Acta Leop. (n.ser.) 25 (161): 47, 1962 [1963] (Grolle 1962a). Bas.: Chiloscyphus hexagonus Nees, Syn. Hepat. 2: 177, 1845 (Gottsche et al. 1845a).

*** Leptoscyphus jackii (Steph.) Grolle, Nova Acta Leop. (n.ser.) 25 (161): 48, 1962 [1963] (Grolle 1962a). Bas.: Leioscyphus jackii Steph., Hedwigia 31 (1): 21, 1892 (Jack and Stephani 1892).

*** Leptoscyphus physocalyx (Hampe et Gottsche) Gottsche, Bot. Zeitung (Berlin) Beil. 16: 33, 1858 (Gottsche 1858). Bas.: Jungermannia physocalyx Hampe et Gottsche, Linnaea 20 (3): 326, 1847 (Hampe 1847).

*** Leptoscyphus sotiauxii Vanderp., Schäf.-Verw. et D.G.Long, Taxon 59 (1): 183, 2010 (Vanderpoorten et al. 2010).

** **sect. Leptoscyphus**

*** Leptoscyphus aequatus (Hook.f. et Taylor) Mitt., Hooker’s J. Bot. Kew Gard. Misc. 3: 358, 1851 (Mitten 1851). Bas.: Jungermannia aequata Hook.f. et Taylor, London J. Bot. 3: 465, 1844 (Hooker and Taylor 1844b).

*** Leptoscyphus intermedius Grolle, Nova Acta Leop. (n.ser.) 25 (161): 32, 1962 [1963] (Grolle 1962a).

*** Leptoscyphus lambinonii Vanderp., Schäf.-Verw. et D.G.Long, Taxon 59 (1): 179, 2010 (Vanderpoorten et al. 2010).

*** Leptoscyphus obcordatus (Spruce) Grolle, Nova Acta Leop. (n.ser.) 25 (161): 33, 1962 [1963] (Grolle 1962a). Bas.: Leioscyphus obcordatus Spruce, Trans. & Proc. Bot. Soc. Edinburgh 15: 446, 1885 (Spruce 1885).

*** Leptoscyphus ovatus (Spruce) Grolle, Nova Acta Leop. (n.ser.) 25 (161): 45, 1962 [1963] (Grolle 1962a). Bas.: Leioscyphus ovatus Spruce, J. Linn. Soc., Bot. 30 (210): 357, 1895 (Gepp 1895b).

*** Leptoscyphus porphyrius (Nees) Grolle, Österr. Bot. Z. 117 (1): 3, 1969 (Grolle 1969a). Bas.: Chiloscyphus porphyrius Nees, Syn. Hepat. 2: 185, 1845 (Gottsche et al. 1845a).

*** Leptoscyphus porphyrius subsp. azoricus (H.Buch et Perss.) Vanderp. et Heinrichs, Taxon 59 (1): 181, 2010 (Vanderpoorten et al. 2010). Bas.: Mylia azorica H.Buch et Perss., Bryophyt. Azoren Madeira: 7, 1941 (Buch and Persson 1941).

** **subg. Physoscyphus Grolle**, Nova Acta Leop. (n.ser.) 25 (161): 51, 1962 (Grolle 1962a).

** **sect. Homaloscyphus Grolle**, Nova Acta Leop. (n.ser.) 25 (161): 58, 1962 (Grolle 1962a).

*** Leptoscyphus chilensis (De Not.) Hässel, J. Hattori Bot. Lab. 91: 207, 2001 (Hässel 2001). Bas.: Lophocolea chilensis De Not., Mem. Reale Accad. Sci. Torino (ser. 2) 16: 222, 1857 (De Notaris 1857).

* Leptoscyphus difficilis (Steph.) Fulford, Mem. New York Bot. Gard. 11 (4): 534, 1976 (Fulford 1976). Bas.: Chiloscyphus difficilis Steph., Biblioth. Bot. 87 (2): 222, 1916 (Stephani 1916a). ^146^

*** Leptoscyphus diversifolius (Gottsche) Grolle, Nova Acta Leop. (n.ser.) 25 (161): 58, 1962 [1963] (Grolle 1962a). Bas.: Lophocolea diversifolia Gottsche, Syn. Hepat. 2: 166, 1845 (Gottsche et al. 1845a).

*** Leptoscyphus expansus (Lehm.) Grolle, Nova Acta Leop. (n.ser.) 25 (161): 60, 1962 [1963] (Grolle 1962a). Bas.: Jungermannia expansa Lehm., Linnaea 4: 361, 1829 (Lehmann 1829).

*** Leptoscyphus hedbergii (S.W.Arnell) R.M.Schust., Amer. Midl. Naturalist 62 (1): 13, 1959 (Schuster 1959a). Bas.: Mylia hedbergii S.W.Arnell, Ark. Bot. (n.ser.) 3 (16): 547, 1956 (Arnell 1956e).

* Leptoscyphus huidobroanus (Mont.) Gottsche, Bot. Zeitung (Berlin) Beil. 16: 33, 1858 (Gottsche 1858). Bas.: Chiloscyphus huidobroanus Mont., Ann. Sci. Nat. Bot. (sér. 3) 4: 352, 1845 (Montagne 1845b). ^147^

*** Leptoscyphus huonicus Piippo, Acta Bot. Fenn. 131: 152, 1985 (Piippo 1985a).

*** Leptoscyphus magellanicus (Gola) Hässel, J. Hattori Bot. Lab. 91: 214, 2001 (Hässel 2001). Bas.: Lophozia magellanica Gola, Nuovo Giorn. Bot. Ital. (n.ser.) 29 (1/4): 165, 1922 [1923] (Gola 1922).

** **sect. Physoscyphus Grolle**, Nova Acta Leop. (n.ser.) 25 (161): 51, 1962 (Grolle 1962a).

*** Leptoscyphus amphibolius (Nees) Grolle, Nova Acta Leop. (n.ser.) 25 (161): 54, 1962 [1963] (Grolle 1962a). Bas.: Jungermannia amphibolia Nees, Fl. Bras. (Martius) 1 (1): 334, 1833 (Nees 1833a).

*** Leptoscyphus infuscatus (Mitt.) E.W.Jones ex Grolle, Nova Acta Leop. (n.ser.) 25 (161): 52, 1962 [1963] (Grolle 1962a). Bas.: Leioscyphus infuscatus Mitt., J. Linn. Soc., Bot. 22 (146): 321, 1886 (Mitten 1886b).

** **subg. Spinoscyphus Vanderp., Schäf.-Verw. et D.G.Long**, Taxon 59 (1): 183, 2010 (Vanderpoorten et al. 2010).

*** Leptoscyphus cleefii Fulford, Mem. New York Bot. Gard. 11 (4): 534, 1976 (Fulford 1976).

*** Leptoscyphus spectabilis (Steph.) Grolle, J. Bryol. 11 (2): 328, 1980 [1981] (Grolle 1980c). Bas.: Lophocolea spectabilis Steph., Bull. Herb. Boissier (sér. 2) 7 (4): 302 (166), 1907 (Stephani 1907d).

***Incertae sedis***

*** Leptoscyphus autoicus (J.J.Engel et Gradst.) Vanderp. et Gradst., J. Bryol. 34 (4): 252, 2012 (Vanderpoorten et al. 2012). Bas.: Physotheca autoica J.J.Engel et Gradst., Taxon 52 (4): 764, 2003 (Engel and Gradstein 2003).

** Leptoscyphus beckettianus (Steph.) R.M.Schust. ex J.J.Engel, Nova Hedwigia 93 (3/4): 402, 2011 (Engel 2011). Bas.: Chiloscyphus beckettianus Steph., Bull. Herb. Boissier (sér. 2) 8 (1): 59 (235), 1908 (Stephani 1908k).

** Leptoscyphus compactus (Colenso) J.J.Engel, Nova Hedwigia 100 (3/4): 579, 2015 (Engel 2015a). Bas.: Chiloscyphus compactus Colenso, Trans. & Proc. New Zealand Inst. 21: 63, 1889 (Colenso 1889).

** Leptoscyphus erraticus (W.Martin et E.A.Hodgs.) J.J.Engel, Nova Hedwigia 99 (1/2): 168, 2014 (Engel 2014). Bas.: Chiloscyphus erraticus W.Martin et E.A.Hodgs., Trans. & Proc. Roy. Soc. New Zealand 78 (4): 497, 1950 (Martin 1950).

*** Leptoscyphus horizontalis (Hook.) Kühnem., Revista Centro Estud. Doct. Ci. Nat. 1: 176, 1937 (Kühnemann 1937). Bas.: Jungermannia horizontalis Hook., Musci Exot. 1: tab. 96, 1818 (Hooker 1818).

** Leptoscyphus normalis (Steph.) J.J.Engel, Nova Hedwigia 100 (3/4): 579, 2015 (Engel 2015a). Bas.: Lophocolea normalis Steph., Sp. Hepat. (Stephani) 6: 285, 1922 (Stephani 1922).

** Leptoscyphus physanthus (Hook.f. et Taylor) J.J.Engel, Nova Hedwigia 99 (1/2): 168, 2014 (Engel 2014). Bas.: Jungermannia physantha Hook.f. et Taylor, London J. Bot. 3: 561, 1844 (Hooker and Taylor 1844d).

** Leptoscyphus subemarginatus (Hook.f. et Taylor) J.J.Engel, Bryologist 94 (4): 436, 1991 (Engel 1991b). Bas.: Lophocolea subemarginata Hook.f. et Taylor, London J. Bot. 5: 367, 1846 (Taylor 1846b).

*** Leptoscyphus trapezoïdes (Mont.) L.Söderstr., Phytotaxa 112 (1): 27, 2013 (Söderström et al. 2013f). Bas.: Lophocolea trapezoïdes Mont., Ann. Sci. Nat. Bot. (sér. 2) 19: 251, 1843 (Montagne 1843).

*** **Lophocolea (Dumort.) Dumort.**, Recueil Observ. Jungerm.: 17, 1835 (Dumortier 1835). Bas.: Jungermannia sect. Lophocolea Dumort., Syll. Jungerm. Europ.: 59, 1831 (Dumortier 1831).

*** Lophocolea aberrans Lindenb. et Gottsche, Syn. Hepat. 5: 696, 1847 (Gottsche et al. 1847).

*** Lophocolea aequifolia Nees et Mont., Ann. Sci. Nat. Bot. (sér. 2) 5: 55, 1836 (Nees and Montagne 1836).

* Lophocolea angustistipula Steph., Sp. Hepat. (Stephani) 6: 260, 1922 (Stephani 1922).

*** Lophocolea anisoloba (J.J.Engel et Glenny) L.Söderstr., Phytotaxa 112 (1): 25, 2013 (Söderström et al. 2013f). Bas.: Chiloscyphus anisolobus J.J.Engel et Glenny, Bryologist 111 (1): 118, 2008 (Engel and Glenny 2008b).

** Lophocolea anomala Steph., Sp. Hepat. (Stephani) 6: 300, 1922 (Stephani 1922).

** Lophocolea anomoda (Mont.) Steph., Hedwigia 32 (5): 327, 1893 (Stephani 1893d). Bas.: Chiloscyphus anomodus Mont., Ann. Sci. Nat. Bot. (sér. 3) 4: 352, 1845 (Montagne 1845b).

** Lophocolea apalachicola R.M.Schust., Hepat. Anthocerotae N. Amer. 4: 195, 1980 (Schuster 1980c).

*** Lophocolea aperticaulis (J.J.Engel) L.Söderstr., Phytotaxa 112 (1): 25, 2013 (Söderström et al. 2013f). Bas.: Chiloscyphus aperticaulis J.J.Engel, J. Hattori Bot. Lab. 95: 229, 2004 (Engel 2004b).

** Lophocolea aphelophylla (Hässel) Váňa, Phytotaxa 112 (1): 25, 2013 (Söderström et al. 2013f). Bas.: Chiloscyphus aphelophyllus Hässel, J. Hattori Bot. Lab. 98: 123, 2005 (Hässel 2005).

** Lophocolea apophylla (Hässel) Váňa, Phytotaxa 112 (1): 25, 2013 (Söderström et al. 2013f). Bas.: Chiloscyphus apophyllus Hässel, J. Hattori Bot. Lab. 98: 126, 2005 (Hässel 2005).

*** Lophocolea appalachiana R.M.Schust., Hepat. Anthocerotae N. Amer. 4: 208, 1980 (Schuster 1980c).

** Lophocolea ascensionis Steph., Sp. Hepat. (Stephani) 6: 261, 1922 (Stephani 1922).

** Lophocolea asperrima Steph., Bull. Herb. Boissier (sér. 2) 6 (11): 949 (129), 1906 (Stephani 1906c).

** Lophocolea atra Gola, Nuovo Giorn. Bot. Ital. (n.ser.) 29 (1/4): 167, 1922 [1923] (Gola 1922).

*** Lophocolea attenuata Steph., Bih. Kongl. Svenska Vetensk.-Akad. Handl. 26 (III, 6): 34, 1900 (Stephani 1900b).

*** Lophocolea australis Gottsche, Linnaea 28 (5): 553, 1856 [1857] (Gottsche 1856).

** Lophocolea autoica Steph., Sp. Hepat. (Stephani) 6: 262, 1922 (Stephani 1922).

** Lophocolea baldwinii Steph., Bull. Herb. Boissier (sér. 2) 6 (11): 950 (130), 1906 (Stephani 1906c).

** Lophocolea bartlettii H.A.Mill., Ark. Bot. (n.ser.) 5 (2): 506, 1963 (Miller 1963).

** Lophocolea bewsii (Sim) Grolle, Trans. Brit. Bryol. Soc. 3 (4): 588, 1959 (Grolle 1959b). Bas.: Leptoscyphus bewsii Sim, Trans. Roy. Soc. South Africa 15 (1): 103, 1926 (Sim 1926).

** Lophocolea bicuspidata Steph., Sp. Hepat. (Stephani) 6: 263, 1922 (Stephani 1922).

*** Lophocolea bidentata (L.) Dumort., Recueil Observ. Jungerm.: 17, 1835 (Dumortier 1835). Bas.: Jungermannia bidentata L., Sp. Pl. 1: 1132, 1753 (Linnaeus 1753). ^148^

** Lophocolea bifidistipula Steph., Sp. Hepat. (Stephani) 6: 264, 1922 (Stephani 1922).

*** Lophocolea bispinosa (Hook.f. et Taylor) Gottsche, Lindenb. et Nees, Syn. Hepat. 2: 162, 1845 (Gottsche et al. 1845a). Bas.: Jungermannia bispinosa Hook.f. et Taylor, London J. Bot. 3: 378, 1844 (Hooker and Taylor 1844a).

** Lophocolea bootanensis Steph., Sp. Hepat. (Stephani) 6: 265, 1922 (Stephani 1922).

** Lophocolea boulyana Steph., Sp. Hepat. (Stephani) 6: 264, 1922 (Stephani 1922).

** Lophocolea bowiena Steph., J. & Proc. Roy. Soc. New South Wales 48 (1/2): 117, 1914 (Stephani and Watts 1914).

*** Lophocolea brookwoodiana Paton et Sheahan, J. Bryol. 28 (3): 163, 2006 (Paton and Sheahan 2006).

** Lophocolea caespitans Steph., Bull. Herb. Boissier (sér. 2) 6 (11): 949 (129), 1906 (Stephani 1906c).

*** Lophocolea calcarea Steph., Bull. Herb. Boissier (sér. 2) 6 (10): 884 (109), 1906 (Stephani 1906d).

** Lophocolea caledonica Steph., Sp. Hepat. (Stephani) 6: 267, 1922 (Stephani 1922).

*** Lophocolea canaliculata (Gottsche, Lindenb. et Nees) Steph., Bull. Herb. Boissier (sér. 2) 6 (9): 786 (86), 1906 (Stephani 1906e). Bas.: Chiloscyphus canaliculatus Gottsche, Lindenb. et Nees, Syn. Hepat. 5: 710, 1847 (Gottsche et al. 1847).

*** Lophocolea canaliculata var. concava (J.J.Engel) L.Söderstr., Phytotaxa 112 (1): 26, 2013 (Söderström et al. 2013f). Bas.: Chiloscyphus canaliculatus var. concavus J.J.Engel, Fieldiana, Bot. (n.ser.) 48: 107, 2010 (Engel 2010).

** Lophocolea cervicornis Steph., Biblioth. Bot. 87 (2): 219, 1916 (Stephani 1916a).

** Lophocolea ciliifera Steph., Bull. Herb. Boissier (sér. 2) 6 (8): 660 (76), 1906 (Stephani 1906h).

* Lophocolea coadunata (Sw.) Mont., Voy. Amér. Mérid., Bot. 7 (1): 76, 1839 (Montagne 1839b). Bas.: Jungermannia coadunata Sw., Fl. Ind. Occid. 3: 1850, 1806 (Swartz 1806). ^149^

** Lophocolea concreta Mont., Ann. Sci. Nat. Bot. (sér. 3) 4: 350, 1845 (Montagne 1845b).

** Lophocolea convexula Mitt., Fl. vit.: 405, 1871 [1873] (Mitten 1871).

** Lophocolea corticola Steph., Sp. Hepat. (Stephani) 6: 268, 1922 (Stephani 1922).

*** Lophocolea decurrens Herzog, Trans. & Proc. Roy. Soc. New Zealand 65 (3): 352, 1936 (Herzog 1936b).

* Lophocolea deningeri Herzog, Beih. Bot. Centralbl. 38 (2): 321, 1921 (Herzog 1921).

** Lophocolea dentiflora Steph., Bull. Herb. Boissier (sér. 2) 6 (7): 550 (64), 1906 (Stephani 1906f).

** Lophocolea difformis Nees, Syn. Hepat. 2: 166, 1845 (Gottsche et al. 1845a).

** Lophocolea discedens (Lehm. et Lindenb.) Lehm. et Lindenb., Syn. Hepat. 2: 167, 1845 (Gottsche et al. 1845a). Bas.: Jungermannia discedens Lehm. et Lindenb., Nov. Stirp. Pug. 5: 3, 1833 (Lehmann 1833).

* Lophocolea dusenii Steph., Cat. Afr. Pl. (Hiern) 2 (2): 314, 1901 (Stephani 1901d).

*** Lophocolea erosa (J.J.Engel) L.Söderstr., Phytotaxa 112 (1): 26, 2013 (Söderström et al. 2013f). Bas.: Chiloscyphus erosus J.J.Engel, Phytologia 83 (1): 43, 1997 [1998] (Engel 1997).

*** Lophocolea excisifolia Steph., J. & Proc. Roy. Soc. New South Wales 48 (1/2): 118, 1914 (Stephani and Watts 1914).

*** Lophocolea fertilis (J.J.Engel) L.Söderstr., Phytotaxa 112 (1): 26, 2013 (Söderström et al. 2013f). Bas.: Chiloscyphus fertilis J.J.Engel, Phytologia 83 (1): 43, 1997 [1998] (Engel 1997).

** Lophocolea flavicans Steph., Sp. Hepat. (Stephani) 6: 300, 1922 (Stephani 1922).

*** Lophocolea floribunda Steph., Bull. Herb. Boissier (sér. 2) 6 (10): 886 (111), 1906 (Stephani 1906d).

** Lophocolea foliicola Spruce, Trans. & Proc. Bot. Soc. Edinburgh 15: 428, 1885 (Spruce 1885).

** Lophocolea fragillima Steph., Sp. Hepat. (Stephani) 6: 273, 1922 (Stephani 1922).

*** Lophocolea fragmentissima R.M.Schust., Phytologia 39 (4): 245, 1978 (Schuster 1978a).

*** Lophocolea fragrans (Moris et De Not.) Gottsche, Lindenb. et Nees, Syn. Hepat. 2: 166, 1845 (Gottsche et al. 1845a). Bas.: Jungermannia fragrans Moris et De Not., Fl. Caprariae: 177, 1839 (Moris and De Notaris 1839).

** Lophocolea glaziovii Steph., Bull. Herb. Boissier (sér. 2) 6 (11): 961 (141), 1906 (Stephani 1906c).

** Lophocolea gollanii (Steph.) Váňa, Phytotaxa 112 (1): 26, 2013 (Söderström et al. 2013f). Bas.: Chiloscyphus gollanii Steph., Bull. Herb. Boissier (sér. 2) 7 (10): 837 (209), 1907 (Stephani 1907b).

** Lophocolea granatensis Gottsche, Ann. Sci. Nat. Bot. (sér. 5) 1: 126, 1864 (Gottsche 1864).

** Lophocolea griffithiana Steph., Sp. Hepat. (Stephani) 6: 274, 1922 (Stephani 1922).

** Lophocolea hahnii Steph., Bull. Herb. Boissier (sér. 2) 6 (8): 660 (76), 1906 (Stephani 1906h).

** Lophocolea haskarliana Gottsche, Syn. Hepat. 2: 153, 1845 (Gottsche et al. 1845a).

*** Lophocolea hattorii (J.J.Engel) L.Söderstr., Phytotaxa 112 (1): 26, 2013 (Söderström et al. 2013f). Bas.: Chiloscyphus hattorii J.J.Engel, J. Hattori Bot. Lab. 74: 29, 1993 (Engel 1993).

** Lophocolea hawaica Steph., Bull. Herb. Boissier (sér. 2) 6 (11): 945 (125), 1906 (Stephani 1906c).

** Lophocolea heterodonta Steph., Sp. Hepat. (Stephani) 6: 275, 1922 (Stephani 1922).

** Lophocolea heteromorpha Steph., Sp. Hepat. (Stephani) 6: 275, 1922 (Stephani 1922).

*** Lophocolea heterophylla (Schrad.) Dumort., Recueil Observ. Jungerm.: 17, 1835 (Dumortier 1835). Bas.: Jungermannia heterophylla Schrad., J. Bot. (Schrader) 5: 66, 1802 [1803] (Schrader 1802).

** Lophocolea heterophylla subsp. cladogyna R.M.Schust., Hepat. Anthocerotae N. Amer. 4: 223, 1980 (Schuster 1980c).

** Lophocolea horikawana S.Hatt., Bull. Tokyo Sci. Mus. 11: 50, 1944 (Hattori 1944d).

** Lophocolea howeana Steph., J. & Proc. Roy. Soc. New South Wales 48 (1/2): 118, 1914 (Stephani and Watts 1914).

* Lophocolea humifusa (Hook.f. et Taylor) Gottsche, Lindenb. et Nees, Syn. Hepat. 5: 695, 1847 (Gottsche et al. 1847). Bas.: Jungermannia humifusa Hook.f. et Taylor, London J. Bot. 3: 472, 1844 (Hooker and Taylor 1844b).

** Lophocolea humistrata (Hook.f. et Taylor) Gottsche, Lindenb. et Nees, Syn. Hepat. 5: 701, 1847 (Gottsche et al. 1847). Bas.: Jungermannia humistrata Hook.f. et Taylor, London J. Bot. 4: 82, 1845 (Hooker and Taylor 1845).

** Lophocolea itoana Inoue, J. Jap. Bot. 31 (11): 340, 1956 (Inoue 1956).

** Lophocolea javanica Schiffn., Hep. Fl. Buitenzorg: 178, 1900 (Schiffner 1900a).

** Lophocolea koponenii (Piippo) Váňa, Phytotaxa 112 (1): 26, 2013 (Söderström et al. 2013f). Bas.: Chiloscyphus koponenii Piippo, Ann. Bot. Fenn. 35 (1): 55, 1998 (Piippo 1998).

** Lophocolea kurzii Sande Lac., Ann. Mus. Bot. Lugduno-Batavi 1: 296, 1864 (Sande Lacoste 1864).

* Lophocolea kurzii var. siamensis N.Kitag., Acta Phytotax. Geobot. 30 (1/3): 33, 1979 (Kitagawa 1979b).

** Lophocolea laceristipula Steph., Sp. Hepat. (Stephani) 6: 281, 1922 (Stephani 1922).

** Lophocolea latistipula Steph., Sp. Hepat. (Stephani) 6: 281, 1922 (Stephani 1922).

*** Lophocolea lauterbachii Steph., Bull. Herb. Boissier (sér. 2) 6 (11): 938 (118), 1906 (Stephani 1906c).

* Lophocolea laxissima Herzog, Ann. Bryol. 5: 77, 1932 (Herzog 1932b).

** Lophocolea ledermannii Steph., Sp. Hepat. (Stephani) 6: 300, 1922 (Stephani 1922).

*** Lophocolea lenta (Hook.f. et Taylor) Gottsche, Lindenb. et Nees, Syn. Hepat. 2: 162, 1845 (Gottsche et al. 1845a). Bas.: Jungermannia lenta Hook.f. et Taylor, London J. Bot. 3: 379, 1844 (Hooker and Taylor 1844a).

*** Lophocolea leptantha (Hook.f. et Taylor) Gottsche, Lindenb. et Nees, Syn. Hepat. 5: 694, 1847 (Gottsche et al. 1847). Bas.: Jungermannia leptantha Hook.f. et Taylor, London J. Bot. 3: 471, 1844 (Hooker and Taylor 1844b).

*** Lophocolea liebmanniana Gottsche, Mexik. Leverm.: 113, 1863 (Gottsche 1863).

** Lophocolea lindmannii Steph., Bull. Herb. Boissier (sér. 2) 6 (11): 960 (140), 1906 (Stephani 1906c).

*** Lophocolea longiciliata Herzog, Memoranda Soc. Fauna Fl. Fennica 27: 96, 1952 (Herzog 1952c).

** Lophocolea lucida (Spreng.) Mont., Voy. Amér. Mérid., Bot. 7 (2): 78, 1839 (Montagne 1839a). Bas.: Jungermannia lucida Spreng. Nov. Stirp. Pug. 5: 2, 1833 (Lehmann 1833).

** Lophocolea madagascariensis Gottsche, Abh. Naturwiss. Vereins Bremen 7: 344, 1882 (Gottsche 1882).

** Lophocolea magna (Udar et V.Nath) Váňa, Phytotaxa 183 (4): 291, 2014 (Váňa et al. 2014a). Bas.: Cephaloziella magna Udar et V.Nath, Geophytology 6 (1): 105, 1976 (Udar and Nath 1976).

*** Lophocolea mediinfrons (J.J.Engel et Braggins) L.Söderstr., Phytotaxa 112 (1): 26, 2013 (Söderström et al. 2013f). Bas.: Chiloscyphus mediinfrons J.J.Engel et Braggins, Fieldiana, Bot. (n.ser.) 48: 119, 2010 (Engel 2010).

** Lophocolea micronesica Inoue et H.A.Mill., Bull. Natl. Sci. Mus. Tokyo (n.ser.) 11 (1): 4, 1968 (Inoue and Miller 1968).

* Lophocolea microstipula Steph., Bih. Kongl. Svenska Vetensk.-Akad. Handl. 26 (III, 6): 43, 1900 (Stephani 1900b). ^150^

*** Lophocolea minor Nees, Naturgesch. Eur. Leberm. 2: 330, 1836 (Nees 1836).

** Lophocolea minutistipula Steph., Sp. Hepat. (Stephani) 6: 283, 1922 (Stephani 1922).

* Lophocolea mollis (Nees) Nees, Syn. Hepat. 2: 158, 1845 (Gottsche et al. 1845a). Bas.: Jungermannia mollis Nees, Enum. Pl. Crypt. Javae: 24, 1830 (Nees 1830). ^151^

* Lophocolea morobeana Piippo, Acta Bot. Fenn. 131: 160, 1985 (Piippo 1985a). ^152^

** Lophocolea muhavurensis (S.W.Arnell) S.W.Arnell ex Pócs, Acta Bot. Acad. Sci. Hung. 25 (3/4): 227, 1979 [1980] (Bizot and Pócs 1979). Bas.: Chiloscyphus muhavurensis S.W.Arnell, Ark. Bot. (n.ser.) 3 (16): 526, 1956 (Arnell 1956e).

*** Lophocolea muricata (Lehm.) Nees, Syn. Hepat. 2: 169, 1845 (Gottsche et al. 1845a). Bas.: Jungermannia muricata Lehm., Linnaea 4: 363, 1829 (Lehmann 1829).

* Lophocolea muricata var. major Pearson, Forh. Vidensk.-Selsk. Kristiania 1892 (14): 10, 1893 (Pearson 1893).

** Lophocolea nakajimae S.Hatt. et Inoue, J. Hattori Bot. Lab. 21: 221, 1959 (Inoue 1959c).

*** Lophocolea novae-zeelandiae (Lehm. et Lindenb.) Nees, Syn. Hepat. 2: 168, 1845 (Gottsche et al. 1845a). Bas.: Jungermannia novae-zeelandiae Lehm. et Lindenb., Nov. Stirp. Pug. 6: 33, 1834 (Lehmann 1834).

*** Lophocolea novae-zeelandiae var. meridionalis (Steph.) L.Söderstr., Phytotaxa 112 (1): 26, 2013 (Söderström et al. 2013f). Bas.: Lophocolea meridionalis Steph., Bull. Herb. Boissier (sér. 2) 6 (10): 888 (113), 1906 (Stephani 1906d).

** Lophocolea orbigniana Nees et Mont., Ann. Sci. Nat. Bot. (sér. 2) 5: 55, 1836 (Nees and Montagne 1836).

** Lophocolea papulimarginata H.A.Mill., Phytologia 47 (4): 323, 1981 (Miller 1981). *Nom. nov. pro Lophocolea papulosa* Steph., Sp. Hepat. (Stephani) 6: 286, 1922 (Stephani 1922), *nom. illeg*.

** Lophocolea parca (Gottsche) Fulford et Sharp, Mem. New York Bot. Gard. 63: 19, 1990 (Fulford and Sharp 1990). Bas.: Jungermannia parca Gottsche, Mexik. Leverm.: 94, 1863 (Gottsche 1863).

** Lophocolea parva Steph., Sp. Hepat. (Stephani) 6: 287, 1922 (Stephani 1922).

*** Lophocolea parvispinea (J.J.Engel) L.Söderstr., Phytotaxa 112 (1): 26, 2013 (Söderström et al. 2013f). Bas.: Chiloscyphus parvispineus J.J.Engel, Phytologia 83 (1): 44, 1997 [1998] (Engel 1997).

** Lophocolea parvistipula Steph., Sp. Hepat. (Stephani) 6: 287, 1922 (Stephani 1922).

*** Lophocolea patulistipa Steph., Kungl. Svenska Vetensk.-Akad. Handl. (n.ser.) 46 (9): 50, 1911 (Stephani 1911b).

*** Lophocolea perpusilla (Hook.f. et Taylor) Gottsche, Lindenb. et Nees, Syn. Hepat. 2: 163, 1845 (Gottsche et al. 1845a). Bas.: Jungermannia perpusilla Hook.f. et Taylor, London J. Bot. 3: 380, 1844 (Hooker and Taylor 1844a).

** Lophocolea piacenzai (Gola) Váňa, Phytotaxa 112 (1): 26, 2013 (Söderström et al. 2013f). Bas.: Lophozia piacenzai Gola, Atti Reale Accad. Sci. Torino, Cl. Sci. Fis. Mat. Nat. 49: 759, 1914 (Gola 1914b).

** Lophocolea pilistipula Steph., Sp. Hepat. (Stephani) 6: 288, 1922 (Stephani 1922).

* Lophocolea pinnatistipula Steph., Biblioth. Bot. 87 (2): 220, 1916 (Stephani 1916a).

** Lophocolea platensis C.Massal., Atti Accad. Sci. Med. Nat. Ferrara 80 (3/4): 12, 1906 (Massalongo 1906a).

** Lophocolea purpurea Steph., Sp. Hepat. (Stephani) 6: 289, 1922 (Stephani 1922).

** Lophocolea pusilla Steph., Sp. Hepat. (Stephani) 6: 290, 1922 (Stephani 1922).

** Lophocolea randii S.W.Arnell, Svensk Bot. Tidskr. 47 (3): 420, 1953 (Arnell 1953c).

** Lophocolea rara Steph., Sp. Hepat. (Stephani) 6: 290, 1922 (Stephani 1922).

** Lophocolea rectangularis Herzog, Rev. Bryol. Lichénol. 23 (1/2): 43, 1954 (Herzog 1954).

** Lophocolea rectangulata Mitt., Fl. vit.: 404, 1871 [1873] (Mitten 1871). ^153^

*** Lophocolea rupicola Steph., Bull. Herb. Boissier (sér. 2) 6 (10): 874 (99), 1906 (Stephani 1906d).

*** Lophocolea sabuletorum (Hook.f. et Taylor) Gottsche, Lindenb. et Nees, Syn. Hepat. 5: 697, 1847 (Gottsche et al. 1847). Bas.: Jungermannia sabuletorum Hook.f. et Taylor, London J. Bot. 3: 469, 1844 (Hooker and Taylor 1844b).

** Lophocolea salacensis Steph., Bull. Herb. Boissier (sér. 2) 6 (11): 943 (123), 1906 (Stephani 1906c).

** Lophocolea savesiana Steph., Bull. Herb. Boissier (sér. 2) 6 (11): 942 (122), 1906 (Stephani 1906c).

*** Lophocolea semiteres (Lehm.) Mitt., J. Linn. Soc., Bot. 16 (91): 188, 1877 (Mitten 1877). Bas.: Jungermannia semiteres Lehm., Linnaea 4: 363, 1829 (Lehmann 1829). ^154^

*** Lophocolea semiteres var. retusa (J.J.Engel) L.Söderstr., Phytotaxa 112 (1): 27, 2013 (Söderström et al. 2013f). Bas.: Chiloscyphus semiteres var. retusus J.J.Engel, Phytologia 83 (1): 44, 1997 [1998] (Engel 1997).

** Lophocolea serrata Mitt., St. Helena: 368, 1875 (Mitten 1875).

** Lophocolea siamensis Steph., Sp. Hepat. (Stephani) 6: 293, 1922 (Stephani 1922).

** Lophocolea sikkimensis (Steph.) Herzog et Grolle, Rev. Bryol. Lichénol. 27 (3/4): 164, 1958 [1959] (Herzog and Grolle 1958). Bas.: Herpocladium sikkimense Steph., Sp. Hepat. (Stephani) 6: 349, 1922 (Stephani 1922).

* Lophocolea silvestris Gottsche, Abh. Naturwiss. Vereins Bremen 7: 345, 1882 (Gottsche 1882).

** Lophocolea steetziae De Not., Epat. Borneo: 20, 1874 (De Notaris 1874).

*** Lophocolea striatella (C.Massal.) Schiffn., Leberm., Forschungsr. Gazelle 4 (4): 13, 1890 (Schiffner 1890). Bas.: Chiloscyphus striatellus C.Massal., Nuovo Giorn. Bot. Ital. 17 (3): 232, 1885 (Massalongo 1885).

** Lophocolea subbidentata Herzog, Rev. Bryol. Lichénol. 23 (1/2): 43, 1954 (Herzog 1954).

** Lophocolea subcostata Steph., Sp. Hepat. (Stephani) 6: 295, 1922 (Stephani 1922).

*** Lophocolea subporosa Mitt., Bot. antarct. voy. II (Fl. Nov.-Zel. 2): 137, 1854 (Mitten 1854).

** Lophocolea subporosa var. inflexifolia (Steph.) L.Söderstr., Phytotaxa 112 (1): 27, 2013 (Söderström et al. 2013f). Bas.: Lophocolea inflexifolia Steph., Sp. Hepat. (Stephani) 6: 278, 1922 (Stephani 1922).

** Lophocolea subviridis (Hook.f. et Taylor) Gottsche, Lindenb. et Nees, Syn. Hepat. 5: 699, 1847 (Gottsche et al. 1847). Bas.: Jungermannia subviridis Hook.f. et Taylor, London J. Bot. 3: 473, 1844 (Hooker and Taylor 1844b).

** Lophocolea sumatrana Schiffn., Denkschr. Kaiserl. Akad. Wiss., Math.-Naturwiss. Kl. 70: 195, 1900 [1901] (Schiffner 1900c).

*** Lophocolea sylvatica Mitt., Rep. Challenger, Bot. 1 (3, 1): 84, 1884 (Mitten 1884b).

** Lophocolea tenera Ångstr., Öfvers. Kongl. Vetensk.-Akad. Förh. 33 (7): 79, 1876 [1877] (Ångström 1876).

** Lophocolea tenerrima Spruce, Trans. & Proc. Bot. Soc. Edinburgh 15: 439, 1885 (Spruce 1885).

** Lophocolea teptepensis Piippo, Acta Bot. Fenn. 131: 163, 1985 (Piippo 1985a).

*** Lophocolea textilis (Hook.f. et Taylor) Gottsche, Lindenb. et Nees, Syn. Hepat. 5: 696, 1847 (Gottsche et al. 1847). Bas.: Jungermannia textilis Hook.f. et Taylor, London J. Bot. 3: 468, 1844 (Hooker and Taylor 1844b).

** Lophocolea textiloidea J.J.Engel, Phytologia 41 (5): 311, 1979 (Engel 1979a). *Nom. nov. pro Chiloscyphus lucidus* Mitt., J. Linn. Soc., Bot. 15 (82): 64, 1876 (Mitten 1876a), *nom. illeg*.

*** Lophocolea trichocoleoides (Glenny, J.J.Engel et He-Nygrén) L.Söderstr., Phytotaxa 112 (1): 27, 2013 (Söderström et al. 2013f). Bas.: Chiloscyphus trichocoleoides Glenny, J.J.Engel et He-Nygrén, J. Bryol. 31 (2): 100, 2009 (Glenny et al. 2009).

** Lophocolea tricuspidata Herzog, Rev. Bryol. Lichénol. 11 (1): 17, 1938 [1939] (Herzog 1938a).

*** Lophocolea tristaniana S.W.Arnell, Results Norweg. Sci. Exped. Tristan da Cunha 42: 19, 1958 (Arnell 1958b).

* Lophocolea undulata Mont., Ann. Sci. Nat. Bot. (sér. 3) 4: 351, 1845 (Montagne 1845b).

*** Lophocolea villosa Mitt., Sp. Hepat. (Stephani) 6: 299, 1922 (Stephani 1922).

** Lophocolea wacei (S.W.Arnell ex J.J.Engel et Váňa) Váňa et L.Söderstr., Phytotaxa 112 (1): 27, 2013 (Söderström et al. 2013f). Bas.: Chiloscyphus wacei S.W.Arnell ex J.J.Engel et Váňa, Mem. New York Bot. Gard. 105: 48, 2013 (Váňa and Engel 2013).

*** Lophocolea wambana Piippo, Acta Bot. Fenn. 131: 163, 1985 (Piippo 1985a).

** Lophocolea werthii (J.J.Engel et R.M.Schust.) Váňa et L.Söderstr., Phytotaxa 112 (1): 27, 2013 (Söderström et al. 2013f). Bas.: Chiloscyphus werthii J.J.Engel et R.M.Schust., Nova Hedwigia 39: 425, 1984 [1985] (Engel and Schuster 1984).

** Lophocolea widgrenii Steph., Bull. Herb. Boissier (sér. 2) 7 (1): 66 (154), 1907 (Stephani 1907c). *Nom. nov. pro Lophocolea pallida* Ångstr., Öfvers. Kongl. Vetensk.-Akad. Förh. 33 (7): 80, 1876 [1877] (Ångström 1876), *nom. illeg*.

** **Otoscyphus J.J.Engel, Bardat et Thouvenot**, Cryptog. Bryol. 33 (3): 280, 2012 (Engel et al. 2012).

*** Otoscyphus crassicaulis (Steph.) J.J.Engel, Bardat et Thouvenot, Cryptog. Bryol. 33 (3): 280, 2012 (Engel et al. 2012). Bas.: Lophocolea crassicaulis Steph., Sp. Hepat. (Stephani) 6: 268, 1922 (Stephani 1922).

*** **Pachyglossa Herzog et Grolle**, Rev. Bryol. Lichénol. 27 (3/4): 150, 1958 [1959] (Herzog and Grolle 1958).

*** Pachyglossa austrigena (Hook.f. et Taylor) L.Söderstr., Phytotaxa 112 (1): 24, 2013 (Söderström et al. 2013f). Bas.: Jungermannia austrigena Hook.f. et Taylor, London J. Bot. 3: 466, 1844 (Hooker and Taylor 1844b).

*** Pachyglossa austrigena subsp. okaritana (Steph.) L.Söderstr., Phytotaxa 112 (1): 24, 2013 (Söderström et al. 2013f). Bas.: Lophocolea okaritana Steph., Bull. Herb. Boissier (sér. 2) 6 (9): 785 (85), 1906 (Stephani 1906e).

*** Pachyglossa boveana (C.Massal.) L.Söderstr., Phytotaxa 112 (1): 24, 2013 (Söderström et al. 2013f). Bas.: Lophocolea boveana C.Massal., Nuovo Giorn. Bot. Ital. 17 (3): 225, 1885 (Massalongo 1885).

** Pachyglossa dissitifolia Herzog et Grolle, Rev. Bryol. Lichénol. 27 (3/4): 155, 1958 [1959] (Herzog and Grolle 1958).

* Pachyglossa exilis (Herzog et Grolle) Hässel et Solari, Transecta botánica de la Patagonia austral: 324, 1985 (Hässel and Solari 1985). Bas.: Pachyglossa spegazziniana var. exilis Herzog et Grolle, Rev. Bryol. Lichénol. 27 (3/4): 159, 1958 [1959] (Herzog and Grolle 1958). ^155^

** Pachyglossa fissa (Mitt.) Herzog et Grolle, Rev. Bryol. Lichénol. 28 (3/4): 346, 1959 [1960] (Grolle 1959c). Bas.: Herpocladium fissum Mitt., J. Linn. Soc., Bot. 15 (82): 69, 1876 (Mitten 1876a).

*** Pachyglossa gottscheoides (Besch. et C.Massal.) L.Söderstr., Phytotaxa 112 (1): 25, 2013 (Söderström et al. 2013f). Bas.: Lophocolea gottscheoides Besch. et C.Massal., Bull. Mens. Soc. Linn. Paris 1 (79): 631, 1886 (Bescherelle and Massalongo 1886).

** Pachyglossa grolleana Váňa, Cryptog. Bryol. 26 (1): 86, 2005 (Váňa and Gremmen 2005).

** Pachyglossa otiphylla (Hook.f. et Taylor) Váňa, Phytotaxa 112 (1): 25, 2013 (Söderström et al. 2013f). Bas.: Jungermannia otiphylla Hook.f. et Taylor, London J. Bot. 3: 466, 1844 (Hooker and Taylor 1844b).

** Pachyglossa spegazziniana (C.Massal.) Herzog et Grolle, Rev. Bryol. Lichénol. 27 (3/4): 159, 1958 [1959] (Herzog and Grolle 1958). Bas.: Lophocolea spegazziniana C.Massal., Nuovo Giorn. Bot. Ital. 17 (3): 225, 1885 (Massalongo 1885).

** Pachyglossa tenacifolia (Hook.f. et Taylor) Herzog et Grolle, Rev. Bryol. Lichénol. 27 (3/4): 153, 1958 [1959] (Herzog and Grolle 1958). Bas.: Jungermannia tenacifolia Hook.f. et Taylor, Bot. Antarct. Voy. I (Fl. Antarct. 1): 152, 1845 (Taylor and Hooker 1845).

** **Perdusenia Hässel**, Revista Mus. Argent. Ci. Nat., Bernardino Rivadavia Inst. Nac. Invest. Ci. Nat. Bot. 7 (2): 11, 1989 (Hässel 1989b).

** Perdusenia rheophila Hässel, Revista Mus. Argent. Ci. Nat., Bernardino Rivadavia Inst. Nac. Invest. Ci. Nat. Bot. 7 (2): 11, 1989 (Hässel 1989b).

** **Pigafettoa C.Massal.**, Nuovo Giorn. Bot. Ital. 17 (3): 237, 1885 (Massalongo 1885).

** Pigafettoa crenulata C.Massal., Nuovo Giorn. Bot. Ital. 17 (3): 237, 1885 (Massalongo 1885).

** **Platycaulis R.M.Schust.**, Phytologia 39 (4): 245, 1978 (Schuster 1978a).

*** Platycaulis renifolius R.M.Schust., Phytologia 39 (4): 245, 1978 (Schuster 1978a).

*** **Stolonivector J.J.Engel**, J. Hattori Bot. Lab. 69: 80, 1991 (Engel 1991a).

** Stolonivector clasmatocoleoides J.J.Engel, Nova Hedwigia 88 (3/4): 339, 2009 (Engel 2009).

*** Stolonivector fiordlandiae (E.A.Hodgs.) J.J.Engel, J. Hattori Bot. Lab. 69: 82, 1991 (Engel 1991a). Bas.: Lophocolea fiordlandiae E.A.Hodgs., Trans. Roy. Soc. New Zealand 80 (3/4): 340, 1952 [1953] (Hodgson 1952).

** Stolonivector fiordlandiae var. nodulosus J.J.Engel, Nova Hedwigia 93 (3/4): 403, 2011 (Engel 2011).

*** Stolonivector gremmenii (Váňa) Váňa, Phytotaxa 112 (1): 28, 2013 (Söderström et al. 2013f). Bas.: Chiloscyphus gremmenii Váňa, Cryptog. Bryol. 26 (1): 81, 2005 (Váňa and Gremmen 2005).

** Stolonivector obtusilobus J.J.Engel, Nova Hedwigia 88 (3/4): 337, 2009 (Engel 2009).

** Stolonivector waipouensis J.J.Engel, J. Hattori Bot. Lab. 93: 70, 2003 (Engel 2003).

** **Xenocephalozia R.M.Schust.**, Nova Hedwigia 10 (1/2): 25, 1965 (Schuster 1965b).

** Xenocephalozia navicularis (Steph.) R.M.Schust., Nova Hedwigia 10 (1/2): 25, 1965 (Schuster 1965b). Bas.: Lophocolea navicularis Steph., Bih. Kongl. Svenska Vetensk.-Akad. Handl. 26 (III, 6): 43, 1900 (Stephani 1900b).

######## *** Mastigophoraceae R.M.Schust.

** **Dendromastigophora R.M.Schust.**, Mem. New York Bot. Gard. 45: 738, 1987 (Schuster 1987a).

*** Dendromastigophora flagellifera (Hook.) R.M.Schust., Mem. New York Bot. Gard. 45: 738, 1987 (Schuster 1987a). Bas.: Jungermannia flagellifera Hook., Musci Exot. 1: tab. 59, 1818 (Hooker 1818).

*** **Mastigophora Nees**, Naturgesch. Eur. Leberm. 3: 89, 1838 (Nees 1838b) nom. conserv. ^156^

* Mastigophora appendiculata Steph., Sp. Hepat. (Stephani) 6: 368, 1922 (Stephani 1922).

* Mastigophora attenuata (Taylor) Trevis., Mem. Reale Ist. Lombardo Sci. (Ser. 3), C. Sci. Mat. 4 (13): 416, 1877 (Trevisan 1877). Bas.: Lepidozia attenuata Taylor, London J. Bot. 5: 369, 1846 (Taylor 1846b).

** Mastigophora caledonica Steph., Rev. Bryol. 35 (2): 31, 1908 (Stephani 1908l).

*** Mastigophora diclados (Brid. ex F.Weber) Nees, Naturgesch. Eur. Leberm. 3: 18, 1838 (Nees 1838b). Bas.: Jungermannia diclados Brid. ex F.Weber, Hist. Musc. Hepat. Prodr.: 56, 1815 (Weber 1815).

** Mastigophora diclados var. borneensis (De Not.) Schiffn., Nova Acta Acad. Caes. Leop.-Carol. German. Nat. Cur. 60 (2): 251, 1893 (Schiffner 1893a). Bas.: Sendtnera diclados var. borneensis De Not., Epat. Borneo: 42, 1874 (De Notaris 1874).

** Mastigophora diclados var. ramentifissa Herzog, Trans. Brit. Bryol. Soc. 1 (4): 315, 1950 (Herzog 1950a).

* Mastigophora diclados var. villosa Herzog, Ann. Bryol. 5: 82, 1932 (Herzog 1932b).

* Mastigophora guineensis Steph., Sp. Hepat. (Stephani) 6: 369, 1923 (Stephani 1923).

* Mastigophora humillima (Taylor) Trevis., Mem. Reale Ist. Lombardo Sci. (Ser. 3), C. Sci. Mat. 4 (13): 416, 1877 (Trevisan 1877). Bas.: Lepidozia humillima Taylor, London J. Bot. 5: 369, 1846 (Taylor 1846b).

* Mastigophora pyramidana Steph., Sp. Hepat. (Stephani) 6: 369, 1923 (Stephani 1923).

** Mastigophora sepikiana Piippo, Ann. Bot. Fenn. 23 (1): 2, 1986 (Piippo 1986b).

** Mastigophora tuberculata D.H.Mill. et H.A.Mill., J. Hattori Bot. Lab. 75: 181, 1994 (Miller and Miller 1994).

* Mastigophora valida Steph., Sp. Hepat. (Stephani) 6: 369, 1923 (Stephani 1923).

** Mastigophora viridula (Nees) Trevis., Mem. Reale Ist. Lombardo Sci. (Ser. 3), C. Sci. Mat. 4 (13): 416, 1877 (Trevisan 1877). Bas.: Jungermannia viridula Nees, Flora 6 (2): 30, 1823 (Link 1823).

*** Mastigophora woodsii (Hook.) Nees, Naturgesch. Eur. Leberm. 3: 95, 1838 (Nees 1838b). Bas.: Jungermannia woodsii Hook., Brit. Jungermann.: tab. 66, 1814 (Hooker 1814).

######## *** Plagiochilaceae Müll.Frib.

by L. Söderström

The placement of Pedinophyllopsis in Plagiochilaceae follows He-Nygrén and Piippo (2003). The inclusion of Pseudolophocolea in the family follows Söderström et al. (2013b). The subgeneric division of Plagiochila follows the review by Söderström et al. (2015b).

** **Acrochila R.M.Schust.**, J. Hattori Bot. Lab. 26: 285, 1963 (Schuster 1963b).

*** Acrochila biserialis (Lehm. et Lindenb.) Grolle, J. Jap. Bot. 39 (8): 236, 1964 (Grolle 1964g). Bas.: Plagiochila biserialis Lehm. et Lindenb., Sp. Hepat. (Lindenberg) 5: 126, 1843 (Lindenberg 1843).

** Acrochila caledonica (Steph.) Inoue, J. Jap. Bot. 42 (6): 182, 1967 (Inoue 1967d). Bas.: Plagiochila caledonica Steph., Rev. Bryol. 35 (2): 32, 1908 (Stephani 1908l).

** **Chiastocaulon Carl**, Flora 126: 58, 1931 (Carl 1931a).

*** Chiastocaulon dendroides (Nees) Carl, Flora 126: 59, 1931 (Carl 1931a). Bas.: Jungermannia dendroides Nees, Enum. Pl. Crypt. Javae: 77, 1830 (Nees 1830).

** **Dinckleria Trevis.**, Mem. Reale Ist. Lombardo Sci. (Ser. 3), C. Sci. Mat. 4 (13): 421, 1877 (Trevisan 1877).

*** Dinckleria fruticella (Hook.f. et Taylor) J.J.Engel et Heinrichs, Cryptog. Bryol. 29 (2): 194, 2008 (Engel and Heinrichs 2008). Bas.: Jungermannia fruticella Hook.f. et Taylor, London J. Bot. 3: 565, 1844 (Hooker and Taylor 1844d).

*** Dinckleria pleurata (Hook.f. et Taylor) Trevis., Mem. Reale Ist. Lombardo Sci. (Ser. 3), C. Sci. Mat. 4 (13): 421, 1877 (Trevisan 1877). Bas.: Jungermannia pleurata Hook.f. et Taylor, London J. Bot. 3: 372, 1844 (Hooker and Taylor 1844a).

** **Pedinophyllopsis R.M.Schust. et Inoue**, Phytologia 47 (4): 311, 1981 (Schuster and Engel 1981).

** Pedinophyllopsis abdita (Sull.) R.M.Schust. et Inoue, Phytologia 47 (4): 311, 1981 (Schuster and Engel 1981). Bas.: Plagiochila abdita Sull., Hooker’s J. Bot. Kew Gard. Misc. 2: 317, 1850 (Sullivant 1850).

** **Pedinophyllum Lindb. ex Nordst.**, Bot. Not. 1874: 156, 1874 (Nordstedt 1874).

*** Pedinophyllum autoicum (Steph.) Inoue, Bull. Natl. Sci. Mus. Tokyo (n.ser.) 9 (4): 575, 1966 (Inoue 1966b). Bas.: Plagiochila autoica Steph., Sp. Hepat. (Stephani) 6: 126, 1917 (Stephani 1917a).

*** Pedinophyllum interruptum (Nees) Kaal., Nyt Mag. Naturvidensk. 33 (1): 190, 1893 (Kaalaas 1893a). Bas.: Jungermannia interrupta Nees, Naturgesch. Eur. Leberm. 1: 165, 1833 (Nees 1833c).

*** Pedinophyllum monoicum (Steph.) Grolle, Nova Hedwigia 2: 287, 1960 (Grolle 1960b). Bas.: Plagiochila monoica Steph., Bull. Herb. Boissier (sér. 2) 3 (4): 331 (315), 1903 (Stephani 1903c).

** Pedinophyllum truncatum (Steph.) Inoue, J. Hattori Bot. Lab. 23: 35, 1960 [1961] (Inoue 1960). Bas.: Clasmatocolea truncata Steph., Bull. Herb. Boissier 5 (2): 87, 1897 (Stephani 1897b).

*** **Plagiochila (Dumort.) Dumort.**, Recueil Observ. Jungerm.: 14, 1835 (Dumortier 1835) nom. conserv. Bas.: Radula sect. Plagiochila Dumort., Syll. Jungerm. Europ.: 42, 1831 (Dumortier 1831).

*** Plagiochila heteromalla (Lehm. et Lindenb.) Lindenb., Sp. Hepat. (Lindenberg) 2-4: 83, 1840 (Lindenberg 1840). Bas.: Jungermannia heteromalla Lehm. et Lindenb., Nov. Stirp. Pug. 6: 62, 1834 (Lehmann 1834).

*** **sect. Adianthoideae Lindenb.**, Monogr. hep. gen. Plagiochilae: xx, 1844 (Lindenberg 1844).

*** Plagiochila adianthoides (Sw.) Lindenb., Sp. Hepat. (Lindenberg) 2-4: 77, 1840 (Lindenberg 1840). Bas.: Jungermannia adianthoides Sw., Prodr. (Swartz): 142, 1788 (Swartz 1788).

* Plagiochila adianthoides var. aspergillifera Spruce, Trans. & Proc. Bot. Soc. Edinburgh 15: 474, 1885 (Spruce 1885).

*** Plagiochila cristata (Sw.) Lindenb., Sp. Hepat. (Lindenberg) 1: 33, 1839 (Lindenberg 1839). Bas.: Jungermannia cristata Sw., Prodr. (Swartz): 143, 1788 (Swartz 1788).

*** Plagiochila grandicrista Steph., Bull. Herb. Boissier (sér. 2) 5 (10): 931 (581), 1905 (Stephani 1905f).

*** Plagiochila herminieri Steph., Bull. Herb. Boissier (sér. 2) 5 (8): 748 (547), 1905 (Stephani 1905i).

*** **sect. Africanae Heinrichs**, Taxon 54 (2): 319, 2005 (Heinrichs et al. 2005).

*** Plagiochila barteri Mitt., J. Linn. Soc., Bot. 22 (146): 320, 1886 (Mitten 1886b).

** Plagiochila barteri var. valida (Steph.) Vanden Berghen, Bull. Jard. Bot. Natl. Belg. 51 (1/2): 73, 1981 (Vanden Berghen 1981). Bas.: Plagiochila valida Steph., Bull. Herb. Boissier (sér. 2) 4 (6): 587 (438), 1904 (Stephani 1904b).

*** Plagiochila colorans Steph., Wiss. Ergebn. Deut. Zentr.-Afr. Exped. (1907-08), Bot. 2: 116, 1911 (Stephani 1911a).

*** **sect. Arrectae Carl**, Ann. Bryol., Suppl. 2: 52, 1931 (Carl 1931b).

* Plagiochila arnelliana Steph., Bull. Herb. Boissier (sér. 2) 2 (10): 861 (230), 1902 (Stephani 1902g). ^157^

*** Plagiochila badia Mitt., Rep. Challenger, Bot. 1 (3, 1): 84, 1884 (Mitten 1884b).

*** Plagiochila bidens Gottsche, Ann. Sci. Nat. Bot. (sér. 4) 8: 322, 1857 (Gottsche 1857).

*** Plagiochila bifaria (Sw.) Lindenb., Sp. Hepat. (Lindenberg) 5: 127, 1843 (Lindenberg 1843). Bas.: Jungermannia bifaria Sw., Prodr. (Swartz): 145, 1788 (Swartz 1788).

** Plagiochila bifaria var. rosea (R.M.Schust.) Heinrichs, Org. Divers. Evol. 4 (1/2): 112, 2004 (Heinrichs et al. 2004). Bas.: Rhodoplagiochila rosea R.M.Schust., Phytologia 39 (4): 247, 1978 (Schuster 1978a).

*** Plagiochila chacabucensis Steph., Kungl. Svenska Vetensk.-Akad. Handl. (n.ser.) 46 (9): 27, 1911 (Stephani 1911b).

*** Plagiochila emeiensis Grolle et M.L.So, Bryologist 101 (2): 282, 1998 (Grolle and So 1998a).

* Plagiochila fragilis Taylor, London J. Bot. 7: 198, 1848 (Taylor 1848a). ^158^

*** Plagiochila lunata S.W.Arnell, Bot. Not. 115: 204, 1962 (Arnell 1962a).

*** Plagiochila pachyloma Taylor, London J. Bot. 5: 267, 1846 (Taylor 1846a).

* Plagiochila pachyloma var. elatior Spruce, Trans. & Proc. Bot. Soc. Edinburgh 15: 480, 1885 (Spruce 1885).

** Plagiochila papillifolia Steph., Biblioth. Bot. 87 (2): 207, 1916 (Stephani 1916a).

*** Plagiochila parviramifera Inoue, J. Hattori Bot. Lab. 46: 317, 1979 (Mizutani 1979a).

*** Plagiochila pseudoattenuata S.W.Arnell, Bot. Not. 115: 206, 1962 (Arnell 1962a).

*** Plagiochila punctata (Taylor) Taylor, London J. Bot. 5: 261, 1846 (Taylor 1846a). Bas.: Jungermannia punctata Taylor, Trans. Bot. Soc. Edinburgh 1: 179, 1844 (Taylor 1844b).

*** Plagiochila renauldii Steph., Bull. Herb. Boissier (sér. 2) 4 (2): 156 (408), 1904 (Stephani 1904f).

*** Plagiochila retrorsa Gottsche, Mexik. Leverm.: 67, 1863 (Gottsche 1863).

*** Plagiochila rubescens (Lehm. et Lindenb.) Lindenb., Sp. Hepat. (Lindenberg) 2-4: 46, 1840 (Lindenberg 1840). Bas.: Jungermannia rubescens Lehm. et Lindenb., Nov. Stirp. Pug. 6: 63, 1834 (Lehmann 1834).

*** Plagiochila sichuanensis Grolle et M.L.So, Bryologist 101 (2): 284, 1998 (Grolle and So 1998a).

*** Plagiochila spinulosa (Dicks.) Dumort., Recueil Observ. Jungerm.: 15, 1835 (Dumortier 1835). Bas.: Jungermannia spinulosa Dicks., Fasc. Pl. Crypt. Brit. 2: 14, 1790 (Dickson 1790).

** Plagiochila sticticola Mont. et Gottsche, Ann. Sci. Nat. Bot. (sér. 4) 6: 198, 1856 (Montagne 1856a).

*** Plagiochila stricta Lindenb., Sp. Hepat. (Lindenberg) 1: 20, 1839 (Lindenberg 1839).

*** Plagiochila tronadoris Herzog, Darwiniana 11 (2): 214, 1957 (Herzog 1957b).

*** Plagiochila uncialis (Hook.f. et Taylor) Gottsche, Lindenb. et Nees, Syn. Hepat. 5: 628, 1847 (Gottsche et al. 1847). Bas.: Jungermannia uncialis Hook.f. et Taylor, London J. Bot. 3: 459, 1844 (Hooker and Taylor 1844b).

*** Plagiochila wilmsiana Steph., Sp. Hepat. (Stephani) 6: 240, 1921 (Stephani 1921).

* **sect. Caducifoliae J.J.Engel et G.L.Merr.**, Nova Hedwigia 96 (3/4): 407, 2013 (Engel and Smith Merrill 2013).

** Plagiochila caducifolia Inoue et R.M.Schust., J. Hattori Bot. Lab. 34: 71, 1971 (Inoue and Schuster 1971).

* **sect. Cardotiae Inoue**, Bull. Natl. Sci. Mus. Tokyo (n.ser.) 8 (3): 386, 1965 (Inoue 1965a).

*** Plagiochila cumingiana Steph., Bull. Herb. Boissier (sér. 2) 4 (1): 32 (404), 1904 (Stephani 1904g).

*** Plagiochila denticulata Mitt., J. Proc. Linn. Soc., Bot. 5 (18): 95, 1860 [1861] (Mitten 1860c).

*** Plagiochila fragillima Steph., Bull. Herb. Boissier (sér. 2) 3 (6): 522 (326), 1903 (Stephani 1903d).

*** Plagiochila pseudorenitens Schiffn., Österr. Bot. Z. 49 (4): 132, 1899 (Schiffner 1899b).

*** Plagiochila stevensiana Steph., Bull. Herb. Boissier (sér. 2) 3 (2): 110 (290), 1903 (Stephani 1903b).

* **sect. Cobanae Carl**, Ann. Bryol., Suppl. 2: 79, 1931 (Carl 1931b).

** Plagiochila cobana Steph., Sp. Hepat. (Stephani) 6: 138, 1918 (Stephani 1918).

** Plagiochila detecta M.L.So et Grolle, Nova Hedwigia 71 (3/4): 391, 2000 (So and Grolle 2000b).

*** Plagiochila singularis Schiffn., Hep. Fl. Buitenzorg: 158, 1900 (Schiffner 1900a).

*** Plagiochila tagawae Inoue, J. Hattori Bot. Lab. 38: 561, 1974 (Inoue 1974a).

** Plagiochila tixieri Inoue, Bull. Natl. Sci. Mus. Tokyo, B 1 (3): 87, 1975 (Inoue 1975a).

*** Plagiochila zhuensis Grolle et M.L.So, Bryologist 102 (2): 200, 1999 (Grolle and So 1999c).

*** **sect. Cucullatae Schiffn.**, Hep. Fl. Buitenzorg: 107, 1900 (Schiffner 1900a).

*** Plagiochila bantamensis (Reinw., Blume et Nees) Mont., Voy. Amér. Mérid., Bot. 7 (2): 82, 1839 (Montagne 1839a). Bas.: Jungermannia bantamensis Reinw., Blume et Nees, Nova Acta Phys.-Med. Acad. Caes. Leop.-Carol. Nat. Cur. 12 (1): 235, 1824 [1825] (Reinwardt et al. 1824a).

*** Plagiochila blepharophora (Nees) Lindenb., Sp. Hepat. (Lindenberg) 2-4: 102, 1840 (Lindenberg 1840). Bas.: Jungermannia blepharophora Nees, Enum. Pl. Crypt. Javae: 71, 1830 (Nees 1830).

** Plagiochila chauviniana Mont., Ann. Sci. Nat. Bot. (sér. 3) 11: 34, 1849 (Montagne 1849).

** Plagiochila clavatosaccata Steph., Bull. Herb. Boissier (sér. 2) 4 (1): 25 (397), 1904 (Stephani 1904g).

** Plagiochila grossispina Steph., Sp. Hepat. (Stephani) 6: 162, 1918 (Stephani 1918).

*** Plagiochila integerrima Steph., Bot. Jahrb. Syst. 8 (2): 83, 1886 (Stephani 1886d).

* Plagiochila integrilobula Schiffn., Hep. Fl. Buitenzorg: 170, 1900 (Schiffner 1900a). ^159^

** Plagiochila johannis-winkleri Herzog, Mitt. Inst. Allg. Bot. Hamburg 7 (3): 187, 1931 (Herzog 1931a).

* Plagiochila kuhliana Sande Lac., Ann. Mus. Bot. Lugduno-Batavi 1: 292, 1864 (Sande Lacoste 1864). ^160^

** Plagiochila kurzii Steph., Bull. Herb. Boissier (sér. 2) 3 (2): 112 (292), 1903 (Stephani 1903b).

** Plagiochila reischeckiana Steph., Bull. Herb. Boissier (sér. 2) 3 (4): 331 (315), 1903 (Stephani 1903c).

*** Plagiochila sandei Dozy ex Sande Lac., Plagiochila Sandei: 5, 1856 (Sande Lacoste 1856c).

*** Plagiochila sciophila Nees, Sp. Hepat. (Lindenberg) 2-4: 100, 1840 (Lindenberg 1840).

** Plagiochila sciophila subsp. ciliigera (R.M.Schust.) L.Söderstr., Phytotaxa 208 (1): 84, 2015 (Söderström et al. 2015b). Bas.: Plagiochila japonica subsp. ciliigera R.M.Schust., Amer. Midl. Naturalist 62 (2): 354, 1959 (Schuster 1959b).

* Plagiochila stephanii Schiffn., Hep. Fl. Buitenzorg: 166, 1900 (Schiffner 1900a). ^161^

** Plagiochila subplana Lindenb., Sp. Hepat. (Lindenberg) 2-4: 73, 1840 (Lindenberg 1840).

** Plagiochila vitiensis Mitt., Bonplandia 9 (24): 367, 1861 (Mitten 1861).

*** **sect. Denticulatae Schiffn.**, Hep. Fl. Buitenzorg: 106, 1900 (Schiffner 1900a).

*** Plagiochila alternans Lindenb. et Gottsche, Syn. Hepat. 5: 648, 1847 (Gottsche et al. 1847).

** Plagiochila ansata (Hook.f. et Taylor) Gottsche, Lindenb. et Nees, Syn. Hepat. 5: 649, 1847 (Gottsche et al. 1847). Bas.: Jungermannia ansata Hook.f. et Taylor, London J. Bot. 3: 457, 1844 (Hooker and Taylor 1844b).

*** Plagiochila banksiana Gottsche, Ann. Sci. Nat. Bot. (sér. 4) 8: 329, 1857 (Gottsche 1857).

** Plagiochila banksiana var. echinophora Inoue et R.M.Schust., J. Hattori Bot. Lab. 34: 62, 1971 (Inoue and Schuster 1971).

** Plagiochila chonotica Taylor, London J. Bot. 5: 260, 1846 (Taylor 1846a).

** Plagiochila equitans Gottsche, Ann. Sci. Nat. Bot. (sér. 4) 8: 331, 1857 (Gottsche 1857).

* Plagiochila fragmentissima Inoue et R.M.Schust., J. Hattori Bot. Lab. 34: 155, 1971 (Inoue and Schuster 1971). ^162^

*** Plagiochila gigantea Lindenb., Sp. Hepat. (Lindenberg) 2-4: 115, 1840 (Lindenberg 1840). *Nom. nov. pro Jungermannia gigantea* Hook., Musci Exot. 1: tab. 93, 1818 (Hooker 1818), *nom. illeg*.

** Plagiochila gigantea var. inermis J.J.Engel et G.L.Merr., Nova Hedwigia 91 (3/4): 513, 2010 (Engel and Smith Merrill 2010).

*** Plagiochila gregaria (Hook.f. et Taylor) Gottsche, Lindenb. et Nees, Syn. Hepat. 5: 654, 1847 (Gottsche et al. 1847). Bas.: Jungermannia gregaria Hook.f. et Taylor, London J. Bot. 3: 564, 1844 (Hooker and Taylor 1844d).

** Plagiochila hookeriana Lindenb., Sp. Hepat. (Lindenberg) 2-4: 81, 1840 (Lindenberg 1840).

** Plagiochila latifrons Gottsche et Hampe, Linnaea 27 (5): 553, 1854 (Hampe 1854).

** Plagiochila minutula (Hook.f. et Taylor) Gottsche, Lindenb. et Nees, Syn. Hepat. 5: 652, 1847 (Gottsche et al. 1847). Bas.: Jungermannia minutula Hook.f. et Taylor, London J. Bot. 3: 459, 1844 (Hooker and Taylor 1844b).

*** Plagiochila nobilis Gottsche, Bot. Zeitung (Berlin) Beil. 16: 37, 1858 (Gottsche 1858).

** Plagiochila obovata Steph., Kungl. Svenska Vetensk.-Akad. Handl. (n.ser.) 46 (9): 33, 1911 (Stephani 1911b).

*** Plagiochila ovata Lindenb. et Gottsche, Syn. Hepat. 5: 656, 1847 (Gottsche et al. 1847).

*** Plagiochila retrospectans Lindenb., Sp. Hepat. (Lindenberg) 5: 123, 1843 (Lindenberg 1843). *Nom. nov. pro Jungermannia retrospectans* Nees, Linnaea 6 (4): 619, 1831 (Nees 1831), *nom. illeg*.

** Plagiochila riparia Steph., Kungl. Svenska Vetensk.-Akad. Handl. (n.ser.) 46 (9): 34, 1911 (Stephani 1911b).

** Plagiochila rutlandii Steph., Bull. Herb. Boissier (sér. 2) 4 (8): 776 (454), 1904 (Stephani 1904c).

** Plagiochila subpectinata Besch. et C.Massal., Bull. Mens. Soc. Linn. Paris 1 (79): 628, 1886 (Bescherelle and Massalongo 1886).

*** **sect. Durae Carl**, Ann. Bryol., Suppl. 2: 123, 1931 (Carl 1931b).

** Plagiochila acanthocaulis Sull., Hooker’s J. Bot. Kew Gard. Misc. 2: 317, 1850 (Sullivant 1850).

** Plagiochila angulata Steph., Bih. Kongl. Svenska Vetensk.-Akad. Handl. 26 (III, 6): 26, 1900 (Stephani 1900b).

** Plagiochila bicornuta Steph., Bot. Jahrb. Syst. 23 (1/2, 3): 305, 1896 (Stephani 1896a).

** Plagiochila crozetensis Kaal., Nyt Mag. Naturvidensk. 49 (2/3): 92, 1911 (Kaalaas 1911).

*** Plagiochila deltoidea Lindenb., Sp. Hepat. (Lindenberg) 5: 132, 1843 (Lindenberg 1843).

** Plagiochila deltoidea var. densa J.J.Engel et G.L.Merr., Nova Hedwigia 91 (3/4): 506, 2010 (Engel and Smith Merrill 2010).

** Plagiochila dura De Not., Mem. Reale Accad. Sci. Torino (ser. 2) 16: 214, 1857 (De Notaris 1857).

** Plagiochila heterodonta (Hook.f. et Taylor) Gottsche, Lindenb. et Nees, Syn. Hepat. 5: 638, 1847 (Gottsche et al. 1847). Bas.: Jungermannia heterodonta Hook.f. et Taylor, London J. Bot. 3: 460, 1844 (Hooker and Taylor 1844b).

*** Plagiochila ramosissima (Hook.) Lindenb., Sp. Hepat. (Lindenberg) 2-4: 87, 1840 (Lindenberg 1840). Bas.: Jungermannia ramosissima Hook., Musci Exot. 1: tab. 92, 1818 (Hooker 1818).

*** **sect. Duseniae Carl**, Ann. Bryol., Suppl. 2: 126, 1931 (Carl 1931b).

** Plagiochila dusenii Steph., Bull. Herb. Boissier (sér. 2) 4 (10): 979 (475), 1904 (Stephani 1904a).

** Plagiochila elata Taylor, London J. Bot. 5: 259, 1846 (Taylor 1846a).

** Plagiochila lechleri Gottsche, Ann. Sci. Nat. Bot. (sér. 4) 8: 325, 1857 (Gottsche 1857).

** Plagiochila validissima Steph., Biblioth. Bot. 87 (2): 214, 1916 (Stephani 1916a).

* **sect. Flexicaules Carl**, Ann. Bryol., Suppl. 2: 127, 1931 (Carl 1931b).

** Plagiochila flexicaulis Mont., Syn. Hepat. 5: 629, 1847 (Gottsche et al. 1847).

*** **sect. Fruticosae Inoue**, Gen. Plagiochila SE Asia: 50, 1984 (Inoue 1984b).

*** Plagiochila assamica Steph., Sp. Hepat. (Stephani) 6: 125, 1917 (Stephani 1917a).

** Plagiochila benitoi Inoue ex Piippo, J. Hattori Bot. Lab. 72: 122, 1992 (Piippo and Tan 1992).

*** Plagiochila frondescens (Nees) Lindenb., Sp. Hepat. (Lindenberg) 2-4: 52, 1840 (Lindenberg 1840). Bas.: Jungermannia frondescens Nees, Linnaea 6 (4): 610, 1831 (Nees 1831).

*** Plagiochila fruticosa Mitt., J. Proc. Linn. Soc., Bot. 5 (18): 94, 1860 [1861] (Mitten 1860c).

** Plagiochila mauiensis Steph., Sp. Hepat. (Stephani) 6: 184, 1921 (Stephani 1921).

*** Plagiochila pulcherrima Horik., J. Sci. Hiroshima Univ., Ser. B, Div. 2, Bot. 1: 63, 1931 (Horikawa 1931a).

** Plagiochila tamiensis Steph., Sp. Hepat. (Stephani) 6: 235, 1921 (Stephani 1921).

*** **sect. Fuscoluteae Carl**, Ann. Bryol., Suppl. 2: 46, 1931 (Carl 1931b).

*** Plagiochila aerea Taylor, London J. Bot. 5: 263, 1846 (Taylor 1846a).

*** Plagiochila dependula Taylor, London J. Bot. 5: 265, 1846 (Taylor 1846a).

*** Plagiochila fuscolutea Taylor, London J. Bot. 5: 263, 1846 (Taylor 1846a).

** Plagiochila heterophylla Lindenb., Nov. Stirp. Pug. 10: 2, 1857 (Lehmann 1857).

** Plagiochila heterophylla var. beauverdii (Steph.) Heinrichs, Bryophyt. Biblioth. 58: 148, 2002 (Heinrichs 2002). Bas.: Plagiochila beauverdii Steph., Biblioth. Bot. 87 (2): 191, 1916 (Stephani 1916a).

*** Plagiochila longiramea Steph., Biblioth. Bot. 87 (2): 204, 1916 (Stephani 1916a).

*** Plagiochila paraphyllina Herzog, Hedwigia 74 (2): 89, 1934 (Herzog 1934a).

*** Plagiochila rudischusteri H.Rob., Beih. Nova Hedwigia 90: 199, 1988 (Robinson 1988).

*** Plagiochila subbidentata Taylor, Ann. Mag. Nat. Hist. 20 (135): 381, 1847 (Taylor 1847a).

*** Plagiochila tabinensis Steph., Bull. Herb. Boissier (sér. 2) 2 (10): 862 (231), 1902 (Stephani 1902g).

*** **sect. Glaucescentes Carl**, Ann. Bryol., Suppl. 2: 70, 1931 (Carl 1931b).

*** Plagiochila buchtiniana Steph., Sp. Hepat. (Stephani) 6: 135, 1918 (Stephani 1918).

*** Plagiochila diversifolia Lindenb. et Gottsche, Syn. Hepat. 5: 640, 1847 (Gottsche et al. 1847).

*** Plagiochila longispina Lindenb. et Gottsche, Syn. Hepat. 5: 642, 1847 (Gottsche et al. 1847).

*** **sect. Hylacoetes Carl**, Ann. Bryol., Suppl. 2: 50, 1931 (Carl 1931b).

*** Plagiochila amicta Steph., Bull. Herb. Boissier (sér. 2) 5 (9): 895 (561), 1905 (Stephani 1905g).

*** Plagiochila boryana Gottsche, Bull. Soc. Roy. Bot. Belgique 31: 118, 1892 (Stephani 1892c).

*** Plagiochila breuteliana Lindenb., Sp. Hepat. (Lindenberg) 5: 150, 1843 (Lindenberg 1843).

*** Plagiochila canelensis Steph., Bull. Herb. Boissier (sér. 2) 5 (10): 926 (576), 1905 (Stephani 1905f).

*** Plagiochila cucullifolia J.B.Jack et Steph., Hedwigia 31 (1): 24, 1892 (Jack and Stephani 1892).

*** Plagiochila cucullifolia var. anomala Heinrichs et Gradst., Pl. Syst. Evol. 242 (1/4): 208, 2003 (Heinrichs et al. 2003).

*** Plagiochila dimorpha Lindenb. et Gottsche, Syn. Hepat. 5: 627, 1847 (Gottsche et al. 1847).

*** Plagiochila dominicensis Taylor, London J. Bot. 5: 270, 1846 (Taylor 1846a).

*** Plagiochila ecuadorica (Inoue) L.Söderstr., Phytotaxa 208 (1): 84, 2015 (Söderström et al. 2015b). Bas.: Steereochila ecuadorica Inoue, Mem. New York Bot. Gard. 45: 279, 1987 (Inoue 1987a).

*** Plagiochila ensiformis Taylor, London J. Bot. 5: 265, 1846 (Taylor 1846a).

*** Plagiochila flabelliflora Steph., Bull. Herb. Boissier (sér. 2) 2 (10): 880 (249), 1902 (Stephani 1902g).

** Plagiochila guevarii H.Rob., Bryologist 70 (1): 48, 1967 (Robinson 1967).

** Plagiochila husnotii Steph., Bull. Herb. Boissier (sér. 2) 5 (2): 178 (506), 1905 (Stephani 1905d).

*** Plagiochila macrostachya Lindenb., Sp. Hepat. (Lindenberg) 2-4: 75, 1840 (Lindenberg 1840).

*** Plagiochila patriciae Heinrichs et H.Anton, Bryophyt. Biblioth. 58: 107, 2002 (Heinrichs 2002).

*** Plagiochila superba (Nees ex Spreng.) Mont. et Nees, Voy. Amér. Mérid., Bot. 7 (2): 81, 1839 (Montagne 1839a). Bas.: Jungermannia superba Nees ex Spreng. Syst. Veg. (ed. 16) [Sprengel] 4 (2): 326, 1827 (Sprengel 1827b).

*** Plagiochila superba var. macrotricha (Spruce) Heinrichs, Bryophyt. Biblioth. 58: 114, 2002 (Heinrichs 2002). Bas.: Plagiochila macrotricha Spruce, Trans. & Proc. Bot. Soc. Edinburgh 15: 476, 1885 (Spruce 1885).

*** Plagiochila turgida Herzog, Hedwigia 72 (6): 196, 1932 (Herzog 1932c).

*** Plagiochila vincentina Lindenb., Sp. Hepat. (Lindenberg) 2-4: 39, 1840 (Lindenberg 1840).

* **sect. Jacquinotiae Hässel**, Nova Hedwigia 89 (1/2): 85, 2009 (Hässel 2009).

** Plagiochila jacquinotii Mont., Voy. Pole Sud, Bot. 1: 273, 1845 (Montagne 1845c).

* **sect. Kaalaasiae Carl**, Ann. Bryol., Suppl. 2: 103, 1931 (Carl 1931b).

** Plagiochila amboynensis Taylor, London J. Bot. 5: 260, 1846 (Taylor 1846a).

* **sect. Longiflorae Carl**, Ann. Bryol., Suppl. 2: 130, 1931 (Carl 1931b).

** Plagiochila longiflora Mont., Syn. Hepat. 5: 651, 1847 (Gottsche et al. 1847).

* **sect. Oligodontes Carl**, Ann. Bryol., Suppl. 2: 130, 1931 (Carl 1931b).

** Plagiochila lophocoleoides Mont., Ann. Sci. Nat. Bot. (sér. 3) 4: 348, 1845 (Montagne 1845b).

*** **sect. Peculiares Schiffn.**, Hep. Fl. Buitenzorg: 107, 1900 (Schiffner 1900a).

** Plagiochila aspericaulis Grolle et M.L.So, Syst. Bot. 24 (3): 307, 1999 (Grolle and So 1999b).

** Plagiochila caulimammillosa Grolle et M.L.So, J. Bryol. 20 (1): 42, 1998 (Grolle and So 1998c).

*** Plagiochila devexa Steph., Bull. Herb. Boissier (sér. 2) 3 (4): 340 (324), 1903 (Stephani 1903c). *Nom. nov. pro Plagiochila deflexa* Mitt., J. Proc. Linn. Soc., Bot. 5 (18): 97, 1860 [1861] (Mitten 1860c), *nom. illeg*.

*** Plagiochila durelii Schiffn., Österr. Bot. Z. 49 (4): 131, 1899 (Schiffner 1899b).

** Plagiochila durelii subsp. guizhouensis Grolle et M.L.So, Syst. Bot. 24 (3): 304, 1999 (Grolle and So 1999b).

** Plagiochila grollei Inoue, Bull. Natl. Sci. Mus. Tokyo (n.ser.) 8 (3): 384, 1965 (Inoue 1965a).

** Plagiochila hoei Inoue, J. Hattori Bot. Lab. 40: 421, 1976 (Inoue 1976b).

** Plagiochila hyalodermica Grolle et M.L.So, Bryologist 100 (4): 470, 1997 (Grolle and So 1997b).

** Plagiochila magna Inoue, J. Hattori Bot. Lab. 28: 216, 1965 (Inoue 1965c).

** Plagiochila paraphyllosa Grolle et M.L.So, Syst. Bot. 24 (3): 298, 1999 (Grolle and So 1999b).

** Plagiochila peculiaris Schiffn., Hep. Fl. Buitenzorg: 157, 1900 (Schiffner 1900a).

*** Plagiochila perserrata Herzog, Symb. Sin. 5: 19, 1930 (Nicholson et al. 1930).

** Plagiochila philippinensis Steph., Bull. Herb. Boissier (sér. 2) 3 (6): 526 (330), 1903 (Stephani 1903d).

*** Plagiochila pseudopoeltii Inoue, Bull. Natl. Sci. Mus. Tokyo (n.ser.) 8 (3): 382, 1965 (Inoue 1965a).

*** Plagiochila renitens (Nees) Lindenb., Sp. Hepat. (Lindenberg) 2-4: 90, 1840 (Lindenberg 1840). Bas.: Jungermannia renitens Nees, Enum. Pl. Crypt. Javae: 76, 1830 (Nees 1830).

** Plagiochila semidecurrens (Lehm. et Lindenb.) Lindenb., Sp. Hepat. (Lindenberg) 5: 142, 1843 (Lindenberg 1843). Bas.: Jungermannia semidecurrens Lehm. et Lindenb., Nov. Stirp. Pug. 4: 21, 1832 (Lehmann 1832).

** Plagiochila semidecurrens var. alaskana (A.Evans) Inoue, J. Hattori Bot. Lab. 28: 216, 1965 (Inoue 1965c). Bas.: Plagiochila alaskana A.Evans, Bull. Torrey Bot. Club 41 (12): 590, 1914 [1915] (Evans 1914a).

*** Plagiochila vexans Schiffn. ex Steph., Sp. Hepat. (Stephani) 6: 237, 1921 (Stephani 1921).

*** Plagiochila zangii Grolle et M.L.So, Bryologist 100 (4): 467, 1997 (Grolle and So 1997b).

*** Plagiochila zonata Steph., Mém. Soc. Nat. Sci. Nat. Math. Cherbourg 29: 225, 1894 (Stephani 1894b).

*** **sect. Plagiochila**

** Plagiochila arctica Bryhn et Kaal., Rep. Second Norweg. Arctic Exped. 11: 41, 1906 (Bryhn 1906).

* Plagiochila arctica var. intermedia R.M.Schust., Amer. Midl. Naturalist 62 (1): 152, 1959 (Schuster 1959a).

*** Plagiochila asplenioides (L.) Dumort., Recueil Observ. Jungerm.: 14, 1835 (Dumortier 1835). Bas.: Jungermannia asplenioides L., Sp. Pl. 1: 1131, 1753 (Linnaeus 1753).

*** Plagiochila bischleriana Grolle et M.L.So, Cryptog. Bryol. Lichénol. 18 (3): 191, 1997 (Grolle and So 1997a).

** Plagiochila britannica Paton, J. Bryol. 10 (3): 245, 1979 (Paton 1979b).

*** Plagiochila chinensis Steph., Mém. Soc. Nat. Sci. Nat. Math. Cherbourg 29: 223, 1894 (Stephani 1894b).

*** Plagiochila circumdentata Steph., Bull. Herb. Boissier (sér. 2) 4 (8): 778 (456), 1904 (Stephani 1904c).

** Plagiochila circumdentata var. carinata J.J.Engel et G.L.Merr., Nova Hedwigia 91 (3/4): 511, 2010 (Engel and Smith Merrill 2010).

** Plagiochila circumserrata Inoue et Grolle, Bull. Natl. Sci. Mus. Tokyo, B 5 (1): 27, 1979 (Inoue 1979c).

** Plagiochila columbiana A.Evans, Bot. Gaz. 21 (4): 189, 1896 (Evans 1896).

*** Plagiochila delavayi Steph., Mém. Soc. Nat. Sci. Nat. Math. Cherbourg 29: 224, 1894 (Stephani 1894b).

*** Plagiochila elegans Mitt., J. Proc. Linn. Soc., Bot. 5 (18): 97, 1860 [1861] (Mitten 1860c).

*** Plagiochila hakkodensis Steph., Bull. Herb. Boissier 5 (2): 103, 1897 (Stephani 1897b).

** Plagiochila korthalsiana Molk., Ned. Kruidk. Arch. 3: 416, 1854 [1855] (Sande Lacoste 1854).

** Plagiochila loriana Steph., Bull. Herb. Boissier (sér. 2) 3 (7): 608 (354), 1903 (Stephani 1903e).

** Plagiochila microdentata M.L.So, New Zealand J. Bot. 38 (3): 426, 2000 (So 2000b).

** Plagiochila mundaliensis Steph., Bull. Herb. Boissier (sér. 2) 3 (6): 536 (340), 1903 (Stephani 1903d).

** Plagiochila orbicularis (S.Hatt.) S.Hatt., J. Hattori Bot. Lab. 3: 26, 1948 [1950] (Hattori 1948b). Bas.: Plagiochila ovalifolia var. orbicularis S.Hatt., Bull. Tokyo Sci. Mus. 11: 61, 1944 (Hattori 1944d).

*** Plagiochila ovalifolia Mitt., Trans. Linn. Soc. London, Bot. 3 (3): 193, 1891 (Mitten 1891).

*** Plagiochila porelloides (Torr. ex Nees) Lindenb., Sp. Hepat. (Lindenberg) 2-4: 61, 1840 (Lindenberg 1840). Bas.: Jungermannia porelloides Torr. ex Nees, Naturgesch. Eur. Leberm. 1: 170, 1833 (Nees 1833c).

** Plagiochila porelloides var. norvegica (H.H.Blom et Holten) Schumacker et Váňa, Identif. keys liverw. hornw. Europe: 131, 2005 (Schumacker and Váňa 2005). Bas.: Plagiochila norvegica H.H.Blom et Holten, Lindbergia 14 (1): 8, 1988 (Blom and Holten 1988).

** Plagiochila porelloides var. subarctica (Jørg.) Lammes, Fl. Fenn. 6: 54, 1977 (Koponen et al. 1977). Bas.: Plagiochila asplenioides var. subarctica Jørg., Bergens Mus. Skr. (n.ser.) 16: 173, 1934 (Jørgensen 1934).

** Plagiochila schofieldiana Inoue, Bull. Natl. Sci. Mus. Tokyo (n.ser.) 15 (1): 183, 1972 (Inoue 1972c).

*** Plagiochila secretifolia Mitt., J. Proc. Linn. Soc., Bot. 5 (18): 98, 1860 [1861] (Mitten 1860c).

** Plagiochila spinosa M.L.So, New Zealand J. Bot. 38 (3): 428, 2000 (So 2000b).

** Plagiochila sumatrana Schiffn., Denkschr. Kaiserl. Akad. Wiss., Math.-Naturwiss. Kl. 70: 183, 1900 [1901] (Schiffner 1900c).

** Plagiochila taiwanensis Inoue, Bull. Natl. Sci. Mus. Tokyo, B 8 (4): 136, 1982 (Inoue 1982).

*** Plagiochila trapezoidea Lindenb., Sp. Hepat. (Lindenberg) 2-4: 112, 1840 (Lindenberg 1840). *Nom. nov. pro* Jungermannia asplenioides β australis Nees, Enum. Pl. Crypt. Javae: 73, 1830 (Nees 1830).

** Plagiochila uniformis Mitt., J. Proc. Linn. Soc., Bot. 5 (18): 98, 1860 [1861] (Mitten 1860c).

** **sect. Poeltiae Inoue**, Bull. Natl. Sci. Mus. Tokyo (n.ser.) 8 (3): 394, 1965 (Inoue 1965a).

*** Plagiochila biondiana C.Massal., Hepat. Shen-si: 15, 1897 (Massalongo 1897).

*** Plagiochila carringtonii (Balf. ex Carrington) Grolle, Trans. Brit. Bryol. Soc. 4 (4): 656, 1964 (Grolle 1964i). Bas.: Adelanthus carringtonii Balf. ex Carrington, Trans. Bot. Soc. Edinburgh 10: 380, 1870 (Carrington 1870).

** Plagiochila carringtonii subsp. lobuchensis Grolle, Trans. Brit. Bryol. Soc. 4 (4): 660, 1964 (Grolle 1964i).

*** Plagiochila duthiana Steph., Bull. Herb. Boissier (sér. 2) 3 (6): 527 (331), 1903 (Stephani 1903d).

** Plagiochila erlangensis M.L.So, Haussknechtia, Beih. 9: 350, 1999 (So 1999).

*** Plagiochila poeltii Inoue et Grolle, Trans. Brit. Bryol. Soc. 4 (4): 656, 1964 (Grolle 1964i).

*** Plagiochila recurvata (W.E.Nicholson) Grolle, Trans. Brit. Bryol. Soc. 4 (4): 654, 1964 (Grolle 1964i). Bas.: Jamesoniella carringtonii var. recurvata W.E.Nicholson, Symb. Sin. 5: 13, 1930 (Nicholson et al. 1930).

*** Plagiochila retusa Mitt., J. Proc. Linn. Soc., Bot. 5 (18): 96, 1860 [1861] (Mitten 1860c).

*** Plagiochila wallichiana Steph., Bull. Herb. Boissier (sér. 2) 3 (6): 523 (327), 1903 (Stephani 1903d).

** Plagiochila wangii Inoue, J. Jap. Bot. 37 (6): 187, 1962 (Inoue 1962c).

** Plagiochila yulungensis Piippo, Ann. Bot. Fenn. 34 (4): 283, 1997 (Piippo 1997).

*** **sect. Rutilantes Carl**, Ann. Bryol., Suppl. 2: 83, 1931 (Carl 1931b).

*** Plagiochila bicuspidata Gottsche, Mexik. Leverm.: 43, 1863 (Gottsche 1863).

** Plagiochila caduciloba H.L.Blomq., Bryologist 42 (5): 114, 1939 (Blomquist 1939).

** Plagiochila chimborazensis Spruce, Trans. & Proc. Bot. Soc. Edinburgh 15: 469, 1885 (Spruce 1885).

* Plagiochila cuneata Lindenb. et Gottsche, Syn. Hepat. 5: 632, 1847 (Gottsche et al. 1847). ^163^

*** Plagiochila debilis Mitt., J. Proc. Linn. Soc., Bot. 5 (18): 97, 1860 [1861] (Mitten 1860c).

*** Plagiochila deflexa Mont. et Gottsche, Ann. Sci. Nat. Bot. (sér. 4) 6: 192, 1856 (Montagne 1856a).

*** Plagiochila defolians Grolle et M.L.So, Syst. Bot. 23 (4): 459, 1998 [1999] (Grolle and So 1998b).

*** Plagiochila exigua (Taylor) Taylor, London J. Bot. 5: 264, 1846 (Taylor 1846a). Bas.: Jungermannia exigua Taylor, Trans. Bot. Soc. Edinburgh 1: 179, 1844 (Taylor 1844b).

** Plagiochila fracta Pócs, Phytotaxa 195 (2): 183, 2015 (Pócs 2015a).

*** Plagiochila ghatiensis Steph., Sp. Hepat. (Stephani) 6: 159, 1918 (Stephani 1918).

*** Plagiochila grossa Grolle et M.L.So, Syst. Bot. 23 (4): 461, 1998 [1999] (Grolle and So 1998b).

*** Plagiochila gymnocalycina (Lehm. et Lindenb.) Mont. et Nees, Voy. Amér. Mérid., Bot. 7 (2): 81, 1839 (Montagne 1839a). Bas.: Jungermannia gymnocalycina Lehm. et Lindenb., Nov. Stirp. Pug. 5: 28, 1833 (Lehmann 1833).

** Plagiochila gymnocalycina var. surinamensis (Molk.) Heinrichs et D.S.Rycroft, J. Hattori Bot. Lab. 100: 139, 2006 (Heinrichs et al. 2006). Bas.: Plagiochila surinamensis Molk., Syn. hepat. jav.: 103, 1856 [1857] (Sande Lacoste 1856b).

** Plagiochila loriloba Herzog ex Carl, Ann. Bryol., Suppl. 2: 47, 1931 (Carl 1931b).

** Plagiochila maderensis Gottsche ex Steph., Bull. Herb. Boissier (sér. 2) 4 (4): 350 (426), 1904 (Stephani 1904e).

** Plagiochila oresitropha Spruce, Trans. & Proc. Bot. Soc. Edinburgh 15: 467, 1885 (Spruce 1885).

*** Plagiochila pectinata Lindenb., Sp. Hepat. (Lindenberg) 1: 14, 1839 (Lindenberg 1839).

*** Plagiochila rutilans Lindenb., Sp. Hepat. (Lindenberg) 2-4: 47, 1840 (Lindenberg 1840).

*** Plagiochila rutilans var. moritziana (Lindenb. et Gottsche) Heinrichs, Bryologist 105 (2): 197, 2002 (Heinrichs et al. 2002). Bas.: Plagiochila moritziana Lindenb. et Gottsche, Linnaea 20 (3): 323, 1847 (Hampe 1847).

*** Plagiochila rutilans var. standleyi (Herzog ex Carl) Heinrichs et D.S.Rycroft, Bryologist 104 (3): 357, 2001 (Heinrichs et al. 2001). Bas.: Plagiochila standleyi Herzog ex Carl, Ann. Bryol., Suppl. 2: 80, 1931 (Carl 1931b).

* Plagiochila steyermarkii H.Rob., Bryologist 68 (1): 93, 1965 (Robinson 1965). ^164^

*** Plagiochila trichostoma Gottsche, Ann. Sci. Nat. Bot. (sér. 5) 1: 113, 1864 (Gottsche 1864).

* **sect. Strombifoliae Inoue et R.M.Schust.**, J. Hattori Bot. Lab. 34: 130, 1971 (Inoue and Schuster 1971).

** Plagiochila strombifolia Taylor, Nov. Stirp. Pug. 8: 5, 1844 (Lehmann 1844).

*** **sect. Tayloriae Carl**, Ann. Bryol., Suppl. 2: 140, 1931 (Carl 1931b).

*** Plagiochila annotina Lindenb., Sp. Hepat. (Lindenberg) 1: 34, 1839 (Lindenberg 1839).

*** Plagiochila baylisii Inoue et R.M.Schust., J. Hattori Bot. Lab. 34: 150, 1971 (Inoue and Schuster 1971).

*** Plagiochila bazzanioides J.J.Engel et G.L.Merr., Novon 9 (1): 29, 1999 (Engel and Smith Merrill 1999b).

*** Plagiochila chenii Grolle et M.L.So, Syst. Bot. 25 (1): 6, 2000 (Grolle and So 2000).

*** Plagiochila circinalis (Lehm. et Lindenb.) Lehm., Sp. Hepat. (Lindenberg) 5: 124, 1843 (Lindenberg 1843). Bas.: Jungermannia circinalis Lehm. et Lindenb., Nov. Stirp. Pug. 4: 64, 1832 (Lehmann 1832).

** Plagiochila circinalis var. hemicardia (Hook.f. et Taylor) J.J.Engel et G.L.Merr., Nova Hedwigia 91 (3/4): 512, 2010 (Engel and Smith Merrill 2010). Bas.: Jungermannia hemicardia Hook.f. et Taylor, London J. Bot. 3: 371, 1844 (Hooker and Taylor 1844a).

*** Plagiochila colensoi Hook.f. et Taylor, London J. Bot. 5: 269, 1846 (Taylor 1846a).

** Plagiochila colensoi var. quinquespina (Steph.) J.J.Engel et G.L.Merr., Nova Hedwigia 91 (3/4): 504, 2010 (Engel and Smith Merrill 2010). Bas.: Plagiochila quinquespina Steph., Bull. Herb. Boissier (sér. 2) 3 (4): 328 (312), 1903 (Stephani 1903c).

** Plagiochila corticola Steph., Mém. Soc. Nat. Sci. Nat. Math. Cherbourg 29: 224, 1894 (Stephani 1894b).

** Plagiochila fasciculata Lindenb., Sp. Hepat. (Lindenberg) 1: 7, 1839 (Lindenberg 1839).

*** Plagiochila fusca Sande Lac., Ned. Kruidk. Arch. 3: 417, 1854 [1855] (Sande Lacoste 1854).

** Plagiochila fuscella (Hook.f. et Taylor) Gottsche, Lindenb. et Nees, Syn. Hepat. 5: 648, 1847 (Gottsche et al. 1847). Bas.: Jungermannia fuscella Hook.f. et Taylor, London J. Bot. 3: 373, 1844 (Hooker and Taylor 1844a).

** Plagiochila fuscella var. novae-zelandiae (E.A.Hodgs.) J.J.Engel et G.L.Merr., Nova Hedwigia 89 (3/4): 294, 2009 (Engel and Smith Merrill 2009). Bas.: Plagiochila retrospectans var. novae-zelandiae E.A.Hodgs., Trans. & Proc. Roy. Soc. New Zealand 73 (4): 293, 1944 (Hodgson 1944).

*** Plagiochila gracilis Lindenb. et Gottsche, Syn. Hepat. 5: 632, 1847 (Gottsche et al. 1847).

*** Plagiochila gymnoclada Sande Lac., Plagiochila Sandei: 6, 1856 (Sande Lacoste 1856c).

*** Plagiochila himalayana Schiffn., Österr. Bot. Z. 49 (4): 131, 1899 (Schiffner 1899b).

** Plagiochila incurvicolla (Hook.f. et Taylor) Gottsche, Lindenb. et Nees, Syn. Hepat. 5: 651, 1847 (Gottsche et al. 1847). Bas.: Jungermannia incurvicolla Hook.f. et Taylor, London J. Bot. 3: 564, 1844 (Hooker and Taylor 1844d).

** Plagiochila incurvicolla var. lonchoscypha (Herzog) J.J.Engel et G.L.Merr., Nova Hedwigia 91 (3/4): 505, 2010 (Engel and Smith Merrill 2010). Bas.: Plagiochila lonchoscypha Herzog, Trans. & Proc. Roy. Soc. New Zealand 68 (1): 42, 1938 (Herzog 1938c).

** Plagiochila microdictyon Mitt., Bot. antarct. voy. II (Fl. Nov.-Zel. 2): 131, 1854 (Mitten 1854).

** Plagiochila monospiris Inoue et Grolle, J. Hattori Bot. Lab. 36: 489, 1972 [1973] (Inoue 1972b).

*** Plagiochila nitens Inoue, Willdenowia 18 (2): 561, 1989 (Inoue 1989b).

** Plagiochila pseudocapillaris Inoue, Bull. Natl. Sci. Mus. Tokyo (n.ser.) 11 (3): 302, 1968 (Inoue 1968a).

*** Plagiochila pseudofirma Herzog, Symb. Sin. 5: 17, 1930 (Nicholson et al. 1930).

** Plagiochila radiculosa Mitt., Bot. antarct. voy. II (Fl. Nov.-Zel. 2): 132, 1854 (Mitten 1854).

** Plagiochila spathulifolia Mitt., J. Proc. Linn. Soc., Bot. 5 (18): 96, 1860 [1861] (Mitten 1860c).

*** Plagiochila stephensoniana Mitt., Bot. antarct. voy. II (Fl. Nov.-Zel. 2): 133, 1854 (Mitten 1854).

*** **sect. Trabeculatae S.Hatt. ex Inoue**, J. Hattori Bot. Lab. 20: 75, 1958 (Inoue 1958).

** Plagiochila austinii A.Evans, Rhodora 16 (184): 68, 1914 (Evans 1914b).

*** Plagiochila flexuosa Mitt., J. Proc. Linn. Soc., Bot. 5 (18): 94, 1860 [1861] (Mitten 1860c).

** Plagiochila sullivantii Gottsche, Bot. Gaz. 21 (4): 191, 1896 (Evans 1896).

** Plagiochila sullivantii var. spinigera R.M.Schust., Amer. Midl. Naturalist 62 (2): 323, 1959 (Schuster 1959b).

*** Plagiochila trabeculata Steph., Bull. Herb. Boissier (sér. 2) 3 (2): 103 (283), 1903 (Stephani 1903b).

*** **sect. Vagae Lindenb.**, Monogr. hep. gen. Plagiochilae: xv, 1844 (Lindenberg 1844).

*** Plagiochila abietina (Nees) Mont. et Nees, Voy. Amér. Mérid., Bot. 7 (2): 81, 1839 (Montagne 1839a). Bas.: Jungermannia abietina Nees, Enum. Pl. Crypt. Javae: 76, 1830 (Nees 1830).

** Plagiochila abrupta Lehm. et Lindenb., Sp. Hepat. (Lindenberg) 2-4: 106, 1840 (Lindenberg 1840).

** Plagiochila aequatorialis Gottsche, Ann. Sci. Nat. Bot. (sér. 4) 8: 334, 1857 (Gottsche 1857).

*** Plagiochila africana Steph., Bull. Herb. Boissier (sér. 2) 2 (12): 973 (263), 1902 (Stephani 1902h).

*** Plagiochila akiyamae Inoue, Bull. Natl. Sci. Mus. Tokyo, B 12 (3): 73, 1986 (Inoue 1986).

*** Plagiochila angusta Lindenb., Sp. Hepat. (Lindenberg) 5: 148, 1843 (Lindenberg 1843).

*** Plagiochila angustitexta Steph., Bull. Herb. Boissier (sér. 2) 2 (12): 977 (267), 1902 (Stephani 1902h).

*** Plagiochila arbuscula (Brid. ex Lehm. et Lindenb.) Lindenb., Sp. Hepat. (Lindenberg) 1: 23, 1839 (Lindenberg 1839). Bas.: Jungermannia arbuscula Brid. ex Lehm. et Lindenb., Nov. Stirp. Pug. 4: 63, 1832 (Lehmann 1832).

** Plagiochila arbuscula var. rekohuensis J.J.Engel et G.L.Merr., Nova Hedwigia 91 (3/4): 509, 2010 (Engel and Smith Merrill 2010).

* Plagiochila aspera Steph., Sp. Hepat. (Stephani) 6: 125, 1917 (Stephani 1917a). ^165^

** Plagiochila aspleniformis R.M.Schust., Amer. Midl. Naturalist 63 (1): 51, 1960 (Schuster 1960c).

** Plagiochila beddomei Steph., Bull. Herb. Boissier (sér. 2) 3 (10): 876 (361), 1903 (Stephani 1903f).

*** Plagiochila boivinii Steph., Bull. Herb. Boissier (sér. 2) 2 (12): 987 (277), 1902 (Stephani 1902h).

*** Plagiochila bryopteroides Spruce, Trans. & Proc. Bot. Soc. Edinburgh 15: 499, 1885 (Spruce 1885).

** Plagiochila contigua Gottsche, Mexik. Leverm.: 30, 1863 (Gottsche 1863).

*** Plagiochila corrugata (Nees) Nees et Mont., Ann. Sci. Nat. Bot. (sér. 2) 5: 52, 1836 (Nees and Montagne 1836). Bas.: Jungermannia corrugata Nees, Fl. Bras. (Martius) 1 (1): 378, 1833 (Nees 1833a).

*** Plagiochila cuspidata Steph., Sp. Hepat. (Stephani) 6: 144, 1918 (Stephani 1918).

** Plagiochila cymata Inoue et Grolle, Bull. Natl. Sci. Mus. Tokyo, B 1 (3): 95, 1975 (Inoue 1975a).

** Plagiochila deflexirama Taylor, London J. Bot. 5: 262, 1846 (Taylor 1846a).

*** Plagiochila dichotoma (P.Beauv.) Nees et Mont., Ann. Sci. Nat. Bot. (sér. 2) 5: 53, 1836 (Nees and Montagne 1836). Bas.: Carpolepidum dichotomum P.Beauv., Fl. Oware 1 (3): 23, 1805 (Palisot de Beauvois 1805b).

* Plagiochila dichotoma var. fluitans Spruce, Trans. & Proc. Bot. Soc. Edinburgh 15: 490, 1885 (Spruce 1885).

*** Plagiochila dissecta Steph., Bull. Herb. Boissier (sér. 2) 3 (7): 600 (346), 1903 (Stephani 1903e).

*** Plagiochila disticha (Lehm. et Lindenb.) Lindenb., Sp. Hepat. (Lindenberg) 2-4: 107, 1840 (Lindenberg 1840). Bas.: Jungermannia disticha Lehm. et Lindenb., Nov. Stirp. Pug. 6: 64, 1834 (Lehmann 1834).

** Plagiochila distinctifolia Lindenb., Sp. Hepat. (Lindenberg) 1: 17, 1839 (Lindenberg 1839).

*** Plagiochila divergens Steph., Hedwigia 30 (6): 268, 1891 (Stephani 1891c).

*** Plagiochila drepanophylla Sande Lac., Syn. hepat. jav.: 103, 1856 [1857] (Sande Lacoste 1856b).

* Plagiochila drepanophylla var. minor Gottsche, Abh. Naturwiss. Vereins Bremen 7: 341, 1882 (Gottsche 1882).

*** Plagiochila effusa Steph., Bot. Jahrb. Syst. 20 (3): 310, 1895 (Stephani 1895a).

* Plagiochila effusa var. decurrens Steph., Bot. Jahrb. Syst. 20 (3): 310, 1895 (Stephani 1895a).

*** Plagiochila ericicola Steph., Bull. Herb. Boissier (sér. 2) 4 (6): 590 (441), 1904 (Stephani 1904b).

** Plagiochila exinnovata Steph., Bull. Herb. Boissier (sér. 2) 3 (7): 600 (346), 1903 (Stephani 1903e).

** Plagiochila fastigiata Lindenb. et Gottsche, Syn. Hepat. 5: 657, 1847 (Gottsche et al. 1847).

*** Plagiochila flabellata Steph., Bot. Jahrb. Syst. 8 (2): 82, 1886 (Stephani 1886d).

** Plagiochila floridana A.Evans, Bot. Gaz. 21 (4): 190, 1896 (Evans 1896).

*** Plagiochila fordiana Steph., Bull. Herb. Boissier (sér. 2) 3 (2): 104 (284), 1903 (Stephani 1903b).

** Plagiochila francana Steph., Sp. Hepat. (Stephani) 6: 157, 1918 (Stephani 1918).

*** Plagiochila furcifolia Mitt., Trans. Linn. Soc. London, Bot. 3 (3): 194, 1891 (Mitten 1891).

*** Plagiochila fusifera Taylor, London J. Bot. 5: 268, 1846 (Taylor 1846a).

*** Plagiochila hampeana Gottsche, Bot. Zeitung (Berlin) Beil. 16: 38, 1858 (Gottsche 1858).

* Plagiochila heterospina Steph., J. & Proc. Roy. Soc. New South Wales 48 (1/2): 128, 1914 (Stephani and Watts 1914).

* Plagiochila heterostipa Steph., Hedwigia 31 (3): 129, 1892 (Stephani 1892d). ^166^

** Plagiochila incerta Gottsche, Ann. Sci. Nat. Bot. (sér. 4) 8: 324, 1857 (Gottsche 1857).

*** Plagiochila indica Mitt. ex Steph., Bull. Herb. Boissier (sér. 2) 3 (6): 532 (336), 1903 (Stephani 1903d).

** Plagiochila invisa (R.M.Schust.) R.M.Schust., Hepat. Anthocerotae N. Amer. 4: 513, 1980 (Schuster 1980c). Bas.: Plagiochila ludoviciana var. invisa R.M.Schust., Amer. Midl. Naturalist 63 (1): 101, 1960 (Schuster 1960c).

*** Plagiochila javanica (Sw.) Nees et Mont., Ann. Sci. Nat. Bot. (sér. 2) 5: 52, 1836 (Nees and Montagne 1836). Bas.: Jungermannia javanica Sw., Meth. Musc.: 35, 1781 (Swartz 1781).

*** Plagiochila junghuhniana Sande Lac., Ned. Kruidk. Arch. 3: 416, 1854 [1855] (Sande Lacoste 1854).

*** Plagiochila khasiana Mitt., J. Proc. Linn. Soc., Bot. 5 (18): 95, 1860 [1861] (Mitten 1860c).

** Plagiochila kiaeri Gottsche, Abh. Naturwiss. Vereins Bremen 7: 341, 1882 (Gottsche 1882).

** Plagiochila kiaeri var. capensis (Steph.) M.Wigginton et Grolle, Bryophyt. Biblioth. 50: 182, 1996 (Wigginton and Grolle 1996). Bas.: Plagiochila capensis Steph., Bull. Herb. Boissier (sér. 2) 4 (4): 350 (426), 1904 (Stephani 1904e).

** Plagiochila kiaeri var. myriocarpa (Pearson) Pócs, J. E. Afr. Nat. Hist. 96 (1): 38, 2007 (Pócs and Luke 2007). Bas.: Plagiochila myriocarpa Pearson, Ark. Bot. 19 (5): 5, 1924 (Pearson 1924b).

*** Plagiochila kunmingensis Piippo, Ann. Bot. Fenn. 34 (4): 281, 1997 (Piippo 1997).

** Plagiochila kurokawae Inoue, J. Hattori Bot. Lab. 32: 104, 1969 (Inoue 1969).

*** Plagiochila laetevirens Lindenb., Sp. Hepat. (Lindenberg) 2-4: 101, 1840 (Lindenberg 1840).

** Plagiochila lamellistipula Spruce, Trans. & Proc. Bot. Soc. Edinburgh 15: 491, 1885 (Spruce 1885).

*** Plagiochila lastii Mitt., J. Linn. Soc., Bot. 22 (146): 320, 1886 (Mitten 1886b).

** Plagiochila latifolia Steph., Bull. Herb. Boissier (sér. 2) 5 (8): 742 (541), 1905 (Stephani 1905i).

* Plagiochila loloënsis Steph., Bull. Herb. Boissier (sér. 2) 4 (2): 166 (418), 1904 (Stephani 1904f). ^167^

** Plagiochila manillana Mont. et Gottsche, Ann. Sci. Nat. Bot. (sér. 4) 6: 189, 1856 (Montagne 1856a).

** Plagiochila massalongoana Schiffn., Hep. Fl. Buitenzorg: 136, 1900 (Schiffner 1900a).

** Plagiochila mastigophoroides Inoue, J. Hattori Bot. Lab. 32: 99, 1969 (Inoue 1969).

** Plagiochila metcalfii Steph., Bull. Herb. Boissier (sér. 2) 3 (6): 533 (337), 1903 (Stephani 1903d).

** Plagiochila micropteryx Gottsche, Ann. Sci. Nat. Bot. (sér. 5) 1: 107, 1864 (Gottsche 1864).

** Plagiochila miradorensis Gottsche, Mexik. Leverm.: 31, 1863 (Gottsche 1863).

** Plagiochila miradorensis var. convoluta R.M.Schust., Amer. Midl. Naturalist 63 (1): 113, 1960 (Schuster 1960c).

* Plagiochila moenkemeyeri Steph., Bull. Herb. Boissier (sér. 2) 4 (2): 160 (412), 1904 (Stephani 1904f). ^168^

*** Plagiochila montagnei Nees, Ann. Sci. Nat. Bot. (sér. 2) 5: 53, 1836 (Nees and Montagne 1836).

** Plagiochila morobensis Inoue et Piippo, Ann. Bot. Fenn. 26 (2): 203, 1989 (Piippo 1989b).

** Plagiochila multipinnula Herzog et S.Hatt., J. Hattori Bot. Lab. 14: 36, 1955 (Herzog and Noguchi 1955).

*** Plagiochila neckeroidea Mitt., Trans. Linn. Soc. London 23 (1): 57, 1860 (Mitten 1860a).

*** Plagiochila nepalensis Lindenb., Sp. Hepat. (Lindenberg) 2-4: 93, 1840 (Lindenberg 1840).

** Plagiochila norfolkiensis Steph., Bull. Herb. Boissier (sér. 2) 3 (10): 877 (362), 1903 (Stephani 1903f).

*** Plagiochila obtusa Lindenb., Sp. Hepat. (Lindenberg) 2-4: 42, 1840 (Lindenberg 1840).

* Plagiochila owaihiensis Nees et Lindenb., Sp. Hepat. (Lindenberg) 1: 30, 1839 (Lindenberg 1839). ^169^

** Plagiochila pacifica Mitt., Fl. vit.: 407, 1871 [1873] (Mitten 1871).

** Plagiochila parallela Steph., Bull. Herb. Boissier (sér. 2) 2 (8): 686 (223), 1902 (Stephani 1902a).

*** Plagiochila parvifolia Lindenb., Sp. Hepat. (Lindenberg) 1: 28, 1839 (Lindenberg 1839).

*** Plagiochila patentissima Lindenb., Sp. Hepat. (Lindenberg) 2-4: 64, 1840 (Lindenberg 1840).

*** Plagiochila patula (Sw.) Lindenb., Sp. Hepat. (Lindenberg) 1: 21, 1839 (Lindenberg 1839). Bas.: Jungermannia patula Sw., Fl. Ind. Occid. 3: 1844, 1806 (Swartz 1806).

** Plagiochila patula var. brevifolia Gottsche, Mexik. Leverm.: 10, 1863 (Gottsche 1863).

** Plagiochila paucidens Steph., Bull. Herb. Boissier (sér. 2) 3 (2): 117 (297), 1903 (Stephani 1903b).

** Plagiochila pensilis Spruce, Trans. & Proc. Bot. Soc. Edinburgh 15: 497, 1885 (Spruce 1885).

** Plagiochila peradenyensis Schiffn., Denkschr. Kaiserl. Akad. Wiss., Math.-Naturwiss. Kl. 70: 172, 1900 [1901] (Schiffner 1900c).

** Plagiochila perdentata M.L.So et Grolle, Syst. Bot. 26 (3): 460, 2001 (So and Grolle 2001).

*** Plagiochila pinniflora Steph., Hedwigia 30 (5): 212, 1891 (Stephani 1891a).

*** Plagiochila praemorsa Steph., Bot. Jahrb. Syst. 8 (2): 92, 1886 (Stephani 1886d).

** Plagiochila propinqua Sande Lac., Plagiochila Sandei: 5, 1856 (Sande Lacoste 1856c).

** Plagiochila purpurascens Steph., Sp. Hepat. (Stephani) 6: 197, 1921 (Stephani 1921).

*** Plagiochila raddiana Lindenb., Sp. Hepat. (Lindenberg) 1: 9, 1839 (Lindenberg 1839).

* Plagiochila ragazzii Gola, Ann. Bot. (Rome) 13 (1): 67, 1914 (Gola 1914a).

*** Plagiochila repanda (Schwägr.) Lindenb., Sp. Hepat. (Lindenberg) 2-4: 62, 1840 (Lindenberg 1840). Bas.: Jungermannia repanda Schwägr., Hist. Musc. Hepat. Prodr.: 26, 1814 (Schwägrichen 1814).

** Plagiochila repanda var. perrotana (Steph.) Vanden Berghen, Bull. Jard. Bot. Natl. Belg. 51 (1/2): 65, 1981 (Vanden Berghen 1981). Bas.: Plagiochila perrotana Steph., Bull. Herb. Boissier (sér. 2) 4 (6): 586 (437), 1904 (Stephani 1904b).

*** Plagiochila rodriguezii Steph., Bot. Gaz. 15 (11): 290, 1890 (Stephani 1890c).

*** Plagiochila rudolfii Pócs, Beih. Nova Hedwigia 90: 223, 1988 (Pócs 1988).

*** Plagiochila salacensis Gottsche, Natuurk. Tijdschr. Ned.-Indië 4: 576, 1853 (Gottsche 1853).

*** Plagiochila salvadorica Steph., Hedwigia 30 (6): 272, 1891 (Stephani 1891c).

** Plagiochila serrialata Herzog, Hedwigia 72 (6): 212, 1932 (Herzog 1932c).

*** Plagiochila shangaica Steph., Sp. Hepat. (Stephani) 6: 216, 1921 (Stephani 1921).

*** Plagiochila simplex (Sw.) Lindenb., Sp. Hepat. (Lindenberg) 2-4: 54, 1840 (Lindenberg 1840). Bas.: Jungermannia simplex Sw., Prodr. (Swartz): 143, 1788 (Swartz 1788).

*** Plagiochila squamulosa Mitt., J. Proc. Linn. Soc., Bot. 7 (27): 165, 1863 (Mitten 1863).

** Plagiochila squamulosa var. crispulocaudata (Gottsche) Vanden Berghen, Bull. Jard. Bot. Natl. Belg. 51 (1/2): 74, 1981 (Vanden Berghen 1981). Bas.: Plagiochila crispulocaudata Gottsche, Abh. Naturwiss. Vereins Bremen 7: 340, 1882 (Gottsche 1882).

** Plagiochila squamulosa var. sinuosa (Mitt.) Vanden Berghen, Bull. Jard. Bot. Natl. Belg. 51 (1/2): 75, 1981 (Vanden Berghen 1981). Bas.: Plagiochila sinuosa Mitt., J. Linn. Soc., Bot. 22 (146): 319, 1886 (Mitten 1886b).

** Plagiochila streimannii Inoue, J. Jap. Bot. 63 (11): 365, 1988 (Inoue 1988c).

*** Plagiochila strictifolia Steph., Hedwigia 30 (5): 210, 1891 (Stephani 1891a).

** Plagiochila subflabellata Colenso, Trans. & Proc. New Zealand Inst. 21: 51, 1889 (Colenso 1889).

** Plagiochila subjavanica M.L.So, Austral. Syst. Bot. 13 (5): 804, 2000 (So 2000a).

*** Plagiochila subtropica Steph., Bull. Soc. Roy. Bot. Belgique 38 (1): 46, 1899 (Stephani 1899h).

** Plagiochila tamariscina Steph., Bull. Herb. Boissier (sér. 2) 2 (8): 685 (222), 1902 (Stephani 1902a).

** Plagiochila tecta Inoue et Grolle, J. Hattori Bot. Lab. 33: 319, 1970 (Inoue 1970a).

*** Plagiochila terebrans Nees et Mont., Sp. Hepat. (Lindenberg) 2-4: 98, 1840 (Lindenberg 1840).

*** Plagiochila teysmannii Sande Lac., Plagiochila Sandei: 6, 1856 (Sande Lacoste 1856c).

** Plagiochila thyoides Spruce, Trans. & Proc. Bot. Soc. Edinburgh 15: 498, 1885 (Spruce 1885).

** Plagiochila tocarema Gottsche, Ann. Sci. Nat. Bot. (sér. 5) 1: 106, 1864 (Gottsche 1864).

** Plagiochila ulata Inoue et Grolle, Bull. Natl. Sci. Mus. Tokyo, B 1 (3): 91, 1975 (Inoue 1975a).

** Plagiochila undata Sull., Amer. J. Sci. Arts (ser. 2) 1 (1): 73, 1846 (Gray 1846).

** Plagiochila undata subsp. crispata (Gottsche) R.M.Schust., Amer. Midl. Naturalist 63 (1): 122, 1960 (Schuster 1960c). Bas.: Plagiochila crispata Gottsche, Mexik. Leverm.: 71, 1863 (Gottsche 1863).

** Plagiochila ungarangana Sande Lac., Syn. hepat. jav.: 10, 1856 [1857] (Sande Lacoste 1856b).

*** Plagiochila virginica A.Evans, Prelim. cat. fl. W. Virginia: 497, 1892 (Evans 1892a).

** Plagiochila virginica var. caroliniana R.M.Schust., Amer. Midl. Naturalist 63 (1): 15, 1960 (Schuster 1960c).

** Plagiochila virginica var. euryphylla R.M.Schust., Amer. Midl. Naturalist 63 (1): 21, 1960 (Schuster 1960c).

*** Plagiochila wightii Nees, Sp. Hepat. (Lindenberg) 2-4: 43, 1840 (Lindenberg 1840).

** Plagiochila wilhelmina Inoue, J. Hattori Bot. Lab. 33: 317, 1970 (Inoue 1970a).

* **sect. Zanteniae (Inoue) Inoue**, Gen. Plagiochila SE Asia: 45, 1984 (Inoue 1984b). Bas.: Plagiochila subsect. Zanteniae Inoue, J. Hattori Bot. Lab. 32: 109, 1969 (Inoue 1969).

** Plagiochila zantenii Inoue, J. Hattori Bot. Lab. 32: 107, 1969 (Inoue 1969).

***Incertae sedis***

* Plagiochila abscendens Gottsche, Ann. Sci. Nat. Bot. (sér. 5) 1: 104, 1864 (Gottsche 1864).

** Plagiochila aculeata (Hook.f. et Taylor) Gottsche, Lindenb. et Nees, Syn. Hepat. 5: 627, 1847 (Gottsche et al. 1847). Bas.: Jungermannia aculeata Hook.f. et Taylor, London J. Bot. 3: 578, 1844 (Hooker and Taylor 1844c).

** Plagiochila acuta Steph., Bull. Herb. Boissier (sér. 2) 3 (7): 607 (353), 1903 (Stephani 1903e).

* Plagiochila albertii Steph., Biblioth. Bot. 87 (2): 188, 1916 (Stephani 1916a).

* Plagiochila aliena Gottsche, Mexik. Leverm.: 22, 1863 (Gottsche 1863). ^170^

** Plagiochila allionii Steph., Sp. Hepat. (Stephani) 6: 120, 1917 (Stephani 1917a).

* Plagiochila ambigua Lindenb. et Hampe, Linnaea 24 (6): 640, 1851 [1852] (Hampe 1851a).

* Plagiochila ampliata Steph., Biblioth. Bot. 87 (2): 189, 1916 (Stephani 1916a).

** Plagiochila andicola Mont. et Gottsche, Ann. Sci. Nat. Bot. (sér. 4) 6: 187, 1856 (Montagne 1856a).

** Plagiochila andina Steph., Sp. Hepat. (Stephani) 6: 121, 1917 (Stephani 1917a).

* Plagiochila angolensis Steph., Sp. Hepat. (Stephani) 6: 122, 1917 (Stephani 1917a).

* Plagiochila angusteoblonga Steph., Biblioth. Bot. 87 (2): 189, 1916 (Stephani 1916a).

** Plagiochila angustisedens Steph., Bull. Herb. Boissier (sér. 2) 5 (8): 743 (542), 1905 (Stephani 1905i).

** Plagiochila angustispina Steph., Bull. Herb. Boissier (sér. 2) 2 (8): 671 (208), 1902 (Stephani 1902a).

** Plagiochila apicalis Gottsche, Mexik. Leverm.: 29, 1863 (Gottsche 1863).

** Plagiochila appalachiana Inoue, J. Hattori Bot. Lab. 40: 415, 1976 (Inoue 1976b). *Nom. nov. pro *Plagiochila yokogurensis subsp. fragilifolia R.M.Schust., J. Hattori Bot. Lab. 18: 18, 1957 (Schuster 1957c).

* Plagiochila arcta S.Winkl., Rev. Bryol. Lichénol. 42 (3): 818, 1976 (Winkler 1976).

** Plagiochila arcuata Lindenb., Sp. Hepat. (Lindenberg) 2-4: 91, 1840 (Lindenberg 1840).

** Plagiochila arenacea Schiffn., Österr. Akad. Wiss., Math.-Naturwiss. Kl., Denkschr. 111: 53, 1964 (Schiffner and Arnell 1964).

* Plagiochila artsii Pócs, J. Hattori Bot. Lab. 100: 334, 2006 (Pócs 2006a).

** Plagiochila atrovirens Taylor, London J. Bot. 5: 266, 1846 (Taylor 1846a).

** Plagiochila balansae Gottsche, Contr. Étude Plagiochila: 149, 1928 (Dugas 1928).

** Plagiochila baldwinii Austin, Trans. Connecticut Acad. Arts 8 (15): 257, 1891 (Evans 1891).

* Plagiochila bamingensis Steph., Bull. Mus. Natl. Hist. Nat. 18 (2): 117, 1912 (Corbière 1912).

** Plagiochila bancroftii Steph., Sp. Hepat. (Stephani) 6: 127, 1917 (Stephani 1917a).

*** Plagiochila barbadensis Steph., Bull. Herb. Boissier (sér. 2) 5 (9): 897 (563), 1905 (Stephani 1905g).

* Plagiochila barbata Steph., Biblioth. Bot. 87 (2): 190, 1916 (Stephani 1916a).

* Plagiochila barbeyi Steph., Biblioth. Bot. 87 (2): 190, 1916 (Stephani 1916a).

* Plagiochila batava Steph., Sp. Hepat. (Stephani) 6: 128, 1917 (Stephani 1917a).

* Plagiochila berggrenii Steph., Biblioth. Bot. 87 (2): 191, 1916 (Stephani 1916a).

** Plagiochila beskeana Steph., Bull. Herb. Boissier (sér. 2) 2 (10): 863 (232), 1902 (Stephani 1902g).

** Plagiochila bialata Mitt., Fl. vit.: 407, 1871 [1873] (Mitten 1871).

* Plagiochila biapiculata Steph., Bull. Herb. Boissier (sér. 2) 5 (9): 891 (557), 1905 (Stephani 1905g).

* Plagiochila bicaudata Steph., Sp. Hepat. (Stephani) 6: 130, 1918 (Stephani 1918).

** Plagiochila biciliata Steph., Bull. Herb. Boissier (sér. 2) 3 (6): 529 (333), 1903 (Stephani 1903d).

** Plagiochila bicornis Hampe et Gottsche, Linnaea 25 (3): 338, 1852 [1853] (Hampe and Gottsche 1852).

* Plagiochila bidentula Steph., Sp. Hepat. (Stephani) 6: 130, 1918 (Stephani 1918).

** Plagiochila binghamiae A.Evans, Trans. Connecticut Acad. Arts 18 (5): 304, 1914 (Evans 1914c).

** Plagiochila binominata Steph., Sp. Hepat. (Stephani) 6: 131, 1918 (Stephani 1918).

** Plagiochila bitexta Dugas, Contr. Étude Plagiochila: 58, 1928 (Dugas 1928).

** Plagiochila blepharobasis Herzog, Hedwigia 72 (6): 216, 1932 (Herzog 1932c).

** Plagiochila bogotensis Gottsche, Ann. Sci. Nat. Bot. (sér. 5) 1: 98, 1864 (Gottsche 1864).

* Plagiochila boliviana Spruce, Mem. Torrey Bot. Club 1 (3): 137, 1890 (Spruce 1890).

* Plagiochila borneensis Steph., Sp. Hepat. (Stephani) 6: 132, 1918 (Stephani 1918). ^171^

** Plagiochila brassii Inoue et Grolle, J. Hattori Bot. Lab. 36: 492, 1972 [1973] (Inoue 1972b).

* Plagiochila brevicalycina Lindenb. et Gottsche, Linnaea 20 (3): 322, 1847 (Hampe 1847).

** Plagiochila brunneola Steph., Bull. Herb. Boissier (sér. 2) 4 (2): 164 (416), 1904 (Stephani 1904f).

*** Plagiochila bryhnii Steph., Biblioth. Bot. 87 (2): 192, 1916 (Stephani 1916a).

** Plagiochila bunburii Taylor, London J. Bot. 5: 269, 1846 (Taylor 1846a).

* Plagiochila byssacea Hampe, Linnaea 20 (3): 326, 1847 (Hampe 1847).

** Plagiochila caducidentata R.M.Schust., Phytologia 39 (4): 247, 1978 (Schuster 1978a).

** Plagiochila caldana Steph., Bull. Herb. Boissier (sér. 2) 2 (10): 879 (248), 1902 (Stephani 1902g).

** Plagiochila callaensis Steph., Sp. Hepat. (Stephani) 6: 136, 1918 (Stephani 1918).

* Plagiochila camusii Steph., Biblioth. Bot. 87 (2): 193, 1916 (Stephani 1916a).

* Plagiochila capillicaulis Steph., Biblioth. Bot. 87 (2): 193, 1916 (Stephani 1916a).

** Plagiochila caribbeania R.M.Schust., Hepat. Anthocerotae N. Amer. 4: 412, 1980 (Schuster 1980c).

* Plagiochila cava Steph., Bull. Herb. Boissier (sér. 2) 4 (12): 1213 (501), 1904 (Stephani 1904h).

** Plagiochila ceylanica Mitt., J. Proc. Linn. Soc., Bot. 5 (18): 98, 1860 [1861] (Mitten 1860c).

** Plagiochila chacoënsis Herzog, Feddes Repert. Spec. Nov. Regni Veg. 55 (1): 7, 1952 (Herzog 1952h).

** Plagiochila chiloënsis Steph., Bih. Kongl. Svenska Vetensk.-Akad. Handl. 26 (III, 6): 27, 1900 (Stephani 1900b).

** Plagiochila chinantlana Gottsche, Mexik. Leverm.: 12, 1863 (Gottsche 1863).

* Plagiochila chiovendae Gola, Ann. Bot. (Rome) 13 (1): 67, 1914 (Gola 1914a).

* Plagiochila cinchonae Steph., Bull. Herb. Boissier (sér. 2) 5 (10): 920 (570), 1905 (Stephani 1905f).

* Plagiochila circumvoluta Gerola, Lav. Bot. Ist. Bot. Univ. Padova 12: 475, 1947 (Gerola 1947).

*** Plagiochila cleefii Inoue, Stud. Cryptog. S. Peru: 95, 1987 (Inoue 1987c).

* Plagiochila concava Nees, Sp. Hepat. (Lindenberg) 2-4: 70, 1840 (Lindenberg 1840).

** Plagiochila conduplicata Steph., Sp. Hepat. (Stephani) 6: 139, 1918 (Stephani 1918).

* Plagiochila conferta Steph., Sp. Hepat. (Stephani) 6: 139, 1918 (Stephani 1918).

** Plagiochila confertissima Steph., Bull. Herb. Boissier (sér. 2) 5 (2): 182 (510), 1905 (Stephani 1905d).

** Plagiochila connata Lindenb. et Gottsche, Syn. Hepat. 5: 645, 1847 (Gottsche et al. 1847).

** Plagiochila contorta Lindenb. et Hampe, Linnaea 24 (3): 301, 1851 [1852] (Hampe 1851b).

** Plagiochila convoluta Steph., Sp. Hepat. (Stephani) 6: 141, 1918 (Stephani 1918).

** Plagiochila convolutifolia Steph., Sp. Hepat. (Stephani) 6: 142, 1918 (Stephani 1918).

*** Plagiochila corymbulosa Pearson, Forh. Vidensk.-Selsk. Kristiania 1887 (9): 14, 1887 (Pearson 1887b).

** Plagiochila costariensis Horik., Acta Phytotax. Geobot. 13: 213, 1943 (Horikawa 1943). *Nom. nov. pro Plagiochila pinnata* Steph., Bull. Herb. Boissier (sér. 2) 5 (8): 749 (548), 1905 (Stephani 1905i), *nom. illeg*.

** Plagiochila crispabilis Lindenb., Sp. Hepat. (Lindenberg) 1: 15, 1839 (Lindenberg 1839).

* Plagiochila crispabilis var. minima Lindenb., Sp. Hepat. (Lindenberg) 5: 155, 1843 (Lindenberg 1843).

** Plagiochila cristophylla Steph., Bull. Herb. Boissier (sér. 2) 3 (2): 117 (297), 1903 (Stephani 1903b).

*** Plagiochila cuatrecasii H.Rob., Bryologist 70 (1): 47, 1967 (Robinson 1967).

** Plagiochila cubensis Steph., Sp. Hepat. (Stephani) 6: 143, 1918 (Stephani 1918).

** Plagiochila cucullata Lindenb. et Gottsche, Syn. Hepat. 5: 642, 1847 (Gottsche et al. 1847).

* Plagiochila cuervina Gottsche, Ann. Sci. Nat. Bot. (sér. 5) 1: 96, 1864 (Gottsche 1864).

** Plagiochila delapsa Inoue, Beih. Nova Hedwigia 90: 171, 1988 (Inoue 1988a).

* Plagiochila delognei Steph., Biblioth. Bot. 87 (2): 196, 1916 (Stephani 1916a).

** Plagiochila denigrata Inoue, Willdenowia 18 (2): 557, 1989 (Inoue 1989b).

** Plagiochila densa Herzog, Hedwigia 72 (6): 222, 1932 (Herzog 1932c).

** Plagiochila densiflora Herzog, Hedwigia 72 (6): 226, 1932 (Herzog 1932c).

** Plagiochila densiramosa Steph., Biblioth. Bot. 87 (2): 196, 1916 (Stephani 1916a).

** Plagiochila deppeana Steph., Bull. Herb. Boissier (sér. 2) 5 (9): 886 (552), 1905 (Stephani 1905g).

** Plagiochila desciscens Steph., Bull. Herb. Boissier (sér. 2) 2 (10): 867 (236), 1902 (Stephani 1902g).

* Plagiochila dilatata Steph., Bull. Herb. Boissier (sér. 2) 5 (9): 887 (553), 1905 (Stephani 1905g).

* Plagiochila distans Colenso, Trans. & Proc. New Zealand Inst. 19: 283, 1887 (Colenso 1887). ^172^

** Plagiochila divaricata Lindenb., Sp. Hepat. (Lindenberg) 5: 147, 1843 (Lindenberg 1843).

* Plagiochila doerfleri Steph., Biblioth. Bot. 87 (2): 196, 1916 (Stephani 1916a).

** Plagiochila dolichoblasta Herzog, Trans. Brit. Bryol. Soc. 1 (4): 286, 1950 (Herzog 1950a).

** Plagiochila dussiana Steph., Symb. Antill. (Urban) 3 (2): 277, 1902 (Stephani 1902e).

** Plagiochila echinata R.M.Schust., Amer. Midl. Naturalist 62 (2): 341, 1959 (Schuster 1959b).

** Plagiochila echinella Gottsche, Ann. Sci. Nat. Bot. (sér. 4) 8: 332, 1857 (Gottsche 1857).

* Plagiochila ecuadorensis Steph., Sp. Hepat. (Stephani) 6: 149, 1918 (Stephani 1918).

* Plagiochila effuseintricata Steph., Biblioth. Bot. 87 (2): 197, 1916 (Stephani 1916a).

** Plagiochila eggersii Inoue, Bull. Natl. Sci. Mus. Tokyo, B 14 (4): 138, 1988 (Inoue 1988b).

** Plagiochila ekmanii S.W.Arnell, Bryologist 59 (4): 274, 1956 (Arnell 1956c).

* Plagiochila electa Steph., Bull. Herb. Boissier (sér. 2) 5 (8): 739 (538), 1905 (Stephani 1905i).

* Plagiochila elegantula Herzog, Feddes Repert. Spec. Nov. Regni Veg. 57 (1/2): 161, 1955 (Herzog 1955).

* Plagiochila emarginatobidentula Steph., Biblioth. Bot. 87 (2): 197, 1916 (Stephani 1916a).

* Plagiochila erythraeae Herzog, Hedwigia 78 (3/4): 224, 1938 (Herzog 1938d).

** Plagiochila estrellensis Herzog, Repert. Spec. Nov. Regni Veg. 21 (1/7): 23, 1925 (Herzog 1925a).

** Plagiochila eurydictyon Herzog, Hedwigia 72 (6): 207, 1932 (Herzog 1932c).

* Plagiochila excisa Steph., Sp. Hepat. (Stephani) 6: 153, 1918 (Stephani 1918).

** Plagiochila exesa Lindenb. et Gottsche, Syn. Hepat. 5: 629, 1847 (Gottsche et al. 1847).

* Plagiochila exilis Colenso, Trans. & Proc. New Zealand Inst. 19: 282, 1887 (Colenso 1887). ^173^

** Plagiochila expansa Gottsche, Mexik. Leverm.: 18, 1863 (Gottsche 1863).

** Plagiochila facallonia Steph., Bull. Herb. Boissier (sér. 2) 2 (10): 871 (240), 1902 (Stephani 1902g).

* Plagiochila falcato-oblonga Steph., Biblioth. Bot. 87 (2): 198, 1916 (Stephani 1916a).

** Plagiochila fallax Lindenb. et Hampe, Linnaea 24 (3): 300, 1851 [1852] (Hampe 1851b).

** Plagiochila faxinensis Schiffn., Österr. Akad. Wiss., Math.-Naturwiss. Kl., Denkschr. 111: 60, 1964 (Schiffner and Arnell 1964).

** Plagiochila fendleri Mont., Ann. Sci. Nat. Bot. (sér. 4) 6: 198, 1856 (Montagne 1856a).

* Plagiochila filicicola Steph., Bull. Herb. Boissier (sér. 2) 4 (4): 351 (427), 1904 (Stephani 1904e).

** Plagiochila fissidentoides Taylor, London J. Bot. 5: 264, 1846 (Taylor 1846a).

* Plagiochila flabellifrons Spruce, Trans. & Proc. Bot. Soc. Edinburgh 15: 488, 1885 (Spruce 1885).

** Plagiochila flava Steph., Sp. Hepat. (Stephani) 6: 156, 1918 (Stephani 1918).

** Plagiochila flavescens (Gottsche, Lindenb. et Nees) Gottsche, Mexik. Leverm.: 52, 1863 (Gottsche 1863). Bas.: Plagiochila guilleminiana β flavescens Gottsche, Lindenb. et Nees, Syn. Hepat. 5: 644, 1847 (Gottsche et al. 1847).

* Plagiochila flavorufescens Steph., Biblioth. Bot. 87 (2): 199, 1916 (Stephani 1916a).

** Plagiochila footei A.Evans, Trans. Connecticut Acad. Arts 18 (5): 306, 1914 (Evans 1914c).

* Plagiochila formosa Nees, Contr. Étude Plagiochila: 147, 1928 (Dugas 1928).

** Plagiochila fragmentata R.M.Schust., Phytologia 45 (5): 421, 1980 (Schuster 1980b).

* Plagiochila frausa Gottsche, Mexik. Leverm.: 66, 1863 (Gottsche 1863).

* Plagiochila frausa var. boliviana Spruce, Mem. Torrey Bot. Club 1 (3): 134, 1890 (Spruce 1890).

** Plagiochila frayjorgensis Hässel, Bol. Soc. Argent. Bot. 22 (1/4): 103, 1983 (Hässel 1983). *Nom. nov. pro Plagiochila modesta* Herzog, Rev. Bryol. Lichénol. 23 (1/2): 34, 1954 (Herzog 1954), *nom. illeg*.

* Plagiochila gaffatensis Gottsche ex Schweinf., Beitr. Fl. Aethiop.: 227, 1866 [1867] (Schweinfurth 1866).

** Plagiochila gaudichaudii Mont. et Gottsche, Ann. Sci. Nat. Bot. (sér. 4) 6: 193, 1856 (Montagne 1856a).

** Plagiochila geniculata Lindenb., Sp. Hepat. (Lindenberg) 5: 131, 1843 (Lindenberg 1843).

* Plagiochila geppii Steph., Biblioth. Bot. 87 (2): 199, 1916 (Stephani 1916a).

** Plagiochila germana Gottsche, Mexik. Leverm.: 34, 1863 (Gottsche 1863).

** Plagiochila germanii Steph., Bull. Herb. Boissier (sér. 2) 5 (10): 938 (588), 1905 (Stephani 1905f).

* Plagiochila gibbiflora Steph., Bull. Herb. Boissier (sér. 2) 4 (6): 590 (441), 1904 (Stephani 1904b).

** Plagiochila gittinsii Inoue, Bull. Natl. Sci. Mus. Tokyo, B 3 (4): 139, 1977 (Inoue 1977a).

* Plagiochila glauca Carl, Ann. Bryol., Suppl. 2: 129, 1931 (Carl 1931b). ^174^

* Plagiochila gracilicaulis Spruce, Mem. Torrey Bot. Club 1 (3): 132, 1890 (Spruce 1890).

** Plagiochila gracillima Austin, Trans. Connecticut Acad. Arts 8 (15): 256, 1891 (Evans 1891).

* Plagiochila granatensis Gottsche, Ann. Sci. Nat. Bot. (sér. 5) 1: 111, 1864 (Gottsche 1864).

** Plagiochila granditexta Steph., Bull. Herb. Boissier (sér. 2) 4 (2): 165 (417), 1904 (Stephani 1904f).

** Plagiochila grateloupii Mont., Ann. Sci. Nat. Bot. (sér. 4) 6: 188, 1856 (Montagne 1856a).

** Plagiochila guatemalensis Steph., Sp. Hepat. (Stephani) 6: 163, 1918 (Stephani 1918).

** Plagiochila guttisquama Inoue et Grolle, Bull. Natl. Sci. Mus. Tokyo, B 5 (1): 29, 1979 (Inoue 1979c).

** Plagiochila gymnocalyx Inoue, Trop. Bryol. 1: 34, 1989 (Gradstein and Florschütz-de Waard 1989).

** Plagiochila haeseliae Inoue, Stud. Cryptog. S. Chile: 97, 1984 (Inoue 1984a).

* Plagiochila hans-meyeri Steph., Sp. Hepat. (Stephani) 6: 164, 1918 (Stephani 1918).

** Plagiochila harlingii S.W.Arnell, Svensk Bot. Tidskr. 56 (2): 346, 1962 (Arnell 1962b).

** Plagiochila haumanii Herzog, Repert. Spec. Nov. Regni Veg. 41 (14/25): 285, 1937 (Herzog 1937).

** Plagiochila hawaica Steph., Bull. Herb. Boissier (sér. 2) 3 (7): 598 (344), 1903 (Stephani 1903e).

** Plagiochila heteracantha Steph., Sp. Hepat. (Stephani) 6: 166, 1918 (Stephani 1918).

* Plagiochila heterofolia Steph., Biblioth. Bot. 87 (2): 200, 1916 (Stephani 1916a).

* Plagiochila hieronymii Steph., Biblioth. Bot. 87 (2): 201, 1916 (Stephani 1916a).

** Plagiochila hiroshiana Pócs, J. Hattori Bot. Lab. 100: 335, 2006 (Pócs 2006a).

** Plagiochila hoehnei Herzog, Hedwigia 72 (6): 220, 1932 (Herzog 1932c).

* Plagiochila holstii Steph., Hedwigia 45 (4): 214, 1906 (Stephani 1906b). *Nom. nov. pro Plagiochila prostrata* Steph., Bull. Herb. Boissier (sér. 2) 4 (2): 168 (420), 1904 (Stephani 1904f), *nom. illeg*.

** Plagiochila horrida Gottsche, Mexik. Leverm.: 74, 1863 (Gottsche 1863).

* Plagiochila huatuscana Gottsche, Mexik. Leverm.: 24, 1863 (Gottsche 1863).

** Plagiochila huerlimannii Inoue, J. Hattori Bot. Lab. 33: 307, 1970 (Inoue 1970b).

* Plagiochila humboldtiana Gottsche, Ann. Sci. Nat. Bot. (sér. 5) 1: 112, 1864 (Gottsche 1864).

** Plagiochila hyalina Lindenb., Syn. Hepat. 5: 640, 1847 (Gottsche et al. 1847).

* Plagiochila incisa Dugas, Contr. Étude Plagiochila: 112, 1928 (Dugas 1928).

** Plagiochila inflata Steph., Bull. Herb. Boissier (sér. 2) 3 (11): 961 (376), 1903 (Stephani 1903a).

* Plagiochila informifolia Steph., Biblioth. Bot. 87 (2): 202, 1916 (Stephani 1916a).

** Plagiochila infuscata Mitt., J. Linn. Soc., Bot. 15 (82): 63, 1876 (Mitten 1876a).

* Plagiochila injasutiensis S.W.Arnell, Bot. Not. 110: 404, 1957 (Arnell 1957d).

** Plagiochila inouei Grolle, J. Bryol. 10 (3): 269, 1979 (Grolle 1979b). *Nom. nov. pro Plagiochila nudiuscula* Inoue, Bull. Natl. Sci. Mus. Tokyo, B 3 (2): 45, 1977 (Inoue 1977c), *nom. illeg*.

** Plagiochila insularia Mitt., St. Helena: 366, 1875 (Mitten 1875).

** Plagiochila intertexta Hook.f. et Taylor, London J. Bot. 5: 267, 1846 (Taylor 1846a).

* Plagiochila inuensis Steph., Biblioth. Bot. 87 (2): 202, 1916 (Stephani 1916a).

** Plagiochila irregularis Gottsche, Mexik. Leverm.: 14, 1863 (Gottsche 1863).

** Plagiochila itatiajensis Steph., Bull. Herb. Boissier (sér. 2) 2 (10): 874 (243), 1902 (Stephani 1902g).

** Plagiochila jamaicensis Lindenb. et Hampe, Linnaea 24 (3): 302, 1851 [1852] (Hampe 1851b).

** Plagiochila jaramillii H.Rob., Bryologist 70 (1): 48, 1967 (Robinson 1967).

* Plagiochila karstenii Steph., Hedwigia 45 (4): 214, 1906 (Stephani 1906b). *Nom. nov. pro Plagiochila patentispina* Steph., Bull. Herb. Boissier (sér. 2) 4 (1): 28 (400), 1904 (Stephani 1904g), *nom. illeg*. ^175^

* Plagiochila kaulfussiana Steph., Sp. Hepat. (Stephani) 6: 172, 1918 (Stephani 1918).

** Plagiochila keckiana Steph., Bull. Herb. Boissier (sér. 2) 5 (4): 358 (526), 1905 (Stephani 1905j).

** Plagiochila kerneriana S.W.Arnell, Österr. Akad. Wiss., Math.-Naturwiss. Kl., Denkschr. 111: 60, 1964 (Schiffner and Arnell 1964).

** Plagiochila koponenii Inoue et Piippo, Ann. Bot. Fenn. 26 (2): 216, 1989 (Piippo 1989b).

* Plagiochila lacerifolia Steph., Biblioth. Bot. 87 (2): 202, 1916 (Stephani 1916a).

* Plagiochila lachenaudii Steph., Biblioth. Bot. 87 (2): 202, 1916 (Stephani 1916a).

* Plagiochila laciniosa Dugas, Contr. Étude Plagiochila: 68, 1928 (Dugas 1928).

* Plagiochila laevifolia Gola, Ann. Bot. (Rome) 6 (2): 273, 1907 (Gola 1907).

** Plagiochila lansbergii Gottsche, Mexik. Leverm.: 13, 1863 (Gottsche 1863).

** Plagiochila latitrigona Schiffn., Österr. Akad. Wiss., Math.-Naturwiss. Kl., Denkschr. 111: 48, 1964 (Schiffner and Arnell 1964).

* Plagiochila laxiramea Steph., Biblioth. Bot. 87 (2): 203, 1916 (Stephani 1916a).

* Plagiochila lecontei Steph., Bull. Herb. Boissier (sér. 2) 4 (2): 165 (417), 1904 (Stephani 1904f). ^176^

* Plagiochila ledermanniana Beauverd, Sp. Hepat. (Stephani) 6: 572, 1924 (Stephani 1924). *Nom. nov. pro Plagiochila cucullifolia* Steph., Sp. Hepat. (Stephani) 6: 243, 1922 (Stephani 1922), *nom. illeg*. ^177^

* Plagiochila ledieui Steph., Bull. Herb. Boissier (sér. 2) 4 (2): 161 (413), 1904 (Stephani 1904f). ^178^

** Plagiochila lehmanniana S.W.Arnell, Svensk Bot. Tidskr. 55 (1): 205, 1961 (Arnell 1961).

** Plagiochila liebmanniana Lehm. et Lindenb., Sp. Hepat. (Lindenberg) 2-4: 97, 1840 (Lindenberg 1840).

* Plagiochila lignicola Spruce, Mem. Torrey Bot. Club 1 (3): 135, 1890 (Spruce 1890).

* Plagiochila lindaui Steph., Biblioth. Bot. 87 (2): 204, 1916 (Stephani 1916a).

* Plagiochila lindigiana Gottsche, Ann. Sci. Nat. Bot. (sér. 5) 1: 105, 1864 (Gottsche 1864).

** Plagiochila lingua Steph., Bull. Herb. Boissier (sér. 2) 2 (8): 677 (214), 1902 (Stephani 1902a).

* Plagiochila longifissa Steph., Bull. Herb. Boissier (sér. 2) 5 (9): 891 (557), 1905 (Stephani 1905g).

* Plagiochila lotsyana Steph., Biblioth. Bot. 87 (2): 205, 1916 (Stephani 1916a).

* Plagiochila luteola Steph., Bull. Herb. Boissier (sér. 2) 5 (2): 175 (503), 1905 (Stephani 1905d).

** Plagiochila lutescens Steph., Sp. Hepat. (Stephani) 6: 179, 1921 (Stephani 1921).

** Plagiochila luzonensis Grolle et M.L.So, Bryologist 102 (1): 69, 1999 (Grolle and So 1999a).

* Plagiochila macra Taylor, London J. Bot. 7: 198, 1848 (Taylor 1848a).

** Plagiochila macrifolia Taylor, London J. Bot. 5: 270, 1846 (Taylor 1846a).

* Plagiochila macrifolia var. angustifolia Spruce, Trans. & Proc. Bot. Soc. Edinburgh 15: 470, 1885 (Spruce 1885).

*** Plagiochila martiana (Nees) Lindenb., Sp. Hepat. (Lindenberg) 1: 12, 1839 (Lindenberg 1839). Bas.: Jungermannia martiana Nees, Linnaea 6 (4): 617, 1831 (Nees 1831).

* Plagiochila matanga Steph., Sp. Hepat. (Stephani) 6: 183, 1921 (Stephani 1921).

** Plagiochila maunakeana Steph., Sp. Hepat. (Stephani) 6: 186, 1921 (Stephani 1921).

** Plagiochila maximiliana Gottsche, Mexik. Leverm.: 18, 1863 (Gottsche 1863).

** Plagiochila meghalayensis K.K.Rawat et S.C.Srivast., Geophytology 35 (1/2): 49, 2005 (Rawat and Srivastava 2005).

** Plagiochila meridana Gottsche, Ann. Sci. Nat. Bot. (sér. 4) 8: 328, 1857 (Gottsche 1857).

** Plagiochila microdonta Mitt., J. Proc. Linn. Soc., Bot. 5 (18): 97, 1860 [1861] (Mitten 1860c).

* Plagiochila mildbreadiana Beauverd, Sp. Hepat. (Stephani) 6: 571, 1924 (Stephani 1924). *Nom. nov. pro Plagiochila andongensis* Steph., Sp. Hepat. (Stephani) 6: 122, 1917 (Stephani 1917a), *nom. illeg*.

** Plagiochila minarum Herzog, Hedwigia 72 (6): 207, 1932 (Herzog 1932c).

** Plagiochila minutiretis Reimers, Repert. Spec. Nov. Regni Veg. 21 (8/20): 264, 1925 (Reimers 1925).

** Plagiochila miqueliana Lehm. et Lindenb., Sp. Hepat. (Lindenberg) 2-4: 95, 1840 (Lindenberg 1840).

** Plagiochila molliuscula Inoue, Ann. Bot. Fenn. 13 (3): 134, 1976 (Engel 1976a).

** Plagiochila mollusca Lehm., Nov. Stirp. Pug. 10: 4, 1857 (Lehmann 1857).

** Plagiochila moniliformis R.M.Schust., Phytologia 39 (4): 247, 1978 (Schuster 1978a).

** Plagiochila muelleriana Gottsche, Mexik. Leverm.: 38, 1863 (Gottsche 1863).

* Plagiochila multiflora Steph., Pflanzenw. Ost-Afrikas C: 64, 1895 (Stephani 1895d). ^179^

** Plagiochila mutila Gottsche, Ann. Sci. Nat. Bot. (sér. 4) 8: 327, 1857 (Gottsche 1857).

* Plagiochila naranjoënsis Steph., Bull. Herb. Boissier (sér. 2) 2 (8): 687 (224), 1902 (Stephani 1902a).

** Plagiochila neesiana Lindenb., Sp. Hepat. (Lindenberg) 2-4: 71, 1840 (Lindenberg 1840).

* Plagiochila neglecta Steph., Bull. Herb. Boissier (sér. 2) 5 (4): 351 (519), 1905 (Stephani 1905j).

* Plagiochila negrensis Spruce, Trans. & Proc. Bot. Soc. Edinburgh 15: 466, 1885 (Spruce 1885).

* Plagiochila negrii Gola, Ann. Bot. (Rome) 13 (1): 66, 1914 (Gola 1914a).

* Plagiochila nigricaulis Steph., Biblioth. Bot. 87 (2): 206, 1916 (Stephani 1916a).

* Plagiochila notha Steph., Bull. Herb. Boissier (sér. 2) 5 (8): 737 (536), 1905 (Stephani 1905i).

* Plagiochila nova Steph., Sp. Hepat. (Stephani) 6: 189, 1921 (Stephani 1921).

** Plagiochila nudifolia (Steph.) Grolle, Feddes Repert. 82 (1): 89, 1971 (Grolle 1971b). Bas.: Jamesoniella nudifolia Steph., Biblioth. Bot. 87 (2): 184, 1916 (Stephani 1916a).

** Plagiochila nutans Steph., Bull. Herb. Boissier (sér. 2) 3 (11): 960 (375), 1903 (Stephani 1903a).

* Plagiochila obliquetruncata Steph., Biblioth. Bot. 87 (2): 207, 1916 (Stephani 1916a).

** Plagiochila oblonga Inoue, Bull. Natl. Sci. Mus. Tokyo (n.ser.) 8 (3): 398, 1965 (Inoue 1965a).

** Plagiochila oerstediana Lindenb. et Hampe, Linnaea 24 (3): 301, 1851 [1852] (Hampe 1851b).

** Plagiochila olivacea Steph., Bull. Herb. Boissier (sér. 2) 5 (2): 190 (518), 1905 (Stephani 1905d).

* Plagiochila ornata Wilson ex Lindenb., Sp. Hepat. (Lindenberg) 5: 164, 1843 (Lindenberg 1843).

* Plagiochila ovato-obconica Steph., Sp. Hepat. (Stephani) 6: 191, 1921 (Stephani 1921).

* Plagiochila ovifolia Steph., Sp. Hepat. (Stephani) 6: 191, 1921 (Stephani 1921).

** Plagiochila palangiensis S.C.Srivast., K.K.Rawat et P.K.Verma, Natl. Acad. Sci. Lett. 29 (7/8): 267, 2006 (Srivastava et al. 2006).

** Plagiochila panamensis Inoue, Bull. Natl. Sci. Mus. Tokyo, B 15 (3): 94, 1989 (Inoue 1989a).

** Plagiochila paramicola Herzog, Beih. Bot. Centralbl. 61B (3): 563, 1942 (Herzog 1942d).

* Plagiochila parvitexta Steph., Bull. Herb. Boissier (sér. 2) 2 (8): 674 (211), 1902 (Stephani 1902a).

** Plagiochila parvivittata Inoue, Bull. Natl. Sci. Mus. Tokyo, B 13 (2): 50, 1987 (Inoue 1987b).

* Plagiochila parvula Steph., Akad. Wiss. Wien, Math.-Naturwiss. Kl., Denkschr. 88: 726, 1913 (Stephani 1913b).

* Plagiochila pastasensis Steph., Sp. Hepat. (Stephani) 6: 197, 1921 (Stephani 1921).

** Plagiochila patentispina Steph., Bull. Herb. Boissier (sér. 2) 2 (10): 877 (246), 1902 (Stephani 1902g).

** Plagiochila patuloides Schiffn., Österr. Akad. Wiss., Math.-Naturwiss. Kl., Denkschr. 111: 58, 1964 (Schiffner and Arnell 1964).

** Plagiochila paucidentata Mont. et Gottsche, Ann. Sci. Nat. Bot. (sér. 4) 6: 197, 1856 (Montagne 1856a).

** Plagiochila paupercula Gottsche, Mexik. Leverm.: 22, 1863 (Gottsche 1863).

* Plagiochila pearceana Steph., Bull. Herb. Boissier (sér. 2) 5 (8): 742 (541), 1905 (Stephani 1905i).

** Plagiochila pellucida Lindenb. et Gottsche, Linnaea 20 (3): 321, 1847 (Hampe 1847).

** Plagiochila perrottetiana Mont. et Gottsche, Ann. Sci. Nat. Bot. (sér. 4) 6: 195, 1856 (Montagne 1856a).

** Plagiochila perrottetiana var. minor Gottsche, Ann. Sci. Nat. Bot. (sér. 5) 1: 101, 1864 (Gottsche 1864).

** Plagiochila pilifera Steph., Sp. Hepat. (Stephani) 6: 198, 1921 (Stephani 1921).

** Plagiochila pinnatidens Steph., Sp. Hepat. (Stephani) 6: 199, 1921 (Stephani 1921).

** Plagiochila pittieri Steph., Bull. Herb. Boissier (sér. 2) 2 (8): 673 (210), 1902 (Stephani 1902a).

** Plagiochila platyphylla Herzog, Hedwigia 72 (6): 223, 1932 (Herzog 1932c).

** Plagiochila polopolensis Herzog, Hedwigia 67 (6): 261, 1927 (Herzog 1927).

* Plagiochila prostrata Steph., Bull. Herb. Boissier (sér. 2) 2 (8): 668 (205), 1902 (Stephani 1902a).

* Plagiochila pseudopatula Herzog, Hedwigia 72 (6): 227, 1932 (Herzog 1932c).

** Plagiochila pseudoradicans Herzog, Repert. Spec. Nov. Regni Veg. 21 (1/7): 24, 1925 (Herzog 1925a).

** Plagiochila ptychanthoidea Steph., Bull. Herb. Boissier (sér. 2) 3 (2): 121 (301), 1903 (Stephani 1903b).

** Plagiochila pulchella Steph., Bull. Herb. Boissier (sér. 2) 2 (10): 871 (240), 1902 (Stephani 1902g).

** Plagiochila pulvinata Steph., Bull. Herb. Boissier (sér. 2) 3 (6): 526 (330), 1903 (Stephani 1903d).

** Plagiochila ratkowskiana Inoue, Brunonia 3 (1): 141, 1980 (Inoue 1980).

** Plagiochila regeliana Steph., Bull. Herb. Boissier (sér. 2) 2 (8): 675 (212), 1902 (Stephani 1902a).

** Plagiochila remyana Steph., Bull. Herb. Boissier (sér. 2) 3 (11): 963 (378), 1903 (Stephani 1903a).

*** Plagiochila revolvens Mitt., Hooker’s J. Bot. Kew Gard. Misc. 3: 358, 1851 (Mitten 1851).

** Plagiochila rigidula Lindenb. et Gottsche, Linnaea 20 (3): 323, 1847 (Hampe 1847).

** Plagiochila rosana Steph., Bull. Herb. Boissier (sér. 2) 5 (9): 892 (558), 1905 (Stephani 1905g).

* Plagiochila rubricaulis Steph., Bot. Jahrb. Syst. 20 (3): 311, 1895 (Stephani 1895a).

* Plagiochila rufifolia Steph., Biblioth. Bot. 87 (2): 209, 1916 (Stephani 1916a).

** Plagiochila rufoviridis Spruce, Mem. Torrey Bot. Club 1 (3): 136, 1890 (Spruce 1890).

* Plagiochila rusbyi Spruce, Mem. Torrey Bot. Club 1 (3): 135, 1890 (Spruce 1890).

* Plagiochila sabensis Steph., Sp. Hepat. (Stephani) 6: 214, 1921 (Stephani 1921).

** Plagiochila sachapatensis Steph., Bull. Herb. Boissier (sér. 2) 2 (8): 679 (216), 1902 (Stephani 1902a).

** Plagiochila salazariae Inoue, Bull. Natl. Sci. Mus. Tokyo, B 15 (3): 91, 1989 (Inoue 1989a).

** Plagiochila saltuensis Spruce ex Steph., Bull. Herb. Boissier (sér. 2) 5 (10): 927 (577), 1905 (Stephani 1905f).

** Plagiochila saltuensis var. spinosissima Herzog, Feddes Repert. Spec. Nov. Regni Veg. 57 (1/2): 163, 1955 (Herzog 1955).

* Plagiochila samoana Steph., Akad. Wiss. Wien, Math.-Naturwiss. Kl., Denkschr. 85: 197, 1910 (Stephani 1910a).

** Plagiochila sarmentosa (Lehm. et Lindenb.) Lindenb., Sp. Hepat. (Lindenberg) 2-4: 86, 1840 (Lindenberg 1840). Bas.: Jungermannia sarmentosa Lehm. et Lindenb., Linnaea 9 (4): 427, 1835 (Lehmann 1835).

* Plagiochila schiffneri Steph., Biblioth. Bot. 87 (2): 209, 1916 (Stephani 1916a).

* Plagiochila schinzei Steph., Biblioth. Bot. 87 (2): 209, 1916 (Stephani 1916a).

* Plagiochila schmidtii Steph., Biblioth. Bot. 87 (2): 209, 1916 (Stephani 1916a).

* Plagiochila schraderbergii Steph., Sp. Hepat. (Stephani) 6: 244, 1922 (Stephani 1922). ^180^

** Plagiochila schubertiana Steph., Sp. Hepat. (Stephani) 6: 208, 1921 (Stephani 1921).

** Plagiochila schusteri Inoue et Grolle, J. Hattori Bot. Lab. 33: 326, 1970 (Inoue 1970a).

** Plagiochila scissifolia Steph., Bull. Herb. Boissier (sér. 2) 2 (10): 865 (234), 1902 (Stephani 1902g).

* Plagiochila scotica Macvicar ex Steph., Sp. Hepat. (Stephani) 6: 209, 1921 (Stephani 1921). ^181^

** Plagiochila semiamplexicaulis Steph., Bull. Herb. Boissier (sér. 2) 5 (10): 936 (586), 1905 (Stephani 1905f).

** Plagiochila semiermis Dugas, Contr. Étude Plagiochila: 66, 1928 (Dugas 1928).

** Plagiochila silvatica Gottsche, Ann. Sci. Nat. Bot. (sér. 5) 1: 108, 1864 (Gottsche 1864).

* Plagiochila similis Steph., Biblioth. Bot. 87 (2): 210, 1916 (Stephani 1916a).

** Plagiochila sisparensis Steph., Sp. Hepat. (Stephani) 6: 207, 1921 (Stephani 1921).

* Plagiochila slateri Steph., Biblioth. Bot. 87 (2): 211, 1916 (Stephani 1916a).

** Plagiochila solitaria Gottsche, Sp. Hepat. (Stephani) 6: 225, 1921 (Stephani 1921).

*** Plagiochila spinifera Ångstr., Öfvers. Kongl. Vetensk.-Akad. Förh. 30 (5): 113, 1873 (Ångström 1873).

* Plagiochila sprucei Steph., Bull. Herb. Boissier (sér. 2) 2 (10): 860 (229), 1902 (Stephani 1902g).

* Plagiochila staudtiana Steph., Bull. Herb. Boissier (sér. 2) 4 (4): 352 (428), 1904 (Stephani 1904e).

** Plagiochila stipata Steph., Sp. Hepat. (Stephani) 6: 209, 1921 (Stephani 1921).

* Plagiochila stolzii Steph., Sp. Hepat. (Stephani) 6: 244, 1922 (Stephani 1922).

** Plagiochila subcontigua Herzog, Hedwigia 72 (6): 230, 1932 (Herzog 1932c).

* Plagiochila subconvoluta Gottsche, Mexik. Leverm.: 24, 1863 (Gottsche 1863).

* Plagiochila subdenudata Steph., Bull. Herb. Boissier (sér. 2) 2 (8): 673 (210), 1902 (Stephani 1902a).

** Plagiochila subedentata Steph., Bull. Herb. Boissier (sér. 2) 2 (8): 679 (216), 1902 (Stephani 1902a).

** Plagiochila subfragilis Inoue, Stud. Cryptog. S. Peru: 98, 1987 (Inoue 1987c).

* Plagiochila subhyalina Steph., Biblioth. Bot. 87 (2): 212, 1916 (Stephani 1916a).

** Plagiochila subligulata Steph., Sp. Hepat. (Stephani) 6: 224, 1921 (Stephani 1921).

** Plagiochila sublyallii M.L.So, New Zealand J. Bot. 39 (1): 109, 2001 (So 2001b).

* Plagiochila subrara Herzog, Hedwigia 74 (2): 87, 1934 (Herzog 1934a).

* Plagiochila subrotundifolia Steph., Bull. Herb. Boissier (sér. 2) 5 (10): 935 (585), 1905 (Stephani 1905f).

** Plagiochila subundulata Lindenb., Sp. Hepat. (Lindenberg) 5: 137, 1843 (Lindenberg 1843).

* Plagiochila suringarii Steph., Sp. Hepat. (Stephani) 6: 208, 1921 (Stephani 1921).

** Plagiochila sylvicultrix Spruce, Trans. & Proc. Bot. Soc. Edinburgh 15: 486, 1885 (Spruce 1885).

* Plagiochila symmetrica Steph., Bull. Herb. Boissier (sér. 2) 2 (10): 876 (245), 1902 (Stephani 1902g).

** Plagiochila tabinana Gottsche, Mexik. Leverm.: 46, 1863 (Gottsche 1863).

** Plagiochila talinayi S.W.Arnell, Svensk Bot. Tidskr. 50 (2): 312, 1956 (Arnell 1956d).

** Plagiochila tambillensis Loitl., Diagn. pl. nov.: 22, 1894 (Szyszylowicz 1894).

** Plagiochila tarapotensis Steph., Bull. Herb. Boissier (sér. 2) 2 (10): 863 (232), 1902 (Stephani 1902g).

* Plagiochila tenera Steph., Sp. Hepat. (Stephani) 6: 231, 1921 (Stephani 1921).

** Plagiochila tenuis Lindenb., Sp. Hepat. (Lindenberg) 2-4: 50, 1840 (Lindenberg 1840).

* Plagiochila tenuispica Steph., Sp. Hepat. (Stephani) 6: 234, 1921 (Stephani 1921).

** Plagiochila thamniopsis Spruce, Bull. Soc. Bot. France (Congr. Bot.) 36: cc, 1889 [1890] (Spruce 1889).

* Plagiochila thollonii Steph., Bull. Herb. Boissier (sér. 2) 2 (12): 980 (270), 1902 (Stephani 1902h).

** Plagiochila thrausta Inoue et Grolle, Bull. Natl. Sci. Mus. Tokyo, B 5 (1): 34, 1979 (Inoue 1979c).

* Plagiochila tonduzana Steph., Sp. Hepat. (Stephani) 6: 227, 1921 (Stephani 1921).

** Plagiochila tortuosa Lindenb. et Gottsche, Mexik. Leverm.: 70, 1863 (Gottsche 1863).

* Plagiochila tovarina Gottsche, Ann. Sci. Nat. Bot. (sér. 5) 1: 102, 1864 (Gottsche 1864).

* Plagiochila trabeculatospinosa Herzog, Hedwigia 72 (6): 213, 1932 (Herzog 1932c).

* Plagiochila trabutii Steph., Biblioth. Bot. 87 (2): 213, 1916 (Stephani 1916a).

** Plagiochila trianae Gottsche, Ann. Sci. Nat. Bot. (sér. 5) 1: 114, 1864 (Gottsche 1864).

** Plagiochila trichomanes Spruce, Bull. Soc. Bot. France (Congr. Bot.) 36: cc, 1889 [1890] (Spruce 1889).

* Plagiochila tricuspis Steph., Sp. Hepat. (Stephani) 6: 229, 1921 (Stephani 1921).

** Plagiochila tristaniana Váňa et J.J.Engel, Mem. New York Bot. Gard. 105: 89, 2013 (Váňa and Engel 2013). *Nom. nov. pro Plagiochila fragilifolia* S.W.Arnell, Results Norweg. Sci. Exped. Tristan da Cunha 42: 25, 1958 (Arnell 1958b), *nom. illeg*.

** Plagiochila tristis Steph., Bull. Herb. Boissier (sér. 2) 2 (10): 880 (249), 1902 (Stephani 1902g).

** Plagiochila trollii Herzog, Hedwigia 74 (2): 90, 1934 (Herzog 1934a).

** Plagiochila truncata Gottsche, Mexik. Leverm.: 25, 1863 (Gottsche 1863).

** Plagiochila uleana Steph., Bull. Herb. Boissier (sér. 2) 2 (10): 868 (237), 1902 (Stephani 1902g).

** Plagiochila umbrosioides L.Söderstr., Phytotaxa 208 (1): 85, 2015 (Söderström et al. 2015b). *Nom. nov. pro Plagiochila umbrosa* Steph., Sp. Hepat. (Stephani) 6: 235, 1921 (Stephani 1921), *nom. illeg*.

* Plagiochila unduavensis Steph., Bull. Herb. Boissier (sér. 2) 2 (10): 882 (251), 1902 (Stephani 1902g).

* Plagiochila usambarana Steph., Bull. Herb. Boissier (sér. 2) 2 (12): 980 (270), 1902 (Stephani 1902h).

** Plagiochila variedentata Steph., Bull. Herb. Boissier (sér. 2) 2 (10): 887 (256), 1902 (Stephani 1902g).

** Plagiochila vastifolia Steph., Bull. Herb. Boissier (sér. 2) 2 (8): 680 (217), 1902 (Stephani 1902a).

** Plagiochila velata Inoue et Piippo, Ann. Bot. Fenn. 26 (2): 207, 1989 (Piippo 1989b).

* Plagiochila venezuelana Steph., Bull. Herb. Boissier (sér. 2) 5 (8): 749 (548), 1905 (Stephani 1905i).

* Plagiochila ventricosotrigona Steph., Biblioth. Bot. 87 (2): 214, 1916 (Stephani 1916a).

* Plagiochila verrucosa Steph., Bull. Herb. Boissier (sér. 2) 5 (9): 885 (551), 1905 (Stephani 1905g).

* Plagiochila vetustisilva Steph., Sp. Hepat. (Stephani) 6: 245, 1922 (Stephani 1922). ^182^

* Plagiochila viminea Spruce, Mem. Torrey Bot. Club 1 (3): 134, 1890 (Spruce 1890).

* Plagiochila viridis Steph., Sp. Hepat. (Stephani) 6: 239, 1921 (Stephani 1921).

** Plagiochila viridonigra (E.A.Hodgs.) Inoue, Bryologist 68 (2): 218, 1965 (Inoue 1965b). Bas.: Syzygiella viridonigra E.A.Hodgs., Rec. Domin. Mus. 4 (11): 120, 1962 (Hodgson 1962a).

** Plagiochila vittiana Inoue, Beih. Nova Hedwigia 90: 171, 1988 (Inoue 1988a).

** Plagiochila vittifolia Steph., Sp. Hepat. (Stephani) 6: 239, 1921 (Stephani 1921).

** Plagiochila vulcanica Steph., Bull. Herb. Boissier (sér. 2) 2 (8): 671 (208), 1902 (Stephani 1902a).

** Plagiochila wacei S.W.Arnell ex Váňa et J.J.Engel, Mem. New York Bot. Gard. 105: 89, 2013 (Váňa and Engel 2013).

* Plagiochila wallisiana Steph., Bull. Herb. Boissier (sér. 2) 5 (10): 936 (586), 1905 (Stephani 1905f).

** Plagiochila wattsiana J.J.Engel et G.L.Merr., Nova Hedwigia 91 (3/4): 511, 2010 (Engel and Smith Merrill 2010). *Nom. nov. pro Plagiochila wattsii* Steph., Sp. Hepat. (Stephani) 6: 240, 1921 (Stephani 1921), *nom. illeg*.

** Plagiochila wettsteiniana S.W.Arnell, Österr. Akad. Wiss., Math.-Naturwiss. Kl., Denkschr. 111: 53, 1964 (Schiffner and Arnell 1964).

* Plagiochila weymouthiana Steph., Biblioth. Bot. 87 (2): 215, 1916 (Stephani 1916a).

** Plagiochila wiemanniana S.W.Arnell, Österr. Akad. Wiss., Math.-Naturwiss. Kl., Denkschr. 111: 54, 1964 (Schiffner and Arnell 1964).

*** Plagiochila winteri Steph., Bull. Herb. Boissier (sér. 2) 2 (12): 981 (271), 1902 (Stephani 1902h).

** Plagiochila wrightii Steph., Bull. Herb. Boissier (sér. 2) 2 (8): 681 (218), 1902 (Stephani 1902a).

** Plagiochila xalapensis Gottsche, Mexik. Leverm.: 21, 1863 (Gottsche 1863).

* Plagiochila xanthochroma Spruce, Trans. & Proc. Bot. Soc. Edinburgh 15: 489, 1885 (Spruce 1885).

* Plagiochila yoshinagana Steph., Sp. Hepat. (Stephani) 6: 242, 1922 (Stephani 1922).

** Plagiochila zacuapana Gottsche, Mexik. Leverm.: 20, 1863 (Gottsche 1863).

** **Plagiochilidium Herzog**, Mitt. Inst. Allg. Bot. Hamburg 7 (3): 186, 1931 (Herzog 1931a).

** Plagiochilidium bidentulum (Steph.) Grolle, J. Hattori Bot. Lab. 65: 408, 1988 (Grolle 1988c). Bas.: Tylimanthus bidentulus Steph., Bull. Herb. Boissier (sér. 2) 5 (12): 1134 (6), 1905 (Stephani 1905b).

** **Plagiochilion S.Hatt.**, Biosphaera 1 (1): 7, 1947 (Hattori 1947a).

*** Plagiochilion braunianum (Nees) S.Hatt., Biosphaera 1 (1): 7, 1947 (Hattori 1947a). Bas.: Jungermannia brauniana Nees, Enum. Pl. Crypt. Javae: 80, 1830 (Nees 1830).

** Plagiochilion combinatum (Mitt.) Inoue, J. Hattori Bot. Lab. 27: 55, 1964 (Inoue 1964b). Bas.: Plagiochila combinata Mitt., Fl. vit.: 408, 1871 [1873] (Mitten 1871).

*** Plagiochilion conjugatum (Hook.) R.M.Schust., J. Hattori Bot. Lab. 26: 285, 1963 (Schuster 1963b). Bas.: Jungermannia conjugata Hook., Musci Exot. 1: tab. 91, 1818 (Hooker 1818).

** Plagiochilion fimbriatum (Mitt.) Inoue, J. Hattori Bot. Lab. 27: 57, 1964 (Inoue 1964b). Bas.: Plagiochila fimbriata Mitt., J. Proc. Linn. Soc., Bot. 5 (18): 97, 1860 [1861] (Mitten 1860c).

** Plagiochilion giulianettii (Steph.) Inoue, J. Hattori Bot. Lab. 27: 57, 1964 (Inoue 1964b). Bas.: Plagiochila giulianettii Steph., Bull. Herb. Boissier (sér. 2) 4 (1): 30 (402), 1904 (Stephani 1904g).

** Plagiochilion herzogii Inoue, Bull. Natl. Sci. Mus. Tokyo (n.ser.) 14 (2): 270, 1971 (Inoue 1971c).

** Plagiochilion intermedium R.M.Schust., Phytologia 45 (5): 421, 1980 (Schuster 1980b).

** Plagiochilion mayebarae S.Hatt., J. Hattori Bot. Lab. 3: 39, 1948 [1950] (Hattori 1948a).

*** Plagiochilion oppositum (Reinw., Blume et Nees) S.Hatt., Biosphaera 1 (1): 7, 1947 (Hattori 1947a). Bas.: Jungermannia opposita Reinw., Blume et Nees, Nova Acta Phys.-Med. Acad. Caes. Leop.-Carol. Nat. Cur. 12 (1): 236, 1824 [1825] (Reinwardt et al. 1824a).

** Plagiochilion pachycephalum (De Not.) Inoue, J. Hattori Bot. Lab. 27: 55, 1964 (Inoue 1964b). Bas.: Plagiochila pachycephala De Not., Epat. Borneo: 14, 1874 (De Notaris 1874).

*** Plagiochilion proliferum (Mitt.) R.M.Schust., J. Hattori Bot. Lab. 26: 285, 1963 (Schuster 1963b). Bas.: Plagiochila prolifera Mitt., Bot. antarct. voy. II (Fl. Nov.-Zel. 2): 130, 1854 (Mitten 1854).

** Plagiochilion theriotanum (Steph.) Inoue, J. Hattori Bot. Lab. 27: 59, 1964 (Inoue 1964b). Bas.: Plagiochila theriotana Steph., Sp. Hepat. (Stephani) 6: 228, 1921 (Stephani 1921).

** **Pseudolophocolea R.M.Schust. et J.J.Engel**, Lindbergia 8 (2): 71, 1982 (Schuster and Engel 1982). Based on: Pseudolophocolea R.M.Schust. et J.J.Engel, Phytologia 47 (4): 310, 1981 (Schuster and Engel 1981).

** Pseudolophocolea denticulata R.M.Schust. et J.J.Engel, Lindbergia 8 (2): 73, 1982 (Schuster and Engel 1982). Based on: Pseudolophocolea denticulata R.M.Schust. et J.J.Engel, Phytologia 47 (4): 311, 1981 (Schuster and Engel 1981), *nom. inval*.

** **Xenochila R.M.Schust.**, Amer. Midl. Naturalist 62 (1): 15, 1959 (Schuster 1959a).

** Xenochila integrifolia (Mitt.) Inoue, Bull. Natl. Sci. Mus. Tokyo (n.ser.) 6 (4): 373, 1963 (Inoue 1963). Bas.: Plagiochila integrifolia Mitt., J. Proc. Linn. Soc., Bot. 5 (18): 96, 1860 [1861] (Mitten 1860c).

######## *** Pseudolepicoleaceae Fulford et J.Taylor

by M. von Konrat

Blepharostoma was recognized as an element within Pseudolepicoleaceae by Crandall-Stotler et al. (2009), but we follow the concept of Frey and Stech (2008) with Blepharostoma as the single genus in Blepharostomataceae.

*** **Archeophylla R.M.Schust.**, J. Hattori Bot. Lab. 26: 263, 1963 (Schuster 1963b).

** Archeophylla paradoxa R.M.Schust., Trans. Brit. Bryol. Soc. 4 (5): 810, 1965 (Schuster 1965c).

** Archeophylla pungens (Herzog) R.M.Schust., Candollea 21 (1): 86, 1966 (Schuster 1966e). Bas.: Blepharostoma pungens Herzog, Rev. Bryol. Lichénol. 29 (3/4): 189, 1960 [1961] (Herzog 1960).

*** Archeophylla schusteri (E.A.Hodgs. et Allison) R.M.Schust., J. Hattori Bot. Lab. 26: 263, 1963 (Schuster 1963b). Bas.: Temnoma schusteri E.A.Hodgs. et Allison, Trans. Roy. Soc. New Zealand, Bot. 1 (12): 147, 1962 (Hodgson and Allison 1962).

*** **Castanoclobos J.J.Engel et Glenny**, Novon 17 (4): 424, 2007 (Engel and Glenny 2007).

*** Castanoclobos julaceus (Hatcher ex J.J.Engel) J.J.Engel et Glenny, Novon 17 (4): 425, 2007 (Engel and Glenny 2007). Bas.: Leiomitra julacea Hatcher ex J.J.Engel, Novon 9 (1): 26, 1999 (Engel 1999a).

** **Chaetocolea Spruce**, Trans. & Proc. Bot. Soc. Edinburgh 15: 346, 1885 (Spruce 1885).

*** Chaetocolea palmata Spruce, Trans. & Proc. Bot. Soc. Edinburgh 15: 346, 1885 (Spruce 1885).

*** **Herzogiaria Fulford ex Hässel**, Lindbergia 7 (1): 23, 1981 (Hässel 1981).

*** Herzogiaria teres (Steph.) Fulford ex Hässel, Lindbergia 7 (1): 24, 1981 (Hässel 1981). Bas.: Lepicolea teres Steph., Bih. Kongl. Svenska Vetensk.-Akad. Handl. 26 (III, 17): 26, 1901 (Stephani 1901b).

** **Isophyllaria E.A.Hodgs. et Allison**, Trans. Roy. Soc. New Zealand, Bot. 3 (4): 68, 1965 (Hodgson 1965).

*** Isophyllaria attenuata (Rodway) E.A.Hodgs., J. Roy. Soc. New Zealand 2 (1): 111, 1972 (Hodgson 1972). Bas.: Isotachis attenuata Rodway, Pap. & Proc. Roy. Soc. Tasmania 1916: 47, 1917 (Rodway 1917a).

** Isophyllaria fuegiana (Hässel) R.M.Schust., Beih. Nova Hedwigia 118: 141, 2000 (Schuster 2000a). Bas.: Fulfordiella fuegiana Hässel, Comun. Mus. Argent. Ci. Nat. “Bernardino Rivadavia,” Ci. Bot. 2 (9): 48, 1974 (Hässel 1974).

*** **Pseudolepicolea Fulford et J.Taylor**, Nova Hedwigia 1 (3/4): 412, 1959 [1960] (Fulford and Taylor 1959b).

** Pseudolepicolea andoi (R.M.Schust.) Inoue, Bull. Natl. Sci. Mus. Tokyo, B 4 (3): 94, 1978 (Inoue 1978a). Bas.: Lophochaete andoi R.M.Schust., J. Hattori Bot. Lab. 26: 261, 1963 (Schuster 1963b).

** Pseudolepicolea fryei (Perss.) Grolle et Ando, Hikobia 3 (3): 180, 1963 (Ando 1963). Bas.: Lepicolea fryei Perss., Bryologist 49 (2): 47, 1946 (Persson 1946).

*** Pseudolepicolea grolleana (R.M.Schust.) Grolle, Ann. Bot. Fenn. 21 (1): 30, 1984 (Piippo 1984a). Bas.: Archeochaete grolleana R.M.Schust., Nova Hedwigia 15: 441, 1968 (Schuster 1968b).

*** Pseudolepicolea kuehnemannii (R.M.Schust.) Hässel, Fl. Criptog. Tierra del Fuego 15: 125, 1975 (Hässel and Solari 1975). Bas.: Archeochaete kuehnemannii R.M.Schust., J. Hattori Bot. Lab. 26: 262, 1963 (Schuster 1963b).

*** Pseudolepicolea quadrilaciniata (Sull.) Fulford et J.Taylor, Nova Hedwigia 1 (3/4): 413, 1959 [1960] (Fulford and Taylor 1959b). Bas.: Sendtnera quadrilaciniata Sull., Hooker’s J. Bot. Kew Gard. Misc. 2: 317, 1850 (Sullivant 1850).

*** Pseudolepicolea temnomoides (R.M.Schust.) Váňa et J.J.Engel, Mem. New York Bot. Gard. 105: 92, 2013 (Váňa and Engel 2013). Bas.: Archeochaete temnomoides R.M.Schust., Candollea 21 (1): 129, 1966 (Schuster 1966e).

** Pseudolepicolea trollii (Herzog) Grolle et Ando, Hikobia 3 (3): 177, 1963 (Ando 1963). Bas.: Blepharostoma trollii Herzog, Ann. Bryol. 12: 80, 1939 (Herzog 1939b).

* Pseudolepicolea trollii var. darjeelingensis S.Hatt. et Mizut., J. Hattori Bot. Lab. 31: 252, 1968 (Hattori and Mizutani 1968).

*** **Temnoma Mitt.**, Handb. N. Zeal. fl. 2: 750, 1867 (Hooker 1867).

*** Temnoma angustifolium R.M.Schust., Candollea 21 (2): 279, 1966 [1967] (Schuster 1966c).

*** Temnoma chaetophyllum R.M.Schust., Phytologia 39 (4): 239, 1978 (Schuster 1978a).

** Temnoma palmatum (Lindb. ex Pearson) R.M.Schust., Bryologist 62 (4): 240, 1959 [1960] (Schuster 1959c). Bas.: Blepharostoma palmatum Lindb. ex Pearson, J. Bot. 25: 193, 1887 (Pearson 1887a).

** Temnoma palmatum var. cuneatum R.M.Schust., Candollea 21 (2): 347, 1966 [1967] (Schuster 1966c).

** Temnoma palmatum var. laxifolium R.M.Schust., Candollea 21 (2): 345, 1966 [1967] (Schuster 1966c).

** Temnoma palmatum var. pseudospiniferum R.M.Schust., Candollea 21 (2): 348, 1966 [1967] (Schuster 1966c).

** Temnoma patagonicum R.M.Schust., Candollea 21 (2): 313, 1966 [1967] (Schuster 1966c).

*** Temnoma paucisetigerum R.M.Schust., Candollea 21 (2): 266, 1966 [1967] (Schuster 1966c).

*** Temnoma pilosum (A.Evans) R.M.Schust., Bryologist 62 (4): 240, 1959 [1960] (Schuster 1959c). Bas.: Blepharostoma pilosum A.Evans, Bull. Torrey Bot. Club 25 (8): 413, 1898 (Evans 1898).

*** Temnoma pulchellum (Hook.) Mitt., Handb. N. Zeal. fl. 2: 753, 1867 (Hooker 1867). Bas.: Jungermannia pulchella Hook., Musci Exot. 1: tab. 94, 1818 (Hooker 1818).

*** Temnoma quadrifidum (Mitt.) Mitt. ex E.A.Hodgs. et Allison, Trans. Roy. Soc. New Zealand, Bot. 1 (12): 142, 1962 (Hodgson and Allison 1962). Bas.: Jungermannia quadrifida Mitt., Bot. antarct. voy. II (Fl. Nov.-Zel. 2): 128, 1854 (Mitten 1854).

*** Temnoma quadripartitum (Hook.) Mitt., J. Linn. Soc., Bot. 15 (82): 68, 1876 (Mitten 1876a). Bas.: Jungermannia quadripartita Hook., Musci Exot. 2: tab. 117, 1820 (Hooker 1820).

** Temnoma quadripartitum var. pseudopungens R.M.Schust., Candollea 21 (2): 312, 1966 [1967] (Schuster 1966c).

** Temnoma quadripartitum var. randii (S.W.Arnell) R.M.Schust., Candollea 21 (2): 307, 1966 [1967] (Schuster 1966c). Bas.: Lepidozia randii S.W.Arnell, Svensk Bot. Tidskr. 47 (3): 417, 1953 (Arnell 1953c).

*** Temnoma setigerum (Lindenb.) R.M.Schust., Nova Hedwigia 5: 35, 1963 (Schuster 1963c). Bas.: Jungermannia setigera Lindenb., Syn. Hepat. 1: 131, 1844 (Gottsche et al. 1844).

** Temnoma setigerum var. hawaiicum Inoue, Bull. Natl. Sci. Mus. Tokyo (n.ser.) 17 (3): 228, 1974 (Inoue 1974b).

** Temnoma townrowii R.M.Schust., Candollea 21 (2): 351, 1966 [1967] (Schuster 1966c).

######## *** Trichocoleaceae Nakai

by T. Katagiri

The treatment of Trichocoleaceae follows what was outlined in Katagiri and Deguchi (2012) and Katagiri et al. (2012, 2013). Recent nomenclatural and taxonomic notes can also be found in Katagiri (2013).

*** **Eotrichocolea R.M.Schust.**, J. Hattori Bot. Lab. 26: 252, 1963 (Schuster 1963b).

*** Eotrichocolea furukii T.Katag., Bryologist 115 (4): 519, 2012 (Katagiri et al. 2012).

*** Eotrichocolea polyacantha (Hook.f. et Taylor) R.M.Schust., J. Hattori Bot. Lab. 26: 264, 1963 (Schuster 1963b). Bas.: Jungermannia polyacantha Hook.f. et Taylor, London J. Bot. 3: 290 [390], 1844 (Hooker and Taylor 1844a).

*** **Leiomitra Lindb.**, Acta Soc. Sci. Fenn. 10: 515, 1875 (Lindberg 1875).

** **subg. Brachygyna R.M.Schust.**, Nova Hedwigia 73 (3/4): 480, 2001 (Schuster 2001b).

** Leiomitra mastigophoroides R.M.Schust., Phytologia 45 (5): 416, 1980 (Schuster 1980b).

** **subg. Leiomitra**, Nova Hedwigia 73 (3/4): 480, 2001 (Schuster 2001b).

*** Leiomitra breviseta (Steph.) R.M.Schust., Beih. Nova Hedwigia 118: 152, 2000 (Schuster 2000a). Bas.: Trichocolea breviseta Steph., Sp. Hepat. (Stephani) 4: 60, 1909 (Stephani 1909d).

*** Leiomitra capillata Lindb., Acta Soc. Sci. Fenn. 10: 515, 1875 (Lindberg 1875).

*** Leiomitra elegans (Lehm.) Hässel, Novon 12 (4): 465, 2002 (Hässel 2002). Bas.: Trichocolea elegans Lehm., Nov. Stirp. Pug. 10: 8, 1857 (Lehmann 1857).

** Leiomitra elliottii (Steph.) R.M.Schust., Nova Hedwigia 73 (3/4): 469, 2001 (Schuster 2001b). Bas.: Trichocolea elliottii Steph., Sp. Hepat. (Stephani) 4: 55, 1909 (Stephani 1909d).

*** Leiomitra flaccida Spruce, Trans. & Proc. Bot. Soc. Edinburgh 15: 349, 1885 (Spruce 1885).

** Leiomitra hirticaulis R.M.Schust., Nova Hedwigia 73 (3/4): 472, 2001 (Schuster 2001b).

*** Leiomitra lanata (Hook.) R.M.Schust., Phytologia 45 (5): 417, 1980 (Schuster 1980b). Bas.: Jungermannia lanata Hook., Musci Exot. 2: tab. 116, 1820 (Hooker 1820).

*** Leiomitra merrillana (Steph.) T.Katag., Bryologist 115 (4): 488, 2012 (Katagiri and Deguchi 2012). Bas.: Trichocolea merrillana Steph., Sp. Hepat. (Stephani) 6: 374, 1923 (Stephani 1923).

*** Leiomitra paraphyllina Spruce, Trans. & Proc. Bot. Soc. Edinburgh 15: 350, 1885 (Spruce 1885).

** Leiomitra robusta (Steph.) R.M.Schust., Nova Hedwigia 73 (3/4): 469, 2001 (Schuster 2001b). Bas.: Trichocolea robusta Steph., Sp. Hepat. (Stephani) 4: 58, 1909 (Stephani 1909d).

** Leiomitra smaragdina Hässel, Novon 12 (4): 467, 2002 (Hässel 2002).

*** Leiomitra tomentosa (Sw.) Lindb., Acta Soc. Sci. Fenn. 10: 515, 1875 (Lindberg 1875). Bas.: Jungermannia tomentosa Sw., Prodr. (Swartz): 145, 1788 (Swartz 1788).

*** **Trichocolea Dumort.**, Commentat. Bot. (Dumortier): 113, 1822 (Dumortier 1822) nom. conserv.

** Trichocolea argentea Herzog, Arch. Bot. São Paulo 1 (2): 40, 1925 (Herzog 1925b).

*** Trichocolea brevifissa Steph., Sp. Hepat. (Stephani) 4: 54, 1909 (Stephani 1909d).

** Trichocolea comptonii Pearson, J. Linn. Soc., Bot. 46 (305): 27, 1922 (Pearson 1922b).

*** Trichocolea filicaulis Steph., Sp. Hepat. (Stephani) 4: 59, 1909 (Stephani 1909d).

* Trichocolea floccosa Herzog et Hatcher, Lloydia 20 (3): 148, 1957 [1958] (Hatcher 1957). ^183^

** Trichocolea geniculata Pearson, J. Linn. Soc., Bot. 46 (305): 28, 1922 (Pearson 1922b).

** Trichocolea gracillima Austin, Bot. Gaz. 3 (1): 6, 1878 (Austin 1878).

*** Trichocolea hatcheri E.A.Hodgs., Trans. Roy. Soc. New Zealand, Bot. 3 (4): 69, 1965 (Hodgson 1965).

*** Trichocolea iriomotensis T.Katag., Hattoria 4: 6, 2013 (Katagiri et al. 2013).

*** Trichocolea japonica T.Katag., Bryologist 114 (4): 744, 2011 (Katagiri et al. 2011).

*** Trichocolea magna T.Katag., Hattoria 4: 7, 2013 (Katagiri et al. 2013).

** Trichocolea minutifolia Steph., J. & Proc. Roy. Soc. New South Wales 48 (1/2): 134, 1914 (Stephani and Watts 1914).

*** Trichocolea mollissima (Hook.f. et Taylor) Gottsche, Ann. Sci. Nat. Bot. (sér. 5) 1: 132, 1864 (Gottsche 1864). Bas.: Jungermannia mollissima Hook.f. et Taylor, London J. Bot. 3: 290 [390], 1844 (Hooker and Taylor 1844a).

*** Trichocolea pluma (Reinw., Blume et Nees) Mont., Voy. Bonite, Bot. 1: 238, 1846 (Montagne 1846). Bas.: Jungermannia pluma Reinw., Blume et Nees, Nova Acta Phys.-Med. Acad. Caes. Leop.-Carol. Nat. Cur. 12 (1): 209, 1824 [1825] (Reinwardt et al. 1824a).

*** Trichocolea rigida R.M.Schust., Nova Hedwigia 15: 447, 1968 (Schuster 1968b).

*** Trichocolea rudimentaris Steph., Sp. Hepat. (Stephani) 6: 376, 1923 (Stephani 1923).

* Trichocolea sprucei Steph., Sp. Hepat. (Stephani) 4: 59, 1909 (Stephani 1909d). *Nom. nov. pro Trichocolea gracillima* Spruce, J. Linn. Soc., Bot. 30 (210): 353, 1895 (Gepp 1895b), *nom. illeg*. ^184^

*** Trichocolea tomentella (Ehrh.) Dumort., Syll. Jungerm. Europ.: 67, 1831 (Dumortier 1831). Bas.: Jungermannia tomentella Ehrh., Hannover. Mag. 21 (18): 277, 1783 (Ehrhart 1783).

** Trichocolea udarii D.K.Singh, Bull. Bot. Surv. India 25: 177, 1983 [1985] (Singh 1983a).

** Trichocolea wattsiana Steph., J. & Proc. Roy. Soc. New South Wales 48 (1/2): 135, 1914 (Stephani and Watts 1914).

####### Myliineae J.J.Engel et Braggins ex Crand.-Stotl., Váňa, Stotler et J.J.Engel

######## *** Myliaceae Schljakov

by L. Söderström

Söderström et al. (2015c) outlines the controversy as to the placement of Mylia anomala where they advocate recognition at the subgeneric level. It has previously been recognized as a segregate genus, Leiomylia J.J.Engel and Braggins (cf. also Shaw et al. 2015).

*** **Mylia Gray**, Nat. Arr. Brit. Pl. 1: 693, 1821 (Gray 1821) nom. conserv.

** **subg. Anomalae (R.M.Schust. ex Potemkin) L.Söderstr.**, Phytotaxa 202 (1): 70, 2015 (Söderström et al. 2015c). Bas.: Mylia sect. Anomalae R.M.Schust. ex Potemkin, Arctoa 2: 1, 1993 (Potemkin and Kazanovsky 1993).

*** Mylia anomala (Hook.) Gray, Nat. Arr. Brit. Pl. 1: 693, 1821 (Gray 1821). Bas.: Jungermannia anomala Hook., Brit. Jungermann.: tab. 34, 1813 (Hooker 1813).

** **subg. Mylia**

*** Mylia taylorii (Hook.) Gray, Nat. Arr. Brit. Pl. 1: 693, 1821 (Gray 1821). Bas.: Jungermannia taylorii Hook., Brit. Jungermann.: tab. 57, 1813 (Hooker 1813).

*** Mylia verrucosa Lindb., Acta Soc. Sci. Fenn. 10: 236, 1872 [1873] (Lindberg 1872b).

*** Mylia verrucosa subsp. nuda (Inoue et B.Y.Yang) Potemkin et Kazan., Arctoa 2: 5, 1993 (Potemkin and Kazanovsky 1993). Bas.: Mylia nuda Inoue et B.Y.Yang, Taiwania 12 (1): 35, 1966 (Inoue and Yang 1966).

####### Perssoniellineae R.M.Schust.

######## *** Schistochilaceae H.Buch

by X. He and D. Glenny

He et al. (2014b) provided an historical account of Schistochilaceae summarizing studies that have showed that the phylogenetic structure of the family does not match units that have resulted from morphologically-based investigations. Further studies are needed until a natural division of the family can be proposed. Thus, we follow the broad concept of He and Glenny (2010) and He and Sun (2013) here, treating Schistochilaceae as comprising a single genus as discussed by He et al. (2014b).

*** **Schistochila Dumort.**, Recueil Observ. Jungerm.: 15, 1835 (Dumortier 1835).

** Schistochila acuminata Steph., Sp. Hepat. (Stephani) 4: 81, 1909 (Stephani 1909d).

*** Schistochila aequiloba Steph., Sp. Hepat. (Stephani) 4: 80, 1909 (Stephani 1909d).

*** Schistochila alata (Lehm.) Schiffn., Hepat. (Engl.-Prantl): 111, 1893 (Schiffner 1893b). Bas.: Jungermannia alata Lehm., Linnaea 4: 359, 1829 (Lehmann 1829).

*** Schistochila aligera (Nees et Blume) J.B.Jack et Steph., Hedwigia 31 (1): 12, 1892 (Jack and Stephani 1892). Bas.: Jungermannia aligera Nees et Blume, Nova Acta Phys.-Med. Acad. Caes. Leop.-Carol. Nat. Cur. 11: 135, 1823 (Blume and Nees 1823).

* Schistochila aligera var. laxa (Nees) Schiffn., Consp. Hepat. Arch. Ind.: 212, 1898 (Schiffner 1898b). Bas.: Jungermannia aligera γ laxa Nees, Enum. Pl. Crypt. Javae: 68, 1830 (Nees 1830).

*** Schistochila altissima E.A.Hodgs., Trans. Roy. Soc. New Zealand, Bot. 3 (4): 85, 1965 (Hodgson 1965).

** Schistochila altissima subsp. polystratosa R.M.Schust. et J.J.Engel, Phytologia 30 (4): 241, 1975 (Schuster and Engel 1975).

*** Schistochila antara Grolle, J. Hattori Bot. Lab. 29: 249, 1966 (Grolle 1966h).

*** Schistochila appendiculata (Hook.) Dumort. ex Trevis., Mem. Reale Ist. Lombardo Sci. (Ser. 3), C. Sci. Mat. 4 (13): 392, 1877 (Trevisan 1877). Bas.: Jungermannia appendiculata Hook., Musci Exot. 1: tab. 15, 1818 (Hooker 1818).

* Schistochila baileyana Steph., Sp. Hepat. (Stephani) 4: 85, 1909 (Stephani 1909d). ^185^

*** Schistochila balfouriana (Hook.f. et Taylor) Steph., Sp. Hepat. (Stephani) 4: 91, 1909 (Stephani 1909d). Bas.: Jungermannia balfouriana Hook.f. et Taylor, London J. Bot. 3: 556, 1844 (Hooker and Taylor 1844d).

*** Schistochila beccariana (De Not.) Trevis., Mem. Reale Ist. Lombardo Sci. (Ser. 3), C. Sci. Mat. 4 (13): 392, 1877 (Trevisan 1877). Bas.: Gottschea beccariana De Not., Epat. Borneo: 9, 1874 (De Notaris 1874).

*** Schistochila berggrenii (J.J.Engel et R.M.Schust.) Xiao L.He et Glenny, Austral. Syst. Bot. 23 (4): 237, 2010 (He and Glenny 2010). Bas.: Pachyschistochila berggrenii J.J.Engel et R.M.Schust., J. Hattori Bot. Lab. 58: 476, 1985 (Schuster and Engel 1985).

*** Schistochila berteroana (Hook.) Steph., Sp. Hepat. (Stephani) 4: 96, 1909 (Stephani 1909d). Bas.: Jungermannia berteroana Hook., Bot. Misc. 2: 148, 1831 (Hooker 1831).

*** Schistochila blumei (Nees) Trevis., Mem. Reale Ist. Lombardo Sci. (Ser. 3), C. Sci. Mat. 4 (13): 392, 1877 (Trevisan 1877). Bas.: Jungermannia blumei Nees, Nova Acta Phys.-Med. Acad. Caes. Leop.-Carol. Nat. Cur. 11: 136, 1823 (Blume and Nees 1823).

** Schistochila brassii Grolle, J. Hattori Bot. Lab. 31: 5, 1968 (Grolle 1968a).

** Schistochila caledonica Steph., Sp. Hepat. (Stephani) 4: 77, 1909 (Stephani 1909d).

*** Schistochila carnosa (Mitt.) Steph., Sp. Hepat. (Stephani) 4: 93, 1909 (Stephani 1909d). Bas.: Gottschea carnosa Mitt., J. Linn. Soc., Bot. 15 (82): 72, 1876 (Mitten 1876a).

*** Schistochila caudata R.M.Schust. et J.J.Engel, Phytologia 30 (4): 242, 1975 (Schuster and Engel 1975).

*** Schistochila childii (R.M.Schust. et J.J.Engel) Xiao L.He et Glenny, Austral. Syst. Bot. 23 (4): 237, 2010 (He and Glenny 2010). Bas.: Pachyschistochila childii R.M.Schust. et J.J.Engel, J. Hattori Bot. Lab. 58: 471, 1985 (Schuster and Engel 1985).

*** Schistochila chlorophylla (Colenso) E.A.Hodgs., Trans. & Proc. Roy. Soc. New Zealand 71 (3): 192, 1941 (Hodgson 1941). Bas.: Gottschea chlorophylla Colenso, Trans. & Proc. New Zealand Inst. 18: 240, 1886 (Colenso 1886b).

*** Schistochila ciliata (Mitt.) Steph., Sp. Hepat. (Stephani) 4: 87, 1909 (Stephani 1909d). Bas.: Gottschea ciliata Mitt., Bot. antarct. voy. II (Fl. Nov.-Zel. 2): 151, 1854 (Mitten 1854).

*** Schistochila compacta (Colenso) E.A.Hodgs., Trans. & Proc. Roy. Soc. New Zealand 71 (3): 192, 1941 (Hodgson 1941). Bas.: Gottschea compacta Colenso, Trans. & Proc. New Zealand Inst. 16: 349, 1884 (Colenso 1884).

*** Schistochila conchophylla Herzog ex E.A.Hodgs. et Allison, Trans. & Proc. Roy. Soc. New Zealand 71 (3): 191, 1941 (Hodgson 1941).

** Schistochila conchophylla var. multidentata (J.J.Engel) Xiao L.He et Glenny, Phytotaxa 173 (1): 92, 2014 (He et al. 2014b). Bas.: Gottschea conchophylla var. multidentata J.J.Engel, Nova Hedwigia 93 (3/4): 407, 2011 (Engel 2011).

*** Schistochila congoana Steph., Sp. Hepat. (Stephani) 4: 71, 1909 (Stephani 1909d).

** Schistochila cookei (H.A.Mill.) R.M.Schust., J. Hattori Bot. Lab. 42: 274, 1977 (Schuster and Engel 1977). Bas.: Fulfordistria cookei H.A.Mill., Phytologia 20 (5): 320, 1970 (Miller 1970).

** Schistochila crinita Grolle, J. Hattori Bot. Lab. 31: 7, 1968 (Grolle 1968a).

*** Schistochila cristata Steph., Hedwigia 28 (4): 274, 1889 (Stephani 1889c).

*** Schistochila cunninghamii Steph., Bih. Kongl. Svenska Vetensk.-Akad. Handl. 26 (III, 17): 27, 1901 (Stephani 1901b).

** Schistochila doriae (De Not.) Trevis., Mem. Reale Ist. Lombardo Sci. (Ser. 3), C. Sci. Mat. 4 (13): 392, 1877 (Trevisan 1877). Bas.: Gottschea doriae De Not., Epat. Borneo: 10, 1874 (De Notaris 1874).

** Schistochila engleriana Steph., Sp. Hepat. (Stephani) 4: 69, 1909 (Stephani 1909d).

*** Schistochila exalata Herzog, Rev. Bryol. Lichénol. 29 (3/4): 191, 1960 [1961] (Herzog 1960).

** Schistochila fijiensis H.Buch et Herzog, Memoranda Soc. Fauna Fl. Fennica 27: 94, 1952 (Herzog 1952c).

*** Schistochila glaucescens (Hook.) A.Evans, Rev. Bryol. 32 (4): 60, 1905 (Evans 1905c). Bas.: Jungermannia glaucescens Hook., Musci Exot. 1: tab. 39, 1818 (Hooker 1818).

** Schistochila hattorii Grolle, J. Hattori Bot. Lab. 29: 247, 1966 (Grolle 1966h).

** Schistochila integerrima Steph., Sp. Hepat. (Stephani) 6: 492, 1924 (Stephani 1924).

** Schistochila isotachyphylla (J.J.Engel et R.M.Schust.) Xiao L.He et Glenny, Austral. Syst. Bot. 23 (4): 237, 2010 (He and Glenny 2010). Bas.: Paraschistochila isotachyphylla J.J.Engel et R.M.Schust., J. Hattori Bot. Lab. 58: 429, 1985 (Schuster and Engel 1985).

*** Schistochila kirkiana Steph., Sp. Hepat. (Stephani) 4: 86, 1909 (Stephani 1909d).

*** Schistochila kunkelii S.W.Arnell, Ark. Bot. (n.ser.) 4 (1): 12, 1957 (Arnell 1957b).

** Schistochila lacerata Steph., Sp. Hepat. (Stephani) 6: 492, 1924 (Stephani 1924).

*** Schistochila lamellata (Hook.) Dumort. ex A.Evans, Contr. U.S. Natl. Herb. 1 (5): 141, 1892 (Evans 1892b). Bas.: Jungermannia lamellata Hook., Musci Exot. 1: tab. 49, 1818 (Hooker 1818).

*** Schistochila laminigera (Hook.f. et Taylor) A.Evans, Contr. U.S. Natl. Herb. 1 (5): 141, 1892 (Evans 1892b). Bas.: Jungermannia laminigera Hook.f. et Taylor, London J. Bot. 3: 456, 1844 (Hooker and Taylor 1844b).

*** Schistochila latiloba (R.M.Schust. et J.J.Engel) Xiao L.He et Glenny, Austral. Syst. Bot. 23 (4): 237, 2010 (He and Glenny 2010). Bas.: Pachyschistochila latiloba R.M.Schust. et J.J.Engel, J. Hattori Bot. Lab. 58: 493, 1985 (Schuster and Engel 1985).

*** Schistochila lehmanniana (Lindenb.) Steph., Sp. Hepat. (Stephani) 4: 86, 1909 (Stephani 1909d). Bas.: Jungermannia lehmanniana Lindenb., Nov. Stirp. Pug. 4: 60, 1832 (Lehmann 1832).

*** Schistochila leucophylla (Lehm. ex Gottsche, Lindenb. et Nees) Steph., Sp. Hepat. (Stephani) 4: 98, 1910 (Stephani 1910b). Bas.: Gottschea leucophylla Lehm. ex Gottsche, Lindenb. et Nees, Syn. Hepat. 1: 17, 1844 (Gottsche et al. 1844).

** Schistochila macrodonta W.E.Nicholson, Symb. Sin. 5: 29, 1930 (Nicholson et al. 1930).

** Schistochila minor C.Gao et Y.H.Wu, J. Hattori Bot. Lab. 95: 267, 2004 (Gao and Wu 2004).

*** Schistochila monticola R.M.Schust., Bull. Natl. Sci. Mus. Tokyo (n.ser.) 14 (4): 623, 1971 (Schuster 1971c).

*** Schistochila muricata E.A.Hodgs. et Allison, Trans. & Proc. Roy. Soc. New Zealand 71 (3): 186, 1941 (Hodgson 1941).

*** Schistochila nadeaudiana Steph., Sp. Hepat. (Stephani) 4: 76, 1909 (Stephani 1909d).

** Schistochila neesii (Mont.) Lindb., J. Linn. Soc., Bot. 13 (67): 194, 1872 [1873] (Lindberg 1872a). Bas.: Gottschea neesii Mont., Ann. Sci. Nat. Bot. (sér. 2) 19: 244, 1843 (Montagne 1843).

*** Schistochila nitidissima R.M.Schust., Bull. Natl. Sci. Mus. Tokyo (n.ser.) 11 (1): 27, 1968 (Schuster 1968a).

*** Schistochila nivicola (R.M.Schust. et J.J.Engel) Xiao L.He et Glenny, Austral. Syst. Bot. 23 (4): 237, 2010 (He and Glenny 2010). Bas.: Pachyschistochila nivicola R.M.Schust. et J.J.Engel, J. Hattori Bot. Lab. 58: 512, 1985 (Schuster and Engel 1985).

*** Schistochila nobilis (Hook.) Trevis., Mem. Reale Ist. Lombardo Sci. (Ser. 3), C. Sci. Mat. 4 (13): 392, 1877 (Trevisan 1877). Bas.: Jungermannia nobilis Hook., Musci Exot. 1: tab. 11, 1818 (Hooker 1818).

*** Schistochila nuda Horik., J. Sci. Hiroshima Univ., Ser. B, Div. 2, Bot. 2: 215, 1934 (Horikawa 1934).

*** Schistochila pachyphylla (Lehm.) Steph., Sp. Hepat. (Stephani) 4: 99, 1910 (Stephani 1910b). Bas.: Jungermannia pachyphylla Lehm., Nov. Stirp. Pug. 6: 61, 1834 (Lehmann 1834).

*** Schistochila papillifera R.M.Schust., Bull. Natl. Sci. Mus. Tokyo (n.ser.) 11 (1): 27, 1968 (Schuster 1968a).

*** Schistochila parvistipula Rodway, Pap. & Proc. Roy. Soc. Tasmania 1916: 47, 1917 (Rodway 1917a).

*** Schistochila pellucida R.M.Schust. et J.J.Engel, J. Hattori Bot. Lab. 58: 286, 1985 (Schuster and Engel 1985).

*** Schistochila piligera Steph., Bot. Gaz. 15 (11): 291, 1890 (Stephani 1890c).

*** Schistochila pinnatifolia (Hook.) Trevis., Mem. Reale Ist. Lombardo Sci. (Ser. 3), C. Sci. Mat. 4 (13): 392, 1877 (Trevisan 1877). Bas.: Jungermannia pinnatifolia Hook., Musci Exot. 2: tab. 114, 1820 (Hooker 1820).

*** Schistochila pluriciliata R.M.Schust. et J.J.Engel, J. Hattori Bot. Lab. 58: 395, 1985 (Schuster and Engel 1985).

*** Schistochila pseudociliata R.M.Schust., Bull. Natl. Sci. Mus. Tokyo (n.ser.) 14 (4): 623, 1971 (Schuster 1971c).

*** Schistochila quadrifida A.Evans, Contr. U.S. Natl. Herb. 1 (5): 141, 1892 (Evans 1892b).

** Schistochila ramentacea Steph., Sp. Hepat. (Stephani) 6: 494, 1924 (Stephani 1924).

*** Schistochila reflexa (Mont.) Steph., Sp. Hepat. (Stephani) 4: 97, 1910 (Stephani 1910b). Bas.: Gottschea reflexa Mont., Ann. Sci. Nat. Bot. (sér. 3) 4: 347, 1845 (Montagne 1845b).

*** Schistochila reflexistipula J.J.Engel et R.M.Schust., Phytologia 30 (4): 245, 1975 (Schuster and Engel 1975).

*** Schistochila reinwardtii (Nees) Schiffn., Consp. Hepat. Arch. Ind.: 218, 1898 (Schiffner 1898b). Bas.: Jungermannia reinwardtii Nees, Enum. Pl. Crypt. Javae: 66, 1830 (Nees 1830).

*** Schistochila repleta (Hook.f. et Taylor) Steph., Sp. Hepat. (Stephani) 4: 90, 1909 (Stephani 1909d). Bas.: Jungermannia repleta Hook.f. et Taylor, London J. Bot. 3: 557, 1844 (Hooker and Taylor 1844d).

** Schistochila rubriseta Steph., Sp. Hepat. (Stephani) 4: 78, 1909 (Stephani 1909d).

** Schistochila schultzei Steph., Sp. Hepat. (Stephani) 6: 494, 1924 (Stephani 1924).

*** Schistochila sciophila R.M.Schust., Bull. Natl. Sci. Mus. Tokyo (n.ser.) 14 (4): 632, 1971 (Schuster 1971c).

*** Schistochila sciurea (Nees) Schiffn., Nova Acta Acad. Caes. Leop.-Carol. German. Nat. Cur. 60 (2): 251, 1893 (Schiffner 1893a). Bas.: Jungermannia sciurea Nees, Enum. Pl. Crypt. Javae: 34, 1830 (Nees 1830).

*** Schistochila simulans (C.Massal.) Xiao L.He et Yu Sun, Polish Bot. J. 58 (2): 473, 2013 (He and Sun 2013). Bas.: Cephalozia simulans C.Massal., Nuovo Giorn. Bot. Ital. 17 (3): 236, 1885 (Massalongo 1885).

*** Schistochila spegazziniana (C.Massal.) Steph., Bih. Kongl. Svenska Vetensk.-Akad. Handl. 26 (III, 6): 60, 1900 (Stephani 1900b). Bas.: Gottschea spegazziniana C.Massal., Nuovo Giorn. Bot. Ital. 17 (3): 206, 1885 (Massalongo 1885).

*** Schistochila sphagnoides (Schwägr.) Lindb. ex Steph., Sp. Hepat. (Stephani) 4: 70, 1909 (Stephani 1909d). Bas.: Jungermannia sphagnoides Schwägr., Hist. Musc. Hepat. Prodr.: 23, 1814 (Schwägrichen 1814).

*** Schistochila splachnophylla (Hook.f. et Taylor) Steph., Bih. Kongl. Svenska Vetensk.-Akad. Handl. 26 (III, 17): 28, 1901 (Stephani 1901b). Bas.: Jungermannia splachnophylla Hook.f. et Taylor, London J. Bot. 3: 455, 1844 (Hooker and Taylor 1844b).

** Schistochila stratosa (Mont.) A.Evans, Contr. U.S. Natl. Herb. 1 (5): 141, 1892 (Evans 1892b). Bas.: Gottschea stratosa Mont., Ann. Sci. Nat. Bot. (sér. 3) 4: 346, 1845 (Montagne 1845b).

*** Schistochila subhyalina R.M.Schust., Phytologia 30 (4): 246, 1975 (Schuster and Engel 1975).

** Schistochila subhyalina var. grandidentata (J.J.Engel et R.M.Schust.) Xiao L.He et Glenny, Austral. Syst. Bot. 23 (4): 237, 2010 (He and Glenny 2010). Bas.: Pachyschistochila subhyalina var. grandidentata J.J.Engel et R.M.Schust., J. Hattori Bot. Lab. 58: 507, 1985 (Schuster and Engel 1985).

*** Schistochila subimmersa J.J.Engel et R.M.Schust., Phytologia 30 (4): 247, 1975 (Schuster and Engel 1975).

*** Schistochila succulenta (J.J.Engel et R.M.Schust.) Xiao L.He et Glenny, Austral. Syst. Bot. 23 (4): 237, 2010 (He and Glenny 2010). Bas.: Pachyschistochila succulenta J.J.Engel et R.M.Schust., J. Hattori Bot. Lab. 58: 517, 1985 (Schuster and Engel 1985).

*** Schistochila tasmanica Steph., Sp. Hepat. (Stephani) 4: 86, 1909 (Stephani 1909d).

*** Schistochila trispiralis R.M.Schust., Bull. Natl. Sci. Mus. Tokyo (n.ser.) 11 (1): 28, 1968 (Schuster 1968a).

*** Schistochila tuloides (Hook.f. et Taylor) Steph., Sp. Hepat. (Stephani) 4: 89, 1909 (Stephani 1909d). Bas.: Jungermannia tuloides Hook.f. et Taylor, London J. Bot. 3: 558, 1844 (Hooker and Taylor 1844d).

*** Schistochila undulatifolia Piippo, Ann. Bot. Fenn. 23 (1): 8, 1986 (Piippo 1986b).

*** Schistochila virescens R.M.Schust., Phytologia 30 (4): 248, 1975 (Schuster and Engel 1975).

*** Schistochila vitreocincta (Herzog) Xiao L.He et Glenny, Austral. Syst. Bot. 23 (4): 237, 2010 (He and Glenny 2010). Bas.: Perssoniella vitreocincta Herzog, Ark. Bot. (n.ser.) 2 (4): 265, 1952 (Herzog 1952g).

** Schistochila volans Grolle, J. Hattori Bot. Lab. 29: 244, 1966 (Grolle 1966h).

*** Schistochila yakushimensis N.Ohnishi et Deguchi, Bryologist 106 (3): 451, 2003 (Ohnishi and Deguchi 2003).

** Schistochila zantenii Grolle, J. Hattori Bot. Lab. 29: 243, 1966 (Grolle 1966h).

###### Porellales Schljakov

####### Jubulineae Müll.Frib.

######## *** Frullaniaceae Lorch

by M. von Konrat, J. Hentschel, J. Uribe, P. Sukkharak, J. Heinrichs, J. Larraín, R. Stotler and L. Zhang

The subgeneric treatment of Frullaniaceae follows Hentschel et al. (2015), which includes a slight departure from formal infrageneric ranks where they apply clades referred to as Diastaloba I, II, III, and IV that appear to represent distinct subgenera.

*** **Frullania Raddi**, Jungermanniogr. Etrusca: 9, 1818 (Raddi 1818a).

*** **subg. Chonanthelia Spruce**, Trans. & Proc. Bot. Soc. Edinburgh 15: 8, 1884 (Spruce 1884).

* Frullania flammea Taylor, Trans. & Proc. Bot. Soc. Edinburgh 15: 29, 1884 (Spruce 1884).

*** Frullania lindmanii Steph., Bih. Kongl. Svenska Vetensk.-Akad. Handl. 23 (III, 2): 19, 1897 (Stephani 1897a).

** Frullania paranensis Steph., Sp. Hepat. (Stephani) 4: 607, 1911 (Stephani 1911e).

** Frullania spegazzinii M.E.Reiner, Bol. Soc. Argent. Bot. 25 (3/4): 310, 1988 (Reiner 1988).

*** **sect. Chonanthelia (Spruce) Yuzawa ex Hentschel et von Konrat**, Phytotaxa 220 (2): 129, 2015 (Hentschel et al. 2015). Bas.: Frullania subg. Chonanthelia Spruce, Trans. & Proc. Bot. Soc. Edinburgh 15: 8, 1884 (Spruce 1884).

*** Frullania gibbosa Nees, Syn. Hepat. 3: 411, 1845 (Gottsche et al. 1845b).

*** **sect. Cladocarpicae Spruce**, Trans. & Proc. Bot. Soc. Edinburgh 15: 43, 1884 (Spruce 1884).

*** Frullania beauverdii Steph., Biblioth. Bot. 87 (2): 241, 1916 (Stephani 1916a).

*** Frullania blepharozia Spruce, Trans. & Proc. Bot. Soc. Edinburgh 15: 18, 1884 (Spruce 1884).

*** Frullania bogotensis Steph., Sp. Hepat. (Stephani) 4: 327, 1910 (Stephani 1910b).

* Frullania brachycarpa Spruce, Bull. Soc. Bot. France (Congr. Bot.) 36: cciv, 1889 [1890] (Spruce 1889).

*** Frullania confertiloba Steph., Sp. Hepat. (Stephani) 4: 326, 1910 (Stephani 1910b).

*** Frullania decidua Spruce, Trans. & Proc. Bot. Soc. Edinburgh 15: 30, 1884 (Spruce 1884).

*** Frullania ecklonii (Spreng.) Gottsche, Lindenb. et Nees, Syn. Hepat. 3: 413, 1845 (Gottsche et al. 1845b). Bas.: Jungermannia ecklonii Spreng. Syst. Veg. (ed. 16) [Sprengel] 4 (2): 324, 1827 (Sprengel 1827b).

* Frullania ecklonii var. robustior (Gottsche, Lindenb. et Nees) Sim, Trans. Roy. Soc. South Africa 15 (1): 39, 1926 (Sim 1926). Bas.: Frullania ecklonii a robustior Gottsche, Lindenb. et Nees, Syn. Hepat. 5: 771, 1847 (Gottsche et al. 1847).

* Frullania ecklonii var. rufescens Gottsche, Lindenb. et Nees, Syn. Hepat. 5: 771, 1847 (Gottsche et al. 1847).

* Frullania ecklonii var. tenerior (Gottsche, Lindenb. et Nees) Sim, Trans. Roy. Soc. South Africa 15 (1): 39, 1926 (Sim 1926). Bas.: Frullania ecklonii b tenerior Gottsche, Lindenb. et Nees, Syn. Hepat. 5: 771, 1847 (Gottsche et al. 1847).

*** Frullania holostipula S.Hatt. et D.G.Griffin, Misc. Bryol. Lichenol. 8 (3): 47, 1978 (Hattori and Griffin 1978).

*** Frullania megalostipa Spruce, Trans. & Proc. Bot. Soc. Edinburgh 15: 15, 1884 (Spruce 1884).

*** Frullania obscura (Sw.) Mont., Ann. Sci. Nat. Bot. (sér. 2) 14: 333, 1840 (Montagne 1840a). Bas.: Jungermannia obscura Sw., Fl. Ind. Occid. 3: 1869, 1806 (Swartz 1806).

*** Frullania obscura var. spiniloba (Steph.) Hentschel et von Konrat, Phytotaxa 220 (2): 136, 2015 (Hentschel et al. 2015). Bas.: Frullania spiniloba Steph., Sp. Hepat. (Stephani) 4: 336, 1910 (Stephani 1910b).

*** Frullania ringens Spruce, Trans. & Proc. Bot. Soc. Edinburgh 15: 17, 1884 (Spruce 1884).

*** Frullania rio-janeirensis (Raddi) Ångstr., Öfvers. Kongl. Vetensk.-Akad. Förh. 33 (7): 88, 1876 [1877] (Ångström 1876). Bas.: Frullanoides rio-janeirensis Raddi, Critt. Brasil.: 13, 1822 (Raddi 1822).

*** Frullania sphaerocephala Spruce, Trans. & Proc. Bot. Soc. Edinburgh 15: 17, 1884 (Spruce 1884).

** Frullania tunguraguana L.Clark et Frye, Bryologist 55 (2): 133, 1952 (Clark and Frye 1952). *Nom. nov. pro Frullania brachyclada* Spruce, Trans. & Proc. Bot. Soc. Edinburgh 15: 15, 1884 (Spruce 1884), *nom. illeg*.

*** **sect. Pluricarinatae (Yuzawa, Mues et S.Hatt.) Hentschel et von Konrat**, Phytotaxa 220 (2): 129, 2015 (Hentschel et al. 2015). Bas.: Frullania ser. Pluricarinatae Yuzawa, Mues et S.Hatt., J. Hattori Bot. Lab. 63: 428, 1987 (Yuzawa et al. 1987).

*** Frullania albertii Steph., Biblioth. Bot. 87 (2): 237, 1916 (Stephani 1916a).

*** Frullania arsenii Steph., Sp. Hepat. (Stephani) 6: 530, 1924 (Stephani 1924).

** Frullania bonariensis M.E.Reiner, Bol. Soc. Argent. Bot. 25 (3/4): 313, 1988 (Reiner 1988).

*** Frullania cuencensis Taylor, London J. Bot. 5: 406, 1846 (Taylor 1846b).

*** Frullania depressa Mitt., J. Proc. Linn. Soc., Bot. 7 (27): 168, 1863 (Mitten 1863).

*** Frullania dusenii Steph., Arch. Mus. Nac. Rio de Janeiro 13: 115 (9), 1905 (Stephani 1905c).

*** Frullania gradsteinii Yuzawa, Mues et S.Hatt., J. Hattori Bot. Lab. 63: 429, 1987 (Yuzawa et al. 1987).

*** Frullania haematocysta Spruce, Trans. & Proc. Bot. Soc. Edinburgh 15: 54, 1884 (Spruce 1884).

*** Frullania jelskii Loitl., Diagn. pl. nov.: 18, 1894 (Loitlesberger 1894).

*** Frullania laxiflora Spruce, Trans. & Proc. Bot. Soc. Edinburgh 15: 26, 1884 (Spruce 1884).

* Frullania laxiflora var. crossii Spruce, Trans. & Proc. Bot. Soc. Edinburgh 15: 27, 1884 (Spruce 1884).

*** Frullania neurota Taylor, London J. Bot. 5: 400, 1846 (Taylor 1846b).

*** Frullania planifolia Steph., Sp. Hepat. (Stephani) 4: 337, 1910 (Stephani 1910b).

*** Frullania pluricarinata Gottsche, Ann. Sci. Nat. Bot. (sér. 5) 1: 168, 1864 (Gottsche 1864).

** Frullania sandvicensis Ångstr., Öfvers. Kongl. Vetensk.-Akad. Förh. 29 (4): 28, 1872 (Ångström 1872).

*** Frullania standaertii Steph., Sp. Hepat. (Stephani) 4: 342, 1910 (Stephani 1910b).

*** Frullania stenostipa Spruce, Trans. & Proc. Bot. Soc. Edinburgh 15: 29, 1884 (Spruce 1884).

*** Frullania tetraptera Nees et Mont., Ann. Sci. Nat. Bot. (sér. 2) 9: 47, 1838 (Montagne 1838).

** Frullania trinervis (Lehm.) Drège, Flora, Beig. 26: 186, 1843 (Drège 1843). Bas.: Jungermannia trinervis Lehm., Linnaea 9 (4): 426, 1835 (Lehmann 1835). ^186^

*** Frullania winteri Steph., Sp. Hepat. (Stephani) 4: 338, 1910 (Stephani 1910b).

*** Frullania winteri var. vanderhammenii (Haarbrink) Yuzawa, J. Hattori Bot. Lab. 70: 233, 1991 (Yuzawa 1991). Bas.: Frullania vanderhammenii Haarbrink, Lindbergia 7 (1): 56, 1981 (Haarbrink 1981).

*** **subg. Diastaloba Spruce**, Trans. & Proc. Bot. Soc. Edinburgh 15: 55, 1884 (Spruce 1884).

** Frullania antaresensis S.Hatt., J. Hattori Bot. Lab. 47: 92, 1980 (Hattori 1980d).

* Frullania armatifolia Verd., Bull. Jard. Bot. Buitenzorg (sér. 3) 12 (1): 61, 1932 (Verdoorn 1932a).

*** Frullania curvilobula Schäf.-Verw., D.F.Peralta et S.M.Siqueira, Phytotaxa 57 (4): 27, 2012 (Schäfer-Verwimp et al. 2012).

** Frullania gracilicaulis S.Hatt., J. Hattori Bot. Lab. 43: 421, 1977 [1978] (Hattori 1977b).

** Frullania humbertii Vanden Berghen, Bull. Jard. Bot. Natl. Belg. 46 (1/2): 24, 1976 (Vanden Berghen 1976b).

** Frullania hypoleucula S.Hatt., J. Hattori Bot. Lab. 57: 413, 1984 (Hattori 1984a).

** Frullania incurva S.Hatt., J. Hattori Bot. Lab. 65: 431, 1988 (Hattori 1988a).

** Frullania klotzschii Nees, Sp. Hepat. (Stephani) 4: 558, 1911 (Stephani 1911e).

** Frullania letestui Vanden Berghen, Bull. Jard. Bot. Natl. Belg. 46 (1/2): 38, 1976 (Vanden Berghen 1976b).

* Frullania miradorensis Lindenb. et Gottsche, Syn. Hepat. 5: 781, 1847 (Gottsche et al. 1847).

* Frullania odontostipa Spruce, Mem. Torrey Bot. Club 1 (3): 120, 1890 (Spruce 1890).

*** Frullania pilibracteola S.Hatt., J. Hattori Bot. Lab. 43: 428, 1977 [1978] (Hattori 1977b).

*** Frullania pilistipula Steph., Sp. Hepat. (Stephani) 4: 648, 1911 (Stephani 1911e).

*** Frullania ramuligera (Nees) Mont., Ann. Sci. Nat. Bot. (sér. 2) 18: 14, 1842 (Montagne 1842b). Bas.: Jungermannia ramuligera Nees, Enum. Pl. Crypt. Javae: 52, 1830 (Nees 1830).

** Frullania subpilibracteola S.Hatt., J. Hattori Bot. Lab. 43: 434, 1977 [1978] (Hattori 1977b).

** Frullania subtilissima (Nees ex Mont.) Lindenb., Syn. Hepat. 3: 443, 1845 (Gottsche et al. 1845b). Bas.: Frullania atrata β subtilissima Nees ex Mont., Ann. Sci. Nat. Bot. (sér. 2) 14: 333, 1840 (Montagne 1840a).

** Frullania vandenberghenii Pócs, Acta Bot. Acad. Sci. Hung. 25 (3/4): 229, 1979 [1980] (Bizot and Pócs 1979). *Nom. nov. pro Frullania epiphylla* Vanden Berghen, Bull. Jard. Bot. Natl. Belg. 46 (1/2): 30, 1976 (Vanden Berghen 1976b), *nom. illeg*.

**grp. Diastaloba I**

** Frullania akiyamae S.Hatt., J. Hattori Bot. Lab. 60: 240, 1986 (Hattori 1986b).

** Frullania apiculata (Reinw., Blume et Nees) Nees, Syn. Hepat. 3: 452, 1845 (Gottsche et al. 1845b). Bas.: Jungermannia apiculata Reinw., Blume et Nees, Nova Acta Phys.-Med. Acad. Caes. Leop.-Carol. Nat. Cur. 12 (1): 222, 1824 [1825] (Reinwardt et al. 1824a).

** Frullania apiculata var. goebelii Schiffn., Nova Acta Acad. Caes. Leop.-Carol. German. Nat. Cur. 60 (2): 222, 1893 (Schiffner 1893a).

*** Frullania armitiana Steph., Sp. Hepat. (Stephani) 4: 538, 1911 (Stephani 1911e).

** Frullania armitiana var. inflexula S.Hatt., J. Hattori Bot. Lab. 65: 415, 1988 (Hattori 1988a).

** Frullania aterrima (Hook.f. et Taylor) Gottsche, Lindenb. et Nees, Syn. Hepat. 3: 450, 1845 (Gottsche et al. 1845b). Bas.: Jungermannia aterrima Hook.f. et Taylor, London J. Bot. 3: 395, 1844 (Hooker and Taylor 1844a).

*** Frullania attenuata Steph., Sp. Hepat. (Stephani) 4: 538, 1911 (Stephani 1911e).

*** Frullania bella Steph., Sp. Hepat. (Stephani) 4: 643, 1911 (Stephani 1911e).

** Frullania changii S.Hatt. et C.Gao, J. Jap. Bot. 60 (1): 1, 1985 (Hattori and Gao 1985).

* Frullania claviloba Steph., Sp. Hepat. (Stephani) 4: 651, 1911 (Stephani 1911e). ^187^

*** Frullania colliculosa von Konrat, Braggins, Hentschel et Heinrichs, Nova Hedwigia 91 (3/4): 494, 2010 (von Konrat et al. 2010b).

*** Frullania cordistipula (Reinw., Blume et Nees) Nees, Voy. Amér. Mérid. 7 (2): 68, 1839 (Montagne 1839a). Bas.: Jungermannia cordistipula Reinw., Blume et Nees, Nova Acta Phys.-Med. Acad. Caes. Leop.-Carol. Nat. Cur. 12 (1): 220, 1824 [1825] (Reinwardt et al. 1824a). ^188^

** Frullania cordistipula var. dentistipula S.Hatt., J. Hattori Bot. Lab. 60: 242, 1986 (Hattori 1986b).

* Frullania cordistipula var. mutica (Gottsche, Lindenb. et Nees) Schiffn., Consp. Hepat. Arch. Ind.: 323, 1898 (Schiffner 1898b). Bas.: Frullania cordistipula β mutica Gottsche, Lindenb. et Nees, Syn. Hepat. 3: 454, 1845 (Gottsche et al. 1845b).

* Frullania crenatiloba Steph., Sp. Hepat. (Stephani) 6: 551, 1924 (Stephani 1924). ^189^

*** Frullania curvistipula Steph., Sp. Hepat. (Stephani) 4: 548, 1911 (Stephani 1911e).

** Frullania curvistipula var. falcatidentata S.Hatt., Misc. Bryol. Lichenol. 9 (6): 124, 1982 (Hattori 1982a).

** Frullania curvistipula var. lamii Verd., Ann. Bryol., Suppl. 1: 91, 1930 (Verdoorn 1930c).

** Frullania cuspidifolia Steph., Sp. Hepat. (Stephani) 4: 543, 1911 (Stephani 1911e).

* Frullania degelii S.W.Arnell, Svensk Bot. Tidskr. 53 (4): 503, 1959 (Arnell 1959).

** Frullania dentifera S.Hatt. et Streimann, J. Hattori Bot. Lab. 59: 102, 1985 (Hattori and Streimann 1985).

*** Frullania dentiloba S.Hatt., J. Jap. Bot. 50 (6): 161, 1975 (Hattori 1975a).

** Frullania durifolia Steph., Hedwigia 33 (3): 162, 1894 (Stephani 1894d).

** Frullania exilis Taylor, London J. Bot. 5: 405, 1846 (Taylor 1846b).

** Frullania gabonensis Vanden Berghen, Bull. Jard. Bot. Natl. Belg. 46 (1/2): 81, 1976 (Vanden Berghen 1976b).

*** Frullania gracilis (Reinw., Blume et Nees) Nees, Syn. Hepat. 3: 452, 1845 (Gottsche et al. 1845b). Bas.: Jungermannia gracilis Reinw., Blume et Nees, Nova Acta Phys.-Med. Acad. Caes. Leop.-Carol. Nat. Cur. 12 (1): 221, 1824 [1825] (Reinwardt et al. 1824a).

* Frullania gracilis var. brevior (Gottsche, Lindenb. et Nees) Schiffn., Consp. Hepat. Arch. Ind.: 327, 1898 (Schiffner 1898b). Bas.: Frullania gracilis β brevior Gottsche, Lindenb. et Nees, Syn. Hepat. 3: 453, 1845 (Gottsche et al. 1845b).

** Frullania gracilis var. vittata S.Hatt., J. Hattori Bot. Lab. 60: 243, 1986 (Hattori 1986b).

** Frullania gracilis subsp. zennoskei S.Hatt. et Thaithong, J. Hattori Bot. Lab. 44: 183, 1978 (Hattori and Thaithong 1978b).

*** Frullania hasskarliana Lindenb., Syn. Hepat. 3: 453, 1845 (Gottsche et al. 1845b).

** Frullania hasskarliana var. gracilis S.Hatt., J. Hattori Bot. Lab. 60: 243, 1986 (Hattori 1986b).

** Frullania hasskarliana var. parvidentata S.Hatt., J. Hattori Bot. Lab. 60: 245, 1986 (Hattori 1986b).

*** Frullania hattorii von Konrat et Braggins, New Zealand J. Bot. 41 (1): 56, 2003 (von Konrat and Braggins 2003).

*** Frullania hodgsoniae von Konrat, Braggins, Hentschel et Heinrichs, Nova Hedwigia 91 (3/4): 492, 2010 (von Konrat et al. 2010b). *Nom. nov. pro* Frullania aterrima var. lepida E.A.Hodgs., Trans. & Proc. Roy. Soc. New Zealand 77 (3): 386, 1949 (Hodgson 1949).

** Frullania hottana S.Hatt., J. Hattori Bot. Lab. 40: 479, 1976 (Hattori 1976d).

*** Frullania inconstans Verd., Ann. Bryol., Suppl. 1: 83, 1930 (Verdoorn 1930c).

** Frullania inconstans var. grossedentata Kamim. et S.Hatt., J. Hattori Bot. Lab. 37: 530, 1973 (Hattori and Kamimura 1973).

*** Frullania johnsonii Steph., Hedwigia 33 (3): 163, 1894 (Stephani 1894d).

** Frullania macgregorii Steph., Hedwigia 33 (3): 154, 1894 (Stephani 1894d).

** Frullania macgregorii var. rostellula (S.Hatt.) S.Hatt., J. Hattori Bot. Lab. 51: 220, 1982 (Hattori 1982d). Bas.: Frullania reimersii var. rostellula S.Hatt., J. Hattori Bot. Lab. 38: 258, 1974 (Hattori 1974c).

** Frullania madens Steph., Sp. Hepat. (Stephani) 6: 553, 1924 (Stephani 1924).

** Frullania mehrana S.Hatt., Recent Adv. Bot.: 66, 1976 (Hattori 1976e).

** Frullania motoyana Steph., Sp. Hepat. (Stephani) 4: 646, 1911 (Stephani 1911e).

* Frullania multilacera Steph., Sp. Hepat. (Stephani) 4: 650, 1911 (Stephani 1911e). ^190^

** Frullania multilacera subsp. gracilior S.Hatt., Mem. New York Bot. Gard. 45: 547, 1987 (Hattori 1987a).

** Frullania multilacera var. lacerissima S.Hatt., J. Hattori Bot. Lab. 39: 294, 1975 (Hattori 1975d).

** Frullania multilaceroides S.Hatt., Mem. New York Bot. Gard. 45: 549, 1987 (Hattori 1987a).

** Frullania neosheana S.Hatt., J. Hattori Bot. Lab. 45: 350, 1979 (Hattori 1979b).

** Frullania papillata Steph., Sp. Hepat. (Stephani) 4: 615, 1911 (Stephani 1911e).

** Frullania pulogensis Steph., Sp. Hepat. (Stephani) 4: 545, 1911 (Stephani 1911e).

*** Frullania purpurea Steph., Sp. Hepat. (Stephani) 4: 626, 1911 (Stephani 1911e).

** Frullania reimersii Verd., Ann. Bryol., Suppl. 1: 84, 1930 (Verdoorn 1930c).

** Frullania saepisdentata S.Hatt. et Streimann, J. Hattori Bot. Lab. 59: 116, 1985 (Hattori and Streimann 1985).

** Frullania schiffneri Verd., Ann. Bryol. 2: 150, 1929 (Verdoorn 1929a).

** Frullania schusterana S.Hatt., J. Hattori Bot. Lab. 36: 411, 1972 [1973] (Hattori 1972b).

** Frullania seriatifolia Steph., Hedwigia 33 (3): 167, 1894 (Stephani 1894d).

** Frullania serrata Gottsche, Syn. Hepat. 3: 453, 1845 (Gottsche et al. 1845b).

** Frullania serrata var. ceramensis S.Hatt., J. Hattori Bot. Lab. 60: 247, 1986 (Hattori 1986b).

** Frullania serrata subsp. grolleana (S.Hatt.) S.Hatt., J. Hattori Bot. Lab. 51: 225, 1982 (Hattori 1982d). Bas.: Frullania grolleana S.Hatt., J. Hattori Bot. Lab. 36: 416, 1972 [1973] (Hattori 1972b).

** Frullania serrata var. hamatispina (S.Hatt.) S.Hatt., J. Hattori Bot. Lab. 51: 225, 1982 (Hattori 1982d). Bas.: Frullania serrata subsp. hamatispina S.Hatt., J. Hattori Bot. Lab. 38: 262, 1974 (Hattori 1974c).

** Frullania serrata var. pertenuis (Nees) Schiffn., Consp. Hepat. Arch. Ind.: 342, 1898 (Schiffner 1898b). Bas.: Jungermannia cordistipula γ pertenuis Nees, Enum. Pl. Crypt. Javae: 49, 1830 (Nees 1830).

** Frullania serrata subsp. spinistipula S.Hatt., J. Hattori Bot. Lab. 51: 225, 1982 (Hattori 1982d). *Nom. nov. pro Frullania spinistipula* S.Hatt., J. Hattori Bot. Lab. 36: 413, 1972 [1973] (Hattori 1972b), *nom. illeg*.

** Frullania setacea S.Hatt., J. Hattori Bot. Lab. 65: 447, 1988 (Hattori 1988a).

** Frullania sheana S.Hatt., J. Hattori Bot. Lab. 45: 356, 1979 (Hattori 1979b).

** Frullania simmondsii Steph., J. & Proc. Roy. Soc. New South Wales 48 (1/2): 109, 1914 (Stephani and Watts 1914).

*** Frullania sinuata Sande Lac., Ned. Kruidk. Arch. 3: 424, 1854 [1855] (Sande Lacoste 1854).

** Frullania steereana S.Hatt., Mem. New York Bot. Gard. 45: 553, 1987 (Hattori 1987a).

** Frullania stipatiloba Steph., Hedwigia 33 (3): 168, 1894 (Stephani 1894d).

** Frullania subdentata Steph., Sp. Hepat. (Stephani) 4: 545, 1911 (Stephani 1911e).

** Frullania subdentata var. latistipula (S.Hatt.) S.Hatt., J. Hattori Bot. Lab. 51: 228, 1982 (Hattori 1982d). Bas.: Frullania curvistipula var. latistipula S.Hatt., J. Hattori Bot. Lab. 44: 530, 1978 (Hattori 1978b).

** Frullania submultilacera S.Hatt. et Koike, J. Hattori Bot. Lab. 75: 190, 1994 (Koike 1994).

** Frullania subocellata S.Hatt., J. Hattori Bot. Lab. 60: 248, 1986 (Hattori 1986b).

*** Frullania taxodiocola R.M.Schust., Phytologia 53 (5): 364, 1983 (Schuster 1983b).

*** Frullania ternatensis Gottsche, Syn. Hepat. 4: 465, 1846 (Gottsche et al. 1846).

** Frullania ternatensis var. non-appendiculata S.Hatt., J. Hattori Bot. Lab. 38: 174, 1974 (Hattori 1974a).

*** Frullania trichodes Mitt., Bonplandia 10 (2): 19, 1862 (Mitten 1862).

** Frullania vaga Mitt., Fl. vit.: 418, 1871 [1873] (Mitten 1871).

*** Frullania vaginata (Sw.) Nees, Syn. Hepat. 4: 465, 1846 (Gottsche et al. 1846). Bas.: Jungermannia vaginata Sw., Meth. Musc.: 35, 1781 (Swartz 1781).

* Frullania vaginata var. nigricans (Gottsche, Lindenb. et Nees) Schiffn., Consp. Hepat. Arch. Ind.: 348, 1898 (Schiffner 1898b). Bas.: Frullania vaginata β nigricans Gottsche, Lindenb. et Nees, Syn. Hepat. 4: 465, 1846 (Gottsche et al. 1846).

* Frullania van-zantenii Kamim. et S.Hatt., J. Hattori Bot. Lab. 37: 528, 1973 (Hattori and Kamimura 1973). ^191^

** Frullania venusta S.Hatt., J. Hattori Bot. Lab. 38: 217, 1974 (Hattori 1974d).

** Frullania verdoorniana S.Hatt., J. Hattori Bot. Lab. 37: 122, 1973 (Hattori 1973b).

*** Frullania vitalii Yuzawa et S.Hatt., J. Jap. Bot. 63 (1): 30, 1988 (Yuzawa and Hattori 1988a).

** Frullania vittata S.Hatt., J. Hattori Bot. Lab. 38: 270, 1974 (Hattori 1974c).

** Frullania vivipara Pócs, Fieldiana, Bot. (n.ser.) 47: 151, 2008 (Pócs 2008a).

** Frullania wairua von Konrat et Braggins, New Zealand J. Bot. 43 (4): 886, 2005 (von Konrat and Braggins 2005).

** Frullania warnckeana S.Hatt., J. Hattori Bot. Lab. 38: 213, 1974 (Hattori 1974d).

** Frullania warnckeana var. dentosa S.Hatt., Misc. Bryol. Lichenol. 7 (8): 162, 1977 (Hattori 1977a).

**grp. Diastaloba II**

*** Frullania baumannii S.Hatt., J. Hattori Bot. Lab. 43: 410, 1977 [1978] (Hattori 1977b).

*** Frullania congesta Gottsche, Lindenb. et Nees, Syn. Hepat. 3: 451, 1845 (Gottsche et al. 1845b). *Nom. nov. pro Jungermannia congesta* Hook.f. et Taylor, London J. Bot. 3: 396, 1844 (Hooker and Taylor 1844a), *nom. illeg*.

** Frullania ocellata S.Hatt. et Kamim., J. Hattori Bot. Lab. 37: 531, 1973 (Hattori and Kamimura 1973).

*** Frullania ptychantha Mont., Ann. Sci. Nat. Bot. (sér. 2) 19: 257, 1843 (Montagne 1843).

*** Frullania repandistipula Sande Lac., Ned. Kruidk. Arch. 3: 422, 1854 [1855] (Sande Lacoste 1854).

** Frullania repandistipula subsp. queenslandica S.Hatt., Mem. New York Bot. Gard. 45: 550, 1987 (Hattori 1987a).

** Frullania repandistipula subsp. spinibractea S.Hatt., Bull. Natl. Sci. Mus. Tokyo, B 1 (4): 150, 1975 (Hattori 1975f).

**grp. Diastaloba III**

** Frullania grossiclava Steph., Sp. Hepat. (Stephani) 4: 384, 1910 (Stephani 1910b).

** Frullania loricata Pearson, Forh. Vidensk.-Selsk. Kristiania 1890 (2): 6, 1891 (Pearson 1891).

* Frullania loricata var. laxa Pearson, Forh. Vidensk.-Selsk. Kristiania 1890 (2): 8, 1891 (Pearson 1891).

*** Frullania usambarana Schiffn., Hedwigia 33 (3): 160, 1894 (Stephani 1894d).

** Frullania usambarana var. reducta Vanden Berghen, Bull. Jard. Bot. Natl. Belg. 46 (1/2): 91, 1976 (Vanden Berghen 1976b).

**grp. Diastaloba IV**

* Frullania brunea (Spreng.) Drège, Flora, Beig. 26: 186, 1843 (Drège 1843). Bas.: Jungermannia brunea Spreng. Syst. Veg. (ed. 16) [Sprengel] 4 (2): 325, 1827 (Sprengel 1827b). ^192^

*** Frullania caulisequa (Nees) Mont., Ann. Sci. Nat. Bot. (sér. 2) 12: 51, 1839 (Montagne 1839c). Bas.: Jungermannia caulisequa Nees, Fl. Bras. (Martius) 1 (1): 373, 1833 (Nees 1833a).

*** Frullania grossifolia Steph., Sp. Hepat. (Stephani) 4: 633, 1911 (Stephani 1911e).

*** Frullania hypoleuca Nees, Observ. bot.: 470, 1843 (Gottsche et al. 1843).

*** Frullania lindenbergii Lehm., Nov. Stirp. Pug. 8: 17, 1844 (Lehmann 1844).

* Frullania lindenbergii var. fusca Gottsche, Lindenb. et Nees, Syn. Hepat. 5: 780, 1847 (Gottsche et al. 1847).

** Frullania ponapensis S.Hatt. et Koike, J. Hattori Bot. Lab. 75: 186, 1994 (Koike 1994).

* Frullania tricarinata Sande Lac., Plagiochila Sandei: 10, 1856 (Sande Lacoste 1856c). ^193^

* **subg. Diversitextae (Kamim.) S.Hatt.**, J. Hattori Bot. Lab. 59: 154, 1985 (Hattori and Lin 1985a). Bas.: Frullania subsect. Diversitextae Kamim., J. Hattori Bot. Lab. 24: 80, 1961 (Kamimura 1961).

*** Frullania diversitexta Steph., Bull. Herb. Boissier 5 (2): 89, 1897 (Stephani 1897b).

*** **subg. Frullania**

* Frullania amamiensis Kamim., Bull. Kochi Gakuen Jun. Coll. 1: 51, 1970 (Kamimura 1970).

** Frullania amplicrania Steph., Sp. Hepat. (Stephani) 4: 404, 1910 (Stephani 1910b).

*** Frullania ampullifera J.B.Jack et Steph., Hedwigia 33 (3): 139, 1894 (Stephani 1894d).

*** Frullania anderssonii Ångstr., Öfvers. Kongl. Vetensk.-Akad. Förh. 30 (5): 144, 1873 (Ångström 1873).

** Frullania angustistipa Steph., Akad. Wiss. Wien, Math.-Naturwiss. Kl., Denkschr. 81: 297, 1907 (Stephani 1907a).

** Frullania aposinensis S.Hatt. et P.J.Lin, J. Hattori Bot. Lab. 59: 131, 1985 (Hattori and Lin 1985a). *Nom. nov. pro Frullania chinensis* Steph., Sp. Hepat. (Stephani) 4: 469, 1911 (Stephani 1911e), *nom. illeg*.

** Frullania appendistipula S.Hatt., J. Hattori Bot. Lab. 36: 424, 1972 [1973] (Hattori 1972b).

** Frullania appendistipula var. spinifera S.Hatt., J. Hattori Bot. Lab. 38: 226, 1974 (Hattori 1974c).

** Frullania auriculata S.Hatt., Bull. Natl. Sci. Mus. Tokyo, B 11 (1): 11, 1985 (Hattori 1985).

** Frullania benjaminiana Inoue, Bull. Natl. Sci. Mus. Tokyo, B 1 (3): 109, 1975 (Hattori 1975b).

** Frullania bergmanii S.Hatt., J. Hattori Bot. Lab. 38: 192, 1974 (Hattori 1974d).

*** Frullania berthoumieui Steph., Hedwigia 33 (3): 140, 1894 (Stephani 1894d).

** Frullania bhutanensis S.Hatt., Fl. E. Himalaya 2: 232, 1971 (Hattori 1971a).

** Frullania blastopetala S.Hatt., J. Hattori Bot. Lab. 57: 407, 1984 (Hattori 1984a).

*** Frullania bonincola S.Hatt., J. Hattori Bot. Lab. 44: 551, 1978 (Hattori 1978b). *Nom. nov. pro Frullania viridis* Horik., Sci. Rep. Tôhoku Imp. Univ., Ser. 4, Biol. 5 (4): 646, 1929 [1930] (Horikawa 1929c), *nom. illeg*.

* Frullania brevicalycina Steph., Hedwigia 33 (3): 141, 1894 (Stephani 1894d).

** Frullania brittoniae A.Evans, Trans. Connecticut Acad. Arts 10 (1): 15, 1899 (Evans 1899).

*** Frullania bullata Steph., Sp. Hepat. (Stephani) 4: 371, 1910 (Stephani 1910b).

*** Frullania caffraria Steph., Hedwigia 33 (3): 141, 1894 (Stephani 1894d).

*** Frullania calcarata Ångstr., Öfvers. Kongl. Vetensk.-Akad. Förh. 30 (5): 137, 1873 (Ångström 1873).

** Frullania carrii Kamim. et S.Hatt., J. Hattori Bot. Lab. 37: 520, 1973 (Hattori and Kamimura 1973).

** Frullania chenii S.Hatt. et P.J.Lin, J. Jap. Bot. 60 (4): 106, 1985 (Hattori and Lin 1985b).

** Frullania chilensis Steph., Hedwigia 33 (3): 142, 1894 (Stephani 1894d).

*** Frullania chodatii Beauverd, Sp. Hepat. (Stephani) 6: 533, 1924 (Stephani 1924). Based on: Frullania grandistipula Steph., Sp. Hepat. (Stephani) 6: 533, 1924 (Stephani 1924), *nom. inval*.

*** Frullania cobrensis Gottsche, Hedwigia 33 (3): 142, 1894 (Stephani 1894d).

** Frullania consociata Steph., Sp. Hepat. (Stephani) 4: 461, 1911 (Stephani 1911e).

* Frullania contracta Steph., Sp. Hepat. (Stephani) 4: 469, 1911 (Stephani 1911e). ^194^

* Frullania cornuta Steph., Sp. Hepat. (Stephani) 4: 467, 1911 (Stephani 1911e). ^195^

*** Frullania crassitexta Steph., Sp. Hepat. (Stephani) 4: 423, 1910 (Stephani 1910b).

*** Frullania crispiplicata Yuzawa et S.Hatt., J. Jap. Bot. 58 (2): 43, 1983 (Yuzawa and Hattori 1983).

** Frullania cristata S.Hatt., J. Hattori Bot. Lab. 49: 165, 1981 (Hattori 1981a).

* Frullania cuneiloba Nees, Syn. Hepat. 3: 427, 1845 (Gottsche et al. 1845b).

** Frullania cyparioides (Schwägr.) Nees, Naturgesch. Eur. Leberm. 3: 210, 1838 (Nees 1838b). Bas.: Jungermannia cyparioides Schwägr., Hist. Musc. Hepat. Prodr.: 14, 1814 (Schwägrichen 1814).

*** Frullania davurica Hampe ex Gottsche, Lindenb. et Nees, Syn. Hepat. 3: 422, 1845 (Gottsche et al. 1845b).

** Frullania debilis Steph. ex S.Hatt., J. Hattori Bot. Lab. 38: 190, 1974 (Hattori 1974d).

** Frullania deppii Lehm., Nov. Stirp. Pug. 8: 15, 1844 (Lehmann 1844).

** Frullania diptera (Lehm.) Drège, Flora, Beig. 26: 186, 1843 (Drège 1843). Bas.: Jungermannia diptera Lehm., Linnaea 9 (4): 425, 1835 (Lehmann 1835).

*** Frullania duthiana Steph., Sp. Hepat. (Stephani) 4: 351, 1910 (Stephani 1910b).

** Frullania duthiana var. appendiculata S.Hatt., Fl. E. Himalaya 2: 234, 1971 (Hattori 1971a).

** Frullania duthiana var. laevis S.Hatt., Bull. Univ. Mus. Univ. Tokyo 8: 233, 1975 (Hattori 1975e).

** Frullania duthiana var. szechuanensis S.Hatt. et C.Gao, J. Jap. Bot. 60 (1): 2, 1985 (Hattori and Gao 1985).

** Frullania echinantha S.Hatt., J. Hattori Bot. Lab. 38: 233, 1974 (Hattori 1974c).

** Frullania echinatella S.Hatt., J. Hattori Bot. Lab. 65: 423, 1988 (Hattori 1988a).

* Frullania elegans Lehm., Nov. Stirp. Pug. 10: 16, 1857 (Lehmann 1857).

*** Frullania elephantum S.Hatt., J. Hattori Bot. Lab. 43: 416, 1977 [1978] (Hattori 1977b).

** Frullania epiphylla S.Hatt., J. Hattori Bot. Lab. 38: 235, 1974 (Hattori 1974c).

* Frullania epiphylla subsp. fijiensis S.Hatt., Bull. Natl. Sci. Mus. Tokyo, B 11 (1): 14, 1985 (Hattori 1985).

** Frullania erostrata S.Hatt., J. Hattori Bot. Lab. 38: 237, 1974 (Hattori 1974c).

* Frullania esenbeckiana Beauverd, Sp. Hepat. (Stephani) 6: 570, 1924 (Stephani 1924). *Nom. nov. pro Frullania grossiloba* Steph., Sp. Hepat. (Stephani) 4: 531, 1911 (Stephani 1911e), *nom. illeg*.

** Frullania evelynae S.Hatt. et Thaithong, J. Hattori Bot. Lab. 44: 179, 1978 (Hattori and Thaithong 1978b).

* Frullania evelynae var. devendrae Sushil K.Singh et Barbhuiya, Taiwania 57 (2): 109, 2012 (Singh and Barbhuiya 2012).

* Frullania evelynae var. srivastavae Sushil K.Singh et Barbhuiya, Taiwania 57 (2): 112, 2012 (Singh and Barbhuiya 2012).

*** Frullania eymae S.Hatt., J. Hattori Bot. Lab. 39: 284, 1975 (Hattori 1975d).

** Frullania eymae var. crispidentata S.Hatt. et Streimann, J. Hattori Bot. Lab. 59: 105, 1985 (Hattori and Streimann 1985).

*** Frullania falciloba Lehm., Nov. Stirp. Pug. 8: 20, 1844 (Lehmann 1844).

** Frullania falsicornuta S.Hatt., J. Hattori Bot. Lab. 60: 212, 1986 (Hattori 1986e).

** Frullania fauriana Steph., Hedwigia 33 (3): 144, 1894 (Stephani 1894d).

** Frullania fengyangshanensis R.L.Zhu et M.L.So, Bryologist 100 (3): 356, 1997 (Zhu and So 1997b).

** Frullania ferdinandi-muelleri Steph., Sp. Hepat. (Stephani) 4: 417, 1910 (Stephani 1910b).

* Frullania flexuosa S.Hatt., J. Hattori Bot. Lab. 54: 146, 1983 (Hattori 1983). ^196^

** Frullania fuscovirens Steph., Sp. Hepat. (Stephani) 4: 401, 1910 (Stephani 1910b).

** Frullania fuscovirens var. gemmipara (R.M.Schust. et S.Hatt.) S.Hatt. et P.J.Lin, J. Hattori Bot. Lab. 59: 135, 1985 (Hattori and Lin 1985a). Bas.: Frullania gemmipara R.M.Schust. et S.Hatt., J. Hattori Bot. Lab. 44: 547, 1978 (Hattori 1978b).

** Frullania gaoligongensis X.L.Bai et C.Gao, Hikobia 13 (1): 87, 1999 (Bai and Gao 1999).

** Frullania gemmulosa S.Hatt. et Thaithong, J. Hattori Bot. Lab. 43: 449, 1977 [1978] (Hattori et al. 1977).

*** Frullania gigantea Steph., Sp. Hepat. (Stephani) 4: 467, 1911 (Stephani 1911e).

** Frullania giraldiana C.Massal., Hepat. Shen-si: 41, 1897 (Massalongo 1897). ^197^

** Frullania giraldiana var. handelii (Verd.) S.Hatt., J. Hattori Bot. Lab. 36: 123, 1972 [1973] (Hattori 1972c). Bas.: Frullania nepalensis var. handelii Verd., Symb. Sin. 5: 41, 1930 (Nicholson et al. 1930).

** Frullania globosa S.Hatt. et Streimann, J. Hattori Bot. Lab. 59: 107, 1985 (Hattori and Streimann 1985).

** Frullania grandilobula S.Hatt. et Piippo, Acta Bot. Fenn. 133: 45, 1986 (Hattori and Piippo 1986).

*** Frullania grandistipula Lindenb., Syn. Hepat. 3: 430, 1845 (Gottsche et al. 1845b).

** Frullania hainanensis S.Hatt. et P.J.Lin, J. Jap. Bot. 61 (10): 307, 1986 (Hattori and Lin 1986).

** Frullania hamatiloba Steph., Sp. Hepat. (Stephani) 4: 400, 1910 (Stephani 1910b).

** Frullania handelii Verd., Symb. Sin. 5: 36, 1930 (Nicholson et al. 1930).

** Frullania handel-mazzettii S.Hatt., J. Hattori Bot. Lab. 49: 150, 1981 (Hattori 1981a).

** Frullania hattoriantha Udar et V.Nath, Misc. Bryol. Lichenol. 9 (2): 44, 1981 (Udar and Nath 1981).

* Frullania hebridensis Steph., Sp. Hepat. (Stephani) 4: 469, 1911 (Stephani 1911e). ^198^

** Frullania hicksiae S.Hatt., Cryptog. Bryol. Lichénol. 5 (1/2): 182, 1984 (Hattori 1984c).

** Frullania higuchii Yuzawa, Koike et S.Hatt., J. Hattori Bot. Lab. 75: 194, 1994 (Yuzawa and Koike 1994).

** Frullania hiroshii S.Hatt., Bull. Natl. Sci. Mus. Tokyo, B 6 (1): 34, 1980 (Hattori 1980b).

* Frullania hirtiflora Spruce, Trans. & Proc. Bot. Soc. Edinburgh 15: 35, 1884 (Spruce 1884).

*** Frullania howeana Steph., J. & Proc. Roy. Soc. New South Wales 48 (1/2): 107, 1914 (Stephani and Watts 1914).

** Frullania ignatovii Sofronova, Mamontov et Potemkin, Novosti Sist. Nizš. Rast. 47: 335, 2013 (Sofronova et al. 2013).

** Frullania incisoduthiana S.Hatt., J. Hattori Bot. Lab. 46: 391, 1979 (Mizutani 1979c).

* Frullania incisoduthiana var. parva S.Hatt., J. Hattori Bot. Lab. 46: 391, 1979 (Mizutani 1979c).

* Frullania incisostipula Steph., Sp. Hepat. (Stephani) 6: 541, 1924 (Stephani 1924).

** Frullania inflexiloba S.Hatt., J. Hattori Bot. Lab. 57: 415, 1984 (Hattori 1984a).

** Frullania inouei S.Hatt., Bull. Natl. Sci. Mus. Tokyo, B 6 (1): 36, 1980 (Hattori 1980b).

*** Frullania irregularis S.Hatt. et Piippo, Acta Bot. Fenn. 133: 46, 1986 (Hattori and Piippo 1986).

*** Frullania jackii Gottsche, Hepat. Eur., Leberm. 29-30: no. 294, 1863 (Gottsche and Rabenhorst 1863).

** Frullania jacobsii S.Hatt., Bull. Natl. Sci. Mus. Tokyo, B 12 (4): 129, 1986 (Hattori 1986c).

* Frullania jacquinotii Gottsche, J. Bot. (Morot) 12: 138, 1898 (Bescherelle 1898).

** Frullania jovetiana von Konrat et Hentschel, Phytotaxa 220 (2): 135, 2015 (Hentschel et al. 2015). Based on: Frullania pseudericoides S.Hatt., Bull. Natl. Sci. Mus. Tokyo, B 12 (4): 132, 1986 (Hattori 1986c), *nom. illeg*.

** Frullania kagoshimensis Steph., Sp. Hepat. (Stephani) 4: 353, 1910 (Stephani 1910b).

** Frullania kagoshimensis subsp. hunanensis (S.Hatt.) S.Hatt. et P.J.Lin, J. Hattori Bot. Lab. 59: 137, 1985 (Hattori and Lin 1985a). Bas.: Frullania hunanensis S.Hatt., J. Hattori Bot. Lab. 49: 152, 1981 (Hattori 1981a).

** Frullania kalimantanensis S.Hatt., Bull. Natl. Sci. Mus. Tokyo, B 12 (4): 127, 1986 (Hattori 1986c).

** Frullania kashyapii Verd., Ann. Bryol. 5: 162, 1932 (Dixon et al. 1932).

** Frullania kitagawana S.Hatt., J. Hattori Bot. Lab. 57: 417, 1984 (Hattori 1984a).

** Frullania laeviperiantha X.L.Bai et C.Gao, Phytotaxa 220 (2): 136, 2015 (Hentschel et al. 2015). Based on: Frullania laeviperiantha X.L.Bai et C.Gao, Nova Hedwigia 70 (1/2): 135, 2000 (Bai and Gao 2000), *nom. inval*.

* Frullania lancistyla Steph., Sp. Hepat. (Stephani) 4: 389, 1910 (Stephani 1910b).

* Frullania latiflora Spruce, Trans. & Proc. Bot. Soc. Edinburgh 15: 34, 1884 (Spruce 1884).

** Frullania latogaleata Herzog, Trans. Brit. Bryol. Soc. 1 (3): 189, 1949 (Herzog 1949b).

** Frullania lepida S.Hatt. et Piippo, Acta Bot. Fenn. 133: 48, 1986 (Hattori and Piippo 1986).

* Frullania levieri Steph., Sp. Hepat. (Stephani) 4: 388, 1910 (Stephani 1910b).

** Frullania linii S.Hatt., J. Hattori Bot. Lab. 49: 155, 1981 (Hattori 1981a).

* Frullania longistyla Yuzawa et S.Hatt., J. Jap. Bot. 63 (11): 361, 1988 (Yuzawa and Hattori 1988b). ^199^

* Frullania ludoviciae Steph., Rev. Bryol. 35 (2): 29, 1908 (Stephani 1908l). ^200^

** Frullania lushanensis S.Hatt. et P.J.Lin, J. Hattori Bot. Lab. 59: 137, 1985 (Hattori and Lin 1985a).

* Frullania macularis Taylor, London J. Bot. 5: 403, 1846 (Taylor 1846b).

** Frullania maymyoensis Svihla, Bryologist 61 (4): 376, 1958 [1959] (Svihla 1958).

** Frullania microauriculata Verd., Ann. Bryol. 2: 126, 1929 (Verdoorn 1929a).

** Frullania microauriculata var. rotundior Verd., Ann. Jard. Bot. Buitenzorg 40: 141, 1929 (Verdoorn 1929b).

** Frullania microrhyncha L.Clark et Svihla, Bryologist 53 (1): 63, 1950 (Clark and Svihla 1950).

** Frullania mizoramensis Sushil K.Singh et Barbhuiya, Taiwania 57 (2): 106, 2012 (Singh and Barbhuiya 2012).

* Frullania montana Steph., Sp. Hepat. (Stephani) 4: 455, 1911 (Stephani 1911e). ^201^

** Frullania multituberculata Hentschel et von Konrat, Phytotaxa 220 (2): 136, 2015 (Hentschel et al. 2015). *Nom. nov. pro Frullania kalimantanensis* Piippo et S.Hatt., J. Hattori Bot. Lab. 72: 117, 1992 (Piippo and Tan 1992), *nom. illeg*.

** Frullania mutilata Steph., Sp. Hepat. (Stephani) 4: 673, 1911 (Stephani 1911e).

** Frullania nadeaudii Steph., Sp. Hepat. (Stephani) 4: 465, 1911 (Stephani 1911e).

*** Frullania nepalensis (Spreng.) Lehm. et Lindenb., Syn. Hepat. 3: 422, 1845 (Gottsche et al. 1845b). Bas.: Jungermannia nepalensis Spreng. Syst. Veg. (ed. 16) [Sprengel] 4 (2): 324, 1827 (Sprengel 1827b). ^202^

*** Frullania nicholsonii E.A.Hodgs., Trans. & Proc. Roy. Soc. New Zealand 77 (3): 368, 1949 (Hodgson 1949). *Nom. nov. pro Frullania berggrenii* W.E.Nicholson, Bryologist 28 (2): 17, 1925 (Nicholson 1925), *nom. illeg*.

** Frullania nigricaulis (Reinw., Blume et Nees) Nees, Syn. Hepat. 3: 457, 1845 (Gottsche et al. 1845b). Bas.: Jungermannia nigricaulis Reinw., Blume et Nees, Nova Acta Phys.-Med. Acad. Caes. Leop.-Carol. Nat. Cur. 12 (1): 225, 1824 [1825] (Reinwardt et al. 1824a).

* Frullania nigricaulis var. elongata Verd., Ann. Bryol., Suppl. 1: 155, 1930 (Verdoorn 1930c).

** Frullania nivimontana S.Hatt., Bull. Natl. Sci. Mus. Tokyo, B 8 (3): 95, 1982 (Hattori 1982c).

*** Frullania nobilis Steph., Hedwigia 33 (3): 154, 1894 (Stephani 1894d).

** Frullania nobilis var. cochleata (Steph.) S.Hatt., J. Hattori Bot. Lab. 59: 111, 1985 (Hattori and Streimann 1985). Bas.: Frullania cochleata Steph., Sp. Hepat. (Stephani) 4: 681, 1911 (Stephani 1911e).

** Frullania novocurvirostris S.Hatt., J. Hattori Bot. Lab. 49: 370, 1981 (Hattori 1981b). *Nom. nov. pro Frullania curvirostris* J.B.Jack et Steph., Hedwigia 33 (3): 143, 1894 (Stephani 1894d), *nom. illeg*.

** Frullania oahuensis Hampe ex Gottsche, Lindenb. et Nees, Observ. bot.: 471, 1843 (Gottsche et al. 1843).

** Frullania obovata S.Hatt., Bull. Natl. Sci. Mus. Tokyo, B 8 (3): 97, 1982 (Hattori 1982c).

** Frullania obtusangula Hentschel et von Konrat, Phytotaxa 220 (2): 137, 2015 (Hentschel et al. 2015).

** Frullania okinawensis Kamim., Misc. Bryol. Lichenol. 9 (4): 90, 1982 (Kamimura 1982).

** Frullania orbicularis Austin, Proc. Acad. Nat. Sci. Philadelphia 21: 227, 1869 (Austin 1869).

*** Frullania orientalis Sande Lac., Plagiochila Sandei: 10, 1856 (Sande Lacoste 1856c).

** Frullania orinocensis Spruce, Trans. & Proc. Bot. Soc. Edinburgh 15: 30, 1884 (Spruce 1884).

*** Frullania ornithocephala (Reinw., Blume et Nees) Nees, Syn. Hepat. 3: 425, 1845 (Gottsche et al. 1845b). Bas.: Jungermannia ornithocephala Reinw., Blume et Nees, Nova Acta Phys.-Med. Acad. Caes. Leop.-Carol. Nat. Cur. 12 (1): 216, 1824 [1825] (Reinwardt et al. 1824a).

* Frullania ornithocephala var. major (Nees) Schiffn., Consp. Hepat. Arch. Ind.: 336, 1898 (Schiffner 1898b). Bas.: Jungermannia ornithocephala β major Nees, Enum. Pl. Crypt. Javae: 47, 1830 (Nees 1830).

** Frullania ornithocephala var. pilosa Verd., Ann. Bryol. 2: 129, 1929 (Verdoorn 1929a).

** Frullania ornithocephala var. tuberculosa S.Hatt., J. Hattori Bot. Lab. 38: 179, 1974 (Hattori 1974a).

** Frullania pachyderma S.Hatt., J. Hattori Bot. Lab. 44: 525, 1978 (Hattori 1978b).

** Frullania pallidevirens Steph., Sp. Hepat. (Stephani) 4: 454, 1911 (Stephani 1911e).

** Frullania pariharii S.Hatt. et Thaithong, J. Jap. Bot. 53 (5): 130, 1978 (Hattori and Thaithong 1978a).

* Frullania parvifolia Steph., Sp. Hepat. (Stephani) 4: 354, 1910 (Stephani 1910b).

** Frullania pauciramea Steph., Sp. Hepat. (Stephani) 4: 458, 1911 (Stephani 1911e).

** Frullania pauciramea var. pauciramella S.Hatt. et Piippo, Acta Bot. Fenn. 133: 50, 1986 (Hattori and Piippo 1986).

** Frullania paucirameoides S.Hatt. et Piippo, Acta Bot. Fenn. 133: 51, 1986 (Hattori and Piippo 1986).

** Frullania pedicellata Steph., Bull. Herb. Boissier 5 (2): 90, 1897 (Stephani 1897b).

*** Frullania physantha Mitt., J. Proc. Linn. Soc., Bot. 5 (18): 121, 1860 [1861] (Mitten 1860c).

** Frullania piptophylla S.Hatt., J. Hattori Bot. Lab. 47: 87, 1980 (Hattori 1980d).

** Frullania piptophylla var. minor S.Hatt., J. Hattori Bot. Lab. 60: 247, 1986 (Hattori 1986b).

** Frullania piptophylloides S.Hatt., J. Hattori Bot. Lab. 47: 90, 1980 (Hattori 1980d).

* Frullania plicata Hentschel et von Konrat, Phytotaxa 220 (2): 137, 2015 (Hentschel et al. 2015). *Nom. nov. pro Frullania acutiloba* Gerola, Lav. Bot. Ist. Bot. Univ. Padova 12: 477, 1947 (Gerola 1947), *nom. illeg*.

** Frullania pocsantha Thaithong et S.Hatt., Bull. Natl. Sci. Mus. Tokyo, B 3 (4): 149, 1977 (Thaithong and Hattori 1977).

** Frullania pran-nathii M.Dey et D.K.Singh, J. Jap. Bot. 83 (5): 281, 2008 (Dey and Singh 2008).

** Frullania pringlei Fulford et Sharp, Mem. New York Bot. Gard. 63: 45, 1990 (Fulford and Sharp 1990). *Nom. nov. pro Frullania spicata* Steph., Sp. Hepat. (Stephani) 4: 392, 1910 (Stephani 1910b), *nom. illeg*.

** Frullania prominula S.Hatt. et Streimann, J. Hattori Bot. Lab. 59: 112, 1985 (Hattori and Streimann 1985).

** Frullania propaginea S.Hatt. et Streimann, J. Hattori Bot. Lab. 59: 114, 1985 (Hattori and Streimann 1985).

* Frullania pseudericoides S.Hatt., J. Hattori Bot. Lab. 51: 256, 1982 (Hattori 1982d). *Nom. nov. pro* Frullania sharpii subsp. subrostrata S.Hatt., J. Hattori Bot. Lab. 38: 265, 1974 (Hattori 1974c).

** Frullania pseudoschensiana S.Hatt., J. Hattori Bot. Lab. 47: 101, 1980 (Hattori 1980d).

** Frullania pseudoschensiana var. darjeelingensis S.Hatt., J. Hattori Bot. Lab. 49: 149, 1981 (Hattori 1981a).

** Frullania pullei Verd., Nova Guinea 14: 542, 1930 (Verdoorn 1930b).

** Frullania pusilla Mitt., Fl. vit.: 417, 1871 [1873] (Mitten 1871).

*** Frullania pycnantha (Hook.f. et Taylor) Gottsche, Lindenb. et Nees, Syn. Hepat. 3: 411, 1845 (Gottsche et al. 1845b). Bas.: Jungermannia pycnantha Hook.f. et Taylor, London J. Bot. 3: 566, 1844 (Hooker and Taylor 1844d).

** Frullania queenslandica Steph., Sp. Hepat. (Stephani) 4: 424, 1910 (Stephani 1910b).

** Frullania recurvistipula S.Hatt., Bull. Natl. Sci. Mus. Tokyo, B 1 (3): 114, 1975 (Hattori 1975b).

*** Frullania reflexistipula Sande Lac., Ned. Kruidk. Arch. 3: 422, 1854 [1855] (Sande Lacoste 1854).

** Frullania reflexistipula var. squarrosa S.Hatt. et Piippo, Acta Bot. Fenn. 133: 54, 1986 (Hattori and Piippo 1986).

** Frullania remotidens S.Hatt., J. Hattori Bot. Lab. 51: 257, 1982 (Hattori 1982d).

* Frullania reptans Mitt., Bot. antarct. voy. II (Fl. Nov.-Zel. 2): 161, 1855 (Mitten 1855).

*** Frullania retusa Mitt., J. Proc. Linn. Soc., Bot. 5 (18): 119, 1860 [1861] (Mitten 1860c).

** Frullania retusa var. gymnantha S.Hatt. et Thaithong, J. Jap. Bot. 53 (5): 131, 1978 (Hattori and Thaithong 1978a).

** Frullania retusa var. hirsuta S.Hatt. et Thaithong, J. Hattori Bot. Lab. 44: 191, 1978 (Hattori and Thaithong 1978b).

*** Frullania rhystocolea Herzog, Symb. Sin. 5: 39, 1930 (Nicholson et al. 1930).

** Frullania rhytidantha S.Hatt., J. Hattori Bot. Lab. 47: 97, 1980 (Hattori 1980d).

** Frullania rigida Steph., Sp. Hepat. (Stephani) 4: 371, 1910 (Stephani 1910b).

*** Frullania riparia Hampe, Nov. Stirp. Pug. 7: 14, 1838 (Lehmann 1838).

** Frullania rizalii Piippo et S.Hatt., J. Hattori Bot. Lab. 72: 119, 1992 (Piippo and Tan 1992).

*** Frullania rostellata Mitt., Handb. N. Zeal. fl. 2: 755, 1867 (Hooker 1867).

** Frullania rubella Gottsche, Hedwigia 28 (3): 159, 1889 (Stephani 1889d).

** Frullania rubella var. elongata (Steph.) S.Hatt., J. Hattori Bot. Lab. 54: 166, 1983 (Hattori 1983). Bas.: Frullania elongata Steph., Sp. Hepat. (Stephani) 4: 423, 1910 (Stephani 1910b).

** Frullania rupicola Steph., Sp. Hepat. (Stephani) 6: 534, 1924 (Stephani 1924).

** Frullania saipanensis S.Hatt. et Koike, J. Hattori Bot. Lab. 75: 188, 1994 (Koike 1994).

*** Frullania schensiana C.Massal., Hepat. Shen-si: 40, 1897 (Massalongo 1897).

** Frullania schusteri S.Hatt., Beih. Nova Hedwigia 90: 154, 1988 (Hattori 1988b).

** Frullania scottiana S.Hatt., Mem. New York Bot. Gard. 45: 551, 1987 (Hattori 1987a).

** Frullania setchellii Pearson, Univ. Calif. Publ. Bot. 10 (4): 326, 1923 (Pearson 1923).

** Frullania shanensis Svihla, Bryologist 60 (4): 359, 1957 (Svihla 1957).

** Frullania sharpantha Udar et Ad.Kumar, Misc. Bryol. Lichenol. 9 (9): 192, 1983 (Udar and Kumar 1983a).

** Frullania sharpii S.Hatt., J. Hattori Bot. Lab. 38: 180, 1974 (Hattori 1974a).

** Frullania sinensis Steph., Nuovo Giorn. Bot. Ital. (n.ser.) 13 (4): 349, 1906 (Levier 1906).

** Frullania sinosphaerantha S.Hatt. et P.J.Lin, J. Hattori Bot. Lab. 59: 144, 1985 (Hattori and Lin 1985a).

** Frullania sphaerantha S.Hatt., J. Hattori Bot. Lab. 47: 99, 1980 (Hattori 1980d).

** Frullania sphaerolobulata S.H.Lin, Tunghai Journal 38: 104, 1997 (Lin and Chen 1997).

*** Frullania spinifera Taylor, London J. Bot. 5: 407, 1846 (Taylor 1846b).

** Frullania spinigastria S.Hatt., J. Hattori Bot. Lab. 45: 358, 1979 (Hattori 1979b).

** Frullania spiniplica S.Hatt., J. Hattori Bot. Lab. 36: 428, 1972 [1973] (Hattori 1972b).

*** Frullania spongiosa Steph., Hedwigia 33 (3): 147, 1894 (Stephani 1894d).

** Frullania squamuligera Spruce, Trans. & Proc. Bot. Soc. Edinburgh 15: 33, 1884 (Spruce 1884).

*** Frullania squarrosula (Hook.f. et Taylor) Gottsche, Lindenb. et Nees, Syn. Hepat. 3: 412, 1845 (Gottsche et al. 1845b). Bas.: Jungermannia squarrosula Hook.f. et Taylor, London J. Bot. 4: 88, 1845 (Hooker and Taylor 1845).

** Frullania subcaduca S.Hatt., J. Hattori Bot. Lab. 38: 267, 1974 (Hattori 1974c).

** Frullania subclavata Steph., Sp. Hepat. (Stephani) 4: 354, 1910 (Stephani 1910b).

** Frullania subnigricaulis S.Hatt., J. Hattori Bot. Lab. 37: 89, 1973 (Hattori 1973c).

** Frullania subnigricaulis var. subtruncata S.Hatt., J. Hattori Bot. Lab. 39: 308, 1975 (Hattori 1975d).

* Frullania subpedicellata S.Hatt., J. Hattori Bot. Lab. 47: 93, 1980 (Hattori 1980d). ^203^

** Frullania subsquarrosa S.Hatt., J. Hattori Bot. Lab. 36: 429, 1972 [1973] (Hattori 1972b).

** Frullania subvalida S.Hatt. et Thaithong, J. Jap. Bot. 53 (6): 173, 1978 (Hattori and Thaithong 1978c).

*** Frullania svihlana S.Hatt., J. Hattori Bot. Lab. 54: 180, 1983 (Hattori 1983).

* Frullania taiheizana Horik., J. Sci. Hiroshima Univ., Ser. B, Div. 2, Bot. 2: 241, 1934 (Horikawa 1934).

** Frullania tamsuina Steph., Sp. Hepat. (Stephani) 4: 444, 1910 (Stephani 1910b).

*** Frullania taradakensis Steph., Sp. Hepat. (Stephani) 4: 352, 1910 (Stephani 1910b).

** Frullania tenuirostris Steph., Sp. Hepat. (Stephani) 4: 462, 1911 (Stephani 1911e).

** Frullania togashiana S.Hatt., Bull. Natl. Sci. Mus. Tokyo, B 1 (3): 118, 1975 (Hattori 1975b).

** Frullania tubercularis S.Hatt. et P.J.Lin, J. Jap. Bot. 60 (4): 107, 1985 (Hattori and Lin 1985b).

*** Frullania usamiensis Steph., Bull. Herb. Boissier 5 (2): 91, 1897 (Stephani 1897b).

** Frullania valdiviensis J.B.Jack et Steph., Hedwigia 33 (3): 149, 1894 (Stephani 1894d).

** Frullania valida Steph., Sp. Hepat. (Stephani) 4: 402, 1910 (Stephani 1910b).

** Frullania variegata Steph., Hedwigia 33 (3): 149, 1894 (Stephani 1894d).

* Frullania victoriensis Steph., Sp. Hepat. (Stephani) 4: 418, 1910 (Stephani 1910b).

*** Frullania vittiana S.Hatt., Bryologist 90 (4): 368, 1987 [1988] (Hattori 1987b).

** Frullania wangii S.Hatt. et P.J.Lin, J. Hattori Bot. Lab. 59: 146, 1985 (Hattori and Lin 1985a).

*** Frullania yuennanensis Steph., Hedwigia 33 (3): 161, 1894 (Stephani 1894d).

** Frullania yuennanensis var. siamensis (N.Kitag., Thaithong et S.Hatt.) S.Hatt. et P.J.Lin, J. Hattori Bot. Lab. 59: 148, 1985 (Hattori and Lin 1985a). Bas.: Frullania siamensis N.Kitag., Thaithong et S.Hatt., J. Hattori Bot. Lab. 43: 452, 1977 [1978] (Hattori et al. 1977).

** Frullania yuzawana S.Hatt., J. Hattori Bot. Lab. 49: 157, 1981 (Hattori 1981a).

** Frullania zangii S.Hatt. et P.J.Lin, J. Hattori Bot. Lab. 59: 149, 1985 (Hattori and Lin 1985a).

** Frullania zennoskeana S.Hatt., J. Jap. Bot. 59 (10): 308, 1984 (Hattori 1984b).

** **sect. Acutilobae Verd.**, Ann. Bryol., Suppl. 1: 44, 1930 (Verdoorn 1930c).

** Frullania allanii E.A.Hodgs., Trans. & Proc. Roy. Soc. New Zealand 77 (3): 371, 1949 (Hodgson 1949).

*** Frullania clavata (Hook.f. et Taylor) Gottsche, Lindenb. et Nees, Syn. Hepat. 3: 428, 1845 (Gottsche et al. 1845b). Bas.: Jungermannia clavata Hook.f. et Taylor, London J. Bot. 4: 88, 1845 (Hooker and Taylor 1845).

** Frullania hamaticoma Steph., Hedwigia 28 (3): 158, 1889 (Stephani 1889d).

*** Frullania monocera (Hook.f. et Taylor) Gottsche, Lindenb. et Nees, Syn. Hepat. 3: 418, 1845 (Gottsche et al. 1845b). Bas.: Jungermannia monocera Hook.f. et Taylor, London J. Bot. 4: 89, 1845 (Hooker and Taylor 1845).

** Frullania monocera var. acutiloba (Mitt.) Hentschel et von Konrat, Phytotaxa 220 (2): 136, 2015 (Hentschel et al. 2015). Bas.: Frullania acutiloba Mitt., J. Proc. Linn. Soc., Bot. 5 (18): 120, 1860 [1861] (Mitten 1860c).

** Frullania monocera var. depauperata S.Hatt., J. Hattori Bot. Lab. 57: 419, 1984 (Hattori 1984a).

** Frullania monocera var. schiffneri (Verd.) S.Hatt., J. Hattori Bot. Lab. 46: 120, 1979 (Hattori 1979a). Bas.: Frullania acutiloba var. schiffneri Verd., Ann. Bryol. 2: 123, 1929 (Verdoorn 1929a).

*** Frullania monocera var. subhampeana (E.A.Hodgs.) Hentschel et von Konrat, Phytotaxa 220 (2): 136, 2015 (Hentschel et al. 2015). Bas.: Frullania subhampeana E.A.Hodgs., Trans. & Proc. Roy. Soc. New Zealand 77 (3): 370, 1949 (Hodgson 1949).

*** Frullania monocera var. undulata (Kamim.) Hentschel et von Konrat, Phytotaxa 220 (2): 136, 2015 (Hentschel et al. 2015). Bas.: Frullania undulata Kamim., J. Hattori Bot. Lab. 24: 50, 1961 (Kamimura 1961).

** Frullania osumiensis (S.Hatt.) S.Hatt., J. Hattori Bot. Lab. 16: 87, 1956 (Iwatsuki and Hattori 1956). Bas.: Frullania hampeana var. osumiensis S.Hatt., Bull. Tokyo Sci. Mus. 11: 144, 1944 (Hattori 1944d).

* Frullania pallidula S.Hatt., Beih. Nova Hedwigia 90: 152, 1988 (Hattori 1988b).

** Frullania pseudomonocera S.Hatt., J. Hattori Bot. Lab. 60: 216, 1986 (Hattori 1986e).

** Frullania seriata Gottsche, Hedwigia 28 (3): 160, 1889 (Stephani 1889d).

** Frullania spinistipula Steph., Sp. Hepat. (Stephani) 4: 463, 1911 (Stephani 1911e).

** Frullania streimannii S.Hatt., J. Hattori Bot. Lab. 54: 176, 1983 (Hattori 1983).

*** **sect. Australes Verd.**, Ann. Bryol., Suppl. 1: 58, 1930 (Verdoorn 1930c).

*** Frullania anomala E.A.Hodgs., Trans. & Proc. Roy. Soc. New Zealand 77 (3): 374, 1949 (Hodgson 1949).

* Frullania baileyana Steph., Sp. Hepat. (Stephani) 4: 417, 1910 (Stephani 1910b).

*** Frullania baladina Gottsche, Hedwigia 33 (3): 140, 1894 (Stephani 1894d). ^204^

* Frullania belmorensis Steph., J. & Proc. Roy. Soc. New South Wales 48 (1/2): 106, 1914 (Stephani and Watts 1914).

*** Frullania campanulata Sande Lac., Ned. Kruidk. Arch. 3: 422, 1854 [1855] (Sande Lacoste 1854).

** Frullania campanulata var. caduca Verd., Ann. Bryol., Suppl. 1: 41, 1930 (Verdoorn 1930c).

** Frullania campanulata var. malesiaca (Verd.) S.Hatt., Bull. Natl. Sci. Mus. Tokyo, B 1 (4): 158, 1975 (Hattori 1975f). Bas.: Frullania malesiaca Verd., Ann. Bryol., Suppl. 1: 59, 1930 (Verdoorn 1930c).

* Frullania cataractarum Steph., Sp. Hepat. (Stephani) 4: 657, 1911 (Stephani 1911e).

* Frullania crawfordii Steph., Hedwigia 33 (3): 143, 1894 (Stephani 1894d).

*** Frullania dentata S.Hatt., J. Hattori Bot. Lab. 38: 231, 1974 (Hattori 1974c).

** Frullania dentata var. secernens S.Hatt., J. Hattori Bot. Lab. 65: 422, 1988 (Hattori 1988a).

*** Frullania errans Verd., Ann. Bryol., Suppl. 1: 59, 1930 (Verdoorn 1930c).

** Frullania errans var. angulistipula S.Hatt., J. Hattori Bot. Lab. 36: 431, 1972 [1973] (Hattori 1972b).

*** Frullania fugax (Hook.f. et Taylor) Gottsche, Lindenb. et Nees, Syn. Hepat. 3: 445, 1845 (Gottsche et al. 1845b). Bas.: Jungermannia fugax Hook.f. et Taylor, London J. Bot. 4: 87, 1845 (Hooker and Taylor 1845). ^205^

** Frullania fulfordiae S.Hatt., Bryologist 90 (4): 365, 1987 [1988] (Hattori 1987b).

*** Frullania glomerata (Lehm. et Lindenb.) Nees et Mont., Ann. Sci. Nat. Bot. (sér. 2) 9: 46, 1838 (Montagne 1838). Bas.: Jungermannia glomerata Lehm. et Lindenb., Nov. Stirp. Pug. 5: 21, 1833 (Lehmann 1833).

*** Frullania incumbens Mitt., Bot. antarct. voy. II (Fl. Nov.-Zel. 2): 162, 1855 (Mitten 1855).

*** Frullania inflexa Mitt., J. Proc. Linn. Soc., Bot. 5 (18): 120, 1860 [1861] (Mitten 1860c).

** Frullania media (E.A.Hodgs.) S.Hatt., J. Hattori Bot. Lab. 54: 153, 1983 (Hattori 1983). Bas.: Frullania fugax var. media E.A.Hodgs., Trans. & Proc. Roy. Soc. New Zealand 77 (3): 375, 1949 (Hodgson 1949).

** Frullania mizutanii Kamim. et S.Hatt., J. Hattori Bot. Lab. 37: 524, 1973 (Hattori and Kamimura 1973).

*** Frullania obscurifolia Mitt., Philos. Trans. 168: 400, 1879 (Mitten 1879).

*** Frullania patagonica Steph., Kungl. Svenska Vetensk.-Akad. Handl. (n.ser.) 46 (9): 88, 1911 (Stephani 1911b).

** Frullania pentapleura Taylor, London J. Bot. 5: 402, 1846 (Taylor 1846b).

** Frullania polyptera Taylor, London J. Bot. 5: 401, 1846 (Taylor 1846b).

** Frullania polyptera var. angustata (Mitt.) S.Hatt., Bull. Natl. Sci. Mus. Tokyo, B 1 (2): 74, 1975 (Hattori 1975g). Bas.: Frullania angustata Mitt., J. Proc. Linn. Soc., Bot. 5 (18): 122, 1860 [1861] (Mitten 1860c).

** Frullania probosciphora Taylor, London J. Bot. 5: 402, 1846 (Taylor 1846b).

** Frullania pulchella Herzog, Rev. Bryol. Lichénol. 23 (1/2): 60, 1954 (Herzog 1954).

*** Frullania sinskeana J.J.Engel et B.C.Tan, J. Hattori Bot. Lab. 60: 335, 1986 (Tan and Engel 1986). *Nom. nov. pro Frullania spathulistipa* Steph., Sp. Hepat. (Stephani) 4: 415, 1910 (Stephani 1910b), *nom. illeg*.

** Frullania socotrana Mitt., Trans. Roy Soc. Edinburgh 31: 335, 1888 (Mitten 1888).

* Frullania solanderiana Colenso, Trans. & Proc. New Zealand Inst. 21: 75, 1889 (Colenso 1889). ^206^

** Frullania subincumbens S.Hatt., Bryologist 90 (4): 367, 1987 [1988] (Hattori 1987b).

* Frullania subtropica Steph., Sp. Hepat. (Stephani) 4: 416, 1910 (Stephani 1910b).

* Frullania tjibodensis S.Hatt. et Thaithong, J. Jap. Bot. 52 (10): 289, 1977 (Hattori and Thaithong 1977). ^207^

** Frullania tuyamae S.Hatt. et Thaithong, J. Jap. Bot. 53 (6): 175, 1978 (Hattori and Thaithong 1978c).

*** **sect. Frullania**

*** Frullania appalachiana R.M.Schust., Phytologia 53 (5): 366, 1983 (Schuster 1983b).

*** Frullania azorica Sim-Sim, Sérgio, Mues et Kraut, Cryptog. Bryol. Lichénol. 16 (2): 112, 1995 (Sim-Sim et al. 1995).

*** Frullania catalinae A.Evans, Trans. Connecticut Acad. Arts 10 (1): 11, 1899 (Evans 1899).

*** Frullania dilatata (L.) Dumort., Recueil Observ. Jungerm.: 13, 1835 (Dumortier 1835). Bas.: Jungermannia dilatata L., Sp. Pl. 1: 1133, 1753 (Linnaeus 1753).

** Frullania dilatata subsp. asiatica S.Hatt., J. Jap. Bot. 57 (9): 258, 1982 (Hattori 1982b).

*** Frullania eboracensis Lehm., Nov. Stirp. Pug. 8: 14, 1844 (Lehmann 1844). ^208^

*** Frullania ericoides (Nees) Mont., Ann. Sci. Nat. Bot. (sér. 2) 12: 51, 1839 (Montagne 1839c). Bas.: Jungermannia ericoides Nees, Fl. Bras. (Martius) 1 (1): 346, 1833 (Nees 1833a). ^209^

* Frullania ericoides var. laxa (Gottsche, Lindenb. et Nees) Schiffn., Consp. Hepat. Arch. Ind.: 324, 1898 (Schiffner 1898b). Bas.: Frullania squarrosa γ laxa Gottsche, Lindenb. et Nees, Syn. Hepat. 5: 772, 1847 (Gottsche et al. 1847).

* Frullania ericoides var. minor Kamim., Bull. Kochi Gakuen Jun. Coll. 2: 22, 1971 (Kamimura 1971).

** Frullania ericoides var. verrucosa (Kamim.) Hentschel et von Konrat, Phytotaxa 220 (2): 134, 2015 (Hentschel et al. 2015). Bas.: Frullania squarrosa var. verrucosa Kamim., J. Hattori Bot. Lab. 24: 19, 1961 (Kamimura 1961).

*** Frullania fragilifolia (Taylor) Gottsche, Lindenb. et Nees, Syn. Hepat. 3: 437, 1845 (Gottsche et al. 1845b). Bas.: Jungermannia fragilifolia Taylor, Ann. Mag. Nat. Hist. 12 (76): 172, 1843 (Taylor 1843).

** Frullania fuegiana Steph., Sp. Hepat. (Stephani) 4: 428, 1910 (Stephani 1910b).

** Frullania hattoriana J.D.Godfrey et G.Godfrey, J. Hattori Bot. Lab. 48: 321, 1980 (Godfrey and Godfrey 1980).

** Frullania koponenii S.Hatt., Ann. Bot. Fenn. 15 (2): 111, 1978 (Koponen et al. 1978).

*** Frullania muscicola Steph., Hedwigia 33 (3): 146, 1894 (Stephani 1894d).

*** Frullania oakesiana Austin, Proc. Acad. Nat. Sci. Philadelphia 21: 225, 1869 (Austin 1869).

** Frullania oakesiana subsp. takayuensis (Steph.) R.M.Schust., Hepat. Anthocerotae N. Amer. 5: 195, 1992 (Schuster 1992b). Bas.: Frullania takayuensis Steph., Sp. Hepat. (Stephani) 4: 399, 1910 (Stephani 1910b).

*** Frullania parvistipula Steph., Sp. Hepat. (Stephani) 4: 397, 1910 (Stephani 1910b).

** Frullania sabaliana R.M.Schust., Phytologia 53 (5): 365, 1983 (Schuster 1983b).

** Frullania semivillosa Lindenb. et Gottsche, Syn. Hepat. 5: 774, 1847 (Gottsche et al. 1847).

** Frullania stylifera (R.M.Schust.) R.M.Schust., Hepat. Anthocerotae N. Amer. 5: 210, 1992 (Schuster 1992b). Bas.: Frullania inflata var. stylifera R.M.Schust., Phytologia 53 (5): 366, 1983 (Schuster 1983b).

* Frullania subdilatata C.Massal., Nuovo Giorn. Bot. Ital. (n.ser.) 13 (4): 349, 1906 (Levier 1906).

*** Frullania virginica Lehm., Nov. Stirp. Pug. 8: 19, 1844 (Lehmann 1844).

** **sect. Irregulares E.A.Hodgs. ex S.Hatt.**, J. Hattori Bot. Lab. 54: 143, 1983 (Hattori 1983).

* Frullania astrolabea Steph., Sp. Hepat. (Stephani) 4: 460, 1910 (Stephani 1910b). ^210^

*** Frullania deplanata Mitt., Bot. antarct. voy. II (Fl. Nov.-Zel. 2): 161, 1855 (Mitten 1855).

*** Frullania morobensis S.Hatt. et Streimann, J. Hattori Bot. Lab. 59: 109, 1985 (Hattori and Streimann 1985).

*** Frullania patula Mitt., Bot. antarct. voy. II (Fl. Nov.-Zel. 2): 159, 1854 (Mitten 1854).

*** Frullania scandens Mont., Ann. Sci. Nat. Bot. (sér. 2) 19: 258, 1843 (Montagne 1843).

** **sect. Planae R.M.Schust.**, Phytologia 57 (5): 372, 1985 (Schuster 1985a).

*** Frullania plana Sull., Mem. Amer. Acad. Arts (n.ser.) 4: 175, 1849 (Sullivant 1849).

*** **subg. Homotropantha Spruce**, Trans. & Proc. Bot. Soc. Edinburgh 15: 35, 1884 (Spruce 1884).

*** Frullania deflexa Mitt., Bonplandia 10 (2): 19, 1862 (Mitten 1862).

*** Frullania integristipula (Nees) Nees, Syn. Hepat. 3: 431, 1845 (Gottsche et al. 1845b). Bas.: Jungermannia integristipula Nees, Enum. Pl. Crypt. Javae: 54, 1830 (Nees 1830).

** Frullania integristipula var. emarginata Verd., Ann. Bryol. 2: 153, 1929 (Verdoorn 1929a).

** Frullania macrophylla S.Hatt., J. Hattori Bot. Lab. 47: 220, 1980 (Hattori 1980a).

** Frullania sabahana S.Hatt., J. Hattori Bot. Lab. 40: 493, 1976 (Hattori 1976d).

** Frullania sackawana Steph., Bull. Herb. Boissier 5 (2): 91, 1897 (Stephani 1897b).

** Frullania sarawakensis S.Hatt., J. Hattori Bot. Lab. 40: 496, 1976 (Hattori 1976d).

** Frullania umbonata Mitt., Sp. Hepat. (Stephani) 4: 579, 1911 (Stephani 1911e).

*** Frullania utriculata Steph., Hedwigia 33 (3): 152, 1894 (Stephani 1894d).

*** **sect. Fallaces Verd.**, Rev. Bryol. Lichénol. 1: 112, 1928 (Verdoorn 1928a).

* Frullania fallax Gottsche, Syn. Hepat. 3: 432, 1845 (Gottsche et al. 1845b).

*** Frullania intermedia (Reinw., Blume et Nees) Nees, Syn. Hepat. 3: 434, 1845 (Gottsche et al. 1845b). Bas.: Jungermannia intermedia Reinw., Blume et Nees, Nova Acta Phys.-Med. Acad. Caes. Leop.-Carol. Nat. Cur. 12 (1): 218, 1824 [1825] (Reinwardt et al. 1824a). ^211^

** Frullania intermedia subsp. morokensis (Steph.) S.Hatt., J. Hattori Bot. Lab. 47: 194, 1980 (Hattori 1980a). Bas.: Frullania morokensis Steph., Sp. Hepat. (Stephani) 4: 578, 1911 (Stephani 1911e).

* Frullania intermedia var. non-apiculata S.Hatt., J. Hattori Bot. Lab. 39: 291, 1975 (Hattori 1975d). ^212^

** Frullania novoguineensis Schiffn., Leberm., Forschungsr. Gazelle 4 (4): 37, 1890 (Schiffner 1890).

** Frullania regularis Schiffn., Leberm., Forschungsr. Gazelle 4 (4): 38, 1890 (Schiffner 1890).

*** **sect. Nodulosae Verd.**, Rev. Bryol. Lichénol. 1: 116, 1928 (Verdoorn 1928a).

* Frullania brotheri Steph., Hedwigia 33 (3): 150, 1894 (Stephani 1894d). ^213^

** Frullania hamata Steph., Sp. Hepat. (Stephani) 4: 582, 1911 (Stephani 1911e).

** Frullania leeuwenii Verd., Nova Guinea 14: 545, 1930 (Verdoorn 1930b).

*** Frullania nodulosa (Reinw., Blume et Nees) Nees, Syn. Hepat. 3: 433, 1845 (Gottsche et al. 1845b). Bas.: Jungermannia nodulosa Reinw., Blume et Nees, Nova Acta Phys.-Med. Acad. Caes. Leop.-Carol. Nat. Cur. 12 (1): 217, 1824 [1825] (Reinwardt et al. 1824a).

** **sect. Remotilobae Verd.**, Rev. Bryol. Lichénol. 1: 119, 1928 (Verdoorn 1928a).

*** Frullania heteromorpha Schiffn., Leberm., Forschungsr. Gazelle 4 (4): 38, 1890 (Schiffner 1890).

** Frullania remotiloba Steph., Hedwigia 33 (3): 152, 1894 (Stephani 1894d).

* **subg. Mammillosae S.Hatt.**, J. Hattori Bot. Lab. 60: 226, 1986 (Hattori 1986e).

** Frullania huerlimannii S.Hatt., Bull. Natl. Sci. Mus. Tokyo, B 2 (3): 84, 1976 (Hattori 1976b).

* Frullania huerlimannii var. dioica S.Hatt., J. Hattori Bot. Lab. 57: 413, 1984 (Hattori 1984a).

** Frullania involvens S.Hatt. et Kamim., J. Hattori Bot. Lab. 37: 526, 1973 (Hattori and Kamimura 1973).

** Frullania iriomotensis S.Hatt., J. Jap. Bot. 55 (5): 133, 1980 (Hattori 1980c).

** Frullania mammillosa S.Hatt., J. Hattori Bot. Lab. 43: 424, 1977 [1978] (Hattori 1977b).

*** Frullania meijeri S.Hatt., Bull. Natl. Sci. Mus. Tokyo (n.ser.) 17 (4): 307, 1974 (Hattori 1974b).

** Frullania notarisii Steph., Sp. Hepat. (Stephani) 4: 651, 1911 (Stephani 1911e).

** Frullania papulosa Steph., Sp. Hepat. (Stephani) 4: 654, 1911 (Stephani 1911e).

** Frullania rudolfiana S.Hatt., J. Hattori Bot. Lab. 36: 437, 1972 [1973] (Hattori 1972b).

*** Frullania thiersiae S.Hatt., Beih. Nova Hedwigia 90: 156, 1988 (Hattori 1988b).

** Frullania tixieri S.Hatt., J. Jap. Bot. 51 (7): 193, 1976 (Hattori 1976a).

*** **subg. Meteoriopsis Spruce**, Trans. & Proc. Bot. Soc. Edinburgh 15: 37, 1884 (Spruce 1884).

* Frullania caldensis Ångstr., Öfvers. Kongl. Vetensk.-Akad. Förh. 33 (7): 89, 1876 [1877] (Ångström 1876).

** Frullania evoluta Mitt., J. Proc. Linn. Soc., Bot. 5 (18): 122, 1860 [1861] (Mitten 1860c).

*** Frullania tagawana (S.Hatt. et Thaithong) S.Hatt., J. Hattori Bot. Lab. 59: 160, 1985 (Hattori and Lin 1985a). Bas.: Frullania evoluta var. tagawana S.Hatt. et Thaithong, J. Hattori Bot. Lab. 43: 441, 1977 [1978] (Hattori et al. 1977).

*** **sect. Intumescentes R.M.Schust.**, Phytologia 57 (5): 370, 1985 (Schuster 1985a).

*** Frullania aculeata Taylor, London J. Bot. 5: 407, 1846 (Taylor 1846b).

** Frullania ambronnii Steph., Biblioth. Bot. 87 (2): 242, 1916 (Stephani 1916a).

*** Frullania atrata (Sw.) Nees ex Mont., Ann. Sci. Nat. Bot. (sér. 2) 12: 51, 1839 (Montagne 1839c). Bas.: Jungermannia atrata Sw., Prodr. (Swartz): 144, 1788 (Swartz 1788).

*** Frullania beyrichiana (Lehm. et Lindenb.) Lehm. et Lindenb., Syn. Hepat. 3: 460, 1845 (Gottsche et al. 1845b). Bas.: Jungermannia beyrichiana Lehm. et Lindenb., Nov. Stirp. Pug. 5: 25, 1833 (Lehmann 1833).

*** Frullania bicornistipula Spruce, Trans. & Proc. Bot. Soc. Edinburgh 15: 46, 1884 (Spruce 1884).

*** Frullania brasiliensis Raddi, Critt. Brasil.: 12, 1822 (Raddi 1822). ^214^

** Frullania brasiliensis var. elegantula Spruce, Trans. & Proc. Bot. Soc. Edinburgh 15: 50, 1884 (Spruce 1884).

* Frullania breuteliana Gottsche, Syn. Hepat. 3: 461, 1845 (Gottsche et al. 1845b). ^215^

* Frullania compacta Gottsche, Sp. Hepat. (Stephani) 4: 493, 1911 (Stephani 1911e). ^216^

* Frullania crenulifolia J.B.Jack et Steph., Hedwigia 31 (1): 14, 1892 (Jack and Stephani 1892).

** Frullania crispiloba Steph., Hedwigia 33 (3): 156, 1894 (Stephani 1894d).

** Frullania curviramea Steph., Sp. Hepat. (Stephani) 4: 684, 1911 (Stephani 1911e).

*** Frullania ecuadorensis Steph., Sp. Hepat. (Stephani) 4: 526, 1911 (Stephani 1911e).

** Frullania formosa Spruce, Trans. & Proc. Bot. Soc. Edinburgh 15: 46, 1884 (Spruce 1884).

* Frullania granatensis Gottsche, Ann. Sci. Nat. Bot. (sér. 5) 1: 173 (79), 1864 (Gottsche 1864). ^217^

*** Frullania griffithsiana Gottsche, Syn. Hepat. 4: 466, 1846 (Gottsche et al. 1846).

* Frullania guadalupensis Gottsche, Sp. Hepat. (Stephani) 4: 496, 1911 (Stephani 1911e).

* Frullania gualaquizana Steph., Sp. Hepat. (Stephani) 4: 531, 1911 (Stephani 1911e).

** Frullania hamiflora Herzog et L.Clark, Bryologist 56 (3): 180, 1953 (Clark and Schultz 1953).

* Frullania humilis Spruce, Mem. Torrey Bot. Club 1 (3): 119, 1890 (Spruce 1890).

*** Frullania intumescens (Lehm. et Lindenb.) Lehm. et Lindenb., Syn. Hepat. 3: 460, 1845 (Gottsche et al. 1845b). Bas.: Jungermannia intumescens Lehm. et Lindenb., Nov. Stirp. Pug. 6: 52, 1834 (Lehmann 1834).

* Frullania laticaulis Spruce, Mem. Torrey Bot. Club 1 (3): 120, 1890 (Spruce 1890).

*** Frullania lobatohastata Steph., Sp. Hepat. (Stephani) 4: 499, 1911 (Stephani 1911e).

* Frullania longistipula var. apiculata Demaret et Vanden Berghen, Bull. Jard. Bot. État Bruxelles 20 (1): 4, 1950 (Demaret and Vanden Berghen 1950).

*** Frullania macrocephala (Lehm. et Lindenb.) Lehm. et Lindenb., Syn. Hepat. 3: 460, 1845 (Gottsche et al. 1845b). Bas.: Jungermannia macrocephala Lehm. et Lindenb., Nov. Stirp. Pug. 5: 20, 1833 (Lehmann 1833).

** Frullania madothecoides Spruce, Trans. & Proc. Bot. Soc. Edinburgh 15: 47, 1884 (Spruce 1884).

** Frullania meridana Steph., Sp. Hepat. (Stephani) 4: 500, 1911 (Stephani 1911e).

* Frullania microcephala Gottsche, Mexik. Leverm.: 251, 1863 (Gottsche 1863).

*** Frullania montagnei Gottsche, Syn. Hepat. 3: 456, 1845 (Gottsche et al. 1845b).

** Frullania moritziana Lindenb. et Gottsche, Syn. Hepat. 5: 782, 1847 (Gottsche et al. 1847).

** Frullania osculatiana De Not., Mem. Reale Accad. Sci. Torino (ser. 2) 16: 236, 1857 (De Notaris 1857).

*** Frullania paradoxa Lehm. et Lindenb., Syn. Hepat. 3: 462, 1845 (Gottsche et al. 1845b).

** Frullania pearceana Steph., Sp. Hepat. (Stephani) 4: 515, 1911 (Stephani 1911e).

*** Frullania pittieri Steph., Primit. fl. costar.: 113, 1892 [1893] (Stephani 1892e).

** Frullania rigescens Spruce, Trans. & Proc. Bot. Soc. Edinburgh 15: 52, 1884 (Spruce 1884).

*** Frullania setigera Steph., Hedwigia 33 (3): 159, 1894 (Stephani 1894d).

** Frullania speciosa Herzog, Memoranda Soc. Fauna Fl. Fennica 25: 59, 1950 (Herzog 1950c).

** Frullania supradecomposita (Lehm. et Lindenb.) Lehm. et Lindenb., Syn. Hepat. 3: 431, 1845 (Gottsche et al. 1845b). Bas.: Jungermannia supradecomposita Lehm. et Lindenb., Nov. Stirp. Pug. 5: 23, 1833 (Lehmann 1833).

* Frullania trianae Gottsche, Ann. Sci. Nat. Bot. (sér. 5) 1: 173, 1864 (Gottsche 1864).

** Frullania triquetra Lindenb. et Gottsche, Syn. Hepat. 5: 780, 1847 (Gottsche et al. 1847).

* Frullania trollii Herzog, Beih. Bot. Centralbl. 61B (3): 575, 1942 (Herzog 1942d).

*** Frullania uleana Steph., Hedwigia 33 (3): 155, 1894 (Stephani 1894d).

** **sect. Meteoriopsis Uribe, von Konrat et Hentschel**, Phytotaxa 220 (2): 132, 2015 (Hentschel et al. 2015).

*** Frullania convoluta Lindenb. et Hampe, Linnaea 24 (3): 303, 1851 [1852] (Hampe 1851b).

* Frullania convoluta var. ampliata Herzog, Rev. Bryol. Lichénol. 20 (1/2): 129, 1951 [1952] (Herzog 1951a).

*** Frullania darwinii Gradst. et Uribe, Cryptog. Bryol. 25 (4): 296, 2004 (Uribe 2004).

*** Frullania dulimensis Uribe, Cryptog. Bryol. 27 (3): 309, 2006 (Uribe 2006).

*** Frullania grandifolia Steph., Sp. Hepat. (Stephani) 4: 684, 1911 (Stephani 1911e).

*** Frullania peruviana Gottsche, Syn. Hepat. 4: 465, 1846 (Gottsche et al. 1846).

*** Frullania phalangiflora Steph., Biblioth. Bot. 87 (2): 247, 1916 (Stephani 1916a).

*** Frullania weberbaueri Steph., Sp. Hepat. (Stephani) 4: 510, 1911 (Stephani 1911e).

*** **sect. Obtusilobae Verd.**, Ann. Bryol., Suppl. 1: 81, 1930 (Verdoorn 1930c).

** Frullania angulata Mitt., J. Proc. Linn. Soc., Bot. 7 (27): 169, 1863 (Mitten 1863).

** Frullania angulata var. laciniata Vanden Berghen, Bull. Jard. Bot. Natl. Belg. 46 (1/2): 72, 1976 (Vanden Berghen 1976b).

** Frullania apicalis Mitt., Philos. Trans. 168: 401, 1879 (Mitten 1879).

** Frullania apicalis var. camerunensis Vanden Berghen, Bull. Jard. Bot. Natl. Belg. 46 (1/2): 53, 1976 (Vanden Berghen 1976b).

* Frullania borbonica Lindenb., Syn. Hepat. 3: 455, 1845 (Gottsche et al. 1845b). ^218^

*** Frullania capensis Gottsche, Syn. Hepat. 3: 449, 1845 (Gottsche et al. 1845b).

** Frullania donnellii Austin, Bull. Torrey Bot. Club 6 (52): 301, 1879 (Austin 1879).

** Frullania eplicata Steph., Sp. Hepat. (Stephani) 4: 679, 1911 (Stephani 1911e).

** Frullania imerinensis Steph., Sp. Hepat. (Stephani) 4: 484, 1911 (Stephani 1911e).

*** Frullania kunzei (Lehm. et Lindenb.) Lehm. et Lindenb., Syn. Hepat. 3: 449, 1845 (Gottsche et al. 1845b). Bas.: Jungermannia kunzei Lehm. et Lindenb., Nov. Stirp. Pug. 6: 50, 1834 (Lehmann 1834).

** Frullania kunzei var. maritima R.M.Schust., Phytotaxa 220 (2): 135, 2015 (Hentschel et al. 2015). Based on: Frullania kunzei var. maritima R.M.Schust., J. Hattori Bot. Lab. 70: 145, 1991 (Schuster 1991b), *nom. inval*.

** Frullania longistipula Steph., Bull. Soc. Roy. Bot. Belgique, Compt. Rend. 30 (2): 199, 1891 [1892] (Stephani 1891b).

*** Frullania meyeniana Lindenb., Syn. Hepat. 3: 455, 1845 (Gottsche et al. 1845b).

** Frullania meyeniana var. dioica S.Hatt., J. Hattori Bot. Lab. 43: 426, 1977 [1978] (Hattori 1977b).

** Frullania onraedtii Vanden Berghen, Bull. Jard. Bot. Natl. Belg. 46 (1/2): 60, 1976 (Vanden Berghen 1976b).

** Frullania papuana Verd., Ann. Bryol., Suppl. 1: 82, 1930 (Verdoorn 1930c).

*** Frullania schimperi Nees, Syn. Hepat. 3: 454, 1845 (Gottsche et al. 1845b). ^219^

** Frullania schimperi var. laciniata Vanden Berghen, Bull. Jard. Bot. Natl. Belg. 46 (1/2): 59, 1976 (Vanden Berghen 1976b).

*** **subg. Microfrullania (R.M.Schust.) R.M.Schust.**, Hepat. Anthocerotae N. Amer. 5: 34, 1992 (Schuster 1992b). Bas.: Neohattoria subg. Microfrullania R.M.Schust., J. Hattori Bot. Lab. 33: 280, 1970 (Schuster 1970a).

*** Frullania fertilis De Not., Mem. Reale Accad. Sci. Torino (ser. 2) 16: 235, 1857 (De Notaris 1857).

*** Frullania knightbridgei von Konrat et de Lange, PhytoKeys 8: 28, 2012 (von Konrat et al. 2012b).

*** Frullania magellanica F.Weber et Nees, Syn. Hepat. 3: 446, 1845 (Gottsche et al. 1845b). *Nom. nov. pro Jungermannia magellanica* Spreng. Ann. Wetterauischen Ges. Gesammte Naturk. 1: 25, 1809 (Sprengel 1809), *nom. illeg*.

** Frullania magellanica subsp. tristaniana (S.W.Arnell) Váňa et J.J.Engel, Mem. New York Bot. Gard. 105: 59, 2013 (Váňa and Engel 2013). Bas.: Frullania tristaniana S.W.Arnell, Results Norweg. Sci. Exped. Tristan da Cunha 42: 9, 1958 (Arnell 1958b).

* Frullania matafaoica H.A.Mill., Phytologia 47 (4): 322, 1981 (Miller 1981). *Nom. nov. pro Frullania minutissima* Pearson, Amer. Samoa: 140, 1924 (Pearson 1924a), *nom. illeg*.

*** Frullania toropuku von Konrat, de Lange et Larraín, Polish Bot. J. 58 (2): 439, 2013 (von Konrat et al. 2013).

*** **sect. Amphijubula (R.M.Schust.) von Konrat, Hentschel, Heinrichs et Braggins**, Bryologist 114 (1): 53, 2011 (von Konrat et al. 2011). Bas.: Amphijubula R.M.Schust., J. Hattori Bot. Lab. 33: 298, 1970 (Schuster 1970a).

*** Frullania lobulata (Hook.) Hook. et Nees, Syn. Hepat. 3: 445, 1845 (Gottsche et al. 1845b). Bas.: Jungermannia lobulata Hook., Musci Exot. 2: tab. 119, 1820 (Hooker 1820).

*** Frullania microcaulis Gola, Nuovo Giorn. Bot. Ital. (n.ser.) 29 (1/4): 172, 1922 [1923] (Gola 1922).

*** Frullania truncatistyla von Konrat, Hentschel, Heinrichs et Braggins, Bryologist 114 (1): 63, 2011 (von Konrat et al. 2011).

*** **sect. Microfrullania (R.M.Schust.) von Konrat et Hentschel**, Phytotaxa 220 (2): 133, 2015 (Hentschel et al. 2015). Bas.: Neohattoria sect. Microfrullania R.M.Schust., J. Hattori Bot. Lab. 33: 288, 1970 (Schuster 1970a).

*** Frullania chevalieri (R.M.Schust.) R.M.Schust., Hepat. Anthocerotae N. Amer. 5: 34, 1992 (Schuster 1992b). Bas.: Neohattoria chevalieri R.M.Schust., J. Hattori Bot. Lab. 33: 289, 1970 (Schuster 1970a). ^220^

*** Frullania microscopica Pearson, J. Linn. Soc., Bot. 46 (305): 33, 1922 (Pearson 1922b).

** Frullania neocaledonica J.J.Engel, Novon 9 (3): 344, 1999 (Engel and Smith Merrill 1999a). *Nom. nov. pro Neohattoria caledonica* R.M.Schust., J. Hattori Bot. Lab. 33: 291, 1970 (Schuster 1970a).

*** Frullania parhamii (R.M.Schust.) R.M.Schust. ex von Konrat, L.Söderstr. et A.Hagborg, Telopea 13 (3): 407, 2011 (Söderström et al. 2011a). Bas.: Neohattoria parhamii R.M.Schust., J. Hattori Bot. Lab. 26: 243, 1963 (Schuster 1963b).

*** **sect. Regulares Verd.**, Ann. Bryol., Suppl. 1: 133, 1930 (Verdoorn 1930c).

*** Frullania junghuhniana Gottsche, Syn. Hepat. 3: 444, 1845 (Gottsche et al. 1845b). ^221^

** Frullania junghuhniana var. bisexualis S.Hatt., J. Hattori Bot. Lab. 40: 485, 1976 (Hattori 1976d).

** Frullania junghuhniana var. tenella (Sande Lac.) Grolle et S.Hatt., Misc. Bryol. Lichenol. 9 (6): 123, 1982 (Hattori 1982a). Bas.: Frullania tenella Sande Lac., Ned. Kruidk. Arch. 3: 423, 1854 [1855] (Sande Lacoste 1854).

** Frullania mcveanii S.Hatt., J. Hattori Bot. Lab. 37: 55, 1973 (Hattori 1973a).

** Frullania pseudomeyeniana S.Hatt., J. Hattori Bot. Lab. 60: 231, 1986 (Hattori 1986e).

*** Frullania rostrata (Hook.f. et Taylor) Gottsche, Lindenb. et Nees, Syn. Hepat. 3: 445, 1845 (Gottsche et al. 1845b). Bas.: Jungermannia rostrata Hook.f. et Taylor, London J. Bot. 4: 87, 1845 (Hooker and Taylor 1845). ^222^

** Frullania scalaris S.Hatt., J. Hattori Bot. Lab. 43: 432, 1977 [1978] (Hattori 1977b).

** **subg. Saccophora Verd.**, Ann. Bryol. 2: 121, 1929 (Verdoorn 1929a).

*** Frullania gaudichaudii (Nees et Mont.) Nees et Mont., Syn. Hepat. 3: 435, 1845 (Gottsche et al. 1845b). Bas.: Jubula gaudichaudii Nees et Mont., Ann. Sci. Nat. Bot. (sér. 2) 5: 64, 1836 (Nees and Montagne 1836).

** Frullania gaudichaudii var. ceylanica (Nees) S.Hatt., J. Hattori Bot. Lab. 47: 104, 1980 (Hattori 1980d). Bas.: Frullania ceylanica Nees, Syn. Hepat. 3: 436, 1845 (Gottsche et al. 1845b).

*** Frullania hedrantha S.Hatt. et Kamim., J. Hattori Bot. Lab. 37: 519, 1973 (Hattori and Kamimura 1973).

** Frullania immersa Steph., Bot. Jahrb. Syst. 23 (1/2, 3): 315, 1896 (Stephani 1896a).

** Frullania pancheri Gottsche, Hedwigia 33 (3): 159, 1894 (Stephani 1894d).

** Frullania papillilobula S.Hatt., Bull. Natl. Sci. Mus. Tokyo, B 1 (4): 141, 1975 (Hattori 1975f).

** Frullania sublignosa Steph., Hedwigia 33 (3): 148, 1894 (Stephani 1894d).

* **subg. Steerea (S.Hatt. et Kamim.) R.M.Schust.**, Hepat. Anthocerotae N. Amer. 5: 32, 1992 (Schuster 1992b). Bas.: Steerea S.Hatt. et Kamim., J. Hattori Bot. Lab. 34: 429, 1971 (Hattori and Kamimura 1971).

*** Frullania clemensiana Verd., Ned. Kruidk. Arch. (ser. 3) 42 (2): 493, 1932 (Verdoorn 1932b).

*** **subg. Thyopsiella Spruce**, Trans. & Proc. Bot. Soc. Edinburgh 15: 41, 1884 (Spruce 1884).

*** Frullania acicularis Hentschel et von Konrat, Phytotaxa 220 (2): 134, 2015 (Hentschel et al. 2015). *Nom. nov. pro* Frullania tamarisci var. azorica J.-P.Frahm, Trop. Bryol. 27: 102, 2006 (Frahm 2006). ^223^

** Frullania alstonii Verd., Ann. Bryol., Suppl. 1: 76, 1930 (Verdoorn 1930c).

** Frullania aoshimensis Horik., Sci. Rep. Tôhoku Imp. Univ., Ser. 4, Biol. 4 (1): 64, 1929 (Horikawa 1929b).

*** Frullania appendiculata Steph., Bull. Herb. Boissier 5 (2): 88, 1897 (Stephani 1897b). ^224^

*** Frullania asagrayana Mont., Ann. Sci. Nat. Bot. (sér. 2) 18: 14, 1842 (Montagne 1842b). ^225^

*** Frullania calcarifera Steph., Bol. Soc. Brot. 4: 241, 1886 [1887] (Henriques 1886). ^226^

*** Frullania californica (M.Howe) A.Evans, Trans. Connecticut Acad. Arts 10 (1): 25, 1899 (Evans 1899). Bas.: Frullania asagrayana var. californica M.Howe, Erythea 2 (6): 98, 1894 (Howe 1894).

*** Frullania densiloba Steph. ex A.Evans, Proc. Wash. Acad. Sci. 8: 157, 1906 (Evans 1906b).

*** Frullania franciscana M.Howe, Erythea 2 (6): 99, 1894 (Howe 1894).

** Frullania iwatsukii S.Hatt., J. Hattori Bot. Lab. 35: 240, 1972 (Hattori 1972a).

*** Frullania microphylla (Gottsche) Pearson, J. Bot. 32: 328, 1894 (Pearson 1894). Bas.: Frullania tamarisci var. microphylla Gottsche, Hepat. Eur., Leberm. 21-22: no. 109 [209], 1862 (Rabenhorst 1862).

*** Frullania moniliata (Reinw., Blume et Nees) Mont., Ann. Sci. Nat. Bot. (sér. 2) 18: 13, 1842 (Montagne 1842b). Bas.: Jungermannia moniliata Reinw., Blume et Nees, Nova Acta Phys.-Med. Acad. Caes. Leop.-Carol. Nat. Cur. 12 (1): 224, 1824 [1825] (Reinwardt et al. 1824a). ^227^

*** Frullania nisquallensis Sull., Mem. Amer. Acad. Arts (n.ser.) 4: 175, 1849 (Sullivant 1849).

*** Frullania polysticta Lindenb., Syn. Hepat. 3: 440, 1845 (Gottsche et al. 1845b).

** Frullania pseudoalstonii Tsudo et J.Haseg., Bryol. Res. 9 (3): 44, 2006 (Tsudo and Hasegawa 2006).

** Frullania punctata Reimers, Hedwigia 71 (1/2): 36, 1931 (Reimers 1931).

** Frullania schaefer-verwimpii Yuzawa et S.Hatt., J. Jap. Bot. 64 (2): 37, 1989 (Yuzawa and Hattori 1989).

** Frullania selwyniana Pearson, List. Canad. Hepat.: 1, 1890 (Pearson 1890).

*** Frullania sergiae Sim-Sim, Fontinha, Mues et Lion, Nova Hedwigia 71 (1/2): 186, 2000 (Sim-Sim et al. 2000).

*** Frullania subarctica Vilnet, Borovich. et Bakalin, Phytotaxa 173 (1): 67, 2014 (Vilnet et al. 2014). ^228^

*** Frullania tamarisci (L.) Dumort., Recueil Observ. Jungerm.: 13, 1835 (Dumortier 1835). Bas.: Jungermannia tamarisci L., Sp. Pl. 1: 1134, 1753 (Linnaeus 1753).

*** Frullania teneriffae (F.Weber) Nees, Naturgesch. Eur. Leberm. 3: 239, 1838 (Nees 1838b). Bas.: Jungermannia teneriffae F.Weber, Hist. Musc. Hepat. Prodr.: 23, 1815 (Weber 1815).

** Frullania trigona L.Clark, Jovet-Ast et Frye, Bryologist 50 (1): 52, 1947 (Clark et al. 1947).

***Incertae sedis***

* Frullania affinis Nees et Mont., Ann. Sci. Nat. Bot. (sér. 2) 19: 257, 1843 (Montagne 1843). ^229^

* Frullania allionii Steph., Sp. Hepat. (Stephani) 4: 394, 1910 (Stephani 1910b).

* Frullania alpina Steph., Sp. Hepat. (Stephani) 4: 533, 1911 (Stephani 1911e).

** Frullania alternans Nees, Syn. Hepat. 3: 430, 1845 (Gottsche et al. 1845b).

* Frullania apertilobula Gerola, Lav. Bot. Ist. Bot. Univ. Padova 12: 478, 1947 (Gerola 1947).

** Frullania armata Herzog et L.Clark, Bryologist 57 (1): 36, 1954 (Clark 1954).

*** Frullania bolanderi Austin, Proc. Acad. Nat. Sci. Philadelphia 21: 226, 1869 (Austin 1869).

*** Frullania boveana C.Massal., Nuovo Giorn. Bot. Ital. 17 (3): 244, 1885 (Massalongo 1885).

* Frullania caespitans Beauverd, Sp. Hepat. (Stephani) 6: 537, 1924 (Stephani 1924). *Nom. nov. pro Frullania campanulata* Steph., Biblioth. Bot. 87 (2): 242, 1916 (Stephani 1916a), *nom. illeg*.

* Frullania canaliculata Gottsche, Sp. Hepat. (Stephani) 4: 391, 1910 (Stephani 1910b).

** Frullania capillaris Steph., Sp. Hepat. (Stephani) 4: 616, 1911 (Stephani 1911e).

* Frullania cavallii Gola, Ann. Bot. (Rome) 6 (2): 275, 1907 (Gola 1907).

* Frullania chiapasana Steph., Sp. Hepat. (Stephani) 4: 392, 1910 (Stephani 1910b).

* Frullania chilcootiensis Steph., Bot. Jahrb. Syst. 8 (2): 98, 1886 (Stephani 1886a).

* Frullania chiovendae Gola, Ann. Bot. (Rome) 13 (1): 71, 1914 (Gola 1914a).

** Frullania ciliata Lindenb. et Gottsche, Syn. Hepat. 5: 775, 1847 (Gottsche et al. 1847).

** Frullania cinchonae Gottsche, Syn. Hepat. 3: 455, 1845 (Gottsche et al. 1845b).

* Frullania complicata Steph., Sp. Hepat. (Stephani) 4: 671, 1911 (Stephani 1911e).

* Frullania cordaeana Lindenb., Syn. Hepat. 3: 463, 1845 (Gottsche et al. 1845b).

** Frullania cuneatistipula Steph., Sp. Hepat. (Stephani) 6: 538, 1924 (Stephani 1924).

* Frullania cuspiloba Steph., Sp. Hepat. (Stephani) 4: 394, 1910 (Stephani 1910b).

* Frullania dispar Nees, Syn. Hepat. 3: 429, 1845 (Gottsche et al. 1845b).

* Frullania guatemalensis Steph., Sp. Hepat. (Stephani) 4: 497, 1911 (Stephani 1911e).

*** Frullania inflata Gottsche, Syn. Hepat. 3: 424, 1845 (Gottsche et al. 1845b).

** Frullania inflata var. communis R.M.Schust., Phytologia 57 (5): 372, 1985 (Schuster 1985a).

** Frullania inflata var. dioica S.Hatt. et Thaithong, J. Hattori Bot. Lab. 44: 185, 1978 (Hattori and Thaithong 1978b).

** Frullania inflata var. mayebarae (S.Hatt.) K.Yamada, Misc. Bryol. Lichenol. 6 (9): 163, 1974 (Yamada 1974). Bas.: Frullania mayebarae S.Hatt., Bot. Mag. (Tokyo) 65 (763/764): 13, 1952 (Hattori 1952a).

* Frullania laetevirens Hampe ex Gottsche, Lindenb. et Nees, Syn. Hepat. 3: 420, 1845 (Gottsche et al. 1845b).

* Frullania larjiana Sushil K.Singh et D.K.Singh, J. Bryol. 27 (2): 105, 2005 (Singh and Singh 2005).

* Frullania larjiana var. didyhatii S.N.Srivast. et M.Rai, Geophytology 41 (1/2): 109, 2011 (Srivastava and Rai 2011).

* Frullania leana Austin, Proc. Acad. Nat. Sci. Philadelphia 21: 227, 1869 (Austin 1869).

* Frullania madagascariensis Gottsche, Abh. Naturwiss. Vereins Bremen 7: 364, 1882 (Gottsche 1882).

* Frullania mauritiana Austin, Proc. Acad. Nat. Sci. Philadelphia 21: 227, 1869 (Austin 1869).

*** Frullania mirabilis J.B.Jack et Steph., Hedwigia 31 (1): 15, 1892 (Jack and Stephani 1892).

** Frullania monoica Steph., Bih. Kongl. Svenska Vetensk.-Akad. Handl. 26 (III, 6): 67, 1900 (Stephani 1900b).

* Frullania ocanniensis Steph., Sp. Hepat. (Stephani) 6: 542, 1924 (Stephani 1924).

*** Frullania platycalyx Herzog, Feddes Repert. Spec. Nov. Regni Veg. 55 (1): 10, 1952 (Herzog 1952h).

* Frullania pyricalycina Steph., Sp. Hepat. (Stephani) 4: 394, 1910 (Stephani 1910b).

** Frullania quillotensis (Nees et Mont.) Nees et Mont., Syn. Hepat. 3: 427, 1845 (Gottsche et al. 1845b). Bas.: Jubula quillotensis Nees et Mont., Ann. Sci. Nat. Bot. (sér. 2) 5: 64, 1836 (Nees and Montagne 1836).

** Frullania reicheana Steph., Sp. Hepat. (Stephani) 4: 427, 1910 (Stephani 1910b).

* Frullania sabanetica Gottsche, Ann. Sci. Nat. Bot. (sér. 5) 1: 170, 1864 (Gottsche 1864).

* Frullania semienana Gola, Ann. Bot. (Rome) 13 (1): 71, 1914 (Gola 1914a).

** Frullania serrifolia Steph., Sp. Hepat. (Stephani) 4: 526, 1911 (Stephani 1911e).

** Frullania subpyricalycina Herzog, Rev. Bryol. Lichénol. 23 (1/2): 57, 1954 (Herzog 1954).

** Frullania subtruncata Steph., Sp. Hepat. (Stephani) 4: 389, 1910 (Stephani 1910b).

** Frullania turfosa Lindenb. et Gottsche, Syn. Hepat. 5: 779, 1847 (Gottsche et al. 1847).

** Frullania udarii V.Nath et Ajit P.Singh, Curr. Sci. 91 (6): 744, 2006 (Nath and Singh 2006).

** Frullania valparaisiana Lehm., Nov. Stirp. Pug. 10: 17, 1857 (Lehmann 1857).

** Frullania wagneri Steph., Sp. Hepat. (Stephani) 4: 392, 1910 (Stephani 1910b).

######## *** Jubulaceae H.Klinggr.

by J. Hentschel and M. von Konrat

The systematic placement of Neohattoria herzogii has been contentious since its description six decades ago. It has been interpreted as either a member of the genus Frullania or segregated into its own genus, Neohattoria, due to morphological similarities with both Frullania and Jubula. We follow Larrain et al. (2015) that provided molecular evidence supporting the recognition of the genus Neohattoria and its inclusion within the Jubulaceae, together with Jubula and Nipponolejeunea.

*** **Jubula Dumort.**, Commentat. Bot. (Dumortier): 112, 1822 (Dumortier 1822) nom. conserv.

*** Jubula blepharophylla Grolle, J. Hattori Bot. Lab. 28: 47, 1965 (Grolle 1965b).

** Jubula hattorii Udar et V.Nath, Misc. Bryol. Lichenol. 8 (3): 49, 1978 (Udar and Nath 1978).

** Jubula hattorii var. muthukuzhiana A.E.D.Daniels et P.Daniel, Bryofl. Southernm. W Ghats: 181, 2013 (Daniels and Daniel 2013).

** Jubula himalayensis S.C.Srivast. et D.Sharma, Proc. Indian Acad. Sci. Pl. Sci. 100 (2): 85, 1990 (Srivastava and Sharma 1990).

*** Jubula hutchinsiae (Hook.) Dumort., Syll. Jungerm. Europ.: 36, 1831 (Dumortier 1831). Bas.: Jungermannia hutchinsiae Hook., Brit. Jungermann.: tab. 1, 1812 (Hooker 1812). ^230^

** Jubula hutchinsiae subsp. australiae Pócs et A.Cairns, Nova Hedwigia 86 (1/2): 232, 2008 (Pócs and Cairns 2008).

** Jubula hutchinsiae subsp. bogotensis (Steph.) Verd., Ann. Cryptog. Exot. 1 (2): 215, 1928 (Verdoorn 1928b). Bas.: Jubula bogotensis Steph., Sp. Hepat. (Stephani) 4: 687, 1911 (Stephani 1911e). ^231^

*** Jubula hutchinsiae subsp. caucasica Konstant. et Vilnet, Arctoa 20: 234, 2011 (Konstantinova and Vilnet 2011).

*** Jubula hutchinsiae subsp. japonica (Steph.) Horik. et Ando, J. Sci. Hiroshima Univ., Ser. B, Div. 2, Bot. 6: 300, 1954 (Horikawa and Ando 1954). Bas.: Jubula japonica Steph., Bull. Herb. Boissier 5 (2): 92, 1897 (Stephani 1897b).

*** Jubula hutchinsiae subsp. javanica (Steph.) Verd., Ann. Cryptog. Exot. 1 (2): 216, 1928 (Verdoorn 1928b). Bas.: Jubula javanica Steph., Sp. Hepat. (Stephani) 4: 688, 1911 (Stephani 1911e). ^232^

*** Jubula hutchinsiae subsp. pennsylvanica (Steph.) Verd., Ann. Cryptog. Exot. 1 (2): 215, 1928 (Verdoorn 1928b). Bas.: Frullania pennsylvanica Steph., Hedwigia 22 (10): 147, 1883 (Stephani 1883).

** Jubula kwangsiensis C.Gao et K.C.Chang, Bull. Bot. Res., Harbin 4 (3): 89, 1984 (Chang and Gao 1984).

*** **Neohattoria Kamim.**, J. Jap. Bot. 37 (7): 218, 1962 (Kamimura 1962). *Nom. nov. pro Hattoria* Kamim., J. Hattori Bot. Lab. 24: 93, 1961 (Kamimura 1961).

*** Neohattoria herzogii (S.Hatt.) Kamim., J. Jap. Bot. 37 (7): 218, 1962 (Kamimura 1962). Bas.: Frullania herzogii S.Hatt., Feddes Repert. Spec. Nov. Regni Veg. 58: 53, 1955 (Hattori 1955).

*** **Nipponolejeunea S.Hatt.**, Bull. Tokyo Sci. Mus. 11: 124, 1944 (Hattori 1944d).

*** Nipponolejeunea pilifera (Steph.) S.Hatt., Bull. Tokyo Sci. Mus. 11: 125, 1944 (Hattori 1944d). Bas.: Pycnolejeunea pilifera Steph., Sp. Hepat. (Stephani) 5: 624, 1914 (Stephani 1914b).

*** Nipponolejeunea subalpina (Horik.) S.Hatt., Bull. Tokyo Sci. Mus. 11: 125, 1944 (Hattori 1944d). Bas.: Pycnolejeunea subalpina Horik., J. Jap. Bot. 15: 360, 1939 (Horikawa 1939).

######## *** Lejeuneaceae Cavers

by S.R. Gradstein, T. Pócs and R.-L. Zhu with contributions by G. Dauphin (Ceratolejeunea), X. He (Pycnolejeunea), A.-L. Ilkiu-Borges (Prionolejeunea), E. Reiner-Drehwald (Rectolejeunea, Lejeunea), A. Sass-Gyarmati (Lopholejeunea), A. Schäfer-Verwimp (Diplasiolejeunea) and P. Sukkharak (Thysananthus)

The subdivision of Lejeuneaceae follows Gradstein (2013c) with updates from Heinrichs et al. (2013, 2014) and Schäfer-Verwimp et al. (2014). Some nomenclatural and taxonomic notes can also be found in Gradstein (2013b), Shu and Zhu (2014), Pócs et al. (2015a, 2015c), Qui et al. (2014) and Söderström et al. (2015a).

######### *** Lejeuneoideae

########## *** trib. Brachiolejeuneeae

########### *** subtrib. Brachiolejeuneinae Gradst.

** **Acanthocoleus R.M.Schust.**, Bull. Torrey Bot. Club 97 (6): 339, 1970 [1971] (Schuster 1970b).

*** Acanthocoleus aberrans (Lindenb. et Gottsche) Kruijt, Bryophyt. Biblioth. 36: 62, 1988 (Kruijt 1988). Bas.: Lejeunea aberrans Lindenb. et Gottsche, Syn. Hepat. 5: 751, 1847 (Gottsche et al. 1847).

*** Acanthocoleus aberrans var. laevis Gradst., Fl. Neotrop. Monogr. 62: 193, 1994 (Gradstein 1994).

*** Acanthocoleus chrysophyllus (Lehm.) Kruijt, Bryophyt. Biblioth. 36: 72, 1988 (Kruijt 1988). Bas.: Jungermannia chrysophylla Lehm., Linnaea 9 (4): 423, 1835 (Lehmann 1835).

** Acanthocoleus elgonensis Gyarmati et Pócs, Cryptog. Bryol. 35 (2): 120, 2014 (Sass-Gyarmati and Pócs 2014).

*** Acanthocoleus gilvus (Gottsche) Kruijt, Bryophyt. Biblioth. 36: 79, 1988 (Kruijt 1988). Bas.: Lejeunea gilva Gottsche, Abh. Naturwiss. Vereins Bremen 7: 353, 1882 (Gottsche 1882).

*** Acanthocoleus javanicus (Steph.) Kruijt, Bryophyt. Biblioth. 36: 85, 1988 (Kruijt 1988). Bas.: Dicranolejeunea javanica Steph., Sp. Hepat. (Stephani) 5: 169, 1912 (Stephani 1912c).

*** Acanthocoleus juddii Kruijt, Bryophyt. Biblioth. 36: 93, 1988 (Kruijt 1988).

** Acanthocoleus madagascariensis (Steph.) Kruijt, Bryophyt. Biblioth. 36: 98, 1988 (Kruijt 1988). Bas.: Dicranolejeunea madagascariensis Steph., Sp. Hepat. (Stephani) 5: 158, 1912 (Stephani 1912c).

*** Acanthocoleus trigonus (Nees et Mont.) Gradst., Contr. Univ. Michigan Herb. 18: 101, 1992 (Gradstein 1992b). Bas.: Lejeunea trigona Nees et Mont., Ann. Sci. Nat. Bot. (sér. 2) 5: 61, 1836 (Nees and Montagne 1836).

** Acanthocoleus yoshinaganus (S.Hatt.) Kruijt, Bryophyt. Biblioth. 36: 105, 1988 (Kruijt 1988). Bas.: Lopholejeunea subfusca var. yoshinagana S.Hatt., Bot. Mag. (Tokyo) 58 (686): 38, 1944 (Hattori 1944a).

*** **Blepharolejeunea S.W.Arnell**, Svensk Bot. Tidskr. 56 (2): 335, 1962 (Arnell 1962b).

*** Blepharolejeunea chimantaensis van Slageren et Kruijt, Beih. Nova Hedwigia 80: 126, 1985 (van Slageren and Kruijt 1985).

*** Blepharolejeunea fuegiana (Besch. et C.Massal.) Gradst., Beih. Nova Hedwigia 80: 130, 1985 (van Slageren and Kruijt 1985). Bas.: Lejeunea fuegiana Besch. et C.Massal., Bull. Mens. Soc. Linn. Paris 1 (79): 638, 1886 (Bescherelle and Massalongo 1886).

*** Blepharolejeunea incongrua (Lindenb. et Gottsche) van Slageren et Kruijt, Beih. Nova Hedwigia 80: 133, 1985 (van Slageren and Kruijt 1985). Bas.: Lejeunea incongrua Lindenb. et Gottsche, Syn. Hepat. 5: 750, 1847 (Gottsche et al. 1847).

*** Blepharolejeunea saccata (Steph.) van Slageren et Kruijt, Beih. Nova Hedwigia 80: 138, 1985 (van Slageren and Kruijt 1985). Bas.: Dicranolejeunea saccata Steph., Hedwigia 35 (3): 78, 1896 (Stephani 1896b).

*** Blepharolejeunea securifolia (Steph.) R.M.Schust., Phytologia 45 (5): 424, 1980 (Schuster 1980b). Bas.: Brachiolejeunea securifolia Steph., Sp. Hepat. (Stephani) 5: 128, 1912 (Stephani 1912c).

*** **Brachiolejeunea (Spruce) Schiffn.**, Hepat. (Engl.-Prantl): 128, 1893 (Schiffner 1893b). Bas.: Lejeunea subg. Brachiolejeunea Spruce, Trans. & Proc. Bot. Soc. Edinburgh 15: 129, 1884 (Spruce 1884).

*** Brachiolejeunea conduplicata (Steph.) Gradst., Fl. Neotrop. Monogr. 62: 175, 1994 (Gradstein 1994). Bas.: Archilejeunea conduplicata Steph., Sp. Hepat. (Stephani) 4: 712, 1911 (Stephani 1911e).

*** Brachiolejeunea fernandeziana S.W.Arnell, Ark. Bot. (n.ser.) 4 (1): 18, 1957 (Arnell 1957b).

*** Brachiolejeunea laxifolia (Taylor) Schiffn., Hepat. (Engl.-Prantl): 128, 1893 (Schiffner 1893b). Bas.: Phragmicoma laxifolia Taylor, London J. Bot. 6: 341, 1847 (Taylor 1847b).

*** Brachiolejeunea leiboldiana (Gottsche et Lindenb.) Schiffn., Hedwigia 33 (4): 182, 1894 (Schiffner 1894). Bas.: Phragmicoma leiboldiana Gottsche et Lindenb., Syn. Hepat. 2: 296, 1845 (Gottsche et al. 1845a).

*** Brachiolejeunea phyllorhiza (Nees) Kruijt et Gradst., Nova Hedwigia 43 (3/4): 299, 1986 (Kruijt and Gradstein 1986). Bas.: Jungermannia phyllorhiza Nees, Fl. Bras. (Martius) 1 (1): 348, 1833 (Nees 1833a).

*** Brachiolejeunea spruceana (C.Massal.) Schiffn., Hepat. (Engl.-Prantl): 128, 1893 (Schiffner 1893b). Bas.: Lejeunea spruceana C.Massal., Nuovo Giorn. Bot. Ital. 17 (3): 246, 1885 (Massalongo 1885).

*** **Dicranolejeunea (Spruce) Schiffn.**, Hepat. (Engl.-Prantl): 128, 1893 (Schiffner 1893b). Bas.: Lejeunea subg. Dicranolejeunea Spruce, Trans. & Proc. Bot. Soc. Edinburgh 15: 138, 1884 (Spruce 1884).

*** Dicranolejeunea axillaris (Nees et Mont.) Schiffn., Hepat. (Engl.-Prantl): 128, 1893 (Schiffner 1893b). Bas.: Lejeunea axillaris Nees et Mont., Ann. Sci. Nat. Bot. (sér. 2) 5: 59, 1836 (Nees and Montagne 1836).

* Dicranolejeunea bovonei Gola, Nuovo Giorn. Bot. Ital. (n.ser.) 27 (2/4): 248, 1920 (Gola 1920).

*** **Lindigianthus Kruijt et Gradst.**, Beih. Nova Hedwigia 80: 165, 1985 (Kruijt and Gradstein 1985).

*** Lindigianthus cipaconeus (Gottsche) Kruijt et Gradst., Beih. Nova Hedwigia 80: 166, 1985 (Kruijt and Gradstein 1985). Bas.: Lejeunea cipaconea Gottsche, Ann. Sci. Nat. Bot. (sér. 5) 1: 150, 1864 (Gottsche 1864).

*** **Odontolejeunea (Spruce) Schiffn.**, Hepat. (Engl.-Prantl): 127, 1893 (Schiffner 1893b). Bas.: Lejeunea subg. Odontolejeunea Spruce, Trans. & Proc. Bot. Soc. Edinburgh 15: 142, 1884 (Spruce 1884).

*** Odontolejeunea decemdentata (Spruce) Steph., Sp. Hepat. (Stephani) 5: 175, 1912 (Stephani 1912c). Bas.: Lejeunea decemdentata Spruce, Trans. & Proc. Bot. Soc. Edinburgh 15: 148, 1884 (Spruce 1884).

*** Odontolejeunea lunulata (F.Weber) Schiffn., Hepat. (Engl.-Prantl): 128, 1893 (Schiffner 1893b). Bas.: Jungermannia lunulata F.Weber, Hist. Musc. Hepat. Prodr.: 33, 1815 (Weber 1815).

*** Odontolejeunea rhomalea (Spruce) Steph., Sp. Hepat. (Stephani) 5: 176, 1912 (Stephani 1912c). Bas.: Lejeunea rhomalea Spruce, Trans. & Proc. Bot. Soc. Edinburgh 15: 147, 1884 (Spruce 1884).

########### *** subtrib. Stictolejeuneinae Gradst.

*** **Neurolejeunea (Spruce) Schiffn.**, Hepat. (Engl.-Prantl): 131, 1893 (Schiffner 1893b). Bas.: Lejeunea subg. Neurolejeunea Spruce, Trans. & Proc. Bot. Soc. Edinburgh 15: 84, 1884 (Spruce 1884).

*** Neurolejeunea breutelii (Gottsche) A.Evans, Bull. Torrey Bot. Club 34 (1): 13, 1907 (Evans 1907b). Bas.: Lejeunea breutelii Gottsche, Syn. Hepat. 3: 324, 1845 (Gottsche et al. 1845b).

** Neurolejeunea breutelii var. africana Pócs, Herzogia 28 (1): 63, 2015 (Pócs et al. 2015b).

*** Neurolejeunea catenulata (Nees) Schiffn., Hepat. (Engl.-Prantl): 131, 1893 (Schiffner 1893b). Bas.: Lejeunea catenulata Nees, Syn. Hepat. 3: 323, 1845 (Gottsche et al. 1845b).

*** Neurolejeunea sastreana Gradst., Bryologist 92 (3): 345, 1989 (Gradstein 1989).

*** Neurolejeunea seminervis (Spruce) Schiffn., Hepat. (Engl.-Prantl): 131, 1893 (Schiffner 1893b). Bas.: Lejeunea seminervis Spruce, Trans. & Proc. Bot. Soc. Edinburgh 15: 84, 1884 (Spruce 1884).

*** **Stictolejeunea (Spruce) Schiffn.**, Hepat. (Engl.-Prantl): 131, 1893 (Schiffner 1893b). Bas.: Lejeunea subg. Stictolejeunea Spruce, Trans. & Proc. Bot. Soc. Edinburgh 15: 81, 1884 (Spruce 1884).

*** **subg. Leptostictolejeunea R.M.Schust.**, Phytologia 56 (2): 70, 1984 (Schuster 1984).

*** Stictolejeunea balfourii (Mitt.) E.W.Jones, J. Bryol. 9 (1): 50, 1976 (Jones 1976). Bas.: Lejeunea balfourii Mitt., Philos. Trans. 168: 398, 1879 (Mitten 1879).

*** Stictolejeunea balfourii var. bekkeri Gradst., Beih. Nova Hedwigia 80: 214, 1985 (Gradstein 1985b).

*** Stictolejeunea iwatsukii Mizut., J. Hattori Bot. Lab. 44: 134, 1978 (Mizutani 1978).

*** **subg. Stictolejeunea**

*** Stictolejeunea squamata (Willd.) Schiffn., Hepat. (Engl.-Prantl): 131, 1893 (Schiffner 1893b). Bas.: Jungermannia squamata Willd. ex F.Weber, Hist. Musc. Hepat. Prodr.: 33, 1815 (Weber 1815).

########## *** trib. Lejeuneeae Dumort.

** **Dactylophorella R.M.Schust.**, Phytologia 45 (5): 427, 1980 (Schuster 1980b).

*** Dactylophorella muricata (Gottsche) R.M.Schust., Phytologia 45 (5): 427, 1980 (Schuster 1980b). Bas.: Lejeunea muricata Gottsche, Syn. Hepat. 3: 348, 1845 (Gottsche et al. 1845b).

*** **Metalejeunea Grolle**, Bryophyt. Biblioth. 48: 17, 1995 (Grolle 1995).

** Metalejeunea crassitexta (J.B.Jack et Steph.) Pócs, Telopea 13 (3): 456, 2011 (Pócs et al. 2011). Bas.: Microlejeunea crassitexta J.B.Jack et Steph., Bot. Centralbl. 60 (4): 106, 1894 (Jack and Stephani 1894).

*** Metalejeunea cucullata (Reinw., Blume et Nees) Grolle, Bryophyt. Biblioth. 48: 100, 1995 (Grolle 1995). Bas.: Jungermannia cucullata Reinw., Blume et Nees, Nova Acta Phys.-Med. Acad. Caes. Leop.-Carol. Nat. Cur. 12 (1): 227, 1824 [1825] (Reinwardt et al. 1824a).

*** Metalejeunea winkleri R.L.Zhu et Grolle, Nova Hedwigia 74 (3/4): 498, 2002 (Zhu and Grolle 2002).

*** **Pictolejeunea Grolle**, Feddes Repert. 88 (4): 248, 1977 (Grolle 1977b).

*** Pictolejeunea levis Grolle et M.E.Reiner, J. Bryol. 27 (3): 281, 2005 (Grolle and Reiner-Drehwald 2005).

*** Pictolejeunea mizutanii Grolle, Feddes Repert. 88 (4): 255, 1977 (Grolle 1977b). *Nom. nov. pro Cheilolejeunea picta* Mizut., J. Hattori Bot. Lab. 33: 226, 1970 (Mizutani 1970).

** Pictolejeunea piconii Pócs, Acta Bot. Hung. 49 (1/2): 110, 2007 (Pócs 2007).

*** Pictolejeunea picta (Steph.) Grolle, Feddes Repert. 88 (4): 252, 1977 (Grolle 1977b). Bas.: Prionolejeunea picta Steph., Sp. Hepat. (Stephani) 5: 223, 1913 (Stephani 1913a).

*** Pictolejeunea reginae Ilk.-Borg., Brittonia 54 (4): 318, 2002 [2003] (Ilkiu-Borges 2002).

*** Pictolejeunea sprucei Grolle, Feddes Repert. 88 (4): 249, 1977 (Grolle 1977b).

########### ** subtrib. Ceratolejeuneinae Gradst.

*** **Ceratolejeunea (Spruce) J.B.Jack et Steph.**, Hedwigia 31 (1): 16, 1892 (Jack and Stephani 1892). Bas.: Lejeunea subg. Ceratolejeunea Spruce, Trans. & Proc. Bot. Soc. Edinburgh 15: 198, 1884 (Spruce 1884). ^233^

*** **subg. Ceratolejeunea**

*** Ceratolejeunea andringitrae Pócs, Polish Bot. J. 56 (2): 144, 2011 (Pócs 2011c).

** Ceratolejeunea atlantica Alvarenga et Ilk.-Borg., Nova Hedwigia 86 (1/2): 238, 2008 (Ilkiu-Borges and Alvarenga 2008).

** Ceratolejeunea belangeriana (Gottsche) Steph., Sp. Hepat. (Stephani) 5: 396, 1913 (Stephani 1913a). Bas.: Lejeunea belangeriana Gottsche, Syn. Hepat. 3: 398, 1845 (Gottsche et al. 1845b).

** Ceratolejeunea beninensis E.W.Jones et Vanden Berghen, Bull. Jard. Bot. État Bruxelles 21 (1/2): 63, 1951 (Vanden Berghen 1951b).

*** Ceratolejeunea brevinervis (Spruce) A.Evans, Bull. Torrey Bot. Club 32 (6): 282, 1905 (Evans 1905a). Bas.: Lejeunea brevinervis Spruce, J. Linn. Soc., Bot. 30 (210): 342, 1895 (Gepp 1895b).

*** Ceratolejeunea ceratantha (Nees et Mont.) Schiffn., Bot. Jahrb. Syst. 23 (5): 582, 1897 (Schiffner 1897). Bas.: Lejeunea ceratantha Nees et Mont., Ann. Sci. Nat. Bot. (sér. 2) 14: 335, 1840 (Montagne 1840a).

** Ceratolejeunea coalita (Ångstr.) Steph., Sp. Hepat. (Stephani) 5: 402, 1913 (Stephani 1913a). Bas.: Lejeunea coalita Ångstr., Öfvers. Kongl. Vetensk.-Akad. Förh. 30 (5): 135, 1873 (Ångström 1873).

*** Ceratolejeunea coarina (Gottsche) Schiffn., Hepat. (Engl.-Prantl): 125, 1893 (Schiffner 1893b). Bas.: Lejeunea coarina Gottsche, Syn. Hepat. 3: 395, 1845 (Gottsche et al. 1845b).

*** Ceratolejeunea confusa R.M.Schust., J. Elisha Mitchell Sci. Soc. 72 (2): 313, 1956 (Schuster 1956a).

*** Ceratolejeunea cornuta (Lindenb.) Steph., Pflanzenw. Ost-Afrikas C: 65, 1895 (Stephani 1895d). Bas.: Jungermannia cornuta Lindenb., Syn. hepat. eur: 23, 1829 (Lindenberg 1829).

*** Ceratolejeunea cubensis (Mont.) Schiffn., Hepat. (Engl.-Prantl): 125, 1893 (Schiffner 1893b). Bas.: Lejeunea cubensis Mont., Hist. Phys. Cuba, Bot., Pl. Cell.: 481, 1842 (Montagne 1842a).

*** Ceratolejeunea dentistipula Steph., Sp. Hepat. (Stephani) 5: 407, 1913 (Stephani 1913a).

*** Ceratolejeunea fallax (Lehm. et Lindenb.) Bonner, Candollea 14: 189, 1953 (Bonner 1953a). Bas.: Jungermannia fallax Lehm. et Lindenb., Nov. Stirp. Pug. 5: 17, 1833 (Lehmann 1833).

*** Ceratolejeunea filaria (Taylor) Steph., Sp. Hepat. (Stephani) 5: 412, 1913 (Stephani 1913a). Bas.: Lejeunea filaria Taylor, Nov. Stirp. Pug. 8: 28, 1844 (Lehmann 1844).

** Ceratolejeunea floribunda Steph., Sp. Hepat. (Stephani) 5: 412, 1913 (Stephani 1913a).

*** Ceratolejeunea guianensis (Nees et Mont.) Steph., Sp. Hepat. (Stephani) 5: 416, 1913 (Stephani 1913a). Bas.: Lejeunea guianensis Nees et Mont., Ann. Sci. Nat. Bot. (sér. 2) 14: 335, 1840 (Montagne 1840a).

* Ceratolejeunea karstenii Steph., Sp. Hepat. (Stephani) 5: 420, 1913 (Stephani 1913a).

* Ceratolejeunea kuerschneri Eb.Fisch. et Vanderp., Beih. Nova Hedwigia 138: 87, 2010 (Fischer and Vanderpoorten 2010). ^234^

*** Ceratolejeunea laetefusca (Austin) R.M.Schust., J. Elisha Mitchell Sci. Soc. 72 (2): 306, 1956 (Schuster 1956a). Bas.: Lejeunea laetefusca Austin, Bot. Bull. (Hanover) 1 (8): 36, 1876 (Austin 1876a).

* Ceratolejeunea ledermannii Steph., Sp. Hepat. (Stephani) 6: 399, 1923 (Stephani 1923).

*** Ceratolejeunea malleigera (Spruce) Steph., Sp. Hepat. (Stephani) 5: 422, 1913 (Stephani 1913a). Bas.: Lejeunea malleigera Spruce, Mem. Torrey Bot. Club 1 (3): 123, 1890 (Spruce 1890).

** Ceratolejeunea maranhensis Silva Brito et Ilk.-Borg., Nova Hedwigia 95 (3/4): 424, 2012 (Brito and Ilkiu-Borges 2012).

** Ceratolejeunea minor Mizut., J. Hattori Bot. Lab. 49: 311, 1981 (Mizutani 1981).

*** Ceratolejeunea minuta G.Dauphin, Fl. Neotrop. Monogr. 90: 66, 2003 (Dauphin 2003).

** Ceratolejeunea moniliata Herzog, Mitt. Inst. Allg. Bot. Hamburg 7 (3): 205, 1931 (Herzog 1931a).

** Ceratolejeunea oculata (Gottsche) Steph., Bull. Herb. Boissier 5 (10): 842, 1897 (Stephani 1897c). Bas.: Lejeunea oculata Gottsche, Syn. Hepat. 3: 357, 1845 (Gottsche et al. 1845b).

*** Ceratolejeunea oxygonia Steph., Sp. Hepat. (Stephani) 5: 429, 1913 (Stephani 1913a).

*** Ceratolejeunea papuliflora Steph., Sp. Hepat. (Stephani) 5: 430, 1913 (Stephani 1913a).

*** Ceratolejeunea patentissima (Hampe et Gottsche) A.Evans, Bull. Torrey Bot. Club 32 (6): 286, 1905 (Evans 1905a). Bas.: Lejeunea patentissima Hampe et Gottsche, Linnaea 25 (3): 355, 1852 [1853] (Hampe and Gottsche 1852).

*** Ceratolejeunea pungens Steph., Sp. Hepat. (Stephani) 5: 434, 1913 (Stephani 1913a).

*** Ceratolejeunea rubiginosa Steph., Hedwigia 34 (5): 237, 1895 (Stephani 1895b).

*** Ceratolejeunea saroltae Pócs, Polish Bot. J. 56 (2): 150, 2011 (Pócs 2011c).

* Ceratolejeunea sinensis P.C.Chen et P.C.Wu, Acta Phytotax. Sin. 9 (3): 232, 1964 (Chen and Wu 1964). ^235^

** Ceratolejeunea singapurensis (Lindenb.) Schiffn., Consp. Hepat. Arch. Ind.: 273, 1898 (Schiffner 1898b). Bas.: Lejeunea singapurensis Lindenb., Syn. Hepat. 3: 397, 1845 (Gottsche et al. 1845b).

*** Ceratolejeunea spinosa (Gottsche) Steph., Hedwigia 34 (5): 238, 1895 (Stephani 1895b). Bas.: Lejeunea spinosa Gottsche, Syn. Hepat. 3: 402, 1845 (Gottsche et al. 1845b).

*** Ceratolejeunea temnantha (Spruce) M.E.Reiner, Cryptog. Bryol. 32 (2): 95, 2011 (Reiner-Drehwald 2011). Bas.: Lejeunea temnantha Spruce, Trans. & Proc. Bot. Soc. Edinburgh 15: 250, 1884 (Spruce 1884).

*** Ceratolejeunea umbonata Steph., Sp. Hepat. (Stephani) 5: 446, 1913 (Stephani 1913a).

** Ceratolejeunea vitiensis Steph., Sp. Hepat. (Stephani) 5: 448, 1913 (Stephani 1913a).

** Ceratolejeunea zenkeri Steph., Sp. Hepat. (Stephani) 5: 449, 1914 (Stephani 1914b).

*** **subg. Ceratophora R.M.Schust.**, J. Elisha Mitchell Sci. Soc. 72 (2): 294, 1956 (Schuster 1956a).

*** Ceratolejeunea desciscens (Sande Lac.) Schiffn., Hepat. (Engl.-Prantl): 126, 1893 (Schiffner 1893b). Bas.: Lejeunea desciscens Sande Lac., Syn. hepat. jav.: 107, 1856 [1857] (Sande Lacoste 1856b).

*** Ceratolejeunea globulifera Herzog, Rev. Bryol. Lichénol. 13: 23, 1942 (Herzog 1942e).

*** Ceratolejeunea grandiloba J.B.Jack et Steph., Hedwigia 31 (1): 16, 1892 (Jack and Stephani 1892).

** Ceratolejeunea grandiloba subsp. inflata (Mizut.) Gradst., Phytotaxa 81 (1): 5, 2013 (Gradstein 2013d). Bas.: Ceratolejeunea inflata Mizut., J. Hattori Bot. Lab. 49: 313, 1981 (Mizutani 1981).

*** Ceratolejeunea szyszylowiczii (Loitl.) Steph., Sp. Hepat. (Stephani) 5: 443, 1913 (Stephani 1913a). Bas.: Lejeunea szyszylowiczii Loitl., Diagn. pl. nov.: 19, 1894 (Loitlesberger 1894).

***Incertae sedis***

** Ceratolejeunea aliena Herzog, Trans. Brit. Bryol. Soc. 2 (1): 71, 1952 (Herzog 1952a).

*** **Luteolejeunea Piippo**, Acta Bot. Fenn. 132: 56, 1986 (Piippo 1986a).

*** Luteolejeunea herzogii (Buchloh) Piippo, Acta Bot. Fenn. 132: 57, 1986 (Piippo 1986a). Bas.: Stictolejeunea herzogii Buchloh, Nova Hedwigia 3 (4): 515, 1961 (Buchloh 1961).

*** **Otigoniolejeunea (Spruce) Schiffn.**, Hepat. (Engl.-Prantl): 125, 1893 (Schiffner 1893b). Bas.: Lejeunea subg. Otigoniolejeunea Spruce, Trans. & Proc. Bot. Soc. Edinburgh 15: 226, 1884 (Spruce 1884).

* Otigoniolejeunea crenulata Steph., Sp. Hepat. (Stephani) 6: 408, 1923 (Stephani 1923). ^236^

*** Otigoniolejeunea huctumalcensis (Lindenb. et Gottsche) Y.M.Wei, R.L.Zhu et Gradst., Phytotaxa 162 (4): 237, 2014 (Wei et al. 2014). Bas.: Lejeunea huctumalcensis Lindenb. et Gottsche, Syn. Hepat. 5: 762, 1847 (Gottsche et al. 1847).

* Otigoniolejeunea ledermannii Steph., Sp. Hepat. (Stephani) 6: 409, 1923 (Stephani 1923). ^237^

*** Otigoniolejeunea portoricensis (Hampe et Gottsche) Y.M.Wei, R.L.Zhu et Gradst., Phytotaxa 162 (4): 237, 2014 (Wei et al. 2014). Bas.: Lejeunea portoricensis Hampe et Gottsche, Linnaea 25 (3): 352, 1852 [1853] (Hampe and Gottsche 1852).

########### *** subtrib. Cheilolejeuneinae Gradst.

* **Aureolejeunea R.M.Schust.**, Phytologia 39 (6): 428, 1978 (Schuster 1978b). ^238^

*** Aureolejeunea aurifera R.M.Schust., Phytologia 39 (6): 429, 1978 (Schuster 1978b).

*** Aureolejeunea lumae (Herzog) van Slageren, Meded. Bot. Mus. Herb. Rijks Univ. Utrecht 544: 121, 1985 (van Slageren 1985). Bas.: Brachiolejeunea lumae Herzog, Beih. Bot. Centralbl. 60B (1/2): 15, 1939 (Herzog 1939c).

*** Aureolejeunea paramicola (Herzog) R.M.Schust., Phytologia 61 (7): 446, 1987 (Schuster 1987c). Bas.: Brachiolejeunea paramicola Herzog, Hedwigia 74 (2): 95, 1934 (Herzog 1934a).

*** Aureolejeunea quinquecarinata R.M.Schust., Phytologia 39 (6): 429, 1978 (Schuster 1978b).

*** Aureolejeunea tonduzana (Steph.) Gradst., Phytotaxa 76 (3): 46, 2013 (Gradstein 2013a). Bas.: Archilejeunea tonduzana Steph., Sp. Hepat. (Stephani) 4: 721, 1911 (Stephani 1911e).

*** **Cheilolejeunea (Spruce) Steph.**, Bot. Gaz. 15 (11): 284, 1890 (Stephani 1890c). Bas.: Lejeunea subg. Cheilolejeunea Spruce, Trans. & Proc. Bot. Soc. Edinburgh 15: 251, 1884 (Spruce 1884). ^239^

** Cheilolejeunea sandvicensis (Prantl) Steph., Bull. Herb. Boissier 5 (10): 842, 1897 (Stephani 1897c). Bas.: Lejeunea sandvicensis Prantl, Hedwigia 29: xvii, 1890 (Prantl 1890).

** **subg. Cheilolejeunea**

*** Cheilolejeunea acanthina (Spruce) Gradst. et Ilk.-Borg., Mem. New York Bot. Gard. 76 (4): 62, 2009 (Gradstein and Ilkiu-Borges 2009). Bas.: Lejeunea acanthina Spruce, Trans. & Proc. Bot. Soc. Edinburgh 15: 182, 1884 (Spruce 1884).

*** Cheilolejeunea adnata (Kunze ex Lehm.) Grolle, J. Bryol. 9 (4): 529, 1977 [1978] (Grolle 1977a). Bas.: Jungermannia adnata Kunze ex Lehm., Nov. Stirp. Pug. 6: 46, 1834 (Lehmann 1834).

** Cheilolejeunea adnata var. autoica Gradst. et Ilk.-Borg., Mem. New York Bot. Gard. 76 (4): 64, 2009 (Gradstein and Ilkiu-Borges 2009).

** Cheilolejeunea albovirens (Hook.f. et Taylor) E.A.Hodgs., Rec. Domin. Mus. 4 (11): 127, 1962 (Hodgson 1962a). Bas.: Jungermannia albovirens Hook.f. et Taylor, London J. Bot. 3: 397, 1844 (Hooker and Taylor 1844a).

*** Cheilolejeunea aneogyna (Spruce) A.Evans, Trans. Connecticut Acad. Arts 10 (8): 440, 1900 (Evans 1900a). Bas.: Lejeunea aneogyna Spruce, Trans. & Proc. Bot. Soc. Edinburgh 15: 254, 1884 (Spruce 1884).

** Cheilolejeunea ascensionis (Hook.f. et Taylor) Grolle, Haussknechtia 4: 45, 1988 (Grolle 1988a). Bas.: Jungermannia ascensionis Hook.f. et Taylor, London J. Bot. 4: 91, 1845 (Hooker and Taylor 1845).

*** Cheilolejeunea asperiflora (Spruce) Gradst. et Ilk.-Borg., Mem. New York Bot. Gard. 76 (4): 62, 2009 (Gradstein and Ilkiu-Borges 2009). Bas.: Lejeunea asperiflora Spruce, Trans. & Proc. Bot. Soc. Edinburgh 15: 183, 1884 (Spruce 1884).

** Cheilolejeunea australis Solari, Comun. Mus. Argent. Ci. Nat. “Bernardino Rivadavia,” Ci. Bot. 2 (11): 70, 1981 (Solari 1981).

** Cheilolejeunea baumannii Hürl., Bauhinia 11 (3): 160, 1995 (Hürlimann 1995).

*** Cheilolejeunea beyrichii (Lindenb.) M.E.Reiner, Nova Hedwigia 83 (3/4): 474, 2006 (Reiner-Drehwald 2006). Bas.: Lejeunea beyrichii Lindenb., Syn. Hepat. 3: 371, 1845 (Gottsche et al. 1845b).

** Cheilolejeunea boninensis Mizut., J. Hattori Bot. Lab. 51: 153, 1982 (Mizutani 1982).

*** Cheilolejeunea caducifolia (Gradst. et Schäf.-Verw.) W.Ye et R.L.Zhu, J. Bryol. 32 (4): 280, 2010 (Ye and Zhu 2010). Bas.: Leucolejeunea caducifolia Gradst. et Schäf.-Verw., J. Hattori Bot. Lab. 74: 64, 1993 (Gradstein et al. 1993).

** Cheilolejeunea camerunensis S.W.Arnell, Svensk Bot. Tidskr. 52 (1): 63, 1958 (Arnell 1958a).

** Cheilolejeunea celebensis (Steph.) Mizut., J. Hattori Bot. Lab. 36: 157, 1972 [1973] (Mizutani 1972a). Bas.: Trachylejeunea celebensis Steph., Sp. Hepat. (Stephani) 5: 312, 1913 (Stephani 1913a).

** Cheilolejeunea chenii R.L.Zhu et M.L.So, Taxon 48 (4): 663, 1999 (Zhu et al. 1999). *Nom. nov. pro Neurolejeunea fukiensis* P.C.Chen et P.C.Wu, Acta Phytotax. Sin. 9 (3): 227, 1964 (Chen and Wu 1964).

*** Cheilolejeunea clypeata (Schwein.) W.Ye et R.L.Zhu, J. Bryol. 32 (4): 280, 2010 (Ye and Zhu 2010). Bas.: Jungermannia clypeata Schwein., Spec. Fl. Amer. Crypt.: 12, 1821 (Schweinitz 1821).

** Cheilolejeunea compressa (Herzog) Grolle, Bryophyt. Biblioth. 48: 38, 1995 (Grolle 1995). Bas.: Strepsilejeunea compressa Herzog, Bot. Not. 100 (4): 325, 1947 (Herzog 1947).

*** Cheilolejeunea conchifolia (A.Evans) W.Ye et R.L.Zhu, J. Bryol. 32 (4): 280, 2010 (Ye and Zhu 2010). Bas.: Archilejeunea conchifolia A.Evans, Mem. Torrey Bot. Club 8 (2): 128, 1902 (Evans 1902a).

*** Cheilolejeunea cordigera (Steph.) Grolle, J. Bryol. 9 (4): 530, 1977 [1978] (Grolle 1977a). Bas.: Hygrolejeunea cordigera Steph., Hedwigia 35 (3): 100, 1896 (Stephani 1896b).

** Cheilolejeunea coronalis (Gottsche) R.M.Schust., Phytologia 45 (5): 431, 1980 (Schuster 1980b). Bas.: Lejeunea coronalis Gottsche, Syn. Hepat. 3: 361, 1845 (Gottsche et al. 1845b).

* Cheilolejeunea curvatilobula (Herzog) Grolle, J. Bryol. 21 (1): 41, 1999 (Grolle and Reiner-Drehwald 1999). Bas.: Harpalejeunea curvatilobula Herzog, Feddes Repert. Spec. Nov. Regni Veg. 57 (1/2): 184, 1955 (Herzog 1955).

*** Cheilolejeunea decursiva (Sande Lac.) R.M.Schust., Beih. Nova Hedwigia 9: 112, 1963 (Schuster 1963a). Bas.: Lejeunea decursiva Sande Lac., Ned. Kruidk. Arch. 3: 522, 1855 (Sande Lacoste 1855).

*** Cheilolejeunea discoidea (Lehm. et Lindenb.) R.M.Schust. et Kachroo, J. Linn. Soc., Bot. 56 (368): 509, 1961 (Kachroo and Schuster 1961). Bas.: Jungermannia discoidea Lehm. et Lindenb., Nov. Stirp. Pug. 6: 47, 1834 (Lehmann 1834).

** Cheilolejeunea diversifolia Augier, Ann. Fac. Sci. Univ. Féd. Cameroun 11: 66, 1972 (Augier 1972).

** Cheilolejeunea ecarinata Vanden Berghen, Bull. Jard. Bot. Natl. Belg. 54 (1/2): 11, 1984 (Vanden Berghen 1984b).

** Cheilolejeunea erostrata R.M.Schust., Phytologia 39 (6): 427, 1978 (Schuster 1978b).

*** Cheilolejeunea exinnovata E.W.Jones, J. Bryol. 12 (1): 37, 1982 (Jones 1982).

*** Cheilolejeunea fragrantissima (Spruce) R.M.Schust., Phytologia 45 (5): 431, 1980 (Schuster 1980b). Bas.: Lejeunea fragrantissima Spruce, Trans. & Proc. Bot. Soc. Edinburgh 15: 243, 1884 (Spruce 1884).

* Cheilolejeunea fukiensis (P.C.Chen et P.C.Wu) Piippo, J. Hattori Bot. Lab. 68: 133, 1990 (Piippo 1990). Bas.: Euosmolejeunea fukiensis P.C.Chen et P.C.Wu, Acta Phytotax. Sin. 9 (3): 232, 1964 (Chen and Wu 1964). ^240^

* Cheilolejeunea galliotii Steph., Sp. Hepat. (Stephani) 5: 656, 1914 (Stephani 1914b).

** Cheilolejeunea germanii (Besch. et Spruce) Grolle, Acta Bot. Fenn. 125: 64, 1984 (Grolle and Piippo 1984). Bas.: Lejeunea germanii Besch. et Spruce, Bull. Soc. Bot. France (Congr. Bot.) 36: clxxxvii, 1889 [1890] (Bescherelle and Spruce 1889).

** Cheilolejeunea ghatensis G.Asthana, S.C.Srivast. et A.K.Asthana, Lindbergia 20 (2/3): 132, 1995 [1996] (Asthana et al. 1995).

*** Cheilolejeunea gradsteinii (Grolle et Piippo) W.Ye et R.L.Zhu, J. Bryol. 32 (4): 280, 2010 (Ye and Zhu 2010). Bas.: Leucolejeunea gradsteinii Grolle et Piippo, Ann. Bot. Fenn. 27 (2): 122, 1990 (Grolle and Piippo 1990).

** Cheilolejeunea grandibracteata Steph., Sp. Hepat. (Stephani) 5: 657, 1914 (Stephani 1914b).

** Cheilolejeunea hamlinii Grolle, Wiss. Z. Friedrich-Schiller-Univ. Jena, Math.-Naturwiss. Reihe 31 (2): 212, 1982 (Grolle 1982). *Nom. nov. pro Strepsilejeunea curnowii* Steph., Hedwigia 35 (3): 129, 1896 (Stephani 1896b).

** Cheilolejeunea hawaica Steph., Bull. Herb. Boissier 5 (10): 847, 1897 (Stephani 1897c).

* Cheilolejeunea herzogiana Steph., Biblioth. Bot. 87 (2): 267, 1916 (Stephani 1916a).

** Cheilolejeunea implexicaulis (Hook.f. et Taylor) R.M.Schust., J. Hattori Bot. Lab. 26: 245, 1963 (Schuster 1963b). Bas.: Jungermannia implexicaulis Hook.f. et Taylor, London J. Bot. 3: 397, 1844 (Hooker and Taylor 1844a).

*** Cheilolejeunea insecta Grolle et Gradst., Taxon 50 (4): 1071, 2001 [2002] (Grolle et al. 2001).

** Cheilolejeunea insignis Jovet-Ast et Tixier, Rev. Bryol. Lichénol. 31 (1/2): 25, 1962 (Jovet-Ast and Tixier 1962).

** Cheilolejeunea intricata (Steph.) J.J.Engel, Bryologist 79 (4): 514, 1976 [1977] (Engel 1976b). Bas.: Harpalejeunea intricata Steph., Sp. Hepat. (Stephani) 5: 269, 1913 (Stephani 1913a).

** Cheilolejeunea invaginata R.M.Schust., Phytologia 39 (6): 427, 1978 (Schuster 1978b).

** Cheilolejeunea jamaicensis Steph., Hedwigia 34 (5): 241, 1895 (Stephani 1895b).

** Cheilolejeunea japonica (Horik.) W.Ye et R.L.Zhu, J. Bryol. 32 (4): 280, 2010 (Ye and Zhu 2010). Bas.: Archilejeunea japonica Horik., J. Sci. Hiroshima Univ., Ser. B, Div. 2, Bot. 1: 84, 1932 (Horikawa 1932a).

** Cheilolejeunea kitagawae W.Ye et R.L.Zhu, J. Bryol. 32 (4): 280, 2010 (Ye and Zhu 2010). *Nom. nov. pro Leucolejeunea paroica* N.Kitag., Acta Phytotax. Geobot. 18 (7): 190, 1960 (Kitagawa 1960a).

*** Cheilolejeunea lacerata C.J.Bastos et Gradst., J. Bryol. 28 (2): 133, 2006 (Bastos and Gradstein 2006).

** Cheilolejeunea larsenii Mizut., Dansk Bot. Ark. 27 (1): 95, 1969 (Hattori and Mizutani 1969).

* Cheilolejeunea laurentii Steph., Sp. Hepat. (Stephani) 5: 647, 1914 (Stephani 1914b). ^241^

** Cheilolejeunea leptophylla (Ångstr.) Steph., Sp. Hepat. (Stephani) 5: 657, 1914 (Stephani 1914b). Bas.: Lejeunea leptophylla Ångstr., Öfvers. Kongl. Vetensk.-Akad. Förh. 33 (7): 86, 1876 [1877] (Ångström 1876).

* Cheilolejeunea longiflora (Taylor) R.M.Schust., Phytologia 45 (5): 431, 1980 (Schuster 1980b). Bas.: Lejeunea longiflora Taylor, London J. Bot. 5: 396, 1846 (Taylor 1846b). ^242^

** Cheilolejeunea longispina (Herzog) R.M.Schust., Beih. Nova Hedwigia 9: 111, 1963 (Schuster 1963a). Bas.: Harpalejeunea longispina Herzog, Rev. Bryol. Lichénol. 23 (1/2): 61, 1954 (Herzog 1954).

** Cheilolejeunea loriana (Steph.) W.Ye et R.L.Zhu, J. Bryol. 32 (4): 280, 2010 (Ye and Zhu 2010). Bas.: Symbiezidium lorianum Steph., Sp. Hepat. (Stephani) 5: 106, 1912 (Stephani 1912c).

** Cheilolejeunea ludoviciae Steph., Sp. Hepat. (Stephani) 5: 668, 1914 (Stephani 1914b).

** Cheilolejeunea lurida (Lindenb.) Steph., Sp. Hepat. (Stephani) 5: 658, 1914 (Stephani 1914b). Bas.: Lejeunea lurida Lindenb., Syn. Hepat. 3: 379, 1845 (Gottsche et al. 1845b).

** Cheilolejeunea macroloba (Herzog) Grolle, Wiss. Z. Friedrich-Schiller-Univ. Jena, Math.-Naturwiss. Reihe 31 (2): 212, 1982 (Grolle 1982). Bas.: Strepsilejeunea macroloba Herzog, Nat. Hist. Juan Fernandez (Botany) 2 (5): 742, 1942 (Herzog 1942a).

*** Cheilolejeunea malaccensis (G.Hoffm.) Xiao L.He, Ann. Bot. Fenn. 33 (1): 59, 1996 (He 1996b). Bas.: Pycnolejeunea malaccensis G.Hoffm., Ann. Bryol. 8: 118, 1935 (Hoffman 1935).

** Cheilolejeunea mammifera R.M.Schust., Phytologia 45 (5): 429, 1980 (Schuster 1980b).

*** Cheilolejeunea mariana (Gottsche) B.M.Thiers et Gradst., Mem. New York Bot. Gard. 52: 75, 1989 (Thiers and Gradstein 1989). Bas.: Lejeunea mariana Gottsche, Syn. Hepat. 3: 337, 1845 (Gottsche et al. 1845b).

** Cheilolejeunea mexicana Steph., Sp. Hepat. (Stephani) 6: 417, 1923 (Stephani 1923).

* Cheilolejeunea micholitzii (Steph.) R.M.Schust. et Kachroo, J. Linn. Soc., Bot. 56 (368): 509, 1961 (Kachroo and Schuster 1961). Bas.: Pycnolejeunea micholitzii Steph., Sp. Hepat. (Stephani) 5: 627, 1914 (Stephani 1914b). ^243^

** Cheilolejeunea microscypha (Hook.f. et Taylor) M.Wigginton, J. Bryol. 34 (4): 269, 2012 (Wigginton 2012). Bas.: Jungermannia microscypha Hook.f. et Taylor, London J. Bot. 4: 90, 1845 (Hooker and Taylor 1845).

*** Cheilolejeunea mizutanii W.Ye et R.L.Zhu, J. Bryol. 32 (4): 281, 2010 (Ye and Zhu 2010). *Nom. nov. pro Hygrolejeunea decurrens* Steph., Hedwigia 35 (3): 101, 1896 (Stephani 1896b).

*** Cheilolejeunea neblinensis Ilk.-Borg. et Gradst., Nova Hedwigia 87 (3/4): 522, 2008 (Ilkiu-Borges and Gradstein 2008).

** Cheilolejeunea nietneri (Steph.) Mizut., J. Hattori Bot. Lab. 37: 191, 1973 (Mizutani 1973). Bas.: Harpalejeunea nietneri Steph., Sp. Hepat. (Stephani) 6: 392, 1923 (Stephani 1923).

*** Cheilolejeunea norisiae G.Dauphin et Gradst., J. Bryol. 25 (4): 259, 2003 (Dauphin and Gradstein 2003).

** Cheilolejeunea novaezelandiae R.M.Schust., Phytologia 56 (7): 459, 1985 (Schuster 1985c).

*** Cheilolejeunea obcordata Herzog, Mitt. Inst. Allg. Bot. Hamburg 7 (3): 209, 1931 (Herzog 1931a).

** Cheilolejeunea obtruncata (Mont.) Solari, J. Hattori Bot. Lab. 54: 539, 1983 (Solari 1983a). Bas.: Lejeunea obtruncata Mont., Ann. Sci. Nat. Bot. (sér. 3) 4: 354, 1845 (Montagne 1845b).

** Cheilolejeunea obtusa (Herzog) Solari, J. Hattori Bot. Lab. 54: 537, 1983 (Solari 1983a). Bas.: Harpalejeunea obtusa Herzog, Rev. Bryol. Lichénol. 29 (3/4): 193, 1960 [1961] (Herzog 1960).

** Cheilolejeunea obtusifolia (Steph.) S.Hatt., J. Hattori Bot. Lab. 18: 116, 1957 (Hattori 1957a). Bas.: Harpalejeunea obtusifolia Steph., Sp. Hepat. (Stephani) 5: 265, 1913 (Stephani 1913a).

** Cheilolejeunea occlusa (Herzog) T.Kodama et N.Kitag., Bull. Osaka Mus. Nat. Hist. 28: 40, 1974 (Kitagawa and Kodama 1974). Bas.: Strepsilejeunea occlusa Herzog, Trans. Brit. Bryol. Soc. 1 (4): 320, 1950 (Herzog 1950a).

** Cheilolejeunea orientalis (Gottsche) Mizut., J. Hattori Bot. Lab. 35: 399, 1972 (Mizutani 1972b). Bas.: Lejeunea orientalis Gottsche, Syn. Hepat. 3: 371, 1845 (Gottsche et al. 1845b).

** Cheilolejeunea ovistipula Steph., Hedwigia 34 (5): 244, 1895 (Stephani 1895b).

** Cheilolejeunea panurensis (Spruce) Steph., Sp. Hepat. (Stephani) 5: 660, 1914 (Stephani 1914b). Bas.: Lejeunea panurensis Spruce, Trans. & Proc. Bot. Soc. Edinburgh 15: 255, 1884 (Spruce 1884).

** Cheilolejeunea papillata Solari, Comun. Mus. Argent. Ci. Nat. “Bernardino Rivadavia,” Ci. Bot. 2 (11): 72, 1981 (Solari 1981).

** Cheilolejeunea paroica Mizut., J. Hattori Bot. Lab. 46: 364, 1979 (Mizutani 1979b).

** Cheilolejeunea piriflora Schiffn., Bot. Jahrb. Syst. 23 (5): 592, 1897 (Schiffner 1897). Based on: Lejeunea piriflora Gottsche ex Pol., J. Bot. 15: 227, 1877 (Polakowski 1877), *nom. inval*.

*** Cheilolejeunea polystachya (Spruce) Gradst. et Ilk.-Borg., Mem. New York Bot. Gard. 76 (4): 62, 2009 (Gradstein and Ilkiu-Borges 2009). Bas.: Lejeunea polystachya Spruce, Trans. & Proc. Bot. Soc. Edinburgh 15: 250, 1884 (Spruce 1884).

** Cheilolejeunea renneri (G.Hoffm.) Xiao L.He, Ann. Bot. Fenn. 33 (1): 62, 1996 (He 1996b). Bas.: Pycnolejeunea renneri G.Hoffm., Ann. Bryol. 8: 117, 1935 (Hoffman 1935).

*** Cheilolejeunea rotundistipula (Lindenb. ex Lehm.) Malombe, Acta Bot. Hung. 51 (3/4): 322, 2009 (Malombe 2009). Bas.: Jungermannia rotundistipula Lindenb. ex Lehm., Linnaea 4: 360, 1829 (Lehmann 1829).

** Cheilolejeunea rufescens (Lindenb.) Grolle, Wiss. Z. Friedrich-Schiller-Univ. Jena, Math.-Naturwiss. Reihe 31 (2): 212, 1982 (Grolle 1982). Bas.: Lejeunea rufescens Lindenb., Syn. Hepat. 3: 366, 1845 (Gottsche et al. 1845b).

** Cheilolejeunea stenoschiza (Ångstr.) A.Evans, Trans. Connecticut Acad. Arts 10 (8): 436, 1900 (Evans 1900a). Bas.: Lejeunea stenoschiza Ångstr., Öfvers. Kongl. Vetensk.-Akad. Förh. 29 (4): 26, 1872 (Ångström 1872).

** Cheilolejeunea subcrenulata (Spruce) R.M.Schust., Phytologia 45 (5): 431, 1980 (Schuster 1980b). Bas.: Lejeunea subcrenulata Spruce, Trans. & Proc. Bot. Soc. Edinburgh 15: 245, 1884 (Spruce 1884).

* Cheilolejeunea suborbicularis (Herzog) H.A.Mill., Bonner et Bischl., Nova Hedwigia 4: 559, 1962 [1963] (Miller et al. 1962). Bas.: Microlejeunea suborbicularis Herzog, Memoranda Soc. Fauna Fl. Fennica 26: 65, 1950 [1951] (Herzog 1950b).

*** Cheilolejeunea suprema (Grolle et Piippo) W.Ye et R.L.Zhu, J. Bryol. 32 (4): 281, 2010 (Ye and Zhu 2010). Bas.: Leucolejeunea suprema Grolle et Piippo, Ann. Bot. Fenn. 27 (2): 123, 1990 (Grolle and Piippo 1990).

** Cheilolejeunea surrepens (Mitt.) E.W.Jones, J. Bryol. 9 (1): 49, 1976 (Jones 1976). Bas.: Lejeunea surrepens Mitt., Philos. Trans. 168: 399, 1879 (Mitten 1879).

*** Cheilolejeunea turgida (Mitt.) W.Ye et R.L.Zhu, J. Bryol. 32 (4): 281, 2010 (Ye and Zhu 2010). Bas.: Lejeunea turgida Mitt., J. Proc. Linn. Soc., Bot. 5 (18): 110, 1860 [1861] (Mitten 1860c).

* Cheilolejeunea ulugurica Malombe, Eb.Fisch. et Pócs, Acta Biol. Pl. Agr. 1: 24, 2010 [2011] (Malombe et al. 2010). ^244^

*** Cheilolejeunea unciloba (Lindenb.) Malombe, Acta Bot. Hung. 51 (3/4): 325, 2009 (Malombe 2009). Bas.: Lejeunea unciloba Lindenb., Syn. Hepat. 3: 331, 1845 (Gottsche et al. 1845b).

** Cheilolejeunea upoluensis S.W.Arnell, Svensk Bot. Tidskr. 50 (3): 516, 1956 (Arnell 1956a).

** Cheilolejeunea valenciae (Steph.) Xiao L.He, Ann. Bot. Fenn. 33 (1): 55, 1996 (He 1996a). Bas.: Pycnolejeunea valenciae Steph., Sp. Hepat. (Stephani) 5: 605, 1914 (Stephani 1914b).

** Cheilolejeunea virescens (Lehm. et Lindenb.) Steph., Sp. Hepat. (Stephani) 5: 662, 1914 (Stephani 1914b). Bas.: Lejeunea virescens Lehm. et Lindenb., Nov. Stirp. Pug. 7: 21, 1838 (Lehmann 1838).

* Cheilolejeunea viridis Steph., Sp. Hepat. (Stephani) 5: 673, 1914 (Stephani 1914b).

** Cheilolejeunea warnstorffii (Steph.) Solari, Darwiniana 20 (3/4): 387, 1976 (Solari 1976). Bas.: Strepsilejeunea warnstorffii Steph., Hedwigia 35 (3): 131, 1896 (Stephani 1896b).

* Cheilolejeunea wrightii Steph., Sp. Hepat. (Stephani) 5: 662, 1914 (Stephani 1914b).

*** Cheilolejeunea xanthocarpa (Lehm. et Lindenb.) Malombe, Acta Bot. Hung. 51 (3/4): 326, 2009 (Malombe 2009). Bas.: Jungermannia xanthocarpa Lehm. et Lindenb., Nov. Stirp. Pug. 5: 8, 1833 (Lehmann 1833).

** Cheilolejeunea xanthophylla (Lindenb.) Steph., Sp. Hepat. (Stephani) 5: 663, 1914 (Stephani 1914b). Bas.: Lejeunea xanthophylla Lindenb., Syn. Hepat. 3: 370, 1845 (Gottsche et al. 1845b).

** **subg. Euosmolejeunea (Spruce) Kachroo**, Ceylon J. Sci., Biol. Sci. 8 (1): 6, 1968 (Kachroo 1968). Bas.: Lejeunea subg. Euosmolejeunea Spruce, Trans. & Proc. Bot. Soc. Edinburgh 15: 241, 1884 (Spruce 1884).

** Cheilolejeunea aciculifera R.M.Schust., Phytologia 45 (5): 428, 1980 (Schuster 1980b).

** Cheilolejeunea acutangula (Nees) Grolle, J. Hattori Bot. Lab. 45: 173, 1979 (Grolle 1979c). Bas.: Jungermannia acutangula Nees, Fl. Bras. (Martius) 1 (1): 357, 1833 (Nees 1833a).

*** Cheilolejeunea asperrima (Steph.) Grolle, J. Hattori Bot. Lab. 58: 197, 1985 (Grolle 1985b). Bas.: Taxilejeunea asperrima Steph., Biblioth. Bot. 87 (2): 259, 1916 (Stephani 1916a).

** Cheilolejeunea birmensis (Steph.) Mizut., J. Hattori Bot. Lab. 27: 139, 1964 (Mizutani 1964a). Bas.: Strepsilejeunea birmensis Steph., Sp. Hepat. (Stephani) 5: 286, 1913 (Stephani 1913a).

** Cheilolejeunea campbelliensis (Steph.) R.M.Schust., J. Hattori Bot. Lab. 26: 245, 1963 (Schuster 1963b). Bas.: Strepsilejeunea campbelliensis Steph., Hedwigia 35 (3): 128, 1896 (Stephani 1896b).

** Cheilolejeunea cedercreutzii (H.Buch et Perss.) Grolle, Feddes Repert. 87 (3/4): 188, 1976 (Grolle 1976a). Bas.: Euosmolejeunea cedercreutzii H.Buch et Perss., Bryophyt. Azoren Madeira: 9, 1941 (Buch and Persson 1941).

** Cheilolejeunea choachina (Gottsche) Gradst., Mem. New York Bot. Gard. 84: 69, 1999 (Gradstein 1999). Bas.: Lejeunea choachina Gottsche, Ann. Sci. Nat. Bot. (sér. 5) 1: 156, 1864 (Gottsche 1864).

*** Cheilolejeunea clausa (Nees et Mont.) R.M.Schust., Hepat. Anthocerotae N. Amer. 4: 863, 1980 (Schuster 1980c). Bas.: Lejeunea clausa Nees et Mont., Ann. Sci. Nat. Bot. (sér. 2) 14: 337, 1840 (Montagne 1840a).

** Cheilolejeunea clavata Mizut., J. Hattori Bot. Lab. 46: 361, 1979 (Mizutani 1979b).

*** Cheilolejeunea comans (Spruce) R.M.Schust., Phytologia 45 (5): 431, 1980 (Schuster 1980b). Bas.: Lejeunea comans Spruce, Trans. & Proc. Bot. Soc. Edinburgh 15: 246, 1884 (Spruce 1884).

** Cheilolejeunea convexa (S.W.Arnell) S.W.Arnell, Hepat. South Africa: 209, 1963 (Arnell 1963b). Bas.: Lejeunea convexa S.W.Arnell, Bot. Not. 106: 272, 1953 (Arnell 1953b).

** Cheilolejeunea cookiensis (Steph.) R.M.Schust. et Kachroo, J. Linn. Soc., Bot. 56 (368): 509, 1961 (Kachroo and Schuster 1961). Bas.: Pycnolejeunea cookiensis Steph., Sp. Hepat. (Stephani) 5: 617, 1914 (Stephani 1914b).

** Cheilolejeunea cordistipula (Steph.) Grolle ex E.W.Jones, J. Bryol. 13 (3): 395, 1985 (Jones 1985). Bas.: Strepsilejeunea cordistipula Steph., Sp. Hepat. (Stephani) 5: 276, 1913 (Stephani 1913a).

** Cheilolejeunea fischeri Malombe, Acta Bot. Hung. 51 (3/4): 319, 2009 (Malombe 2009).

** Cheilolejeunea gaoi R.L.Zhu, M.L.So et Grolle, Bryologist 103 (3): 499, 2000 (Zhu et al. 2000).

** Cheilolejeunea hallingii B.M.Thiers, Brittonia 44 (2): 160, 1992 (Thiers 1992a).

*** Cheilolejeunea inflexa (Hampe) Grolle, J. Hattori Bot. Lab. 45: 174, 1979 (Grolle 1979c). Bas.: Lejeunea inflexa Hampe, Nov. Stirp. Pug. 7: 22, 1838 (Lehmann 1838).

*** Cheilolejeunea intertexta (Lindenb.) Steph., Bull. Herb. Boissier 5 (2): 79, 1897 (Stephani 1897b). Bas.: Lejeunea intertexta Lindenb., Syn. Hepat. 3: 379, 1845 (Gottsche et al. 1845b).

*** Cheilolejeunea krakakammae (Lindenb.) R.M.Schust., Beih. Nova Hedwigia 9: 112, 1963 (Schuster 1963a). Bas.: Lejeunea krakakammae Lindenb., Syn. Hepat. 3: 353, 1845 (Gottsche et al. 1845b).

*** Cheilolejeunea laciniata D.F.Peralta et M.E.Reiner, Bryologist 116 (1): 54, 2013 (Peralta and Reiner-Drehwald 2013).

*** Cheilolejeunea laevicalyx (J.B.Jack et Steph.) Grolle, J. Hattori Bot. Lab. 65: 403, 1988 (Grolle 1988c). Bas.: Strepsilejeunea laevicalyx J.B.Jack et Steph., Sp. Hepat. (Stephani) 5: 283, 1913 (Stephani 1913a).

** Cheilolejeunea laeviuscula (Mitt.) Steph., Sp. Hepat. (Stephani) 5: 668, 1914 (Stephani 1914b). Bas.: Lejeunea laeviuscula Mitt., J. Proc. Linn. Soc., Bot. 5 (18): 114, 1860 [1861] (Mitten 1860c).

*** Cheilolejeunea lindenbergii (Gottsche) Mizut., J. Hattori Bot. Lab. 33: 226, 1970 (Mizutani 1970). Bas.: Lejeunea lindenbergii Gottsche, Syn. Hepat. 3: 336, 1845 (Gottsche et al. 1845b).

** Cheilolejeunea mimosa (Hook.f. et Taylor) R.M.Schust., J. Hattori Bot. Lab. 26: 245, 1963 (Schuster 1963b). Bas.: Jungermannia mimosa Hook.f. et Taylor, London J. Bot. 3: 398, 1844 (Hooker and Taylor 1844a).

** Cheilolejeunea nana R.M.Schust., Phytologia 39 (6): 426, 1978 (Schuster 1978b).

** Cheilolejeunea ngongensis Malombe et Pócs, Acta Bot. Hung. 51 (3/4): 317, 2009 (Malombe 2009).

** Cheilolejeunea nipponica (S.Hatt.) S.Hatt., Misc. Bryol. Lichenol. 1 (14): 1, 1957 (Hattori 1957c). Bas.: Strepsilejeunea nipponica S.Hatt., Bull. Tokyo Sci. Mus. 11: 134, 1944 (Hattori 1944d).

*** Cheilolejeunea omphalogastria Pócs, Trop. Bryol. 9: 131, 1994 (Pócs 1994b).

*** Cheilolejeunea oncophylla (Ångstr.) Grolle et M.E.Reiner, J. Bryol. 19 (4): 781, 1997 (Grolle and Reiner-Drehwald 1997). Bas.: Lejeunea oncophylla Ångstr., Öfvers. Kongl. Vetensk.-Akad. Förh. 33 (7): 86, 1876 [1877] (Ångström 1876).

** Cheilolejeunea ornata C.J.Bastos, J. Bryol. 33 (1): 86, 2011 (Bastos 2011).

** Cheilolejeunea osumiensis (S.Hatt.) Mizut., Misc. Bryol. Lichenol. 8 (7): 148, 1980 (Mizutani 1980). Bas.: Euosmolejeunea osumiensis S.Hatt., Bull. Tokyo Sci. Mus. 11: 105, 1944 (Hattori 1944d).

** Cheilolejeunea pluriplicata (Pearson) R.M.Schust., Phytologia 45 (5): 430, 1980 (Schuster 1980b). Bas.: Lejeunea pluriplicata Pearson, Forh. Vidensk.-Selsk. Kristiania 1887 (9): 5, 1887 (Pearson 1887b).

** Cheilolejeunea pocsii E.W.Jones, J. Bryol. 15 (1): 156, 1988 (Jones 1988).

** Cheilolejeunea polyantha A.Evans, Mem. Torrey Bot. Club 8 (2): 141, 1902 (Evans 1902a).

** Cheilolejeunea polyantha var. caduciloba R.M.Schust., Hepat. Anthocerotae N. Amer. 4: 887, 1980 (Schuster 1980c).

*** Cheilolejeunea revoluta (Herzog) Gradst. et Grolle, J. Hattori Bot. Lab. 74: 59, 1993 (Gradstein et al. 1993). Bas.: Pycnolejeunea revoluta Herzog, Feddes Repert. Spec. Nov. Regni Veg. 57 (1/2): 193, 1955 (Herzog 1955).

*** Cheilolejeunea rigidula (Nees ex Mont.) R.M.Schust., Castanea 36 (2): 102, 1971 (Schuster 1971a). Bas.: Lejeunea rigidula Nees ex Mont., Ann. Sci. Nat. Bot. (sér. 2) 14: 336, 1840 (Montagne 1840a).

** Cheilolejeunea ruwenzorensis (S.W.Arnell) R.M.Schust., Beih. Nova Hedwigia 9: 112, 1963 (Schuster 1963a). Bas.: Euosmolejeunea ruwenzorensis S.W.Arnell, Ark. Bot. (n.ser.) 3 (16): 529, 1956 (Arnell 1956e).

** Cheilolejeunea ryukyuensis Mizut., J. Hattori Bot. Lab. 51: 162, 1982 (Mizutani 1982).

** Cheilolejeunea subopaca (Mitt.) Mizut., J. Hattori Bot. Lab. 26: 183, 1963 (Mizutani 1963). Bas.: Lejeunea subopaca Mitt., J. Proc. Linn. Soc., Bot. 5 (18): 116, 1860 [1861] (Mitten 1860c).

*** Cheilolejeunea trifaria (Reinw., Blume et Nees) Mizut., J. Hattori Bot. Lab. 27: 132, 1964 (Mizutani 1964b). Bas.: Jungermannia trifaria Reinw., Blume et Nees, Nova Acta Phys.-Med. Acad. Caes. Leop.-Carol. Nat. Cur. 12 (1): 226, 1824 [1825] (Reinwardt et al. 1824a).

* Cheilolejeunea udarii G.Asthana, S.C.Srivast. et A.K.Asthana, Lindbergia 20 (2/3): 142, 1995 [1996] (Asthana et al. 1995). ^245^

** Cheilolejeunea usambarana (Steph.) Grolle, J. Hattori Bot. Lab. 46: 344, 1979 (Grolle 1979d). Bas.: Strepsilejeunea usambarana Steph., Akad. Wiss. Wien, Math.-Naturwiss. Kl., Denkschr. 88: 731, 1913 (Stephani 1913b).

** Cheilolejeunea verrucosa Steph., Sp. Hepat. (Stephani) 5: 673, 1914 (Stephani 1914b).

** **subg. Renilejeunea R.M.Schust.**, Beih. Nova Hedwigia 9: 112, 1963 (Schuster 1963a).

*** Cheilolejeunea montagnei (Gottsche ex Mont.) R.M.Schust., Beih. Nova Hedwigia 9: 112, 1963 (Schuster 1963a). Bas.: Lejeunea montagnei Gottsche ex Mont., Ann. Sci. Nat. Bot. (sér. 2) 19: 261, 1843 (Montagne 1843).

** **subg. Xenolejeunea Kachroo et R.M.Schust.**, J. Linn. Soc., Bot. 56 (368): 496, 1961 (Kachroo and Schuster 1961).

** Cheilolejeunea ceylanica (Gottsche) R.M.Schust. et Kachroo, J. Linn. Soc., Bot. 56 (368): 509, 1961 (Kachroo and Schuster 1961). Bas.: Lejeunea ceylanica Gottsche, Syn. Hepat. 3: 359, 1845 (Gottsche et al. 1845b).

** Cheilolejeunea eximia (Jovet-Ast et Tixier) R.L.Zhu et M.L.So, Beih. Nova Hedwigia 121: 114, 2001 (Zhu and So 2001). Bas.: Pycnolejeunea eximia Jovet-Ast et Tixier, Rev. Bryol. Lichénol. 31 (1/2): 31, 1962 (Jovet-Ast and Tixier 1962).

*** Cheilolejeunea falsinervis (Sande Lac.) R.M.Schust. et Kachroo, J. Linn. Soc., Bot. 56 (368): 509, 1961 (Kachroo and Schuster 1961). Bas.: Lejeunea falsinervis Sande Lac., Ned. Kruidk. Arch. 3: 421, 1854 [1855] (Sande Lacoste 1854).

** Cheilolejeunea gardneri (Mitt.) Mizut., J. Hattori Bot. Lab. 26: 181, 1963 (Mizutani 1963). Bas.: Lejeunea gardneri Mitt., J. Proc. Linn. Soc., Bot. 5 (18): 115, 1860 [1861] (Mitten 1860c).

** Cheilolejeunea gigantea (Steph.) R.M.Schust. et Kachroo, J. Linn. Soc., Bot. 56 (368): 509, 1961 (Kachroo and Schuster 1961). Bas.: Pycnolejeunea gigantea Steph., Hedwigia 35 (3): 125, 1896 (Stephani 1896b).

** Cheilolejeunea huerlimannii Tixier, Misc. Bryol. Lichenol. 9 (9): 184, 1983 (Tixier 1983b).

** Cheilolejeunea hyalomarginata R.L.Zhu et Frank Müll., Bryologist 115 (2): 218, 2012 (Zhu and Müller 2012).

*** Cheilolejeunea incisa (Gottsche) R.M.Schust. et Kachroo, J. Linn. Soc., Bot. 56 (368): 509, 1961 (Kachroo and Schuster 1961). Bas.: Lejeunea incisa Gottsche, Syn. Hepat. 3: 360, 1845 (Gottsche et al. 1845b).

** Cheilolejeunea incisa var. teretiflora B.M.Thiers, J. Hattori Bot. Lab. 82: 321, 1997 (Thiers 1997a).

** Cheilolejeunea longidens (Steph.) R.M.Schust. et Kachroo, J. Linn. Soc., Bot. 56 (368): 509, 1961 (Kachroo and Schuster 1961). Bas.: Pycnolejeunea longidens Steph., Sp. Hepat. (Stephani) 5: 634, 1914 (Stephani 1914b).

** Cheilolejeunea meyeniana (Nees, Lindenb. et Gottsche) R.M.Schust. et Kachroo, J. Linn. Soc., Bot. 56 (368): 509, 1961 (Kachroo and Schuster 1961). Bas.: Lejeunea meyeniana Nees, Lindenb. et Gottsche, Observ. bot.: 472, 1843 (Gottsche et al. 1843).

** Cheilolejeunea obtusilobula (S.Hatt.) S.Hatt., Misc. Bryol. Lichenol. 1 (14): 2, 1957 (Hattori 1957c). Bas.: Pycnolejeunea obtusilobula S.Hatt., J. Hattori Bot. Lab. 3: 44, 1948 [1950] (Hattori 1948a).

** Cheilolejeunea parvidens B.M.Thiers, J. Hattori Bot. Lab. 82: 323, 1997 (Thiers 1997a).

** Cheilolejeunea streimannii Pócs et Ninh, Acta Bot. Hung. 47 (1/2): 162, 2005 (Pócs and Ninh 2005).

*** Cheilolejeunea trapezia (Nees) Kachroo et R.M.Schust., J. Linn. Soc., Bot. 56 (368): 509, 1961 (Kachroo and Schuster 1961). Bas.: Jungermannia trapezia Nees, Enum. Pl. Crypt. Javae: 41, 1830 (Nees 1830).

** Cheilolejeunea ventricosa (Schiffn. ex P.Syd.) Xiao L.He, Acta Bot. Fenn. 163: 60, 1999 (He 1999). Bas.: Pycnolejeunea ventricosa Schiffn. ex P.Syd., Just’s Bot. Jahresber. 19: 246, 1894 (Sydow 1894).

*** Cheilolejeunea vittata (Steph. ex G.Hoffm.) R.M.Schust. et Kachroo, J. Linn. Soc., Bot. 56 (368): 509, 1961 (Kachroo and Schuster 1961). Bas.: Pycnolejeunea vittata Steph. ex G.Hoffm., Ann. Bryol. 8: 115, 1935 (Hoffman 1935).

***Incertae sedis***

** Cheilolejeunea gottscheana C.J.Bastos, J. Bryol. 34 (4): 316, 2012 (Bastos 2012b). *Nom. nov. pro Strepsilejeunea lindenbergii* Steph., Hedwigia 35 (3): 130, 1896 (Stephani 1896b).

* Cheilolejeunea heteroclada (Spruce) Schiffn., Hepat. (Engl.-Prantl): 124, 1893 (Schiffner 1893b). Bas.: Lejeunea heteroclada Spruce, Trans. & Proc. Bot. Soc. Edinburgh 15: 256, 1884 (Spruce 1884). ^246^

** Cheilolejeunea minutilobula Amakawa, J. Jap. Bot. 35 (12): 365, 1960 (Amakawa 1960a).

** Cheilolejeunea norrisii (Grolle) M.A.M.Renner, Bryologist 115 (4): 550, 2012 (Renner 2012). Bas.: Lejeunea norrisii Grolle, Wiss. Z. Friedrich-Schiller-Univ. Jena, Math.-Naturwiss. Reihe 31 (2): 215, 1982 (Grolle 1982).

** Cheilolejeunea oscilla M.A.M.Renner, Bryologist 115 (4): 551, 2012 (Renner 2012).

** Cheilolejeunea papulosa Schiffn., Hepat. (Engl.-Prantl): 124, 1893 (Schiffner 1893b). *Nom. nov. pro Lejeunea papulosa* Spruce, Trans. & Proc. Bot. Soc. Edinburgh 15: 258, 1884 (Spruce 1884), *nom. illeg*.

*** Cheilolejeunea riparia (Steph.) M.E.Reiner, Nova Hedwigia 95 (3/4): 467, 2012 (Reiner-Drehwald and Grolle 2012). Bas.: Potamolejeunea riparia Steph., Sp. Hepat. (Stephani) 5: 639, 1914 (Stephani 1914b).

*** Cheilolejeunea rotalis (Hook.f. et Taylor) M.Wigginton, J. Bryol. 34 (4): 270, 2012 (Wigginton 2012). Bas.: Jungermannia rotalis Hook.f. et Taylor, London J. Bot. 4: 89, 1845 (Hooker and Taylor 1845).

** Cheilolejeunea tenerrima (Steph.) C.J.Bastos, J. Bryol. 34 (4): 317, 2012 (Bastos 2012b). Bas.: Strepsilejeunea tenerrima Steph., Sp. Hepat. (Stephani) 5: 286, 1913 (Stephani 1913a).

** Cheilolejeunea urubuensis (Zartman et I.L.Ackerman) R.L.Zhu et Y.M.Wei, Phytotaxa 152 (1): 50, 2013 (Wei et al. 2013). Bas.: Vitalianthus urubuensis Zartman et I.L.Ackerman, Bryologist 105 (2): 267, 2002 (Zartman and Ackerman 2002).

* **Cyrtolejeunea A.Evans**, Bull. Torrey Bot. Club 30 (10): 553, 1903 (Evans 1903c).

*** Cyrtolejeunea holostipa (Spruce) A.Evans, Bull. Torrey Bot. Club. 30 (10): 553, 1903 (Evans 1903c). Bas.: Lejeunea holostipa Spruce, Trans. & Proc. Bot. Soc. Edinburgh 15: 171 (Spruce 1884).

** **Cystolejeunea A.Evans**, Bull. Torrey Bot. Club 33 (1): 16, 1906) (Evans 1906a)

*** Cystolejeunea lineata (Lehm. et Lindenb.) A.Evans, Bull. Torrey Bot. Club. 33 (1): 17, 1906 (Evans 1906a). Bas.: Jungermannia lineata Lehm. et Lindenb., Nov. Stirp. Pug. 4: 53, 1832 (Lehmann 1832).

* **Omphalanthus Lindenb. et Nees**, Syn. Hepat. 2: 303, 1845 (Gottsche et al. 1845a). ^247^

** Omphalanthus baracoensis Mustelier, M.E.Reiner et Gradst., J. Bryol. 29 (2): 95, 2007 (Reiner-Drehwald et al. 2007).

*** Omphalanthus filiformis (Sw.) Nees, Syn. Hepat. 2: 304, 1845 (Gottsche et al. 1845a). Bas.: Jungermannia filiformis Sw., Prodr. (Swartz): 144, 1788 (Swartz 1788).

** Omphalanthus filiformis var. platycoleus (Herzog) Gradst., Phytotaxa 76 (3): 46, 2013 (Gradstein 2013a). Bas.: Omphalanthus platycoleus Herzog, Feddes Repert. Spec. Nov. Regni Veg. 57 (1/2): 171, 1955 (Herzog 1955).

** Omphalanthus filiformis var. wallisii (Prantl) Gradst., Phytotaxa 76 (3): 46, 2013 (Gradstein 2013a). Bas.: Lejeunea wallisii Prantl, Hedwigia 31: xvii, 1892 (Prantl 1892).

** Omphalanthus huanucensis (Gottsche) Gradst., Beih. Nova Hedwigia 80: 109, 1985 (Gradstein and Buskes 1985). Bas.: Lejeunea huanucensis Gottsche, Syn. Hepat. 3: 335, 1845 (Gottsche et al. 1845b).

*** Omphalanthus jackii (Prantl) Gradst., Proc. Kon. Ned. Akad. Wetensch. C 80: 410, 1977 (Gradstein et al. 1977). Bas.: Lejeunea jackii Prantl, Hedwigia 31: xvii, 1892 (Prantl 1892).

*** Omphalanthus ovalis (Lindenb. et Gottsche) Gradst., Proc. Kon. Ned. Akad. Wetensch. C 80: 411, 1977 (Gradstein et al. 1977). Bas.: Lejeunea ovalis Lindenb. et Gottsche, Syn. Hepat. 5: 754, 1847 (Gottsche et al. 1847).

*** Omphalanthus roccatii (Gola) R.M.Schust., Beih. Nova Hedwigia 9: 96, 1963 (Schuster 1963a). Bas.: Acrolejeunea roccatii Gola, Ann. Bot. (Rome) 6 (2): 275, 1907 (Gola 1907).

########### *** subtrib. Cololejeuneinae Gradst.

** **Aphanotropis Herzog**, Trans. Brit. Bryol. Soc. 2 (1): 63, 1952 (Herzog 1952b).

*** Aphanotropis saxicola Herzog, Trans. Brit. Bryol. Soc. 2 (1): 63, 1952 (Herzog 1952b).

** **Calatholejeunea K.I.Goebel**, Ann. Jard. Bot. Buitenzorg 39: 8, 1928 (Goebel 1928).

*** Calatholejeunea lamii Mizut., J. Hattori Bot. Lab. 56: 334, 1984 (Mizutani 1984a).

*** Calatholejeunea paradoxa (Schiffn.) K.I.Goebel, Ann. Jard. Bot. Buitenzorg 39: 8, 1928 (Goebel 1928). Bas.: Lejeunea paradoxa Schiffn., Nova Acta Acad. Caes. Leop.-Carol. German. Nat. Cur. 60 (2): 243, 1893 (Schiffner 1893a).

*** **Cololejeunea (Spruce) Steph.**, Hedwigia 30 (5): 208, 1891 (Stephani 1891a). Bas.: Lejeunea subg. Cololejeunea Spruce, Trans. & Proc. Bot. Soc. Edinburgh 15: 291, 1884 (Spruce 1884). ^248^

** Cololejeunea micrandroecia (Spruce) M.Menzel, Willdenowia 14: 492, 1984 [1985] (Menzel 1984). Bas.: Lejeunea micrandroecia Spruce, Trans. & Proc. Bot. Soc. Edinburgh 15: 298, 1884 (Spruce 1884).

* **subg. Aphanolejeunea (A.Evans) Pócs**, Phytotaxa 202 (1): 64, 2015 (Pócs et al. 2015c). Bas.: Aphanolejeunea A.Evans, Bull. Torrey Bot. Club 38 (6): 272, 1911 (Evans 1911).

*** Cololejeunea berneckerae Pócs, Polish Bot. J. 54 (1): 4, 2009 (Pócs and Bernecker 2009). *Nom. nov. pro Aphanolejeunea pocsii* Bern.-Lück., Nova Hedwigia 66 (1/2): 168, 1998 (Bernecker-Lücking 1998).

*** Cololejeunea cingens (Herzog) Bernecker et Pócs, Polish Bot. J. 54 (1): 4, 2009 (Pócs and Bernecker 2009). Bas.: Aphanolejeunea cingens Herzog, Svensk Bot. Tidskr. 46 (1): 104, 1952 (Herzog 1952e).

*** Cololejeunea cornutissima (R.M.Schust.) Stotler et Crand.-Stotl., Bryologist 80 (3): 411, 1977 (Stotler and Crandall-Stotler 1977). Bas.: Aphanolejeunea cornutissima R.M.Schust., Bryologist 59 (3): 217, 1956 (Schuster 1956c).

*** Cololejeunea costaricensis (Bern.-Lück.) Bernecker et Pócs, Polish Bot. J. 54 (1): 5, 2009 (Pócs and Bernecker 2009). Bas.: Aphanolejeunea costaricensis Bern.-Lück., Nova Hedwigia 66 (1/2): 164, 1998 (Bernecker-Lücking 1998).

*** Cololejeunea cubensis Pócs, Polish Bot. J. 54 (1): 5, 2009 (Pócs and Bernecker 2009). *Nom. nov. pro Aphanolejeunea evansii* Herzog, Beih. Bot. Centralbl. 61B (3): 583, 1942 (Herzog 1942d).

*** Cololejeunea gracilis (Jovet-Ast) Pócs, Cryptog. Bryol. 29 (3): 233, 2008 (Dauphin et al. 2008). Bas.: Aphanolejeunea gracilis Jovet-Ast, Rev. Bryol. Lichénol. 16 (1/2): 21, 1947 [1948] (Jovet-Ast 1947b).

** Cololejeunea gracilis var. linearifolia (R.M.Schust.) Pócs, Acta Bot. Hung. 56 (1/2): 189, 2014 (Pócs et al. 2014). Bas.: Aphanolejeunea gracilis var. linearifolia R.M.Schust., Phytologia 45 (5): 434, 1980 (Schuster 1980b).

*** Cololejeunea grossepapillosa (Horik.) N.Kitag., Hikobia, Suppl. 1: 68, 1981 (Kitagawa 1981a). Bas.: Aphanolejeunea grossepapillosa Horik., J. Sci. Hiroshima Univ., Ser. B, Div. 2, Bot. 1: 92, 1932 (Horikawa 1932a).

** Cololejeunea iwatsukiana (Pócs) Pócs, Polish Bot. J. 54 (1): 6, 2009 (Pócs and Bernecker 2009). Bas.: Aphanolejeunea iwatsukiana Pócs, Hikobia 11: 457, 1994 (Pócs 1994d).

*** Cololejeunea jovetastiana (Pócs) Pócs, Polish Bot. J. 54 (1): 6, 2009 (Pócs and Bernecker 2009). Bas.: Aphanolejeunea jovetastiana Pócs, Cryptog. Bryol. Lichénol. 5 (3): 251, 1984 (Pócs 1984a).

** Cololejeunea lisowskii (Pócs) Pócs, Polish Bot. J. 54 (1): 6, 2009 (Pócs and Bernecker 2009). Bas.: Aphanolejeunea lisowskii Pócs, Cryptog. Bryol. Lichénol. 5 (3): 259, 1984 (Pócs 1984a).

*** Cololejeunea madeirensis Schiffn., Hedwigia 41 (5): 279, 1902 (Schiffner 1902).

*** Cololejeunea microscopica (Taylor) Schiffn., Hepat. (Engl.-Prantl): 122, 1893 (Schiffner 1893b). Bas.: Jungermannia microscopica Taylor, Mackay, Fl. Hibern. 2: 59, 1836 (Taylor 1836a).

*** Cololejeunea microscopica var. africana (Pócs) Pócs et Bernecker, Cryptog. Bryol. 29 (3): 234, 2008 (Dauphin et al. 2008). Bas.: Aphanolejeunea exigua var. africana Pócs, Cryptog. Bryol. Lichénol. 5 (3): 247, 1984 (Pócs 1984a).

*** Cololejeunea microscopica var. exigua (A.Evans) Pócs, Mem. New York Bot. Gard. 76 (4): 73, 2009 (Gradstein and Ilkiu-Borges 2009). Bas.: Aphanolejeunea exigua A.Evans, Bull. Torrey Bot. Club 38 (6): 273, 1911 (Evans 1911).

*** Cololejeunea minuscula Pócs, Polish Bot. J. 54 (1): 7, 2009 (Pócs and Bernecker 2009). *Nom. nov. pro Aphanolejeunea minuta* R.M.Schust., Hepat. Anthocerotae N. Amer. 4: 1310, 1980 (Schuster 1980c).

** Cololejeunea moramangae Tixier, Bull. Acad. Malgache (n.ser) 55 (1/2): 241, 1977 [1979] (Tixier 1977a).

*** Cololejeunea norrisii (Pócs) Pócs, Polish Bot. J. 54 (1): 7, 2009 (Pócs and Bernecker 2009). Bas.: Aphanolejeunea norrisii Pócs, Acta Bot. Fenn. 165: 95, 1999 (Pócs and Piippo 1999).

*** Cololejeunea papillosa (K.I.Goebel) Mizut., J. Hattori Bot. Lab. 29: 156, 1966 (Mizutani 1966). Bas.: Physocolea papillosa K.I.Goebel, Ann. Jard. Bot. Buitenzorg 39: 41, 1928 (Goebel 1928).

*** Cololejeunea sicifolia (Gottsche ex A.Evans) Pócs et Bernecker, Polish Bot. J. 54 (1): 8, 2009 (Pócs and Bernecker 2009). Bas.: Aphanolejeunea sicifolia Gottsche ex A.Evans, Bull. Torrey Bot. Club 38 (6): 277, 1911 (Evans 1911).

*** Cololejeunea sicifolia subsp. jamaicensis (R.M.Schust.) Bernecker et Pócs, Polish Bot. J. 54 (1): 8, 2009 (Pócs and Bernecker 2009). Bas.: Aphanolejeunea jamaicensis R.M.Schust., Phytologia 45 (5): 434, 1980 (Schuster 1980b).

*** Cololejeunea sintenisii (Steph.) Pócs, Cryptog. Bryol. 29 (3): 235, 2008 (Dauphin et al. 2008). Bas.: Aphanolejeunea sintenisii Steph., Sp. Hepat. (Stephani) 5: 861, 1916 (Stephani 1916b).

*** Cololejeunea subsphaeroidea (R.M.Schust.) Pócs, Cryptog. Bryol. 29 (3): 235, 2008 (Dauphin et al. 2008). Bas.: Aphanolejeunea subsphaeroidea R.M.Schust., Phytologia 39 (6): 431, 1978 (Schuster 1978b).

** Cololejeunea thiersiae (Pócs) Pócs, Polish Bot. J. 54 (1): 9, 2009 (Pócs and Bernecker 2009). Bas.: Aphanolejeunea thiersiae Pócs, Hikobia 11: 459, 1994 (Pócs 1994d).

** Cololejeunea veillonii Tixier, Nova Hedwigia 31: 757, 1979 (Tixier 1979b).

*** Cololejeunea winkleri (M.I.Morales et A.Lücking) Pócs, Mem. New York Bot. Gard. 76 (4): 78, 2009 (Gradstein and Ilkiu-Borges 2009). Bas.: Aphanolejeunea winkleri M.I.Morales et A.Lücking, Nova Hedwigia 60 (1/2): 120, 1995 (Morales and Lücking 1995).

* **subg. Austrocololejeunea Tixier**, Nova Hedwigia 31: 776, 1979 (Tixier 1979b).

** Cololejeunea australis Tixier, Nova Hedwigia 31: 781, 1979 (Tixier 1979b).

** Cololejeunea caledonica Gottsche, Hedwigia 34 (5): 246, 1895 (Stephani 1895b).

** Cololejeunea sophiana Tixier, Bot. Not. 128: 428, 1975 [1976] (Tixier 1975a).

** Cololejeunea virotana Tixier, Nova Hedwigia 31: 777, 1979 (Tixier 1979b).

* **subg. Chlorocolea R.M.Schust.**, Beih. Nova Hedwigia 9: 178, 1963 (Schuster 1963a).

*** Cololejeunea ceratilobula (P.C.Chen) R.M.Schust., Beih. Nova Hedwigia 9: 179, 1963 (Schuster 1963a). Bas.: Leptocolea ceratilobula P.C.Chen, Feddes Repert. Spec. Nov. Regni Veg. 58: 49, 1955 (Chen 1955).

*** Cololejeunea desciscens Steph., Hedwigia 34 (5): 248, 1895 (Stephani 1895b).

*** Cololejeunea linopteroides H.Rob., Bryologist 67 (4): 457, 1964 (Robinson 1964).

* Cololejeunea rotundilobula (P.C.Wu et P.J.Lin) Piippo, J. Hattori Bot. Lab. 68: 134, 1990 (Piippo 1990). Bas.: Pedinolejeunea rotundilobula P.C.Wu et P.J.Lin, Acta Phytotax. Sin. 16 (2): 69, 1978 (Wu and Lin 1978). ^249^

*** Cololejeunea sigmoidea Jovet-Ast et Tixier, Rev. Bryol. Lichénol. 31 (1/2): 27, 1962 (Jovet-Ast and Tixier 1962).

** Cololejeunea sigmoidea var. dubia Tixier, Bryophyt. Biblioth. 27: 114, 1985 (Tixier 1985a).

*** Cololejeunea standleyi Herzog, Rev. Bryol. Lichénol. 20 (1/2): 172, 1951 [1952] (Herzog 1951a).

*** Cololejeunea stylilobula Tixier, Phytotaxa 202 (1): 65, 2015 (Pócs et al. 2015c). Based on: Cololejeunea stylilobula Tixier, Bryophyt. Biblioth. 27: 119, 1985 (Tixier 1985a), *nom. inval*.

*** Cololejeunea zangii R.L.Zhu et M.L.So, Syst. Bot. 24 (4): 501, 1999 [2000] (Zhu and So 1999b).

*** **subg. Chlorolejeunea Benedix**, Feddes Repert. Spec. Nov. Regni Veg. Beih. 134: 81, 1953 (Benedix 1953). ^250^

** Cololejeunea lacinulata Benedix, Feddes Repert. Spec. Nov. Regni Veg. Beih. 134: 82, 1953 (Benedix 1953).

*** Cololejeunea madothecoides (Steph.) Benedix, Feddes Repert. Spec. Nov. Regni Veg. Beih. 134: 81, 1953 (Benedix 1953). Bas.: Physocolea madothecoides Steph., Sp. Hepat. (Stephani) 5: 898, 1916 (Stephani 1916b).

** Cololejeunea ombrophila Tixier, Nat. Hist. Bull. Siam Soc. 23 (4): 550, 1970 (Tixier 1970a).

*** Cololejeunea stotleriana Gradst., Ilk.-Borg. et Vanderp., Bryologist 114 (1): 13, 2011 (Gradstein et al. 2011).

*** **subg. Chondriolejeunea Benedix**, Feddes Repert. Spec. Nov. Regni Veg. Beih. 134: 75, 1953 (Benedix 1953). ^251^

*** Cololejeunea chinii Tixier, Nat. Hist. Bull. Siam Soc. 24 (3/4): 445, 1973 (Tixier 1973b).

*** Cololejeunea pseudostipulata Schiffn. ex P.Syd., Just’s Bot. Jahresber. 19: 246, 1894 (Sydow 1894). Based on: Cololejeunea pseudostipulata Schiffn., Leberm., Forschungsr. Gazelle 4 (4): 33, 1890 (Schiffner 1890), *nom. inval*.

*** Cololejeunea shimizui N.Kitag., Acta Phytotax. Geobot. 23 (5/6): 185, 1969 (Kitagawa 1969b).

*** Cololejeunea shimizui var. phangngana N.Kitag., Acta Phytotax. Geobot. 23 (5/6): 187, 1969 (Kitagawa 1969b).

*** **subg. Cololejeunea**, ^252^

** Cololejeunea albodentata P.C.Chen et P.C.Wu, Acta Phytotax. Sin. 9 (3): 252, 1964 (Chen and Wu 1964).

** Cololejeunea armata Tixier, Gard. Bull. Singapore 26 (1): 149, 1972 (Tixier 1972b).

*** Cololejeunea bhutanica Grolle et Mizut., J. Bryol. 15 (2): 281, 1989 (Grolle 1989e).

** Cololejeunea biddlecomiae (Austin) A.Evans, Mem. Torrey Bot. Club 8 (2): 168, 1902 (Evans 1902a). Bas.: Lejeunea biddlecomiae Austin, List. Canad. Hepat.: 5, 1890 (Pearson 1890).

* Cololejeunea caihuaella But et P.C.Wu, Hepat. Fl. Hong Kong: 132, 2009 (Wu and But 2009).

*** Cololejeunea calcarea (Lib.) Steph., Bot. Gaz. 17 (6): 171, 1892 (Stephani 1892f). Bas.: Lejeunea calcarea Lib., Ann. Gen. Sci. Phys. 6: 373, 1820 (Libert 1820).

** Cololejeunea capuronii Tixier, Bull. Acad. Malgache (n.ser) 55 (1/2): 241, 1977 [1979] (Tixier 1977a).

** Cololejeunea dinghuiana R.L.Zhu et Y.F.Wang, J. E. China Norm. Univ., Nat. Sci. Ed. 2: 91, 1992 (Zhu and Wang 1992).

** Cololejeunea dolichodonta Tixier, Bryophyt. Biblioth. 27: 215, 1985 (Tixier 1985a).

*** Cololejeunea dozyana (Sande Lac.) Schiffn., Hedwigia 39 (4): 199, 1900 (Schiffner 1900b). Bas.: Lejeunea dozyana Sande Lac., Ned. Kruidk. Arch. 3: 522, 1855 (Sande Lacoste 1855).

*** Cololejeunea elegans Steph., Hedwigia 30 (5): 208, 1891 (Stephani 1891a).

** Cololejeunea falcidentata R.M.Schust., Nova Hedwigia 15: 507, 1968 (Schuster 1968b).

** Cololejeunea filidens Benedix, Feddes Repert. Spec. Nov. Regni Veg. Beih. 134: 49, 1953 (Benedix 1953).

* Cololejeunea frahmii Tixier, Trop. Bryol. 11: 63, 1995 (Tixier 1995b). ^253^

*** Cololejeunea grolleana Pócs, J. Hattori Bot. Lab. 48: 312, 1980 (Pócs 1980b).

*** Cololejeunea haskarliana (Lehm.) Schiffn., Consp. Hepat. Arch. Ind.: 244, 1898 (Schiffner 1898b). Bas.: Lejeunea haskarliana Lehm., Nov. Stirp. Pug. 8: 26, 1844 (Lehmann 1844).

*** Cololejeunea hyalina G.Asthana et S.C.Srivast., Bryophyt. Biblioth. 60: 25, 2003 (Asthana and Srivastava 2003).

* Cololejeunea kahuziensis Tixier, Trop. Bryol. 11: 63, 1995 (Tixier 1995b). ^254^

*** Cololejeunea karnatakensis G.Asthana et S.C.Srivast., Bryophyt. Biblioth. 60: 26, 2003 (Asthana and Srivastava 2003).

** Cololejeunea kodamae Kamim., Feddes Repert. Spec. Nov. Regni Veg. 58: 55, 1955 (Kamimura 1955).

*** Cololejeunea kolombangarae Pócs, Acta Bryolichenol. Asiat. 4: 67, 2011 (Pócs and Piippo 2011).

** Cololejeunea kolombangarae subsp. sepikensis Pócs, Acta Bryolichenol. Asiat. 4: 68, 2011 (Pócs and Piippo 2011).

*** Cololejeunea konratii Pócs, Acta Bot. Hung. 54 (1/2): 156, 2012 (Pócs 2012b).

*** Cololejeunea kuciana Pócs et Schäf.-Verw., Polish Bot. J. 57 (1): 51, 2012 (Pócs and Schäfer-Verwimp 2012).

*** Cololejeunea longiana Grolle et Mizut., J. Bryol. 15 (2): 284, 1989 (Grolle 1989e).

*** Cololejeunea macounii (Spruce) A.Evans, Mem. Torrey Bot. Club 8 (2): 171, 1902 (Evans 1902a). Bas.: Lejeunea macounii Spruce, Bull. Torrey Bot. Club 17 (10): 259, 1890 (Underwood 1890).

** Cololejeunea magillii Pócs, J. Hattori Bot. Lab. 74: 49, 1993 (Pócs 1993).

*** Cololejeunea malanjae Steph., Bot. Jahrb. Syst. 30 (2): 261, 1901 (Stephani 1901g).

** Cololejeunea mamillata (Ångstr.) E.A.Hodgs., Trans. Roy. Soc. New Zealand, Bot. 3 (11): 184, 1967 (Hodgson 1967). Bas.: Lejeunea mamillata Ångstr., Öfvers. Kongl. Vetensk.-Akad. Förh. 29 (4): 14, 1872 (Ångström 1872).

** Cololejeunea mizutaniana Udar et G.Srivast., Misc. Bryol. Lichenol. 9 (7): 138, 1983 (Udar and Srivastava 1983).

*** Cololejeunea mocambiquensis S.W.Arnell, Mitt. Thüring. Bot. Ges. 1 (1): 7, 1955 [1956] (Arnell 1955a).

** Cololejeunea nanhutashanensis J.D.Yang et S.H.Lin, Phytotaxa 177 (1): 56, 2014 (Yang and Lin 2014).

*** Cololejeunea nilgiriensis G.Asthana et S.C.Srivast., Bryophyt. Biblioth. 60: 27, 2003 (Asthana and Srivastava 2003).

*** Cololejeunea ornata A.Evans, Bryologist 41 (4): 73, 1938 (Evans 1938c).

** Cololejeunea parva Vanden Berghen, Bull. Jard. Bot. Natl. Belg. 47 (1/2): 239, 1977 (Vanden Berghen 1977).

*** Cololejeunea planiflora Benedix, Feddes Repert. Spec. Nov. Regni Veg. Beih. 134: 60, 1953 (Benedix 1953).

*** Cololejeunea platyneura (Spruce) A.Evans, Mem. Torrey Bot. Club 8 (2): 172, 1902 (Evans 1902a). Bas.: Lejeunea platyneura Spruce, Trans. & Proc. Bot. Soc. Edinburgh 15: 299, 1884 (Spruce 1884).

*** Cololejeunea pluridentata P.C.Wu et J.S.Lou, Acta Phytotax. Sin. 16 (4): 105, 1978 (Wu and Lou 1978).

** Cololejeunea pretiosa Benedix, Feddes Repert. Spec. Nov. Regni Veg. Beih. 134: 62, 1953 (Benedix 1953).

*** Cololejeunea pseudocristallina P.C.Chen et P.C.Wu, Acta Phytotax. Sin. 9 (3): 257, 1964 (Chen and Wu 1964).

** Cololejeunea pseudoplagiophylla P.C.Wu et J.S.Lou, Acta Phytotax. Sin. 16 (4): 106, 1978 (Wu and Lou 1978).

** Cololejeunea pseudoschmidtii Tixier, Gard. Bull. Singapore 26 (1): 145, 1972 (Tixier 1972b).

** Cololejeunea ramromensis Pócs, Biodiv., biogeogr., nat. conserv. Wallacea N. Guinea II: 113, 2014 (Chantanaorrapint and Pócs 2014).

*** Cololejeunea rosellata Mizut., J. Hattori Bot. Lab. 29: 159, 1966 (Mizutani 1966).

*** Cololejeunea rossettiana (C.Massal.) Schiffn., Hepat. (Engl.-Prantl): 122, 1893 (Schiffner 1893b). Bas.: Lejeunea rossettiana C.Massal., Nuovo Giorn. Bot. Ital. 21 (3): 487, 1889 (Massalongo 1889).

*** Cololejeunea runssorensis (Steph.) Pócs, Acta Bot. Acad. Sci. Hung. 21 (3/4): 371, 1975 (Pócs 1975). Bas.: Aphanolejeunea runssorensis Steph., Sp. Hepat. (Stephani) 5: 858, 1916 (Stephani 1916b).

** Cololejeunea schaeferi Grolle, J. Bryol. 13 (4): 488, 1986 (Grolle 1986b).

*** Cololejeunea schmidtii Steph., Bot. Tidsskr. 24 (3): 278, 1902 (Stephani 1902b).

** Cololejeunea schmidtii var. acutepapillosa Pócs, Acta Bot. Hung. 54 (1/2): 165, 2012 (Pócs 2012b).

** Cololejeunea selaginellicola Tixier, Gard. Bull. Singapore 26 (1): 149, 1972 (Tixier 1972b).

*** Cololejeunea serrata (Steph.) Benedix, Feddes Repert. Spec. Nov. Regni Veg. Beih. 134: 53, 1953 (Benedix 1953). Bas.: Physocolea serrata Steph., Sp. Hepat. (Stephani) 5: 905, 1916 (Stephani 1916b).

** Cololejeunea shikokiana (Horik.) S.Hatt., Bull. Tokyo Sci. Mus. 11: 101, 1944 (Hattori 1944d). Bas.: Physocolea shikokiana Horik., Bot. Mag. (Tokyo) 46 (544): 182, 1932 (Horikawa 1932b).

** Cololejeunea spinosa (Horik.) Pandé et R.N.Misra, J. Indian Bot. Soc. 22 (2/4): 166, 1943 (Pandé and Misra 1943). Bas.: Physocolea spinosa Horik., J. Sci. Hiroshima Univ., Ser. B, Div. 2, Bot. 1: 70, 1931 (Horikawa 1931a).

*** Cololejeunea stellaris Pócs, Acta Bryolichenol. Asiat. 4: 70, 2011 (Pócs and Piippo 2011).

** Cololejeunea subkodamae Mizut., J. Hattori Bot. Lab. 60: 448, 1986 (Mizutani 1986a).

** Cololejeunea tamasii Schäf.-Verw., Phytotaxa 60: 9, 2012 (Schäfer-Verwimp 2012).

** Cololejeunea tanneri Pócs, Acta Bot. Hung. 31 (1/4): 126, 1985 (Pócs 1985).

*** Cololejeunea tenella Benedix, Feddes Repert. Spec. Nov. Regni Veg. Beih. 134: 55, 1953 (Benedix 1953).

** Cololejeunea tenella var. dentiloba Onr., Bull. Jard. Bot. Natl. Belg. 59 (3/4): 436, 1989 (Onraedt 1989).

** Cololejeunea thiersiana Tixier, Cryptog. Bryol. Lichénol. 16 (3): 229, 1995 (Tixier 1995a). Based on: Cololejeunea thiersiana Tixier, Candollea 46 (2): 291, 1991 (Tixier 1991), *nom. inval*.

** Cololejeunea tuiwawana Pócs, Acta Bot. Hung. 54 (1/2): 165, 2012 (Pócs 2012b).

*** Cololejeunea verrucosa Steph., Hedwigia 34 (5): 253, 1895 (Stephani 1895b).

** Cololejeunea verrucosa var. rectispina (Herzog) Benedix, Feddes Repert. Spec. Nov. Regni Veg. Beih. 134: 55, 1953 (Benedix 1953). Bas.: Physocolea verrucosa var. rectispina Herzog, Mitt. Inst. Allg. Bot. Hamburg 7 (3): 214, 1931 (Herzog 1931a).

*** Cololejeunea zenkeri (Steph.) E.W.Jones, Trans. Brit. Bryol. Soc. 2 (3): 420, 1954 (Jones 1954c). Bas.: Aphanolejeunea zenkeri Steph., Sp. Hepat. (Stephani) 5: 858, 1916 (Stephani 1916b).

* **subg. Cryptolejeunea Benedix**, Feddes Repert. Spec. Nov. Regni Veg. Beih. 134: 77, 1953 (Benedix 1953).

*** Cololejeunea angustiflora (Steph.) Mizut., J. Hattori Bot. Lab. 28: 113, 1965 (Mizutani 1965). Bas.: Leptocolea angustiflora Steph., Sp. Hepat. (Stephani) 5: 848, 1916 (Stephani 1916b).

** Cololejeunea drepanolejeuneoides (Horik.) R.M.Schust., Beih. Nova Hedwigia 9: 174, 1963 (Schuster 1963a). Bas.: Boninoleptocolea drepanolejeuneoides Horik., Bot. Mag. (Tokyo) 50 (598): 558, 1936 (Horikawa 1936). ^255^

** Cololejeunea hattoriana Mizut. et Pócs, Ann. Bot. Fenn. 31 (3): 188, 1994 (Pócs et al. 1994). *Nom. nov. pro Campylolejeunea pusilla* Mizut., J. Hattori Bot. Lab. 29: 154, 1966 (Mizutani 1966).

*** Cololejeunea inflectens (Mitt.) Benedix, Feddes Repert. Spec. Nov. Regni Veg. Beih. 134: 79, 1953 (Benedix 1953). Bas.: Lejeunea inflectens Mitt., J. Proc. Linn. Soc., Bot. 5 (18): 117, 1860 [1861] (Mitten 1860c).

** Cololejeunea mouensis (Tixier) H.A.Mill., Phytologia 47 (4): 322, 1981 (Miller 1981). Bas.: Campylolejeunea mouensis Tixier, Nova Hedwigia 31: 727, 1979 (Tixier 1979b).

*** Cololejeunea vesicaria (Sande Lac.) Schiffn., Consp. Hepat. Arch. Ind.: 247, 1898 (Schiffner 1898b). Bas.: Lejeunea vesicaria Sande Lac., Syn. hepat. jav.: 74, 1856 [1857] (Sande Lacoste 1856b).

* **subg. Diaphanae R.M.Schust.**, J. Elisha Mitchell Sci. Soc. 72 (1): 103, 1956 (Schuster 1956b).

*** Cololejeunea amphibola B.M.Thiers, Beih. Nova Hedwigia 90: 130, 1988 (Thiers 1988).

** Cololejeunea antillana Pócs, Polish Bot. J. 54 (1): 3, 2009 (Pócs and Bernecker 2009). *Nom. nov. pro Aphanolejeunea longifolia* Jovet-Ast, Rev. Bryol. Lichénol. 16 (1/2): 23, 1947 [1948] (Jovet-Ast 1947b).

* Cololejeunea augieri Tixier, Trop. Bryol. 11: 48, 1995 (Tixier 1995b).

*** Cololejeunea azorica V.Allorge et Jovet-Ast, Mitt. Thüring. Bot. Ges. 1 (2/3): 17, 1955 (Allorge and Jovet-Ast 1955).

*** Cololejeunea camillii (Lehm.) A.Evans, Bryologist 15 (4): 59, 1912 (Evans 1912a). Bas.: Lejeunea camillii Lehm., Nov. Stirp. Pug. 10: 15, 1857 (Lehmann 1857).

*** Cololejeunea contractiloba A.Evans, Amer. J. Bot. 5 (3): 131, 1918 (Evans 1918).

*** Cololejeunea cornuta E.W.Jones, Trans. Brit. Bryol. Soc. 2 (3): 436, 1954 (Jones 1954c).

** Cololejeunea crenata (A.Evans) Pócs, Polish Bot. J. 54 (1): 5, 2009 (Pócs and Bernecker 2009). Bas.: Aphanolejeunea crenata A.Evans, Bull. Torrey Bot. Club 38 (6): 276, 1911 (Evans 1911).

*** Cololejeunea diaphana A.Evans, Bull. Torrey Bot. Club 32 (4): 184, 1905 (Evans 1905b).

*** Cololejeunea erostrata (Herzog) Bernecker et Pócs, Polish Bot. J. 54 (1): 5, 2009 (Pócs and Bernecker 2009). Bas.: Physocolea erostrata Herzog, Beih. Bot. Centralbl. 61B (3): 582, 1942 (Herzog 1942d).

** Cololejeunea gradsteinii M.J.Lai et R.L.Zhu, Ann. Bot. Fenn. 45 (5): 334, 2008 (Lai et al. 2008). *Nom. nov. pro Cololejeunea pusilla* Tixier, Nat. Hist. Bull. Siam Soc. 23 (4): 552, 1970 (Tixier 1970a), *nom. illeg*.

** Cololejeunea koponenii (Pócs) Pócs, Polish Bot. J. 54 (1): 6, 2009 (Pócs and Bernecker 2009). Bas.: Aphanolejeunea koponenii Pócs, Acta Bot. Fenn. 165: 91, 1999 (Pócs and Piippo 1999).

*** Cololejeunea lanceolata E.W.Jones, Trans. Brit. Bryol. Soc. 2 (3): 428, 1954 (Jones 1954c).

*** Cololejeunea moralesiae (Bern.-Lück.) Bernecker et Pócs, Polish Bot. J. 54 (1): 7, 2009 (Pócs and Bernecker 2009). Bas.: Aphanolejeunea moralesiae Bern.-Lück., Nova Hedwigia 66 (1/2): 166, 1998 (Bernecker-Lücking 1998).

*** Cololejeunea morobensis (Pócs) Pócs, Polish Bot. J. 54 (1): 7, 2009 (Pócs and Bernecker 2009). Bas.: Aphanolejeunea morobensis Pócs, Ann. Bot. Fenn. 31 (3): 180, 1994 (Pócs et al. 1994).

*** Cololejeunea obtusifolia (E.W.Jones) Tixier, Trop. Bryol. 11: 42, 1995 (Tixier 1995b). Bas.: Cololejeunea pusilla var. obtusifolia E.W.Jones, Trans. Brit. Bryol. Soc. 2 (3): 427, 1954 (Jones 1954c).

* Cololejeunea obtusifolia var. madecassa (Tixier) Pócs, Phytotaxa 202 (1): 64, 2015 (Pócs et al. 2015c). Bas.: Cololejeunea androphylla var. madecassa Tixier, Bull. Acad. Malgache (n.ser) 55 (1/2): 216, 1977 [1979] (Tixier 1977a).

*** Cololejeunea papilliloba (Steph.) Steph., Hedwigia 34 (5): 250, 1895 (Stephani 1895b). Bas.: Lejeunea papilliloba Steph., Hedwigia 29 (2): 73, 1890 (Stephani 1890b).

** Cololejeunea papulosa R.M.Schust., Phytologia 45 (5): 433, 1980 (Schuster 1980b).

*** Cololejeunea paucifolia (Spruce) Bernecker et Pócs, Polish Bot. J. 54 (1): 8, 2009 (Pócs and Bernecker 2009). Bas.: Lejeunea paucifolia Spruce, Bull. Soc. Bot. France (Congr. Bot.) 36: cxciv, 1889 [1890] (Spruce 1889).

*** Cololejeunea peponiformis Mizut., J. Hattori Bot. Lab. 33: 258, 1970 (Mizutani 1970).

** Cololejeunea pseudocuspidata Tixier, Bauhinia 8 (4): 228, 1987 (Hürlimann 1987).

*** Cololejeunea pterocolea Herzog, Svensk Bot. Tidskr. 46 (1): 103, 1952 (Herzog 1952e).

*** Cololejeunea pusilla Steph., Hedwigia 34 (5): 251, 1895 (Stephani 1895b).

** Cololejeunea spathulata Jovet-Ast, Rev. Bryol. Lichénol. 29 (1/2): 36, 1960 (Jovet-Ast 1960).

** Cololejeunea taurifolia Inoue et H.A.Mill., Bull. Natl. Sci. Mus. Tokyo (n.ser.) 8 (2): 152, 1965 (Inoue and Miller 1965).

*** Cololejeunea wightii Steph., Hedwigia 34 (5): 253, 1895 (Stephani 1895b).

*** Cololejeunea yelitzae Pócs et Bernecker, Acta Bot. Hung. 55 (3/4): 386, 2013 (Pócs and Bernecker 2013).

* **subg. Leptocolea (Spruce) Schiffn.**, Hepat. (Engl.-Prantl): 122, 1893 (Schiffner 1893b). Bas.: Lejeunea sect. Leptocolea Spruce, Trans. & Proc. Bot. Soc. Edinburgh 15: 294, 1884 (Spruce 1884).

** Cololejeunea acuminata Mizut., J. Hattori Bot. Lab. 33: 261, 1970 (Mizutani 1970).

*** Cololejeunea aequabilis (Sande Lac.) Schiffn., Consp. Hepat. Arch. Ind.: 242, 1898 (Schiffner 1898b). Bas.: Lejeunea aequabilis Sande Lac., Ann. Mus. Bot. Lugduno-Batavi 1: 310, 1864 (Sande Lacoste 1864).

** Cololejeunea altimontana Pócs, Acta Bryolichenol. Asiat. 4: 78, 2011 (Pócs and Piippo 2011).

*** Cololejeunea amaniensis Pócs, Acta Bot. Hung. 31 (1/4): 120, 1985 (Pócs 1985).

*** Cololejeunea angustibracteata Schiffn. ex P.Syd., Just’s Bot. Jahresber. 19: 246, 1894 (Sydow 1894). Based on: Cololejeunea angustibracteata Schiffn., Leberm., Forschungsr. Gazelle 4 (4): 34, 1890 (Schiffner 1890), *nom. inval*.

*** Cololejeunea apiculata (E.W.Jones) R.M.Schust., Beih. Nova Hedwigia 9: 174, 1963 (Schuster 1963a). Bas.: Leptocolea apiculata E.W.Jones, Trans. Brit. Bryol. Soc. 2 (3): 415, 1954 (Jones 1954c).

* Cololejeunea arrectifolia (Mitt.) Steph., Bot. Jahrb. Syst. 23 (1/2, 3): 309, 1896 (Stephani 1896a). Bas.: Lejeunea arrectifolia Mitt., Fl. vit.: 415, 1871 [1873] (Mitten 1871).

** Cololejeunea aurantia (Tixier) Thouvenot, Cryptog. Bryol. 32 (4): 290, 2011 (Thouvenot et al. 2011). Bas.: Jovetastella aurantia Tixier, Cryptog. Bryol. Lichénol. 3 (1): 29, 1982 (Tixier 1982).

** Cololejeunea bandamiae Tixier, Bull. Acad. Malgache (n.ser) 55 (1/2): 220, 1977 [1979] (Tixier 1977a).

** Cololejeunea bebourensis Tixier, Bull. Acad. Malgache (n.ser) 55 (1/2): 229, 1977 [1979] (Tixier 1977a).

** Cololejeunea bergmansiana Tixier, Bull. Jard. Bot. Natl. Belg. 59 (3/4): 440, 1989 (Tixier 1989).

** Cololejeunea bidentula (Steph.) E.W.Jones, Trans. Brit. Bryol. Soc. 2 (3): 423, 1954 (Jones 1954c). Bas.: Physocolea bidentula Steph., Sp. Hepat. (Stephani) 5: 868, 1916 (Stephani 1916b).

** Cololejeunea bifalcata Pócs, Acta Bot. Hung. 54 (1/2): 146, 2012 (Pócs 2012b).

*** Cololejeunea blepharophylla Pócs, Acta Bot. Hung. 54 (1/2): 149, 2012 (Pócs 2012b).

** Cololejeunea borhidiana Pócs, J. Hattori Bot. Lab. 48: 305, 1980 (Pócs 1980b).

** Cololejeunea bosseriana Tixier, Bull. Acad. Malgache (n.ser) 55 (1/2): 235, 1977 [1979] (Tixier 1977a).

** Cololejeunea calcarata E.W.Jones, Bull. Brit. Mus. (Nat. Hist.), Bot. 11 (3): 235, 1983 (Jones and Harrington 1983).

* Cololejeunea camusii Tixier, Bull. Acad. Malgache (n.ser) 55 (1/2): 227, 1977 [1979] (Tixier 1977a).

*** Cololejeunea ceatocarpa (Ångstr.) Steph., Bull. Herb. Boissier 5 (10): 842, 1897 (Stephani 1897c). Bas.: Lejeunea ceatocarpa Ångstr., Öfvers. Kongl. Vetensk.-Akad. Förh. 29 (4): 27, 1872 (Ångström 1872).

*** Cololejeunea ceylanica Onr., Acta Bot. Acad. Sci. Hung. 25 (1/2): 107, 1979 (Onraedt 1979).

* Cololejeunea chamlongiana Tixier, Ann. Fac. Sci. Univ. Phnom Penh 3: 181, 1970 (Tixier 1970b). ^256^

*** Cololejeunea chenii Tixier, Bryophyt. Biblioth. 27: 219, 1985 (Tixier 1985a). *Nom. nov. pro* Cololejeunea plagiophylla var. grossipapillosa P.C.Chen et P.C.Wu, Acta Phytotax. Sin. 9 (3): 254, 1964 (Chen and Wu 1964).

*** Cololejeunea ciliata Pócs, Ann. Bot. Fenn. 31 (3): 182, 1994 (Pócs et al. 1994).

** Cololejeunea comptonii (Pearson) H.A.Mill., Phytologia 47 (4): 321, 1981 (Miller 1981). Bas.: Leptocolea comptonii Pearson, J. Linn. Soc., Bot. 46 (305): 40, 1922 (Pearson 1922b).

** Cololejeunea cookei A.Evans, Trans. Connecticut Acad. Arts 10 (8): 447, 1900 (Evans 1900a).

** Cololejeunea cordiflora Steph., Hedwigia 34 (5): 246, 1895 (Stephani 1895b).

** Cololejeunea crateris Pócs, Acta Bot. Hung. 54 (1/2): 153, 2012 (Pócs 2012b).

* Cololejeunea crenulata (Pearson) H.A.Mill., Phytologia 47 (4): 321, 1981 (Miller 1981). Bas.: Leptocolea crenulata Pearson, J. Linn. Soc., Bot. 46 (305): 41, 1922 (Pearson 1922b). ^257^

** Cololejeunea cucullifolia (Herzog) E.A.Hodgs., Rec. Domin. Mus. 4 (11): 127, 1962 (Hodgson 1962a). Bas.: Physocolea cucullifolia Herzog, Trans. & Proc. Roy. Soc. New Zealand 68 (1): 45, 1938 (Herzog 1938c).

** Cololejeunea cuneifolia Steph., Hedwigia 31 (4): 166, 1892 (Stephani 1892g).

* Cololejeunea dadeuniana Tixier, Ann. Fac. Sci. Univ. Phnom Penh 3: 186, 1970 (Tixier 1970b).

** Cololejeunea decemplicata (Steph.) Tixier, Bull. Acad. Malgache (n.ser) 55 (1/2): 213, 1977 [1979] (Tixier 1977a). Bas.: Physocolea decemplicata Steph., Sp. Hepat. (Stephani) 5: 869, 1916 (Stephani 1916b).

** Cololejeunea decliviloba Steph., Hedwigia 34 (5): 247, 1895 (Stephani 1895b).

** Cololejeunea dentata (E.W.Jones) R.M.Schust., Beih. Nova Hedwigia 9: 175, 1963 (Schuster 1963a). Bas.: Leptocolea dentata E.W.Jones, Trans. Brit. Bryol. Soc. 2 (2): 161, 1953 (Jones 1953a).

** Cololejeunea denticulata (Horik.) S.Hatt., Bull. Tokyo Sci. Mus. 11: 99, 1944 (Hattori 1944d). Bas.: Physocolea denticulata Horik., J. Sci. Hiroshima Univ., Ser. B, Div. 2, Bot. 2: 287, 1934 (Horikawa 1934).

* Cololejeunea dentilobula (Steph.) R.M.Schust., J. Hattori Bot. Lab. 26: 241, 1963 (Schuster 1963b). Bas.: Physocolea dentilobula Steph., Sp. Hepat. (Stephani) 5: 892, 1916 (Stephani 1916b). ^258^

*** Cololejeunea dianae M.Wigginton, J. Bryol. 28 (4): 364, 2006 (Wigginton 2006).

** Cololejeunea dilatata (Steph.) Mizut., J. Hattori Bot. Lab. 28: 113, 1965 (Mizutani 1965). Bas.: Leptocolea dilatata Steph., Sp. Hepat. (Stephani) 5: 850, 1916 (Stephani 1916b).

*** Cololejeunea diplasiolejeunoides Tixier, Bryologist 82 (4): 602, 1979 (Tixier 1979d).

*** Cololejeunea distalopapillata (E.W.Jones) R.M.Schust., Beih. Nova Hedwigia 9: 173, 1963 (Schuster 1963a). Bas.: Leptocolea distalopapillata E.W.Jones, Trans. Brit. Bryol. Soc. 3 (2): 202, 1957 (Jones 1957b).

* Cololejeunea effusa (Mitt.) Steph., Bot. Jahrb. Syst. 23 (1/2, 3): 309, 1896 (Stephani 1896a). Bas.: Lejeunea effusa Mitt., Fl. vit.: 415, 1871 [1873] (Mitten 1871). ^259^

*** Cololejeunea elephantorum Tixier, Ann. Fac. Sci. Univ. Phnom Penh 3: 185, 1970 (Tixier 1970b).

** Cololejeunea ellipsoidea R.M.Schust., Nova Hedwigia 15: 508, 1968 (Schuster 1968b).

*** Cololejeunea equialbi Tixier, Ann. Fac. Sci. Univ. Phnom Penh 3: 178, 1970 (Tixier 1970b).

*** Cololejeunea filicis (Herzog) Piippo, J. Hattori Bot. Lab. 68: 133, 1990 (Piippo 1990). Bas.: Leptocolea filicis Herzog, Symb. Sin. 5: 52, 1930 (Nicholson et al. 1930).

* Cololejeunea fischeri Tixier, Trop. Bryol. 11: 59, 1995 (Tixier 1995b). ^260^

** Cololejeunea flavicans (Steph.) Mizut., J. Hattori Bot. Lab. 28: 115, 1965 (Mizutani 1965). Bas.: Physocolea flavicans Steph., Sp. Hepat. (Stephani) 5: 893, 1916 (Stephani 1916b).

* Cololejeunea fredericii Onr., Rev. Bryol. Lichénol. 44 (1): 80, 1978 (Onraedt 1978). ^261^

** Cololejeunea fusca (Steph.) Mizut., J. Hattori Bot. Lab. 28: 120, 1965 (Mizutani 1965). Bas.: Physocolea fusca Steph., Sp. Hepat. (Stephani) 5: 893, 1916 (Stephani 1916b).

** Cololejeunea gottschei (Steph.) Pandé, K.P.Srivast. et Ahmad, J. Indian Bot. Soc. 36 (3): 345, 1957 (Pandé et al. 1957). Bas.: Physocolea gottschei Steph., Sp. Hepat. (Stephani) 5: 894, 1916 (Stephani 1916b).

*** Cololejeunea grossestyla M.Wigginton, J. Bryol. 28 (4): 369, 2006 (Wigginton 2006).

*** Cololejeunea grushvitzkiana Pócs, Bot. Zhurn. (Moscow & Leningrad) 56 (5): 674, 1971 (Pócs 1971).

*** Cololejeunea hainanensis R.L.Zhu, J. Hattori Bot. Lab. 78: 87, 1995 (Zhu 1995).

*** Cololejeunea harrisii Pócs, Acta Bot. Acad. Sci. Hung. 21 (3/4): 357, 1975 (Pócs 1975).

*** Cololejeunea hildebrandii (Austin) Steph., Bull. Herb. Boissier 5 (10): 842, 1897 (Stephani 1897c). Bas.: Lejeunea hildebrandii Austin, Bot. Bull. (Hanover) 1 (8): 35, 1876 (Austin 1876a).

*** Cololejeunea hirta Steph., Bull. Misc. Inform. Kew 1899 (151/152): 125, 1899 (MacGregor 1899).

** Cololejeunea hodgsoniae (Herzog) E.A.Hodgs., Trans. Roy. Soc. New Zealand, Bot. 3 (11): 184, 1967 (Hodgson 1967). Bas.: Physocolea hodgsoniae Herzog, Trans. & Proc. Roy. Soc. New Zealand 68 (1): 46, 1938 (Herzog 1938c).

*** Cololejeunea horikawana (S.Hatt.) Mizut., J. Hattori Bot. Lab. 24: 254, 1961 (Mizutani 1961). Bas.: Leptocolea horikawana S.Hatt., J. Jap. Bot. 18 (11): 653, 1942 (Hattori 1942).

*** Cololejeunea huerlimannii Tixier, Nova Hedwigia 31: 773, 1979 (Tixier 1979b).

** Cololejeunea inflexifolia R.M.Schust., Phytologia 56 (7): 458, 1985 (Schuster 1985c).

*** Cololejeunea iradieri M.Infante et Heras, Trop. Bryol. 17: 14, 1999 (Infante and Heras 1999).

** Cololejeunea irianensis Tixier, Bull. Jard. Bot. Natl. Belg. 59 (3/4): 440, 1989 (Tixier 1989).

*** Cololejeunea johannis-winkleri (Herzog) R.L.Zhu, Nova Hedwigia 79 (3/4): 528, 2004 (Zhu et al. 2004). Bas.: Leptocolea johannis-winkleri Herzog, Mitt. Inst. Allg. Bot. Hamburg 7 (3): 214, 1931 (Herzog 1931a).

** Cololejeunea kegelii Steph., Hedwigia 34 (5): 249, 1895 (Stephani 1895b).

** Cololejeunea khanii Tixier, Dacca Univ. Stud., B 15: 9, 1967 (Tixier 1967).

* Cololejeunea kohkongensis Tixier, Bryophyt. Biblioth. 27: 302, 1985 (Tixier 1985a). ^262^

*** Cololejeunea lichenyae R.D.Porley, N.G.Hodgetts et M.Wigginton, J. Bryol. 29 (1): 7, 2007 (Wigginton et al. 2007).

* Cololejeunea lobulilineata Tixier, Trop. Bryol. 11: 42, 1995 (Tixier 1995b). ^263^

*** Cololejeunea longifolia (Mitt.) Benedix ex Mizut., J. Hattori Bot. Lab. 26: 184, 1963 (Mizutani 1963). Bas.: Lejeunea longifolia Mitt., J. Proc. Linn. Soc., Bot. 5 (18): 117, 1860 [1861] (Mitten 1860c).

*** Cololejeunea magna (Tixier) M.Infante et Heras, Trop. Bryol. 17: 17, 1999 (Infante and Heras 1999). Bas.: Cololejeunea harrisii var. magna Tixier, Trop. Bryol. 11: 56, 1995 (Tixier 1995b).

** Cololejeunea magnifica Pócs, Acta Bryolichenol. Asiat. 4: 88, 2011 (Pócs and Piippo 2011).

** Cololejeunea magnilobula (Horik.) S.Hatt., Bull. Tokyo Sci. Mus. 11: 99, 1944 (Hattori 1944d). Bas.: Physocolea magnilobula Horik., J. Sci. Hiroshima Univ., Ser. B, Div. 2, Bot. 2: 288, 1934 (Horikawa 1934).

* Cololejeunea micronesica H.A.Mill. et Bonner, Beih. Nova Hedwigia 11: 63, 1963 (Miller et al. 1963). ^264^

** Cololejeunea mooreaensis Tixier, Bauhinia 8 (4): 226, 1987 (Hürlimann 1987).

* Cololejeunea ninguana Tixier, Nova Hedwigia 31: 776, 1979 (Tixier 1979b). ^265^

** Cololejeunea obcordata (Austin) A.Evans, Trans. Connecticut Acad. Arts 10 (8): 448, 1900 (Evans 1900a). Bas.: Lejeunea obcordata Austin, Bot. Bull. (Hanover) 1 (8): 36, 1876 (Austin 1876a).

*** Cololejeunea obliqua (Nees et Mont.) Schiffn., Bot. Jahrb. Syst. 23 (5): 586, 1897 (Schiffner 1897). Bas.: Lejeunea obliqua Nees et Mont., Ann. Sci. Nat. Bot. (sér. 2) 19: 264, 1843 (Montagne 1843).

* Cololejeunea oblongiperianthia (P.C.Wu et J.S.Lou) Piippo, J. Hattori Bot. Lab. 68: 134, 1990 (Piippo 1990). Bas.: Leptocolea oblongiperianthia P.C.Wu et J.S.Lou, Acta Phytotax. Sin. 16 (4): 109, 1978 (Wu and Lou 1978). ^266^

** Cololejeunea oleana Sim, Trans. Roy. Soc. South Africa 15 (1): 49, 1926 (Sim 1926).

** Cololejeunea ovalifolia A.Evans, Trans. Connecticut Acad. Arts 10 (8): 450, 1900 (Evans 1900a).

** Cololejeunea panchoana Tixier, Ann. Fac. Sci. Univ. Phnom Penh 3: 183, 1970 (Tixier 1970b).

*** Cololejeunea paniensis (Tixier) Grolle, J. Bryol. 8 (4): 485, 1975 (Grolle 1975d). Bas.: Jovetastella paniensis Tixier, Rev. Bryol. Lichénol. 39 (4): 662, 1973 [1974] (Tixier 1973c).

** Cololejeunea papuliflora Steph., Akad. Wiss. Wien, Math.-Naturwiss. Kl., Denkschr. 85: 199, 1910 (Stephani 1910a).

** Cololejeunea pentagona (Mitt.) Steph., Bot. Jahrb. Syst. 23 (1/2, 3): 309, 1896 (Stephani 1896a). Bas.: Lejeunea pentagona Mitt., Fl. vit.: 416, 1871 [1873] (Mitten 1871).

*** Cololejeunea plagiochiliana Tixier, Bot. Not. 128: 428, 1975 [1976] (Tixier 1975a).

** Cololejeunea planiuscula Tixier, Cryptog. Bryol. Lichénol. 16 (3): 229, 1995 (Tixier 1995a). Based on: Cololejeunea planiuscula Tixier, Candollea 46 (2): 289, 1991 (Tixier 1991), *nom. inval*.

*** Cololejeunea pseudoserrata Tixier, Nova Hedwigia 31: 770, 1979 (Tixier 1979b).

* Cololejeunea pteroporum Tixier, Bryophyt. Biblioth. 27: 271, 1985 (Tixier 1985a). ^267^

** Cololejeunea pulchella (Mitt.) R.M.Schust., J. Hattori Bot. Lab. 26: 241, 1963 (Schuster 1963b). Bas.: Lejeunea pulchella Mitt., Bot. antarct. voy. II (Fl. Nov.-Zel. 2): 157, 1854 (Mitten 1854).

** Cololejeunea pulchella var. stylifera R.M.Schust., Phytologia 56 (7): 458, 1985 (Schuster 1985c).

*** Cololejeunea quadridentata (S.Hatt.) Grolle, Acta Bot. Fenn. 125: 65, 1984 (Grolle and Piippo 1984). Bas.: Leptocolea quadridentata S.Hatt., Bot. Mag. (Tokyo) 64 (755/756): 117, 1951 (Hattori 1951c).

** Cololejeunea retusula (Mitt.) H.A.Mill., Phytologia 47 (4): 322, 1981 (Miller 1981). Bas.: Lejeunea retusula Mitt., Fl. vit.: 416, 1871 [1873] (Mitten 1871).

** Cololejeunea salgadoi Onr., Bull. Jard. Bot. Natl. Belg. 59 (3/4): 433, 1989 (Onraedt 1989).

*** Cololejeunea sanctae-helenae M.Wigginton, J. Bryol. 28 (4): 366, 2006 (Wigginton 2006).

** Cololejeunea serrulata Steph., Hedwigia 34 (5): 252, 1895 (Stephani 1895b).

*** Cololejeunea setiloba A.Evans, Bryologist 16 (4): 51, 1913 (Evans 1913).

*** Cololejeunea siangensis G.Asthana et S.C.Srivast., Bryophyt. Biblioth. 60: 57, 2003 (Asthana and Srivastava 2003).

*** Cololejeunea skottsbergii Herzog, Nat. Hist. Juan Fernandez (Botany) 2 (5): 749, 1942 (Herzog 1942a).

*** Cololejeunea societatis Tixier, Bauhinia 8 (4): 230, 1987 (Hürlimann 1987).

* Cololejeunea spathulifolia (Steph.) H.A.Mill., Phytologia 47 (4): 322, 1981 (Miller 1981). Bas.: Leptocolea spathulifolia Steph., Sp. Hepat. (Stephani) 5: 855, 1916 (Stephani 1916b). ^268^

** Cololejeunea spruceana Tixier, Cryptog. Bryol. Lichénol. 16 (3): 229, 1995 (Tixier 1995a). Based on: Cololejeunea spruceana Tixier, Candollea 46 (2): 286, 1991 (Tixier 1991), *nom. inval*.

** Cololejeunea stenophylla Herzog, Bot. Not. 100 (4): 330, 1947 (Herzog 1947).

*** Cololejeunea streimannii Pócs, Acta Bryolichenol. Asiat. 4: 96, 2011 (Pócs and Piippo 2011).

** Cololejeunea streimannii subsp. solomonensis Pócs, Acta Bryolichenol. Asiat. 4: 96, 2011 (Pócs and Piippo 2011).

*** Cololejeunea subalpina Pócs, Acta Bryolichenol. Asiat. 4: 98, 2011 (Pócs and Piippo 2011).

** Cololejeunea subcristata A.Evans, Bryologist 20 (2): 24, 1917 (Evans 1917b).

* Cololejeunea takamakae Tixier, Bryophyt. Biblioth. 27: 319, 1985 (Tixier 1985a). ^269^

*** Cololejeunea tanzaniae Pócs, J. Hattori Bot. Lab. 48: 312, 1980 (Pócs 1980b).

** Cololejeunea tenuiparietata Tixier, Trop. Bryol. 11: 56, 1995 (Tixier 1995b).

** Cololejeunea teurnoumensis Tixier, Ann. Fac. Sci. Univ. Phnom Penh 3: 183, 1970 (Tixier 1970b).

*** Cololejeunea timoi Pócs, Acta Bryolichenol. Asiat. 4: 98, 2011 (Pócs and Piippo 2011).

** Cololejeunea touwii Pócs, Acta Bryolichenol. Asiat. 4: 101, 2011 (Pócs and Piippo 2011).

*** Cololejeunea tranninhiana Tixier, Ann. Hist.-Nat. Mus. Natl. Hung. 66: 97, 1974 (Tixier 1974).

*** Cololejeunea trichomanis (Gottsche) Besch., Rev. Bryol. 19 (1): 14, 1892 (Bescherelle 1892). Bas.: Lejeunea trichomanis Gottsche, Abh. Naturwiss. Vereins Bremen 7: 362, 1882 (Gottsche 1882).

* Cololejeunea tuksapiana Tixier, Ann. Fac. Sci. Univ. Phnom Penh 3: 186, 1970 (Tixier 1970b). ^270^

** Cololejeunea vulcania Tixier, Ann. Fac. Sci. Univ. Phnom Penh 3: 181, 1970 (Tixier 1970b).

*** Cololejeunea yoshinagana (S.Hatt.) Mizut., J. Hattori Bot. Lab. 24: 250, 1961 (Mizutani 1961). Bas.: Leptocolea yoshinagana S.Hatt., Bull. Tokyo Sci. Mus. 11: 115, 1944 (Hattori 1944d).

* **subg. Metzgeriopsis (K.I.Goebel) Pócs**, Acta Bryolichenol. Asiat. 4: 106, 2011 (Pócs and Piippo 2011). Bas.: Metzgeriopsis K.I.Goebel, Ann. Jard. Bot. Buitenzorg 7 (1): 54, 1888 (Goebel 1888).

*** Cololejeunea metzgeriopsis (K.I.Goebel) Gradst., R.Wilson, Ilk.-Borg. et Heinrichs, Bot. J. Linn. Soc. 151 (3): 306, 2006 (Gradstein et al. 2006). Bas.: Lejeunea metzgeriopsis K.I.Goebel, Flora 72 (1): 2, 1889 (Goebel 1889).

*** **subg. Pedinolejeunea Benedix ex Mizut.**, J. Hattori Bot. Lab. 24: 240, 1961 (Mizutani 1961).

** Cololejeunea abnormis Mizut., J. Hattori Bot. Lab. 33: 260, 1970 (Mizutani 1970).

*** Cololejeunea adhaesiva (Mitt.) R.M.Schust., Beih. Nova Hedwigia 9: 177, 1963 (Schuster 1963a). Bas.: Lejeunea adhaesiva Mitt., J. Linn. Soc., Bot. 22 (146): 325, 1886 (Mitten 1886b).

** Cololejeunea adnata Tixier, Bull. Acad. Malgache (n.ser) 55 (1/2): 194, 1977 [1979] (Tixier 1977a).

*** Cololejeunea africana (Steph.) R.M.Schust., Beih. Nova Hedwigia 9: 173, 1963 (Schuster 1963a). Bas.: Physocolea africana Steph., Sp. Hepat. (Stephani) 5: 867, 1916 (Stephani 1916b).

** Cololejeunea ambeliensis Tixier, Bryophyt. Biblioth. 27: 142, 1985 (Tixier 1985a).

** Cololejeunea amieuensis Tixier, Nova Hedwigia 31: 748, 1979 (Tixier 1979b).

** Cololejeunea andapania Tixier, Bull. Acad. Malgache (n.ser) 55 (1/2): 178, 1977 [1979] (Tixier 1977a).

*** Cololejeunea angulata (Steph.) Mizut., J. Hattori Bot. Lab. 28: 108, 1965 (Mizutani 1965). Bas.: Leptocolea angulata Steph., Sp. Hepat. (Stephani) 5: 847, 1916 (Stephani 1916b).

*** Cololejeunea ankaiana Tixier, Bryophyt. Biblioth. 27: 62, 1985 (Tixier 1985a).

** Cololejeunea attilana Pócs, Magyar Bot. Kut. Ezredf. Tanul. Borhidi: 186, 2002 (Pócs 2002a).

*** Cololejeunea auriculata (E.W.Jones) R.M.Schust., Beih. Nova Hedwigia 9: 177, 1963 (Schuster 1963a). Bas.: Leptocolea auriculata E.W.Jones, Trans. Brit. Bryol. Soc. 2 (2): 152, 1953 (Jones 1953b).

** Cololejeunea autoica (Steph.) Grolle, Bryophyt. Biblioth. 48: 43, 1995 (Grolle 1995). Bas.: Physocolea autoica Steph., Sp. Hepat. (Stephani) 5: 867, 1916 (Stephani 1916b).

*** Cololejeunea bekkeri Tixier, Cryptog. Bryol. Lichénol. 16 (3): 229, 1995 (Tixier 1995a). Based on: Cololejeunea bekkeri Tixier, Candollea 46 (2): 269, 1991 (Tixier 1991), *nom. inval*.

*** Cololejeunea bischleriana Tixier, Bradea 3 (6): 36, 1980 (Tixier 1980a).

** Cololejeunea bolovenensis Tixier, Nat. Hist. Bull. Siam Soc. 24 (3/4): 442, 1973 (Tixier 1973b).

*** Cololejeunea borbonica Tixier, Bull. Acad. Malgache (n.ser) 55 (1/2): 188, 1977 [1979] (Tixier 1977a).

* Cololejeunea brunelii Tixier, Dacca Univ. Stud., B 15: 10, 1967 (Tixier 1967). ^271^

*** Cololejeunea cardiocarpa (Mont.) A.Evans, Mem. Torrey Bot. Club 8 (2): 172, 1902 (Evans 1902a). Bas.: Lejeunea cardiocarpa Mont., Hist. Phys. Cuba, Bot., Pl. Cell.: 476, 1842 (Montagne 1842a).

* Cololejeunea chittagongensis Tixier, Bryophyt. Biblioth. 27: 97, 1985 (Tixier 1985a). ^272^

*** Cololejeunea cocoscola Tixier, Cryptog. Bryol. Lichénol. 14 (3): 353, 1993 (Tixier 1993).

** Cololejeunea cremersii Tixier, Cryptog. Bryol. Lichénol. 16 (3): 229, 1995 (Tixier 1995a). Based on: Cololejeunea cremersii Tixier, Candollea 46 (2): 271, 1991 (Tixier 1991), *nom. inval*.

** Cololejeunea cristata (Steph.) R.M.Schust., Beih. Nova Hedwigia 9: 173, 1963 (Schuster 1963a). Bas.: Physocolea cristata Steph., Sp. Hepat. (Stephani) 5: 869, 1916 (Stephani 1916b).

** Cololejeunea cuneata (Lehm. et Lindenb.) Herzog, Bot. Not. 100 (4): 320, 1947 (Herzog 1947). Bas.: Jungermannia cuneata Lehm. et Lindenb., Nov. Stirp. Pug. 4: 56, 1832 (Lehmann 1832).

* Cololejeunea deroinii Tixier, Cryptog. Bryol. Lichénol. 14 (3): 355, 1993 (Tixier 1993).

* Cololejeunea deslooveri Vanden Berghen, Bull. Jard. Bot. Natl. Belg. 47 (1/2): 227, 1977 (Vanden Berghen 1977). ^273^

* Cololejeunea dzumacensis Tixier, Nova Hedwigia 31: 754, 1979 (Tixier 1979b). ^274^

*** Cololejeunea ecuadoriensis Pócs, Acta Bot. Hung. 44 (3/4): 372, 2002 (Pócs 2002b).

* Cololejeunea epiphylla G.Asthana et A.Shukla, Cryptog. Bryol. 31 (3): 218, 2010 (Asthana and Shukla 2010). ^275^

** Cololejeunea fissilobula Herzog, Trans. Brit. Bryol. Soc. 1 (4): 323, 1950 (Herzog 1950a).

** Cololejeunea florencei Tixier, Cryptog. Bryol. Lichénol. 14 (3): 355, 1993 (Tixier 1993).

** Cololejeunea foliicola S.C.Srivast. et G.Srivast., Proc. Indian Acad. Sci. Pl. Sci. 99 (2): 86, 1989 (Srivastava and Srivastava 1989a).

** Cololejeunea fructumarginata Tixier, Bryophyt. Biblioth. 27: 58, 1985 (Tixier 1985a).

*** Cololejeunea furcilobulata (Berrie et E.W.Jones) R.M.Schust., Beih. Nova Hedwigia 9: 178, 1963 (Schuster 1963a). Bas.: Leptocolea furcilobulata Berrie et E.W.Jones, Trans. Brit. Bryol. Soc. 2 (3): 417, 1954 (Jones 1954c).

*** Cololejeunea geissleriana Tixier, Bradea 3 (6): 37, 1980 (Tixier 1980a).

** Cololejeunea georgiana Tixier, Bryophyt. Biblioth. 27: 145, 1985 (Tixier 1985a).

*** Cololejeunea guadelupensis Tixier, Cryptog. Bryol. Lichénol. 16 (3): 229, 1995 (Tixier 1995a). Based on: Cololejeunea guadelupensis Tixier, Candollea 46 (2): 276, 1991 (Tixier 1991), *nom. inval*.

*** Cololejeunea hebridensis Tixier, Bot. Not. 128: 425, 1975 [1976] (Tixier 1975a).

*** Cololejeunea hinidumae Onr., Acta Bot. Acad. Sci. Hung. 25 (1/2): 109, 1979 (Onraedt 1979).

* Cololejeunea hoabinhiana Tixier, Ann. Hist.-Nat. Mus. Natl. Hung. 66: 91, 1974 (Tixier 1974). ^276^

** Cololejeunea hoeana Tixier, Bryophyt. Biblioth. 27: 56, 1985 (Tixier 1985a).

** Cololejeunea hungii Tixier, Ann. Hist.-Nat. Mus. Natl. Hung. 66: 91, 1974 (Tixier 1974).

*** Cololejeunea indosinica Tixier, Bryophyt. Biblioth. 27: 63, 1985 (Tixier 1985a).

** Cololejeunea inoueana Mizut., J. Hattori Bot. Lab. 57: 440, 1984 (Mizutani 1984c).

** Cololejeunea japonica (Schiffn.) Mizut., J. Hattori Bot. Lab. 24: 241, 1961 (Mizutani 1961). Bas.: Leptocolea japonica Schiffn., Ann. Bryol. 2: 92, 1929 (Schiffner 1929).

*** Cololejeunea jonesii Pócs, Acta Bot. Acad. Sci. Hung. 21 (3/4): 361, 1975 (Pócs 1975).

*** Cololejeunea kapingaensis H.A.Mill., Bryologist 59 (3): 170, 1956 (Miller 1956).

** Cololejeunea kiriromensis Tixier, Bryophyt. Biblioth. 27: 147, 1985 (Tixier 1985a).

*** Cololejeunea kulenensis Tixier, Bryophyt. Biblioth. 27: 71, 1985 (Tixier 1985a). *Nom. nov. pro Leptocolea verdoornii* Herzog, Ann. Bryol. 5: 97, 1932 (Herzog 1932a).

** Cololejeunea laevigata (Mitt.) R.M.Schust., J. Hattori Bot. Lab. 26: 241, 1963 (Schuster 1963b). Bas.: Lejeunea laevigata Mitt., Bot. antarct. voy. II (Fl. Nov.-Zel. 2): 157, 1854 (Mitten 1854).

*** Cololejeunea lanciloba Steph., Hedwigia 34 (5): 250, 1895 (Stephani 1895b).

*** Cololejeunea latilobula (Herzog) Tixier, Bryophyt. Biblioth. 27: 156, 1985 (Tixier 1985a). Bas.: Leptocolea latilobula Herzog, Symb. Sin. 5: 54, 1930 (Nicholson et al. 1930).

*** Cololejeunea latistyla R.L.Zhu, Hikobia 11: 544, 1994 (Zhu et al. 1994).

*** Cololejeunea leloutrei (E.W.Jones) R.M.Schust., Beih. Nova Hedwigia 9: 173, 1963 (Schuster 1963a). Bas.: Leptocolea leloutrei E.W.Jones, Trans. Brit. Bryol. Soc. 2 (2): 146, 1953 (Jones 1953b).

** Cololejeunea leloutrei var. microlobulata Tixier, Bryophyt. Biblioth. 27: 78, 1985 (Tixier 1985a).

** Cololejeunea leloutrei var. ulugurica Pócs ex Tixier, Bryophyt. Biblioth. 27: 74, 1985 (Tixier 1985a).

*** Cololejeunea lemuriana Tixier, Bull. Acad. Malgache (n.ser) 55 (1/2): 191, 1977 [1979] (Tixier 1977a).

** Cololejeunea littoralis Tixier, Bryophyt. Biblioth. 27: 163, 1985 (Tixier 1985a).

** Cololejeunea longistylis A.Evans, Trans. Connecticut Acad. Arts 10 (8): 453, 1900 (Evans 1900a).

*** Cololejeunea magnistyla (Horik.) Mizut., J. Hattori Bot. Lab. 24: 243, 1961 (Mizutani 1961). Bas.: Leptocolea magnistyla Horik., J. Sci. Hiroshima Univ., Ser. B, Div. 2, Bot. 1: 131, 1932 (Horikawa 1932c).

* Cololejeunea malaccensis Tixier, Bryophyt. Biblioth. 27: 42, 1985 (Tixier 1985a). ^277^

** Cololejeunea malayana Tixier, Bryophyt. Biblioth. 27: 154, 1985 (Tixier 1985a).

*** Cololejeunea marginata (Lehm. et Lindenb.) Pearson, Forh. Vidensk.-Selsk. Kristiania 1892 (8): 9, 1892 (Pearson 1892). Bas.: Jungermannia marginata Lehm. et Lindenb., Nov. Stirp. Pug. 5: 11, 1833 (Lehmann 1833).

* Cololejeunea maritima Tixier, Nova Hedwigia 31: 752, 1979 (Tixier 1979b).

*** Cololejeunea minutilobula Herzog, Rev. Bryol. Lichénol. 20 (1/2): 171, 1951 [1952] (Herzog 1951a).

*** Cololejeunea nigerica (E.W.Jones) R.M.Schust., Beih. Nova Hedwigia 9: 177, 1963 (Schuster 1963a). Bas.: Leptocolea nigerica E.W.Jones, Trans. Brit. Bryol. Soc. 2 (2): 150, 1953 (Jones 1953b).

** Cololejeunea occidentalis (E.W.Jones) Vanden Berghen, Rev. Bryol. Lichénol. 44 (4): 449, 1978 (Vanden Berghen 1978). Bas.: Leptocolea cristata var. occidentalis E.W.Jones, Trans. Brit. Bryol. Soc. 2 (2): 149, 1953 (Jones 1953b).

** Cololejeunea onraedtii Tixier, Bull. Acad. Malgache (n.ser) 55 (1/2): 202, 1977 [1979] (Tixier 1977a).

*** Cololejeunea pacifica Pócs, Acta Bot. Hung. 54 (1/2): 158, 2012 (Pócs 2012b).

** Cololejeunea panamensis G.Dauphin et Pócs, Trop. Bryol. 27: 76, 2006 (Dauphin et al. 2006).

*** Cololejeunea paucimarginata Tixier, Bryophyt. Biblioth. 27: 100, 1985 (Tixier 1985a).

** Cololejeunea perakensis Tixier, Bryophyt. Biblioth. 27: 95, 1985 (Tixier 1985a).

*** Cololejeunea plagiophylla Benedix, Feddes Repert. Spec. Nov. Regni Veg. Beih. 134: 73, 1953 (Benedix 1953).

*** Cololejeunea planissima (Mitt.) Abeyw., Ceylon J. Sci., Biol. Sci. 2 (1): 73, 1959 (Abeywickrama 1959). Bas.: Lejeunea planissima Mitt., J. Proc. Linn. Soc., Bot. 5 (18): 117, 1860 [1861] (Mitten 1860c).

** Cololejeunea planissima var. chagosensis Pócs, J. Bryol. 28 (1): 14, 2006 (Seaward et al. 2006).

** Cololejeunea praeruptorum Tixier, Bull. Acad. Malgache (n.ser) 55 (1/2): 205, 1977 [1979] (Tixier 1977a).

** Cololejeunea producta (Mitt.) S.Hatt., Fl. E. Himalaya: 533, 1966 (Hattori 1966c). Bas.: Lejeunea producta Mitt., J. Proc. Linn. Soc., Bot. 5 (18): 117, 1860 [1861] (Mitten 1860c).

* Cololejeunea punctata (Gottsche) Pearson, Forh. Vidensk.-Selsk. Kristiania 1892 (14): 9, 1893 (Pearson 1893). Bas.: Lejeunea punctata Gottsche, Abh. Naturwiss. Vereins Bremen 7: 361, 1882 (Gottsche 1882). ^278^

*** Cololejeunea raduliloba Steph., Hedwigia 34 (5): 251, 1895 (Stephani 1895b).

* Cololejeunea reineckeana Steph., Bot. Jahrb. Syst. 23 (1/2, 3): 309, 1896 (Stephani 1896a).

** Cololejeunea saltuum Tixier, Bull. Acad. Malgache (n.ser) 55 (1/2): 188, 1977 [1979] (Tixier 1977a).

** Cololejeunea sambiroana Tixier, Bryologist 82 (4): 608, 1979 (Tixier 1979d).

*** Cololejeunea saroltae Pócs, Acta Bot. Hung. 54 (1/2): 160, 2012 (Pócs 2012b).

*** Cololejeunea schusteri Pócs, Acta Bot. Hung. 44 (3/4): 376, 2002 (Pócs 2002b).

** Cololejeunea schwabei Herzog, J. Hattori Bot. Lab. 14: 54, 1955 (Herzog and Noguchi 1955).

*** Cololejeunea selangorensis Tixier, Bryophyt. Biblioth. 27: 166, 1985 (Tixier 1985a).

*** Cololejeunea shibiensis Mizut., J. Hattori Bot. Lab. 57: 437, 1984 (Mizutani 1984c).

*** Cololejeunea smitinandii Tixier, Bryophyt. Biblioth. 27: 131, 1985 (Tixier 1985a). Based on: Cololejeunea smitinandii Tixier, Nat. Hist. Bull. Siam Soc. 24 (3/4): 439, 1973 (Tixier 1973b), *nom. inval*.

** Cololejeunea stoniana Tixier, Bot. Not. 128: 429, 1975 [1976] (Tixier 1975a).

*** Cololejeunea stylosa Steph., Trans. Connecticut Acad. Arts 10 (8): 454, 1900 (Evans 1900a). Based on: Cololejeunea stylosa Steph., Hedwigia 27 (11/12): 289, 1888 (Stephani 1888c), *nom. inval*.

*** Cololejeunea subcardiocarpa Tixier, Bradea 3 (6): 39, 1980 (Tixier 1980a).

** Cololejeunea subinflata Tixier, Bull. Acad. Malgache (n.ser) 55 (1/2): 182, 1977 [1979] (Tixier 1977a).

*** Cololejeunea submarginata Tixier, Bradea 3 (6): 40, 1980 (Tixier 1980a).

** Cololejeunea subminutilobula Mizut., J. Hattori Bot. Lab. 24: 282, 1961 (Mizutani 1961). *Nom. nov. pro Leptocolea minutilobula* Horik., J. Sci. Hiroshima Univ., Ser. B, Div. 2, Bot. 1: 16, 1931 (Horikawa 1931b).

** Cololejeunea subscariosa (Spruce) Pócs, Acta Bot. Hung. 56 (1/2): 197, 2014 (Pócs et al. 2014). Bas.: Lejeunea subscariosa Spruce, Trans. & Proc. Bot. Soc. Edinburgh 15: 300, 1884 (Spruce 1884).

** Cololejeunea subtriapiculata Tixier, Nova Hedwigia 31: 744, 1979 (Tixier 1979b).

*** Cololejeunea succinea Tixier, Bull. Acad. Malgache (n.ser) 55 (1/2): 194, 1977 [1979] (Tixier 1977a).

*** Cololejeunea surinamensis Tixier, Bradea 3 (6): 42, 1980 (Tixier 1980a).

** Cololejeunea tahitensis Tixier, Cryptog. Bryol. Lichénol. 14 (3): 359, 1993 (Tixier 1993).

** Cololejeunea tamatavensis Tixier, Bull. Acad. Malgache (n.ser) 55 (1/2): 191, 1977 [1979] (Tixier 1977a).

* Cololejeunea taprobanea Tixier, Bryophyt. Biblioth. 27: 158, 1985 (Tixier 1985a).

* Cololejeunea thailandensis Tixier, Nat. Hist. Bull. Siam Soc. 24 (3/4): 441, 1973 (Tixier 1973b).

** Cololejeunea triapiculata (Herzog) Tixier, Gard. Bull. Singapore 25 (3): 344, 1971 (Tixier 1971). Bas.: Leptocolea triapiculata Herzog, Ann. Bryol. 5: 95, 1932 (Herzog 1932a).

* Cololejeunea tribracteata Tixier, Trop. Bryol. 11: 46, 1995 (Tixier 1995b). ^279^

* Cololejeunea tridentata Tixier, Bryophyt. Biblioth. 27: 83, 1985 (Tixier 1985a).

* Cololejeunea uchimae Amakawa, J. Jap. Bot. 33 (5): 142, 1958 (Amakawa 1958a). ^280^

*** Cololejeunea verwimpii Tixier, Cryptog. Bryol. Lichénol. 16 (3): 230, 1995 (Tixier 1995a).

*** Cololejeunea vidaliana Tixier, Nat. Hist. Bull. Siam Soc. 24 (3/4): 444, 1973 (Tixier 1973b).

** Cololejeunea vietnamensis Tixier, Bryophyt. Biblioth. 27: 127, 1985 (Tixier 1985a).

** Cololejeunea vitaliana Tixier, Cryptog. Bryol. Lichénol. 16 (3): 230, 1995 (Tixier 1995a).

*** Cololejeunea yakusimensis (S.Hatt.) Mizut., J. Hattori Bot. Lab. 57: 430, 1984 (Mizutani 1984c). Bas.: Leptocolea lanciloba var. yakusimensis S.Hatt., J. Jap. Bot. 18 (11): 655, 1942 (Hattori 1942).

* **subg. Protocolea R.M.Schust.**, Beih. Nova Hedwigia 9: 171, 1963 (Schuster 1963a).

** Cololejeunea chuahiana Pócs, Polish Bot. J. 47 (1): 11, 2002 (Pócs 2002c). ^281^

** Cololejeunea dauphinii R.L.Zhu, J. Bryol. 28 (3): 277, 2006 (Zhu 2006b). *Nom. nov. pro Cololejeunea tixieri* M.I.Morales et G.Dauphin, Trop. Bryol. 14: 133, 1998 (Morales and Dauphin 1998), *nom. illeg*. ^282^

** Cololejeunea disciflora Tixier, Bryologist 82 (4): 604, 1979 (Tixier 1979d). ^283^

*** **subg. Taeniolejeunea (Zwickel) Benedix**, Feddes Repert. Spec. Nov. Regni Veg. Beih. 134: 21, 1953 (Benedix 1953). Bas.: Taeniolejeunea Zwickel, Ann. Bryol. 6: 106, 1933 (Zwickel 1933).

* Cololejeunea amoena Benedix, Feddes Repert. Spec. Nov. Regni Veg. Beih. 134: 25, 1953 (Benedix 1953). ^284^

*** Cololejeunea appressa (A.Evans) Benedix, Feddes Repert. Spec. Nov. Regni Veg. Beih. 134: 31, 1953 (Benedix 1953). Bas.: Leptocolea appressa A.Evans, Bull. Torrey Bot. Club 39 (12): 606, 1912 [1913] (Evans 1912d).

* Cololejeunea bachmaensis Tixier, Rev. Bryol. Lichénol. 36 (3/4): 560, 1969 [1970] (Tixier 1969).

* Cololejeunea bontocensis Tixier, Rev. Bryol. Lichénol. 36 (3/4): 583, 1969 [1970] (Tixier 1969). ^285^

* Cololejeunea crassipapillata Tixier, Rev. Bryol. Lichénol. 36 (3/4): 565, 1969 [1970] (Tixier 1969).

** Cololejeunea eustacei Pócs, J. Bryol. 29 (2): 83, 2007 (Müller and Pócs 2007).

*** Cololejeunea falcata (Horik.) Benedix, Feddes Repert. Spec. Nov. Regni Veg. Beih. 134: 29, 1953 (Benedix 1953). Bas.: Physocolea falcata Horik., J. Sci. Hiroshima Univ., Ser. B, Div. 2, Bot. 1: 22, 1931 (Horikawa 1931b).

** Cololejeunea falcata var. madecassa Tixier, Bryologist 82 (4): 606, 1979 (Tixier 1979d).

* Cololejeunea flavida P.C.Wu et J.S.Lou, Acta Phytotax. Sin. 16 (4): 103, 1978 (Wu and Lou 1978). ^286^

** Cololejeunea flavovittata Pócs, Acta Bryolichenol. Asiat. 4: 120, 2011 (Pócs and Piippo 2011).

*** Cololejeunea floccosa (Lehm. et Lindenb.) Schiffn., Consp. Hepat. Arch. Ind.: 243, 1898 (Schiffner 1898b). Bas.: Jungermannia floccosa Lehm. et Lindenb., Nov. Stirp. Pug. 5: 26, 1833 (Lehmann 1833).

* Cololejeunea floccosa var. amoenoides Tixier, Cryptog. Bryol. Lichénol. 2 (1): 59, 1981 (Tixier 1981).

** Cololejeunea floccosa var. angustibracteata Tixier, Cryptog. Bryol. Lichénol. 2 (1): 65, 1981 (Tixier 1981).

** Cololejeunea floccosa var. aurita Benedix, Feddes Repert. Spec. Nov. Regni Veg. Beih. 134: 24, 1953 (Benedix 1953).

** Cololejeunea floccosa var. conivens Benedix, Feddes Repert. Spec. Nov. Regni Veg. Beih. 134: 24, 1953 (Benedix 1953).

* Cololejeunea floccosa var. ocellata Tixier, Cryptog. Bryol. Lichénol. 2 (1): 69, 1981 (Tixier 1981).

** Cololejeunea floccosa var. plicata Tixier, Cryptog. Bryol. Lichénol. 2 (1): 65, 1981 (Tixier 1981).

** Cololejeunea floccosa var. trivittata Tixier, Cryptog. Bryol. Lichénol. 2 (1): 62, 1981 (Tixier 1981).

** Cololejeunea gresicola Tixier, Rev. Bryol. Lichénol. 36 (3/4): 569, 1969 [1970] (Tixier 1969).

** Cololejeunea gynophthalma Benedix, Feddes Repert. Spec. Nov. Regni Veg. Beih. 134: 32, 1953 (Benedix 1953).

*** Cololejeunea inflata Steph., Hedwigia 34 (5): 249, 1895 (Stephani 1895b).

** Cololejeunea khiavensis Tixier, Rev. Bryol. Lichénol. 36 (3/4): 557, 1969 [1970] (Tixier 1969).

** Cololejeunea koratensis Tixier, Rev. Bryol. Lichénol. 36 (3/4): 585, 1969 [1970] (Tixier 1969).

* Cololejeunea manlinensis Tixier, Rev. Bryol. Lichénol. 36 (3/4): 563, 1969 [1970] (Tixier 1969).

** Cololejeunea maquilingensis Tixier, Rev. Bryol. Lichénol. 36 (3/4): 579, 1969 [1970] (Tixier 1969).

** Cololejeunea mutabilis Benedix, Feddes Repert. Spec. Nov. Regni Veg. Beih. 134: 27, 1953 (Benedix 1953).

** Cololejeunea nakajimae S.Hatt., J. Hattori Bot. Lab. 10: 57, 1953 (Hattori and Kodama 1953).

*** Cololejeunea ocellata (Horik.) Benedix, Feddes Repert. Spec. Nov. Regni Veg. Beih. 134: 38, 1953 (Benedix 1953). Bas.: Leptocolea ocellata Horik., J. Sci. Hiroshima Univ., Ser. B, Div. 2, Bot. 1: 86, 1932 (Horikawa 1932a).

*** Cololejeunea ocelloides (Horik.) Mizut., J. Hattori Bot. Lab. 24: 277, 1961 (Mizutani 1961). Bas.: Leptocolea ocelloides Horik., J. Sci. Hiroshima Univ., Ser. B, Div. 2, Bot. 2: 280, 1934 (Horikawa 1934).

*** Cololejeunea peraffinis (Schiffn.) Schiffn., Consp. Hepat. Arch. Ind.: 245, 1898 (Schiffner 1898b). Bas.: Lejeunea peraffinis Schiffn., Nova Acta Acad. Caes. Leop.-Carol. German. Nat. Cur. 60 (2): 242, 1893 (Schiffner 1893a).

* Cololejeunea peraffinis var. ciconiae Tixier, Bull. Jard. Bot. Natl. Belg. 59 (3/4): 444, 1989 (Tixier 1989).

** Cololejeunea peraffinis var. elegans Benedix, Feddes Repert. Spec. Nov. Regni Veg. Beih. 134: 35, 1953 (Benedix 1953).

** Cololejeunea peraffinis var. serrulata Schiffn. ex Benedix, Feddes Repert. Spec. Nov. Regni Veg. Beih. 134: 35, 1953 (Benedix 1953).

* Cololejeunea polisiana Tixier, Rev. Bryol. Lichénol. 36 (3/4): 550, 1969 [1970] (Tixier 1969). ^287^

*** Cololejeunea pseudofloccosa (Horik.) Benedix, Feddes Repert. Spec. Nov. Regni Veg. Beih. 134: 36, 1953 (Benedix 1953). Bas.: Leptocolea pseudofloccosa Horik., J. Sci. Hiroshima Univ., Ser. B, Div. 2, Bot. 1: 87, 1932 (Horikawa 1932a).

*** Cololejeunea pseudostephanii Tixier, Rev. Bryol. Lichénol. 36 (3/4): 571, 1969 [1970] (Tixier 1969).

** Cololejeunea setosa Mizut., J. Hattori Bot. Lab. 29: 163, 1966 (Mizutani 1966).

*** Cololejeunea sharpii Mizut., J. Hattori Bot. Lab. 39: 258, 1975 (Mizutani 1975).

*** Cololejeunea siamensis Steph., Bot. Tidsskr. 24 (3): 279, 1902 (Stephani 1902b).

*** Cololejeunea sphaerodonta Mizut., J. Hattori Bot. Lab. 29: 165, 1966 (Mizutani 1966).

*** Cololejeunea stephanii Schiffn. ex Benedix, Feddes Repert. Spec. Nov. Regni Veg. Beih. 134: 40, 1953 (Benedix 1953).

** Cololejeunea subfloccosa Mizut., J. Hattori Bot. Lab. 57: 168, 1984 (Mizutani 1984b).

** Cololejeunea subocelloides Mizut., J. Hattori Bot. Lab. 57: 163, 1984 (Mizutani 1984b).

* Cololejeunea tamdaoensis Tixier, Rev. Bryol. Lichénol. 36 (3/4): 567, 1969 [1970] (Tixier 1969).

** Cololejeunea verdoornii (S.Hatt.) S.Hatt., J. Hattori Bot. Lab. 17: 75, 1956 [1957] (Hattori 1956b). Bas.: Taeniolejeunea verdoornii S.Hatt., J. Jap. Bot. 17: 459, 1941 (Hattori 1941).

** Cololejeunea yipii R.L.Zhu, Beih. Nova Hedwigia 121: 346, 2001 (Zhu and So 2001).

** Cololejeunea zantenorum Pócs, Acta Bryolichenol. Asiat. 4: 127, 2011 (Pócs and Piippo 2011).

***Incertae sedis***

** Cololejeunea conchifolia (Gottsche) Gradst., J. Hattori Bot. Lab. 45: 109, 1979 (Gradstein and Hekking 1979). Bas.: Lejeunea conchifolia Gottsche, Ann. Sci. Nat. Bot. (sér. 5) 1: 163, 1864 (Gottsche 1864). ^288^

** Cololejeunea dankiaensis Tixier, Phytotaxa 220 (2): 199, 2015 (Söderström et al. 2015d). Based on: Cololejeunea dankiaensis Tixier, Rev. Bryol. Lichénol. 36 (3/4): 581, 1969 [1970] (Tixier 1969), *nom. inval*.

** Cololejeunea ensifera Tixier, Phytotaxa 220 (2): 199, 2015 (Söderström et al. 2015d). Based on: Cololejeunea ensifera Tixier, Rev. Bryol. Lichénol. 36 (3/4): 562, 1969 [1970] (Tixier 1969), *nom. inval*.

** Cololejeunea hamata Steph., Hedwigia 34 (5): 248, 1895 (Stephani 1895b).

** Cololejeunea herzogii K.I.Goebel, Biblioth. Bot. 87 (2): 269, 1916 (Stephani 1916a).

** Cololejeunea jamesii (Austin) M.E.Reiner et Pócs, Phytotaxa 208 (1): 98, 2015 (Pócs et al. 2015a). Bas.: Lejeunea jamesii Austin, Bull. Torrey Bot. Club 6 (30): 158, 1877 (Austin 1877).

** Cololejeunea sublatistyla Jian Wang bis et R.L.Zhu, Phytotaxa 161 (2): 165, 2014 (Wang et al. 2014a).

** Cololejeunea tixieri Onr., Bull. Jard. Bot. Natl. Belg. 59 (3/4): 436, 1989 (Onraedt 1989).

* Cololejeunea variifolia (Mitt.) Steph., Bot. Jahrb. Syst. 23 (1/2, 3): 309, 1896 (Stephani 1896a). Bas.: Lejeunea variifolia Mitt., Fl. vit.: 415, 1871 [1873] (Mitten 1871).

*** **Colura (Dumort.) Dumort.**, Recueil Observ. Jungerm.: 12, 1835 (Dumortier 1835). Bas.: Lejeunea sect. Colura Dumort., Syll. Jungerm. Europ.: 32, 1831 (Dumortier 1831).

** **subg. Colura**

** **sect. Colura**

*** Colura berghenii Jovet-Ast, Rev. Bryol. Lichénol. 22 (2/3): 245, 1953 [1954] (Jovet-Ast 1953).

*** Colura calyptrifolia (Hook.) Dumort., Recueil Observ. Jungerm.: 12, 1835 (Dumortier 1835). Bas.: Jungermannia calyptrifolia Hook., Brit. Jungermann.: tab. 43, 1813 (Hooker 1813).

*** Colura hedbergiana Pócs, J. Bryol. 14 (3): 499, 1987 (Jones and Pócs 1987).

*** Colura humbertii Jovet-Ast, Rev. Bryol. Lichénol. 22 (2/3): 251, 1953 [1954] (Jovet-Ast 1953).

*** Colura irrorata (Spruce) Heinrichs, Y.Yu, Schäf.-Verw. et Pócs, Phytotaxa 66: 58, 2012 (Heinrichs et al. 2012c). Bas.: Myriocolea irrorata Spruce, Trans. & Proc. Bot. Soc. Edinburgh 15: 305, 1884 (Spruce 1884).

** Colura junghuhniana (Prantl) Steph., Sp. Hepat. (Stephani) 5: 938, 1916 (Stephani 1916b). Bas.: Lejeunea junghuhniana Prantl, Hedwigia 29: xvi, 1890 (Prantl 1890).

** Colura medusa J.Eggers et Pócs, Chenia 11: 22, 2013 (Pócs 2013).

** Colura mizutanii Pócs, Chenia 11: 22, 2013 (Pócs 2013).

*** Colura rhynchophora Jovet-Ast, Rev. Bryol. Lichénol. 17 (1/4): 27, 1948 [1949] (Jovet-Ast 1948).

*** Colura tenuicornis (A.Evans) Steph., Sp. Hepat. (Stephani) 5: 942, 1916 (Stephani 1916b), *nom. conserv*. Bas.: Colurolejeunea tenuicornis A.Evans, Trans. Connecticut Acad. Arts 10 (8): 455, 1900 (Evans 1900a).

** **sect. Gamolepis Jovet-Ast**, Cryptog. Bryol. Lichénol. 4 (3): 207, 1983 (Jovet-Ast 1983).

*** Colura cristata Jovet-Ast, Rev. Bryol. Lichénol. 22 (2/3): 291, 1953 [1954] (Jovet-Ast 1953).

*** Colura greig-smithii Jovet-Ast, Rev. Bryol. Lichénol. 22 (2/3): 293, 1953 [1954] (Jovet-Ast 1953).

*** Colura inflata K.I.Goebel, Ann. Jard. Bot. Buitenzorg 39: 11, 1928 (Goebel 1928).

*** Colura jovet-astiae Grolle, J. Hattori Bot. Lab. 28: 44, 1965 (Grolle 1965b). *Nom. nov. pro Colura undulata* Jovet-Ast, Rev. Bryol. Lichénol. 30 (1/2): 7, 1961 (Jovet-Ast 1961), *nom. illeg*.

*** Colura meijeri Jovet-Ast, Rev. Bryol. Lichénol. 22 (2/3): 290, 1953 [1954] (Jovet-Ast 1953).

*** Colura obvoluta Jovet-Ast, Cryptog. Bryol. Lichénol. 4 (3): 207, 1983 (Jovet-Ast 1983).

*** Colura ornata K.I.Goebel, Ann. Jard. Bot. Buitenzorg 9 (1): 26, 1890 [1891] (Goebel 1890).

*** Colura palawanensis Jovet-Ast, Rev. Bryol. Lichénol. 22 (2/3): 305, 1953 [1954] (Jovet-Ast 1953).

*** Colura valida Jovet-Ast, Rev. Bryol. Lichénol. 30 (1/2): 6, 1961 (Jovet-Ast 1961).

*** Colura verdoornii Herzog et Jovet-Ast, Rev. Bryol. Lichénol. 22 (2/3): 288, 1953 [1954] (Jovet-Ast 1953).

** **sect. Harmophyllum Grolle**, J. Hattori Bot. Lab. 28: 44, 1965 (Grolle 1965b).

*** Colura andoi Gradst. et Jovet-Ast, Hikobia 9: 355, 1986 (Gradstein 1986).

*** Colura ari (Steph.) Steph., Sp. Hepat. (Stephani) 5: 936, 1916 (Stephani 1916b). Bas.: Colurolejeunea ari Steph., Hedwigia 35 (3): 73, 1896 (Stephani 1896b).

*** Colura australiensis Jovet-Ast, Rev. Bryol. Lichénol. 22 (2/3): 260, 1953 [1954] (Jovet-Ast 1953).

*** Colura brevistyla Herzog, Beih. Bot. Centralbl. 38 (2): 331, 1921 (Herzog 1921).

*** Colura calderae Pócs, J. Bryol. 29 (2): 84, 2007 (Müller and Pócs 2007).

*** Colura clementis Grolle, J. Hattori Bot. Lab. 28: 45, 1965 (Grolle 1965b).

*** Colura conica (Sande Lac.) K.I.Goebel, Ann. Jard. Bot. Buitenzorg 39: 3, 1928 (Goebel 1928). Bas.: Lejeunea conica Sande Lac., Ann. Mus. Bot. Lugduno-Batavi 1: 311, 1864 (Sande Lacoste 1864).

*** Colura corynophora (Nees, Lindenb. et Gottsche) Trevis., Mem. Reale Ist. Lombardo Sci. (Ser. 3), C. Sci. Mat. 4 (13): 402, 1877 (Trevisan 1877). Bas.: Lejeunea corynophora Nees, Lindenb. et Gottsche, Observ. bot.: 474, 1843 (Gottsche et al. 1843).

*** Colura crenulata Grolle, J. Hattori Bot. Lab. 28: 46, 1965 (Grolle 1965b).

*** Colura crispiloba Jovet-Ast, Cryptog. Bryol. Lichénol. 4 (3): 205, 1983 (Jovet-Ast 1983).

** Colura cylindrica Herzog, Svensk Bot. Tidskr. 46 (1): 106, 1952 (Herzog 1952e).

*** Colura cymbalifera Herzog et Jovet-Ast, Rev. Bryol. Lichénol. 22 (2/3): 268, 1953 [1954] (Jovet-Ast 1953).

*** Colura digitalis (Mitt.) Steph., Sp. Hepat. (Stephani) 5: 931, 1916 (Stephani 1916b). Bas.: Lejeunea digitalis Mitt., J. Linn. Soc., Bot. 22 (146): 325, 1886 (Mitten 1886b).

** Colura digitalis var. mucronata Pócs, Fragm. Florist. Geobot. 40 (1): 263, 1995 (Pócs 1995).

*** Colura dusenii Steph., Sp. Hepat. (Stephani) 5: 931, 1916 (Stephani 1916b). Based on: Colurolejeunea dusenii Steph., Hedwigia 31 (4): 168, 1892 (Stephani 1892g).

*** Colura fastigiata Jovet-Ast, Rev. Bryol. Lichénol. 27 (1/2): 28, 1958 (Jovet-Ast 1958).

*** Colura fistulosa Jovet-Ast, Cryptog. Bryol. Lichénol. 4 (3): 211, 1983 (Jovet-Ast 1983).

*** Colura hattoriana Pócs, J. Hattori Bot. Lab. 74: 47, 1993 (Pócs 1993).

*** Colura heimii Jovet-Ast, Rev. Bryol. Lichénol. 22 (2/3): 275, 1953 [1954] (Jovet-Ast 1953).

*** Colura hemisphaerica Jovet-Ast, Rev. Bryol. Lichénol. 22 (2/3): 267, 1953 [1954] (Jovet-Ast 1953).

*** Colura herzogii Jovet-Ast, Rev. Bryol. Lichénol. 22 (2/3): 261, 1953 [1954] (Jovet-Ast 1953).

*** Colura inuii Horik., J. Sci. Hiroshima Univ., Ser. B, Div. 2, Bot. 1: 68, 1931 (Horikawa 1931a).

** Colura koponenii Pócs, Chenia 11: 29, 2013 (Pócs 2013).

** Colura mauritiana Pócs, Cryptog. Bryol. Lichénol. 18 (3): 196, 1997 (Pócs 1997a).

*** Colura maxima Jovet-Ast, Rev. Bryol. Lichénol. 22 (2/3): 284, 1953 [1954] (Jovet-Ast 1953).

*** Colura mosenii Steph., Sp. Hepat. (Stephani) 5: 940, 1916 (Stephani 1916b).

** Colura norrisii Pócs, Chenia 11: 29, 2013 (Pócs 2013).

*** Colura obesa Jovet-Ast, Rev. Bryol. Lichénol. 22 (2/3): 273, 1953 [1954] (Jovet-Ast 1953).

*** Colura pallida Steph., Sp. Hepat. (Stephani) 5: 941, 1916 (Stephani 1916b).

*** Colura pluridentata Jovet-Ast, Rev. Bryol. Lichénol. 22 (2/3): 265, 1953 [1954] (Jovet-Ast 1953).

*** Colura speciosa Jovet-Ast, Rev. Bryol. Lichénol. 22 (2/3): 307, 1953 [1954] (Jovet-Ast 1953).

** Colura streimannii Pócs, Polish Bot. J. 60 (1): 7, 2015 (Pócs 2015b).

*** Colura superba (Mont.) Steph., Sp. Hepat. (Stephani) 5: 941, 1916 (Stephani 1916b). Bas.: Lejeunea superba Mont., Ann. Sci. Nat. Bot. (sér. 3) 10: 115, 1848 (Montagne 1848).

*** Colura thomeensis Pócs, Bryologist 114 (2): 363, 2011 (Pócs 2011b).

*** Colura tortifolia (Nees et Mont.) Trevis., Mem. Reale Ist. Lombardo Sci. (Ser. 3), C. Sci. Mat. 4 (13): 402, 1877 (Trevisan 1877). Bas.: Lejeunea tortifolia Nees et Mont., Ann. Sci. Nat. Bot. (sér. 2) 19: 265, 1843 (Montagne 1843).

*** Colura tutuilana (Pearson) H.A.Mill., Phytologia 47 (4): 322, 1981 (Miller 1981). Bas.: Colurolejeunea tutuilana Pearson, Amer. Samoa: 151, 1924 (Pearson 1924a).

*** Colura ulei Jovet-Ast, Rev. Bryol. Lichénol. 22 (2/3): 270, 1953 [1954] (Jovet-Ast 1953).

*** Colura vitiensis Pócs et J.Eggers, Polish Bot. J. 52 (2): 88, 2007 (Pócs and Eggers 2007).

** **sect. Oidocorys Jovet-Ast ex Grolle**, J. Hattori Bot. Lab. 32: 140, 1969 (Grolle 1969c).

*** Colura itatyana Steph., Sp. Hepat. (Stephani) 5: 932, 1916 (Stephani 1916b).

*** Colura naumannii (Schiffn.) Steph., Sp. Hepat. (Stephani) 5: 935, 1916 (Stephani 1916b). Bas.: Colurolejeunea naumannii Schiffn., Hepat. (Engl.-Prantl): 121, 1893 (Schiffner 1893b).

*** Colura ornithocephala Herzog, Svensk Bot. Tidskr. 46 (1): 107, 1952 (Herzog 1952e).

*** Colura pulcherrima Jovet-Ast, Rev. Bryol. Lichénol. 22 (2/3): 235, 1953 [1954] (Jovet-Ast 1953).

** Colura pulcherrima var. bartlettii Jovet-Ast, Cryptog. Bryol. Lichénol. 1 (3): 278, 1980 (Jovet-Ast 1980).

*** Colura saccophylla E.A.Hodgs. et Herzog, Trans. & Proc. Roy. Soc. New Zealand 77 (2): 253, 1949 (Herzog 1949a).

*** Colura schusteri Grolle, J. Hattori Bot. Lab. 32: 140, 1969 (Grolle 1969c).

** **subg. Glotta Grolle et R.L.Zhu**, J. Hattori Bot. Lab. 92: 187, 2002 (Grolle and Zhu 2002).

** **sect. Glotta Grolle et R.L.Zhu**, J. Hattori Bot. Lab. 92: 187, 2002 (Grolle and Zhu 2002).

*** Colura bicornis Jovet-Ast, Rev. Bryol. Lichénol. 25 (3/4): 272, 1956 [1957] (Jovet-Ast 1956).

*** Colura bisvoluta Herzog et Jovet-Ast, Rev. Bryol. Lichénol. 22 (2/3): 228, 1953 [1954] (Jovet-Ast 1953).

*** Colura inornata Jovet-Ast, Rev. Bryol. Lichénol. 25 (3/4): 275, 1956 [1957] (Jovet-Ast 1956).

*** Colura karstenii K.I.Goebel, Pflanzenbiol. Schilderungen 2 (1): 153, 1891 (Goebel 1891).

*** Colura queenslandica B.M.Thiers, Brittonia 39 (2): 175, 1987 (Thiers 1987b).

*** Colura saroltae Pócs, J. Bryol. 14 (3): 497, 1987 (Jones and Pócs 1987).

*** Colura strophiolata Jovet-Ast, Rev. Bryol. Lichénol. 42 (4): 917, 1976 (Jovet-Ast 1976).

*** Colura usambarica E.W.Jones, J. Bryol. 14 (3): 495, 1987 (Jones and Pócs 1987).

** **sect. Heterophyllum Jovet-Ast**, Cryptog. Bryol. Lichénol. 4 (3): 213, 1983 (Jovet-Ast 1983).

*** Colura acroloba (Prantl) Jovet-Ast, Rev. Bryol. Lichénol. 22 (2/3): 297, 1953 [1954] (Jovet-Ast 1953). Bas.: Lejeunea acroloba Prantl, Hedwigia 29: xiv, 1890 (Prantl 1890).

*** Colura corniantha Grolle, J. Hattori Bot. Lab. 28: 44, 1965 (Grolle 1965b). *Nom. nov. pro Colura cornuta* Jovet-Ast, Rev. Bryol. Lichénol. 30 (1/2): 11, 1961 (Jovet-Ast 1961), *nom. illeg*.

** Colura denticulata Jovet-Ast, Rev. Bryol. Lichénol. 23 (1/2): 2, 1954 (Jovet-Ast 1954).

*** Colura galeata Jovet-Ast, Rev. Bryol. Lichénol. 35 (1/4): 146, 1967 [1968] (Jovet-Ast 1967a).

** Colura imperfecta Steph., Sp. Hepat. (Stephani) 5: 938, 1916 (Stephani 1916b).

*** Colura siamensis Jovet-Ast, Rev. Bryol. Lichénol. 35 (1/4): 139, 1967 [1968] (Jovet-Ast 1967b).

*** Colura tixieri Jovet-Ast, Rev. Bryol. Lichénol. 30 (1/2): 9, 1961 (Jovet-Ast 1961).

*** Colura vietnamensis Jovet-Ast et Tixier, Rev. Bryol. Lichénol. 27 (3/4): 205, 1958 [1959] (Jovet-Ast and Tixier 1958).

***Incertae sedis***

** Colura amboinensis Steph., Sp. Hepat. (Stephani) 5: 935, 1916 (Stephani 1916b).

*** Colura hirta Steph., Sp. Hepat. (Stephani) 5: 932, 1916 (Stephani 1916b).

*** **Diplasiolejeunea (Spruce) Schiffn.**, Hepat. (Engl.-Prantl): 121, 1893 (Schiffner 1893b). Bas.: Lejeunea subg. Diplasiolejeunea Spruce, Trans. & Proc. Bot. Soc. Edinburgh 15: 301, 1884 (Spruce 1884). ^289^

*** Diplasiolejeunea utriculata Steph., Sp. Hepat. (Stephani) 5: 920, 1916 (Stephani 1916b).

** **subg. Austrolejeuneopsis R.M.Schust.**, Bull. Torrey Bot. Club 97 (6): 349, 1970 (Schuster 1970b).

*** Diplasiolejeunea alata Jovet-Ast, Rev. Bryol. Lichénol. 17 (1/4): 31, 1948 [1949] (Jovet-Ast 1948).

*** Diplasiolejeunea eggersii Pócs, Bryologist 109 (3): 408, 2006 (Pócs 2006b).

*** Diplasiolejeunea erostrata Schäf.-Verw., Cryptog. Bryol. 25 (1): 3, 2004 (Schäfer-Verwimp 2004).

* Diplasiolejeunea guadalupensis Steph., Sp. Hepat. (Stephani) 5: 924, 1916 (Stephani 1916b). ^290^

* Diplasiolejeunea heimii Jovet-Ast, Rev. Bryol. Lichénol. 29 (1/2): 39, 1960 (Jovet-Ast 1960). ^291^

** Diplasiolejeunea involuta S.Winkl., Rev. Bryol. Lichénol. 35 (1/4): 320, 1967 [1968] (Winkler 1967).

** Diplasiolejeunea involuta subsp. andicola Pócs, Cryptog. Bryol. Lichénol. 19 (1): 12, 1998 (León et al. 1998).

*** Diplasiolejeunea johnsonii A.Evans, Bull. Torrey Bot. Club 39 (12): 603, 1912 [1913] (Evans 1912d).

** Diplasiolejeunea johnsonii var. mexicana Jovet-Ast, Rev. Bryol. Lichénol. 29 (1/2): 39, 1960 (Jovet-Ast 1960).

* Diplasiolejeunea montecristensis S.Winkl., Rev. Bryol. Lichénol. 35 (1/4): 321, 1967 [1968] (Winkler 1967). ^292^

*** Diplasiolejeunea papilionacea R.M.Schust., Phytologia 39 (6): 431, 1978 (Schuster 1978b).

*** Diplasiolejeunea pauckertii (Nees) Steph., Sp. Hepat. (Stephani) 5: 924, 1916 (Stephani 1916b). Bas.: Lejeunea pauckertii Nees, Syn. Hepat. 3: 392, 1845 (Gottsche et al. 1845b).

*** Diplasiolejeunea pluridentata Schäf.-Verw., Haussknechtia 8: 71, 2001 (Schäfer- Verwimp 2001b).

*** Diplasiolejeunea replicata (Spruce) Steph., Sp. Hepat. (Stephani) 5: 926, 1916 (Stephani 1916b). Bas.: Lejeunea replicata Spruce, Trans. & Proc. Bot. Soc. Edinburgh 15: 302, 1884 (Spruce 1884).

** Diplasiolejeunea rudolphiana Steph., Hedwigia 35 (3): 79, 1896 (Stephani 1896b). ^293^

*** Diplasiolejeunea runssorensis Steph., Bot. Jahrb. Syst. 20 (3): 318, 1895 (Stephani 1895a).

*** Diplasiolejeunea unidentata (Lehm. et Lindenb.) Schiffn., Bot. Jahrb. Syst. 23 (5): 583, 1897 (Schiffner 1897). Bas.: Jungermannia unidentata Lehm. et Lindenb., Nov. Stirp. Pug. 6: 48, 1834 (Lehmann 1834).

** **subg. Diplasiolejeunea**

*** Diplasiolejeunea armatiloba Steph., Hedwigia 35 (3): 80, 1896 (Stephani 1896b).

*** Diplasiolejeunea borhidiana Reyes Montoya, Acta Bot. Acad. Sci. Hung. 28 (1/2): 177, 1982 [1983] (Reyes 1982).

*** Diplasiolejeunea brunnea Steph., Sp. Hepat. (Stephani) 5: 922, 1916 (Stephani 1916b).

** Diplasiolejeunea caribea Tixier, Bryophyt. Biblioth. 27: 377, 1985 (Tixier 1985a).

*** Diplasiolejeunea cavifolia Steph., Sp. Hepat. (Stephani) 5: 918, 1916 (Stephani 1916b). *Nom. nov. pro Lejeunea cavifolia* Steph., Bot. Jahrb. Syst. 8 (2): 89, 1886 (Stephani 1886d), *nom. illeg*.

*** Diplasiolejeunea cobrensis Steph., Sp. Hepat. (Stephani) 5: 923, 1916 (Stephani 1916b).

** Diplasiolejeunea cobrensis subsp. antsirananae Pócs, Candollea 56 (1): 72, 2001 (Pócs 2001).

** Diplasiolejeunea cogoensis M.Infante, Heras et Pócs, Trop. Bryol. 17: 9, 1999 (Infante et al. 1999).

** Diplasiolejeunea cubensis Tixier, Bryophyt. Biblioth. 27: 389, 1985 (Tixier 1985a).

* Diplasiolejeunea glaziovii Tixier, Bryophyt. Biblioth. 27: 397, 1985 (Tixier 1985a). ^294^

*** Diplasiolejeunea ingekarolae Schäf.-Verw., Herzogia 19: 239, 2006 (Schäfer-Verwimp 2006).

** Diplasiolejeunea jonesii Tixier, Acta Bot. Hung. 30 (1/2): 21, 1984 (Tixier 1984).

*** Diplasiolejeunea leiocarpa Jovet-Ast, Rev. Bryol. Lichénol. 16 (1/2): 34, 1947 [1948] (Jovet-Ast 1947b).

*** Diplasiolejeunea malleiformis (A.Evans) Tixier, Bryophyt. Biblioth. 27: 351, 1985 (Tixier 1985a). Bas.: Diplasiolejeunea pellucida var. malleiformis A.Evans, Bull. Torrey Bot. Club 39 (5): 215, 1912 (Evans 1912c).

** Diplasiolejeunea palustrium Tixier, Acta Bot. Hung. 30 (1/2): 19, 1984 (Tixier 1984).

*** Diplasiolejeunea pellucida (C.F.W.Meissn. ex Spreng.) Schiffn., Hepat. (Engl.-Prantl): 121, 1893 (Schiffner 1893b). Bas.: Jungermannia pellucida C.F.W.Meissn. ex Spreng. Syst. Veg. (ed. 16) [Sprengel] 4 (2): 325, 1827 (Sprengel 1827b).

** Diplasiolejeunea phyllarthronii Tixier, Acta Bot. Hung. 30 (1/2): 19, 1984 (Tixier 1984).

*** Diplasiolejeunea pocsii Reyes Montoya, Acta Bot. Acad. Sci. Hung. 28 (1/2): 173, 1982 [1983] (Reyes 1982).

** **subg. Physolejeunea R.M.Schust.**, Bull. Torrey Bot. Club 97 (6): 348, 1970 (Schuster 1970b).

*** Diplasiolejeunea andringitrae Schäf.-Verw., Cryptog. Bryol. 27 (4): 447, 2006 (Pócs and Schäfer-Verwimp 2006).

*** Diplasiolejeunea cornuta Steph., Sp. Hepat. (Stephani) 5: 918, 1916 (Stephani 1916b).

*** Diplasiolejeunea jovet-astiae Grolle, Feddes Repert. 73 (2): 84, 1966 (Grolle 1966b).

*** Diplasiolejeunea kraussiana (Lindenb.) Steph., Sp. Hepat. (Stephani) 5: 919, 1916 (Stephani 1916b). Bas.: Lejeunea kraussiana Lindenb., Syn. Hepat. 3: 393, 1845 (Gottsche et al. 1845b).

*** Diplasiolejeunea ornata Pócs et Schäf.-Verw., Cryptog. Bryol. 27 (4): 440, 2006 (Pócs and Schäfer-Verwimp 2006).

*** Diplasiolejeunea patelligera Herzog, Svensk Bot. Tidskr. 42 (3): 240, 1948 (Herzog 1948).

*** Diplasiolejeunea plicatiloba (Hook.f. et Taylor) Grolle, J. Hattori Bot. Lab. 45: 175, 1979 (Grolle 1979c). Bas.: Jungermannia plicatiloba Hook.f. et Taylor, London J. Bot. 4: 92, 1845 (Hooker and Taylor 1845).

*** Diplasiolejeunea pusilla Grolle, Feddes Repert. 86 (1/2): 79, 1975 (Grolle 1975a).

*** Diplasiolejeunea ranomafanae Pócs, Cryptog. Bryol. 27 (4): 444, 2006 (Pócs and Schäfer-Verwimp 2006).

*** Diplasiolejeunea villaumei Steph., Sp. Hepat. (Stephani) 5: 921, 1916 (Stephani 1916b).

*** Diplasiolejeunea zakiae Tixier, Lindbergia 4 (1/2): 123, 1977 (Tixier 1977b).

***Incertae sedis***

*** Diplasiolejeunea albifolia (Taylor) E.W.Jones, Trans. Brit. Bryol. Soc. 2 (3): 393, 1954 (Jones 1954a). Bas.: Lejeunea albifolia Taylor, London J. Bot. 5: 399, 1846 (Taylor 1846b).

*** Diplasiolejeunea aulae E.W.Jones, J. Bryol. 7 (4): 552, 1973 [1974] (Jones 1973).

** Diplasiolejeunea auriculata Tixier, Rev. Bryol. Lichénol. 45 (2): 210, 1979 (Tixier 1979c).

*** Diplasiolejeunea buckii Grolle, Beitr. Phytotax. 15: 105, 1992 (Grolle 1992a).

** Diplasiolejeunea columbica Tixier, Cryptog. Bryol. Lichénol. 4 (3): 233, 1983 (Tixier 1983a).

* Diplasiolejeunea comorensis Tixier, Rev. Bryol. Lichénol. 45 (2): 213, 1979 (Tixier 1979c).

** Diplasiolejeunea cyanguguensis Tixier, Trop. Bryol. 11: 67, 1995 (Tixier 1995b).

*** Diplasiolejeunea deslooveri Vanden Berghen, Bull. Jard. Bot. Natl. Belg. 47 (1/2): 217, 1977 (Vanden Berghen 1977).

** Diplasiolejeunea ensifera Tixier, Rev. Bryol. Lichénol. 45 (2): 215, 1979 (Tixier 1979c).

* Diplasiolejeunea evansii Tixier, Bryophyt. Biblioth. 27: 360, 1985 (Tixier 1985a). ^295^

** Diplasiolejeunea gradsteinii Tixier, Trop. Bryol. 11: 67, 1995 (Tixier 1995b).

*** Diplasiolejeunea grandirostrata Schäf.-Verw., Cryptog. Bryol. 25 (1): 7, 2004 (Schäfer-Verwimp 2004).

*** Diplasiolejeunea grolleana Reyes Montoya, Acta Bot. Acad. Sci. Hung. 28 (1/2): 175, 1982 [1983] (Reyes 1982).

** Diplasiolejeunea hamata Tixier, Rev. Bryol. Lichénol. 45 (2): 217, 1979 (Tixier 1979c).

** Diplasiolejeunea insignis Tixier, Lindbergia 4 (1/2): 120, 1977 (Tixier 1977b).

* Diplasiolejeunea integerrima Tixier, Rev. Bryol. Lichénol. 45 (2): 219, 1979 (Tixier 1979c). ^296^

*** Diplasiolejeunea lanceolata Grolle, Beitr. Phytotax. 15: 107, 1992 (Grolle 1992a).

*** Diplasiolejeunea latipuensis Tixier, Cryptog. Bryol. Lichénol. 16 (3): 230, 1995 (Tixier 1995a). Based on: Diplasiolejeunea latipuensis Tixier, Candollea 46 (2): 294, 1991 (Tixier 1991), *nom. inval*.

** Diplasiolejeunea lemuriana Tixier, Lindbergia 4 (1/2): 120, 1977 (Tixier 1977b).

** Diplasiolejeunea longilobula Herzog, Trans. Brit. Bryol. Soc. 1 (4): 325, 1950 (Herzog 1950a).

** Diplasiolejeunea magnistipula Tixier, Ann. Fac. Sci. Yaoundé 20: 7, 1975 (Tixier 1975b).

*** Diplasiolejeunea mayaykuensis Schäf.-Verw. et Heinrichs, Polish Bot. J. 58 (1): 143, 2013 (Schäfer-Verwimp et al. 2013).

*** Diplasiolejeunea onraedtii Grolle, Feddes Repert. 89 (5/6): 301, 1978 (Grolle 1978a).

** Diplasiolejeunea ramicola Tixier, Rev. Bryol. Lichénol. 45 (2): 222, 1979 (Tixier 1979c).

** Diplasiolejeunea riclefgrollei Schäf.-Verw., Cryptog. Bryol. 26 (1): 37, 2005 (Schäfer-Verwimp 2005).

** Diplasiolejeunea subcornuta Tixier, Lindbergia 4 (1/2): 122, 1977 (Tixier 1977b).

*** Diplasiolejeunea symoensii Vanden Berghen, Bull. Soc. Roy. Bot. Belgique 92: 126, 1960 (Vanden Berghen 1960b).

** **Haplolejeunea Grolle**, J. Hattori Bot. Lab. 39: 205, 1975 (Grolle 1975b).

*** Haplolejeunea cucullata (Steph.) Grolle, J. Hattori Bot. Lab. 45: 176, 1979 (Grolle 1979c). Bas.: Cheilolejeunea cucullata Steph., Sp. Hepat. (Stephani) 5: 644, 1914 (Stephani 1914b).

** Haplolejeunea sticta Grolle, J. Hattori Bot. Lab. 39: 205, 1975 (Grolle 1975b).

** **Macrocolura R.M.Schust.**, J. Hattori Bot. Lab. 75: 233, 1994 (Schuster 1994).

*** Macrocolura sagittistipula (Spruce) R.M.Schust., J. Hattori Bot. Lab. 75: 233, 1994 (Schuster 1994). Bas.: Lejeunea sagittistipula Spruce, Trans. & Proc. Bot. Soc. Edinburgh 15: 304, 1884 (Spruce 1884).

*** **Myriocoleopsis Schiffn.**, Hedwigia 81 (5/6): 234, 1944 (Schiffner 1944).

*** Myriocoleopsis fluviatilis (Steph.) M.E.Reiner et Gradst., J. Bryol. 19 (3): 639, 1997 (Reiner-Drehwald and Gradstein 1997). Bas.: Cololejeunea fluviatilis Steph., Hedwigia 34 (5): 248, 1895 (Stephani 1895b).

*** Myriocoleopsis gymnocolea (Spruce) M.E.Reiner et Gradst., J. Bryol. 19 (3): 640, 1997 (Reiner-Drehwald and Gradstein 1997). Bas.: Lejeunea gymnocolea Spruce, Trans. & Proc. Bot. Soc. Edinburgh 15: 296, 1884 (Spruce 1884).

*** Myriocoleopsis minutissima (Sm.) R.L.Zhu, Y.Yu et Pócs, Phytotaxa 183 (4): 293, 2014 (Yu et al. 2014). Bas.: Jungermannia minutissima Sm., Engl. Bot. 23: tab. 1633, 1806 (Smith and Sowerby 1806).

*** Myriocoleopsis minutissima subsp. myriocarpa (Nees et Mont.) R.L.Zhu, Y.Yu et Pócs, Phytotaxa 183 (4): 294, 2014 (Yu et al. 2014). Bas.: Lejeunea myriocarpa Nees et Mont., Hist. Phys. Cuba, Bot., Pl. Cell.: 473, 1842 (Montagne 1842a).

** Myriocoleopsis vuquangensis (Pócs et Ninh) Pócs, Trop. Bryol. 31: 124, 2010 (Pócs 2010a). Bas.: Cololejeunea vuquangensis Pócs et Ninh, Acta Bot. Hung. 47 (1/2): 156, 2005 (Pócs and Ninh 2005).

** **Nephelolejeunea Grolle**, J. Hattori Bot. Lab. 37: 252, 1973 (Grolle 1973b).

** Nephelolejeunea bidentata B.M.Thiers ex L.Söderstr. et A.Hagborg, Phytotaxa 202 (1): 65, 2015 (Pócs et al. 2015c). Based on: Austrolejeunea bidentata B.M.Thiers, Bryologist 88 (4): 350, 1985 [1986] (Thiers 1985), *nom. inval*.

** Nephelolejeunea carcharias M.A.M.Renner, Syst. Bot. 34 (4): 621, 2009 (Renner et al. 2009).

*** Nephelolejeunea conchophylla Grolle, J. Hattori Bot. Lab. 48: 164, 1980 (Grolle 1980a).

** Nephelolejeunea fragilis (R.M.Schust.) L.Söderstr. et A.Hagborg, Phytotaxa 202 (1): 65, 2015 (Pócs et al. 2015c). Bas.: Cololejeunea fragilis R.M.Schust., Phytologia 56 (7): 458, 1985 (Schuster 1985c).

*** Nephelolejeunea hamata Grolle, J. Hattori Bot. Lab. 48: 167, 1980 (Grolle 1980a).

*** Nephelolejeunea hispida R.M.Schust. ex L.Söderstr. et A.Hagborg, Phytotaxa 202 (1): 66, 2015 (Pócs et al. 2015c). Based on: Austrolejeunea hispida R.M.Schust., Phytologia 47 (4): 305, 1981 (Schuster 1981a), *nom. inval*.

*** Nephelolejeunea jarmaniana Grolle ex L.Söderstr. et A.Hagborg, Phytotaxa 202 (1): 66, 2015 (Pócs et al. 2015c). Based on: Austrolejeunea jarmaniana Grolle, Nova Hedwigia 55 (1/2): 112, 1992 (Grolle 1992b), *nom. inval*.

*** Nephelolejeunea nudipes (Hook.f. et Taylor) L.Söderstr. et A.Hagborg, Phytotaxa 202 (1): 66, 2015 (Pócs et al. 2015c). Bas.: Jungermannia nudipes Hook.f. et Taylor, London J. Bot. 3: 568, 1844 (Hooker and Taylor 1844d).

** Nephelolejeunea occidentalis Pócs ex L.Söderstr. et A.Hagborg, Phytotaxa 202 (1): 66, 2015 (Pócs et al. 2015c). Based on: Austrolejeunea occidentalis Pócs, J. Hattori Bot. Lab. 99: 187, 2006 (Pócs 2006d), *nom. inval*.

*** Nephelolejeunea papillosa Glenny, New Zealand J. Bot. 34 (2): 195, 1996 (Glenny 1996).

*** Nephelolejeunea radulifolia (C.Massal.) L.Söderstr. et A.Hagborg, Phytotaxa 202 (1): 66, 2015 (Pócs et al. 2015c). Bas.: Lejeunea radulifolia C.Massal., Nuovo Giorn. Bot. Ital. 17 (3): 248, 1885 (Massalongo 1885).

** Nephelolejeunea secunda M.A.M.Renner ex L.Söderstr. et A.Hagborg, Phytotaxa 202 (1): 66, 2015 (Pócs et al. 2015c). Based on: Austrolejeunea secunda M.A.M.Renner, Bryologist 113 (4): 782, 2010 (Renner 2010), *nom. inval*.

*** Nephelolejeunea talinayi (S.W.Arnell) Grolle, J. Hattori Bot. Lab. 37: 253, 1973 (Grolle 1973b). Bas.: Harpalejeunea talinayi S.W.Arnell, Svensk Bot. Tidskr. 50 (2): 309, 1956 (Arnell 1956d).

** **Schusterolejeunea Grolle**, J. Bryol. 11 (1): 105, 1980 (Grolle 1980b). *Nom. nov. pro Cladocolea* R.M.Schust., Beih. Nova Hedwigia 9: 155, 1963 (Schuster 1963a).

*** Schusterolejeunea inundata (Spruce) Grolle, J. Bryol. 11 (1): 105, 1980 (Grolle 1980b). Bas.: Lejeunea inundata Spruce, Trans. & Proc. Bot. Soc. Edinburgh 15: 278, 1884 (Spruce 1884).

** **Siphonolejeunea Herzog**, Nat. Hist. Juan Fernandez (Botany) 2 (5): 744, 1942 (Herzog 1942a).

** Siphonolejeunea elegantissima (Steph.) Grolle, J. Hattori Bot. Lab. 41: 405, 1976 (Grolle 1976b). Bas.: Trachylejeunea elegantissima Steph., Hedwigia 28 (4): 262, 1889 (Stephani 1889c).

** Siphonolejeunea neesii (Mont.) Bischl., Nova Hedwigia 17: 338, 1969 (Bischler 1969). Bas.: Lejeunea neesii Mont., Ann. Sci. Nat. Bot. (sér. 2) 5: 62, 1836 (Nees and Montagne 1836).

*** Siphonolejeunea schiffneri (Schiffn.) Herzog, Svensk Bot. Tidskr. 42 (3): 230, 1948 (Herzog 1948). Bas.: Pycnolejeunea schiffneri Schiffn., Consp. Hepat. Arch. Ind.: 260, 1898 (Schiffner 1898b).

** **Tuyamaella S.Hatt.**, J. Hattori Bot. Lab. 5: 60, 1951 (Hattori 1951d).

** Tuyamaella angulistipa (Steph.) R.M.Schust. et Kachroo, J. Linn. Soc., Bot. 56 (368): 508, 1961 (Kachroo and Schuster 1961). Bas.: Pycnolejeunea angulistipa Steph., Hedwigia 35 (3): 123, 1896 (Stephani 1896b).

** Tuyamaella borneensis Tixier, Rev. Bryol. Lichénol. 39 (2): 241, 1973 (Tixier 1973a).

** Tuyamaella hattorii Tixier, Rev. Bryol. Lichénol. 31 (3/4): 188, 1962 [1963] (Tixier 1962).

** Tuyamaella jackii (Steph.) Tixier, Rev. Bryol. Lichénol. 39 (2): 235, 1973 (Tixier 1973a). Bas.: Pycnolejeunea jackii Steph., Sp. Hepat. (Stephani) 5: 611, 1914 (Stephani 1914b).

*** Tuyamaella molischii (Schiffn.) S.Hatt., J. Hattori Bot. Lab. 5: 62, 1951 (Hattori 1951d). Bas.: Pycnolejeunea molischii Schiffn., Ann. Bryol. 2: 97, 1929 (Schiffner 1929).

** Tuyamaella molischii var. brevistipa P.C.Wu et P.J.Lin, Acta Phytotax. Sin. 16 (2): 65, 1978 (Wu and Lin 1978).

** Tuyamaella molischii var. taiwanensis R.L.Zhu et M.L.So, Nova Hedwigia 70 (1/2): 190, 2000 (Zhu and So 2000).

** Tuyamaella serratistipa S.Hatt., Bot. Mag. (Tokyo) 64 (755/756): 118, 1951 (Hattori 1951c).

########### ** subtrib. Cyclolejeuneinae Gradst.

*** **Bromeliophila R.M.Schust.**, J. Hattori Bot. Lab. 75: 226, 1994 (Schuster 1994).

*** Bromeliophila helenae Gradst., Cryptog. Bryol. Lichénol. 18 (3): 218, 1997 (Gradstein 1997).

*** Bromeliophila natans (Steph.) R.M.Schust., J. Hattori Bot. Lab. 75: 226, 1994 (Schuster 1994). Bas.: Peltolejeunea natans Steph., Hedwigia 44 (4): 228, 1905 (Stephani 1905a).

*** **Cyclolejeunea A.Evans**, Bull. Torrey Bot. Club 31 (4): 193, 1904 (Evans 1904a).

*** Cyclolejeunea accedens (Gottsche) A.Evans, Bull. Torrey Bot. Club 31 (4): 201, 1904 (Evans 1904a). Bas.: Lejeunea accedens Gottsche, Syn. Hepat. 3: 339, 1845 (Gottsche et al. 1845b).

*** Cyclolejeunea chitonia (Taylor) A.Evans, Bull. Torrey Bot. Club 31 (4): 194, 1904 (Evans 1904a). Bas.: Lejeunea chitonia Taylor, Nov. Stirp. Pug. 8: 27, 1844 (Lehmann 1844).

*** Cyclolejeunea convexistipa (Lehm. et Lindenb.) A.Evans, Bull. Torrey Bot. Club 31 (4): 198, 1904 (Evans 1904a). Bas.: Jungermannia convexistipa Lehm. et Lindenb., Nov. Stirp. Pug. 6: 43, 1834 (Lehmann 1834).

* Cyclolejeunea ecuadorensis Steph., Sp. Hepat. (Stephani) 5: 194, 1913 (Stephani 1913a).

*** Cyclolejeunea foliorum (Nees) Grolle, J. Hattori Bot. Lab. 65: 403, 1988 (Grolle 1988c). Bas.: Lejeunea foliorum Nees, Syn. Hepat. 3: 326, 1845 (Gottsche et al. 1845b).

* Cyclolejeunea integerrima (Steph.) Steph., Sp. Hepat. (Stephani) 5: 195, 1913 (Stephani 1913a). Bas.: Odontolejeunea integerrima Steph., Hedwigia 44 (4): 227, 1905 (Stephani 1905a).

*** Cyclolejeunea luteola (Spruce) Grolle, Wiss. Z. Friedrich-Schiller-Univ. Jena, Math.-Naturwiss. Reihe 33: 761, 1984 (Grolle 1984b). Bas.: Lejeunea luteola Spruce, Trans. & Proc. Bot. Soc. Edinburgh 15: 205, 1884 (Spruce 1884).

*** Cyclolejeunea peruviana (Lehm. et Lindenb.) A.Evans, Bull. Torrey Bot. Club 31 (4): 196, 1904 (Evans 1904a). Bas.: Jungermannia peruviana Lehm. et Lindenb., Nov. Stirp. Pug. 5: 18, 1833 (Lehmann 1833).

* Cyclolejeunea spectabilis Steph., Sp. Hepat. (Stephani) 5: 193, 1913 (Stephani 1913a).

*** **Prionolejeunea (Spruce) Schiffn.**, Hepat. (Engl.-Prantl): 127, 1893 (Schiffner 1893b). Bas.: Lejeunea subg. Prionolejeunea Spruce, Trans. & Proc. Bot. Soc. Edinburgh 15: 152, 1884 (Spruce 1884).

*** Prionolejeunea aemula (Gottsche) A.Evans, Bull. Torrey Bot. Club 31 (4): 219, 1904 (Evans 1904a). Bas.: Lejeunea aemula Gottsche, Syn. Hepat. 3: 338, 1845 (Gottsche et al. 1845b).

*** Prionolejeunea ampliretis Herzog, Feddes Repert. Spec. Nov. Regni Veg. 57 (1/2): 178, 1955 (Herzog 1955).

*** Prionolejeunea arguta (Nees) Steph., Sp. Hepat. (Stephani) 5: 204, 1913 (Stephani 1913a). Bas.: Lejeunea arguta Nees, Syn. Hepat. 3: 338, 1845 (Gottsche et al. 1845b).

*** Prionolejeunea decora (Taylor) Steph., Sp. Hepat. (Stephani) 5: 207, 1913 (Stephani 1913a). Bas.: Lejeunea decora Taylor, London J. Bot. 5: 393, 1846 (Taylor 1846b).

*** Prionolejeunea denticulata (F.Weber) Schiffn., Hepat. (Engl.-Prantl): 127, 1893 (Schiffner 1893b). Bas.: Jungermannia denticulata F.Weber, Hist. Musc. Hepat. Prodr.: 30, 1815 (Weber 1815).

*** Prionolejeunea diversitexta (Hampe et Gottsche) Steph., Sp. Hepat. (Stephani) 5: 209, 1913 (Stephani 1913a). Bas.: Lejeunea diversitexta Hampe et Gottsche, Linnaea 25 (3): 357, 1852 [1853] (Hampe and Gottsche 1852).

*** Prionolejeunea exauriculata A.Evans, Bull. Torrey Bot. Club 31 (4): 223, 1904 (Evans 1904a).

*** Prionolejeunea galliotii Steph., Sp. Hepat. (Stephani) 6: 388, 1923 (Stephani 1923).

*** Prionolejeunea grata (Gottsche) Schiffn., Hepat. (Engl.-Prantl): 127, 1893 (Schiffner 1893b). Bas.: Lejeunea grata Gottsche, Abh. Naturwiss. Vereins Bremen 7: 359, 1882 (Gottsche 1882).

*** Prionolejeunea grollei Ilk.-Borg. et Schäf.-Verw., Cryptog. Bryol. 26 (1): 29, 2005 (Ilkiu-Borges and Schäfer-Verwimp 2005).

*** Prionolejeunea guadalupensis (Lindenb.) Steph., Sp. Hepat. (Stephani) 5: 211, 1913 (Stephani 1913a). Bas.: Lejeunea guadalupensis Lindenb., Syn. Hepat. 3: 340, 1845 (Gottsche et al. 1845b).

*** Prionolejeunea limpida Herzog, Hedwigia 67 (6): 252, 1927 (Herzog 1927).

*** Prionolejeunea magnistipula Herzog, Feddes Repert. Spec. Nov. Regni Veg. 57 (1/2): 176, 1955 (Herzog 1955).

*** Prionolejeunea meissneri (Gottsche) Steph., Sp. Hepat. (Stephani) 5: 212, 1913 (Stephani 1913a). Bas.: Lejeunea meissneri Gottsche, Syn. Hepat. 3: 340, 1845 (Gottsche et al. 1845b).

*** Prionolejeunea mucronata (Sande Lac.) Steph., Hedwigia 35 (3): 119, 1896 (Stephani 1896b). Bas.: Lejeunea mucronata Sande Lac., Syn. hepat. jav.: 106, 1856 [1857] (Sande Lacoste 1856b).

*** Prionolejeunea muricatoserrulata (Spruce) Steph., Sp. Hepat. (Stephani) 5: 223, 1913 (Stephani 1913a). Bas.: Lejeunea muricatoserrulata Spruce, Trans. & Proc. Bot. Soc. Edinburgh 15: 155, 1884 (Spruce 1884).

*** Prionolejeunea principensis Vanden Berghen, Rev. Bryol. Lichénol. 29 (1/2): 65, 1960 (Vanden Berghen 1960a).

*** Prionolejeunea recurvula (Spruce) Steph., Sp. Hepat. (Stephani) 5: 224, 1913 (Stephani 1913a). Bas.: Lejeunea recurvula Spruce, Trans. & Proc. Bot. Soc. Edinburgh 15: 155, 1884 (Spruce 1884).

*** Prionolejeunea scaberula (Spruce) Steph., Sp. Hepat. (Stephani) 5: 214, 1913 (Stephani 1913a). Bas.: Lejeunea scaberula Spruce, Trans. & Proc. Bot. Soc. Edinburgh 15: 159, 1884 (Spruce 1884).

*** Prionolejeunea schlimiana (Gottsche) Steph., Sp. Hepat. (Stephani) 5: 214, 1913 (Stephani 1913a). Bas.: Lejeunea schlimiana Gottsche, Ann. Sci. Nat. Bot. (sér. 5) 1: 154, 1864 (Gottsche 1864).

*** Prionolejeunea trachyodes (Spruce) Steph., Sp. Hepat. (Stephani) 5: 215, 1913 (Stephani 1913a). Bas.: Lejeunea trachyodes Spruce, J. Linn. Soc., Bot. 30 (210): 338, 1895 (Gepp 1895b).

**Excluded from the genus**

* Prionolejeunea corbisieri Pearson, Natuurw. Tijdschr. 4 (5/6): 128, 1922 (Pearson 1922a). ^297^

* Prionolejeunea maculata Herzog, Feddes Repert. Spec. Nov. Regni Veg. 57 (1/2): 180, 1955 (Herzog 1955). ^298^

########### ** subtrib. Drepanolejeuneinae Gradst.

** **Capillolejeunea S.W.Arnell**, Svensk Bot. Tidskr. 59 (1): 69, 1965 (Arnell 1965).

** Capillolejeunea geisslerae (Pócs) R.L.Zhu, Qiong He, Y.M.Wei et Pócs, Phytotaxa 175 (3): 167, 2014 (He et al. 2014a). Bas.: Drepanolejeunea geisslerae Pócs, Candollea 56 (1): 70, 2001 (Pócs 2001).

** Capillolejeunea mascarena S.W.Arnell, Svensk Bot. Tidskr. 59 (1): 69, 1965 (Arnell 1965).

*** **Drepanolejeunea (Spruce) Steph.**, Hedwigia 30 (5): 209, 1891 (Stephani 1891a). Bas.: Lejeunea subg. Drepanolejeunea Spruce, Trans. & Proc. Bot. Soc. Edinburgh 15: 186, 1884 (Spruce 1884).

** **subg. Acantholejeunea R.M.Schust.**, Beih. Nova Hedwigia 9: 115, 1963 (Schuster 1963a).

** Drepanolejeunea dentistipula Steph., Sp. Hepat. (Stephani) 5: 343, 1913 (Stephani 1913a).

** Drepanolejeunea spinistipula Herzog, Svensk Bot. Tidskr. 42 (3): 238, 1948 (Herzog 1948).

*** **subg. Drepanolejeunea**

*** Drepanolejeunea aculeata Bischl., Rev. Bryol. Lichénol. 33 (1/2): 68, 1964 (Bischler 1964).

** Drepanolejeunea anderssonii (Ångstr.) A.Evans, Trans. Connecticut Acad. Arts 10 (8): 429, 1900 (Evans 1900a). Bas.: Lejeunea anderssonii Ångstr., Öfvers. Kongl. Vetensk.-Akad. Förh. 29 (4): 24, 1872 (Ångström 1872).

*** Drepanolejeunea andina Herzog, Svensk Bot. Tidskr. 51 (1): 196, 1957 (Herzog 1957a).

*** Drepanolejeunea angustifolia (Mitt.) Grolle, J. Jap. Bot. 40 (7): 206, 1965 (Grolle 1965d). Bas.: Lejeunea angustifolia Mitt., J. Proc. Linn. Soc., Bot. 5 (18): 116, 1860 [1861] (Mitten 1860c).

*** Drepanolejeunea ankasica E.W.Jones, Bull. Brit. Mus. (Nat. Hist.), Bot. 11 (3): 245, 1983 (Jones and Harrington 1983).

*** Drepanolejeunea anoplantha (Spruce) Steph., Sp. Hepat. (Stephani) 5: 325, 1913 (Stephani 1913a). Bas.: Lejeunea anoplantha Spruce, Trans. & Proc. Bot. Soc. Edinburgh 15: 189, 1884 (Spruce 1884).

*** Drepanolejeunea appalachiana R.M.Schust., J. Elisha Mitchell Sci. Soc. 83 (4): 219, 1967 (Schuster 1967a).

*** Drepanolejeunea araucariae Steph., Hedwigia 35 (3): 80, 1896 (Stephani 1896b).

** Drepanolejeunea araucariae var. chilensis Herzog, Acta Horti Gotob. 15: 161, 1943 (Herzog 1943a).

** Drepanolejeunea aucklandica Steph., Sp. Hepat. (Stephani) 5: 358, 1913 (Stephani 1913a).

*** Drepanolejeunea aurita Bischl., Rev. Bryol. Lichénol. 33 (1/2): 146, 1964 (Bischler 1964).

** Drepanolejeunea bakeri Herzog, Ann. Bryol. 3: 148, 1930 (Herzog 1930b).

*** Drepanolejeunea bidens (Prantl) A.Evans, Bull. Torrey Bot. Club 30 (1): 29, 1903 (Evans 1903b). Bas.: Lejeunea bidens Prantl, Hedwigia 29: xiv, 1890 (Prantl 1890).

*** Drepanolejeunea biocellata A.Evans, Bull. Torrey Bot. Club 30 (1): 22, 1903 (Evans 1903b).

** Drepanolejeunea blumei Steph., Hedwigia 35 (3): 81, 1896 (Stephani 1896b).

** Drepanolejeunea brunnea Mizut., J. Hattori Bot. Lab. 33: 231, 1970 (Mizutani 1970).

** Drepanolejeunea caledonica Steph., Sp. Hepat. (Stephani) 5: 342, 1913 (Stephani 1913a).

*** Drepanolejeunea campanulata (Spruce) Steph., Sp. Hepat. (Stephani) 5: 328, 1913 (Stephani 1913a). Bas.: Lejeunea campanulata Spruce, Trans. & Proc. Bot. Soc. Edinburgh 15: 192, 1884 (Spruce 1884).

** Drepanolejeunea canceroides H.A.Mill. et Bonner, Beih. Nova Hedwigia 11: 53, 1963 (Miller et al. 1963).

** Drepanolejeunea capulata (Taylor) J.B.Jack et Steph., Hedwigia 31 (1): 13, 1892 (Jack and Stephani 1892). Bas.: Lejeunea capulata Taylor, London J. Bot. 5: 394, 1846 (Taylor 1846b).

** Drepanolejeunea capulata var. flagellifera S.W.Arnell, Österr. Akad. Wiss., Math.-Naturwiss. Kl., Denkschr. 111: 100, 1964 (Schiffner and Arnell 1964).

** Drepanolejeunea ciliata Mizut., J. Hattori Bot. Lab. 33: 232, 1970 (Mizutani 1970).

*** Drepanolejeunea crassiretis A.Evans, Bull. Torrey Bot. Club 30 (1): 25, 1903 (Evans 1903b).

*** Drepanolejeunea crucianella (Taylor) A.Evans, Bull. Torrey Bot. Club 30 (1): 33, 1903 (Evans 1903b). Bas.: Lejeunea crucianella Taylor, London J. Bot. 5: 393, 1846 (Taylor 1846b).

*** Drepanolejeunea cultrella (Mitt.) Steph., Sp. Hepat. (Stephani) 5: 324, 1913 (Stephani 1913a). Bas.: Lejeunea cultrella Mitt., J. Proc. Linn. Soc., Bot. 7 (27): 168, 1863 (Mitten 1863).

** Drepanolejeunea cutervoensis (Loitl.) Grolle, J. Hattori Bot. Lab. 69: 186, 1991 (Grolle 1991). Bas.: Lejeunea cutervoensis Loitl., Diagn. pl. nov.: 21, 1894 (Loitlesberger 1894).

** Drepanolejeunea decurviloba Steph., Sp. Hepat. (Stephani) 6: 397, 1923 (Stephani 1923).

* Drepanolejeunea deslooveri Vanden Berghen, Bull. Jard. Bot. Natl. Belg. 47 (1/2): 210, 1977 (Vanden Berghen 1977). ^299^

* Drepanolejeunea dissitifolia A.Evans, Bull. Torrey Bot. Club 30 (1): 28, 1903 (Evans 1903b).

** Drepanolejeunea elegans Herzog, Ann. Bryol. 9: 128, 1936 [1937] (Herzog 1936a).

** Drepanolejeunea erecta (Steph.) Mizut., J. Hattori Bot. Lab. 40: 442, 1976 (Mizutani 1976b). Bas.: Leptolejeunea erecta Steph., Bull. Soc. Roy. Bot. Belgique 38 (1): 44, 1899 (Stephani 1899h).

** Drepanolejeunea evansii Bischl. ex L.Söderstr., A.Hagborg et von Konrat, Phytotaxa 65: 47, 2012 (Söderström et al. 2012c). Based on: Drepanolejeunea evansii Bischl., Rev. Bryol. Lichénol. 33 (1/2): 75, 1964 (Bischler 1964), *nom. inval*.

*** Drepanolejeunea fragilis Bischl. ex L.Söderstr., A.Hagborg et von Konrat, Phytotaxa 65: 47, 2012 (Söderström et al. 2012c). Based on: Drepanolejeunea fragilis Bischl., Rev. Bryol. Lichénol. 33 (1/2): 123, 1964 (Bischler 1964), *nom. inval*.

** Drepanolejeunea fulfordiae L.Söderstr., Phytotaxa 208 (1): 98, 2015 (Pócs et al. 2015a). *Nom. nov. pro Drepanolejeunea papillosa* Fulford, Mem. New York Bot. Gard. 23: 843, 1972 (Fulford 1972), *nom. illeg*.

*** Drepanolejeunea granatensis (Prantl) Bischl., Rev. Bryol. Lichénol. 33 (1/2): 150, 1964 (Bischler 1964). Bas.: Lejeunea granatensis Prantl, Hedwigia 31: xvi, 1892 (Prantl 1892).

** Drepanolejeunea grandis Herzog, Ann. Bryol. 12: 113, 1939 (Herzog 1939a).

*** Drepanolejeunea grollei M.E.Reiner et Schäf.-Verw., Candollea 51 (2): 475, 1996 (Reiner-Drehwald and Schäfer-Verwimp 1996).

** Drepanolejeunea grossidens Steph., Sp. Hepat. (Stephani) 5: 358, 1913 (Stephani 1913a). Based on: Drepanolejeunea grossidens Steph., Hedwigia 28 (3): 168, 1889 (Stephani 1889d), *nom. inval*.

*** Drepanolejeunea hamatifolia (Hook.) Schiffn., Hepat. (Engl.-Prantl): 126, 1893 (Schiffner 1893b). Bas.: Jungermannia hamatifolia Hook., Brit. Jungermann.: tab. 51, 1813 (Hooker 1813).

** Drepanolejeunea hamulata Steph., Sp. Hepat. (Stephani) 5: 331, 1913 (Stephani 1913a).

*** Drepanolejeunea helenae Pócs, Cryptog. Bryol. Lichénol. 18 (3): 198, 1997 (Pócs 1997a).

** Drepanolejeunea herzogii R.L.Zhu et M.L.So, Beih. Nova Hedwigia 121: 181, 2001 (Zhu and So 2001). *Nom. nov. pro Strepsilejeunea ocellata* Herzog, Memoranda Soc. Fauna Fl. Fennica 26: 57, 1950 [1951] (Herzog 1950b).

*** Drepanolejeunea inchoata (C.F.W.Meissn.) Steph., Primit. fl. costar.: 115, 1892 [1893] (Stephani 1892e). Bas.: Jungermannia inchoata C.F.W.Meissn., Nov. Stirp. Pug. 5: 19, 1833 (Lehmann 1833).

** Drepanolejeunea inchoata var. palmicola Pócs, Acta Bot. Hung. 51 (3/4): 381, 2009 (Schäfer-Verwimp and Pócs 2009).

** Drepanolejeunea inchoata var. roraimae (Steph. ex Zwickel) Bischl., Rev. Bryol. Lichénol. 33 (1/2): 48, 1964 (Bischler 1964). Bas.: Drepanolejeunea roraimae Steph. ex Zwickel, Ann. Bryol. 6: 121, 1933 (Zwickel 1933).

*** Drepanolejeunea infundibulata (Spruce) A.Evans, Bull. Torrey Bot. Club 30 (1): 35, 1903 (Evans 1903b). Bas.: Lejeunea infundibulata Spruce, Trans. & Proc. Bot. Soc. Edinburgh 15: 191, 1884 (Spruce 1884).

*** Drepanolejeunea integribracteata Bischl., Rev. Bryol. Lichénol. 33 (1/2): 142, 1964 (Bischler 1964).

** Drepanolejeunea laevis (Mitt.) Steph., Sp. Hepat. (Stephani) 5: 357, 1913 (Stephani 1913a). Bas.: Lejeunea laevis Mitt., Fl. vit.: 416, 1871 [1873] (Mitten 1871).

* Drepanolejeunea lancifolia (Gottsche) J.B.Jack et Steph., Hedwigia 31 (1): 13, 1892 (Jack and Stephani 1892). Bas.: Lejeunea lancifolia Gottsche, Ann. Sci. Nat. Bot. (sér. 5) 1: 155, 1864 (Gottsche 1864). ^300^

*** Drepanolejeunea lichenicola (Spruce) Steph., Sp. Hepat. (Stephani) 5: 335, 1913 (Stephani 1913a). Bas.: Lejeunea lichenicola Spruce, Trans. & Proc. Bot. Soc. Edinburgh 15: 191, 1884 (Spruce 1884).

** Drepanolejeunea longii Grolle et R.L.Zhu, Ann. Bot. Fenn. 36 (2): 115, 1999 (Grolle and Zhu 1999).

** Drepanolejeunea macrodonta (Mitt.) Steph., Sp. Hepat. (Stephani) 5: 357, 1913 (Stephani 1913a). Bas.: Lejeunea macrodonta Mitt., J. Proc. Linn. Soc., Bot. 5 (18): 116, 1860 [1861] (Mitten 1860c).

** Drepanolejeunea mawtmiana Ajit P.Singh et V.Nath, Hepat. Khasi Jaintia Hills: E. Himal.: 251, 2007 (Singh and Nath 2007b).

** Drepanolejeunea microcarpa Pearson, J. Linn. Soc., Bot. 46 (305): 36, 1922 (Pearson 1922b).

** Drepanolejeunea moluccensis Herzog, Ann. Bryol. 7: 88, 1934 (Herzog 1934b).

*** Drepanolejeunea mosenii (Steph.) Bischl., Rev. Bryol. Lichénol. 35 (1/4): 118, 1967 [1968] (Bischler 1967). Bas.: Leptolejeunea mosenii Steph., Sp. Hepat. (Stephani) 5: 372, 1913 (Stephani 1913a).

* Drepanolejeunea obliqua Steph., Hedwigia 35 (3): 82, 1896 (Stephani 1896b). ^301^

** Drepanolejeunea obtriangulata T.Kodama, J. Hattori Bot. Lab. 41: 381, 1976 (Kodama 1976).

** Drepanolejeunea obtusifolia T.Yamag., J. Jap. Bot. 59 (11): 332, 1984 (Yamaguchi 1984).

*** Drepanolejeunea orthophylla (Nees et Mont.) Bischl., Rev. Bryol. Lichénol. 35 (1/4): 102, 1967 [1968] (Bischler 1967). Bas.: Lejeunea orthophylla Nees et Mont., Ann. Sci. Nat. Bot. (sér. 2) 19: 265, 1843 (Montagne 1843).

*** Drepanolejeunea palmifolia (Nees) Schiffn., Hepat. (Engl.-Prantl): 126, 1893 (Schiffner 1893b). Bas.: Jungermannia palmifolia Nees, Fl. Bras. (Martius) 1 (1): 366, 1833 (Nees 1833a).

*** Drepanolejeunea pentadactyla (Mont.) Steph., Sp. Hepat. (Stephani) 5: 357, 1913 (Stephani 1913a). Bas.: Lejeunea pentadactyla Mont., Ann. Sci. Nat. Bot. (sér. 3) 10: 113, 1848 (Montagne 1848).

** Drepanolejeunea perissodonta (Spruce) Bischl., Rev. Bryol. Lichénol. 33 (1/2): 73, 1964 (Bischler 1964). Bas.: Lejeunea inchoata var. perissodonta Spruce, J. Linn. Soc., Bot. 30 (210): 340, 1895 (Gepp 1895b).

*** Drepanolejeunea physifolia (Gottsche) Pearson, Forh. Vidensk.-Selsk. Kristiania 1892 (8): 8, 1892 (Pearson 1892). Bas.: Lejeunea physifolia Gottsche, Abh. Naturwiss. Vereins Bremen 7: 357, 1882 (Gottsche 1882).

** Drepanolejeunea pinnatiloba Schiffn., Bot. Jahrb. Syst. 23 (5): 591, 1897 (Schiffner 1897).

** Drepanolejeunea pleiodictya Herzog, Ann. Bryol. 7: 89, 1934 (Herzog 1934b).

** Drepanolejeunea propagulifera Herzog, Mitt. Inst. Allg. Bot. Hamburg 7 (3): 204, 1931 (Herzog 1931a).

** Drepanolejeunea pseudoneura (A.Evans) Grolle, J. Hattori Bot. Lab. 65: 405, 1988 (Grolle 1988c). Bas.: Harpalejeunea pseudoneura A.Evans, Trans. Connecticut Acad. Arts 10 (8): 427, 1900 (Evans 1900a).

** Drepanolejeunea pterocalyx (Herzog) Bischl., Rev. Bryol. Lichénol. 35 (1/4): 114, 1967 [1968] (Bischler 1967). Bas.: Leptolejeunea pterocalyx Herzog, Feddes Repert. Spec. Nov. Regni Veg. 57 (1/2): 187, 1955 (Herzog 1955).

** Drepanolejeunea pungens Bischl., Rev. Bryol. Lichénol. 33 (1/2): 104, 1964 (Bischler 1964).

** Drepanolejeunea ramentiflora Steph., Sp. Hepat. (Stephani) 5: 338, 1913 (Stephani 1913a).

* Drepanolejeunea ruandensis Vanden Berghen, Bull. Soc. Roy. Bot. Belgique 93: 63, 1961 (Vanden Berghen 1961). ^302^

*** Drepanolejeunea senticosa Bischl., Rev. Bryol. Lichénol. 33 (1/2): 96, 1964 (Bischler 1964).

** Drepanolejeunea sikkimensis (Udar et U.S.Awasthi) Grolle, J. Hattori Bot. Lab. 55: 503, 1984 (Grolle 1984a). Bas.: Leptolejeunea sikkimensis Udar et U.S.Awasthi, Misc. Bryol. Lichenol. 8 (6): 115, 1979 (Udar and Awasthi 1979).

** Drepanolejeunea spinosa Herzog, Feddes Repert. Spec. Nov. Regni Veg. 57 (1/2): 185, 1955 (Herzog 1955).

** Drepanolejeunea spinosocornuta Steph., Sp. Hepat. (Stephani) 5: 351, 1913 (Stephani 1913a).

* Drepanolejeunea subdissitifolia Herzog, Memoranda Soc. Fauna Fl. Fennica 25: 65, 1950 (Herzog 1950c).

** Drepanolejeunea submuricata R.M.Schust., Phytotaxa 208 (1): 98, 2015 (Pócs et al. 2015a). Based on: Drepanolejeunea submuricata R.M.Schust., Nova Hedwigia 62 (1/2): 34, 1996 (Schuster 1996c), *nom. inval*.

** Drepanolejeunea subquadrata (Mitt.) Steph., Bot. Jahrb. Syst. 23 (1/2, 3): 311, 1896 (Stephani 1896a). Bas.: Lejeunea subquadrata Mitt., Fl. vit.: 416, 1871 [1873] (Mitten 1871).

** Drepanolejeunea subvittata (Herzog) Grolle, J. Hattori Bot. Lab. 69: 186, 1991 (Grolle 1991). Bas.: Harpalejeunea subvittata Herzog, Svensk Bot. Tidskr. 51 (1): 195, 1957 (Herzog 1957a).

* Drepanolejeunea tenax K.I.Goebel, Ann. Jard. Bot. Buitenzorg 39: 15, 1928 (Goebel 1928).

** Drepanolejeunea tenera K.I.Goebel, Ann. Jard. Bot. Buitenzorg 39: 20, 1928 (Goebel 1928). ^303^

* Drepanolejeunea tenera var. litoceras Herzog, Ann. Bryol. 7: 85, 1934 (Herzog 1934b).

*** Drepanolejeunea ternatensis (Gottsche) Schiffn., Hepat. (Engl.-Prantl): 126, 1893 (Schiffner 1893b). Bas.: Lejeunea ternatensis Gottsche, Syn. Hepat. 3: 346, 1845 (Gottsche et al. 1845b).

** Drepanolejeunea teysmannii (Gottsche) Steph., Hedwigia 35 (3): 84, 1896 (Stephani 1896b). Bas.: Lejeunea teysmannii Gottsche, Abh. Naturwiss. Vereins Bremen 7: 360, 1882 (Gottsche 1882).

** Drepanolejeunea tridactyla (Gottsche) Steph., Sp. Hepat. (Stephani) 5: 354, 1913 (Stephani 1913a). Bas.: Lejeunea tridactyla Gottsche, Syn. Hepat. 3: 347, 1845 (Gottsche et al. 1845b).

*** Drepanolejeunea trigonophylla Steph., Hedwigia 35 (3): 85, 1896 (Stephani 1896b).

** Drepanolejeunea tristaniana S.W.Arnell, Results Norweg. Sci. Exped. Tristan da Cunha 42: 7, 1958 (Arnell 1958b).

** Drepanolejeunea tuyamae S.Hatt., Bot. Mag. (Tokyo) 64 (755/756): 116, 1951 (Hattori 1951c).

** Drepanolejeunea ualanensis Inoue et H.A.Mill., Bull. Natl. Sci. Mus. Tokyo (n.ser.) 8 (2): 148, 1965 (Inoue and Miller 1965).

** Drepanolejeunea ungulata (Steph.) Grolle, J. Hattori Bot. Lab. 69: 187, 1991 (Grolle 1991). Bas.: Harpalejeunea ungulata Steph., Sp. Hepat. (Stephani) 5: 264, 1913 (Stephani 1913a).

** Drepanolejeunea urceolata R.M.Schust., Phytologia 39 (6): 427, 1978 (Schuster 1978b).

** Drepanolejeunea valiae Jovet-Ast, Rev. Bryol. Lichénol. 18 (1/2): 38, 1949 (Jovet-Ast 1949a).

** Drepanolejeunea vandenberghenii Buchb. et Eb.Fisch., J. Bryol. 26 (4): 273, 2004 (Buchbender and Fischer 2004).

*** Drepanolejeunea vesiculosa (Mitt.) Steph., Sp. Hepat. (Stephani) 5: 356, 1913 (Stephani 1913a). Bas.: Lejeunea vesiculosa Mitt., J. Proc. Linn. Soc., Bot. 5 (18): 116, 1860 [1861] (Mitten 1860c).

** Drepanolejeunea yulensis Steph., Sp. Hepat. (Stephani) 5: 356, 1913 (Stephani 1913a).

*** **subg. Kolpolejeunea Grolle**, J. Hattori Bot. Lab. 40: 193, 1976 (Grolle 1976c).

*** Drepanolejeunea intermedia Zwickel, Ann. Bryol. 6: 119, 1933 (Zwickel 1933).

*** Drepanolejeunea lyrata Grolle, J. Hattori Bot. Lab. 40: 199, 1976 (Grolle 1976c).

*** Drepanolejeunea madagascariensis (Steph.) Grolle, Lindbergia 2 (3/4): 232, 1974 (Grolle and Onraedt 1974). Bas.: Leptolejeunea madagascariensis Steph., Sp. Hepat. (Stephani) 5: 363, 1913 (Stephani 1913a).

*** Drepanolejeunea pocsii Grolle, J. Hattori Bot. Lab. 40: 209, 1976 (Grolle 1976c).

** Drepanolejeunea symoensii Vanden Berghen et Grolle, J. Hattori Bot. Lab. 49: 86, 1981 (Grolle 1981). Based on: Leptolejeunea symoensii Vanden Berghen, Bull. Soc. Roy. Bot. Belgique 93: 58, 1961 (Vanden Berghen 1961), *nom. inval*.

** Drepanolejeunea symoensii var. minor Tixier, Trop. Bryol. 11: 25, 1995 (Tixier 1995b).

*** Drepanolejeunea trematodes (Nees) Bischl., Rev. Bryol. Lichénol. 35 (1/4): 125, 1967 [1968] (Bischler 1967). Bas.: Lejeunea trematodes Nees, Ann. Sci. Nat. Bot. (sér. 2) 5: 63, 1836 (Nees and Montagne 1836).

** **subg. Pristolejeunea Grolle**, J. Hattori Bot. Lab. 40: 193, 1976 (Grolle 1976c).

** Drepanolejeunea actinogyna Inuthai, R.L.Zhu et Chantanaorr., Bryologist 117 (2): 165, 2014 (Inuthai et al. 2014).

*** Drepanolejeunea fissicornua Steph., Sp. Hepat. (Stephani) 5: 344, 1913 (Stephani 1913a).

* Drepanolejeunea hampeana Steph., Sp. Hepat. (Stephani) 5: 345, 1913 (Stephani 1913a). *Nom. nov. pro Drepanolejeunea hampeana* Steph., Hedwigia 29 (2): 70, 1890 (Stephani 1890b), *nom. inval*.

** Drepanolejeunea laciniata Qiong He et R.L.Zhu, Cryptog. Bryol. 33 (3): 292, 2012 (He et al. 2012a).

*** Drepanolejeunea levicornua Steph., Sp. Hepat. (Stephani) 5: 347, 1913 (Stephani 1913a).

* Drepanolejeunea longicornua (Herzog) Mizut., J. Hattori Bot. Lab. 68: 368, 1990 (Mizutani 1990). Bas.: Drepanolejeunea levicornua var. longicornua Herzog, Ann. Bryol. 3: 142, 1930 (Herzog 1930b).

* Drepanolejeunea nymanii Steph., Sp. Hepat. (Stephani) 5: 348, 1913 (Stephani 1913a).

** Drepanolejeunea pulla (Mitt.) Grolle, J. Hattori Bot. Lab. 46: 349, 1979 (Grolle 1979d). Bas.: Lejeunea pulla Mitt., J. Proc. Linn. Soc., Bot. 5 (18): 116, 1860 [1861] (Mitten 1860c).

* Drepanolejeunea serricalyx Herzog, Ann. Bryol. 9: 126, 1936 [1937] (Herzog 1936a).

*** Drepanolejeunea thwaitesiana (Mitt.) Steph., Sp. Hepat. (Stephani) 5: 350, 1913 (Stephani 1913a). Bas.: Lejeunea thwaitesiana Mitt., J. Proc. Linn. Soc., Bot. 5 (18): 117, 1860 [1861] (Mitten 1860c). ^304^

** Drepanolejeunea thwaitesiana var. zhengii R.L.Zhu, Beih. Nova Hedwigia 121: 197, 2001 (Zhu and So 2001).

*** Drepanolejeunea tricornua Herzog, Ann. Bryol. 9: 124, 1936 [1937] (Herzog 1936a).

*** **subg. Rhaphidolejeunea (Herzog) Grolle et R.L.Zhu**, Nova Hedwigia 70 (3/4): 376, 2000 (Grolle and Zhu 2000). Bas.: Rhaphidolejeunea Herzog, Mitth. Thüring. Bot. Vereins 50: 104, 1943 (Herzog 1943c).

** Drepanolejeunea bidoupensis Pócs, Cryptog. Bryol. 34 (3): 293, 2013 (Pócs et al. 2013).

*** Drepanolejeunea bischlerae (Grolle) Grolle et R.L.Zhu, Nova Hedwigia 70 (3/4): 391, 2000 (Grolle and Zhu 2000). Bas.: Rhaphidolejeunea bischlerae Grolle, J. Hattori Bot. Lab. 38: 653, 1974 (Grolle 1974b).

*** Drepanolejeunea commutata Grolle et R.L.Zhu, Nova Hedwigia 70 (3/4): 377, 2000 (Grolle and Zhu 2000).

*** Drepanolejeunea cyclops (Sande Lac.) Grolle et R.L.Zhu, Nova Hedwigia 70 (3/4): 391, 2000 (Grolle and Zhu 2000). Bas.: Lejeunea cyclops Sande Lac., Ann. Mus. Bot. Lugduno-Batavi 1: 310, 1864 (Sande Lacoste 1864).

*** Drepanolejeunea fleischeri (Steph.) Grolle et R.L.Zhu, Nova Hedwigia 70 (3/4): 379, 2000 (Grolle and Zhu 2000). Bas.: Leptolejeunea fleischeri Steph., Sp. Hepat. (Stephani) 5: 382, 1913 (Stephani 1913a).

** Drepanolejeunea foliicola Horik., J. Sci. Hiroshima Univ., Ser. B, Div. 2, Bot. 1: 85, 1932 (Horikawa 1932a).

** Drepanolejeunea longicruris (Steph.) Grolle et R.L.Zhu, Nova Hedwigia 70 (3/4): 392, 2000 (Grolle and Zhu 2000). Bas.: Leptolejeunea longicruris Steph., Hedwigia 35 (3): 106, 1896 (Stephani 1896b).

*** Drepanolejeunea polyrhiza (Nees) Grolle et R.L.Zhu, Nova Hedwigia 70 (3/4): 392, 2000 (Grolle and Zhu 2000). Bas.: Lejeunea polyrhiza Nees, Syn. Hepat. 3: 403, 1845 (Gottsche et al. 1845b).

*** Drepanolejeunea siamensis (Bischl.) Grolle et R.L.Zhu, Nova Hedwigia 70 (3/4): 393, 2000 (Grolle and Zhu 2000). Bas.: Rhaphidolejeunea siamensis Bischl., Rev. Bryol. Lichénol. 36 (1/2): 86, 1968 [1969] (Bischler 1968).

*** Drepanolejeunea spicata (Steph.) Grolle et R.L.Zhu, Nova Hedwigia 70 (3/4): 384, 2000 (Grolle and Zhu 2000). Bas.: Leptolejeunea spicata Steph., Hedwigia 35 (3): 108, 1896 (Stephani 1896b).

*** Drepanolejeunea tibetana (P.C.Wu et J.S.Lou) Grolle et R.L.Zhu, Nova Hedwigia 70 (3/4): 386, 2000 (Grolle and Zhu 2000). Bas.: Rhaphidolejeunea tibetana P.C.Wu et J.S.Lou, Acta Phytotax. Sin. 16 (4): 102, 1978 (Wu and Lou 1978).

*** Drepanolejeunea yunnanensis (P.C.Chen) Grolle et R.L.Zhu, Nova Hedwigia 70 (3/4): 388, 2000 (Grolle and Zhu 2000). Bas.: Rhaphidolejeunea yunnanensis P.C.Chen, Feddes Repert. Spec. Nov. Regni Veg. 58: 44, 1955 (Chen 1955).

***Incertae sedis***

*** Drepanolejeunea dactylophora (Nees, Lindenb. et Gottsche) J.B.Jack et Steph., Hedwigia 31 (1): 12, 1892 (Jack and Stephani 1892). Bas.: Lejeunea dactylophora Nees, Lindenb. et Gottsche, Observ. bot.: 473, 1843 (Gottsche et al. 1843).

*** Drepanolejeunea dactylophora var. submuricata Herzog, Ann. Bryol. 4: 92, 1931 (Herzog 1931b).

* Drepanolejeunea devendrae Sushil K.Singh et M.Dey, Nelumbo 54: 20, 2012 (Singh and Dey 2012).

* Drepanolejeunea integerrima Herzog, Rev. Bryol. Lichénol. 20 (1/2): 146, 1951 [1952] (Herzog 1951a). ^305^

** **Vitalianthus R.M.Schust. et Giancotti**, Nova Hedwigia 57 (3/4): 447, 1993 (Schuster and Giancotti 1993).

*** Vitalianthus bischlerianus (K.C.Pôrto et Grolle) R.M.Schust. et Giancotti, Nova Hedwigia 57 (3/4): 448, 1993 (Schuster and Giancotti 1993). Bas.: Drepanolejeunea bischleriana K.C.Pôrto et Grolle, Cryptog. Bryol. Lichénol. 8 (4): 301, 1987 (Pôrto and Grolle 1987).

** Vitalianthus guangxianus R.L.Zhu, Qiong He et Y.M.Wei, J. Bryol. 34 (1): 32, 2012 (He et al. 2012b).

########### ** subtrib. Echinolejeuneinae Gradst.

*** **Anoplolejeunea (Spruce) Schiffn.**, Hepat. (Engl.-Prantl): 131, 1893 (Schiffner 1893b). Bas.: Lejeunea subg. Anoplolejeunea Spruce, Trans. & Proc. Bot. Soc. Edinburgh 15: 129, 1884 (Spruce 1884).

*** Anoplolejeunea conferta (C.F.W.Meissn. ex Spreng.) A.Evans, Bull. Torrey Bot. Club 35 (4): 175, 1908 (Evans 1908a). Bas.: Jungermannia conferta C.F.W.Meissn. ex Spreng. Syst. Veg. (ed. 16) [Sprengel] 4 (2): 325, 1827 (Sprengel 1827b).

*** **Echinolejeunea R.M.Schust.**, Beih. Nova Hedwigia 9: 187, 1963 (Schuster 1963a).

*** Echinolejeunea papillata (Mitt.) R.M.Schust. ex Hamlin, Rec. Domin. Mus. 7: 260, 1972 (Hamlin 1972). Bas.: Lejeunea papillata Mitt., Bot. antarct. voy. II (Fl. Nov.-Zel. 2): 158, 1854 (Mitten 1854).

** **Kymatolejeunea Grolle**, Wiss. Z. Friedrich-Schiller-Univ. Jena, Math.-Naturwiss. Reihe 32 (6): 1005, 1984 (Grolle 1984c).

*** Kymatolejeunea bartlettii Grolle, Wiss. Z. Friedrich-Schiller-Univ. Jena, Math.-Naturwiss. Reihe 32 (6): 1005, 1984 (Grolle 1984c).

########### ** subtrib. Leiolejeuneinae Schäf.-Verw. et Heinrichs

** **Leiolejeunea A.Evans**, Bull. Torrey Bot. Club 35 (8): 377, 1908 (Evans 1908b).

*** Leiolejeunea grandiflora A.Evans, Bull. Torrey Bot. Club 35 (8): 378, 1908 (Evans 1908b).

########### *** subtrib. Lejeuneinae Gradst.

*** **Harpalejeunea (Spruce) Schiffn.**, Hepat. (Engl.-Prantl): 126, 1893 (Schiffner 1893b). Bas.: Lejeunea subg. Harpalejeunea Spruce, Trans. & Proc. Bot. Soc. Edinburgh 15: 164, 1884 (Spruce 1884).

** **subg. Cleefiolejeunea Grolle et M.E.Reiner**, J. Bryol. 21 (1): 33, 1999 (Grolle and Reiner-Drehwald 1999).

*** Harpalejeunea grandis Grolle et M.E.Reiner, J. Bryol. 21 (1): 32, 1999 (Grolle and Reiner-Drehwald 1999).

** **subg. Harpalejeunea**

* Harpalejeunea acuta S.Winkl., Rev. Bryol. Lichénol. 42 (3): 812, 1976 (Winkler 1976). ^306^

*** Harpalejeunea ancistrodes (Spruce) Schiffn., Hepat. (Engl.-Prantl): 127, 1893 (Schiffner 1893b). Bas.: Lejeunea ancistrodes Spruce, Trans. & Proc. Bot. Soc. Edinburgh 15: 169, 1884 (Spruce 1884).

** Harpalejeunea buenaventurae Herzog, Feddes Repert. Spec. Nov. Regni Veg. 57 (1/2): 183, 1955 (Herzog 1955).

*** Harpalejeunea cinchonae (Nees) Schiffn., Hepat. (Engl.-Prantl): 127, 1893 (Schiffner 1893b). Bas.: Lejeunea cinchonae Nees, Syn. Hepat. 3: 342, 1845 (Gottsche et al. 1845b).

** Harpalejeunea cinchonae var. strigulosa Herzog, Svensk Bot. Tidskr. 51 (1): 192, 1957 (Herzog 1957a).

** Harpalejeunea decurvicuspis (Besch. et C.Massal.) P.Syd., Just’s Bot. Jahresber. 19: 246, 1894 (Sydow 1894). Bas.: Lejeunea decurvicuspis Besch. et C.Massal., Bull. Mens. Soc. Linn. Paris 1 (79): 639, 1886 (Bescherelle and Massalongo 1886).

** Harpalejeunea emarginata Jovet-Ast, Rev. Bryol. Lichénol. 16 (1/2): 38, 1947 [1948] (Jovet-Ast 1947b).

** Harpalejeunea exocellata Herzog, Rev. Bryol. Lichénol. 20 (1/2): 144, 1951 [1952] (Herzog 1951a).

** Harpalejeunea grandistipula R.M.Schust., J. Hattori Bot. Lab. 87: 290, 1999 (Schuster 1999c).

*** Harpalejeunea harpaphylla (Herzog) Bischl., Rev. Bryol. Lichénol. 33 (1/2): 164, 1964 (Bischler 1964). Bas.: Drepanolejeunea harpaphylla Herzog, Svensk Bot. Tidskr. 46 (1): 93, 1952 (Herzog 1952e).

** Harpalejeunea herzogii Jovet-Ast, Feddes Repert. Spec. Nov. Regni Veg. 58: 19, 1955 (Jovet-Ast 1955).

** Harpalejeunea longibracteata (Spruce) Steph., Sp. Hepat. (Stephani) 5: 254, 1913 (Stephani 1913a). Bas.: Lejeunea longibracteata Spruce, Bull. Soc. Bot. France (Congr. Bot.) 36: cciii, 1889 [1890] (Spruce 1889).

** Harpalejeunea marginalis (Hook.f. et Taylor) Steph., Sp. Hepat. (Stephani) 5: 271, 1913 (Stephani 1913a). Bas.: Jungermannia marginalis Hook.f. et Taylor, London J. Bot. 4: 91, 1845 (Hooker and Taylor 1845).

*** Harpalejeunea molleri (Steph.) Grolle, Taxon 38 (1): 89, 1989 (Grolle 1989c). Bas.: Lejeunea molleri Steph., Hedwigia 26 (1): 3, 1887 (Stephani 1887).

** Harpalejeunea molleri subsp. integra (R.M.Schust.) Damsh., Ill. Fl. Nord. Liverw. Hornw.: 615, 2002 (Damsholt 2002). Bas.: Harpalejeunea ovata subsp. integra R.M.Schust., J. Elisha Mitchell Sci. Soc. 83 (4): 199, 1967 (Schuster 1967a).

*** Harpalejeunea oxyphylla (Nees et Mont.) Steph., Sp. Hepat. (Stephani) 5: 255, 1913 (Stephani 1913a). Bas.: Lejeunea oxyphylla Nees et Mont., Ann. Sci. Nat. Bot. (sér. 2) 19: 264, 1843 (Montagne 1843).

*** Harpalejeunea parasitica (Hook.f. et Taylor) Steph., Sp. Hepat. (Stephani) 5: 268, 1913 (Stephani 1913a). Bas.: Jungermannia parasitica Hook.f. et Taylor, London J. Bot. 3: 477, 1844 (Hooker and Taylor 1844b).

** Harpalejeunea pinaundensis Grolle, J. Hattori Bot. Lab. 46: 44, 1979 (Grolle 1979a).

** Harpalejeunea reflexula A.Evans, Bull. Torrey Bot. Club 35 (8): 375, 1908 (Evans 1908b).

** Harpalejeunea scabra Gradst. et Schäf.-Verw., Cryptog. Bryol. 32 (2): 102, 2011 (Gradstein and Schäfer-Verwimp 2011).

*** Harpalejeunea schiffneri S.W.Arnell, Österr. Akad. Wiss., Math.-Naturwiss. Kl., Denkschr. 111: 102, 1964 (Schiffner and Arnell 1964).

* Harpalejeunea solitaria (Gottsche) Steph., Sp. Hepat. (Stephani) 5: 246, 1913 (Stephani 1913a). Bas.: Lejeunea solitaria Gottsche, Abh. Naturwiss. Vereins Bremen 7: 356, 1882 (Gottsche 1882).

*** Harpalejeunea stricta (Lindenb. et Gottsche) Steph., Sp. Hepat. (Stephani) 5: 259, 1913 (Stephani 1913a). Bas.: Lejeunea stricta Lindenb. et Gottsche, Syn. Hepat. 5: 756, 1847 (Gottsche et al. 1847).

** Harpalejeunea subacuta A.Evans, Bull. Torrey Bot. Club 30 (10): 547, 1903 (Evans 1903c).

* Harpalejeunea tenuicuspis (Spruce) Schiffn., Hepat. (Engl.-Prantl): 127, 1893 (Schiffner 1893b). Bas.: Lejeunea tenuicuspis Spruce, Trans. & Proc. Bot. Soc. Edinburgh 15: 170, 1884 (Spruce 1884). ^307^

*** Harpalejeunea tridens (Besch. et Spruce) Steph., Sp. Hepat. (Stephani) 5: 263, 1913 (Stephani 1913a). Bas.: Lejeunea tridens Besch. et Spruce, Bull. Soc. Bot. France (Congr. Bot.) 36: clxxxi, 1889 [1890] (Bescherelle and Spruce 1889).

** Harpalejeunea uncinata Steph., Hedwigia 35 (3): 97, 1896 (Stephani 1896b).

* Harpalejeunea uncinata var. setulosa Herzog, Feddes Repert. Spec. Nov. Regni Veg. 57 (1/2): 183, 1955 (Herzog 1955).

***Incertae sedis***

* Harpalejeunea grossearmata Steph., Biblioth. Bot. 87 (2): 256, 1916 (Stephani 1916a). ^308^

* Harpalejeunea renneri Herzog, Memoranda Soc. Fauna Fl. Fennica 26: 54, 1950 [1951] (Herzog 1950b). ^309^

* Harpalejeunea spruceana Steph., Biblioth. Bot. 87 (2): 257, 1916 (Stephani 1916a). ^310^

* Harpalejeunea vitrea Herzog, Memoranda Soc. Fauna Fl. Fennica 27: 94, 1952 (Herzog 1952c). ^311^

** **Hattoriolejeunea Mizut.**, J. Hattori Bot. Lab. 61: 303, 1986 [1987] (Mizutani 1986b).

** Hattoriolejeunea akiyamae Mizut., J. Hattori Bot. Lab. 61: 303, 1986 [1987] (Mizutani 1986b).

*** **Lejeunea Lib.**, Ann. Gen. Sci. Phys. 6: 373, 1820 (Libert 1820) nom. conserv. ^312^

** Lejeunea tunquiniensis M.E.Reiner et Drehwald, Nova Hedwigia 100 (3/4): 584, 2015 (Reiner-Drehwald 2015).

** **subg. Lejeunea**

*** Lejeunea abyssinica (Gola) Cufod., Phyton (Horn) 4: 75, 1952 (Cufodontis 1952). Bas.: Eulejeunea abyssinica Gola, Ann. Bot. (Rome) 13 (1): 70, 1914 (Gola 1914a).

*** Lejeunea acanthogona Spruce, Trans. & Proc. Bot. Soc. Edinburgh 15: 177, 1884 (Spruce 1884).

** Lejeunea acuminata (Lehm. et Lindenb.) Lehm., Nov. Stirp. Pug. 7: 22, 1838 (Lehmann 1838). Bas.: Jungermannia acuminata Lehm. et Lindenb., Nov. Stirp. Pug. 6: 49, 1834 (Lehmann 1834).

** Lejeunea acuta Mitt., J. Proc. Linn. Soc., Bot. 7 (27): 167, 1863 (Mitten 1863).

** Lejeunea acutata (Steph.) Solari, J. Hattori Bot. Lab. 54: 541, 1983 (Solari 1983a). Bas.: Strepsilejeunea acutata Steph., Hedwigia 35 (3): 127, 1896 (Stephani 1896b).

*** Lejeunea adpressa Nees, Repert. Pharm. 76: 45, 1842 (von Flotow et al. 1842). ^313^

*** Lejeunea aethiopica E.W.Jones, J. Bryol. 13 (3): 387, 1985 (Jones 1985).

** Lejeunea alaskana (R.M.Schust. et Steere) Inoue et Steere, J. Hattori Bot. Lab. 44: 330, 1978 (Steere and Inoue 1978). Bas.: Hygrolejeunea alaskana R.M.Schust. et Steere, Bull. Torrey Bot. Club 85 (3): 190, 1958 (Schuster and Steere 1958).

*** Lejeunea alata Gottsche, Syn. Hepat. 3: 406, 1845 (Gottsche et al. 1845b).

** Lejeunea alata var. patriciae Pócs, Candollea 56 (1): 72, 2001 (Pócs 2001).

*** Lejeunea albescens (Steph.) Mizut., J. Hattori Bot. Lab. 33: 245, 1970 (Mizutani 1970). Bas.: Taxilejeunea albescens Steph., Hedwigia 35 (3): 132, 1896 (Stephani 1896b).

* Lejeunea albiflora Colenso, Trans. & Proc. New Zealand Inst. 21: 72, 1889 (Colenso 1889).

** Lejeunea aloba Sande Lac., Plagiochila Sandei: 10, 1856 (Sande Lacoste 1856c).

** Lejeunea alobifolia H.A.Mill., Phytologia 47 (4): 323, 1981 (Miller 1981). *Nom. nov. pro Lejeunea aloba* Steph., Sp. Hepat. (Stephani) 5: 767, 1915 (Stephani 1915b), *nom. illeg*.

** Lejeunea amaniensis E.W.Jones, J. Bryol. 13 (3): 392, 1985 (Jones 1985).

** Lejeunea ambigua Lindenb. et Gottsche, Syn. Hepat. 5: 764, 1847 (Gottsche et al. 1847).

* Lejeunea amentulifera Steph., Sp. Hepat. (Stephani) 5: 707, 1915 (Stephani 1915b).

** Lejeunea androgyna R.M.Schust., Phytologia 45 (5): 432, 1980 (Schuster 1980b).

* Lejeunea angulifolia Mitt., Philos. Trans. 168: 400, 1879 (Mitten 1879). ^314^

*** Lejeunea angusta (Lehm. et Lindenb.) Mont., Hist. Phys. Cuba, Bot., Pl. Cell.: 469, 1842 (Montagne 1842a). Bas.: Jungermannia angusta Lehm. et Lindenb., Nov. Stirp. Pug. 4: 52, 1832 (Lehmann 1832).

** Lejeunea anisophylla Mont., Ann. Sci. Nat. Bot. (sér. 2) 19: 263, 1843 (Montagne 1843).

*** Lejeunea anomala Lindenb. et Gottsche, Linnaea 24 (6): 636, 1851 [1852] (Lindenberg and Gottsche 1851a).

* Lejeunea antillana Steph., Hedwigia 27 (11/12): 281, 1888 (Stephani 1888c).

** Lejeunea aphanes Spruce, J. Bot. 19: 36, 1881 (Spruce 1881b).

*** Lejeunea apiculata Sande Lac., Ned. Kruidk. Arch. 3: 421, 1854 [1855] (Sande Lacoste 1854).

** Lejeunea aquatica Horik., Sci. Rep. Tôhoku Imp. Univ., Ser. 4, Biol. 5 (4): 643, 1929 [1930] (Horikawa 1929c).

** Lejeunea aquatica var. apiculata S.Hatt., Bull. Tokyo Sci. Mus. 11: 108, 1944 (Hattori 1944d).

** Lejeunea armitii (Steph.) Steph., Sp. Hepat. (Stephani) 5: 768, 1915 (Stephani 1915b). Bas.: Eulejeunea armitii Steph., Hedwigia 28 (3): 169, 1889 (Stephani 1889d).

* Lejeunea asperifolia Steph., Sp. Hepat. (Stephani) 5: 708, 1915 (Stephani 1915b).

*** Lejeunea asperrima Spruce, Trans. & Proc. Bot. Soc. Edinburgh 15: 160, 1884 (Spruce 1884).

** Lejeunea asperula (Steph.) Mizut., J. Hattori Bot. Lab. 33: 236, 1970 (Mizutani 1970). Bas.: Taxilejeunea asperula Steph., Sp. Hepat. (Stephani) 5: 499, 1914 (Stephani 1914b).

** Lejeunea asprella Spruce, Trans. & Proc. Bot. Soc. Edinburgh 15: 175, 1884 (Spruce 1884).

* Lejeunea atheatostipa Spruce, J. Bot. 33: 83, 1895 (Gepp 1895a).

** Lejeunea barbata (Herzog) R.L.Zhu et M.J.Lai, Ann. Bot. Fenn. 48 (5): 376, 2011 (Wang et al. 2011). Bas.: Rectolejeunea barbata Herzog, J. Hattori Bot. Lab. 14: 49, 1955 (Herzog and Noguchi 1955).

*** Lejeunea bermudiana (A.Evans) R.M.Schust., Hepat. Anthocerotae N. Amer. 4: 1105, 1980 (Schuster 1980c). Bas.: Crossotolejeunea bermudiana A.Evans, Bull. Torrey Bot. Club 33 (3): 132, 1906 (Evans 1906c).

** Lejeunea bidentula Herzog, Symb. Sin. 5: 51, 1930 (Nicholson et al. 1930).

** Lejeunea biformis Gottsche, Ann. Sci. Nat. Bot. (sér. 5) 1: 162 (68), 1864 (Gottsche 1864).

** Lejeunea blepharogona Spruce, Trans. & Proc. Bot. Soc. Edinburgh 15: 178, 1884 (Spruce 1884).

** Lejeunea blomquistii R.M.Schust., J. Elisha Mitchell Sci. Soc. 78 (1): 64, 1962 (Schuster 1962b).

*** Lejeunea boliviensis (Steph.) R.L.Zhu et M.E.Reiner, Bryologist 107 (2): 237, 2004 (Zhu and Reiner-Drehwald 2004). Bas.: Strepsilejeunea boliviensis Steph., Biblioth. Bot. 87 (2): 257, 1916 (Stephani 1916a).

** Lejeunea boryana Mont., Ann. Sci. Nat. Bot. (sér. 2) 9: 47, 1838 (Montagne 1838).

** Lejeunea brenanii E.W.Jones, J. Bryol. 10 (4): 391, 1979 (Jones 1979).

*** Lejeunea calcicola R.M.Schust., J. Elisha Mitchell Sci. Soc. 73 (2): 404, 1957 (Schuster 1957d).

** Lejeunea calcicola var. mexicana R.M.Schust., J. Elisha Mitchell Sci. Soc. 73 (2): 408, 1957 (Schuster 1957d).

** Lejeunea canariensis (Steph.) Steph., Sp. Hepat. (Stephani) 5: 802, 1915 (Stephani 1915b). Bas.: Eulejeunea canariensis Steph., Mém. Soc. Bot. France 7: 42, 1907 (Pitard and Corbière 1907).

*** Lejeunea cancellata Nees et Mont., Hist. Phys. Cuba, Bot., Pl. Cell.: 472, 1842 (Montagne 1842a).

** Lejeunea cantabrigiensis E.W.Jones, J. Bryol. 15 (4): 669, 1989 (Jones 1989).

*** Lejeunea capensis Gottsche, Syn. Hepat. 3: 374, 1845 (Gottsche et al. 1845b).

* Lejeunea caroliniana Austin, Bot. Bull. (Hanover) 1 (8): 36, 1876 (Austin 1876a). ^315^

*** Lejeunea catinulifera Spruce, Trans. & Proc. Bot. Soc. Edinburgh 15: 233, 1884 (Spruce 1884).

*** Lejeunea caulicalyx (Steph.) M.E.Reiner et Goda, J. Hattori Bot. Lab. 89: 13, 2000 (Reiner-Drehwald and Goda 2000). Bas.: Crossotolejeunea caulicalyx Steph., Sp. Hepat. (Stephani) 5: 237, 1913 (Stephani 1913a).

*** Lejeunea cavifolia (Ehrh.) Lindb., Revis. crit. icon.: 43, 1871 (Lindberg 1871). Bas.: Jungermannia cavifolia Ehrh., Beitr. Naturk. (Ehrhart) 4: 45, 1789 (Ehrhart 1789).

** Lejeunea caviloba (Steph.) Besch., J. Bot. (Morot) 12: 140, 1898 (Bescherelle 1898). Bas.: Eulejeunea caviloba Steph., Hedwigia 35 (3): 86, 1896 (Stephani 1896b).

*** Lejeunea cerina (Lehm. et Lindenb.) Lehm. et Lindenb., Syn. Hepat. 3: 391, 1845 (Gottsche et al. 1845b). Bas.: Jungermannia cerina Lehm. et Lindenb., Nov. Stirp. Pug. 5: 16, 1833 (Lehmann 1833).

** Lejeunea chaishanensis S.H.Lin, Yushania 9: 7, 1992 (Lin and Yang 1992).

** Lejeunea cladogyna A.Evans, Amer. J. Bot. 5 (3): 134, 1918 (Evans 1918).

** Lejeunea clavata Lindenb., Syn. Hepat. 3: 379, 1845 (Gottsche et al. 1845b).

** Lejeunea claviformis Lindenb. ex Steph., Sp. Hepat. (Stephani) 5: 727, 1915 (Stephani 1915b).

** Lejeunea cochleata Spruce, Trans. & Proc. Bot. Soc. Edinburgh 15: 273, 1884 (Spruce 1884).

** Lejeunea cocoes Mitt., J. Proc. Linn. Soc., Bot. 5 (18): 114, 1860 [1861] (Mitten 1860c).

*** Lejeunea colensoana (Steph.) M.A.M.Renner, Austral. Syst. Bot. 23 (6): 455, 2010 (Renner et al. 2010b). Bas.: Taxilejeunea colensoana Steph., Hedwigia 35 (3): 132, 1896 (Stephani 1896b).

** Lejeunea compacta (Steph.) Steph., Sp. Hepat. (Stephani) 5: 771, 1915 (Stephani 1915b). Bas.: Eulejeunea compacta Steph., Bull. Herb. Boissier 5 (2): 93, 1897 (Stephani 1897b).

** Lejeunea concinnula Spruce et Steph., J. Bot. 25: 39, 1887 (Spruce 1887a).

** Lejeunea connatistipula (Steph.) Steph., Sp. Hepat. (Stephani) 5: 772, 1915 (Stephani 1915b). Bas.: Eulejeunea connatistipula Steph., Hedwigia 35 (3): 87, 1896 (Stephani 1896b).

** Lejeunea contracta Mizut., J. Hattori Bot. Lab. 33: 248, 1970 (Mizutani 1970).

** Lejeunea controversa Gottsche, Hepat. Eur., Leberm. 56-57: no 556, 1873 (Gottsche and Rabenhorst 1873b).

** Lejeunea convexiloba M.L.So et R.L.Zhu, Bryologist 101 (1): 137, 1998 (So and Zhu 1998).

** Lejeunea corcovadae (Steph.) Bischl., Nova Hedwigia 5 (1/2): 406, 1963 (Bischler et al. 1963). Bas.: Microlejeunea corcovadae Steph., Sp. Hepat. (Stephani) 5: 820, 1915 (Stephani 1915b).

** Lejeunea cordiflora Spruce, Trans. & Proc. Bot. Soc. Edinburgh 15: 283, 1884 (Spruce 1884).

** Lejeunea corralensis A.Evans, Ann. Bryol. 3: 86, 1930 (Evans 1930b).

*** Lejeunea corynantha Spruce, J. Linn. Soc., Bot. 30 (210): 344, 1895 (Gepp 1895b).

** Lejeunea crassiretis Mitt., Fl. vit.: 414, 1871 [1873] (Mitten 1871).

*** Lejeunea cristulata (Steph.) M.E.Reiner et Goda, J. Hattori Bot. Lab. 89: 21, 2000 (Reiner-Drehwald and Goda 2000). Bas.: Crossotolejeunea cristulata Steph., Hedwigia 35 (3): 75, 1896 (Stephani 1896b).

*** Lejeunea cristuliflora (Steph.) M.E.Reiner et Goda, J. Hattori Bot. Lab. 89: 19, 2000 (Reiner-Drehwald and Goda 2000). Bas.: Crossotolejeunea cristuliflora Steph., Sp. Hepat. (Stephani) 5: 231, 1913 (Stephani 1913a).

** Lejeunea cuspidistipula (Steph.) Steph. ex Watts, Proc. Linn. Soc. New South Wales (ser. 2) 27 (108): 493, 1903 (Watts 1903). Bas.: Eulejeunea cuspidistipula Steph., Hedwigia 35 (3): 88, 1896 (Stephani 1896b).

** Lejeunea cyanomontana R.M.Schust., Phytologia 45 (5): 432, 1980 (Schuster 1980b).

** Lejeunea cyanophora R.M.Schust., J. Hattori Bot. Lab. 26: 246, 1963 (Schuster 1963b).

** Lejeunea cyathearum E.W.Jones, J. Bryol. 8 (1): 86, 1974 (Jones 1974).

*** Lejeunea cyathophora Mitt., Hooker’s J. Bot. Kew Gard. Misc. 3: 359, 1851 (Mitten 1851).

** Lejeunea denticalyx (Steph.) Steph., Sp. Hepat. (Stephani) 5: 794, 1915 (Stephani 1915b). Bas.: Eulejeunea denticalyx Steph., Hedwigia 28 (3): 169, 1889 (Stephani 1889d).

** Lejeunea denticuspis (Steph.) Mizut., J. Hattori Bot. Lab. 36: 160, 1972 [1973] (Mizutani 1972a). Bas.: Strepsilejeunea denticuspis Steph., Hedwigia 35 (3): 129, 1896 (Stephani 1896b).

** Lejeunea denudata (Pearson) J.J.Engel, Bryologist 78 (3): 361, 1975 (Engel 1975). Bas.: Eulejeunea denudata Pearson, J. Linn. Soc., Bot. 46 (305): 39, 1922 (Pearson 1922b).

*** Lejeunea deplanata Nees, Syn. Hepat. 3: 368, 1845 (Gottsche et al. 1845b).

** Lejeunea deplanata var. cuspidata (Steph.) M.E.Reiner, Nova Hedwigia 91 (3/4): 529, 2010 (Reiner-Drehwald 2010). Bas.: Pycnolejeunea cuspidata Steph., Sp. Hepat. (Stephani) 5: 605, 1914 (Stephani 1914b).

** Lejeunea diaphana Spruce, Trans. & Proc. Bot. Soc. Edinburgh 15: 168, 1884 (Spruce 1884).

** Lejeunea dimorpha T.Kodama, J. Hattori Bot. Lab. 41: 384, 1976 (Kodama 1976).

** Lejeunea dipterocarpa E.W.Jones, J. Bryol. 7 (1): 44, 1972 (Jones 1972).

*** Lejeunea discreta Lindenb., Syn. Hepat. 3: 361, 1845 (Gottsche et al. 1845b).

* Lejeunea disjecta Spruce, J. Linn. Soc., Bot. 30 (210): 347, 1895 (Gepp 1895b).

** Lejeunea diversicuspis Spruce, Trans. & Proc. Bot. Soc. Edinburgh 15: 176, 1884 (Spruce 1884).

** Lejeunea drummondii Taylor, London J. Bot. 5: 400, 1846 (Taylor 1846b).

** Lejeunea ecarinata (Steph.) J.M.Coult., Barnes et Arthur, Bot. Gaz. 15 (12): 349, 1890 (Coulter et al. 1890). Bas.: Eulejeunea ecarinata Steph., Bot. Gaz. 15 (11): 283, 1890 (Stephani 1890c).

** Lejeunea eckloniana Lindenb., Syn. Hepat. 3: 381, 1845 (Gottsche et al. 1845b).

** Lejeunea eifrigii Mizut., J. Hattori Bot. Lab. 33: 244, 1970 (Mizutani 1970). *Nom. nov. pro Taxilejeunea acutiloba* Eifrig, Monogr. Stud. Indomal. Art. Taxilejeunea: 94, 1937 (Eifrig 1937).

* Lejeunea elongella Gottsche, Ann. Sci. Nat. Bot. (sér. 5) 1: 161, 1864 (Gottsche 1864).

*** Lejeunea erostrata M.E.Reiner et Goda, J. Hattori Bot. Lab. 89: 25, 2000 (Reiner-Drehwald and Goda 2000). *Nom. nov. pro Crossotolejeunea parva* Steph., Sp. Hepat. (Stephani) 5: 241, 1913 (Stephani 1913a).

*** Lejeunea exilis (Reinw., Blume et Nees) Grolle, J. Hattori Bot. Lab. 46: 353, 1979 (Grolle 1979d). Bas.: Jungermannia exilis Reinw., Blume et Nees, Nova Acta Phys.-Med. Acad. Caes. Leop.-Carol. Nat. Cur. 12 (1): 227, 1824 [1825] (Reinwardt et al. 1824a).

** Lejeunea exilis var. abnormis (Herzog) G.E.Lee, Polish Bot. J. 58 (1): 61, 2013 (Lee and Gradstein 2013). Bas.: Byssolejeunea abnormis Herzog, Hedwigia 80 (1/2): 84, 1941 (Herzog 1941b).

** Lejeunea fernandeziana S.W.Arnell, Ark. Bot. (n.ser.) 4 (1): 16, 1957 (Arnell 1957b).

** Lejeunea firma Mitt., J. Proc. Linn. Soc., Bot. 5 (18): 112, 1860 [1861] (Mitten 1860c).

** Lejeunea fissistipula (Steph.) Steph., Sp. Hepat. (Stephani) 5: 775, 1915 (Stephani 1915b). Bas.: Eulejeunea fissistipula Steph., Hedwigia 35 (3): 88, 1896 (Stephani 1896b).

** Lejeunea flagellaris Spruce, Trans. & Proc. Bot. Soc. Edinburgh 15: 273, 1884 (Spruce 1884).

*** Lejeunea flava (Sw.) Nees, Naturgesch. Eur. Leberm. 3: 277, 1838 (Nees 1838b). Bas.: Jungermannia flava Sw., Prodr. (Swartz): 144, 1788 (Swartz 1788). ^316^

** Lejeunea flava subsp. moorei (Lindb.) R.M.Schust., J. Elisha Mitchell Sci. Soc. 73 (1): 161, 1957 (Schuster 1957a). Bas.: Lejeunea moorei Lindb., Acta Soc. Sci. Fenn. 10: 487, 1875 (Lindberg 1875).

** Lejeunea flava subsp. orientalis R.M.Schust., J. Elisha Mitchell Sci. Soc. 73 (1): 161, 1957 (Schuster 1957a).

** Lejeunea flava var. pellucida Lindenb. et Gottsche, Linnaea 24 (6): 634, 1851 [1852] (Lindenberg and Gottsche 1851a).

** Lejeunea flava subsp. tabularis (Spreng.) S.W.Arnell, Hepat. South Africa: 199, 1963 (Arnell 1963b). Bas.: Jungermannia tabularis Spreng. Syst. Veg. (ed. 16) [Sprengel] 4 (2): 325, 1827 (Sprengel 1827b).

*** Lejeunea flavovirens Ångstr., Öfvers. Kongl. Vetensk.-Akad. Förh. 30 (5): 144, 1873 (Ångström 1873).

** Lejeunea fleischeri (Steph.) Mizut., J. Hattori Bot. Lab. 33: 238, 1970 (Mizutani 1970). Bas.: Hygrolejeunea fleischeri Steph., Sp. Hepat. (Stephani) 5: 560, 1914 (Stephani 1914b).

** Lejeunea floridana A.Evans, Bull. Torrey Bot. Club 32 (4): 185, 1905 (Evans 1905b).

*** Lejeunea fulfordiae (Jovet-Ast) R.L.Zhu, Syst. Bot. 33 (4): 617, 2008 (Zhu and Cheng 2008). Bas.: Amblyolejeunea fulfordiae Jovet-Ast, Rev. Bryol. Lichénol. 17 (1/4): 25, 1948 [1949] (Jovet-Ast 1948).

** Lejeunea fusagasugana Gottsche, Ann. Sci. Nat. Bot. (sér. 5) 1: 158, 1864 (Gottsche 1864).

** Lejeunea galeata Spruce, Trans. & Proc. Bot. Soc. Edinburgh 15: 172, 1884 (Spruce 1884).

** Lejeunea gayana Gottsche, Ann. Sci. Nat. Bot. (sér. 5) 1: 157, 1864 (Gottsche 1864).

** Lejeunea gibbiloba (Steph.) H.A.Mill., Phytologia 47 (4): 323, 1981 (Miller 1981). Bas.: Eulejeunea gibbiloba Steph., Sp. Hepat. (Stephani) 6: 418, 1923 (Stephani 1923).

*** Lejeunea glaucescens Gottsche, Syn. Hepat. 3: 378, 1845 (Gottsche et al. 1845b).

** Lejeunea glaucescens var. acrogyna R.M.Schust., J. Elisha Mitchell Sci. Soc. 73 (2): 400, 1957 (Schuster 1957d).

* Lejeunea glaucescens var. obsoleta R.M.Schust., J. Elisha Mitchell Sci. Soc. 73 (2): 395, 1957 (Schuster 1957d).

** Lejeunea globosiflora (Steph.) Steph., Sp. Hepat. (Stephani) 5: 795, 1915 (Stephani 1915b). Bas.: Eulejeunea globosiflora Steph., Bih. Kongl. Svenska Vetensk.-Akad. Handl. 26 (III, 6): 65, 1900 (Stephani 1900b).

** Lejeunea gomphocalyx Spruce, Trans. & Proc. Bot. Soc. Edinburgh 15: 174, 1884 (Spruce 1884).

** Lejeunea gracilicaulis Spruce, Mem. Torrey Bot. Club 1 (3): 126, 1890 (Spruce 1890).

** Lejeunea gracilipes (Taylor) Spruce, Trans. & Proc. Bot. Soc. Edinburgh 15: 213, 1884 (Spruce 1884). Bas.: Omphalanthus gracilipes Taylor, London J. Bot. 5: 385, 1846 (Taylor 1846b).

** Lejeunea gracilis Steph., Sp. Hepat. (Stephani) 5: 777, 1915 (Stephani 1915b).

** Lejeunea gradsteiniana Pócs, Acta Biol. Pl. Agr. 1: 55, 2010 [2011] (Pócs 2010d). *Nom. nov. pro Ceratolejeunea aberrans* Steph., Sp. Hepat. (Stephani) 6: 399, 1923 (Stephani 1923).

** Lejeunea gradsteinii G.E.Lee, Damanhuri et Latiff, Acta Biol. Pl. Agr. 1: 29, 2010 [2011] (Lee et al. 2010).

* Lejeunea grossecristata (Steph.) E.W.Jones, Trans. Brit. Bryol. Soc. 5 (3): 556, 1968 (Jones 1968). Bas.: Hygrolejeunea grossecristata Steph., Hedwigia 35 (3): 102, 1896 (Stephani 1896b).

*** Lejeunea grossiretis (Steph.) M.E.Reiner et Goda, J. Hattori Bot. Lab. 89: 27, 2000 (Reiner-Drehwald and Goda 2000). Bas.: Crossotolejeunea grossiretis Steph., Hedwigia 35 (3): 75, 1896 (Stephani 1896b).

** Lejeunea grossistipula Steph., Sp. Hepat. (Stephani) 5: 739, 1915 (Stephani 1915b).

*** Lejeunea grossitexta (Steph.) M.E.Reiner et Goda, J. Hattori Bot. Lab. 89: 29, 2000 (Reiner-Drehwald and Goda 2000). Bas.: Crossotolejeunea grossitexta Steph., Sp. Hepat. (Stephani) 5: 240, 1913 (Stephani 1913a).

* Lejeunea grossiuscula Gottsche ex Steph., Sp. Hepat. (Stephani) 5: 739, 1915 (Stephani 1915b).

** Lejeunea hahnii Solari, J. Hattori Bot. Lab. 54: 543, 1983 (Solari 1983a). *Nom. nov. pro Microlejeunea grandistipula* Steph., Hedwigia 35 (3): 114, 1896 (Stephani 1896b).

* Lejeunea haitica Nees et Mont., Ann. Sci. Nat. Bot. (sér. 2) 19: 263, 1843 (Montagne 1843).

** Lejeunea hawaikiana M.A.M.Renner et de Lange, New Zealand J. Bot. 49 (3): 431, 2011 (Renner and de Lange 2011). *Nom. nov. pro Stenolejeunea acuminata* R.M.Schust., J. Hattori Bot. Lab. 89: 156, 2000 (Schuster 2000b).

** Lejeunea helenae Pearson, Forh. Vidensk.-Selsk. Kristiania 1886 (3): 6, 1886 (Pearson 1886).

** Lejeunea helmsiana (Steph.) Steph., Sp. Hepat. (Stephani) 5: 796, 1915 (Stephani 1915b). Bas.: Eulejeunea helmsiana Steph., Hedwigia 35 (3): 89, 1896 (Stephani 1896b).

*** Lejeunea hepaticola (Steph.) Steph., Sp. Hepat. (Stephani) 5: 714, 1915 (Stephani 1915b). Bas.: Eulejeunea hepaticola Steph., Hedwigia 27 (2): 60, 1888 (Stephani 1888a).

** Lejeunea hibernica Bischl., H.A.Mill. et Bonner ex Grolle, Lindbergia 3 (1/2): 48, 1975 [1976] (Grolle 1975c). Based on: Lejeunea hibernica Bischl., H.A.Mill. et Bonner, Nova Hedwigia 3 (4): 455, 1961 [1962] (Bischler et al. 1961), *nom. inval*.

** Lejeunea hodgsoniana Grolle ex R.J.Lewington, Bever. et M.A.M.Renner, PhytoKeys 29: 2, 2013 (Lewington et al. 2013).

** Lejeunea howeana Grolle, Wiss. Z. Friedrich-Schiller-Univ. Jena, Math.-Naturwiss. Reihe 31 (2): 219, 1982 (Grolle 1982). *Nom. nov. pro Cheilolejeunea wattsiana* Steph., J. & Proc. Roy. Soc. New South Wales 48 (1/2): 102, 1914 (Stephani and Watts 1914).

** Lejeunea hui R.L.Zhu, Beih. Nova Hedwigia 121: 134, 2001 (Zhu and So 2001).

** Lejeunea humefacta Spruce, Trans. & Proc. Bot. Soc. Edinburgh 15: 275, 1884 (Spruce 1884).

** Lejeunea hyalina (Steph.) L.Söderstr. et A.Hagborg, Phytotaxa 220 (2): 188, 2015 (Söderström et al. 2015a). Bas.: Pycnolejeunea hyalina Steph., Sp. Hepat. (Stephani) 5: 614, 1914 (Stephani 1914b).

** Lejeunea ibadana A.J.Harr. et E.W.Jones, J. Bryol. 12 (1): 40, 1982 (Jones 1982).

** Lejeunea immersa Spruce, Trans. & Proc. Bot. Soc. Edinburgh 15: 186, 1884 (Spruce 1884).

** Lejeunea increscens Spruce, Mem. Torrey Bot. Club 1 (3): 124, 1890 (Spruce 1890).

* Lejeunea inflatiloba (Steph.) H.A.Mill., Bonner et Bischl., Nova Hedwigia 14 (1): 67, 1967 (Miller et al. 1967). Bas.: Microlejeunea inflatiloba Steph., Sp. Hepat. (Stephani) 6: 422, 1923 (Stephani 1923).

*** Lejeunea inflexiloba Prantl, Hedwigia 31: xvi, 1892 (Prantl 1892). Based on: Crossotolejeunea inflexiloba J.B.Jack et Steph., Hedwigia 31 (1): 16, 1892 (Jack and Stephani 1892), *nom. inval*.

*** Lejeunea intricata Prantl, Hedwigia 31: xvi, 1892 (Prantl 1892). Based on: Crossotolejeunea intricata J.B.Jack et Steph., Hedwigia 31 (1): 17, 1892 (Jack and Stephani 1892), *nom. inval*.

*** Lejeunea isophylla E.W.Jones, Trans. Brit. Bryol. Soc. 5 (3): 559, 1968 (Jones 1968).

** Lejeunea japonica Mitt., Trans. Linn. Soc. London, Bot. 3 (3): 203, 1891 (Mitten 1891).

** Lejeunea jardinii Spruce, Bull. Soc. Bot. France (Congr. Bot.) 36: ccv, 1889 [1890] (Spruce 1889).

** Lejeunea julacea Steph., Sp. Hepat. (Stephani) 5: 715, 1915 (Stephani 1915b).

*** Lejeunea jungneri (Steph.) Steph., Cat. Afr. Pl. (Hiern) 2 (2): 318, 1901 (Stephani 1901d). Bas.: Eulejeunea jungneri Steph., Hedwigia 35 (3): 90, 1896 (Stephani 1896b).

** Lejeunea kashyapii M.Dey, D.K.Singh et D.Singh, J. Bryol. 30 (2): 126, 2008 (Dey et al. 2008).

* Lejeunea kilimandjarensis Steph., Sp. Hepat. (Stephani) 5: 716, 1915 (Stephani 1915b).

** Lejeunea kinabalensis Mizut., J. Hattori Bot. Lab. 33: 246, 1970 (Mizutani 1970).

** Lejeunea kodamae Ikegami et Inoue, J. Jap. Bot. 36 (1): 7, 1961 (Inoue 1961a).

** Lejeunea konosensis Mizut., J. Hattori Bot. Lab. 71: 127, 1992 (Mizutani 1992).

** Lejeunea kuerschneriana Pócs, Beih. Nova Hedwigia 138: 100, 2010 (Pócs 2010b).

*** Lejeunea laeta (Lehm. et Lindenb.) Lehm. et Lindenb., Syn. Hepat. 3: 380, 1845 (Gottsche et al. 1845b). Bas.: Jungermannia laeta Lehm. et Lindenb., Nov. Stirp. Pug. 6: 45, 1834 (Lehmann 1834).

** Lejeunea laii R.L.Zhu, J. Bryol. 30 (2): 173, 2008 (Wang and Zhu 2008). *Nom. nov. pro Microlejeunea ramulosa* Herzog, J. Hattori Bot. Lab. 14: 51, 1955 (Herzog and Noguchi 1955).

*** Lejeunea lamacerina (Steph.) Schiffn., Hedwigia 41 (5): 278, 1902 (Schiffner 1902). Bas.: Eulejeunea lamacerina Steph., Hedwigia 35 (3): 91, 1896 (Stephani 1896b). ^317^

** Lejeunea lamacerina subsp. gemminata R.M.Schust., J. Elisha Mitchell Sci. Soc. 73 (1): 168, 1957 (Schuster 1957a).

** Lejeunea latilobula (Herzog) R.L.Zhu et M.L.So, J. Bryol. 24 (2): 168, 2002 (Zhu and So 2002). Bas.: Taxilejeunea latilobula Herzog, Symb. Sin. 5: 50, 1930 (Nicholson et al. 1930).

** Lejeunea leptalea Spruce, Trans. & Proc. Bot. Soc. Edinburgh 15: 272, 1884 (Spruce 1884).

* Lejeunea leratii (Steph.) Mizut., J. Hattori Bot. Lab. 33: 243, 1970 (Mizutani 1970). Bas.: Hygrolejeunea leratii Steph., Sp. Hepat. (Stephani) 5: 562, 1914 (Stephani 1914b). ^318^

** Lejeunea leucosis Besch. et Spruce, Bull. Soc. Bot. France (Congr. Bot.) 36: clxxxviii, 1889 [1890] (Bescherelle and Spruce 1889).

* Lejeunea litoralis Steph., Sp. Hepat. (Stephani) 5: 778, 1915 (Stephani 1915b).

** Lejeunea lomana E.W.Jones, Bull. Brit. Mus. (Nat. Hist.), Bot. 11 (3): 257, 1983 (Jones and Harrington 1983).

** Lejeunea longicollis S.W.Arnell, Results Norweg. Sci. Exped. Tristan da Cunha 42: 12, 1958 (Arnell 1958b).

** Lejeunea longilobula Pócs, Beih. Nova Hedwigia 138: 112, 2010 (Pócs 2010b). *Nom. nov. pro* Lejeunea halei subsp. africana Pócs, J. Bryol. 29 (2): 89, 2007 (Müller and Pócs 2007).

** Lejeunea lowriana Steph., Sp. Hepat. (Stephani) 5: 779, 1915 (Stephani 1915b).

*** Lejeunea lumbricoides (Nees) Nees, Syn. Hepat. 3: 342, 1845 (Gottsche et al. 1845b). Bas.: Jungermannia lumbricoides Nees, Enum. Pl. Crypt. Javae: 40, 1830 (Nees 1830).

** Lejeunea lunatigastria Tixier, Ann. Hist.-Nat. Mus. Natl. Hung. 66: 98, 1974 (Tixier 1974).

** Lejeunea lyratiflora Prantl, Hedwigia 31: xvi, 1892 (Prantl 1892). Based on: Hygrolejeunea lyratiflora Steph., Hedwigia 31 (4): 169, 1892 (Stephani 1892g), *nom. inval*.

** Lejeunea magohukui Mizut., Misc. Bryol. Lichenol. 7 (7): 133, 1977 (Mizutani 1977).

** Lejeunea mandonii (Steph.) Müll.Frib., Leberm. Eur. 2 (9): 1281, 1958 (Müller 1958). Bas.: Microlejeunea mandonii Steph., Hedwigia 35 (3): 115, 1896 (Stephani 1896b).

*** Lejeunea masoalae Pócs, Beih. Nova Hedwigia 138: 103, 2010 (Pócs 2010b).

** Lejeunea massalongoana (Schiffn. ex P.Syd.) Solari, J. Hattori Bot. Lab. 54: 542, 1983 (Solari 1983a).Bas.: Harpalejeunea massalongoana Schiffn. ex P.Syd., Just’s Bot. Jahresber. 19: 246, 1894 (Sydow 1894).

** Lejeunea megalantha Spruce, Trans. & Proc. Bot. Soc. Edinburgh 15: 172, 1884 (Spruce 1884).

** Lejeunea mehrana M.Dey, D.K.Singh et D.Singh, J. Bryol. 30 (2): 128, 2008 (Dey et al. 2008).

*** Lejeunea meridensis Ilk.-Borg., Nova Hedwigia 80 (1/2): 59, 2005 (Ilkiu-Borges 2005).

** Lejeunea micholitzii Mizut., J. Hattori Bot. Lab. 33: 236, 1970 (Mizutani 1970). *Nom. nov. pro Hygrolejeunea parvisaccata* Steph., Sp. Hepat. (Stephani) 5: 567, 1914 (Stephani 1914b).

** Lejeunea microloba Taylor, London J. Bot. 5: 399, 1846 (Taylor 1846b).

*** Lejeunea mimula Hürl., Bauhinia 11 (1): 12, 1993 (Hürlimann 1993). *Nom. nov. pro Hygrolejeunea luteola* Steph., Sp. Hepat. (Stephani) 5: 553, 1914 (Stephani 1914b).

*** Lejeunea minutiloba A.Evans, Bull. Torrey Bot. Club 44 (11): 525, 1917 (Evans 1917d).

** Lejeunea minutiloba var. heterogyna R.M.Schust., J. Elisha Mitchell Sci. Soc. 73 (2): 425, 1957 (Schuster 1957d).

** Lejeunea mizutanii Grolle, J. Hattori Bot. Lab. 45: 178, 1979 (Grolle 1979c). *Nom. nov. pro Cheilolejeunea zollingeri* Steph., Hedwigia 34 (5): 245, 1895 (Stephani 1895b).

** Lejeunea molkenboeriana Sande Lac., Ned. Kruidk. Arch. 3: 421, 1854 [1855] (Sande Lacoste 1854).

*** Lejeunea monimiae (Steph.) Steph., Sp. Hepat. (Stephani) 5: 747, 1915 (Stephani 1915b). Bas.: Eulejeunea monimiae Steph., Hedwigia 35 (3): 91, 1896 (Stephani 1896b).

*** Lejeunea multidentata M.E.Reiner et Mustelier, J. Bryol. 26 (2): 103, 2004 (Reiner-Drehwald and Mustelier 2004).

* Lejeunea musae (Spreng.) Gottsche, Lindenb. et Nees, Syn. Hepat. 3: 407, 1845 (Gottsche et al. 1845b). Bas.: Jungermannia musae Spreng. Ann. Wetterauischen Ges. Gesammte Naturk. 1: 25, 1809 (Sprengel 1809).

** Lejeunea muscicola Spruce, Trans. & Proc. Bot. Soc. Edinburgh 15: 281, 1884 (Spruce 1884).

* Lejeunea muscicola var. palmicola Spruce, Trans. & Proc. Bot. Soc. Edinburgh 15: 282, 1884 (Spruce 1884).

** Lejeunea neelgherriana Gottsche, Syn. Hepat. 3: 354, 1845 (Gottsche et al. 1845b).

** Lejeunea nemoralis Gottsche, Ann. Sci. Nat. Bot. (sér. 5) 1: 159 (65), 1864 (Gottsche 1864).

** Lejeunea nepalensis Steph., Sp. Hepat. (Stephani) 5: 780, 1915 (Stephani 1915b).

* Lejeunea nesiotica Besch. et Spruce, Bull. Soc. Bot. France (Congr. Bot.) 36: clxxxviii, 1889 [1890] (Bescherelle and Spruce 1889).

** Lejeunea neumanniana Nees, Repert. Pharm. 76: 44, 1842 (von Flotow et al. 1842).

** Lejeunea nietneri (Steph.) Steph. ex Watts, Proc. Linn. Soc. New South Wales (ser. 2) 26 (104): 633, 1902 (Watts 1902). Bas.: Eulejeunea nietneri Steph., Hedwigia 35 (3): 91, 1896 (Stephani 1896b).

** Lejeunea notata Gottsche, Abh. Naturwiss. Vereins Bremen 7: 361, 1882 (Gottsche 1882).

** Lejeunea nymannii Steph., Sp. Hepat. (Stephani) 5: 781, 1915 (Stephani 1915b).

** Lejeunea obfusca Mitt., J. Proc. Linn. Soc., Bot. 5 (18): 114, 1860 [1861] (Mitten 1860c).

** Lejeunea obidensis Spruce, Trans. & Proc. Bot. Soc. Edinburgh 15: 277, 1884 (Spruce 1884).

* Lejeunea obscura Mitt., J. Proc. Linn. Soc., Bot. 5 (18): 112, 1860 [1861] (Mitten 1860c). ^319^

** Lejeunea obtusata Gottsche, Abh. Naturwiss. Vereins Bremen 7: 354, 1882 (Gottsche 1882).

** Lejeunea okomuensis E.W.Jones, Trans. Brit. Bryol. Soc. 5 (4): 787, 1969 (Jones 1969).

*** Lejeunea oligoclada Spruce, Bull. Soc. Bot. France (Congr. Bot.) 36: cxcix, 1889 [1890] (Spruce 1889).

*** Lejeunea oracola M.A.M.Renner, Austral. Syst. Bot. 23 (6): 448, 2010 (Renner et al. 2010b).

** Lejeunea osculatiana De Not., Mem. Reale Accad. Sci. Torino (ser. 2) 16: 233, 1857 (De Notaris 1857).

** Lejeunea otiana S.Hatt., Bot. Mag. (Tokyo) 65 (763/764): 15, 1952 (Hattori 1952a).

** Lejeunea ovalifolia Steph., Sp. Hepat. (Stephani) 5: 751, 1915 (Stephani 1915b).

** Lejeunea pacifica Mont., Ann. Sci. Nat. Bot. (sér. 2) 19: 262, 1843 (Montagne 1843).

** Lejeunea pallescens Mitt., Hooker’s J. Bot. Kew Gard. Misc. 3: 360, 1851 (Mitten 1851).

** Lejeunea pallida Lindenb. et Gottsche, Syn. Hepat. 5: 762, 1847 (Gottsche et al. 1847).

* Lejeunea pallidissima Gola, Nuovo Giorn. Bot. Ital. (n.ser.) 27 (2/4): 247, 1920 (Gola 1920). ^320^

*** Lejeunea papilionacea Prantl, Hedwigia 31: xvii, 1892 (Prantl 1892). Based on: Hygrolejeunea papilionacea Steph., Hedwigia 31 (4): 169, 1892 (Stephani 1892g), *nom. inval*.

* Lejeunea paratropa Spruce, Trans. & Proc. Bot. Soc. Edinburgh 15: 176, 1884 (Spruce 1884). ^321^

** Lejeunea patagonica (Steph.) Steph., Sp. Hepat. (Stephani) 5: 797, 1915 (Stephani 1915b). Bas.: Eulejeunea patagonica Steph., Bih. Kongl. Svenska Vetensk.-Akad. Handl. 26 (III, 6): 66, 1900 (Stephani 1900b).

** Lejeunea patens Lindb., Acta Soc. Sci. Fenn. 10: 482, 1875 (Lindberg 1875).

*** Lejeunea patersonii (Steph.) Steph., Sp. Hepat. (Stephani) 5: 784, 1915 (Stephani 1915b). Bas.: Eulejeunea patersonii Steph., Hedwigia 35 (3): 92, 1896 (Stephani 1896b).

*** Lejeunea patriciae Schäf.-Verw., Candollea 56 (1): 64, 2001 (Schäfer-Verwimp 2001a). *Nom. nov. pro Lejeunea pilifera* Tixier, Gard. Bull. Singapore 25 (3): 351, 1971 (Tixier 1971), *nom. illeg*.

*** Lejeunea paucidentata (Steph.) Grolle, J. Hattori Bot. Lab. 69: 191, 1991 (Grolle 1991). Bas.: Crossotolejeunea paucidentata Steph., Hedwigia 35 (3): 76, 1896 (Stephani 1896b).

*** Lejeunea pectinella Mizut., J. Hattori Bot. Lab. 33: 239, 1970 (Mizutani 1970).

** Lejeunea perigonialis Gottsche, Mexik. Leverm.: 223, 1863 (Gottsche 1863).

*** Lejeunea perpapillosa M.E.Reiner et K.C.Pôrto, Nova Hedwigia 85 (3/4): 542, 2007 (Reiner-Drehwald and Pôrto 2007).

* Lejeunea pertusa (Corda ex Nees et Mont.) Gottsche, Lindenb. et Nees, Syn. Hepat. 3: 407, 1845 (Gottsche et al. 1845b). Bas.: Jungermannia pertusa Corda ex Nees et Mont., Ann. Sci. Nat. Bot. (sér. 2) 5: 63, 1836 (Nees and Montagne 1836).

*** Lejeunea phyllobola Nees et Mont., Hist. Phys. Cuba, Bot., Pl. Cell.: 471, 1842 (Montagne 1842a).

* Lejeunea phyllobola var. turgidula Spruce, Trans. & Proc. Bot. Soc. Edinburgh 15: 259, 1884 (Spruce 1884).

*** Lejeunea planiloba A.Evans, Proc. Wash. Acad. Sci. 8: 147, 1906 (Evans 1906b).

** Lejeunea polilloensis Steph., Sp. Hepat. (Stephani) 5: 786, 1915 (Stephani 1915b).

** Lejeunea praetervisa Steph., Sp. Hepat. (Stephani) 5: 752, 1915 (Stephani 1915b).

** Lejeunea primordialis (Hook.f. et Taylor) Gottsche, Lindenb. et Nees, Syn. Hepat. 3: 375, 1845 (Gottsche et al. 1845b). Bas.: Jungermannia primordialis Hook.f. et Taylor, London J. Bot. 4: 92, 1845 (Hooker and Taylor 1845).

** Lejeunea princeps (Steph.) Mizut., J. Hattori Bot. Lab. 34: 454, 1971 (Mizutani 1971a). Bas.: Hygrolejeunea princeps Steph., Sp. Hepat. (Stephani) 5: 568, 1914 (Stephani 1914b).

** Lejeunea procumbens Mitt., Fl. vit.: 413, 1871 [1873] (Mitten 1871).

** Lejeunea propagulifera Gradst., Phytotaxa 9: 54, 2010 (Söderström et al. 2010a). *Nom. nov. pro Trachylejeunea schiffneri* Herzog, Svensk Bot. Tidskr. 42 (3): 239, 1948 (Herzog 1948).

** Lejeunea pteridis Besch. et Spruce, Bull. Soc. Bot. France (Congr. Bot.) 36: clxxxvii, 1889 [1890] (Bescherelle and Spruce 1889).

*** Lejeunea ptosimophylla C.Massal., Nuovo Giorn. Bot. Ital. 13 (2): 123, 1881 (Massalongo 1881).

*** Lejeunea puiggariana Steph., Sp. Hepat. (Stephani) 5: 754, 1915 (Stephani 1915b).

*** Lejeunea pulverulenta (Steph.) M.E.Reiner, Cryptog. Bryol. 26 (1): 60, 2005 (Reiner-Drehwald 2005b). Bas.: Taxilejeunea pulverulenta Steph., Sp. Hepat. (Stephani) 5: 477, 1914 (Stephani 1914b).

*** Lejeunea pulvinata Nees et Mont., Ann. Sci. Nat. Bot. (sér. 2) 5: 61, 1836 (Nees and Montagne 1836). *Nom. nov. pro Jungermannia pulvinata* Lehm. et Lindenb., Nov. Stirp. Pug. 5: 15, 1833 (Lehmann 1833), *nom. illeg*.

*** Lejeunea raddiana Lindenb., Syn. Hepat. 3: 342, 1845 (Gottsche et al. 1845b).

** Lejeunea radicans Lindenb. et Gottsche, Syn. Hepat. 5: 766, 1847 (Gottsche et al. 1847).

** Lejeunea ramosissima Steph., Bot. Jahrb. Syst. 8 (2): 88, 1886 (Stephani 1886d).

** Lejeunea rara Steph., Sp. Hepat. (Stephani) 5: 798, 1915 (Stephani 1915b). ^322^

* Lejeunea ravenelii Austin, Bot. Bull. (Hanover) 1 (8): 35, 1876 (Austin 1876a). ^323^

** Lejeunea recurva M.E.Reiner, Polish Bot. J. 58 (2): 423, 2013 (Reiner-Drehwald et al. 2013). *Nom. nov. pro Hygrolejeunea herzogii* Steph., Biblioth. Bot. 87 (2): 265, 1916 (Stephani 1916a), *nom. illeg*.

*** Lejeunea reflexistipula (Lehm. et Lindenb.) Lehm. et Lindenb., Syn. Hepat. 3: 335, 1845 (Gottsche et al. 1845b). Bas.: Jungermannia reflexistipula Lehm. et Lindenb., Nov. Stirp. Pug. 5: 10, 1833 (Lehmann 1833).

** Lejeunea reflexistipula var. costaricensis (Steph.) M.E.Reiner, Nova Hedwigia 81 (3/4): 408, 2005 (Reiner-Drehwald 2005a). Bas.: Hygrolejeunea costaricensis Steph., Hedwigia 35 (3): 100, 1896 (Stephani 1896b).

*** Lejeunea reinerae Ilk.-Borg., Nova Hedwigia 80 (1/2): 61, 2005 (Ilkiu-Borges 2005). *Nom. nov. pro Echinocolea herzogii* Mizut. et Grolle, Bot. Mag. (Tokyo) 77 (915): 333, 1964 (Grolle 1964d).

** Lejeunea resupinata (Steph.) Steph., Sp. Hepat. (Stephani) 5: 757, 1915 (Stephani 1915b). Bas.: Eulejeunea resupinata Steph., Bih. Kongl. Svenska Vetensk.-Akad. Handl. 23 (III, 2): 22, 1897 (Stephani 1897a).

** Lejeunea reticulata Herzog, Nat. Hist. Juan Fernandez (Botany) 2 (5): 747, 1942 (Herzog 1942a).

*** Lejeunea rhigophila M.A.M.Renner, Austral. Syst. Bot. 23 (6): 453, 2010 (Renner et al. 2010b).

** Lejeunea rhodesiae (Sim) R.M.Schust., J. Hattori Bot. Lab. 25: 71, 1962 (Schuster 1962a). Bas.: Stylolejeunea rhodesiae Sim, Trans. Roy. Soc. South Africa 15 (1): 68, 1926 (Sim 1926).

*** Lejeunea rionegrensis Spruce, Trans. & Proc. Bot. Soc. Edinburgh 15: 579, 1885 (Spruce 1885). *Nom. nov. pro Lejeunea implexa* Spruce, Trans. & Proc. Bot. Soc. Edinburgh 15: 240, 1884 (Spruce 1884), *nom. inval*.

** Lejeunea riparia Mitt., J. Proc. Linn. Soc., Bot. 5 (18): 113, 1860 [1861] (Mitten 1860c).

* Lejeunea rothii (Schwägr.) Gottsche, Lindenb. et Nees, Syn. Hepat. 3: 407, 1845 (Gottsche et al. 1845b). Bas.: Jungermannia rothii Schwägr., Hist. Musc. Hepat. Prodr.: 17, 1814 (Schwägrichen 1814).

*** Lejeunea rotundifolia Mitt., Hooker’s J. Bot. Kew Gard. Misc. 3: 359, 1851 (Mitten 1851).

** Lejeunea sanctae-helenae M.Wigginton, J. Bryol. 29 (1): 12, 2007 (Wigginton 2007).

** Lejeunea schusteri Grolle, Haussknechtia 8: 60, 2001 (Grolle 2001). *Nom. nov. pro Rectolejeunea denudata* R.M.Schust., J. Hattori Bot. Lab. 89: 143, 2000 (Schuster 2000c).

** Lejeunea semiscabrida Gottsche, Ann. Sci. Nat. Bot. (sér. 5) 1: 154 (60), 1864 (Gottsche 1864).

** Lejeunea semperi Steph., Sp. Hepat. (Stephani) 5: 788, 1915 (Stephani 1915b).

** Lejeunea seriata Lindenb. et Gottsche, Syn. Hepat. 5: 762, 1847 (Gottsche et al. 1847).

** Lejeunea sessiliflora (Steph.) Grolle, Wiss. Z. Friedrich-Schiller-Univ. Jena, Math.-Naturwiss. Reihe 37: 171, 1988 (Grolle 1988b). Bas.: Macrolejeunea sessiliflora Steph., Sp. Hepat. (Stephani) 5: 512, 1914 (Stephani 1914b).

** Lejeunea setacea (Steph.) Steph., Sp. Hepat. (Stephani) 5: 719, 1914 (Stephani 1914b). Bas.: Eulejeunea setacea Steph., Bull. Mus. Natl. Hist. Nat. 18 (2): 120, 1912 (Corbière 1912).

** Lejeunea setiloba Spruce, Trans. & Proc. Bot. Soc. Edinburgh 15: 281, 1884 (Spruce 1884).

** Lejeunea sharpii (R.M.Schust.) R.M.Schust., Hepat. Anthocerotae N. Amer. 4: 991, 1980 (Schuster 1980c). Bas.: Taxilejeunea sharpii R.M.Schust., J. Elisha Mitchell Sci. Soc. 81 (1): 41, 1965 (Schuster 1965a).

** Lejeunea siccata Spruce, Trans. & Proc. Bot. Soc. Edinburgh 15: 284, 1884 (Spruce 1884).

** Lejeunea silvatica Gottsche, Ann. Sci. Nat. Bot. (sér. 5) 1: 159 (65), 1864 (Gottsche 1864).

** Lejeunea smaragdina Besch. et Spruce, Bull. Soc. Bot. France (Congr. Bot.) 36: clxxxii, 1889 [1890] (Bescherelle and Spruce 1889).

*** Lejeunea soae R.L.Zhu, Y.M.Wei, L.Söderstr., A.Hagborg et von Konrat, Phytotaxa 81 (1): 1, 2013 (Zhu et al. 2013). *Nom. nov. pro Trachylejeunea chinensis* Herzog, Symb. Sin. 5: 49, 1930 (Nicholson et al. 1930).

** Lejeunea solanicola Spruce, Trans. & Proc. Bot. Soc. Edinburgh 15: 280, 1884 (Spruce 1884).

*** Lejeunea sordida (Nees) Nees, Naturgesch. Eur. Leberm. 3: 278, 1838 (Nees 1838b). Bas.: Jungermannia sordida Nees, Enum. Pl. Crypt. Javae: 41, 1830 (Nees 1830).

*** Lejeunea spiniloba Lindenb. et Gottsche, Syn. Hepat. 5: 770, 1847 (Gottsche et al. 1847).

** Lejeunea spinuliflora Spruce, Trans. & Proc. Bot. Soc. Edinburgh 15: 177, 1884 (Spruce 1884).

** Lejeunea sporadica Besch. et Spruce, Bull. Soc. Bot. France (Congr. Bot.) 36: clxxx, 1889 [1890] (Bescherelle and Spruce 1889).

* Lejeunea squarrosa (Steph.) Steph., Sp. Hepat. (Stephani) 5: 719, 1915 (Stephani 1915b). Bas.: Eulejeunea squarrosa Steph., Bot. Jahrb. Syst. 20 (3): 317, 1895 (Stephani 1895a).

** Lejeunea squarrosula (Herzog) Solari, J. Hattori Bot. Lab. 54: 543, 1983 (Solari 1983a). Bas.: Strepsilejeunea squarrosula Herzog, Nat. Hist. Juan Fernandez (Botany) 2 (5): 741, 1942 (Herzog 1942a).

* Lejeunea stephaniana Mizut., J. Hattori Bot. Lab. 27: 143, 1964 (Mizutani 1964a). *Nom. nov. pro Strepsilejeunea heterophylla* Steph., Sp. Hepat. (Stephani) 6: 395, 1923 (Stephani 1923). ^324^

** Lejeunea stevensiana (Steph.) Mizut., J. Hattori Bot. Lab. 34: 452, 1971 (Mizutani 1971a). Bas.: Taxilejeunea stevensiana Steph., Hedwigia 35 (3): 136, 1896 (Stephani 1896b).

** Lejeunea subacuta Mitt., J. Proc. Linn. Soc., Bot. 5 (18): 113, 1860 [1861] (Mitten 1860c).

* Lejeunea subaquatica Schiffn., Arch. Hydrobiol., suppl. 21 (3/4): 397, 1955 (Schiffner 1955).

* Lejeunea subbifida Steph. ex Duss, Enum. musc. Antilles franç., Hép.: 10, 1903 (Duss 1903). *Nom. nov. pro Odontolejeunea subbifida* Steph., Symb. Antill. (Urban) 3 (2): 277, 1902 (Stephani 1902e), *nom. illeg*.

** Lejeunea subigiensis (Steph.) Steph., Hedwigia 35 (3): 94, 1896 (Stephani 1896b). Bas.: Eulejeunea subigiensis Steph., Hedwigia 35 (3): 94, 1896 (Stephani 1896b).

** Lejeunea subrufula Spruce, Trans. & Proc. Bot. Soc. Edinburgh 15: 289, 1884 (Spruce 1884).

** Lejeunea subsessilis Spruce, Trans. & Proc. Bot. Soc. Edinburgh 15: 282, 1884 (Spruce 1884).

*** Lejeunea subspathulata Spruce, Trans. & Proc. Bot. Soc. Edinburgh 15: 173, 1884 (Spruce 1884).

** Lejeunea succulenta Herzog, Rev. Bryol. Lichénol. 20 (1/2): 171, 1951 [1952] (Herzog 1951a).

** Lejeunea suffruticola Spruce, Trans. & Proc. Bot. Soc. Edinburgh 15: 279, 1884 (Spruce 1884).

** Lejeunea syoshii Inoue, Bull. Natl. Sci. Mus. Tokyo, B 3 (4): 143, 1977 (Inoue 1977b).

*** Lejeunea talamancensis M.E.Reiner et Schäf.-Verw., Nova Hedwigia 87 (3/4): 414, 2008 (Reiner-Drehwald and Schäfer-Verwimp 2008).

*** Lejeunea tamasii M.E.Reiner, N.Salazar et C.Chung, Polish Bot. J. 58 (2): 420, 2013 (Reiner-Drehwald et al. 2013).

** Lejeunea tamaspocsii G.E.Lee, Polish Bot. J. 58 (1): 66, 2013 (Lee and Gradstein 2013).

*** Lejeunea tapajosensis Spruce, Trans. & Proc. Bot. Soc. Edinburgh 15: 223, 1884 (Spruce 1884).

** Lejeunea tarapotensis Spruce, Trans. & Proc. Bot. Soc. Edinburgh 15: 282, 1884 (Spruce 1884).

** Lejeunea tenella Taylor, London J. Bot. 5: 398, 1846 (Taylor 1846b).

** Lejeunea thallophora (Eifrig) Gradst., Phytotaxa 9: 54, 2010 (Söderström et al. 2010a). Bas.: Taxilejeunea thallophora Eifrig, Monogr. Stud. Indomal. Art. Taxilejeunea: 99, 1937 (Eifrig 1937).

*** Lejeunea tonduzana (Steph.) M.E.Reiner, Polish Bot. J. 58 (2): 421, 2013 (Reiner-Drehwald et al. 2013). Bas.: Hygrolejeunea tonduzana Steph., Hedwigia 35 (3): 105, 1896 (Stephani 1896b).

** Lejeunea trachygona Spruce, Trans. & Proc. Bot. Soc. Edinburgh 15: 175, 1884 (Spruce 1884).

*** Lejeunea trinitensis Lindenb., Syn. Hepat. 3: 381, 1845 (Gottsche et al. 1845b).

** Lejeunea trukensis H.A.Mill. et Bonner, Beih. Nova Hedwigia 11: 56, 1963 (Miller et al. 1963).

** Lejeunea tuberculosa Steph., Sp. Hepat. (Stephani) 5: 790, 1915 (Stephani 1915b).

** Lejeunea tumida Mitt., Bot. antarct. voy. II (Fl. Nov.-Zel. 2): 157, 1854 (Mitten 1854).

** Lejeunea uleana (Steph.) Steph., Sp. Hepat. (Stephani) 5: 765, 1915 (Stephani 1915b). Bas.: Eulejeunea uleana Steph., Hedwigia 35 (3): 94, 1896 (Stephani 1896b).

*** Lejeunea umbilicata (Nees) Nees, Observ. bot.: 472, 1843 (Gottsche et al. 1843). Bas.: Jungermannia umbilicata Nees, Enum. Pl. Crypt. Javae: 42, 1830 (Nees 1830).

** Lejeunea utriculata (Steph.) Mizut., J. Hattori Bot. Lab. 33: 242, 1970 (Mizutani 1970). Bas.: Pycnolejeunea utriculata Steph., Hedwigia 35 (3): 126, 1896 (Stephani 1896b).

** Lejeunea vesicata Mitt., Fl. vit.: 415, 1871 [1873] (Mitten 1871).

*** Lejeunea villaumei (Steph.) Grolle, J. Bryol. 9 (4): 536, 1977 [1978] (Grolle 1977a). Bas.: Otigoniolejeunea villaumei Steph., Sp. Hepat. (Stephani) 5: 516, 1914 (Stephani 1914b).

** Lejeunea vojtkoi Pócs, Beih. Nova Hedwigia 138: 107, 2010 (Pócs 2010b).

** Lejeunea vulgariformis Gottsche, Abh. Naturwiss. Vereins Bremen 7: 355, 1882 (Gottsche 1882).

** Lejeunea wattsiana (Steph.) H.A.Mill., Bonner et Bischl., Nova Hedwigia 14 (1): 66, 1967 (Miller et al. 1967). Bas.: Microlejeunea wattsiana Steph., Sp. Hepat. (Stephani) 5: 834, 1916 (Stephani 1916b).

* Lejeunea wichurae (Steph.) Steph., Sp. Hepat. (Stephani) 5: 792, 1915 (Stephani 1915b). Bas.: Eulejeunea wichurae Steph., Hedwigia 35 (3): 94, 1896 (Stephani 1896b).

** Lejeunea wightii Lindenb., Syn. Hepat. 3: 379, 1845 (Gottsche et al. 1845b).

*** Lejeunea xiphophylla (Herzog) M.E.Reiner, Nova Hedwigia 87 (3/4): 409, 2008 (Reiner-Drehwald and Schäfer-Verwimp 2008). Bas.: Taxilejeunea xiphophylla Herzog, Svensk Bot. Tidskr. 46 (1): 99, 1952 (Herzog 1952e).

** **subg. Nanolejeunea R.M.Schust.**, Hepat. Anthocerotae N. Amer. 4: 1092, 1980 (Schuster 1980c).

** Lejeunea curviloba Steph., Sp. Hepat. (Stephani) 5: 774, 1915 (Stephani 1915b).

*** Lejeunea laetevirens Nees et Mont., Hist. Phys. Cuba, Bot., Pl. Cell.: 469, 1842 (Montagne 1842a).

** Lejeunea pallidevirens S.Hatt., J. Hattori Bot. Lab. 12: 80, 1954 (Hattori 1954). *Nom. nov. pro* Microlejeunea rotundistipula var. pallida S.Hatt., J. Hattori Bot. Lab. 5: 53, 1951 (Hattori 1951d).

** Lejeunea parva (S.Hatt.) Mizut., Misc. Bryol. Lichenol. 5 (10/12): 178, 1971 (Mizutani 1971b). Bas.: Microlejeunea rotundistipula f. parva S.Hatt., Bull. Tokyo Sci. Mus. 11: 123, 1944 (Hattori 1944d).

*** Lejeunea prionoides Spruce, Trans. & Proc. Bot. Soc. Edinburgh 15: 238, 1884 (Spruce 1884).

*** Lejeunea ramulosa Spruce, Trans. & Proc. Bot. Soc. Edinburgh 15: 274, 1884 (Spruce 1884).

*** **subg. Neopotamolejeunea (M.E.Reiner) Gradst. et M.E.Reiner**, Syst. Bot. 32 (3): 487, 2007 (Gradstein and Reiner-Drehwald 2007). Bas.: Neopotamolejeunea M.E.Reiner, Nova Hedwigia 71 (3/4): 449, 2000 (Reiner-Drehwald 2000).

** Lejeunea juruana Gradst. et M.E.Reiner, Syst. Bot. 32 (3): 488, 2007 (Gradstein and Reiner-Drehwald 2007). *Nom. nov. pro Potamolejeunea uleana* Steph., Sp. Hepat. (Stephani) 5: 641, 1914 (Stephani 1914b).

*** Lejeunea polyantha Mont., Ann. Sci. Nat. Bot. (sér. 4) 5: 350, 1856 (Montagne 1856c).

** Lejeunea tenera (Sw.) Gottsche, Lindenb. et Nees, Syn. Hepat. 3: 406, 1845 (Gottsche et al. 1845b). Bas.: Jungermannia tenera Sw., Prodr. (Swartz): 143, 1788 (Swartz 1788).

*** Lejeunea topoensis Gradst. et M.E.Reiner, Syst. Bot. 32 (3): 488, 2007 (Gradstein and Reiner-Drehwald 2007).

** **subg. Papillolejeunea (Pócs) R.M.Schust.**, J. Hattori Bot. Lab. 85: 84, 1998 (Schuster 1998a). Bas.: Papillolejeunea Pócs, Trop. Bryol. 13: 2, 1997 (Pócs 1997b).

** Lejeunea balazsii (Pócs) R.M.Schust., J. Hattori Bot. Lab. 85: 84, 1998 (Schuster 1998a). Bas.: Papillolejeunea balazsii Pócs, Trop. Bryol. 13: 3, 1997 (Pócs 1997b).

** Lejeunea candida (Pócs) R.M.Schust., J. Hattori Bot. Lab. 85: 84, 1998 (Schuster 1998a). Bas.: Papillolejeunea candida Pócs, Trop. Bryol. 13: 8, 1997 (Pócs 1997b).

** Lejeunea falcata (Pócs et J.Eggers) Pócs, Phytotaxa 208 (1): 99, 2015 (Pócs et al. 2015a). Bas.: Papillolejeunea falcata Pócs et J.Eggers, Bryobrothera 5: 163, 1999 (Pócs and Eggers 1999).

** Lejeunea koponenii (Pócs et J.Eggers) Pócs, Phytotaxa 208 (1): 99, 2015 (Pócs et al. 2015a). Bas.: Papillolejeunea koponenii Pócs et J.Eggers, Bryobrothera 5: 159, 1999 (Pócs and Eggers 1999).

** Lejeunea papuana (Pócs) R.M.Schust., J. Hattori Bot. Lab. 85: 84, 1998 (Schuster 1998a). Bas.: Papillolejeunea papuana Pócs, Trop. Bryol. 13: 14, 1997 (Pócs 1997b).

** Lejeunea touwii (Pócs) R.M.Schust., J. Hattori Bot. Lab. 85: 84, 1998 (Schuster 1998a). Bas.: Papillolejeunea touwii Pócs, Trop. Bryol. 13: 11, 1997 (Pócs 1997b).

***Incertae sedis***

* Lejeunea apiahyna (Steph.) Sushil K.Singh, Phytotaxa 96 (1): 63, 2013 (Singh 2013). Bas.: Otigoniolejeunea apiahyna Steph., Sp. Hepat. (Stephani) 5: 514, 1914 (Stephani 1914b).

* Lejeunea aptycta Gottsche, Syn. Hepat. 3: 369, 1845 (Gottsche et al. 1845b).

** Lejeunea asthenica Spruce, Trans. & Proc. Bot. Soc. Edinburgh 15: 222, 1884 (Spruce 1884).

* Lejeunea bethanica Gottsche, Syn. Hepat. 3: 381, 1845 (Gottsche et al. 1945b)

** Lejeunea bombonasensis Spruce, Trans. & Proc. Bot. Soc. Edinburgh 15: 222, 1884 (Spruce 1884).

** Lejeunea bornmuelleri (Steph.) M.E.Reiner, Nova Hedwigia 95 (3/4): 471, 2012 (Reiner-Drehwald and Grolle 2012). Bas.: Rectolejeunea bornmuelleri Steph., Sp. Hepat. (Stephani) 5: 682, 1914 (Stephani 1914b).

** Lejeunea caracensis Lindenb., Syn. Hepat. 3: 355, 1845 (Gottsche et al. 1845b).

** Lejeunea caripensis Lindenb. et Gottsche, Syn. Hepat. 5: 758, 1847 (Gottsche et al. 1847).

** Lejeunea chamissonis Lindenb., Syn. Hepat. 3: 378, 1845 (Gottsche et al. 1845b).

** Lejeunea chimborazensis Spruce, Trans. & Proc. Bot. Soc. Edinburgh 15: 215, 1884 (Spruce 1884).

*** Lejeunea combuensis O.S.Moura, Ilk.-Borg. et M.E.Reiner, Nova Hedwigia 95 (1/2): 198, 2012 (Moura et al. 2012).

** Lejeunea concava Lindenb. et Gottsche, Syn. Hepat. 5: 759, 1847 (Gottsche et al. 1847).

*** Lejeunea conformis Nees et Mont., Ann. Sci. Nat. Bot. (sér. 2) 19: 260, 1843 (Montagne 1843).

** Lejeunea cordistipula Lindenb. et Gottsche, Syn. Hepat. 5: 758, 1847 (Gottsche et al. 1847).

** Lejeunea cyrtotis Spruce, Trans. & Proc. Bot. Soc. Edinburgh 15: 229, 1884 (Spruce 1884).

*** Lejeunea debilis (Lehm. et Lindenb.) Nees et Mont., Ann. Sci. Nat. Bot. (sér. 2) 5: 60, 1836 (Nees and Montagne 1836). Bas.: Jungermannia debilis Lehm. et Lindenb., Nov. Stirp. Pug. 4: 51, 1832 (Lehmann 1832).

** Lejeunea devendrae (Sushil K.Singh) P.K.Verma et K.K.Rawat, J. Bryol. 36 (2): 161, 2014 (Verma and Rawat 2014). Bas.: Rectolejeunea devendrae Sushil K.Singh, Indian J. Forest. 34 (3): 341, 2011 (Singh 2011).

** Lejeunea dictyocalyx Spruce, Trans. & Proc. Bot. Soc. Edinburgh 15: 218, 1884 (Spruce 1884).

** Lejeunea dipterota (Eifrig) G.E.Lee, Polish Bot. J. 58 (1): 61, 2013 (Lee and Gradstein 2013). Bas.: Taxilejeunea dipterota Eifrig, Monogr. Stud. Indomal. Art. Taxilejeunea: 96, 1937 (Eifrig 1937).

*** Lejeunea drehwaldii Heinrichs et Schäf.-Verw., Phytotaxa 69: 14, 2012 (Heinrichs et al. 2012b). *Nom. nov. pro Sphaerolejeunea umbilicata* Herzog, Ann. Bryol. 11: 88, 1938 (Herzog 1938b).

** Lejeunea duncaniae (Sim) M.E.Reiner, Phytotaxa 208 (1): 98, 2015 (Pócs et al. 2015a). Bas.: Stylolejeunea duncaniae Sim, Trans. Roy. Soc. South Africa 15 (1): 68, 1926 (Sim 1926).

** Lejeunea edentata L.Söderstr., Phytotaxa 208 (1): 98, 2015 (Pócs et al. 2015a). *Nom. nov. pro Cyclolejeunea marginata* R.M.Schust., Phytologia 39 (6): 430, 1978 (Schuster 1978b).

* Lejeunea emarginuliflora Gottsche ex Steph., Sp. Hepat. (Stephani) 5: 734, 1915 (Stephani 1915b). ^325^

* Lejeunea epibrya Taylor, London J. Bot. 7: 199, 1848 (Taylor 1848a).

** Lejeunea estrellamontana M.A.M.Renner et Pócs, Phytotaxa 81 (1): 9, 2013 (Renner et al. 2013d). *Nom. nov. pro Stenolejeunea fissistipula* R.M.Schust., J. Hattori Bot. Lab. 89: 167, 2000 (Schuster 2000b).

* Lejeunea fawcettiae D.J.Carr, Proc. Roy. Soc. Victoria 117 (2): 325, 2005 (Carr 2005). Based on: Lejeunea fawcettiae D.J.Carr, Proc. Roy. Soc. Victoria 116 (2): 229, 2004 (Carr 2004), *nom. inval*.

** Lejeunea flaccida Lindenb. et Gottsche, Syn. Hepat. 5: 758, 1847 (Gottsche et al. 1847).

** Lejeunea flavida Mitt., J. Proc. Linn. Soc., Bot. 5 (18): 113, 1860 [1861] (Mitten 1860c).

** Lejeunea florida Spruce, Trans. & Proc. Bot. Soc. Edinburgh 15: 221, 1884 (Spruce 1884).

** Lejeunea fulva Spruce, Trans. & Proc. Bot. Soc. Edinburgh 15: 237, 1884 (Spruce 1884).

** Lejeunea gottscheana Lindenb., Syn. Hepat. 3: 382, 1845 (Gottsche et al. 1845b).

** Lejeunea graminicolor Spruce, J. Linn. Soc., Bot. 30 (210): 343, 1895 (Gepp 1895b).

*** Lejeunea grolleana (Bernecker) R.L.Zhu et W.Ye, J. Syst. Evol. 51 (4): 472, 2013 (Ye et al. 2013b). Bas.: Oryzolejeunea grolleana Bernecker, Haussknechtia, Beih. 9: 37, 1999 (Bernecker-Lücking 1999).

*** Lejeunea herminieri (Steph.) R.L.Zhu, Phytotaxa 208 (1): 99, 2015 (Pócs et al. 2015a). Bas.: Archilejeunea herminieri Steph., Sp. Hepat. (Stephani) 4: 714, 1911 (Stephani 1911e).

** Lejeunea heterocheila Taylor, London J. Bot. 5: 394, 1846 (Taylor 1846b).

* Lejeunea hygrophila Gottsche, Ann. Sci. Nat. Bot. (sér. 5) 1: 157, 1864 (Gottsche 1864).

** Lejeunea laevicalyx Gottsche, Mexik. Leverm.: 221, 1863 (Gottsche 1863).

* Lejeunea laxa (Nees) Lindenb., Syn. Hepat. 3: 378, 1845 (Gottsche et al. 1845b). Bas.: Jungermannia thymifolia δ laxa Nees, Enum. Pl. Crypt. Javae: 43, 1830 (Nees 1830).

** Lejeunea leiantha Spruce, J. Linn. Soc., Bot. 30 (210): 345, 1895 (Gepp 1895b).

** Lejeunea leptoscypha Spruce, Bull. Soc. Bot. France (Congr. Bot.) 36: ccv, 1889 [1890] (Spruce 1889).

*** Lejeunea lusoria (Lindenb. et Gottsche) Steph., Hedwigia 29 (3): 141, 1890 (Stephani 1890d). Bas.: Omphalanthus lusorius Lindenb. et Gottsche, Syn. Hepat. 5: 747, 1847 (Gottsche et al. 1847).

** Lejeunea luzonensis (Steph.) R.L.Zhu et M.J.Lai, Ann. Bot. Fenn. 48 (5): 376, 2011 (Wang et al. 2011). Bas.: Taxilejeunea luzonensis Steph., Hedwigia 35 (3): 134, 1896 (Stephani 1896b).

** Lejeunea macrorhyncha Spruce, Trans. & Proc. Bot. Soc. Edinburgh 15: 220, 1884 (Spruce 1884).

** Lejeunea malangensis (Herzog) R.L.Zhu et Y.M.Wei, J. Bryol. 34 (4): 319, 2012 (Zhu and Wei 2012). Bas.: Trachylejeunea malangensis Herzog, Mitt. Inst. Allg. Bot. Hamburg 7 (3): 203, 1931 (Herzog 1931a).

** Lejeunea marasmodes Spruce, Mem. Torrey Bot. Club 1 (3): 125, 1890 (Spruce 1890).

** Lejeunea morobensis (Grolle) M.A.M.Renner et Pócs, Phytotaxa 81 (1): 9, 2013 (Renner et al. 2013d). Bas.: Stenolejeunea morobensis Grolle, J. Hattori Bot. Lab. 29: 76, 1966 (Grolle 1966e).

** Lejeunea novoguineensis Schiffn., Nova Acta Acad. Caes. Leop.-Carol. German. Nat. Cur. 60 (2): 238, 1893 (Schiffner 1893a).

*** Lejeunea obtusangula Spruce, Trans. & Proc. Bot. Soc. Edinburgh 15: 221, 1884 (Spruce 1884).

* Lejeunea oerstediana Lindenb. et Hampe, Linnaea 24 (6): 641, 1851 [1852] (Hampe 1851a). ^326^

** Lejeunea paraensis Spruce, Trans. & Proc. Bot. Soc. Edinburgh 15: 224, 1884 (Spruce 1884).

** Lejeunea parviloba Ångstr., Öfvers. Kongl. Vetensk.-Akad. Förh. 33 (7): 87, 1876 [1877] (Ångström 1876).

* Lejeunea pfleidereri Sushil K.Singh, Phytotaxa 96 (1): 63, 2013 (Singh 2013). *Nom. nov. pro Otigoniolejeunea indica* Steph., Sp. Hepat. (Stephani) 6: 408, 1923 (Stephani 1923).

* Lejeunea prominula Gottsche, Ann. Sci. Nat. Bot. (sér. 5) 1: 157, 1864 (Gottsche 1864).

*** Lejeunea pterigonia (Lehm. et Lindenb.) Mont., Ann. Sci. Nat. Bot. (sér. 2) 14: 337, 1840 (Montagne 1840a). Bas.: Jungermannia pterigonia Lehm. et Lindenb., Nov. Stirp. Pug. 6: 44, 1834 (Lehmann 1834).

** Lejeunea quinqueumbonata Spruce, Trans. & Proc. Bot. Soc. Edinburgh 15: 230, 1884 (Spruce 1884).

** Lejeunea quinqueumbonata var. rotundata (Herzog) Sushil K.Singh, Phytotaxa 96 (1): 64, 2013 (Singh 2013). Bas.: Otigoniolejeunea quinqueumbonata var. rotundata Herzog, Rev. Bryol. Lichénol. 20 (1/2): 154, 1951 [1952] (Herzog 1951a).

** Lejeunea remotifolia Hampe ex Steph., Sp. Hepat. (Stephani) 5: 756, 1915 (Stephani 1915b).

*** Lejeunea ruthii (A.Evans) R.M.Schust., J. Hattori Bot. Lab. 25: 23, 1962 (Schuster 1962a). Bas.: Microlejeunea ruthii A.Evans, Mem. Torrey Bot. Club 8 (2): 161, 1902 (Evans 1902a).

** Lejeunea ruthii var. alata R.M.Schust., Hepat. Anthocerotae N. Amer. 4: 1063, 1980 (Schuster 1980c).

** Lejeunea sikorae (Steph.) Steph., Bull. Soc. Roy. Bot. Belgique, Mém. 32: 120, 1893 [1894] (Renauld and Cardot 1893). Bas.: Taxilejeunea sikorae Steph., Bull. Soc. Roy. Bot. Belgique, Compt. Rend. 32 (2): 34, 1893 [1894] (Stephani 1893e).

** Lejeunea srivastavae P.K.Verma et K.K.Rawat, Taiwania 58 (1): 8, 2013 (Verma and Rawat 2013).

** Lejeunea stenodentata M.A.M.Renner et Pócs, Phytotaxa 81 (1): 8, 2013 (Renner et al. 2013d). *Nom. nov. pro Drepanolejeunea dentata* Steph., Hedwigia 35 (3): 82, 1896 (Stephani 1896b).

*** Lejeunea subelobata Carrington et Pearson, Proc. Linn. Soc. New South Wales (ser. 2) 2 (4): 1039, 1888 (Carrington and Pearson 1888a).

** Lejeunea subolivacea Mizut., J. Hattori Bot. Lab. 28: 121, 1965 (Mizutani 1965). *Nom. nov. pro Eulejeunea olivacea* Steph., Hedwigia 29 (2): 85, 1890 (Stephani 1890b).

** Lejeunea subplana (Steph.) C.J.Bastos, J. Bryol. 36 (3): 249, 2014 (Bastos 2014). Bas.: Trachylejeunea subplana Steph., Sp. Hepat. (Stephani) 5: 310, 1913 (Stephani 1913a).

*** Lejeunea sulphurea (Lehm. et Lindenb.) Spruce, Trans. & Proc. Bot. Soc. Edinburgh 15: 217, 1884 (Spruce 1884). Bas.: Jungermannia sulphurea Lehm. et Lindenb., Nov. Stirp. Pug. 5: 14, 1833 (Lehmann 1833).

** Lejeunea terricola Spruce, Bull. Soc. Bot. France (Congr. Bot.) 36: cxci, 1889 [1890] (Spruce 1889).

** Lejeunea urbanii (Steph.) Steph., Sp. Hepat. (Stephani) 5: 766, 1915 (Stephani 1915b). Bas.: Eulejeunea urbanii Steph., Hedwigia 27 (11/12): 301, 1888 (Stephani 1888b).

** Lejeunea venezuelana (R.M.Schust.) R.L.Zhu et W.Ye, J. Syst. Evol. 51 (4): 473, 2013 (Ye et al. 2013b). Bas.: Cyrtolejeunea venezuelana R.M.Schust., Phytologia 39 (6): 426, 1978 (Schuster 1978b).

** Lejeunea viridis R.M.Schust. ex L.Söderstr. et A.Hagborg, Phytotaxa 208 (1): 99, 2015 (Pócs et al. 2015a). Based on: Prionocolea viridissima R.M.Schust., J. Hattori Bot. Lab. 75: 215, 1994 (Schuster 1994), *nom. inval*.

* Lejeunea zacuapana (Steph.) Prantl, Hedwigia 29: xviii, 1890 (Prantl 1890). Bas.: Eulejeunea zacuapana Steph., Hedwigia 29 (2): 87, 1890 (Stephani 1890b).

**Excluded from the genus**

* Lejeunea elegans Gottsche, Syn. Hepat. 3: 364, 1845 (Gottsche et al. 1845b). ^327^

* Lejeunea hieronymii Spruce, Bull. Soc. Bot. France (Congr. Bot.) 36: cciii, 1889 [1890] (Spruce 1889). ^328^

* Lejeunea proboscidea Gottsche, Mexik. Leverm.: 225, 1863 (Gottsche 1863). ^329^

* Lejeunea scabriflora Loitl., Diagn. pl. nov.: 22, 1894 (Szyszylowicz 1894). ^330^

* Lejeunea trochantha Spruce, Bull. Soc. Bot. France (Congr. Bot.) 36: cxcii, 1889 [1890] (Spruce 1889). ^331^

** **Microlejeunea (Spruce) Steph.**, Hedwigia 27 (2): 61, 1888 (Stephani 1888a). Bas.: Lejeunea subg. Microlejeunea Spruce, Trans. & Proc. Bot. Soc. Edinburgh 15: 286, 1884 (Spruce 1884).

*** Microlejeunea acutifolia Steph., Hedwigia 35 (3): 113, 1896 (Stephani 1896b).

** Microlejeunea africana Steph., Hedwigia 27 (2): 61, 1888 (Stephani 1888a).

** Microlejeunea aligera (Mitt.) Steph., Sp. Hepat. (Stephani) 5: 827, 1915 (Stephani 1915b). Bas.: Lejeunea aligera Mitt., J. Proc. Linn. Soc., Bot. 5 (18): 113, 1860 [1861] (Mitten 1860c).

** Microlejeunea ankasica E.W.Jones, J. Bryol. 10 (4): 394, 1979 (Jones 1979).

** Microlejeunea aphanella (Spruce) Steph., Sp. Hepat. (Stephani) 5: 816, 1915 (Stephani 1915b). Bas.: Lejeunea aphanella Spruce, Trans. & Proc. Bot. Soc. Edinburgh 15: 290, 1884 (Spruce 1884).

** Microlejeunea atsuana Steph., Hedwigia 35 (3): 113, 1896 (Stephani 1896b).

** Microlejeunea bischlerae (B.M.Thiers) B.M.Thiers, Phytotaxa 65: 59, 2012 (Thiers et al. 2012). Bas.: Lejeunea bischlerae B.M.Thiers, Cryptog. Bryol. Lichénol. 18 (3): 223, 1997 (Thiers 1997b).

*** Microlejeunea bullata (Taylor) Steph., Hedwigia 29 (2): 90, 1890 (Stephani 1890b). Bas.: Lejeunea bullata Taylor, London J. Bot. 5: 398, 1846 (Taylor 1846b).

* Microlejeunea byssoides (Gottsche) Pearson, Forh. Vidensk.-Selsk. Kristiania 1892 (8): 9, 1892 (Pearson 1892). Bas.: Lejeunea byssoides Gottsche, Abh. Naturwiss. Vereins Bremen 7: 358, 1882 (Gottsche 1882).

** Microlejeunea capillaris (Gottsche) Steph., Sp. Hepat. (Stephani) 5: 819, 1915 (Stephani 1915b). Bas.: Lejeunea capillaris Gottsche, Ann. Sci. Nat. Bot. (sér. 5) 1: 163, 1864 (Gottsche 1864).

** Microlejeunea cochlearifolia Steph., Hedwigia 27 (3/4): 113, 1888 (Stephani 1888d). ^332^

** Microlejeunea colombiana Bischl., Nova Hedwigia 5 (1/2): 373, 1963 (Bischler et al. 1963).

** Microlejeunea constricta (Grolle) Grolle, J. Bryol. 21 (1): 41, 1999 (Grolle and Reiner-Drehwald 1999). Bas.: Harpalejeunea constricta Grolle, Nova Hedwigia 16: 150, 1968 (Grolle 1968d).

** Microlejeunea crenulifolia (Gottsche) Steph., Hedwigia 35 (3): 114, 1896 (Stephani 1896b). Bas.: Lejeunea crenulifolia Gottsche, Mexik. Leverm.: 227, 1863 (Gottsche 1863).

*** Microlejeunea cystifera Herzog, Memoranda Soc. Fauna Fl. Fennica 25: 68, 1950 (Herzog 1950c).

** Microlejeunea dispar Jovet-Ast, Rev. Bryol. Lichénol. 27 (3/4): 191, 1959 (Jovet-Ast 1959).

*** Microlejeunea epiphylla Bischl., Nova Hedwigia 5 (1/2): 378, 1963 (Bischler et al. 1963).

*** Microlejeunea filicuspis (Steph.) Heinrichs, Schäf.-Verw., Pócs et S.Dong, Phytotaxa 85 (2): 52, 2013 (Dong et al. 2013). Bas.: Drepanolejeunea filicuspis Steph., Sp. Hepat. (Stephani) 5: 344, 1913 (Stephani 1913a).

*** Microlejeunea fischeri (Tixier) Heinrichs, Schäf.-Verw., Pócs et S.Dong, Phytotaxa 85 (2): 52, 2013 (Dong et al. 2013). Bas.: Harpalejeunea fischeri Tixier, Trop. Bryol. 11: 29, 1995 (Tixier 1995b).

** Microlejeunea fissistipula Steph., Sp. Hepat. (Stephani) 5: 810, 1915 (Stephani 1915b).

*** Microlejeunea globosa (Spruce) Steph., Sp. Hepat. (Stephani) 5: 821, 1915 (Stephani 1915b). Bas.: Lejeunea globosa Spruce, Bull. Soc. Bot. France (Congr. Bot.) 36: cxciii, 1889 [1890] (Spruce 1889).

** Microlejeunea herzogiana Steph., Biblioth. Bot. 87 (2): 266, 1916 (Stephani 1916a).

** Microlejeunea indica (Udar et U.S.Awasthi) Y.M.Wei et R.L.Zhu, Phytotaxa 97 (2): 63, 2013 (Wei and Zhu 2013b). Bas.: Lejeunea indica Udar et U.S.Awasthi, Cryptog. Bryol. Lichénol. 2 (3): 345, 1981 (Udar and Awasthi 1981).

** Microlejeunea inflata Steph., Sp. Hepat. (Stephani) 5: 811, 1915 (Stephani 1915b).

** Microlejeunea kinabaluensis (Mizut.) Grolle, J. Bryol. 21 (1): 42, 1999 (Grolle and Reiner-Drehwald 1999). Bas.: Harpalejeunea kinabaluensis Mizut., J. Hattori Bot. Lab. 37: 200, 1973 (Mizutani 1973).

*** Microlejeunea latitans (Hook.f. et Taylor) Heinrichs, Schäf.-Verw., Pócs et S.Dong, Phytotaxa 85 (2): 52, 2013 (Dong et al. 2013). Bas.: Jungermannia latitans Hook.f. et Taylor, London J. Bot. 3: 399, 1844 (Hooker and Taylor 1844a).

** Microlejeunea lunulatiloba Horik., J. Sci. Hiroshima Univ., Ser. B, Div. 2, Bot. 1: 27, 1931 (Horikawa 1931b). ^333^

* Microlejeunea magnilobula Gola, Ann. Bot. (Rome) 6 (2): 274, 1907 (Gola 1907).

** Microlejeunea mammillosa (Mizut.) Grolle, J. Bryol. 21 (1): 42, 1999 (Grolle and Reiner-Drehwald 1999). Bas.: Harpalejeunea mammillosa Mizut., J. Hattori Bot. Lab. 37: 192, 1973 (Mizutani 1973).

** Microlejeunea minutissima (Mizut.) Grolle, J. Bryol. 21 (1): 42, 1999 (Grolle and Reiner-Drehwald 1999). Bas.: Harpalejeunea minutissima Mizut., J. Hattori Bot. Lab. 37: 202, 1973 (Mizutani 1973).

* Microlejeunea minutistipula Steph., Wiss. Ergebn. Deut. Zentr.-Afr. Exped. (1907–08), Bot. 2: 131, 1911 (Stephani 1911a). ^334^

*** Microlejeunea moniliata (Mizut.) R.L.Zhu et Y.M.Wei, Cryptog. Bryol. 34 (3): 308, 2013 (Wei and Zhu 2013a). Bas.: Lejeunea moniliata Mizut., J. Hattori Bot. Lab. 46: 357, 1979 (Mizutani 1979b).

* Microlejeunea nepalensis Steph., Sp. Hepat. (Stephani) 5: 832, 1915 (Stephani 1915b).

** Microlejeunea nyandaruensis Pócs, Polish Bot. J. 47 (1): 14, 2002 (Pócs 2002c).

** Microlejeunea oblongistipula (Gottsche) Pearson, Forh. Vidensk.-Selsk. Kristiania 1892 (8): 9, 1892 (Pearson 1892). Bas.: Lejeunea oblongistipula Gottsche, Abh. Naturwiss. Vereins Bremen 7: 357, 1882 (Gottsche 1882).

** Microlejeunea ocellata (Herzog) Grolle, Haussknechtia 8: 60, 2001 (Grolle 2001). Bas.: Rectolejeunea ocellata Herzog, Trans. & Proc. Roy. Soc. New Zealand 77 (2): 255, 1949 (Herzog 1949a).

* Microlejeunea ovistipula Steph., Wiss. Ergebn. Deut. Zentr.-Afr. Exped. (1907-08), Bot. 2: 131, 1911 (Stephani 1911a). ^335^

** Microlejeunea papulosa (Gottsche) Pearson, Forh. Vidensk.-Selsk. Kristiania 1892 (8): 9, 1892 (Pearson 1892). Bas.: Lejeunea papulosa Gottsche, Abh. Naturwiss. Vereins Bremen 7: 358, 1882 (Gottsche 1882).

** Microlejeunea perpusilla (Spruce) Steph., Hedwigia 35 (3): 113, 1896 (Stephani 1896b). Bas.: Lejeunea perpusilla Spruce, J. Bot. 19: 36, 1881 (Spruce 1881b).

** Microlejeunea punctiformis (Taylor) Steph., Hedwigia 29 (2): 90, 1890 (Stephani 1890b). Bas.: Lejeunea punctiformis Taylor, London J. Bot. 5: 398, 1846 (Taylor 1846b). ^336^

** Microlejeunea pyriformis (Lindenb. et Gottsche) Steph., Sp. Hepat. (Stephani) 5: 824, 1915 (Stephani 1915b). Bas.: Lejeunea pyriformis Lindenb. et Gottsche, Syn. Hepat. 5: 767, 1847 (Gottsche et al. 1847).

** Microlejeunea spinosa (Mizut.) Grolle, J. Bryol. 21 (1): 43, 1999 (Grolle and Reiner-Drehwald 1999). Bas.: Harpalejeunea spinosa Mizut., J. Hattori Bot. Lab. 37: 196, 1973 (Mizutani 1973).

*** Microlejeunea squarrosa (Steph.) Heinrichs, Schäf.-Verw., Pócs et S.Dong, Phytotaxa 85 (2): 51, 2013 (Dong et al. 2013). Bas.: Strepsilejeunea squarrosa Steph., Hedwigia 35 (3): 130, 1896 (Stephani 1896b).

** Microlejeunea strasbergii Bardat et Ah-Peng, Bryologist 114 (4): 669, 2011 (Ah-Peng and Bardat 2011).

*** Microlejeunea subulistipa Steph., Hedwigia 35 (3): 115, 1896 (Stephani 1896b).

* Microlejeunea szechuanensis P.C.Chen, Feddes Repert. Spec. Nov. Regni Veg. 58: 46, 1955 (Chen 1955). ^337^

** Microlejeunea udarii P.K.Verma et S.C.Srivast., J. Bombay Nat. Hist. Soc. 108 (2): 122, 2011 [2012] (Verma and Srivastava 2011).

*** Microlejeunea ulicina (Taylor) Steph., Hedwigia 29 (2): 88, 1890 (Stephani 1890b). Bas.: Jungermannia ulicina Taylor, Trans. Bot. Soc. Edinburgh 1 (1/4): 115, 1844 (Taylor 1844a).

* Microlejeunea usambarensis Steph., Akad. Wiss. Wien, Math.-Naturwiss. Kl., Denkschr. 88: 730, 1913 (Stephani 1913b).

** Microlejeunea valenciana Steph., Sp. Hepat. (Stephani) 5: 815, 1915 (Stephani 1915b).

* Microlejeunea victoriensis D.J.Carr, Proc. Roy. Soc. Victoria 117 (2): 322, 2005 (Carr 2005).

*** Microlejeunea wallichiana (Lehm.) R.L.Zhu et Y.M.Wei, Cryptog. Bryol. 34 (3): 308, 2013 (Wei and Zhu 2013a). Bas.: Jungermannia wallichiana Lehm., Nov. Stirp. Pug. 3: 5, 1831 (Lehmann 1831).

* **Taxilejeunea (Spruce) Steph.**, Hedwigia 28 (4): 262, 1889 (Stephani 1889c) nom. rejic. Bas.: Lejeunea subg. Taxilejeunea Spruce, Trans. & Proc. Bot. Soc. Edinburgh 15: 212, 1884 (Spruce 1884).

** Taxilejeunea acutifolia (Steph.) Steph., Sp. Hepat. (Stephani) 5: 481, 1914 (Stephani 1914b). Bas.: Dicranolejeunea acutifolia Steph., Hedwigia 35 (3): 76, 1896 (Stephani 1896b).

** Taxilejeunea antillana Steph., Sp. Hepat. (Stephani) 5: 482, 1914 (Stephani 1914b).

** Taxilejeunea apiculata (Gottsche) J.B.Jack et Steph., Hedwigia 31 (1): 13, 1892 (Jack and Stephani 1892). Bas.: Omphalanthus apiculatus Gottsche, Ann. Sci. Nat. Bot. (sér. 5) 1: 149, 1864 (Gottsche 1864).

** Taxilejeunea argentina Steph., Sp. Hepat. (Stephani) 5: 482, 1914 (Stephani 1914b).

** Taxilejeunea arsenii Steph., Sp. Hepat. (Stephani) 6: 400, 1923 (Stephani 1923).

** Taxilejeunea auriculata Steph., Sp. Hepat. (Stephani) 5: 459, 1914 (Stephani 1914b).

** Taxilejeunea berteroana Steph., Sp. Hepat. (Stephani) 5: 483, 1914 (Stephani 1914b).

** Taxilejeunea beyrichiana Steph., Sp. Hepat. (Stephani) 5: 460, 1914 (Stephani 1914b).

** Taxilejeunea biapiculata Steph., Sp. Hepat. (Stephani) 5: 460, 1914 (Stephani 1914b).

* Taxilejeunea boliviana Steph., Biblioth. Bot. 87 (2): 260, 1916 (Stephani 1916a).

** Taxilejeunea brasiliensis Steph., Hedwigia 35 (3): 132, 1896 (Stephani 1896b).

** Taxilejeunea coilantha Herzog, Rev. Bryol. Lichénol. 20 (1/2): 159, 1951 [1952] (Herzog 1951a).

* Taxilejeunea compressiuscula Steph., Sp. Hepat. (Stephani) 5: 501, 1914 (Stephani 1914b).

* Taxilejeunea convoluta Herzog, Biblioth. Bot. 88: 31, 1920 [1921] (Herzog 1920).

* Taxilejeunea cuervi (Gottsche) Steph., Sp. Hepat. (Stephani) 5: 486, 1914 (Stephani 1914b). Bas.: Omphalanthus cuervi Gottsche, Ann. Sci. Nat. Bot. (sér. 5) 1: 147, 1864 (Gottsche 1864).

* Taxilejeunea cuneistipula Steph., Sp. Hepat. (Stephani) 6: 401, 1923 (Stephani 1923).

* Taxilejeunea cuspidata Steph., Biblioth. Bot. 87 (2): 260, 1916 (Stephani 1916a).

** Taxilejeunea decurrens Steph., Sp. Hepat. (Stephani) 5: 487, 1914 (Stephani 1914b).

** Taxilejeunea deflexa Steph., Sp. Hepat. (Stephani) 5: 502, 1914 (Stephani 1914b).

** Taxilejeunea densiflora A.Evans, Bull. Torrey Bot. Club 48 (4): 121, 1921 (Evans 1921b).

** Taxilejeunea diaphana (Lehm.) Steph., Sp. Hepat. (Stephani) 5: 463, 1914 (Stephani 1914b). Bas.: Omphalanthus diaphanus Lehm., Nov. Stirp. Pug. 10: 12, 1857 (Lehmann 1857).

* Taxilejeunea dissitifolia Steph., Symb. Antill. 2: 472, 1901 (Stephani 1901f).

** Taxilejeunea eggersiana Schiffn., Bot. Jahrb. Syst. 23 (5): 579, 1897 (Schiffner 1897). Based on: Taxilejeunea eggersiana Steph., Hedwigia 27 (11/12): 285, 1888 (Stephani 1888c), *nom. inval*.

* Taxilejeunea elobulata Sim, Trans. Roy. Soc. South Africa 15 (1): 66, 1926 (Sim 1926).

** Taxilejeunea fissistipula Steph., Sp. Hepat. (Stephani) 5: 488, 1914 (Stephani 1914b).

** Taxilejeunea foliicola Steph., Sp. Hepat. (Stephani) 5: 466, 1914 (Stephani 1914b).

** Taxilejeunea furcicornuta Grolle, J. Bryol. 8 (1): 93, 1974 (Grolle 1974a).

** Taxilejeunea fuscorufa Steph., Hedwigia 35 (3): 133, 1896 (Stephani 1896b).

** Taxilejeunea galapagensis Onr., Misc. Bryol. Lichenol. 9 (6): 117, 1982 (Onraedt 1982).

* Taxilejeunea ghatensis P.K.Verma et S.C.Srivast., Proc. Natl. Acad. Sci. India, B 77 (2): 211, 2007 (Verma and Srivastava 2007).

* Taxilejeunea giulianettii Steph., Sp. Hepat. (Stephani) 5: 502, 1914 (Stephani 1914b).

** Taxilejeunea gomphocalyx Herzog, Beih. Bot. Centralbl. 61B (3): 580, 1942 (Herzog 1942d).

* Taxilejeunea grandifolia Steph., Biblioth. Bot. 87 (2): 261, 1916 (Stephani 1916a).

* Taxilejeunea grandistipula Steph., Sp. Hepat. (Stephani) 5: 504, 1914 (Stephani 1914b).

* Taxilejeunea hamatifolia Steph., Biblioth. Bot. 87 (2): 261, 1916 (Stephani 1916a).

** Taxilejeunea himalayensis Herzog, Ann. Bryol. 12: 86, 1939 (Herzog 1939b).

* Taxilejeunea immersa Eifrig, Monogr. Stud. Indomal. Art. Taxilejeunea: 96, 1937 (Eifrig 1937).

** Taxilejeunea irregularis Steph., Sp. Hepat. (Stephani) 5: 490, 1914 (Stephani 1914b).

** Taxilejeunea jamaicensis A.Evans, Bull. Torrey Bot. Club 48 (4): 117, 1921 (Evans 1921b).

** Taxilejeunea jeringii Steph., Hedwigia 35 (3): 134, 1896 (Stephani 1896b).

** Taxilejeunea killipii Herzog, Feddes Repert. Spec. Nov. Regni Veg. 57 (1/2): 190, 1955 (Herzog 1955).

* Taxilejeunea laevis (Gottsche) Steph., Hedwigia 44 (4): 229, 1905 (Stephani 1905a). Bas.: Omphalanthus laevis Gottsche, Ann. Sci. Nat. Bot. (sér. 5) 1: 148, 1864 (Gottsche 1864).

* Taxilejeunea langiana Pearson, Ann. Bryol. 4: 102, 1931 (Pearson 1931b).

** Taxilejeunea lindbergiana Steph., Sp. Hepat. (Stephani) 5: 491, 1914 (Stephani 1914b).

** Taxilejeunea linguifolia Steph., Sp. Hepat. (Stephani) 5: 471, 1914 (Stephani 1914b).

* Taxilejeunea maxima Steph., Sp. Hepat. (Stephani) 5: 473, 1914 (Stephani 1914b).

** Taxilejeunea mexicana Steph., Sp. Hepat. (Stephani) 6: 402, 1923 (Stephani 1923).

* Taxilejeunea microstipula Steph., Sp. Hepat. (Stephani) 5: 493, 1914 (Stephani 1914b).

* Taxilejeunea mucronata Steph., Sp. Hepat. (Stephani) 5: 473, 1914 (Stephani 1914b).

** Taxilejeunea multiflora Steph., Hedwigia 35 (3): 135, 1896 (Stephani 1896b).

* Taxilejeunea muscicola Steph., Biblioth. Bot. 87 (2): 263, 1916 (Stephani 1916a).

* Taxilejeunea nilgiriensis P.K.Verma et S.C.Srivast., Proc. Natl. Acad. Sci. India, B 77 (2): 207, 2007 (Verma and Srivastava 2007).

** Taxilejeunea nymannii Steph., Sp. Hepat. (Stephani) 5: 507, 1914 (Stephani 1914b).

** Taxilejeunea obtusifolia Steph., Sp. Hepat. (Stephani) 5: 474, 1914 (Stephani 1914b).

** Taxilejeunea papuliflora Steph., Sp. Hepat. (Stephani) 5: 494, 1914 (Stephani 1914b).

** Taxilejeunea parvibracteata Steph., Hedwigia 35 (3): 136, 1896 (Stephani 1896b).

** Taxilejeunea parvistipula Steph., Sp. Hepat. (Stephani) 6: 403, 1923 (Stephani 1923).

* Taxilejeunea paucidens Steph., Biblioth. Bot. 87 (2): 263, 1916 (Stephani 1916a).

* Taxilejeunea pendula Steph., Biblioth. Bot. 87 (2): 263, 1916 (Stephani 1916a).

* Taxilejeunea peruviana Steph., Sp. Hepat. (Stephani) 5: 475, 1914 (Stephani 1914b).

** Taxilejeunea planilobula Herzog, Rev. Bryol. Lichénol. 20 (1/2): 160, 1951 [1952] (Herzog 1951a).

** Taxilejeunea pulchriflora Pearson, Ark. Bot. 19 (5): 15, 1924 (Pearson 1924b).

* Taxilejeunea pusilla Steph., Biblioth. Bot. 87 (2): 264, 1916 (Stephani 1916a).

** Taxilejeunea renistipula (Lindenb.) Steph., Hedwigia 29 (3): 142, 1890 (Stephani 1890d). Bas.: Omphalanthus renistipulus Lindenb., Syn. Hepat. 3: 308, 1845 (Gottsche et al. 1845b).

* Taxilejeunea rufescens Steph., Biblioth. Bot. 87 (2): 264, 1916 (Stephani 1916a).

*** Taxilejeunea serpillifolioides (Raddi) D.P.Costa, J. Bryol. 31 (4): 230, 2009 (Costa 2009). Bas.: Jungermannia serpillifolioides Raddi, Critt. Brasil.: 17, 1822 (Raddi 1822).

** Taxilejeunea setchellii Pearson, Amer. Samoa: 146, 1924 (Pearson 1924a).

** Taxilejeunea speciosa Herzog, Feddes Repert. Spec. Nov. Regni Veg. 57 (1/2): 188, 1955 (Herzog 1955).

* Taxilejeunea splendida Eifrig, Monogr. Stud. Indomal. Art. Taxilejeunea: 88, 1937 (Eifrig 1937).

** Taxilejeunea stephanii Eifrig, Monogr. Stud. Indomal. Art. Taxilejeunea: 90, 1937 (Eifrig 1937). *Nom. nov. pro Hygrolejeunea nymanii* Steph., Sp. Hepat. (Stephani) 5: 564, 1914 (Stephani 1914b).

** Taxilejeunea steyermarkii H.Rob., Phytologia 34 (1): 67, 1976 (Robinson 1976a).

** Taxilejeunea surinamensis (Lindenb. et Gottsche) Steph., Hedwigia 29 (3): 142, 1890 (Stephani 1890d). Bas.: Omphalanthus surinamensis Lindenb. et Gottsche, Linnaea 24 (6): 628, 1851 [1852] (Lindenberg and Gottsche 1851a).

* Taxilejeunea suringarii Steph., Sp. Hepat. (Stephani) 5: 479, 1914 (Stephani 1914b).

** Taxilejeunea tenerrima Steph., Sp. Hepat. (Stephani) 6: 406, 1923 (Stephani 1923).

** Taxilejeunea tenuiplica Steph., Sp. Hepat. (Stephani) 5: 480, 1914 (Stephani 1914b).

* Taxilejeunea tjibodensis (Steph.) Eifrig, Monogr. Stud. Indomal. Art. Taxilejeunea: 91, 1937 (Eifrig 1937). Bas.: Hygrolejeunea tjibodensis Steph., Sp. Hepat. (Stephani) 5: 571, 1914 (Stephani 1914b).

** Taxilejeunea tonduzana Steph., Sp. Hepat. (Stephani) 5: 498, 1914 (Stephani 1914b).

* Taxilejeunea uleana Steph., Hedwigia 35 (3): 136, 1896 (Stephani 1896b).

* Taxilejeunea umbonata Steph., Sp. Hepat. (Stephani) 5: 481, 1914 (Stephani 1914b).

* Taxilejeunea urbanii Steph., Symb. Antill. (Urban) 3 (2): 278, 1902 (Stephani 1902e).

** Taxilejeunea vallis-gratiae Steph., Hedwigia 35 (3): 137, 1896 (Stephani 1896b).

########### ** subtrib. Lepidolejeuneinae Gradst.

*** **Lepidolejeunea R.M.Schust.**, Beih. Nova Hedwigia 9: 139, 1963 (Schuster 1963a). ^338^

** **subg. Kingiolejeunea (H.Rob.) R.M.Schust.**, Phytologia 45 (5): 425, 1980 (Schuster 1980b). Bas.: Kingiolejeunea H.Rob., Bryologist 70 (1): 53, 1967 (Robinson 1967).

** Lepidolejeunea auriculata Schäf.-Verw. et Heinrichs, Taxon 64 (2): 224, 2015 (Heinrichs et al. 2015).

*** Lepidolejeunea cordifissa (Taylor) M.E.Reiner, Nova Hedwigia 83 (3/4): 478, 2006 (Reiner-Drehwald 2006). Bas.: Lejeunea cordifissa Taylor, London J. Bot. 5: 395, 1846 (Taylor 1846b).

*** Lepidolejeunea grossepapulosa (Steph.) Piippo, Acta Bot. Fenn. 132: 49, 1986 (Piippo 1986a). Bas.: Prionolejeunea grossepapulosa Steph., Sp. Hepat. (Stephani) 5: 220, 1913 (Stephani 1913a).

*** Lepidolejeunea involuta (Gottsche) Grolle, J. Hattori Bot. Lab. 55: 504, 1984 (Grolle 1984a). Bas.: Lejeunea involuta Gottsche, Syn. Hepat. 3: 350, 1845 (Gottsche et al. 1845b).

** **subg. Lepidolejeunea**

*** Lepidolejeunea bidentula (Steph.) R.M.Schust., Phytologia 45 (5): 425, 1980 (Schuster 1980b). Bas.: Pycnolejeunea bidentula Steph., Bot. Centralbl. 60 (4): 107, 1894 (Jack and Stephani 1894).

*** Lepidolejeunea bidentula var. novae-caledoniae Piippo, Acta Bot. Fenn. 132: 26, 1986 (Piippo 1986a).

*** Lepidolejeunea falcata (Herzog) R.M.Schust., Beih. Nova Hedwigia 9: 139, 1963 (Schuster 1963a). Bas.: Hygrolejeunea falcata Herzog, Ark. Bot. (n.ser.) 3 (3): 57, 1953 (Herzog 1953a).

*** Lepidolejeunea graeffei (J.B.Jack et Steph.) R.M.Schust., Phytologia 45 (5): 425, 1980 (Schuster 1980b). Bas.: Archilejeunea graeffei J.B.Jack et Steph., Bot. Centralbl. 60 (4): 104, 1894 (Jack and Stephani 1894).

*** Lepidolejeunea integristipula (J.B.Jack et Steph.) R.M.Schust., Phytologia 45 (5): 425, 1980 (Schuster 1980b). Bas.: Pycnolejeunea integristipula J.B.Jack et Steph., Bot. Centralbl. 60 (4): 107, 1894 (Jack and Stephani 1894).

** **subg. Perilejeunea (Kachroo et R.M.Schust.) R.M.Schust.**, Phytologia 45 (5): 424, 1980 (Schuster 1980b). Bas.: Pycnolejeunea subg. Perilejeunea Kachroo et R.M.Schust., J. Linn. Soc., Bot. 56 (368): 493, 1961 (Kachroo and Schuster 1961).

** Lepidolejeunea cuspidata (Gottsche) Heinrichs et Schäf.-Verw., Taxon 64 (2): 224, 2015 (Heinrichs et al. 2015). Bas.: Lejeunea cuspidata Gottsche, Syn. Hepat. 3: 351, 1845 (Gottsche et al. 1845b).

*** Lepidolejeunea delessertii (Nees et Mont.) Grolle, J. Hattori Bot. Lab. 55: 505, 1984 (Grolle 1984a). Bas.: Lejeunea delessertii Nees et Mont., Ann. Sci. Nat. Bot. (sér. 2) 19: 260, 1843 (Montagne 1843).

*** Lepidolejeunea eluta (Nees) R.M.Schust., Beih. Nova Hedwigia 9: 139, 1963 (Schuster 1963a). Bas.: Jungermannia eluta Nees, Fl. Bras. (Martius) 1 (1): 362, 1833 (Nees 1833a).

*** Lepidolejeunea serrulata (Steph.) Grolle, J. Hattori Bot. Lab. 55: 505, 1984 (Grolle 1984a). Bas.: Trachylejeunea serrulata Steph., Sp. Hepat. (Stephani) 5: 300, 1913 (Stephani 1913a).

*** Lepidolejeunea sullivantii (Gottsche) M.E.Reiner, Nova Hedwigia 83 (3/4): 479, 2006 (Reiner-Drehwald 2006). Bas.: Lejeunea sullivantii Gottsche, Mexik. Leverm.: 196, 1863 (Gottsche 1863).

***Incertae sedis***

*** Lepidolejeunea borneensis (Steph.) R.M.Schust., Phytologia 45 (5): 425, 1980 (Schuster 1980b). Bas.: Hygrolejeunea borneensis Steph., Sp. Hepat. (Stephani) 5: 557, 1914 (Stephani 1914b).

*** Lepidolejeunea longilobula (Amakawa) Xiao L.He, Acta Bot. Fenn. 163: 59, 1999 (He 1999). Bas.: Pycnolejeunea longilobula Amakawa, J. Jap. Bot. 40 (10): 307, 1965 (Amakawa 1965).

*** **Otolejeunea Grolle et Tixier**, Nova Hedwigia 32: 609, 1980 (Tixier 1980b).

*** **subg. Allorgella (Tixier) Grolle**, Haussknechtia 2: 53, 1985 (Grolle 1985a). Bas.: Allorgella Tixier, Nova Hedwigia 32: 612, 1980 (Tixier 1980b).

*** Otolejeunea australiensis B.M.Thiers, Brittonia 44 (2): 162, 1992 (Thiers 1992a).

*** Otolejeunea hoana (Tixier) Grolle, Haussknechtia 2: 54, 1985 [1986] (Grolle 1985a). Bas.: Allorgella hoana Tixier, Nova Hedwigia 32: 615, 1980 (Tixier 1980b).

*** Otolejeunea rabenorii Tixier, Nova Hedwigia 46 (3/4): 376, 1988 (Tixier 1988).

*** Otolejeunea schmidii (Tixier) Grolle, Haussknechtia 2: 54, 1985 [1986] (Grolle 1985a). Bas.: Allorgella schmidii Tixier, Nova Hedwigia 32: 613, 1980 (Tixier 1980b).

*** Otolejeunea schnellii (Tixier) R.L.Zhu et M.L.So, Ann. Bot. Fenn. 34 (4): 287, 1997 (Zhu and So 1997a). Bas.: Allorgella schnellii Tixier, Cryptog. Bryol. Lichenol. 16 (3): 230, 1995 (Tixier 1995a).

*** Otolejeunea semperiana (Steph.) Grolle, Haussknechtia 2: 53, 1985 [1986] (Grolle 1985a). Bas.: Prionolejeunea semperiana Steph., Sp. Hepat. (Stephani) 5: 227, 1913 (Stephani 1913a).

** Otolejeunea subana Pocs, Acta Acad. Ped. Agr., Sect. Biol. 25: 50, 2004 (Pocs 2004).

*** Otolejeunea zantenii Grolle, Haussknechtia 2: 54, 1985 [1986] (Grolle 1985a).

*** **subg. Otolejeunea**

*** Otolejeunea moniliata Grolle, Nova Hedwigia 32: 609, 1980 (Tixier 1980b). World checklist of hornworts and liverworts 353

*** **subg. Phoxolejeunea Grolle**, Haussknechtia 2: 49, 1985 (Grolle 1985a).

** Otolejeunea philippinensis R.L.Zhu et M.L.So, Syst. Bot. 23 (2): 231, 1998 (Zhu and So 1998).

*** Otolejeunea streimannii Grolle, Haussknechtia 2: 49, 1985 [1986] (Grolle 1985a).

*** **Rectolejeunea A.Evans**, Bull. Torrey Bot. Club 33 (1): 8, 1906 (Evans 1906a).

* Rectolejeunea colombiana R.M.Schust., J. Hattori Bot. Lab. 89: 146, 2000 (Schuster 2000c).

*** Rectolejeunea emarginuliflora (Schiffn.) A.Evans, Bull. Torrey Bot. Club 33 (1): 14, 1906 (Evans 1906a). Bas.: Cheilolejeunea emarginuliflora Schiffn., Bot. Jahrb. Syst. 23 (5): 585, 1897 (Schiffner 1897).

*** Rectolejeunea flagelliformis A.Evans, Bull. Torrey Bot. Club 33 (1): 9, 1906 (Evans 1906a).

* Rectolejeunea flagelliformis subsp. hamata R.M.Schust., J. Hattori Bot. Lab. 89: 137, 2000 (Schuster 2000c).

*** Rectolejeunea queenslandica (B.M.Thiers) Xiao L.He, Ann. Bot. Fenn. 34 (2): 129, 1997 (He 1997). Bas.: Lepidolejeunea queenslandica B.M.Thiers, Mem. New York Bot. Gard. 45: 556, 1987 (Thiers 1987a).

** Rectolejeunea truncatilobula C.J.Bastos, J. Bryol. 34 (2): 144, 2012 (Bastos 2012a).

*** Rectolejeunea versifolia (Schiffn.) L.Söderstr. et A.Hagborg, Phytotaxa 220 (2): 188, 2015 (Söderström et al. 2015a). Bas.: Cheilolejeunea versifolia Schiffn., Bot. Jahrb. Syst. 23 (5): 597, 1897 (Schiffner 1897).

**Excluded from the genus**

* Rectolejeunea lindenbergii Steph., Sp. Hepat. (Stephani) 5: 689, 1914 (Stephani 1914b). ^339^

* Rectolejeunea lindigiana Steph., Sp. Hepat. (Stephani) 5: 690, 1914 (Stephani 1914b). ^340^

* Rectolejeunea pachyderma R.M.Schust., J. Hattori Bot. Lab. 89: 148, 2000 (Schuster 2000c). ^341^

########### ** subtrib. Leptolejeuneinae Heinrichs et Schäf.-Verw.

*** **Leptolejeunea (Spruce) Steph.**, Hedwigia 30 (6): 270, 1891 (Stephani 1891c). Bas.: Lejeunea subg. Leptolejeunea Spruce, Trans. & Proc. Bot. Soc. Edinburgh 15: 193, 1884 (Spruce 1884).

*** Leptolejeunea amphiophthalma Zwickel, Ann. Bryol. 6: 117, 1933 (Zwickel 1933).

** Leptolejeunea apiculata (Horik.) S.Hatt., J. Hattori Bot. Lab. 5: 46, 1951 (Hattori 1951d). Bas.: Drepanolejeunea apiculata Horik., J. Sci. Hiroshima Univ., Ser. B, Div. 2, Bot. 2: 266, 1934 (Horikawa 1934).

** Leptolejeunea arunachalensis Sudipa Das et D.K.Singh, J. Jap. Bot. 83 (6): 343, 2008 (Das and Singh 2008).

*** Leptolejeunea astroidea (Mitt.) Steph., Sp. Hepat. (Stephani) 5: 363, 1913 (Stephani 1913a). Bas.: Lejeunea astroidea Mitt., Trans. Linn. Soc. London 23 (1): 58, 1860 (Mitten 1860a).

** Leptolejeunea australis Steph., Sp. Hepat. (Stephani) 5: 389, 1913 (Stephani 1913a). Based on: Leptolejeunea australis Steph., Hedwigia 28 (3): 173, 1889 (Stephani 1889d), *nom. inval*.

** Leptolejeunea balansae Steph., Hedwigia 35 (3): 105, 1896 (Stephani 1896b).

* Leptolejeunea borneensis Herzog, Flora 135: 394, 1942 (Herzog 1942c). ^342^

** Leptolejeunea brasiliensis Bischl., Nova Hedwigia 17: 301, 1969 (Bischler 1969).

* Leptolejeunea convexistipa Bischl., Nova Hedwigia 17: 325, 1969 (Bischler 1969).

* Leptolejeunea curvatifolia Steph., Sp. Hepat. (Stephani) 6: 398, 1923 (Stephani 1923). ^343^

** Leptolejeunea denticulata Steph., Sp. Hepat. (Stephani) 5: 389, 1913 (Stephani 1913a). Based on: Leptolejeunea denticulata Steph., Hedwigia 28 (3): 174, 1889 (Stephani 1889d), *nom. inval*.

** Leptolejeunea dentistipula Steph., Sp. Hepat. (Stephani) 5: 379, 1913 (Stephani 1913a).

** Leptolejeunea diversilobulata Bischl., Nova Hedwigia 17: 313, 1969 (Bischler 1969).

** Leptolejeunea dolabriformis Pearson, J. Linn. Soc., Bot. 46 (305): 37, 1922 (Pearson 1922b).

*** Leptolejeunea elliptica (Lehm. et Lindenb.) Besch., Rev. Bryol. 19 (1): 14, 1892 (Bescherelle 1892). Bas.: Jungermannia elliptica Lehm. et Lindenb., Nov. Stirp. Pug. 5: 13, 1833 (Lehmann 1833).

* Leptolejeunea emarginata (Horik.) S.Hatt., J. Hattori Bot. Lab. 5: 46, 1951 (Hattori 1951d). Bas.: Drepanolejeunea emarginata Horik., J. Sci. Hiroshima Univ., Ser. B, Div. 2, Bot. 2: 267, 1934 (Horikawa 1934). ^344^

*** Leptolejeunea epiphylla (Mitt.) Steph., Sp. Hepat. (Stephani) 5: 380, 1913 (Stephani 1913a). Bas.: Lejeunea epiphylla Mitt., J. Proc. Linn. Soc., Bot. 5 (18): 118, 1860 [1861] (Mitten 1860c).

*** Leptolejeunea exocellata (Spruce) A.Evans, Bull. Torrey Bot. Club 29 (8): 498, 1902 (Evans 1902c). Bas.: Lejeunea exocellata Spruce, Trans. & Proc. Bot. Soc. Edinburgh 15: 195, 1884 (Spruce 1884).

** Leptolejeunea foliicola Steph., Hedwigia 35 (3): 106, 1896 (Stephani 1896b).

** Leptolejeunea integristipula Steph., Sp. Hepat. (Stephani) 6: 398, 1923 (Stephani 1923).

* Leptolejeunea jamaicensis R.M.Schust., J. Elisha Mitchell Sci. Soc. 83 (4): 229, 1967 (Schuster 1967a).

** Leptolejeunea lancifolia Steph., Sp. Hepat. (Stephani) 5: 382, 1913 (Stephani 1913a). *Nom. nov. pro Lejeunea lancifolia* Mitt., Fl. vit.: 415, 1871 [1873] (Mitten 1871), *nom. illeg*.

** Leptolejeunea latifolia Herzog, Memoranda Soc. Fauna Fl. Fennica 26: 58, 1950 [1951] (Herzog 1950b).

** Leptolejeunea lepinii Steph., Sp. Hepat. (Stephani) 5: 383, 1913 (Stephani 1913a).

** Leptolejeunea ligulata Herzog, Flora 135: 429, 1942 (Herzog 1942c).

*** Leptolejeunea maculata (Mitt.) Schiffn., Consp. Hepat. Arch. Ind.: 275, 1898 (Schiffner 1898b). Bas.: Lejeunea maculata Mitt., J. Proc. Linn. Soc., Bot. 5 (18): 118, 1860 [1861] (Mitten 1860c).

* Leptolejeunea massartiana Schiffn. ex Herzog, Flora 135: 421, 1942 (Herzog 1942c). ^345^

** Leptolejeunea micronesica Inoue et H.A.Mill., Bull. Natl. Sci. Mus. Tokyo (n.ser.) 8 (2): 149, 1965 (Inoue and Miller 1965).

** Leptolejeunea minima Herzog, Memoranda Soc. Fauna Fl. Fennica 26: 60, 1950 [1951] (Herzog 1950b).

** Leptolejeunea mirikana M.Dey et D.K.Singh, Taiwania 55 (4): 355, 2010 (Dey and Singh 2010).

*** Leptolejeunea moniliata Steph., Sp. Hepat. (Stephani) 5: 371, 1913 (Stephani 1913a).

*** Leptolejeunea obfuscata (Spruce) Steph., Sp. Hepat. (Stephani) 5: 373, 1913 (Stephani 1913a). Bas.: Lejeunea obfuscata Spruce, Trans. & Proc. Bot. Soc. Edinburgh 15: 579, 1885 (Spruce 1885).

* Leptolejeunea punctata Herzog, Flora 135: 432, 1942 (Herzog 1942c).

*** Leptolejeunea radicosa (Nees ex Mont.) Grolle, J. Hattori Bot. Lab. 45: 178, 1979 (Grolle 1979c). Bas.: Lejeunea radicosa Nees ex Mont., Hist. Phys. Cuba, Bot., Pl. Cell.: 475, 1842 (Montagne 1842a).

* Leptolejeunea renneri Herzog, Flora 135: 422, 1942 (Herzog 1942c).

* Leptolejeunea revoluta P.C.Chen, Feddes Repert. Spec. Nov. Regni Veg. 58: 44, 1955 (Chen 1955). ^346^

** Leptolejeunea rosulans Steph., Sp. Hepat. (Stephani) 5: 390, 1913 (Stephani 1913a). Based on: Leptolejeunea rosulans Steph., Hedwigia 28 (3): 174, 1889 (Stephani 1889d), *nom. inval*.

** Leptolejeunea serratifolia Schiffn., Bot. Jahrb. Syst. 23 (5): 594, 1897 (Schiffner 1897).

** Leptolejeunea serrulata Herzog, Flora 135: 426, 1942 (Herzog 1942c).

** Leptolejeunea spinistipula (Mizut.) Xiao L.He, Ann. Bot. Fenn. 34 (2): 127, 1997 (He 1997). Bas.: Pycnolejeunea spinistipula Mizut., J. Hattori Bot. Lab. 33: 255, 1970 (Mizutani 1970).

** Leptolejeunea subdentata Schiffn. ex Herzog, Flora 135: 403, 1942 (Herzog 1942c).

*** Leptolejeunea subrotundifolia Herzog, Flora 135: 424, 1942 (Herzog 1942c).

*** Leptolejeunea tridentata Bischl., Nova Hedwigia 17: 335, 1969 (Bischler 1969).

* Leptolejeunea trigonostipa (Spruce) Steph., Sp. Hepat. (Stephani) 5: 376, 1913 (Stephani 1913a). Bas.: Lejeunea trigonostipa Spruce, Trans. & Proc. Bot. Soc. Edinburgh 15: 197, 1884 (Spruce 1884).

** Leptolejeunea tripuncta (Mitt.) Steph., Sp. Hepat. (Stephani) 5: 388, 1913 (Stephani 1913a). Bas.: Lejeunea tripuncta Mitt., Fl. vit.: 415, 1871 [1873] (Mitten 1871).

** Leptolejeunea truncatifolia Steph., Sp. Hepat. (Stephani) 5: 388, 1913 (Stephani 1913a).

* Leptolejeunea udarii M.Dey et D.K.Singh, Taiwania 55 (4): 359, 2010 (Dey and Singh 2010). ^347^

*** Leptolejeunea vitrea (Nees) Schiffn., Hepat. (Engl.-Prantl): 126, 1893 (Schiffner 1893b). Bas.: Jungermannia vitrea Nees, Enum. Pl. Crypt. Javae: 56, 1830 (Nees 1830).

########### ** subtrib. Pycnolejeuneinae Heinrichs et Schäf.Verw.

** **Pycnolejeunea (Spruce) Schiffn.**, Hepat. (Engl.-Prantl): 124, 1893 (Schiffner 1893b). Bas.: Lejeunea subg. Pycnolejeunea Spruce, Trans. & Proc. Bot. Soc. Edinburgh 15: 246, 1884 (Spruce 1884).

* Pycnolejeunea anotomensis Tixier, Bull. Soc. Hist. Nat. Afrique N. 63: 10, 1972 (Tixier 1972a). ^348^

** Pycnolejeunea borneensis Steph., Sp. Hepat. (Stephani) 5: 632, 1914 (Stephani 1914b).

* Pycnolejeunea cavistipula (Steph.) Mizut., J. Hattori Bot. Lab. 36: 161, 1972 [1973] (Mizutani 1972a). Bas.: Strepsilejeunea cavistipula Steph., Hedwigia 35 (3): 128, 1896 (Stephani 1896b). ^349^

* Pycnolejeunea connivens Schiffn. ex P.Syd., Just’s Bot. Jahresber. 19: 246, 1894 (Sydow 1894). Based on: Pycnolejeunea connivens Gottsche ex Schiffn., Leberm., Forschungsr. Gazelle 4 (4): 32, 1890 (Schiffner 1890), *nom. inval*.

*** Pycnolejeunea contigua (Nees) Grolle, J. Hattori Bot. Lab. 45: 179, 1979 (Grolle 1979c). Bas.: Jungermannia contigua Nees, Fl. Bras. (Martius) 1 (1): 360, 1833 (Nees 1833a).

* Pycnolejeunea convexifolia (Mitt.) Steph., Sp. Hepat. (Stephani) 5: 636, 1914 (Stephani 1914b). Bas.: Lejeunea convexifolia Mitt., Fl. vit.: 416, 1871 [1873] (Mitten 1871). ^350^

*** Pycnolejeunea decurviloba Steph., Hedwigia 35 (3): 125, 1896 (Stephani 1896b).

*** Pycnolejeunea densistipula (Lehm. et Lindenb.) Steph., Sp. Hepat. (Stephani) 5: 602, 1914 (Stephani 1914b). Bas.: Lejeunea densistipula Lehm. et Lindenb., Nov. Stirp. Pug. 7: 20, 1838 (Lehmann 1838).

** Pycnolejeunea gradsteinii Ilk.-Borg., Bol. Inst. Bot. (São Paulo) 21 (1): 1, 2011 (Ilkiu-Borges 2011).

** Pycnolejeunea grandiocellata Steph., Bot. Tidsskr. 24 (3): 279, 1902 (Stephani 1902b).

* Pycnolejeunea grossiloba Steph., Sp. Hepat. (Stephani) 5: 629, 1914 (Stephani 1914b). ^351^

*** Pycnolejeunea macroloba (Nees et Mont.) Schiffn., Hepat. (Engl.-Prantl): 124, 1893 (Schiffner 1893b). Bas.: Lejeunea macroloba Nees et Mont., Ann. Sci. Nat. Bot. (sér. 2) 19: 260, 1843 (Montagne 1843).

*** Pycnolejeunea monophthalma (R.M.Schust.) Xiao L.He, Acta Bot. Fenn. 163: 52, 1999 (He 1999). Bas.: Trachylejeunea monophthalma R.M.Schust., Bull. Torrey Bot. Club 97 (6): 345, 1970 [1971] (Schuster 1970b).

** Pycnolejeunea novae-caledoniae (Steph.) Horik., Acta Phytotax. Geobot. 13: 214, 1943 (Horikawa 1943). Bas.: Archilejeunea novae-caledoniae Steph., Sp. Hepat. (Stephani) 4: 729, 1911 (Stephani 1911e).

* Pycnolejeunea palmicola Steph., Sp. Hepat. (Stephani) 6: 413, 1923 (Stephani 1923). ^352^

*** Pycnolejeunea papillosa Xiao L.He, Acta Bot. Fenn. 163: 55, 1999 (He 1999).

** Pycnolejeunea porrectilobula C.J.Bastos et O.Yano, Nova Hedwigia 74 (3/4): 440, 2002 (Bastos and Yano 2002).

** Pycnolejeunea retusa R.M.Schust., J. Hattori Bot. Lab. 100: 402, 2006 (Schuster 2006).

* Pycnolejeunea schlimiana Steph., Sp. Hepat. (Stephani) 5: 615, 1914 (Stephani 1914b). ^353^

*** Pycnolejeunea schwaneckei (Steph.) Schiffn. ex P.Syd., Just’s Bot. Jahresber. 19: 246, 1894 (Sydow 1894). Bas.: Lejeunea schwaneckei Steph., Hedwigia 27 (11/12): 290, 1888 (Stephani 1888c).

** Pycnolejeunea sphaeroides (Sande Lac.) J.B.Jack et Steph., Bot. Centralbl. 60 (4): 107, 1894 (Jack and Stephani 1894). Bas.: Lejeunea sphaeroides Sande Lac., Ann. Mus. Bot. Lugduno-Batavi 1: 309, 1864 (Sande Lacoste 1864).

########### ** subtrib. Xylolejeuneinae Heinrichs et Schäf.Verw.

*** **Xylolejeunea Xiao L.He et Grolle**, Ann. Bot. Fenn. 38 (1): 27, 2001 (He and Grolle 2001).

*** Xylolejeunea aquarius (Spruce) Xiao L.He et Grolle, Ann. Bot. Fenn. 38 (1): 29, 2001 (He and Grolle 2001). Bas.: Lejeunea aquarius Spruce, Trans. & Proc. Bot. Soc. Edinburgh 15: 185, 1884 (Spruce 1884).

*** Xylolejeunea crenata (Nees et Mont.) Xiao L.He et Grolle, Ann. Bot. Fenn. 38 (1): 36, 2001 (He and Grolle 2001). Bas.: Lejeunea crenata Nees et Mont., Ann. Sci. Nat. Bot. (sér. 2) 9: 48, 1838 (Montagne 1838).

*** Xylolejeunea grolleana (Pócs) Xiao L.He et Grolle, Ann. Bot. Fenn. 38 (1): 32, 2001 (He and Grolle 2001). Bas.: Trachylejeunea grolleana Pócs, Haussknechtia, Beih. 9: 285, 1999 (Pócs 1999).

*** Xylolejeunea muricella Xiao L.He et Grolle, Ann. Bot. Fenn. 38 (1): 34, 2001 (He and Grolle 2001).

########## ** trib. Symbiezidieae Gradst.

*** **Symbiezidium Trevis.**, Mem. Reale Ist. Lombardo Sci. (Ser. 3), C. Sci. Mat. 4 (13): 402, 1877 (Trevisan 1877).

*** Symbiezidium barbiflorum (Lindenb. et Gottsche) A.Evans, Bull. Torrey Bot. Club 34 (11): 540, 1907 [1908] (Evans 1907a). Bas.: Lejeunea barbiflora Lindenb. et Gottsche, Linnaea 24 (6): 630, 1851 [1852] (Lindenberg and Gottsche 1851a).

*** Symbiezidium dentatum Herzog, Feddes Repert. Spec. Nov. Regni Veg. 57 (1/2): 175, 1955 (Herzog 1955).

*** Symbiezidium madagascariense Steph., Sp. Hepat. (Stephani) 5: 99, 1912 (Stephani 1912c).

*** Symbiezidium transversale (Sw.) Trevis., Mem. Reale Ist. Lombardo Sci. (Ser. 3), C. Sci. Mat. 4 (13): 403, 1877 (Trevisan 1877). Bas.: Jungermannia transversalis Sw., Prodr. (Swartz): 144, 1788 (Swartz 1788).

*** Symbiezidium transversale var. hookerianum (Gottsche, Lindenb. et Nees) Gradst. et J.Beek, Beih. Nova Hedwigia 80: 237, 1985 (Gradstein and van Beek 1985). Bas.: Lejeunea transversalis β hookeriana Gottsche, Lindenb. et Nees, Syn. Hepat. 3: 311, 1845 (Gottsche et al. 1845b).

######### *** Ptychanthoideae Mizut.

*** **Acrolejeunea (Spruce) Schiffn.**, Hepat. (Engl.-Prantl): 128, 1893 (Schiffner 1893b) nom. conserv. Bas.: Lejeunea subg. Acrolejeunea Spruce, Trans. & Proc. Bot. Soc. Edinburgh 15: 115, 1884 (Spruce 1884).

*** Acrolejeunea sandvicensis (Gottsche) Steph., Bot. Jahrb. Syst. 23 (1/2, 3): 312, 1896 (Stephani 1896a). Bas.: Phragmicoma sandvicensis Gottsche, Ann. Sci. Nat. Bot. (sér. 4) 8: 344, 1857 (Gottsche 1857).

** **sect. Acrolejeunea**

*** Acrolejeunea emergens (Mitt.) Steph., Pflanzenw. Ost-Afrikas C: 65, 1895 (Stephani 1895d). Bas.: Phragmicoma emergens Mitt., Philos. Trans. 168: 397, 1879 (Mitten 1879).

*** Acrolejeunea emergens var. confertissima (Steph.) Gradst., Bryophyt. Biblioth. 4: 76, 1975 (Gradstein 1975). Bas.: Acrolejeunea confertissima Steph., Hedwigia 31 (4): 165, 1892 (Stephani 1892g).

*** Acrolejeunea heterophylla (A.Evans) Grolle et Gradst., J. Hattori Bot. Lab. 38: 332, 1974 (Gradstein 1974a). Bas.: Ptychocoleus heterophyllus A.Evans, Amer. J. Bot. 5 (3): 144, 1918 (Evans 1918).

*** Acrolejeunea torulosa (Lehm. et Lindenb.) Schiffn., Hepat. (Engl.-Prantl): 128, 1893 (Schiffner 1893b). Bas.: Jungermannia torulosa Lehm. et Lindenb., Nov. Stirp. Pug. 6: 41, 1834 (Lehmann 1834).

** **sect. Minores (Verd.) L.Söderstr. et A.Hagborg**, Bryophyte Diversity Evol. 36 (1): 41, 2014 (Wang et al. 2014b). Bas.: Ptychocoleus sect. Minores Verd., Ann. Bryol., Suppl. 4: 132, 1934 (Verdoorn 1934a).

*** Acrolejeunea arcuata (Nees) Grolle et Gradst., J. Hattori Bot. Lab. 38: 332, 1974 (Gradstein 1974a). Bas.: Jungermannia arcuata Nees, Enum. Pl. Crypt. Javae: 38, 1830 (Nees 1830).

** Acrolejeunea arcuata subsp. gradsteinii M.A.M.Renner, Phytotaxa 83 (1): 42, 2013 (Renner 2013).

*** Acrolejeunea fertilis (Reinw., Blume et Nees) Schiffn., Hepat. (Engl.-Prantl): 128, 1893 (Schiffner 1893b). Bas.: Jungermannia fertilis Reinw., Blume et Nees, Nova Acta Phys.-Med. Acad. Caes. Leop.-Carol. Nat. Cur. 12 (1): 211, 1824 [1825] (Reinwardt et al. 1824a).

*** Acrolejeunea parvula (Mizut.) Gradst., Bryophyt. Biblioth. 4: 115, 1975 (Gradstein 1975). Bas.: Ptychocoleus parvulus Mizut., Dansk Bot. Ark. 27 (1): 97, 1969 (Hattori and Mizutani 1969).

*** Acrolejeunea pycnoclada (Taylor) Schiffn., Hepat. (Engl.-Prantl): 128, 1893 (Schiffner 1893b). Bas.: Ptychanthus pycnocladus Taylor, London J. Bot. 5: 385, 1846 (Taylor 1846b).

*** Acrolejeunea pycnoclada subsp. latistipula Gradst., Bryophyt. Biblioth. 4: 113, 1975 (Gradstein 1975).

*** Acrolejeunea tjibodensis (Verd.) Grolle et Gradst., J. Hattori Bot. Lab. 38: 333, 1974 (Gradstein 1974a). Bas.: Ptychocoleus tjibodensis Verd., Recueil Trav. Bot. Neerl. 30: 227, 1933 (Verdoorn 1933b).

** **sect. Pusillae Gradst.**, Bryophyt. Biblioth. 4: 59, 1975 (Gradstein 1975).

*** Acrolejeunea pusilla (Steph.) Grolle et Gradst., J. Hattori Bot. Lab. 38: 332, 1974 (Gradstein 1974a). Bas.: Archilejeunea pusilla Steph., Sp. Hepat. (Stephani) 4: 731, 1911 (Stephani 1911e).

*** Acrolejeunea sikkimensis (Mizut.) Gradst., Bryophyt. Biblioth. 4: 83, 1975 (Gradstein 1975). Bas.: Ptychocoleus sikkimensis Mizut., Fl. E. Himalaya: 532, 1966 (Hattori 1966c).

** **sect. Recurvatae Jian Wang bis et Gradst.**, Bryophyte Diversity Evol. 36 (1): 39, 2014 (Wang et al. 2014b).

*** Acrolejeunea recurvata Gradst., Bryophyt. Biblioth. 4: 79, 1975 (Gradstein 1975).

** **sect. Regulares (Verd.) Gradst.**, Bryophyt. Biblioth. 4: 63, 1975 (Gradstein 1975). Bas.: Ptychocoleus sect. Regulares Verd., Ann. Bryol., Suppl. 4: 143, 1934 (Verdoorn 1934a).

*** Acrolejeunea allisonii Gradst., Bryophyt. Biblioth. 4: 103, 1975 (Gradstein 1975).

*** Acrolejeunea aulacophora (Mont.) Steph., Bot. Jahrb. Syst. 20 (3): 317, 1895 (Stephani 1895a). Bas.: Phragmicoma aulacophora Mont., Ann. Sci. Nat. Bot. (sér. 2) 19: 259, 1843 (Montagne 1843).

*** Acrolejeunea mollis (Hook.f. et Taylor) Schiffn., Hedwigia 33 (4): 178, 1894 (Schiffner 1894). Bas.: Ptychanthus mollis Hook.f. et Taylor, London J. Bot. 5: 384, 1846 (Taylor 1846b).

*** Acrolejeunea securifolia (Nees) Steph., Hedwigia 34 (2): 59, 1895 (Stephani 1895c). Bas.: Jungermannia securifolia Nees, Prodr. Fl. Norfolk.: 5, 1833 (Endlicher 1833).

*** Acrolejeunea securifolia subsp. caledonica (Steph.) Gradst., Bryophyt. Biblioth. 4: 100, 1975 (Gradstein 1975). Bas.: Ptychocoleus caledonicus Steph., Sp. Hepat. (Stephani) 5: 39, 1912 (Stephani 1912c).

*** Acrolejeunea securifolia subsp. hartmannii (Steph.) Gradst., Bryophyt. Biblioth. 4: 99, 1975 (Gradstein 1975). Bas.: Ptychocoleus hartmannii Steph., Sp. Hepat. (Stephani) 5: 44, 1912 (Stephani 1912c).

*** Acrolejeunea securifolia subsp. pallida (Ångstr.) Gradst., Bryophyt. Biblioth. 4: 101, 1975 (Gradstein 1975). Bas.: Phragmicoma pallida Ångstr., Öfvers. Kongl. Vetensk.-Akad. Förh. 30 (5): 132, 1873 (Ångström 1873).

** **sect. Trocholejeunea (Schiffn.) Jian Wang bis et Gradst.**, Bryophyte Diversity Evol. 36 (1): 38, 2014 (Wang et al. 2014b). Bas.: Trocholejeunea Schiffn., Ann. Bryol. 5: 160, 1932 (Dixon et al. 1932).

*** Acrolejeunea crassicaulis (Steph.) Jian Wang bis et Gradst., Bryophyte Diversity Evol. 36 (1): 38, 2014 (Wang et al. 2014b). Bas.: Hygrolejeunea crassicaulis Steph., Sp. Hepat. (Stephani) 5: 550, 1914 (Stephani 1914b).

*** Acrolejeunea infuscata (Mitt.) Jian Wang bis et Gradst., Bryophyte Diversity Evol. 36 (1): 38, 2014 (Wang et al. 2014b). Bas.: Lejeunea infuscata Mitt., J. Proc. Linn. Soc., Bot. 5 (18): 111, 1860 [1861] (Mitten 1860c).

** Acrolejeunea meghalayensis (Ajit P.Singh et V.Nath) Jian Wang bis et Gradst., Bryophyte Diversity Evol. 36 (1): 39, 2014 (Wang et al. 2014b). Bas.: Trocholejeunea meghalayensis Ajit P.Singh et V.Nath, J. Jap. Bot. 83 (1): 2, 2008 (Singh and Nath 2008).

** Acrolejeunea sinensis (Jian Wang bis, R.L.Zhu et Gradst.) Jian Wang bis et Gradst., Bryophyte Diversity Evol. 36 (1): 39, 2014 (Wang et al. 2014b). Bas.: Trocholejeunea sinensis Jian Wang bis, R.L.Zhu et Gradst., Phytotaxa 174 (5): 296, 2014 (Wang et al. 2014c).

**Excluded from the genus**

* Acrolejeunea abnormis (Gottsche) Pearson, Forh. Vidensk.-Selsk. Kristiania 1892 (8): 4, 1892 (Pearson 1892). Bas.: Phragmicoma abnormis Gottsche, Abh. Naturwiss. Vereins Bremen 7: 352, 1882 (Gottsche 1882). ^354^

* Acrolejeunea comptonii Pearson, J. Linn. Soc., Bot. 46 (305): 33, 1922 (Pearson 1922b). ^355^

* Acrolejeunea inflexa (Gottsche) Pearson, Forh. Vidensk.-Selsk. Kristiania 1892 (8): 4, 1892 (Pearson 1892). Bas.: Phragmicoma inflexa Gottsche, Abh. Naturwiss. Vereins Bremen 7: 351, 1882 (Gottsche 1882). ^356^

*** **Archilejeunea (Spruce) Steph.**, Hedwigia 27 (3/4): 113, 1888 (Stephani 1888d). Bas.: Lejeunea subg. Archilejeunea Spruce, Trans. & Proc. Bot. Soc. Edinburgh 15: 88, 1884 (Spruce 1884). ^357^

*** Archilejeunea abbreviata (Mitt.) Vanden Berghen, Rev. Bryol. Lichénol. 20 (1/2): 117, 1951 (Vanden Berghen 1951c). Bas.: Lejeunea abbreviata Mitt., J. Proc. Linn. Soc., Bot. 7 (27): 167, 1863 (Mitten 1863).

** Archilejeunea africana Steph., Sp. Hepat. (Stephani) 4: 705, 1911 (Stephani 1911e).

** Archilejeunea alata Steph., Bull. Soc. Roy. Bot. Belgique, Compt. Rend. 32 (2): 33, 1893 [1894] (Stephani 1893e).

** Archilejeunea amakawana Inoue, J. Jap. Bot. 41 (1): 16, 1966 (Inoue 1966d). *Nom. nov. pro Archilejeunea falcata* Amakawa, J. Jap. Bot. 39 (5): 137, 1964 (Amakawa 1964b), *nom. illeg*.

*** Archilejeunea auberiana (Mont.) Steph., Hedwigia 29 (3): 134, 1890 (Stephani 1890d). Bas.: Lejeunea auberiana Mont., Hist. Phys. Cuba, Bot., Pl. Cell.: 483, 1842 (Montagne 1842a).

*** Archilejeunea autoica Vanden Berghen, Rev. Bryol. Lichénol. 20 (1/2): 119, 1951 (Vanden Bergen 1951b).

*** Archilejeunea badia (Spruce) Steph., Sp. Hepat. (Stephani) 4: 711, 1911 (Stephani 1911e). Bas.: Lejeunea badia Spruce, Trans. & Proc. Bot. Soc. Edinburgh 15: 92, 1884 (Spruce 1884).

* Archilejeunea bilabiata (Mitt.) Steph., Sp. Hepat. (Stephani) 4: 723, 1911 (Stephani 1911e). Bas.: Phragmicoma bilabiata Mitt., Fl. vit.: 412, 1871 [1873] (Mitten 1871). ^358^

*** Archilejeunea bischleriana Gradst., Fl. Neotrop. Monogr. 62: 62, 1994 (Gradstein 1994).

** Archilejeunea bongardii Steph., Hedwigia 29 (1): 20, 1890 (Stephani 1890a).

** Archilejeunea brachyantha J.B.Jack et Steph., Bot. Centralbl. 60 (4): 104, 1894 (Jack and Stephani 1894).

** Archilejeunea brevilobula Steph., Sp. Hepat. (Stephani) 4: 706, 1911 (Stephani 1911e).

*** Archilejeunea crispistipula (Spruce) Steph., Sp. Hepat. (Stephani) 4: 712, 1911 (Stephani 1911e). Bas.: Lejeunea crispistipula Spruce, Trans. & Proc. Bot. Soc. Edinburgh 15: 93, 1884 (Spruce 1884).

* Archilejeunea eberhardtii Steph., Sp. Hepat. (Stephani) 4: 725, 1911 (Stephani 1911e).

** Archilejeunea elobulata Steph., Sp. Hepat. (Stephani) 4: 707, 1911 (Stephani 1911e).

** Archilejeunea gradsteinii X.Q.Shi et R.L.Zhu, Nova Hedwigia 100 (3/4): 592, 2015 (Shi and Zhu 2015).

** Archilejeunea incrassata Steph., Rev. Bryol. 35 (2): 30, 1908 (Stephani 1908l).

** Archilejeunea jonesii Vanden Berghen, Rev. Bryol. Lichénol. 20 (1/2): 116, 1951 (Vanden Berghen 1951c).

*** Archilejeunea juliformis (Nees) Gradst., Bryophyt. Biblioth. 4: 127, 1975 (Gradstein 1975). Bas.: Jungermannia juliformis Nees, Fl. Bras. (Martius) 1 (1): 351, 1833 (Nees 1833a).

*** Archilejeunea kiushiana (Horik.) Verd., Ann. Bryol., Suppl. 4: 46, 1934 (Verdoorn 1934a). Bas.: Lopholejeunea kiushiana Horik., J. Sci. Hiroshima Univ., Ser. B, Div. 2, Bot. 1: 129, 1932 (Horikawa 1932c).

*** Archilejeunea ludoviciana (De Not.) P.Geissler et Gradst., J. Hattori Bot. Lab. 75: 202, 1994 (Geissler and Gradstein 1994). Bas.: Phragmicoma ludoviciana De Not., Nov. Stirp. Pug. 10: 11, 1857 (Lehmann 1857).

*** Archilejeunea ludoviciana subsp. porelloides (Spruce) Gradst., Fl. Neotrop. Monogr. 62: 58, 1994 (Gradstein 1994). Bas.: Lejeunea porelloides Spruce, Trans. & Proc. Bot. Soc. Edinburgh 15: 90, 1884 (Spruce 1884).

** Archilejeunea nebeliana Gradst. et Schäf.-Verw., Cryptog. Bryol. 33 (2): 108, 2012 (Gradstein and Schäfer-Verwimp 2012).

*** Archilejeunea olivacea (Hook.f. et Taylor) Steph., Hedwigia 29 (3): 134, 1890 (Stephani 1890d). Bas.: Jungermannia olivacea Hook.f. et Taylor, London J. Bot. 3: 568, 1844 (Hooker and Taylor 1844d).

*** Archilejeunea parviflora (Nees) Steph., Hedwigia 29 (3): 134, 1890 (Stephani 1890d). Bas.: Jungermannia parviflora Nees, Fl. Bras. (Martius) 1 (1): 353, 1833 (Nees 1833a).

** Archilejeunea planifolia (Horik.) Mizut., J. Hattori Bot. Lab. 73: 175, 1993 (Mizutani 1993). Bas.: Leucolejeunea planifolia Horik., J. Sci. Hiroshima Univ., Ser. B, Div. 2, Bot. 1: 199, 1933 (Horikawa 1933). ^359^

*** Archilejeunea planiuscula (Mitt.) Steph., Sp. Hepat. (Stephani) 4: 731, 1911 (Stephani 1911e). Bas.: Lejeunea planiuscula Mitt., J. Proc. Linn. Soc., Bot. 5 (18): 111, 1860 [1861] (Mitten 1860c).

**Excluded from the genus**

* Archilejeunea negrensis Steph., Sp. Hepat. (Stephani) 4: 716, 1911 (Stephani 1911e). ^360^

* Archilejeunea ovata Herzog, Rev. Bryol. Lichénol. 20 (1/2): 130, 1951 [1952] (Herzog 1951a). ^361^

*** **Bryopteris (Nees) Lindenb.**, Syn. Hepat. 2: 284, 1845 (Gottsche et al. 1845a). Bas.: Frullania subg. Bryopteris Nees, Naturgesch. Eur. Leberm. 3: 211, 1838 (Nees 1838b).

*** Bryopteris diffusa (Sw.) Nees, Syn. Hepat. 2: 286, 1845 (Gottsche et al. 1845a). Bas.: Jungermannia diffusa Sw., Prodr. (Swartz): 144, 1788 (Swartz 1788).

*** Bryopteris filicina (Sw.) Nees, Syn. Hepat. 2: 284, 1845 (Gottsche et al. 1845a). Bas.: Jungermannia filicina Sw., Prodr. (Swartz): 145, 1788 (Swartz 1788).

* Bryopteris fissiloba Steph., Sp. Hepat. (Stephani) 6: 568, 1924 (Stephani 1924). ^362^

*** Bryopteris gaudichaudii Gottsche, Ann. Sci. Nat. Bot. (sér. 4) 8: 340, 1857 (Gottsche 1857).

*** **Caudalejeunea Schiffn.**, Hepat. (Engl.-Prantl): 129, 1893 (Schiffner 1893b).

** **subg. Acaudalejeunea R.M.Schust.**, Hepat. Anthocerotae N. Amer. 4: 779, 1980 (Schuster 1980c).

*** Caudalejeunea grolleana Gradst., Acta Bot. Neerl. 23 (3): 334, 1974 (Gradstein 1974b).

** **subg. Caudalejeunea**

** Caudalejeunea africana (Steph.) Schiffn., Hepat. (Engl.-Prantl): 129, 1893 (Schiffner 1893b). Bas.: Thysananthus africanus Steph., Bot. Jahrb. Syst. 8 (2): 93, 1886 (Stephani 1886d).

*** Caudalejeunea hanningtonii (Mitt.) Schiffn., Hepat. (Engl.-Prantl): 129, 1893 (Schiffner 1893b). Bas.: Lejeunea hanningtonii Mitt., J. Linn. Soc., Bot. 22 (146): 324, 1886 (Mitten 1886b).

** Caudalejeunea katangensis Vanden Berghen, Explor. Hydrobiol. Lac Bangweolo Luapula: 94, 1972 (Vanden Berghen 1972b).

*** Caudalejeunea lehmanniana (Gottsche) A.Evans, Bull. Torrey Bot. Club 34 (11): 554, 1907 [1908] (Evans 1907a). Bas.: Lejeunea lehmanniana Gottsche, Syn. Hepat. 3: 325, 1845 (Gottsche et al. 1845b).

** Caudalejeunea lewallei Vanden Berghen, Bull. Jard. Bot. Natl. Belg. 42 (4): 434, 1972 (Vanden Berghen 1972a).

** **subg. Vermilejeunea R.M.Schust.**, Hepat. Anthocerotae N. Amer. 4: 778, 1980 (Schuster 1980c).

*** Caudalejeunea cristiloba (Steph.) Gradst., Acta Bot. Neerl. 23 (3): 340, 1974 (Gradstein 1974b). Bas.: Acrolejeunea cristiloba Steph., Hedwigia 34 (2): 56, 1895 (Stephani 1895c).

*** Caudalejeunea cristiloba subsp. samoana (Steph.) Gradst., Acta Bot. Neerl. 23 (3): 343, 1974 (Gradstein 1974b). Bas.: Caudalejeunea samoana Steph., Akad. Wiss. Wien, Math.-Naturwiss. Kl., Denkschr. 81: 296, 1907 (Stephani 1907a).

** Caudalejeunea dusenii Steph., Sp. Hepat. (Stephani) 5: 11, 1912 (Stephani 1912c).

*** Caudalejeunea reniloba (Gottsche) Steph., Sp. Hepat. (Stephani) 5: 16, 1912 (Stephani 1912c). Bas.: Phragmicoma reniloba Gottsche, Syn. Hepat. 2: 301, 1845 (Gottsche et al. 1845a).

** Caudalejeunea yangambiensis (Vanden Berghen) E.W.Jones, Trans. Brit. Bryol. Soc. 3 (2): 192, 1957 (Jones 1957b). Bas.: Ptychocoleus yangambiensis Vanden Berghen, Bull. Soc. Roy. Bot. Belgique 84: 61, 1951 (Vanden Berghen 1951a).

***Incertae sedis***

** Caudalejeunea acutifolia Gerola, Lav. Bot. Ist. Bot. Univ. Padova 12: 478, 1947 (Gerola 1947).

* Caudalejeunea mauritiana Horik., Acta Phytotax. Geobot. 13: 214, 1943 (Horikawa 1943). *Nom. nov. pro Dicranolejeunea africana* Steph., Sp. Hepat. (Stephani) 5: 158, 1912 (Stephani 1912c).

** Caudalejeunea streimannii Gyarmati, Trop. Bryol. 22: 129, 2002 (Sass-Gyarmati 2002).

** Caudalejeunea tridentata R.L.Zhu, Y.M.Wei et Qiong He, Bryologist 114 (3): 469, 2011 (Zhu et al. 2011).

** **Cephalantholejeunea R.M.Schust.**, Hepat. Anthocerotae N. Amer. 4: 798, 1980 (Schuster 1980c).

*** Cephalantholejeunea temnanthoides (R.M.Schust.) R.M.Schust., Hepat. Anthocerotae N. Amer. 4: 807, 1980 (Schuster 1980c). Bas.: Potamolejeunea temnanthoides R.M.Schust., Beih. Nova Hedwigia 9: 123, 1963 (Schuster 1963a).

** **Cephalolejeunea Mizut.**, J. Hattori Bot. Lab. 46: 359, 1979 (Mizutani 1979b).

*** Cephalolejeunea parvilobula Mizut., J. Hattori Bot. Lab. 46: 359, 1979 (Mizutani 1979b).

*** **Frullanoides Raddi**, Critt. Brasil.: 13, 1822 (Raddi 1822).

*** Frullanoides bahamensis (A.Evans) van Slageren, Meded. Bot. Mus. Herb. Rijks Univ. Utrecht 544: 81, 1985 (van Slageren 1985). Bas.: Brachiolejeunea bahamensis A.Evans, Bull. Torrey Bot. Club 35 (8): 383, 1908 (Evans 1908b).

*** Frullanoides corticalis (Lehm. et Lindenb.) van Slageren, Meded. Bot. Mus. Herb. Rijks Univ. Utrecht 544: 84, 1985 (van Slageren 1985). Bas.: Jungermannia corticalis Lehm. et Lindenb., Nov. Stirp. Pug. 4: 50, 1832 (Lehmann 1832).

*** Frullanoides densifolia Raddi, Critt. Brasil.: 14, 1822 (Raddi 1822).

*** Frullanoides densifolia subsp. grandidentata (L.Clark) van Slageren, Meded. Bot. Mus. Herb. Rijks Univ. Utrecht 544: 95, 1985 (van Slageren 1985). Bas.: Brachiolejeunea grandidentata L.Clark, Proc. Calif. Acad. Sci. (ser. 4) 27 (18): 595, 1953 (Clark 1953).

*** Frullanoides laciniatiflora (Loitl.) van Slageren, Meded. Bot. Mus. Herb. Rijks Univ. Utrecht 544: 100, 1985 (van Slageren 1985). Bas.: Lejeunea laciniatiflora Loitl., Diagn. pl. nov.: 19, 1894 (Loitlesberger 1894).

*** Frullanoides liebmaniana (Lindenb. et Gottsche) van Slageren, Meded. Bot. Mus. Herb. Rijks Univ. Utrecht 544: 102, 1985 (van Slageren 1985). Bas.: Phragmicoma liebmaniana Lindenb. et Gottsche, Syn. Hepat. 5: 744, 1847 (Gottsche et al. 1847).

*** Frullanoides mexicana van Slageren, Meded. Bot. Mus. Herb. Rijks Univ. Utrecht 544: 106, 1985 (van Slageren 1985).

*** Frullanoides tristis (Steph.) van Slageren, Meded. Bot. Mus. Herb. Rijks Univ. Utrecht 544: 110, 1985 (van Slageren 1985). Bas.: Brachiolejeunea tristis Steph., Sp. Hepat. (Stephani) 5: 112, 1912 (Stephani 1912c).

*** **Fulfordianthus Gradst.**, Bryologist 95 (1): 44, 1992 (Gradstein 1992a).

*** Fulfordianthus evansii (Fulford) Gradst., Bryologist 95 (1): 46, 1992 (Gradstein 1992a). Bas.: Thysananthus evansii Fulford, Bull. Torrey Bot. Club 68 (1): 34, 1941 (Fulford 1941).

*** Fulfordianthus pterobryoides (Spruce) Gradst., Bryologist 95 (1): 44, 1992 (Gradstein 1992a). Bas.: Lejeunea pterobryoides Spruce, Trans. & Proc. Bot. Soc. Edinburgh 15: 109, 1884 (Spruce 1884).

*** **Lopholejeunea (Spruce) Steph.**, Bot. Gaz. 15 (11): 285, 1890 (Stephani 1890c) nom. rejic. Bas.: Lejeunea subg. Lopholejeunea Spruce, Trans. & Proc. Bot. Soc. Edinburgh 15: 119, 1884 (Spruce 1884).

** **subg. Lopholejeunea**

** Lopholejeunea borbonica Steph., Hedwigia 35 (3): 109, 1896 (Stephani 1896b).

** Lopholejeunea jonesii Vanden Berghen, Bull. Jard. Bot. État Bruxelles 20 (2): 178, 1950 (Vanden Berghen 1950).

** Lopholejeunea laciniata E.W.Jones, Trans. Brit. Bryol. Soc. 3 (2): 194, 1957 (Jones 1957b).

** Lopholejeunea minima Vanden Berghen, Bull. Jard. Bot. Natl. Belg. 54 (3/4): 437, 1984 (Vanden Berghen 1984a).

* Lopholejeunea obtusilacera Herzog, Bull. Jard. Bot. État Bruxelles 20 (2): 172, 1950 (Vanden Berghen 1950).

* Lopholejeunea paramultilacera Vanden Berghen, Bull. Jard. Bot. Natl. Belg. 54 (3/4): 435, 1984 (Vanden Berghen 1984a).

* Lopholejeunea quinquecarinata Vanden Berghen, Bull. Jard. Bot. Natl. Belg. 54 (3/4): 408, 1984 (Vanden Berghen 1984a).

** Lopholejeunea renistipula (Mitt.) Steph., Sp. Hepat. (Stephani) 5: 94, 1912 (Stephani 1912c). Bas.: Phragmicoma renistipula Mitt., Fl. vit.: 413, 1871 [1873] (Mitten 1871).

** Lopholejeunea revoluta E.W.Jones, Trans. Brit. Bryol. Soc. 3 (2): 207, 1957 (Jones 1957b).

** **sect. Eulophae Verd.**, Ann. Bryol., Suppl. 4: 87, 1934 (Verdoorn 1934a).

*** Lopholejeunea applanata (Reinw., Blume et Nees) Schiffn., Hepat. (Engl.-Prantl): 129, 1893 (Schiffner 1893b). Bas.: Jungermannia applanata Reinw., Blume et Nees, Nova Acta Phys.-Med. Acad. Caes. Leop.-Carol. Nat. Cur. 12 (1): 210, 1824 [1825] (Reinwardt et al. 1824a).

*** Lopholejeunea borneensis (Steph.) Verd., Ann. Bryol., Suppl. 4: 83, 1934 (Verdoorn 1934a). Bas.: Mastigolejeunea borneensis Steph., Sp. Hepat. (Stephani) 4: 777, 1912 (Stephani 1912b).

*** Lopholejeunea erugata B.M.Thiers, Brittonia 36 (2): 174, 1984 (Thiers 1984). *Nom. nov. pro Ptychocoleus inermis* Steph., Sp. Hepat. (Stephani) 5: 27, 1912 (Stephani 1912c).

*** Lopholejeunea eulopha (Taylor) Schiffn., Hepat. (Engl.-Prantl): 129, 1893 (Schiffner 1893b). Bas.: Lejeunea eulopha Taylor, London J. Bot. 5: 391, 1846 (Taylor 1846b).

*** Lopholejeunea evansiana Verd., Nova Guinea 18: 4, 1934 (Verdoorn 1934d).

*** Lopholejeunea grollei R.L.Zhu et Gradst., Monogr. Syst. Bot. Missouri Bot. Gard. 74: 36, 2005 (Zhu and Gradstein 2005).

*** Lopholejeunea herzogiana Verd., Recueil Trav. Bot. Neerl. 30: 217, 1933 (Verdoorn 1933b).

*** Lopholejeunea hispidissima Steph., Sp. Hepat. (Stephani) 5: 80, 1912 (Stephani 1912c).

*** Lopholejeunea loheri Steph., Sp. Hepat. (Stephani) 5: 77, 1912 (Stephani 1912c).

*** Lopholejeunea minuta R.L.Zhu et Gradst., Nova Hedwigia 78 (3/4): 436, 2004 (Zhu and Gradstein 2004).

*** Lopholejeunea nigricans (Lindenb.) Schiffn., Consp. Hepat. Arch. Ind.: 293, 1898 (Schiffner 1898b). Bas.: Lejeunea nigricans Lindenb., Syn. Hepat. 3: 316, 1845 (Gottsche et al. 1845b).

*** Lopholejeunea plicatiscypha (Hook.f. et Taylor) Steph., Sp. Hepat. (Stephani) 5: 96, 1912 (Stephani 1912c). Bas.: Phragmicoma plicatiscypha Hook.f. et Taylor, London J. Bot. 5: 386, 1846 (Taylor 1846b).

* Lopholejeunea proxima Steph., Sp. Hepat. (Stephani) 5: 89, 1912 (Stephani 1912c). ^363^

*** Lopholejeunea streimannii B.M.Thiers et Gradst., Mem. New York Bot. Gard. 52: 37, 1989 (Thiers and Gradstein 1989).

** Lopholejeunea vojtkoana Gyarmati, Nova Hedwigia 87 (3/4): 480, 2008 (Sass-Gyarmati 2008).

** **sect. Lopholejeunea**

*** Lopholejeunea ceylanica Steph., Sp. Hepat. (Stephani) 5: 86, 1912 (Stephani 1912c).

*** Lopholejeunea horticola Schiffn., Ann. Bryol. 6: 133, 1933 (Herzog et al. 1933).

*** Lopholejeunea latilobula Verd., Nova Guinea 18: 4, 1934 (Verdoorn 1934d).

*** Lopholejeunea magna Mizut., J. Hattori Bot. Lab. 32: 131, 1969 (Mizutani 1969).

*** Lopholejeunea recurvata Mizut., J. Hattori Bot. Lab. 46: 369, 1979 (Mizutani 1979b).

*** Lopholejeunea soae R.L.Zhu et Gradst., Monogr. Syst. Bot. Missouri Bot. Gard. 74: 69, 2005 (Zhu and Gradstein 2005).

*** Lopholejeunea subfusca (Nees) Schiffn., Bot. Jahrb. Syst. 23 (5): 593, 1897 (Schiffner 1897). Bas.: Jungermannia subfusca Nees, Enum. Pl. Crypt. Javae: 36, 1830 (Nees 1830).

** Lopholejeunea subfusca var. elongata Vanden Berghen, Bull. Jard. Bot. Natl. Belg. 54 (3/4): 445, 1984 (Vanden Berghen 1984a).

*** Lopholejeunea wiltensii Steph., Hedwigia 35 (3): 112, 1896 (Stephani 1896b).

* Lopholejeunea yapensis Steph., Sp. Hepat. (Stephani) 5: 81, 1912 (Stephani 1912c). ^364^

*** Lopholejeunea zollingeri (Steph.) Schiffn., Consp. Hepat. Arch. Ind.: 296, 1898 (Schiffner 1898b). Bas.: Lejeunea zollingeri Steph., Hedwigia 29 (1): 14, 1890 (Stephani 1890a).

** **subg. Pholianthus B.M.Thiers et Gradst.**, Mem. New York Bot. Gard. 52: 25, 1989 (Thiers and Gradstein 1989).

*** Lopholejeunea colensoi Steph., Sp. Hepat. (Stephani) 5: 97, 1912 (Stephani 1912c).

*** Lopholejeunea pocsii Gyarmati, Cryptog. Bryol. 26 (4): 404, 2005 (Sass-Gyarmati 2005).

** **subg. Pteryganthus B.M.Thiers**, Brittonia 35 (1): 85, 1983 (Thiers 1983).

** Lopholejeunea grandicrista Steph., Bull. Soc. Roy. Bot. Belgique, Compt. Rend. 32 (2): 34, 1893 [1894] (Stephani 1893e).

*** Lopholejeunea leioptera Gyarmati, Candollea 56 (1): 80, 2001 (Sass-Gyarmati 2001).

** Lopholejeunea onraedtii Vanden Berghen, Bull. Jard. Bot. Natl. Belg. 54 (3/4): 452, 1984 (Vanden Berghen 1984a).

** Lopholejeunea sphaerophora (Lehm. et Lindenb.) Steph., Sp. Hepat. (Stephani) 5: 68, 1912 (Stephani 1912c). Bas.: Jungermannia sphaerophora Lehm. et Lindenb., Nov. Stirp. Pug. 5: 9, 1833 (Lehmann 1833).

* Lopholejeunea tixieriana Vanden Berghen, Bull. Jard. Bot. Natl. Belg. 54 (3/4): 454, 1984 (Vanden Berghen 1984a).

** Lopholejeunea utriculata Steph., Sp. Hepat. (Stephani) 5: 69, 1912 (Stephani 1912c).

***Incertae sedis***

** Lopholejeunea lepidoscypha Kiaer et Pearson, Forh. Vidensk.-Selsk. Kristiania 1892 (8): 5, 1892 (Pearson 1892).

* Lopholejeunea multilacera Steph., Bot. Gaz. 15 (11): 285, 1890 (Stephani 1890c).

** Lopholejeunea udarii M.Dey et D.K.Singh, Nelumbo 53: 197, 2011 (Dey and Singh 2011).

**Excluded from the genus**

* Lopholejeunea aberrantia Horik., J. Sci. Hiroshima Univ., Ser. B, Div. 2, Bot. 2: 256, 1934 (Horikawa 1934). ^365^

* Lopholejeunea vietnamica Tixier, Ann. Hist.-Nat. Mus. Natl. Hung. 66: 99, 1974 (Tixier 1974). ^366^

*** **Marchesinia Gray**, Nat. Arr. Brit. Pl. 1: 689, 1821 (Gray 1821) nom. conserv.

*** **subg. Marchesinia**

*** Marchesinia mackaii (Hook.) Gray, Nat. Arr. Brit. Pl. 1: 689, 1821 (Gray 1821). Bas.: Jungermannia mackaii Hook., Brit. Jungermann.: tab. 53, 1813 (Hooker 1813).

*** **subg. Marchesiniopsis R.M.Schust.**, J. Hattori Bot. Lab. 72: 358, 1992 (Schuster 1992a).

*** Marchesinia bongardiana (Lehm. et Lindenb.) Trevis., Mem. Reale Ist. Lombardo Sci. (Ser. 3), C. Sci. Mat. 4 (13): 405, 1877 (Trevisan 1877). Bas.: Lejeunea bongardiana Lehm. et Lindenb., Nov. Stirp. Pug. 7: 18, 1838 (Lehmann 1838).

*** Marchesinia brachiata (Sw.) Schiffn., Hepat. (Engl.-Prantl): 128, 1893 (Schiffner 1893b). Bas.: Jungermannia brachiata Sw., Prodr. (Swartz): 144, 1788 (Swartz 1788).

*** Marchesinia deslooveri Vanden Berghen, Rev. Bryol. Lichénol. 42 (4): 926, 1976 (Vanden Berghen 1976a).

*** Marchesinia excavata (Mitt.) Schiffn., Hepat. (Engl.-Prantl): 128, 1893 (Schiffner 1893b). Bas.: Lejeunea excavata Mitt., Trans. Linn. Soc. London 23 (1): 58, 1860 (Mitten 1860a).

*** Marchesinia languida (Nees et Mont.) Steph., Sp. Hepat. (Stephani) 5: 149, 1912 (Stephani 1912c). Bas.: Lejeunea languida Nees et Mont., Ann. Sci. Nat. Bot. (sér. 2) 5: 59, 1836 (Nees and Montagne 1836).

*** Marchesinia nobilis (Gottsche) X.Q.Shi, R.L.Zhu et Gradst., Phytotaxa 195 (3): 249, 2015 (Shi et al. 2015b). Bas.: Lejeunea nobilis Gottsche, Abh. Naturwiss. Vereins Bremen 7: 353, 1882 (Gottsche 1882).

*** Marchesinia robusta (Mitt.) Schiffn., Hepat. (Engl.-Prantl): 128, 1893 (Schiffner 1893b). Bas.: Lejeunea robusta Mitt., Hooker’s J. Bot. Kew Gard. Misc. 3: 359, 1851 (Mitten 1851).

** **Mastigolejeunea (Spruce) Steph.**, Hedwigia 30 (5): 206, 1891 (Stephani 1891a). Bas.: Lejeunea subg. Mastigolejeunea Spruce, Trans. & Proc. Bot. Soc. Edinburgh 15: 100, 1884 (Spruce 1884).

*** Mastigolejeunea auriculata (Wilson et Hook.) Steph., Bot. Gaz. 17 (6): 171, 1892 (Stephani 1892f). Bas.: Jungermannia auriculata Wilson et Hook., Musci Amer., S. States: no. 170, 1841 (Wilson 1841; non vidi).

** Mastigolejeunea auriculata var. rhodesica (Vanden Berghen) Sukkharak et Gradst., Nova Hedwigia 99 (3/4): 297, 2014 (Sukkharak and Gradstein 2014). Bas.: Brachiolejeunea rhodesica Vanden Berghen, Bull. Jard. Bot. État Bruxelles 21 (1/2): 94, 1951 (Vanden Berghen 1951d).

*** Mastigolejeunea calcarata (Steph.) Verd., Blumea 1 (1): 230, 1934 (Verdoorn 1934b). Bas.: Archilejeunea calcarata Steph., Sp. Hepat. (Stephani) 4: 724, 1911 (Stephani 1911e).

*** Mastigolejeunea florea (Mitt.) Paris, Rev. Bryol. 33 (3): 42, 1906 (Paris 1906b). Bas.: Phragmicoma florea Mitt., J. Linn. Soc., Bot. 22 (146): 323, 1886 (Mitten 1886b).

*** Mastigolejeunea frauenfeldii (Reichardt) Verd., Blumea 1 (1): 230, 1934 (Verdoorn 1934b). Bas.: Thysananthus frauenfeldii Reichardt, Verh. K.K. Zool.-Bot. Ges. Wien 16: 958, 1866 (Reichardt 1866).

** Mastigolejeunea gradsteinii Sukkharak, J. Bryol. 36 (1): 56, 2014 (Sukkharak 2014).

*** Mastigolejeunea humilis (Gottsche) Schiffn., Hepat. (Engl.-Prantl): 129, 1893 (Schiffner 1893b). Bas.: Phragmicoma humilis Gottsche, Syn. Hepat. 2: 299, 1845 (Gottsche et al. 1845a). ^367^

*** Mastigolejeunea indica Steph., Sp. Hepat. (Stephani) 4: 776, 1912 (Stephani 1912b).

*** Mastigolejeunea innovans (Spruce) Steph., Sp. Hepat. (Stephani) 4: 765, 1912 (Stephani 1912b). Bas.: Lejeunea innovans Spruce, Trans. & Proc. Bot. Soc. Edinburgh 15: 103, 1884 (Spruce 1884).

*** Mastigolejeunea ligulata (Lehm. et Lindenb.) Schiffn., Hepat. (Engl.-Prantl): 129, 1893 (Schiffner 1893b). Bas.: Jungermannia ligulata Lehm. et Lindenb., Nov. Stirp. Pug. 6: 39, 1834 (Lehmann 1834).

*** Mastigolejeunea nigra Steph., Hedwigia 30 (5): 206, 1891 (Stephani 1891a).

*** Mastigolejeunea plicatiflora (Spruce) Steph., Sp. Hepat. (Stephani) 4: 766, 1912 (Stephani 1912b). Bas.: Lejeunea plicatiflora Spruce, Trans. & Proc. Bot. Soc. Edinburgh 15: 104, 1884 (Spruce 1884).

*** Mastigolejeunea recondita (Steph.) Mizut., J. Hattori Bot. Lab. 32: 134, 1969 (Mizutani 1969). Bas.: Ptycholejeunea recondita Steph., Hedwigia 35 (3): 122, 1896 (Stephani 1896b).

*** Mastigolejeunea repleta (Taylor) A.Evans, Mem. Torrey Bot. Club 8 (2): 131, 1902 (Evans 1902a). Bas.: Lejeunea repleta Taylor, London J. Bot. 5: 392, 1846 (Taylor 1846b).

*** Mastigolejeunea truncata Mizut., J. Hattori Bot. Lab. 61: 292, 1986 [1987] (Mizutani 1986c).

** Mastigolejeunea turgida Steph., Hedwigia 31 (4): 170, 1892 (Stephani 1892g).

*** Mastigolejeunea virens (Ångstr.) Steph., Sp. Hepat. (Stephani) 4: 776, 1912 (Stephani 1912b), *nom. conserv*. Bas.: Thysananthus virens Ångstr., Öfvers. Kongl. Vetensk.-Akad. Förh. 30 (5): 131, 1873 (Ångström 1873).

** **Phaeolejeunea Mizut.**, J. Hattori Bot. Lab. 31: 130, 1968 (Mizutani 1968).

*** Phaeolejeunea amicorum (Hürl.) Pócs, Fieldiana, Bot. (n.ser.) 47: 140, 2008 (Pócs 2008b). Bas.: Phaeolejeunea etesseana subsp. amicorum Hürl., Bauhinia 9 (4): 263, 1991 (Hürlimann 1991).

** Phaeolejeunea etesseana (Steph.) Mizut., J. Hattori Bot. Lab. 31: 133, 1968 (Mizutani 1968). Bas.: Brachiolejeunea etesseana Steph., Sp. Hepat. (Stephani) 5: 133, 1912 (Stephani 1912c).

** Phaeolejeunea inermis (Steph.) Mizut., J. Hattori Bot. Lab. 31: 134, 1968 (Mizutani 1968). Bas.: Lopholejeunea inermis Steph., Sp. Hepat. (Stephani) 5: 92, 1912 (Stephani 1912c).

*** Phaeolejeunea latistipula (Schiffn. ex P.Syd.) Mizut., J. Hattori Bot. Lab. 31: 131, 1968 (Mizutani 1968). Bas.: Hygrolejeunea latistipula Schiffn. ex P.Syd., Just’s Bot. Jahresber. 19: 246, 1894 (Sydow 1894).

*** **Ptychanthus Nees**, Naturgesch. Eur. Leberm. 3: 211, 1838 (Nees 1838b).

** Ptychanthus africanus Steph., Wiss. Ergebn. Deut. Zentr.-Afr. Exped. (1907-08), Bot. 2: 131, 1911 (Stephani 1911a).

* Ptychanthus stephensonianus (Mitt.) Steph., Sp. Hepat. (Stephani) 4: 754, 1912 (Stephani 1912b). Bas.: Lejeunea stephensoniana Mitt., Bot. antarct. voy. II (Fl. Nov.-Zel. 2): 155, 1854 (Mitten 1854). ^368^

*** Ptychanthus striatus (Lehm. et Lindenb.) Nees, Naturgesch. Eur. Leberm. 3: 212, 1838 (Nees 1838b). Bas.: Jungermannia striata Lehm. et Lindenb., Nov. Stirp. Pug. 4: 16, 1832 (Lehmann 1832).

** Ptychanthus striatus var. intermedius (Gottsche) Verd., Ann. Bryol., Suppl. 4: 122, 1934 (Verdoorn 1934a). Bas.: Ptychanthus intermedius Gottsche, Natuurk. Tijdschr. Ned.-Indië 4: 576, 1853 (Gottsche 1853).

*** **Schiffneriolejeunea Verd.**, Ann. Bryol. 6: 89, 1933 (Verdoorn 1933a).

** **sect. Pappeanae R.M.Schust. ex Gradst. et Vanden Berghen**, Beih. Nova Hedwigia 80: 174, 1985 (Gradstein and Vanden Berghen 1985). Based on: Phragmilejeunea R.M.Schust., J. Hattori Bot. Lab. 11: 27, 1954 (Schuster and Hattori 1954).

*** Schiffneriolejeunea fragilis Gradst. et E.W.Jones, J. Bryol. 12 (1): 45, 1982 (Jones 1982).

*** Schiffneriolejeunea madagascariensis (Steph.) Gradst., J. Hattori Bot. Lab. 38: 333, 1974 (Gradstein 1974a). Bas.: Ptychocoleus madagascariensis Steph., Sp. Hepat. (Stephani) 5: 27, 1912 (Stephani 1912c).

*** Schiffneriolejeunea pappeana var. bidentata Gradst. et Vanden Berghen, Beih. Nova Hedwigia 80: 182, 1985 (Gradstein and Vanden Berghen 1985).

*** Schiffneriolejeunea pappeana var. integra Gradst. et Vanden Berghen, Beih. Nova Hedwigia 80: 182, 1985 (Gradstein and Vanden Berghen 1985).

*** Schiffneriolejeunea parviloba (Steph.) Gradst., J. Hattori Bot. Lab. 38: 335, 1974 (Gradstein 1974a). Bas.: Acrolejeunea parviloba Steph., Bot. Gaz. 15 (11): 286, 1890 (Stephani 1890c).

*** Schiffneriolejeunea pappeana (Nees) Gradst., J. Hattori Bot. Lab. 38: 335, 1974 (Gradstein 1974a). Bas.: Phragmicoma pappeana Nees, Syn. Hepat. 2: 296, 1845 (Gottsche et al. 1845a).

** **sect. Schiffneriolejeunea**, Occas. Pap. Farlow Herb. Cryptog. Bot. 16: 72, 1981 (Gradstein and Terken 1981).

*** Schiffneriolejeunea altimontana Vanden Berghen, Rev. Bryol. Lichénol. 42 (4): 923, 1976 (Vanden Berghen 1976a).

*** Schiffneriolejeunea amazonica Gradst., Beih. Nova Hedwigia 80: 25, 1985 (Gradstein 1985a).

*** Schiffneriolejeunea cumingiana (Mont.) Gradst., J. Hattori Bot. Lab. 38: 333, 1974 (Gradstein 1974a). Bas.: Phragmicoma cumingiana Mont., London J. Bot. 4: 7, 1845 (Montagne 1845a).

*** Schiffneriolejeunea ferruginea (Steph.) Gradst., J. Hattori Bot. Lab. 38: 333, 1974 (Gradstein 1974a). Bas.: Acrolejeunea ferruginea Steph., Hedwigia 34 (2): 57, 1895 (Stephani 1895c).

*** Schiffneriolejeunea occulta (Steph.) Gradst., J. Hattori Bot. Lab. 38: 333, 1974 (Gradstein 1974a). Bas.: Ptychocoleus occultus Steph., Sp. Hepat. (Stephani) 5: 25, 1912 (Stephani 1912c).

*** Schiffneriolejeunea omphalanthoides Verd., Ann. Bryol. 6: 91, 1933 (Verdoorn 1933c).

*** Schiffneriolejeunea tumida (Nees) Gradst., J. Hattori Bot. Lab. 38: 335, 1974 (Gradstein 1974a). Bas.: Ptychanthus tumidus Nees, Naturgesch. Eur. Leberm. 3: 213, 1838 (Nees 1838b).

*** Schiffneriolejeunea tumida var. haskarliana (Gottsche) Gradst. et Terken, Occas. Pap. Farlow Herb. Cryptog. Bot. 16: 77, 1981 (Gradstein and Terken 1981). Bas.: Phragmicoma haskarliana Gottsche, Syn. Hepat. 2: 299, 1845 (Gottsche et al. 1845a).

*** Schiffneriolejeunea nymannii (Steph.) Gradst. et Terken, Occas. Pap. Farlow Herb. Cryptog. Bot. 16: 79, 1981 (Gradstein and Terken 1981). Bas.: Archilejeunea nymannii Steph., Sp. Hepat. (Stephani) 4: 730, 1911 (Stephani 1911e).

*** Schiffneriolejeunea polycarpa (Nees) Gradst., J. Hattori Bot. Lab. 38: 335, 1974 (Gradstein 1974a). Bas.: Jungermannia polycarpa Nees, Fl. Bras. (Martius) 1 (1): 350, 1833 (Nees 1833a).

*** Schiffneriolejeunea pulopenangensis (Gottsche) Gradst., J. Hattori Bot. Lab. 38: 335, 1974 (Gradstein 1974a). Bas.: Phragmicoma pulopenangensis Gottsche, Syn. Hepat. 2: 299, 1845 (Gottsche et al. 1845a).

** **Spruceanthus Verd.**, Ann. Bryol., Suppl. 4: 151, 1934 (Verdoorn 1934a).

*** Spruceanthus macrostipulus (Steph.) Gradst., Trop. Bryol. 4: 13, 1991 (Gradstein 1991). Bas.: Mastigolejeunea macrostipula Steph., Sp. Hepat. (Stephani) 4: 767, 1912 (Stephani 1912b).

** Spruceanthus mamillilobulus (Herzog) Verd., Hepat. Select. Crit. 9: no. 447, 1936 (Verdoorn 1936; non vidi). Bas.: Ptychanthus mamillilobulus Herzog, Symb. Sin. 5: 44, 1930 (Nicholson et al. 1930).

*** Spruceanthus pluriplicatus (Steph.) Gradst., Schriftenreihe Mensch, Kultur Umwelt z. Bergl. W Neug. 7: 14, 1981 (Hiepko and Schultze-Motel 1981). Bas.: Brachiolejeunea pluriplicata Steph., Sp. Hepat. (Stephani) 5: 135, 1912 (Stephani 1912c).

*** Spruceanthus polymorphus (Sande Lac.) Verd., Ann. Bryol., Suppl. 4: 155, 1934 (Verdoorn 1934a). Bas.: Phragmicoma polymorpha Sande Lac., Ned. Kruidk. Arch. 3: 420, 1854 [1855] (Sande Lacoste 1854).

*** Spruceanthus semirepandus (Nees) Verd., Ann. Bryol., Suppl. 4: 153, 1934 (Verdoorn 1934a). Bas.: Jungermannia semirepanda Nees, Enum. Pl. Crypt. Javae: 39, 1830 (Nees 1830).

*** Spruceanthus sulcatus (Nees) Gradst., Beih. Nova Hedwigia 80: 26, 1985 (Gradstein 1985a). Bas.: Jungermannia sulcata Nees, Enum. Pl. Crypt. Javae: 36, 1830 (Nees 1830).

*** Spruceanthus theobromae (Spruce) Gradst., Beih. Nova Hedwigia 80: 26, 1985 (Gradstein 1985a). Bas.: Lejeunea theobromae Spruce, Trans. & Proc. Bot. Soc. Edinburgh 15: 99, 1884 (Spruce 1884).

*** Spruceanthus thozetianus (Gottsche et F.Muell.) B.M.Thiers et Gradst., Mem. New York Bot. Gard. 52: 62, 1989 (Thiers and Gradstein 1989). Bas.: Phragmicoma thozetiana Gottsche et F.Muell., Fragm. (Mueller): 63, 1880 (Gottsche 1880).

*** **Thysananthus Lindenb.**, Nov. Stirp. Pug. 8: 24, 1844 (Lehmann 1844).

** **sect. Thysananthus**

** **subsect. Anguiformes Sukkharak**, Phytotaxa 193 (1): 37, 2015 (Sukkharak 2015).

*** Thysananthus anguiformis (Hook.f. et Taylor) Gottsche, Lindenb. et Nees, Syn. Hepat. 2: 289, 1845 (Gottsche et al. 1845a). Bas.: Jungermannia anguiformis Hook.f. et Taylor, London J. Bot. 3: 567, 1844 (Hooker and Taylor 1844d).

*** Thysananthus pancheri (Steph.) Hürl., Bauhinia 9 (2): 167, 1989 (Hürlimann 1989). Bas.: Mastigolejeunea pancheri Steph., Sp. Hepat. (Stephani) 4: 771, 1912 (Stephani 1912b).

** **subsect. Thysananthus**

*** Thysananthus aculeatus Herzog, Ann. Bryol. 4: 89, 1931 (Herzog 1931b).

*** Thysananthus amazonicus (Spruce) Schiffn., Hepat. (Engl.-Prantl): 130, 1893 (Schiffner 1893b). Bas.: Lejeunea amazonica Spruce, Trans. & Proc. Bot. Soc. Edinburgh 15: 106, 1884 (Spruce 1884).

*** Thysananthus appendiculatus Steph., Sp. Hepat. (Stephani) 4: 794, 1912 (Stephani 1912b).

** Thysananthus ciliaris (Sande Lac.) Sukkharak, Nova Hedwigia 99 (3/4): 339, 2014 (Sukkharak and Gradstein 2014). Bas.: Phragmicoma ciliaris Sande Lac., Ann. Mus. Bot. Lugduno-Batavi 1: 307, 1864 (Sande Lacoste 1864).

*** Thysananthus comosus Lindenb., Nov. Stirp. Pug. 8: 25, 1844 (Lehmann 1844).

*** Thysananthus convolutus Lindenb., Syn. Hepat. 2: 288, 1845 (Gottsche et al. 1845a).

** Thysananthus convolutus var. laceratus (Steph.) Sukkharak, Phytotaxa 193 (1): 30, 2015 (Sukkharak 2015). Bas.: Thysananthus laceratus Steph., Sp. Hepat. (Stephani) 4: 796, 1912 (Stephani 1912b).

*** Thysananthus discretus Sukkharak et Gradst., Cryptog. Bryol. 31 (2): 113, 2010 (Sukkharak and Gradstein 2010).

*** Thysananthus gottschei (J.B.Jack et Steph.) Steph., Sp. Hepat. (Stephani) 4: 787, 1912 (Stephani 1912b). Bas.: Thysanolejeunea gottschei J.B.Jack et Steph., Hedwigia 31 (1): 20, 1892 (Jack and Stephani 1892).

** Thysananthus gottschei var. continuus Sukkharak, Phytotaxa 193 (1): 33, 2015 (Sukkharak 2015).

*** Thysananthus spathulistipus (Reinw., Blume et Nees) Lindenb., Syn. Hepat. 2: 287, 1845 (Gottsche et al. 1845a). Bas.: Jungermannia spathulistipa Reinw., Blume et Nees, Nova Acta Phys.-Med. Acad. Caes. Leop.-Carol. Nat. Cur. 12 (1): 212, 1824 [1825] (Reinwardt et al. 1824a).

** **sect. Vittatae Verd.**, Ann. Bryol., Suppl. 4: 182, 1934 (Verdoorn 1934a).

** **subsect. Sandeanthus (B.M.Thiers et Gradst.) Sukkharak**, Phytotaxa 193 (1): 43, 2015 (Sukkharak 2015). Bas.: Thysananthus subg. Sandeanthus B.M.Thiers et Gradst., Mem. New York Bot. Gard. 52: 66, 1989 (Thiers and Gradstein 1989).

*** Thysananthus mollis Steph., Sp. Hepat. (Stephani) 4: 798, 1912 (Stephani 1912b).

*** Thysananthus montanus Gradst., Xiao L.He et Piippo, Acta Bot. Fenn. 174: 77, 2002 (Gradstein et al. 2002).

*** Thysananthus retusus (Reinw., Blume et Nees) B.M.Thiers et Gradst., Mem. New York Bot. Gard. 52: 67, 1989 (Thiers and Gradstein 1989). Bas.: Jungermannia retusa Reinw., Blume et Nees, Nova Acta Phys.-Med. Acad. Caes. Leop.-Carol. Nat. Cur. 12 (1): 214, 1824 [1825] (Reinwardt et al. 1824a).

** Thysananthus retusus subsp. sellingii (Herzog) Sukkharak, Phytotaxa 193 (1): 47, 2015 (Sukkharak 2015). Bas.: Mastigolejeunea sellingii Herzog, Ark. Bot. (n.ser.) 3 (3): 60, 1953 (Herzog 1953a).

** **subsect. Vittatae (Verd.) Sukkharak**, Phytotaxa 193 (1): 40, 2015 (Sukkharak 2015). Bas.: Thysananthus sect. Vittatae Verd., Ann. Bryol., Suppl. 4: 182, 1934 (Verdoorn 1934a).

*** Thysananthus fruticosus (Lindenb. et Gottsche) Schiffn., Hepat. (Engl.-Prantl): 130, 1893 (Schiffner 1893b). Bas.: Bryopteris fruticosa Lindenb. et Gottsche, Syn. Hepat. 5: 737, 1847 (Gottsche et al. 1847).

** **Tuzibeanthus S.Hatt.**, Biosphaera 1 (1): 7, 1947 (Hattori 1947a).

*** Tuzibeanthus chinensis (Steph.) Mizut., J. Hattori Bot. Lab. 24: 151, 1961 (Mizutani 1961). Bas.: Ptychanthus chinensis Steph., Sp. Hepat. (Stephani) 4: 744, 1911 (Stephani 1911e).

** **Verdoornianthus Gradst.**, Bryologist 80 (4): 607, 1977 [1978] (Gradstein 1977).

*** Verdoornianthus griffinii Gradst., Bryologist 80 (4): 609, 1977 [1978] (Gradstein 1977).

*** Verdoornianthus marsupiifolius (Spruce) Gradst., Bryologist 80 (4): 609, 1977 [1978] (Gradstein 1977). Bas.: Lejeunea marsupiifolia Spruce, Trans. & Proc. Bot. Soc. Edinburgh 15: 118, 1884 (Spruce 1884).

####### Porellineae R.M.Schust.

######## *** Goebeliellaceae Verd.

by M. von Konrat and M.A.M. Renner

*** **Goebeliella Steph.**, Hedwigia 51 (1): 61, 1911 (Stephani 1911c).

*** Goebeliella cornigera (Mitt.) Steph., Hedwigia 51 (1): 62, 1911 (Stephani 1911c). Bas.: Frullania cornigera Mitt., Bot. antarct. voy. II (Fl. Nov.-Zel. 2): 163, 1855 (Mitten 1855).

######## *** Lepidolaenaceae Nakai

by M. von Konrat

The generic composition of Lepidolaenaceae follows Crandall-Stotler et al. (2009), except that the genus Jubulopsis was reduced to a synonym of Lepidolaena by von Konrat et al. (2012a).

*** **Gackstroemia Trevis.**, Mem. Reale Ist. Lombardo Sci. (Ser. 3), C. Sci. Mat. 4 (13): 397, 1877 (Trevisan 1877).

** **subg. Gackstroemia**

** Gackstroemia ljungneri (Herzog) Grolle, J. Hattori Bot. Lab. 30: 17, 1967 (Grolle 1967a). Bas.: Lepidolaena ljungneri Herzog, Ark. Bot. 29A (21): 6, 1940 (Herzog 1940).

*** Gackstroemia magellanica (Lam.) Trevis., Mem. Reale Ist. Lombardo Sci. (Ser. 3), C. Sci. Mat. 4 (13): 397, 1877 (Trevisan 1877). Bas.: Jungermannia magellanica Lam., Encycl. (Lamarck) 3 (1): 284, 1789 (Lamarck 1789).

*** Gackstroemia novae-zelandiae R.M.Schust. et J.J.Engel, Phytotaxa 118 (1): 10, 2013 (Engel 2013a).

*** Gackstroemia weindorferi (Herzog) Grolle, J. Hattori Bot. Lab. 30: 20, 1967 (Grolle 1967a). Bas.: Lepidolaena weindorferi Herzog, Ann. Bryol. 6: 103, 1933 (Verdoorn 1933a).

** **subg. Hariotiella (Besch. et C.Massal. ex Schiffn.) Grolle**, J. Hattori Bot. Lab. 30: 12, 1967 (Grolle 1967a). Bas.: Lepidolaena subg. Hariotiella Besch. et C.Massal. ex Schiffn., Hepat. (Engl.-Prantl): 110, 1893 (Schiffner 1893b).

*** Gackstroemia hariotiana (Besch. et C.Massal.) Grolle, J. Hattori Bot. Lab. 30: 12, 1967 (Grolle 1967a). Bas.: Polyotus hariotianus Besch. et C.Massal., Bull. Mens. Soc. Linn. Paris 1 (79): 639, 1886 (Bescherelle and Massalongo 1886).

*** Gackstroemia patagonica (Steph.) Grolle, J. Hattori Bot. Lab. 30: 14, 1967 (Grolle 1967a). Bas.: Lepidolaena patagonica Steph., Kungl. Svenska Vetensk.-Akad. Handl. (n.ser.) 46 (9): 76, 1911 (Stephani 1911b).

** Gackstroemia schwabei (Herzog) Grolle, J. Hattori Bot. Lab. 30: 16, 1967 (Grolle 1967a). Bas.: Lepidolaena schwabei Herzog, Rev. Bryol. Lichénol. 29 (3/4): 191, 1960 [1961] (Herzog 1960).

***Incertae sedis***

*** Gackstroemia alpina R.M.Schust., J. Hattori Bot. Lab. 36: 349, 1972 [1973] (Schuster 1972).

*** **Lepidogyna R.M.Schust.**, Phytologia 45 (5): 419, 1980 (Schuster 1980b).

*** Lepidogyna hodgsoniae (Grolle) R.M.Schust., Phytologia 45 (5): 419, 1980 (Schuster 1980b). Bas.: Lepidolaena hodgsoniae Grolle, J. Hattori Bot. Lab. 30: 29, 1967 (Grolle 1967a).

*** Lepidogyna menziesii (Hook.) R.M.Schust., Phytologia 45 (5): 419, 1980 (Schuster 1980b). Bas.: Jungermannia menziesii Hook., Musci Exot. 2: tab. 118, 1820 (Hooker 1820).

*** **Lepidolaena Dumort.**, Recueil Observ. Jungerm.: 13, 1835 (Dumortier 1835). ^369^

*** Lepidolaena berggrenii E.A.Hodgs., Trans. Roy. Soc. New Zealand 87 (3/4): 205, 1959 (Hodgson 1959).

** Lepidolaena brachyclada (Lehm.) Trevis., Mem. Reale Ist. Lombardo Sci. (Ser. 3), C. Sci. Mat. 4 (13): 393, 1877 (Trevisan 1877). Bas.: Frullania brachyclada Lehm., Nov. Stirp. Pug. 8: 21, 1844 (Lehmann 1844).

*** Lepidolaena clavigera (Hook.) Dumort. ex Trevis., Mem. Reale Ist. Lombardo Sci. (Ser. 3), C. Sci. Mat. 4 (13): 393, 1877 (Trevisan 1877). Bas.: Jungermannia clavigera Hook., Musci Exot. 1: tab. 70, 1818 (Hooker 1818).

*** Lepidolaena novae-zelandiae (E.A.Hodgs. et S.W.Arnell) von Konrat, L.Söderstr. et A.Hagborg, Phytotaxa 65: 51, 2012 (von Konrat et al. 2012a). Bas.: Jubula novae-zelandiae E.A.Hodgs. et S.W.Arnell, Trans. Roy. Soc. New Zealand, Bot. 3 (4): 90, 1965 (Hodgson 1965).

*** Lepidolaena palpebrifolia (Hook.) Dumort. ex Trevis., Mem. Reale Ist. Lombardo Sci. (Ser. 3), C. Sci. Mat. 4 (13): 393, 1877 (Trevisan 1877). Bas.: Jungermannia palpebrifolia Hook., Musci Exot. 1: tab. 71, 1818 (Hooker 1818).

*** Lepidolaena reticulata (Hook.f. et Taylor) Trevis., Mem. Reale Ist. Lombardo Sci. (Ser. 3), C. Sci. Mat. 4 (13): 393, 1877 (Trevisan 1877). Bas.: Jungermannia reticulata Hook.f. et Taylor, London J. Bot. 3: 395, 1844 (Hooker and Taylor 1844a).

*** Lepidolaena taylorii (Gottsche) Trevis., Mem. Reale Ist. Lombardo Sci. (Ser. 3), C. Sci. Mat. 4 (13): 393, 1877 (Trevisan 1877). Bas.: Polyotus taylorii Gottsche, Syn. Hepat. 2: 246, 1845 (Gottsche et al. 1845a).

######## *** Porellaceae Cavers *nom. conserv.*

*** **Ascidiota C.Massal.**, Nuovo Giorn. Bot. Ital. (n.ser.) 5 (2): 256, 1898 (Massalongo 1898).

*** Ascidiota blepharophylla C.Massal., Nuovo Giorn. Bot. Ital. (n.ser.) 5 (2): 257, 1898 (Massalongo 1898).

** Ascidiota blepharophylla subsp. alaskana Steere et R.M.Schust., Bull. Torrey Bot. Club 87 (3): 213, 1960 (Steere and Schuster 1960).

*** **Porella L.**, Sp. Pl. 1: 1106, 1753 (Linnaeus 1753).

** Porella abyssinica (Nees) Trevis., Mem. Reale Ist. Lombardo Sci. (Ser. 3), C. Sci. Mat. 4 (13): 407, 1877 (Trevisan 1877). Bas.: Madotheca abyssinica Nees, Syn. Hepat. 2: 281, 1845 (Gottsche et al. 1845a).

** Porella abyssinica var. hoehnelii (Steph.) Pócs, Fragm. Florist. Geobot. 39 (1): 229, 1994 (Pócs 1994a). Bas.: Porella hoehnelii Steph., Hedwigia 30 (6): 266, 1891 (Stephani 1891c).

** Porella acutifolia (Lehm. et Lindenb.) Trevis., Mem. Reale Ist. Lombardo Sci. (Ser. 3), C. Sci. Mat. 4 (13): 408, 1877 (Trevisan 1877). Bas.: Madotheca acutifolia Lehm. et Lindenb., Nov. Stirp. Pug. 7: 8, 1838 (Lehmann 1838).

** Porella acutifolia var. hattoriana (Pócs) S.Hatt., Misc. Bryol. Lichenol. 8 (4): 79, 1979 (Hattori 1979c). Bas.: Porella plumosa var. hattoriana Pócs, J. Hattori Bot. Lab. 31: 82, 1968 (Pócs 1968).

* Porella acutifolia var. linguifolia (Steph.) M.L.So, Syst. Bot. 27 (1): 5, 2002 (So 2002a). Bas.: Madotheca linguifolia Steph., Sp. Hepat. (Stephani) 4: 291, 1910 (Stephani 1910b). ^370^

** Porella acutifolia subsp. tosana (Steph.) S.Hatt., J. Hattori Bot. Lab. 44: 100, 1978 (Hattori 1978a). Bas.: Madotheca tosana Steph., Bull. Herb. Boissier 5 (2): 97, 1897 (Stephani 1897b).

* Porella andica (Gottsche) Hässel, Beih. Nova Hedwigia 134: 452, 2009 (Hässel and Rubies 2009). Bas.: Madotheca andica Gottsche, Ann. Sci. Nat. Bot. (sér. 4) 8: 339, 1857 (Gottsche 1857). ^371^

*** Porella arboris-vitae (With.) Grolle, Trans. Brit. Bryol. Soc. 5 (4): 770, 1969 (Grolle 1969b). Bas.: Jungermannia arboris-vitae With., Bot. arr. veg. Gr. Brit. 2: 697, 1776 (Withering 1776).

** Porella arboris-vitae subsp. nitidula (C.Massal.) S.Hatt., J. Hattori Bot. Lab. 40: 123, 1976 (Hattori 1976f). Bas.: Madotheca nitidula C.Massal., Bull. Soc. Bot. Ital. 1906: 141, 1906 (Massalongo 1906b).

** Porella baueri (Schiffn.) C.E.O.Jensen, Danmarks mosser: 240, 1915 (Jensen 1915). Bas.: Madotheca baueri Schiffn., Sitzungsber. deutsch. naturwiss.-med. Vereins Böhmen “Lotos” Prag 48: 346, 1900 (Schiffner 1900d).

** Porella bolanderi (Austin) Pearson, List. Canad. Hepat.: 7, 1890 (Pearson 1890). Bas.: Madotheca bolanderi Austin, Bull. Torrey Bot. Club 3 (3): 14, 1872 (Austin 1872).

** Porella borellii (Gola) Parihar, Univ. Allahabad Stud., Bot. 1961-2: 15, 1962 (Parihar 1962). Bas.: Madotheca borellii Gola, Atti Reale Accad. Sci. Torino, Cl. Sci. Fis. Mat. Nat. 49: 760, 1914 (Gola 1914b).

*** Porella brachiata (Taylor) Spruce, Trans. & Proc. Bot. Soc. Edinburgh 15: 334, 1885 (Spruce 1885). Bas.: Madotheca brachiata Taylor, London J. Bot. 6: 341, 1847 (Taylor 1847b).

*** Porella brasiliensis (Raddi) Schiffn., Nova Acta Acad. Caes. Leop.-Carol. German. Nat. Cur. 60 (2): 246, 1893 (Schiffner 1893a). Bas.: Schulthesia brasiliensis Raddi, Critt. Brasil.: 10, 1822 (Raddi 1822).

* Porella brasiliensis var. ciliata (Gottsche, Lindenb. et Nees) Schiffn., Nova Acta Acad. Caes. Leop.-Carol. German. Nat. Cur. 60 (2): 246, 1893 (Schiffner 1893a). Bas.: Madotheca brasiliensis β ciliata Gottsche, Lindenb. et Nees, Syn. Hepat. 2: 271, 1845 (Gottsche et al. 1845a).

* Porella brasiliensis var. laevior (Gottsche, Lindenb. et Nees) Schiffn., Nova Acta Acad. Caes. Leop.-Carol. German. Nat. Cur. 60 (2): 246, 1893 (Schiffner 1893a). Bas.: Madotheca brasiliensis α laevior Gottsche, Lindenb. et Nees, Syn. Hepat. 2: 271, 1845 (Gottsche et al. 1845a).

** Porella caespitans (Steph.) S.Hatt., J. Hattori Bot. Lab. 33: 50, 1970 (Hattori 1970). Bas.: Madotheca caespitans Steph., Mém. Soc. Nat. Sci. Nat. Math. Cherbourg 29: 218, 1894 (Stephani 1894b). ^372^

** Porella caespitans var. cordifolia (Steph.) S.Hatt. ex T.Katag. et T.Yamag., Bryol. Res. 10 (5): 133, 2011 (Katagiri and Yamaguchi 2011). Bas.: Madotheca cordifolia Steph., Sp. Hepat. (Stephani) 4: 315, 1910 (Stephani 1910b).

** Porella caespitans subsp. latior (S.Hatt.) S.Hatt., J. Hattori Bot. Lab. 40: 127, 1976 (Hattori 1976f). Bas.: Porella acutifolia subsp. latior S.Hatt., J. Hattori Bot. Lab. 32: 325, 1969 (Hattori 1969).

** Porella caespitans var. nipponica S.Hatt., J. Hattori Bot. Lab. 33: 57, 1970 (Hattori 1970).

** Porella caespitans var. reflexigastria (Pócs) S.Hatt., J. Hattori Bot. Lab. 40: 127, 1976 (Hattori 1976f). Bas.: Porella reflexigastria Pócs, J. Hattori Bot. Lab. 31: 71, 1968 (Pócs 1968).

** Porella campylophylla (Lehm. et Lindenb.) Trevis., Mem. Reale Ist. Lombardo Sci. (Ser. 3), C. Sci. Mat. 4 (13): 408, 1877 (Trevisan 1877). Bas.: Jungermannia campylophylla Lehm. et Lindenb., Nov. Stirp. Pug. 6: 40, 1834 (Lehmann 1834).

** Porella campylophylla subsp. lancistipula (Steph.) S.Hatt., J. Hattori Bot. Lab. 44: 102, 1978 (Hattori 1978a). Bas.: Madotheca lancistipula Steph., Sp. Hepat. (Stephani) 6: 524, 1924 (Stephani 1924).

** Porella campylophylla var. ligulifera (Taylor) S.Hatt., J. Hattori Bot. Lab. 32: 333, 1969 (Hattori 1969). Bas.: Madotheca ligulifera Taylor, Nov. Stirp. Pug. 8: 10, 1844 (Lehmann 1844).

** Porella campylophylla var. tixieri (Pócs) S.Hatt., J. Hattori Bot. Lab. 40: 128, 1976 (Hattori 1976f). Bas.: Porella plumosa var. tixieri Pócs, J. Hattori Bot. Lab. 31: 82, 1968 (Pócs 1968).

** Porella canariensis (F.Weber) Underw., Rep. (Annual) Missouri Bot. Gard. 8: 186, 1897 (Trelease 1897). Bas.: Jungermannia platyphylla var. canariensis F.Weber, Hist. Musc. Hepat. Prodr.: 16, 1815 (Weber 1815).

* Porella capehorniensis Swails, Nova Hedwigia 19: 244, 1970 (Swails 1970). ^373^

** Porella capensis (Gottsche) Mitt., J. Linn. Soc., Bot. 22 (146): 323, 1886 (Mitten 1886b). Bas.: Madotheca capensis Gottsche, Syn. Hepat. 2: 270, 1845 (Gottsche et al. 1845a).

* Porella caucasica Steph., Bot. Centralbl. 50 (3): 71, 1892 (Stephani 1892a).

** Porella chenii S.Hatt., J. Hattori Bot. Lab. 30: 129, 1967 (Hattori 1967).

*** Porella chilensis (Lehm. et Lindenb.) Trevis., Mem. Reale Ist. Lombardo Sci. (Ser. 3), C. Sci. Mat. 4 (13): 407, 1877 (Trevisan 1877). Bas.: Jungermannia chilensis Lehm. et Lindenb., Nov. Stirp. Pug. 6: 36, 1834 (Lehmann 1834).

* Porella chilensis var. antucensis (Gottsche) Hässel, Beih. Nova Hedwigia 134: 452, 2009 (Hässel and Rubies 2009). Bas.: Madotheca chilensis β antucensis Gottsche, Ann. Sci. Nat. Bot. (sér. 4) 8: 340, 1857 (Gottsche 1857).

** Porella chilensis var. fernandeziensis (Herzog) Swails, Nova Hedwigia 19: 236, 1970 (Swails 1970). Bas.: Madotheca chilensis f. fernandeziensis Herzog, Nat. Hist. Juan Fernandez (Botany) 2 (5): 736, 1942 (Herzog 1942a).

** Porella chilensis var. microloba (Herzog) Swails, Nova Hedwigia 19: 236, 1970 (Swails 1970). Bas.: Madotheca chilensis f. microloba Herzog, Nat. Hist. Juan Fernandez (Botany) 2 (5): 736, 1942 (Herzog 1942a).

** Porella chinensis (Steph.) S.Hatt., J. Hattori Bot. Lab. 30: 131, 1967 (Hattori 1967). Bas.: Madotheca chinensis Steph., Mém. Soc. Nat. Sci. Nat. Math. Cherbourg 29: 218, 1894 (Stephani 1894b).

** Porella chinensis var. crispata Udar et Shaheen, Misc. Bryol. Lichenol. 9 (4): 74, 1982 (Udar and Shaheen 1982).

** Porella chinensis var. decurrens (Steph.) S.Hatt., J. Hattori Bot. Lab. 44: 102, 1978 (Hattori 1978a). Bas.: Madotheca decurrens Steph., Sp. Hepat. (Stephani) 4: 289, 1910 (Stephani 1910b).

** Porella chinensis var. hattorii Udar et Shaheen, Misc. Bryol. Lichenol. 9 (7): 146, 1983 (Udar and Shaheen 1983a).

** Porella chinensis var. irregularis (Steph.) S.Hatt., J. Hattori Bot. Lab. 39: 270, 1975 (Hattori 1975c). Bas.: Madotheca irregularis Steph., Sp. Hepat. (Stephani) 4: 304, 1910 (Stephani 1910b).

** Porella circinnata Lindb., Not. Sällsk. Fauna Fl. Fenn. Förh. 13: 355, 1874 (Lindberg 1874a).

*** Porella cordaeana (Huebener) Moore, Proc. Roy. Irish Acad. (ser. 2) 2: 618, 1877 (Moore 1877). Bas.: Jungermannia cordaeana Huebener, Hepaticol. germ.: 291, 1834 (Hübener 1834).

** Porella cranfordii Steph., Hedwigia 28 (4): 270, 1889 (Stephani 1889c).

*** Porella crispata (Hook.) Trevis., Mem. Reale Ist. Lombardo Sci. (Ser. 3), C. Sci. Mat. 4 (13): 407, 1877 (Trevisan 1877). Bas.: Jungermannia crispata Hook., Pl. crypt. (Hooker): tab. 4b, 1816 (Hooker 1816b).

** Porella cucullistipula Steph., Bull. Soc. Roy. Bot. Belgique, Compt. Rend. 32 (2): 38, 1893 [1894] (Stephani 1893e).

** Porella densifolia (Steph.) S.Hatt., J. Jap. Bot. 20: 109, 1944 (Hattori 1944c). Bas.: Madotheca densifolia Steph., Mém. Soc. Nat. Sci. Nat. Math. Cherbourg 29: 219, 1894 (Stephani 1894b). ^374^

** Porella densifolia subsp. andamana S.Hatt., J. Hattori Bot. Lab. 32: 346, 1969 (Hattori 1969).

** Porella densifolia subsp. appendiculata (Steph.) S.Hatt., J. Hattori Bot. Lab. 32: 343, 1969 (Hattori 1969). Bas.: Madotheca appendiculata Steph., Sp. Hepat. (Stephani) 4: 301, 1910 (Stephani 1910b).

** Porella densifolia var. oviloba (Steph.) N.Kitag., Acta Phytotax. Geobot. 19 (2/3): 64, 1962 (Kitagawa 1962b). Bas.: Madotheca oviloba Steph., Sp. Hepat. (Stephani) 4: 312, 1910 (Stephani 1910b).

** Porella densifolia var. paraphyllina (P.C.Chen) Pócs, J. Hattori Bot. Lab. 31: 84, 1968 (Pócs 1968). Bas.: Madotheca paraphyllina P.C.Chen, Feddes Repert. Spec. Nov. Regni Veg. 58: 42, 1955 (Chen 1955).

** Porella densifolia var. pilosa S.Hatt. et K.C.Chang, Bull. Bot. Res., Harbin 8 (2): 44, 1988 (Chang 1988).

** Porella densifolia var. robusta (Steph.) S.Hatt., J. Hattori Bot. Lab. 32: 343, 1969 (Hattori 1969). Bas.: Madotheca robusta Steph., Sp. Hepat. (Stephani) 4: 313, 1910 (Stephani 1910b).

*** Porella elegantula (Mont.) E.A.Hodgs., Svensk Bot. Tidskr. 42 (3): 277, 1948 (Hodgson and Sainsbury 1948). Bas.: Madotheca elegantula Mont., Ann. Sci. Nat. Bot. (sér. 2) 19: 255, 1843 (Montagne 1843).

** Porella faurieri (Steph.) S.Hatt., J. Jap. Bot. 20: 109, 1944 (Hattori 1944c). Bas.: Madotheca faurieri Steph., Sp. Hepat. (Stephani) 4: 315, 1910 (Stephani 1910b).

** Porella fengii P.C.Chen et S.Hatt., J. Hattori Bot. Lab. 30: 133, 1967 (Hattori 1967).

** Porella geheebii (Steph.) S.Hatt., Bot. Mag. (Tokyo) 64 (755/756): 114, 1951 (Hattori 1951c). Bas.: Madotheca geheebii Steph., Sp. Hepat. (Stephani) 4: 290, 1910 (Stephani 1910b).

** Porella gracillima Mitt., Trans. Linn. Soc. London, Bot. 3 (3): 202, 1891 (Mitten 1891).

** Porella grandifolia (Steph.) S.Hatt., J. Hattori Bot. Lab. 30: 136, 1967 (Hattori 1967). Bas.: Madotheca grandifolia Steph., Sp. Hepat. (Stephani) 4: 289, 1910 (Stephani 1910b).

** Porella grandiloba Lindb., Acta Soc. Sci. Fenn. 10: 234, 1872 [1873] (Lindberg 1872b).

** Porella grollei S.Hatt., J. Hattori Bot. Lab. 34: 411, 1971 (Hattori 1971b).

** Porella handelii S.Hatt., J. Hattori Bot. Lab. 33: 65, 1970 (Hattori 1970).

** Porella hattorii Udar et Shaheen, Lindbergia 9 (1): 70, 1983 (Udar and Shaheen 1983b).

** Porella hoeana S.Hatt., Misc. Bryol. Lichenol. 7 (5): 86, 1976 (Hattori 1976c).

* Porella imbricata Lour., Fl. Cochinch. 2: 683, 1790 (Loureiro 1790).

** Porella inaequalis (Gottsche) Perss., Arch. Soc. Zool. Bot. Fenn. “Vanamo”, suppl. 9: 225, 1955 (Persson 1955). Bas.: Madotheca inaequalis Gottsche, Sp. Hepat. (Stephani) 4: 251, 1910 (Stephani 1910b).

** Porella japonica (Sande Lac.) Mitt., Trans. Linn. Soc. London, Bot. 3 (3): 202, 1891 (Mitten 1891). Bas.: Madotheca japonica Sande Lac., Syn. hepat. jav.: 105, 1856 [1857] (Sande Lacoste 1856b).

** Porella japonica subsp. appalachiana R.M.Schust., Hepat. Anthocerotae N. Amer. 4: 682, 1980 (Schuster 1980c).

** Porella japonica var. densespinosa S.Hatt. et M.X.Zhang, J. Jap. Bot. 60 (11): 324, 1985 (Hattori and Zhang 1985).

** Porella javanica (Gottsche) Inoue, J. Hattori Bot. Lab. 30: 60, 1967 (Inoue 1967a). Bas.: Madotheca javanica Gottsche, Sp. Hepat. (Stephani) 4: 290, 1910 (Stephani 1910b).

** Porella latifolia J.S.Lou et Q.Li, Acta Phytotax. Sin. 25 (6): 482, 1987 (Lou 1987).

*** Porella leiboldii (Lehm.) Trevis., Mem. Reale Ist. Lombardo Sci. (Ser. 3), C. Sci. Mat. 4 (13): 407, 1877 (Trevisan 1877). Bas.: Madotheca leiboldii Lehm., Nov. Stirp. Pug. 8: 11, 1844 (Lehmann 1844).

** Porella longifolia (Steph.) S.Hatt., J. Hattori Bot. Lab. 32: 351, 1969 (Hattori 1969). Bas.: Madotheca longifolia Steph., Sp. Hepat. (Stephani) 4: 305, 1910 (Stephani 1910b).

** Porella madagascariensis (Nees et Mont.) Trevis., Mem. Reale Ist. Lombardo Sci. (Ser. 3), C. Sci. Mat. 4 (13): 407, 1877 (Trevisan 1877). Bas.: Lejeunea madagascariensis Nees et Mont., Ann. Sci. Nat. Bot. (sér. 2) 5: 57, 1836 (Nees and Montagne 1836).

* Porella maxima (Steph.) M.L.So, Syst. Bot. 27 (1): 11, 2002 (So 2002a). Bas.: Madotheca maxima Steph., Sp. Hepat. (Stephani) 4: 291, 1910 (Stephani 1910b). ^375^

*** Porella mexicana (Hampe ex Gottsche, Lindenb. et Nees) Trevis., Mem. Reale Ist. Lombardo Sci. (Ser. 3), C. Sci. Mat. 4 (13): 407, 1877 (Trevisan 1877). Bas.: Madotheca mexicana Hampe ex Gottsche, Lindenb. et Nees, Syn. Hepat. 2: 273, 1845 (Gottsche et al. 1845a).

** Porella montantii (Steph.) E.W.Jones, Trans. Brit. Bryol. Soc. 4 (3): 460, 1963 (Jones 1963). Bas.: Madotheca montantii Steph., Sp. Hepat. (Stephani) 4: 259, 1910 (Stephani 1910b).

*** Porella navicularis (Lehm. et Lindenb.) Pfeiff., Fl. Niederhessen 2: 234, 1855 (Pfeiffer 1855). Bas.: Jungermannia navicularis Lehm. et Lindenb., Nov. Stirp. Pug. 6: 38, 1834 (Lehmann 1834).

** Porella nitens (Steph.) S.Hatt., Fl. E. Himalaya: 525, 1966 (Hattori 1966c). Bas.: Madotheca nitens Steph., Mém. Soc. Nat. Sci. Nat. Math. Cherbourg 29: 220, 1894 (Stephani 1894b).

** Porella oblongifolia S.Hatt., J. Jap. Bot. 19 (7): 200, 1943 (Hattori 1943c). ^376^

*** Porella obtusata (Taylor) Trevis., Mem. Reale Ist. Lombardo Sci. (Ser. 3), C. Sci. Mat. 4 (13): 407, 1877 (Trevisan 1877). Bas.: Madotheca obtusata Taylor, London J. Bot. 5: 380, 1846 (Taylor 1846b).

** Porella obtusata var. macroloba (Steph.) S.Hatt. et M.X.Zhang, J. Jap. Bot. 60 (11): 325, 1985 (Hattori and Zhang 1985). Bas.: Madotheca macroloba Steph., Sp. Hepat. (Stephani) 4: 292, 1910 (Stephani 1910b).

** Porella obtusiloba S.Hatt., J. Hattori Bot. Lab. 33: 69, 1970 (Hattori 1970).

** Porella perrottetiana (Mont.) Trevis., Mem. Reale Ist. Lombardo Sci. (Ser. 3), C. Sci. Mat. 4 (13): 408, 1877 (Trevisan 1877). Bas.: Madotheca perrottetiana Mont., Ann. Sci. Nat. Bot. (sér. 2) 18: 15, 1842 (Montagne 1842b).

** Porella perrottetiana var. angustifolia Pócs, J. Hattori Bot. Lab. 31: 75, 1968 (Pócs 1968).

** Porella perrottetiana var. ciliatodentata (P.C.Chen et P.C.Wu) S.Hatt., J. Hattori Bot. Lab. 30: 144, 1967 (Hattori 1967). Bas.: Porella ciliatodentata P.C.Chen et P.C.Wu, Obs. fl. Hwangs.: 8, 1965 (Chen and Wu 1965).

** Porella perrottetiana var. triciliata (Steph.) Pócs, J. Hattori Bot. Lab. 31: 75, 1968 (Pócs 1968). Bas.: Madotheca triciliata Steph., Sp. Hepat. (Stephani) 4: 308, 1910 (Stephani 1910b).

*** Porella pinnata L., Sp. Pl. 1: 1106, 1753 (Linnaeus 1753).

** Porella planifolia J.S.Lou, Coll. Pap. Quing-Zang Huang-Den 1: 277, 1983 (Lou and Wang 1983).

*** Porella platyphylla (L.) Pfeiff., Fl. Niederhessen 2: 234, 1855 (Pfeiffer 1855). Bas.: Jungermannia platyphylla L., Sp. Pl. 1: 1134, 1753 (Linnaeus 1753).

*** Porella platyphylloidea (Schwein.) Lindb., Morgonbladet (Helsinki) 1876 (287, 10 Dec.): 1, 1876 (Lindberg 1876c). Bas.: Jungermannia platyphylloidea Schwein., Spec. Fl. Amer. Crypt.: 9, 1821 (Schweinitz 1821).

** Porella plicata J.S.Lou, Acta Phytotax. Sin. 18 (1): 119, 1980 (Lou and Wu 1980).

** Porella plumosa (Mitt.) Parihar, Univ. Allahabad Stud., Bot. 1961-2: 17, 1962 (Parihar 1962). Bas.: Madotheca plumosa Mitt., J. Proc. Linn. Soc., Bot. 5 (18): 108, 1860 [1861] (Mitten 1860c).

** Porella prolixa (Gottsche) E.W.Jones, Trans. Brit. Bryol. Soc. 4 (3): 460, 1963 (Jones 1963). Bas.: Madotheca prolixa Gottsche, Sp. Hepat. (Stephani) 4: 260, 1910 (Stephani 1910b).

** Porella pulcherrima Herzog et S.Hatt., Bull. Natl. Sci. Mus. Tokyo, B 12 (1): 34, 1986 (Hattori 1986a).

*** Porella reflexa (Lehm. et Lindenb.) Trevis., Mem. Reale Ist. Lombardo Sci. (Ser. 3), C. Sci. Mat. 4 (13): 408, 1877 (Trevisan 1877). Bas.: Jungermannia reflexa Lehm. et Lindenb., Nov. Stirp. Pug. 5: 5, 1833 (Lehmann 1833).

** Porella revoluta (Lehm. et Lindenb.) Trevis., Mem. Reale Ist. Lombardo Sci. (Ser. 3), C. Sci. Mat. 4 (13): 407, 1877 (Trevisan 1877). Bas.: Jungermannia revoluta Lehm. et Lindenb., Nov. Stirp. Pug. 4: 18, 1832 (Lehmann 1832).

** Porella revoluta var. propinqua (C.Massal.) S.Hatt., J. Hattori Bot. Lab. 30: 148, 1967 (Hattori 1967). Bas.: Madotheca propinqua C.Massal., Hepat. Shen-si: 27, 1897 (Massalongo 1897).

** Porella roellii Steph., Bot. Centralbl. 45: 203, 1891 (Röll 1891).

*** Porella saccata M.L.So, New Zealand J. Bot. 43 (1): 302, 2005 (So 2005b).

** Porella sichuanensis S.Hatt. et K.C.Chang, Bull. Bot. Res., Harbin 8 (2): 43, 1988 (Chang 1988).

** Porella spinulosa (Steph.) S.Hatt., J. Hattori Bot. Lab. 33: 74, 1970 (Hattori 1970). Bas.: Madotheca spinulosa Steph., Sp. Hepat. (Stephani) 6: 529, 1924 (Stephani 1924).

*** Porella squamulifera (Taylor) Trevis., Mem. Reale Ist. Lombardo Sci. (Ser. 3), C. Sci. Mat. 4 (13): 407, 1877 (Trevisan 1877). Bas.: Madotheca squamulifera Taylor, London J. Bot. 5: 378, 1846 (Taylor 1846b).

** Porella stephaniana (C.Massal.) S.Hatt., J. Hattori Bot. Lab. 5: 81, 1951 (Hattori 1951b). Bas.: Madotheca stephaniana C.Massal., Hepat. Shen-si: 23, 1897 (Massalongo 1897). ^377^

** Porella subdentata (Mitt.) Steph., Hedwigia 30 (5): 203, 1891 (Stephani 1891a). Bas.: Madotheca subdentata Mitt., J. Proc. Linn. Soc., Bot. 7 (27): 167, 1863 (Mitten 1863).

** Porella subdentata var. camerunensis E.W.Jones, Trans. Brit. Bryol. Soc. 4 (3): 456, 1963 (Jones 1963).

** Porella subobtusa (Steph.) S.Hatt., J. Jap. Bot. 20: 111, 1944 (Hattori 1944c). Bas.: Madotheca subobtusa Steph., Sp. Hepat. (Stephani) 4: 311, 1910 (Stephani 1910b).

** Porella subparaphyllina J.S.Lou, Acta Phytotax. Sin. 25 (6): 483, 1987 (Lou 1987).

*** Porella subsquarrosa (Nees et Mont.) Trevis., Mem. Reale Ist. Lombardo Sci. (Ser. 3), C. Sci. Mat. 4 (13): 407, 1877 (Trevisan 1877). Bas.: Lejeunea subsquarrosa Nees et Mont., Ann. Sci. Nat. Bot. (sér. 2) 5: 57, 1836 (Nees and Montagne 1836).

*** Porella swailsii Grolle, J. Bryol. 10 (3): 270, 1979 (Grolle 1979b). *Nom. nov. pro Madotheca apiculata* Herzog, Feddes Repert. Spec. Nov. Regni Veg. 57 (1/2): 197, 1955 (Herzog 1955).

*** Porella swartziana (F.Weber) Trevis., Mem. Reale Ist. Lombardo Sci. (Ser. 3), C. Sci. Mat. 4 (13): 407, 1877 (Trevisan 1877). Bas.: Jungermannia swartziana F.Weber, Hist. Musc. Hepat. Prodr.: 18, 1815 (Weber 1815).

** Porella triquetra (Steph.) E.W.Jones, Trans. Brit. Bryol. Soc. 4 (3): 454, 1963 (Jones 1963). Bas.: Madotheca triquetra Steph., Bot. Jahrb. Syst. 20 (3): 321, 1895 (Stephani 1895a).

** Porella truncata J.S.Lou, Acta Phytotax. Sin. 18 (1): 119, 1980 (Lou and Wu 1980).

** Porella ulophylla (Steph.) S.Hatt., Bull. Tokyo Sci. Mus. 11: 92, 1944 (Hattori 1944d). Bas.: Madotheca ulophylla Steph., Bull. Herb. Boissier 5 (2): 97, 1897 (Stephani 1897b).

** Porella undatorevoluta J.S.Lou, Acta Phytotax. Sin. 25 (6): 485, 1987 (Lou 1987).

** Porella urceolata S.Hatt., J. Hattori Bot. Lab. 33: 66, 1970 (Hattori 1970).

** Porella urogea (C.Massal.) S.Hatt., J. Hattori Bot. Lab. 32: 349, 1969 (Hattori 1969). Bas.: Madotheca urogea C.Massal., Hepat. Shen-si: 28, 1897 (Massalongo 1897).

** Porella vallis-gratiae (Gottsche) E.W.Jones, Trans. Brit. Bryol. Soc. 4 (3): 450, 1963 (Jones 1963). Bas.: Madotheca vallis-gratiae Gottsche, Sp. Hepat. (Stephani) 4: 261, 1910 (Stephani 1910b).

** Porella variabilis (Kashyap et R.S.Chopra) Parihar, Univ. Allahabad Stud., Bot. 1961-2: 17, 1962 (Parihar 1962). Bas.: Madotheca variabilis Kashyap et R.S.Chopra, Liverworts W. Himal. 2: 33, 1932 (Kashyap and Chopra 1932).

** Porella vernicosa Lindb., Acta Soc. Sci. Fenn. 10: 223, 1872 [1873] (Lindberg 1872b).

** Porella viridissima (Mitt.) Grolle, J. Hattori Bot. Lab. 36: 83, 1972 [1973] (Grolle and Schultze-Motel 1972). Bas.: Madotheca viridissima Mitt., Fl. vit.: 411, 1871 [1873] (Mitten 1871).

** Porella wataugensis (Sull.) Underw. ex M.Howe, Bull. Torrey Bot. Club 24 (11): 519, 1897 (Howe 1897b). Bas.: Madotheca wataugensis Sull., Musc. Hepat. U.S.: 700, 1856 (Sullivant 1856).

####### Radulineae R.M.Schust.

######## *** Radulaceae Müll.Frib.

by M.A.M. Renner

The treatment of Radulaceae follows Devos et al. (2011). Taxonomic and nomenclatural notes can also be found in Renner et al. (2013b, 2014).

*** **Radula Dumort.**, Commentat. Bot. (Dumortier): 112, 1822 (Dumortier 1822) nom. conserv.

*** **subg. Amentuloradula Devos, M.A.M.Renner, Gradst., A.J.Shaw et Vanderp.**, Taxon 60 (6): 1630, 2011 (Devos et al. 2011).

*** Radula amentulosa Mitt., Bonplandia 9 (24): 367, 1861 (Mitten 1861).

*** Radula aneurismalis (Hook.f. et Taylor) Gottsche, Lindenb. et Nees, Syn. Hepat. 2: 262, 1845 (Gottsche et al. 1845a). Bas.: Jungermannia aneurismalis Hook.f. et Taylor, London J. Bot. 4: 86, 1845 (Hooker and Taylor 1845).

** Radula ceylanica K.Yamada, J. Jap. Bot. 50 (12): 373, 1975 (Yamada 1975b).

*** Radula fissifolia Steph., Sp. Hepat. (Stephani) 6: 507, 1924 (Stephani 1924).

*** Radula formosa (C.F.W.Meissn. ex Spreng.) Nees, Syn. Hepat. 2: 258, 1845 (Gottsche et al. 1845a). Bas.: Jungermannia formosa C.F.W.Meissn. ex Spreng. Syst. Veg. (ed. 16) [Sprengel] 4 (2): 325, 1827 (Sprengel 1827b).

*** Radula helix (Hook.f. et Taylor) Gottsche, Lindenb. et Nees, Syn. Hepat. 2: 260, 1845 (Gottsche et al. 1845a). Bas.: Jungermannia helix Hook.f. et Taylor, London J. Bot. 3: 475, 1844 (Hooker and Taylor 1844b).

*** Radula hicksiae K.Yamada, Cryptog. Bryol. Lichénol. 5 (1/2): 191, 1984 (Yamada 1984a).

*** Radula iwatsukii K.Yamada, J. Hattori Bot. Lab. 45: 275, 1979 (Yamada 1979b).

** Radula morobeana K.Yamada et Piippo, Ann. Bot. Fenn. 26 (4): 358, 1989 (Yamada and Piippo 1989).

** Radula multiamentula E.A.Hodgs., Rec. Domin. Mus. 4 (11): 122, 1962 (Hodgson 1962a).

*** Radula ornata E.A.Br. et Pócs, Telopea 9 (3): 436, 2001 (Brown and Pócs 2001).

*** Radula physoloba Mont., Ann. Sci. Nat. Bot. (sér. 2) 19: 255, 1843 (Montagne 1843).

*** Radula pseudoscripta M.A.M.Renner, New Zealand J. Bot. 44 (3): 340, 2006 (Renner 2006).

** Radula queenslandica K.Yamada, J. Hattori Bot. Lab. 62: 192, 1987 (Yamada 1987).

*** Radula scariosa Mitt., Bonplandia 9 (24): 367, 1861 (Mitten 1861).

*** Radula splendida M.A.M.Renner et Devos, Nova Hedwigia 90 (1/2): 113, 2010 (Renner et al. 2010a).

* Radula squarrosa K.Yamada, J. Jap. Bot. 65 (1): 1, 1990 (Yamada 1990). ^378^

*** Radula thiersiae K.Yamada, J. Hattori Bot. Lab. 62: 198, 1987 (Yamada 1987).

*** Radula uvifera (Hook.f. et Taylor) Gottsche, Lindenb. et Nees, Syn. Hepat. 2: 258, 1845 (Gottsche et al. 1845a). Bas.: Jungermannia uvifera Hook.f. et Taylor, London J. Bot. 3: 292 [392], 1844 (Hooker and Taylor 1844a).

* Radula vagans Steph., Kungl. Svenska Vetensk.-Akad. Handl. (n.ser.) 46 (9): 85, 1911 (Stephani 1911b).

** Radula verrucosa K.Yamada, J. Hattori Bot. Lab. 45: 277, 1979 (Yamada 1979b).

*** **subg. Cladoradula Spruce**, Trans. & Proc. Bot. Soc. Edinburgh 15: 315, 1885 (Spruce 1885).

** Radula auriculata Steph., Bull. Herb. Boissier 5 (2): 105, 1897 (Stephani 1897b).

* Radula bipinnata Mitt., J. Proc. Linn. Soc., Bot. 7 (27): 166, 1863 (Mitten 1863). ^379^

*** Radula boryana (F.Weber) Nees ex Mont., Ann. Sci. Nat. Bot. (sér. 2) 18: 13, 1842 (Montagne 1842b). Bas.: Jungermannia boryana F.Weber, Hist. Musc. Hepat. Prodr.: 58, 1815 (Weber 1815).

*** Radula campanigera Mont., London J. Bot. 3: 634, 1844 (Montagne 1844a).

*** Radula campanigera subsp. obiensis (S.Hatt.) K.Yamada, J. Hattori Bot. Lab. 45: 309, 1979 (Yamada 1979b). Bas.: Radula obiensis S.Hatt., Bull. Tokyo Sci. Mus. 11: 83, 1944 (Hattori 1944d).

** Radula chinensis Steph., Nuovo Giorn. Bot. Ital. (n.ser.) 13 (4): 355, 1906 (Levier 1906).

*** Radula gottscheana Taylor, London J. Bot. 5: 374, 1846 (Taylor 1846b).

*** Radula perrottetii Gottsche, Hedwigia 23 (10): 154, 1884 (Stephani 1884a).

** Radula tenax Lindb., Acta Soc. Sci. Fenn. 10: 492, 1875 (Lindberg 1875).

*** **subg. Dactyloradula Devos, M.A.M.Renner, Gradst., A.J.Shaw et Vanderp.**, Taxon 60 (6): 1630, 2011 (Devos et al. 2011).

*** Radula brunnea Steph., Sp. Hepat. (Stephani) 4: 232, 1910 (Stephani 1910b).

*** **subg. Metaradula R.M.Schust.**, Phytologia 56 (2): 69, 1984 (Schuster 1984).

** Radula acuminata Steph., Sp. Hepat. (Stephani) 4: 230, 1910 (Stephani 1910b). ^380^

** Radula aguirrei R.M.Schust., Phytotaxa 202 (1): 70, 2015 (Söderström et al. 2015c). Based on: Radula aguirrei R.M.Schust., J. Hattori Bot. Lab. 70: 56, 1991 (Schuster 1991a), *nom. inval*.

** Radula anisotoma M.A.M.Renner, PhytoKeys 27: 30, 2013 (Renner et al. 2013a).

*** Radula assamica Steph., Hedwigia 23 (10): 151, 1884 (Stephani 1884a).

*** Radula australiana K.Yamada, J. Hattori Bot. Lab. 51: 323, 1982 (Yamada 1982a).

*** Radula buccinifera (Hook.f. et Taylor) Gottsche, Lindenb. et Nees, Syn. Hepat. 2: 261, 1845 (Gottsche et al. 1845a). Bas.: Jungermannia buccinifera Hook.f. et Taylor, London J. Bot. 3: 580, 1844 (Hooker and Taylor 1844c).

*** Radula demissa M.A.M.Renner, PhytoKeys 27: 53, 2013 (Renner et al. 2013a).

** Radula evansii Castle, Ann. Bryol. 11: 37, 1938 (Castle 1938).

** Radula flaccida Lindenb. et Gottsche, Syn. Hepat. 5: 726, 1847 (Gottsche et al. 1847).

** Radula flaccida var. brachycalyx Spruce, Trans. & Proc. Bot. Soc. Edinburgh 15: 321, 1885 (Spruce 1885).

*** Radula forficata M.A.M.Renner, Austral. Syst. Bot. 26 (4): 307, 2013 (Renner et al. 2013c).

** Radula grevilleana Taylor, Ann. Mag. Nat. Hist. 20 (135): 380, 1847 (Taylor 1847a).

*** Radula imposita M.A.M.Renner, PhytoKeys 27: 65, 2013 (Renner et al. 2013a).

** Radula jovetiana K.Yamada, Cryptog. Bryol. Lichénol. 5 (1/2): 193, 1984 (Yamada 1984a).

** Radula kilgourii M.A.M.Renner, Austral. Syst. Bot. 26 (4): 313, 2013 (Renner et al. 2013c).

** Radula loriana Castle, J. Hattori Bot. Lab. 21: 6, 1959 (Castle 1959).

*** Radula mammosa Spruce, Mem. Torrey Bot. Club 1 (3): 127, 1890 (Spruce 1890).

*** Radula mittenii Steph., Hedwigia 23 (10): 148, 1884 (Stephani 1884a).

*** Radula myriopoda M.A.M.Renner, Austral. Syst. Bot. 26 (4): 323, 2013 (Renner et al. 2013c).

*** Radula notabilis M.A.M.Renner, PhytoKeys 27: 77, 2013 (Renner et al. 2013a).

** Radula nymannii Steph., Sp. Hepat. (Stephani) 4: 229, 1910 (Stephani 1910b).

** Radula protensa Lindenb., Bot. Zeitung (Berlin) 6 (25): 462, 1848 (Meissner 1848).

** Radula protensa var. erectilobula Schiffn., Nova Acta Acad. Caes. Leop.-Carol. German. Nat. Cur. 60 (2): 247, 1893 (Schiffner 1893a).

** Radula pseudoflaccida E.W.Jones, J. Bryol. 9 (4): 501, 1977 [1978] (Jones 1977).

** Radula psychosis M.A.M.Renner, Austral. Syst. Bot. 26 (4): 328, 2013 (Renner et al. 2013c).

*** Radula ratkowskiana K.Yamada, J. Jap. Bot. 59 (3): 94, 1984 (Yamada 1984b).

*** Radula robinsonii Steph., Sp. Hepat. (Stephani) 4: 214, 1910 (Stephani 1910b).

** Radula stenocalyx Mont., Ann. Sci. Nat. Bot. (sér. 4) 3 (5): 315, 1855 (Montagne 1855).

*** Radula strangulata Hook.f. et Taylor, London J. Bot. 5: 377, 1846 (Taylor 1846b).

* Radula tjibodensis K.I.Goebel, Ann. Jard. Bot. Buitenzorg 7 (1): 53, 1888 (Goebel 1888). ^381^

*** Radula ventricosa Steph., Sp. Hepat. (Stephani) 4: 187, 1910 (Stephani 1910b).

*** Radula yanoella R.M.Schust., Phytologia 56 (2): 72, 1984 (Schuster 1984).

*** **subg. Odontoradula K.Yamada**, J. Hattori Bot. Lab. 45: 209, 1979 (Yamada 1979b).

** Radula acuta Mitt., Fl. vit.: 410, 1871 [1873] (Mitten 1871).

** Radula allisonii Castle, Rev. Bryol. Lichénol. 31 (3/4): 148, 1962 [1963] (Castle 1962).

** Radula amoena Herzog, Mitt. Inst. Allg. Bot. Hamburg 7 (3): 192, 1931 (Herzog 1931a).

** Radula anceps Sande Lac., Ned. Kruidk. Arch. 3: 419, 1854 [1855] (Sande Lacoste 1854).

* Radula crenulata Schiffn., Leberm., Forschungsr. Gazelle 4 (4): 21, 1890 (Schiffner 1890). ^382^

*** Radula cuspidata Steph., Sp. Hepat. (Stephani) 4: 156, 1910 (Stephani 1910b).

*** Radula decora Gottsche, Hedwigia 23 (10): 145, 1884 (Stephani 1884a).

** Radula emarginata K.Yamada et Piippo, Ann. Bot. Fenn. 26 (4): 352, 1989 (Yamada and Piippo 1989).

** Radula kojana Steph., Bull. Herb. Boissier 5 (2): 105, 1897 (Stephani 1897b).

*** Radula lacerata Steph., Rev. Bryol. 35 (2): 33, 1908 (Stephani 1908l).

*** Radula novae-hollandiae Hampe, Nov. Stirp. Pug. 7: 24, 1838 (Lehmann 1838).

*** Radula ocellata K.Yamada, J. Hattori Bot. Lab. 45: 209, 1979 (Yamada 1979b).

*** Radula plicata Mitt., Bot. antarct. voy. II (Fl. Nov.-Zel. 2): 154, 1854 (Mitten 1854).

** Radula pugioniformis M.A.M.Renner, PhytoKeys 27: 84, 2013 (Renner et al. 2013a).

*** Radula pulchella Mitt., Hedwigia 23 (10): 149, 1884 (Stephani 1884a).

*** Radula retroflexa Taylor, London J. Bot. 5: 378, 1846 (Taylor 1846b).

*** Radula tasmanica Steph., Sp. Hepat. (Stephani) 4: 212, 1910 (Stephani 1910b).

*** Radula weymouthiana Steph., Sp. Hepat. (Stephani) 4: 190, 1910 (Stephani 1910b).

*** **subg. Radula**

*** Radula acutiloba Steph., Hedwigia 28 (4): 271, 1889 (Stephani 1889c). ^383^

** Radula appressa Mitt., Philos. Trans. 168: 397, 1879 (Mitten 1879).

*** Radula aquilegia (Hook.f. et Taylor) Gottsche, Lindenb. et Nees, Syn. Hepat. 2: 260, 1845 (Gottsche et al. 1845a). Bas.: Jungermannia aquilegia Hook.f. et Taylor, London J. Bot. 3: 291 [391], 1844 (Hooker and Taylor 1844a).

** Radula australis Austin, Bot. Bull. (Hanover) 1 (7): 32, 1876 (Austin 1876b).

** Radula borneensis Steph., Sp. Hepat. (Stephani) 4: 209, 1910 (Stephani 1910b).

** Radula caduca K.Yamada, J. Hattori Bot. Lab. 45: 225, 1979 (Yamada 1979b).

*** Radula carringtonii J.B.Jack, Flora 64 (25): 385, 1881 (Jack 1881).

*** Radula complanata (L.) Dumort., Syll. Jungerm. Europ.: 38, 1831 (Dumortier 1831). Bas.: Jungermannia complanata L., Sp. Pl. 1: 1133, 1753 (Linnaeus 1753).

** Radula constricta Steph., Sp. Hepat. (Stephani) 6: 506, 1924 (Stephani 1924).

** Radula evelynae K.Yamada, J. Jap. Bot. 50 (4): 115, 1975 (Yamada 1975a).

** Radula fendleri Gottsche, Hedwigia 23 (10): 146, 1884 (Stephani 1884a). ^384^

*** Radula grandis Steph., J. Linn. Soc., Bot. 29 (201): 271, 1892 (Stephani 1892b).

** Radula japonica Gottsche, Hedwigia 23 (10): 152, 1884 (Stephani 1884a).

** Radula javanica Gottsche, Syn. Hepat. 2: 257, 1845 (Gottsche et al. 1845a).

*** Radula jonesii Bouman, Dirkse et K.Yamada, J. Bryol. 15 (1): 161, 1988 (Bouman et al. 1988).

*** Radula lindenbergiana Gottsche ex C.Hartm., Handb. Skand. fl. (ed.9): 98, 1864 (Hartman 1864).

** Radula madagascariensis Gottsche, Abh. Naturwiss. Vereins Bremen 7: 349, 1882 (Gottsche 1882).

** Radula marojezica E.W.Jones, J. Bryol. 17 (2): 307, 1992 (Jones 1992).

* Radula multiflora Gottsche ex Schiffn., Leberm., Forschungsr. Gazelle 4 (4): 20, 1890 (Schiffner 1890). ^385^

** Radula novoguineensis K.Yamada et Piippo, Ann. Bot. Fenn. 26 (4): 360, 1989 (Yamada and Piippo 1989).

** Radula obconica Sull., Manual (Gray): 688, 1848 (Gray 1848).

** Radula obtusiloba Steph., Bull. Herb. Boissier 5 (2): 105, 1897 (Stephani 1897b).

** Radula obtusiloba subsp. polyclada (A.Evans) S.Hatt., J. Hattori Bot. Lab. 29: 275, 1966 (Hattori 1966d). Bas.: Radula polyclada A.Evans, Bull. Torrey Bot. Club 41 (12): 607, 1914 [1915] (Evans 1914a).

* Radula oceania Castle, Rev. Bryol. Lichénol. 33 (3/4): 390, 1965 (Castle 1965). ^386^

** Radula oreopsis M.A.M.Renner, Telopea 17: 123, 2014 (Renner 2014).

** Radula portoricensis Steph., Hedwigia 27 (11/12): 298, 1888 (Stephani 1888c).

*** Radula prolifera Arnell, Ark. Bot. 13 (2): 12, 1913 (Arnell 1913).

*** Radula quadrata Gottsche, Syn. Hepat. 2: 255, 1845 (Gottsche et al. 1845a).

*** Radula reflexa Nees et Mont., Ann. Sci. Nat. Bot. (sér. 2) 19: 255, 1843 (Montagne 1843).

** Radula sharpii K.Yamada, J. Jap. Bot. 60 (9): 260, 1985 (Yamada 1985a).

** Radula sumatrana Steph., Sp. Hepat. (Stephani) 4: 204, 1910 (Stephani 1910b).

** Radula tokiensis Steph., Hedwigia 23 (10): 150, 1884 (Stephani 1884a).

*** Radula van-zantenii K.Yamada, J. Hattori Bot. Lab. 45: 260, 1979 (Yamada 1979b).

** Radula varilobula Castle, J. Hattori Bot. Lab. 21: 19, 1959 (Castle 1959).

*** Radula wichurae Steph., Sp. Hepat. (Stephani) 4: 168, 1910 (Stephani 1910b).

*** **subg. Volutoradula Devos, M.A.M.Renner, Gradst., A.J.Shaw et Vanderp.**, Taxon 60 (6): 1629, 2011 (Devos et al. 2011).

** Radula ankefinensis Gottsche, Hedwigia 23 (10): 152, 1884 (Stephani 1884a).

** Radula antilleana Castle, J. Hattori Bot. Lab. 21: 48, 1959 (Castle 1959).

** Radula comorensis Steph., Hedwigia 23 (9): 132, 1884 (Stephani 1884c).

** Radula cubensis K.Yamada, J. Hattori Bot. Lab. 54: 241, 1983 (Yamada 1983).

** Radula diversifolia Steph., Sp. Hepat. (Stephani) 4: 212, 1910 (Stephani 1910b).

*** Radula eggersii K.Yamada, J. Hattori Bot. Lab. 82: 339, 1997 (Yamada 1997).

** Radula episcia Spruce, Trans. & Proc. Bot. Soc. Edinburgh 15: 318, 1885 (Spruce 1885).

** Radula floridana Castle, Rev. Bryol. Lichénol. 36 (1/2): 1, 1968 [1969] (Castle 1968).

** Radula fulvifolia (Hook.f. et Taylor) Gottsche, Lindenb. et Nees, Syn. Hepat. 2: 261, 1845 (Gottsche et al. 1845a). Bas.: Jungermannia fulvifolia Hook.f. et Taylor, London J. Bot. 4: 85, 1845 (Hooker and Taylor 1845).

*** Radula hastata Steph., Sp. Hepat. (Stephani) 4: 163, 1910 (Stephani 1910b).

* Radula holstiana Steph., Bot. Jahrb. Syst. 20 (3): 320, 1895 (Stephani 1895a).

*** Radula holtii Spruce, J. Bot. 25: 209, 1887 (Spruce 1887b).

** Radula husnotii Castle, J. Hattori Bot. Lab. 21: 45, 1959 (Castle 1959).

** Radula inflexa Gottsche, Hedwigia 23 (10): 148, 1884 (Stephani 1884a).

** Radula kegelii Gottsche ex Steph., Hedwigia 23 (10): 152, 1884 (Stephani 1884a).

** Radula macroloba Steph., Bull. Soc. Roy. Bot. Belgique 31: 121, 1892 (Stephani 1892c).

** Radula mazarunensis K.Yamada, Trop. Bryol. 1: 38, 1989 (Gradstein and Florschütz-de Waard 1989).

** Radula mexicana Lindenb. et Gottsche, Mexik. Leverm.: 150, 1863 (Gottsche 1863).

** Radula microloba Gottsche, Syn. Hepat. 2: 259, 1845 (Gottsche et al. 1845a).

** Radula neotropica Castle, J. Hattori Bot. Lab. 21: 31, 1959 (Castle 1959).

*** Radula nudicaulis Steph., Sp. Hepat. (Stephani) 4: 174, 1910 (Stephani 1910b).

* Radula nudicaulis var. delicatula P.Allorge et V.Allorge, Rev. Bryol. Lichénol. 19 (1/2): 106, 1950 (Allorge and Allorge 1950).

** Radula pocsii K.Yamada, J. Hattori Bot. Lab. 54: 245, 1983 (Yamada 1983).

** Radula recubans Taylor, London J. Bot. 5: 376, 1846 (Taylor 1846b).

** Radula saccatiloba Steph., Hedwigia 23 (8): 129, 1884 (Stephani 1884b).

** Radula schaefer-verwimpii K.Yamada, J. Jap. Bot. 65 (1): 3, 1990 (Yamada 1990).

** Radula schofieldiana K.Yamada, J. Hattori Bot. Lab. 82: 337, 1997 (Yamada 1997).

** Radula stipatiflora Steph., Sp. Hepat. (Stephani) 4: 159, 1910 (Stephani 1910b).

** Radula striata Mitt., Hedwigia 23 (10): 155, 1884 (Stephani 1884a).

** Radula subinflata Lindenb. et Gottsche, Syn. Hepat. 5: 724, 1847 (Gottsche et al. 1847).

** Radula sullivantii Austin, Hepat. bor.-amer.: 22, 1873 (Austin 1873).

** Radula tenera Mitt., Hedwigia 23 (10): 149, 1884 (Stephani 1884a).

*** Radula voluta Taylor, Syn. Hepat. 2: 255, 1845 (Gottsche et al. 1845a).

***Incertae sedis***

*** Radula acutangula Steph., Bull. Herb. Boissier 5 (10): 848, 1897 (Stephani 1897c).

** Radula angulata Steph., Hedwigia 23 (8): 114, 1884 (Stephani 1884b).

** Radula bogotensis Steph., Hedwigia 23 (8): 115, 1884 (Stephani 1884b).

*** Radula bolanderi Gottsche, Hedwigia 23 (10): 145, 1884 (Stephani 1884a).

** Radula boninensis Furuki et K.Yamada, J. Jap. Bot. 61 (10): 312, 1986 (Furuki and Yamada 1986).

** Radula brasilica K.Yamada, J. Hattori Bot. Lab. 74: 35, 1993 (Yamada 1993).

** Radula caespitosa Steph., Hedwigia 27 (3/4): 107, 1888 (Stephani 1888d).

** Radula campanulata Lindenb. et Gottsche, Syn. Hepat. 2: 256, 1845 (Gottsche et al. 1845a).

** Radula castlei Grolle, Bryologist 73 (4): 662, 1970 (Grolle 1970a).

*** Radula cavifolia Hampe ex Gottsche, Lindenb. et Nees, Syn. Hepat. 2: 259, 1845 (Gottsche et al. 1845a).

** Radula cochabambaensis K.Yamada, J. Hattori Bot. Lab. 74: 37, 1993 (Yamada 1993).

** Radula conferta Lindenb. et Gottsche, Syn. Hepat. 5: 729, 1847 (Gottsche et al. 1847).

*** Radula cordata Mitt., Fl. vit.: 410, 1871 [1873] (Mitten 1871).

** Radula costaricensis Gottsche, J. Bot. 15: 226, 1877 (Polakowski 1877).

*** Radula curvilobula M.L.So, J. Hattori Bot. Lab. 98: 176, 2005 (So 2005a).

* Radula decurrens Mitt., Fl. vit.: 410, 1871 [1873] (Mitten 1871). ^387^

** Radula densifolia Castle, Rev. Bryol. Lichénol. 33 (3/4): 385, 1965 (Castle 1965).

** Radula diaphana K.I.Goebel, Organogr. Pfl., ed. 2, 2 (1): 677, 1915 (Goebel 1915).

** Radula dolabrata K.Yamada, J. Jap. Bot. 60 (9): 257, 1985 (Yamada 1985a).

** Radula elliottii Castle, J. Hattori Bot. Lab. 21: 12, 1959 (Castle 1959).

** Radula falcata Steph., Hedwigia 23 (8): 115, 1884 (Stephani 1884b).

** Radula fauriana Steph., Sp. Hepat. (Stephani) 4: 207, 1910 (Stephani 1910b).

** Radula fernandezana Steph., Kungl. Svenska Vetensk.-Akad. Handl. (n.ser.) 46 (9): 84, 1911 (Stephani 1911b).

** Radula flavifolia (Hook.f. et Taylor) Gottsche, Lindenb. et Nees, Syn. Hepat. 2: 259, 1845 (Gottsche et al. 1845a). Bas.: Jungermannia flavifolia Hook.f. et Taylor, London J. Bot. 3: 476, 1844 (Hooker and Taylor 1844b).

** Radula fujitae Furuki, Bryol. Res. 9 (5): 143, 2007 (Furuki 2007).

** Radula galapagona Steph., Sp. Hepat. (Stephani) 4: 176, 1910 (Stephani 1910b).

** Radula gedena Gottsche, Hedwigia 23 (10): 146, 1884 (Stephani 1884a).

** Radula gracilis Mitt., Hedwigia 23 (10): 147, 1884 (Stephani 1884a).

** Radula gradsteinii K.Yamada, Trop. Bryol. 1: 37, 1989 (Gradstein and Florschütz-de Waard 1989).

** Radula grandifolia Steph., Sp. Hepat. (Stephani) 4: 184, 1910 (Stephani 1910b).

** Radula grollei K.Yamada et Piippo, Ann. Bot. Fenn. 26 (4): 379, 1989 (Yamada and Piippo 1989).

** Radula guyanensis K.Yamada, Trop. Bryol. 1: 38, 1989 (Gradstein and Florschütz-de Waard 1989).

** Radula hattorii K.Yamada, J. Jap. Bot. 60 (9): 259, 1985 (Yamada 1985a).

*** Radula hawaiica M.L.So, J. Hattori Bot. Lab. 98: 177, 2005 (So 2005a).

* Radula hedingeri K.I.Goebel, Ann. Jard. Bot. Buitenzorg 7 (1): 51, 1888 (Goebel 1888).

*** Radula inouei K.Yamada, J. Hattori Bot. Lab. 45: 262, 1979 (Yamada 1979b).

*** Radula involvens Spruce, Trans. & Proc. Bot. Soc. Edinburgh 15: 325, 1885 (Spruce 1885).

*** Radula iwatsukiana K.Yamada, J. Hattori Bot. Lab. 58: 114, 1985 (Yamada 1985b).

*** Radula jamaicensis Pearson, Ann. Bryol. 4: 103, 1931 (Pearson 1931b).

** Radula jamesonii Taylor, London J. Bot. 5: 375, 1846 (Taylor 1846b).

** Radula kinabaluensis K.Yamada, Misc. Bryol. Lichenol. 6 (6): 97, 1973 (Yamada 1973b).

*** Radula kitagawae K.Yamada, J. Hattori Bot. Lab. 58: 116, 1985 (Yamada 1985b).

** Radula koponenii K.Yamada et Piippo, Ann. Bot. Fenn. 26 (4): 364, 1989 (Yamada and Piippo 1989).

** Radula kurzii Steph., Hedwigia 23 (10): 153, 1884 (Stephani 1884a).

** Radula laxiramea Steph., Sp. Hepat. (Stephani) 4: 178, 1910 (Stephani 1910b).

** Radula leiboldii Steph., Hedwigia 23 (8): 116, 1884 (Stephani 1884b).

** Radula lewisii K.Yamada, J. Hattori Bot. Lab. 74: 39, 1993 (Yamada 1993).

*** Radula ligula Steph., Sp. Hepat. (Stephani) 4: 228, 1910 (Stephani 1910b).

*** Radula lingulata Gottsche, Syn. Hepat. 2: 260, 1845 (Gottsche et al. 1845a).

** Radula longiloba K.Yamada, J. Hattori Bot. Lab. 54: 243, 1983 (Yamada 1983).

* Radula longispica Steph., Sp. Hepat. (Stephani) 4: 183, 1910 (Stephani 1910b). ^388^

*** Radula marginata Gottsche, Lindenb. et Nees, Syn. Hepat. 2: 261, 1845 (Gottsche et al. 1845a). *Nom. nov. pro Jungermannia marginata* Hook.f. et Taylor, London J. Bot. 3: 566, 1844 (Hooker and Taylor 1844a), *nom. illeg*.

*** Radula mauiensis M.L.So, J. Hattori Bot. Lab. 98: 178, 2005 (So 2005a).

** Radula microlobula Castle, J. Hattori Bot. Lab. 21: 35, 1959 (Castle 1959).

** Radula minutilobula K.Yamada et Piippo, Ann. Bot. Fenn. 26 (4): 377, 1989 (Yamada and Piippo 1989).

** Radula mizutanii K.Yamada, J. Jap. Bot. 48 (5): 134, 1973 (Yamada 1973a).

*** Radula nigra Pearson, J. Linn. Soc., Bot. 46 (305): 31, 1922 (Pearson 1922b).

** Radula nilgiriensis Udar et D.Kumar, J. Indian Bot. Soc. 61: 177, 1982 (Udar and Kumar 1982b).

** Radula norrisii K.Yamada et Piippo, Ann. Bot. Fenn. 26 (4): 374, 1989 (Yamada and Piippo 1989).

** Radula novivrieseana K.Yamada, J. Hattori Bot. Lab. 51: 326, 1982 (Yamada 1982a).

*** Radula novocaledonica Hürl. et K.Yamada, J. Jap. Bot. 54 (8): 238, 1979 (Hürlimann and Yamada 1979).

*** Radula novocaledoniensis K.Yamada, J. Hattori Bot. Lab. 58: 120, 1985 (Yamada 1985b).

** Radula obovata Castle, J. Hattori Bot. Lab. 21: 16, 1959 (Castle 1959).

** Radula obscura Mitt., J. Proc. Linn. Soc., Bot. 5 (18): 107, 1860 [1861] (Mitten 1860c).

** Radula okamurana Steph., Sp. Hepat. (Stephani) 4: 209, 1910 (Stephani 1910b).

** Radula onraedtii K.Yamada, Misc. Bryol. Lichenol. 8 (6): 113, 1979 (Yamada 1979a).

** Radula opaciuscula (Spruce) Castle, J. Hattori Bot. Lab. 21: 22, 1959 (Castle 1959). Bas.: Radula episcia var. opaciuscula Spruce, Trans. & Proc. Bot. Soc. Edinburgh 15: 319, 1885 (Spruce 1885).

** Radula ovalilobula K.Yamada, J. Hattori Bot. Lab. 45: 257, 1979 (Yamada 1979b).

** Radula oyamensis Steph., Hedwigia 23 (10): 149, 1884 (Stephani 1884a).

* Radula paganii Castle, J. Hattori Bot. Lab. 21: 33, 1959 (Castle 1959). ^389^

*** Radula pallens (Sw.) Nees ex Mont., Voy. Amér. Mérid. 7 (2): 71, 1839 (Montagne 1839a). Bas.: Jungermannia pallens Sw., Prodr. (Swartz): 143, 1788 (Swartz 1788).

*** Radula pandei Udar et Dh.Kumar, Lindbergia 9 (2): 133, 1983 (Udar and Kumar 1983b).

** Radula patens K.Yamada, Cryptog. Bryol. Lichénol. 5 (1/2): 197, 1984 (Yamada 1984a).

** Radula peruviana K.Yamada, Beih. Nova Hedwigia 88: 79, 1987 (Schultze-Motel and Menzel 1987).

** Radula philippinensis K.Yamada, J. Hattori Bot. Lab. 45: 299, 1979 (Yamada 1979b).

* Radula pinnulata Mitt., Fl. vit.: 410, 1871 [1873] (Mitten 1871). ^390^

** Radula pseudostachya Spruce, Trans. & Proc. Bot. Soc. Edinburgh 15: 319, 1885 (Spruce 1885).

** Radula punctata Steph., Hedwigia 23 (8): 135, 1884 (Stephani 1884b).

** Radula pusilla Spruce, Trans. & Proc. Bot. Soc. Edinburgh 15: 320, 1885 (Spruce 1885).

*** Radula rhombiloba Steph., Sp. Hepat. (Stephani) 4: 204, 1910 (Stephani 1910b).

* Radula rupicola K.Yamada, J. Hattori Bot. Lab. 58: 124, 1985 (Yamada 1985b). ^391^

** Radula santacruziana K.Yamada et Gradst., Trop. Bryol. 4: 67, 1991 (Yamada and Gradstein 1991).

** Radula silvestris Gottsche, Abh. Naturwiss. Vereins Bremen 7: 349, 1882 (Gottsche 1882).

** Radula sinskeana K.Yamada, J. Hattori Bot. Lab. 74: 41, 1993 (Yamada 1993).

** Radula sinuata Gottsche ex Steph., Sp. Hepat. (Stephani) 4: 161, 1910 (Stephani 1910b).

* Radula socorana Gerola, Lav. Bot. Ist. Bot. Univ. Padova 12: 475, 1947 (Gerola 1947).

** Radula sonsonensis Steph., Sp. Hepat. (Stephani) 4: 201, 1910 (Stephani 1910b).

** Radula stellatogemmipara C.Gao et Y.H.Wu, Nova Hedwigia 80 (1/2): 239, 2005 (Gao and Wu 2005).

** Radula subsimplex Steph., Hedwigia 23 (8): 130, 1884 (Stephani 1884b).

** Radula subsquarrosa S.W.Arnell, Ark. Bot. (n.ser.) 4 (1): 15, 1957 (Arnell 1957b).

** Radula tabularis Steph., Hedwigia 23 (9): 131, 1884 (Stephani 1884c).

** Radula taylorii Steph., Hedwigia 23 (9): 133, 1884 (Stephani 1884c).

** Radula tectiloba Steph., Hedwigia 27 (11/12): 298, 1888 (Stephani 1888c).

** Radula tenuis K.Yamada, J. Hattori Bot. Lab. 54: 247, 1983 (Yamada 1983).

** Radula underwoodii Castle, J. Hattori Bot. Lab. 21: 37, 1959 (Castle 1959).

** Radula venezuelensis K.Yamada, Misc. Bryol. Lichenol. 9 (6): 122, 1982 (Yamada 1982b).

*** Radula vieillardii Gottsche, Hedwigia 23 (10): 150, 1884 (Stephani 1884a).

** Radula visianica C.Massal., Ann. Bot. (Rome) 1 (4): 298, 1904 (Massalongo 1904).

** Radula vrieseana Sande Lac., Ann. Mus. Bot. Lugduno-Batavi 1: 305, 1864 (Sande Lacoste 1864).

** Radula wrightii Castle, J. Hattori Bot. Lab. 21: 15, 1959 (Castle 1959).

** Radula xalapensis Nees et Mont., Ann. Sci. Nat. Bot. (sér. 2) 5: 56, 1836 (Nees and Montagne 1836).

###### Ptilidiales Schljakov

####### ** Herzogianthaceae Stotler et Crand.-Stotl.

** **Herzogianthus R.M.Schust.**, J. Hattori Bot. Lab. 23: 71, 1960 [1961] (Schuster 1960b).

* Herzogianthus sanguineus R.M.Schust., Phytologia 56 (7): 457, 1985 (Schuster 1985c). ^392^

*** Herzogianthus vaginatus (Herzog) R.M.Schust., J. Hattori Bot. Lab. 23: 71, 1960 [1961] (Schuster 1960b). Bas.: Blepharostoma vaginatum Herzog, Trans. & Proc. Roy. Soc. New Zealand 65 (3): 355, 1936 (Herzog 1936b).

####### ** Neotrichocoleaceae Inoue

** **Neotrichocolea S.Hatt.**, J. Hattori Bot. Lab. 2: 9, 1947 [1948] (Hattori 1947b).

** Neotrichocolea bissetii (Mitt.) S.Hatt., J. Hattori Bot. Lab. 2: 10, 1947 [1948] (Hattori 1947b). Bas.: Mastigophora bissetii Mitt., Trans. Linn. Soc. London, Bot. 3 (3): 200, 1891 (Mitten 1891).

** **Trichocoleopsis S.Okamura**, Bot. Mag. (Tokyo) 25 (293): 159, 1911 (Okamura 1911).

** Trichocoleopsis sacculata (Mitt.) S.Okamura, Bot. Mag. (Tokyo) 25 (293): 159, 1911 (Okamura 1911). Bas.: Blepharozia sacculata Mitt., Trans. Linn. Soc. London, Bot. 3 (3): 200, 1891 (Mitten 1891).

####### *** Ptilidiaceae H.Klinggr.

by L. Söderström

Ptilidiaceae was recently studied molecularly by Kreier et al. (2010) showing the occurrence of a possibly undescribed cryptic species from the Himalayas.

*** **Ptilidium Nees**, Naturgesch. Eur. Leberm. 1: 95, 1833 (Nees 1833c).

*** Ptilidium californicum (Austin) Pearson, List. Canad. Hepat.: 7, 1890 (Pearson 1890). Bas.: Lepidozia californica Austin, Bull. Torrey Bot. Club 6 (3): 19, 1875 (Austin 1875b).

*** Ptilidium ciliare (L.) Hampe, Prod. fl. hercyn.: 76, 1836 (Hampe 1836). Bas.: Jungermannia ciliaris L., Sp. Pl. 1: 1134, 1753 (Linnaeus 1753).

*** Ptilidium pulcherrimum (Weber) Vain., Meddel. Soc. Fauna Fl. Fenn. 3: 88, 1878 (Vainio 1878). Bas.: Jungermannia pulcherrima Weber, Spic. Fl. Goett.: 150, 1778 (Weber 1778).

##### Metzgeriidae Barthol.-Began

###### Metzgeriales Chalaud

####### *** Aneuraceae H.Klinggr.

by M. Nebel

A molecular phylogeny of Aneuraceae was recently published by Preussing et al. (2010). Their study revealed Verdoorniaceae to be nested within Aneuraceae and Cryptothallus within Aneura (see also Wickett and Goffinet 2008). Nomenclatural and taxonomic notes can also be found in Söderström et al. (2010a), Söderström et al. (2012a) and Nebel et al. (2013).

*** **Aneura Dumort.**, Commentat. Bot. (Dumortier): 115, 1822 (Dumortier 1822). ^393^

* Aneura amboinensis Steph., Bull. Herb. Boissier 7 (9): 678 (219), 1899 (Stephani 1899e).

* Aneura augustae Steph., Sp. Hepat. (Stephani) 6: 430, 1923 (Stephani 1923).

* Aneura biflora Colenso, Trans. & Proc. New Zealand Inst. 17: 262, 1885 (Colenso 1885).

*** Aneura blasioides (Horik.) Furuki, J. Hattori Bot. Lab. 70: 311, 1991 (Furuki 1991). Bas.: Riccardia blasioides Horik., J. Sci. Hiroshima Univ., Ser. B, Div. 2, Bot. 1: 197, 1933 (Horikawa 1933).

** Aneura brasiliensis (Ångstr.) Steph., Hedwigia 32 (3): 137, 1893 (Stephani 1893b). Bas.: Pseudoneura brasiliensis Ångstr., Öfvers. Kongl. Vetensk.-Akad. Förh. 33 (7): 91, 1876 [1877] (Ångström 1876).

* Aneura brevissima Steph., Sp. Hepat. (Stephani) 6: 21, 1917 (Stephani 1917a).

** Aneura cerebrata Hewson, Proc. Linn. Soc. New South Wales (ser. 2) 94 (2): 185, 1970 (Hewson 1970b).

*** Aneura crateriformis Furuki et D.G.Long, J. Bryol. 18 (2): 281, 1994 (Furuki and Long 1994).

* Aneura crinita C.Massal., Bull. Soc. Bot. Ital. 1917 (8/9): 81, 1917 (Massalongo 1917). ^394^

** Aneura crumii L.Söderstr., A.Hagborg et von Konrat, Phytotaxa 65: 43, 2012 (Söderström et al. 2012a). *Nom. nov. pro Cryptothallus hirsutus* H.A.Crum, Bryologist 99 (4): 438, 1996 (Crum and Bruce 1996).

* Aneura densa Steph., Sp. Hepat. (Stephani) 6: 24, 1917 (Stephani 1917a).

* Aneura denticulata Mitt. ex Thurn, Timehri 5: 222, 1886 (Thurn 1886). ^395^

** Aneura eachamensis Hewson, Proc. Linn. Soc. New South Wales (ser. 2) 94 (2): 184, 1970 (Hewson 1970b).

** Aneura erronea Steph., Sp. Hepat. (Stephani) 6: 20, 1917 (Stephani 1917a).

** Aneura eskuchei Hässel, Veröff. Geobot. Inst. ETH Stiftung Rübel Zürich 91: 294, 1986 (Hässel 1986b).

** Aneura gemmifera Furuki, J. Hattori Bot. Lab. 70: 309, 1991 (Furuki 1991).

* Aneura giangena Hewson, Proc. Linn. Soc. New South Wales (ser. 2) 94 (2): 190, 1970 (Hewson 1970b).

** Aneura gibbsiana Steph., Sp. Hepat. (Stephani) 6: 28, 1917 (Stephani 1917a).

** Aneura glaucescens Steph., Sp. Hepat. (Stephani) 6: 28, 1917 (Stephani 1917a).

* Aneura goebeliana Steph., Sp. Hepat. (Stephani) 6: 28, 1917 (Stephani 1917a).

*** Aneura hirsuta Furuki, J. Hattori Bot. Lab. 70: 317, 1991 (Furuki 1991).

* Aneura hunsteinii Steph., Sp. Hepat. (Stephani) 6: 431, 1923 (Stephani 1923).

** Aneura imbricata Colenso, Trans. & Proc. New Zealand Inst. 16: 359, 1884 (Colenso 1884).

** Aneura kaguaensis Hewson, Proc. Linn. Soc. New South Wales (ser. 2) 94 (2): 190, 1970 (Hewson 1970b).

** Aneura keniae Gola, Mem. Reale Accad. Sci. Torino (ser. 2) 65 (1): 3, 1916 (Gola 1916).

* Aneura latemultifida Steph., Sp. Hepat. (Stephani) 6: 431, 1923 (Stephani 1923).

** Aneura latissima Spruce, Trans. & Proc. Bot. Soc. Edinburgh 15: 544, 1885 (Spruce 1885).

* Aneura ledermannii Steph., Sp. Hepat. (Stephani) 6: 431, 1923 (Stephani 1923).

** Aneura macrostachya Spruce, Trans. & Proc. Bot. Soc. Edinburgh 15: 550, 1885 (Spruce 1885).

*** Aneura marianensis Furuki, Bryologist 97 (1): 87, 1994 (Furuki 1994b).

*** Aneura maxima (Schiffn.) Steph., Bull. Herb. Boissier 7 (10): 760 (270), 1899 (Stephani 1899f). Bas.: Riccardia maxima Schiffn., Denkschr. Kaiserl. Akad. Wiss., Math.-Naturwiss. Kl. 67: 177, 1898 (Schiffner 1898a).

*** Aneura mirabilis (Malmb.) Wickett et Goffinet, Bot. J. Linn. Soc. 156 (1): 11, 2008 (Wickett and Goffinet 2008). Bas.: Cryptothallus mirabilis Malmb., Ann. Bryol. 6: 122, 1933 (von Malmborg 1933).

** Aneura novaecaledoniae R.M.Schust., Phytologia 56 (7): 451, 1985 (Schuster 1985c).

*** Aneura novaguineensis Hewson, Proc. Linn. Soc. New South Wales (ser. 2) 94 (2): 189, 1970 (Hewson 1970b).

* Aneura nymannii Steph., Sp. Hepat. (Stephani) 6: 35, 1917 (Stephani 1917a).

** Aneura pellucida Colenso, Trans. & Proc. New Zealand Inst. 18: 252, 1886 (Colenso 1886b).

*** Aneura pinguis (L.) Dumort., Syll. Jungerm. Europ.: 86, 1831 (Dumortier 1831). Bas.: Jungermannia pinguis L., Sp. Pl. 1: 1136, 1753 (Linnaeus 1753).

** Aneura polyantha Colenso, Trans. & Proc. New Zealand Inst. 17: 262, 1885 (Colenso 1885).

** Aneura punctata Colenso, Trans. & Proc. New Zealand Inst. 18: 254, 1886 (Colenso 1886b).

** Aneura rodwayi Hewson, Proc. Linn. Soc. New South Wales (ser. 2) 94 (2): 188, 1970 (Hewson 1970b).

* Aneura roraimensis Steph., Trans. Linn. Soc. London, Bot. 6 (1): 94, 1901 (Stephani 1901e).

** Aneura rotangicola Steph., Sp. Hepat. (Stephani) 6: 432, 1923 (Stephani 1923).

* Aneura serrulata Gottsche ex Steph., Sp. Hepat. (Stephani) 6: 42, 1917 (Stephani 1917a).

** Aneura sharpii Inoue et N.G.Mill., Bull. Natl. Sci. Mus. Tokyo, B 11 (3): 96, 1985 (Inoue and Miller 1985). ^396^

* Aneura singalangana (Schiffn.) Steph., Bull. Herb. Boissier 7 (10): 751 (261), 1899 (Stephani 1899f). Bas.: Riccardia singalangana Schiffn., Denkschr. Kaiserl. Akad. Wiss., Math.-Naturwiss. Kl. 67: 174, 1898 (Schiffner 1898a). ^397^

** Aneura subcanaliculata R.M.Schust., J. Hattori Bot. Lab. 67: 60, 1989 (Schuster 1989).

* Aneura subledermannii Steph., Sp. Hepat. (Stephani) 6: 432, 1923 (Stephani 1923).

* Aneura subtenerrima Steph., Sp. Hepat. (Stephani) 6: 432, 1923 (Stephani 1923).

* Aneura vincentina Steph., Sp. Hepat. (Stephani) 6: 45, 1917 (Stephani 1917a).

** **Lobatiriccardia (Mizut. et S.Hatt.) Furuki**, J. Hattori Bot. Lab. 70: 319, 1991 (Furuki 1991). Bas.: Riccardia subg. Lobatiriccardia Mizut. et S.Hatt., J. Hattori Bot. Lab. 18: 38, 1957 (Mizutani and Hattori 1957).

*** Lobatiriccardia alterniloba (Hook.f. et Taylor) Furuki, J. Hattori Bot. Lab. 70: 319, 1991 (Furuki 1991). Bas.: Jungermannia alterniloba Hook.f. et Taylor, London J. Bot. 3: 572, 1844 (Hooker and Taylor 1844d).

** Lobatiriccardia alterniloba var. gigantea (Steph.) Nebel, Phytotaxa 81 (1): 10, 2013 (Nebel et al. 2013). Bas.: Aneura gigantea Steph., J. & Proc. Roy. Soc. New South Wales 48 (1/2): 95, 1914 (Stephani and Watts 1914).

** Lobatiriccardia alterniloba var. robusta (Rodway) Nebel, Phytotaxa 81 (1): 10, 2013 (Nebel et al. 2013). Bas.: Aneura alterniloba f. robusta Rodway, Tasm. Bryoph.: 12, 1917 (Rodway 1917b).

*** Lobatiriccardia athertonensis (Hewson) Furuki, J. Hattori Bot. Lab. 70: 319, 1991 (Furuki 1991). Bas.: Aneura athertonensis Hewson, Proc. Linn. Soc. New South Wales (ser. 2) 94 (2): 188, 1970 (Hewson 1970b).

*** Lobatiriccardia coronopus (De Not.) Furuki, J. Hattori Bot. Lab. 100: 90, 2006 (Furuki 2006a). Bas.: Aneura coronopus De Not., Hedwigia 32 (1): 19, 1893 (Stephani 1893a).

** Lobatiriccardia coronopus subsp. australis (R.M.Schust.) Nebel, Preussing, Schäf.-Verw. et D.Quandt, Taxon 59 (5): 1434, 2010 (Preussing et al. 2010). Bas.: Aneura lobata subsp. australis R.M.Schust., Phytologia 56 (7): 451, 1985 (Schuster 1985c).

** Lobatiriccardia oberwinkleri Nebel, Preussing, Schäf.-Verw. et D.Quandt, Taxon 59 (5): 1435, 2010 (Preussing et al. 2010).

** Lobatiriccardia subaquatica (R.M.Schust.) Nebel, Phytotaxa 81 (1): 10, 2013 (Nebel et al. 2013). Bas.: Aneura subaquatica R.M.Schust., Phytologia 56 (7): 450, 1985 (Schuster 1985c).

** Lobatiriccardia verdoornioides Nebel, Preussing, Schäf.-Verw. et D.Quandt, Taxon 59 (5): 1437, 2010 (Preussing et al. 2010).

*** Lobatiriccardia yakusimensis (S.Hatt.) Furuki, J. Hattori Bot. Lab. 70: 321, 1991 (Furuki 1991). Bas.: Riccardia lobata var. yakusimensis S.Hatt., J. Hattori Bot. Lab. 6: 10, 1951 [1952] (Hattori 1951a).

*** Lobatiriccardia yunnanensis Furuki et D.G.Long, J. Bryol. 29 (3): 161, 2007 (Furuki and Long 2007).

*** **Riccardia Gray**, Nat. Arr. Brit. Pl. 1: 679, 1821 (Gray 1821) nom. conserv. ^398^

** Riccardia pectinata var. fasciculata (Steph.) Hürl., Bauhinia 5 (4): 208, 1976 (Hürlimann 1976). Bas.: Aneura fasciculata Steph., Sp. Hepat. (Stephani) 6: 25, 1917 (Stephani 1917a).

** **subg. Arceoneura Hässel**, Revista Mus. Argent. Ci. Nat., Bernardino Rivadavia Inst. Nac. Invest. Ci. Nat. Bot. 4 (1): 204, 1972 (Hässel 1972a).

** Riccardia marionensis R.M.Schust., J. Hattori Bot. Lab. 67: 65, 1989 (Schuster 1989).

*** Riccardia prehensilis (Hook.f. et Taylor) C.Massal., Nuovo Giorn. Bot. Ital. 17 (3): 255, 1885 (Massalongo 1885). Bas.: Jungermannia prehensilis Hook.f. et Taylor, London J. Bot. 3: 480, 1844 (Hooker and Taylor 1844b).

** **subg. Corioneura Furuki**, J. Hattori Bot. Lab. 70: 394, 1991 (Furuki 1991).

** Riccardia argentolimbata Hewson et Grolle, J. Hattori Bot. Lab. 29: 70, 1966 (Grolle 1966i).

*** Riccardia hattorii Furuki, J. Hattori Bot. Lab. 75: 257, 1994 (Furuki 1994a).

** **subg. Hyaloneura R.M.Schust.**, Phytologia 56 (7): 452, 1985 (Schuster 1985c).

* Riccardia albomarginata (Steph.) Schiffn., Consp. Hepat. Arch. Ind.: 53, 1898 (Schiffner 1898b). Bas.: Aneura albomarginata Steph., Hedwigia 32 (1): 18, 1893 (Stephani 1893a). ^399^

** Riccardia canaliculata (Nees) Kuntze, Revis. Gen. Pl. 2: 838, 1891 (Kuntze 1891). Bas.: Jungermannia canaliculata Nees, Enum. Pl. Crypt. Javae: 10, 1830 (Nees 1830).

** Riccardia pindensis Hewson, Proc. Linn. Soc. New South Wales (ser. 2) 95 (1): 87, 1970 (Hewson 1970a).

** **subg. Lophoneura Hässel**, Revista Mus. Argent. Ci. Nat., Bernardino Rivadavia Inst. Nac. Invest. Ci. Nat. Bot. 4 (1): 218, 1972 (Hässel 1972a).

** Riccardia fuegiensis C.Massal., Nuovo Giorn. Bot. Ital. 17 (3): 255, 1885 (Massalongo 1885).

** **subg. Neoneura Furuki**, J. Hattori Bot. Lab. 70: 385, 1991 (Furuki 1991).

*** Riccardia spongiosa Furuki, J. Hattori Bot. Lab. 70: 385, 1991 (Furuki 1991).

** **subg. Phycaneura R.M.Schust.**, J. Hattori Bot. Lab. 26: 294, 1963 (Schuster 1963b).

** Riccardia aequicellularis (Steph.) Hewson, Proc. Linn. Soc. New South Wales (ser. 2) 95 (1): 79, 1970 (Hewson 1970a). Bas.: Aneura aequicellularis Steph., J. & Proc. Roy. Soc. New South Wales 48 (1/2): 95, 1914 (Stephani and Watts 1914).

** Riccardia asperulata R.M.Schust., J. Hattori Bot. Lab. 27: 209, 1964 (Schuster 1964a).

** **subg. Riccardia**

*** Riccardia aeruginosa Furuki, J. Hattori Bot. Lab. 70: 345, 1991 (Furuki 1991).

*** Riccardia arcuata Furuki, J. Hattori Bot. Lab. 70: 361, 1991 (Furuki 1991).

*** Riccardia chamedryfolia (With.) Grolle, Trans. Brit. Bryol. Soc. 5 (4): 772, 1969 (Grolle 1969b). Bas.: Jungermannia chamedryfolia With., Bot. arr. veg. Gr. Brit. 2: 699, 1776 (Withering 1776).

*** Riccardia cochleata (Hook.f. et Taylor) Kuntze, Revis. Gen. Pl. 2: 838, 1891 (Kuntze 1891). Bas.: Riccia cochleata Hook.f. et Taylor, London J. Bot. 4: 96, 1845 (Hooker and Taylor 1845).

*** Riccardia eriocaula (Hook.) C.Massal., Nuovo Giorn. Bot. Ital. 17 (3): 256, 1885 (Massalongo 1885). Bas.: Jungermannia eriocaula Hook., Musci Exot. 1: tab. 72, 1818 (Hooker 1818).

*** Riccardia flavovirens Furuki, J. Hattori Bot. Lab. 70: 333, 1991 (Furuki 1991).

*** Riccardia fruticosa (Steph.) Furuki, Nat. Hist. Res. 5 (1): 1, 1998 (Furuki 1998). Bas.: Aneura fruticosa Steph., Sp. Hepat. (Stephani) 6: 27, 1917 (Stephani 1917a).

*** Riccardia glauca Furuki, J. Hattori Bot. Lab. 70: 352, 1991 (Furuki 1991).

** Riccardia kodamae Mizut. et S.Hatt., J. Hattori Bot. Lab. 18: 57, 1957 (Mizutani and Hattori 1957).

*** Riccardia latifrons (Lindb.) Lindb., Acta Soc. Sci. Fenn. 10: 513, 1875 (Lindberg 1875). Bas.: Aneura latifrons Lindb., Bot. Not. 26: 62, 1873 (Anonymous 1873).

** Riccardia latifrons subsp. arctica R.M.Schust. et Damsh., J. Hattori Bot. Lab. 62: 303, 1987 (Schuster 1987d).

** Riccardia latifrons var. miyakeana (Schiffn.) Furuki, J. Hattori Bot. Lab. 70: 375, 1991 (Furuki 1991). Bas.: Riccardia miyakeana Schiffn., Österr. Bot. Z. 49 (11): 388, 1899 (Schiffner 1899c).

** Riccardia nagasakiensis (Steph.) S.Hatt., Bull. Tokyo Sci. Mus. 11: 164, 1944 (Hattori 1944d). Bas.: Aneura nagasakiensis Steph., Sp. Hepat. (Stephani) 6: 34, 1917 (Stephani 1917a).

*** Riccardia palmata (Hedw.) Carruth., J. Bot. 3: 302, 1865 (Carruthers 1865). Bas.: Jungermannia palmata Hedw., Theoria generat.: 87, 1784 (Hedwig 1784), *nom. conserv*.

** Riccardia planiflora (Steph.) S.Hatt., Bull. Tokyo Sci. Mus. 11: 164, 1944 (Hattori 1944d). Bas.: Aneura planiflora Steph., Sp. Hepat. (Stephani) 6: 38, 1917 (Stephani 1917a).

** Riccardia planiflora var. aequatorialis Furuki, Nat. Hist. Res. 4 (2): 77, 1997 (Furuki 1997).

** Riccardia pseudodendroceros R.M.Schust., Phytologia 56 (7): 452, 1985 (Schuster 1985c).

*** Riccardia pumila Furuki, J. Hattori Bot. Lab. 70: 361, 1991 (Furuki 1991).

** Riccardia pusilla Grolle, J. Jap. Bot. 41 (8): 231, 1966 (Grolle 1966d). *Nom. nov. pro Riccardia nana* Mizut. et S.Hatt., J. Hattori Bot. Lab. 18: 53, 1957 (Mizutani and Hattori 1957), *nom. illeg*.

*** Riccardia subalpina Furuki, J. Hattori Bot. Lab. 70: 367, 1991 (Furuki 1991).

** Riccardia tamariscina (Steph.) Schiffn., Consp. Hepat. Arch. Ind.: 58, 1898 (Schiffner 1898b). Bas.: Aneura tamariscina Steph., Hedwigia 32 (1): 27, 1893 (Stephani 1893a).

*** Riccardia vitrea Furuki, J. Hattori Bot. Lab. 70: 327, 1991 (Furuki 1991).

** **sect. Alcicornia Hässel**, Revista Mus. Argent. Ci. Nat., Bernardino Rivadavia Inst. Nac. Invest. Ci. Nat. Bot. 4 (1): 17, 1972 (Hässel 1972a).

** Riccardia alcicornis (Hook.f. et Taylor) Trevis., Mem. Reale Ist. Lombardo Sci. (Ser. 3), C. Sci. Mat. 4 (13): 431, 1877 (Trevisan 1877). Bas.: Jungermannia alcicornis Hook.f. et Taylor, London J. Bot. 3: 479, 1844 (Hooker and Taylor 1844b).

** Riccardia conimitra (Steph.) A.Evans, Trans. Connecticut Acad. Arts 25 (2): 156, 1921 (Evans 1921a). Bas.: Aneura conimitra Steph., Bull. Herb. Boissier 7 (10): 749 (259), 1899 (Stephani 1899f).

** Riccardia corralensis (Steph.) A.Evans, Trans. Connecticut Acad. Arts 25 (2): 143, 1921 (Evans 1921a). Bas.: Aneura corralensis Steph., Bull. Herb. Boissier 7 (10): 742 (252), 1899 (Stephani 1899f).

*** Riccardia furtiva E.A.Br. et Braggins, J. Hattori Bot. Lab. 66: 35, 1989 (Brown and Braggins 1989).

** Riccardia fuscobrunnea (Steph.) A.Evans, Trans. Connecticut Acad. Arts 25 (2): 152, 1921 (Evans 1921a). Bas.: Aneura fuscobrunnea Steph., Kungl. Svenska Vetensk.-Akad. Handl. (n.ser.) 46 (9): 7, 1911 (Stephani 1911b).

** Riccardia longioleata Hässel, Revista Mus. Argent. Ci. Nat., Bernardino Rivadavia Inst. Nac. Invest. Ci. Nat. Bot. 4 (1): 30, 1972 (Hässel 1972a).

*** Riccardia multicorpora E.A.Br., J. Hattori Bot. Lab. 66: 40, 1989 (Brown and Braggins 1989).

* Riccardia umida E.A.Br., J. Hattori Bot. Lab. 66: 38, 1989 (Brown and Braggins 1989).

** **sect. Crassantia Hässel**, Revista Mus. Argent. Ci. Nat., Bernardino Rivadavia Inst. Nac. Invest. Ci. Nat. Bot. 4 (1): 41, 1972 (Hässel 1972a).

*** Riccardia aequitexta (Steph.) E.A.Br., J. Hattori Bot. Lab. 66: 78, 1989 (Brown and Braggins 1989). Bas.: Aneura aequitexta Steph., J. Linn. Soc., Bot. 29 (201): 263, 1892 (Stephani 1892b).

*** Riccardia alba (Colenso) E.A.Br., J. Hattori Bot. Lab. 66: 66, 1989 (Brown and Braggins 1989). Bas.: Aneura alba Colenso, Trans. & Proc. New Zealand Inst. 16: 357, 1884 (Colenso 1884).

** Riccardia amnicola Hässel, Revista Mus. Argent. Ci. Nat., Bernardino Rivadavia Inst. Nac. Invest. Ci. Nat. Bot. 4 (1): 92, 1972 (Hässel 1972a).

** Riccardia bipinnatifida (Colenso) Hewson, Proc. Linn. Soc. New South Wales (ser. 2) 95 (1): 93, 1970 (Hewson 1970a). Bas.: Aneura bipinnatifida Colenso, Trans. & Proc. New Zealand Inst. 16: 358, 1884 (Colenso 1884).

** Riccardia calva (Schiffn.) A.Evans, Trans. Connecticut Acad. Arts 25 (2): 134, 1921 (Evans 1921a). Bas.: Aneura calva Schiffn., Leberm., Forschungsr. Gazelle 4 (4): 42, 1890 (Schiffner 1890).

*** Riccardia colensoi (Steph.) W.Martin, Trans. & Proc. Roy. Soc. New Zealand 78 (4): 499, 1950 (Martin 1950). Bas.: Aneura colensoi Steph., J. Linn. Soc., Bot. 29 (201): 264, 1892 (Stephani 1892b).

*** Riccardia crassa (Schwägr.) C.Massal., Nuovo Giorn. Bot. Ital. 17 (3): 254, 1885 (Massalongo 1885). Bas.: Jungermannia crassa Schwägr., Hist. Musc. Hepat. Prodr.: 31, 1814 (Schwägrichen 1814).

** Riccardia crassicrispa (Steph.) A.Evans, Trans. Connecticut Acad. Arts 25 (2): 194, 1921 (Evans 1921a). Bas.: Aneura crassicrispa Steph., Kungl. Svenska Vetensk.-Akad. Handl. (n.ser.) 46 (9): 6, 1911 (Stephani 1911b).

** Riccardia diderma Hässel, Revista Mus. Argent. Ci. Nat., Bernardino Rivadavia Inst. Nac. Invest. Ci. Nat. Bot. 4 (1): 56, 1972 (Hässel 1972a).

** Riccardia diversiflora A.Evans, Trans. Connecticut Acad. Arts 25 (2): 167, 1921 (Evans 1921a).

** Riccardia diversiflora subsp. paucigyna R.M.Schust., J. Hattori Bot. Lab. 67: 97, 1989 (Schuster 1989).

** Riccardia duriuscula Hässel, Revista Mus. Argent. Ci. Nat., Bernardino Rivadavia Inst. Nac. Invest. Ci. Nat. Bot. 4 (1): 82, 1972 (Hässel 1972a).

** Riccardia falsifloribunda Hässel, Revista Mus. Argent. Ci. Nat., Bernardino Rivadavia Inst. Nac. Invest. Ci. Nat. Bot. 4 (1): 119, 1972 (Hässel 1972a).

** Riccardia floribunda (Steph.) A.Evans, Trans. Connecticut Acad. Arts 25 (2): 182, 1921 (Evans 1921a). Bas.: Aneura floribunda Steph., Bull. Herb. Boissier 7 (10): 749 (259), 1899 (Stephani 1899f).

** Riccardia fluvigena Hässel, Revista Mus. Argent. Ci. Nat., Bernardino Rivadavia Inst. Nac. Invest. Ci. Nat. Bot. 4 (1): 73, 1972 (Hässel 1972a).

** Riccardia georgiensis (Steph.) Hässel, Lindbergia 1 (1/2): 80, 1971 [1972] (Grolle 1971a). Bas.: Aneura georgiensis Steph., Wiss. Ergebn. Schwed. Südpolar-Exped. [1901–1903] 4 (1): 2, 1905 (Stephani 1905e).

** Riccardia georgiensis subsp. sympodea R.M.Schust., J. Hattori Bot. Lab. 67: 83, 1989 (Schuster 1989).

*** Riccardia graeffei (Steph.) Hewson, Proc. Linn. Soc. New South Wales (ser. 2) 95 (1): 118, 1970 (Hewson 1970a). Bas.: Aneura graeffei Steph., Hedwigia 32 (1): 21, 1893 (Stephani 1893a).

*** Riccardia marginata (Colenso) Pearson, Univ. Calif. Publ. Bot. 10 (4): 309, 1923 (Pearson 1923). Bas.: Aneura marginata Colenso, Trans. & Proc. New Zealand Inst. 18: 253, 1886 (Colenso 1886b).

*** Riccardia marginata var. pacifica Furuki, J. Hattori Bot. Lab. 70: 380, 1991 (Furuki 1991).

** Riccardia mycophora A.Evans, Trans. Connecticut Acad. Arts 25 (2): 175, 1921 (Evans 1921a).

** Riccardia negeri (Steph.) A.Evans, Trans. Connecticut Acad. Arts 25 (2): 172, 1921 (Evans 1921a). Bas.: Aneura negeri Steph., Bull. Herb. Boissier 7 (10): 747 (257), 1899 (Stephani 1899f).

** Riccardia nitida (Colenso) E.A.Hodgs., Trans. Roy. Soc. New Zealand, Bot. 3 (4): 92, 1965 (Hodgson 1965). Bas.: Aneura nitida Colenso, Trans. & Proc. New Zealand Inst. 18: 253, 1886 (Colenso 1886b).

** Riccardia papillosa (C.Massal. et Steph.) Hässel, Revista Mus. Argent. Ci. Nat., Bernardino Rivadavia Inst. Nac. Invest. Ci. Nat. Bot. 4 (1): 60, 1972 (Hässel 1972a). Bas.: Aneura papillosa C.Massal. et Steph., Atti Reale Ist. Veneto Sci. Lett. Arti 87 (2): 241, 1928 (Massalongo 1928).

** Riccardia regularis (Steph.) Kühnem., Revista Centro Estud. Doct. Ci. Nat. 1: 171, 1937 (Kühnemann 1937). Bas.: Aneura regularis Steph., Kungl. Svenska Vetensk.-Akad. Handl. (n.ser.) 46 (9): 9, 1911 (Stephani 1911b).

** Riccardia rivularis Hässel, Revista Mus. Argent. Ci. Nat., Bernardino Rivadavia Inst. Nac. Invest. Ci. Nat. Bot. 4 (1): 52, 1972 (Hässel 1972a).

** Riccardia saxicola Hässel, Revista Mus. Argent. Ci. Nat., Bernardino Rivadavia Inst. Nac. Invest. Ci. Nat. Bot. 4 (1): 137, 1972 (Hässel 1972a).

** Riccardia spectabilis (Steph.) A.Evans, Trans. Connecticut Acad. Arts 25 (2): 140, 1921 (Evans 1921a). Bas.: Aneura spectabilis Steph., Bull. Herb. Boissier 7 (10): 746 (256), 1899 (Stephani 1899f).

** Riccardia spegazziniana C.Massal., Nuovo Giorn. Bot. Ital. 17 (3): 254, 1885 (Massalongo 1885).

** Riccardia tenax (Steph.) A.Evans, Trans. Connecticut Acad. Arts 25 (2): 186, 1921 (Evans 1921a). Bas.: Aneura tenax Steph., Bull. Herb. Boissier 7 (10): 755 (265), 1899 (Stephani 1899f).

** Riccardia tenerrima (Steph.) A.Evans, Trans. Connecticut Acad. Arts 25 (2): 164, 1921 (Evans 1921a). Bas.: Aneura tenerrima Steph., Kungl. Svenska Vetensk.-Akad. Handl. (n.ser.) 46 (9): 9, 1911 (Stephani 1911b).

** Riccardia theliophora Hässel, Revista Mus. Argent. Ci. Nat., Bernardino Rivadavia Inst. Nac. Invest. Ci. Nat. Bot. 4 (1): 96, 1972 (Hässel 1972a).

** **sect. Pallidevirida Hässel**, Revista Mus. Argent. Ci. Nat., Bernardino Rivadavia Inst. Nac. Invest. Ci. Nat. Bot. 4 (1): 142, 1972 (Hässel 1972a).

** Riccardia granulata (Steph.) A.Evans, Trans. Connecticut Acad. Arts 25 (2): 192, 1921 (Evans 1921a). Bas.: Aneura granulata Steph., Hedwigia 32 (1): 21, 1893 (Stephani 1893a).

** Riccardia intercellula E.A.Br., J. Hattori Bot. Lab. 66: 58, 1989 (Brown and Braggins 1989).

** Riccardia opuntiiformis S.W.Arnell, Arch. Soc. Zool. Bot. Fenn. “Vanamo” 9: 52, 1954 (Arnell 1954b).

** Riccardia pallidevirens (Steph.) A.Evans, Trans. Connecticut Acad. Arts 25 (2): 189, 1921 (Evans 1921a). Bas.: Aneura pallidevirens Steph., Bull. Herb. Boissier 7 (10): 762 (272), 1899 (Stephani 1899f).

*** Riccardia pennata E.A.Br., J. Hattori Bot. Lab. 66: 53, 1989 (Brown and Braggins 1989).

*** Riccardia perspicua E.A.Br., J. Hattori Bot. Lab. 66: 55, 1989 (Brown and Braggins 1989).

** Riccardia xylophila Hässel, Revista Mus. Argent. Ci. Nat., Bernardino Rivadavia Inst. Nac. Invest. Ci. Nat. Bot. 4 (1): 156, 1972 (Hässel 1972a).

** **sect. Riccardia**

*** Riccardia australis (Lehm.) Hewson, Proc. Linn. Soc. New South Wales (ser. 2) 95 (1): 110, 1970 (Hewson 1970a). Bas.: Sarcomitrium australe Lehm., Nov. Stirp. Pug. 10: 19, 1857 (Lehmann 1857).

** Riccardia autoica (Steph.) A.Evans, Trans. Connecticut Acad. Arts 25 (2): 159, 1921 (Evans 1921a). Bas.: Aneura autoica Steph., Bull. Herb. Boissier 7 (9): 691 (232), 1899 (Stephani 1899e).

** Riccardia breviala E.A.Br., J. Hattori Bot. Lab. 66: 46, 1989 (Brown and Braggins 1989).

** Riccardia breviramosa (Steph.) A.Evans, Nat. Hist. Juan Fernandez (Botany) 2 (20): 559, 1930 (Evans 1930a). Bas.: Aneura breviramosa Steph., Kungl. Svenska Vetensk.-Akad. Handl. (n.ser.) 46 (9): 6, 1911 (Stephani 1911b).

** Riccardia filicina (Colenso) E.A.Hodgs., Rec. Domin. Mus. 4 (11): 129, 1962 (Hodgson 1962a). Bas.: Aneura filicina Colenso, Trans. & Proc. New Zealand Inst. 16: 358, 1884 (Colenso 1884).

** Riccardia leptostachya A.Evans, Nat. Hist. Juan Fernandez (Botany) 2 (20): 570, 1930 (Evans 1930a).

** Riccardia lobulata (Colenso) E.A.Hodgs., Trans. Roy. Soc. New Zealand, Bot. 3 (4): 92, 1965 (Hodgson 1965). Bas.: Zoopsis lobulata Colenso, Trans. & Proc. New Zealand Inst. 18: 250, 1886 (Colenso 1886b).

** Riccardia mejlandii S.W.Arnell, Results Norweg. Sci. Exped. Tristan da Cunha 42: 30, 1958 (Arnell 1958b).

*** Riccardia multifida (L.) Gray, Nat. Arr. Brit. Pl. 1: 684, 1821 (Gray 1821). Bas.: Jungermannia multifida L., Sp. Pl. 1: 1136, 1753 (Linnaeus 1753).

*** Riccardia multifida subsp. decrescens (Steph.) Furuki, J. Hattori Bot. Lab. 70: 341, 1991 (Furuki 1991). Bas.: Aneura decrescens Steph., Bull. Herb. Boissier 7 (9): 686 (227), 1899 (Stephani 1899e).

** Riccardia multifida subsp. synoica R.M.Schust., J. Hattori Bot. Lab. 62: 319, 1987 (Schuster 1987d).

** Riccardia multioleata Hässel, Revista Mus. Argent. Ci. Nat., Bernardino Rivadavia Inst. Nac. Invest. Ci. Nat. Bot. 4 (1): 195, 1972 (Hässel 1972a).

*** Riccardia papulosa (Steph.) E.A.Br., J. Hattori Bot. Lab. 66: 49, 1989 (Brown and Braggins 1989). Bas.: Aneura papulosa Steph., Hedwigia 32 (1): 25, 1893 (Stephani 1893a).

** Riccardia patens Hässel, Revista Mus. Argent. Ci. Nat., Bernardino Rivadavia Inst. Nac. Invest. Ci. Nat. Bot. 4 (1): 165, 1972 (Hässel 1972a).

** Riccardia polyclada (Mitt.) Hässel, Bryologist 109 (1): 34, 2006 (Hässel 2006b). Bas.: Aneura polyclada Mitt., Trans. Linn. Soc. London, Bot. 2 (13): 297, 1887 (Mitten 1887).

** Riccardia statensis Hässel, Revista Mus. Argent. Ci. Nat., Bernardino Rivadavia Inst. Nac. Invest. Ci. Nat. Bot. 4 (1): 177, 1972 (Hässel 1972a).

** Riccardia thaxteri A.Evans, Trans. Connecticut Acad. Arts 25 (2): 126, 1921 (Evans 1921a).

** Riccardia tristaniana S.W.Arnell, Results Norweg. Sci. Exped. Tristan da Cunha 42: 31, 1958 (Arnell 1958b).

** **subg. Spinella (Schiffn.) Hässel**, Revista Mus. Argent. Ci. Nat., Bernardino Rivadavia Inst. Nac. Invest. Ci. Nat. Bot. 4 (1): 214, 1972 (Hässel 1972a). Bas.: Spinella Schiffn., Leberm., Forschungsr. Gazelle 4 (4): 41, 1890 (Schiffner 1890).

** Riccardia spinulifera C.Massal., Nuovo Giorn. Bot. Ital. 17 (3): 254, 1885 (Massalongo 1885).

** **subg. Thornoneura Furuki**, J. Hattori Bot. Lab. 70: 382, 1991 (Furuki 1991).

* Riccardia baumannii Hürl., Bauhinia 5 (4): 203, 1976 (Hürlimann 1976). ^400^

** Riccardia deguchii Furuki et K.T.Yong, Hikobia 16 (3): 285, 2013 (Furuki et al. 2013).

*** Riccardia grossitexta (Steph.) Furuki, J. Hattori Bot. Lab. 70: 382, 1991 (Furuki 1991). Bas.: Aneura grossitexta Steph., Sp. Hepat. (Stephani) 6: 29, 1917 (Stephani 1917a).

** Riccardia inconspicua (Steph.) Reeb et Bardat, Cryptog. Bryol. 35 (1): 61, 2014 (Reeb and Bardat 2014). Bas.: Aneura inconspicua Steph., Hedwigia 32 (1): 23, 1893 (Stephani 1893a).

** **subg. Trichothallia Hässel**, Revista Mus. Argent. Ci. Nat., Bernardino Rivadavia Inst. Nac. Invest. Ci. Nat. Bot. 4 (1): 224, 1972 (Hässel 1972a).

** Riccardia hyalitricha Hässel, Revista Mus. Argent. Ci. Nat., Bernardino Rivadavia Inst. Nac. Invest. Ci. Nat. Bot. 4 (1): 225, 1972 (Hässel 1972a).

***Incertae sedis***

** Riccardia aberrans (Steph.) Gradst., J. Hattori Bot. Lab. 45: 129, 1979 (Gradstein and Hekking 1979). Bas.: Aneura aberrans Steph., Hedwigia 32 (1): 18, 1893 (Stephani 1893a).

** Riccardia agumana Hewson, Proc. Linn. Soc. New South Wales (ser. 2) 95 (1): 82, 1970 (Hewson 1970a).

** Riccardia algoides (Taylor) Meenks, J. Hattori Bot. Lab. 62: 168, 1987 (Meenks 1987). Bas.: Metzgeria algoides Taylor, London J. Bot. 5: 410, 1846 (Taylor 1846b).

*** Riccardia amazonica (Spruce) Schiffn. ex Gradst. et Hekking, J. Hattori Bot. Lab. 45: 129, 1979 (Gradstein and Hekking 1979). Bas.: Aneura amazonica Spruce, Trans. & Proc. Bot. Soc. Edinburgh 15: 545, 1885 (Spruce 1885).

*** Riccardia andina (Spruce) Herzog, Svensk Bot. Tidskr. 46 (1): 65, 1952 (Herzog 1952e). Bas.: Aneura andina Spruce, Trans. & Proc. Bot. Soc. Edinburgh 15: 548, 1885 (Spruce 1885).

** Riccardia angustata Horik., J. Sci. Hiroshima Univ., Ser. B, Div. 2, Bot. 2: 126, 1934 (Horikawa 1934).

** Riccardia angustealata (Steph.) Hewson, Proc. Linn. Soc. New South Wales (ser. 2) 95 (1): 111, 1970 (Hewson 1970a). Bas.: Aneura angustealata Steph., Sp. Hepat. (Stephani) 6: 20, 1917 (Stephani 1917a).

** Riccardia angustissima (Steph.) H.A.Mill., Phytologia 47 (4): 323, 1981 (Miller 1981). Bas.: Aneura angustissima Steph., Sp. Hepat. (Stephani) 6: 20, 1917 (Stephani 1917a).

** Riccardia aspera (Steph.) Grolle, J. Hattori Bot. Lab. 30: 117, 1967 (Grolle 1967b). Bas.: Aneura aspera Steph., Sp. Hepat. (Stephani) 6: 21, 1917 (Stephani 1917a).

* Riccardia baldwinii (Steph.) H.A.Mill., Ark. Bot. (n.ser.) 5 (2): 527, 1963 (Miller 1963). Bas.: Aneura baldwinii Steph., Bull. Herb. Boissier 7 (10): 743 (253), 1899 (Stephani 1899f). ^401^

** Riccardia barbiflora (Steph.) Piippo, J. Hattori Bot. Lab. 68: 134, 1990 (Piippo 1990). Bas.: Aneura barbiflora Steph., Mém. Soc. Nat. Sci. Nat. Math. Cherbourg 29: 209, 1894 (Stephani 1894b).

** Riccardia bogotensis (Gottsche) Pagán, Bryologist 42 (1): 6, 1939 (Pagán 1939a). Bas.: Pseudoneura bogotensis Gottsche, Ann. Sci. Nat. Bot. (sér. 5) 1: 184, 1864 (Gottsche 1864).

** Riccardia boliviensis (Steph.) Meenks, J. Hattori Bot. Lab. 62: 170, 1987 (Meenks 1987). Bas.: Aneura boliviensis Steph., Biblioth. Bot. 87 (2): 176, 1916 (Stephani 1916a).

** Riccardia bongeriana Hewson, Proc. Linn. Soc. New South Wales (ser. 2) 95 (1): 101, 1970 (Hewson 1970a).

** Riccardia brunnea (Steph.) S.Hatt., Bull. Tokyo Sci. Mus. 11: 164, 1944 (Hattori 1944d). Bas.: Aneura brunnea Steph., Sp. Hepat. (Stephani) 6: 21, 1917 (Stephani 1917a).

** Riccardia calcarea (Steph.) Meenks, J. Hattori Bot. Lab. 62: 170, 1987 (Meenks 1987). Bas.: Aneura calcarea Steph., Bull. Herb. Boissier 7 (10): 756 (266), 1899 (Stephani 1899f).

** Riccardia capillacea (Steph.) Meenks et C.De Jong, Cryptog. Bryol. Lichénol. 6 (1): 6, 1985 (Meenks and de Jong 1985). Bas.: Aneura capillacea Steph., Biblioth. Bot. 87 (2): 174, 1916 (Stephani 1916a).

** Riccardia capillacea var. dentata Meenks, J. Hattori Bot. Lab. 62: 170, 1987 (Meenks 1987).

** Riccardia cardotii (Steph.) Pandé et S.C.Srivast., Biol. Mem. 1 (1/2): 131, 1976 (Srivastava and Udar 1976). Bas.: Aneura cardotii Steph., Bull. Soc. Roy. Bot. Belgique, Mém. 41 (1): 118, 1904 [1905] (Stephani 1904d).

** Riccardia cataractarum (Spruce) Schiffn., Österr. Akad. Wiss., Math.-Naturwiss. Kl., Denkschr. 111: 18, 1964 (Schiffner and Arnell 1964). Bas.: Aneura cataractarum Spruce, Bull. Soc. Bot. France (Congr. Bot.) 36: cxcv, 1889 [1890] (Spruce 1889).

*** Riccardia cervicornis (Spruce) Herzog ex Gradst. et Hekking, J. Hattori Bot. Lab. 45: 129, 1979 (Gradstein and Hekking 1979). Bas.: Aneura cervicornis Spruce, Trans. & Proc. Bot. Soc. Edinburgh 15: 550, 1885 (Spruce 1885).

** Riccardia changbaishanensis C.Gao, Fl. Hepat. Chin. Boreali-Orient.: 209, 1981 (Gao and Chang 1981).

** Riccardia chinensis C.Gao, Fl. Hepat. Chin. Boreali-Orient.: 209, 1981 (Gao and Chang 1981).

** Riccardia ciliolata (Spruce) Horik., J. Sci. Hiroshima Univ., Ser. B, Div. 2, Bot. 1: 198, 1933 (Horikawa 1933). Bas.: Aneura ciliolata Spruce, Trans. & Proc. Bot. Soc. Edinburgh 15: 547, 1885 (Spruce 1885).

** Riccardia columbica (Steph.) Hässel ex Gradst. et Hekking, J. Hattori Bot. Lab. 45: 130, 1979 (Gradstein and Hekking 1979). Bas.: Aneura columbica Steph., Sp. Hepat. (Stephani) 6: 22, 1917 (Stephani 1917a).

** Riccardia comata (Steph.) H.A.Mill., Phytologia 47 (4): 323, 1981 (Miller 1981). Bas.: Aneura comata Steph., Sp. Hepat. (Stephani) 6: 22, 1917 (Stephani 1917a).

** Riccardia compacta (Steph.) S.W.Arnell, Bot. Not. 105: 141, 1952 (Arnell 1952b). Bas.: Aneura compacta Steph., Hedwigia 32 (1): 19, 1893 (Stephani 1893a).

** Riccardia comptonii (Pearson) H.A.Mill., Phytologia 47 (4): 323, 1981 (Miller 1981). Bas.: Aneura comptonii Pearson, J. Linn. Soc., Bot. 46 (305): 17, 1922 (Pearson 1922b).

** Riccardia costata (Steph.) Hürl., Bauhinia 5 (4): 206, 1976 (Hürlimann 1976). Bas.: Aneura costata Steph., Sp. Hepat. (Stephani) 6: 23, 1917 (Stephani 1917a).

*** Riccardia crassicaulis (Steph.) Meenks et C.De Jong, Cryptog. Bryol. Lichénol. 6 (1): 8, 1985 (Meenks and de Jong 1985). Bas.: Aneura crassicaulis Steph., Biblioth. Bot. 87 (2): 174, 1916 (Stephani 1916a).

** Riccardia crassiretis Schiffn., Denkschr. Kaiserl. Akad. Wiss., Math.-Naturwiss. Kl. 67: 173, 1898 (Schiffner 1898a).

** Riccardia crenulata Schiffn., Denkschr. Kaiserl. Akad. Wiss., Math.-Naturwiss. Kl. 67: 173, 1898 (Schiffner 1898a).

** Riccardia crenuliformis R.M.Schust., J. Hattori Bot. Lab. 67: 72, 1989 (Schuster 1989).

* Riccardia decolyana Schiffn., J. Indian Bot. Soc. 38 (4): 538, 1959 [1960] (Schiffner et al. 1959). ^402^

** Riccardia densiramea (Steph.) S.Hatt., Bull. Tokyo Sci. Mus. 11: 164, 1944 (Hattori 1944d). Bas.: Aneura densiramea Steph., Sp. Hepat. (Stephani) 6: 24, 1917 (Stephani 1917a).

** Riccardia devexa Schiffn., Österr. Akad. Wiss., Math.-Naturwiss. Kl., Denkschr. 111: 12, 1964 (Schiffner and Arnell 1964).

** Riccardia diablotina (Spruce) Pagán, Bryologist 45 (4): 80, 1942 (Pagán 1942b). Bas.: Aneura diablotina Spruce, J. Linn. Soc., Bot. 30 (210): 366, 1895 (Gepp 1895b).

*** Riccardia digitiloba (Spruce) Pagán, Bryologist 42 (1): 6, 1939 (Pagán 1939a). Bas.: Aneura digitiloba Spruce, Bull. Soc. Bot. France (Congr. Bot.) 36: cci, 1889 [1890] (Spruce 1889).

** Riccardia dilatata (Spruce) Schäf.-Verw. et Pócs, Cryptog. Bryol. 31 (4): 389, 2010 (Schäfer-Verwimp 2010). Bas.: Aneura dilatata Spruce, J. Linn. Soc., Bot. 30 (210): 368, 1895 (Gepp 1895b).

* Riccardia diminuta Schiffn., Denkschr. Kaiserl. Akad. Wiss., Math.-Naturwiss. Kl. 67: 170, 1898 (Schiffner 1898a). ^403^

* Riccardia diminuta var. thermarum Schiffn., Denkschr. Kaiserl. Akad. Wiss., Math.-Naturwiss. Kl. 67: 172, 1898 (Schiffner 1898a).

** Riccardia distans (Spruce) Pagán, Bryologist 45 (4): 80, 1942 (Pagán 1942b). Bas.: Aneura distans Spruce, J. Linn. Soc., Bot. 30 (210): 367, 1895 (Gepp 1895b).

** Riccardia elata (Steph.) Schiffn., Denkschr. Kaiserl. Akad. Wiss., Math.-Naturwiss. Kl. 67: 169, 1898 (Schiffner 1898a). Bas.: Aneura elata Steph., Hedwigia 32 (1): 19, 1893 (Stephani 1893a).

* Riccardia elata var. flaccida Schiffn., Denkschr. Kaiserl. Akad. Wiss., Math.-Naturwiss. Kl. 67: 170, 1898 (Schiffner 1898a).

* Riccardia elata var. intercedens Schiffn., Denkschr. Kaiserl. Akad. Wiss., Math.-Naturwiss. Kl. 67: 170, 1898 (Schiffner 1898a).

** Riccardia elegans (Steph.) Hürl., Bauhinia 5 (4): 196, 1976 (Hürlimann 1976). Bas.: Aneura elegans Steph., Sp. Hepat. (Stephani) 6: 25, 1917 (Stephani 1917a).

** Riccardia elisabethae Thouvenot et Reeb, Telopea 17: 229, 2014 (Thouvenot and Reeb 2014).

*** Riccardia emarginata (Steph.) K.G.Hell, Bol. Fac. Filos. Univ. São Paulo, Bot. 25: 100, 1969 (Hell 1969). Bas.: Aneura emarginata Steph., Hedwigia 32 (1): 20, 1893 (Stephani 1893a).

** Riccardia erosa (Steph.) E.W.Jones, Trans. Brit. Bryol. Soc. 3 (1): 83, 1956 (Jones 1956). Bas.: Aneura erosa Steph., Hedwigia 30 (6): 269, 1891 (Stephani 1891c).

** Riccardia fastigiata (Lehm.) Trevis., Mem. Reale Ist. Lombardo Sci. (Ser. 3), C. Sci. Mat. 4 (13): 431, 1877 (Trevisan 1877). Bas.: Jungermannia fastigiata Lehm., Linnaea 4: 370, 1829 (Lehmann 1829).

** Riccardia fendleri (Steph.) Pagán, Bryologist 45 (4): 80, 1942 (Pagán 1942b). Bas.: Aneura fendleri Steph., Hedwigia 32 (1): 20, 1893 (Stephani 1893a).

** Riccardia flaccida (Steph.) S.Hatt., Bull. Tokyo Sci. Mus. 11: 164, 1944 (Hattori 1944d). Bas.: Aneura flaccida Steph., Sp. Hepat. (Stephani) 6: 26, 1917 (Stephani 1917a).

** Riccardia flaccidissima Schiffn., Denkschr. Kaiserl. Akad. Wiss., Math.-Naturwiss. Kl. 67: 167, 1898 (Schiffner 1898a).

** Riccardia flagellaris (A.Gepp) H.A.Mill., Phytologia 47 (4): 323, 1981 (Miller 1981). Bas.: Aneura flagellaris A.Gepp, J. Linn. Soc., Bot. 39 (270): 194, 1909 (Gibbs 1909).

** Riccardia flagellifrons C.Gao, Fl. Hepat. Chin. Boreali-Orient.: 209, 1981 (Gao and Chang 1981).

** Riccardia fleischeri (Steph.) H.A.Mill., Ark. Bot. (n.ser.) 5 (2): 527, 1963 (Miller 1963). Bas.: Aneura fleischeri Steph., Sp. Hepat. (Stephani) 6: 26, 1917 (Stephani 1917a).

** Riccardia foliacea Meenks et C.De Jong, Cryptog. Bryol. Lichénol. 6 (1): 8, 1985 (Meenks and de Jong 1985).

** Riccardia formosensis (Steph.) Horik., J. Sci. Hiroshima Univ., Ser. B, Div. 2, Bot. 2: 125, 1934 (Horikawa 1934). Bas.: Aneura formosensis Steph., Sp. Hepat. (Stephani) 6: 27, 1917 (Stephani 1917a).

*** Riccardia fucoidea (Sw.) C.Massal., Nuovo Giorn. Bot. Ital. 17 (3): 256, 1885 (Massalongo 1885). Bas.: Jungermannia fucoidea Sw., Prodr. (Swartz): 145, 1788 (Swartz 1788).

** Riccardia geniana Hewson, Proc. Linn. Soc. New South Wales (ser. 2) 95 (1): 76, 1970 (Hewson 1970a).

*** Riccardia glaziovii (Spruce) Meenks, J. Hattori Bot. Lab. 62: 173, 1987 (Meenks 1987). Bas.: Aneura glaziovii Spruce, Bull. Soc. Bot. France (Congr. Bot.) 36: cci, 1889 [1890] (Spruce 1889).

** Riccardia gogolensis (Steph.) Hewson, Proc. Linn. Soc. New South Wales (ser. 2) 95 (1): 102, 1970 (Hewson 1970a). Bas.: Aneura gogolensis Steph., Bull. Herb. Boissier 7 (9): 689 (230), 1899 (Stephani 1899e).

** Riccardia gracilis (Steph.) R.M.Schust., J. Hattori Bot. Lab. 26: 295, 1963 (Schuster 1963b). Bas.: Aneura gracilis Steph., Bull. Herb. Boissier 7 (10): 752 (262), 1899 (Stephani 1899f).

** Riccardia grandiflora (Steph.) H.A.Mill., Ark. Bot. (n.ser.) 5 (2): 528, 1963 (Miller 1963). Bas.: Aneura grandiflora Steph., Sp. Hepat. (Stephani) 6: 29, 1917 (Stephani 1917a).

*** Riccardia grollei Furuki, Haussknechtia, Beih. 9: 139, 1999 (Furuki 1999).

** Riccardia grossidens (Steph.) Pagán, Bryologist 45 (4): 80, 1942 (Pagán 1942b). Bas.: Aneura grossidens Steph., Hedwigia 32 (1): 23, 1893 (Stephani 1893a).

** Riccardia gunniana (Steph.) H.A.Mill., Phytologia 47 (4): 323, 1981 (Miller 1981). Bas.: Aneura gunniana Steph., J. & Proc. Roy. Soc. New South Wales 48 (1/2): 96, 1914 (Stephani and Watts 1914).

** Riccardia hamatiflora (Steph.) H.A.Mill., Ark. Bot. (n.ser.) 5 (2): 526, 1963 (Miller 1963). Bas.: Aneura hamatiflora Steph., Bull. Herb. Boissier 5 (10): 844, 1897 (Stephani 1897c).

*** Riccardia hans-meyeri (Steph.) Meenks et C.De Jong, Cryptog. Bryol. Lichénol. 6 (1): 12, 1985 (Meenks and de Jong 1985). Bas.: Aneura hans-meyeri Steph., Sp. Hepat. (Stephani) 6: 29, 1917 (Stephani 1917a).

** Riccardia hans-meyeri var. dentata Meenks, J. Hattori Bot. Lab. 62: 173, 1987 (Meenks 1987).

** Riccardia hawaica (Steph.) H.A.Mill., Ark. Bot. (n.ser.) 5 (2): 528, 1963 (Miller 1963). Bas.: Aneura hawaica Steph., Sp. Hepat. (Stephani) 6: 30, 1917 (Stephani 1917a).

** Riccardia hebridensis (Steph.) H.A.Mill., Phytologia 47 (4): 323, 1981 (Miller 1981). Bas.: Aneura hebridensis Steph., J. & Proc. Roy. Soc. New South Wales 48 (1/2): 96, 1914 (Stephani and Watts 1914).

** Riccardia herzogiana (Steph.) Meenks et C.De Jong, Cryptog. Bryol. Lichénol. 6 (1): 12, 1985 (Meenks and de Jong 1985). Bas.: Aneura herzogiana Steph., Biblioth. Bot. 87 (2): 175, 1916 (Stephani 1916a).

** Riccardia heteroclada Schiffn., Denkschr. Kaiserl. Akad. Wiss., Math.-Naturwiss. Kl. 67: 175, 1898 (Schiffner 1898a).

* Riccardia hirtiflora (Steph.) Schiffn., Österr. Akad. Wiss., Math.-Naturwiss. Kl., Denkschr. 111: 19, 1964 (Schiffner and Arnell 1964). Bas.: Aneura hirtiflora Steph., Arch. Mus. Nac. Rio de Janeiro 13: 116, 1905 (Stephani 1905c). ^404^

** Riccardia humilis (Gottsche) O.Yano, J. Hattori Bot. Lab. 56: 530, 1984 (Yano 1984). Bas.: Pseudoneura humilis Gottsche, Mexik. Leverm.: 260, 1863 (Gottsche 1863).

** Riccardia hyalina (Steph.) H.A.Mill., Phytologia 47 (4): 324, 1981 (Miller 1981). Bas.: Aneura hyalina Steph., Sp. Hepat. (Stephani) 6: 31, 1917 (Stephani 1917a).

** Riccardia hydra Hürl., Bauhinia 5 (4): 210, 1976 (Hürlimann 1976).

** Riccardia hymenophylloides Schiffn., Denkschr. Kaiserl. Akad. Wiss., Math.-Naturwiss. Kl. 67: 175, 1898 (Schiffner 1898a).

* Riccardia hymenophylloides var. flaccida Schiffn., Denkschr. Kaiserl. Akad. Wiss., Math.-Naturwiss. Kl. 67: 176, 1898 (Schiffner 1898a).

*** Riccardia hymenophytoides (Spruce) Meenks, Beih. Nova Hedwigia 88: 101, 1987 (Schultze-Motel and Menzel 1987). Bas.: Aneura hymenophytoides Spruce, Trans. & Proc. Bot. Soc. Edinburgh 15: 549, 1885 (Spruce 1885).

** Riccardia hypipamensis Hewson, Proc. Linn. Soc. New South Wales (ser. 2) 95 (1): 97, 1970 (Hewson 1970a).

** Riccardia ibana Hewson, Proc. Linn. Soc. New South Wales (ser. 2) 95 (1): 78, 1970 (Hewson 1970a).

*** Riccardia incurvata Lindb., Helsingf. Dagbl. 1878 (315, 18 Nov.): 2, 1878 (Lindberg 1878).

** Riccardia innovans (Steph.) Pagán, Bryologist 45 (4): 80, 1942 (Pagán 1942b). Bas.: Aneura innovans Steph., Symb. Antill. 2: 470, 1901 (Stephani 1901f).

** Riccardia insularis Schiffn., Deutsche Südpolar-Exped. 1901-1903, 8 (bot.) 1: 66, 1906 (Schiffner 1906a).

** Riccardia intricata (Steph.) H.A.Mill., Phytologia 47 (4): 324, 1981 (Miller 1981). Bas.: Aneura intricata Steph., Bot. Jahrb. Syst. 23 (1/2, 3): 301, 1896 (Stephani 1896a).

** Riccardia jackii Schiffn., Denkschr. Kaiserl. Akad. Wiss., Math.-Naturwiss. Kl. 67: 165, 1898 (Schiffner 1898a).

* Riccardia jackii var. densa Schiffn., Denkschr. Kaiserl. Akad. Wiss., Math.-Naturwiss. Kl. 67: 165, 1898 (Schiffner 1898a).

** Riccardia judithae Meenks et C.De Jong, Cryptog. Bryol. Lichénol. 6 (1): 12, 1985 (Meenks and de Jong 1985).

*** Riccardia jugata R.M.Schust., J. Hattori Bot. Lab. 62: 305, 1987 (Schuster 1987d).

* Riccardia karstenii (Steph.) Schiffn., Denkschr. Kaiserl. Akad. Wiss., Math.-Naturwiss. Kl. 67: 177, 1898 (Schiffner 1898a). Bas.: Aneura karstenii Steph., Hedwigia 32 (1): 23, 1893 (Stephani 1893a).

** Riccardia laticostata (Spruce) Schiffn., Österr. Akad. Wiss., Math.-Naturwiss. Kl., Denkschr. 111: 12, 1964 (Schiffner and Arnell 1964). Bas.: Aneura laticostata Spruce, J. Linn. Soc., Bot. 30 (210): 367, 1895 (Gepp 1895b).

** Riccardia latifrondoides Schiffn., Denkschr. Kaiserl. Akad. Wiss., Math.-Naturwiss. Kl. 67: 168, 1898 (Schiffner 1898a).

** Riccardia lepidomitra (Spruce) Gradst., J. Hattori Bot. Lab. 45: 130, 1979 (Gradstein and Hekking 1979). Bas.: Aneura lepidomitra Spruce, Trans. & Proc. Bot. Soc. Edinburgh 15: 549, 1885 (Spruce 1885).

** Riccardia leptophylla (Spruce) Herzog, Svensk Bot. Tidskr. 46 (1): 65, 1952 (Herzog 1952e). Bas.: Aneura leptophylla Spruce, Trans. & Proc. Bot. Soc. Edinburgh 15: 544, 1885 (Spruce 1885).

** Riccardia leptothallus R.M.Schust., J. Hattori Bot. Lab. 67: 87, 1989 (Schuster 1989).

** Riccardia levieri Schiffn., Österr. Bot. Z. 49 (4): 130, 1899 (Schiffner 1899b).

** Riccardia lichenoides (Steph.) H.A.Mill., Phytologia 47 (4): 324, 1981 (Miller 1981). Bas.: Aneura lichenoides Steph., Bot. Jahrb. Syst. 23 (1/2, 3): 301, 1896 (Stephani 1896a).

** Riccardia ligulata (Steph.) Pócs et Schäf.-Verw., Cryptog. Bryol. 31 (4): 390, 2010 (Schäfer-Verwimp 2010). Bas.: Aneura ligulata Steph., Sp. Hepat. (Stephani) 6: 32, 1917 (Stephani 1917a).

** Riccardia lilliena (Steph.) H.A.Mill., Ark. Bot. (n.ser.) 5 (2): 527, 1963 (Miller 1963). Bas.: Aneura lilliena Steph., Sp. Hepat. (Stephani) 6: 33, 1917 (Stephani 1917a).

** Riccardia limbata (Steph.) E.W.Jones, Trans. Brit. Bryol. Soc. 3 (1): 79, 1956 (Jones 1956). Bas.: Aneura limbata Steph., Hedwigia 30 (5): 203, 1891 (Stephani 1891a).

** Riccardia loefgrenii Schiffn., Österr. Akad. Wiss., Math.-Naturwiss. Kl., Denkschr. 111: 14, 1964 (Schiffner and Arnell 1964).

** Riccardia longiflora (Steph.) Hewson, Proc. Linn. Soc. New South Wales (ser. 2) 95 (1): 88, 1970 (Hewson 1970a). Bas.: Aneura longiflora Steph., Bull. Herb. Boissier 7 (10): 746 (256), 1899 (Stephani 1899f).

** Riccardia longispica (Steph.) Pearson, Forh. Vidensk.-Selsk. Kristiania 1892 (14): 4, 1893 (Pearson 1893). Bas.: Aneura longispica Steph., Bot. Gaz. 15 (11): 281, 1890 (Stephani 1890c).

** Riccardia loriana (Steph.) H.A.Mill., Ark. Bot. (n.ser.) 5 (2): 528, 1963 (Miller 1963). Bas.: Aneura loriana Steph., Bull. Herb. Boissier 7 (10): 733 (243), 1899 (Stephani 1899f).

** Riccardia macdonaldiana Hewson, Proc. Linn. Soc. New South Wales (ser. 2) 95 (1): 93, 1970 (Hewson 1970a).

** Riccardia macrantha (Pearson) H.A.Mill., Phytologia 47 (4): 324, 1981 (Miller 1981). Bas.: Aneura macrantha Pearson, J. Linn. Soc., Bot. 46 (305): 17, 1922 (Pearson 1922b).

*** Riccardia magnicellularis Furuki, J. Hattori Bot. Lab. 100: 93, 2006 (Furuki 2006a).

*** Riccardia metzgeriiformis (Steph.) R.M.Schust., J. Hattori Bot. Lab. 26: 295, 1963 (Schuster 1963b). Bas.: Aneura metzgeriiformis Steph., Bull. Herb. Boissier 7 (10): 753 (263), 1899 (Stephani 1899f).

** Riccardia microscopica (Nees) Kuntze, Revis. Gen. Pl. 2: 838, 1891 (Kuntze 1891). Bas.: Aneura microscopica Nees, Syn. Hepat. 4: 500, 1846 (Gottsche et al. 1846).

** Riccardia minuta (Steph.) W.Martin, Trans. & Proc. Roy. Soc. New Zealand 78 (4): 499, 1950 (Martin 1950). Bas.: Aneura minuta Steph., Sp. Hepat. (Stephani) 6: 34, 1917 (Stephani 1917a).

* Riccardia multifidoides Schiffn., Denkschr. Kaiserl. Akad. Wiss., Math.-Naturwiss. Kl. 67: 166, 1898 (Schiffner 1898a). ^405^

** Riccardia multispica (Steph.) S.Hatt., Bull. Tokyo Sci. Mus. 11: 164, 1944 (Hattori 1944d). Bas.: Aneura multispica Steph., Sp. Hepat. (Stephani) 6: 34, 1917 (Stephani 1917a).

** Riccardia nadeaudii (Steph.) Hürl., Bauhinia 5 (4): 201, 1976 (Hürlimann 1976). Bas.: Aneura nadeaudii Steph., Bull. Herb. Boissier 7 (10): 750 (260), 1899 (Stephani 1899f).

** Riccardia newellana (Steph.) H.A.Mill., Ark. Bot. (n.ser.) 5 (2): 528, 1963 (Miller 1963). Bas.: Aneura newellana Steph., Sp. Hepat. (Stephani) 6: 35, 1917 (Stephani 1917a).

** Riccardia nigra (Steph.) H.A.Mill., Ark. Bot. (n.ser.) 5 (2): 527, 1963 (Miller 1963). Bas.: Aneura nigra Steph., Sp. Hepat. (Stephani) 6: 35, 1917 (Stephani 1917a).

** Riccardia nobilis (Steph.) Schiffn., Consp. Hepat. Arch. Ind.: 56, 1898 (Schiffner 1898b). Bas.: Aneura nobilis Steph., Hedwigia 32 (1): 24, 1893 (Stephani 1893a).

** Riccardia novo-amstelodamensis Schiffn., Deutsche Südpolar-Exped. 1901-1903, 8 (bot.) 1: 65, 1906 (Schiffner 1906a).

** Riccardia nudiflora (Steph.) Grolle, Bryophyt. Biblioth. 48: 130, 1995 (Grolle 1995). Bas.: Aneura nudiflora Steph., Bot. Gaz. 15 (11): 282, 1890 (Stephani 1890c).

** Riccardia obtusa S.W.Arnell, Bot. Not. 105: 142, 1952 (Arnell 1952b).

** Riccardia obtusifrons (Steph.) H.A.Mill., Ark. Bot. (n.ser.) 5 (2): 527, 1963 (Miller 1963). Bas.: Aneura obtusifrons Steph., Sp. Hepat. (Stephani) 6: 36, 1917 (Stephani 1917a).

** Riccardia omkaliensis Hewson, Proc. Linn. Soc. New South Wales (ser. 2) 95 (1): 97, 1970 (Hewson 1970a).

*** Riccardia pallida (Spruce) Meenks et C.De Jong, Cryptog. Bryol. Lichénol. 6 (1): 15, 1985 (Meenks and de Jong 1985). Bas.: Aneura pallida Spruce, Trans. & Proc. Bot. Soc. Edinburgh 15: 547, 1885 (Spruce 1885).

** Riccardia palmatifida (Steph.) H.A.Mill., Phytologia 47 (4): 324, 1981 (Miller 1981). Bas.: Aneura palmatifida Steph., Sp. Hepat. (Stephani) 6: 36, 1917 (Stephani 1917a).

* Riccardia palmatiformis Schiffn., J. Indian Bot. Soc. 38 (4): 538, 1959 [1960] (Schiffner et al. 1959). ^406^

** Riccardia papillata (Gottsche) Hässel ex Gradst. et Hekking, J. Hattori Bot. Lab. 45: 130, 1979 (Gradstein and Hekking 1979). Bas.: Pseudoneura papillata Gottsche, Ann. Sci. Nat. Bot. (sér. 5) 1: 185, 1864 (Gottsche 1864).

** Riccardia paramorum Meenks, J. Hattori Bot. Lab. 62: 176, 1987 (Meenks 1987).

*** Riccardia parasitans (Steph.) Meenks et C.De Jong, Cryptog. Bryol. Lichénol. 6 (1): 17, 1985 (Meenks and de Jong 1985). Bas.: Aneura parasitans Steph., Biblioth. Bot. 87 (2): 175, 1916 (Stephani 1916a).

** Riccardia parvula Schiffn., Denkschr. Kaiserl. Akad. Wiss., Math.-Naturwiss. Kl. 67: 172, 1898 (Schiffner 1898a).

** Riccardia pauciramea (Steph.) H.A.Mill., Ark. Bot. (n.ser.) 5 (2): 527, 1963 (Miller 1963). Bas.: Aneura pauciramea Steph., Bull. Herb. Boissier 5 (10): 845, 1897 (Stephani 1897c).

** Riccardia paulensis Schiffn., Österr. Akad. Wiss., Math.-Naturwiss. Kl., Denkschr. 111: 16, 1964 (Schiffner and Arnell 1964).

** Riccardia pectinata (Austin) H.A.Mill., Ark. Bot. (n.ser.) 5 (2): 526, 1963 (Miller 1963). Bas.: Aneura pectinata Austin, Bull. Torrey Bot. Club 5 (3): 15, 1874 (Austin 1874).

** Riccardia pellucida Piippo, J. Hattori Bot. Lab. 68: 134, 1990 (Piippo 1990). *Nom. nov. pro Aneura pellucida* Steph., Sp. Hepat. (Stephani) 6: 37, 1917 (Stephani 1917a), *nom. illeg*.

** Riccardia pembaiensis (Steph.) Hürl., Bauhinia 5 (4): 205, 1976 (Hürlimann 1976). Bas.: Aneura pembaiensis Steph., Sp. Hepat. (Stephani) 6: 37, 1917 (Stephani 1917a).

** Riccardia pengagensis Hewson, Proc. Linn. Soc. New South Wales (ser. 2) 95 (1): 108, 1970 (Hewson 1970a).

** Riccardia perssonii S.C.Srivast. et Udar, Lindbergia 4 (1/2): 127, 1977 (Srivastava and Udar 1977).

*** Riccardia philippinensis Furuki, J. Hattori Bot. Lab. 100: 94, 2006 (Furuki 2006a).

** Riccardia phleganiana Hewson, Proc. Linn. Soc. New South Wales (ser. 2) 95 (1): 80, 1970 (Hewson 1970a).

** Riccardia plana (Steph.) Hürl., Bauhinia 5 (4): 205, 1976 (Hürlimann 1976). Bas.: Aneura plana Steph., Sp. Hepat. (Stephani) 6: 38, 1917 (Stephani 1917a).

* Riccardia plana var. minor (Pearson) Hürl. ex H.A.Mill., H.Whittier et B.Whittier, Bryophyt. Biblioth. 25: 303, 1983 (Miller et al. 1983). Bas.: Aneura plana var. minor Pearson, J. Linn. Soc., Bot. 46 (305): 16, 1922 (Pearson 1922b).

** Riccardia planifrons (Spruce) Pagán, Bryologist 45 (4): 80, 1942 (Pagán 1942b). Bas.: Aneura planifrons Spruce, J. Linn. Soc., Bot. 30 (210): 368, 1895 (Gepp 1895b).

*** Riccardia plumiformis (Spruce) Hässel ex Meenks, Beih. Nova Hedwigia 88: 101, 1987 (Schultze-Motel and Menzel 1987). Bas.: Aneura plumiformis Spruce, Trans. & Proc. Bot. Soc. Edinburgh 15: 548, 1885 (Spruce 1885).

** Riccardia plumosa (Mitt.) E.O.Campb., J. Roy. Soc. New Zealand 1 (1): 24, 1971 (Campbell 1971). Bas.: Sarcomitrium plumosum Mitt., Bonplandia 10 (2): 19, 1862 (Mitten 1862).

*** Riccardia poeppigiana (Lehm. et Lindenb.) Hässel ex Meenks et C.De Jong, Cryptog. Bryol. Lichénol. 6 (1): 17, 1985 (Meenks and de Jong 1985). Bas.: Jungermannia poeppigiana Lehm. et Lindenb., Nov. Stirp. Pug. 6: 23, 1834 (Lehmann 1834).

** Riccardia porcina (Hewson) L.Söderstr., Phytotaxa 202 (1): 70, 2015 (Söderström et al. 2015c). Bas.: Riccardia bliklika var. porcina Hewson, Proc. Linn. Soc. New South Wales (ser. 2) 95 (1): 84, 1970 (Hewson 1970a).

** Riccardia portoricensis (Steph.) Pagán, Bryologist 42 (1): 7, 1939 (Pagán 1939a). Bas.: Aneura portoricensis Steph., Bull. Herb. Boissier 7 (10): 739 (249), 1899 (Stephani 1899f).

** Riccardia punahuina (Steph.) H.A.Mill., Ark. Bot. (n.ser.) 5 (2): 526, 1963 (Miller 1963). Bas.: Aneura punahuina Steph., Sp. Hepat. (Stephani) 6: 39, 1917 (Stephani 1917a).

** Riccardia ramosissima (Steph.) Grolle, Bryophyt. Biblioth. 48: 130, 1995 (Grolle 1995). Bas.: Aneura ramosissima Steph., Bull. Soc. Roy. Bot. Belgique, Compt. Rend. 30 (2): 196, 1891 [1892] (Stephani 1891b).

** Riccardia regina Meenks et C.De Jong, Cryptog. Bryol. Lichénol. 6 (1): 17, 1985 (Meenks and de Jong 1985).

*** Riccardia regnellii (Ångstr.) K.G.Hell, Bol. Fac. Filos. Univ. São Paulo, Bot. 25: 110, 1969 (Hell 1969). Bas.: Pseudoneura regnellii Ångstr., Öfvers. Kongl. Vetensk.-Akad. Förh. 33 (7): 90, 1876 [1877] (Ångström 1876).

** Riccardia reyesiana Meenks, Acta Bot. Hung. 32 (1/4): 207, 1986 (Meenks 1986).

** Riccardia riccioides Pearson, Univ. Calif. Publ. Bot. 10 (4): 310, 1923 (Pearson 1923).

* Riccardia rigida Schiffn., Denkschr. Kaiserl. Akad. Wiss., Math.-Naturwiss. Kl. 67: 172, 1898 (Schiffner 1898a).

** Riccardia robbinsii Hewson et Grolle, J. Hattori Bot. Lab. 29: 72, 1966 (Grolle 1966i).

** Riccardia robusta (Steph.) H.A.Mill., Phytologia 47 (4): 324, 1981 (Miller 1981). Bas.: Aneura robusta Steph., Sp. Hepat. (Stephani) 6: 40, 1917 (Stephani 1917a).

** Riccardia rockii (Steph.) H.A.Mill., Ark. Bot. (n.ser.) 5 (2): 527, 1963 (Miller 1963). Bas.: Aneura rockii Steph., Sp. Hepat. (Stephani) 6: 41, 1917 (Stephani 1917a).

** Riccardia rupicola (Steph.) Hewson, Proc. Linn. Soc. New South Wales (ser. 2) 95 (1): 99, 1970 (Hewson 1970a). Bas.: Aneura rupicola Steph., Sp. Hepat. (Stephani) 6: 41, 1917 (Stephani 1917a).

** Riccardia russellii R.M.Schust., J. Hattori Bot. Lab. 67: 93, 1989 (Schuster 1989).

** Riccardia saccatiflora (Steph.) S.W.Arnell, Bot. Not. 105: 144, 1952 (Arnell 1952b). Bas.: Aneura saccatiflora Steph., Bot. Gaz. 15 (11): 282, 1890 (Stephani 1890c).

** Riccardia santapaui Udar et S.C.Srivast., Rev. Bryol. Lichénol. 39 (1): 155, 1973 (Udar and Srivastava 1973).

*** Riccardia schwaneckei (Steph.) Pagán, Bryologist 42 (1): 7, 1939 (Pagán 1939a). Bas.: Aneura schwaneckei Steph., Hedwigia 27 (11/12): 278, 1888 (Stephani 1888c).

** Riccardia singapurensis Schiffn., Denkschr. Kaiserl. Akad. Wiss., Math.-Naturwiss. Kl. 67: 165, 1898 (Schiffner 1898a).

** Riccardia smaragdina Meenks et C.De Jong, Cryptog. Bryol. Lichénol. 6 (1): 20, 1985 (Meenks and de Jong 1985).

*** Riccardia sprucei (Steph.) Meenks et C.De Jong, Cryptog. Bryol. Lichénol. 6 (1): 22, 1985 (Meenks and de Jong 1985). Bas.: Aneura sprucei Steph., Bull. Herb. Boissier 5 (10): 844, 1897 (Stephani 1897c).

** Riccardia squamifera Schiffn., Österr. Akad. Wiss., Math.-Naturwiss. Kl., Denkschr. 111: 19, 1964 (Schiffner and Arnell 1964).

* Riccardia stipatiflora (Steph.) Pagán, Bryologist 45 (4): 81, 1942 (Pagán 1942b). Bas.: Aneura stipatiflora Steph., Hedwigia 32 (1): 27, 1893 (Stephani 1893a).

** Riccardia stricta R.M.Schust., J. Hattori Bot. Lab. 62: 326, 1987 (Schuster 1987d).

** Riccardia subantarctica Grolle et L.Söderstr., Phytotaxa 202 (1): 70, 2015 (Söderström et al. 2015c). *Nom. nov. pro Riccardia pauciramea* R.M.Schust., J. Hattori Bot. Lab. 67: 102, 1989 (Schuster 1989), *nom. illeg*.

** Riccardia subexalata Schiffn., Denkschr. Kaiserl. Akad. Wiss., Math.-Naturwiss. Kl. 67: 163, 1898 (Schiffner 1898a).

* Riccardia subexalata var. procera Schiffn., Denkschr. Kaiserl. Akad. Wiss., Math.-Naturwiss. Kl. 67: 164, 1898 (Schiffner 1898a).

* Riccardia submersa (Hook.f. et Taylor) Trevis., Mem. Reale Ist. Lombardo Sci. (Ser. 3), C. Sci. Mat. 4 (13): 431, 1877 (Trevisan 1877). Bas.: Jungermannia multifida var. submersa Hook.f. et Taylor, London J. Bot. 4: 94, 1845 (Hooker and Taylor 1845).

** Riccardia submultifida Horik., J. Sci. Hiroshima Univ., Ser. B, Div. 2, Bot. 2: 128, 1934 (Horikawa 1934).

** Riccardia subpalmata (Steph.) Hürl., Bauhinia 5 (4): 194, 1976 (Hürlimann 1976). Bas.: Aneura subpalmata Steph., Sp. Hepat. (Stephani) 6: 43, 1917 (Stephani 1917a).

** Riccardia subsimplex (Steph.) Pagán, Bryologist 45 (4): 81, 1942 (Pagán 1942b). Bas.: Aneura subsimplex Steph., Hedwigia 32 (1): 27, 1893 (Stephani 1893a).

** Riccardia sumatrana Schiffn., Denkschr. Kaiserl. Akad. Wiss., Math.-Naturwiss. Kl. 67: 173, 1898 (Schiffner 1898a).

** Riccardia tahitensis (Steph.) Hürl., Bauhinia 5 (4): 201, 1976 (Hürlimann 1976). Bas.: Aneura tahitensis Steph., Bull. Herb. Boissier 7 (10): 728 (238), 1899 (Stephani 1899f).

* Riccardia tenella Hewson, Proc. Linn. Soc. New South Wales (ser. 2) 95 (1): 95, 1970 (Hewson 1970a).

*** Riccardia tenuicula (Spruce) Meenks, Beih. Nova Hedwigia 88: 101, 1987 (Schultze-Motel and Menzel 1987). Bas.: Aneura tenuicula Spruce, Trans. & Proc. Bot. Soc. Edinburgh 15: 545, 1885 (Spruce 1885).

** Riccardia tenuis (Steph.) Schiffn., Denkschr. Kaiserl. Akad. Wiss., Math.-Naturwiss. Kl. 67: 172, 1898 (Schiffner 1898a). Bas.: Aneura tenuis Steph., Hedwigia 32 (1): 28, 1893 (Stephani 1893a).

* Riccardia tjibodensis Schiffn., Denkschr. Kaiserl. Akad. Wiss., Math.-Naturwiss. Kl. 67: 165, 1898 (Schiffner 1898a).

*** Riccardia trichomanoides (Spruce) Hässel ex Meenks, Beih. Nova Hedwigia 88: 101, 1987 (Schultze-Motel and Menzel 1987). Bas.: Aneura trichomanoides Spruce, Trans. & Proc. Bot. Soc. Edinburgh 15: 547, 1885 (Spruce 1885).

** Riccardia trukensis H.A.Mill. et Bonner, Beih. Nova Hedwigia 11: 70, 1963 (Miller et al. 1963).

** Riccardia tumbareriensis Hewson, Proc. Linn. Soc. New South Wales (ser. 2) 95 (1): 87, 1970 (Hewson 1970a).

** Riccardia upoluna (Steph.) Grolle, J. Hattori Bot. Lab. 36: 77, 1972 [1973] (Grolle and Schultze-Motel 1972). Bas.: Aneura upoluna Steph., Denkschr. Kaiserl. Akad. Wiss., Math.-Naturwiss. Kl. 91: 165, 1915 (Stephani 1915a).

** Riccardia valida (Steph.) J.J.Engel, Bryologist 78 (3): 362, 1975 (Engel 1975). Bas.: Aneura valida Steph., Sp. Hepat. (Stephani) 6: 44, 1917 (Stephani 1917a).

** Riccardia venosa (Steph.) Hürl., Bauhinia 5 (4): 208, 1976 (Hürlimann 1976). Bas.: Aneura venosa Steph., Sp. Hepat. (Stephani) 6: 45, 1917 (Stephani 1917a).

* Riccardia villosa (Steph.) Pandé et S.C.Srivast., Biol. Mem. 1 (1/2): 129, 1976 (Srivastava and Udar 1976). Bas.: Aneura villosa Steph., Sp. Hepat. (Stephani) 6: 45, 1917 (Stephani 1917a).

** Riccardia virens (Steph.) Hürl., Bauhinia 5 (4): 209, 1976 (Hürlimann 1976). Bas.: Aneura virens Steph., Sp. Hepat. (Stephani) 6: 45, 1917 (Stephani 1917a).

** Riccardia virgata (Gottsche) Pagán, Bryologist 42 (1): 7, 1939 (Pagán 1939a). Bas.: Aneura virgata Gottsche, Hedwigia 27 (11/12): 277, 1888 (Stephani 1888c).

** Riccardia wallisii (Steph.) Gradst., J. Hattori Bot. Lab. 45: 130, 1979 (Gradstein and Hekking 1979). Bas.: Aneura wallisii Steph., Hedwigia 32 (1): 28, 1893 (Stephani 1893a).

** Riccardia wettsteinii Schiffn., Denkschr. Kaiserl. Akad. Wiss., Math.-Naturwiss. Kl. 67: 162, 1898 (Schiffner 1898a).

* Riccardia wettsteinii var. angustilimbia Schiffn., Denkschr. Kaiserl. Akad. Wiss., Math.-Naturwiss. Kl. 67: 163, 1898 (Schiffner 1898a).

* Riccardia wettsteinii var. crassa Schiffn., Denkschr. Kaiserl. Akad. Wiss., Math.-Naturwiss. Kl. 67: 163, 1898 (Schiffner 1898a).

* Riccardia wettsteinii var. procera Schiffn., Denkschr. Kaiserl. Akad. Wiss., Math.-Naturwiss. Kl. 67: 162, 1898 (Schiffner 1898a).

* Riccardia wettsteinii var. tenuiretis Schiffn., Denkschr. Kaiserl. Akad. Wiss., Math.-Naturwiss. Kl. 67: 162, 1898 (Schiffner 1898a).

** Riccardia womersleyana Hewson, Proc. Linn. Soc. New South Wales (ser. 2) 95 (1): 98, 1970 (Hewson 1970a).

** **Verdoornia R.M.Schust.**, J. Hattori Bot. Lab. 26: 291, 1963 (Schuster 1963b).

*** Verdoornia succulenta R.M.Schust., J. Hattori Bot. Lab. 26: 291, 1963 (Schuster 1963b).

####### *** Metzgeriaceae H.Klinggr.

by D. P. Costa

The treatment of Metzgeriaceae follows what was outlined in Hewson (1982), Kuwahara (1986), Crandall-Stotler and Stotler (2000), and Costa (2008).

*** **Metzgeria Raddi**, Jungermanniogr. Etrusca: 34, 1818 (Raddi 1818a). ^407^

*** Metzgeria acuminata Steph., Bull. Herb. Boissier 7 (12): 934 (282), 1899 (Stephani 1899g).

*** Metzgeria adscendens Steph. ex K.I.Goebel, Flora 77: 427, 1893 (Goebel 1893a).

*** Metzgeria agnewiae Kuwah., Bryologist 76 (4): 569, 1973 (Kuwahara 1973b). ^408^

*** Metzgeria albinea Spruce, Bull. Soc. Bot. France (Congr. Bot.) 36: cci, 1889 [1890] (Spruce 1889).

*** Metzgeria albinea var. aberrans Schiffn., Österr. Akad. Wiss., Math.-Naturwiss. Kl., Denkschr. 111: 27, 1964 (Schiffner and Arnell 1964).

*** Metzgeria albinea var. angusta (Steph.) D.P.Costa et Gradst., Bryologist 103 (4): 757, 2000 [2001] (Costa and Gradstein 2000). Bas.: Metzgeria angusta Steph., Bull. Herb. Boissier 7 (12): 944 (292), 1899 (Stephani 1899g).

*** Metzgeria allionii Steph., Sp. Hepat. (Stephani) 6: 47, 1917 (Stephani 1917a).

*** Metzgeria alpina R.M.Schust. et J.J.Engel, Brittonia 40 (2): 203, 1988 (Engel and Schuster 1988).

*** Metzgeria americana Masuzaki, Hikobia 15 (4): 441, 2010 (Masuzaki et al. 2010a).

*** Metzgeria attenuata Steph., Biblioth. Bot. 87 (2): 177, 1916 (Stephani 1916a).

*** Metzgeria aurantiaca Steph., Bull. Herb. Boissier 7 (12): 938 (286), 1899 (Stephani 1899g).

*** Metzgeria auriculata Grolle et Kuwah., Bryophyt. Biblioth. 28: 194, 1986 (Kuwahara 1986).

*** Metzgeria bahiensis Schiffn., Österr. Bot. Z. 61 (7/8): 262, 1911 (Schiffner 1911).

*** Metzgeria bartlettii Kuwah., Mem. New York Bot. Gard. 45: 561, 1987 (Kuwahara 1987).

*** Metzgeria bischlerae Kuwah., J. Hattori Bot. Lab. 40: 264, 1976 (Kuwahara 1976a).

*** Metzgeria bracteata Spruce, Trans. & Proc. Bot. Soc. Edinburgh 15: 553, 1885 (Spruce 1885).

*** Metzgeria brasiliensis Schiffn., Österr. Akad. Wiss., Math.-Naturwiss. Kl., Denkschr. 111: 22, 1964 (Schiffner and Arnell 1964).

*** Metzgeria chilensis Steph., Bull. Herb. Boissier 7 (12): 937 (285), 1899 (Stephani 1899g).

*** Metzgeria ciliata Raddi, Critt. Brasil.: 17, 1822 (Raddi 1822). ^409^

*** Metzgeria claviflora Spruce, Trans. & Proc. Bot. Soc. Edinburgh 15: 556, 1885 (Spruce 1885).

*** Metzgeria cleefii Kuwah., Proc. Kon. Ned. Akad. Wetensch. C 85 (3): 360, 1982 (Kuwahara 1982).

** Metzgeria comata Steph., Bull. Herb. Boissier 7 (12): 939 (287), 1899 (Stephani 1899g).

*** Metzgeria conjugata Lindb., Acta Soc. Sci. Fenn. 10: 495, 1875 (Lindberg 1875). ^410^

*** Metzgeria consanguinea Schiffn., Nova Acta Acad. Caes. Leop.-Carol. German. Nat. Cur. 60 (2): 271, 1893 (Schiffner 1893a).

*** Metzgeria convoluta Steph., Bull. Herb. Boissier 7 (12): 940 (288), 1899 (Stephani 1899g).

** Metzgeria coorgensis S.C.Srivast. et S.Srivast., Phytotaxonomy 4: 81, 2004 (Srivastava and Srivastava 2004).

** Metzgeria corralensis Steph., Bull. Herb. Boissier 7 (12): 933 (281), 1899 (Stephani 1899g).

*** Metzgeria crassipilis (Lindb.) A.Evans, Rhodora 11 (130): 188, 1909 (Evans 1909). Bas.: Metzgeria furcata subsp. crassipilis Lindb., Acta Soc. Fauna Fl. Fenn. 1 (2): 42, 1877 (Lindberg 1877b).

*** Metzgeria cratoneura Schiffn., Österr. Akad. Wiss., Math.-Naturwiss. Kl., Denkschr. 111: 24, 1964 (Schiffner and Arnell 1964).

*** Metzgeria cylindra Kuwah., Bryologist 81 (3): 405, 1978 (Kuwahara 1978).

** Metzgeria decrescens Steph., Bull. Herb. Boissier 7 (12): 932 (280), 1899 (Stephani 1899g).

*** Metzgeria dichotoma (Sw.) Nees, Syn. Hepat. 4: 504, 1846 (Gottsche et al. 1846). Bas.: Jungermannia dichotoma Sw., Prodr. (Swartz): 145, 1788 (Swartz 1788).

** Metzgeria divaricata A.Evans, Proc. Amer. Acad. Arts 58 (7): 288, 1923 (Evans 1923a).

*** Metzgeria dorsipara (Herzog) Kuwah., J. Hattori Bot. Lab. 40: 269, 1976 (Kuwahara 1976a). Bas.: Metzgeria violacea var. dorsipara Herzog, Svensk Bot. Tidskr. 51 (1): 187, 1957 (Herzog 1957a).

*** Metzgeria duricosta Steph., Sp. Hepat. (Stephani) 6: 50, 1917 (Stephani 1917a).

** Metzgeria engelii Kuwah., Hikobia 8 (3/4): 275, 1980 (Kuwahara 1980a).

** Metzgeria epiphylla A.Evans, Proc. Amer. Acad. Arts 58 (7): 303, 1923 (Evans 1923a).

*** Metzgeria filicina Mitt., Hooker’s J. Bot. Kew Gard. Misc. 3: 361, 1851 (Mitten 1851).

** Metzgeria flavovirens Colenso, Trans. & Proc. New Zealand Inst. 21: 79, 1889 (Colenso 1889).

* Metzgeria foliicola Schiffn., Denkschr. Kaiserl. Akad. Wiss., Math.-Naturwiss. Kl. 67: 181, 1898 (Schiffner 1898a). ^411^

*** Metzgeria francana Steph., Sp. Hepat. (Stephani) 6: 51, 1917 (Stephani 1917a).

** Metzgeria frontipilis Lindb., Acta Soc. Fauna Fl. Fenn. 1 (2): 14, 1877 (Lindberg 1877b).

*** Metzgeria fruticola Spruce, Trans. & Proc. Bot. Soc. Edinburgh 15: 554, 1885 (Spruce 1885).

*** Metzgeria furcata (L.) Corda, Gen. hepat.: 654, 1829 (Corda 1829). Bas.: Jungermannia furcata L., Sp. Pl. 1: 1136, 1753 (Linnaeus 1753). ^412^

* Metzgeria furcata var. expansa Douin, Rev. Bryol. 30 (3): 47, 1903 (Douin 1903).

* Metzgeria furcata var. pacifica Brinkm., Bryologist 34 (2): 15, 1931 (Brinkman 1931).

*** Metzgeria grandiflora A.Evans, Torreya 16 (3): 68, 1916 (Evans 1916).

** Metzgeria hasselii Kuwah., J. Hattori Bot. Lab. 40: 509, 1976 (Kuwahara 1976c).

** Metzgeria hebridensis Steph., Sp. Hepat. (Stephani) 6: 51, 1917 (Stephani 1917a).

*** Metzgeria hegewaldii Kuwah., Nova Hedwigia 34: 784, 1981 (Kuwahara 1981).

*** Metzgeria herminieri Schiffn., Österr. Bot. Z. 61 (7/8): 261, 1911 (Schiffner 1911).

* Metzgeria heteroramea Steph., Biblioth. Bot. 87 (2): 178, 1916 (Stephani 1916a).

** Metzgeria imberbis J.B.Jack et Steph., Hedwigia 34 (6): 316, 1895 (Jack and Stephani 1895).

*** Metzgeria inflata Steph., Bull. Herb. Boissier 7 (12): 936 (284), 1899 (Stephani 1899g).

*** Metzgeria jamesonii Kuwah., Bryophyt. Biblioth. 28: 157, 1986 (Kuwahara 1986).

** Metzgeria kanaii Kuwah., Fl. E. Himalaya 2: 240, 1971 (Hattori 1971a).

*** Metzgeria kinabaluensis Masuzaki, Hikobia 16 (1): 59, 2011 (Masuzaki 2011). Based on: Apometzgeria pubescens var. kinabaluensis Kuwah., J. Hattori Bot. Lab. 28: 166, 1965 (Kuwahara 1965), *nom. inval*.

** Metzgeria kuwaharae Piippo, Acta Bot. Fenn. 143: 8, 1991 (Piippo 1991).

** Metzgeria laciniata Kuwah., Bryologist 81 (3): 406, 1978 (Kuwahara 1978).

*** Metzgeria lechleri Steph., Bull. Herb. Boissier 7 (12): 942 (290), 1899 (Stephani 1899g).

*** Metzgeria leptoneura Spruce, Trans. & Proc. Bot. Soc. Edinburgh 15: 555, 1885 (Spruce 1885).

** Metzgeria leptoneura var. breviseta (Schiffn.) O.Yano, J. Hattori Bot. Lab. 56: 532, 1984 (Yano 1984). Bas.: Metzgeria hamata var. breviseta Schiffn., Österr. Akad. Wiss., Math.-Naturwiss. Kl., Denkschr. 111: 27, 1964 (Schiffner and Arnell 1964).

** Metzgeria leptoneura var. polychaeta R.M.Schust., Phytotaxa 202 (1): 70, 2015 (Söderström et al. 2015c). Based on: Metzgeria leptoneura var. polychaeta R.M.Schust., J. Hattori Bot. Lab. 70: 150, 1991 (Schuster 1991b), *nom. inval*.

*** Metzgeria liebmaniana Lindenb. et Gottsche, Syn. Hepat. 4: 505, 1846 (Gottsche et al. 1846).

*** Metzgeria lindbergii Schiffn., Denkschr. Kaiserl. Akad. Wiss., Math.-Naturwiss. Kl. 67: 182, 1898 (Schiffner 1898a).

** Metzgeria litoralis J.J.Engel et Kuwah., Bryologist 76 (2): 293, 1973 (Engel and Kuwahara 1973).

*** Metzgeria longitexta Steph., Bull. Herb. Boissier 7 (12): 940 (288), 1899 (Stephani 1899g).

** Metzgeria macrospora Kuwah., J. Hattori Bot. Lab. 32: 17, 1969 (Kuwahara 1969a).

** Metzgeria macveanii Kuwah., Rev. Bryol. Lichénol. 36 (3/4): 539, 1969 [1970] (Kuwahara 1969b).

*** Metzgeria maegdefraui Kuwah., Hikobia 8 (3/4): 269, 1980 (Kuwahara 1980b).

** Metzgeria magellanica Schiffn., Leberm., Forschungsr. Gazelle 4 (4): 43, 1890 (Schiffner 1890).

*** Metzgeria metaensis Kuwah., Proc. Kon. Ned. Akad. Wetensch. C 85 (3): 375, 1982 (Kuwahara 1982).

*** Metzgeria mexicana Steph., Sp. Hepat. (Stephani) 6: 55, 1917 (Stephani 1917a). ^413^

** Metzgeria monoica Kuwah. et J.J.Engel, Hikobia 8 (3/4): 284, 1980 (Kuwahara 1980a).

*** Metzgeria myriopoda Lindb., Acta Soc. Fauna Fl. Fenn. 1 (2): 22, 1877 (Lindberg 1877b).

*** Metzgeria neotropica Kuwah., Nova Hedwigia 34: 779, 1981 (Kuwahara 1981).

*** Metzgeria nudifrons Steph., Hedwigia 31 (3): 126, 1892 (Stephani 1892d).

*** Metzgeria parviinvolucrata Kuwah., Nova Hedwigia 34: 774, 1981 (Kuwahara 1981).

** Metzgeria patagonica Steph., Bull. Herb. Boissier 7 (12): 940 (288), 1899 (Stephani 1899g).

*** Metzgeria polytricha Spruce, Trans. & Proc. Bot. Soc. Edinburgh 15: 553, 1885 (Spruce 1885).

*** Metzgeria procera Mitt., Bot. antarct. voy. II (Fl. Nov.-Zel. 2): 166, 1855 (Mitten 1855).

*** Metzgeria psilocraspeda Schiffn., Österr. Akad. Wiss., Math.-Naturwiss. Kl., Denkschr. 111: 25, 1964 (Schiffner and Arnell 1964).

*** Metzgeria pubescens (Schrank) Raddi, Jungermanniogr. Etrusca: 35, 1818 (Raddi 1818a). Bas.: Jungermannia pubescens Schrank, Prim. Fl. Salisb.: 231, 1792 (Schrank 1792).

*** Metzgeria pulvinata Steph., Biblioth. Bot. 87 (2): 179, 1916 (Stephani 1916a).

** Metzgeria quadrifaria Steph., Bull. Herb. Boissier 7 (12): 953 (301), 1899 (Stephani 1899g).

** Metzgeria raoi S.C.Srivast. et S.Srivast., Phytotaxonomy 4: 83, 2004 (Srivastava and Srivastava 2004).

** Metzgeria rigida Lindb., Acta Soc. Fauna Fl. Fenn. 1 (2): 43, 1877 (Lindberg 1877b).

** Metzgeria robinsonii Steph., Sp. Hepat. (Stephani) 6: 60, 1917 (Stephani 1917a).

** Metzgeria roivainenii Kuwah., J. Hattori Bot. Lab. 40: 516, 1976 (Kuwahara 1976c).

*** Metzgeria rufula Spruce, Trans. & Proc. Bot. Soc. Edinburgh 15: 555, 1885 (Spruce 1885).

** Metzgeria saccata Mitt., J. Linn. Soc., Bot. 22 (145): 241, 1886 (Mitten 1886a).

** Metzgeria saxbyi Pearson, Ann. Cryptog. Exot. 4 (2): 70, 1931 (Pearson 1931a).

** Metzgeria scobina Mitt., J. Linn. Soc., Bot. 22 (145): 243, 1886 (Mitten 1886a).

*** Metzgeria scyphigera A.Evans, Trans. Connecticut Acad. Arts 18 (5): 299, 1914 (Evans 1914c).

*** Metzgeria senjoana Masuzaki, Hikobia 15 (4): 445, 2010 (Masuzaki et al. 2010a).

** Metzgeria setigera R.M.Schust. ex Crand.-Stotl. et L.Söderstr., Phytotaxa 202 (1): 69, 2015 (Söderström et al. 2015c). Based on: Metzgeria furcata var. setigera R.M.Schust., J. Hattori Bot. Lab. 70: 149, 1991 (Schuster 1991b), *nom. inval*.

** Metzgeria sikkimensis S.C.Srivast. et K.K.Rawat, Geophytology 31 (1/2): 71, 2001 [2003] (Srivastava and Rawat 2001).

* Metzgeria simplex Lorb. ex Müll.Frib., Hedwigia 80 (1/2): 115, 1941 (Müller 1941).

*** Metzgeria sinuata Loitl., Diagn. pl. nov.: 25, 1894 (Loitlesberger 1894).

** Metzgeria sparrei Kuwah., Hikobia 8 (3/4): 278, 1980 (Kuwahara 1980a).

*** Metzgeria spindleri Steph., Biblioth. Bot. 87 (2): 179, 1916 (Stephani 1916a).

*** Metzgeria subaneura Schiffn., Österr. Akad. Wiss., Math.-Naturwiss. Kl., Denkschr. 111: 22, 1964 (Schiffner and Arnell 1964).

** Metzgeria submarginata M.L.So, New Zealand J. Bot. 40 (2): 201, 2002 (So 2002b).

** Metzgeria subundulata (Lindb.) Kuwah., Bryologist 86 (3): 276, 1983 [1984] (Kuwahara 1983). Bas.: Metzgeria furcata subsp. subundulata Lindb., Acta Soc. Fauna Fl. Fenn. 1 (2): 42, 1877 (Lindberg 1877b).

** Metzgeria temperata Kuwah., J. Hattori Bot. Lab. 40: 219, 1976 (Kuwahara 1976b).

*** Metzgeria uncigera A.Evans, Ann. Bot. (Oxford) 24 (2): 276, 1910 (Evans 1910).

*** Metzgeria undulata Kuwah., Nova Hedwigia 34: 792, 1981 (Kuwahara 1981).

*** Metzgeria violacea (Ach.) Dumort., Recueil Observ. Jungerm.: 26, 1835 (Dumortier 1835). Bas.: Jungermannia violacea Ach., Beitr. Naturk. (Weber & Mohr) 1: 77, 1805 (Acharius 1805).

** **Steereella Kuwah.**, Amer. J. Bot. 60 (6): 602, 1973 (Kuwahara 1973a).

*** Steereella lilliana (Steph.) Kuwah., Bryophyt. Biblioth. 28: 179, 1986 (Kuwahara 1986). Bas.: Metzgeria lilliana Steph., Sp. Hepat. (Stephani) 6: 53, 1917 (Stephani 1917a).

*** Steereella linearis (Sw.) Kuwah., Amer. J. Bot. 60 (6): 604, 1973 (Kuwahara 1973a). Bas.: Jungermannia linearis Sw., Prodr. (Swartz): 145, 1788 (Swartz 1788).

** **Vandiemenia Hewson**, J. Hattori Bot. Lab. 52: 163, 1982 (Hewson 1982).

** Vandiemenia ratkowskiana Hewson, J. Hattori Bot. Lab. 52: 163, 1982 (Hewson 1982).

###### Pleuroziales Schljakov

####### *** Pleuroziaceae Müll.Frib.

by B. Thiers

*** **Pleurozia Dumort.**, Recueil Observ. Jungerm.: 15, 1835 (Dumortier 1835).

** **subg. Constantifolia B.M.Thiers**, Bryologist 96 (4): 526, 1993 (Thiers 1993).

*** Pleurozia conchifolia (Hook. et Arn.) Austin, Bull. Torrey Bot. Club 5 (3): 16, 1874 (Austin 1874). Bas.: Jungermannia conchifolia Hook. et Arn., Bot. Beechey Voy. 3: 110, 1832 (Hooker and Arnott 1832).

** Pleurozia conchifolia var. papillosa B.M.Thiers, Bryologist 96 (4): 528, 1993 (Thiers 1993).

*** Pleurozia purpurea Lindb., Acta Soc. Fauna Fl. Fenn. 1 (2): 27, 1877 (Lindberg 1877b). Based on: Jungermannia purpurea Lightf., Fl. Scot. 2: 778, 1777 (Lightfoot 1777), *nom. illeg*.

** **subg. Diversifolia B.M.Thiers**, Bryologist 96 (4): 531, 1993 (Thiers 1993).

*** Pleurozia acinosa (Mitt.) Trevis., Mem. Reale Ist. Lombardo Sci. (Ser. 3), C. Sci. Mat. 4 (13): 412, 1877 (Trevisan 1877). Bas.: Physiotium acinosum Mitt., J. Proc. Linn. Soc., Bot. 5 (18): 102, 1860 [1861] (Mitten 1860c).

*** Pleurozia articulata (Lindb.) Lindb. et Lackström, Hepat. Scand. Exsicc.: 5 (adnot.), 1874 (Lindberg and Lackström 1874). Bas.: Physiotium articulatum Lindb., Öfvers. Förh. Finska Vetensk.-Soc. 12 (2): 78, 1870 (Lindberg 1870).

*** Pleurozia caledonica (Gottsche) Steph., Rev. Bryol. 33 (2): 29 (Paris 1906a). Bas.: Physiotium caledonicum Gottsche, Hedwigia 25 (2/3): 81, 1886 (Jack 1886).

*** Pleurozia curiosa B.M.Thiers, Bryologist 96 (4): 537, 1993 (Thiers 1993).

*** Pleurozia heterophylla Steph. ex Fulford, Mem. New York Bot. Gard. 23: 842, 1972 (Fulford 1972).

*** Pleurozia johannis-winkleri Herzog, Mitt. Inst. Allg. Bot. Hamburg 7 (3): 195, 1931 (Herzog 1931a).

*** Pleurozia paradoxa (J.B.Jack) Schiffn., Hepat. (Engl.-Prantl): 115, 1893 (Schiffner 1893b). Bas.: Physiotium paradoxum J.B.Jack, Hedwigia 25 (2/3): 85, 1886 (Jack 1886).

*** Pleurozia subinflata (Austin) Austin, Bull. Torrey Bot. Club 5 (3): 16, 1874 (Austin 1874). Bas.: Physiotium subinflatum Austin, Proc. Acad. Nat. Sci. Philadelphia 21: 224, 1869 (Austin 1869).

** **subg. Pleurozia**

*** Pleurozia gigantea (F.Weber) Lindb., Hepat. Scand. Exsicc.: no. 5, 1874 (Lindberg and Lackström 1874). Bas.: Jungermannia gigantea F.Weber, Hist. Musc. Hepat. Prodr.: 57, 1815 (Weber 1815).

***Incertae sedis***

** Pleurozia pocsii Frank Müll., Polish Bot. J. 58 (1): 50, 2013 (Müller 2013).

##### Pelliidae He-Nygrén, Juslén, Ahonen, Glenny et Piippo

###### Fossombroniales Schljakov

####### Calyculariineae He-Nygrén, Juslén, Ahonen, Glenny et Piippo

######## *** Calyculariaceae He-Nygrén, Juslén, Ahonen, Glenny et Piippo

*** **Calycularia Mitt.**, J. Proc. Linn. Soc., Bot. 5 (18): 122, 1860 [1861] (Mitten 1860c).

*** Calycularia crispula Mitt., J. Proc. Linn. Soc., Bot. 5 (18): 122, 1860 [1861] (Mitten 1860c).

*** Calycularia laxa Lindb. et Arnell, Kongl. Svenska Vetensk.-Akad. Handl. (n.ser.) 23 (5): 66, 1889 (Lindberg and Arnell 1889).

####### Fossombroniineae R.M.Schust. ex Stotler et Crand.-Stotl.

######## ** Allisoniaceae Schljakov

*** **Allisonia Herzog**, Hedwigia 80 (1/2): 77, 1941 (Herzog 1941a).

*** Allisonia cockaynei (K.I.Goebel) R.M.Schust., J. Hattori Bot. Lab. 26: 294, 1963 (Schuster 1963b). Bas.: Moerckia cockaynei K.I.Goebel, Flora 96: 190, 1906 (Goebel 1906).

######## *** Fossombroniaceae Hazsl. *nom. conserv.*

by R. Stotler, B. J. Crandall-Stotler and D. C. Cargill

*** **Fossombronia Raddi**, Jungermanniogr. Etrusca: 29, 1818 (Raddi 1818a).

*** Fossombronia alaskana Steere et Inoue, Bryologist 77 (1): 66, 1974 (Steere and Inoue 1974).

*** Fossombronia alata G.A.M.Scott et D.C.Pike, J. Hattori Bot. Lab. 56: 340, 1984 (Scott and Pike 1984).

*** Fossombronia altilamellosa G.A.M.Scott et D.C.Pike, Fl. Austral. Suppl. 21: 114, 2003 (McCarthy 2003). Based on: Fossombronia altilamellosa G.A.M.Scott et D.C.Pike, J. Hattori Bot. Lab. 62: 367, 1987 (Scott and Pike 1987), *nom. inval*.

*** Fossombronia angulifolia Perold, Bothalia 28 (2): 159, 1998 (Perold 1998b).

*** Fossombronia angulosa (Dicks.) Raddi, Jungermanniogr. Etrusca: 29, 1818 (Raddi 1818a). Bas.: Jungermannia angulosa Dicks., Fasc. Pl. Crypt. Brit. 1: 7, 1785 (Dickson 1785).

** Fossombronia areolata G.A.M.Scott et D.C.Pike, J. Hattori Bot. Lab. 62: 368, 1987 (Scott and Pike 1987).

** Fossombronia auricolor G.A.M.Scott et D.C.Pike, Fl. Austral. Suppl. 21: 114, 2003 (McCarthy 2003). Based on: Fossombronia auricolor G.A.M.Scott et D.C.Pike, J. Hattori Bot. Lab. 62: 370, 1987 (Scott and Pike 1987), *nom. inval*.

*** Fossombronia australis Mitt., J. Linn. Soc., Bot. 15 (82): 73, 1876 (Mitten 1876a).

*** Fossombronia caespitiformis (Raddi) De Not. ex Rabenh., Hepat. Eur., Leberm. 13-14: no. 123, 1860 [1861] (Rabenhorst 1860). Bas.: Fossombronia angulosa var. caespitiformis Raddi, Jungermanniogr. Etrusca: 30, 1818 (Raddi 1818a).

*** Fossombronia caespitiformis subsp. multispira (Schiffn.) J.R.Bray et Cargill, Bryologist 106 (1): 131, 2003 (Stotler et al. 2003). Bas.: Fossombronia caespitiformis var. multispira Schiffn., Österr. Bot. Z. 67 (4/5): 152, 1918 (Schiffner 1918).

*** Fossombronia caledonica Steph., Sp. Hepat. (Stephani) 6: 71, 1917 (Stephani 1917a).

*** Fossombronia cederbergensis Perold, Bothalia 28 (1): 1, 1998 (Perold 1998c).

*** Fossombronia cerebriformis G.A.M.Scott et D.C.Pike, J. Hattori Bot. Lab. 56: 343, 1984 (Scott and Pike 1984).

*** Fossombronia crassifolia Spruce, Trans. & Proc. Bot. Soc. Edinburgh 15: 527, 1885 (Spruce 1885).

*** Fossombronia crispa Nees, Syn. Hepat. 4: 469, 1846 (Gottsche et al. 1846).

* Fossombronia crispula (Brot.) R.M.Schust., Hepat. Anthocerotae N. Amer. 5: 383, 1992 (Schuster 1992b). Bas.: Jungermannia crispula Brot., Fl. lusit. 2: 422, 1804 [1805] (Brotero 1804). ^414^

*** Fossombronia cristula Austin, Proc. Acad. Nat. Sci. Philadelphia 21: 228, 1869 (Austin 1869).

*** Fossombronia cultriformis G.A.M.Scott et D.C.Pike, J. Hattori Bot. Lab. 62: 371, 1987 (Scott and Pike 1987).

*** Fossombronia densa G.A.M.Scott et D.C.Pike, J. Hattori Bot. Lab. 62: 372, 1987 (Scott and Pike 1987).

*** Fossombronia densilamellata S.W.Arnell, Bot. Not. 105: 317, 1952 (Arnell 1952a).

*** Fossombronia echinata Macvicar, Rev. Bryol. 38 (4): 73, 1911 (Macvicar 1911).

*** Fossombronia elsieae Perold, Bothalia 29 (1): 25, 1999 (Perold 1999c).

*** Fossombronia fernandeziensis Steph., Kungl. Svenska Vetensk.-Akad. Handl. (n.ser.) 46 (9): 15, 1911 (Stephani 1911b).

*** Fossombronia fimbriata Paton, J. Bryol. 8 (1): 1, 1974 (Paton 1974).

*** Fossombronia fleischeri Osterwald ex Loeske, Verh. Bot. Vereins Prov. Brandenburg 70: 125, 1928 (Loeske 1928).

*** Fossombronia foveolata Lindb., Helsingf. Dagbl. 1873 (353, 28 Dec): 2, 1873 (Lindberg 1873b).

** Fossombronia fuhreri G.A.M.Scott et D.C.Pike, J. Hattori Bot. Lab. 62: 374, 1987 (Scott and Pike 1987).

*** Fossombronia gemmifera Perold, Bothalia 27 (1): 19, 1997 (Perold 1997a).

*** Fossombronia glenii Perold, Bothalia 27 (1): 20, 1997 (Perold 1997a).

** Fossombronia grandis Steph., Mém. Herb. Boissier 16: 29 (383), 1900 (Stephani 1900a).

* Fossombronia gregaria Colenso, Trans. & Proc. New Zealand Inst. 20: 252, 1888 (Colenso 1888). ^415^

* Fossombronia grossepapillata Steph., J. & Proc. Roy. Soc. New South Wales 48 (1/2): 105, 1914 (Stephani and Watts 1914). ^416^

*** Fossombronia hamatohirta Steph., Hedwigia 33 (1): 8, 1894 (Stephani 1894a).

** Fossombronia hewsoniae G.A.M.Scott et D.C.Pike, J. Hattori Bot. Lab. 62: 375, 1987 (Scott and Pike 1987).

*** Fossombronia himalayensis Kashyap, New Phytol. 14 (1): 4, 1915 (Kashyap 1915).

*** Fossombronia hyalorhiza Perold, Bothalia 29 (1): 83, 1999 (Perold 1999d).

*** Fossombronia incurva Lindb., Helsingf. Dagbl. 1873 (273, 7 Oct): 2, 1873 (Lindberg 1873c).

*** Fossombronia indica Steph., Sp. Hepat. (Stephani) 6: 73, 1917 (Stephani 1917a).

* Fossombronia integerrima Steph., Mém. Herb. Boissier 16: 40 (394), 1900 (Stephani 1900a). ^417^

* Fossombronia integrifolia Steph., Sp. Hepat. (Stephani) 6: 73, 1917 (Stephani 1917a). ^418^

*** Fossombronia intestinalis Taylor, London J. Bot. 5: 408, 1846 (Taylor 1846b).

*** Fossombronia japonica Schiffn., Österr. Bot. Z. 49 (11): 389, 1899 (Schiffner 1899c).

** Fossombronia laciniata G.A.M.Scott et D.C.Pike, J. Hattori Bot. Lab. 62: 375, 1987 (Scott and Pike 1987).

*** Fossombronia lamellata Steph., Hedwigia 33 (1): 9, 1894 (Stephani 1894a).

*** Fossombronia leucoxantha (Lehm.) Lehm. et Lindenb., Syn. Hepat. 4: 469, 1846 (Gottsche et al. 1846). Bas.: Jungermannia leucoxantha Lehm., Linnaea 4: 368, 1829 (Lehmann 1829).

*** Fossombronia longiseta (Austin) Austin, Hepat. bor.-amer.: 30, 1873 (Austin 1873). Bas.: Androcryphia longiseta Austin, Proc. Acad. Nat. Sci. Philadelphia 21: 228, 1869 (Austin 1869).

*** Fossombronia lophoclada Spruce, Trans. & Proc. Bot. Soc. Edinburgh 15: 529, 1885 (Spruce 1885).

** Fossombronia lophoscypha Hässel, Beih. Nova Hedwigia 131: 16, 2007 (Hässel and Villagrán 2007).

*** Fossombronia luetzelburgiana K.I.Goebel, Flora 105: 55, 1912 (Goebel 1912).

*** Fossombronia macrocalyx Steph., Sp. Hepat. (Stephani) 6: 74, 1917 (Stephani 1917a).

* Fossombronia macrophylla Colenso, Trans. & Proc. New Zealand Inst. 18: 285, 1886 (Colenso 1886a).

** Fossombronia magnaspora G.A.M.Scott et D.C.Pike, Fl. Austral. Suppl. 21: 114, 2003 (McCarthy 2003). Based on: Fossombronia magnaspora G.A.M.Scott et D.C.Pike, J. Hattori Bot. Lab. 62: 377, 1987 (Scott and Pike 1987), *nom. inval*.

*** Fossombronia marindae Perold, Bothalia 29 (1): 86, 1999 (Perold 1999d).

** Fossombronia maritima (Paton) Paton, J. Bryol. 18 (2): 367, 1994 (Paton 1994). Bas.: Fossombronia pusilla var. maritima Paton, J. Bryol. 7 (3): 244, 1973 (Paton 1973).

*** Fossombronia marshii J.R.Bray et Stotler, Phytologia 92 (2): 230, 2010 (Stotler et al. 2010).

** Fossombronia microlamellata G.A.M.Scott et D.C.Pike, J. Hattori Bot. Lab. 56: 347, 1984 (Scott and Pike 1984).

*** Fossombronia mittenii Tind., J. Bot. 36: 44, 1898 (Tindall 1898).

*** Fossombronia montaguensis S.W.Arnell, Bot. Not. 105: 316, 1952 (Arnell 1952a).

*** Fossombronia monticola Perold, Bothalia 29 (1): 87, 1999 (Perold 1999d).

*** Fossombronia mylioides Inoue, J. Hattori Bot. Lab. 37: 296, 1973 (Inoue 1973).

* Fossombronia nigricaulis Colenso, Trans. & Proc. New Zealand Inst. 18: 248, 1886 (Colenso 1886b). ^419^

** Fossombronia nyikaensis Perold, Bothalia 31 (1): 48, 2001 (Perold 2001c).

*** Fossombronia papillata Steph., Hedwigia 28 (3): 157, 1889 (Stephani 1889d).

*** Fossombronia paranapanemae Schiffn., Österr. Akad. Wiss., Math.-Naturwiss. Kl., Denkschr. 111: 34, 1964 (Schiffner and Arnell 1964).

*** Fossombronia peruviana Gottsche et Hampe, Linnaea 27 (5): 555, 1854 (Hampe 1854).

*** Fossombronia porphyrorhiza (Nees) Prosk., Bryologist 58 (3): 197, 1955 (Proskauer 1955). Bas.: Jungermannia porphyrorhiza Nees, Fl. Bras. (Martius) 1 (1): 343, 1833 (Nees 1833a).

** Fossombronia pulvinata Steph., Wiss. Ergebn. Deut. Zentr.-Afr. Exped. (1907-08), Bot. 2: 113, 1911 (Stephani 1911a).

*** Fossombronia punctata G.A.M.Scott et D.C.Pike, J. Hattori Bot. Lab. 56: 341, 1984 (Scott and Pike 1984).

*** Fossombronia purpureospora G.A.M.Scott et D.C.Pike, Fl. Austral. Suppl. 21: 114, 2003 (McCarthy 2003). Based on: Fossombronia purpureospora G.A.M.Scott et D.C.Pike, J. Hattori Bot. Lab. 62: 379, 1987 (Scott and Pike 1987), *nom. inval*.

*** Fossombronia pusilla (L.) Nees, Naturgesch. Eur. Leberm. 3: 319, 1838 (Nees 1838b). Bas.: Jungermannia pusilla L., Sp. Pl. 1: 1136, 1753 (Linnaeus 1753).

*** Fossombronia renateae Perold, Bothalia 29 (1): 77, 1999 (Perold 1999b).

*** Fossombronia reticulata Steph., Hedwigia 33 (1): 9, 1894 (Stephani 1894a).

* Fossombronia rosulata Colenso, Trans. & Proc. New Zealand Inst. 18: 248, 1886 (Colenso 1886b).

** Fossombronia rudis G.A.M.Scott et D.C.Pike, Beih. Nova Hedwigia 90: 110, 1988 (Scott and Pike 1988a).

*** Fossombronia ruminata Cargill, Phytotaxa 65: 45, 2012 (Cargill et al. 2012). *Nom. nov. pro Fossombronia maritima* G.A.M.Scott et D.C.Pike, Fl. Austral. Suppl. 21: 114, 2003 (McCarthy 2003), *nom. illeg*.

** Fossombronia rupestris G.A.M.Scott et D.C.Pike, Fl. Austral. Suppl. 21: 114, 2003 (McCarthy 2003). Based on: Fossombronia rupestris G.A.M.Scott et D.C.Pike, J. Hattori Bot. Lab. 62: 381, 1987 (Scott and Pike 1987), *nom. inval*.

*** Fossombronia rwandaensis Perold, Bothalia 28 (1): 45, 1998 (Perold 1998a).

*** Fossombronia scrobiculata G.A.M.Scott et D.C.Pike, J. Hattori Bot. Lab. 56: 341, 1984 (Scott and Pike 1984).

*** Fossombronia spinifolia Steph., Mém. Herb. Boissier 16: 35 (389), 1900 (Stephani 1900a).

* Fossombronia spinosa Perold, Bothalia 29 (1): 29, 1999 (Perold 1999c).

*** Fossombronia stephanii Schiffn. ex Steph., Mém. Herb. Boissier 16: 27 (381), 1900 (Stephani 1900a).

*** Fossombronia straussiana Perold, Bothalia 27 (1): 24, 1997 (Perold 1997a).

* Fossombronia subsaccata Steph., Sp. Hepat. (Stephani) 6: 75, 1917 (Stephani 1917a). ^420^

*** Fossombronia swaziensis Perold, Bothalia 28 (2): 162, 1998 (Perold 1998b).

** Fossombronia tesselata G.A.M.Scott et D.C.Pike, J. Hattori Bot. Lab. 62: 382, 1987 (Scott and Pike 1987).

*** Fossombronia texana Lindb., Acta Soc. Sci. Fenn. 10: 533, 1875 (Lindberg 1875).

*** Fossombronia truncata G.A.M.Scott et D.C.Pike, Fl. Austral. Suppl. 21: 114, 2003 (McCarthy 2003). Based on: Fossombronia truncata G.A.M.Scott et D.C.Pike, J. Hattori Bot. Lab. 62: 383, 1987 (Scott and Pike 1987), *nom. inval*.

*** Fossombronia tumida Mitt., J. Linn. Soc., Bot. 16 (91): 193, 1877 (Mitten 1877).

** Fossombronia valparaisiana Hässel, Beih. Nova Hedwigia 131: 14, 2007 (Hässel and Villagrán 2007).

** Fossombronia vermiculata G.A.M.Scott et D.C.Pike, J. Hattori Bot. Lab. 56: 345, 1984 (Scott and Pike 1984).

*** Fossombronia wattsii Steph., Sp. Hepat. (Stephani) 6: 75, 1917 (Stephani 1917a).

*** Fossombronia wondraczekii (Corda) Dumort. ex Lindb., Helsingf. Dagbl. 1873 (273, 7 Oct): 2, 1873 (Lindberg 1873c). Bas.: Jungermannia wondraczekii Corda, Deutschl. Fl. (Sturm), Abt. 2, Cryptog.: 30, 1830 (Corda 1830).

*** Fossombronia wrightii Austin, Bot. Bull. (Hanover) 1 (8): 36, 1876 (Austin 1876a).

** Fossombronia zuurbergensis Perold, Bothalia 31 (1): 25, 2001 (Perold 2001b).

######## *** Petalophyllaceae Stotler et Crand.-Stotl.

by R. Stotler and B.J. Crandall-Stotler

*** **Petalophyllum Nees et Gottsche**, Nov. Stirp. Pug. 8: 29, 1844 (Lehmann 1844).

*** Petalophyllum americanum C.H.Ford et Crand.-Stotl., Novon 12 (3): 335, 2002 (Crandall-Stotler et al. 2002).

*** Petalophyllum hodgsoniae C.H.Ford et Crand.-Stotl., Novon 12 (3): 336, 2002 (Crandall-Stotler et al. 2002).

*** Petalophyllum indicum Kashyap, J. Indian Bot. Soc. 7: 14, 1928 (Kashyap 1928).

*** Petalophyllum preissii Gottsche, Nov. Stirp. Pug. 8: 30, 1844 (Lehmann 1844).

*** Petalophyllum ralfsii (Wilson) Nees et Gottsche, Nov. Stirp. Pug. 8: 30, 1844 (Lehmann 1844). Bas.: Jungermannia ralfsii Wilson, Suppl. Engl. Bot. 4: tab. 2874, 1849 (Borrer et al. 1849).

*** **Sewardiella Kashyap**, New Phytol. 14 (1): 5, 1915 (Kashyap 1915).

*** Sewardiella tuberifera Kashyap, New Phytol. 14 (1): 5, 1915 (Kashyap 1915).

Makinoiineae He-Nygrén, Juslén, Ahonen, Glenny et Piippo

######## *** Makinoaceae Nakai

by R. Stotler and B.J. Crandall-Stotler

*** **Makinoa Miyake**, Bot. Mag. (Tokyo) 13 (144): 23, 1899 (Miyake 1899).

*** Makinoa crispata (Steph.) Miyake, Bot. Mag. (Tokyo) 13 (144): 23, 1899 (Miyake 1899). Bas.: Pellia crispata Steph., Bull. Herb. Boissier 5 (2): 103, 1897 (Stephani 1897b).

###### Pallaviciniales W.Frey et M.Stech

####### Pallaviciniineae R.M.Schust.

######## *** Hymenophytaceae R.M.Schust.

by R. Stotler and B.J. Crandall-Stotler

*** **Hymenophyton Dumort.**, Recueil Observ. Jungerm.: 25, 1835 (Dumortier 1835).

*** Hymenophyton flabellatum (Labill.) Dumort. ex Trevis., Mem. Reale Ist. Lombardo Sci. (Ser. 3), C. Sci. Mat. 4 (13): 430, 1877 (Trevisan 1877). Bas.: Jungermannia flabellata Labill., Nov. Holl. Pl. 2: 109, 1806 (Labillardière 1806).

*** Hymenophyton leptopodum (Hook.f. et Taylor) A.Evans, Trans. Connecticut Acad. Arts 8 (16): 274, 1893 (Evans 1893). Bas.: Jungermannia leptopoda Hook.f. et Taylor, London J. Bot. 3: 571, 1844 (Hooker and Taylor 1844d).

*** Hymenophyton pedicellatum Steph., Kungl. Svenska Vetensk.-Akad. Handl. (n.ser.) 46 (9): 11, 1911 (Stephani 1911b).

######## *** Moerckiaceae K.I.Goebel ex Stotler et Crand.-Stotl.

by B.J. Crandall-Stotler and R. Stotler

Mamontov et al. (2015) showed that the family Moerckiaceae is heterogeneous and they moved Hattorianthus and Moerckia flotoviana to their new family Cordaeaceae. However, the type of Moerckiaceae (Moerckia hibernica) was not included in the study and only provisionally left in Moerckia. We prefer to follow Crandall-Stotler & Stotler (2007) until Moerckia hibernica is further studied.

*** **Hattorianthus R.M.Schust. et Inoue**, Bull. Natl. Sci. Mus. Tokyo, B 1 (3): 103, 1975 (Schuster and Inoue 1975).

*** Hattorianthus erimonus (Steph.) R.M.Schust. et Inoue, Bull. Natl. Sci. Mus. Tokyo, B 1 (3): 106, 1975 (Schuster and Inoue 1975). Bas.: Pallavicinia erimona Steph., Bull. Herb. Boissier 5 (2): 102, 1897 (Stephani 1897b).

*** **Moerckia Gottsche**, Hepat. Eur., Leberm. 13-14: no. 121, 1860 [1861] (Rabenhorst 1860).

*** Moerckia blyttii (Mørch) Brockm., Arch. Vereins Freunde Naturgesch. Mecklenburg 17: 190, 1863 (Brockmüller 1863). Bas.: Jungermannia blyttii Mørch, Fl. Danica 12: 6, 1830 (Hornemann 1830).

*** Moerckia flotoviana (Nees) Schiffn., Österr. Bot. Z. 51 (2): 43, 1901 (Schiffner 1901). Bas.: Cordaea flotoviana Nees, Flora 16 (26): 405, 1833 (Nees 1833b).

*** Moerckia hibernica (Hook.) Gottsche, Hepat. Eur., Leberm. 13-14: no. 121, 1860 [1861] (Rabenhorst 1860). Bas.: Jungermannia hibernica Hook., Brit. Jungermann.: tab. 78, 1816 (Hooker 1816a).

######## *** Pallaviciniaceae Mig.

by R. Stotler and B.J. Crandall-Stotler

######### ** Pallavicinioideae Mig. ex Grolle

*** **Jensenia Lindb.**, Not. Sällsk. Fauna Fl. Fenn. Förh. 9: 13, 1868 (Lindberg 1868a).

*** Jensenia connivens (Colenso) Grolle, Rev. Bryol. Lichénol. 33 (1/2): 228, 1964 [1965] (Grolle 1964j). Bas.: Symphyogyna connivens Colenso, Trans. & Proc. New Zealand Inst. 20: 254, 1888 (Colenso 1888).

* Jensenia crassifrons (Steph.) S.Schuette et Stotler, J. Hattori Bot. Lab. 97: 300, 2005 (Schuette and Stotler 2005). Bas.: Pallavicinia crassifrons Steph., Mém. Herb. Boissier 11: 21 (325), 1900 (Stephani 1900c). ^421^

*** Jensenia decipiens (Mitt.) Grolle, Rev. Bryol. Lichénol. 33 (1/2): 228, 1964 [1965] (Grolle 1964j). Bas.: Steetzia decipiens Mitt., J. Proc. Linn. Soc., Bot. 5 (18): 123, 1860 [1861] (Mitten 1860c).

*** Jensenia difformis (Nees) Grolle, Rev. Bryol. Lichénol. 33 (1/2): 228, 1964 [1965] (Grolle 1964j). Bas.: Jungermannia difformis Nees, Fl. Bras. (Martius) 1 (1): 329, 1833 (Nees 1833a).

*** Jensenia florschuetzii Gronde, Proc. Kon. Ned. Akad. Wetensch. C 83 (3): 273, 1980 (Van der Gronde 1980).

*** Jensenia spinosa (Lindenb. et Gottsche) Grolle, Acta Bot. Fenn. 133: 65, 1986 (Grolle and Piippo 1986). Bas.: Symphyogyna spinosa Lindenb. et Gottsche, Syn. Hepat. 5: 786, 1847 (Gottsche et al. 1847).

*** Jensenia wallisii (J.B.Jack et Steph.) Grolle, Rev. Bryol. Lichénol. 33 (1/2): 228, 1964 [1965] (Grolle 1964j). Bas.: Pallavicinia wallisii J.B.Jack et Steph., Hedwigia 31 (1): 23, 1892 (Jack and Stephani 1892).

*** **Pallavicinia Gray**, Nat. Arr. Brit. Pl. 1: 775, 1821 (Gray 1821) nom. conserv.

*** Pallavicinia ambigua (Mitt.) Steph., Mém. Herb. Boissier 11: 8 (312), 1900 (Stephani 1900a). Bas.: Steetzia ambigua Mitt., J. Proc. Linn. Soc., Bot. 5 (18): 123, 1860 [1861] (Mitten 1860c).

** Pallavicinia baldwinii (Austin) A.Evans, Trans. Connecticut Acad. Arts 8 (15): 259, 1891 (Evans 1891). Bas.: Steetzia baldwinii Austin, Bull. Torrey Bot. Club 6 (52): 303, 1879 (Austin 1879).

* Pallavicinia bipinnata Steph., Sp. Hepat. (Stephani) 6: 62, 1917 (Stephani 1917a).

** Pallavicinia camisassai Gola, Mem. Reale Accad. Sci. Torino (ser. 2) 65 (1): 4, 1916 (Gola 1916).

** Pallavicinia cylindrica (Austin) A.Evans, Trans. Connecticut Acad. Arts 8 (15): 259, 1891 (Evans 1891). Bas.: Steetzia cylindrica Austin, Bull. Torrey Bot. Club 5 (3): 17, 1874 (Austin 1874).

** Pallavicinia himalayensis Schiffn., Mém. Herb. Boissier 11: 13 (317), 1900 (Stephani 1900c).

*** Pallavicinia indica Schiffn., Denkschr. Kaiserl. Akad. Wiss., Math.-Naturwiss. Kl. 67: 183, 1898 (Schiffner 1898a).

*** Pallavicinia levieri Schiffn., Denkschr. Kaiserl. Akad. Wiss., Math.-Naturwiss. Kl. 67: 184, 1898 (Schiffner 1898a).

*** Pallavicinia lyellii (Hook.) Gray, Nat. Arr. Brit. Pl. 1: 775, 1821 (Gray 1821). Bas.: Jungermannia lyellii Hook., Brit. Jungermann.: tab. 77, 1816 (Hooker 1816a).

** Pallavicinia pilifera Steph., Hedwigia 30 (6): 271, 1891 (Stephani 1891c).

** Pallavicinia purpurea Steph., Sp. Hepat. (Stephani) 6: 64, 1917 (Stephani 1917a).

** Pallavicinia ridleyi Steph., Sp. Hepat. (Stephani) 6: 64, 1917 (Stephani 1917a).

*** Pallavicinia rubristipa Schiffn., Österr. Bot. Z. 56 (1): 24, 1906 (Schiffner 1906b).

*** Pallavicinia subciliata (Austin) Steph., Mém. Herb. Boissier 11: 9 (313), 1900 (Stephani 1900c). Bas.: Steetzia subciliata Austin, Bull. Torrey Bot. Club 6 (52): 303, 1879 (Austin 1879).

*** Pallavicinia xiphoides (Hook.f. et Taylor) Trevis., Mem. Reale Ist. Lombardo Sci. (Ser. 3), C. Sci. Mat. 4 (13): 427, 1877 (Trevisan 1877). Bas.: Jungermannia xiphoides Hook.f. et Taylor, London J. Bot. 3: 569, 1844 (Hooker and Taylor 1844d).

*** **Podomitrium Mitt.**, Bot. antarct. voy. II (Fl. Nov.-Zel. 2): 164, 1855 (Mitten 1855).

*** Podomitrium malaccense (Steph.) Campb., Amer. J. Bot. 2 (5): 199, 1915 (Campbell 1915). Bas.: Hymenophyton malaccense Steph., Hedwigia 34 (2): 46, 1895 (Stephani 1895c).

*** Podomitrium marginatum (Steph.) Hürl., Bauhinia 4 (1): 78, 1968 [1969] (Hürlimann 1968). Bas.: Hymenophyton marginatum Steph., Sp. Hepat. (Stephani) 6: 61, 1917 (Stephani 1917a).

*** Podomitrium phyllanthus (Hook.) Mitt., Bot. antarct. voy. II (Fl. Nov.-Zel. 2): 164, 1855 (Mitten 1855). Bas.: Jungermannia phyllanthus Hook., Musci Exot. 1: tab. 95, 1818 (Hooker 1818).

######### ** Symphyogynoideae R.M.Schust. ex Grolle

** **Greeneothallus Hässel**, J. Bryol. 11 (1): 115, 1980 (Hässel 1980).

*** Greeneothallus gemmiparus Hässel, J. Bryol. 11 (1): 115, 1980 (Hässel 1980).

*** **Seppeltia Grolle**, J. Hattori Bot. Lab. 60: 276, 1986 (Grolle and Seppelt 1986).

*** Seppeltia succuba Grolle, J. Hattori Bot. Lab. 60: 276, 1986 (Grolle and Seppelt 1986).

*** **Symphyogyna Nees et Mont.**, Ann. Sci. Nat. Bot. (sér. 2) 5: 66, 1836 (Nees and Montagne 1836).

*** Symphyogyna apiculispina Steph., Biblioth. Bot. 87 (2): 180, 1916 (Stephani 1916a).

*** Symphyogyna aspera Steph. ex F.A.McCormick, Bot. Gaz. 58 (5): 403, 1914 (McCormick 1914).

** Symphyogyna atronervia Taylor, London J. Bot. 5: 409, 1846 (Taylor 1846b).

** Symphyogyna bogotensis Steph., Mém. Herb. Boissier 11: 42 (346), 1900 (Stephani 1900c). Based on: Symphyogyna hymenophyllum var. bogotensis Gottsche, Ann. Sci. Nat. Bot. (sér. 5) 1: 181, 1864 (Gottsche 1864), *nom. inval*.

* Symphyogyna boliviensis Steph., Biblioth. Bot. 87 (2): 180, 1916 (Stephani 1916a).

*** Symphyogyna brasiliensis Nees et Mont., Ann. Sci. Nat. Bot. (sér. 2) 5: 67, 1836 (Nees and Montagne 1836). *Nom. nov. pro Jungermannia brasiliensis* Nees, Enum. Pl. Crypt. Javae: 11, 1830 (Nees 1830), *nom. illeg*.

** Symphyogyna brasiliensis var. angustior (Gottsche, Lindenb. et Nees) Gottsche, Ann. Sci. Nat. Bot. (sér. 5) 1: 183, 1864 (Gottsche 1864). Bas.: Symphyogyna brasiliensis β angustior Gottsche, Lindenb. et Nees, Syn. Hepat. 4: 484, 1846 (Gottsche et al. 1846).

** Symphyogyna brasiliensis var. subsinuata Schiffn., Österr. Akad. Wiss., Math.-Naturwiss. Kl., Denkschr. 111: 32, 1964 (Schiffner and Arnell 1964).

*** Symphyogyna brongniartii Mont., Ann. Sci. Nat. Bot. (sér. 2) 19: 265, 1843 (Montagne 1843).

*** Symphyogyna circinata Nees et Mont., Ann. Sci. Nat. Bot. (sér. 2) 5: 69, 1836 (Nees and Montagne 1836).

*** Symphyogyna digitisquama Steph., Mém. Herb. Boissier 11: 31 (335), 1900 (Stephani 1900c).

** Symphyogyna fuscovirens A.Evans, Trans. Connecticut Acad. Arts 28 (6): 320, 1927 (Evans 1927).

*** Symphyogyna hochstetteri Nees et Mont., Ann. Sci. Nat. Bot. (sér. 2) 5: 68, 1836 (Nees and Montagne 1836).

*** Symphyogyna hymenophyllum (Hook.) Nees et Mont., Ann. Sci. Nat. Bot. (sér. 2) 5: 68, 1836 (Nees and Montagne 1836). Bas.: Jungermannia hymenophyllum Hook., Musci Exot. 1: tab. 14, 1818 (Hooker 1818).

** Symphyogyna hymenophyllum var. heterogenum Spruce, Trans. & Proc. Bot. Soc. Edinburgh 15: 536, 1885 (Spruce 1885).

** Symphyogyna ignambiensis Hürl., Bauhinia 4 (1): 79, 1968 [1969] (Hürlimann 1968).

** Symphyogyna interrupta Carrington et Pearson, Proc. Linn. Soc. New South Wales (ser. 2) 2 (4): 1053, 1888 (Carrington and Pearson 1888a).

** Symphyogyna irregularis Steph., Mém. Herb. Boissier 11: 29 (333), 1900 (Stephani 1900c).

** Symphyogyna lacerosquama Steph., Sp. Hepat. (Stephani) 6: 67, 1917 (Stephani 1917a).

*** Symphyogyna leptothelia Taylor, London J. Bot. 5: 408, 1846 (Taylor 1846b).

** Symphyogyna lindmanii A.Evans, Trans. Connecticut Acad. Arts 28 (6): 316, 1927 (Evans 1927).

*** Symphyogyna luetzelburgii Herzog, Repert. Spec. Nov. Regni Veg. 21 (1/7): 22, 1925 (Herzog 1925a).

*** Symphyogyna marginata Steph., Mém. Herb. Boissier 11: 30 (334), 1900 (Stephani 1900c).

*** Symphyogyna mexicana Steph., Rev. Bryol. 36 (6): 140, 1909 (Stephani 1909b).

** Symphyogyna multiflora Steph., J. & Proc. Roy. Soc. New South Wales 48 (1/2): 133, 1914 (Stephani and Watts 1914).

* Symphyogyna paucidens Steph., Kungl. Svenska Vetensk.-Akad. Handl. (n.ser.) 46 (9): 13, 1911 (Stephani 1911b). ^422^

*** Symphyogyna podophylla (Thunb.) Nees et Mont., Flora 29 (9): 135, 1846 (Krauss 1846). Bas.: Jungermannia podophylla Thunb., Prodr. Pl. Cap. 2: 174, 1800 (Thunberg 1800).

* Symphyogyna purpureolimbata E.A.Hodgs., Trans. Roy. Soc. New Zealand, Bot. 3 (4): 95, 1965 (Hodgson 1965).

*** Symphyogyna rectidens Grolle, Acta Bot. Fenn. 133: 68, 1986 (Grolle and Piippo 1986).

** Symphyogyna rhodina (Hook.f. et Taylor) Gottsche, Lindenb. et Nees, Syn. Hepat. 4: 487, 1846 (Gottsche et al. 1846). Bas.: Jungermannia rhodina Hook.f. et Taylor, London J. Bot. 4: 93, 1845 (Hooker and Taylor 1845).

*** Symphyogyna rubescens Steph., Mém. Herb. Boissier 11: 29 (333), 1900 (Stephani 1900c).

*** Symphyogyna rubritincta A.Evans, Trans. Connecticut Acad. Arts 27 (1): 38, 1925 (Evans 1925).

** Symphyogyna semi-involucrata Austin, Bull. Torrey Bot. Club 5 (3): 15, 1874 (Austin 1874).

** Symphyogyna similis Grolle, Acta Bot. Fenn. 133: 70, 1986 (Grolle and Piippo 1986).

*** Symphyogyna sinuata (Sw.) Nees et Mont., Voy. Amér. Mérid., Bot. 7 (1): 61, 1839 (Montagne 1839b). Bas.: Jungermannia sinuata Sw., Prodr. (Swartz): 145, 1788 (Swartz 1788).

*** Symphyogyna subsimplex Mitt., Bot. antarct. voy. II (Fl. Nov.-Zel. 2): 166, 1855 (Mitten 1855).

*** Symphyogyna tenuinervis (Hook.f. et Taylor) Grolle, J. Hattori Bot. Lab. 61: 253, 1986 [1987] (Grolle 1986a). Bas.: Jungermannia tenuinervis Hook.f. et Taylor, London J. Bot. 3: 570, 1844 (Hooker and Taylor 1844d).

*** Symphyogyna trivittata Spruce, J. Linn. Soc., Bot. 30 (210): 365, 1895 (Gepp 1895b).

* Symphyogyna ulvoides (Reinw., Blume et Nees) Nees, Syn. Hepat. 4: 487, 1846 (Gottsche et al. 1846). Bas.: Jungermannia ulvoides Reinw., Blume et Nees, Nova Acta Phys.-Med. Acad. Caes. Leop.-Carol. Nat. Cur. 12 (1): 196, 1824 [1825] (Reinwardt et al. 1824a). ^423^

*** Symphyogyna undulata Colenso, Trans. & Proc. New Zealand Inst. 16: 356, 1884 (Colenso 1884).

** Symphyogyna volkensii Steph., Mém. Herb. Boissier 11: 35 (339), 1900 (Stephani 1900c).

** **Symphyogynopsis Grolle**, Acta Bot. Fenn. 133: 72, 1986 (Grolle and Piippo 1986).

*** Symphyogynopsis gottscheana (Mont. et Nees) Grolle, J. Hattori Bot. Lab. 63: 441, 1987 (Grolle 1987). Bas.: Symphyogyna gottscheana Mont. et Nees, Syn. Hepat. 4: 484, 1846 (Gottsche et al. 1846).

** **Xenothallus R.M.Schust.**, J. Hattori Bot. Lab. 26: 293, 1963 (Schuster 1963b).

*** Xenothallus vulcanicola R.M.Schust., J. Hattori Bot. Lab. 26: 293, 1963 (Schuster 1963b).

######## ** Sandeothallaceae R.M.Schust.

** **Sandeothallus R.M.Schust.**, Nova Hedwigia 36: 10, 1982 (Schuster 1982).

** Sandeothallus japonicus (Inoue) Crand.-Stotl. et Stotler, Beih. Nova Hedwigia 131: 58, 2007 (Crandall-Stotler and Stotler 2007). Bas.: Moerckia japonica Inoue, Bull. Natl. Sci. Mus. Tokyo, B 11 (1): 8, 1985 (Inoue 1985).

** Sandeothallus radiculosus (Schiffn.) R.M.Schust., Nova Hedwigia 36: 11, 1982 (Schuster 1982). Bas.: Moerckia radiculosa Schiffn., Österr. Bot. Z. 51 (2): 48, 1901 (Schiffner 1901).

####### Phyllothalliineae R.M.Schust.

######## *** Phyllothalliaceae E.A.Hodgs. ex T.Katag.

*** **Phyllothallia E.A.Hodgs.**, Trans. Roy. Soc. New Zealand, Bot. 2 (19): 247, 1964 (Hodgson 1964).

*** Phyllothallia fuegiana R.M.Schust., Trans. Brit. Bryol. Soc. 5 (2): 284, 1967 (Schuster 1967d).

*** Phyllothallia nivicola E.A.Hodgs., Trans. Roy. Soc. New Zealand, Bot. 2 (19): 247, 1964 (Hodgson 1964).

###### Pelliales He-Nygrén, Juslén, Ahonen, Glenny et Piippo

####### *** Noterocladaceae W.Frey et M.Stech

Frey and Stech (2005a) proposed Noterocladaceae as a monogeneric family based on molecular and morphological evidence, which was later corroborated by Crandall-Stotler et al. (2010).

*** **Noteroclada Taylor ex Hook.f. et Wilson**, London J. Bot. 3: 166, 1844 (Hooker and Wilson 1844).

*** Noteroclada confluens Taylor, London J. Bot. 3: 166, 1844 (Hooker and Wilson 1844).

**Excluded from the genus**

* Noteroclada longiuscula Colenso, Trans. & Proc. New Zealand Inst. 19: 299, 1887 (Colenso 1887). ^424^

####### *** Pelliaceae H.Klinggr.

Frey and Stech (2005b) recognized Pelliaceae as a monogeneric family, which was later supported by Crandall-Stotler et al. (2010).

** **Pellia Raddi**, Jungermanniogr. Etrusca: 38, 1818 (Raddi 1818a) nom. conserv.

*** **subg. Apopellia Grolle**, J. Bryol. 12 (3): 427, 1983 (Grolle 1983a).

** Pellia alpicola R.M.Schust. ex L.Söderstr., A.Hagborg et von Konrat, Phytotaxa 76 (3): 39, 2013 (Söderström et al. 2013d). Based on: Pellia endiviifolia subsp. alpicola R.M.Schust., J. Hattori Bot. Lab. 70: 145, 1991 (Schuster 1991b), *nom. inval*.

*** Pellia endiviifolia (Dicks.) Dumort., Recueil Observ. Jungerm.: 27, 1835 (Dumortier 1835). Bas.: Jungermannia endiviifolia Dicks., Fasc. Pl. Crypt. Brit. 4: 19, 1801 (Dickson 1801).

** Pellia megaspora R.M.Schust., J. Bryol. 11 (3): 419, 1981 (Schuster 1981b).

*** **subg. Pellia**

** Pellia appalachiana R.M.Schust., Phytotaxa 76 (3): 39, 2013 (Söderström et al. 2013d). Based on: Pellia appalachiana R.M.Schust., J. Hattori Bot. Lab. 70: 145, 1991 (Schuster 1991b), *nom. inval*.

*** Pellia epiphylla (L.) Corda, Gen. hepat.: 654, 1829 (Corda 1829). Bas.: Jungermannia epiphylla L., Sp. Pl. 1: 1135, 1753 (Linnaeus 1753).

** Pellia epiphylla subsp. borealis (Lorb.) Messe, Bull. Soc. Roy. Bot. Belgique 114 (1): 13, 1981 (Messe 1981). Bas.: Pellia borealis Lorb., Jahrb. Wiss. Bot. 80: 697, 1934 (Lorbeer 1934).

*** Pellia neesiana (Gottsche) Limpr., Hedwigia 15 (2): 18, 1876 (Limpricht 1876). Bas.: Pellia epiphylla f. neesiana Gottsche, Hedwigia 6 (5): 69, 1867 (Gottsche 1867).

***Incertae sedis***

* Pellia cordaeana Trevis., Mem. Reale Ist. Lombardo Sci. (Ser. 3), C. Sci. Mat. 4 (13): 433, 1877 (Trevisan 1877).

* Pellia crispa P.Kumm., Führer Leberm.: 60, 1875 (Kummer 1875).

* Pellia gottscheana Kreh, Nova Acta Acad. Caes. Leop.-Carol. German. Nat. Cur. 90 (4): 237, 1909 (Kreh 1909).

* Pellia longifolia P.Kumm., Führer Leberm.: 60, 1875 (Kummer 1875).

* Pellia undulata P.Kumm., Führer Leberm.: 60, 1875 (Kummer 1875).

#### Marchantiopsida Cronquist, Takht. et W.Zimm.

##### Blasiidae He-Nygrén, Juslén, Ahonen, Glenny et Piippo

###### Blasiales Stotler et Crand.-Stotl.

####### *** Blasiaceae H.Klinggr.

by L. Söderström

*** **Blasia L.**, Sp. Pl. 1: 1138, 1753 (Linnaeus 1753).

*** Blasia pusilla L., Sp. Pl. 1: 1138, 1753 (Linnaeus 1753).

*** **Cavicularia Steph.**, Bull. Herb. Boissier 5 (2): 87, 1897 (Stephani 1897b).

*** Cavicularia densa Steph., Bull. Herb. Boissier 5 (2): 87, 1897 (Stephani 1897b).

##### Marchantiidae Engl.

###### Lunulariales H.Klinggr.

####### *** Lunulariaceae H.Klinggr.

by D.G. Long

*** **Lunularia Adans.**, Fam. Pl. (Adanson) 2: 15, 1763 (Adanson 1763).

*** Lunularia cruciata (L.) Dumort. ex Lindb., Not. Sällsk. Fauna Fl. Fenn. Förh. 9: 298, 1868 (Lindberg 1868b). Bas.: Marchantia cruciata L., Sp. Pl. 1: 1137, 1753 (Linnaeus 1753).

* Lunularia cruciata subsp. thaxteri (A.Evans et Herzog) R.M.Schust., Hepat. Anthocerotae N. Amer. 6: 91, 1992 (Schuster 1992d). Bas.: Lunularia thaxteri A.Evans et Herzog, Arch. Esc. Fárm. Fac. Ci. Méd. Córdoba 7: 5, 1938 (Herzog and Hosseus 1938). ^425^

###### Marchantiales Limpr.

####### *** Aytoniaceae Cavers

by D.G. Long

The genera of Aytoniaceae follow the treatment of Bischler-Causse et al. (2005) with the exception of Asterella and Mannia which were re-defined by Schill et al. (2010).

*** **Asterella P.Beauv.**, Dict. Sci. Nat. [F. Cuvier] 3: 257, 1805 (Palisot de Beauvois 1805a) nom. conserv. ^426^

** **subg. Asterella**

** **sect. Asterella**

*** Asterella tenella (L.) P.Beauv., Dict. Sci. Nat. [F. Cuvier] 3: 258, 1805 (Palisot de Beauvois 1805a). Bas.: Marchantia tenella L., Sp. Pl. 1: 1137, 1753 (Linnaeus 1753).

** **sect. Brachyblepharis (Nees) D.G.Long**, J. Bryol. 22 (2): 113, 2000 (Grolle and Long 2000). Bas.: Fimbraria subg. Brachyblepharis Nees, Syn. Hepat. 4: 569, 1846 (Gottsche et al. 1846).

*** Asterella abyssinica (Gottsche) Grolle, Explor. Hydrobiol. Lac Bangweolo Luapula: 170, 1972 (Vanden Berghen 1972b). Bas.: Fimbraria abyssinica Gottsche, Syn. Hepat. 4: 569, 1846 (Gottsche et al. 1846).

*** Asterella africana (Mont.) Underw. ex A.Evans, Contr. U.S. Natl. Herb. 20: 250, 1920 (Evans 1920). Bas.: Fimbraria africana Mont., Hist. Nat. Îles Canaries 3 (2): 61, 1840 (Montagne 1840b).

*** Asterella blumeana (Nees) Kachroo, J. Gauhati India Univ. 3: 130, 1952 (Kachroo 1952). Bas.: Fimbraria blumeana Nees, Syn. Hepat. 4: 564, 1846 (Gottsche et al. 1846).

*** Asterella chilensis (Nees et Mont.) A.Evans, Bull. Torrey Bot. Club 46 (12): 469, 1919 (Evans 1919b). Bas.: Fimbraria chilensis Nees et Mont., Ann. Sci. Nat. Bot. (sér. 2) 9: 41, 1838 (Montagne 1838).

*** Asterella cruciata (Steph.) Horik., Hikobia 1 (2): 79, 1951 (Horikawa 1951c). Bas.: Fimbraria cruciata Steph., Sp. Hepat. (Stephani) 6: 12, 1917 (Stephani 1917a).

*** Asterella dissoluta (Steph.) Grolle, Wiss. Z. Friedrich-Schiller-Univ. Jena, Math.-Naturwiss. Reihe 38 (2): 237, 1989 (Grolle 1989b). Bas.: Fimbraria dissoluta Steph., Pflanzenw. Ost-Afrikas C: 62, 1895 (Stephani 1895d).

*** Asterella dominicensis S.W.Arnell, Bryologist 61 (2): 140, 1958 (Arnell 1958c).

*** Asterella khasyana (Griff.) Grolle, Khumbu Himal 1 (4): 267, 1966 (Grolle 1966k). Bas.: Octokepos khasyanus Griff., Not. pl. asiat. 2: 343, 1849 (Griffith 1849).

*** Asterella leptophylla (Mont.) Grolle, Feddes Repert. 87 (3/4): 246, 1976 (Grolle 1976a). Bas.: Fimbraria leptophylla Mont., Ann. Sci. Nat. Bot. (sér. 2) 18: 16, 1842 (Montagne 1842b).

*** Asterella limbata D.G.Long et Grolle, J. Bryol. 18 (2): 287, 1994 (Long and Grolle 1994).

** Asterella shimizuana Inoue, Bull. Natl. Sci. Mus. Tokyo (n.ser.) 10 (3): 361, 1967 (Inoue 1967b). ^427^

*** Asterella tenera (Mitt.) R.M.Schust., J. Hattori Bot. Lab. 26: 298, 1963 (Schuster 1963b). Bas.: Fimbraria tenera Mitt., Bot. antarct. voy. II (Fl. Nov.-Zel. 2): 170, 1855 (Mitten 1855).

* Asterella tenerrima (Steph.) H.A.Mill., Phytologia 47 (4): 320, 1981 (Miller 1981). Bas.: Fimbraria tenerrima Steph., Sp. Hepat. (Stephani) 6: 17, 1917 (Stephani 1917a). ^428^

*** Asterella venosa (Lehm. et Lindenb.) A.Evans, Contr. U.S. Natl. Herb. 20: 286, 1920 (Evans 1920). Bas.: Fimbraria venosa Lehm. et Lindenb., Nov. Stirp. Pug. 4: 29, 1832 (Lehmann 1832).

** **subg. Phragmoblepharis Grolle**, Feddes Repert. 87 (3/4): 246, 1976 (Grolle 1976a).

*** Asterella australis (Hook.f. et Taylor) Verd., Ann. Bryol. 5: 126, 1932 (Verdoorn 1932c). Bas.: Fimbraria australis Hook.f. et Taylor, London J. Bot. 3: 573, 1844 (Hooker and Taylor 1844d).

*** Asterella bachmannii (Steph.) S.W.Arnell, Hepat. South Africa: 62, 1963 (Arnell 1963b). Bas.: Fimbraria bachmannii Steph., Hedwigia 33 (1): 7, 1894 (Stephani 1894a).

*** Asterella bolanderi (Austin) Underw., Bot. Gaz. 20 (2): 61, 1895 (Underwood 1895). Bas.: Fimbraria bolanderi Austin, Proc. Acad. Nat. Sci. Philadelphia 21: 230, 1869 (Austin 1869).

* Asterella caucasica (Steph.) H.Buch, A.Evans et Verd., Ann. Bryol. 10: 8, 1937 [1938] (Buch et al. 1937). Bas.: Fimbraria caucasica Steph., Bull. Herb. Boissier 7 (3): 206 (132), 1899 (Stephani 1899b). ^429^

*** Asterella conocephala (Steph.) R.M.Schust., J. Hattori Bot. Lab. 26: 298, 1963 (Schuster 1963b). Bas.: Fimbraria conocephala Steph., Bull. Herb. Boissier 7 (3): 205 (131), 1899 (Stephani 1899b).

** Asterella coronata (Steph.) H.A.Mill., Phytologia 47 (4): 319, 1981 (Miller 1981). Bas.: Fimbraria coronata Steph., Sp. Hepat. (Stephani) 6: 12, 1917 (Stephani 1917a).

** Asterella dioica (Steph.) H.A.Mill., Phytologia 47 (4): 319, 1981 (Miller 1981). Bas.: Fimbraria dioica Steph., J. & Proc. Roy. Soc. New South Wales 48 (1/2): 104, 1914 (Stephani and Watts 1914).

* Asterella dognyensis H.A.Mill., Phytologia 47 (4): 319, 1981 (Miller 1981). *Nom. nov. pro Fimbraria umbonata* Steph., Sp. Hepat. (Stephani) 6: 17, 1917 (Stephani 1917a), *nom. illeg*. ^430^

*** Asterella drummondii (Taylor) R.M.Schust. ex D.G.Long, J. Bryol. 21 (1): 76, 1999 (Long 1999b). Bas.: Fimbraria drummondii Taylor, London J. Bot. 5: 412, 1846 (Taylor 1846b).

*** Asterella echinella (Gottsche) Underw., Bot. Gaz. 20 (2): 62, 1895 (Underwood 1895). Bas.: Fimbraria echinella Gottsche, Mexik. Leverm.: 271, 1863 (Gottsche 1863).

*** Asterella elegans (Spreng.) Trevis., Rendiconti Reale Ist. Lombardo Sci. (ser. 2) 7: 785, 1874 (Trevisan 1874). Bas.: Fimbraria elegans Spreng. Syst. Veg. (ed. 16) [Sprengel] 4 (1): 235, 1827 (Sprengel 1827a).

*** Asterella heteroflora (Steph.) H.A.Mill., Phytologia 47 (4): 320, 1981 (Miller 1981). Bas.: Fimbraria heteroflora Steph., Sp. Hepat. (Stephani) 6: 14, 1917 (Stephani 1917a).

*** Asterella innovans (Austin) H.A.Mill., Ark. Bot. (n.ser.) 5 (2): 529, 1963 (Miller 1963). Bas.: Marchantia innovans Austin, Bull. Torrey Bot. Club 5 (3): 14, 1874 (Austin 1874).

*** Asterella lateralis M.Howe, Bull. Torrey Bot. Club 25 (4): 189, 1898 (Howe 1898b).

*** Asterella lindenbergiana (Corda ex Nees) Lindb. ex Arnell, Lebermoosstud. nördl. Norwegen: 2, 1892 (Arnell 1892). Bas.: Fimbraria lindenbergiana Corda ex Nees, Naturgesch. Eur. Leberm. 4: 266, 1838 (Nees 1838a).

*** Asterella linearis (Steph.) M.Howe, Bull. Torrey Bot. Club 25 (4): 191, 1898 (Howe 1898b). Bas.: Fimbraria linearis Steph., Bot. Jahrb. Syst. 20 (3): 302, 1895 (Stephani 1895a).

*** Asterella longebarbata (Steph.) H.A.Mill., Phytologia 47 (4): 320, 1981 (Miller 1981). Bas.: Fimbraria longebarbata Steph., Hedwigia 28 (3): 156, 1889 (Stephani 1889d).

*** Asterella macropoda (Spruce) A.Evans, Bull. Torrey Bot. Club 46 (12): 472, 1919 (Evans 1919b). Bas.: Fimbraria macropoda Spruce, Trans. & Proc. Bot. Soc. Edinburgh 15: 564, 1885 (Spruce 1885).

*** Asterella marginata (Nees) S.W.Arnell, Hepat. South Africa: 63, 1963 (Arnell 1963b). Bas.: Fimbraria marginata Nees, Horae Phys. Berol.: 44, 1820 (Nees 1820).

** Asterella muelleri (Gottsche) R.M.Schust., J. Hattori Bot. Lab. 26: 298, 1963 (Schuster 1963b). Bas.: Fimbraria muelleri Gottsche, Bull. Herb. Boissier 7 (3): 203 (129), 1899 (Stephani 1899b).

*** Asterella multiflora (Steph.) Kachroo, J. Hattori Bot. Lab. 19: 3, 1958 (Kachroo 1958). Bas.: Fimbraria multiflora Steph., Bull. Herb. Boissier 7 (3): 198 (124), 1899 (Stephani 1899b).

*** Asterella mussuriensis (Kashyap) Verd., Ann. Bryol. 8: 156, 1935 (Verdoorn 1935). Bas.: Fimbraria mussuriensis Kashyap, J. Bombay Nat. Hist. Soc. 24 (2): 345, 1916 (Kashyap 1916).

*** Asterella mussuriensis subsp. crassa (Shimizu et S.Hatt.) D.G.Long, Lindbergia 26 (1): 44, 2001 (Long 2001). Bas.: Asterella crassa Shimizu et S.Hatt., J. Hattori Bot. Lab. 8: 48, 1952 (Shimizu and Hattori 1952).

*** Asterella pappii (Gola) Grolle, Feddes Repert. 87 (3/4): 246, 1976 (Grolle 1976a). Bas.: Fimbraria pappii Gola, Ann. Bot. (Rome) 13 (1): 65, 1914 (Gola 1914a).

*** Asterella persica (Steph.) M.Howe, Bull. Torrey Bot. Club 25 (4): 191, 1898 (Howe 1898b). Bas.: Fimbraria persica Steph., Hedwigia 33 (1): 7, 1894 (Stephani 1894a).

* Asterella preussii (Schiffn.) M.Howe, Bull. Torrey Bot. Club 25 (4): 191, 1898 (Howe 1898b). Bas.: Fimbraria preussii Schiffn., Bot. Jahrb. Syst. 20 (3): 303, 1895 (Stephani 1895a). ^431^

** Asterella setisquama (Steph.) R.M.Schust., J. Hattori Bot. Lab. 26: 298, 1963 (Schuster 1963b). Bas.: Fimbraria setisquama Steph., Hedwigia 28 (3): 156, 1889 (Stephani 1889d).

*** Asterella syngenesica (Bory) Grolle, Lindbergia 2 (3/4): 230, 1974 (Grolle and Onraedt 1974). Bas.: Marchantia syngenesica Bory, Voy. îles Afrique 2: 95, 1804 (Bory 1804).

** Asterella tasmanica (Steph.) R.M.Schust., J. Hattori Bot. Lab. 26: 298, 1963 (Schuster 1963b). Bas.: Fimbraria tasmanica Steph., Bull. Herb. Boissier 7 (3): 206 (132), 1899 (Stephani 1899b).

*** Asterella versicolor A.Evans, Contr. U.S. Natl. Herb. 20: 307, 1920 (Evans 1920).

*** Asterella vulcanica (Schiffn.) Kachroo et Bapna, J. Indian Bot. Soc. 56 (1): 75, 1977 (Kachroo et al. 1977). Bas.: Hypenantron vulcanicum Schiffn., Denkschr. Kaiserl. Akad. Wiss., Math.-Naturwiss. Kl. 67: 155, 1898 (Schiffner 1898a).

*** Asterella whiteleggeana (Steph.) R.M.Schust., J. Hattori Bot. Lab. 26: 298, 1963 (Schuster 1963b). Bas.: Fimbraria whiteleggeana Steph., Hedwigia 28 (3): 155, 1889 (Stephani 1889d).

*** Asterella wilmsii (Steph.) S.W.Arnell, Hepat. South Africa: 62, 1963 (Arnell 1963b). Bas.: Fimbraria wilmsii Steph., Hedwigia 31 (3): 122, 1892 (Stephani 1892d).

** **subg. Saccatae (Grolle) D.G.Long**, J. Bryol. 22 (2): 113, 2000 (Grolle and Long 2000). Bas.: Asterella subg. Phragmoblepharis sect. Saccatae Grolle, Feddes Repert. 87 (3/4): 346, 1976 (Grolle 1976a).

*** Asterella alpina (Steph.) D.G.Long, J. Hattori Bot. Lab. 93: 9, 2003 (Gradstein et al. 2003). Bas.: Fimbraria alpina Steph., Bull. Herb. Boissier 7 (3): 211 (137), 1899 (Stephani 1899b).

*** Asterella grollei D.G.Long, Bryologist 102 (2): 169, 1999 (Long 1999a).

*** Asterella muscicola (Steph.) S.W.Arnell, Mitt. Bot. Staatssamml. München 2 (16): 263, 1957 (Arnell 1957c). Bas.: Fimbraria muscicola Steph., Hedwigia 31 (3): 121, 1892 (Stephani 1892d).

*** Asterella palmeri (Austin) Underw., Bot. Gaz. 20 (2): 63, 1895 (Underwood 1895). Bas.: Fimbraria palmeri Austin, Bull. Torrey Bot. Club 6 (7): 47, 1875 (Austin 1875c).

*** Asterella pringlei Underw., Bot. Gaz. 20 (2): 64, 1895 (Underwood 1895).

*** Asterella rugosa A.Evans, Contr. U.S. Natl. Herb. 20: 289, 1920 (Evans 1920).

*** Asterella saccata (Wahlenb.) A.Evans, Contr. U.S. Natl. Herb. 20: 276, 1920 (Evans 1920). Bas.: Marchantia saccata Wahlenb., Mag. Neuesten Entdeck. Gesammten Naturk. Ges. Naturf. Freunde Berlin 5: 296, 1811 (Wahlenberg 1811).

** **subg. Wallichianae D.G.Long**, Lindbergia 26 (1): 43, 2001 (Long 2001).

** **sect. Californicae D.G.Long**, J. Hattori Bot. Lab. 97: 257, 2005 (Long 2005).

*** Asterella californica (Hampe ex Austin) Underw., Bot. Gaz. 20 (2): 60, 1895 (Underwood 1895). Bas.: Fimbraria californica Hampe ex Austin, Hepat. bor.-amer.: 33, 1873 (Austin 1873).

** **sect. Wallichianae D.G.Long**, Phytotaxa 173 (1): 87, 2014 (Long et al. 2014).

*** Asterella wallichiana (Lehm. et Lindenb.) Grolle, Khumbu Himal 1 (4): 262, 1966 (Grolle 1966k). Bas.: Fimbraria wallichiana Lehm. et Lindenb., Nov. Stirp. Pug. 4: 4, 1832 (Lehmann 1832).

** **Cryptomitrium Austin ex Underw.**, Bull. Illinois State Lab. Nat. Hist. 2 (1): 36, 1884 (Underwood 1884).

*** Cryptomitrium himalayense Kashyap, New Phytol. 14 (1): 2, 1915 (Kashyap 1915).

*** Cryptomitrium oreades Perold, Bothalia 24 (2): 149, 1994 (Perold 1994).

*** Cryptomitrium tenerum (Hook.) Austin ex Underw., Bull. Illinois State Lab. Nat. Hist. 2 (1): 36, 1884 (Underwood 1884). Bas.: Marchantia tenera Hook., Syn. Pl. (Kunth) 1: 45, 1822 (Kunth 1822).

*** **Mannia Corda**, Gen. hepat.: 646, 1829 (Corda 1829) nom. conserv.

** **subg. Mannia**

*** Mannia androgyna (L.) A.Evans, Chron. Bot. 4: 224, 1938 (Evans 1938a). Bas.: Marchantia androgyna L., Sp. Pl. 1: 1138, 1753 (Linnaeus 1753).

*** Mannia californica (Gottsche) L.C.Wheeler, Bryologist 37 (5): 88, 1934 [1935] (Wheeler 1934). Bas.: Grimaldia californica Underw., Bot. Gaz. 13 (5): 114, 1888 (Underwood 1888).

*** Mannia controversa (Meyl.) D.B.Schill, Edinburgh J. Bot. 65 (1): 36, 2008 (Schill et al. 2008). Bas.: Grimaldia controversa Meyl., Beitr. Kryptogamenfl. Schweiz 6 (4): 87, 1924 (Meylan 1924).

*** Mannia controversa subsp. asiatica D.B.Schill et D.G.Long, Edinburgh J. Bot. 65 (1): 45, 2008 (Schill et al. 2008).

*** Mannia fragrans (Balb.) Frye et L.Clark, Univ. Wash. Publ. Biol. 6 (1): 62, 1937 (Frye and Clark 1937). Bas.: Marchantia fragrans Balb., Mem. Acad. Sci. Turin, Sci. Phys. 15: 76, 1804 (Balbis 1804).

** Mannia fragrans subsp. orientalis R.M.Schust., Hepat. Anthocerotae N. Amer. 6: 201, 1992 (Schuster 1992d). *Nom. nov. pro Mannia barbifrons* Shimizu et S.Hatt., J. Hattori Bot. Lab. 10: 49, 1953 (Shimizu and Hattori 1953b).

* Mannia perssonii Udar et V.Chandra, Canad. J. Bot. 43 (1): 150, 1965 (Udar and Chandra 1965). ^432^

*** Mannia sibirica (Müll.Frib.) Frye et L.Clark, Univ. Wash. Publ. Biol. 6 (1): 66, 1937 (Frye and Clark 1937). Bas.: Grimaldia pilosa var. sibirica Müll.Frib., Lebermoose 1 (5): 265, 1907 (Müller 1907a).

** **subg. Neesiella (Schiffn.) D.B.Schill et D.G.Long**, Bryologist 113 (1): 175, 2010 (Schill et al. 2010). Bas.: Neesiella Schiffn., Hepat. (Engl.-Prantl): 32, 1893 (Schiffner 1893b).

*** Mannia gracilis (F.Weber) D.B.Schill et D.G.Long, Bryologist 113 (1): 173, 2010 (Schill et al. 2010). Bas.: Marchantia gracilis F.Weber, Hist. Musc. Hepat. Prodr.: 105, 1815 (Weber 1815).

* Mannia hegewaldii Bischl., Fl. Neotrop. Monogr. 97: 184, 2005 (Bischler-Causse et al. 2005). ^433^

*** Mannia pilosa (Hornem.) Frye et L.Clark, Univ. Wash. Publ. Biol. 6 (1): 64, 1937 (Frye and Clark 1937). Bas.: Marchantia pilosa Hornem., Fl. Danica 8 (24): 7, tab. 1426, 1810 (Hornemann 1810).

*** Mannia triandra (Scop.) Grolle, J. Bryol. 8 (4): 487, 1975 (Grolle 1975d). Bas.: Marchantia triandra Scop., Fl. Carniol. (ed. 2) 2: 354, 1772 (Scopoli 1772).

***Incertae sedis***

* Mannia paradoxa R.M.Schust., Phytologia 57 (6): 410, 1985 (Schuster 1985b). ^434^

*** **Plagiochasma Lehm.**, Nov. Stirp. Pug. 4: 13, 1832 (Lehmann 1832) nom. conserv.

* Plagiochasma megacarpon (Griff.) Steph., Bull. Herb. Boissier 6 (10): 789 (86), 1898 (Stephani 1898c). Bas.: Antrocephalus megacarpon Griff., Not. pl. asiat. 2: 338, 1849 (Griffith 1849). ^435^

** **subg. Micropylum Bischl.**, Rev. Bryol. Lichénol. 43 (1): 103, 1977 (Bischler 1977).

*** Plagiochasma rupestre (J.R.Forst. et G.Forst.) Steph., Bull. Herb. Boissier 6 (10): 783 (80), 1898 (Stephani 1898c). Bas.: Aytonia rupestris J.R.Forst. et G.Forst., Char. gen. pl., ed. 2: 148, 1776 (Forster and Forster 1776).

*** Plagiochasma rupestre var. volkii Bischl., Rev. Bryol. Lichénol. 44 (3): 289, 1978 (Bischler 1978).

** **subg. Plagiochasma**

*** Plagiochasma appendiculatum Lehm. et Lindenb., Nov. Stirp. Pug. 4: 14, 1832 (Lehmann 1832).

*** Plagiochasma argentinicum Bischl., Rev. Bryol. Lichénol. 45 (3): 301, 1979 (Bischler 1979a).

*** Plagiochasma beccarianum Steph., Bull. Herb. Boissier 6 (10): 781 (78), 1898 (Stephani 1898c).

*** Plagiochasma cordatum Lehm. et Lindenb., Nov. Stirp. Pug. 4: 13, 1832 (Lehmann 1832).

*** Plagiochasma crenulatum Gottsche, Mexik. Leverm.: 266, 1863 (Gottsche 1863).

*** Plagiochasma cuneatum A.Evans, Amer. J. Bot. 19 (7): 627, 1932 (Evans 1932a).

*** Plagiochasma eximium (Schiffn.) Steph., Bull. Herb. Boissier 6 (10): 781 (78), 1898 (Stephani 1898c). Bas.: Aytonia eximia Schiffn., Bot. Jahrb. Syst. 20 (3): 300, 1895 (Stephani 1895a).

*** Plagiochasma intermedium Lindenb. et Gottsche, Syn. Hepat. 4: 513, 1846 (Gottsche et al. 1846).

*** Plagiochasma jamaicense (Haynes) A.Evans, Bull. Torrey Bot. Club 42 (5): 292, 1915 (Evans 1915). Bas.: Aytonia jamaicensis Haynes, Bull. Torrey Bot. Club 34 (2): 58, 1907 (Haynes 1907).

*** Plagiochasma japonicum (Steph.) C.Massal., Hepat. Shen-si: 47, 1897 (Massalongo 1897). Bas.: Aytonia japonica Steph., Bull. Herb. Boissier 5 (2): 84, 1897 (Stephani 1897b).

*** Plagiochasma landii A.Evans, Bull. Torrey Bot. Club 42 (5): 298, 1915 (Evans 1915).

*** Plagiochasma microcephalum (Steph.) Steph., Bull. Herb. Boissier 6 (10): 781 (78), 1898 (Stephani 1898c). Bas.: Aytonia microcephala Steph., Bot. Jahrb. Syst. 20 (3): 301, 1895 (Stephani 1895a).

*** Plagiochasma microcephalum var. tunesicum Bischl., Rev. Bryol. Lichénol. 44 (3): 247, 1978 (Bischler 1978).

*** Plagiochasma muenchianum Steph., Sp. Hepat. (Stephani) 6: 9, 1917 (Stephani 1917a).

*** Plagiochasma pterospermum C.Massal., Hepat. Shen-si: 46, 1897 (Massalongo 1897).

*** Plagiochasma wrightii Sull., Musc. Hepat. U.S.: 688, 1856 (Sullivant 1856).

***Incertae sedis***

** Plagiochasma udarii A.Alam et S.C.Srivast., Indian J. Forest. 32 (4): 631, 2009 (Alam and Srivastava 2009).

*** **Reboulia Raddi**, Opusc. Sci. 2 (6): 357, 1818 (Raddi 1818b) nom. conserv.

*** Reboulia hemisphaerica (L.) Raddi, Opusc. Sci. 2 (6): 357, 1818 (Raddi 1818b). Bas.: Marchantia hemisphaerica L., Sp. Pl. 1: 1138, 1753 (Linnaeus 1753).

** Reboulia hemisphaerica subsp. acrogyna (R.M.Schust.) R.M.Schust., Hepat. Anthocerotae N. Amer. 6: 168, 1992 (Schuster 1992d). Bas.: Asterella bolanderi subsp. acrogyna R.M.Schust., Phytologia 57 (6): 410, 1985 (Schuster 1985b).

** Reboulia hemisphaerica subsp. australis R.M.Schust., Phytologia 56 (7): 460, 1985 (Schuster 1985c).

** Reboulia hemisphaerica subsp. dioica R.M.Schust., Phytologia 56 (7): 462, 1985 (Schuster 1985c).

** Reboulia hemisphaerica var. fissisquama Herzog, Symb. Sin. 5: 5, 1930 (Nicholson et al. 1930).

** Reboulia hemisphaerica subsp. orientalis R.M.Schust., Phytologia 56 (7): 461, 1985 (Schuster 1985c).

** Reboulia hemisphaerica var. turkestanica C.E.O.Jensen ex Herzog, Symb. Sin. 5: 5, 1930 (Nicholson et al. 1930).

####### *** Cleveaceae Cavers

by D.G. Long

The genera of Cleveaceae were re-defined by Rubasinghe et al. (2011a) based on molecular evidence.

*** **Athalamia Falc.**, Ann. Mag. Nat. Hist. (ser. 2) 1 (5): 375, 1848 (Anonymous 1848).

* Athalamia dioica Kashyap, J. Bombay Nat. Hist. Soc. 24 (2): 348, 1916 (Kashyap 1916).

*** Athalamia pinguis Falc., Ann. Mag. Nat. Hist. (ser. 2) 1 (5): 375, 1848 (Anonymous 1848).

* Athalamia pulcherrima (Steph.) S.Hatt., J. Hattori Bot. Lab. 12: 54, 1954 (Shimizu and Hattori 1954). Bas.: Clevea pulcherrima Steph., Bot. Jahrb. Syst. 20 (3): 303, 1895 (Stephani 1895a).

*** **Clevea Lindb.**, Not. Sällsk. Fauna Fl. Fenn. Förh. 9: 289, 1868 (Lindberg 1868b).

*** Clevea hyalina (Sommerf.) Lindb., Not. Sällsk. Fauna Fl. Fenn. Förh. 9: 291, 1868 (Lindberg 1868b). Bas.: Marchantia hyalina Sommerf., Mag. Naturvidensk. 11 (2): 234, 1833 (Sommerfeldt 1833).

* Clevea hyalina var. californica M.Howe, Mem. Torrey Bot. Club 7: 38, 1899 (Howe 1899).

* Clevea pedicellata (Griff.) Lindb., Acta Soc. Fauna Fl. Fenn. 2 (3): 11, 1882 (Lindberg 1882). Bas.: Plagiochasma pedicellatum Griff., Not. pl. asiat. 2: 331, 1849 (Griffith 1849).

*** Clevea pusilla (Steph.) Rubas. et D.G.Long, J. Bryol. 33 (2): 167, 2011 (Rubasinghe et al. 2011b). Bas.: Gollaniella pusilla Steph., Hedwigia 44 (2): 74, 1905 (Stephani 1905h).

*** Clevea spathysii (Lindenb.) Müll.Frib., Hedwigia 79 (1/2): 75, 1940 (Müller 1940). Bas.: Marchantia spathysii Lindenb., Syn. hepat. eur: 104, 1829 (Lindenberg 1829).

*** **Peltolepis Lindb.**, Morgonbladet (Helsinki) 1876 (106, 9 May): 1, 1876 (Elfving 1876).

** Peltolepis japonica (Shimizu et S.Hatt.) S.Hatt., J. Hattori Bot. Lab. 14: 103, 1955 (Shimizu and Hattori 1955). Bas.: Peltolepis quadrata var. japonica Shimizu et S.Hatt., J. Hattori Bot. Lab. 12: 69, 1954 (Shimizu and Hattori 1954).

*** Peltolepis quadrata (Saut.) Müll.Frib., Hedwigia 79 (1/2): 74, 1940 (Müller 1940). Bas.: Sauteria quadrata Saut., Flora 43 (22): 351, 1860 (Sauter 1860).

*** **Sauteria Nees**, Naturgesch. Eur. Leberm. 4: 139, 1838 (Nees 1838a).

** **sect. Sauchia (Kashyap) R.M.Schust.**, Phytologia 57 (6): 411, 1985 (Schuster 1985b). Bas.: Sauchia Kashyap, J. Bombay Nat. Hist. Soc. 24 (2): 347, 1916 (Kashyap 1916).

* Sauteria japonica (Shimizu et S.Hatt.) S.Hatt., J. Hattori Bot. Lab. 12: 62, 1954 (Shimizu and Hattori 1954). Bas.: Sauchia japonica Shimizu et S.Hatt., J. Hattori Bot. Lab. 9: 32, 1953 (Shimizu and Hattori 1953a). ^436^

*** Sauteria spongiosa (Kashyap) S.Hatt., J. Hattori Bot. Lab. 12: 62, 1954 (Shimizu and Hattori 1954). Bas.: Sauchia spongiosa Kashyap, J. Bombay Nat. Hist. Soc. 24 (2): 347, 1916 (Kashyap 1916).

** **sect. Sauteria**

*** Sauteria alpina (Nees) Nees, Naturgesch. Eur. Leberm. 4: 143, 1838 (Nees 1838a). Bas.: Lunularia alpina Nees, Flora 13 (25): 399, 1830 (Nees and Bischoff 1830).

* Sauteria inflata C.Gao et K.C.Chang, Acta Bot. Yunnan. 3 (4): 389, 1981 (Gao et al. 1981). ^437^

***Incertae sedis***

* Sauteria chilensis (Lindenb.) Grolle, J. Hattori Bot. Lab. 58: 200, 1985 (Grolle 1985b). Bas.: Grimaldia chilensis Lindenb., Voy. Amér. Mérid. 7 (2): 53, 1839 (Montagne 1839a).

* Sauteria crassipes Austin, Proc. Acad. Nat. Sci. Philadelphia 21: 229, 1869 (Austin 1869).

* Sauteria nyikaensis Perold, Bothalia 33 (2): 167, 2003 (Perold 2003).

####### *** Conocephalaceae Müll.Frib. ex Grolle

by D.G. Long

*** **Conocephalum Hill**, Gener. Nat. Hist. 2 Hist. pl. (ed. 2): 118, 1773 (Hill 1773) nom. conserv.

** **subg. Conocephalum**

*** Conocephalum conicum (L.) Dumort., Commentat. Bot. (Dumortier): 115, 1822 (Dumortier 1822). Bas.: Marchantia conica L., Sp. Pl. 1: 1138, 1753 (Linnaeus 1753).

*** Conocephalum salebrosum Szweyk., Buczk. et Odrzyk., Pl. Syst. Evol. 253 (1/4): 146, 2005 (Szweykowski et al. 2005).

** **subg. Sandea (Lindb.) Inoue**, Ill. Jap. Hep. 2: 192, 1976 (Inoue 1976a). Bas.: Sandea Lindb., Acta Soc. Fauna Fl. Fenn. 2 (5): 3, 1884 (Lindberg 1884).

*** Conocephalum japonicum (Thunb.) Grolle, J. Hattori Bot. Lab. 55: 501, 1984 (Grolle 1984a). Bas.: Lichen japonicus Thunb., Fl. Jap. (Thunberg): 344, 1784 (Thunberg 1784).

####### ** Corsiniaceae Engl.

by D.G. Long

######## ** Corsinioideae Schiffn.

*** **Corsinia Raddi**, Opusc. Sci. 2 (6): 354, 1818 (Raddi 1818b).

*** Corsinia coriandrina (Spreng.) Lindb., Hepaticol. Utveckl.: 30, 1877 (Lindberg 1877c). Bas.: Riccia coriandrina Spreng. Anleit. Kenntn. Gew. 3: 320, 1804 (Sprengel 1804).

######## *** Cronisioideae R.M.Schust.

*** **Cronisia Berk.**, Introd. crypt. bot.: 434, 1857 (Berkeley 1857).

*** Cronisia fimbriata (Nees) Whittem. et Bischl., Cryptog. Bryol. 22 (3): 170, 2001 (Bischler and Whittemore 2001). Bas.: Riccia fimbriata Nees, Fl. Bras. (Martius) 1 (1): 301, 1833 (Nees 1833a).

*** Cronisia weddellii (Mont.) Grolle, J. Bryol. 9 (4): 532, 1977 [1978] (Grolle 1977a). Bas.: Boschia weddellii Mont., Ann. Sci. Nat. Bot. (sér. 4) 5: 352, 1856 (Montagne 1856c).

####### *** Cyathodiaceae Stotler et Crand.-Stotl.

by D.G. Long

*** **Cyathodium Kunze**, Nov. Stirp. Pug. 6: 17, 1834 (Lehmann 1834).

*** Cyathodium aureonitens (Griff.) Mitten, J. Linn. Soc., Bot. 22 (146): 327 (Mitten 1886b). Bas.: Synhymenium aureonitens Griff., Not. pl. asiat. 2: 344, 1849 (Griffith 1849).

*** Cyathodium bischlerianum N.Salazar, Bryologist 104 (1): 141, 2001 (Salazar 2001).

*** Cyathodium cavernarum Kunze, Nov. Stirp. Pug. 6: 18, 1834 (Lehmann 1834).

** Cyathodium denticulatum Udar et S.C.Srivast., Geophytology 1 (2): 166, 1971 (Udar and Srivastava 1971).

*** Cyathodium foetidissimum Schiffn., Denkschr. Kaiserl. Akad. Wiss., Math.-Naturwiss. Kl. 67: 154, 1898 (Schiffner 1898a).

*** Cyathodium indicum Udar et D.K.Singh, J. Bryol. 10 (2): 139, 1978 [1979] (Udar and Singh 1978).

*** Cyathodium mehranum D.K.Singh, Misc. Bryol. Lichenol. 9 (8): 173, 1983 (Singh 1983b).

*** Cyathodium smaragdinum Schiffn., Ann. Jard. Bot. Buitenzorg, suppl. 3: 480, 1910 (Schiffner 1910c).

*** Cyathodium spruceanum Prosk., Bryologist 54 (4): 243, 1951 [1952] (Proskauer 1951b).

* Cyathodium spurium (Dicks.) Lindb. ex Braithw., J. Bot. 16: 55, 1878 (Braithwaite 1878). Bas.: Riccia spuria Dicks., Fasc. Pl. Crypt. Brit. 4: 20, 1801 (Dickson 1801). ^438^

*** Cyathodium steerei Hässel, Rev. Bryol. Lichénol. 30 (3/4): 223, 1961 (Hässel 1961).

*** Cyathodium tuberculatum Udar et D.K.Singh, Bryologist 79 (2): 235, 1976 (Udar and Singh 1976).

*** Cyathodium tuberosum Kashyap, New Phytol. 13 (6/7): 210, 1914 (Kashyap 1914a).

####### *** Dumortieraceae D.G.Long

by D.G. Long

*** **Dumortiera Nees**, Nova Acta Phys.-Med. Acad. Caes. Leop.-Carol. Nat. Cur. 12 (1): 410, 1824 [1825] (Reinwardt et al. 1824b).

*** Dumortiera hirsuta (Sw.) Nees, Nova Acta Phys.-Med. Acad. Caes. Leop.-Carol. Nat. Cur. 12 (1): 410, 1824 [1825] (Reinwardt et al. 1824b). Bas.: Marchantia hirsuta Sw., Prodr. (Swartz): 145, 1788 (Swartz 1788). ^439^

* Dumortiera hirsuta subsp. nepalensis (Taylor) R.M.Schust., Hepat. Anthocerotae N. Amer. 6: 386, 1992 (Schuster 1992d). Bas.: Hygrophila nepalensis Taylor, Trans. Linn. Soc. London 17 (3): 392, 1836 (Taylor 1836b).

* Dumortiera hirsuta subsp. tatunoi Horik., J. Sci. Hiroshima Univ., Ser. B, Div. 2, Bot. 6: 38, 1951 (Horikawa 1951a).

####### *** Exormothecaceae Müll.Frib. ex Grolle

by D.G. Long

*** **Aitchisoniella Kashyap**, New Phytol. 13 (6/7): 219, 1914 (Kashyap 1914a).

*** Aitchisoniella himalayensis Kashyap, New Phytol. 13 (6/7): 219, 1914 (Kashyap 1914a).

*** **Exormotheca Mitt.**, Nat. hist. Azores: 325, 1870 (Mitten 1870).

** **subg. Corbierella (Douin et Trab.) Schiffn.**, Hedwigia 81 (1/2): 71, 1942 (Schiffner 1942). Bas.: Corbierella Douin et Trab., Rev. Gén. Bot. 31: 326, 1919 (Douin and Trabut 1919).

*** Exormotheca bischlerae Furuki et Higuchi, Cryptog. Bryol. 27 (1): 98, 2006 (Furuki and Higuchi 2006).

*** Exormotheca holstii Steph., Bull. Herb. Boissier 7 (3): 219 (145), 1899 (Stephani 1899b).

*** Exormotheca welwitschii Steph., Bull. Herb. Boissier 7 (3): 220 (146), 1899 (Stephani 1899b).

** **subg. Exormotheca**

*** Exormotheca pustulosa Mitt., Nat. hist. Azores: 326, 1870 (Mitten 1870).

***Incertae sedis***

*** Exormotheca bulbigena Bornefeld, O.H.Volk et R.Wolf, Bothalia 26 (2): 159, 1996 (Bornefeld et al. 1996).

*** Exormotheca ceylonensis Meijer, J. Hattori Bot. Lab. 16: 72, 1956 (Meijer 1956).

* Exormotheca gollanii Steph., Sp. Hepat. (Stephani) 6: 18, 1917 (Stephani 1917a).

*** Exormotheca tuberifera Kashyap, New Phytol. 13 (9): 309, 1914 (Kashyap 1914b).

*** **Stephensoniella Kashyap**, New Phytol. 13 (9): 312, 1914 (Kashyap 1914b).

*** Stephensoniella brevipedunculata Kashyap, New Phytol. 13 (9): 312, 1914 (Kashyap 1914b).

####### *** Marchantiaceae Lindl.

by D.G. Long

The treatment of Marchantiaceae is mainly following Bischler (1984, 1989b) and Bischler-Causse (1993).

####### ** Bucegioideae R.M.Schust.

*** **Bucegia Radian**, Bull. Herb. Inst. Bot. Bucarest 3-4: 3, 1903 (Radian 1903).

*** Bucegia romanica Radian, Bull. Herb. Inst. Bot. Bucarest 3-4: 4, 1903 (Radian 1903).

####### ** Marchantioideae Schiffn.

*** **Marchantia L.**, Sp. Pl. 1: 1137, 1753 (Linnaeus 1753).

** **subg. Chlamidium (Corda) Bischl.**, Cryptog. Bryol. Lichénol. 3 (4): 362, 1982 (Bischler 1982). Bas.: Chlamidium Corda, Gen. hepat.: 647, 1829 (Corda 1829).

*** Marchantia breviloba A.Evans, Trans. Connecticut Acad. Arts 21 (3): 265, 1917 (Evans 1917a).

*** Marchantia inflexa Nees et Mont., Ann. Sci. Nat. Bot. (sér. 2) 9: 43, 1838 (Montagne 1838).

** **sect. Chlamidium (Corda) Nees**, Naturgesch. Eur. Leberm. 4: 101, 1838 (Nees 1838a). Bas.: Chlamidium Corda, Gen. hepat.: 647, 1829 (Corda 1829).

*** Marchantia chenopoda L., Sp. Pl. 1: 1137, 1753 (Linnaeus 1753).

*** Marchantia crenata Austin, Bull. Torrey Bot. Club 5 (3): 14, 1874 (Austin 1874).

*** Marchantia foliacea Mitt., Bot. antarct. voy. II (Fl. Nov.-Zel. 2): 168, 1855 (Mitten 1855).

*** Marchantia formosana Horik., J. Sci. Hiroshima Univ., Ser. B, Div. 2, Bot. 2: 121, 1934 (Horikawa 1934).

*** Marchantia globosa Brid., Hist. Musc. Hepat. Prodr.: 102, 1815 (Weber 1815).

*** Marchantia hexaptera Reichardt, Verh. K.K. Zool.-Bot. Ges. Wien 16: 957, 1866 (Reichardt 1866).

*** Marchantia linearis Lehm. et Lindenb., Nov. Stirp. Pug. 4: 8, 1832 (Lehmann 1832).

*** Marchantia miqueliana Lehm., Nov. Stirp. Pug. 10: 20, 1857 (Lehmann 1857).

*** Marchantia novoguineensis Bischl., Bryophyt. Biblioth. 38: 130, 1989 (Bischler 1989b).

*** Marchantia pappeana Lehm., Nov. Stirp. Pug. 10: 21, 1857 (Lehmann 1857).

** Marchantia pappeana subsp. robusta (Steph.) Bischl., Bryophyt. Biblioth. 45: 91, 1993 (Bischler-Causse 1993). Bas.: Marchantia robusta Steph., Candollea 14: 111, 1953 (Bonner 1953b).

*** Marchantia pileata Mitt., Bot. antarct. voy. II (Fl. Nov.-Zel. 2): 169, 1855 (Mitten 1855).

*** Marchantia pinnata Steph., Candollea 14: 109, 1953 (Bonner 1953b).

*** Marchantia rubribarba Steph., Bull. Herb. Boissier 7 (5): 400 (172), 1899 (Stephani 1899c).

*** Marchantia vitiensis Steph., Bull. Herb. Boissier 7 (7): 520 (182), 1899 (Stephani 1899d).

** **sect. Paleaceae Bischl.**, Bryophyt. Biblioth. 38: 90, 1989 (Bischler 1989b).

*** Marchantia paleacea Bertol., Opusc. Sci. 1: 242, 1817 (Bertoloni 1817).

*** Marchantia paleacea subsp. diptera (Nees et Mont.) Inoue, J. Jap. Bot. 64 (7): 194, 1989 (Inoue 1989c). Bas.: Marchantia diptera Nees et Mont., Ann. Sci. Nat. Bot. (sér. 2) 19: 243, 1843 (Montagne 1843).

** **sect. Papillatae Bischl.**, Cryptog. Bryol. Lichénol. 10 (1): 69, 1989 (Bischler 1989a).

*** Marchantia debilis K.I.Goebel, Organogr. Pfl., ed. 2, 2 (1): 901, 1915 (Goebel 1915).

*** Marchantia emarginata Reinw., Blume et Nees, Nova Acta Phys.-Med. Acad. Caes. Leop.-Carol. Nat. Cur. 12 (1): 192, 1824 [1825] (Reinwardt et al. 1824a).

*** Marchantia emarginata subsp. lecordiana (Steph.) Bischl., Cryptog. Bryol. Lichénol. 10 (1): 78, 1989 (Bischler 1989a). Bas.: Marchantia lecordiana Steph., Bull. Herb. Boissier 7 (7): 525 (187), 1899 (Stephani 1899d).

*** Marchantia emarginata subsp. tosana (Steph.) Bischl., Cryptog. Bryol. Lichénol. 10 (1): 77, 1989 (Bischler 1989a). Bas.: Marchantia tosana Steph., Bull. Herb. Boissier 5 (2): 99, 1897 (Stephani 1897b).

*** Marchantia papillata Raddi, Critt. Brasil.: 20, 1822 (Raddi 1822).

*** Marchantia papillata subsp. grossibarba (Steph.) Bischl., Cryptog. Bryol. Lichénol. 10 (1): 78, 1989 (Bischler 1989a). Bas.: Marchantia grossibarba Steph., Mém. Soc. Nat. Sci. Nat. Math. Cherbourg 29: 221, 1894 (Stephani 1894b).

** **subg. Marchantia**

*** Marchantia plicata Nees et Mont., Ann. Sci. Nat. Bot. (sér. 2) 9: 43, 1838 (Montagne 1838).

** **sect. Berteroanae R.M.Schust.**, Hepat. Anthocerotae N. Amer. 6: 310, 1992 (Schuster 1992d).

*** Marchantia berteroana Lehm. et Lindenb., Nov. Stirp. Pug. 6: 21, 1834 (Lehmann 1834).

** **sect. Marchantia**

*** Marchantia polymorpha L., Sp. Pl. 1: 1137, 1753 (Linnaeus 1753).

*** Marchantia polymorpha subsp. montivagans Bischl. et Boissel.-Dub., J. Bryol. 16 (3): 364, 1991 (Bischler-Causse and Boisselier-Dubayle 1991).

*** Marchantia polymorpha subsp. ruderalis Bischl. et Boissel.-Dub., J. Bryol. 16 (3): 364, 1991 (Bischler-Causse and Boisselier-Dubayle 1991).

** **subg. Protomarchantia R.M.Schust.**, Phytologia 57 (6): 410, 1985 (Schuster 1985b).

** **sect. Protomarchantia (R.M.Schust.) L.Söderstr.**, Phytotaxa 202 (1): 69, 2015 (Söderström et al. 2015c). Bas.: Marchantia subg. Protomarchantia R.M.Schust., Phytologia 57 (6): 410, 1985 (Schuster 1985b).

*** Marchantia acaulis Steph., Bull. Herb. Boissier 7 (7): 533 (195), 1899 (Stephani 1899d).

*** Marchantia antiqua Steph., Candollea 14: 103, 1953 (Bonner 1953b).

*** Marchantia carrii Bischl., Bryophyt. Biblioth. 38: 256, 1989 (Bischler 1989b).

*** Marchantia geminata Reinw., Blume et Nees, Nova Acta Phys.-Med. Acad. Caes. Leop.-Carol. Nat. Cur. 12 (1): 194, 1824 [1825] (Reinwardt et al. 1824a).

*** Marchantia hartlessiana Steph., Candollea 14: 107, 1953 (Bonner 1953b).

*** Marchantia macropora Mitt., Bot. antarct. voy. II (Fl. Nov.-Zel. 2): 169, 1855 (Mitten 1855).

*** Marchantia philippinensis Bischl., Bryophyt. Biblioth. 38: 245, 1989 (Bischler 1989b).

*** Marchantia solomonensis Bischl., Bryophyt. Biblioth. 38: 281, 1989 (Bischler 1989b).

*** Marchantia streimannii Bischl., Bryophyt. Biblioth. 38: 250, 1989 (Bischler 1989b).

*** Marchantia subintegra Mitt., J. Proc. Linn. Soc., Bot. 5 (18): 125, 1860 [1861] (Mitten 1860c).

*** Marchantia treubii Schiffn., Denkschr. Kaiserl. Akad. Wiss., Math.-Naturwiss. Kl. 67: 160, 1898 (Schiffner 1898a).

*** Marchantia wallisii J.B.Jack et Steph., Bull. Herb. Boissier 7 (7): 520 (182), 1899 (Stephani 1899d).

** **sect. Subgeminatae Bischl.**, Bryophyt. Biblioth. 38: 219, 1989 (Bischler 1989b).

*** Marchantia subgeminata Steph., Bull. Herb. Boissier 7 (7): 530 (192), 1899 (Stephani 1899d).

***Incertae sedis***

* Marchantia assamica Griff., Not. pl. asiat. 2: 327, 1849 (Griffith 1849). ^440^

* Marchantia balboi Gola, Mem. Reale Accad. Sci. Torino (ser. 2) 65 (1): 2, 1916 (Gola 1916). ^441^

* Marchantia balboi var. acutisquamata Gerola, Lav. Bot. Ist. Bot. Univ. Padova 12: 472, 1947 (Gerola 1947). ^442^

* Marchantia cagnii Gola, Ann. Bot. (Rome) 6 (2): 271, 1907 (Gola 1907). ^443^

* Marchantia cengiana Gerola, Lav. Bot. Ist. Bot. Univ. Padova 12: 473, 1947 (Gerola 1947). ^444^

* Marchantia friedrichsthaliana Trevis., Mem. Reale Ist. Lombardo Sci. (Ser. 3), C. Sci. Mat. 4 (13): 438, 1877 (Trevisan 1877).

* Marchantia keniae Gola, Mem. Reale Accad. Sci. Torino (ser. 2) 65 (1): 3, 1916 (Gola 1916). ^445^

* Marchantia papyracea Gola, Ann. Bot. (Rome) 6 (2): 271, 1907 (Gola 1907). ^446^

* Marchantia quadriloba Steph., Candollea 14: 110, 1953 (Bonner 1953b).

* Marchantia sellae Gola, Ann. Bot. (Rome) 6 (2): 271, 1907 (Gola 1907). ^447^

** Marchantia stoloniscyphulus (C.Gao et K.C.Chang) Piippo, J. Hattori Bot. Lab. 68: 134, 1990 (Piippo 1990). Bas.: Marchantiopsis stoloniscyphulus C.Gao et K.C.Chang, Bull. Bot. Res., Harbin 2 (4): 114, 1982 (Gao and Chang 1982).

* Marchantia trilocularis Roth, Tent. Fl. Germ. 1: 487, 1788 (Roth 1788). ^448^

* Marchantia tusui Gola, Mem. Reale Accad. Sci. Torino (ser. 2) 65 (1): 3, 1916 (Gola 1916). ^449^

** **Preissia Corda**, Gen. hepat.: 647, 1829 (Corda 1829).

*** Preissia quadrata (Scop.) Nees, Naturgesch. Eur. Leberm. 4: 135, 1838 (Nees 1838a). Bas.: Marchantia quadrata Scop., Fl. Carniol. (ed. 2) 2: 355, 1772 (Scopoli 1772).

** Preissia quadrata subsp. hyperborea R.M.Schust., Phytologia 57 (6): 410, 1985 (Schuster 1985b).

####### *** Monocleaceae A.B.Frank

by D.G. Long

*** **Monoclea Hook.**, Musci Exot. 2: tab. clxxiv, 1820 (Hooker 1820).

*** Monoclea forsteri Hook., Musci Exot. 2: tab. clxxiv, 1820 (Hooker 1820).

*** Monoclea gottschei Lindb., Rev. Bryol. 13 (6): 102, 1886 (Lindberg 1886).

*** Monoclea gottschei subsp. elongata Gradst. et Mues, Pl. Syst. Evol. 180 (1/2): 133, 1992 (Gradstein et al. 1992).

####### *** Monosoleniaceae Inoue

by D.G.Long

*** **Monosolenium Griff.**, Not. pl. asiat. 2: 341, 1849 (Griffith 1849).

*** Monosolenium tenerum Griff., Not. pl. asiat. 2: 341, 1849 (Griffith 1849).

####### *** Oxymitraceae Müll.Frib. ex Grolle

by D.G. Long

*** **Oxymitra Bisch. ex Lindenb.**, Syn. hepat. eur: 124, 1829 (Lindenberg 1829).

*** Oxymitra cristata Garside, Bothalia 23 (2): 211, 1993 (Perold 1993). Based on: Oxymitra cristata Garside, J. S. African Bot. 24: 83, 1958 (Garside 1958), *nom. inval*.

*** Oxymitra incrassata (Brot.) Sérgio et Sim-Sim, J. Bryol. 15 (4): 662, 1989 (Sérgio and Sim-Sim 1989). Bas.: Riccia incrassata Brot., Fl. lusit. 2: 428, 1804 [1805] (Brotero 1804).

####### *** Ricciaceae Rchb.

by R. Stotler, B.J. Crandall-Stotler and D.C. Cargill

** **Riccia L.**, Sp. Pl. 1: 1138, 1753 (Linnaeus 1753) nom. conserv.

** **subg. Chartaceae Perold**, Bothalia 16 (1): 29, 1986 (Volk and Perold 1986c).

*** Riccia schelpei O.H.Volk et Perold, Bothalia 16 (1): 29, 1986 (Volk and Perold 1986c).

** **subg. Leptoriccia R.M.Schust.**, Phytologia 56 (2): 72, 1984 (Schuster 1984).

*** Riccia membranacea Gottsche et Lindenb., Syn. Hepat. 4: 608, 1846 (Gottsche et al. 1846).

** **subg. Riccia**

*** Riccia albida Sull. ex Austin, Proc. Acad. Nat. Sci. Philadelphia 21: 231, 1869 (Austin 1869).

*** Riccia albopunctata Jovet-Ast, Cryptog. Bryol. Lichénol. 12 (3): 237, 1991 (Jovet-Ast 1991).

*** Riccia australis Steph., Bull. Herb. Boissier 6 (4): 337 (29), 1898 (Stephani 1898a).

*** Riccia boliviensis Jovet-Ast, Cryptog. Bryol. Lichénol. 12 (3): 242, 1991 (Jovet-Ast 1991).

*** Riccia brasiliensis Schiffn., Österr. Akad. Wiss., Math.-Naturwiss. Kl., Denkschr. 111: 6, 1964 (Schiffner and Arnell 1964).

*** Riccia breutelii Hampe, Bull. Herb. Boissier 6 (4): 325 (17), 1898 (Stephani 1898a).

*** Riccia brittonii M.Howe, Ann. Missouri Bot. Gard. 2 (1/2): 50, 1915 (Britton 1915).

* Riccia chudoana Steph., Sp. Hepat. (Stephani) 6: 1, 1917 (Stephani 1917a).

*** Riccia coracina Jovet-Ast, Cryptog. Bryol. 24 (3): 212, 2003 (Jovet-Ast 2003).

*** Riccia corrugata Jovet-Ast, Cryptog. Bryol. 21 (4): 308, 2000 (Jovet-Ast 2000).

*** Riccia crassivenia Jovet-Ast, Cryptog. Bryol. 21 (4): 312, 2000 (Jovet-Ast 2000).

*** Riccia cubensis S.W.Arnell, Bryologist 61 (2): 142, 1958 (Arnell 1958c).

*** Riccia discolor Lehm. et Lindenb., Nov. Stirp. Pug. 4: 1, 1832 (Lehmann 1832).

*** Riccia ekmanii S.W.Arnell, Bryologist 61 (2): 143, 1958 (Arnell 1958c).

*** Riccia elliottii Steph., Bull. Herb. Boissier 6 (4): 324 (16), 1898 (Stephani 1898a).

*** Riccia enyae Jovet-Ast, Cryptog. Bryol. Lichénol. 12 (3): 230, 1991 (Jovet-Ast 1991).

*** Riccia erythrocarpa Jovet-Ast, Cryptog. Bryol. Lichénol. 12 (3): 257, 1991 (Jovet-Ast 1991).

*** Riccia fruchartii Steph., Bull. Herb. Boissier 6 (4): 330 (22), 1898 (Stephani 1898a).

*** Riccia gangetica Ahmad ex L.Söderstr., A.Hagborg et von Konrat, Phytotaxa 65: 57, 2012 (Söderström et al. 2012f). Based on: Riccia gangetica Ahmad, Curr. Sci. 11 (11): 433, 1942 (Ahmad 1942), *nom. inval*.

*** Riccia grandis Nees, Fl. Bras. (Martius) 1 (1): 300, 1833 (Nees 1833a).

*** Riccia helenae Jovet-Ast, J. Hattori Bot. Lab. 74: 96, 1993 (Jovet-Ast 1993a).

** Riccia hirta (Austin) Underw., Bot. Gaz. 19 (7): 274, 1894 (Underwood 1894). Bas.: Riccia arvensis var. hirta Austin, Proc. Acad. Nat. Sci. Philadelphia 21: 232, 1869 (Austin 1869).

*** Riccia horrida Jovet-Ast, Cryptog. Bryol. Lichénol. 12 (3): 226, 1991 (Jovet-Ast 1991).

*** Riccia hortorum Bory, Nova Acta Phys.-Med. Acad. Caes. Leop.-Carol. Nat. Cur. 18 (1): 435, 1836 [1837] (Lindenberg 1836).

*** Riccia howellii M.Howe, Proc. Calif. Acad. Sci. (ser. 4) 21 (17): 202, 1934 (Howe 1934).

* Riccia ianthina Jovet-Ast, Rev. Bryol. Lichénol. 44 (4): 418, 1978 (Jovet-Ast 1978). ^450^

*** Riccia inflexa Taylor, London J. Bot. 5: 417, 1846 (Taylor 1846b).

*** Riccia iodocheila M.Howe, Proc. Calif. Acad. Sci. (ser. 4) 21 (17): 200, 1934 (Howe 1934).

*** Riccia lanceolata Steph., Hedwigia 27 (3/4): 110, 1888 (Stephani 1888d).

*** Riccia lindmanii Steph., Bih. Kongl. Svenska Vetensk.-Akad. Handl. 23 (III, 2): 29, 1897 (Stephani 1897a).

** Riccia macallisteri M.Howe, Bryologist 20 (3): 35, 1917 (Howe 1917).

*** Riccia macrospora Steph., Bull. Herb. Boissier 6 (4): 328 (20), 1898 (Stephani 1898a).

** Riccia mamillata Trab. ex Steph., Rev. Bryol. 16 (5): 65, 1889 (Stephani 1889b).

*** Riccia mauryana Steph., Bull. Herb. Boissier 6 (4): 327 (19), 1898 (Stephani 1898a).

*** Riccia olgensis Na-Thalang, Brunonia 3 (1): 100, 1980 (Na-Thalang 1980).

*** Riccia planobiconvexa Steph., Bih. Kongl. Svenska Vetensk.-Akad. Handl. 23 (III, 2): 29, 1897 (Stephani 1897a).

*** Riccia ridleyi A.Gepp, J. Linn. Soc., Bot. 27 (181): 74, 1890 (Gepp 1890).

*** Riccia sanguineisporis Jovet-Ast, Cryptog. Bryol. Lichénol. 12 (3): 253, 1991 (Jovet-Ast 1991).

*** Riccia squamata Nees, Fl. Bras. (Martius) 1 (1): 302, 1833 (Nees 1833a).

*** Riccia subdepilata Jovet-Ast, Cryptog. Bryol. Lichénol. 12 (3): 228, 1991 (Jovet-Ast 1991).

*** Riccia subplana Steph., Symb. Antill. (Urban) 3 (2): 275, 1902 (Stephani 1902e).

*** Riccia taeniiformis Jovet-Ast, Cryptog. Bryol. Lichénol. 12 (3): 270, 1991 (Jovet-Ast 1991).

*** Riccia viannae Jovet-Ast, Cryptog. Bryol. Lichénol. 12 (3): 261, 1991 (Jovet-Ast 1991).

*** Riccia vitalii Jovet-Ast, Mem. New York Bot. Gard. 45: 285, 1987 (Jovet-Ast 1987).

*** Riccia weinionis Steph., Bull. Herb. Boissier 6 (4): 326 (18), 1898 (Stephani 1898a).

** **sect. Pilifer O.H.Volk**, Mitt. Bot. Staatssamml. München 19: 455, 1983 (Volk 1983).

*** Riccia alatospora O.H.Volk et Perold, Bothalia 15 (3/4): 534, 1985 (Volk and Perold 1985).

*** Riccia albomarginata Bisch. ex C.Krauss, Flora 29 (9): 135, 1846 (Krauss 1846).

*** Riccia albovestita O.H.Volk, Mitt. Bot. Staatssamml. München 17: 245, 1981 (Volk 1981).

*** Riccia ampullacea Perold, Bothalia 20 (2): 168, 1990 (Perold 1990e).

*** Riccia concava Bisch. ex C.Krauss, Flora 29 (9): 135, 1846 (Krauss 1846).

*** Riccia elongata Perold, Bothalia 20 (2): 167, 1990 (Perold 1990e).

*** Riccia furfuracea Perold, Bothalia 20 (2): 176, 1990 (Perold 1990b).

*** Riccia hantamensis Perold, Bothalia 19 (2): 157, 1989 (Perold 1989b).

*** Riccia hirsuta O.H.Volk et Perold, Bothalia 16 (2): 187, 1986 (Volk and Perold 1986a).

*** Riccia namaquensis Perold, Bothalia 20 (2): 180, 1990 (Perold 1990b).

*** Riccia parvoareolata O.H.Volk et Perold, Bothalia 15 (1/2): 117, 1984 (Volk and Perold 1984).

*** Riccia pulveracea Perold, Bothalia 20 (2): 185, 1990 (Perold 1990c).

*** Riccia radicosa Pearson, Natuurw. Tijdschr. 4 (5/6): 142, 1922 (Pearson 1922a).

*** Riccia simii Perold, Bothalia 20 (1): 36, 1990 (Perold 1990a).

*** Riccia trachyglossa Perold, Bothalia 20 (2): 172, 1990 (Perold 1990e).

*** Riccia villosa Steph., Akad. Wiss. Wien, Math.-Naturwiss. Kl., Denkschr. 88: 724, 1913 (Stephani 1913b).

*** Riccia vitrea Perold, Bothalia 20 (2): 178, 1990 (Perold 1990b).

** **sect. Riccia**

*** Riccia albolimbata S.W.Arnell, Mitt. Bot. Staatssamml. München 2 (16): 264, 1957 (Arnell 1957c).

*** Riccia alboporosa Perold, Bothalia 19 (1): 12, 1989 (Perold 1989c).

*** Riccia albornata O.H.Volk et Perold, Bothalia 18 (2): 160, 1988 (Volk et al. 1988).

*** Riccia angolensis Steph., Bull. Herb. Boissier 6 (4): 323 (15), 1898 (Stephani 1898a).

*** Riccia argenteolimbata O.H.Volk et Perold, Bothalia 18 (2): 155, 1988 (Volk et al. 1988).

** Riccia atlantica Sérgio et Perold, J. Bryol. 17 (1): 127, 1992 (Sérgio and Perold 1992).

*** Riccia atromarginata Levier, Nuovo Giorn. Bot. Ital. 21 (2): 291, 1889 (Martelli 1889).

** Riccia atromarginata var. jovet-astiae Rauh et Buchloh, Rev. Bryol. Lichénol. 30 (1/2): 77, 1961 (Rauh and Buchloh 1961).

*** Riccia atropurpurea Sim, Trans. Roy. Soc. South Africa 15 (1): 11, 1926 (Sim 1926).

*** Riccia beyrichiana Hampe, Nov. Stirp. Pug. 7: 1, 1838 (Lehmann 1838).

*** Riccia bicarinata Lindb., Rev. Bryol. 4 (3): 41, 1877 (Lindberg 1877d).

*** Riccia bicolorata Perold, Bothalia 20 (2): 188, 1990 (Perold 1990c).

*** Riccia bifurca Hoffm., Deutschl. Fl., Theil 2 (Hoffm.): 95, 1795 [1796] (Hoffmann 1795).

*** Riccia billardierei Mont. et Nees, Syn. Hepat. 4: 602, 1846 (Gottsche et al. 1846).

*** Riccia breidleri Jur. ex Steph., Hedwigia 24 (1): 6, 1885 (Stephani 1885e).

*** Riccia californica Austin, Bull. Torrey Bot. Club 6 (7): 46, 1875 (Austin 1875c).

*** Riccia campbelliana M.Howe, Mem. Torrey Bot. Club 7: 26, 1899 (Howe 1899).

*** Riccia ciliata Hoffm., Deutschl. Fl., Theil 2 (Hoffm.): 95, 1795 [1796] (Hoffmann 1795).

*** Riccia ciliifera Link, Syn. hepat. eur: 119, 1829 (Lindenberg 1829).

* Riccia congoana Steph., Bull. Herb. Boissier 6 (4): 328 (20), 1898 (Stephani 1898a). ^451^

*** Riccia crinita Taylor, London J. Bot. 5: 415, 1846 (Taylor 1846b).

*** Riccia crozalsii Levier, Rev. Bryol. 29 (4): 73, 1902 (Levier 1902).

* Riccia crustata Trab., Bull. Soc. Hist. Nat. Afrique N. 7: 87, 1916 (Trabut 1916). ^452^

** Riccia dictyospora M.Howe, Bull. Torrey Bot. Club 28 (3): 163, 1901 (Howe 1901a).

*** Riccia glauca L., Sp. Pl. 1: 1139, 1753 (Linnaeus 1753).

** Riccia glauca var. ciliaris Warnst., Verh. Bot. Vereins Prov. Brandenburg 27 (1): 87, 1886 (Warnstorf 1886).

** Riccia gothica Damsh. et Hallingb., Lindbergia 12 (2/3): 100, 1986 [1987] (Damsholt and Hallingbäck 1986).

*** Riccia gougetiana Durieu et Mont., Ann. Sci. Nat. Bot. (sér. 3) 11: 35, 1849 (Montagne 1849).

** Riccia gougetiana var. armatissima Levier ex Müll.Frib., Lebermoose 1 (3): 161, 1907 (Müller 1907b).

*** Riccia lamellosa Raddi, Opusc. Sci. 2 (6): 351, 1818 (Raddi 1818b).

*** Riccia ligula Steph., Bull. Herb. Boissier 6 (4): 315 (7), 1898 (Stephani 1898a).

*** Riccia limbata Bisch. ex C.Krauss, Flora 29 (9): 135, 1846 (Krauss 1846).

*** Riccia macrocarpa Levier, Bull. Soc. Bot. Ital. 1894: 114, 1894 (Levier 1894).

*** Riccia mammifera O.H.Volk et Perold, Bothalia 16 (2): 176, 1986 (Volk and Perold 1986b).

*** Riccia melitensis C.Massal., Bull. Soc. Bot. Ital. 1913 (2/3): 52, 1913 (Massalongo 1913).

*** Riccia michelii Raddi, Opusc. Sci. 2 (6): 352, 1818 (Raddi 1818b).

*** Riccia microciliata O.H.Volk et Perold, Bothalia 16 (2): 173, 1986 (Volk and Perold 1986b).

*** Riccia montana Perold, Bothalia 19 (1): 9, 1989 (Perold 1989c).

*** Riccia natalensis Sim, Trans. Roy. Soc. South Africa 15 (1): 9, 1926 (Sim 1926).

*** Riccia nigrella DC., Fl. Franç. (DC. & Lamarck), 5 (6): 193, 1815 (De Candolle and Lamarck 1815).

*** Riccia okahandjana S.W.Arnell, Mitt. Bot. Staatssamml. München 2 (16): 268, 1957 (Arnell 1957c).

** Riccia ozarkiana McGregor, Bryologist 63 (1): 30, 1960 (McGregor 1960).

*** Riccia papillosa Moris, Stirp. Sard. Elench.: 18, 1829 (Moris 1829).

*** Riccia pottsiana Sim, Trans. Roy. Soc. South Africa 15 (1): 10, 1926 (Sim 1926).

*** Riccia rosea O.H.Volk et Perold, Bothalia 16 (2): 181, 1986 (Volk and Perold 1986d).

* Riccia runssorensis Steph., Bull. Herb. Boissier 6 (4): 330 (22), 1898 (Stephani 1898a). ^453^

*** Riccia sommieri Levier, Isola Giglio: 119, 1900 (Bottini et al. 1900).

*** Riccia sorocarpa Bisch., Bem. Leberm.: 145, 1835 (Bischoff 1835).

** Riccia sorocarpa var. heegii Schiffn., Hedwigia 53 (1/2): 36, 1912 (Schiffner 1912a).

*** Riccia subbifurca Warnst. ex Croz., Rev. Bryol. 30 (4): 62, 1903 (Crozals 1903b).

** Riccia tenella D.L.Jacobs, Bryologist 52 (4): 168, 1949 [1950] (Jacobs 1949).

*** Riccia trabutiana Steph., Rev. Bryol. 16 (5): 65, 1889 (Stephani 1889b).

*** Riccia violacea M.Howe, Ann. Missouri Bot. Gard. 2 (1/2): 51, 1915 (Britton 1915).

*** Riccia violacea var. laevis Jovet-Ast, Cryptog. Bryol. Lichénol. 10 (2): 100, 1989 (Jovet-Ast 1989).

*** Riccia warnstorfii Limpr. ex Warnst., Verh. Bot. Vereins Prov. Brandenburg 27 (1): 85, 1886 (Warnstorf 1886).

** **subg. Ricciella (A.Braun) Boulay**, Musc. France 2: 198, 1904 (Boulay 1904). Bas.: Ricciella A.Braun, Flora 4 (2): 756, 1821 (Braun 1821).

*** Riccia cancellata Taylor, London J. Bot. 5: 414, 1846 (Taylor 1846b).

*** Riccia cincta Jovet-Ast, Cryptog. Bryol. 21 (4): 303, 2000 (Jovet-Ast 2000).

*** Riccia cruciata Kashyap, J. Bombay Nat. Hist. Soc. 24 (2): 349, 1916 (Kashyap 1916).

*** Riccia eburnea Jovet-Ast, Cryptog. Bryol. 21 (4): 300, 2000 (Jovet-Ast 2000).

*** Riccia hasskarliana Steph., Bull. Herb. Boissier 6 (5): 374 (49), 1898 (Stephani 1898b).

*** Riccia junghuhniana Nees et Lindenb., Syn. Hepat. 4: 609, 1846 (Gottsche et al. 1846).

** Riccia junghuhniana var. simplex Schiffn., Hep. Fl. Buitenzorg: 14, 1900 (Schiffner 1900a).

*** Riccia mangalorica Ahmad ex Jovet-Ast, Cryptog. Bryol. 24 (3): 223, 2003 (Jovet-Ast 2003). Based on: Riccia mangalorica Ahmad, Curr. Sci. 11 (11): 433, 1942 (Ahmad 1942), *nom. inval*.

*** Riccia multifida (Steph.) Steph., Bull. Herb. Boissier 6 (5): 365 (40), 1898 (Stephani 1898b). Bas.: Ricciella multifida Steph., Hedwigia 28 (4): 273, 1889 (Stephani 1889c).

** Riccia polycarpa (Trab.) Jelenc, Bull. Trimestriel Geogr. Archeol. Oran 73 (228): 88, 1950 (Jelenc 1950). Bas.: Ricciella polycarpa Trab., Mém. Soc. Hist. Nat. Afrique N. 3: 36, 1933 (Maire 1933).

*** Riccia porosa Taylor, London J. Bot. 5: 416, 1846 (Taylor 1846b).

*** Riccia pullulans Jovet-Ast, Cryptog. Bryol. Lichénol. 18 (3): 183, 1997 (Jovet-Ast 1997).

** **sect. Ricciella (A.Braun) Bisch.**, Bem. Leberm.: 160, 1835 (Bischoff 1835). Bas.: Ricciella A.Braun, Flora 4 (2): 756, 1821 (Braun 1821).

* Riccia bahiensis Steph., Bull. Herb. Boissier 6 (5): 375 (50), 1898 (Stephani 1898b). ^454^

*** Riccia canaliculata Hoffm., Deutschl. Fl., Theil 2 (Hoffm.): 96, 1795 [1796] (Hoffmann 1795).

*** Riccia chiapasensis Jovet-Ast, Cryptog. Bryol. Lichénol. 14 (3): 235, 1993 (Jovet-Ast 1993b).

*** Riccia crassifrons Spruce, Trans. & Proc. Bot. Soc. Edinburgh 15: 570, 1885 (Spruce 1885).

*** Riccia duplex Lorb. ex Müll.Frib., Hedwigia 80 (1/2): 100, 1941 (Müller 1941).

** Riccia duplex var. megaspora Na-Thalang, Brunonia 3 (1): 128, 1980 (Na-Thalang 1980).

*** Riccia dussiana Steph., Symb. Antill. (Urban) 3 (2): 275, 1902 (Stephani 1902e).

*** Riccia fluitans L., Sp. Pl. 1: 1139, 1753 (Linnaeus 1753).

*** Riccia frostii Austin, Bull. Torrey Bot. Club 6 (3): 17, 1875 (Austin 1875b).

* Riccia frostii var. crystallinoides Schiffn., Ann. K. K. Naturhist. Hofmus. 27: 503, 1913 (Schiffner 1913).

*** Riccia geissleriana Jovet-Ast, Cryptog. Bryol. Lichénol. 14 (3): 236, 1993 (Jovet-Ast 1993b).

*** Riccia hegewaldiana Jovet-Ast, Cryptog. Bryol. Lichénol. 14 (3): 238, 1993 (Jovet-Ast 1993b).

*** Riccia huebeneriana Lindenb., Nova Acta Phys.-Med. Acad. Caes. Leop.-Carol. Nat. Cur. 18 (1): 504d, 1836 [1837] (Lindenberg 1836).

* Riccia huebeneriana subsp. sullivantii (Austin) R.M.Schust., Hepat. Anthocerotae N. Amer. 6: 457, 1992 (Schuster 1992d). Bas.: Riccia sullivantii Austin, Proc. Acad. Nat. Sci. Philadelphia 21: 233, 1869 (Austin 1869).

*** Riccia jovet-astiae E.Vianna, Bol. Inst. Bioci. Univ. Fed. Rio Grande do Sul 38: 165, 1985 (Vianna 1985).

*** Riccia limicola Jovet-Ast, Rev. Bryol. Lichénol. 44 (4): 422, 1978 (Jovet-Ast 1978).

*** Riccia paraguayensis Spruce, Bull. Soc. Bot. France (Congr. Bot.) 36: cxcvi, 1889 [1890] (Spruce 1889).

*** Riccia paranaensis Hässel, Opera Lilloana 7: 228, 1962 [1963] (Hässel 1962). ^455^

*** Riccia perennis Steph., Bull. Herb. Boissier 6 (5): 372 (47), 1898 (Stephani 1898b).

*** Riccia purpurascens Lehm., Linnaea 4: 371, 1829 (Lehmann 1829).

** Riccia rhenana Lorb. ex Müll.Frib., Hedwigia 80 (1/2): 94, 1941 (Müller 1941).

** Riccia rhenana var. violacea M.F.Boiko, Chornom. Bot. J. 7: 93, 2011 (Boiko 2011).

*** Riccia stricta (Lindenb.) Perold, Bothalia 20 (2): 197, 1990 (Perold 1990d). Bas.: Riccia fluitans var. stricta Lindenb., Nova Acta Phys.-Med. Acad. Caes. Leop.-Carol. Nat. Cur. 18 (1): 444, 1836 [1837] (Lindenberg 1836).

** **sect. Spongodes Nees**, Naturgesch. Eur. Leberm. 4: 391, 1838 (Nees 1838a).

*** Riccia bullosa Link, Syn. hepat. eur: 119, 1829 (Lindenberg 1829).

*** Riccia cavernosa Hoffm., Deutschl. Fl., Theil 2 (Hoffm.): 95, 1795 [1796] (Hoffmann 1795).

*** Riccia crystallina L., Sp. Pl. 1: 1138, 1753 (Linnaeus 1753).

*** Riccia cupulifera A.V.Duthie, Trans. Roy. Soc. South Africa 24 (2): 116, 1936 (Duthie and Garside 1936).

*** Riccia garsidei Sim, Trans. Roy. Soc. South Africa 15 (1): 13, 1926 (Sim 1926).

*** Riccia moenkemeyeri Steph., Bot. Jahrb. Syst. 8 (2): 95, 1886 (Stephani 1886d).

*** Riccia rubricollis Garside et A.V.Duthie ex Perold, Bothalia 21 (1): 51, 1991 (Perold 1991a).

*** Riccia volkii S.W.Arnell, Mitt. Bot. Staatssamml. München 2 (16): 271, 1957 (Arnell 1957c).

*** Riccia vulcanicola Eb.Fisch., Trop. Bryol. 8: 70, 1993 (Fischer 1993).

** **subg. Thallocarpus (Lindb.) Jovet-Ast**, Cryptog. Bryol. Lichénol. 14 (3): 220, 1993 (Jovet-Ast 1993b). Bas.: Thallocarpus Lindb., Not. Sällsk. Fauna Fl. Fenn. Förh. 13: 377, 1874 (Lindberg 1874a).

*** Riccia curtisii (Austin) Austin, Bull. Torrey Bot. Club 6 (52): 305, 1879 (Austin 1879). Bas.: Cryptocarpus curtisii Austin, Proc. Acad. Nat. Sci. Philadelphia 21: 231, 1869 (Austin 1869).

** Riccia leptothallus R.M.Schust., J. Hattori Bot. Lab. 71: 271, 1992 (Schuster 1992c).

*** Riccia perssonii Sultan Khan, Svensk Bot. Tidskr. 49 (3): 433, 1955 (Kahn 1955).

** **subg. Triseriata Jovet-Ast**, Cryptog. Bryol. Lichénol. 17 (2): 132, 1996 (Jovet-Ast 1996).

*** Riccia singularis Jovet-Ast, Cryptog. Bryol. Lichénol. 17 (2): 127, 1996 (Jovet-Ast 1996).

***Incertae sedis***

** Riccia abuensis Bapna, Trans. Brit. Bryol. Soc. 4 (2): 249, 1962 (Bapna 1962).

** Riccia acutisulca Steph., Sp. Hepat. (Stephani) 6: 1, 1917 (Stephani 1917a).

* Riccia amboinensis Schiffn., Leberm., Forschungsr. Gazelle 4 (4): 44, 1890 (Schiffner 1890).

** Riccia aravalliensis Pandé et Udar, J. Indian Bot. Soc. 36 (3): 249, 1957 (Pandé and Udar 1957).

* Riccia arnellii Sultan Khan, Bryologist 60 (1): 29, 1957 (Khan 1957).

** Riccia asprella Carrington et Pearson, Proc. Linn. Soc. New South Wales (ser. 2) 2 (4): 1059, 1888 (Carrington and Pearson 1888a).

* Riccia asservanda De Not. ex Lamothe, Rech. Anat. Taxinom. Gamét. Marchantiales: 138, 1919 (Lamothe 1919).

** Riccia attenuata Pandé, Proc. Natl. Inst. Sci. India B 25 (2): 92, 1959 (Pandé and Udar 1959).

* Riccia balansae Steph., Bull. Herb. Boissier 6 (5): 370 (45), 1898 (Stephani 1898b).

* Riccia bialbistrata Hässel, Opera Lilloana 7: 243, 1962 [1963] (Hässel 1962). ^456^

** Riccia biokoensis Perold, Nova Hedwigia 64 (1/2): 244, 1997 (Perold 1997b).

** Riccia blackii Na-Thalang, Brunonia 3 (1): 81, 1980 (Na-Thalang 1980).

*** Riccia caroliniana Na-Thalang, Brunonia 3 (1): 72, 1980 (Na-Thalang 1980).

** Riccia cartilaginosa Steph., Hedwigia 28 (4): 272, 1889 (Stephani 1889c).

* Riccia chartacea K.I.Goebel, Organogr. Pfl., ed. 2, 2 (1): 630, 1915 (Goebel 1915).

** Riccia chinensis Herzog, Symb. Sin. 5: 1, 1930 (Nicholson et al. 1930).

** Riccia collata Na-Thalang, Brunonia 3 (1): 122, 1980 (Na-Thalang 1980).

** Riccia compacta Garside, Trans. Roy. Soc. South Africa 27 (1): 17, 1939 (Duthie and Garside 1939).

** Riccia convexa Steph., Sp. Hepat. (Stephani) 6: 2, 1917 (Stephani 1917a).

* Riccia coronata Sim, Trans. Roy. Soc. South Africa 15 (1): 9, 1926 (Sim 1926). ^457^

** Riccia crassa Steph., Bull. Herb. Boissier 6 (5): 376 (51), 1898 (Stephani 1898b).

** Riccia crenatodentata O.H.Volk, Nova Hedwigia 46 (1/2): 27, 1988 (Volk 1988).

** Riccia delavayi Steph., Bull. Herb. Boissier 6 (5): 367 (42), 1898 (Stephani 1898b).

** Riccia deserticola Steph., Bull. Herb. Boissier 6 (5): 373 (48), 1898 (Stephani 1898b).

*** Riccia erubescens Perold, J. Bryol. 16 (3): 371, 1991 (Perold 1991b).

** Riccia esulcata Steph., Sp. Hepat. (Stephani) 6: 2, 1917 (Stephani 1917a).

** Riccia fertilissima Steph., Sp. Hepat. (Stephani) 6: 2, 1917 (Stephani 1917a).

* Riccia gemmifera O.H.Volk, Nova Hedwigia 39: 131, 1984 (Volk 1984). ^458^

** Riccia grollei Udar, Curr. Sci. 34 (4): 126, 1965 (Udar 1965). *Nom. nov. pro Riccia tuberculata* Pandé et Udar, Proc. Natl. Inst. Sci. India B 24 (2): 83, 1958 (Pandé and Udar 1958), *nom. illeg*.

** Riccia handelii Schiffn., Symb. sin. 2: 81, 1937 (Schiffner 1937).

** Riccia hawaiiensis Hürl., Phytologia 61 (5): 339, 1986 (Hürlimann 1986).

** Riccia indica Udar et A.Gupta, Proc. V Indian Geophytol. Conf., Special Publ.: 307, 1984 (Udar and Gupta 1984).

** Riccia indira-gandhiensis Dabhade et A.Hasan, J. Bombay Nat. Hist. Soc. 83 (2): 400, 1986 (Dabhade and Hasan 1986).

* Riccia intermedia Roum., Mém. Soc. Arts Sci. Carcassonne 5: 198, 1888 (Roumeguère 1888). ^459^

** Riccia jodhpurensis Bapna, Bot. Not. 114 (2): 181, 1961 (Bapna 1961).

** Riccia kirinensis C.Gao et K.C.Chang, Acta Phytotax. Sin. 16 (4): 117, 1978 (Gao and Chang 1978).

*** Riccia laxisquamata (Steph.) Steph., Bull. Herb. Boissier 6 (5): 371 (46), 1898 (Stephani 1898b). Bas.: Ricciella laxisquamata Steph., Bot. Jahrb. Syst. 20 (3): 299, 1895 (Stephani 1895a).

** Riccia liaoningensis C.Gao et K.C.Chang, Acta Phytotax. Sin. 16 (4): 113, 1978 (Gao and Chang 1978).

** Riccia linearis (Schiffn.) Steph., Bull. Herb. Boissier 6 (5): 371 (46), 1898 (Stephani 1898b). Bas.: Ricciella linearis Schiffn., Leberm., Forschungsr. Gazelle 4 (4): 43, 1890 (Schiffner 1890).

** Riccia luticola Na-Thalang, Brunonia 3 (1): 123, 1980 (Na-Thalang 1980).

** Riccia mamrensis Perold, Cryptog. Bryol. 26 (1): 68, 2005 (Perold 2005).

* Riccia marginata Lindb., Meddel. Soc. Fauna Fl. Fenn. 1: 106, 1876 [1877] (Lindberg 1876b). ^460^

** Riccia melanospora Kashyap, Liverworts W. Himal. 1: 94, 1929 (Kashyap 1929).

** Riccia miyakeana Schiffn., Österr. Bot. Z. 49 (11): 386, 1899 (Schiffner 1899c).

** Riccia muscicola Steph., Hedwigia 24 (1): 4, 1885 (Stephani 1885e).

*** Riccia nigerica E.W.Jones, Trans. Brit. Bryol. Soc. 3 (2): 225, 1957 (Jones 1957a).

* Riccia nigrescens Mont., Voy. Amér. Mérid., Bot. 7 (1): 15, 1839 (Montagne 1839b). ^461^

** Riccia nipponica S.Hatt., J. Hattori Bot. Lab. 9: 38, 1953 (Shimizu and Hattori 1953a).

** Riccia novo-hannoverana Schiffn., Leberm., Forschungsr. Gazelle 4 (4): 44, 1890 (Schiffner 1890).

** Riccia numeensis Steph., Bull. Herb. Boissier 6 (4): 343 (35), 1898 (Stephani 1898a).

* Riccia obtusa Meijer, J. Hattori Bot. Lab. 20: 113, 1958 (Meijer 1958). ^462^

*** Riccia oerstediana Lindenb. et Hampe, Linnaea 24 (3): 304, 1851 [1852] (Hampe 1851b).

** Riccia pandei Udar, J. Indian Bot. Soc. 38 (1): 149, 1959 (Udar 1959).

*** Riccia papillispora Steph., Bull. Herb. Boissier 6 (4): 334 (26), 1898 (Stephani 1898a).

** Riccia papulosa (Steph.) Steph., Bull. Herb. Boissier 6 (5): 377 (52), 1898 (Stephani 1898b). Bas.: Ricciella papulosa Steph., Hedwigia 28 (4): 273, 1889 (Stephani 1889c).

** Riccia papulosa var. variabilis Na-Thalang, Brunonia 3 (1): 112, 1980 (Na-Thalang 1980).

** Riccia pathankotensis Kashyap, J. Bombay Nat. Hist. Soc. 24 (2): 349, 1916 (Kashyap 1916).

* Riccia perthiana Steph. ex K.I.Goebel, Organogr. Pfl., ed. 2, 2 (1): 630, 1915 (Goebel 1915).

* Riccia prominens Meijer, J. Hattori Bot. Lab. 20: 111, 1958 (Meijer 1958). ^463^

** Riccia pseudofluitans C.Gao et K.C.Chang, Acta Phytotax. Sin. 16 (4): 116, 1978 (Gao and Chang 1978).

** Riccia pubescens S.Hatt., Nat. Sci. Mus. 14 (6): 141, 1943 (Hattori 1943a).

*** Riccia radiata Perold, Bothalia 34 (1): 23, 2004 (Perold 2004).

** Riccia rechingeri Steph., Akad. Wiss. Wien, Math.-Naturwiss. Kl., Denkschr. 81: 288, 1907 (Stephani 1907a).

* Riccia reticulatula Udar, Bull. Bot. Soc. Univ. Saugar 13: 49, 1961 (Udar 1961).

** Riccia rorida Na-Thalang, Brunonia 3 (1): 101, 1980 (Na-Thalang 1980).

* Riccia saharensis Steph. ex Jovet-Ast, Rev. Bryol. Lichénol. 26 (1/2): 62, 1957 (Gillett and Jovet-Ast 1957). ^464^

** Riccia satoi S.Hatt., Bot. Mag. (Tokyo) 62 (733/734): 109, 1949 (Hattori 1949).

* Riccia schroederi Steph., 52 (5): 304, 1912 (Stephani 1912a).

*** Riccia schweinfurthii Steph., Bull. Herb. Boissier 6 (4): 339 (31), 1898 (Stephani 1898a).

** Riccia sibayenii Perold, Bothalia 31 (1): 151, 2001 (Perold 2001a).

*** Riccia somaliensis Perold, J. Bryol. 16 (3): 367, 1991 (Perold 1991b).

** Riccia spongiosula Na-Thalang, Brunonia 3 (1): 113, 1980 (Na-Thalang 1980).

* Riccia subtilis (Steph.) Steph., Bull. Herb. Boissier 6 (5): 364 (39), 1898 (Stephani 1898b). Bas.: Ricciella subtilis Steph., Bih. Kongl. Svenska Vetensk.-Akad. Handl. 23 (III, 2): 31, 1897 (Stephani 1897a).

** Riccia sumatrana Meijer, J. Hattori Bot. Lab. 20: 114, 1958 (Meijer 1958).

*** Riccia symoensii Vanden Berghen, Explor. Hydrobiol. Lac Bangweolo Luapula: 191, 1972 (Vanden Berghen 1972b).

** Riccia tasmanica Steph. ex Rodway, Tasm. Bryoph.: 4, 1917 (Rodway 1917b).

*** Riccia tomentosa O.H.Volk et Perold, Bothalia 20 (1): 25, 1990 (Volk and Perold 1990).

** Riccia treubiana Steph., Bull. Herb. Boissier 6 (4): 323 (15), 1898 (Stephani 1898a).

* Riccia treubiana var. subrubescens Schiffn., Hep. Fl. Buitenzorg: 16, 1900 (Schiffner 1900a).

* Riccia triangularis Steph., Bull. Mus. Natl. Hist. Nat. 18 (2): 116, 1912 (Corbière 1912).

* Riccia tuberculata Poir., Encycl. (Lamarck) 6: 199, 1804 (Lamarck and Poiret 1804). ^465^

** Riccia udarii Kanwal, J. Indian Bot. Soc. 58 (3): 282, 1979 (Kanwal 1979).

* Riccia velenovskyi Kavina, Arch. Přír. Výzk. Čech 16 (2): 75, 1915 (Kavina 1915). ^466^

** Riccia velimalaiana A.E.D.Daniels et P.Daniel, Bull. Bot. Surv. India 44 (1/4): 139, 2002 [2003] (Daniels and Daniel 2002).

** Riccia victoriensis Steph., Bull. Herb. Boissier 6 (5): 370, 1898 (Stephani 1898b).

** Riccia weymouthiana Steph. ex Rodway, Tasm. Bryoph.: 5, 1917 (Rodway 1917b).

** Riccia wichurae Steph., Bull. Herb. Boissier 6 (4): 330 (22), 1898 (Stephani 1898a).

** **Ricciocarpos Corda**, Gen. hepat.: 651, 1829 (Corda 1829).

*** Ricciocarpos natans (L.) Corda, Gen. hepat.: 651, 1829 (Corda 1829). Bas.: Riccia natans L., Syst. Nat., ed. 10., 2: 1339, 1759 (Linnaeus 1759).

####### *** Targioniaceae Dumort.

by D.G. Long

*** **Targionia L.**, Sp. Pl. 1: 1136, 1753 (Linnaeus 1753).

** **subg. Prototargionia R.M.Schust.**, Hepat. Anthocerotae N. Amer. 6: 69, 1992 (Schuster 1992d).

** Targionia stellaris (Müll.Frib.) Hässel, Opera Lilloana 7: 74, 1962 [1963] (Hässel 1962). Bas.: Grimaldia stellaris Müll.Frib., Feddes Repert. Spec. Nov. Regni Veg. 58: 61, 1955 (Müller 1955).

** **subg. Targionia**

*** Targionia hypophylla L., Sp. Pl. 1: 1136, 1753 (Linnaeus 1753).

** Targionia hypophylla subsp. linealis W.Frey et Kürschner, Nova Hedwigia 57 (1/2): 127, 1993 (Frey and Kürschner 1993).

** Targionia lorbeeriana Müll.Frib., Hedwigia 79 (1/2): 78, 1940 (Müller 1940).

***Incertae sedis***

* Targionia dioica Schiffn., Denkschr. Kaiserl. Akad. Wiss., Math.-Naturwiss. Kl. 67: 154, 1898 (Schiffner 1898a). ^467^

* Targionia elongata Bisch., Syn. Hepat. 4: 576, 1846 (Gottsche et al. 1846).

* Targionia fiorii Gola, Ann. Bot. (Rome) 13 (1): 62, 1914 (Gola 1914a).

* Targionia formosica Horik., J. Jap. Bot. 11: 499, 1935 (Horikawa 1935).

* Targionia indica Udar et A.Gupta, Geophytology 13 (1): 83, 1983 (Udar and Gupta 1983).

####### *** Wiesnerellaceae Inoue

by D.G.Long

*** **Wiesnerella Schiffn.**, Österr. Bot. Z. 46 (3): 86, 1896 (Schiffner 1896b).

*** Wiesnerella denudata (Mitt.) Steph., Bull. Herb. Boissier 7 (5): 382 (154), 1899 (Stephani 1899c). Bas.: Dumortiera denudata Mitt., J. Proc. Linn. Soc., Bot. 5 (18): 125, 1860 [1861] (Mitten 1860c).

* Wiesnerella fasciaria C.Gao et K.C.Chang, Acta Bot. Yunnan. 3 (4): 391, 1981 (Gao et al. 1981).

###### Neohodgsoniales D.G.Long

####### *** Neohodgsoniaceae D.G.Long

by D.G. Long

*** **Neohodgsonia Perss.**, Bot. Not. 107 (1): 40, 1954 (Persson 1954). *Nom. nov. pro Hodgsonia* Perss., Hodgsonia Leafl. Stockholm: 1, 1953 (Persson 1953).

*** Neohodgsonia mirabilis (Perss.) Perss., Bot. Not. 107 (1): 40, 1954 (Persson 1954). Bas.: Hodgsonia mirabilis Perss., Hodgsonia Leafl. Stockholm: 1, 1953 (Persson 1953).

###### Sphaerocarpales Cavers

####### *** Monocarpaceae D.J.Carr ex Schelpe

by D.G. Long

*** **Monocarpus D.J.Carr**, Austral. J. Bot. 4 (2): 176, 1956 (Carr 1956).

*** Monocarpus sphaerocarpus D.J.Carr, Austral. J. Bot. 4 (2): 176, 1956 (Carr 1956).

####### *** Riellaceae Engl.

by J. G. Segarra-Moragues and F. Puche

*** **Austroriella Cargill et J.Milne**, Polish Bot. J. 58 (1): 72, 2013 (Cargill and Milne 2013).

*** Austroriella salta J.Milne et Cargill, Polish Bot. J. 58 (1): 72, 2013 (Cargill and Milne 2013).

*** **Riella Mont.**, Ann. Sci. Nat. Bot. (sér. 3) 18: 11, 1852 (Montagne 1852).

*** **subg. Riella**

*** Riella alatospora Wigglesw., J. Linn. Soc., Bot. 51 (339): 317, 1937 (Wigglesworth 1937).

*** Riella americana M.Howe et Underw., Bull. Torrey Bot. Club 30 (4): 218, 1903 (Howe and Underwood 1903).

* Riella battandieri Trab., Rev. Bryol. 13 (3): 35, 1886 (Trabut 1886).

*** Riella bialata Trab., Rev. Bryol. 35 (4): 96, 1908 (Trabut 1908).

*** Riella capensis Cavers, Rev. Bryol. 30 (5): 81, 1903 (Cavers 1903).

*** Riella choconensis Hässel, Symp. Biol. Hung. 35: 341, 1987 (Hässel 1987).

* Riella cyrenaica Maire, Bull. Soc. Hist. Nat. Afrique N. 30 (5): 312, 1939 (Maire and Weiler 1939).

*** Riella echinospora Wigglesw., J. Linn. Soc., Bot. 51 (339): 321, 1937 (Wigglesworth 1937).

* Riella gallica Balansa ex Trab., Rev. Gén. Bot. 3 (35): 450, 1891 (Trabut 1891).

*** Riella halophila Banwell, Trans. Brit. Bryol. Soc. 1 (5): 475, 1951 (Banwell 1951).

*** Riella helicophylla (Bory et Mont.) Mont., Ann. Sci. Nat. Bot. (sér. 3) 18: 12, 1852 (Montagne 1852). Bas.: Duriaea helicophylla Bory et Mont., Ann. Sci. Nat. Bot. (sér. 3) 1: 229, 1844 (Bory and Montagne 1844).

* Riella helicophylla var. macrocarpa P.Allorge, Sched. Br. Iber. (ser. 2): 4, 1929 (Allorge 1929).

* Riella indica Steph. ex Kashyap, J. Bombay Nat. Hist. Soc. 25 (2): 279, 1917 (Kashyap 1917).

*** Riella notarisii (Mont.) Mont., Ann. Sci. Nat. Bot. (sér. 3) 18: 12, 1852 (Montagne 1852). Bas.: Sphaerocarpos notarisii Mont., Ann. Sci. Nat. Bot. (sér. 2) 9: 39, 1838 (Montagne 1838). ^468^

** Riella numidica Trab., Bull. Soc. Hist. Nat. Afrique N. 25 (9): 391, 1934 [1935] (Trabut 1934).

*** Riella pampae Hässel, Revista Mus. Argent. Ci. Nat., Bernardino Rivadavia Inst. Nac. Invest. Ci. Nat. Bot. 5 (9): 207, 1979 (Hässel 1979).

*** Riella parisii Gottsche, Hepat. Eur., Leberm. 38-39: no. 375, 1867 (Gottsche and Rabenhorst 1867).

*** Riella purpureospora Wigglesw., J. Linn. Soc., Bot. 51 (339): 312, 1937 (Wigglesworth 1937).

* Riella reuteri Mont., Ann. Sci. Nat. Bot. (sér. 3) 18: 12, 1852 (Montagne 1852).

* Riella sersuensis Trab., Bull. Soc. Hist. Nat. Afrique N. 25 (9): 392, 1934 [1935] (Trabut 1934).

** Riella spiculata J.Taylor, Kew Bull. 9 (1): 45, 1954 (Taylor 1954).

*** Riella trigonospora Segarra et Puche, S. African J. Bot. 94: 175, 2014 (Segarra-Moragues and Puche 2014).

*** Riella undulata Hässel, Symp. Biol. Hung. 35: 341, 1987 (Hässel 1987).

*** **subg. Trabutiella Porsild**, Bot. Tidsskr. 24 (3): 327, 1902 (Porsild 1902).

*** Riella affinis M.Howe et Underw., Bull. Torrey Bot. Club 30 (4): 221, 1903 (Howe and Underwood 1903).

*** Riella cossoniana Trab., Atlas fl. Alger 1: 6, 1886 (Battandier and Trabut 1886).

*** Riella echinata (Müll.Frib.) Segarra, Puche et Sabovlj., Phytotaxa 159 (3): 165, 2014 (Segarra-Moragues et al. 2014). Bas.: Riella cossoniana var. echinata Müll.Frib., Rev. Bryol. Lichénol. 22 (3/4): 132, 1953 [1954] (Müller 1953).

*** Riella gamundiae Hässel, Rev. Bryol. Lichénol. 38 (3/4): 580, 1972 [1973] (Hässel 1972b).

*** Riella heliospora Segarra, Puche et Sabovlj., Syst. Bot. 37 (2): 315, 2012 (Segarra-Moragues et al. 2012).

*** Riella mediterranea Segarra, Puche, Sabovlj., M.Infante et Heras, Phytotaxa 159 (3): 170, 2014 (Segarra-Moragues et al. 2014).

####### *** Sphaerocarpaceae Heeg

by D. Long

*** **Geothallus Campb.**, Bot. Gaz. 21 (1): 13, 1896 (Campbell 1896).

*** Geothallus tuberosus Campb., Bot. Gaz. 21 (1): 13, 1896 (Campbell 1896).

*** **Sphaerocarpos Boehm.**, Def. gen. pl., ed. 3: 501, 1760 (Ludwig 1760).

** **subg. Austrosphaerocarpos R.M.Schust.**, Hepat. Anthocerotae N. Amer. 5: 813, 1992 (Schuster 1992b).

*** Sphaerocarpos stipitatus Bisch. ex Lindenb., Nova Acta Phys.-Med. Acad. Caes. Leop.-Carol. Nat. Cur. 18 (1): 504i, 1836 [1837] (Lindenberg 1836).

** **subg. Sphaerocarpos**

*** Sphaerocarpos michelii Bellardi, App. fl. pedem.: 52, 1792 (Bellardi 1792).

** Sphaerocarpos texanus Austin, Bull. Torrey Bot. Club 6 (30): 158, 1877 (Austin 1877).

***Incertae sedis***

*** Sphaerocarpos cristatus M.Howe, Mem. Torrey Bot. Club 7: 66, 1899 (Howe 1899).

*** Sphaerocarpos donnellii Austin, Bull. Torrey Bot. Club 6 (30): 157, 1877 (Austin 1877).

*** Sphaerocarpos drewiae Wigglesw., Univ. Calif. Publ. Bot. 16 (3): 129, 1929 (Wigglesworth 1929).

*** Sphaerocarpos europaeus Lorb., Jahrb. Wiss. Bot. 80: 665, 1934 (Lorbeer 1934). ^469^

*** Sphaerocarpos hians Haynes, Bull. Torrey Bot. Club 37 (5): 225, 1910 (Haynes 1910).

** Sphaerocarpos muccilloi E.Vianna, Lindbergia 7 (1): 58, 1981 (Vianna 1981).

## Names in genera not currently accepted

The following taxa in unsupported genera are all poorly understood. We list them here rather than making new combinations for names we do not know the status of.

**Acrostolia Dumort.**, Recueil Observ. Jungerm.: 26, 1835 (Dumortier 1835). ^470^

* Acrostolia alata (Gottsche et Rabenh.) Trevis., Mem. Reale Ist. Lombardo Sci. (Ser. 3), C. Sci. Mat. 4 (13): 431, 1877 (Trevisan 1877). Bas.: Pseudoneura alata Gottsche et Rabenh., Hepat. Eur., Leberm. 56-57: no. 560, 1873 (Gottsche and Rabenhorst 1873b).

* Acrostolia brevifolia (Gottsche et Rabenh.) Trevis., Mem. Reale Ist. Lombardo Sci. (Ser. 3), C. Sci. Mat. 4 (13): 431, 1877 (Trevisan 1877). Bas.: Pseudoneura brevifolia Gottsche et Rabenh., Hepat. Eur., Leberm. 56-57: no. 560, 1873 (Gottsche and Rabenhorst 1873b).

**Aphanolejeunea A.Evans**, Bull. Torrey Bot. Club 38 (6): 272, 1911 (Evans 1911).

* Aphanolejeunea lancifera R.M.Schust., Phytologia 45 (5): 434, 1980 (Schuster 1980b). ^471^

* Aphanolejeunea minima Tixier, Ann. Fac. Sci. Yaoundé 20: 7, 1975 (Tixier 1975b). ^472^

**Aspiromitus Steph.**, Sp. Hepat. (Stephani) 5: 957, 1916 (Stephani 1916b). ^473^

* Aspiromitus asper Schiffn., Arch. Hydrobiol., suppl. 21 (3/4): 402, 1955 (Schiffner 1955).

* Aspiromitus bullosus Schiffn., Arch. Hydrobiol., suppl. 21 (3/4): 403, 1955 (Schiffner 1955).

* Aspiromitus crenatifrons Steph., Sp. Hepat. (Stephani) 5: 968, 1916 (Stephani 1916b).

* Aspiromitus lobatus Schiffn., Arch. Hydrobiol., suppl. 21 (3/4): 405, 1955 (Schiffner 1955).

* Aspiromitus squamulosus Schiffn., Arch. Hydrobiol., suppl. 21 (3/4): 407, 1955 (Schiffner 1955).

**Crossotolejeunea (Spruce) Schiffn.**, Hepat. (Engl.-Prantl): 127, 1893 (Schiffner 1893b). Bas.: Lejeunea subg. Crossotolejeunea Spruce, Trans. & Proc. Bot. Soc. Edinburgh 15: 161, 1884 (Spruce 1884). ^474^

* Crossotolejeunea curvifolia Steph., Hedwigia 35 (3): 75, 1896 (Stephani 1896b). ^475^

**Eulejeunea Steph.**, Hedwigia 27 (2): 60, 1888 (Stephani 1888a). ^476^

* Eulejeunea setulosa Steph., Sp. Hepat. (Stephani) 6: 421, 1923 (Stephani 1923).

* Eulejeunea subpililoba Steph., Sp. Hepat. (Stephani) 6: 420, 1923 (Stephani 1923). ^477^

**Euosmolejeunea (Spruce) Steph.**, Hedwigia 28 (3): 170, 1889 (Stephani 1889d). Bas.: Lejeunea subg. Euosmolejeunea Spruce, Trans. & Proc. Bot. Soc. Edinburgh 15: 241, 1884 (Spruce 1884). ^478^

* Euosmolejeunea parvistipula (Lindenb. et Gottsche) Steph., Hedwigia 29 (1): 80, 1890 (Stephani 1890a). Bas.: Lejeunea parvistipula Lindenb. et Gottsche, Syn. Hepat. 5: 761, 1847 (Gottsche et al. 1847).

* Euosmolejeunea tenerrima (Nees) Steph., Sp. Hepat. (Stephani) 5: 589, 1914 (Stephani 1914b). Bas.: Jungermannia sordida var. tenerrima Nees, Fl. Bras. (Martius) 1 (1): 363, 1833 (Nees 1833a). ^479^

**Fimbraria Nees**, Horae Phys. Berol.: 44, 1820 (Nees 1820) nom. illeg. ^480^

* Fimbraria gigantea Steph., Bull. Herb. Boissier 7 (2): 93 (106), 1899 (Stephani 1899a). ^481^

* Fimbraria incrassata Steph., Bull. Herb. Boissier 7 (2): 87 (100), 1899 (Stephani 1899a). ^482^

* Fimbraria kamerunensis Steph., Sp. Hepat. (Stephani) 6: 14, 1917 (Stephani 1917a). ^483^

* Fimbraria pirottae Gola, Ann. Bot. (Rome) 13 (1): 64, 1914 (Gola 1914a).

**Hygrolejeunea (Spruce) Schiffn.**, Hepat. (Engl.-Prantl): 124, 1893 (Schiffner 1893b). Bas.: Lejeunea subg. Hygrolejeunea Spruce, Trans. & Proc. Bot. Soc. Edinburgh 15: 230, 1884 (Spruce 1884). ^484^

* Hygrolejeunea cubensis Steph., Sp. Hepat. (Stephani) 5: 533, 1914 (Stephani 1914b).

* Hygrolejeunea harpaphylla Steph., Sp. Hepat. (Stephani) 5: 552, 1914 (Stephani 1914b).

* Hygrolejeunea pacifica Steph., Sp. Hepat. (Stephani) 6: 411, 1923 (Stephani 1923).

* Hygrolejeunea parvicalycina Steph., Hedwigia 35 (3): 103, 1896 (Stephani 1896b).

* Hygrolejeunea parvistipula Steph., Sp. Hepat. (Stephani) 5: 568, 1914 (Stephani 1914b).

* Hygrolejeunea patellirostris Steph., Hedwigia 35 (3): 103, 1896 (Stephani 1896b). ^485^

* Hygrolejeunea staudtiana Steph., Sp. Hepat. (Stephani) 5: 528, 1914 (Stephani 1914b). ^486^

**Hypenantron Corda**, Gen. hepat.: 648, 1829 (Corda 1829). ^487^

* Hypenantron brachypus Steph. ex Lamothe, Rech. Anat. Taxinom. Gamét. Marchantiales: 107, 1919 (Lamothe 1919).

* Hypenantron brasiliense Steph. ex Lamothe, Rech. Anat. Taxinom. Gamét. Marchantiales: 104, 1919 (Lamothe 1919).

**Jamesoniella (Spruce) Carrington**, Cat. Brit. Moss. Hepat.: 25, 1881 (Carrington 1881). Bas.: Jungermannia subg. Jamesoniella Spruce, J. Bot. 14: 230, 1876 (Spruce 1876a). ^488^

* Jamesoniella convoluta Steph., Sp. Hepat. (Stephani) 6: 433, 1924 (Stephani 1924).

**Kingiolejeunea H.Rob.**, Bryologist 70 (1): 53, 1967 (Robinson 1967). ^489^

* Kingiolejeunea guayanensis H.Rob., Bol. Soc. Venez. Ci. Nat. 32 (132/133): 259, 1976 (Robinson 1976b).

**Leptocolea (Spruce) A.Evans**, Bull. Torrey Bot. Club 38 (6): 261, 1911 (Evans 1911). Bas.: Lejeunea sect. Leptocolea Spruce, Trans. & Proc. Bot. Soc. Edinburgh 15: 294, 1884 (Spruce 1884). ^490^

* Leptocolea sumatrana Herzog, Ann. Bryol. 5: 96, 1932 (Herzog 1932a). ^491^

**Mastigobryum (Nees) Lindenb. et Gottsche**, Syn. Hepat. 2: 214, 1845 (Gottsche et al. 1845a) nom. illeg. Bas.: Herpetium sect. Mastigobryum Nees, Naturgesch. Eur. Leberm. 3: 43, 1838 (Nees 1838b). ^492^

* Mastigobryum aberrans Steph., Sp. Hepat. (Stephani) 6: 486, 1924 (Stephani 1924).

* Mastigobryum asperum Steph., J. & Proc. Roy. Soc. New South Wales 48 (1/2): 121, 1914 (Stephani and Watts 1914).

* Mastigobryum deningeri Herzog, Beih. Bot. Centralbl. 38 (2): 322, 1921 (Herzog 1921).

* Mastigobryum karstenii Steph., Bull. Herb. Boissier (sér. 2) 8 (12): 952 (502), 1908 (Stephani 1908b).

* Mastigobryum ledermannii Steph., Sp. Hepat. (Stephani) 6: 486, 1924 (Stephani 1924).

* Mastigobryum londbergii Steph., Sp. Hepat. (Stephani) 6: 487, 1924 (Stephani 1924).

* Mastigobryum longifolium Steph., Sp. Hepat. (Stephani) 6: 487, 1924 (Stephani 1924).

* Mastigobryum minutitextum Steph., Sp. Hepat. (Stephani) 6: 487, 1924 (Stephani 1924).

* Mastigobryum multidens Steph., Sp. Hepat. (Stephani) 6: 487, 1924 (Stephani 1924).

* Mastigobryum muscicola Steph., Sp. Hepat. (Stephani) 6: 486, 1924 (Stephani 1924).

* Mastigobryum nigricans Herzog, Beih. Bot. Centralbl. 38 (2): 322, 1921 (Herzog 1921).

* Mastigobryum nipuranum Steph., Sp. Hepat. (Stephani) 6: 485, 1924 (Stephani 1924).

* Mastigobryum palmicola Steph., Sp. Hepat. (Stephani) 6: 488, 1924 (Stephani 1924).

* Mastigobryum rajanum Herzog, Mitt. Inst. Allg. Bot. Hamburg 7 (3): 190, 1931 (Herzog 1931a).

* Mastigobryum ribehanum Steph., Sp. Hepat. (Stephani) 6: 488, 1924 (Stephani 1924).

* Mastigobryum ruficaule Beauverd, Sp. Hepat. (Stephani) 6: 485, 1924 (Stephani 1924).

* Mastigobryum schraderbergii Steph., Sp. Hepat. (Stephani) 6: 489, 1924 (Stephani 1924).

* Mastigobryum squamulistipum Steph., Sp. Hepat. (Stephani) 6: 480, 1924 (Stephani 1924).

* Mastigobryum subhyalinum Steph., Sp. Hepat. (Stephani) 6: 482, 1924 (Stephani 1924).

* Mastigobryum venezuelanum Molk., Syn. hepat. jav.: 104, 1856 [1857] (Sande Lacoste 1856b).

* Mastigobryum vermiculare Herzog, Hedwigia 66 (6): 339, 1926 (Herzog 1926).

**Nemoursia Mérat**, Ann. Agric. Franç. (ser. 4) 2 (7): 10, 1840 (Mérat 1840).

* Nemoursia tuberculata Mérat, Ann. Agric. Franç. (ser. 4) 2 (7): 10, 1840 (Mérat 1840). ^493^

**Physocolea (Spruce) Steph.**, Sp. Hepat. (Stephani) 5: 863, 1916 (Stephani 1916b). Bas.: Lejeunea sect. Physocolea Spruce, Trans. & Proc. Bot. Soc. Edinburgh 15: 292, 1884 (Spruce 1884). ^494^

* Physocolea tambillensis (Loitl.) Steph., Sp. Hepat. (Stephani) 5: 885, 1916 (Stephani 1916b). Bas.: Lejeunea tambillensis Loitl., Diagn. pl. nov.: 20, 1894 (Loitlesberger 1894). ^495^

**Plectocolea (Mitt.) Mitt.**, Fl. vit.: 405, 1871 [1873] (Mitten 1871). Bas.: Solenostoma subg. Plectocolea Mitt., J. Linn. Soc., Bot. 8 (31): 156, 1864 [1865] (Mitten 1864a). ^496^

* Plectocolea subamoena S.Winkl., Rev. Bryol. Lichénol. 42 (3): 821, 1976 (Winkler 1976). ^497^

**Polyotus Gottsche**, Syn. Hepat. 2: 244, 1845 (Gottsche et al. 1845a) nom. illeg. ^498^

* Polyotus peckianus Austin, Proc. Acad. Nat. Sci. Philadelphia 21: 224, 1869 (Austin 1869).

**Schisma Dumort.**, Commentat. Bot. (Dumortier): 114, 1822 (Dumortier 1822) nom. illeg. ^499^

* Schisma orizabense (Gottsche) Steph., Sp. Hepat. (Stephani) 4: 19, 1909 (Stephani 1909d). Bas.: Sendtnera orizabensis Gottsche, Mexik. Leverm.: 139, 1863 (Gottsche 1863).

* Schisma uleanum Steph., Hedwigia 44 (4): 225, 1905 (Stephani 1905a).

**Strepsilejeunea (Spruce) Schiffn.**, Hepat. (Engl.-Prantl): 127, 1893 (Schiffner 1893b). Bas.: Lejeunea sect. Strepsilejeunea Spruce, Trans. & Proc. Bot. Soc. Edinburgh 15: 168, 1884 (Spruce 1884). ^500^

* Strepsilejeunea apollinea (Gottsche) Steph., Sp. Hepat. (Stephani) 5: 291, 1913 (Stephani 1913a). Bas.: Lejeunea apollinea Gottsche, Fragm. (Mueller): 64, 1880 (Gottsche 1880).

* Strepsilejeunea durelii Schiffn., Österr. Bot. Z. 49 (6): 206, 1899 (Schiffner 1899a).

* Strepsilejeunea hamatifolia Steph., Sp. Hepat. (Stephani) 6: 396, 1923 (Stephani 1923).

* Strepsilejeunea lanceolata (Gottsche) Steph., Sp. Hepat. (Stephani) 5: 283, 1913 (Stephani 1913a). Bas.: Lejeunea lanceolata Gottsche, Syn. Hepat. 3: 353, 1845 (Gottsche et al. 1845b).

* Strepsilejeunea muscicola Herzog, Hedwigia 74 (2): 96, 1934 (Herzog 1934a).

* Strepsilejeunea novae-guineae Steph., Sp. Hepat. (Stephani) 6: 397, 1923 (Stephani 1923).

* Strepsilejeunea obtusistipula Steph., Biblioth. Bot. 87 (2): 258, 1916 (Stephani 1916a).

* Strepsilejeunea papulifolia Steph., Biblioth. Bot. 87 (2): 259, 1916 (Stephani 1916a).

* Strepsilejeunea pectiniformis (Gottsche) Steph., Sp. Hepat. (Stephani) 5: 285, 1913 (Stephani 1913a). Bas.: Lejeunea pectiniformis Gottsche, Ann. Sci. Nat. Bot. (sér. 5) 1: 156, 1864 (Gottsche 1864).

* Strepsilejeunea renistipula Steph., Sp. Hepat. (Stephani) 5: 289, 1913 (Stephani 1913a).

* Strepsilejeunea vatovae Gerola, Lav. Bot. Ist. Bot. Univ. Padova 12: 479, 1947 (Gerola 1947).

**Thysanolejeunea (Spruce) Steph.**, Hedwigia 31 (1): 20, 1892 (Jack and Stephani 1892). Bas.: Lejeunea subg. Thysanolejeunea Spruce, Trans. & Proc. Bot. Soc. Edinburgh 15: 105, 1885 (Spruce 1885).

* Thysanolejeunea africana Sim, Trans. Roy. Soc. South Africa 15 (1): 50, 1926 (Sim 1926). ^501^

**Trachylejeunea (Spruce) Steph.**, Hedwigia 28 (4): 262, 1889 (Stephani 1889c) nom. rejic. Bas.: Lejeunea subg. Trachylejeunea Spruce, Trans. & Proc. Bot. Soc. Edinburgh 15: 180, 1884 (Spruce 1884).

* Trachylejeunea conifera Steph., Sp. Hepat. (Stephani) 5: 302, 1913 (Stephani 1913a).

* Trachylejeunea cristuliflora Steph., Hedwigia 35 (3): 137, 1896 (Stephani 1896b).

* Trachylejeunea englishii Steph., Bull. Misc. Inform. Kew 1899 (151/152): 126, 1899 (MacGregor 1899). ^502^

* Trachylejeunea jamaicensis Pearson, Ann. Bryol. 4: 98, 1931 (Pearson 1931b).

* Trachylejeunea kusaiensis Inoue et H.A.Mill., Bull. Natl. Sci. Mus. Tokyo (n.ser.) 8 (2): 147, 1965 (Inoue and Miller 1965).

## Footnotes

1 Anthoceros includes Aspiromitus, but some taxa have neither been transferred nor synonymized. They are listed in the “Names in genera not currently accepted” section below.

2 Anthoceros agrestis is conserved against the earlier Anthoceros nagasakiensis Steph. 1916. Anthoceros cristatus Steph. 1916 is also an earlier synonym and a proposal to conserve Anthoceros agrestis against it is in progress.

3 Anthoceros angustifolius (type from Europe) has neither been recognized in any recent treatment nor synonymized. The name may have priority once its identity is determined.

4 Anthoceros ferdinandi-muelleri is similar to Anthoceros punctatus, but Anthoceros still needs to be revised for Australia.

5 Anthoceros helmsii was treated as conspecific with Anthoceros muscoides by Campbell (1982), but it is in need of a revision.

6 Anthoceros javanicoides belongs to the Anthoceros punctatus species complex (Söderström et al. 2010a).

7 Anthoceros punctatus is a species complex in need of a revision.

8 Anthoceros aethyopicus is probably a Phaeoceros species (Wigginton 2009).

9 Anthoceros brunnthaleri was annotated by Hasegawa in 1993 as probably conspecific with Phymatoceros bulbiculosus but the synonymy seems not to have been published.

10 Anthoceros floribundus type specimen in G was annotated by Hasegawa in 1993 as conspecific with Anthoceros parvifrons which is a Phaeoceros species.

11

Anthoceros mildbraedii is probably conspecific with Phaeoceros carolinianus (Wigginton and Grolle 1996).

12

Anthoceros parvifrons is annotated as a Phaeoceros species by Hasegawa in 1993 but the new combination has never been published.

13

Anthoceros pseudocostus is a Phaeoceros species of uncertain status (Grolle 1995).

14

Anthoceros rossoi is a Phaeoceros species of uncertain status (Wigginton 2009).

15

Dendroceros adglutinatus is conspecific with Dendroceros crispus in Proskauer (1960), but later authors accept it.

16

Dendroceros cucullatus was treated as conspecific with Dendroceros difficilis by Hasegawa (1980), but re-instated by Chantanaorrapint et al. (2014).

17

Dendroceros exalatus was reported from the Moluccas in Stephani (1909c), but Hasegawa (1980) states that this is a Brazilian species. Dendroceros has not been recently revised for Brazil.

18

Dendroceros reticulus was mentioned by Hasegawa (1980). He did not synonymise it with Dendroceros javanicus, he just stated that Herzog thought it similar to Dendroceros elegans which is conspecific with Dendroceros javanicus.

19

Megaceros flavens and Megaceros jamesonii are probably conspecific with Nothoceros vincentianus (Villarreal et al. 2010a).

20

Mesoceros was described from a collection that contains a mixture of both Anthoceros and Phaeoceros material.

21

Phaeoceros dendroceroides may belong to Phaeomegaceros

22

Phaeoceros microsporus may be conspecific with Phaeoceros mohrii in Schuster (1992d), but he did not study any material.

23

Phaeoceros striatisporus was nested within Phaeoceros in Li et al. (2011). Morphologically it was supposed to differ by its unique spores which, however, turns out to be fungal infections (Villarreal & Renner 2012).

24

Syzygiella includes Jamesoniella (cf. Feldberg et al. 2010a), but one taxon has neither been transferred nor synonymized. It is listed in the “Names in genera not currently accepted” section below.

25

Syzygiella securifolia is a sister taxon to Syzygiella subintegerrima (Feldberg et al. 2010b) and it is debatable if they are different enough to warrant separation on the species level. Morphologically they are very similar (Söderström et al. 2010a).

26

Anastrophyllum ellipticum is only tentatively placed in the genus following Mamontov & Vilnet (2013).

27

Anastrophyllum lignicola is possibly conspecific with Anastrophyllum ellipticum and its generic placement is uncertain.

28

Barbilophozia sudetica was transferred from Lophozia by Söderström et al. (2010a), but transferred to the new genus Pseudolophozia Konstant. and Vilnet (sister to Barbilophozia) by Konstantinova and Vilnet (2009).

29

Gymnocolea inflata subsp. acutiloba is a problematic taxon that sometimes has been treated as conspecific with Gymnocolea inflata, sometimes as a separate species.

30

Plicanthus birmensis seems to be closely related to Tetralophozia (Vilnet et al. 2010), but Grolle (1995) considered it a possibly depauperate form of Plicanthus hirtellus. However, it may be a good species, possibly of the genus Tetralophozia.

31

Plicanthus giganteus is often treated as a subspecies of Plicanthus hirtellus (e.g. Pócs and Lye 1999).

32

Schizophyllopsis was originally published as Schizophyllum (R.M.Schust.) Váňa et L.Söderstr. (Váňa et al. 2013k), but that is a later homonym of Schizophyllum Fr. (1815; Fungus) and Nutt. (1841; Asteraceae).

33

Cephalozia neesiana may be conspecific with Cephalozia hamatiloba (Váňa 1988).

34

Cephalozia hians is probably an Isotachis or Lophocolea species.

35

Cephalozia indica is a Lophocolea species and probably conspecific with “Cephalozia” kodaikanalensis.

36

Cephalozia kodaikanalensis is a Lophocolea species, possibly conspecific with “Cephalozia” indica.

37

Cephalozia parvifolia is a Cephaloziella species although the type specimen has not been found.

38

Cephalozia tricuspidata is a Lepidozia species (Schiffner 1898b).

39

Fuscocephaloziopsis lunulifolia is a species complex also including Fuscocephaloziopsis affinis and Fuscocephaloziopsis schusteri.

40

Odontoschisma obcordatum is to be excluded from Odontoschisma but its identity remains unclear as the type is not found. Based on the original description this species might belong to Alobiellopsis (Gradstein & Ilkiu-Borges 2014: 81).

41

Cephaloziella is a large genus and the subgeneric division is unclear. The traditional subdivisions used here do not necessary agree with recent phylogenetic studies.

42 The correct name for the variety was discussed by Söderström et al. (2012d).

43

Cephaloziella villaumei is possibly conspecific with Cephaloziella anthelioides.

44

Cephaloziella meghalayensis is probably conspecific with Cephaloziella acanthophora or Cephaloziella microphylla.

45

Cephaloziella flexuosa may be a form of Plicanthus birmensis or a juvenile form of Tetralophozia filiformis.

46

Cephaloziella pellucida is probably not a Cephaloziella, but the type specimen has not been found.

47

Cephaloziella pygmaea is possibly conspecific with Cephaloziella granatensis (Váňa et al. 2014a).

48

Cephaloziella secundifolia could not be studied by Fulford (1976) and she did not know what it is.

49

Lophozia has many nomenclatural and taxonomic problems. This treatment follows Söderström et al. (2013c).

50

Lophozia jamaicensis is probably not a Lophozia, but the type specimen is missing in Nees’s herbarium (Söderström et al. 2010a).

51

Lophozia pallida may be conspecific with Lophozia guttulata (Bakalin 2003).

52

Lophozia subapiculata may be a form of Lophozia guttulata, but Bakalin (2005) treated it as conspecific with Lophozia ventricosa.

53

Lophozia udarii has recently been described using the generic concept of Lophozia in the wide sense (as e.g. Schuster 1969b). We do not know where to refer the species. It is possibly a Schistochilopsis species close to Schistochilopsis incisa.

54

Lophozia ventricosa is very variable and many segregates at various subspecific levels have been described. The species complex also includes at least Lophozia austrosibirica, Lophozia schusteriana and Lophozia wenzelii.

55

Lophozia wenzelii is closely related to Lophozia ventricosa according to De Roo et al. (2007) and Vilnet et al. (2010) based on molecular and morphological evidence. It is variable and many varieties are described. The variability and relationships to related species needs further study.

56

Lophozia serpens is probably conspecific with Schistochilopsis incisa.

57

Lophoziopsis longidens var. arctica was treated as a form of Lophoziopsis pellucida by Bakalin (2005).

58

Lophoziopsis propagulifera belongs to the Lophoziopsis excisa species complex. It is probably only a modification (cf. discussion by Váňa and Engel 2013).

59

Diplophyllum obtusifolium is a species complex also including Diplophyllum androgynum, Diplophyllum angustifolium, Diplophyllum gemmiparum, Diplophyllum incrassatum and Diplophyllum novum.

60

Scapania degenii is conspecific with Scapania brevicaulis in Potemkin (1998), but recognized by Konstantinova et al. (2009).

61

Scapania glaucoviridis was treated as conspecific with Scapania parvitexta by Potemkin (2002), but accepted by Zuo et al. (2007a).

62

Scapania parvidens was treated as conspecific with Scapania parvitexta by Potemkin (2002), but accepted by Zuo et al. (2007a) and Choi et al. (2012).

63

Scapania scapanioides is possibly conspecific with Scapania glaucocephala (Potemkin 2002).

64

Schistochilopsis nakanishii is possibly conspecific with Schistochilopsis incisa.

65

Schistochilopsis opacifolia is sometimes treated as a subspecies of Schistochilopsis incisa (e.g. Bisang 1991).

66

Acrobolbus bispinosus is a Plagiochila species (Burghardt and Gradstein 2008).

67

Isotachis multiceps is sometimes treated as a Hypoisotachis, but recent molecular studies (Forrest et al. 2006, Cooper et al. 2012b) point towards inclusion in Isotachis.

68

Calypogeia fissa contains two well separated taxa (Buczkowska et al. 2011), but a formal taxonomic treatment is not yet available.

69

Calypogeia lechleri was treated as conspecific with Calypogeia leptoloma by Bischler (1962c) with hesitation (type specimen not found).

70

Calypogeia muelleriana contains two well separated taxa (Buczkowska 2010, Buczkowska and Bączkiewicz 2011). One corresponds well to the type of the species while the name of the other is unknown.

71

Calypogeia sphagnicola is a species complex including at least three genetically and morphologically well separated taxa (Buczkowska et al. 2012b, 2012a). They all need to be compared with types of many existing names in the genus before the correct name can be assigned.

72

Calypogeia amazonica was treated as conspecific with Calypogeia miquelii in Gradstein et al. (1994), but it has been accepted by later authors (e.g. Churchill et al. 2008, Söderström et al. 2013e).

73

Nardia kamtschatica may be conspecific with Nardia assamica (Váňa 1976c), but the type specimen could not be studied.

74

Jackiella unica was identified as a Colura species before the B herbarium burned (Grolle 1966d).

75

Jungermannia was used for almost all leafy liverworts from Linnaeus (1753) and well into the 19th century. Some taxa have neither been transferred nor synonymized. As the names often are very old, they may gain priority over existing names if proven to be a synonym of something. They are listed in the “Names in genera not currently accepted” section below.

76

Jungermannia erectii was described on the basis of sterile material and it is not certain if it belongs in Jungermannia or Solenostoma (Váňa and Long 2009).

77

Jungermannia was used for almost all leafy hepatics from Linnaeus (1753) and well into the 19th century. The following names have not been studied recently and are neither accepted nor synonymized. Many of them may have priority when their identity is determined.

78

Jungermannia brasiliensis is a taxon of uncertain status. Costa (2009) could not find any authentic material and did not know what it is.

79

Jungermannia chinensis may be conspecific with Cephalozia connivens or Cephalozia bicuspidata.

80

Jungermannia incerta is a doubtful taxon (Grolle 1995). The type specimen was burned in B.

81

Jungermannia lescuriana is a Cephaloziella species.

82

Jungermannia longiretis is an Isotachis species.

83

Jungermannia mastigophora is probably a Mastigophora species.

84

Jungermannia michelii is a Fossombronia species.

85

Jungermannia quadridigitata is conspecific with Kurzia pauciflora in Schuster (1969b), but that species does not occur in India (Sharma and Srivastava 1993). It is probably some other Kurzia species.

86

Jungermannia stereocaulis is a doubtful taxon. Engel (1990b) did not find any type specimen and did not know what it is.

87

Jungermannia sullivantiana is a Cephaloziella species.

88

Jungermannia tenuis is a Lejeunea species.

89

Saccogyna tridens is a Bazzania species, but the affinity is unclear and the type specimen was burned in B (Grolle and Piippo 1984).

90

Solenostoma subdivisions are following Shaw et al. (2015), but the subgeneric placement of several species is preliminary and only based on morphological characteristics. Plectocolea is synonymous with Solenostoma, but one taxon has neither been transferred nor synonymized. It is listed in the “Names in genera not currently accepted” section below.

91

Solenostoma rupicola is conspecific with Solenostoma rosulans in Váňa (1975a), but accepted by Váňa and Long (2009).

92

Solenostoma appalachianum is placed in synonymy with Solenostoma pyriflorum by Schuster (1969b) but Shaw et al. (2015) shows that the North American specimens differs from the East Asiatic specimens so named.

93

Solenostoma appressifolium seems to be a species complex (Shaw et al. 2015).

94

Solenostoma novaezelandiae is possibly conspecific with Solenostoma orbiculatum (Engel and Glenny 2008a).

95

Solenostoma pyriflorum is a species complex (Shaw et al. 2015).

96

Solenostoma pyriflorum var. minutissimum was treated as a separate species, Solenostoma rishiriense Amakawa by Bakalin and Vilnet (2009).

97

Solenostoma totopapillosum is possibly conspecific with Solenostoma inundatum (Shaw et al. 2015).

98

Solenostoma ventroversum is possibly conspecific with Solenostoma appressifolium.

99

Herbertus includes Schisma, but a few taxa have neither been transferred nor synonymized. They are listed in the “Names in genera not currently accepted” section below.

100

Herbertus delavayi is a species complex that requires further studies (Bell et al. 2012). It also includes Herbertus borealis and may include several, partly cryptic species.

101

Herbertus juniperoideus is here treated with two subspecies, but they may deserve species rank instead.

102

Herbertus subrotundus does not belong in Herbertus (Juslén 2006a), but it is unclear where it belongs.

103

Lepicolea rara is possibly a species complex and warrants further investigation (Schuster 2000a).

104

Acromastigum is here divided into two subgenera which are supported by recent molecular studies (Heslewood and Brown 2007, Cooper et al. 2011).

105

Acromastigum echinatum may be conspecific with Acromastigum inaequilaterum since all specimens identified by Piippo et al. (2002) belong there, but she did not see the type specimen.

106

Bazzania can not be subdivided based on recent molecular studies, so infrageneric taxa are not used here. The genus includes Mastigobryum, but several taxa have neither been transferred nor synonymized. They are listed in the “Names in genera not currently accepted” section below.

107

Bazzania asymmetrica is conspecific with Bazzania macgregorii in Grolle (1968a), but Kitagawa (1979a) kept them separate.

108

Bazzania corbieri may be conspecific with Bazzania crenata (Meagher 2008).

109

Bazzania crenata from Europe has neither been recognized in any recent treatment nor synonymized. The name may have priority once the identity is established.

110

Bazzania decrescens is a species complex also including also Bazzania comorensis, Bazzania curvidens and Bazzania mascarena (Grolle 1995).

111

Bazzania distans is probably conspecific with Bazzania loricata (Meijer 1960).

112

Bazzania lehmanniana is possibly conspecific with Bazzania arcuata or Bazzania longa (Meagher 2012).

113

Bazzania serpentina is possibly conspecific with Bazzania bidens (Frahm et al. 1990).

114

Bazzania sikkimensis is possibly conspecific with Bazzania sumbavensis (Long and Grolle 1990).

115

Bazzania sumbavensis is possibly conspecific with Bazzania japonica (Söderström et al. 2010a).

116

Bazzania wallichiana seems to be a species complex also including Bazzania francana, Bazzania intermedia and Bazzania marginata (Meagher 2010).

117

Bazzania wattsiana is possibly conspecific with Bazzania tridens (Meagher 2010).

118

Mastigopelma may be better placed in Bazzania, but it is retained here until further studied.

119

Kurzia cucullifolia is of doubtful status (Engel 1978).

120

Lepidozia seems to be a monophyletic genus, but its infrataxonomy still has to be tested using molecular data. Grolle and Piippo (1984) could not study Lepidozia cordistipula and Lepidozia palmicola since the types were destroyed in B.

121

Lepidozia brasiliensis is conspecific with Kurzia brasiliensis in Pócs (1984b), possibly conspecific with Lepidozia cupressina in Gradstein and Costa (2003), but it has later been accepted by many authors (e.g. Schäfer-Verwimp and Pócs 2009)

122

Lepidozia massartiana has not been studied recently. Stephani (1909a) confused it with Telaranea cuneifolia (Söderström et al. 2010a).

123

Lepidozia minor may be conspecific with a known taxon. Lepidozia minor sensu Solari and Hässel (1983) is probably another taxon perhaps without name.

124

Lepidozia pallida is possibly Lepidozia chordulifera since all specimens cited by Stephani (1911b) and examined by Engel (1978) belong to this species, except the supposed type specimen which does not contain any Lepidozia material.

125

Lepidozia paucifolia is conspecific with Lepidozia fauriana in del Rosario (1977), but Tan and Engel (1986) kept them apart.

126

Lepidozia hexiloba is possibly an Arachniopsis or Telaranea species (Wigginton and Grolle 1996).

127

Lepidozia parvistipa is possibly conspecific with Ceramanus clatritexta.

128

Micropterygium trachyphyllum was split into several varieties by Reimers (1933), but they do not seem to have been accepted by any later author and Söderström et al. (2011a) were not sure about their status.

129

Telaranea is a heterogeneous genus and several segregates are warranted. Additional studies are needed to clarify the relationships of the species retained here (Cooper 2013).

130

Zoopsis ciliata was not studied by Engel and Glenny (2008a) and they did not know what it may be.

131

Chiloscyphus pallescens var. fragilis is a problematic taxon sometimes treated as a species, sometimes as conspecific with Chiloscyphus pallescens, sometimes as conspecific with Chiloscyphus polyanthos.

132

Chiloscyphus polyanthos var. rivularis is a problematic taxon sometimes treated as a species, sometimes as conspecific with Chiloscyphus polyanthos.

133

Chiloscyphus is here treated in a very narrow sense (cf. Söderström et al. 2013b), but a large number of species remain to be assigned to other genera. It is unclear if the taxa included here belong to Cryptolophocolea, Lophocolea or some other genus in Lophocoleaceae.

134

Chiloscyphus bifidus is possibly conspecific with Heteroscyphus aselliformis (Schiffner 1900a).

135

Chiloscyphus durus is conspecific with Leptoscyphus expansus in Grolle (1962a), but it probably belongs to one of its segregates. It was accepted by Hässel (1999).

136

Clasmatocolea is here treated without subdivisions although Engel (1980a, 2015) did so.

137

Cryptolophocolea levieri is possibly conspecific with Cryptolophocolea ciliolata (Söderström et al. 2010a).

138

Cryptolophocolea lilliena is possibly conspecific with Cryptolophocolea martiana (Wigginton and Grolle 1996).

139

Cryptolophocolea massalongoana is possibly conspecific with Cryptolophocolea costata (Piippo 1985a).

140

Cryptolophocolea stephanii is doubtfully distinct from Cryptolophocolea ciliolata (Söderström et al. 2010a).

141

Cryptolophocolea thermarum is possibly conspecific with Cryptolophocolea ciliolata (Söderström et al. 2010a).

142

Cryptolophocolea whittieriana is possibly conspecific with Cryptolophocolea helmsiana (Hodgson 1967).

143

Heteroscyphus caesius is possibly conspecific with Heteroscyphus splendens (Söderström et al. 2010a).

144

Heteroscyphus caledonicus is possibly conspecific with Heteroscyphus amboinensis Miller et al. (1983) with a question mark.

145 The subdivision of Leptoscyphus follows Vanderpoorten et al. (2010).

146

Leptoscyphus difficilis is conspecific with Leptoscyphus expansus in Grolle (1962a), but it was accepted by Vanderpoorten et al. (2010).

147

Leptoscyphus huidobroanus is conspecific with Leptoscyphus expansus in Váňa and Engel (2013) rejecting the conclusion by Hässel (2001) that it is an independent species, but it was accepted by Vanderpoorten et al. (2010).

148

Lophocolea bidentata is a species complex as discussed by Váňa and Engel (2013) and it has a complicated nomenclatural history causing many misunderstandings. At least Lophocolea coadunata and Lophocolea humifusa also belong to the complex.

149

Lophocolea coadunata belongs to the Lophocolea bidentata species complex with complicated nomenclature (Váňa and Engel 2013).

150

Lophocolea microstipula is possibly conspecific with Lophocolea sabuletorum (Engel 1978), but it was accepted by Hässel and Rubies (2009).

151

Lophocolea mollis is possibly conspecific with Lophocolea difformis (Grolle 1995).

152

Lophocolea morobeana is conspecific with Chiloscyphus kurzii in Piippo (1987), but it was accepted as very close to Chiloscyphus kurzii by Ariyanti et al. (2009).

153

Lophocolea rectangulata may be a Cryptolophocolea species.

154

Lophocolea semiteres is a species complex also including Lophocolea platensis and Lophocolea undulata.

155

Pachyglossa exilis may be a Chiloscyphus species.

156

Mastigophora includes (Mastigophora appendiculata, Mastigophora guineensis, Mastigophora pyramidana and Mastigophora valida) which Grolle and Piippo (1984) could not study since the types were destroyed in B.

157

Plagiochila arnelliana may be conspecific with Plagiochila bifaria (Heinrichs et al. 1998).

158

Plagiochila fragilis may be conspecific with Plagiochila bifaria (Heinrichs et al. 1998).

159

Plagiochila integrilobula is possibly conspecific with Plagiochila blepharophora (Söderström et al. 2010a).

160

Plagiochila kuhliana is possibly conspecific with Plagiochila sciophila (Söderström et al. 2010a).

161

Plagiochila stephanii is possibly conspecific with Plagiochila blepharophora (Söderström et al. 2010a).

162

Plagiochila fragmentissima may be conspecific with Plagiochila gregaria (M.L. So and D. Glenny, pers. comm.).

163

Plagiochila cuneata is conspecific with Plagiochila bursata in Inoue (1980), but accepted by Grolle and Heinrichs (1999).

164

Plagiochila steyermarkii is conspecific with Plagiochila aerea in Inoue in Gradstein and Hekking (1979), but this was rejected by Grolle and Heinrichs (1999).

165

Plagiochila aspera is conspecific with Plagiochila trigona Steph. (=Plagiochila metcalfii) in Inoue (1970b), but it was accepted by So (2000a, 2001a).

166

Plagiochila heterostipa is possibly conspecific with Plagiochila corymbulosa (Jones 1962).

167

Plagiochila loloënsis is probably an entire-leaved form of Plagiochila moenkemeyeri (Wigginton and Grolle 1996).

168

Plagiochila moenkemeyeri is possibly conspecific with Plagiochila winteri (Jones 1962), but he expressed some doubts on the synonymy.

169

Plagiochila owaihiensis was considered conspecific with Plagiochila remyana by Inoue (1976c), but So (2000a) did not agree.

170

Plagiochila aliena is conspecific with Plagiochila ludoviciana in Schuster (1960c), but others (e.g. Fulford and Sharp 1990, Heinrichs et al. 1998, Dauphin 2005) recognize it.

171

Plagiochila borneensis is a doubtful taxon. No specimen could be found by Inoue (1984b) or So and Grolle (2000a).

172

Plagiochila distans is a doubtful taxon. The type specimen could not be found (Hodgson 1944, Hamlin 1972).

173

Plagiochila exilis is a doubtful taxon. The type specimen could not be found (Hodgson 1944, Hamlin 1972).

174

Plagiochila glauca is a doubtful taxon. Hässel and Rubies (2009) could not find any type material.

175

Plagiochila karstenii is possibly conspecific with Plagiochila bantamensis (So and Grolle 2000a).

176

Plagiochila lecontei is allied to and perhaps conspecific with Plagiochila ledieui and Plagiochila multiflora (Jones 1962).

177

Plagiochila ledermanniana is a doubtful taxon. The type specimen was burned in B (Piippo 1989b).

178

Plagiochila ledieui is allied to and perhaps conspecific with Plagiochila lecontei and Plagiochila multiflora (Jones 1962).

179

Plagiochila multiflora is allied to and perhaps conspecific with Plagiochila lecontei and Plagiochila ledieui (Jones 1962).

180

Plagiochila schraderbergii is a doubtful taxon. The type specimen was burned in B (Piippo 1989b).

181

Plagiochila scotica was described from Europe, but it has neither been recognized in any recent European study nor synonymized.

182

Plagiochila vetustisilva is a doubtful taxon. The type specimen was burned in B (Piippo 1989b).

183

Trichocolea floccosa is treated as a variety of Trichocolea flaccida (=Leiomitra flaccida by Schultze-Motel & Menzel (1987).

184

Trichocolea sprucei is suggested to be a variety of Trichocolea flaccida (=Leiomitra flaccida) in Schultze-Motel & Menzel (1987)

185

Schistochila baileyana is possibly conspecific with Schistochila beccariana (D. Meagher, pers. comm.).

186

Frullania trinervis is possibly a species complex (Vanden Berghen 1976b).

187

Frullania claviloba is possibly conspecific with Frullania gracilis (Söderström et al. 2010a).

188

Frullania cordistipula is a species complex also including Frullania serrata.

189

Frullania crenatiloba is conspecific with Frullania apiculata in Verdoorn (1930c), but it was accepted by So and Wang (2006).

190

Frullania multilacera is possibly conspecific with Frullania vaga.

191

Frullania van-zantenii is possibly conspecific with Frullania serrata.

192

Frullania brunea belongs to the Frullania lindenbergii complex (Vanden Berghen 1976b).

193

Frullania tricarinata is possibly conspecific with Frullania hypoleuca (Söderström et al. 2010a).

194

Frullania contracta is conspecific with Frullania squarrosa in Verdoorn (1930c), but it was accepted by Hattori (1986e).

195

Frullania cornuta is closely related to the very variable Frullania ornithocephala.

196

Frullania flexuosa is possibly conspecific with Frullania rubella.

197

Frullania giraldiana belongs to the Frullania nepalensis complex (Hattori 1973b).

198

Frullania hebridensis belongs to the Frullania ericoides species complex (Verdoorn 1930c, 1930a).

199

Frullania longistyla is closely related to Frullania brevicalycina (Yuzawa and Hattori 1988b).

200

Frullania ludoviciae seems to be close to Frullania pusilla (Hattori 1986e).

201

Frullania montana is possibly conspecific with Frullania reflexistipula.

202

Frullania nepalensis is a species complex (Hattori 1973b).

203

Frullania subpedicellata is possibly conspecific with Frullania pedicellata.

204

Frullania baladina is a species complex also including Frullania fulfordiae.

205

Frullania fugax is a species complex possibly including Frullania baileyana, Frullania belmorensis, Frullania cataractarum, Frullania media and Frullania subtropica (Hattori 1979a).

206

Frullania solanderiana is morphologically similar to Frullania pentapleura and possibly conspecific.

207

Frullania tjibodensis is possibly conspecific with Frullania campanulata (Söderström et al. 2010a).

208

Frullania eboracensis is a species complex also including Frullania appalachiana, Frullania parvistipula and Frullania virginica.

209

Frullania ericoides is enormously variable (Schuster 1992b) and is a species complex with at least two lineages (Hentschel et al. 2009).

210

Frullania astrolabea is possibly conspecific with Frullania scandens (Hattori 1981b).

211

Frullania intermedia is a species complex (Hattori 1980a).

212

Frullania intermedia var. non-apiculata was reduced to a synonym of subsp. intermedia by Hattori (1980a), but it was re-instated by Hattori (1985).

213

Frullania brotheri is probably just a high-elevation form of Frullania nodulosa (Söderström et al. 2010a).

214

Frullania brasiliensis is a species complex with controversial synonymy (Schuster 1992b, Hentschel et al. 2009).

215

Frullania breuteliana is possibly conspecific with Frullania beyrichiana.

216

Frullania compacta is conspecific with Frullania brasiliensis in Stotler (1969), but Schuster (1992b) doubts the correctness of this.

217

Frullania granatensis is possibly conspecific with Frullania beyrichiana, but the type specimen was lost in B (Stotler 1969).

218

Frullania borbonica is possibly conspecific with Frullania apicalis (Vanden Berghen 1976b).

219

Frullania schimperi belongs to a species complex also including Frullania onraedtii, Frullania capensis, Frullania apicalis, Frullania imerinensis and Frullania meyeniana (Vanden Berghen 1976b).

220

Frullania chevalieri is a species complex (Hattori 1984a).

221

Frullania junghuhniana is a species complex (Hattori 1976b).

222

Frullania rostrata is a species complex with several lineages, some of which have been recently described (e.g. von Konrat et al. 2013).

223

Frullania acicularis was shown to be a species separate from Frullania tamarisci (Heinrichs et al. 2010, Vilnet et al. (2014). It may, however, not be the earliest available name.

224

Frullania appendiculata was shown by Vilnet et al. (2014) to be a species separate from Frullania tamarisci. It may, however, not be the earliest available name.

225

Frullania asagrayana consists of two geographically separated clades (Ramaiya et al. 2010).

226

Frullania calcarifera was shown to be a species separate from Frullania tamarisci by Heinrichs et al. (2010) and Vilnet et al. (2014). It may, however, not be the earliest available name.

227

Frullania moniliata is a species complex (Vilnet et al. 2014).

228

Frullania subarctica was shown to be a species separate from the Frullania tamarisci complex (Vilnet et al. 2014), but it is maybe not the oldest name.

229

Frullania affinis is possibly conspecific with Frullania ecklonii (Arnell 1963b).

230

Jubula hutchinsiae has subspecies that may be better recognized at the species level.

231

Jubula hutchinsiae subsp. bogotensis is closely related to subsp. pennsylvanica (Pätsch et al. 2010).

232

Jubula hutchinsiae subsp. javanica includes a cryptic taxon from China (Pätsch et al. 2010).

233 The treatment of Ceratolejeunea follows Dauphin (2003) except that his subg. Caduciloba becomes subg. Ceratolejeunea and his subg. Ceratolejeunea becomes subg. Ceratophora as a consequence of the introduction of ICN Art. 41.4 (cf. Söderström et al. 2015a).

234

Ceratolejeunea kuerschneri is very similar to Ceratolejeunea papuliflora and may be conspecific with it.

235

Ceratolejeunea sinensis may be conspecific with Drepanolejeunea erecta (Zhu et al. 2005).

236

Otigoniolejeunea crenulata is a doubtful taxon. The type specimen was burned in B and it is unclear where the taxon belongs (Grolle and Piippo (1984).

237

Otigoniolejeunea ledermannii is a doubtful taxon. The type specimen was burned in B and it is unclear where the taxon belongs (Grolle and Piippo (1984).

238

Aureolejeunea is nested in Cheilolejeunea (Ye et al. 2011, Gradstein 2013a, Ye et al. 2015). A formal transfer of the genus and its species to Cheilolejeunea is found in Ye et al. (2015).

239

Cheilolejeunea is here organized into subgenera that are maintained for practical purposes. They may not be natural lineages. An improved infrageneric classification based on molecular analysis is in preparation (Ye et al. 2015). The genus includes Euosmolejeunea, Strepsilejeunea and Trachylejeunea, but several taxa have neither been transferred nor synonymized. They are listed under “Names in genera not currently accepted” below.

240

Cheilolejeunea fukiensis may be conspecific with Lejeunea flava (Zhu and So 2001), but they did not see the type specimen.

241

Cheilolejeunea laurentii is not a Cheilolejeunea species (Jones 1954b, Wigginton and Grolle 1996). We do not know where to refer it.

242

Cheilolejeunea longiflora is possibly conspecific with Cheilolejeunea trifaria (Gradstein and Costa 2003).

243

Cheilolejeunea micholitzii is conspecific with Cheilolejeunea longidens in Thiers (1992b), but accepted by Hürlimann (1995).

244

Cheilolejeunea ulugurica is possibly conspecific with Cheilolejeunea chenii (Ye et al. 2013a).

245

Cheilolejeunea udarii is possibly conspecific with Cheilolejeunea krakakammae (Zhu 2006a).

246

Cheilolejeunea heteroclada was considered conspecific with Cheilolejeunea aneogyna by X.-L. He (in Gradstein and Costa 2003), but it was tentatively accepted by Reiner-Drehwald and Grolle (2012). Lejeunea heteroclada var. subandina Spruce may be a different taxon (cf. Reiner-Drehwald & Grolle 2012).

247

Omphalanthus is nested in Cheilolejeunea (Wilson et al. 2007, Ye et al. 2011, Gradstein 2013a). A formal transfer of the genus and its species to Cheilolejeunea is found in Ye et al. (2015).

248

Cololejeunea is here organized into subgenera that are not supported by the molecular study of Yu et al. (2013). They are referred to following the traditional use (e.g. as in Pócs et al. 2014). Leptocolea and Physocolea also belong here, but a few taxa have neither been transferred nor synonymized. They are listed in the “Names in genera not currently accepted” section below.

249

Cololejeunea rotundilobula is possibly conspecific with Cololejeunea sigmoidea (Pócs et al. 2013).

250

Cololejeunea subg. Chlorolejeunea forms a separate clade in Yu et al. (2013) with three sequenced species (Cololejeunea ceratilobula, Cololejeunea madothecoides (the type) and Cololejeunea raduliloba).

251

Cololejeunea subg. Chondriolejeunea is supported by Yu et al. (2013) who included Cololejeunea chinii, Cololejeunea pseudostipulata (the type, not sequenced) and Cololejeunea shimizui.

252 Subg. Cololejeunea includes subg. Aphanolejeunea in the phylogeny by Yu et al. (2013).

253

Cololejeunea frahmii is probably only a depauperate form of Cololejeunea elegans or Cololejeunea zenkeri.

254

Cololejeunea kahuziensis is probably only a depauperate form of Cololejeunea elegans or Cololejeunea zenkeri.

255

Cololejeunea drepanolejeuneoides may based on its description be close to Cololejeunea angustiflora and especially to Cololejeunea inflectens, forming a natural group.

256

Cololejeunea chamlongiana is possibly conspecific with Cololejeunea papuliflora.

257

Cololejeunea crenulata is possibly conspecific with Cololejeunea angustiflora judging from the protologue.

258

Cololejeunea dentilobula is possibly conspecific with Cololejeunea decliviloba judging from Stephani’s Icones (unpublished).

259

Cololejeunea effusa may be conspecific with Cololejeunea trichomanis.

260

Cololejeunea fleischeri is near to or conspecific with Cololejeunea bidentula.

261

Cololejeunea fredericii is possibly conspecific with Cololejeunea polyantha.

262

Cololejeunea kohkongensis may be conspecific with Cololejeunea plagiophylla.

263

Cololejeunea lobulilineata seems to be a depauperate form of some other species, maybe Cololejeunea obtusifolia.

264

Cololejeunea micronesica is possibly conspecific with Cololejeunea cookei *fide* Schuster (1980c, 1983a).

265

Cololejeunea ninguana is possibly conspecific with Cololejeunea decliviloba (Yu et al. 2013).

266

Cololejeunea oblongiperianthia is possibly conspecific with Cololejeunea trichomanis (Zhu and So 2001).

267

Cololejeunea pteroporum is possibly conspecific with Cololejeunea spathulifolia.

268

Cololejeunea spathulifolia is possibly conspecific with Cololejeunea obliqua (Pócs and Piippo 2011).

269

Cololejeunea takamakae is possibly conspecific with Cololejeunea angustiflora.

270

Cololejeunea tuksapiana is possibly conspecific with Cololejeunea aequalis.

271

Cololejeunea brunelii is conspecific with Cololejeunea madothecoides in Tixier (1985a), but it belongs to the Cololejeunea raduliloba, Cololejeunea furcilobulata, Cololejeunea paucimarginata species complex.

272

Cololejeunea chittagongensis is probably conspecific with Cololejeunea schwabei.

273

Cololejeunea deslooveri is possibly conspecific with Cololejeunea cristata.

274

Cololejeunea dzumacensis is possibly conspecific with Cololejeunea lanciloba, but the type specimen has not been localized.

275

Cololejeunea epiphylla is closely related to Cololejeunea chittagongensis and Cololejeunea schwabei.

276

Cololejeunea hoabinhiana is possibly conspecific with Cololejeunea lanciloba.

277

Cololejeunea malaccensis may be conspecific with Cololejeunea stylosa.

278

Cololejeunea punctata is a doubtful taxon. The type specimen has been destroyed and the identity is unclear (Grolle 1995).

279

Cololejeunea tribracteata is possibly conspecific with Cololejeunea africana.

280

Cololejeunea uchimae is conspecific with Cololejeunea raduliloba in Mizutani (1961), but it is accepted in recent Japanese checklists.

281

Cololejeunea chuahiana is probably a Myriocoleopsis species (Yu et al. 2014).

282

Cololejeunea dauphinii is probably a Myriocoleopsis species (Yu et al. 2014).

283

Cololejeunea disciflora is probably a Myriocoleopsis species (Yu et al. 2014).

284

Cololejeunea amoena is possibly conspecific with Cololejeunea floccosa (Söderström et al. 2010a).

285

Cololejeunea bontocensis is possibly conspecific with Cololejeunea pseudostephanii.

286

Cololejeunea flavida may be conspecific with Cololejeunea peraffinis (Zhu and So 2001).

287

Cololejeunea polisiana may be conspecific with Cololejeunea peraffinis.

288

Cololejeunea conchifolia is probably a Myriocoleopsis species (Yu et al. 2014).

289

Diplasiolejeunea is here only provisionally accepted with subgenera following Schuster (1970b), but they are not supported by the molecular phylogeny by Dong et al. (2012) where the type species of subg. Austrolejeuneopsis and Physolejeunea are nested in subg. Diplasiolejeunea.

290

Diplasiolejeunea guadalupensis is a poorly known taxon with some similarities to the Diplasiolejeunea replicata species complex (Schäfer-Verwimp and Reiner-Drehwald 2009).

291

Diplasiolejeunea heimii is possibly conspecific with Diplasiolejeunea replicata (Schäfer-Verwimp and Pócs 2009).

292

Diplasiolejeunea montecristensis is possibly conspecific with Diplasiolejeunea replicata.

293

Diplasiolejeunea rudolphiana and Diplasiolejeunea unidentata are genetically very close and may be conspecific (Dong et al. 2012).

294

Diplasiolejeunea glaziovii may be conspecific with Diplasiolejeunea cavifolia (Dong et al. 2012).

295

Diplasiolejeunea evansii is a doubtful taxon and the type specimen could not be found in PC (Schäfer-Verwimp 2004).

296

Diplasiolejeunea integerrima is a juvenile Acrolejeunea species, but the type is too poor to allow identification (Grolle 1995).

297

Prionolejeunea corbisieri is a Cheilolejeunea species (Ilkiu-Borges 2006).

298

Prionolejeunea maculata is a Cyclolejeunea species (Ilkiu-Borges 2006).

299

Drepanolejeunea deslooveri was treated as conspecific with Drepanolejeunea hamatifolia by Tixier (1995b) and accepted uncritically as such by Wigginton and Grolle (1996) and Wigginton (2002). However, no published supporting evidence for the synonymy has been found, and it was therefore reinstated pending further studies (Wigginton 2009).

300

Drepanolejeunea lancifolia resembles Drepanolejeunea bidens and Drepanolejeunea araucariae, but the type material has not been found (Bischler 1964).

301

Drepanolejeunea obliqua is possibly conspecific with Drepanolejeunea ternatensis (Söderström et al. 2010a).

302

Drepanolejeunea ruandensis is conspecific with Drepanolejeunea cultrella in Vanden Berghen (1972a), but it was accepted by Tixier (1995b) and Wigginton and Grolle (1996).

303

Drepanolejeunea tenera is possibly conspecific with Drepanolejeunea pentadactyla (Söderström et al. 2010a).

304

Drepanolejeunea thwaitesiana is a species complex also including Drepanolejeunea hampeana, Drepanolejeunea longicornua, Drepanolejeunea nymannii and Drepanolejeunea serricalyx.

305

Drepanolejeunea integerrima is a Leptolejeunea species (Bischler 1964), but it is probably a synonym since she did not transfer it.

306

Harpalejeunea acuta is possibly conspecific with Harpalejeunea cinchonae (Grolle and Reiner-Drehwald 1999).

307

Harpalejeunea tenuicuspis is possibly conspecific with Harpalejeunea oxyphylla (Gradstein and Costa 2003, Grolle and Reiner-Drehwald 1999).

308

Harpalejeunea grossearmata is a Lejeunea species (Grolle and Reiner-Drehwald 1999). It was treated as a doubtful taxon by Gradstein et al. (2003).

309

Harpalejeunea renneri is a Lejeunea species and most likely conspecific with something (Grolle and Reiner-Drehwald 1999).

310

Harpalejeunea spruceana is a Lejeunea species (non Lejeunea spruceana C.Massal. 1885) (Grolle and Reiner-Drehwald 1999). It was treated as a doubtful taxon by Gradstein et al. (2003).

311

Harpalejeunea vitrea is a Lejeunea species (non Lejeunea vitrea Nees, Lindenb. et Gottsche 1843) (Grolle and Reiner-Drehwald 1999).

312

Lejeunea is a large genus and its infrageneric classification, which has not been studied in a rigorous manner, needs investigation although Heinrichs et al. (2013) showed two main clades on molecular grounds, provisionally named Lejeunea subg. Lejeunea and Lejeunea subg. Crossotolejeunea. However, their morphology is not yet understood. The subgenera listed here are recently accepted ones. Crossotolejeunea, Eulejeunea, Hygrolejeunea and Taxilejeunea also belong here, but several taxa have neither been transferred nor synonymized. They are listed in the “Names in genera not currently accepted” section below.

313

Lejeunea adpressa from South America is very similar to the palaeotropical Lejeunea anisophylla. The two taxa probably represent a single pantropical species (possibly with two subspecies).

314

Lejeunea angulifolia may be conspecific with Lejeunea cocoes (Pócs 2011a).

315

Lejeunea caroliniana was poorly described and the type specimen so poor that Evans (1902a) could not identify it.

316

Lejeunea flava is a species complex also including Lejeunea grossecristata.

317

Lejeunea lamacerina forms a North American and a Macaronesian/European clade and the separation of two subspecies are justified if not treating them at species level (Heinrichs et al. 2013).

318

Lejeunea leratii is probably distinct from Lejeunea mimula (Lee 2013).

319

Lejeunea obscura is possibly conspecific with Lejeunea aloba (Söderström et al. 2010a).

320

Lejeunea pallidissima is possibly conspecific with Lejeunea flavovirens (Vanden Berghen 1972b).

321

Lejeunea paratropa is closely related to or perhaps conspecific with Lejeunea raddiana Lindenb. (Grolle and Reiner-Drehwald 1999).

322

Lejeunea rara is the valid name for Lejeunea sinclairii Spruce (1884) hom. illeg. (non Mitten 1862 = Thysananthus fruticosus), but Scott and Bradshaw (1985) confused the taxa and considered them conspecific. Grolle (1982) accepted Spruce’s taxon without commenting on any homonym problem.

323

Lejeunea ravenelii was poorly described and the type specimen so poor that Evans (1902a) could not identify it.

324

Lejeunea stephaniana is doubtfully distinct from Lejeunea discreta (Söderström et al. 2010a).

325

Lejeunea emarginuliflora is a Lejeunea (subg. Heterolejeunea) species (Reiner-Drehwald and Grolle 2012).

326

Lejeunea oerstediana is a poorly known taxon often placed in Euosmolejeunea (=Cheilolejeunea). We can not tell where it belongs until the type specimen is re-studied (Söderström et al. 2011a).

327

Lejeunea elegans is a Cheilolejeunea species (Reiner-Drehwald 2006).

328

Lejeunea hieronymii is a Cheilolejeunea species of uncertain status.

329

Lejeunea proboscidea is a Drepanolejeunea species, but Bischler (1964) did not place it to any species.

330

Lejeunea scabriflora was rejected from Dicranolejeunea by Kruijt (1988), but he did not refer it to any other genus.

331

Lejeunea trochantha is a Cheilolejeunea species (Reiner-Drehwald 2006).

332

Microlejeunea cochlearifolia is conspecific with Microlejeunea kamerunensis in Jones (1969), but he used the younger name Microlejeunea kamerunensis Steph. 1915.

333

Microlejeunea lunulatiloba is conspecific with Microlejeunea ulicina in Schuster (1962a), but that is not followed by many recent authors.

334

Microlejeunea minutistipula is probably either Microlejeunea cochlearifolia or Microlejeunea africana (Jones 1969).

335

Microlejeunea ovistipula is possibly conspecific with Lejeunea hepaticola (Jones (1985, Wigginton and Grolle 1996).

336

Microlejeunea punctiformis was treated as conspecific with Microlejeunea ulicina by Long and Grolle (1990), Fang et al. (1998) and Piippo (1990), but Zhu and So (2001) showed that it merits distinction.

337

Microlejeunea szechuanensis may be conspecific with Microlejeunea punctiformis.

338

Lepidolejeunea includes Kingiolejeunea, but one taxon has neither been transferred nor synonymized. It is listed in the “Names in genera not currently accepted” section below.

339

Rectolejeunea lindenbergii is a Lejeunea species (Reiner-Drehwald and Grolle 2012; non Lejeunea lindenbergii Gottsche). However, it may be conspecific with something else.

340

Rectolejeunea lindigiana is a Lejeunea species of doubtful status (Reiner-Drehwald and Grolle 2012).

341

Rectolejeunea pachyderma is a Lejeunea species of doubtful status (Reiner-Drehwald and Grolle 2012).

342

Leptolejeunea borneensis may be conspecific with Leptolejeunea vitrea.

343

Leptolejeunea curvatifolia is possibly conspecific with Drepanolejeunea thwaitesiana, but the type specimen was burned in B and the identity can not be ascertained (Grolle and Piippo 1984).

344

Leptolejeunea emarginata is possibly conspecific with Leptolejeunea apiculata (Zhu and So 2001).

345

Leptolejeunea massartiana is very possibly conspecific with Leptolejeunea elliptica judging from the original description and illustration (Söderström et al. 2010a).

346

Leptolejeunea revoluta may be conspecific with Leptolejeunea elliptica (Zhu and So 2001).

347

Leptolejeunea udarii is possibly conspecific with Leptolejeunea latifolia (He et al. 2013).

348

Pycnolejeunea anotomensis is a Cheilolejeunea species.

349

Pycnolejeunea cavistipula is possibly a Cheilolejeunea species.

350

Pycnolejeunea convexifolia is possibly conspecific with Cheilolejeunea imbricata.

351

Pycnolejeunea grossiloba is a Cheilolejeunea species, but its taxonomic status is unclear (Grolle and Piippo 1984).

352

Pycnolejeunea palmicola is not a Pycnolejeunea species.

353

Pycnolejeunea schlimiana is not a Pycnolejeunea species.

354

Acrolejeunea abnormis was excluded from Acrolejeunea by Gradstein (1975), but the type specimen was not localized.

355

Acrolejeunea comptonii is a Mastigolejeunea species (Gradstein 1975). The type specimen has not been localized.

356

Acrolejeunea inflexa was excluded from Acrolejeunea by Gradstein (1975), but the type specimen was not localized.

357

Archilejeunea is polyphyletic and several nomenclatural and taxonomic will be proposed (Shi et al. 2015a).

358

Archilejeunea bilabiata is a doubtful taxon, the type specimen could not be found (Geissler and Gradstein 1994).

359

Archilejeunea planifolia is conspecific with Archilejeunea kiushiana in Hattori (1952c) and Gradstein and Geissler (1997), but it is a separate species in Mizutani (1993).

360

Archilejeunea negrensis is probably not an Archilejeunea species, but the type specimen is too poor to permit identification (Gradstein and Buskes 1985).

361

Archilejeunea ovata is conspecific with Lopholejeunea nigricans or Lopholejeunea subfusca (Gradstein 1994).

362

Bryopteris fissiloba is probably conspecific with Bryopteris diffusa (Gradstein and Costa 2003). It was not accepted by Hartmann et al. (2006).

363

Lopholejeunea proxima is possibly conspecific with Lopholejeunea nigricans (Zhu and Gradstein 2005).

364

Lopholejeunea yapensis is possibly conspecific with Lopholejeunea subfusca (Mizutani 1985).

365

Lopholejeunea aberrantia is probably an Archilejeunea species, but the type specimen in HIRO is destroyed (Zhu and Gradstein 2005).

366

Lopholejeunea vietnamica is probably an Archilejeunea species (Zhu and Gradstein 2005). The type specimen was not found in PC.

367

Mastigolejeunea humilis has been treated as conspecific with Mastigolejeunea auriculata by many recent authors, but it merits recognition based on molecular and morphological evidence (Sukkharak et al. 2011).

368

Ptychanthus stephensonianus was tentatively treated as a separate species by Thiers and Gradstein (1989). Further work on the infraspecific variation of Ptychanthus striatus is needed to determine the correct status.

369

Lepidolaena includes Polyotus, but a few taxa have neither been transferred nor synonymized. They are listed in the “Names in genera not currently accepted” section below.

370

Porella acutifolia var. linguifolia was accepted by So (2002a), but it is conspecific with Plagiochila viridissima in Hattori (1976f, 1986d). This synonymy is doubtful and further study is necessary to clarify the status.

371

Madotheca andica was transferred to Porella by Hässel (in Hässel and Rubies 2009), but she did not study the type specimen. It may be conspecific with Plagiochila subsquarrosa.

372

Porella caespitans was resolved as polyphyletic in the phylogeny of Hentschel et al. (2007b), suggesting that the species is a complex deserving further study.

373

Porella capehorniensis may be conspecific with Plagiochila setigera and the type specimen is probably not from South America (Hässel and Rubies 2009).

374

Porella densifolia was resolved as paraphyletic in the phylogeny of Hentschel et al. (2007b), suggesting that the species is a complex deserving further study.

375

Porella maxima was accepted by So (2002a), but it was treated as conspecific with Porella viridissima in Hattori (1976f, 1986d). This synonymy is doubtful and further study is necessary to clarify the status.

376

Porella oblongifolia is closely related to Porella densifolia (Hentschel et al. 2007b).

377

Porella stephaniana is closely related to Porella densifolia (Hentschel et al. 2007b).

378

Radula squarrosa is probably conspecific with Radula morobeana.

379

Radula bipinnata is distinct from Radula boryana according to molecular evidence (Devos et al. 2011).

380

Radula acuminata may be conspecific with Radula tjibodensis.

381

Radula tjibodensis is a doubtful taxon. The type specimen has not been found.

382

Radula crenulata is conspecific with Radula acuta in So (2006) and possibly conspecific with Radula apiculata in Yamada and Piippo (1989), but differs morphologically from both and needs to be further studied.

383

Radula acutiloba is tentatively placed in subg. Radula. The specimen labelled Radula acutiloba in Devos et al. (2011) was misidentified and should be Radula australiana.

384

Radula fendleri may be conspecific with Radula madagascariensis.

385

Radula multiflora is similar to, and may be conspecific with Radula reflexa, but it was accepted by Renner and de Lange (2009).

386

Radula oceania was considered conspecific with Radula javanica by So (2006), but molecular data does not support it (Renner 2014).

387

Radula decurrens may be conspecific with Radula reflexa.

388

Radula longispica may be conspecific with Radula javanica.

389

Radula paganii is doubtfully distinct from Radula neotropica.

390

Radula pinnulata was accepted by Yamada (1979b) and, indirectly through comparison with Radula norrisii, by Yamada and Piippo (1989). It is conspecific with Radula javanica according to So (2006).

391

Radula rupicola may be conspecific with Radula vieillardii (So 2006).

392

Herzogianthus sanguineus is possibly conspecific with Herzogianthus vaginatus.

393

Aneura includes also Acrostolia and Pseudoneura, but a few taxa have never been transferred nor synonymized. They are listed in the section “Names in genera not currently accepted” below. The types of Aneura augustae, Aneura hunsteinii, Aneura latemultifida, Aneura ledermannii, Aneura subledermannii and Aneura subtenerrima were destroyed in B and their identity is doubtful (Grolle and Piippo 1984).

394

Aneura crinita (type from Europe) has neither been recognized in any recent European treatment nor synonymized.

395

Aneura denticulata is possibly conspecific with Riccardia ciliolata (Hässel 2006b).

396

Aneura sharpii is conspecific with Aneura maxima in Schuster (1992b), but the taxa are not the same (D.G. Long, pers. comm.).

397

Aneura singalangana may be a form of Riccardia subexalata (Stephani 1899f).

398

Riccardia includes Acrostolia, but a few taxa have neither been transferred nor synonymized. They are listed under “Names in genera not currently accepted” below.

399

Riccardia albomarginata is possibly conspecific with Riccardia canaliculata (Söderström et al. 2010a).

400

Riccardia baumannii is possibly conspecific with Riccardia grossitexta or Riccardia tenuicostata (Furuki 1991, Söderström et al. 2010a).

401

Riccardia baldwinii is possibly conspecific with Riccardia flaccida (Miller et al. 1983).

402

Riccardia decolyana is possibly conspecific with Riccardia multifida (Srivastava and Udar 1976).

403

Riccardia diminuta is possibly conspecific with Riccardia elata (Söderström et al. 2010a).

404

Riccardia hirtiflora is possibly conspecific with Riccardia emarginata (Gradstein and Costa 2003).

405

Riccardia multifidoides is conspecific with Riccardia multifida in Meijer (1959), but that was not based on examination of type material (Söderström et al. 2010a).

406

Riccardia palmatiformis was treated as a doubtful taxon with some affinity with Riccardia levieri by Srivastava and Udar (1976).

407

Metzgeria was divided into many subdivisions proposed by Kuwahara in a series of publications, but they are artificial and not accepted here.

408

Metzgeria agnewiae is conspecific with Metzgeria consanguinea in So (2004), but it was accepted by Costa (2008).

409

Metzgeria ciliata is conspecific with Metzgeria furcata in Grolle (2002) and with Metzgeria dichotoma in Gradstein and Costa (2003), but it was accepted by Costa (2008).

410

Metzgeria conjugata is a complex taxon. Fuselier et al. (2009) demonstrated the occurrence of two clearly separated lineages, a northern North American lineage (possibly corresponding to Metzgeria conjugata s.str.) and a southern North American and European lineage (possibly corresponding to Metzgeria simplex).

411

Metzgeria foliicola is conspecific with Metzgeria albinea in Kuwahara (1986), but it was accepted by So (2002c).

412

Metzgeria furcata includes several cryptic species as demonstrated by Fuselier et al. (2009).

413

Metzgeria mexicana is conspecific with Metzgeria subundulata in Schuster (1992b), but it was accepted by Costa (2008).

414

Fossombronia crispula is based on the description most likely Fossombronia pusilla, but no material has been found.

415

Fossombronia gregaria is possibly conspecific with Fossombronia australis (Scott and Pike 1988b).

416

Fossombronia grossepapillata may be conspecific with Fossombronia papillata, but the type specimen is too immature to allow determination (Scott and Pike 1984).

417

Fossombronia integerrima is possibly conspecific with Fossombronia australis.

418

Fossombronia integrifolia is possibly conspecific with Fossombronia foveolata (Scott and Pike 1984).

419

Fossombronia nigricaulis is possibly conspecific with Fossombronia australis (Scott and Pike 1984).

420

Fossombronia subsaccata has a sterile and unidentifiable type specimen (Scott and Pike 1988b).

421

Jensenia crassifrons is possibly conspecific with Jensenia difformis.

422

Symphyogyna paucidens is a doubtful taxon. Hässel and Rubies (2009) could not find the type specimen.

423

Symphyogyna ulvoides is probably a filmy fern (Schiffner 1900a), but he did not see any type material.

424

Noteroclada longiuscula is neither a Noteroclada nor a Fossombronia species. The type specimen could not be found (Crandall-Stotler et al. 2010).

425

Lunularia cruciata subsp. thaxteri was synonymized with subsp. cruciata by Boisselier-Dubayle et al. (1995), but their subsp. thaxteri specimen was identified with some doubt and originated far from the known range.

426

Asterella includes Fimbraria and Hypenantron, but a few taxa have neither been transferred nor synonymized. They are listed in the “Names in genera not currently accepted” section below.

427

Asterella shimizuana is conspecific with Asterella khasyana in Grolle and Piippo (1984), but it was accepted by Long (2006).

428

Asterella tenerrima is probably conspecific with Asterella tenera.

429

Asterella caucasica is probably conspecific with Asterella whiteleggeana (Grolle 1975c, Scott and Bradshaw 1985).

430

Asterella dognyensis is possibly conspecific with Asterella heteroflora.

431

Asterella preussii was provisionally placed in synonymy with Asterella bachmannii (Wigginton and Grolle 1996).

432

Mannia perssonii is possibly conspecific with Mannia sibirica.

433

Mannia hegewaldii is possibly conspecific with Mannia triandra.

434

Mannia paradoxa is possibly a Reboulia species.

435

Plagiochasma megacarpon may not be a Plagiochasma species. Bischler (1979b) could not find any specimen and did not know where to refer it.

436

Sauteria japonica is possibly conspecific with Sauteria spongiosa.

437

Sauteria inflata is possibly conspecific with Sauteria alpina (Schuster 1992d).

438

Cyathodium spurium may be conspecific with Cyathodium cavernarum (Braithwaite 1878). The type specimen is from Scotland, but it is probably mislabelled as no Cyathodium is known from boreal Europe.

439

Dumortiera hirsuta is a species complex (Forrest et al. 2011).

440

Marchantia assamica is a doubtful taxon. Bischler (1989b) could not find the type specimen.

441

Marchantia balboi is a doubtful taxon. Bischler-Causse (1993) could not find the type specimen.

442

Marchantia balboi var. acutisquamata is a doubtful taxon, Bischler-Causse (1993) could not find the type specimen and did not know what it is.

443

Marchantia cagnii is a doubtful taxon. Bischler-Causse (1993) could not find the type specimen, but the description points to Marchantia paleacea.

444

Marchantia cengiana is a doubtful taxon. Bischler-Causse (1993) could not find the type specimen, but the description points to Marchantia pappeana.

445

Marchantia keniae is a doubtful taxon. Bischler-Causse (1993) could not find the type specimen, but the description points to Marchantia pappeana.

446

Marchantia papyracea is a doubtful taxon. Bischler-Causse (1993) could not find the type specimen, but the description points to Marchantia pappeana.

447

Marchantia sellae is a doubtful taxon. Bischler-Causse (1993) could not find the type specimen, but the description points to Marchantia pappeana or Marchantia paleacea.

448

Marchantia trilocularis is a doubtful taxon which Bischler-Causse (1993) could not place. The name may have priority once its identity is determined.

449

Marchantia tusui is a doubtful taxon. Bischler-Causse (1993) could not find the type specimen, but the description points to Marchantia pappeana.

450

Riccia ianthina seems identical to glabrous phases of Riccia atromarginata (Schuster 1992d). It is related to Riccia iodocheila and Riccia violacea (Bischler-Causse et al. 2005), but it is only known from the type specimen.

451

Riccia congoana is possibly conspecific with Riccia billardierei (Perold 1989a).

452

Riccia crustata may be conspecific with Riccia albida (Jovet-Ast 1986).

453

Riccia runssorensis is possibly conspecific with Riccia macrospora (Seppelt 1998).

454

Riccia bahiensis is conspecific with Riccia cavernosa in Jovet-Ast (1965), but it was accepted by Bischler-Causse et al. (2005). It is only known from the type specimen.

455

Riccia paranaensis may be conspecific with Riccia huebeneriana (Schuster 1992d), but it was accepted by Hässel and Rubies (2009).

456

Riccia bialbistrata is a doubtful taxon closely related to Riccia lindmanii (Bischler-Causse et al. 2005).

457

Riccia coronata is only known from the type specimen, which could not be traced. The description is very brief and it is suspected that it refers to smaller plants of Riccia natalensis (Perold 1999a).

458

Riccia gemmifera is possibly conspecific with Riccia atropurpurea (Perold 1999a).

459

Riccia intermedia (type from France) has neither been recognized in any recent treatment nor synonymized. The name may have priority once its identity is determined.

460

Riccia marginata is possibly conspecific with Riccia beyrichiana (Potemkin and Ahti 2012).

461

Riccia nigrescens is an Anthoceros species (Hässel and Rubies 2009).

462

Riccia obtusa may be conspecific with Riccia gangetica (Söderström et al. 2010a).

463

Riccia prominens may be conspecific with Riccia junghuhniana (Söderström et al. 2010a).

464

Riccia saharensis is possibly conspecific with Riccia argenteolimbata (Perold 1995).

465

Riccia tuberculata (type from France) has neither been recognized in any recent European treatment nor synonymized. The name may have priority once its identity is determined.

466

Riccia velenovskyi (type from Czech Republic) has neither been recognized in any recent European treatment nor synonymized. The name may have priority once its identity is determined.

467

Targionia dioica is probably conspecific with Targionia hypophylla (Söderström et al. 2010a).

468

Riella notarisii is a species complex with poorly defined taxonomic boundaries including Riella battandieri, Riella cyrenaica, Riella gallica, Riella indica, Riella reuteri and Riella sersuensis.

469

Sphaerocarpos europaeus is clearly distinct from Sphaerocarpos texanus (Bell et al. 2013).

470

Acrostolia is congeneric with Riccardia, but a few taxa have neither been transferred nor synonymized.

471

Aphanolejeunea lancifera is a Drepanolejeunea species (Pócs et al. 2014).

472

Aphanolejeunea minima seems to be close to Cololejeunea gracilis based on the drawing in the original description, but the type specimen (not available for study) seems to be very fragmentary, having only reduced leaves, so its identity is uncertain (Pócs and Bernecker 2009).

473

Aspiromitus is congeneric with Anthoceros, but some taxa have neither been transferred nor synonymized.

474

Crossotolejeunea is congeneric with Lejeunea, but one taxon has neither been transferred nor synonymized.

475

Crossotolejeunea curvifolia has doubtful status, the type specimen could not be found in G (Reiner-Drehwald and Goda 2000).

476

Eulejeunea is congeneric with Lejeunea subg. Lejeunea, but a few taxa have neither been transferred nor synonymized.

477

Eulejeunea subpililoba is morphologically similar to Lejeunea spiniloba.

478

Euosmolejeunea is here treated as a subgenus of Cheilolejeunea, but a few taxa have neither been transferred nor synonymized.

479

Euosmolejeunea tenerrima is a doubtful taxon described with three syntypes, two from Brazil and one from Java. The Java material is probably not the same species as the material from Brazil.

480

Fimbraria is congeneric with Asterella, but the following taxa have neither been transferred nor synonymized, and their status is doubtful.

481

Fimbraria gigantea belongs to Asterella subg. Asterella sect. Brachyblepharis.

482

Fimbraria incrassata was provisionally placed in synonymy with Asterella abyssinica (Wigginton and Grolle 1996), and was listed as an unassigned Asterella species by Wigginton (2009).

483

Fimbraria kamerunensis was provisionally placed in synonymy with Asterella abyssinica (Wigginton and Grolle 1996), and was listed as an unassigned Asterella species by Wigginton (2009).

484

Hygrolejeunea is congeneric with Lejeunea, but some taxa have neither been transferred nor synonymized.

485

Hygrolejeunea patellirostris is conspecific with Lejeunea acuta in Tixier (1995b) and Wigginton and Grolle (1996), but it was kept as an unassigned Lejeunea species by Wigginton (2009).

486

Hygrolejeunea staudtiana is likely to be either Lejeunea brenanii or Lejeunea isophylla (Wigginton and Grolle 1996). It was kept as an unassigned Lejeunea species by Wigginton (2009).

487

Hypenantron is congeneric with Asterella, but a few taxa have neither been transferred nor synonymized.

488

Jamesoniella is congeneric with Syzygiella (Feldberg et al. 2010a), but one taxon has neither been transferred nor synonymized. The holotype was burned in B (Grolle 1971b) and it is not clear where it belongs.

489

Kingiolejeunea is congeneric with Lepidolejeunea, but one taxon has neither been transferred nor synonymized.

490

Leptocolea is congeneric with Cololejeunea, but one taxon has neither been transferred nor synonymized.

491

Leptocolea sumatrana is possibly conspecific with Cololejeunea equialbi.

492

Mastigobryum is congeneric with Bazzania, but many species have not been studied recently and thus not transferred. It is possible that some prove to be older names of existing taxa when their identities are known. Grolle and Piippo (1984) could not study Mastigobryum ledermannii, Mastigobryum londbergii and Mastigobryum longifolium since the types were destroyed in B.

493

Nemoursia tuberculata is a Conocephalum species.

494

Physocolea is congeneric with Cololejeunea, but one taxon has neither been transferred nor synonymized.

495

Physocolea tambillensis is possibly conspecific with Myriocoleopsis minutissima.

496

Plectocolea is now regarded a subgenus of Solenostoma, but one taxon has neither been transferred nor synonymized.

497

Plectocolea subamoena is probably conspecific with Solenostoma amoena.

498

Polyotus is a superfluous name for Lepidolaena, but a few taxa have neither been transferred nor synonymized.

499

Schisma is congeneric with Herbertus, but a few taxa have neither been transferred nor synonymized.

500

Strepsilejeunea is congeneric with Cheilolejeunea, but some taxa have neither been transferred nor synonymized.

501

Thysanolejeunea africana may be conspecific with Caudalejeunea africana.

502

Trachylejeunea englishii is not a Trachylejeunea species (see Zhu and So 1999a). The status needs further study.
